# Systematic Exploration of Potential Druggable Genes for Ischemic Stroke Employing Genome‐Wide Mendelian Randomization Analysis

**DOI:** 10.1002/brb3.70857

**Published:** 2025-09-09

**Authors:** Peng Zhang, Yulu He, Qing Zhen, Yan Zhang

**Affiliations:** ^1^ Department of Epidemiology and Biostatistics, School of Public Health Jilin University Changchun China; ^2^ Stroke Center & Clinical Trial and Research Center for Stroke, Department of Neurology the First Hospital of Jilin University Changchun China; ^3^ Genetic Diagnosis Center the First Hospital of Jilin University Changchun China; ^4^ Department of Thoracic Surgery II, Department of Lung Transplantation, Organ Transplantation Center the First Hospital of Jilin University Changchun China

**Keywords:** Bayesian colocalization, druggable gene, ischemic stroke, Mendelian randomization

## Abstract

**Background:**

Ischemic stroke (IS) treatment remains a significant challenge. This study aimed to identify potential druggable genes for IS using a systematic druggable genome‐wide Mendelian Randomization (MR) analysis.

**Methods:**

Two‐sample MR analysis was conducted to identify the causal association between potential druggable genes and IS. This involved integrating data from the druggable genome, expression quantitative trait loci (eQTL), protein quantitative trait loci (pQTL), and genome‐wide association study summary data of IS. Sensitivity and Bayesian colocalization analyses were used to validate the causal relationships. In addition, phenome‐wide MR analysis was used to evaluate the side effects or other indications of the identified druggable genes, and their functions were explored using the Metascape database.

**Results:**

Our MR analysis identified 16 potential druggable genes significantly associated with IS, three of which were significant in the two QTL datasets. Colocalization analysis revealed six druggable genes (two in the blood eQTL [*CALCRL, KCNJ11*], two in the brain eQTL [*NEK3, THSD1*], one in the blood pQTL [*MMP12*], and one in the brain pQTL [*HSD17B12*]) had a PP.H4 greater than 0.75. Phenome‐wide MR analysis indicated that *CALCRL* is correlated with benign breast neoplasms, and *HSD17B12* is associated with essential hypertension and hypertension.

**Conclusions:**

This study identified six potential druggable genes (*CALCRL*, *KCNJ11*, *NEK3*, *THSD1*, *MMP12*, and *HSD17B12*) associated with IS risk. Further research is required to explore the specific roles of these druggable genes in the onset and progression of IS.

## Graphical abstract

This study identified six potential druggable genes (*CALCRL*, *KCNJ11*, *NEK3*, *THSD1*, *MMP12*, and *HSD17B12*) associated with ischemic stroke risk using a systematic druggable genome‐wide Mendelian randomization analysis.

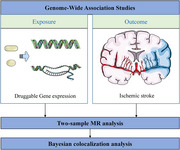

## 1 Background

Stroke is the second leading cause of death and a major cause of disability globally, presenting substantial challenges for affected individuals and exerting a significant burden on societal resources (Saini et al. 2021). Ischemic stroke (IS) accounts for approximately 80%–90% of all stroke incidents (Saini et al. 2021), characterized by the occlusion of cerebral vessels, which leads to localized cerebral tissue ischemic necrosis or infarction due to insufficient blood and oxygen supply.

Currently, the treatment of IS remains challenging. Recombinant tissue plasminogen activator (tPA) is still the most commonly used medication for thrombolytic therapy of IS (Hacke et al. 2008; Jauch et al. 2013). However, its use is limited by a strict treatment time window, resulting in the inability of most patients to receive this treatment. Moreover, thrombolytic therapy is unsuitable for individuals with certain hemorrhagic conditions. For eligible patients who experience acute IS within 24 h of symptom onset (particularly those with large vessel occlusions), endovascular thrombectomy can be utilized for reperfusion (Powers et al. 2019). Nonetheless, whether thrombolytic therapy or endovascular thrombectomy is used, the clinical efficacy of these treatments is often limited, and unfortunately, some patients continue to experience disability posttreatment (Hacke et al. 2008; Goyal et al. 2016). Therefore, it is necessary to explore potential therapeutic targets for IS treatment.

Mendelian randomization (MR) is a method that utilizes genetic variants as instrumental variables (IVs) to elucidate the causal relationship between exposure and outcome (Emdin et al. 2017). Expression quantitative trait loci (eQTL) or protein quantitative trait loci (pQTL) are types of multi‐omics data that reveal the relationships between genetic variations and the expression levels of genes and proteins. The expression levels indicated by eQTL or pQTL within the genomic regions of druggable genes serve as proxies for these genetic influences, representing a type of lifelong exposure (Z. Zhu et al. 2016; Schmidt et al. 2020). MR analysis is commonly used to identify novel therapeutic targets by combining summary data from genome‐wide association studies (GWAS) of disease and QTL studies (Su et al. 2023; Gu et al. 2023; C. Zhang et al. 2024). In this study, we aimed to identify potential druggable genes for IS using MR analysis incorporating druggable genome data, eQTL data, pQTL data, and GWAS summary data related to IS.

## 2 Methods

**FIGURE 1:**
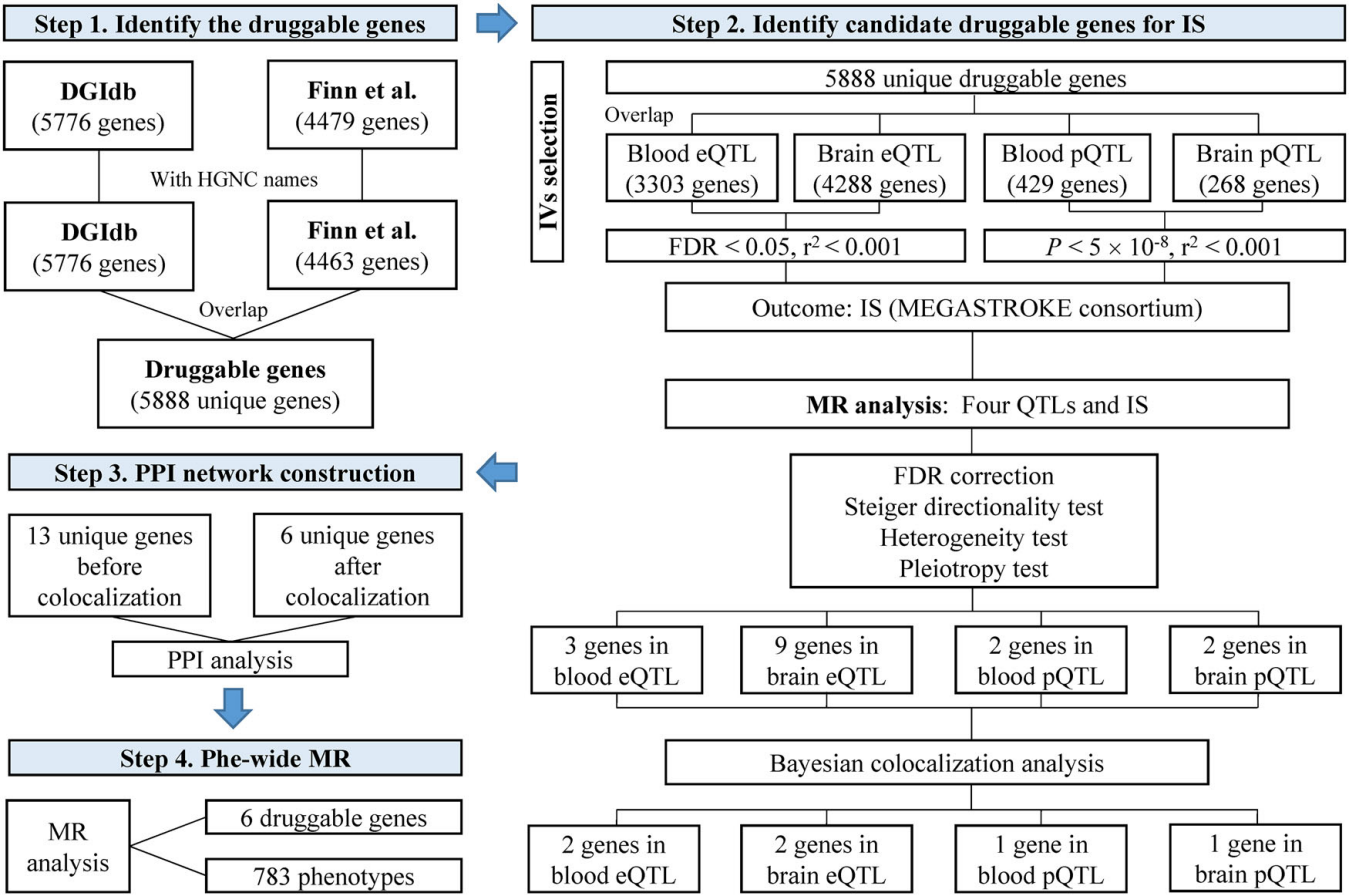
Overview of this study design. DGIdb, Drug‐Gene Interaction Database; eQTL, expression quantitative trait loci; GWAS, genome‐wide association studies; IS, ischemic stroke; MR, Mendelian randomization; Phe‐wide MR, phenome‐wide MR; PPI, protein–protein interaction; pQTL, protein quantitative trait loci.

The overall study design is presented in Figure 1. Summary SNP‐phenotype association data were sourced from published GWAS, where ethical approval and informed consent were obtained in the original studies. The details of the methods are as follows.

### 2.1 Druggable Genes

The identification of druggable genes in this study comes from two sources: the Drug–Gene Interaction Database (DGIdb V.5.0.7, https://www.dgidb.org/) (Freshour et al. 2021) and a recent review on the “druggability” of genes (Finan et al. 2017). The DGIdb, which compiles data on drug–gene associations and genes amenable to drug therapy, gathers its information from a variety of sources, including published works, databases, and online resources. We downloaded a dataset labeled “Categories” from the DGIdb (February 2022), which encompasses all genes identified as druggable across the various sources, linked to their corresponding Entrez gene identifiers (Freshour et al. 2021). In addition, we obtained a list of druggable genes from the attachment of the review by Finan et al. (2017).

### 2.2 eQTL and pQTL Datasets

Data sources for eQTL and pQTL are presented in Table S1. The eQTL data for blood were sourced from the eQTLGen Consortium database (https://eqtlgen.org/), which gathered *cis*‐eQTLs for 16,987 genes from 31,684 blood samples collected from healthy individuals of European descent (Võsa et al. 2021). Data included highly significant *cis*‐eQTL results, identified with a false discovery rate (FDR) less than 0.05, along with information on allele frequencies. For brain eQTL information, we used data from the PsychENCODE Consortium (http://resource.psychencode.org) (Wang et al. 2018), which included data from the prefrontal cortex of 1387 samples, mostly of European ancestry. From this source, we downloaded all significant eQTLs (FDR < 0.05) related to genes exhibiting expression levels exceeding 0.1 fragments per kilobase of transcript per million mapped reads in a minimum of 10 samples and acquired all related single‐nucleotide polymorphism (SNP) information.

The blood pQTL data were sourced from the INTERVAL study, which includes *cis*‐pQTL data for 550 proteins from 3301 individuals of European descent (Sun et al. 2018). Summary statistics for the blood pQTL were obtained from the IEU OpenGWAS Project (https://gwas.mrcieu.ac.uk) (Hemani et al. 2018). Significant SNPs were determined at the threshold of *p* < 5 × 10^−8^. The data on brain *cis*‐pQTLs were obtained from a previous investigation that conducted a proteome‐wide association study using human brain proteomes sourced from the dorsolateral prefrontal cortex of 400 deceased individuals who had donated their brains for research (https://www.synapse.org/Synapse:syn23191787) (Wingo et al. 2021). These individuals were of European descent and part of the Religious Orders Study/Memory and Aging Project (ROS/MAP) cohorts. The genome‐wide significance threshold was set at *p* < 5 × 10^−8^.

### 2.3 IS Dataset

The outcome data of this study were obtained from the MEGASTROKE consortium (http://megastroke.org/), which included information from 440,328 participants of diverse ancestries, consisting of 34,217 cases of IS and 406,111 controls (Malik et al. 2018).

### 2.4 Mendelian Randomization

The R package *TwoSampleMR* (version 0.6.3) was used for the MR analyses. Significant SNPs (FDR < 0.05 for eQTLs; *p* < 5 × 10^−8^ for pQTLs) were selected as IVs. To ensure that the influence of IVs on the outcome was solely due to their effect on exposure, we excluded SNPs directly associated with the outcome. Additionally, to ensure that each selected SNP was independent and eliminate the potential confounding effects of pleiotropy arising from linkage disequilibrium (LD), we adjusted the LD coefficient *r* ^2^ to 0.001 and defined the LD window width at 10 Mb using the clumping function in the *TwoSampleMR* package, referencing the European 1000 Genomes.

After determining the IVs, the same SNPs or their proxies were extracted from the outcome data and harmonized with these IVs. The *F*‐statistics of the IVs were calculated, where an *F*‐statistic < 10 was defined as indicative of a weak IV (Burgess and Thompson 2011). The Wald ratio, or inverse‐variance weighted (IVW) method, was employed to estimate the association between exposure and outcome based on the number of SNPs available (Burgess et al. 2019). To evaluate horizontal pleiotropy, we investigated whether the intercept from the MR‐Egger method differed significantly from zero, provided there were at least three SNPs (Bowden et al. 2015). Additionally, Cochran's *Q* test was employed to assess heterogeneity (Greco et al. 2015). To broadly identify druggable genes for IS, we considered an FDR ≤ 0.1 as significant in this study, which is consistent with standards for exploratory genomic discovery (Wu et al. 2024; Domingo et al. 2018;N. Zhu et al. 2021).

### 2.5 Bayesian Colocalization Analysis

To evaluate evidence against confounding by LD and pleiotropy while assessing whether the genetic associations for gene expression/protein levels (eQTL/pQTL) and IS could be attributed to shared causal variants, we employed a Bayesian colocalization method (Giambartolomei et al. 2014). This method utilized the *coloc* package in R software, which determines the posterior probability of five distinct hypotheses concerning the association of a genetic variant with two traits: (1) PP.H0, no variant is associated with either trait; (2) PP.H1, the variant is linked only to the first trait (eQTL or pQTL) and not to the second (IS); (3) PP.H2, the variant is only associated with the second trait (IS) and not with the first (eQTL or pQTL); (4) PP.H3, the two traits (eQTL/pQTL and IS) are influenced by different variants; (5) PP.H4, both traits (eQTL/pQTL and IS) are influenced by the same variant. A posterior probability exceeding 75% for the last hypothesis suggested that these two traits were influenced by the same genetic variant (Zuber et al. 2022).

### 2.6 Protein–Protein Interaction Network Construction

In order to explore the interactions between potential druggable genes and further understand their mechanisms in the pathogenesis of IS, we conducted protein–protein interaction (PPI) analysis on the druggable genes identified before and after Bayesian analysis. The PPI networks were constructed using the STRING database (version 12.0, available at https://string‐db.org/) (Szklarczyk et al. 2023). The confidence score threshold is set to 0.4 as the minimum required interaction score, while all other parameters remain at the default setting.

### 2.7 Phenome‐Wide Association Analysis

To evaluate potential side effects or other indications of the identified druggable genes, we conducted a Phenome‐wide MR (Phe‐MR) analysis. The data for this analysis were derived from the gene expression data of over 1400 binary phenotypes from over 400,000 participants of European descent in the UK Biobank (W. Zhou et al. 2018). Summary statistics are available at the UKB‐SAIGE GWAS (https://pheweb.org/UKB‐SAIGE/). We included only binary phenotypes with more than 500 cases to ensure statistical significance. Using the MR analysis strategy described above, we explored the relationships between the 783 traits and the identified druggable genes. A Bonferroni‐adjusted significance threshold was applied to account for multiple testing: *p* < 6.39 × 10^−5^ (calculated as *α* = 0.05/783 independent tests).

### 2.8 Function Identification of the Druggable Genes

We further explored the druggable genes identified using the Metascape database (http://metascape.org). The Metascape database integrates multiple authoritative sources, including GO, KEGG, UniProt, and DrugBank (Y. Zhou et al. 2019). This integration enables it to perform not only pathway enrichment and biological process annotation but also gene‐related drug analysis.

## 3 Results

### 3.1 Druggable Genome

We identified 5776 genes categorized as “DRUGGABLE GENOME” from the DGIdb (Table S2). In addition, from a review by Finn et al., we extracted 4463 genes with official names (Table S3). After combining these datasets, we identified 5888 unique druggable genes named by the Human Genome Organization Gene Nomenclature Committee (Table S4).

### 3.2 Candidate Druggable Genes for IS

We cross‐reference the 5888 potential druggable genes with genes in the blood eQTL, brain eQTL, blood pQTL, and brain pQTL datasets; 3303, 4288, 429, and 268 genes remained, respectively. After clumping and harmonizing, 3237 potential druggable genes in the blood eQTL dataset remained for MR analysis, 3501 for brain eQTL, 407 for blood pQTL, and 267 for brain pQTL.

**FIGURE 2:**
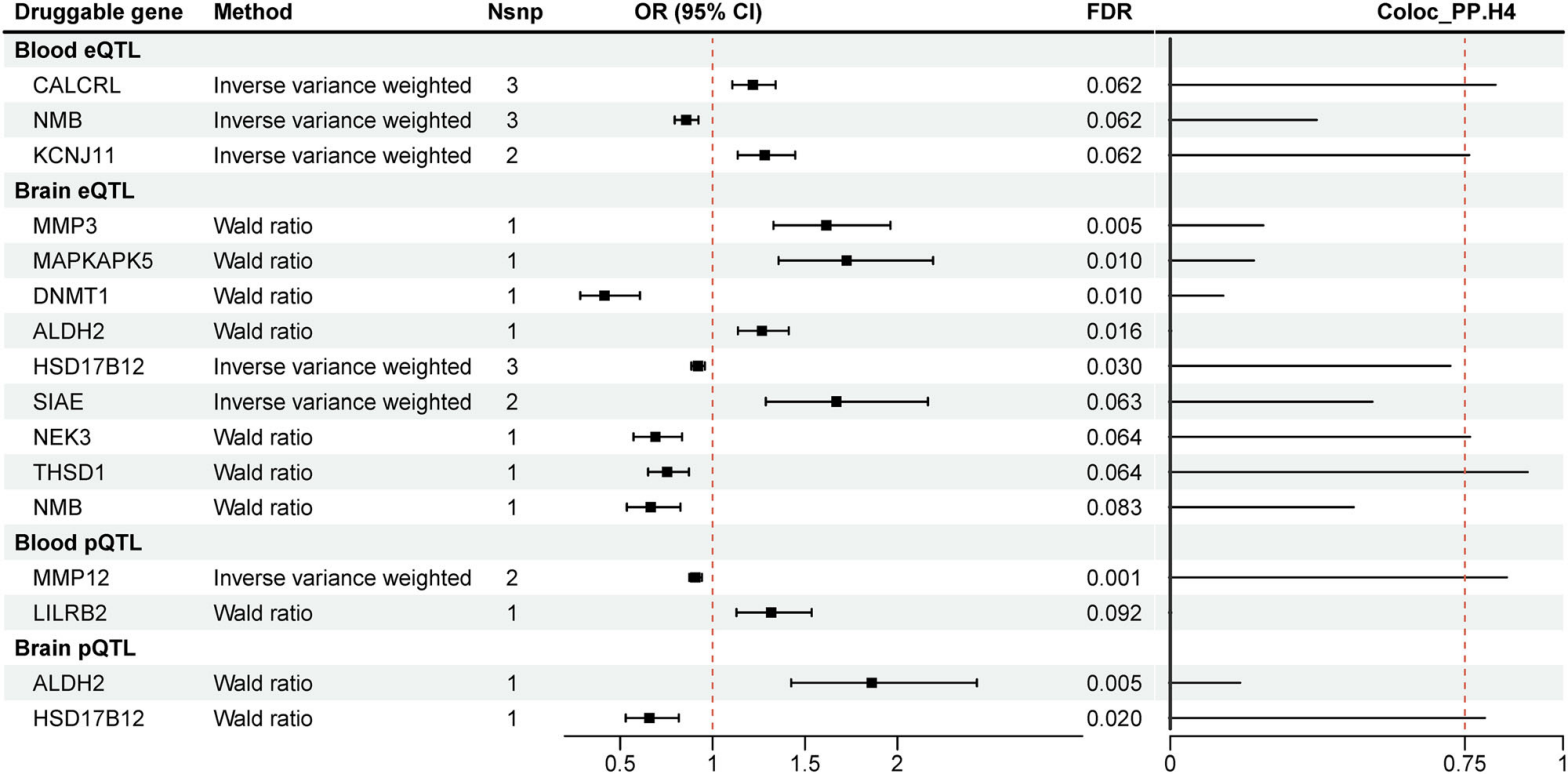
Forest plot of 16 significant druggable genes associated with ischemic stroke from blood and brain. CI, confidence interval; FDR, false discovery rate; OR, odds ratio.

In the MR analysis, we found that 203 druggable genes in the human blood eQTL, 217 in the brain eQTL, 21 in the blood pQTL, and 27 in the brain pQTL had a potential causal relationship with IS (*p* < 0.05) (Tables S5–S8). After multiple corrections, three druggable genes in the blood eQTL, nine in the brain eQTL, two in the blood pQTL, and two in the brain pQTL were confirmed to have a causal relationship with IS at an FDR < 0.1 (Table 1, Figure 2, and Table S9).

**TABLE 1:** MR analysis results of the association between potential druggable genes and ischemic stroke.

Druggable gene	Method	NSNP	Beta	SE	OR (95% CI)	*p* value	*F*‐statistic	FDR
Blood_eQTL								
CALCRL	Inverse variance weighted	3	0.197	0.049	1.218 (1.107–1.341)	5.12e−05	93.14	0.062
NMB	Inverse variance weighted	3	−0.155	0.038	0.857 (0.795–0.923)	5.40e−05	167.42	0.062
KCNJ11	Inverse variance weighted	2	0.249	0.062	1.282 (1.136–1.447)	5.73e−05	97.01	0.062
Brain_eQTL								
MMP3	Wald ratio	1	0.479	0.099	1.615 (1.329–1.962)	1.39e−06	4.06	0.005
MAPKAPK5	Wald ratio	1	0.545	0.122	1.725 (1.357–2.193)	8.60e−06	8.39	0.010
DNMT1	Wald ratio	1	−0.879	0.194	0.415 (0.284–0.607)	6.02e−06	3.94	0.010
ALDH2	Wald ratio	1	0.237	0.055	1.267 (1.137–1.412)	1.82e−05	63.68	0.016
HSD17B12	Inverse variance weighted	3	−0.083	0.020	0.921 (0.885–0.958)	4.26e−05	193.16	0.030
SIAE	Inverse variance weighted	2	0.513	0.132	1.670 (1.288–2.165)	1.08e−04	4.09	0.063
NEK3	Wald ratio	1	−0.370	0.097	0.691 (0.572–0.835)	1.31e−04	22.79	0.064
THSD1	Wald ratio	1	−0.283	0.074	0.754 (0.651–0.872)	1.46e−04	26.54	0.064
NMB	Wald ratio	1	−0.408	0.110	0.665 (0.536–0.826)	2.14e−04	8.14	0.083
Blood_pQTL								
MMP12	Inverse variance weighted	2	−0.097	0.019	0.907 (0.874–0.942)	4.17e−07	57.31	1.70e−04
LILRB2	Wald ratio	1	0.275	0.078	1.317 (1.129–1.536)	4.53e−04	1.66	0.092
Brain_pQTL								
ALDH2	Wald ratio	1	0.621	0.136	1.861 (1.425–2.430)	5.00e−06	19.41	0.005
HSD17B12	Wald ratio	1	−0.418	0.110	0.658 (0.530–0.817)	1.49e−04	48.70	0.020

Abbreviations: CI: confidence interval; eQTL: expression quantitative trait loci; FDR: false discovery rate; OR: odds ratio; pQTL: protein quantitative trait loci; SNP: single nucleotide polymorphism.

To determine whether IS and candidate druggable genes originated from the same genetic variation, we conducted a Bayesian colocalization analysis. The results revealed that six potential druggable genes (two in the blood eQTL [*CALCRL*, *KCNJ11*], two in the brain eQTL [*NEK3*, *THSD1*], one in the blood pQTL [*MMP12*], and one in the brain pQTL [*HSD17B12*]) had PP.H4 greater than 0.75 and passed the colocalization analysis (Table S10 and Figure 2). Higher expression of *CALCRL* and *KCNJ11* was associated with increased IS risk (*CALCRL*: OR = 1.218, 95% confidence interval [CI]: 1.107–1.341; *KCNJ11*: OR = 1.282, 95% CI: 1.136–1.447). Conversely, higher expression of *NEK3*, *THSD1*, *MMP12*, and *HSD17B12* was associated with decreased IS risk, with ORs of 0.691 (95% CI: 0.572–0.835), 0.754 (95% CI: 0.651–0.872), 0.907 (95% CI: 0.874–0.942), and 0.658 (95% CI: 0.530–0.817), respectively.

### 3.3 PPI Network Construction

We initially conducted PPI analysis on 13 unique druggable genes identified before the Bayesian analysis, where gene–gene interactions were identified only between *MMP3* and *MMP12*. Subsequently, PPI analysis was performed on six unique druggable genes identified after Bayesian analysis, which revealed no significant interactions among these genes.

### 3.4 Phenome‐Wide Association Analysis

**FIGURE 3:**
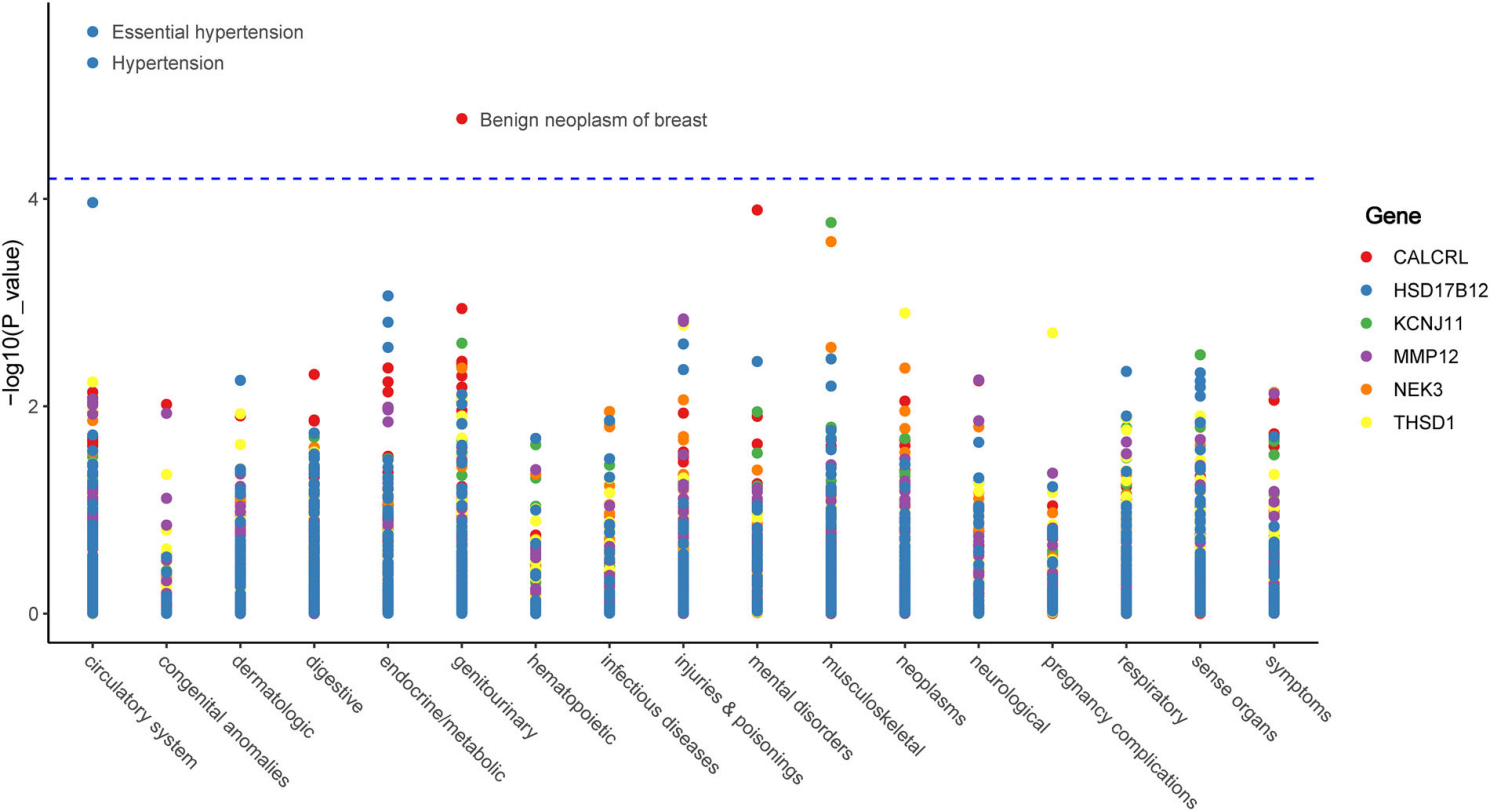
Manhattan plot for phenome‐wide MR results of six potential druggable genes.

We examined the associations between 783 phenotypes (Table S11) and six genes using a significance threshold of *p* < 6.39 × 10^−5^, adjusted by the Bonferroni method for multiple tests (0.05/783). Our findings revealed that an increase in *CALCRL* expression correlated with a lower risk of developing benign breast neoplasms (odds ratio [OR], 0.450; 95% CI: 0.313–0.648). Similarly, elevated levels of *HSD17B12* were associated with decreased risks of essential hypertension (OR: 0.721; 95% CI: 0.629–0.826) and hypertension (OR: 0.728; 95% CI: 0.635–0.834) (Figure 3 and Tables S12–S17).

### 3.5 Function Identification of the Druggable Genes

We explored the biological processes (GO), protein functions (Protein Atlas), and clinical drug development activities related to the six druggable genes (Table 2). Except for *THSD1* and *HSD17B12*, drugs targeting the other four genes (*CALCRL*, *KCNJ11*, *NEK3*, and *MMP12*) have been evaluated for certain diseases. However, none of these have been used to treat IS. According to biological process (GO) analysis, some genes may influence blood glucose levels, blood lipids, and intercellular connections, potentially affecting the occurrence and development of IS.

**TABLE 2:** Function identification of the druggable genes.

Gene symbol	Description	Biological process (GO)	Protein function (protein atlas)	Drug (DrugBank)
CALCRL	Calcitonin receptor‐like receptor	GO:0071324 cellular response to disaccharide stimulus; GO:0071329 cellular response to sucrose stimulus; GO:1990408 calcitonin gene‐related peptide receptor signaling pathway	Disease‐related genes; FDA‐approved drug targets; G‐protein‐coupled receptors; Human disease‐related genes; Plasma proteins; Transporters	Olcegepant; telcagepant; rimegepant; erenumab; ubrogepant; vazegepant
KCNJ11	Potassium inwardly rectifying channel subfamily J member 11	GO:1904638 response to resveratrol; GO:0061762 CAMKK‐AMPK signaling cascade; GO:0035634 response to stilbenoid	Disease‐related genes; FDA‐approved drug targets; Human disease‐related genes; Metabolic proteins; Transporters; Voltage‐gated ion channels	Glimepiride; ibutilide; verapamil; levosimendan; glyburide; diazoxide; thiamylal; isavuconazole
NEK3	NIMA‐related kinase 3	GO:0090043 regulation of tubulin deacetylation; GO:0090311 regulation of protein deacetylation; GO:0030010 establishment of cell polarity	Enzymes	Fostamatinib
THSD1	Thrombospondin type 1 domain containing 1	GO:0048041 focal adhesion assembly; GO:0007044 cell‐substrate junction assembly; GO:0150115 cell‐substrate junction organization	Disease‐related genes	NA
MMP12	Matrix metallopeptidase 12	GO:1904905 negative regulation of endothelial cell‐matrix adhesion via fibronectin; GO:0060309 elastin catabolic process; GO:0060435 bronchiole development	Cancer‐related genes; Enzymes	Acetohydroxamic acid; Marimastat; CP‐271485; PF‐00356231; Batimastat; 2‐[2‐(1,3‐dioxo‐1,3‐dihydro‐2*H*‐isoindol‐2‐yl)ethyl]‐4‐(4′‐ethoxy‐1,1′‐biphenyl‐4‐yl)‐4‐oxobutanoic acid; AE‐941; (1S,5S,7R)‐*N*∼7∼‐(biphenyl‐4‐ylmethyl)‐*N*∼3∼‐hydroxy‐6,8‐dioxa‐3‐azabicyclo[3.2.1]octane‐3,7‐dicarboxamide; *N*‐(biphenyl‐4‐ylsulfonyl)‐d‐leucine; CGS‐27023; *N*‐(dibenzo[*b*,*d*]thiophen‐3‐ylsulfonyl)‐l‐valine; *N*‐oxo‐2‐[(4‐phenylphenyl)sulfonylamino]ethanamide; 2‐[(4‐fluorophenyl)sulfonylamino]‐*N*‐oxo‐ethanamide; *N*‐oxo‐2‐(phenylsulfonylamino)ethanamide; *N*‐isobutyl‐*N*‐[4‐methoxyphenylsulfonyl]glycyl hydroxamic acid; *N*‐[(4‐methoxyphenyl)sulfonyl]‐d‐alanine
HSD17B12	Hydroxysteroid 17‐beta dehydrogenase 12	GO:0019367 fatty acid elongation, saturated fatty acid; GO:0006703 estrogen biosynthetic process; GO:0030497 fatty acid elongation	Enzymes; Metabolic proteins	NA

## 4 Discussion

In this study, we identified 13 unique genes with potential therapeutic targets for IS using eQTL and pQTL data from the human blood and brain samples. Further colocalization analysis provided strong evidence that six genes (*CALCRL*, *KCNJ11*, *NEK3*, *THSD1*, *MMP12*, and *HSD17B12*) showed genetic covariation with IS. In addition, we conducted a Phe‐MR analysis, which revealed the roles of some of these genes in controlling hypertension and benign breast tumors. Furthermore, we constructed a PPI network and investigated gene functions to understand the biological significance and mechanisms of interaction among these druggable genes while exploring their medicinal value.

The human *CALCRL* gene encodes the calcitonin receptor‐like receptor (CRLR), which plays a role in several processes, primarily in G protein‐coupled receptor signaling pathways. The biological effects of *CALCRL* depend on which receptor‐activity‐modifying protein (RAMP) subtype it binds to, allowing *CALCRL* to interact with specific ligands and exert various biological effects (Kotliar et al. 2023). It enables the binding activity of adrenomedullin, adrenomedullin receptor activity, and calcitonin gene‐related peptide (CGRP) receptor activity (McLatchie et al. 1998). Targeted drug discovery efforts focus on developing pharmacological agents that modulate the activity of the CRLR, such as the treatment of migraines (Olesen and Ashina 2011). One such drug is erenumab, a monoclonal antibody targeting the CGRP receptor complex, which has been approved for migraine prevention. Another drug, olcegepant, is a small‐molecule antagonist of the CGRP receptor complex that was used in clinical trials for migraine treatment but was not approved for commercial use. Regarding IS, our MR analysis indicated that higher expression of *CALCRL* was causally associated with the risk of IS. This finding was validated by a recent study (Brabenec et al. 2022), which analyzed the underlying mechanism affecting procalcitonin integrity of the endothelial barrier and VE‐cadherin adhesion junction; the study elucidated the importance of N‐terminal truncation on procalcitonin signaling and determined the potential of CRLR antagonists for vascular barrier protection. The result of the Phe‐MR analysis did not reveal any side effects of the *CALCRL* gene, but it showed a therapeutic effect on benign neoplasms of the breast. Overall, our findings indicate the potential of targeting the *CALCRL* gene in IS treatment.

The *KCNJ11* gene encodes the Kir6.2 subunit of the ATP‐sensitive potassium channels, which are expressed in pancreatic beta cells and are closely associated with insulin secretion and glucose metabolism (Haghvirdizadeh et al. 2015). Mutations in this gene can lead to dysfunction of the ATP‐sensitive potassium channels, thereby affecting insulin secretion and glucose metabolism, potentially resulting in metabolic disorders such as diabetes, neonatal hypoglycemia, and obesity (Bowman et al. 2019). The *KCNJ11* gene may also play an important role in IS. In our study, the expression of the *KCNJ11* gene was positively correlated with the risk of IS. This is consistent with previous research, which indicates that mutations or aberrant expression of the *KCNJ11* gene may lead to an imbalance in cerebral vascular dilation and constriction, thereby affecting cerebral blood flow and ischemic tissue damage in the brain (Gloyn et al. 2006). Furthermore, mutations in the *KCNJ11* gene may result in the dysfunction of ATP‐sensitive potassium channels, impacting the energy metabolism and survival of brain cells. However, the influence of *KCNJ11* on IS, and whether this effect is mediated through diabetes, remains to be further investigated. Consequently, developing medications that target *KCNJ11* appears to be a viable approach. Therefore, further studies are required to investigate how *KCNJ11* influences the risk of IS.

*NEK3* encodes a serine/threonine protein kinase that belongs to the NimA family. Research on *NEK3* has suggested its involvement in various diseases, including cancer, cardiovascular diseases, and neurological disorders. In cancer, *NEK3* has been shown to promote tumor growth and metastasis by regulating cell migration and invasion (Panchal and Evan Prince 2023). In cardiovascular diseases, *NEK3* has been linked to abnormal cardiac left‐right patterning (Y. Zhang et al. 2020). In neurological disorders, *NEK3* has been implicated in the regulation of neuronal development, neuronal morphology, and polarity (Chang et al. 2009). These findings are consistent with our results, which show that *NEK3* was causally associated with the risk of IS. Currently, there is limited research on drug discovery targeting *NEK3*. Our study provides a direction for future drug development targeting *NEK3*.

The *THSD1* gene in humans codes for a protein that possesses a thrombospondin Type 1 domain, commonly found in proteins linked to the complement system and those that form part of the extracellular matrix. Studies have indicated that *THSD1* expression is elevated in different types of cancer, potentially contributing to tumor proliferation and the spread of cancer cells, ultimately exacerbating the condition (Ko et al. 2008; Khamas et al. 2012). In contrast, its role in neurological disorders differs; genetic changes in *THSD1* have been associated with intracranial aneurysms and subarachnoid hemorrhage. These mutations may disrupt the interaction between endothelial cells and the basement membrane, which is crucial for maintaining vascular integrity (Xu et al. 2024; Santiago‐Sim et al. 2016). Furthermore, *THSD1* is identified as a critical regulatory factor in maintaining endothelial barrier integrity during vascular development, offering protection against hemorrhaging in the microvessels of advanced atherosclerotic plaques (Haasdijk et al. 2016). Our findings also indicate that the *THSD1* gene is a protective factor for the risk of IS. The discovery of drugs targeting *THSD1* is still in the early stages of development, and currently, there are no drugs in the market specifically targeting *THSD1*.

*MMP12* is a member of the M10 family of matrix metalloproteinases (MMPs), enzymes that play a crucial role in the degradation of the extracellular matrix during various biological processes. One previous study revealed the role of *MMP12* in poststroke pathogenesis, particularly in the mechanisms of blood–brain barrier disruption, inflammation, apoptosis, and demyelination following cerebral ischemia and reperfusion (Veeravalli 2024). However, there is limited research on the role of *MMP12* in the risk of IS onset. Our findings suggest that *MMP12* may have a protective function in preventing the onset of IS, highlighting the complex interplay between *MMP12* and disease onset and progression. Further investigation into the dose–response relationship of *MMP12* could shed light on these findings. Additionally, the development of therapeutic interventions targeting *MMP12* poststroke may have immediate clinical relevance.

*HSD17B12* is a gene that encodes a crucial enzyme called 17beta‐hydroxysteroid dehydrogenase. *HSD17B12* is involved in fatty acid elongation, which is important for maintaining healthy cell membranes and energy storage (Tsachaki et al. 2020). Research on the role of *HSD17B12* in neurological disorders is limited, with targeted drug development primarily focused on cancer treatment. Our findings revealed that *HSD17B12* prevented the onset of IS and exhibited a prophylactic effect against hypertension and essential hypertension. Therefore, further investigation into the role of *HSD17B12* in the context of IS required.

The strength of our study lies in the use of the largest IS GWAS dataset currently available, which ensures the robustness of our results. Additionally, to broadly identify potential druggable genes associated with IS, we analyzed blood *cis*‐eQTL, brain *cis*‐eQTL, blood *cis*‐pQTL, and brain *cis*‐pQTL data, thereby enhancing the reliability and efficiency of clinical trials for IS drug development. Moreover, our results were subjected to multiple corrections and Bayesian colocalization analysis, which provides stronger evidence of a causal association between IS and druggable genes. Finally, using Phe‐MR analysis, we validated the potential side effects or other indications of druggable genes, offering guidance for subsequent drug development.

However, this study has several limitations. First, although most of the IVs in our MR analysis had *F*‐statistics ≥ 10, the number of SNPs included in many of these IVs was relatively small. This limited our ability to conduct further sensitivity, heterogeneity, and pleiotropic analyses. Future studies should consider using a larger set of SNPs for more comprehensive analyses. Secondly, the sample sizes for some QTL data were small, potentially excluding other potentially druggable genes from the analysis. Third, the GWAS database we used was primarily based on European populations, which restricts the generalizability of our findings to other ethnic groups. Future research involving participants from diverse regions would be valuable. Fourth, our MR analysis assumed a linear relationship between exposure and outcome; however, it is challenging to ascertain whether this assumption is satisfied under the current conditions. Finally, although MR analysis provided evidence of a causal association between druggable genes and IS, the relationship between these genes and diseases may be complex. Therefore, further studies are required to ascertain whether the druggable genes play the same role in the onset and progression of IS to ensure the success of pharmaceutical applications.

## 5 Conclusion

This study identified six potential druggable genes (*CALCRL*, *KCNJ11*, *NEK3*, *THSD1*, *MMP12*, and *HSD17B12*) associated with IS, using MR and colocalization analyses. These findings highlight the significance of druggable genes associated with IS, offering promising clues for more effective treatments and potentially reducing the cost of drug development. Further research is necessary to examine the specific roles of these druggable genes in the onset and progression of IS.

## Author Contributions

**Peng Zhang**: conceptualization, methodology, formal analysis, visualization, writing – review and editing, writing – original draft. **Yulu He**: formal analysis, methodology, visualization, writing – original draft. **Qing Zhen**: conceptualization, writing – review and editing, methodology. **Yan Zhang**: writing–review and editing, methodology, conceptualization, supervision, validation.

## Conflicts of Interest

The authors declare no conflicts of interest.

## Peer Review

The peer review history for this article is available at https://publons.com/publon/10.1002/brb3.70857.

## Supporting information




**Supplementary Tables**: brb370857‐sup‐0001‐TableS1‐S17.pdf

## Data Availability

Data analyzed in this study were a reanalysis of existing data, which are openly available at locations cited in the main text.
