# Supplementary material for: Systematic Exploration of Potential Druggable Genes for Ischemic Stroke Employing Genome‐Wide Mendelian Randomization Analysis
Source: Brain Behav. 2025 Sep 9;15(9):e70857. doi: 10.1002/brb3.70857 (PMC12417969; doi:10.1002/brb3.70857)
Supplement: Supplementary file 1 — Supplementary Tables: brb370857‐sup‐0001‐TableS1‐S17.pdf [file BRB3-15-e70857-s001.pdf]

Table list

Table S1 Data sources of this sutdy.
Table S2 The druggable genes identified from the DGIdb v5.0.7.
Table S3 The druggable genes identified from the review by Finn et al.
Table S4 The unique druggable genes identified by overlaping the two source of the prior druggable genes.
Table S5 MR results of the association between blood eQTL and ischemic stroke (IVW or Wald ratio methods).
Table S6 MR results of the association between brain eQTL and ischemic stroke (IVW or Wald ratio methods).
Table S7 MR results of the association between blood pQTL and ischemic stroke (IVW or Wald ratio methods).
Table S8 MR results of the association between brain pQTL and ischemic stroke (IVW or Wald ratio methods).
Table S9 Detailed MR results of the significant druggable genes (FDR < 0.1) and ischemic stroke.
Table S10 The results of the colocalization analysis.
Table S11 The detail of the 783 phenotypes in the phenome-wide MR analysis.
Table S12 The phenome-wide MR analysis results of CALCRL (IVW or Wald ratio methods).
Table S13 The phenome-wide MR analysis results of KCNJ11 (IVW or Wald ratio methods).
Table S14 The phenome-wide MR analysis results of NEK3 (IVW or Wald ratio methods).
Table S15 The phenome-wide MR analysis results of THSD1 (IVW or Wald ratio methods).
Table S16 The phenome-wide MR analysis results of MMP12 (IVW or Wald ratio methods).
Table S17 The phenome-wide MR analysis results of HSD17B12 (IVW or Wald ratio methods).

Table S1. Data sources of this sutdy.

Dataset	Sample size	Ancestry	PMID	Data source
blood eQTL	31684	mostly European descent	34475573	https://eqtlgen.org/
brain eQTL	1387	mostly European descent	30545857	http://resource.psychencode.org
blood pQTL	3301	European descent	29875488	https://doi.org/10.1038/s41586-018-0175-2.
brain pQTL	376	European descent	33510477	https://www.synapse.org/Synapse:syn23191787

Table S2. The druggable genes identified from the DGIdb v5.0.7.

Number	Gene list
1	SDF2
2	KCNK2
3	ASIC1
4	OBP2A
5	RFPL3
6	GFRA4
7	GUCA2B
8	H4C2
9	SLC5A1
10	FZD10
11	RNASE1
12	SNAP23
13	FRK
14	GNPTG
15	ABCG4
16	SSTR3
17	C4BPA
18	GRIN2B
19	BRCA1
20	PRCP
21	S100A12
22	CD3G
23	KYNU
24	CD37
25	STK36
26	KLHL23
27	FBLN1
28	VWA5A
29	GPR151
30	ICAM4
31	BRD2
32	SLC12A7
33	SIK1
34	ITPR1
35	CCL3L3
36	FFAR1
37	ABCA5
38	CX3CL1
39	IGFBP4
40	PDGFD
41	KLHL10
42	NGF
43	NOG
44	AKR1A1
45	MTNR1A

46 ARTN
47 HNRNPUL1
48 OR5K4
49 C5
50 MYH6
51 EHMT1
52 CCL5
53 TRPM3
54 NPC1L1
55 STK4
56 UTS2
57 PEAK1
58 PTH
59 SERPINB7
60 CYP20A1
61 ENTPD6
62 ABCC5
63 KMO
64 PMPCA
65 HTR3E
66 TMED10
67 CLPSL2
68 OR5D14
69 P4HA1
70 OR5AN1
71 EXTL2
72 PADI1
73 CD68
74 CHST8
75 AGA
76 PRKAB1
77 AQP4
78 SNCA
79 ACKR3
80 WDR5
81 SLC27A4
82 DAPK3
83 LSS
84 NLGN1
85 OR10G4
86 OR13H1
87 OR2T34
88 FMO6P
89 CNGA3
90 TUBB4A
91 PSEN1
92 POLE
93 SLC4A11

94 SMPDL3B
95 CD22
96 OR1C1
97 LPCAT3
98 RXFP2
99 SNAP29
100 NDOR1
101 PGLYRP2
102 LCN8
103 CD200R1
104 CACNA2D2
105 PLAU
106 OR2AG2
107 GART
108 PLCE1
109 SLC10A3
110 OR1D2
111 COQ8B
112 QARS1
113 CTSL
114 GPR68
115 MIP
116 PTH1R
117 NTN3
118 OPN1MW2
119 CHST10
120 OXNAD1
121 EGFL7
122 KLHL6
123 PAPPA
124 AKR1D1
125 GFOD2
126 OR2T1
127 GSTO1
128 NMU
129 CAMK4
130 OXTR
131 OR5K3
132 TRPA1
133 OR2W1
134 ADCY8
135 REG3A
136 RLN1
137 PIK3R4
138 PLA2G10
139 CNTF
140 CTRC
141 NOTCH1

142 FCGR1A
143 ABCG8
144 CLK1
145 FGF17
146 PLBD2
147 IGLV4-60
148 ALDH7A1
149 ALK
150 OR8U1
151 SLC18A1
152 CAPZA2
153 NRBP1
154 ATG4C
155 KCNV1
156 SEZ6L2
157 GALR2
158 GUCY2D
159 IGLV3-25
160 AIMP1
161 CDK4
162 TRPM2
163 ACHE
164 SLC6A18
165 SLC2A10
166 KLK12
167 LGR5
168 GRIN3B
169 TOP1
170 ATP1A3
171 PRELP
172 DUSP7
173 RHCE
174 BBS2
175 IL20RA
176 GPR37L1
177 PAN3
178 ABCC6
179 TMCO3
180 OR52P2P
181 TXNDC11
182 CD55
183 CDK1
184 SCN7A
185 CYP4Z1
186 REG3G
187 EPHB6
188 OR51T1
189 C6ORF15

190 SLC16A13
191 INSL6
192 CAPN11
193 OR2T8
194 AIFM1
195 OR10A6
196 ALDH5A1
197 OR9M1P
198 OR8K5
199 APOF
200 IL18
201 ACR
202 CA12
203 ALDH1B1
204 NEK6
205 GFRA3
206 C1QTNF9
207 OR4C6
208 HSD17B14
209 IFNGR2
210 FBP2
211 GRID1
212 SLC25A39
213 ASIC2
214 FSD1
215 OR10H2
216 THBS3
217 TNFRSF1A
218 TTL
219 DYRK1A
220 CHRM4
221 CDKN1A
222 KCNK9
223 FKBP14
224 MAOA
225 TRIM7
226 SLCO3A1
227 SULF2
228 RELN
229 HNF4G
230 FGF7
231 RTN4R
232 NPY6R
233 WNT3
234 TAS1R3
235 IL17B
236 ADAM22
237 PHOSPHO1

238 RLN2
239 TYR
240 DAGLB
241 LBP
242 ENG
243 NQO1
244 HSD17B7P2
245 C16ORF89
246 GPR156
247 CCNA2
248 PDE4A
249 CYB5R3
250 COL1A1
251 NR4A3
252 IGKV3-7
253 HAPLN1
254 TAAR8
255 DUOX2
256 CRTAC1
257 AIP
258 ASIC4
259 SLC4A2
260 SLC7A7
261 CXCL1
262 ITGA6
263 ADAMTS4
264 MARS2
265 SRD5A2
266 NTM
267 EFEMP1
268 DHRS9
269 DDI1
270 ROS1
271 AGR3
272 USP47
273 GPR153
274 ROR2
275 GPR27
276 OR2T3
277 THSD1
278 SOD2
279 MBOAT1
280 COL2A1
281 PIK3C2A
282 HPR
283 BCAM
284 SLC22A18
285 CYP46A1

286 BPNT1
287 TUBB2A
288 TESK1
289 OR13D3P
290 S1PR2
291 PGA5
292 SLC16A12
293 XBP1
294 ATP2C2
295 CD300E
296 KDM1B
297 HDAC4
298 PIK3IP1
299 NR3C2
300 ALOX12B
301 TNFSF8
302 SCN3B
303 OR8H2
304 GABRE
305 DHRS7
306 TXNDC2
307 COMT
308 PRIMPOL
309 PSMB11
310 UQCC3
311 OGN
312 BACE2
313 RBM5
314 MMRN1
315 APOC2
316 KCNMA1
317 ADH1A
318 SMAD3
319 NPY4R
320 BMP2
321 CSPG4
322 KCNJ8
323 AKR1B15
324 SERPINE1
325 ADGRE3
326 IGLV7-46
327 ATP1A2
328 DCD
329 ABL2
330 TRIM49D2
331 TRIM58
332 CDK8
333 OR4N4

334 SLC16A5
335 PRKCH
336 H3C6
337 CDK11A
338 TGFBR3
339 CD2
340 AGBL1
341 ATP2A2
342 FBLN2
343 CACNG6
344 IL1F10
345 PTPN9
346 HSP90B1
347 SLC13A3
348 ACVR1B
349 FSHR
350 CD34
351 S100A8
352 GMNN
353 OR5E1P
354 INPP5J
355 OR6S1
356 MRC2
357 UGT2A1
358 ABAT
359 CSNK1G1
360 RPS6KB2
361 THPO
362 EGFLAM
363 HYAL3
364 PON1
365 SLC2A3
366 GUCY1B1
367 IKBKB
368 PPP1CA
369 DEFB126
370 DPEP3
371 KCNN4
372 IGLV1-40
373 MMP23B
374 SLC25A15
375 ADAM20
376 KCNV2
377 DAO
378 LAD1
379 TWSG1
380 LRPAP1
381 UHMK1

382 SPSB4
383 ADGRF5
384 CYP2R1
385 PRSS48
386 CHRFAM7A
387 IGKC
388 TNNT2
389 OR52N4
390 OR51S1
391 AR
392 CACNA1C
393 OR4F15
394 ITGA9
395 HLA-DRB1
396 ADCY4
397 NR1H4
398 PSMA3
399 IMPDH2
400 OR51J1
401 NPY5R
402 DRD5P1
403 ATP7B
404 TUBBP5
405 VRK2
406 WNT4
407 GSTM3
408 TRIM38
409 CASP6
410 TNFRSF10B
411 ALOX5AP
412 SRPK2
413 PMS2
414 PON2
415 GPR176
416 PDE1B
417 THBS1
418 CHRNA6
419 LILRB2
420 KCNJ15
421 ARSK
422 GDF9
423 GHRHR
424 KCNK3
425 ITGA1
426 GPIHBP1
427 GRM7
428 CD36
429 DYRK4

430 C1QC
431 SLC4A8
432 CDH5
433 FBN2
434 PIP5K1C
435 HDAC2
436 WT1
437 CD74
438 IGLV2-11
439 MICAL3
440 AMY1A
441 OR2M3
442 NR5A1
443 APAF1
444 BBOX1
445 SLC29A2
446 MAP3K14
447 OR2G3
448 NRXN1
449 P2RY4
450 CRLF2
451 OR9Q2
452 PTGFR
453 OR6N2
454 SCGB3A2
455 CFC1
456 KBTBD6
457 CELA3B
458 MYORG
459 LIPF
460 TP53I3
461 CEACAM1
462 SPSB3
463 IL15RA
464 LAIR1
465 APOH
466 MAPK13
467 MUC6
468 CHRM2
469 NOTCH4
470 CACNG2
471 FKBP8
472 OR8H3
473 CACNA1G
474 CHRNA10
475 THY1
476 CYP7B1
477 HP

478 SERPINA13P
479 FER
480 WNT5A
481 ALDH3B2
482 PDK2
483 SDC1
484 ATP4B
485 EYS
486 CAT
487 KCNJ6
488 OR5M8
489 NTS
490 CYP3A43
491 PAH
492 CASP7
493 HRAS
494 MINPP1
495 MMP9
496 ABL1
497 TEC
498 OR5D3P
499 KCNAB1
500 SPINT4
501 LMCD1
502 PTPN13
503 PARP4
504 OR5H15
505 LOXL1
506 ZG16
507 ADHFE1
508 FCRLA
509 SLC19A1
510 CCR4
511 IL21
512 MSMP
513 SELENOP
514 CCL25
515 RLN3
516 RPN2
517 GPA33
518 OR7E149P
519 BMX
520 PMEL
521 NAAA
522 RIOK3
523 PAK3
524 OR5M5P
525 IL24

526 RPS27
527 SLC28A3
528 NTRK3
529 CCL2
530 SERPINF1
531 HDAC11
532 PIP5K1A
533 PRL
534 SRMS
535 SLC1A6
536 ABCF3
537 HMOX1
538 ALPI
539 GAL
540 OLFM2
541 CASP12
542 OR8U8
543 CD109
544 DDOST
545 CA13
546 PKDREJ
547 CST8
548 STAT3
549 NFATC3
550 CYP2W1
551 OR8B2
552 OXGR1
553 CDK19
554 SLC25A13
555 ALOX15B
556 RNPEP
557 MYLK4
558 OR52H1
559 ABCB9
560 MAP3K4
561 ITGA2B
562 NPSR1
563 LILRA2
564 BMP1
565 PDCD1LG2
566 ACKR4
567 DPP10
568 EWSR1
569 SIGLEC7
570 AZU1
571 LIPH
572 EMILIN1
573 CHRNE

574 GRK4
575 PTPRN2
576 RNGTT
577 LDHB
578 LGALS1
579 GAPDHS
580 FGF5
581 OR13G1
582 EDIL3
583 DEFA1B
584 HCN4
585 IMPG1
586 PTPRJ
587 FUT2
588 IL1RL1
589 LRP1
590 DEFB105B
591 LPAR5
592 ITGB8
593 GPRC5B
594 ADH7
595 CYP27A1
596 SLC25A31
597 ABCA10
598 RXRG
599 CDKL1
600 CHRNB2
601 HSD17B8
602 TARS1
603 SIRT3
604 IFNA10
605 MARK4
606 TXNDC9
607 TAOK2
608 DEFB104B
609 NAMPT
610 S1PR4
611 MYLK
612 FABP2
613 IGLC3
614 UGT2B28
615 MAP3K1
616 PTPN23
617 TAC1
618 CR2
619 SGK3
620 AIFM2
621 RIPK1

622 WNT5B
623 PTH2
624 AHSG
625 KLHL30
626 TMPRSS11D
627 FRZB
628 SLC28A1
629 TMPRSS5
630 CCND3
631 BMP4
632 CCNE2
633 CLCA1
634 TLR6
635 OR6X1
636 IKBKG
637 COL4A2
638 FLNA
639 IL18RAP
640 ALPL
641 TNFSF14
642 GSTA1
643 DHODH
644 PDIA3
645 ACACA
646 TRIM17
647 CYSLTR1
648 SMN1
649 SLC6A17
650 CFHR2
651 SLC4A5
652 CSPG5
653 FGFBP2
654 MBOAT4
655 HSD3BP1
656 CD99
657 UCMA
658 LEFTY1
659 CPN2
660 KLHL26
661 LRRK1
662 FBN3
663 TRIM60
664 VAT1
665 ADAM33
666 OR8B12
667 NFATC4
668 WWOX
669 IGKV1D-39

670 PDE10A
671 ACRBP
672 NPPC
673 CD82
674 SHH
675 DRD4
676 RTN4
677 CD27
678 AAK1
679 INPPL1
680 NME9
681 LILRB4
682 ITIH3
683 CHRNA2
684 ROCK1
685 PRTFDC1
686 CEACAM7
687 OR2H5P
688 TOR2A
689 MAP3K3
690 PLSCR2
691 TSPO2
692 SAA1
693 KCNU1
694 PROC
695 SERPINA11
696 RNPEPL1
697 B4GALT1
698 LY86
699 GSTZ1
700 MKNK2
701 TMX1
702 MTNR1B
703 RABGGTB
704 CHRNB1
705 PRTN3
706 CMYA5
707 DEFB116
708 DHRS1
709 CLCN6
710 SERPINB9
711 CNR1
712 MTRNR2L5
713 ADGRG2
714 AHR
715 TRIM16L
716 PSEN2
717 PLCXD2

718 ADRB3
719 TRIO
720 PRPF4
721 LHCGR
722 OR13D1
723 SLPI
724 GFER
725 CCNA1
726 CXCR5
727 KLK1
728 OPN1MW
729 MYT1
730 SLCO6A1
731 TNFSF11
732 EDAR
733 RTN3
734 LARS1
735 OR5T2
736 PM20D1
737 ABCC12
738 OR2W6P
739 ADA
740 OR1G1
741 GRIK2
742 HDAC1
743 GRIN2A
744 CFHR5
745 IFNA16
746 SLC16A7
747 VIPR1
748 HLA-DPA1
749 FKBP15
750 NXPE1
751 TPCN1
752 EDNRB
753 FOLR2
754 OC90
755 OR4A15
756 SLC9C1
757 IL4R
758 FGF18
759 TNFRSF8
760 PLA2G12A
761 FKBP7
762 ABCB7
763 PTPRZ1
764 PLK3
765 UCP1

766 HDAC8
767 AGRP
768 ADAL
769 UGT2B10
770 TMPRSS6
771 ATP2A1
772 SLC25A3
773 CTSK
774 OR52Z1P
775 CDC14C
776 UGT1A3
777 GLRA4
778 SPP1
779 DBF4
780 ITGB4
781 SST
782 CTSL3P
783 CCNB1
784 KLRC1
785 ATP2B4
786 PRKD3
787 AHCYL2
788 OR10V1
789 DLK1
790 TP53
791 MRGPRF
792 CTSD
793 GSTA4
794 SLC5A4
795 MLX
796 GRIA3
797 SSR2
798 WNK1
799 OPRPN
800 HSDL1
801 CCN2
802 TUBB8
803 CCL8
804 INHBC
805 OPRK1
806 ITLN2
807 IGFBP1
808 CSF1
809 IFNA17
810 CHRDL2
811 CPAMD8
812 LEP
813 MOXD1

814 SFRP2
815 PLAUR
816 ENDOU
817 ADH6
818 GSTM4
819 HVCN1
820 ATP6V1B1
821 OR2A12
822 TRIM27
823 F13B
824 OR10G3
825 IL17RB
826 ANGPTL3
827 OR11G2
828 BCL2L15
829 CA3
830 THRA
831 COL6A5
832 C6
833 BLK
834 IL13RA2
835 MARS1
836 CLK2
837 UPK3A
838 SLC37A1
839 OR8G1
840 CCND1
841 JAK1
842 DAPK2
843 SLC25A33
844 STC1
845 ITPR3
846 SBK3
847 CA5A
848 METAP1D
849 NECTIN2
850 SLC7A5
851 INHBE
852 ABHD12
853 AMHR2
854 KCNK10
855 DEFB115
856 LCN9
857 RRH
858 GLB1
859 CHST6
860 IL4I1
861 PTGR2

862 FCAMR
863 ALDH8A1
864 ANTXR2
865 CACNG3
866 PPIH
867 SCNN1G
868 GFM1
869 KRT8
870 RNLS
871 DYRK3
872 H4C1
873 GPLD1
874 FKRP
875 TMX3
876 GGT7
877 APLNR
878 NR4A1
879 NR6A1
880 IL17A
881 SIK3
882 TIMP1
883 RSPRY1
884 CD33
885 OR4C2P
886 ADAMDEC1
887 SERPINB12
888 OR51B4
889 GH2
890 TMPRSS15
891 NLGN4X
892 NEK10
893 IL34
894 TGFB3
895 NME8
896 IGHG1
897 GPR19
898 SLC25A19
899 ACVR2B
900 DIO2
901 OR2M7
902 COL21A1
903 BSG
904 KLK8
905 ELAVL1
906 ABCC10
907 KSR2
908 HIPK2
909 ADAMTS13

910 FAT3
911 F9
912 ADM2
913 SREBF2
914 LIPJ
915 RAMP1
916 CD1D
917 OPN5
918 ATRAID
919 ENOX1
920 VAMP2
921 PCYOX1L
922 ADGRG3
923 NEU1
924 RBM10
925 GPR45
926 GRM4
927 PHF8
928 JAK3
929 OR4M1
930 SLC7A13
931 CD69
932 C3AR1
933 LOXL4
934 IHH
935 SERPINA3
936 IL10RA
937 CDKN3
938 CCRL2
939 COL4A6
940 SDR42E2
941 XYLT1
942 TUBA1C
943 NID2
944 COL9A2
945 OR7G3
946 SERPINH1
947 GRIA4
948 SLC2A13
949 PLIN2
950 CLEC14A
951 SLC25A41
952 CTSE
953 HYAL2
954 OR2F2
955 EPHA3
956 INPP5B
957 SYT15

958 SLC15A5
959 CTSO
960 EPHA6
961 TFF3
962 DAPK1
963 RARG
964 OR4D10
965 TYRO3
966 ADAM29
967 TBXA2R
968 PRPS2
969 TRPC6
970 ADGRD1
971 SPNS1
972 GGT1
973 NR1H3
974 HSD11B2
975 CNTNAP5
976 MTMR1
977 SEMA3G
978 EEF2K
979 SERPINA4
980 PGLYRP3
981 ANGPTL1
982 ITGB2
983 MAP3K12
984 CRTAP
985 ANXA2
986 EIF2AK4
987 SCN1A
988 PTPRB
989 MAOB
990 CMKLR2
991 AVPR1B
992 HTRA4
993 PPME1
994 NEK8
995 AXL
996 NEK2
997 FKBP1A
998 BTC
999 CLEC10A
1000 ZRANB2
1001 OPTC
1002 GDF11
1003 GPR161
1004 OR4E2
1005 EGF

1006 CNDP2
1007 LGR4
1008 OR51I2
1009 SLC7A3
1010 LMTK3
1011 SLC22A9
1012 KCNK16
1013 MAST1
1014 OR8H1
1015 LEAP2
1016 PNMT
1017 IGHE
1018 ATP2C1
1019 QDPR
1020 IL9R
1021 MAPK7
1022 H3C2
1023 NISCH
1024 OR1S1
1025 CAMK1D
1026 AKR1B1
1027 OR4C5
1028 SCG2
1029 PTGIR
1030 PLA2G12B
1031 MAP3K21
1032 KCNJ4
1033 OR2AT4
1034 C1R
1035 PYGL
1036 OR2AE1
1037 TRPC5
1038 IL7
1039 BTD
1040 PI4KA
1041 MMP24
1042 TUBB2B
1043 HSDL2
1044 IFIT1
1045 HLA-DQA2
1046 NMBR
1047 TAF1A
1048 ZADH2
1049 MASTL
1050 ESRRB
1051 OR10A7
1052 INS
1053 MRGPRE

1054 OR3A4P
1055 GPR139
1056 BMP2K
1057 KLRB1
1058 EFNA5
1059 P2RY6
1060 RNF135
1061 ABCC11
1062 TACR1
1063 SLC22A5
1064 PHKG1
1065 GH1
1066 NPPB
1067 SLC6A14
1068 CD209
1069 EFL1
1070 LAMP2
1071 GPR78
1072 FGFR3
1073 SLC16A8
1074 OR2B6
1075 PDIA5
1076 ART1
1077 DRD1
1078 OR5M11
1079 SERPINB6
1080 MKNK1
1081 CNMD
1082 MEPE
1083 RSPO4
1084 CDC14B
1085 BOLA1
1086 SV2C
1087 CCL1
1088 GPR12
1089 CD320
1090 PTK6
1091 HAPLN2
1092 ECEL1
1093 PENK
1094 IL17F
1095 SIGLEC10
1096 OXER1
1097 C1RL
1098 GPM6A
1099 CACNA1H
1100 ADAMTS12
1101 GRIA1

1102 MINK1
1103 SULT1E1
1104 CD80
1105 SSR1
1106 KDM4A
1107 TRGV3
1108 PGLYRP4
1109 TXNDC8
1110 TMPRSS11A
1111 GPR33
1112 TDO2
1113 SLC25A14
1114 FCGR3A
1115 PIGT
1116 AMY2A
1117 LARS2
1118 FKTN
1119 NDP
1120 GRP
1121 VSIG2
1122 ZAP70
1123 AMPD2
1124 SLC27A1
1125 SLC16A2
1126 PDF
1127 OR10H1
1128 FREM3
1129 PRSS45P
1130 ACP5
1131 GUCY1B2
1132 PTPMT1
1133 PLBD1
1134 PEBP4
1135 OR2H1
1136 LAMB4
1137 GREM1
1138 PLTP
1139 ASPRV1
1140 SCN1B
1141 AGTPBP1
1142 CPE
1143 DHRSX
1144 RELB
1145 PCSK7
1146 GABRR3
1147 NOCT
1148 TUBA8
1149 AMH

1150 OR5BS1P
1151 NOTCH3
1152 NUAK2
1153 PARP8
1154 CORIN
1155 IPP
1156 DEFA6
1157 XCR1
1158 APOBEC3G
1159 OR51E1
1160 PIM2
1161 MAP4K4
1162 TMEFF2
1163 LEFTY2
1164 PMCH
1165 IGKV2D-40
1166 VARS2
1167 CD164
1168 PYGB
1169 OR51H1
1170 CASP9
1171 PI4K2A
1172 NRDC
1173 TREM1
1174 SGK2
1175 THBS4
1176 COL3A1
1177 CYP39A1
1178 VAMP7
1179 ARSD
1180 KLHL33
1181 PRPS1L1
1182 UGT1A6
1183 ELSPBP1
1184 SHPK
1185 CASP4
1186 TNFRSF25
1187 OR4K5
1188 LTB
1189 NPC1
1190 CARTPT
1191 PA2G4
1192 GJB1
1193 TRIM10
1194 OPN1SW
1195 PDGFA
1196 COL8A1
1197 OR52N5

1198 BCL2L2-PABPN1
1199 GRIA2
1200 OR52D1
1201 LCN2
1202 OR51C1P
1203 SELP
1204 GRIK5
1205 PTPN11
1206 TRIM4
1207 CHST12
1208 C1QL1
1209 PROKR2
1210 MMP16
1211 KLHL4
1212 CDK20
1213 NPLOC4
1214 COL7A1
1215 FDFT1
1216 OR2AJ1
1217 UGT1A1
1218 CD19
1219 CDC42BPA
1220 P3H3
1221 ATP2B1
1222 LGALS8
1223 CBLN3
1224 KCND1
1225 VEGFD
1226 MAPK15
1227 ERVK-6
1228 SLC25A23
1229 OR7A10
1230 IVNS1ABP
1231 DCN
1232 SAE1
1233 SLC25A35
1234 OR5B3
1235 GDF3
1236 QPCT
1237 EEPD1
1238 TCN2
1239 SCP2
1240 ANGPT2
1241 HDAC9
1242 RNASE6
1243 DUSP15
1244 OR8D4
1245 EGLN2

1246 CALM3
1247 PRPF4B
1248 ABCE1
1249 RNF31
1250 FGF16
1251 ACVR2A
1252 SNAP25
1253 SERPINI1
1254 MORC1
1255 PARP2
1256 RGS12
1257 CD5L
1258 CTSF
1259 ENPP6
1260 BTK
1261 RFPL2
1262 OR11H13P
1263 TAC4
1264 SFTPB
1265 LRRN3
1266 STYX
1267 AGBL5
1268 PLK5
1269 PPIA
1270 CCL27
1271 PDE6H
1272 DTX2P1
1273 COQ8A
1274 MAP2K4
1275 THRB
1276 ENPP1
1277 PPIAL4D
1278 PSMG3
1279 PADI3
1280 OR8G2P
1281 UGT1A7
1282 SIGLEC5
1283 GABRD
1284 NBL1
1285 PRSS27
1286 CYP1A2
1287 EPB42
1288 BCR
1289 ATP2B3
1290 SCGB2A1
1291 KLHL35
1292 CSNK1A1
1293 ABCA2

1294 ATP1B2
1295 ODR4
1296 PGBD1
1297 SLC25A26
1298 EP300
1299 KDR
1300 GABBR1
1301 OR5M3
1302 RANBP2
1303 GIP
1304 FGF19
1305 CXCL5
1306 MMP8
1307 PTGS1
1308 KLK13
1309 MDK
1310 SCD5
1311 JAML
1312 CSNK1G3
1313 MTOR
1314 DUSP16
1315 IL1R1
1316 CD70
1317 OR7E31P
1318 OR5AQ1P
1319 GALP
1320 RXFP1
1321 TPCN2
1322 H3C10
1323 MCL1
1324 ERG
1325 CNGA4
1326 SLC16A14
1327 OR4N2
1328 CD58
1329 ELOVL3
1330 GPR63
1331 CA11
1332 PTPN18
1333 PPARG
1334 APOC3
1335 IGKV3-11
1336 CD93
1337 INPP5E
1338 NTSR1
1339 GABRA5
1340 FASLG
1341 OR51V1

1342 CFB
1343 KCNB1
1344 FXYD2
1345 OR5G1P
1346 SIGLEC1
1347 CELSR1
1348 ULK3
1349 DEFA1
1350 GSTA2
1351 EPGN
1352 HSD3B1
1353 HEBP1
1354 VIPR2
1355 DAND5
1356 DNASE1
1357 PPY
1358 CHIA
1359 ERN1
1360 WNT6
1361 OTOS
1362 INSL3
1363 SLC2A2
1364 OR2T29
1365 GPR34
1366 CD72
1367 WNK4
1368 RPS6KA5
1369 CXCL12
1370 MFSD3
1371 GLIPR1L1
1372 PTPN14
1373 AGPAT2
1374 CRHR1
1375 KCNK1
1376 CHRND
1377 NEK9
1378 ALCAM
1379 IFNL2
1380 NCR1
1381 PDK1
1382 RNASE4
1383 CDK5
1384 COL4A4
1385 HTR1B
1386 IFNA2
1387 OR4C13
1388 GPR142
1389 SIGLEC12

1390 MATN3
1391 CD7
1392 UQCRC2
1393 OR7C1
1394 TTR
1395 SIRPB1
1396 PRH1
1397 CD48
1398 OR7D2
1399 GMPR2
1400 PRPSAP2
1401 PDE6C
1402 DKK1
1403 FSD1L
1404 APLP1
1405 LTBP3
1406 DNASE1L1
1407 PPBP
1408 CNTN2
1409 PDE5A
1410 NOX5
1411 IL12A
1412 CACNA1B
1413 HLA-DRA
1414 PRKCB
1415 HHIP
1416 H4C9
1417 OR4F5
1418 FES
1419 KCNB2
1420 CA2
1421 UCP2
1422 CDK3
1423 BMP6
1424 RPS6KA3
1425 SLC47A1
1426 PPIG
1427 KAT2B
1428 SLAMF6
1429 HMGB2
1430 IARS1
1431 CPT1B
1432 RAET1G
1433 SULT1A2
1434 CYP1B1
1435 HPX
1436 RPS6KA6
1437 SCUBE1

1438 RARA
1439 FGF10
1440 OR52M1
1441 P2RX3
1442 C4A
1443 KBTBD11
1444 OR51A6P
1445 APOBEC3A
1446 GKN2
1447 CDK14
1448 QRFPR
1449 TEX101
1450 ADGRV1
1451 GRK6
1452 HRH4
1453 VWC2
1454 LAMC3
1455 RSPO2
1456 SFTPA2
1457 CBR3
1458 SLC5A8
1459 SAMD1
1460 NNMT
1461 NRTN
1462 LVRN
1463 SULT1A3
1464 SNRK
1465 PTPRN
1466 CXCL10
1467 NTN4
1468 SAA2
1469 DEFB114
1470 TSSK2
1471 ABCB10
1472 ADORA3
1473 TNFSF15
1474 PGA4
1475 AHCYL1
1476 CCN6
1477 PSMD14
1478 NELL2
1479 KCNH2
1480 UGT1A10
1481 USP7
1482 PRSS42P
1483 ATP1A1
1484 L3MBTL1
1485 CXCL2

1486 PRKAA2
1487 FCRL3
1488 TUBA1A
1489 KDM5C
1490 GPR157
1491 CYP2C18
1492 PARP10
1493 FDCSP
1494 SLC25A44
1495 TUBB8B
1496 SEMA3D
1497 PRLH
1498 UCN2
1499 OR5A2
1500 NR0B1
1501 TXNRD3
1502 OR9A4
1503 TXK
1504 SENP8
1505 MFSD5
1506 SCYL2
1507 CYP4X1
1508 PGC
1509 LIG1
1510 NAGA
1511 SI
1512 CAPZA1
1513 KCNK15
1514 OR10A5
1515 UCN3
1516 OVCH1
1517 CLPS
1518 LAG3
1519 CPM
1520 ADCY10
1521 MAP3K9
1522 TBXAS1
1523 IL20RB
1524 PSMB10
1525 RAMP2
1526 NAALAD2
1527 DLL4
1528 TNIK
1529 PKN1
1530 EPHA5
1531 BTN3A2
1532 F2RL3
1533 CACNA2D4

1534 ITIH4
1535 TNXB
1536 IL18R1
1537 CCR10
1538 BRD4
1539 TMLHE
1540 TOP2A
1541 CYP4F2
1542 NFKBIA
1543 H1-2
1544 RXFP4
1545 FCN2
1546 MAN2B2
1547 TRPM4
1548 SLC1A3
1549 GLP2R
1550 OR10AD1
1551 MMP23A
1552 HCAR1
1553 MRGPRG
1554 ADRA2B
1555 ANGPTL7
1556 CLK4
1557 SLC5A7
1558 ADGRA2
1559 IL26
1560 RASGRP3
1561 HDC
1562 PGLYRP1
1563 MTCH2
1564 PPARA
1565 CYP11B1
1566 RESP18
1567 CA4
1568 THOP1
1569 TXNDC15
1570 A1BG
1571 ACVRL1
1572 KCNS2
1573 ECRG4
1574 SPRYD4
1575 KAZALD1
1576 SLC22A8
1577 SCN8A
1578 STK24
1579 ADA2
1580 PDE4B
1581 H3-3A

1582 EGFR
1583 BAD
1584 CROT
1585 CDK18
1586 MGMT
1587 IGHV1-2
1588 PKD2
1589 NR2C1
1590 SLC34A2
1591 PSMB6
1592 HPGDS
1593 EDA
1594 HTR3C
1595 TMIGD2
1596 LRRK2
1597 KBTBD12
1598 PBK
1599 GUSBP1
1600 GPR52
1601 TENM1
1602 TSPEAR
1603 OR52W1
1604 KLRD1
1605 DNAJC16
1606 FST
1607 GSTP1
1608 HPN
1609 CFH
1610 ADAMTS20
1611 ERAP1
1612 ADAM30
1613 OR51B2
1614 PKHD1
1615 KCNK13
1616 TSTD1
1617 AMPD3
1618 FUT1
1619 SDC2
1620 MATN4
1621 SLC28A2
1622 BIRC2
1623 NPR3
1624 NTN1
1625 RHBG
1626 ASIP
1627 STAT5B
1628 GBE1
1629 FABP4

1630 KLK9
1631 AHCY
1632 CAMK2A
1633 FLRT2
1634 BACE1
1635 DHRS12
1636 OR2T5
1637 ALDH9A1
1638 CA7
1639 ABCG2
1640 CYP17A1
1641 SPP2
1642 OR10G7
1643 DUSP22
1644 TFRC
1645 LAMP1
1646 RAB9A
1647 VSIG4
1648 BRAF
1649 OR4C12
1650 LILRB1
1651 SLC37A2
1652 SPRYD3
1653 ENPEP
1654 ALPG
1655 SLC1A1
1656 CBR4
1657 TNFRSF10A
1658 MFAP1
1659 TAP1
1660 GPR135
1661 CXCR6
1662 CPA2
1663 COLQ
1664 TRIB1
1665 POLA1
1666 PRF1
1667 WNT2B
1668 MLH1
1669 M6PR
1670 ESR2
1671 KLHL1
1672 SCNN1B
1673 APCS
1674 IMPDH1
1675 LAMA2
1676 BCL2L10
1677 TMEM94

1678 CYP2C9
1679 OR5L2
1680 RFPL1
1681 CCL7
1682 PDIA2
1683 MRGPRX4
1684 IFNK
1685 HLA-A
1686 YWHAG
1687 IL17C
1688 NECTIN1
1689 H4C11
1690 OCRL
1691 OR4S2
1692 PLK2
1693 POGLUT1
1694 APOC1
1695 SLC2A8
1696 MFSD10
1697 PRKCD
1698 SLC3A1
1699 ERAP2
1700 FCRL5
1701 IDE
1702 TAB2
1703 GPNMB
1704 OR10G2
1705 KCNQ2
1706 THBD
1707 OR51N1P
1708 CASQ2
1709 CALCB
1710 GPR101
1711 GPRC5D
1712 OR2T10
1713 MGST2
1714 TOP1MT
1715 KCNA5
1716 MAPKAPK2
1717 SERPINB4
1718 HTR1E
1719 SLC4A3
1720 CCL15-CCL14
1721 BRD3
1722 CDNF
1723 OR5H2
1724 C1S
1725 HHAT

1726 OR4H12P
1727 IDS
1728 S100A9
1729 LILRA1
1730 OR1K1
1731 GMPR
1732 CATSPER2
1733 PRSS8
1734 RAPGEF4
1735 FPR2
1736 CTSG
1737 OR2AG1
1738 OR2D2
1739 OR9K1P
1740 FCRL6
1741 HSD17B13
1742 NQO2
1743 OR4P1P
1744 SLC43A2
1745 PCDHA2
1746 CHAT
1747 MAS1
1748 ART4
1749 TPBG
1750 TRIM35
1751 KLHL20
1752 ALDH3A2
1753 APOD
1754 CD274
1755 MAPT
1756 PLD2
1757 PSMA1
1758 FUCA1
1759 COL6A2
1760 ADAM15
1761 MMRN2
1762 ELN
1763 IL3RA
1764 CA10
1765 SENP6
1766 PSG2
1767 NOX1
1768 PAK1
1769 DRD2
1770 UGT1A8
1771 CYP2A6
1772 OR10S1
1773 OR5P1P

1774 PRKACA
1775 ADGRE5
1776 PCSK2
1777 ODC1
1778 MCOLN3
1779 GPX1
1780 CPB1
1781 RARB
1782 MAP3K6
1783 PLCB1
1784 IL2RG
1785 REG1B
1786 AMPD1
1787 SEMG2
1788 DHRS2
1789 CXCR2
1790 APEX2
1791 CASP1
1792 SLC15A2
1793 TNFRSF10D
1794 CYP21A2
1795 WARS2
1796 KIRREL2
1797 CSNK2A2
1798 GGT6
1799 TPSAB1
1800 GABRR1
1801 FBLN7
1802 CRISP2
1803 MTMR7
1804 NPBWR2
1805 SLC3A2
1806 SLC9A7
1807 SLC25A37
1808 OR10D3
1809 MFSD14A
1810 FMO2
1811 ATIC
1812 DMBT1L1
1813 CA8
1814 PLSCR3
1815 MMP11
1816 SERPINB3
1817 IL11RA
1818 DECR1
1819 SPACA5B
1820 HNMT
1821 NFATC1

1822 IMPG2
1823 HHATL
1824 PAPPA2
1825 TECTA
1826 ACY1
1827 KLK3
1828 WFIKKN2
1829 S100B
1830 OR7A15P
1831 OR1E2
1832 RELT
1833 UGT8
1834 CD207
1835 HDAC7
1836 NPFF
1837 AVPR2
1838 MYLK3
1839 MGAM2
1840 OXSR1
1841 SLC16A11
1842 EPHA1
1843 SLC9C2
1844 IL13RA1
1845 COX7B
1846 FUCA2
1847 GGTLC2
1848 GZMM
1849 TUBB1
1850 IGLV3-16
1851 GP1BB
1852 EIF4A1
1853 TAAR1
1854 CRISP1
1855 PDCD4
1856 SLC18A3
1857 PODNL1
1858 ZP1
1859 ARSL
1860 OMG
1861 LRG1
1862 LAMA3
1863 TIMP4
1864 CST6
1865 HSP90AA2P
1866 IL6ST
1867 ATP1B3
1868 BDNF
1869 TXNL1

1870 NLRP3
1871 COQ6
1872 GRPR
1873 ENPP5
1874 OR1J4
1875 FGFR2
1876 DUT
1877 TH
1878 PRKACG
1879 FMO3
1880 SLC25A51
1881 MPST
1882 DMBT1
1883 SMS
1884 GABRA6
1885 RNASE2
1886 MANF
1887 MFSD12
1888 MME
1889 OR5T3
1890 CACNA1I
1891 OR8J3
1892 DNMT1
1893 OR14A16
1894 ATP1A4
1895 PTPRU
1896 PSKH2
1897 ADAMTS18
1898 PLA2G2E
1899 EFNB1
1900 SULT4A1
1901 PRDX4
1902 OR5M1
1903 CYP4F3
1904 NDFIP1
1905 RDH10
1906 ABCA13
1907 TRIM36
1908 HTR2A
1909 HRH1
1910 PGA3
1911 NOD2
1912 MAP2K3
1913 DERL1
1914 BLMH
1915 PROCR
1916 TRPM1
1917 DPEP1

1918 CRH
1919 DSCAM
1920 RORA
1921 HLA-DOB
1922 CGB3
1923 GALC
1924 OR6B1
1925 LAMA5
1926 KHDRBS1
1927 TAB1
1928 NMT1
1929 TUBE1
1930 RB1
1931 CELA2A
1932 STK32B
1933 SLC16A10
1934 SLC22A13
1935 STRADB
1936 CXCL13
1937 THBS2
1938 KNG1
1939 OR4P4
1940 NUP153
1941 CCL21
1942 QSOX1
1943 CD59
1944 SLC17A5
1945 B2M
1946 MAPK1
1947 TIPARP
1948 CNOT6L
1949 LIMK1
1950 JUN
1951 COL5A1
1952 PDZD2
1953 PGF
1954 CYP2A13
1955 OR7E8P
1956 CAPN5
1957 CYP51A1
1958 RYK
1959 OR10D4P
1960 IGHV1-46
1961 CKLF
1962 SERPINF2
1963 GBAP1
1964 CRB2
1965 CXCL14

1966 TAAR9
1967 ADGRB1
1968 KCNK18
1969 OR4C3
1970 SLC16A1
1971 PDE7B
1972 IL17D
1973 BCL2L14
1974 PCSK1N
1975 OR2G2
1976 MFSD4B
1977 SERPINB1
1978 KCNA3
1979 OR52K1
1980 SLC25A25
1981 DRAXIN
1982 NUAK1
1983 CD163L1
1984 OR2T2
1985 OR8B4
1986 OR6Q1
1987 CSNK1G2
1988 SPINK5
1989 SLC22A3
1990 SLAMF8
1991 TXNRD1
1992 MFSD11
1993 CSH1
1994 MARK1
1995 ABCA8
1996 COL10A1
1997 ANGEL1
1998 HSD17B1
1999 PRSS1
2000 GPR83
2001 STATH
2002 MAP4K3
2003 SLC9A4
2004 FGF22
2005 MMP1
2006 KALRN
2007 TNFRSF19
2008 OR5B21
2009 C1QL2
2010 CDH1
2011 OR5L1
2012 NGFR
2013 SYNJ1

2014 PLCH1
2015 SLC25A43
2016 APRT
2017 SLC39A5
2018 SLC37A3
2019 SDR16C5
2020 OGA
2021 ACVR1
2022 CYP2G1P
2023 FBLN5
2024 COL9A3
2025 KLK10
2026 TF
2027 HTN1
2028 PGR
2029 NFATC2
2030 OR5C1
2031 SLC6A8
2032 LTC4S
2033 NR1D1
2034 ADCY6
2035 ITPR2
2036 SLC12A9
2037 PMS2P1
2038 AMTN
2039 DEFB113
2040 CPD
2041 UMOD
2042 SCRG1
2043 IGKV1D-16
2044 CSF3
2045 FFAR3
2046 SERPINE3
2047 IGHD
2048 IGLC2
2049 CCN3
2050 ADAMTS10
2051 LYPD6
2052 OR10AC1
2053 TKT
2054 AKT1
2055 MFSD14B
2056 NPY2R
2057 H3C12
2058 GRM6
2059 ILK
2060 VGF
2061 SLC5A2

2062 ADAMTS14
2063 CGREF1
2064 FPR3
2065 OR1D4
2066 SLC9A1
2067 PREP
2068 ZC3HAV1
2069 HASPIN
2070 PDXK
2071 BIRC5
2072 PNCK
2073 ADH4
2074 SARAF
2075 CATSPER1
2076 IL31
2077 PROKR1
2078 TAOK1
2079 PMP22
2080 PSMB9
2081 ADH5
2082 WNT9B
2083 IGKV2D-29
2084 DOT1L
2085 HCRT
2086 PDE3A
2087 FLT1
2088 METAP2
2089 AKR1C6P
2090 IGKV4-1
2091 ADIPOQ
2092 S100A7
2093 PKD2L1
2094 SLC4A9
2095 ARAF
2096 PLA2G2D
2097 ATP4A
2098 TGM2
2099 UCHL3
2100 SERPINE2
2101 GLP1R
2102 PKN2
2103 OR1E1
2104 HCAR3
2105 PLPBP
2106 CHST11
2107 DCPS
2108 GSTCD
2109 DIO1

2110 KLHL12
2111 OR14A2
2112 FPR1
2113 GPR183
2114 NRK
2115 SELENOS
2116 FAS
2117 KCNK6
2118 TRIB3
2119 PDIA6
2120 IMPA2
2121 GPC6
2122 NOTCH2NLA
2123 VARS1
2124 RSPO1
2125 BICD1
2126 SLC12A6
2127 SLC2A4
2128 ADCY7
2129 TFF2
2130 CD1B
2131 CPXM1
2132 PLK1
2133 ERN2
2134 CDKL5
2135 SRGN
2136 HAPLN4
2137 MBOAT2
2138 ADAM2
2139 METRN
2140 TUFT1
2141 CDH13
2142 SLC9A8
2143 CSN2
2144 SLC6A6
2145 LAMB3
2146 ITGB1
2147 CHID1
2148 DHRS7C
2149 CPZ
2150 APOM
2151 GPR61
2152 ANG
2153 CLEC11A
2154 PYCARD
2155 PTPN7
2156 SEMA4D
2157 PLCD1

2158 CD200
2159 ADK
2160 SPNS3
2161 PI4KAP2
2162 HSP90AB1
2163 OR7E5P
2164 MICAL2
2165 ADAMTS9
2166 MAPK14
2167 CCR9
2168 ABCC4
2169 CACHD1
2170 SV2A
2171 GAN
2172 MFAP4
2173 SLC2A9
2174 OSM
2175 DEFB105A
2176 LY6K
2177 CD244
2178 OR7A17
2179 ATP2B2
2180 SPOCK2
2181 IL17RE
2182 CPXM2
2183 CSN3
2184 CHST4
2185 ASPN
2186 CRISP3
2187 TSSK1B
2188 MFSD2A
2189 SLC25A12
2190 TUB
2191 TSPO
2192 LIPN
2193 KCNJ10
2194 ASH2L
2195 TYMP
2196 HRH2
2197 NR1H2
2198 STK32C
2199 UPP1
2200 GPR152
2201 TSTD2
2202 OR52K2
2203 FAM20C
2204 CYP7A1
2205 MELK

2206 IL17RC
2207 TRIM62
2208 LAP3
2209 KCNK4
2210 CES4A
2211 SCN3A
2212 SLC25A36
2213 PXK
2214 OR8K3
2215 CR1
2216 OR6C76
2217 SLC16A3
2218 PDCD1
2219 NR1D2
2220 SDR42E1
2221 PAM
2222 LAMB1
2223 NPFFR2
2224 CYP4F22
2225 ESAM
2226 POMK
2227 HIPK4
2228 OR4F21
2229 CDK17
2230 SERPINA12
2231 TM2D1
2232 SLC4A1
2233 ABCD1
2234 NRXN2
2235 AKR7L
2236 MSR1
2237 FGF4
2238 ENPP7
2239 MUC15
2240 OR51G1
2241 TTN
2242 NFAT5
2243 CCNH
2244 LCAT
2245 SSC4D
2246 OR3A3
2247 AURKA
2248 CD300LB
2249 GRK7
2250 CLEC3B
2251 G6PC1
2252 IGLV2-23
2253 PXDNL

2254 PCDHB10
2255 SCGB1A1
2256 ADGRG1
2257 DEFB4A
2258 OR13C6P
2259 OR2A7
2260 SLC12A2
2261 EFNA4
2262 P2RX7
2263 KDM2A
2264 SEMA7A
2265 DCLK2
2266 SSTR1
2267 PTPRA
2268 HCK
2269 HSPB1
2270 MBOAT7
2271 OR51A2
2272 OR2W3
2273 ENTPD1
2274 OR5B12
2275 ALDH3B1
2276 PROCA1
2277 TGM1
2278 HABP4
2279 AMELY
2280 VIP
2281 GPC4
2282 SDC4
2283 CBLN4
2284 SEMA3A
2285 CHRNB3
2286 OR10K2
2287 MRGPRX1
2288 RAB35
2289 FPGS
2290 TAFA5
2291 SLITRK4
2292 ADAM7
2293 SLAMF7
2294 TRPC4
2295 NEK4
2296 BMP3
2297 ABCB1
2298 CELA1
2299 PROS1
2300 KCNJ12
2301 DEFB112

2302 VWF
2303 KCNJ14
2304 DPP6
2305 TAAR3P
2306 GBA
2307 OMD
2308 TNFRSF10C
2309 ATF1
2310 CNTNAP2
2311 OR6C68
2312 ACTN4
2313 OR9I1
2314 CES3
2315 GPR65
2316 SCNN1D
2317 ERVK-11
2318 NLGN3
2319 OR2L8
2320 H4C5
2321 OR1A1
2322 OR4D5
2323 MARK3
2324 GAS6
2325 CCL18
2326 P2RY8
2327 CLCNKA
2328 CDC25C
2329 FN1
2330 F5
2331 FCER2
2332 SLC15A4
2333 LDLR
2334 P4HTM
2335 EVI2B
2336 CCDC3
2337 SEMA4B
2338 FGF8
2339 MAP3K7
2340 GPX3
2341 MAPK11
2342 CDK11B
2343 TAAR2
2344 PCDH15
2345 CHRD
2346 COL14A1
2347 TNFSF13
2348 CLCA4
2349 CDH2

2350 PI4KB
2351 ADRA2C
2352 CALCA
2353 FCRL1
2354 NRXN3
2355 GREM2
2356 ADGRL4
2357 GRN
2358 TNFRSF11A
2359 SPRYD7
2360 EPYC
2361 GYPC
2362 KLHL36
2363 CD5
2364 CNGB3
2365 IFNL3
2366 LEPR
2367 OR6P1
2368 HSPH1
2369 MASP1
2370 SEMG1
2371 OR4K17
2372 RABGGTA
2373 VASH1
2374 GP9
2375 MAK
2376 PTPN20
2377 H4-16
2378 MT-ND4
2379 ADGRL2
2380 MTRR
2381 NLGN4Y
2382 TAS2R16
2383 PLA2G2C
2384 GGCX
2385 ENDOD1
2386 CTNNB1
2387 SPINK9
2388 KCND2
2389 NUCB1
2390 CCR6
2391 H4C15
2392 ARNT
2393 LY75
2394 OLR1
2395 FGF1
2396 KDM4C
2397 KLHL17

2398 TEK
2399 NCOA3
2400 NR2C2
2401 GPR15
2402 PTGDS
2403 CCKAR
2404 MDM4
2405 NODAL
2406 USH2A
2407 HNRNPUL2
2408 OR51M1
2409 OTOL1
2410 TK2
2411 ABCC3
2412 DHRS13
2413 AGTR1
2414 CASP10
2415 ALDH1A3
2416 SLC18B1
2417 PTP4A2
2418 BUB1B
2419 OR2M2
2420 GPR4
2421 ATP2A3
2422 H4C4
2423 IKBKE
2424 TAS1R1
2425 PAEP
2426 FCN3
2427 DPP7
2428 TGFA
2429 CSF3R
2430 OR2L13
2431 IGLV1-36
2432 PDGFRB
2433 SMR3A
2434 FGF3
2435 PLOD1
2436 PTPN2
2437 OR1I1
2438 ABCB8
2439 ESRRG
2440 ALOX5
2441 STAT1
2442 DUSP29
2443 OPRL1
2444 KCNJ5
2445 LAMC1

2446 H3C15
2447 SLC4A10
2448 PSMA6
2449 PIP
2450 CSH2
2451 PRSS58
2452 BPIFB3
2453 LTA4H
2454 GABRG2
2455 OR10A4
2456 NR3C1
2457 STK17A
2458 FARS2
2459 SLC6A16
2460 CYP1A1
2461 GPR173
2462 AGBL4
2463 COL27A1
2464 ITGB3
2465 CPT1A
2466 DUSP19
2467 GSTA5
2468 AMY1C
2469 TRIM68
2470 PARP3
2471 OR2A25
2472 GPX7
2473 PTP4A1
2474 BCL2L13
2475 OR10H3
2476 MAPK8
2477 PMS2P4
2478 ABCC2
2479 SEMA3F
2480 CTDSP1
2481 KARS1
2482 KCNJ2
2483 SUOX
2484 IFI30
2485 IGFBP5
2486 MPZL3
2487 OR4A21P
2488 CLCF1
2489 TYK2
2490 CD38
2491 RRM2
2492 OR2A5
2493 DKKL1

2494 FAP
2495 NTSR2
2496 EDN2
2497 NIM1K
2498 CCK
2499 OR7A2P
2500 OR7E90P
2501 SLC25A16
2502 RFPL4A
2503 SQLE
2504 EPX
2505 OR6Y1
2506 H3C11
2507 RNASEL
2508 SCGB1C1
2509 CYP4A11
2510 HCRTR2
2511 TRIM34
2512 PSG1
2513 PSKH1
2514 PPIE
2515 DEFB106B
2516 MAP3K13
2517 MCHR2
2518 NRG3
2519 GHSR
2520 XPNPEP3
2521 NEIL3
2522 TRPM8
2523 FBXO45
2524 FCGBP
2525 TIMP2
2526 IL33
2527 CREG2
2528 PLA2G15
2529 ITK
2530 CRP
2531 ECM2
2532 CPB2
2533 SLC25A11
2534 SLC6A19
2535 SOGA1
2536 GGTLC1
2537 CDC42BPG
2538 TMPRSS9
2539 HSD17B7
2540 FCGR2A
2541 DPP9

2542 SLC12A1
2543 EPDR1
2544 OR51B6
2545 P2RY1
2546 PPARD
2547 SAA4
2548 PIK3CG
2549 GSS
2550 H4C13
2551 BPIFB2
2552 ICAM3
2553 TAFA4
2554 MTMR2
2555 TDP1
2556 AMOT
2557 MPZ
2558 TG
2559 KL
2560 LILRB5
2561 MFNG
2562 FUS
2563 KCNC3
2564 NALCN
2565 TRIM47
2566 TEX14
2567 MMP14
2568 ACP2
2569 TNFRSF13C
2570 DEFB107A
2571 TGFB2
2572 LINGO2
2573 SLC6A9
2574 BPI
2575 CXCL6
2576 LIPG
2577 DMD
2578 COL4A5
2579 BSPRY
2580 TNNI3
2581 VWA3A
2582 PATE2
2583 MC2R
2584 H4C12
2585 CLCN5
2586 CNTNAP3
2587 PRKDC
2588 PRKCA
2589 OR5D13

2590 SELE
2591 IL6
2592 P4HB
2593 OR6K2
2594 IFNA6
2595 MIPEP
2596 IL23A
2597 DHH
2598 FOLR3
2599 HILPDA
2600 ERMAP
2601 LTB4R2
2602 CACNG8
2603 OR5B17
2604 IGLV4-69
2605 NCR3
2606 ALDH16A1
2607 CDKL4
2608 CPA3
2609 DUSP11
2610 TMX4
2611 KRT18
2612 PLGLB1
2613 H6PD
2614 PTPN12
2615 DNASE1L3
2616 CPQ
2617 PIM1
2618 ADAMTSL4
2619 GPC5
2620 DEFA5
2621 SERPINB8
2622 SACS
2623 OR5A1
2624 NECTIN3
2625 PTPRC
2626 CYP24A1
2627 SLC25A32
2628 OR13C8
2629 APP
2630 PDE11A
2631 ADAMTS19
2632 FCGR3B
2633 MMP19
2634 CTSZ
2635 SCN9A
2636 GRIN3A
2637 MCOLN2

2638 IGKV1D-42
2639 BTNL8
2640 LPAR1
2641 GPRC5C
2642 TGFBI
2643 TRIM21
2644 SSH2
2645 EPHB3
2646 FGFRL1
2647 NEK11
2648 CAMP
2649 FGF9
2650 P2RX4
2651 POTEI
2652 DUSP10
2653 SLC10A6
2654 RPTN
2655 TRIM64
2656 PTK7
2657 PLSCR1
2658 SPAM1
2659 TBK1
2660 TNFRSF18
2661 KCNH3
2662 FLVCR1
2663 TRPV4
2664 KCNN3
2665 ADGRA3
2666 MAS1L
2667 CLUL1
2668 DHFR
2669 IL25
2670 MPL
2671 KERA
2672 ADGRE2
2673 APLN
2674 OR4A47
2675 EDN1
2676 PTPN1
2677 SLC25A47
2678 ARSJ
2679 CD79B
2680 CTRB1
2681 RDH12
2682 LRTOMT
2683 FNTB
2684 KCNQ5
2685 TRRAP

2686 BCL2
2687 GCK
2688 ERBIN
2689 C2
2690 SSTR2
2691 OR56B4
2692 PLA2R1
2693 SIRT2
2694 STK16
2695 IGHG2
2696 NPB
2697 EGLN1
2698 C1QTNF9B
2699 KIR2DL4
2700 CST5
2701 P2RY13
2702 TIMP3
2703 OR7E85P
2704 COL4A1
2705 DECR2
2706 VASN
2707 LAMA4
2708 FASN
2709 TULP3
2710 PF4V1
2711 SECTM1
2712 APH1A
2713 SHISA2
2714 FGF6
2715 TRIM72
2716 MALT1
2717 PKD2L2
2718 SCT
2719 STK32A
2720 HTR3D
2721 GDF5
2722 OR52E2
2723 ITLN1
2724 OTOA
2725 ST6GAL1
2726 IFNL1
2727 CFI
2728 IGHA2
2729 XCL2
2730 PORCN
2731 SCN5A
2732 SLC22A20P
2733 SUMF1

2734 ECE1
2735 LTBR
2736 CRIM1
2737 PRR4
2738 GPR119
2739 SENP7
2740 OR7E87P
2741 BMP7
2742 PTPN22
2743 MRGPRD
2744 FFAR2
2745 DNPEP
2746 SCGN
2747 SLC25A10
2748 DGAT1
2749 ACVR1C
2750 MAPK9
2751 ATP12A
2752 MMP21
2753 LACRT
2754 SGSH
2755 HERC1
2756 AGXT2
2757 CES1
2758 IL27
2759 MCHR1
2760 PDIK1L
2761 PRIM1
2762 ITGA4
2763 TYRL
2764 TMEM25
2765 S1PR5
2766 ALDH2
2767 LYPD5
2768 PLA1A
2769 PTPDC1
2770 BDH1
2771 HSD17B11
2772 TRAP1
2773 FMO5
2774 IGFBP6
2775 LAMA1
2776 HLA-G
2777 SLC17A1
2778 IL2RA
2779 GPR3
2780 ANGPTL4
2781 CDCP2

2782 GPRC6A
2783 HTRA1
2784 ST3GAL2
2785 MID1
2786 TAB3
2787 PDE4D
2788 NDUFA4
2789 BPHL
2790 RBKS
2791 APOB
2792 PTPRF
2793 TLN1
2794 RYR1
2795 GPR22
2796 CATSPER3
2797 SLC25A17
2798 TLR1
2799 OR7D1P
2800 PIK3C3
2801 CHADL
2802 ADAM8
2803 FBN1
2804 PRB4
2805 ISG15
2806 PRKY
2807 GLRA3
2808 SLC7A1
2809 GPR26
2810 TACR2
2811 ITGAV
2812 OR51A4
2813 AOPEP
2814 PI15
2815 ALPP
2816 SPEG
2817 PPOX
2818 PAMR1
2819 CEP41
2820 IGKV3-20
2821 XPNPEP2
2822 ADGRL3
2823 RIPK4
2824 PRSS35
2825 P2RX6
2826 OR2S2
2827 AGER
2828 HRC
2829 IGLV10-54

2830 OR2T12
2831 RPN1
2832 MAP3K20
2833 CSNK1D
2834 HYAL1
2835 MUSK
2836 SLC12A4
2837 MLNR
2838 LTBP4
2839 OR10P1
2840 DPT
2841 ICAM2
2842 PRSS3
2843 OR5BB1P
2844 CCL24
2845 CAMK2B
2846 NPS
2847 COL22A1
2848 OR2M4
2849 SEMA6B
2850 MEFV
2851 IFNA7
2852 DNMT3B
2853 CDK5R1
2854 FCGR2C
2855 PRSS29P
2856 PKMYT1
2857 RXFP3
2858 BOC
2859 FSD2
2860 MGP
2861 IFNA4
2862 SLC22A2
2863 CACNA2D1
2864 SLC17A4
2865 NLGN2
2866 MFSD6
2867 RAD51
2868 SOD3
2869 FKBP5
2870 CHRDL1
2871 DAG1
2872 OR2T27
2873 NID1
2874 BMP8A
2875 GPR148
2876 RXRA
2877 CDC42BPB

2878 TNFRSF12A
2879 ACTN1
2880 DSPP
2881 BMPR1B
2882 PRG4
2883 CACNG4
2884 MLKL
2885 SVOPL
2886 CAMKV
2887 IGIP
2888 LRRC4B
2889 GNAS
2890 VIM
2891 TRPV3
2892 TGFB1
2893 CHEK1
2894 SLC15A1
2895 DUSP3
2896 PROZ
2897 GALE
2898 PRH2
2899 OR7E91P
2900 AKR1C1
2901 GPR42
2902 OR4A16
2903 IFNA14
2904 CYB5AP3
2905 CACNA1S
2906 BPIFB1
2907 GAST
2908 HEPH
2909 GAD1
2910 MFSD4A
2911 NTNG1
2912 DRD5
2913 MAP3K2
2914 PROK2
2915 NTF3
2916 HSPG2
2917 TRPC7
2918 ZACN
2919 ALDH3A1
2920 GALR3
2921 STK35
2922 HTR4
2923 DUSP2
2924 ADGRL1
2925 TUBA1B

2926 TMPRSS3
2927 STAT5A
2928 COL6A1
2929 NMUR2
2930 RDH16
2931 LRRC55
2932 SLC7A9
2933 SPINT2
2934 LRIG3
2935 MPO
2936 HPSE
2937 NAPSB
2938 FABP1
2939 OR2G6
2940 S100A13
2941 GPR182
2942 GCLC
2943 PLCZ1
2944 BRS3
2945 CPS1
2946 IL1RL2
2947 NCSTN
2948 VN1R1
2949 ALDH1A2
2950 FKBP6
2951 PCOLCE
2952 PRKCZ
2953 OR52B6
2954 MIF
2955 LY9
2956 KLK6
2957 KIR3DL2
2958 MST1L
2959 IDSP1
2960 CYP4F8
2961 NPPA
2962 NPY
2963 GPR32
2964 ENOX2
2965 PRSS53
2966 MTCH1
2967 GPR158
2968 C7
2969 HKDC1
2970 PPIL2
2971 DEFA3
2972 SMR3B
2973 CDC25A

2974 GRM1
2975 MAP2K7
2976 ABCA1
2977 CACNA1E
2978 ANGPTL6
2979 ABCB6
2980 PDGFRL
2981 OR7C2
2982 IL5RA
2983 GFPT1
2984 SLC45A3
2985 NR2F1
2986 SLCO4A1
2987 TRIM5
2988 UGCG
2989 OR6A2
2990 OR7A5
2991 CIT
2992 EPHB4
2993 LPA
2994 GPBAR1
2995 DNMT3A
2996 FOLH1
2997 OPN3
2998 TPSD1
2999 JAM2
3000 SLC44A4
3001 ITGB5
3002 TLR5
3003 GABRB3
3004 GABRG3
3005 TM4SF1
3006 GP2
3007 IL18BP
3008 SLC13A2
3009 WNT7A
3010 PRKCE
3011 DKK3
3012 OR6C4
3013 IBSP
3014 RRM1
3015 EEF2
3016 ADAM3A
3017 GRK2
3018 COL12A1
3019 RTN4RL1
3020 GPR162
3021 DYRK2

3022 LPL
3023 HAO1
3024 ADAMTS17
3025 WEE1
3026 SPSB2
3027 TEX13A
3028 CCR3
3029 ALOX12
3030 PRMT3
3031 TAFA1
3032 NPM1
3033 CXCL11
3034 GCG
3035 TRIM67
3036 PLGLB2
3037 LYN
3038 HTN3
3039 IGKV3-15
3040 ADAMTS15
3041 CFTR
3042 PSCA
3043 JCHAIN
3044 SPSB1
3045 PSMB8
3046 GANC
3047 CCN1
3048 ULBP1
3049 TNFSF13B
3050 H1-1
3051 OR52E8
3052 VAMP8
3053 YARS2
3054 SLC10A5
3055 CILP2
3056 AKR7A2
3057 FGF20
3058 OR6C75
3059 SULT2A1
3060 PLA2G2A
3061 RDH11
3062 H4C14
3063 GPR21
3064 FGA
3065 BAK1P1
3066 GRK5
3067 MTRNR2L4
3068 OR4G2P
3069 MAP3K11

3070 DUSP4
3071 IGKV5-2
3072 TNNC1
3073 FYN
3074 BGN
3075 UGT1A5
3076 DPYD
3077 WNT8B
3078 ABCF1
3079 ABCA3
3080 RPS6KL1
3081 OR9Q1
3082 GSTO2
3083 CFP
3084 UQCRC1
3085 PTK2B
3086 ABCA9
3087 CAMK2D
3088 AQP1
3089 GSTT2B
3090 ADAM21
3091 PVR
3092 DUSP13
3093 DEFB106A
3094 OR13C5
3095 DPP4
3096 PLXNC1
3097 CYP2A7
3098 TPTE
3099 IL10
3100 KCNK7
3101 CCL17
3102 DDX1
3103 SLC25A22
3104 LTB4R
3105 SLC15A3
3106 FMO1
3107 SLC25A28
3108 MST1
3109 WNT3A
3110 EPOR
3111 GAD2
3112 AURKB
3113 SERPINA10
3114 BCKDK
3115 OR2M5
3116 FAM3B
3117 RGS4

3118 IARS2
3119 CAPN12
3120 AGRN
3121 OR7E97P
3122 SLC2A11
3123 HMGCS1
3124 FGR
3125 MAP3K5
3126 OR1S2
3127 SCYL3
3128 UTS2B
3129 GPR50
3130 F7
3131 HTR5A
3132 TFPI2
3133 EPHX1
3134 MAPKAPK5
3135 F12
3136 CYP2C8
3137 LGALS9
3138 CD46
3139 IGLV3-22
3140 SOD1
3141 CAPN13
3142 CXCL3
3143 ADGRE4P
3144 TNC
3145 OR2C1
3146 GRK1
3147 SFRP1
3148 SLCO2A1
3149 TUBG2
3150 RARS2
3151 ALOX15
3152 IL19
3153 NPTN
3154 BRDT
3155 G6PD
3156 OR8K1
3157 CLDN18
3158 PDE6A
3159 ITGAD
3160 H1-0
3161 EREG
3162 KLHL40
3163 KCNS3
3164 SCPEP1
3165 GPR25

3166 CD47
3167 STAT6
3168 NELL1
3169 OR1F12P
3170 ST3GAL3
3171 COL20A1
3172 GPX5
3173 MCAM
3174 TRAC
3175 SHBG
3176 SCNN1A
3177 GPX2
3178 SIK2
3179 PCSK6
3180 FAM234A
3181 PTPRS
3182 PLCB4
3183 HTR7
3184 IGLV7-43
3185 KCNJ16
3186 SCUBE3
3187 CCL14
3188 ANPEP
3189 OR6C1
3190 AGR2
3191 OR2A2
3192 GNRH1
3193 OR5T1
3194 TRPM6
3195 SLC17A3
3196 RNASET2
3197 KLHL9
3198 NEGR1
3199 ADAM10
3200 KHK
3201 NOD1
3202 SPON2
3203 LGI1
3204 PRSS21
3205 PTGIS
3206 LTK
3207 SULT1C2
3208 OR2T11
3209 SMOC2
3210 OR52E4
3211 SLC22A17
3212 CNP
3213 HMGCS2

3214 MAN2A1
3215 TRIM50
3216 ADGRF3
3217 GAA
3218 OR14I1
3219 CRISPLD2
3220 MMEL1
3221 MYDGF
3222 DPP8
3223 SLC2A12
3224 OAS1
3225 PLCD3
3226 OR56B3P
3227 SLC47A2
3228 MC3R
3229 VKORC1
3230 EPHA2
3231 GPR75
3232 COLEC10
3233 APOE
3234 CYB5R4
3235 OR51E2
3236 EMILIN2
3237 TRIM26
3238 SRD5A3
3239 TSG101
3240 PDILT
3241 PAK2
3242 NDUFS3
3243 SPACA3
3244 SLC37A4
3245 KLHL24
3246 PLA2G1B
3247 OTOP1
3248 ERBB2
3249 MFGE8
3250 SLC7A11
3251 SULF1
3252 IGFBPL1
3253 JAK2
3254 CD14
3255 OR51B5
3256 SLC17A2
3257 GPR150
3258 LOXL2
3259 EDDM3A
3260 LCT
3261 CFHR1

3262 KCNK17
3263 HSD3B2
3264 OR2H2
3265 TFPI
3266 CD81
3267 MAPK10
3268 MCOLN1
3269 EDEM2
3270 AMBN
3271 PSMA7
3272 CD44
3273 SLC25A24
3274 NLN
3275 GFM2
3276 CD84
3277 ALDH1L2
3278 HMGB1
3279 FGFBP1
3280 OR11A1
3281 PIK3CA
3282 SLC5A6
3283 WNT16
3284 PEPD
3285 SLC5A10
3286 STK38
3287 TBC1D23
3288 ENHO
3289 ERBB3
3290 PDXDC1
3291 PTPRD
3292 PLCL1
3293 SLC4A7
3294 SLC22A1
3295 KCNE1
3296 STK3
3297 OLFML3
3298 MYO3B
3299 OR10W1
3300 IRAK2
3301 LAIR2
3302 RNF39
3303 CD300LD
3304 KLHL32
3305 EIF2AK3
3306 CDK10
3307 TAPBPL
3308 PTGER3
3309 CYP4F11

3310 PPP3CA
3311 PKN3
3312 ADAM9
3313 ACKR1
3314 ACTN2
3315 CNGA1
3316 MSLN
3317 BHMT2
3318 FIBCD1
3319 CACNA2D3
3320 TAAR6
3321 GHR
3322 ASTL
3323 OR7E38P
3324 SLC16A4
3325 CDK9
3326 AKR7A3
3327 OR4N5
3328 OVGP1
3329 HCN1
3330 TIE1
3331 SOSTDC1
3332 PTPRG
3333 NETO2
3334 MUC16
3335 CORT
3336 TRPV6
3337 PPIL6
3338 OR12D2
3339 IL1RN
3340 PIGR
3341 ACKR2
3342 DHFR2
3343 NCOR2
3344 ROR1
3345 MC4R
3346 SMC3
3347 GABRA2
3348 GRIN1
3349 OR1L6
3350 ENGASE
3351 ATG4B
3352 CELA3A
3353 ATP13A3
3354 MFSD9
3355 ABI3BP
3356 CD248
3357 PRSS30P

3358 OR56A3
3359 SLC17A7
3360 CNTN1
3361 TRAV8-4
3362 GRIN2D
3363 KIT
3364 ERBB4
3365 HPRT1
3366 ULBP2
3367 RNF123
3368 CD1E
3369 MAPK6
3370 PDGFC
3371 OR10A3
3372 ADAMTS8
3373 GPR85
3374 GRM2
3375 DUSP18
3376 CGA
3377 ADORA2A
3378 PMS2P3
3379 COL4A3
3380 STYXL2
3381 GPR82
3382 CDH3
3383 IL36G
3384 PARP1
3385 PECR
3386 LIPM
3387 HCN2
3388 OR4C11
3389 ROCK2
3390 MS4A1
3391 IL1A
3392 INHA
3393 NPR1
3394 COL17A1
3395 OR5D18
3396 C8B
3397 PASK
3398 CYP2S1
3399 LPAR2
3400 SLC29A1
3401 CYP2C19
3402 ICAM1
3403 BRSK2
3404 TMPRSS13
3405 OR10G9

3406 HSD17B3
3407 ABCG5
3408 SLC22A7
3409 GRM8
3410 CEACAM8
3411 FSTL4
3412 IFNGR1
3413 HPD
3414 ADGRG4
3415 TUBG1
3416 C1QTNF8
3417 AURKAIP1
3418 BPIFC
3419 LILRA4
3420 SIGLEC9
3421 APOC4-APOC2
3422 DGCR6
3423 OR5B2
3424 DYRK1B
3425 MGST3
3426 OR13C9
3427 DUSP9
3428 SPACA5
3429 SLC2A1
3430 SLC6A3
3431 SCARA5
3432 CA9
3433 KCNJ13
3434 AIFM3
3435 OR6J1
3436 VCAN
3437 SLC25A6
3438 MTMR4
3439 ETNPPL
3440 HLA-DPB1
3441 DUSP26
3442 SUCNR1
3443 PARP6
3444 ASIC5
3445 ADCY9
3446 GPR17
3447 CTLA4
3448 EARS2
3449 OR2A42
3450 SLC6A12
3451 VSIR
3452 C3
3453 LRP8

3454 CHRNG
3455 PYY
3456 OR6K4P
3457 PRPSAP1
3458 FZD3
3459 GSR
3460 IGLL1
3461 PCSK5
3462 KCNK12
3463 GPR18
3464 PSMB2
3465 AKT3
3466 AREG
3467 IGLC7
3468 GUCY2C
3469 LGALS7
3470 MAP3K10
3471 OR11H6
3472 CSNK2A1
3473 STX1A
3474 OBP2B
3475 C1QA
3476 INPP5A
3477 PLK4
3478 CTRL
3479 LY96
3480 OR5H14
3481 TRAV29DV5
3482 GABRG1
3483 CRELD2
3484 IGKV3D-15
3485 HSP90AB2P
3486 BST2
3487 COMTD1
3488 VPREB1
3489 SLC24A1
3490 FAM3C
3491 TRIM11
3492 WFDC2
3493 CD86
3494 STK38L
3495 CD151
3496 ABCC13
3497 SLC22A10
3498 INSR
3499 OR4S1
3500 MTRNR2L7
3501 WNT1

3502 TUBBP1
3503 CRY2
3504 MLYCD
3505 INSL4
3506 OR10D1P
3507 SLC7A4
3508 DCLK3
3509 MGLL
3510 CPO
3511 ITGB7
3512 PRNP
3513 KCNAB2
3514 OR5AP2
3515 AOC3
3516 SLCO1B1
3517 DDX3X
3518 KIF11
3519 TMX2
3520 GRM5
3521 CNTNAP4
3522 JAG1
3523 TAF15
3524 ADCYAP1
3525 DDR2
3526 OR8D2
3527 CCR5
3528 IGKV1-5
3529 OR4D1
3530 CILK1
3531 NYX
3532 IK
3533 PI16
3534 KCNC2
3535 PTPRT
3536 CGB2
3537 GSK3A
3538 MBNL1
3539 ALDH6A1
3540 EIF4E
3541 TAS1R2
3542 VMO1
3543 PTGER4
3544 PLAC1
3545 UGT2B17
3546 DIO3
3547 PDE1A
3548 MAN2B1
3549 OR6V1

3550 SLC9A2
3551 OR5AK3P
3552 GPR88
3553 OR6K6
3554 FGFBP3
3555 HTR2C
3556 HTRA2
3557 MUC7
3558 SLC7A10
3559 MDM2
3560 OR52N1
3561 SPR
3562 SMOX
3563 DMP1
3564 RORC
3565 PARP11
3566 TMPRSS12
3567 DHRS4L2
3568 APEX1
3569 IGFALS
3570 OR10A2
3571 NOTCH2
3572 TAP2
3573 KEL
3574 SMPD3
3575 DEFA4
3576 AKT2
3577 MSTN
3578 CCL26
3579 CHRM1
3580 COL24A1
3581 H3C4
3582 MADCAM1
3583 PLAA
3584 ENDOG
3585 OR5H1
3586 IL1RAPL2
3587 BMP8B
3588 CYP4B1
3589 RDH5
3590 CTSC
3591 CDK13
3592 OR56A4
3593 ERP29
3594 BTNL9
3595 H3C13
3596 SLC40A1
3597 OR4D6

3598 LMTK2
3599 RBMX
3600 ITIH6
3601 P3H1
3602 CCNB3
3603 SLC22A24
3604 NSDHL
3605 MITF
3606 KCNH6
3607 MYOC
3608 DCLK1
3609 OR13C3
3610 CHRNA7
3611 IGF1R
3612 IL4
3613 OR1L1
3614 COL28A1
3615 BTN2A1
3616 MMP25
3617 SLC6A5
3618 NUP62
3619 OR6B3
3620 SLC25A40
3621 SLURP1
3622 EPHB2
3623 HSD17B2
3624 CCR2
3625 DKK2
3626 YES1
3627 NMUR1
3628 PRKCI
3629 GALR1
3630 GDF7
3631 CASK
3632 BIRC8
3633 GDF10
3634 RNASE9
3635 CSAD
3636 ACE
3637 OR4C46
3638 LYZ
3639 HSPA5
3640 SLC25A42
3641 DDC
3642 MMP12
3643 PLCD4
3644 COMP
3645 MAPK4

3646 IGLV2-18
3647 SCD
3648 ECM1
3649 OR6C2
3650 SERPINA6
3651 ADRB1
3652 PTGFRN
3653 OR7D4
3654 RETN
3655 TNNI3K
3656 POLR1C
3657 PTPRM
3658 GSN
3659 SLC25A38
3660 MIA
3661 OR6K3
3662 VN2R1P
3663 GPC2
3664 VWA2
3665 MATN1
3666 PSMB3
3667 GPR174
3668 FNDC5
3669 SLC22A14
3670 GALNT1
3671 UTP11
3672 ORM2
3673 ULK1
3674 STIM2
3675 MBTPS1
3676 TDGF1
3677 NPBWR1
3678 KCNN2
3679 OR8B8
3680 DNAJC10
3681 ADAMTS7
3682 SLC25A5
3683 TUBB6
3684 CHGB
3685 CCR7
3686 GPR132
3687 IGHV3-23
3688 TMPRSS2
3689 F13A1
3690 SIGLEC6
3691 SYK
3692 KMT2A
3693 TINAG

3694 FAT4
3695 P2RY2
3696 PRKG2
3697 SIGLEC8
3698 IL12RB2
3699 MFSD8
3700 SLC46A1
3701 OR4X1
3702 DEFB127
3703 KLHL28
3704 CD300LG
3705 SLC18A2
3706 AKR1C3
3707 IGHV7-81
3708 PRKD2
3709 IL15
3710 CD9
3711 CCKBR
3712 SGPL1
3713 MT-CO2
3714 PLCB3
3715 BRPF3
3716 MTRNR2L3
3717 OR14L1P
3718 TRIM41
3719 TTK
3720 MMP13
3721 KCNG3
3722 GIPR
3723 C8A
3724 PLG
3725 ATP7A
3726 LMNA
3727 CD79A
3728 LIPI
3729 LPAR4
3730 OR13A1
3731 CHRM5
3732 INS-IGF2
3733 PLEK
3734 ITGA3
3735 KLHL7
3736 WNT11
3737 CA6
3738 ST14
3739 C5AR1
3740 ESM1
3741 NR5A2

3742 ATP13A2
3743 CHAD
3744 DCK
3745 ARSB
3746 ITGA11
3747 EPAS1
3748 OR6W1P
3749 HTR5BP
3750 SMPDL3A
3751 FCGRT
3752 PTBP1
3753 ITFG1
3754 IRAK1
3755 CPT1C
3756 IGLV5-45
3757 PODN
3758 NAXE
3759 TUSC3
3760 SLC22A25
3761 SPNS2
3762 SRM
3763 SLC6A11
3764 OR51F1
3765 GABRP
3766 TGM6
3767 SIRPG
3768 GRIK1
3769 CD53
3770 SLCO1C1
3771 CMKLR1
3772 DEFB103A
3773 GRIK3
3774 LMLN
3775 OR7E125P
3776 GPX4
3777 PLAT
3778 NFKB2
3779 UBA2
3780 SLC6A20
3781 LAMB2
3782 ADAMTS6
3783 KLHL8
3784 MTR
3785 KCNA6
3786 ABCC1
3787 SLC22A4
3788 CCL20
3789 PPIB

3790 PSMB4
3791 ADGRA1
3792 CRHR2
3793 OR4K2
3794 HIPK1
3795 SLC19A3
3796 ERP44
3797 SPAG11A
3798 LACTB
3799 CLCNKB
3800 PTPN6
3801 KCNK5
3802 ALDH1L1
3803 KLHL38
3804 COL26A1
3805 MAP3K15
3806 HMSD
3807 SLC9A9
3808 C8G
3809 DHRS7B
3810 TNFRSF4
3811 ABCB4
3812 NEK7
3813 H1-4
3814 GLRA2
3815 TUBB3
3816 PTPN3
3817 GGT5
3818 ZP3
3819 MYO3A
3820 STYK1
3821 BTLA
3822 PPP5C
3823 ABCA12
3824 OR10AA1P
3825 BRSK1
3826 OR5F1
3827 MUC4
3828 GPR62
3829 OR5W2
3830 DDR1
3831 POR
3832 OR52E6
3833 PPIF
3834 OR10R2
3835 HAPLN3
3836 PSMB1
3837 GADL1

3838 SLC25A2
3839 SPOCK3
3840 CNDP1
3841 SLIT1
3842 CD4
3843 ACP1
3844 PXDN
3845 RBP4
3846 MAST4
3847 HNRNPA1
3848 IGKV2-30
3849 PPIL4
3850 IGHG4
3851 AVPR1A
3852 BCL2A1
3853 STEAP1
3854 TLR2
3855 GC
3856 CDCP1
3857 GPR84
3858 LIMK2
3859 SPHK1
3860 EGLN3
3861 CYP26C1
3862 CCR8
3863 VWA3B
3864 CHRNA3
3865 MUC2
3866 NTRK1
3867 OR56B1
3868 COL18A1
3869 ELOVL6
3870 OR4D9
3871 PDE4C
3872 GSTM5
3873 BOLA3
3874 EPHA7
3875 AKT1S1
3876 MYLK2
3877 METAP1
3878 IFNB1
3879 TLL2
3880 IL22RA2
3881 LILRA6
3882 CXCR1
3883 TRIM75
3884 MRGPRX2
3885 LCK

3886 PROK1
3887 SLCO5A1
3888 GAK
3889 CAPN14
3890 TRPC3
3891 ITGA10
3892 DEAF1
3893 MC5R
3894 PIM3
3895 TPP1
3896 USP2
3897 HUNK
3898 IL12RB1
3899 PRSS23
3900 AGGF1
3901 OR52A5
3902 OR10K1
3903 OLFML1
3904 HNRNPU
3905 S1PR3
3906 INPP5D
3907 MAP4K2
3908 NCAN
3909 CD3E
3910 HTR3A
3911 MAP2K5
3912 OR51L1
3913 TRH
3914 CELA2B
3915 HRH3
3916 OR11H12
3917 SFN
3918 DUOX1
3919 HMCN1
3920 OR2C3
3921 FKBP1C
3922 BGLAP
3923 MMP15
3924 ALOX15P2
3925 LOX
3926 IMPA1
3927 CDC42
3928 PTN
3929 RYR3
3930 LGMN
3931 SULT1B1
3932 UGT2B4
3933 RTN4IP1

3934 CLCN3
3935 OR51A7
3936 CNTNAP1
3937 PYDC1
3938 IL12B
3939 OR6C3
3940 F2R
3941 KLKB1
3942 ICOSLG
3943 CATSPER4
3944 ITGA5
3945 ADGRF4
3946 ANGPT1
3947 CCDC80
3948 MAP2K6
3949 HPSE2
3950 MST1P2
3951 CGB7
3952 CEACAM16
3953 OR10H5
3954 MAP4K1
3955 IL3
3956 PTPRQ
3957 GPR171
3958 CES5A
3959 PHYKPL
3960 ACP6
3961 OR14K1
3962 DST
3963 OR1M1
3964 MAP2K1
3965 CASP5
3966 CDK7
3967 CTSS
3968 CHEK2
3969 BTN3A1
3970 LGR6
3971 OR3A1
3972 IL16
3973 DHCR7
3974 IGLV11-55
3975 TGFBR1
3976 EMC10
3977 ST3GAL1
3978 GPC3
3979 TRIML2
3980 NAT1
3981 AMY1B

3982 GCGR
3983 KRTDAP
3984 TLR8
3985 TMEM123
3986 DDO
3987 BPIFA2
3988 DPEP2
3989 CLCA3P
3990 ABCA4
3991 PTH2R
3992 HTR2B
3993 EPHA4
3994 STK33
3995 FGG
3996 TAC3
3997 CACNG5
3998 OR2A14
3999 SBK2
4000 KEAP1
4001 PPIAL4A
4002 SLC17A8
4003 OR9A2
4004 HDAC10
4005 OPRM1
4006 THSD4
4007 PLA2G6
4008 H3C1
4009 EPO
4010 PDE12
4011 OR4A13P
4012 RHD
4013 RNASE10
4014 SLC9A3
4015 CTSV
4016 OR6T1
4017 FKBP1B
4018 PAK6
4019 CA14
4020 HBB
4021 CHSY1
4022 AFM
4023 PPT1
4024 AOX1
4025 UCHL1
4026 CD226
4027 NRG4
4028 ADGRF1
4029 KIR3DL3

4030 CYP27C1
4031 ADAM17
4032 EFNB3
4033 OR52J3
4034 GP5
4035 BAMBI
4036 MC1R
4037 ACACB
4038 IFNA5
4039 HIPK3
4040 NAPSA
4041 COL13A1
4042 CDKL2
4043 PLCH2
4044 PDIA4
4045 OR10Z1
4046 MMP3
4047 OR8I2
4048 HSD17B1P1
4049 ULK2
4050 TNK2
4051 OR4C45
4052 BPIFA1
4053 CLCN2
4054 ADAMTS2
4055 NXPH3
4056 BAK1
4057 CWC27
4058 HTR1F
4059 MOCS3
4060 GYPA
4061 H3C14
4062 OLFM3
4063 OR2B3
4064 NR1I2
4065 SSC5D
4066 BAX
4067 CTSA
4068 EPHA10
4069 DHRS11
4070 FAM20A
4071 APEH
4072 REG4
4073 DLD
4074 REG1A
4075 DEFB104A
4076 PARP14
4077 CSNK1E

4078 CCN5
4079 QSOX2
4080 RHO
4081 TRPM7
4082 KLHL13
4083 CD1A
4084 GUSB
4085 KLF5
4086 FCGR2B
4087 IGHM
4088 BTNL3
4089 FCRL4
4090 RDH8
4091 DUSP23
4092 TRHR
4093 CCNE1
4094 OAT
4095 EPRS1
4096 OR7E24
4097 SLC13A1
4098 SERPING1
4099 FGFR4
4100 ANOS1
4101 NMT2
4102 PLA2G2F
4103 TGFBR2
4104 MCCC2
4105 TNFSF12
4106 AOC2
4107 OR11H7
4108 TUBAL3
4109 AMBP
4110 PYROXD1
4111 PTP4A3
4112 NOS2
4113 CA1
4114 TNFRSF1B
4115 OR14C36
4116 CSK
4117 FSTL1
4118 FKBP11
4119 GHRH
4120 OR10R3P
4121 CFLAR
4122 KCNT1
4123 RET
4124 GLUL
4125 LINGO1

4126 SLC25A30
4127 LIPC
4128 AGBL2
4129 SERPINA5
4130 KLK4
4131 PTGDR2
4132 F11R
4133 GABRA1
4134 CBR1
4135 GPC1
4136 DBH
4137 TNFAIP2
4138 OR2T33
4139 MTRNR2L10
4140 IGKV1-39
4141 ADCY1
4142 COPA
4143 DNAJB11
4144 OR51D1
4145 KRT19
4146 PKD1L3
4147 PTGDR
4148 MATK
4149 OR4C15
4150 CNTN4
4151 AADAC
4152 HSD11B1L
4153 TPTE2
4154 MAPKAPK3
4155 OR2Z1
4156 FCAR
4157 HCN3
4158 GRM3
4159 PDGFRA
4160 PRRT3
4161 OBSCN
4162 ITGA8
4163 USP8
4164 CASR
4165 PRADC1
4166 OR10C1
4167 ADAMTS1
4168 H4C6
4169 SOAT1
4170 CD276
4171 MMP10
4172 CHST1
4173 CST9L

4174 CGB5
4175 SLK
4176 OPCML
4177 TLR4
4178 PYGM
4179 IL36B
4180 DEFB107B
4181 ACP3
4182 RIPK3
4183 P2RX2
4184 GRIN2C
4185 P2RY14
4186 TXNRD2
4187 KCND3
4188 TRIM49
4189 PDE8B
4190 SRC
4191 TXN
4192 SUPT16H
4193 SLC25A48
4194 ATP6AP2
4195 H4C3
4196 FMOD
4197 ITGAX
4198 CD40LG
4199 TRIM69
4200 STK31
4201 NEK5
4202 IL31RA
4203 TNFRSF14
4204 CSHL1
4205 CGB8
4206 AKR1B10
4207 RHCG
4208 CCS
4209 ADAMTS3
4210 IL2
4211 OR6F1
4212 TRIM65
4213 CDK6
4214 MARCO
4215 BPIFB6
4216 CEACAM5
4217 KLK14
4218 CAD
4219 TLR9
4220 IFNAR1
4221 LPO

4222 LIF
4223 TBCK
4224 IGLV1-51
4225 MTAP
4226 YBX1
4227 OR2AK2
4228 UGT2B11
4229 COL1A2
4230 FKBP9P1
4231 DUSP8
4232 SLC22A31
4233 C8ORF44-SGK3
4234 SPAG11B
4235 DUSP14
4236 TRIM51
4237 PTPN5
4238 PTGS2
4239 CER1
4240 PROM1
4241 STK10
4242 OR10G6
4243 LRRC17
4244 UGT1A4
4245 NT5C1B
4246 PLP1
4247 RELA
4248 ARSA
4249 TSSK6
4250 HDAC5
4251 OR4D11
4252 CPA5
4253 PRKG1
4254 CAMK1
4255 DUSP6
4256 CX3CR1
4257 OR5P2
4258 TUBA3FP
4259 IGLL5
4260 IGLV1-44
4261 PDK4
4262 TESK2
4263 IL1B
4264 OR1L8
4265 ADAM5
4266 NUCB2
4267 BHMT
4268 CYP26B1
4269 ITIH5

4270 LRRC4
4271 CRLF1
4272 SIRT1
4273 PTPRH
4274 NPEPPS
4275 SPINT3
4276 RNASE13
4277 GALK1
4278 L1CAM
4279 F2RL1
4280 LIPK
4281 HLCS
4282 GSTM2
4283 MARK2
4284 SLC9A6
4285 DUSP28
4286 PRR29
4287 HTR6
4288 RSPO3
4289 RBCK1
4290 HCAR2
4291 DEFB108B
4292 RDH13
4293 CNGB1
4294 NEK1
4295 PDE8A
4296 OR1N2
4297 SMOC1
4298 IDO1
4299 AXIN2
4300 CARD17
4301 OR2F1
4302 COL5A2
4303 IGF1
4304 PRKCG
4305 RPS6KA1
4306 SOST
4307 OR52A1
4308 POLB
4309 TPSG1
4310 MAN2A2
4311 PTAR1
4312 OR4B1
4313 IL20
4314 CCL4L1
4315 CCNT1
4316 ALDH1A1
4317 OR5AU1

4318 LYZL4
4319 ADGRG7
4320 PDE2A
4321 NRBP2
4322 CACNG1
4323 CXCL17
4324 CYP2F1
4325 ADGRB2
4326 KSR1
4327 RNASE7
4328 SLC22A16
4329 CYB5D2
4330 HSD3B7
4331 NPR2
4332 TXNDC5
4333 GHRL
4334 ATP13A5
4335 GPX6
4336 CRYZL1
4337 BST1
4338 GGT2P
4339 EFEMP2
4340 LGALS7B
4341 OR1A2
4342 OR6C6
4343 MMP2
4344 KCNH5
4345 IL1R2
4346 MEP1A
4347 GPR20
4348 TNFRSF11B
4349 GLRA1
4350 SLC25A1
4351 SYNJ2
4352 MAP4K5
4353 APH1B
4354 SLC25A18
4355 SV2B
4356 NR2E1
4357 MELTF
4358 TNR
4359 CD180
4360 PDE6G
4361 TRPC1
4362 KCNJ1
4363 PTAFR
4364 OR1J1
4365 SPINT1

4366 CNTNAP3B
4367 OR2D3
4368 INHBA
4369 WNK3
4370 PRAP1
4371 WEE2
4372 PKM
4373 UBE2N
4374 BTN1A1
4375 GZMA
4376 GDF1
4377 HSD17B12
4378 TMPRSS11B
4379 APOO
4380 NECTIN4
4381 PGD
4382 OR10G8
4383 CLU
4384 DRD5P2
4385 PPID
4386 CCN4
4387 SERPINB2
4388 CEACAM6
4389 RANBP10
4390 EXOG
4391 MMP17
4392 PPIAL4C
4393 PIP4K2C
4394 GKN1
4395 KLK2
4396 KCNH8
4397 SLC46A2
4398 IL7R
4399 SLC43A3
4400 TOP2B
4401 LTF
4402 ICOS
4403 OPN1LW
4404 PIGF
4405 INSL5
4406 SCUBE2
4407 SLC2A5
4408 UBA6
4409 C1QB
4410 HPGD
4411 MMP20
4412 TERT
4413 AMD1

4414 RNASEH1
4415 ADCYAP1R1
4416 MAG
4417 SLC6A7
4418 NT5E
4419 LILRA5
4420 CDC7
4421 AZIN1
4422 ENPP2
4423 CD1C
4424 POMC
4425 OR9K2
4426 ADAM11
4427 HS3ST1
4428 LY6D
4429 GGH
4430 PTGER4P2
4431 PNLIPRP3
4432 ABHD16A
4433 SLC1A2
4434 OR10J6P
4435 SFTPD
4436 SLIT3
4437 TK1
4438 MORC3
4439 ESRRA
4440 ACAN
4441 ITGA2
4442 IGHG3
4443 IGSF8
4444 H3-3B
4445 VKORC1L1
4446 CHRNB4
4447 FURIN
4448 CD96
4449 CCL22
4450 F3
4451 ITGA7
4452 TAOK3
4453 LAMC2
4454 LATS2
4455 TNFRSF13B
4456 SLC1A7
4457 ABCA6
4458 TRIM15
4459 PRAME
4460 OR3A2
4461 CYTL1

4462 NOX4
4463 OR2V2
4464 MTRNR2L8
4465 HDGF
4466 ITGB6
4467 PIGK
4468 ASIC3
4469 APOL1
4470 PARP15
4471 CYB5R2
4472 CLCN4
4473 CR1L
4474 OLFM4
4475 ABCD4
4476 OR10Q1
4477 BTN3A3
4478 BMPR2
4479 SPARC
4480 OR52I1
4481 CYP4Z2P
4482 HSPA1A
4483 PRSS57
4484 RS1
4485 OR8S1
4486 PCYOX1
4487 KCNJ9
4488 RETNLB
4489 ALDH4A1
4490 OLFML2A
4491 KITLG
4492 OR5V1
4493 OR2J3
4494 ATM
4495 JMJD7-PLA2G4B
4496 CYP26A1
4497 PPIC
4498 CAMKK1
4499 MUC13
4500 ELANE
4501 GNLY
4502 CFHR3
4503 SMO
4504 DEFB118
4505 KLHL15
4506 IAPP
4507 ZRANB3
4508 PTGER1
4509 LALBA

4510 FOLR1
4511 MEP1B
4512 ANGPT4
4513 PRSS54
4514 IGFL3
4515 HTR7P1
4516 GLO1
4517 LCTL
4518 STK40
4519 FIBIN
4520 CALR
4521 KCNA2
4522 GAPDH
4523 TULP2
4524 ADAM28
4525 UCP3
4526 PLCG2
4527 CD300LF
4528 FGF2
4529 STK39
4530 DMPK
4531 UGGT1
4532 OR2A3P
4533 MORC2
4534 CPA6
4535 TLR10
4536 PCSK9
4537 CALCRL
4538 DUSP1
4539 IL6R
4540 NRG2
4541 WNT10B
4542 PLA2G7
4543 CXCL16
4544 SPX
4545 CD52
4546 C5AR2
4547 PRSS50
4548 CLCA2
4549 GPR31
4550 RXRB
4551 CCL23
4552 SLC22A11
4553 TRIM25
4554 OR52L2P
4555 CLCN7
4556 BMP15
4557 ADORA1

4558 TRPM5
4559 PREPL
4560 ACLY
4561 CETP
4562 INSRR
4563 OR4D2
4564 CXADR
4565 LNPEP
4566 DCXR
4567 UGT2B7
4568 ADGRD2
4569 PRSS55
4570 CDKL3
4571 AKR1C2
4572 TRIML1
4573 OR52L1
4574 KCNQ3
4575 CACNB3
4576 TUBA3D
4577 CHRNA5
4578 NPY1R
4579 COL9A1
4580 SLC6A1
4581 BMPER
4582 SLC12A8
4583 OR1P1
4584 CRHBP
4585 OR1L3
4586 KCNH7
4587 WARS1
4588 OR10J3
4589 OR56A1
4590 NCAM1
4591 OR10J1
4592 ERCC5
4593 BDKRB1
4594 VN1R2
4595 SLC7A2
4596 HABP2
4597 TNF
4598 LIPA
4599 SMG1
4600 CHL1
4601 REN
4602 ITGAM
4603 NRG1
4604 CAPN3
4605 KCNG1

4606 GALNT2
4607 KLK11
4608 EDNRA
4609 BTN2A2
4610 MAT1A
4611 ATR
4612 CCNB2
4613 CST4
4614 SCGB3A1
4615 HGF
4616 PIK3C2B
4617 ABCF2
4618 PLA2G5
4619 SLC5A9
4620 MYH7
4621 QRFP
4622 KCNT2
4623 HHIPL1
4624 CD8B
4625 SLC11A2
4626 SLC6A15
4627 OR7E22P
4628 CHUK
4629 IFNA21
4630 PRSS2
4631 OR51I1
4632 OR4K13
4633 GNS
4634 MAST3
4635 NOTUM
4636 GZMH
4637 IL13
4638 SIDT2
4639 GPER1
4640 ITM2B
4641 SSTR5
4642 GRK3
4643 AATK
4644 KCNG2
4645 CCL3
4646 TNKS2
4647 GPR35
4648 PRSS36
4649 PTPN21
4650 OR4F17
4651 LDHA
4652 ENAM
4653 BDKRB2

4654 SMN2
4655 GPR87
4656 NR2F6
4657 OR52E5
4658 SLC12A5
4659 OR4M2
4660 CD163
4661 GPR149
4662 EDN3
4663 PTPRK
4664 ANGPTL5
4665 ISLR2
4666 SOAT2
4667 KLHL25
4668 TRIM14
4669 NOS1
4670 CLCN1
4671 OR7E129P
4672 SLIT2
4673 CAMK1G
4674 PDE3B
4675 AMELX
4676 CYP2J2
4677 NCR2
4678 LRRC4C
4679 GUSBP12
4680 C1QTNF6
4681 OR7G1
4682 CLSTN1
4683 UTS2R
4684 OR8J1
4685 ABCD3
4686 GNRH2
4687 TGM3
4688 SLC39A6
4689 HSD11B1
4690 GRIK4
4691 LPAR3
4692 IGKV2-40
4693 IGLV3-12
4694 CMA1
4695 MST1R
4696 ADAMTSL3
4697 TYMS
4698 TSSK4
4699 TTBK1
4700 OR2L3
4701 ITIH2

4702 SCARB1
4703 ZP2
4704 AGBL3
4705 EGFL8
4706 ABCB5
4707 SLC17A6
4708 OR1L4
4709 NPNT
4710 MUCL1
4711 REV3L
4712 OPN4
4713 STK26
4714 CP
4715 KLHL18
4716 CTSW
4717 FGF23
4718 ABCG1
4719 TRIB2
4720 TRBV7-1
4721 OR6N1
4722 HSP90AA1
4723 CACNA1F
4724 CXCR3
4725 MFAP2
4726 MORC4
4727 TNFSF10
4728 IRAK3
4729 POSTN
4730 SLC5A3
4731 CAPN2
4732 IL1RAPL1
4733 TYSND1
4734 ARSF
4735 ANGEL2
4736 TGM4
4737 OR11L1
4738 SCN2B
4739 CD8A
4740 OR8A1
4741 PNP
4742 LYZL2
4743 OLFML2B
4744 TUBA4B
4745 OR2B2
4746 ITIH1
4747 PARP12
4748 COL6A3
4749 IL21R

4750 SLC22A15
4751 FZD9
4752 PI4K2B
4753 CCL11
4754 IL9
4755 DSTYK
4756 FAAH
4757 OR11N1P
4758 GPX8
4759 PIK3R1
4760 AZIN2
4761 AADAT
4762 MAP3K8
4763 KBTBD8
4764 OR10AG1
4765 HEXA
4766 OR5M9
4767 STK25
4768 COL25A1
4769 GPR37
4770 ZAR1L
4771 FGB
4772 CD101
4773 ADAM12
4774 TMPRSS11F
4775 ADAM18
4776 CLK3
4777 POLD1
4778 OR51Q1
4779 CAPN1
4780 PRKCSH
4781 CELSR3
4782 ARSI
4783 GSTA3
4784 CXCL9
4785 CTHRC1
4786 SEMA3E
4787 UGT2B15
4788 CAPN10
4789 KCNS1
4790 CCL28
4791 EGFL6
4792 GABRB2
4793 TRIM49B
4794 CD6
4795 SERPINB10
4796 TYRP1
4797 KLHL2

4798 PTGES
4799 DUSP21
4800 LMAN1
4801 PRSS22
4802 CYP3A4
4803 PSMA2
4804 TPSB2
4805 GPR146
4806 IL1RAP
4807 PCK1
4808 CAPN7
4809 LAMP3
4810 COL11A2
4811 ADRA1D
4812 H4C8
4813 OR5P3
4814 OR56A5
4815 PLCB2
4816 FGF12
4817 SFTPC
4818 TP63
4819 MOS
4820 BOK
4821 CHST5
4822 SIRPA
4823 HMMR
4824 RPS6KA2
4825 DEFB103B
4826 CYP4A22
4827 SLC7A14
4828 MAPK3
4829 MGAT4A
4830 IFNA1
4831 XCL1
4832 GP1BA
4833 SERPINB13
4834 PCSK1
4835 GUCA2A
4836 INPP5K
4837 F2RL2
4838 TXNDC12
4839 SVOP
4840 HLA-C
4841 HAO2
4842 ANXA1
4843 OTC
4844 ADAMTSL1
4845 PPIL3

4846 CSF2
4847 RIOK1
4848 MAN1B1
4849 PRSS33
4850 SIGLEC11
4851 TXNDC16
4852 TRIM43
4853 GDF6
4854 MMP27
4855 GSTK1
4856 KLHL5
4857 OR4F29
4858 SFRP5
4859 SLC25A46
4860 NAE1
4861 ADGRG6
4862 COL19A1
4863 OR10AB1P
4864 MPIG6B
4865 OR10T2
4866 PTGER2
4867 WNT2
4868 NRP1
4869 PLCL2
4870 IL37
4871 VAMP1
4872 OR5AS1
4873 ORM1
4874 DUSP5
4875 BIRC3
4876 CHST7
4877 MGAM
4878 CAPN15
4879 LTBP2
4880 IGLV4-3
4881 NUP210
4882 PSAP
4883 P2RY11
4884 PINK1
4885 SCGB1D4
4886 ADAM32
4887 CRTAM
4888 C9ORF47
4889 ADORA2B
4890 VSTM1
4891 CD302
4892 OR2J2
4893 P2RY12

4894 STKLD1
4895 DCT
4896 CREG1
4897 SORD
4898 OR10X1
4899 LRRTM3
4900 SLC9B1
4901 OR7E102P
4902 HEPHL1
4903 ITGAE
4904 EFTUD2
4905 CPN1
4906 NR0B2
4907 ME1
4908 VWA1
4909 CD300A
4910 PNOC
4911 COL8A2
4912 HGFAC
4913 S1PR1
4914 EFNA1
4915 OR51G2
4916 SLC16A6
4917 MAGEA3
4918 P2RX1
4919 NAGLU
4920 FLT3
4921 MUC5B
4922 OR2T4
4923 DNAJA1
4924 DMKN
4925 DEFB1
4926 SUV39H1
4927 OR8D1
4928 TSHB
4929 PTPRR
4930 LATS1
4931 MAP3K19
4932 VRK1
4933 PARP16
4934 LGALS3
4935 VEGFC
4936 MOK
4937 LUM
4938 PARP9
4939 APOA1
4940 ABCD2
4941 OR2Y1

4942 OR2T35
4943 NKTR
4944 SSH3
4945 GANAB
4946 RNASE8
4947 CYP2U1
4948 CASP2
4949 PRG2
4950 C9
4951 COL6A6
4952 MTRNR2L1
4953 TUBB4B
4954 OR5D17P
4955 SERPINA7
4956 PDGFB
4957 AMY2B
4958 OR4K14
4959 FCRL2
4960 JAM3
4961 SLC25A20
4962 TUBA3E
4963 GLRX3
4964 MMP26
4965 FLT3LG
4966 PSPN
4967 CCL16
4968 OR4K1
4969 APOA4
4970 CASP8
4971 TRIM39
4972 SULT2B1
4973 CPA4
4974 TMED9
4975 TSPAN7
4976 HSD17B4
4977 SSH1
4978 CFD
4979 FGL2
4980 CAMKK2
4981 OR4X2
4982 PPWD1
4983 OR13C2
4984 SLCO1A2
4985 MUC17
4986 SLC6A2
4987 HYOU1
4988 CALM2
4989 FGL1

4990 HAMP
4991 PIK3CB
4992 CYP2D6
4993 IL2RB
4994 HSPA1B
4995 A2M
4996 SMPD2
4997 ST3GAL4
4998 TGM5
4999 GPR39
5000 GRID2
5001 GPR141
5002 DRD3
5003 LTBP1
5004 BPIFB4
5005 IFNAR2
5006 CALM1
5007 THNSL2
5008 CLEC4C
5009 PPM1B
5010 SLC7A8
5011 STYXL1
5012 CD3D
5013 TRIM9
5014 PRKACB
5015 GLI2
5016 TRHDE
5017 PRSS38
5018 KCNN1
5019 IL10RB
5020 ADAMTS16
5021 FGFR1
5022 CHRNA9
5023 UCN
5024 SRD5A1
5025 KBTBD7
5026 MAPK12
5027 SCN2A
5028 PRKD1
5029 FGF21
5030 ATP13A4
5031 GABRA4
5032 PDE1C
5033 VCL
5034 GABBR2
5035 HNF4A
5036 GPD2
5037 XPNPEP1

5038 GZMB
5039 OR5I1
5040 GSTT1
5041 SHARPIN
5042 IL32
5043 ADH1B
5044 OR9G1
5045 IGFBP2
5046 PZP
5047 COL15A1
5048 AZGP1
5049 BLM
5050 PLA2G4A
5051 APOA5
5052 OR2AP1
5053 CAPN6
5054 TNK1
5055 GUCY1A2
5056 NLK
5057 KIR2DL1
5058 TNFRSF17
5059 AVP
5060 PON3
5061 OR6B2
5062 RIOK2
5063 ESR1
5064 CALU
5065 PLD1
5066 CTSH
5067 BMP10
5068 KLHL21
5069 EBP
5070 HTT
5071 SLC25A4
5072 TGM7
5073 ADRA2A
5074 PDPK1
5075 SLC6A13
5076 TPT1
5077 TAFA3
5078 PRB3
5079 MFAP5
5080 CHST3
5081 BUB1
5082 FSHB
5083 ARSG
5084 RNASE3
5085 AGTR2

5086 SPINK6
5087 KCNA7
5088 OR13F1
5089 TNFRSF21
5090 OR52B2
5091 TLK2
5092 ODAM
5093 LRRC26
5094 OR12D3
5095 OR4L1
5096 SPN
5097 RPS6KC1
5098 MTM1
5099 MAN2C1
5100 CASP14
5101 CYP4V2
5102 SERPIND1
5103 CD63
5104 TNKS
5105 SLC5A12
5106 DNASE1L2
5107 CYP2A7P1
5108 CEL
5109 GPHA2
5110 OR14J1
5111 RFPL4B
5112 CSF2RA
5113 SPOCK1
5114 TNFAIP6
5115 SLCO2B1
5116 DHRS4
5117 PRIMA1
5118 PCCB
5119 NR2F2
5120 RPS6KA4
5121 SLC12A3
5122 SRPK3
5123 XDH
5124 NMS
5125 TPH2
5126 CISD1
5127 CCR1
5128 IRAK4
5129 ADM
5130 OR5K2
5131 C6ORF120
5132 FEN1
5133 HDAC3

5134 CYP8B1
5135 OR4A5
5136 SLC16A9
5137 CYP27B1
5138 CDK15
5139 HIF1A
5140 PSMB7
5141 OR5J2
5142 SCN4A
5143 SSTR4
5144 OR6C74
5145 MMP28
5146 KCNC1
5147 IL36RN
5148 SLC25A27
5149 RECQL
5150 A2ML1
5151 SLC10A4
5152 CCL4
5153 FRRS1
5154 HTR1A
5155 SGK1
5156 OR1F1
5157 CRYZ
5158 SMPD1
5159 BSPH1
5160 DHRS3
5161 OR2T6
5162 PDYN
5163 TXN2
5164 ENPP3
5165 PLCG1
5166 CSNK1A1L
5167 F10
5168 CBLIF
5169 OR11H4
5170 KLK5
5171 PATE4
5172 PF4
5173 TRPV2
5174 VCAM1
5175 TUBA3C
5176 PELO
5177 ASGR1
5178 KIR3DL1
5179 CEACAM3
5180 CYP3A5
5181 IGLV8-61

5182 OR52B1P
5183 TMPRSS7
5184 OR5K1
5185 SLC9A5
5186 DUSP12
5187 TECTB
5188 OR6M1
5189 KDM1A
5190 PNLIPRP1
5191 SPINK4
5192 SF3B3
5193 FBP1
5194 MTTP
5195 STS
5196 TDP2
5197 KLHL29
5198 PARM1
5199 FCN1
5200 CYB5R1
5201 CHMP1A
5202 WNK2
5203 IL5
5204 BCAT1
5205 PRSS12
5206 MBL2
5207 ABCA7
5208 ACE2
5209 ADAM19
5210 PDE9A
5211 CYP19A1
5212 CST2
5213 NR4A2
5214 CFHR4
5215 AKR1C8
5216 RDH14
5217 CNGA2
5218 SLCO4C1
5219 OR2A4
5220 MAP2K2
5221 CCL13
5222 TCN1
5223 CTSB
5224 CSN1S1
5225 PDK3
5226 RANBP9
5227 CHI3L2
5228 SERPINB11
5229 CST9

5230 SIAE
5231 MUC3A
5232 REL
5233 TRPV1
5234 IL11
5235 SLC2A7
5236 KCNJ3
5237 UMPS
5238 PPAT
5239 TLR3
5240 SULT1A1
5241 OR52R1
5242 LILRA3
5243 CHRM3
5244 KCNF1
5245 SRPX2
5246 OR1Q1
5247 SLC7A6
5248 OR2B11
5249 GUCY2F
5250 PRKCQ
5251 ADRA1B
5252 CILP
5253 BMP5
5254 OTOG
5255 MGST1
5256 OR5J1P
5257 HAGH
5258 TLR7
5259 FKBP4
5260 MUC5AC
5261 CST3
5262 CARM1
5263 KDSR
5264 CAPN8
5265 COL16A1
5266 GDF2
5267 MATN2
5268 CTBS
5269 NPW
5270 AOAH
5271 LTA
5272 WNT7B
5273 EPHB1
5274 OR4K15
5275 VEGFB
5276 SLC25A45
5277 CYP3A7

5278 FOS
5279 NR2E3
5280 HSD17B10
5281 SERPINI2
5282 OR1N1
5283 FSTL5
5284 GSTM1
5285 CTRB2
5286 IFITM1
5287 OR10H4
5288 NFKB1
5289 TULP1
5290 PSMA4
5291 WNT8A
5292 ADRA1A
5293 TINAGL1
5294 SFRP4
5295 KISS1R
5296 SPINK2
5297 CHST2
5298 LYG2
5299 SCGB1D2
5300 GPRC5A
5301 EMILIN3
5302 CALCR
5303 SLC25A29
5304 ATRN
5305 BCL2L1
5306 SBSN
5307 RGR
5308 OR2K2
5309 IGLV5-37
5310 CCL19
5311 ADCY2
5312 KCNA4
5313 IGF2
5314 ALDH18A1
5315 CD164L2
5316 RYBP
5317 CYP2B6
5318 SLC2A6
5319 OR5AC2
5320 CDK16
5321 KCNC4
5322 SERPINA1
5323 TLK1
5324 UGT1A9
5325 HCRTR1

5326 GALNS
5327 SLC29A3
5328 CHRNA4
5329 OR5H6
5330 STK11
5331 OR4C16
5332 FLRT1
5333 PCOLCE2
5334 SERPINC1
5335 KISS1
5336 CHI3L1
5337 NPTX2
5338 IGFBP3
5339 ADAM23
5340 IFNG
5341 AGT
5342 SCN10A
5343 TSLP
5344 F8
5345 ITFG2
5346 TNFSF4
5347 CD300C
5348 C17ORF99
5349 CDK12
5350 CLEC4M
5351 KBTBD2
5352 OR1B1
5353 PLA2G4B
5354 CHIT1
5355 OTOR
5356 SBK1
5357 GPR55
5358 CDK2
5359 GABRR2
5360 FREM1
5361 CYBB
5362 SLC17A9
5363 DKK4
5364 KLK15
5365 KCNJ11
5366 OR5AR1
5367 SFTA2
5368 RAF1
5369 CD28
5370 SLC10A1
5371 DAGLA
5372 HLA-DRB5
5373 PDE7A

5374 PTK2
5375 OR52B4
5376 LECT2
5377 IFNW1
5378 LOXL3
5379 KCNG4
5380 VEGFA
5381 BPIFA3
5382 RIPK2
5383 SLC5A11
5384 MUC1
5385 AKR1E2
5386 ABCC8
5387 CXCL8
5388 FMO4
5389 EMID1
5390 TUBA4A
5391 OVCH2
5392 GYPE
5393 CNR2
5394 KLHL3
5395 TNFRSF6B
5396 TPH1
5397 WFIKKN1
5398 SMCHD1
5399 PRSS37
5400 TMED1
5401 CELSR2
5402 MFSD6L
5403 OR11H1
5404 MR1
5405 NCEH1
5406 ALDOA
5407 SLC44A1
5408 POLR2C
5409 CHST9
5410 FFAR4
5411 SLC9B2
5412 TPMT
5413 OR1D5
5414 CYSLTR2
5415 PAOX
5416 CHST13
5417 AADACL2
5418 SELPLG
5419 KLK7
5420 EPM2A
5421 TSHR

5422 MMP7
5423 PTPN4
5424 LCN6
5425 HTR3B
5426 ZAR1
5427 OSTN
5428 P2RY10
5429 GYPB
5430 OR6C70
5431 ITGAL
5432 FSTL3
5433 OR13C4
5434 GUCY1A1
5435 ANGPTL2
5436 ADCY3
5437 F2
5438 GDNF
5439 HBEGF
5440 EIF2AK2
5441 FLVCR2
5442 FAT1
5443 MRGPRX3
5444 TPO
5445 FKBP10
5446 FLT4
5447 COL11A1
5448 NR1I3
5449 HAS3
5450 OCLN
5451 CLEC3A
5452 GLRB
5453 OR1J2
5454 KLRK1
5455 MID2
5456 LIPE
5457 HTR1D
5458 CDC14A
5459 RYR2
5460 PRLR
5461 OR5AK2
5462 CACNB4
5463 CBLN1
5464 SCG5
5465 TRIM22
5466 PSENEN
5467 PIK3CD
5468 FDPS
5469 LIFR

5470 INHBB
5471 AOC1
5472 KLRC2
5473 MLH3
5474 CAMK2G
5475 GPI
5476 DZANK1
5477 SLC6A10P
5478 FNTA
5479 OR4Q3
5480 SELL
5481 LYG1
5482 C1QTNF5
5483 GDF15
5484 ICMT
5485 EIF4H
5486 IFNA8
5487 FJX1
5488 VCP
5489 GBA2
5490 PPIAL4G
5491 AKR1C4
5492 CACNB2
5493 KIR2DS4
5494 FKBP9
5495 SLC6A4
5496 TRIM6
5497 PIN1
5498 NPFFR1
5499 RCE1
5500 SLC5A5
5501 VDR
5502 TSSK3
5503 EIF2AK1
5504 RAMP3
5505 KCNQ1
5506 GNRHR2
5507 OR5M10
5508 ATP1B1
5509 OR52N2
5510 CD160
5511 OR8U3
5512 OXT
5513 IGFBP7
5514 NEK3
5515 TMEM9B
5516 SLC22A6
5517 SERPINA2

5518 CST1
5519 CAPN9
5520 H1-5
5521 CADM3
5522 SERPINA9
5523 CA5B
5524 ADCY5
5525 SLC25A34
5526 HSPD1
5527 GGTLC3
5528 ADAMTS5
5529 CCL4L2
5530 AFP
5531 TST
5532 PTX3
5533 OR5D16
5534 MLN
5535 HSP90AA5P
5536 IL22
5537 PIP4K2B
5538 MASP2
5539 CACNA1D
5540 PDE6D
5541 OR9G4
5542 CAPNS1
5543 ECE2
5544 CD177
5545 OR2L2
5546 PAK5
5547 RBBP9
5548 KCNH1
5549 TACR3
5550 PRLHR
5551 XIAP
5552 SLCO1B3
5553 MET
5554 TMPRSS4
5555 CSF2RB
5556 GNRHR
5557 NPVF
5558 ENC1
5559 ALOXE3
5560 GZMK
5561 PAK4
5562 COL5A3
5563 TRIM16
5564 DEFB110
5565 VAT1L

5566 OR6C65
5567 PMPCB
5568 CNTFR
5569 AIPL1
5570 LCN1
5571 HRG
5572 LRRN1
5573 KCNA10
5574 MFSD1
5575 TUBB
5576 ABCB11
5577 SRPK1
5578 GGPS1
5579 TAAR5
5580 SERPINB5
5581 IGSF11
5582 PTHLH
5583 LGALS3BP
5584 ANKK1
5585 IGFL1
5586 SCN11A
5587 GABRA3
5588 PKLR
5589 CD40
5590 C4B
5591 CYP11A1
5592 CASP3
5593 RPS6KB1
5594 OR7G2
5595 CREBBP
5596 MECR
5597 PNLIPRP2
5598 MAST2
5599 FKBP3
5600 SCG3
5601 PI3
5602 LYZL6
5603 OR5M13P
5604 RARS1
5605 CPT2
5606 ERO1A
5607 GPR6
5608 SYCN
5609 ADRB2
5610 SPINK1
5611 BMPR1A
5612 RRM2B
5613 NAT2

5614 MANBA
5615 OR4F4
5616 PGGT1B
5617 APOA2
5618 KCNH4
5619 OPRD1
5620 PMS1
5621 IGF2R
5622 PSMA5
5623 TRPV5
5624 EPHX2
5625 CDA
5626 HDAC6
5627 NOS3
5628 PTPRO
5629 SLC10A2
5630 EHMT2
5631 WNT9A
5632 MVD
5633 FCER1A
5634 FKBP2
5635 GLA
5636 TMSB4X
5637 MAMDC2
5638 ARSH
5639 NTRK2
5640 COL23A1
5641 PDE6B
5642 ADCY10P1
5643 PPIL1
5644 TTBK2
5645 PRKAA1
5646 PNLIP
5647 CD247
5648 TNN
5649 TLL1
5650 TRIM46
5651 MSMB
5652 CPA1
5653 NDUFAF6
5654 SDR9C7
5655 CYP11B2
5656 CD83
5657 MPI
5658 FZD4
5659 OR13J1
5660 ULK4
5661 PRKX

5662 MRC1
5663 YAF2
5664 GSK3B
5665 WIF1
5666 GABRB1
5667 NENF
5668 CST7
5669 F11
5670 KDM4E
5671 PTGR1
5672 STK17B
5673 CHGA
5674 KIR2DL3
5675 KCNAB3
5676 SLAMF1
5677 CNOT6
5678 RORB
5679 CRAT
5680 CRB1
5681 SLC2A14
5682 ANTXR1
5683 TP73
5684 EBPL
5685 VIT
5686 CSF1R
5687 GPR160
5688 SPARCL1
5689 SCGB1D1
5690 HFE
5691 ALB
5692 PIK3C2G
5693 TNFRSF9
5694 OR5AZ1P
5695 BDH2
5696 CXCR4
5697 WDR1
5698 SLC49A3
5699 CACNB1
5700 ZP4
5701 TUBD1
5702 SLC46A3
5703 TMPRSS11E
5704 RHAG
5705 P2RX5
5706 PHEX
5707 NMB
5708 BCHE
5709 IL17RA

5710 CACNA1A
5711 PRPS1
5712 EPHA8
5713 ADH1C
5714 PLXNB1
5715 CCL3L1
5716 SCTR
5717 BCL2L2
5718 YARS1
5719 CTF1
5720 SLC22A12
5721 EBI3
5722 SFTPA1
5723 OR4F6
5724 COCH
5725 BCAN
5726 AURKC
5727 OR8B3
5728 LPAR6
5729 CHRNA1
5730 LHB
5731 IGLV3-27
5732 GABRQ
5733 TFF1
5734 KCNQ4
5735 PTPRE
5736 PLSCR4
5737 TRIM49C
5738 PSMB5
5739 CYB5RL
5740 ADGRG5
5741 AEBP1
5742 PLA2G3
5743 PHKG2
5744 LYZL1
5745 C4BPB
5746 NDNF
5747 FAAH2
5748 CDC25B
5749 KCNA1
5750 CACNG7
5751 SLC25A21
5752 HSD17B6
5753 MERTK
5754 SIGMAR1
5755 ZRANB1
5756 PRB2
5757 SLC41A2

5758	RBP3
5759	HLA-B
5760	CYP2E1
5761	CCND2
5762	CES2
5763	ABCC9
5764	HMGCR
5765	OR10J5
5766	KLHL22
5767	SLC4A4
5768	CYP4F12
5769	OR51F2
5770	HTRA3
5771	LRIT2
5772	WNT10A
5773	EPCAM
5774	SEMA3C
5775	PSMA8
5776	OR52I2

Table S3. The druggable genes identified from the review by Finn et al.

Number	Gene list
1	FGR
2	CFTR
3	CYP51A1
4	LAP3
5	CD38
6	FKBP4
7	CAMKK1
8	RECQL
9	SLC22A16
10	ABCB5
11	CALCR
12	MPO
13	PON1
14	ABCB4
15	ITGAL
16	ITGA2B
17	ABCC8
18	CACNA1G
19	TNFRSF12A
20	MAP3K9
21	TBXA2R
22	MARK4
23	NOS2
24	SLC13A2
25	MATK
26	CD79B
27	SCN4A
28	CACNA2D2
29	SELE
30	FMO3
31	PSMB1
32	CDKL5
33	MGST1
34	GIPR
35	NISCH
36	CD4
37	BTK
38	FYN
39	FMO1
40	MAP4K3
41	GABRA3
42	CD22
43	NR1H4
44	ALOX5
45	MAP4K5

46 CLK1
47 CD6
48 DPEP1
49 NPC1L1
50 CLCA1
51 CLCA4
52 IGF1
53 ATP1A2
54 CYP24A1
55 CD74
56 HGF
57 CYP3A43
58 CPS1
59 GABRA1
60 BIRC3
61 ABCC2
62 VIM
63 CD44
64 SLAMF7
65 PRKCH
66 IFNGR1
67 VRK2
68 MUSK
69 MAP2K3
70 DAPK2
71 CYP46A1
72 CASR
73 FLT4
74 TNC
75 TDP1
76 ADRB1
77 EPHA3
78 ROS1
79 HDAC9
80 TNFRSF9
81 SRD5A2
82 LAMC3
83 PTGER3
84 MAPK9
85 PIK3CB
86 LAMA3
87 KCNQ1
88 KCNH2
89 F7
90 LAMC2
91 CAMK2B
92 RIOK2
93 TBXAS1

94 HDAC7
95 GUCY1B3
96 TNK2
97 CDH3
98 LTK
99 POLD1
100 HAGH
101 CASP8
102 NGFR
103 HIPK2
104 LPAR2
105 CALCRL
106 PKN2
107 GLP2R
108 ERBB3
109 MYLK
110 MAP2K4
111 SLK
112 GSTO2
113 PRKCQ
114 CDK13
115 PDE4A
116 TIE1
117 SLC9A3
118 CLDN18
119 FGFR2
120 TNFRSF1A
121 PKM
122 PRKCZ
123 ROCK1
124 HDAC4
125 FGFR3
126 ABCC9
127 MAOB
128 DRD4
129 PLA2G10
130 GUCY2C
131 CHAT
132 CAMK2A
133 EPHA8
134 SLC12A3
135 MAP4K4
136 RPS6KA2
137 PRKACA
138 RPS6KA6
139 ALDH3A2
140 TFRC
141 HSD17B10

142 MARK2
143 FCGR2B
144 STK10
145 PDE8A
146 ABCB11
147 DHRS9
148 PTGS2
149 MAP3K13
150 TSG101
151 CA12
152 NUAK1
153 SLC12A1
154 TXK
155 KCNQ2
156 TACR2
157 FGF4
158 MARK3
159 PLD1
160 FMO4
161 ICAM3
162 MCAM
163 MAP2K7
164 RARB
165 TOP2B
166 IL4R
167 PAK3
168 TYR
169 FGFR1
170 FAP
171 MKNK1
172 FDFT1
173 KEAP1
174 EPHA6
175 SCTR
176 CHRNA3
177 PSEN1
178 HSP90AA1
179 AFP
180 PTPRC
181 CACNA1S
182 STK17B
183 SLC13A1
184 PGR
185 KCNK2
186 OPRK1
187 GSK3B
188 ITGB5
189 ULK2

190 ITGAE
191 GSTP1
192 SLCO1A2
193 APOB
194 MAP3K4
195 ABCB1
196 AKR1B1
197 MGST2
198 FOLH1
199 CEACAM6
200 HSD17B14
201 ACHE
202 CETP
203 MMP2
204 PTHLH
205 AURKA
206 SULT2B1
207 SLC15A1
208 FKBP1A
209 NOS1
210 GANAB
211 SLC9A1
212 ICAM1
213 IRAK3
214 SI
215 THPO
216 LAMB4
217 LAMB1
218 IL5RA
219 ABCC6
220 ESR1
221 ANGPT2
222 CD200
223 CMA1
224 MYH7
225 SLC7A8
226 SLC22A17
227 TYRO3
228 TGFB2
229 COMT
230 HDAC6
231 FMO2
232 PTGS1
233 PDE6C
234 CYP26A1
235 FKBP5
236 SRPK1
237 HSP90AB1

238 SIRT1
239 JAK2
240 IL12RB1
241 ABL1
242 NRP1
243 MADCAM1
244 MKNK2
245 MAPK1
246 SLC5A1
247 SLC5A4
248 CYP2D6
249 CYB5R3
250 ARSA
251 CACNA1I
252 IL2RB
253 HDAC10
254 KCNK10
255 GSTZ1
256 DHRS7
257 HIF1A
258 SLC10A1
259 BDKRB1
260 RPS6KA5
261 PSMB5
262 APEX1
263 DHRS2
264 GSS
265 MMP9
266 ABHD12
267 CD40
268 HNF4A
269 STK4
270 TUBB1
271 HRH3
272 SLCO4A1
273 NTSR1
274 CHRNA4
275 PTK6
276 CSNK2A1
277 MYLK2
278 HCK
279 PAK7
280 LAMA1
281 RIOK3
282 STS
283 POLA1
284 XIAP
285 CACNA1F

286 BMX
287 PIM2
288 CDK16
289 CD40LG
290 PORCN
291 GLA
292 HTR2A
293 TNFSF13B
294 MLNR
295 STK24
296 FLT1
297 DHRS12
298 MSLN
299 MAPK3
300 DHODH
301 MMP15
302 ABCC1
303 SLC7A5
304 SLC6A2
305 LACTB
306 CSK
307 CD276
308 SGK3
309 CA2
310 RIPK2
311 TRPA1
312 IKBKB
313 PLAT
314 STK3
315 SQLE
316 GSR
317 TNFRSF10A
318 MAN2B1
319 KCNN4
320 MAP4K1
321 TUBB4A
322 CD37
323 FCER2
324 DMPK
325 AURKC
326 CCNE1
327 DYRK1B
328 AKT2
329 PRKD2
330 TGFB1
331 CD33
332 CEACAM5
333 TYK2

334 SULT2A1
335 ATP1A3
336 GRIN2D
337 MAST1
338 JAK3
339 PDE4C
340 ATP4A
341 MAG
342 HAMP
343 GSK3A
344 GRIK5
345 CDK6
346 PIK3CG
347 PON3
348 PON2
349 MET
350 EPHB6
351 GHRHR
352 HSPB1
353 CYP3A5
354 IMPDH1
355 AHR
356 SLC1A1
357 TGFBR1
358 C5
359 ENG
360 TESK1
361 CA9
362 TYRP1
363 MAPK8
364 TNKS2
365 DKK1
366 CYP2C18
367 P2RX1
368 RPS6KB1
369 CHRNE
370 SLC6A4
371 ALDH3A1
372 CCL2
373 HSD17B1
374 HDAC5
375 ABCC3
376 MAP2K6
377 DHRS7B
378 PMP22
379 GABRA4
380 GNRHR
381 UGT2B10

382 SULT1E1
383 NFKB1
384 MAPK10
385 IL2
386 SOD3
387 DCPS
388 CCND1
389 CCKBR
390 FOLR1
391 BIRC2
392 HIPK3
393 CD5
394 SLC22A18
395 CALCA
396 VWF
397 CAMKK2
398 IL23A
399 CYP27B1
400 KRT18
401 METAP2
402 LTA4H
403 ALDH2
404 SCNN1A
405 LTBR
406 VDR
407 IFNG
408 SLCO1B3
409 FRK
410 MAK
411 PPARD
412 OPRM1
413 MAPK14
414 SOD2
415 IL17A
416 IL17F
417 ICK
418 GLP1R
419 ALDH5A1
420 GMNN
421 SLC22A2
422 PDE10A
423 CCND3
424 VEGFA
425 PRPF4B
426 TTK
427 SLC29A1
428 LAMA4
429 GHR

430 HBEGF
431 HMGCR
432 PDE8B
433 CLK4
434 ITK
435 IL12B
436 GABRG2
437 PDE4D
438 PRLR
439 IL4
440 IL5
441 FGF1
442 NR3C1
443 CSNK1A1
444 PDGFRB
445 HRH2
446 CD86
447 GRK7
448 BCHE
449 ABCC5
450 CLCN2
451 IL1A
452 ZAP70
453 GPD2
454 ACVR1
455 ITGB6
456 ITGA4
457 RTN4
458 TACR1
459 FN1
460 GGCX
461 CHST10
462 IL1R2
463 IL1R1
464 ABCB6
465 STK16
466 STK25
467 TPO
468 ODC1
469 PRKD3
470 SDC1
471 AAK1
472 GRIN3B
473 EPHA4
474 MARK1
475 GPX7
476 OPRD1
477 HDAC1

478 PLA2G4A
479 TNNI3K
480 CD2
481 KCNQ4
482 AKT3
483 PLA2G2D
484 APH1A
485 MPL
486 AKR1A1
487 FAAH
488 FMO6P
489 F3
490 TNFSF4
491 HSD11B1
492 SERPINC1
493 NEK2
494 RPS6KA1
495 CHRNB4
496 CTSD
497 STK11
498 KMT2A
499 MMP8
500 TTR
501 CA14
502 CNR1
503 ALDH8A1
504 CTGF
505 ABCG2
506 SPP1
507 CCND2
508 CYP20A1
509 CSF3R
510 PGF
511 TGFB3
512 ALDH6A1
513 ESRRB
514 DNMT3A
515 FKBP1B
516 EPCAM
517 CRHR1
518 TEK
519 CD274
520 IFNA6
521 IFNA8
522 GRIA2
523 TNFSF11
524 TNFRSF10B
525 PTK2B

526 CHRNA2
527 ADRA1A
528 EPHX2
529 TNFRSF8
530 ABCC11
531 IAPP
532 KCNJ8
533 CD80
534 CAT
535 HCRTR1
536 CCR2
537 GHSR
538 PIK3CA
539 ADORA3
540 CXCR4
541 FLT3
542 PLG
543 PTGFR
544 INHBA
545 CHST3
546 CIT
547 PKN1
548 EBPL
549 ATP7B
550 GUCY1B2
551 CDK2
552 AMD1
553 SERPINA7
554 RAB9A
555 MC3R
556 CHST8
557 ABCC10
558 NQO2
559 HIST1H1A
560 RIOK1
561 EREG
562 SLC10A2
563 ABCC4
564 PTGER2
565 GRK4
566 KIR2DL1
567 SRMS
568 OPRL1
569 IL1B
570 PLGLB2
571 GRIA3
572 CD70
573 TNFSF14

574 STK35
575 S1PR4
576 PSMB2
577 F10
578 FFAR1
579 THRA
580 PRKCG
581 AVPR2
582 MAP2K2
583 IL22
584 DYRK2
585 GFER
586 POR
587 SRD5A3
588 KDR
589 PPAT
590 ADORA2A
591 SMO
592 PDE11A
593 EIF2AK4
594 DLL4
595 ALDH1A2
596 SHBG
597 CCNT1
598 PARP2
599 EGLN3
600 SLC27A1
601 STK33
602 SULT4A1
603 CYP2E1
604 PAK4
605 LAMA5
606 MAP3K10
607 DNMT1
608 LATS1
609 ABCB7
610 SLC6A6
611 AOC3
612 CA6
613 TOP2A
614 RARA
615 FMO5
616 RAF1
617 PPARG
618 RAMP1
619 PRKAA1
620 DDC
621 SNAP25

622 SSTR4
623 ATP1A4
624 ALDH3B2
625 CDK8
626 ALOX5AP
627 CHRM3
628 DSTYK
629 DCLK1
630 CCNA1
631 EPHB2
632 GSTT2B
633 IMPA1
634 CA1
635 CCNB1
636 CDK7
637 GSTM1
638 GSTM5
639 GSTM3
640 NGF
641 ROCK2
642 IL6ST
643 IL2RA
644 HRH4
645 SLCO1B1
646 MTNR1B
647 CYP2J2
648 DAGLA
649 PDGFRA
650 CTSL
651 TAOK3
652 UGT2B28
653 SRPK2
654 HTR1B
655 NT5E
656 EPHA7
657 MAP3K7
658 CDK4
659 ACVR1B
660 TEC
661 MDM2
662 CHST5
663 EGLN1
664 GLUL
665 LAMC1
666 CHRND
667 HTR2B
668 CYP27A1
669 EDNRB

670 CHST12
671 GPNMB
672 IL6
673 SCN2A
674 SCN7A
675 BLK
676 IL10
677 CDK9
678 PRPF4
679 GABBR2
680 CTSV
681 IL11RA
682 ALDH1B1
683 PIM1
684 SLC22A7
685 HCRTR2
686 TUBB2A
687 RIPK1
688 TUBB2B
689 TPMT
690 SLCO2B1
691 PRCP
692 SLCO5A1
693 SULF1
694 MMP7
695 MMP13
696 CASP1
697 MAP2K5
698 PAK6
699 ADAM10
700 SLC28A2
701 CYP19A1
702 CLCA2
703 LHCGR
704 CYP1B1
705 SLC5A6
706 CYP2C9
707 CYP2C8
708 KIF11
709 AOX1
710 MSTN
711 CHRNA1
712 ITGAV
713 SLC40A1
714 APH1B
715 PRKG2
716 FGF2
717 BMPR1B

718 PDE5A
719 BMP2K
720 CDKL2
721 GSTCD
722 PPP3CA
723 MTTP
724 SLCO1C1
725 ACVRL1
726 CELA1
727 MAP3K12
728 ITGB7
729 SSTR1
730 ESR2
731 IGF1R
732 CYP11A1
733 CYP1A1
734 ULK3
735 CYP1A2
736 NTRK3
737 SLC5A2
738 MYLK3
739 ABCC12
740 CHST4
741 PDPK1
742 NPC1
743 MINK1
744 TP53
745 CSNK1D
746 PIP4K2B
747 ERBB2
748 PNMT
749 DPP9
750 IFNAR1
751 SOD1
752 SIK1
753 APP
754 AKT1
755 SLC6A3
756 SLC47A1
757 SLC2A5
758 EPHA2
759 PLK4
760 CYP4B1
761 ALDH9A1
762 GPA33
763 RXRG
764 MGST3
765 PVRL4

766 NR1I3
767 ABL2
768 RORC
769 CTSK
770 PIP5K1A
771 ARNT
772 IKBKE
773 TAF1A
774 PARP1
775 PSEN2
776 EPHX1
777 REN
778 PTPN7
779 GPR17
780 SCN1A
781 TRPM8
782 SLC22A14
783 NR1I2
784 AGTR1
785 EPHA5
786 GC
787 CAMK2D
788 CCNA2
789 SRD5A1
790 TSLP
791 IL9
792 GABRA6
793 GABRB2
794 GLRA1
795 MYLK4
796 PLA2G7
797 CYP39A1
798 TPBG
799 TAAR1
800 SLC22A3
801 EGFR
802 TLK2
803 EPHA1
804 CCNB3
805 HDAC8
806 CHST7
807 EBP
808 HTR2C
809 GPC3
810 ADHFE1
811 IFNA5
812 SIGMAR1
813 NTRK2

814 UGCG
815 PTGES
816 NOTCH1
817 CACNA1B
818 CAMK2G
819 HTR7
820 CYP17A1
821 GSTO1
822 ADAM12
823 PAK1
824 NCAM1
825 DRD2
826 HTR3B
827 GRIK4
828 SLC22A8
829 CHEK1
830 SLC22A9
831 MMP3
832 ITGB1
833 FCGR1A
834 LATS2
835 HNMT
836 PDCD4
837 ADRA2A
838 IL18
839 CACNA1C
840 THRB
841 MAT1A
842 EIF4E
843 FER
844 DRD3
845 EDNRA
846 NR3C2
847 PIGF
848 ADAM17
849 GABRA2
850 PDE3B
851 GRIA4
852 MBNL1
853 GRM1
854 MERTK
855 SCN3A
856 CLCA3P
857 CACNA2D1
858 CHST9
859 ANGPT1
860 PRKCA
861 TNIK

862 EPHB1
863 CDK19
864 SLC16A1
865 SLC7A7
866 GRIA1
867 NAT2
868 UGT2B4
869 MMP16
870 SLC28A1
871 FGF18
872 MAPK13
873 MS4A1
874 PHKG2
875 SST
876 GHRL
877 SLC6A1
878 HTR5A
879 MMP14
880 DHRS4
881 DHRS1
882 CACNA1D
883 KIT
884 CCNB2
885 DYRK1A
886 BRAF
887 SLC34A2
888 HPD
889 XDH
890 SLC13A3
891 HTR6
892 CDA
893 IFNAR2
894 IFNGR2
895 GART
896 SV2A
897 CBR1
898 CBR3
899 ALDH4A1
900 ACE
901 TNFRSF13C
902 PTGIR
903 ABCG1
904 ICOSLG
905 ITGB2
906 HIPK4
907 BRSK1
908 TAOK1
909 SIK3

910 CXCR5
911 IL6R
912 CHRNB2
913 FDPS
914 LMNA
915 CCR5
916 PTH1R
917 FGFR4
918 CYP3A4
919 CYP3A7
920 CYP11B1
921 PTGER1
922 GRIN2C
923 ITGA5
924 ALOX15
925 SSTR5
926 RPS6KA4
927 PRKAA2
928 JAK1
929 DHRS3
930 DDR2
931 NCSTN
932 CTSS
933 TGFA
934 GABRG1
935 GABRB1
936 HIPK1
937 SLC22A15
938 CCKAR
939 ATP1A1
940 SLC15A2
941 CXCR1
942 ADORA1
943 TGFBR2
944 HDAC11
945 NUAK2
946 PRKCI
947 FABP1
948 CTLA4
949 ICOS
950 DCLK3
951 CCR1
952 GRIK3
953 PRKCD
954 METAP1
955 MST1R
956 GRM2
957 NPY5R

958 IL15
959 ITGA2
960 HTR4
961 KLKB1
962 CSF2
963 GRIK2
964 DAGLB
965 STK17A
966 STEAP1
967 SHH
968 CTSB
969 ADCY1
970 PHKG1
971 GPER1
972 NOS3
973 CA3
974 CDK5
975 ALDH7A1
976 SYK
977 ABCA1
978 ALDH1A1
979 ATP7A
980 MELK
981 TSHR
982 SLC18A2
983 RET
984 CYP2C19
985 AVPR1A
986 GABRB3
987 PRKCB
988 MC4R
989 HTR3A
990 NNMT
991 PPIB
992 PLK1
993 UGT1A6
994 IGF2
995 RRM1
996 MMP26
997 VKORC1
998 CA4
999 GPX4
1000 DHRS13
1001 CYP2S1
1002 AXL
1003 PSCA
1004 DAPK3
1005 EEF2

1006 TRPV3
1007 TK1
1008 CYP7A1
1009 SOST
1010 SLC22A11
1011 MAP4K2
1012 PTGDR
1013 HIST1H1E
1014 SCN11A
1015 BDKRB2
1016 MTNR1A
1017 FNTA
1018 CHRM1
1019 STAT3
1020 ADAM9
1021 CA7
1022 GSTM4
1023 HTR1E
1024 GRM5
1025 MAP2K1
1026 AR
1027 DHRSX
1028 PCSK9
1029 IL13
1030 CA5B
1031 CXCL10
1032 ADRB2
1033 P2RY12
1034 PTK2
1035 PTAFR
1036 NPR1
1037 KCNK9
1038 IL8
1039 SCN9A
1040 CD52
1041 DRD5
1042 FASN
1043 LINGO1
1044 TACR3
1045 MAP3K2
1046 SIK2
1047 CHRNB1
1048 ANKK1
1049 ADRA1B
1050 CDK1
1051 KRT8
1052 ADORA2B
1053 MGMT

1054 CD14
1055 CDH2
1056 FSHR
1057 GSTA4
1058 CEACAM3
1059 S1PR1
1060 ALK
1061 INSR
1062 PRKCE
1063 GRIK1
1064 UGT2B7
1065 GAA
1066 KCNK3
1067 CHST11
1068 KRT19
1069 PDE7B
1070 NAT1
1071 PPID
1072 PTGER4
1073 BCL2L1
1074 FGG
1075 FGA
1076 FGB
1077 PIK3CD
1078 HDAC3
1079 PAH
1080 BCL2
1081 RRM2
1082 ADRA1D
1083 CYP4F11
1084 LAMB2
1085 SMN1
1086 CCL11
1087 BSG
1088 PDE3A
1089 CYP7B1
1090 RARG
1091 CES2
1092 EGFL7
1093 DHCR7
1094 SLC22A13
1095 ADH6
1096 ADRBK1
1097 ESRRA
1098 ADCY5
1099 CYSLTR1
1100 MAP3K11
1101 VEGFB

1102 CCR9
1103 SULT1B1
1104 UGT2A1
1105 SLC19A1
1106 SLCO4C1
1107 GSTA3
1108 SELP
1109 TNK1
1110 TRHR
1111 UGT8
1112 SLCO2A1
1113 SLC29A2
1114 BRSK2
1115 HRAS
1116 CD248
1117 CA5A
1118 SLC22A1
1119 CHST2
1120 NPPA
1121 CHST1
1122 CCNE2
1123 CHRNA7
1124 PNLIP
1125 P2RY2
1126 AURKAIP1
1127 TUBB6
1128 YES1
1129 MC5R
1130 GPX2
1131 HSD11B2
1132 CLK2
1133 SLCO3A1
1134 MAP3K19
1135 CDK5R1
1136 GRIN1
1137 TYMS
1138 POLE
1139 ULK1
1140 RPS6KA3
1141 CD19
1142 GBA
1143 IL17RA
1144 IMPDH2
1145 HTR3C
1146 HTR1A
1147 P4HTM
1148 CD28
1149 ERBB4

1150 ERN1
1151 GPR35
1152 GAK
1153 AURKB
1154 HTR1F
1155 CYP11B2
1156 HTR1D
1157 GRM8
1158 GPBAR1
1159 TH
1160 F2
1161 PAK2
1162 CYP8B1
1163 SSTR2
1164 SLC47A2
1165 CHRM4
1166 S1PR5
1167 CHST13
1168 AGTR2
1169 MAP3K15
1170 CXCR2
1171 OXTR
1172 NQO1
1173 CHRM2
1174 MAPK15
1175 F2R
1176 MUC16
1177 UTS2R
1178 SLC2A4
1179 GABRG3
1180 LIPF
1181 LIMK2
1182 CSF1R
1183 EPHB3
1184 HCAR2
1185 GSTA5
1186 LCK
1187 ABAT
1188 CAMK1D
1189 PTGDR2
1190 CHST6
1191 EPHA10
1192 RIPK4
1193 GRIN2A
1194 SSTR3
1195 TBK1
1196 CHEK2
1197 CCR4

1198 SCN5A
1199 PRKX
1200 KCNQ3
1201 ADRA2C
1202 IRAK1
1203 ALDH1A3
1204 PRKD1
1205 SRPK3
1206 HIST1H1B
1207 CSF1
1208 TOP1MT
1209 DHRS7C
1210 PDE4B
1211 GSTT1
1212 DRD1
1213 WT1
1214 CHRM5
1215 SLC22A10
1216 F8
1217 CA13
1218 MC2R
1219 IL3RA
1220 SCN10A
1221 MAPK11
1222 MUC1
1223 L3MBTL1
1224 DLK1
1225 PMEL
1226 PRAME
1227 KCNQ5
1228 GRK1
1229 ATP4B
1230 HTR3E
1231 HTR3D
1232 CYP2R1
1233 CYP4F2
1234 CYP4Z1
1235 CYP4F12
1236 GABRA5
1237 BACE1
1238 RXRA
1239 CYP4F8
1240 CYP4F3
1241 BCR
1242 KCNK18
1243 IFNA10
1244 TNFRSF4
1245 MAPT

1246 TNFRSF18
1247 PPARA
1248 RHD
1249 CYP4A11
1250 AKR1C1
1251 NPSR1
1252 EPOR
1253 KCNJ11
1254 CYP26C1
1255 DHRS4L2
1256 ADH1A
1257 HIST1H1C
1258 MAPK12
1259 TUBB4B
1260 PLA2G2A
1261 IFNA2
1262 PDCD1
1263 SELL
1264 DPYD
1265 ADRB3
1266 PLA2G2E
1267 CNR2
1268 LRRK2
1269 KIR2DL4
1270 H1F0
1271 SF3B3
1272 MAOA
1273 HLA-DRB1
1274 MYT1
1275 AKR1C3
1276 CTSE
1277 RYR1
1278 TUBB
1279 PPIA
1280 IARS
1281 ADH7
1282 CD55
1283 EPHB4
1284 FGF16
1285 NCOR2
1286 SULT1A1
1287 SLC6A9
1288 MME
1289 CACNA1H
1290 LAMA2
1291 HDAC2
1292 MMP1
1293 ADH1B

1294 UGT2B15
1295 HRH1
1296 TLR7
1297 TRPV1
1298 DAPK1
1299 CHRNG
1300 ADA
1301 SCN8A
1302 LAMB3
1303 SRC
1304 ABCB8
1305 SULT1A2
1306 SLC22A4
1307 BLM
1308 SLC22A5
1309 HTT
1310 CYP2B6
1311 MAP3K5
1312 CYP2F1
1313 GSTK1
1314 SLC28A3
1315 ELANE
1316 MYH6
1317 DPP4
1318 PDCD1LG2
1319 CFD
1320 CYP2A13
1321 UGT2B17
1322 SLC22A12
1323 ADH5
1324 SLC22A6
1325 IFNA1
1326 IRAK4
1327 AVPR1B
1328 AKR1B10
1329 CYP2A7
1330 ADH4
1331 LPAR1
1332 HSD17B11
1333 SULT1C2
1334 CSF2RA
1335 PIM3
1336 NTRK1
1337 TXNRD1
1338 HLA-DRB5
1339 MMP17
1340 ABCA4
1341 GPX6

1342 F5
1343 GRIN3A
1344 MTOR
1345 PNP
1346 GRM3
1347 RYR3
1348 CES1
1349 CD3E
1350 TOP1
1351 MAP3K3
1352 DMD
1353 DDO
1354 HSD3B2
1355 RXRB
1356 PSMB8
1357 HLA-DRA
1358 SLC44A4
1359 ABHD16A
1360 DDR1
1361 GABBR1
1362 PRSS1
1363 PSENEN
1364 PDE7A
1365 SLCO6A1
1366 SMN2
1367 CRLF2
1368 HLA-A
1369 GPX3
1370 DIO1
1371 TRGV3
1372 TRBV7-1
1373 TRAV29DV5
1374 IGHE
1375 TSSK1B
1376 CHUK
1377 GSTM2
1378 S1PR3
1379 UGT2B11
1380 LTB4R
1381 CSNK1E
1382 IFNA7
1383 WEE2
1384 MUC5AC
1385 GCGR
1386 VAMP2
1387 MAGEA3
1388 ADRA2B
1389 LTA

1390 LTB
1391 DHFR
1392 TRAC
1393 TNF
1394 GPX1
1395 IFNA4
1396 TNFSF12
1397 TLR9
1398 UGT1A5
1399 UGT1A9
1400 TDGF1
1401 UGT1A8
1402 UGT1A1
1403 UGT1A10
1404 UGT1A3
1405 AMY2A
1406 KIR2DL3
1407 GSTA1
1408 GSTA2
1409 UGT1A7
1410 UGT1A4
1411 ADH1C
1412 LYN
1413 CYP2A6
1414 PGA5
1415 MGAM
1416 FNTB
1417 PDF
1418 MC1R
1419 ITGB3
1420 SULT1A3
1421 S1PR2
1422 EGLN2
1423 C8orf44-SGK3
1424 GRIN2B
1425 FUCA2
1426 GCLC
1427 BAD
1428 AOC1
1429 KDM1A
1430 CREBBP
1431 MAP3K14
1432 DBF4
1433 CDKL3
1434 NOX1
1435 CAMK1G
1436 CDK11A
1437 MMP25

1438 DYRK4
1439 SLC6A13
1440 PRSS3
1441 PTBP1
1442 PIK3C2A
1443 PLAUR
1444 PPP5C
1445 BRCA1
1446 CAPN1
1447 ACPP
1448 NR1H3
1449 TYMP
1450 INSRR
1451 NR2E3
1452 UBA6
1453 OTC
1454 SLC18A1
1455 USP2
1456 PARP3
1457 HSPA5
1458 UTS2
1459 RAD51
1460 EIF2AK2
1461 MCOLN3
1462 NPFFR2
1463 SOAT1
1464 CDK14
1465 CDK17
1466 BCAT1
1467 CTSA
1468 SLC12A2
1469 ABCA7
1470 KARS
1471 ME1
1472 ELAVL1
1473 KDM4A
1474 SIRT2
1475 PYGM
1476 MAPK6
1477 POLB
1478 GBA2
1479 TESK2
1480 CSNK2A2
1481 CDC42
1482 MYO3B
1483 IKBKG
1484 SCARB1
1485 GLI2

1486 MGLL
1487 DPP8
1488 ATP12A
1489 ACACB
1490 NAALAD2
1491 ARAF
1492 PIK3C3
1493 ADCYAP1R1
1494 SLC1A3
1495 LIPE
1496 CPB2
1497 KCNN2
1498 PPIE
1499 CAD
1500 PREP
1501 DNAJA1
1502 EIF2AK1
1503 HSD17B2
1504 NOX4
1505 NLK
1506 DNMT3B
1507 F11
1508 MAPKAPK5
1509 P2RX7
1510 RBBP9
1511 BIRC5
1512 RAPGEF4
1513 CPA1
1514 TGM1
1515 SNAP23
1516 MAP3K1
1517 MYO3A
1518 CDC7
1519 SCD
1520 MTAP
1521 XBP1
1522 TSPO
1523 ACR
1524 TAB1
1525 EP300
1526 CTSG
1527 GZMB
1528 CDKL1
1529 PYGL
1530 NFKBIA
1531 SGK2
1532 CTSZ
1533 CDC25B

1534 HAO1
1535 AHCY
1536 LIPG
1537 TLR8
1538 SUV39H1
1539 GLRA2
1540 F9
1541 SMS
1542 GPR50
1543 BRS3
1544 KLF5
1545 PARP4
1546 MLYCD
1547 EEF2K
1548 CTSH
1549 SFRP1
1550 DOT1L
1551 SLC1A6
1552 PTPRS
1553 LIG1
1554 SIGLEC6
1555 SIGLEC5
1556 KCNN1
1557 NAMPT
1558 VIPR2
1559 NOD1
1560 CASP2
1561 SERPINE1
1562 GCK
1563 EIF4H
1564 LIMK1
1565 KDM4C
1566 PKD2L1
1567 BMPR1A
1568 LIPA
1569 MAP3K8
1570 PPIF
1571 WNT3
1572 GALK1
1573 MLX
1574 ALOX12
1575 DUSP3
1576 AREG
1577 MANBA
1578 AADAT
1579 CTSC
1580 P2RX3
1581 CPT1A

1582 SLC1A2
1583 ASIC1
1584 DAO
1585 SLC11A2
1586 TRPV4
1587 GAPDH
1588 LDHB
1589 PRKAB1
1590 GABRR2
1591 STK38
1592 PTP4A1
1593 CCR6
1594 SENP6
1595 GRM6
1596 TARS
1597 LNPEP
1598 KAT2B
1599 UMPS
1600 NEK11
1601 MAPKAPK3
1602 ACVR2B
1603 VIPR1
1604 TNNC1
1605 NKTR
1606 NEK4
1607 TTL
1608 PSMD14
1609 PDE1A
1610 STAT1
1611 IGFBP2
1612 IGFBP5
1613 SLC5A7
1614 PASK
1615 QPCT
1616 KYNU
1617 KISS1R
1618 EPAS1
1619 ATP6V1B1
1620 SCP2
1621 ICMT
1622 AMPD2
1623 SRM
1624 AMPD1
1625 HAO2
1626 KMO
1627 RGS4
1628 CDK18
1629 ECE1

1630 GALE
1631 SLC2A1
1632 SLC5A9
1633 TNNT2
1634 SGK1
1635 PKD2
1636 UCHL3
1637 TRPM6
1638 NEK6
1639 NEK9
1640 IDE
1641 ELOVL3
1642 CPN1
1643 APAF1
1644 KHDRBS1
1645 TNFSF10
1646 ACVR2A
1647 SV2C
1648 XPNPEP2
1649 PLAU
1650 CISD1
1651 ATF1
1652 NR4A1
1653 PDE1B
1654 DBH
1655 KCNJ2
1656 ADCK4
1657 DNPEP
1658 SLC12A5
1659 NCOA3
1660 PCK1
1661 PEPD
1662 F13A1
1663 GRM4
1664 CDKN1A
1665 GLO1
1666 BMP4
1667 TGM3
1668 KDM5C
1669 CAPNS1
1670 UBA2
1671 FFAR2
1672 NR1D1
1673 STAT5A
1674 TRAP1
1675 PTPRB
1676 PIN1
1677 PLA2G5

1678 PKMYT1
1679 PTPN12
1680 MCHR1
1681 GALR3
1682 APOBEC3A
1683 DUT
1684 TPH1
1685 PLD2
1686 SIGLEC9
1687 BCL2L2
1688 TNNI3
1689 KCNA5
1690 LDLR
1691 ACE2
1692 SLC6A8
1693 HSD17B3
1694 IDO1
1695 NR1H2
1696 ACLY
1697 AOC2
1698 G6PC
1699 ACACA
1700 SLC6A11
1701 HSD17B7
1702 PTPRA
1703 BHMT2
1704 CASP9
1705 PDE6A
1706 PIK3C2B
1707 CSNK1G2
1708 LARS
1709 AMPD3
1710 CAMK1
1711 CHIA
1712 PTPN22
1713 LDHA
1714 CCNH
1715 YARS
1716 APLNR
1717 ERCC5
1718 P2RX4
1719 HNRNPA1
1720 NMBR
1721 SMPD2
1722 CPM
1723 GPR55
1724 NEK3
1725 NMT1

1726 TXN
1727 TLR4
1728 FPGS
1729 NR5A1
1730 ENPP2
1731 PLAA
1732 PPIL1
1733 GGH
1734 NEK1
1735 MMP20
1736 MMP27
1737 BCL2L10
1738 BRDT
1739 KHK
1740 PPM1B
1741 RBP4
1742 ATIC
1743 CDK15
1744 PPIG
1745 SENP7
1746 HCN4
1747 NAAA
1748 ENPEP
1749 CASP6
1750 PIK3C2G
1751 VAMP1
1752 TPH2
1753 RB1
1754 SORD
1755 HDC
1756 BCL2A1
1757 MBTPS1
1758 IMPA2
1759 IGFBP4
1760 CACNA1A
1761 BRD4
1762 SIRT3
1763 HUNK
1764 SAE1
1765 CARM1
1766 KLK3
1767 PADI3
1768 PADI1
1769 PGD
1770 MAP3K6
1771 PRKACB
1772 ADC
1773 PTPRF

1774 PPOX
1775 MCL1
1776 PI4KB
1777 DYRK3
1778 KCNN3
1779 PKLR
1780 ACP1
1781 CDC42BPA
1782 CALM2
1783 PTH2R
1784 ACKR3
1785 CTDSP1
1786 NCEH1
1787 SCD5
1788 SNCA
1789 FABP2
1790 GLRA3
1791 PLK2
1792 PIK3R1
1793 BHMT
1794 PAM
1795 FARS2
1796 GABRR1
1797 IGFBP3
1798 IGFBP1
1799 CASK
1800 GPR119
1801 CHRNB3
1802 CHRNA6
1803 NPFFR1
1804 PAOX
1805 ATM
1806 ST14
1807 ADAM33
1808 TAOK2
1809 CSNK1G3
1810 NEK7
1811 QDPR
1812 AKR1C2
1813 ADAM8
1814 KCNJ1
1815 TDO2
1816 MCHR2
1817 PDK1
1818 CAMK4
1819 RASGRP3
1820 GGPS1
1821 STK32B

1822 CPB1
1823 RANBP2
1824 NR4A2
1825 TMPRSS11D
1826 SLC5A10
1827 UCHL1
1828 PDE1C
1829 ADAMTS1
1830 ADAMTS5
1831 AZIN1
1832 ADK
1833 KCNMA1
1834 DCK
1835 HKDC1
1836 SMG1
1837 CPT2
1838 ERG
1839 PSMG3
1840 CDC25C
1841 PLA2G2F
1842 ADAMTS4
1843 SLC5A11
1844 HLCS
1845 C1R
1846 NAE1
1847 RGS12
1848 CALM3
1849 PDE9A
1850 G6PD
1851 LSS
1852 S100B
1853 CCL5
1854 EIF4A1
1855 SLC1A7
1856 CTRC
1857 ALPL
1858 VCAM1
1859 NLRP3
1860 KCNJ9
1861 OXER1
1862 MAPKAPK2
1863 CAPN2
1864 ADCK3
1865 BIRC8
1866 HPGDS
1867 ALPPL2
1868 ALPI
1869 STK36

1870 PTPN13
1871 ALB
1872 CPA3
1873 SNRK
1874 TKT
1875 CDC25A
1876 GUCY1A3
1877 HPGD
1878 NPY1R
1879 PGGT1B
1880 F2RL1
1881 CASP3
1882 TERT
1883 HCN1
1884 FBP1
1885 CYBB
1886 VCP
1887 FAAH2
1888 HPRT1
1889 STK32C
1890 CASP7
1891 PNLIPRP2
1892 SLC6A5
1893 SENP8
1894 WEE1
1895 MAPK7
1896 TK2
1897 HSP90B1
1898 MMP10
1899 ANPEP
1900 SCNN1G
1901 STAT6
1902 PIP4K2C
1903 TGM6
1904 SMAD3
1905 MARS
1906 SLC27A4
1907 NOD2
1908 DPEP2
1909 ENGASE
1910 MVD
1911 KLK1
1912 IGFBP6
1913 SOAT2
1914 CTNNB1
1915 PBK
1916 CX3CR1
1917 ATG4B

1918 SCNN1B
1919 BMP1
1920 FEN1
1921 AXIN2
1922 CTRB1
1923 PPIC
1924 CSNK1G1
1925 STK32A
1926 PROKR1
1927 CHRNA5
1928 AGPAT2
1929 P2RY1
1930 BRD3
1931 YWHAG
1932 USP47
1933 MRGPRX1
1934 GLB1
1935 FABP4
1936 FOS
1937 DCLK2
1938 ELOVL6
1939 CEL
1940 PLA2G1B
1941 FPR2
1942 FPR1
1943 CDC42BPG
1944 RXFP1
1945 LPAR3
1946 P2RY6
1947 C3AR1
1948 RNASEH1
1949 PPIH
1950 EIF2AK3
1951 TPSAB1
1952 PPP1CA
1953 OXSR1
1954 PHF8
1955 RELA
1956 HPSE
1957 KDM2A
1958 LIPM
1959 TNKS
1960 FKBP2
1961 RCE1
1962 STAT5B
1963 KCNH6
1964 PLK3
1965 PHOSPHO1

1966 TUBB8
1967 CTSF
1968 CMKLR1
1969 NR1D2
1970 AMY1B
1971 ATR
1972 PTPN2
1973 LPL
1974 RPS6KB2
1975 SPHK1
1976 RNPEP
1977 DPP7
1978 MAN1B1
1979 KCNA3
1980 NIM1K
1981 NR2C2
1982 GSG2
1983 JUN
1984 UBE2N
1985 RPS27
1986 DHFRL1
1987 MPI
1988 FUCA1
1989 PTPN11
1990 CLK3
1991 CCR8
1992 CSNK1A1L
1993 KCNE1
1994 PRF1
1995 EHMT1
1996 NPM1
1997 BACE2
1998 C1S
1999 FES
2000 KCNB2
2001 CCR3
2002 UPP1
2003 NPBWR1
2004 PTP4A2
2005 KCNJ12
2006 PLA2G6
2007 TXNRD2
2008 LPAR5
2009 KCNH7
2010 DGAT1
2011 NPY2R
2012 PRMT3
2013 SV2B

2014 PRKG1
2015 P4HB
2016 PIP5K1C
2017 FFAR4
2018 PDE2A
2019 CXCR3
2020 PNLIPRP1
2021 MITF
2022 USP7
2023 TRPV2
2024 SLC18A3
2025 AMY1C
2026 P2RX2
2027 PLA2G2C
2028 CLCN1
2029 SBK1
2030 GLRA4
2031 NDUFA4
2032 GRM7
2033 WDR5
2034 PTPN1
2035 ESRRG
2036 WNK3
2037 NEK5
2038 C5AR1
2039 TXNRD3
2040 PLCG2
2041 PPIAL4C
2042 TUBB3
2043 FKBP1C
2044 GFPT1
2045 MGEA5
2046 TLK1
2047 RYR2
2048 STK39
2049 CALM1
2050 MT-CO2
2051 SLC5A3
2052 CDC42BPB
2053 SUCNR1
2054 GRK5
2055 MT-ND4
2056 SREBF2
2057 TGM2
2058 HSD3B1
2059 LIPN
2060 LIPK
2061 LIPJ

2062 BMPR2
2063 BRD2
2064 EHMT2
2065 HSPA1B
2066 HSPA1A
2067 AKT1S1
2068 CPT1B
2069 AREGB
2070 TSSK2
2071 STK38L
2072 ASIC3
2073 LTB4R2
2074 PPME1
2075 DDX3X
2076 PSMB11
2077 PGA4
2078 PGA3
2079 SBK3
2080 KDM4E
2081 PPIAL4G
2082 AMY1A
2083 APOBEC3G
2084 AMY2B
2085 PPIL3
2086 MIF
2087 PLA2G4B
2088 HBB
2089 CDK11B
2090 CDK3
2091 PRKDC
2092 NOX5
2093 HCAR3
2094 PPIAL4B
2095 PPIAL4A
2096 PPIAL4D
2097 GPR142
2098 BCL2L2-PABPN1
2099 CFH
2100 WNT16
2101 M6PR
2102 TFPI
2103 SLC4A1
2104 CRLF1
2105 PROM1
2106 IL32
2107 TENM1
2108 MASP2
2109 CD9

2110 SEMA3G
2111 HFE
2112 MRC2
2113 SLC6A7
2114 LTF
2115 CNTN1
2116 VSIG2
2117 NRXN3
2118 ERP44
2119 FAS
2120 RNASET2
2121 IBSP
2122 GRN
2123 TIMP2
2124 AGA
2125 VCAN
2126 CDH1
2127 C6
2128 RTN4R
2129 USH2A
2130 TG
2131 ADAM28
2132 CP
2133 TNFRSF17
2134 KITLG
2135 ADAMTS6
2136 LTBP1
2137 DKK3
2138 COL23A1
2139 PRSS8
2140 FSTL4
2141 GALC
2142 ITIH4
2143 ITIH1
2144 ATP2B4
2145 WNK1
2146 COL11A1
2147 WNT8A
2148 WISP2
2149 NTN1
2150 COL17A1
2151 ATP2B3
2152 HYAL2
2153 RORA
2154 TGFBR3
2155 SCT
2156 NUCB2
2157 FGF10

2158 MGAT4A
2159 WDR1
2160 PRLH
2161 LPHN1
2162 ACTN1
2163 TRPC5
2164 PVR
2165 CD5L
2166 ST6GAL1
2167 ATP2A3
2168 NTN4
2169 SEMA3A
2170 SEMA3C
2171 FBLN1
2172 ITGA8
2173 LAMP3
2174 AFM
2175 SCGN
2176 CFHR2
2177 CXCL2
2178 COL4A4
2179 PLOD1
2180 CD82
2181 WNT11
2182 B4GALT1
2183 EPDR1
2184 ADAMTS2
2185 NID2
2186 GNAS
2187 EDEM2
2188 DEFB127
2189 CPXM1
2190 SIRPG
2191 OAS1
2192 CHGB
2193 ERP29
2194 CHRD
2195 FLT3LG
2196 DLD
2197 ITGA6
2198 TF
2199 APOH
2200 CCDC80
2201 TRPM7
2202 COL9A3
2203 NXPE1
2204 TLL2
2205 CRTAC1

2206 IL11
2207 NCR2
2208 MLN
2209 PCSK5
2210 MAST3
2211 IGFALS
2212 GGT1
2213 MFNG
2214 PIK3IP1
2215 HMOX1
2216 PDGFB
2217 APOL1
2218 CSF2RB
2219 GZMH
2220 SERPINA4
2221 VRK1
2222 PLTP
2223 PROCR
2224 COL20A1
2225 ANGPT4
2226 SIRPB1
2227 PDYN
2228 JAG1
2229 BPI
2230 CST9L
2231 CST3
2232 WFDC2
2233 SPINT3
2234 CHRDL1
2235 ACP5
2236 FGF9
2237 HAS3
2238 PLA2G15
2239 CRISPLD2
2240 METRN
2241 IL21R
2242 SCG3
2243 CPQ
2244 WISP1
2245 IL7
2246 FGL1
2247 FCGRT
2248 AMH
2249 DKKL1
2250 RETN
2251 OLFM2
2252 ICAM4
2253 FGF21

2254 LILRB5
2255 SCN1B
2256 TFPI2
2257 WNT2
2258 FKBP14
2259 PCOLCE
2260 SFRP4
2261 AEBP1
2262 FKTN
2263 PTGDS
2264 ABCA2
2265 CXCL12
2266 C10orf54
2267 SPOCK2
2268 MINPP1
2269 CSF3
2270 ASIC2
2271 CCL7
2272 COL1A1
2273 PPY
2274 EFTUD2
2275 EFNB3
2276 NMU
2277 CPE
2278 STIM2
2279 BST1
2280 HGFAC
2281 SIAE
2282 NRXN2
2283 ST3GAL4
2284 APOA5
2285 APOA4
2286 APOC3
2287 IL10RA
2288 MDK
2289 CD69
2290 SELPLG
2291 WNT5B
2292 FZD10
2293 IL26
2294 RAB35
2295 BTN3A3
2296 ULBP1
2297 TULP1
2298 CD83
2299 GPLD1
2300 SMOC2
2301 LY86

2302 LIFR
2303 C9
2304 KNG1
2305 HRG
2306 WNT5A
2307 FGF12
2308 HYAL1
2309 PLSCR4
2310 CCL20
2311 POMC
2312 GCG
2313 EFEMP1
2314 WNT6
2315 IL1RL1
2316 IL18R1
2317 IL18RAP
2318 PROC
2319 LCT
2320 PLEK
2321 TNR
2322 PAPPA2
2323 CAPZA1
2324 LEPR
2325 CFHR3
2326 CD58
2327 NR5A2
2328 NID1
2329 ZP4
2330 SLAMF1
2331 CD48
2332 MFAP2
2333 CD160
2334 CR2
2335 CD46
2336 APOA1
2337 RPN2
2338 CASQ2
2339 LTBP2
2340 INSL6
2341 INSL4
2342 TNN
2343 KCNJ5
2344 ENOX1
2345 CLU
2346 NPPB
2347 SCPEP1
2348 PLBD1
2349 PRB2

2350 CRY2
2351 CD244
2352 LY9
2353 SPINK4
2354 ITIH5
2355 LRP1
2356 TUBA1B
2357 TNFAIP6
2358 PLA2G12A
2359 C4BPA
2360 C4BPB
2361 SLC12A4
2362 SDC4
2363 PIGT
2364 MATN4
2365 HIST1H4B
2366 BTN1A1
2367 HIST1H3B
2368 TREM1
2369 SSR1
2370 IL37
2371 C3
2372 DEFB126
2373 CD93
2374 BMP2
2375 PCSK2
2376 GDF5
2377 MMP24
2378 ST3GAL3
2379 PROZ
2380 CCR7
2381 PZP
2382 FGFRL1
2383 WFIKKN1
2384 IL17B
2385 TNFRSF19
2386 ADM2
2387 IGLL1
2388 LIF
2389 VGF
2390 TWSG1
2391 LOXL1
2392 CD68
2393 RNASE1
2394 INS-IGF2
2395 LBP
2396 PVRL2
2397 APOE

2398 APOC1
2399 NCAN
2400 EPO
2401 PXDN
2402 GDF15
2403 COL5A1
2404 ANGPTL6
2405 ULBP2
2406 LILRB2
2407 PYY
2408 F12
2409 NAPSA
2410 NDFIP1
2411 VIMP
2412 CHSY1
2413 LGALS3
2414 MATN3
2415 EMILIN2
2416 SERPINF1
2417 MATN2
2418 CRP
2419 FCRL2
2420 ANGPTL3
2421 CHI3L1
2422 CHIT1
2423 TRPC4
2424 TPT1
2425 PDZD2
2426 C1QTNF6
2427 SFTPD
2428 PEBP4
2429 CD180
2430 CHL1
2431 TRPM1
2432 PTGFRN
2433 NOTCH2
2434 SAA2
2435 CFHR4
2436 CRB1
2437 CFHR5
2438 IL15RA
2439 KLRC1
2440 ACP2
2441 GIF
2442 TCN1
2443 COL4A2
2444 ADAMTS8
2445 ANXA1

2446 CD36
2447 CD63
2448 LACRT
2449 ITGA7
2450 MIP
2451 CD164
2452 KCNK1
2453 WNT10A
2454 EDAR
2455 AOAH
2456 ADAMTS7
2457 CHAD
2458 GH2
2459 CSH1
2460 IL36G
2461 IL1RN
2462 IL36RN
2463 IL36B
2464 IL1F10
2465 NOV
2466 IL33
2467 CCL21
2468 IGFBPL1
2469 TINAG
2470 FGFBP2
2471 THBS1
2472 ITGA11
2473 MSMB
2474 ADAMTS14
2475 SEMA7A
2476 FGF5
2477 IL21
2478 MMRN1
2479 CXCL9
2480 EGF
2481 FBN2
2482 TAPBPL
2483 CD27
2484 COL2A1
2485 INHBE
2486 LUM
2487 KERA
2488 DHH
2489 NPFF
2490 CBLN3
2491 FBLN5
2492 SERPINA10
2493 FGF7

2494 ADAMTS17
2495 PCSK6
2496 HAPLN3
2497 MFGE8
2498 FURIN
2499 ITGAX
2500 ADAMTS18
2501 CDH13
2502 GALNS
2503 KSR1
2504 CTRL
2505 GFOD2
2506 ADCYAP1
2507 ASGR1
2508 TNFRSF11A
2509 COL6A1
2510 IL19
2511 ADAMTS10
2512 MMEL1
2513 CELA2A
2514 CD53
2515 PROK1
2516 DPT
2517 FCGR2A
2518 F13B
2519 FCRL5
2520 HDGF
2521 S100A7
2522 GALNT2
2523 GDF7
2524 PDIA6
2525 TMEFF2
2526 SUMF1
2527 CNTN4
2528 ITGA9
2529 PTPRG
2530 COL8A1
2531 BOC
2532 TRPC1
2533 SLIT2
2534 CORIN
2535 SFRP2
2536 PDGFC
2537 HAPLN1
2538 ADAMTS19
2539 LECT2
2540 PCYOX1L
2541 GFRA3

2542 TNFRSF21
2543 RSPO3
2544 IFNK
2545 GSN
2546 CRB2
2547 LCN9
2548 ADM
2549 SAA4
2550 SCGB1A1
2551 SERPING1
2552 PTPRJ
2553 ESAM
2554 SIDT2
2555 PCDH15
2556 CTF1
2557 GPM6A
2558 CD226
2559 CRIM1
2560 ITPR1
2561 PRSS53
2562 LYZL2
2563 ADAMTS12
2564 CCDC3
2565 ITIH2
2566 BICD1
2567 GUCY1A2
2568 MEPE
2569 BMP3
2570 MR1
2571 BMP6
2572 PLA2R1
2573 CD96
2574 CD8A
2575 KCNJ16
2576 ENPP3
2577 JAM2
2578 WNT7A
2579 DKK2
2580 TTN
2581 WIF1
2582 ADAMTS3
2583 ADAMTSL3
2584 ITGAD
2585 ATP2B2
2586 LYZL4
2587 NRG1
2588 LRP8
2589 ST3GAL2

2590 KCNJ6
2591 KCNJ15
2592 ACAN
2593 TNFRSF14
2594 HIST1H4H
2595 SLAMF8
2596 EDA
2597 FGF17
2598 MPZ
2599 WNT9B
2600 LAD1
2601 STC1
2602 GIP
2603 SCUBE1
2604 PSKH1
2605 CPAMD8
2606 NR2F6
2607 KALRN
2608 CILP2
2609 TFF3
2610 TFF2
2611 TFF1
2612 ADAMTS13
2613 LCN1
2614 MPZL3
2615 AMICA1
2616 PCSK7
2617 FCRL3
2618 LY6K
2619 COL26A1
2620 DMKN
2621 DCD
2622 CD300LG
2623 CXCL16
2624 TNFSF13
2625 ITIH3
2626 DRAXIN
2627 WNT4
2628 SCNN1D
2629 NTNG1
2630 KCNT2
2631 EXTL2
2632 CADM3
2633 IGSF8
2634 SLAMF6
2635 OLFML2B
2636 ACP6
2637 IL20

2638 IL24
2639 FRZB
2640 INHBB
2641 BMP10
2642 NPPC
2643 ANTXR2
2644 COL6A3
2645 POGLUT1
2646 SSR2
2647 IHH
2648 FBLN2
2649 FCRL1
2650 CD200R1
2651 ADAMTS9
2652 DNASE1L3
2653 PCOLCE2
2654 CXCL3
2655 CXCL5
2656 PPBP
2657 PF4
2658 RPN1
2659 LRPAP1
2660 PLXNB1
2661 HHIP
2662 EDIL3
2663 F2RL2
2664 SCGB3A2
2665 SPINK1
2666 ERAP1
2667 EGFLAM
2668 IL3
2669 IL22RA2
2670 PI16
2671 COL1A2
2672 TNFRSF11B
2673 DEFA4
2674 DEFA6
2675 FREM1
2676 MBL2
2677 QSOX2
2678 SERPINA12
2679 NELL1
2680 CACNB2
2681 HTRA1
2682 LIPC
2683 IL25
2684 ADAMTS15
2685 SPINT1

2686 FBN1
2687 C2
2688 TUB
2689 A2ML1
2690 GALR1
2691 SCG5
2692 GREM1
2693 CACNB3
2694 KIR3DL1
2695 SPINT2
2696 SERPINF2
2697 KLK4
2698 KLK2
2699 KLK5
2700 KLK6
2701 ANGPTL4
2702 LTBP3
2703 TNXB
2704 SFTPC
2705 IL7R
2706 NPNT
2707 IL12A
2708 FSTL5
2709 CTRB2
2710 NTSR2
2711 COL4A3
2712 THBS3
2713 EFNA1
2714 UMOD
2715 GP2
2716 ELSPBP1
2717 RNASE6
2718 MUC15
2719 GKN1
2720 NRG4
2721 BTD
2722 MUC3A
2723 ITGAM
2724 SERPINA9
2725 SERPINA6
2726 TMED10
2727 CST5
2728 CST2
2729 KISS1
2730 LILRA3
2731 PKHD1
2732 PDGFD
2733 OBP2B

2734 NRTN
2735 RLN3
2736 MUC7
2737 SOSTDC1
2738 KSR2
2739 COL24A1
2740 DSCAM
2741 COL8A2
2742 PRNP
2743 SCG2
2744 CD8B
2745 PRL
2746 NEGR1
2747 IL16
2748 IL17D
2749 CTSW
2750 EFEMP2
2751 CCL19
2752 HPSE2
2753 ADAMTS20
2754 CST9
2755 SAA1
2756 MST1
2757 CD34
2758 CHRNA9
2759 PODN
2760 KLK15
2761 SEZ6L2
2762 INHBC
2763 APOF
2764 SCUBE2
2765 PCSK1
2766 PRIMA1
2767 CALCB
2768 CREG2
2769 CLEC14A
2770 BDNF
2771 C8G
2772 CD151
2773 KCNJ10
2774 CHID1
2775 ADAMTSL1
2776 BOLA1
2777 PRSS36
2778 CA8
2779 TAS1R2
2780 CALR
2781 GPC5

2782 FCER1A
2783 SYCN
2784 ITLN1
2785 NRXN1
2786 RRH
2787 GREM2
2788 FKRP
2789 FCRL6
2790 ADIPOQ
2791 PENK
2792 AATK
2793 SGSH
2794 EDDM3A
2795 TNFSF15
2796 IFNL1
2797 RNASE10
2798 EPGN
2799 NTM
2800 PAPPA
2801 VMO1
2802 COL18A1
2803 GPC6
2804 OVCH2
2805 GKN2
2806 BMP8A
2807 NOG
2808 IFNL2
2809 OPCML
2810 FAM3B
2811 NPW
2812 CRELD2
2813 SLIT3
2814 EFNA5
2815 COLEC10
2816 KCND2
2817 BPIFC
2818 GAST
2819 FAM19A3
2820 LRRC26
2821 RNLS
2822 TMED9
2823 TNFAIP2
2824 GP1BA
2825 TCN2
2826 NRG3
2827 IFITM1
2828 LAMP1
2829 CD300LF

2830 FCAR
2831 CLCNKA
2832 PRG2
2833 HYAL3
2834 FGF3
2835 CCK
2836 LILRA5
2837 SLIT1
2838 LYPD6
2839 BCAM
2840 COL4A1
2841 HAPLN4
2842 THSD4
2843 SEMA4D
2844 WNT7B
2845 SCGB1C1
2846 EYS
2847 AGRN
2848 BSPH1
2849 ZP3
2850 SERPINA5
2851 KRTDAP
2852 COL25A1
2853 RNASE9
2854 RHCE
2855 QRFP
2856 OSTN
2857 FZD9
2858 OPTC
2859 PRELP
2860 UTS2B
2861 HIST1H4D
2862 SBSN
2863 RELN
2864 VSTM1
2865 HMGB1
2866 NCR1
2867 IL1RAP
2868 SPOCK3
2869 SERPINA3
2870 HIST1H4A
2871 SFTA2
2872 ATP2A1
2873 COL27A1
2874 FLNA
2875 FAM3C
2876 HIST1H3E
2877 HIST1H4C

2878 IGF2R
2879 IFNL3
2880 SERPINA1
2881 IL27
2882 HIST1H3D
2883 COL13A1
2884 ENTPD6
2885 ENPP1
2886 CR1L
2887 SCGB1D4
2888 PSAP
2889 SIRPA
2890 GRK6
2891 HIST1H4F
2892 HIST1H4I
2893 HIST1H3A
2894 HIST1H4E
2895 LPA
2896 SMOC1
2897 CD247
2898 CAPZA2
2899 CR1
2900 FCGR3A
2901 COL11A2
2902 NOTCH4
2903 AGER
2904 NEU1
2905 CSHL1
2906 C6orf25
2907 APOM
2908 NCR3
2909 HLA-C
2910 C6orf15
2911 HLA-G
2912 IL31
2913 PCDHA2
2914 CFI
2915 TMSB4X
2916 KLRC2
2917 C1QTNF9B
2918 DEFA1
2919 IGLV4-60
2920 IGLV11-55
2921 IGHG1
2922 IGHG3
2923 DARC
2924 CSH2
2925 LCAT

2926 KLRK1
2927 CEACAM16
2928 DNASE1
2929 CCL27
2930 ITGA1
2931 COL28A1
2932 CELA2B
2933 TNFRSF25
2934 RPTN
2935 IFI30
2936 FAM19A5
2937 KIR2DS4
2938 HLA-DPB1
2939 C1QTNF5
2940 APOC4-APOC2
2941 ORM2
2942 ORM1
2943 HLA-DPA1
2944 HLA-B
2945 APOC2
2946 HLA-DQA2
2947 DEFA3
2948 LILRA4
2949 DEFA1B
2950 KIR3DL2
2951 TNFRSF13B
2952 AQP1
2953 HLA-DOB
2954 EGFL8
2955 CNTF
2956 TNFRSF6B
2957 CFB
2958 CFHR1
2959 LILRA6
2960 FCGR2C
2961 C4A
2962 INSL3
2963 OC90
2964 INS
2965 HP
2966 GH1
2967 MIA
2968 SEMA3F
2969 CD99
2970 HS3ST1
2971 KLHL13
2972 CYP26B1
2973 PRSS22

2974 ITGA3
2975 LAMP2
2976 CCL18
2977 CCL3
2978 CACNG3
2979 TAC1
2980 CX3CL1
2981 ALDH3B1
2982 CCL26
2983 CEACAM7
2984 CELSR3
2985 PGLYRP1
2986 ST3GAL1
2987 REV3L
2988 KAL1
2989 DCN
2990 HEBP1
2991 IL20RA
2992 ATP2C1
2993 SERPINB1
2994 C8B
2995 KCNG1
2996 BTN3A1
2997 TNFRSF1B
2998 MYOC
2999 VCL
3000 TUBG2
3001 TLL1
3002 TRIO
3003 MSR1
3004 PSMA4
3005 GUCA2B
3006 RRM2B
3007 COL9A2
3008 H6PD
3009 ELN
3010 LY75
3011 OPN3
3012 PTPRN
3013 CBLN4
3014 CHRDL2
3015 RELT
3016 IL17RB
3017 SERPINB3
3018 STYK1
3019 SLC6A16
3020 CA11
3021 GPC1

3022 ATP2C2
3023 CHI3L2
3024 YBX1
3025 CD84
3026 CACNB1
3027 TRPC7
3028 MAST4
3029 GAL
3030 ATP1B3
3031 SLC44A1
3032 FGF22
3033 FSTL3
3034 CNGB1
3035 TRPM5
3036 VASH1
3037 LMCD1
3038 SLC6A15
3039 SPP2
3040 SPEG
3041 HMMR
3042 CYP2W1
3043 CLCN4
3044 NOTCH3
3045 SLC24A1
3046 LMAN1
3047 C19orf10
3048 TUBE1
3049 CELSR1
3050 WNT8B
3051 CACNG5
3052 CACNG4
3053 TUBA3D
3054 KLHL20
3055 GPC4
3056 ACTN2
3057 FKBP6
3058 CST7
3059 TULP3
3060 EDN1
3061 FGF20
3062 P2RY10
3063 PKD2L2
3064 BPIFB2
3065 CLUL1
3066 FKBP7
3067 CEACAM1
3068 DCT
3069 COL5A3

3070 MOK
3071 IMPG2
3072 IL12RB2
3073 COL19A1
3074 TRPM3
3075 P2RX5
3076 EPYC
3077 FAT1
3078 COL16A1
3079 CD59
3080 FCN1
3081 OVGP1
3082 MAST2
3083 TMPRSS11E
3084 SLC6A14
3085 ATRN
3086 SIGLEC1
3087 HEPH
3088 KCNH4
3089 LAG3
3090 LTBP4
3091 LYZ
3092 DNAJB11
3093 GNPTG
3094 CD209
3095 MCOLN1
3096 EFNB1
3097 FCGBP
3098 CECR1
3099 GABRP
3100 BAMBI
3101 TPSD1
3102 KCNK16
3103 CRISP3
3104 BRPF3
3105 PGC
3106 ITPR3
3107 TMED1
3108 KCNK6
3109 HSD3B7
3110 AMELY
3111 HCN2
3112 KLHL22
3113 SERPIND1
3114 SNAP29
3115 MMP11
3116 P2RX6
3117 OSM

3118 GGT5
3119 PPIL2
3120 ADRBK2
3121 PLA2G3
3122 LGALS1
3123 GGTLC2
3124 TIMP3
3125 UPK3A
3126 CHADL
3127 FKBP3
3128 COCH
3129 PSMA3
3130 LGMN
3131 CHGA
3132 PSMA6
3133 BMP7
3134 PSMA7
3135 AVP
3136 TRIB3
3137 RSPO4
3138 PROKR2
3139 OXT
3140 ASIP
3141 CST4
3142 GUCY2F
3143 KCND1
3144 OPN1LW
3145 RS1
3146 PCSK1N
3147 TIMP1
3148 KLHL4
3149 GABRE
3150 ITIH6
3151 SRPX2
3152 OLFM4
3153 CBLN1
3154 CCL22
3155 CCL17
3156 PRSS54
3157 ZP2
3158 PRSS33
3159 XYLT1
3160 PDGFRL
3161 FZD3
3162 DKK4
3163 TULP2
3164 NUCB1
3165 CGB2

3166 LHB
3167 CGB
3168 KCNA7
3169 CLEC4M
3170 IL4I1
3171 LILRB1
3172 LILRA1
3173 GPI
3174 EBI3
3175 APLP1
3176 SIGLEC8
3177 CD79A
3178 CLEC11A
3179 CACNG7
3180 COMP
3181 FKBP8
3182 ITGB8
3183 PTN
3184 NPVF
3185 STX1A
3186 CRHR2
3187 CCL24
3188 NPTX2
3189 PTPRZ1
3190 SPAM1
3191 AGR2
3192 PSMA2
3193 CNTNAP3
3194 OGN
3195 ASPN
3196 ECM2
3197 AMBP
3198 TNFSF8
3199 RLN2
3200 RLN1
3201 KCNT1
3202 RBP3
3203 GDF10
3204 KAZALD1
3205 FGF8
3206 SMC3
3207 LGI1
3208 PSMB3
3209 TUBD1
3210 ICAM2
3211 LGALS3BP
3212 CCL8
3213 CCL1

3214 NAGLU
3215 CACNG1
3216 FAM20A
3217 ODAM
3218 SMR3A
3219 PF4V1
3220 KLHL2
3221 CLCN3
3222 CPZ
3223 GLRB
3224 KLHL5
3225 TECTA
3226 CRTAM
3227 HPX
3228 FOLR3
3229 PVRL1
3230 CD81
3231 SLC6A12
3232 PRR4
3233 FGF6
3234 KCNA1
3235 ART4
3236 MGP
3237 ENDOU
3238 ACRBP
3239 KLRB1
3240 COL12A1
3241 RHAG
3242 BMP5
3243 GPR63
3244 COL9A1
3245 NR2E1
3246 PTK7
3247 IMPG1
3248 WISP3
3249 ENPP5
3250 MEP1A
3251 ERBB2IP
3252 C7
3253 LOX
3254 GZMK
3255 SPARC
3256 IK
3257 THBS4
3258 BTNL8
3259 SLC12A7
3260 PPWD1
3261 SERPINI2

3262 COL7A1
3263 CSPG5
3264 KLHL18
3265 KLHL24
3266 SLC4A3
3267 ADAM23
3268 NRBP1
3269 LOXL3
3270 REG1A
3271 KCNJ13
3272 GNLY
3273 IL1RL2
3274 PCYOX1
3275 CD207
3276 ANGPTL1
3277 QSOX1
3278 KCNC4
3279 IVNS1ABP
3280 PRG4
3281 OLFML3
3282 LGALS8
3283 BMP8B
3284 CTBS
3285 KLHL12
3286 LEPRE1
3287 ARTN
3288 FASLG
3289 NENF
3290 TXNDC12
3291 MUC5B
3292 VAMP8
3293 GHRH
3294 OLFM3
3295 FGF23
3296 C2orf40
3297 FKBP15
3298 NR4A3
3299 KDSR
3300 GPR68
3301 GPR75
3302 KLHL29
3303 TECTB
3304 PRLHR
3305 SFRP5
3306 PCDHB10
3307 GPR31
3308 MASTL
3309 LYZL1

3310 MRC1
3311 HSPH1
3312 KBTBD7
3313 TGFBI
3314 NR2C1
3315 EPX
3316 TEX14
3317 A1BG
3318 CCRL2
3319 CPXM2
3320 PAEP
3321 OBP2A
3322 FMOD
3323 COPA
3324 OPN4
3325 KLHL7
3326 NPY
3327 FKBP9
3328 RAMP3
3329 CNTFR
3330 SFTPA1
3331 SRGN
3332 P4HA1
3333 ITPR2
3334 PRDX4
3335 CD97
3336 MMP19
3337 IL13RA2
3338 COL10A1
3339 PLP1
3340 NRK
3341 ACVR1C
3342 GPR83
3343 INHA
3344 PI3
3345 SLPI
3346 KCNS1
3347 SEMG2
3348 EDN3
3349 SEMG1
3350 KCNK15
3351 VAMP7
3352 IL9R
3353 IL17C
3354 CEACAM8
3355 NDP
3356 CRISP2
3357 SERPINB6

3358 COL21A1
3359 KCNK17
3360 CRISP1
3361 OPN5
3362 CXCL6
3363 SCGB1D2
3364 SCGB2A1
3365 WNT1
3366 GPR18
3367 AMELX
3368 NPBWR2
3369 PSPN
3370 ATG4C
3371 GNRH2
3372 CST8
3373 GFRA4
3374 OTOR
3375 BPIFB1
3376 GRPR
3377 AMOT
3378 SLURP1
3379 GPR42
3380 KIRREL2
3381 FLRT1
3382 CSN1S1
3383 STATH
3384 HTN1
3385 WNK4
3386 CFP
3387 OMG
3388 OMD
3389 EDN2
3390 MASP1
3391 TRPV5
3392 EMR2
3393 F2RL3
3394 TUBA4A
3395 FGL2
3396 SPINK2
3397 CPA4
3398 TAS2R16
3399 LRRC4
3400 CALU
3401 LRRC17
3402 OPN1SW
3403 GDF2
3404 TTBK2
3405 ACKR4

3406 PSMA1
3407 KCNC1
3408 ATP1B2
3409 MMP28
3410 CCL4
3411 KLK14
3412 KLK10
3413 KLK8
3414 RIPK3
3415 ART1
3416 CHRNA10
3417 PRKCSH
3418 DPP6
3419 GDF1
3420 BST2
3421 MAS1
3422 BMP15
3423 ACTN4
3424 CACNG6
3425 HRC
3426 TRPM4
3427 SMPDL3B
3428 PNCK
3429 HABP4
3430 PPIL4
3431 BPIFA2
3432 GGT7
3433 DEFB118
3434 C1QL1
3435 TEX101
3436 CCL25
3437 PPT1
3438 EMR3
3439 KCNC3
3440 LRRC4B
3441 TUBG1
3442 RAMP2
3443 IL13RA1
3444 FSHB
3445 NR0B2
3446 PODNL1
3447 NUP210
3448 FCRLA
3449 CNGA4
3450 ENAM
3451 IGJ
3452 ITGB4
3453 H3F3B

3454 CLEC10A
3455 GUCY2D
3456 SDF2
3457 BCAN
3458 HAPLN2
3459 APCS
3460 NMUR2
3461 GPR12
3462 LGR6
3463 RXFP2
3464 POSTN
3465 KL
3466 PDE6B
3467 RTN3
3468 GGT2
3469 NTS
3470 SPINK5
3471 TMEM66
3472 LOXL2
3473 ADAMDEC1
3474 IRAK2
3475 REG4
3476 TSHB
3477 WNT2B
3478 CD101
3479 FKBP11
3480 FST
3481 ERN2
3482 GRP
3483 KLRD1
3484 C12orf39
3485 PRH2
3486 C5AR2
3487 CSN2
3488 HILPDA
3489 CGA
3490 AMHR2
3491 GDF11
3492 KCNH3
3493 TAAR5
3494 PRADC1
3495 GNS
3496 KLHL36
3497 AGT
3498 SERPINE2
3499 GPR45
3500 ALDH1L2
3501 SCYL2

3502 PLXNC1
3503 SLC41A2
3504 LECT1
3505 THSD1
3506 ITM2B
3507 CFC1
3508 UGGT1
3509 GYPC
3510 OR1J1
3511 ANGPTL2
3512 PSMB7
3513 DERL1
3514 TLN1
3515 IFNA21
3516 CD72
3517 BPHL
3518 CLPS
3519 FGFBP1
3520 TLR2
3521 IL18BP
3522 PI15
3523 TRPC6
3524 FXYD2
3525 CGREF1
3526 EMILIN1
3527 ATRAID
3528 ENTPD1
3529 GPR87
3530 PLA2G12B
3531 CILP
3532 TRPC3
3533 PDE6H
3534 C1RL
3535 LRIG3
3536 LGR5
3537 SLC39A5
3538 GPR84
3539 LPAR6
3540 TSSK4
3541 KCNH5
3542 GPR65
3543 SLC12A6
3544 MFAP1
3545 EFTUD1
3546 DPEP3
3547 OR4D1
3548 SPACA3
3549 ARSG

3550 SLC39A6
3551 GALNT1
3552 MEP1B
3553 SECTM1
3554 MAPK4
3555 FKBP10
3556 SAMD1
3557 COL6A2
3558 TRPM2
3559 LMTK3
3560 CACNG8
3561 FBN3
3562 PSMB6
3563 GPR32
3564 SIGLEC10
3565 PTH2
3566 FCN3
3567 HSPG2
3568 CYR61
3569 PIGK
3570 TINAGL1
3571 KCNA10
3572 PSMA5
3573 CELSR2
3574 ITGA10
3575 GPR161
3576 ATP1B1
3577 CREG1
3578 XCL1
3579 XCL2
3580 HMCN1
3581 TUFT1
3582 ECM1
3583 ADAMTSL4
3584 KCNH1
3585 S100A8
3586 HCN3
3587 LEFTY2
3588 WNT9A
3589 REG3G
3590 THNSL2
3591 C1QL2
3592 FBLN7
3593 CNGA3
3594 LYG1
3595 HSPD1
3596 ABCA12
3597 ACKR2

3598 IGSF11
3599 ALDH1L1
3600 UCN2
3601 MANF
3602 MUC4
3603 AHSG
3604 KLHL8
3605 CYP4V2
3606 ADAMTS16
3607 GZMA
3608 CRHBP
3609 CXCL14
3610 KLHL3
3611 SCUBE3
3612 TTBK1
3613 GPR6
3614 TAAR2
3615 TAAR6
3616 TAAR8
3617 VIP
3618 SLC12A9
3619 NLGN4X
3620 GPR174
3621 LPAR4
3622 IL2RG
3623 RBMX
3624 OPN1MW
3625 NSDHL
3626 GABRQ
3627 GNRH1
3628 PXDNL
3629 CRH
3630 PSKH2
3631 RSPO2
3632 CER1
3633 PLIN2
3634 IFNA16
3635 NR6A1
3636 LCN2
3637 RGR
3638 HABP2
3639 LRRC4C
3640 PAMR1
3641 KLHL35
3642 HYOU1
3643 GGTLC1
3644 ZP1
3645 SCN2B

3646 TMEM25
3647 SOGA1
3648 SPINT4
3649 GPHA2
3650 ALDOA
3651 KLHL1
3652 VEGFC
3653 CNDP1
3654 CACNA2D4
3655 KCNA6
3656 PLBD2
3657 CCL28
3658 DST
3659 TUBA3E
3660 CYSLTR2
3661 GRID2
3662 PTH
3663 KCNK13
3664 UHMK1
3665 SPOCK1
3666 TMEM123
3667 SPARCL1
3668 DSPP
3669 DMP1
3670 CATSPER3
3671 C16orf89
3672 MCOLN2
3673 SEMA3D
3674 THY1
3675 GPR15
3676 ABI3BP
3677 LRRK1
3678 WNT3A
3679 OBSCN
3680 GPR26
3681 MMP21
3682 LY96
3683 PSMA8
3684 CXADR
3685 CA10
3686 CYP2U1
3687 GPR78
3688 VSIG4
3689 OTOA
3690 GPR61
3691 CXCL13
3692 TSPAN7
3693 CDK20

3694 GDF6
3695 KCNS2
3696 CD109
3697 NODAL
3698 NPTN
3699 BUB1B
3700 PDE6D
3701 KLHL40
3702 C8A
3703 TIMP4
3704 IL34
3705 CACNA2D3
3706 SVOPL
3707 GPR153
3708 KCNB1
3709 NRG2
3710 CD1D
3711 CD1A
3712 CD1C
3713 CD1B
3714 CD1E
3715 CPA2
3716 CPA5
3717 NBL1
3718 F11R
3719 PINK1
3720 APOA2
3721 C1QC
3722 PSMB4
3723 CES5A
3724 PGLYRP3
3725 SPON2
3726 AGRP
3727 PIP
3728 LYPD5
3729 NPR2
3730 FNDC5
3731 FCN2
3732 TOR2A
3733 PKN3
3734 NEK8
3735 CD3G
3736 AZGP1
3737 PGLYRP2
3738 SCGB3A1
3739 LYZL6
3740 CCL16
3741 CCL15-CCL14

3742 KLHL10
3743 HCRT
3744 ALDH16A1
3745 SIGLEC11
3746 EMC10
3747 OR7G1
3748 NTN3
3749 TPCN2
3750 FGF19
3751 CYP4A22
3752 KLHL21
3753 MATN1
3754 TSSK3
3755 TM2D1
3756 FCGR3B
3757 BPNT1
3758 PIGR
3759 FCAMR
3760 KCNF1
3761 KCNJ3
3762 H3F3A
3763 BOLA3
3764 PGLYRP4
3765 S100A9
3766 S100A12
3767 ALPP
3768 KBTBD8
3769 FAM19A4
3770 APOA1BP
3771 PROK2
3772 FSTL1
3773 IGFBP7
3774 NEK10
3775 RETNLB
3776 FCRL4
3777 SERPINI1
3778 PTX3
3779 IL17RE
3780 IL17RC
3781 PRRT3
3782 CXCL1
3783 PLSCR2
3784 RYK
3785 UCN
3786 CDCP1
3787 CLEC3B
3788 SLC6A20
3789 LIPH

3790 RHO
3791 MFI2
3792 OTOP1
3793 AIMP1
3794 CAMP
3795 CAMKV
3796 ETNPPL
3797 PRSS12
3798 HMGB2
3799 SCRG1
3800 AGGF1
3801 ESM1
3802 ENPP6
3803 CARTPT
3804 TLR3
3805 GFM2
3806 SLC6A18
3807 GDF9
3808 LEAP2
3809 IL31RA
3810 GPR85
3811 BMPER
3812 KCNK5
3813 LMTK2
3814 HNF4G
3815 KCNV1
3816 DEFA5
3817 DEFB1
3818 GPR146
3819 SPAG11B
3820 CTHRC1
3821 PRKACG
3822 MAMDC2
3823 CPA6
3824 KDM1B
3825 TRPV6
3826 FIGF
3827 WNK2
3828 NLGN4Y
3829 FAT3
3830 GPR101
3831 FOLR2
3832 KBTBD6
3833 OXGR1
3834 UCMA
3835 ENOX2
3836 RNASE7
3837 VWA2

3838 PRAP1
3839 KCNC2
3840 GPR176
3841 JAM3
3842 SVOP
3843 OPN1MW2
3844 SCN3B
3845 SMPD1
3846 ILK
3847 TPP1
3848 OR10A5
3849 SERPINB7
3850 SERPINB8
3851 MFAP4
3852 CLEC3A
3853 SERPINB12
3854 CHRFAM7A
3855 B2M
3856 CATSPER2
3857 GPR182
3858 CACNG2
3859 TAC3
3860 PDIA3
3861 BPIFB6
3862 ISLR2
3863 CCL23
3864 CDK12
3865 CD3D
3866 OR51E2
3867 LPO
3868 KLHL26
3869 LALBA
3870 TUBA1A
3871 TUBA1C
3872 LAIR1
3873 LAIR2
3874 LY6D
3875 TMIGD2
3876 SEMA6B
3877 CYB5D2
3878 GGT6
3879 KLK11
3880 KLK13
3881 CD320
3882 CD300C
3883 CD300A
3884 ABCA3
3885 SLC3A2

3886 ULK4
3887 PNOC
3888 KCNJ4
3889 VASN
3890 KCNV2
3891 MLKL
3892 KCNG4
3893 KLHL30
3894 SCGB1D1
3895 COL3A1
3896 GDNF
3897 GFM1
3898 OR13J1
3899 SFTPB
3900 ENHO
3901 LGALS9
3902 JMJD7-PLA2G4B
3903 SIGLEC7
3904 KLK7
3905 ROR2
3906 PARM1
3907 RSPO1
3908 CXCL11
3909 NR0B1
3910 IL1RAPL1
3911 RNASE2
3912 RNASE3
3913 COL22A1
3914 SDC2
3915 OR4K15
3916 HTRA4
3917 GPR183
3918 GJB1
3919 VPREB1
3920 BUB1
3921 GP9
3922 NLGN1
3923 MUC17
3924 WNT10B
3925 TM4SF1
3926 GUSB
3927 TAS1R3
3928 NLGN2
3929 ALCAM
3930 GPR37L1
3931 GPR25
3932 GYPA
3933 CRTAP

3934 CNGB3
3935 CST1
3936 SEMA3E
3937 SERPINB9
3938 KCNS3
3939 GPR37
3940 HTRA3
3941 GPR27
3942 KBTBD2
3943 CYTL1
3944 TRH
3945 OR7G2
3946 OR1M1
3947 OR8B12
3948 PLAC1
3949 KCNG3
3950 PROL1
3951 SMR3B
3952 NETO2
3953 CSN3
3954 LRG1
3955 CLCN5
3956 KCND3
3957 APLN
3958 SPACA5B
3959 SPACA5
3960 OR1L8
3961 NMUR1
3962 CLSTN1
3963 ENC1
3964 GPR82
3965 GPR34
3966 DEFB4A
3967 ANGPTL7
3968 IFNB1
3969 AQP4
3970 CYP4F22
3971 REG3A
3972 REG1B
3973 OR1A1
3974 GPR22
3975 CXCR6
3976 AZU1
3977 PRSS27
3978 INSL5
3979 MUCL1
3980 KLHL6
3981 SMPDL3A

3982 MOS
3983 OR4D9
3984 COL6A5
3985 CES4A
3986 CES3
3987 MRGPRF
3988 MRGPRD
3989 RXFP4
3990 LRRN3
3991 GPR151
3992 MMRN2
3993 GPR148
3994 KCNK7
3995 C1QB
3996 C1QA
3997 NDNF
3998 OLR1
3999 GLIPR1L1
4000 DAG1
4001 RNASE8
4002 AGR3
4003 PEAK1
4004 TNFRSF10D
4005 TNFRSF10C
4006 CSPG4
4007 XCR1
4008 GPRC6A
4009 TAS1R1
4010 MUC13
4011 WFIKKN2
4012 CD7
4013 CNP
4014 GPR160
4015 KLHL15
4016 TLR10
4017 TLR1
4018 TLR6
4019 SLC6A19
4020 ATP2A2
4021 LINGO2
4022 IL20RB
4023 LEP
4024 FGFBP3
4025 FZD4
4026 BTC
4027 P2RY14
4028 GPR171
4029 GPR149

4030 CD164L2
4031 ZG16
4032 PDIK1L
4033 CATSPER1
4034 CST6
4035 TMEM9B
4036 OR10P1
4037 DPP10
4038 CLCF1
4039 GPR152
4040 NR2F1
4041 SFN
4042 TSPEAR
4043 A2M
4044 LRRN1
4045 KLHL38
4046 OR4K17
4047 OR4K5
4048 TAC4
4049 KBTBD11
4050 OR4F17
4051 DEFB104A
4052 DEFB103A
4053 FIBIN
4054 DEFB104B
4055 DEAF1
4056 IFNW1
4057 DEFB103B
4058 KCNA2
4059 GPR4
4060 OR2B11
4061 CD163
4062 CD163L1
4063 DEFB114
4064 OR4F4
4065 FAM20C
4066 PVRL3
4067 GPR150
4068 TSSK6
4069 SPINK6
4070 VN1R1
4071 SPAG11A
4072 KCNG2
4073 TUBAL3
4074 UCN3
4075 AMBN
4076 OTOS
4077 THBD

4078 GP5
4079 CPN2
4080 CD300LB
4081 LGALS7B
4082 ALOXE3
4083 DAND5
4084 VWA1
4085 FJX1
4086 KLHL28
4087 ALOX12B
4088 SLITRK4
4089 ALOX15B
4090 CDH5
4091 MRGPRX4
4092 MRGPRX3
4093 ABCA13
4094 GPR139
4095 OR10K2
4096 SHISA2
4097 OR51E1
4098 DEFB112
4099 GPR62
4100 HEPHL1
4101 CCL13
4102 FDCSP
4103 GPR135
4104 P2RY13
4105 GPR88
4106 OR8H3
4107 GPR3
4108 OR5AS1
4109 OR4C16
4110 OR4A16
4111 ENPP7
4112 P2RY8
4113 MRGPRG
4114 HIST2H4B
4115 ATP6AP2
4116 KCNA4
4117 KCNJ14
4118 CACNB4
4119 OTOL1
4120 KCNK4
4121 BGN
4122 HIST1H3I
4123 NXPH3
4124 OR2V2
4125 RXFP3

4126 GALR2
4127 RESP18
4128 IGIP
4129 ANXA2
4130 GRID1
4131 OR2T29
4132 GPIHBP1
4133 GPR97
4134 GGTLC3
4135 GAS6
4136 FREM3
4137 GPR19
4138 GABRR3
4139 PLGLB1
4140 OR2T34
4141 PMCH
4142 GPR132
4143 UTP11L
4144 HIST2H3D
4145 DGCR6
4146 KLHL25
4147 FAM19A1
4148 GPR1
4149 MRGPRX2
4150 TUBA8
4151 EMILIN3
4152 OLFML1
4153 GPR39
4154 CNGA2
4155 ARSI
4156 LRRC55
4157 SDR42E2
4158 HIST2H4A
4159 KCNH8
4160 NPB
4161 TMPRSS2
4162 OR2T10
4163 CNTN2
4164 LRTOMT
4165 OR1D2
4166 GPR173
4167 KCNK12
4168 DEFB108B
4169 GDF3
4170 MRGPRE
4171 CCR10
4172 C1QTNF8
4173 PROS1

4174 NELL2
4175 APOO
4176 SDR42E1
4177 CLCNKB
4178 MUC6
4179 SEMA4B
4180 FLRT2
4181 C6orf120
4182 PPIL6
4183 CDNF
4184 NOTUM
4185 KLHL33
4186 SFTPA2
4187 CDK10
4188 ROR1
4189 PDE6G
4190 NR2F2
4191 OLFML2A
4192 NTF3
4193 LYG2
4194 IFIT1
4195 EVI2B
4196 FFAR3
4197 RTN4RL1
4198 OR4F5
4199 BPIFB3
4200 BPIFB4
4201 KLHL32
4202 BTLA
4203 THBS2
4204 CYP4X1
4205 CD300E
4206 BTN3A2
4207 KLK12
4208 DEFB105A
4209 DEFB107A
4210 DEFB106A
4211 DEFB105B
4212 TPCN1
4213 LILRB4
4214 QRFPR
4215 SERPINA11
4216 P2RY4
4217 ZACN
4218 EMID1
4219 GPR141
4220 DEFB106B
4221 FPR3

4222 SBK2
4223 TLR5
4224 ISG15
4225 AMTN
4226 KBTBD12
4227 GABRD
4228 TMEM256-PLSCR3
4229 DMBT1
4230 OVCH1
4231 COL14A1
4232 KLHL17
4233 C17orf99
4234 COL4A5
4235 OTOG
4236 IGFL1
4237 PLSCR1
4238 GPR21
4239 LCTL
4240 IGFL3
4241 VWC2
4242 CATSPER4
4243 NYX
4244 LIPI
4245 CGB5
4246 APOD
4247 IL1RAPL2
4248 S100A13
4249 CXCL17
4250 VN1R2
4251 FAT4
4252 STK40
4253 CGB7
4254 NLGN3
4255 PRTN3
4256 PIK3R4
4257 OR2T3
4258 SLC22A25
4259 VKORC1L1
4260 CLPSL2
4261 CD47
4262 POTEI
4263 PATE2
4264 HCAR1
4265 SLC6A17
4266 ADAM32
4267 HIST1H3J
4268 OR1J2
4269 HIST1H4J

4270 CCL4L2
4271 GUCA2A
4272 DIO3
4273 IPP
4274 PDGFA
4275 GYPE
4276 SPN
4277 GALP
4278 GZMM
4279 COL4A6
4280 MFAP5
4281 SERPINB2
4282 SERPINB13
4283 SLC22A24
4284 OR51C1P
4285 NMB
4286 OR52M1
4287 OCLN
4288 HIST4H4
4289 PRB3
4290 HIST1H4K
4291 ERO1L
4292 AADACL2
4293 KEL
4294 TUBA3C
4295 DEFB107B
4296 CLEC4C
4297 BPIFA1
4298 RPS6KL1
4299 CACNA1E
4300 SLC29A3
4301 CNGA1
4302 HIST1H4L
4303 KLHL9
4304 LRRTM3
4305 EGFL6
4306 MUC2
4307 C9orf96
4308 L1CAM
4309 NAGA
4310 RORB
4311 GP1BB
4312 OR2T5
4313 RAET1G
4314 GPR52
4315 HIST2H3C
4316 HIST1H3H
4317 PNLIPRP3

4318 HIST2H3A
4319 DEFB110
4320 LCN8
4321 LRIT2
4322 NPY4R
4323 SYT15
4324 COL5A2
4325 COL15A1
4326 CD300LD
4327 NMS
4328 OR2H2
4329 MAS1L
4330 OR2H1
4331 OR11A1
4332 OR14J1
4333 GPR20
4334 SPINK9
4335 C11orf83
4336 CD177
4337 CCL4L1
4338 CCL3L1
4339 LGALS7
4340 CDKL4
4341 LGR4
4342 PSMB10
4343 VIT
4344 GPR56
4345 HTN3
4346 SERPINB11
4347 SERPINB4
4348 SERPINB5
4349 RNASE13
4350 COL6A6
4351 COLQ
4352 IGKC
4353 IGKV4-1
4354 IGKV5-2
4355 IGKV2-40
4356 IGKV1D-42
4357 IGLV4-69
4358 IGLV8-61
4359 IGLV10-54
4360 IGLV1-51
4361 IGLV7-46
4362 IGLV5-45
4363 IGLV1-44
4364 IGLV7-43
4365 IGLV1-40

4366 IGLV5-37
4367 IGLV1-36
4368 IGLV3-27
4369 IGLV3-25
4370 IGLV2-23
4371 IGLV3-22
4372 IGLV2-18
4373 IGLV3-16
4374 IGLV3-12
4375 IGLV2-11
4376 IGLV4-3
4377 IGLC2
4378 IGLC3
4379 IGLC7
4380 TRAV8-4
4381 IGHA2
4382 IGHG4
4383 IGHG2
4384 IGHD
4385 IGHM
4386 IGHV1-2
4387 IGHV3-23
4388 IGHV1-46
4389 IGHV7-81
4390 KLK9
4391 NUP62
4392 CGB8
4393 KLHL23
4394 NOTCH2NL
4395 GPC2
4396 CCL14
4397 HEXA
4398 GANC
4399 ANG
4400 NPS
4401 DEFB113
4402 GPR33
4403 MSMP
4404 KCNU1
4405 DEFB116
4406 DEFB115
4407 CKLF
4408 OR1C1
4409 IGKV3D-15
4410 C17orf72
4411 C4B
4412 GPX5
4413 IFNA14

4414 PRB4
4415 CYP21A2
4416 PRH1
4417 PSG1
4418 MTRNR2L4
4419 IFNA17
4420 TAAR9
4421 PATE4
4422 IGKV3-20
4423 LILRA2
4424 PSMB9
4425 C1QTNF9
4426 IGKV1D-16
4427 IGKV3-11
4428 CD302
4429 CORT
4430 KIR3DL3
4431 PSG2
4432 BGLAP
4433 IGKV1-39
4434 SERPINB10
4435 IGKV3-7
4436 IGKV2-30
4437 IGKV2D-29
4438 EFNA4
4439 IGKV1-5
4440 IL10RB
4441 LEFTY1
4442 DDOST
4443 IGKV3-15
4444 MTRNR2L5
4445 GYPB
4446 GPR162
4447 SEPP1
4448 IGKV2D-40
4449 IGKV1D-39
4450 SERPINE3
4451 SIGLEC12
4452 IGLL5
4453 OR52B2
4454 MTRNR2L8
4455 MTRNR2L10
4456 MTRNR2L3
4457 ASIC5
4458 CCL3L3
4459 MTRNR2L1
4460 MTRNR2L7
4461 RNASE4

4462	SLC22A31
4463	GAN

Table S4. The unique druggable genes identified by overlaping the two source of the prior druggable genes.

Number	Gene list
1	SDF2
2	KCNK2
3	ASIC1
4	OBP2A
5	RFPL3
6	GFRA4
7	GUCA2B
8	H4C2
9	SLC5A1
10	FZD10
11	RNASE1
12	SNAP23
13	FRK
14	GNPTG
15	ABCG4
16	SSTR3
17	C4BPA
18	GRIN2B
19	BRCA1
20	PRCP
21	S100A12
22	CD3G
23	KYNU
24	CD37
25	STK36
26	KLHL23
27	FBLN1
28	VWA5A
29	GPR151
30	ICAM4
31	BRD2
32	SLC12A7
33	SIK1
34	ITPR1
35	CCL3L3
36	FFAR1
37	ABCA5
38	CX3CL1
39	IGFBP4
40	PDGFD
41	KLHL10
42	NGF
43	NOG
44	AKR1A1
45	MTNR1A
46	ARTN
47	HNRNPUL1
48	OR5K4
49	C5

50 MYH6
51 EHMT1
52 CCL5
53 TRPM3
54 NPC1L1
55 STK4
56 UTS2
57 PEAK1
58 PTH
59 SERPINB7
60 CYP20A1
61 ENTPD6
62 ABCC5
63 KMO
64 PMPCA
65 HTR3E
66 TMED10
67 CLPSL2
68 OR5D14
69 P4HA1
70 OR5AN1
71 EXTL2
72 PADI1
73 CD68
74 CHST8
75 AGA
76 PRKAB1
77 AQP4
78 SNCA
79 ACKR3
80 WDR5
81 SLC27A4
82 DAPK3
83 LSS
84 NLGN1
85 OR10G4
86 OR13H1
87 OR2T34
88 FMO6P
89 CNGA3
90 TUBB4A
91 PSEN1
92 POLE
93 SLC4A11
94 SMPDL3B
95 CD22
96 OR1C1
97 LPCAT3
98 RXFP2
99 SNAP29
100 NDOR1
101 PGLYRP2

102 LCN8
103 CD200R1
104 CACNA2D2
105 PLAU
106 OR2AG2
107 GART
108 PLCE1
109 SLC10A3
110 OR1D2
111 COQ8B
112 QARS1
113 CTSL
114 GPR68
115 MIP
116 PTH1R
117 NTN3
118 OPN1MW2
119 CHST10
120 OXNAD1
121 EGFL7
122 KLHL6
123 PAPPA
124 AKR1D1
125 GFOD2
126 OR2T1
127 GSTO1
128 NMU
129 CAMK4
130 OXTR
131 OR5K3
132 TRPA1
133 OR2W1
134 ADCY8
135 REG3A
136 RLN1
137 PIK3R4
138 PLA2G10
139 CNTF
140 CTRC
141 NOTCH1
142 FCGR1A
143 ABCG8
144 CLK1
145 FGF17
146 PLBD2
147 IGLV4-60
148 ALDH7A1
149 ALK
150 OR8U1
151 SLC18A1
152 CAPZA2
153 NRBP1

154 ATG4C
155 KCNV1
156 SEZ6L2
157 GALR2
158 GUCY2D
159 IGLV3-25
160 AIMP1
161 CDK4
162 TRPM2
163 ACHE
164 SLC6A18
165 SLC2A10
166 KLK12
167 LGR5
168 GRIN3B
169 TOP1
170 ATP1A3
171 PRELP
172 DUSP7
173 RHCE
174 BBS2
175 IL20RA
176 GPR37L1
177 PAN3
178 ABCC6
179 TMCO3
180 OR52P2P
181 TXNDC11
182 CD55
183 CDK1
184 SCN7A
185 CYP4Z1
186 REG3G
187 EPHB6
188 OR51T1
189 C6ORF15
190 SLC16A13
191 INSL6
192 CAPN11
193 OR2T8
194 AIFM1
195 OR10A6
196 ALDH5A1
197 OR9M1P
198 OR8K5
199 APOF
200 IL18
201 ACR
202 CA12
203 ALDH1B1
204 NEK6
205 GFRA3

206 C1QTNF9
207 OR4C6
208 HSD17B14
209 IFNGR2
210 FBP2
211 GRID1
212 SLC25A39
213 ASIC2
214 FSD1
215 OR10H2
216 THBS3
217 TNFRSF1A
218 TTL
219 DYRK1A
220 CHRM4
221 CDKN1A
222 KCNK9
223 FKBP14
224 MAOA
225 TRIM7
226 SLCO3A1
227 SULF2
228 RELN
229 HNF4G
230 FGF7
231 RTN4R
232 NPY6R
233 WNT3
234 TAS1R3
235 IL17B
236 ADAM22
237 PHOSPHO1
238 RLN2
239 TYR
240 DAGLB
241 LBP
242 ENG
243 NQO1
244 HSD17B7P2
245 C16ORF89
246 GPR156
247 CCNA2
248 PDE4A
249 CYB5R3
250 COL1A1
251 NR4A3
252 IGKV3-7
253 HAPLN1
254 TAAR8
255 DUOX2
256 CRTAC1
257 AIP

258 ASIC4
259 SLC4A2
260 SLC7A7
261 CXCL1
262 ITGA6
263 ADAMTS4
264 MARS2
265 SRD5A2
266 NTM
267 EFEMP1
268 DHRS9
269 DDI1
270 ROS1
271 AGR3
272 USP47
273 GPR153
274 ROR2
275 GPR27
276 OR2T3
277 THSD1
278 SOD2
279 MBOAT1
280 COL2A1
281 PIK3C2A
282 HPR
283 BCAM
284 SLC22A18
285 CYP46A1
286 BPNT1
287 TUBB2A
288 TESK1
289 OR13D3P
290 S1PR2
291 PGA5
292 SLC16A12
293 XBP1
294 ATP2C2
295 CD300E
296 KDM1B
297 HDAC4
298 PIK3IP1
299 NR3C2
300 ALOX12B
301 TNFSF8
302 SCN3B
303 OR8H2
304 GABRE
305 DHRS7
306 TXNDC2
307 COMT
308 PRIMPOL
309 PSMB11

310 UQCC3
311 OGN
312 BACE2
313 RBM5
314 MMRN1
315 APOC2
316 KCNMA1
317 ADH1A
318 SMAD3
319 NPY4R
320 BMP2
321 CSPG4
322 KCNJ8
323 AKR1B15
324 SERPINE1
325 ADGRE3
326 IGLV7-46
327 ATP1A2
328 DCD
329 ABL2
330 TRIM49D2
331 TRIM58
332 CDK8
333 OR4N4
334 SLC16A5
335 PRKCH
336 H3C6
337 CDK11A
338 TGFBR3
339 CD2
340 AGBL1
341 ATP2A2
342 FBLN2
343 CACNG6
344 IL1F10
345 PTPN9
346 HSP90B1
347 SLC13A3
348 ACVR1B
349 FSHR
350 CD34
351 S100A8
352 GMNN
353 OR5E1P
354 INPP5J
355 OR6S1
356 MRC2
357 UGT2A1
358 ABAT
359 CSNK1G1
360 RPS6KB2
361 THPO

362 EGFLAM
363 HYAL3
364 PON1
365 SLC2A3
366 GUCY1B1
367 IKBKB
368 PPP1CA
369 DEFB126
370 DPEP3
371 KCNN4
372 IGLV1-40
373 MMP23B
374 SLC25A15
375 ADAM20
376 KCNV2
377 DAO
378 LAD1
379 TWSG1
380 LRPAP1
381 UHMK1
382 SPSB4
383 ADGRF5
384 CYP2R1
385 PRSS48
386 CHRFAM7A
387 IGKC
388 TNNT2
389 OR52N4
390 OR51S1
391 AR
392 CACNA1C
393 OR4F15
394 ITGA9
395 HLA-DRB1
396 ADCY4
397 NR1H4
398 PSMA3
399 IMPDH2
400 OR51J1
401 NPY5R
402 DRD5P1
403 ATP7B
404 TUBBP5
405 VRK2
406 WNT4
407 GSTM3
408 TRIM38
409 CASP6
410 TNFRSF10B
411 ALOX5AP
412 SRPK2
413 PMS2

414 PON2
415 GPR176
416 PDE1B
417 THBS1
418 CHRNA6
419 LILRB2
420 KCNJ15
421 ARSK
422 GDF9
423 GHRHR
424 KCNK3
425 ITGA1
426 GPIHBP1
427 GRM7
428 CD36
429 DYRK4
430 C1QC
431 SLC4A8
432 CDH5
433 FBN2
434 PIP5K1C
435 HDAC2
436 WT1
437 CD74
438 IGLV2-11
439 MICAL3
440 AMY1A
441 OR2M3
442 NR5A1
443 APAF1
444 BBOX1
445 SLC29A2
446 MAP3K14
447 OR2G3
448 NRXN1
449 P2RY4
450 CRLF2
451 OR9Q2
452 PTGFR
453 OR6N2
454 SCGB3A2
455 CFC1
456 KBTBD6
457 CELA3B
458 MYORG
459 LIPF
460 TP53I3
461 CEACAM1
462 SPSB3
463 IL15RA
464 LAIR1
465 APOH

466 MAPK13
467 MUC6
468 CHRM2
469 NOTCH4
470 CACNG2
471 FKBP8
472 OR8H3
473 CACNA1G
474 CHRNA10
475 THY1
476 CYP7B1
477 HP
478 SERPINA13P
479 FER
480 WNT5A
481 ALDH3B2
482 PDK2
483 SDC1
484 ATP4B
485 EYS
486 CAT
487 KCNJ6
488 OR5M8
489 NTS
490 CYP3A43
491 PAH
492 CASP7
493 HRAS
494 MINPP1
495 MMP9
496 ABL1
497 TEC
498 OR5D3P
499 KCNAB1
500 SPINT4
501 LMCD1
502 PTPN13
503 PARP4
504 OR5H15
505 LOXL1
506 ZG16
507 ADHFE1
508 FCRLA
509 SLC19A1
510 CCR4
511 IL21
512 MSMP
513 SELENOP
514 CCL25
515 RLN3
516 RPN2
517 GPA33

518 OR7E149P
519 BMX
520 PMEL
521 NAAA
522 RIOK3
523 PAK3
524 OR5M5P
525 IL24
526 RPS27
527 SLC28A3
528 NTRK3
529 CCL2
530 SERPINF1
531 HDAC11
532 PIP5K1A
533 PRL
534 SRMS
535 SLC1A6
536 ABCF3
537 HMOX1
538 ALPI
539 GAL
540 OLFM2
541 CASP12
542 OR8U8
543 CD109
544 DDOST
545 CA13
546 PKDREJ
547 CST8
548 STAT3
549 NFATC3
550 CYP2W1
551 OR8B2
552 OXGR1
553 CDK19
554 SLC25A13
555 ALOX15B
556 RNPEP
557 MYLK4
558 OR52H1
559 ABCB9
560 MAP3K4
561 ITGA2B
562 NPSR1
563 LILRA2
564 BMP1
565 PDCD1LG2
566 ACKR4
567 DPP10
568 EWSR1
569 SIGLEC7

570 AZU1
571 LIPH
572 EMILIN1
573 CHRNE
574 GRK4
575 PTPRN2
576 RNGTT
577 LDHB
578 LGALS1
579 GAPDHS
580 FGF5
581 OR13G1
582 EDIL3
583 DEFA1B
584 HCN4
585 IMPG1
586 PTPRJ
587 FUT2
588 IL1RL1
589 LRP1
590 DEFB105B
591 LPAR5
592 ITGB8
593 GPRC5B
594 ADH7
595 CYP27A1
596 SLC25A31
597 ABCA10
598 RXRG
599 CDKL1
600 CHRNB2
601 HSD17B8
602 TARS1
603 SIRT3
604 IFNA10
605 MARK4
606 TXNDC9
607 TAOK2
608 DEFB104B
609 NAMPT
610 S1PR4
611 MYLK
612 FABP2
613 IGLC3
614 UGT2B28
615 MAP3K1
616 PTPN23
617 TAC1
618 CR2
619 SGK3
620 AIFM2
621 RIPK1

622 WNT5B
623 PTH2
624 AHSG
625 KLHL30
626 TMPRSS11D
627 FRZB
628 SLC28A1
629 TMPRSS5
630 CCND3
631 BMP4
632 CCNE2
633 CLCA1
634 TLR6
635 OR6X1
636 IKBKG
637 COL4A2
638 FLNA
639 IL18RAP
640 ALPL
641 TNFSF14
642 GSTA1
643 DHODH
644 PDIA3
645 ACACA
646 TRIM17
647 CYSLTR1
648 SMN1
649 SLC6A17
650 CFHR2
651 SLC4A5
652 CSPG5
653 FGFBP2
654 MBOAT4
655 HSD3BP1
656 CD99
657 UCMA
658 LEFTY1
659 CPN2
660 KLHL26
661 LRRK1
662 FBN3
663 TRIM60
664 VAT1
665 ADAM33
666 OR8B12
667 NFATC4
668 WWOX
669 IGKV1D-39
670 PDE10A
671 ACRBP
672 NPPC
673 CD82

674 SHH
675 DRD4
676 RTN4
677 CD27
678 AAK1
679 INPPL1
680 NME9
681 LILRB4
682 ITIH3
683 CHRNA2
684 ROCK1
685 PRTFDC1
686 CEACAM7
687 OR2H5P
688 TOR2A
689 MAP3K3
690 PLSCR2
691 TSPO2
692 SAA1
693 KCNU1
694 PROC
695 SERPINA11
696 RNPEPL1
697 B4GALT1
698 LY86
699 GSTZ1
700 MKNK2
701 TMX1
702 MTNR1B
703 RABGGTB
704 CHRNB1
705 PRTN3
706 CMYA5
707 DEFB116
708 DHRS1
709 CLCN6
710 SERPINB9
711 CNR1
712 MTRNR2L5
713 ADGRG2
714 AHR
715 TRIM16L
716 PSEN2
717 PLCXD2
718 ADRB3
719 TRIO
720 PRPF4
721 LHCGR
722 OR13D1
723 SLPI
724 GFER
725 CCNA1

726 CXCR5
727 KLK1
728 OPN1MW
729 MYT1
730 SLCO6A1
731 TNFSF11
732 EDAR
733 RTN3
734 LARS1
735 OR5T2
736 PM20D1
737 ABCC12
738 OR2W6P
739 ADA
740 OR1G1
741 GRIK2
742 HDAC1
743 GRIN2A
744 CFHR5
745 IFNA16
746 SLC16A7
747 VIPR1
748 HLA-DPA1
749 FKBP15
750 NXPE1
751 TPCN1
752 EDNRB
753 FOLR2
754 OC90
755 OR4A15
756 SLC9C1
757 IL4R
758 FGF18
759 TNFRSF8
760 PLA2G12A
761 FKBP7
762 ABCB7
763 PTPRZ1
764 PLK3
765 UCP1
766 HDAC8
767 AGRP
768 ADAL
769 UGT2B10
770 TMPRSS6
771 ATP2A1
772 SLC25A3
773 CTSK
774 OR52Z1P
775 CDC14C
776 UGT1A3
777 GLRA4

778 SPP1
779 DBF4
780 ITGB4
781 SST
782 CTSL3P
783 CCNB1
784 KLRC1
785 ATP2B4
786 PRKD3
787 AHCYL2
788 OR10V1
789 DLK1
790 TP53
791 MRGPRF
792 CTSD
793 GSTA4
794 SLC5A4
795 MLX
796 GRIA3
797 SSR2
798 WNK1
799 OPRPN
800 HSDL1
801 CCN2
802 TUBB8
803 CCL8
804 INHBC
805 OPRK1
806 ITLN2
807 IGFBP1
808 CSF1
809 IFNA17
810 CHRDL2
811 CPAMD8
812 LEP
813 MOXD1
814 SFRP2
815 PLAUR
816 ENDOU
817 ADH6
818 GSTM4
819 HVCN1
820 ATP6V1B1
821 OR2A12
822 TRIM27
823 F13B
824 OR10G3
825 IL17RB
826 ANGPTL3
827 OR11G2
828 BCL2L15
829 CA3

830 THRA
831 COL6A5
832 C6
833 BLK
834 IL13RA2
835 MARS1
836 CLK2
837 UPK3A
838 SLC37A1
839 OR8G1
840 CCND1
841 JAK1
842 DAPK2
843 SLC25A33
844 STC1
845 ITPR3
846 SBK3
847 CA5A
848 METAP1D
849 NECTIN2
850 SLC7A5
851 INHBE
852 ABHD12
853 AMHR2
854 KCNK10
855 DEFB115
856 LCN9
857 RRH
858 GLB1
859 CHST6
860 IL4I1
861 PTGR2
862 FCAMR
863 ALDH8A1
864 ANTXR2
865 CACNG3
866 PPIH
867 SCNN1G
868 GFM1
869 KRT8
870 RNLS
871 DYRK3
872 H4C1
873 GPLD1
874 FKRP
875 TMX3
876 GGT7
877 APLNR
878 NR4A1
879 NR6A1
880 IL17A
881 SIK3

882 TIMP1
883 RSPRY1
884 CD33
885 OR4C2P
886 ADAMDEC1
887 SERPINB12
888 OR51B4
889 GH2
890 TMPRSS15
891 NLGN4X
892 NEK10
893 IL34
894 TGFB3
895 NME8
896 IGHG1
897 GPR19
898 SLC25A19
899 ACVR2B
900 DIO2
901 OR2M7
902 COL21A1
903 BSG
904 KLK8
905 ELAVL1
906 ABCC10
907 KSR2
908 HIPK2
909 ADAMTS13
910 FAT3
911 F9
912 ADM2
913 SREBF2
914 LIPJ
915 RAMP1
916 CD1D
917 OPN5
918 ATRAID
919 ENOX1
920 VAMP2
921 PCYOX1L
922 ADGRG3
923 NEU1
924 RBM10
925 GPR45
926 GRM4
927 PHF8
928 JAK3
929 OR4M1
930 SLC7A13
931 CD69
932 C3AR1
933 LOXL4

934 IHH
935 SERPINA3
936 IL10RA
937 CDKN3
938 CCRL2
939 COL4A6
940 SDR42E2
941 XYLT1
942 TUBA1C
943 NID2
944 COL9A2
945 OR7G3
946 SERPINH1
947 GRIA4
948 SLC2A13
949 PLIN2
950 CLEC14A
951 SLC25A41
952 CTSE
953 HYAL2
954 OR2F2
955 EPHA3
956 INPP5B
957 SYT15
958 SLC15A5
959 CTSO
960 EPHA6
961 TFF3
962 DAPK1
963 RARG
964 OR4D10
965 TYRO3
966 ADAM29
967 TBXA2R
968 PRPS2
969 TRPC6
970 ADGRD1
971 SPNS1
972 GGT1
973 NR1H3
974 HSD11B2
975 CNTNAP5
976 MTMR1
977 SEMA3G
978 EEF2K
979 SERPINA4
980 PGLYRP3
981 ANGPTL1
982 ITGB2
983 MAP3K12
984 CRTAP
985 ANXA2

986 EIF2AK4
987 SCN1A
988 PTPRB
989 MAOB
990 CMKLR2
991 AVPR1B
992 HTRA4
993 PPME1
994 NEK8
995 AXL
996 NEK2
997 FKBP1A
998 BTC
999 CLEC10A
1000 ZRANB2
1001 OPTC
1002 GDF11
1003 GPR161
1004 OR4E2
1005 EGF
1006 CNDP2
1007 LGR4
1008 OR51I2
1009 SLC7A3
1010 LMTK3
1011 SLC22A9
1012 KCNK16
1013 MAST1
1014 OR8H1
1015 LEAP2
1016 PNMT
1017 IGHE
1018 ATP2C1
1019 QDPR
1020 IL9R
1021 MAPK7
1022 H3C2
1023 NISCH
1024 OR1S1
1025 CAMK1D
1026 AKR1B1
1027 OR4C5
1028 SCG2
1029 PTGIR
1030 PLA2G12B
1031 MAP3K21
1032 KCNJ4
1033 OR2AT4
1034 C1R
1035 PYGL
1036 OR2AE1
1037 TRPC5

1038 IL7
1039 BTD
1040 PI4KA
1041 MMP24
1042 TUBB2B
1043 HSDL2
1044 IFIT1
1045 HLA-DQA2
1046 NMBR
1047 TAF1A
1048 ZADH2
1049 MASTL
1050 ESRRB
1051 OR10A7
1052 INS
1053 MRGPRE
1054 OR3A4P
1055 GPR139
1056 BMP2K
1057 KLRB1
1058 EFNA5
1059 P2RY6
1060 RNF135
1061 ABCC11
1062 TACR1
1063 SLC22A5
1064 PHKG1
1065 GH1
1066 NPPB
1067 SLC6A14
1068 CD209
1069 EFL1
1070 LAMP2
1071 GPR78
1072 FGFR3
1073 SLC16A8
1074 OR2B6
1075 PDIA5
1076 ART1
1077 DRD1
1078 OR5M11
1079 SERPINB6
1080 MKNK1
1081 CNMD
1082 MEPE
1083 RSPO4
1084 CDC14B
1085 BOLA1
1086 SV2C
1087 CCL1
1088 GPR12
1089 CD320

1090 PTK6
1091 HAPLN2
1092 ECEL1
1093 PENK
1094 IL17F
1095 SIGLEC10
1096 OXER1
1097 C1RL
1098 GPM6A
1099 CACNA1H
1100 ADAMTS12
1101 GRIA1
1102 MINK1
1103 SULT1E1
1104 CD80
1105 SSR1
1106 KDM4A
1107 TRGV3
1108 PGLYRP4
1109 TXNDC8
1110 TMPRSS11A
1111 GPR33
1112 TDO2
1113 SLC25A14
1114 FCGR3A
1115 PIGT
1116 AMY2A
1117 LARS2
1118 FKTN
1119 NDP
1120 GRP
1121 VSIG2
1122 ZAP70
1123 AMPD2
1124 SLC27A1
1125 SLC16A2
1126 PDF
1127 OR10H1
1128 FREM3
1129 PRSS45P
1130 ACP5
1131 GUCY1B2
1132 PTPMT1
1133 PLBD1
1134 PEBP4
1135 OR2H1
1136 LAMB4
1137 GREM1
1138 PLTP
1139 ASPRV1
1140 SCN1B
1141 AGTPBP1

1142 CPE
1143 DHRSX
1144 RELB
1145 PCSK7
1146 GABRR3
1147 NOCT
1148 TUBA8
1149 AMH
1150 OR5BS1P
1151 NOTCH3
1152 NUAK2
1153 PARP8
1154 CORIN
1155 IPP
1156 DEFA6
1157 XCR1
1158 APOBEC3G
1159 OR51E1
1160 PIM2
1161 MAP4K4
1162 TMEFF2
1163 LEFTY2
1164 PMCH
1165 IGKV2D-40
1166 VARS2
1167 CD164
1168 PYGB
1169 OR51H1
1170 CASP9
1171 PI4K2A
1172 NRDC
1173 TREM1
1174 SGK2
1175 THBS4
1176 COL3A1
1177 CYP39A1
1178 VAMP7
1179 ARSD
1180 KLHL33
1181 PRPS1L1
1182 UGT1A6
1183 ELSPBP1
1184 SHPK
1185 CASP4
1186 TNFRSF25
1187 OR4K5
1188 LTB
1189 NPC1
1190 CARTPT
1191 PA2G4
1192 GJB1
1193 TRIM10

1194 OPN1SW
1195 PDGFA
1196 COL8A1
1197 OR52N5
1198 BCL2L2-PABPN1
1199 GRIA2
1200 OR52D1
1201 LCN2
1202 OR51C1P
1203 SELP
1204 GRIK5
1205 PTPN11
1206 TRIM4
1207 CHST12
1208 C1QL1
1209 PROKR2
1210 MMP16
1211 KLHL4
1212 CDK20
1213 NPLOC4
1214 COL7A1
1215 FDFT1
1216 OR2AJ1
1217 UGT1A1
1218 CD19
1219 CDC42BPA
1220 P3H3
1221 ATP2B1
1222 LGALS8
1223 CBLN3
1224 KCND1
1225 VEGFD
1226 MAPK15
1227 ERVK-6
1228 SLC25A23
1229 OR7A10
1230 IVNS1ABP
1231 DCN
1232 SAE1
1233 SLC25A35
1234 OR5B3
1235 GDF3
1236 QPCT
1237 EEPD1
1238 TCN2
1239 SCP2
1240 ANGPT2
1241 HDAC9
1242 RNASE6
1243 DUSP15
1244 OR8D4
1245 EGLN2

1246 CALM3
1247 PRPF4B
1248 ABCE1
1249 RNF31
1250 FGF16
1251 ACVR2A
1252 SNAP25
1253 SERPINI1
1254 MORC1
1255 PARP2
1256 RGS12
1257 CD5L
1258 CTSF
1259 ENPP6
1260 BTK
1261 RFPL2
1262 OR11H13P
1263 TAC4
1264 SFTPB
1265 LRRN3
1266 STYX
1267 AGBL5
1268 PLK5
1269 PPIA
1270 CCL27
1271 PDE6H
1272 DTX2P1
1273 COQ8A
1274 MAP2K4
1275 THRB
1276 ENPP1
1277 PPIAL4D
1278 PSMG3
1279 PADI3
1280 OR8G2P
1281 UGT1A7
1282 SIGLEC5
1283 GABRD
1284 NBL1
1285 PRSS27
1286 CYP1A2
1287 EPB42
1288 BCR
1289 ATP2B3
1290 SCGB2A1
1291 KLHL35
1292 CSNK1A1
1293 ABCA2
1294 ATP1B2
1295 ODR4
1296 PGBD1
1297 SLC25A26

1298 EP300
1299 KDR
1300 GABBR1
1301 OR5M3
1302 RANBP2
1303 GIP
1304 FGF19
1305 CXCL5
1306 MMP8
1307 PTGS1
1308 KLK13
1309 MDK
1310 SCD5
1311 JAML
1312 CSNK1G3
1313 MTOR
1314 DUSP16
1315 IL1R1
1316 CD70
1317 OR7E31P
1318 OR5AQ1P
1319 GALP
1320 RXFP1
1321 TPCN2
1322 H3C10
1323 MCL1
1324 ERG
1325 CNGA4
1326 SLC16A14
1327 OR4N2
1328 CD58
1329 ELOVL3
1330 GPR63
1331 CA11
1332 PTPN18
1333 PPARG
1334 APOC3
1335 IGKV3-11
1336 CD93
1337 INPP5E
1338 NTSR1
1339 GABRA5
1340 FASLG
1341 OR51V1
1342 CFB
1343 KCNB1
1344 FXYD2
1345 OR5G1P
1346 SIGLEC1
1347 CELSR1
1348 ULK3
1349 DEFA1

1350 GSTA2
1351 EPGN
1352 HSD3B1
1353 HEBP1
1354 VIPR2
1355 DAND5
1356 DNASE1
1357 PPY
1358 CHIA
1359 ERN1
1360 WNT6
1361 OTOS
1362 INSL3
1363 SLC2A2
1364 OR2T29
1365 GPR34
1366 CD72
1367 WNK4
1368 RPS6KA5
1369 CXCL12
1370 MFSD3
1371 GLIPR1L1
1372 PTPN14
1373 AGPAT2
1374 CRHR1
1375 KCNK1
1376 CHRND
1377 NEK9
1378 ALCAM
1379 IFNL2
1380 NCR1
1381 PDK1
1382 RNASE4
1383 CDK5
1384 COL4A4
1385 HTR1B
1386 IFNA2
1387 OR4C13
1388 GPR142
1389 SIGLEC12
1390 MATN3
1391 CD7
1392 UQCRC2
1393 OR7C1
1394 TTR
1395 SIRPB1
1396 PRH1
1397 CD48
1398 OR7D2
1399 GMPR2
1400 PRPSAP2
1401 PDE6C

1402 DKK1
1403 FSD1L
1404 APLP1
1405 LTBP3
1406 DNASE1L1
1407 PPBP
1408 CNTN2
1409 PDE5A
1410 NOX5
1411 IL12A
1412 CACNA1B
1413 HLA-DRA
1414 PRKCB
1415 HHIP
1416 H4C9
1417 OR4F5
1418 FES
1419 KCNB2
1420 CA2
1421 UCP2
1422 CDK3
1423 BMP6
1424 RPS6KA3
1425 SLC47A1
1426 PPIG
1427 KAT2B
1428 SLAMF6
1429 HMGB2
1430 IARS1
1431 CPT1B
1432 RAET1G
1433 SULT1A2
1434 CYP1B1
1435 HPX
1436 RPS6KA6
1437 SCUBE1
1438 RARA
1439 FGF10
1440 OR52M1
1441 P2RX3
1442 C4A
1443 KBTBD11
1444 OR51A6P
1445 APOBEC3A
1446 GKN2
1447 CDK14
1448 QRFPR
1449 TEX101
1450 ADGRV1
1451 GRK6
1452 HRH4
1453 VWC2

1454 LAMC3
1455 RSPO2
1456 SFTPA2
1457 CBR3
1458 SLC5A8
1459 SAMD1
1460 NNMT
1461 NRTN
1462 LVRN
1463 SULT1A3
1464 SNRK
1465 PTPRN
1466 CXCL10
1467 NTN4
1468 SAA2
1469 DEFB114
1470 TSSK2
1471 ABCB10
1472 ADORA3
1473 TNFSF15
1474 PGA4
1475 AHCYL1
1476 CCN6
1477 PSMD14
1478 NELL2
1479 KCNH2
1480 UGT1A10
1481 USP7
1482 PRSS42P
1483 ATP1A1
1484 L3MBTL1
1485 CXCL2
1486 PRKAA2
1487 FCRL3
1488 TUBA1A
1489 KDM5C
1490 GPR157
1491 CYP2C18
1492 PARP10
1493 FDCSP
1494 SLC25A44
1495 TUBB8B
1496 SEMA3D
1497 PRLH
1498 UCN2
1499 OR5A2
1500 NR0B1
1501 TXNRD3
1502 OR9A4
1503 TXK
1504 SENP8
1505 MFSD5

1506 SCYL2
1507 CYP4X1
1508 PGC
1509 LIG1
1510 NAGA
1511 SI
1512 CAPZA1
1513 KCNK15
1514 OR10A5
1515 UCN3
1516 OVCH1
1517 CLPS
1518 LAG3
1519 CPM
1520 ADCY10
1521 MAP3K9
1522 TBXAS1
1523 IL20RB
1524 PSMB10
1525 RAMP2
1526 NAALAD2
1527 DLL4
1528 TNIK
1529 PKN1
1530 EPHA5
1531 BTN3A2
1532 F2RL3
1533 CACNA2D4
1534 ITIH4
1535 TNXB
1536 IL18R1
1537 CCR10
1538 BRD4
1539 TMLHE
1540 TOP2A
1541 CYP4F2
1542 NFKBIA
1543 H1-2
1544 RXFP4
1545 FCN2
1546 MAN2B2
1547 TRPM4
1548 SLC1A3
1549 GLP2R
1550 OR10AD1
1551 MMP23A
1552 HCAR1
1553 MRGPRG
1554 ADRA2B
1555 ANGPTL7
1556 CLK4
1557 SLC5A7

1558 ADGRA2
1559 IL26
1560 RASGRP3
1561 HDC
1562 PGLYRP1
1563 MTCH2
1564 PPARA
1565 CYP11B1
1566 RESP18
1567 CA4
1568 THOP1
1569 TXNDC15
1570 A1BG
1571 ACVRL1
1572 KCNS2
1573 ECRG4
1574 SPRYD4
1575 KAZALD1
1576 SLC22A8
1577 SCN8A
1578 STK24
1579 ADA2
1580 PDE4B
1581 H3-3A
1582 EGFR
1583 BAD
1584 CROT
1585 CDK18
1586 MGMT
1587 IGHV1-2
1588 PKD2
1589 NR2C1
1590 SLC34A2
1591 PSMB6
1592 HPGDS
1593 EDA
1594 HTR3C
1595 TMIGD2
1596 LRRK2
1597 KBTBD12
1598 PBK
1599 GUSBP1
1600 GPR52
1601 TENM1
1602 TSPEAR
1603 OR52W1
1604 KLRD1
1605 DNAJC16
1606 FST
1607 GSTP1
1608 HPN
1609 CFH

1610 ADAMTS20
1611 ERAP1
1612 ADAM30
1613 OR51B2
1614 PKHD1
1615 KCNK13
1616 TSTD1
1617 AMPD3
1618 FUT1
1619 SDC2
1620 MATN4
1621 SLC28A2
1622 BIRC2
1623 NPR3
1624 NTN1
1625 RHBG
1626 ASIP
1627 STAT5B
1628 GBE1
1629 FABP4
1630 KLK9
1631 AHCY
1632 CAMK2A
1633 FLRT2
1634 BACE1
1635 DHRS12
1636 OR2T5
1637 ALDH9A1
1638 CA7
1639 ABCG2
1640 CYP17A1
1641 SPP2
1642 OR10G7
1643 DUSP22
1644 TFRC
1645 LAMP1
1646 RAB9A
1647 VSIG4
1648 BRAF
1649 OR4C12
1650 LILRB1
1651 SLC37A2
1652 SPRYD3
1653 ENPEP
1654 ALPG
1655 SLC1A1
1656 CBR4
1657 TNFRSF10A
1658 MFAP1
1659 TAP1
1660 GPR135
1661 CXCR6

1662 CPA2
1663 COLQ
1664 TRIB1
1665 POLA1
1666 PRF1
1667 WNT2B
1668 MLH1
1669 M6PR
1670 ESR2
1671 KLHL1
1672 SCNN1B
1673 APCS
1674 IMPDH1
1675 LAMA2
1676 BCL2L10
1677 TMEM94
1678 CYP2C9
1679 OR5L2
1680 RFPL1
1681 CCL7
1682 PDIA2
1683 MRGPRX4
1684 IFNK
1685 HLA-A
1686 YWHAG
1687 IL17C
1688 NECTIN1
1689 H4C11
1690 OCRL
1691 OR4S2
1692 PLK2
1693 POGLUT1
1694 APOC1
1695 SLC2A8
1696 MFSD10
1697 PRKCD
1698 SLC3A1
1699 ERAP2
1700 FCRL5
1701 IDE
1702 TAB2
1703 GPNMB
1704 OR10G2
1705 KCNQ2
1706 THBD
1707 OR51N1P
1708 CASQ2
1709 CALCB
1710 GPR101
1711 GPRC5D
1712 OR2T10
1713 MGST2

1714 TOP1MT
1715 KCNA5
1716 MAPKAPK2
1717 SERPINB4
1718 HTR1E
1719 SLC4A3
1720 CCL15-CCL14
1721 BRD3
1722 CDNF
1723 OR5H2
1724 C1S
1725 HHAT
1726 OR4H12P
1727 IDS
1728 S100A9
1729 LILRA1
1730 OR1K1
1731 GMPR
1732 CATSPER2
1733 PRSS8
1734 RAPGEF4
1735 FPR2
1736 CTSG
1737 OR2AG1
1738 OR2D2
1739 OR9K1P
1740 FCRL6
1741 HSD17B13
1742 NQO2
1743 OR4P1P
1744 SLC43A2
1745 PCDHA2
1746 CHAT
1747 MAS1
1748 ART4
1749 TPBG
1750 TRIM35
1751 KLHL20
1752 ALDH3A2
1753 APOD
1754 CD274
1755 MAPT
1756 PLD2
1757 PSMA1
1758 FUCA1
1759 COL6A2
1760 ADAM15
1761 MMRN2
1762 ELN
1763 IL3RA
1764 CA10
1765 SENP6

1766 PSG2
1767 NOX1
1768 PAK1
1769 DRD2
1770 UGT1A8
1771 CYP2A6
1772 OR10S1
1773 OR5P1P
1774 PRKACA
1775 ADGRE5
1776 PCSK2
1777 ODC1
1778 MCOLN3
1779 GPX1
1780 CPB1
1781 RARB
1782 MAP3K6
1783 PLCB1
1784 IL2RG
1785 REG1B
1786 AMPD1
1787 SEMG2
1788 DHRS2
1789 CXCR2
1790 APEX2
1791 CASP1
1792 SLC15A2
1793 TNFRSF10D
1794 CYP21A2
1795 WARS2
1796 KIRREL2
1797 CSNK2A2
1798 GGT6
1799 TPSAB1
1800 GABRR1
1801 FBLN7
1802 CRISP2
1803 MTMR7
1804 NPBWR2
1805 SLC3A2
1806 SLC9A7
1807 SLC25A37
1808 OR10D3
1809 MFSD14A
1810 FMO2
1811 ATIC
1812 DMBT1L1
1813 CA8
1814 PLSCR3
1815 MMP11
1816 SERPINB3
1817 IL11RA

1818 DECR1
1819 SPACA5B
1820 HNMT
1821 NFATC1
1822 IMPG2
1823 HHATL
1824 PAPPA2
1825 TECTA
1826 ACY1
1827 KLK3
1828 WFIKKN2
1829 S100B
1830 OR7A15P
1831 OR1E2
1832 RELT
1833 UGT8
1834 CD207
1835 HDAC7
1836 NPFF
1837 AVPR2
1838 MYLK3
1839 MGAM2
1840 OXSR1
1841 SLC16A11
1842 EPHA1
1843 SLC9C2
1844 IL13RA1
1845 COX7B
1846 FUCA2
1847 GGTLC2
1848 GZMM
1849 TUBB1
1850 IGLV3-16
1851 GP1BB
1852 EIF4A1
1853 TAAR1
1854 CRISP1
1855 PDCD4
1856 SLC18A3
1857 PODNL1
1858 ZP1
1859 ARSL
1860 OMG
1861 LRG1
1862 LAMA3
1863 TIMP4
1864 CST6
1865 HSP90AA2P
1866 IL6ST
1867 ATP1B3
1868 BDNF
1869 TXNL1

1870 NLRP3
1871 COQ6
1872 GRPR
1873 ENPP5
1874 OR1J4
1875 FGFR2
1876 DUT
1877 TH
1878 PRKACG
1879 FMO3
1880 SLC25A51
1881 MPST
1882 DMBT1
1883 SMS
1884 GABRA6
1885 RNASE2
1886 MANF
1887 MFSD12
1888 MME
1889 OR5T3
1890 CACNA1I
1891 OR8J3
1892 DNMT1
1893 OR14A16
1894 ATP1A4
1895 PTPRU
1896 PSKH2
1897 ADAMTS18
1898 PLA2G2E
1899 EFNB1
1900 SULT4A1
1901 PRDX4
1902 OR5M1
1903 CYP4F3
1904 NDFIP1
1905 RDH10
1906 ABCA13
1907 TRIM36
1908 HTR2A
1909 HRH1
1910 PGA3
1911 NOD2
1912 MAP2K3
1913 DERL1
1914 BLMH
1915 PROCR
1916 TRPM1
1917 DPEP1
1918 CRH
1919 DSCAM
1920 RORA
1921 HLA-DOB

1922 CGB3
1923 GALC
1924 OR6B1
1925 LAMA5
1926 KHDRBS1
1927 TAB1
1928 NMT1
1929 TUBE1
1930 RB1
1931 CELA2A
1932 STK32B
1933 SLC16A10
1934 SLC22A13
1935 STRADB
1936 CXCL13
1937 THBS2
1938 KNG1
1939 OR4P4
1940 NUP153
1941 CCL21
1942 QSOX1
1943 CD59
1944 SLC17A5
1945 B2M
1946 MAPK1
1947 TIPARP
1948 CNOT6L
1949 LIMK1
1950 JUN
1951 COL5A1
1952 PDZD2
1953 PGF
1954 CYP2A13
1955 OR7E8P
1956 CAPN5
1957 CYP51A1
1958 RYK
1959 OR10D4P
1960 IGHV1-46
1961 CKLF
1962 SERPINF2
1963 GBAP1
1964 CRB2
1965 CXCL14
1966 TAAR9
1967 ADGRB1
1968 KCNK18
1969 OR4C3
1970 SLC16A1
1971 PDE7B
1972 IL17D
1973 BCL2L14

1974 PCSK1N
1975 OR2G2
1976 MFSD4B
1977 SERPINB1
1978 KCNA3
1979 OR52K1
1980 SLC25A25
1981 DRAXIN
1982 NUAK1
1983 CD163L1
1984 OR2T2
1985 OR8B4
1986 OR6Q1
1987 CSNK1G2
1988 SPINK5
1989 SLC22A3
1990 SLAMF8
1991 TXNRD1
1992 MFSD11
1993 CSH1
1994 MARK1
1995 ABCA8
1996 COL10A1
1997 ANGEL1
1998 HSD17B1
1999 PRSS1
2000 GPR83
2001 STATH
2002 MAP4K3
2003 SLC9A4
2004 FGF22
2005 MMP1
2006 KALRN
2007 TNFRSF19
2008 OR5B21
2009 C1QL2
2010 CDH1
2011 OR5L1
2012 NGFR
2013 SYNJ1
2014 PLCH1
2015 SLC25A43
2016 APRT
2017 SLC39A5
2018 SLC37A3
2019 SDR16C5
2020 OGA
2021 ACVR1
2022 CYP2G1P
2023 FBLN5
2024 COL9A3
2025 KLK10

2026 TF
2027 HTN1
2028 PGR
2029 NFATC2
2030 OR5C1
2031 SLC6A8
2032 LTC4S
2033 NR1D1
2034 ADCY6
2035 ITPR2
2036 SLC12A9
2037 PMS2P1
2038 AMTN
2039 DEFB113
2040 CPD
2041 UMOD
2042 SCRG1
2043 IGKV1D-16
2044 CSF3
2045 FFAR3
2046 SERPINE3
2047 IGHD
2048 IGLC2
2049 CCN3
2050 ADAMTS10
2051 LYPD6
2052 OR10AC1
2053 TKT
2054 AKT1
2055 MFSD14B
2056 NPY2R
2057 H3C12
2058 GRM6
2059 ILK
2060 VGF
2061 SLC5A2
2062 ADAMTS14
2063 CGREF1
2064 FPR3
2065 OR1D4
2066 SLC9A1
2067 PREP
2068 ZC3HAV1
2069 HASPIN
2070 PDXK
2071 BIRC5
2072 PNCK
2073 ADH4
2074 SARAF
2075 CATSPER1
2076 IL31
2077 PROKR1

2078 TAOK1
2079 PMP22
2080 PSMB9
2081 ADH5
2082 WNT9B
2083 IGKV2D-29
2084 DOT1L
2085 HCRT
2086 PDE3A
2087 FLT1
2088 METAP2
2089 AKR1C6P
2090 IGKV4-1
2091 ADIPOQ
2092 S100A7
2093 PKD2L1
2094 SLC4A9
2095 ARAF
2096 PLA2G2D
2097 ATP4A
2098 TGM2
2099 UCHL3
2100 SERPINE2
2101 GLP1R
2102 PKN2
2103 OR1E1
2104 HCAR3
2105 PLPBP
2106 CHST11
2107 DCPS
2108 GSTCD
2109 DIO1
2110 KLHL12
2111 OR14A2
2112 FPR1
2113 GPR183
2114 NRK
2115 SELENOS
2116 FAS
2117 KCNK6
2118 TRIB3
2119 PDIA6
2120 IMPA2
2121 GPC6
2122 NOTCH2NLA
2123 VARS1
2124 RSPO1
2125 BICD1
2126 SLC12A6
2127 SLC2A4
2128 ADCY7
2129 TFF2

2130 CD1B
2131 CPXM1
2132 PLK1
2133 ERN2
2134 CDKL5
2135 SRGN
2136 HAPLN4
2137 MBOAT2
2138 ADAM2
2139 METRN
2140 TUFT1
2141 CDH13
2142 SLC9A8
2143 CSN2
2144 SLC6A6
2145 LAMB3
2146 ITGB1
2147 CHID1
2148 DHRS7C
2149 CPZ
2150 APOM
2151 GPR61
2152 ANG
2153 CLEC11A
2154 PYCARD
2155 PTPN7
2156 SEMA4D
2157 PLCD1
2158 CD200
2159 ADK
2160 SPNS3
2161 PI4KAP2
2162 HSP90AB1
2163 OR7E5P
2164 MICAL2
2165 ADAMTS9
2166 MAPK14
2167 CCR9
2168 ABCC4
2169 CACHD1
2170 SV2A
2171 GAN
2172 MFAP4
2173 SLC2A9
2174 OSM
2175 DEFB105A
2176 LY6K
2177 CD244
2178 OR7A17
2179 ATP2B2
2180 SPOCK2
2181 IL17RE

2182 CPXM2
2183 CSN3
2184 CHST4
2185 ASPN
2186 CRISP3
2187 TSSK1B
2188 MFSD2A
2189 SLC25A12
2190 TUB
2191 TSPO
2192 LIPN
2193 KCNJ10
2194 ASH2L
2195 TYMP
2196 HRH2
2197 NR1H2
2198 STK32C
2199 UPP1
2200 GPR152
2201 TSTD2
2202 OR52K2
2203 FAM20C
2204 CYP7A1
2205 MELK
2206 IL17RC
2207 TRIM62
2208 LAP3
2209 KCNK4
2210 CES4A
2211 SCN3A
2212 SLC25A36
2213 PXK
2214 OR8K3
2215 CR1
2216 OR6C76
2217 SLC16A3
2218 PDCD1
2219 NR1D2
2220 SDR42E1
2221 PAM
2222 LAMB1
2223 NPFFR2
2224 CYP4F22
2225 ESAM
2226 POMK
2227 HIPK4
2228 OR4F21
2229 CDK17
2230 SERPINA12
2231 TM2D1
2232 SLC4A1
2233 ABCD1

2234 NRXN2
2235 AKR7L
2236 MSR1
2237 FGF4
2238 ENPP7
2239 MUC15
2240 OR51G1
2241 TTN
2242 NFAT5
2243 CCNH
2244 LCAT
2245 SSC4D
2246 OR3A3
2247 AURKA
2248 CD300LB
2249 GRK7
2250 CLEC3B
2251 G6PC1
2252 IGLV2-23
2253 PXDNL
2254 PCDHB10
2255 SCGB1A1
2256 ADGRG1
2257 DEFB4A
2258 OR13C6P
2259 OR2A7
2260 SLC12A2
2261 EFNA4
2262 P2RX7
2263 KDM2A
2264 SEMA7A
2265 DCLK2
2266 SSTR1
2267 PTPRA
2268 HCK
2269 HSPB1
2270 MBOAT7
2271 OR51A2
2272 OR2W3
2273 ENTPD1
2274 OR5B12
2275 ALDH3B1
2276 PROCA1
2277 TGM1
2278 HABP4
2279 AMELY
2280 VIP
2281 GPC4
2282 SDC4
2283 CBLN4
2284 SEMA3A
2285 CHRNB3

2286 OR10K2
2287 MRGPRX1
2288 RAB35
2289 FPGS
2290 TAFA5
2291 SLITRK4
2292 ADAM7
2293 SLAMF7
2294 TRPC4
2295 NEK4
2296 BMP3
2297 ABCB1
2298 CELA1
2299 PROS1
2300 KCNJ12
2301 DEFB112
2302 VWF
2303 KCNJ14
2304 DPP6
2305 TAAR3P
2306 GBA
2307 OMD
2308 TNFRSF10C
2309 ATF1
2310 CNTNAP2
2311 OR6C68
2312 ACTN4
2313 OR9I1
2314 CES3
2315 GPR65
2316 SCNN1D
2317 ERVK-11
2318 NLGN3
2319 OR2L8
2320 H4C5
2321 OR1A1
2322 OR4D5
2323 MARK3
2324 GAS6
2325 CCL18
2326 P2RY8
2327 CLCNKA
2328 CDC25C
2329 FN1
2330 F5
2331 FCER2
2332 SLC15A4
2333 LDLR
2334 P4HTM
2335 EVI2B
2336 CCDC3
2337 SEMA4B

2338 FGF8
2339 MAP3K7
2340 GPX3
2341 MAPK11
2342 CDK11B
2343 TAAR2
2344 PCDH15
2345 CHRD
2346 COL14A1
2347 TNFSF13
2348 CLCA4
2349 CDH2
2350 PI4KB
2351 ADRA2C
2352 CALCA
2353 FCRL1
2354 NRXN3
2355 GREM2
2356 ADGRL4
2357 GRN
2358 TNFRSF11A
2359 SPRYD7
2360 EPYC
2361 GYPC
2362 KLHL36
2363 CD5
2364 CNGB3
2365 IFNL3
2366 LEPR
2367 OR6P1
2368 HSPH1
2369 MASP1
2370 SEMG1
2371 OR4K17
2372 RABGGTA
2373 VASH1
2374 GP9
2375 MAK
2376 PTPN20
2377 H4-16
2378 MT-ND4
2379 ADGRL2
2380 MTRR
2381 NLGN4Y
2382 TAS2R16
2383 PLA2G2C
2384 GGCX
2385 ENDOD1
2386 CTNNB1
2387 SPINK9
2388 KCND2
2389 NUCB1

2390 CCR6
2391 H4C15
2392 ARNT
2393 LY75
2394 OLR1
2395 FGF1
2396 KDM4C
2397 KLHL17
2398 TEK
2399 NCOA3
2400 NR2C2
2401 GPR15
2402 PTGDS
2403 CCKAR
2404 MDM4
2405 NODAL
2406 USH2A
2407 HNRNPUL2
2408 OR51M1
2409 OTOL1
2410 TK2
2411 ABCC3
2412 DHRS13
2413 AGTR1
2414 CASP10
2415 ALDH1A3
2416 SLC18B1
2417 PTP4A2
2418 BUB1B
2419 OR2M2
2420 GPR4
2421 ATP2A3
2422 H4C4
2423 IKBKE
2424 TAS1R1
2425 PAEP
2426 FCN3
2427 DPP7
2428 TGFA
2429 CSF3R
2430 OR2L13
2431 IGLV1-36
2432 PDGFRB
2433 SMR3A
2434 FGF3
2435 PLOD1
2436 PTPN2
2437 OR1I1
2438 ABCB8
2439 ESRRG
2440 ALOX5
2441 STAT1

2442 DUSP29
2443 OPRL1
2444 KCNJ5
2445 LAMC1
2446 H3C15
2447 SLC4A10
2448 PSMA6
2449 PIP
2450 CSH2
2451 PRSS58
2452 BPIFB3
2453 LTA4H
2454 GABRG2
2455 OR10A4
2456 NR3C1
2457 STK17A
2458 FARS2
2459 SLC6A16
2460 CYP1A1
2461 GPR173
2462 AGBL4
2463 COL27A1
2464 ITGB3
2465 CPT1A
2466 DUSP19
2467 GSTA5
2468 AMY1C
2469 TRIM68
2470 PARP3
2471 OR2A25
2472 GPX7
2473 PTP4A1
2474 BCL2L13
2475 OR10H3
2476 MAPK8
2477 PMS2P4
2478 ABCC2
2479 SEMA3F
2480 CTDSP1
2481 KARS1
2482 KCNJ2
2483 SUOX
2484 IFI30
2485 IGFBP5
2486 MPZL3
2487 OR4A21P
2488 CLCF1
2489 TYK2
2490 CD38
2491 RRM2
2492 OR2A5
2493 DKKL1

2494 FAP
2495 NTSR2
2496 EDN2
2497 NIM1K
2498 CCK
2499 OR7A2P
2500 OR7E90P
2501 SLC25A16
2502 RFPL4A
2503 SQLE
2504 EPX
2505 OR6Y1
2506 H3C11
2507 RNASEL
2508 SCGB1C1
2509 CYP4A11
2510 HCRTR2
2511 TRIM34
2512 PSG1
2513 PSKH1
2514 PPIE
2515 DEFB106B
2516 MAP3K13
2517 MCHR2
2518 NRG3
2519 GHSR
2520 XPNPEP3
2521 NEIL3
2522 TRPM8
2523 FBXO45
2524 FCGBP
2525 TIMP2
2526 IL33
2527 CREG2
2528 PLA2G15
2529 ITK
2530 CRP
2531 ECM2
2532 CPB2
2533 SLC25A11
2534 SLC6A19
2535 SOGA1
2536 GGTLC1
2537 CDC42BPG
2538 TMPRSS9
2539 HSD17B7
2540 FCGR2A
2541 DPP9
2542 SLC12A1
2543 EPDR1
2544 OR51B6
2545 P2RY1

2546 PPARD
2547 SAA4
2548 PIK3CG
2549 GSS
2550 H4C13
2551 BPIFB2
2552 ICAM3
2553 TAFA4
2554 MTMR2
2555 TDP1
2556 AMOT
2557 MPZ
2558 TG
2559 KL
2560 LILRB5
2561 MFNG
2562 FUS
2563 KCNC3
2564 NALCN
2565 TRIM47
2566 TEX14
2567 MMP14
2568 ACP2
2569 TNFRSF13C
2570 DEFB107A
2571 TGFB2
2572 LINGO2
2573 SLC6A9
2574 BPI
2575 CXCL6
2576 LIPG
2577 DMD
2578 COL4A5
2579 BSPRY
2580 TNNI3
2581 VWA3A
2582 PATE2
2583 MC2R
2584 H4C12
2585 CLCN5
2586 CNTNAP3
2587 PRKDC
2588 PRKCA
2589 OR5D13
2590 SELE
2591 IL6
2592 P4HB
2593 OR6K2
2594 IFNA6
2595 MIPEP
2596 IL23A
2597 DHH

2598 FOLR3
2599 HILPDA
2600 ERMAP
2601 LTB4R2
2602 CACNG8
2603 OR5B17
2604 IGLV4-69
2605 NCR3
2606 ALDH16A1
2607 CDKL4
2608 CPA3
2609 DUSP11
2610 TMX4
2611 KRT18
2612 PLGLB1
2613 H6PD
2614 PTPN12
2615 DNASE1L3
2616 CPQ
2617 PIM1
2618 ADAMTSL4
2619 GPC5
2620 DEFA5
2621 SERPINB8
2622 SACS
2623 OR5A1
2624 NECTIN3
2625 PTPRC
2626 CYP24A1
2627 SLC25A32
2628 OR13C8
2629 APP
2630 PDE11A
2631 ADAMTS19
2632 FCGR3B
2633 MMP19
2634 CTSZ
2635 SCN9A
2636 GRIN3A
2637 MCOLN2
2638 IGKV1D-42
2639 BTNL8
2640 LPAR1
2641 GPRC5C
2642 TGFBI
2643 TRIM21
2644 SSH2
2645 EPHB3
2646 FGFRL1
2647 NEK11
2648 CAMP
2649 FGF9

2650 P2RX4
2651 POTEI
2652 DUSP10
2653 SLC10A6
2654 RPTN
2655 TRIM64
2656 PTK7
2657 PLSCR1
2658 SPAM1
2659 TBK1
2660 TNFRSF18
2661 KCNH3
2662 FLVCR1
2663 TRPV4
2664 KCNN3
2665 ADGRA3
2666 MAS1L
2667 CLUL1
2668 DHFR
2669 IL25
2670 MPL
2671 KERA
2672 ADGRE2
2673 APLN
2674 OR4A47
2675 EDN1
2676 PTPN1
2677 SLC25A47
2678 ARSJ
2679 CD79B
2680 CTRB1
2681 RDH12
2682 LRTOMT
2683 FNTB
2684 KCNQ5
2685 TRRAP
2686 BCL2
2687 GCK
2688 ERBIN
2689 C2
2690 SSTR2
2691 OR56B4
2692 PLA2R1
2693 SIRT2
2694 STK16
2695 IGHG2
2696 NPB
2697 EGLN1
2698 C1QTNF9B
2699 KIR2DL4
2700 CST5
2701 P2RY13

2702 TIMP3
2703 OR7E85P
2704 COL4A1
2705 DECR2
2706 VASN
2707 LAMA4
2708 FASN
2709 TULP3
2710 PF4V1
2711 SECTM1
2712 APH1A
2713 SHISA2
2714 FGF6
2715 TRIM72
2716 MALT1
2717 PKD2L2
2718 SCT
2719 STK32A
2720 HTR3D
2721 GDF5
2722 OR52E2
2723 ITLN1
2724 OTOA
2725 ST6GAL1
2726 IFNL1
2727 CFI
2728 IGHA2
2729 XCL2
2730 PORCN
2731 SCN5A
2732 SLC22A20P
2733 SUMF1
2734 ECE1
2735 LTBR
2736 CRIM1
2737 PRR4
2738 GPR119
2739 SENP7
2740 OR7E87P
2741 BMP7
2742 PTPN22
2743 MRGPRD
2744 FFAR2
2745 DNPEP
2746 SCGN
2747 SLC25A10
2748 DGAT1
2749 ACVR1C
2750 MAPK9
2751 ATP12A
2752 MMP21
2753 LACRT

2754 SGSH
2755 HERC1
2756 AGXT2
2757 CES1
2758 IL27
2759 MCHR1
2760 PDIK1L
2761 PRIM1
2762 ITGA4
2763 TYRL
2764 TMEM25
2765 S1PR5
2766 ALDH2
2767 LYPD5
2768 PLA1A
2769 PTPDC1
2770 BDH1
2771 HSD17B11
2772 TRAP1
2773 FMO5
2774 IGFBP6
2775 LAMA1
2776 HLA-G
2777 SLC17A1
2778 IL2RA
2779 GPR3
2780 ANGPTL4
2781 CDCP2
2782 GPRC6A
2783 HTRA1
2784 ST3GAL2
2785 MID1
2786 TAB3
2787 PDE4D
2788 NDUFA4
2789 BPHL
2790 RBKS
2791 APOB
2792 PTPRF
2793 TLN1
2794 RYR1
2795 GPR22
2796 CATSPER3
2797 SLC25A17
2798 TLR1
2799 OR7D1P
2800 PIK3C3
2801 CHADL
2802 ADAM8
2803 FBN1
2804 PRB4
2805 ISG15

2806 PRKY
2807 GLRA3
2808 SLC7A1
2809 GPR26
2810 TACR2
2811 ITGAV
2812 OR51A4
2813 AOPEP
2814 PI15
2815 ALPP
2816 SPEG
2817 PPOX
2818 PAMR1
2819 CEP41
2820 IGKV3-20
2821 XPNPEP2
2822 ADGRL3
2823 RIPK4
2824 PRSS35
2825 P2RX6
2826 OR2S2
2827 AGER
2828 HRC
2829 IGLV10-54
2830 OR2T12
2831 RPN1
2832 MAP3K20
2833 CSNK1D
2834 HYAL1
2835 MUSK
2836 SLC12A4
2837 MLNR
2838 LTBP4
2839 OR10P1
2840 DPT
2841 ICAM2
2842 PRSS3
2843 OR5BB1P
2844 CCL24
2845 CAMK2B
2846 NPS
2847 COL22A1
2848 OR2M4
2849 SEMA6B
2850 MEFV
2851 IFNA7
2852 DNMT3B
2853 CDK5R1
2854 FCGR2C
2855 PRSS29P
2856 PKMYT1
2857 RXFP3

2858 BOC
2859 FSD2
2860 MGP
2861 IFNA4
2862 SLC22A2
2863 CACNA2D1
2864 SLC17A4
2865 NLGN2
2866 MFSD6
2867 RAD51
2868 SOD3
2869 FKBP5
2870 CHRDL1
2871 DAG1
2872 OR2T27
2873 NID1
2874 BMP8A
2875 GPR148
2876 RXRA
2877 CDC42BPB
2878 TNFRSF12A
2879 ACTN1
2880 DSPP
2881 BMPR1B
2882 PRG4
2883 CACNG4
2884 MLKL
2885 SVOPL
2886 CAMKV
2887 IGIP
2888 LRRC4B
2889 GNAS
2890 VIM
2891 TRPV3
2892 TGFB1
2893 CHEK1
2894 SLC15A1
2895 DUSP3
2896 PROZ
2897 GALE
2898 PRH2
2899 OR7E91P
2900 AKR1C1
2901 GPR42
2902 OR4A16
2903 IFNA14
2904 CYB5AP3
2905 CACNA1S
2906 BPIFB1
2907 GAST
2908 HEPH
2909 GAD1

2910 MFSD4A
2911 NTNG1
2912 DRD5
2913 MAP3K2
2914 PROK2
2915 NTF3
2916 HSPG2
2917 TRPC7
2918 ZACN
2919 ALDH3A1
2920 GALR3
2921 STK35
2922 HTR4
2923 DUSP2
2924 ADGRL1
2925 TUBA1B
2926 TMPRSS3
2927 STAT5A
2928 COL6A1
2929 NMUR2
2930 RDH16
2931 LRRC55
2932 SLC7A9
2933 SPINT2
2934 LRIG3
2935 MPO
2936 HPSE
2937 NAPSB
2938 FABP1
2939 OR2G6
2940 S100A13
2941 GPR182
2942 GCLC
2943 PLCZ1
2944 BRS3
2945 CPS1
2946 IL1RL2
2947 NCSTN
2948 VN1R1
2949 ALDH1A2
2950 FKBP6
2951 PCOLCE
2952 PRKCZ
2953 OR52B6
2954 MIF
2955 LY9
2956 KLK6
2957 KIR3DL2
2958 MST1L
2959 IDSP1
2960 CYP4F8
2961 NPPA

2962 NPY
2963 GPR32
2964 ENOX2
2965 PRSS53
2966 MTCH1
2967 GPR158
2968 C7
2969 HKDC1
2970 PPIL2
2971 DEFA3
2972 SMR3B
2973 CDC25A
2974 GRM1
2975 MAP2K7
2976 ABCA1
2977 CACNA1E
2978 ANGPTL6
2979 ABCB6
2980 PDGFRL
2981 OR7C2
2982 IL5RA
2983 GFPT1
2984 SLC45A3
2985 NR2F1
2986 SLCO4A1
2987 TRIM5
2988 UGCG
2989 OR6A2
2990 OR7A5
2991 CIT
2992 EPHB4
2993 LPA
2994 GPBAR1
2995 DNMT3A
2996 FOLH1
2997 OPN3
2998 TPSD1
2999 JAM2
3000 SLC44A4
3001 ITGB5
3002 TLR5
3003 GABRB3
3004 GABRG3
3005 TM4SF1
3006 GP2
3007 IL18BP
3008 SLC13A2
3009 WNT7A
3010 PRKCE
3011 DKK3
3012 OR6C4
3013 IBSP

3014 RRM1
3015 EEF2
3016 ADAM3A
3017 GRK2
3018 COL12A1
3019 RTN4RL1
3020 GPR162
3021 DYRK2
3022 LPL
3023 HAO1
3024 ADAMTS17
3025 WEE1
3026 SPSB2
3027 TEX13A
3028 CCR3
3029 ALOX12
3030 PRMT3
3031 TAFA1
3032 NPM1
3033 CXCL11
3034 GCG
3035 TRIM67
3036 PLGLB2
3037 LYN
3038 HTN3
3039 IGKV3-15
3040 ADAMTS15
3041 CFTR
3042 PSCA
3043 JCHAIN
3044 SPSB1
3045 PSMB8
3046 GANC
3047 CCN1
3048 ULBP1
3049 TNFSF13B
3050 H1-1
3051 OR52E8
3052 VAMP8
3053 YARS2
3054 SLC10A5
3055 CILP2
3056 AKR7A2
3057 FGF20
3058 OR6C75
3059 SULT2A1
3060 PLA2G2A
3061 RDH11
3062 H4C14
3063 GPR21
3064 FGA
3065 BAK1P1

3066 GRK5
3067 MTRNR2L4
3068 OR4G2P
3069 MAP3K11
3070 DUSP4
3071 IGKV5-2
3072 TNNC1
3073 FYN
3074 BGN
3075 UGT1A5
3076 DPYD
3077 WNT8B
3078 ABCF1
3079 ABCA3
3080 RPS6KL1
3081 OR9Q1
3082 GSTO2
3083 CFP
3084 UQCRC1
3085 PTK2B
3086 ABCA9
3087 CAMK2D
3088 AQP1
3089 GSTT2B
3090 ADAM21
3091 PVR
3092 DUSP13
3093 DEFB106A
3094 OR13C5
3095 DPP4
3096 PLXNC1
3097 CYP2A7
3098 TPTE
3099 IL10
3100 KCNK7
3101 CCL17
3102 DDX1
3103 SLC25A22
3104 LTB4R
3105 SLC15A3
3106 FMO1
3107 SLC25A28
3108 MST1
3109 WNT3A
3110 EPOR
3111 GAD2
3112 AURKB
3113 SERPINA10
3114 BCKDK
3115 OR2M5
3116 FAM3B
3117 RGS4

3118 IARS2
3119 CAPN12
3120 AGRN
3121 OR7E97P
3122 SLC2A11
3123 HMGCS1
3124 FGR
3125 MAP3K5
3126 OR1S2
3127 SCYL3
3128 UTS2B
3129 GPR50
3130 F7
3131 HTR5A
3132 TFPI2
3133 EPHX1
3134 MAPKAPK5
3135 F12
3136 CYP2C8
3137 LGALS9
3138 CD46
3139 IGLV3-22
3140 SOD1
3141 CAPN13
3142 CXCL3
3143 ADGRE4P
3144 TNC
3145 OR2C1
3146 GRK1
3147 SFRP1
3148 SLCO2A1
3149 TUBG2
3150 RARS2
3151 ALOX15
3152 IL19
3153 NPTN
3154 BRDT
3155 G6PD
3156 OR8K1
3157 CLDN18
3158 PDE6A
3159 ITGAD
3160 H1-0
3161 EREG
3162 KLHL40
3163 KCNS3
3164 SCPEP1
3165 GPR25
3166 CD47
3167 STAT6
3168 NELL1
3169 OR1F12P

3170 ST3GAL3
3171 COL20A1
3172 GPX5
3173 MCAM
3174 TRAC
3175 SHBG
3176 SCNN1A
3177 GPX2
3178 SIK2
3179 PCSK6
3180 FAM234A
3181 PTPRS
3182 PLCB4
3183 HTR7
3184 IGLV7-43
3185 KCNJ16
3186 SCUBE3
3187 CCL14
3188 ANPEP
3189 OR6C1
3190 AGR2
3191 OR2A2
3192 GNRH1
3193 OR5T1
3194 TRPM6
3195 SLC17A3
3196 RNASET2
3197 KLHL9
3198 NEGR1
3199 ADAM10
3200 KHK
3201 NOD1
3202 SPON2
3203 LGI1
3204 PRSS21
3205 PTGIS
3206 LTK
3207 SULT1C2
3208 OR2T11
3209 SMOC2
3210 OR52E4
3211 SLC22A17
3212 CNP
3213 HMGCS2
3214 MAN2A1
3215 TRIM50
3216 ADGRF3
3217 GAA
3218 OR14I1
3219 CRISPLD2
3220 MMEL1
3221 MYDGF

3222 DPP8
3223 SLC2A12
3224 OAS1
3225 PLCD3
3226 OR56B3P
3227 SLC47A2
3228 MC3R
3229 VKORC1
3230 EPHA2
3231 GPR75
3232 COLEC10
3233 APOE
3234 CYB5R4
3235 OR51E2
3236 EMILIN2
3237 TRIM26
3238 SRD5A3
3239 TSG101
3240 PDILT
3241 PAK2
3242 NDUFS3
3243 SPACA3
3244 SLC37A4
3245 KLHL24
3246 PLA2G1B
3247 OTOP1
3248 ERBB2
3249 MFGE8
3250 SLC7A11
3251 SULF1
3252 IGFBPL1
3253 JAK2
3254 CD14
3255 OR51B5
3256 SLC17A2
3257 GPR150
3258 LOXL2
3259 EDDM3A
3260 LCT
3261 CFHR1
3262 KCNK17
3263 HSD3B2
3264 OR2H2
3265 TFPI
3266 CD81
3267 MAPK10
3268 MCOLN1
3269 EDEM2
3270 AMBN
3271 PSMA7
3272 CD44
3273 SLC25A24

3274 NLN
3275 GFM2
3276 CD84
3277 ALDH1L2
3278 HMGB1
3279 FGFBP1
3280 OR11A1
3281 PIK3CA
3282 SLC5A6
3283 WNT16
3284 PEPD
3285 SLC5A10
3286 STK38
3287 TBC1D23
3288 ENHO
3289 ERBB3
3290 PDXDC1
3291 PTPRD
3292 PLCL1
3293 SLC4A7
3294 SLC22A1
3295 KCNE1
3296 STK3
3297 OLFML3
3298 MYO3B
3299 OR10W1
3300 IRAK2
3301 LAIR2
3302 RNF39
3303 CD300LD
3304 KLHL32
3305 EIF2AK3
3306 CDK10
3307 TAPBPL
3308 PTGER3
3309 CYP4F11
3310 PPP3CA
3311 PKN3
3312 ADAM9
3313 ACKR1
3314 ACTN2
3315 CNGA1
3316 MSLN
3317 BHMT2
3318 FIBCD1
3319 CACNA2D3
3320 TAAR6
3321 GHR
3322 ASTL
3323 OR7E38P
3324 SLC16A4
3325 CDK9

3326 AKR7A3
3327 OR4N5
3328 OVGP1
3329 HCN1
3330 TIE1
3331 SOSTDC1
3332 PTPRG
3333 NETO2
3334 MUC16
3335 CORT
3336 TRPV6
3337 PPIL6
3338 OR12D2
3339 IL1RN
3340 PIGR
3341 ACKR2
3342 DHFR2
3343 NCOR2
3344 ROR1
3345 MC4R
3346 SMC3
3347 GABRA2
3348 GRIN1
3349 OR1L6
3350 ENGASE
3351 ATG4B
3352 CELA3A
3353 ATP13A3
3354 MFSD9
3355 ABI3BP
3356 CD248
3357 PRSS30P
3358 OR56A3
3359 SLC17A7
3360 CNTN1
3361 TRAV8-4
3362 GRIN2D
3363 KIT
3364 ERBB4
3365 HPRT1
3366 ULBP2
3367 RNF123
3368 CD1E
3369 MAPK6
3370 PDGFC
3371 OR10A3
3372 ADAMTS8
3373 GPR85
3374 GRM2
3375 DUSP18
3376 CGA
3377 ADORA2A

3378 PMS2P3
3379 COL4A3
3380 STYXL2
3381 GPR82
3382 CDH3
3383 IL36G
3384 PARP1
3385 PECR
3386 LIPM
3387 HCN2
3388 OR4C11
3389 ROCK2
3390 MS4A1
3391 IL1A
3392 INHA
3393 NPR1
3394 COL17A1
3395 OR5D18
3396 C8B
3397 PASK
3398 CYP2S1
3399 LPAR2
3400 SLC29A1
3401 CYP2C19
3402 ICAM1
3403 BRSK2
3404 TMPRSS13
3405 OR10G9
3406 HSD17B3
3407 ABCG5
3408 SLC22A7
3409 GRM8
3410 CEACAM8
3411 FSTL4
3412 IFNGR1
3413 HPD
3414 ADGRG4
3415 TUBG1
3416 C1QTNF8
3417 AURKAIP1
3418 BPIFC
3419 LILRA4
3420 SIGLEC9
3421 APOC4-APOC2
3422 DGCR6
3423 OR5B2
3424 DYRK1B
3425 MGST3
3426 OR13C9
3427 DUSP9
3428 SPACA5
3429 SLC2A1

3430 SLC6A3
3431 SCARA5
3432 CA9
3433 KCNJ13
3434 AIFM3
3435 OR6J1
3436 VCAN
3437 SLC25A6
3438 MTMR4
3439 ETNPPL
3440 HLA-DPB1
3441 DUSP26
3442 SUCNR1
3443 PARP6
3444 ASIC5
3445 ADCY9
3446 GPR17
3447 CTLA4
3448 EARS2
3449 OR2A42
3450 SLC6A12
3451 VSIR
3452 C3
3453 LRP8
3454 CHRNG
3455 PYY
3456 OR6K4P
3457 PRPSAP1
3458 FZD3
3459 GSR
3460 IGLL1
3461 PCSK5
3462 KCNK12
3463 GPR18
3464 PSMB2
3465 AKT3
3466 AREG
3467 IGLC7
3468 GUCY2C
3469 LGALS7
3470 MAP3K10
3471 OR11H6
3472 CSNK2A1
3473 STX1A
3474 OBP2B
3475 C1QA
3476 INPP5A
3477 PLK4
3478 CTRL
3479 LY96
3480 OR5H14
3481 TRAV29DV5

3482 GABRG1
3483 CRELD2
3484 IGKV3D-15
3485 HSP90AB2P
3486 BST2
3487 COMTD1
3488 VPREB1
3489 SLC24A1
3490 FAM3C
3491 TRIM11
3492 WFDC2
3493 CD86
3494 STK38L
3495 CD151
3496 ABCC13
3497 SLC22A10
3498 INSR
3499 OR4S1
3500 MTRNR2L7
3501 WNT1
3502 TUBBP1
3503 CRY2
3504 MLYCD
3505 INSL4
3506 OR10D1P
3507 SLC7A4
3508 DCLK3
3509 MGLL
3510 CPO
3511 ITGB7
3512 PRNP
3513 KCNAB2
3514 OR5AP2
3515 AOC3
3516 SLCO1B1
3517 DDX3X
3518 KIF11
3519 TMX2
3520 GRM5
3521 CNTNAP4
3522 JAG1
3523 TAF15
3524 ADCYAP1
3525 DDR2
3526 OR8D2
3527 CCR5
3528 IGKV1-5
3529 OR4D1
3530 CILK1
3531 NYX
3532 IK
3533 PI16

3534 KCNC2
3535 PTPRT
3536 CGB2
3537 GSK3A
3538 MBNL1
3539 ALDH6A1
3540 EIF4E
3541 TAS1R2
3542 VMO1
3543 PTGER4
3544 PLAC1
3545 UGT2B17
3546 DIO3
3547 PDE1A
3548 MAN2B1
3549 OR6V1
3550 SLC9A2
3551 OR5AK3P
3552 GPR88
3553 OR6K6
3554 FGFBP3
3555 HTR2C
3556 HTRA2
3557 MUC7
3558 SLC7A10
3559 MDM2
3560 OR52N1
3561 SPR
3562 SMOX
3563 DMP1
3564 RORC
3565 PARP11
3566 TMPRSS12
3567 DHRS4L2
3568 APEX1
3569 IGFALS
3570 OR10A2
3571 NOTCH2
3572 TAP2
3573 KEL
3574 SMPD3
3575 DEFA4
3576 AKT2
3577 MSTN
3578 CCL26
3579 CHRM1
3580 COL24A1
3581 H3C4
3582 MADCAM1
3583 PLAA
3584 ENDOG
3585 OR5H1

3586 IL1RAPL2
3587 BMP8B
3588 CYP4B1
3589 RDH5
3590 CTSC
3591 CDK13
3592 OR56A4
3593 ERP29
3594 BTNL9
3595 H3C13
3596 SLC40A1
3597 OR4D6
3598 LMTK2
3599 RBMX
3600 ITIH6
3601 P3H1
3602 CCNB3
3603 SLC22A24
3604 NSDHL
3605 MITF
3606 KCNH6
3607 MYOC
3608 DCLK1
3609 OR13C3
3610 CHRNA7
3611 IGF1R
3612 IL4
3613 OR1L1
3614 COL28A1
3615 BTN2A1
3616 MMP25
3617 SLC6A5
3618 NUP62
3619 OR6B3
3620 SLC25A40
3621 SLURP1
3622 EPHB2
3623 HSD17B2
3624 CCR2
3625 DKK2
3626 YES1
3627 NMUR1
3628 PRKCI
3629 GALR1
3630 GDF7
3631 CASK
3632 BIRC8
3633 GDF10
3634 RNASE9
3635 CSAD
3636 ACE
3637 OR4C46

3638 LYZ
3639 HSPA5
3640 SLC25A42
3641 DDC
3642 MMP12
3643 PLCD4
3644 COMP
3645 MAPK4
3646 IGLV2-18
3647 SCD
3648 ECM1
3649 OR6C2
3650 SERPINA6
3651 ADRB1
3652 PTGFRN
3653 OR7D4
3654 RETN
3655 TNNI3K
3656 POLR1C
3657 PTPRM
3658 GSN
3659 SLC25A38
3660 MIA
3661 OR6K3
3662 VN2R1P
3663 GPC2
3664 VWA2
3665 MATN1
3666 PSMB3
3667 GPR174
3668 FNDC5
3669 SLC22A14
3670 GALNT1
3671 UTP11
3672 ORM2
3673 ULK1
3674 STIM2
3675 MBTPS1
3676 TDGF1
3677 NPBWR1
3678 KCNN2
3679 OR8B8
3680 DNAJC10
3681 ADAMTS7
3682 SLC25A5
3683 TUBB6
3684 CHGB
3685 CCR7
3686 GPR132
3687 IGHV3-23
3688 TMPRSS2
3689 F13A1

3690 SIGLEC6
3691 SYK
3692 KMT2A
3693 TINAG
3694 FAT4
3695 P2RY2
3696 PRKG2
3697 SIGLEC8
3698 IL12RB2
3699 MFSD8
3700 SLC46A1
3701 OR4X1
3702 DEFB127
3703 KLHL28
3704 CD300LG
3705 SLC18A2
3706 AKR1C3
3707 IGHV7-81
3708 PRKD2
3709 IL15
3710 CD9
3711 CCKBR
3712 SGPL1
3713 MT-CO2
3714 PLCB3
3715 BRPF3
3716 MTRNR2L3
3717 OR14L1P
3718 TRIM41
3719 TTK
3720 MMP13
3721 KCNG3
3722 GIPR
3723 C8A
3724 PLG
3725 ATP7A
3726 LMNA
3727 CD79A
3728 LIPI
3729 LPAR4
3730 OR13A1
3731 CHRM5
3732 INS-IGF2
3733 PLEK
3734 ITGA3
3735 KLHL7
3736 WNT11
3737 CA6
3738 ST14
3739 C5AR1
3740 ESM1
3741 NR5A2

3742 ATP13A2
3743 CHAD
3744 DCK
3745 ARSB
3746 ITGA11
3747 EPAS1
3748 OR6W1P
3749 HTR5BP
3750 SMPDL3A
3751 FCGRT
3752 PTBP1
3753 ITFG1
3754 IRAK1
3755 CPT1C
3756 IGLV5-45
3757 PODN
3758 NAXE
3759 TUSC3
3760 SLC22A25
3761 SPNS2
3762 SRM
3763 SLC6A11
3764 OR51F1
3765 GABRP
3766 TGM6
3767 SIRPG
3768 GRIK1
3769 CD53
3770 SLCO1C1
3771 CMKLR1
3772 DEFB103A
3773 GRIK3
3774 LMLN
3775 OR7E125P
3776 GPX4
3777 PLAT
3778 NFKB2
3779 UBA2
3780 SLC6A20
3781 LAMB2
3782 ADAMTS6
3783 KLHL8
3784 MTR
3785 KCNA6
3786 ABCC1
3787 SLC22A4
3788 CCL20
3789 PPIB
3790 PSMB4
3791 ADGRA1
3792 CRHR2
3793 OR4K2

3794 HIPK1
3795 SLC19A3
3796 ERP44
3797 SPAG11A
3798 LACTB
3799 CLCNKB
3800 PTPN6
3801 KCNK5
3802 ALDH1L1
3803 KLHL38
3804 COL26A1
3805 MAP3K15
3806 HMSD
3807 SLC9A9
3808 C8G
3809 DHRS7B
3810 TNFRSF4
3811 ABCB4
3812 NEK7
3813 H1-4
3814 GLRA2
3815 TUBB3
3816 PTPN3
3817 GGT5
3818 ZP3
3819 MYO3A
3820 STYK1
3821 BTLA
3822 PPP5C
3823 ABCA12
3824 OR10AA1P
3825 BRSK1
3826 OR5F1
3827 MUC4
3828 GPR62
3829 OR5W2
3830 DDR1
3831 POR
3832 OR52E6
3833 PPIF
3834 OR10R2
3835 HAPLN3
3836 PSMB1
3837 GADL1
3838 SLC25A2
3839 SPOCK3
3840 CNDP1
3841 SLIT1
3842 CD4
3843 ACP1
3844 PXDN
3845 RBP4

3846 MAST4
3847 HNRNPA1
3848 IGKV2-30
3849 PPIL4
3850 IGHG4
3851 AVPR1A
3852 BCL2A1
3853 STEAP1
3854 TLR2
3855 GC
3856 CDCP1
3857 GPR84
3858 LIMK2
3859 SPHK1
3860 EGLN3
3861 CYP26C1
3862 CCR8
3863 VWA3B
3864 CHRNA3
3865 MUC2
3866 NTRK1
3867 OR56B1
3868 COL18A1
3869 ELOVL6
3870 OR4D9
3871 PDE4C
3872 GSTM5
3873 BOLA3
3874 EPHA7
3875 AKT1S1
3876 MYLK2
3877 METAP1
3878 IFNB1
3879 TLL2
3880 IL22RA2
3881 LILRA6
3882 CXCR1
3883 TRIM75
3884 MRGPRX2
3885 LCK
3886 PROK1
3887 SLCO5A1
3888 GAK
3889 CAPN14
3890 TRPC3
3891 ITGA10
3892 DEAF1
3893 MC5R
3894 PIM3
3895 TPP1
3896 USP2
3897 HUNK

3898 IL12RB1
3899 PRSS23
3900 AGGF1
3901 OR52A5
3902 OR10K1
3903 OLFML1
3904 HNRNPU
3905 S1PR3
3906 INPP5D
3907 MAP4K2
3908 NCAN
3909 CD3E
3910 HTR3A
3911 MAP2K5
3912 OR51L1
3913 TRH
3914 CELA2B
3915 HRH3
3916 OR11H12
3917 SFN
3918 DUOX1
3919 HMCN1
3920 OR2C3
3921 FKBP1C
3922 BGLAP
3923 MMP15
3924 ALOX15P2
3925 LOX
3926 IMPA1
3927 CDC42
3928 PTN
3929 RYR3
3930 LGMN
3931 SULT1B1
3932 UGT2B4
3933 RTN4IP1
3934 CLCN3
3935 OR51A7
3936 CNTNAP1
3937 PYDC1
3938 IL12B
3939 OR6C3
3940 F2R
3941 KLKB1
3942 ICOSLG
3943 CATSPER4
3944 ITGA5
3945 ADGRF4
3946 ANGPT1
3947 CCDC80
3948 MAP2K6
3949 HPSE2

3950 MST1P2
3951 CGB7
3952 CEACAM16
3953 OR10H5
3954 MAP4K1
3955 IL3
3956 PTPRQ
3957 GPR171
3958 CES5A
3959 PHYKPL
3960 ACP6
3961 OR14K1
3962 DST
3963 OR1M1
3964 MAP2K1
3965 CASP5
3966 CDK7
3967 CTSS
3968 CHEK2
3969 BTN3A1
3970 LGR6
3971 OR3A1
3972 IL16
3973 DHCR7
3974 IGLV11-55
3975 TGFBR1
3976 EMC10
3977 ST3GAL1
3978 GPC3
3979 TRIML2
3980 NAT1
3981 AMY1B
3982 GCGR
3983 KRTDAP
3984 TLR8
3985 TMEM123
3986 DDO
3987 BPIFA2
3988 DPEP2
3989 CLCA3P
3990 ABCA4
3991 PTH2R
3992 HTR2B
3993 EPHA4
3994 STK33
3995 FGG
3996 TAC3
3997 CACNG5
3998 OR2A14
3999 SBK2
4000 KEAP1
4001 PPIAL4A

4002 SLC17A8
4003 OR9A2
4004 HDAC10
4005 OPRM1
4006 THSD4
4007 PLA2G6
4008 H3C1
4009 EPO
4010 PDE12
4011 OR4A13P
4012 RHD
4013 RNASE10
4014 SLC9A3
4015 CTSV
4016 OR6T1
4017 FKBP1B
4018 PAK6
4019 CA14
4020 HBB
4021 CHSY1
4022 AFM
4023 PPT1
4024 AOX1
4025 UCHL1
4026 CD226
4027 NRG4
4028 ADGRF1
4029 KIR3DL3
4030 CYP27C1
4031 ADAM17
4032 EFNB3
4033 OR52J3
4034 GP5
4035 BAMBI
4036 MC1R
4037 ACACB
4038 IFNA5
4039 HIPK3
4040 NAPSA
4041 COL13A1
4042 CDKL2
4043 PLCH2
4044 PDIA4
4045 OR10Z1
4046 MMP3
4047 OR8I2
4048 HSD17B1P1
4049 ULK2
4050 TNK2
4051 OR4C45
4052 BPIFA1
4053 CLCN2

4054 ADAMTS2
4055 NXPH3
4056 BAK1
4057 CWC27
4058 HTR1F
4059 MOCS3
4060 GYPA
4061 H3C14
4062 OLFM3
4063 OR2B3
4064 NR1I2
4065 SSC5D
4066 BAX
4067 CTSA
4068 EPHA10
4069 DHRS11
4070 FAM20A
4071 APEH
4072 REG4
4073 DLD
4074 REG1A
4075 DEFB104A
4076 PARP14
4077 CSNK1E
4078 CCN5
4079 QSOX2
4080 RHO
4081 TRPM7
4082 KLHL13
4083 CD1A
4084 GUSB
4085 KLF5
4086 FCGR2B
4087 IGHM
4088 BTNL3
4089 FCRL4
4090 RDH8
4091 DUSP23
4092 TRHR
4093 CCNE1
4094 OAT
4095 EPRS1
4096 OR7E24
4097 SLC13A1
4098 SERPING1
4099 FGFR4
4100 ANOS1
4101 NMT2
4102 PLA2G2F
4103 TGFBR2
4104 MCCC2
4105 TNFSF12

4106 AOC2
4107 OR11H7
4108 TUBAL3
4109 AMBP
4110 PYROXD1
4111 PTP4A3
4112 NOS2
4113 CA1
4114 TNFRSF1B
4115 OR14C36
4116 CSK
4117 FSTL1
4118 FKBP11
4119 GHRH
4120 OR10R3P
4121 CFLAR
4122 KCNT1
4123 RET
4124 GLUL
4125 LINGO1
4126 SLC25A30
4127 LIPC
4128 AGBL2
4129 SERPINA5
4130 KLK4
4131 PTGDR2
4132 F11R
4133 GABRA1
4134 CBR1
4135 GPC1
4136 DBH
4137 TNFAIP2
4138 OR2T33
4139 MTRNR2L10
4140 IGKV1-39
4141 ADCY1
4142 COPA
4143 DNAJB11
4144 OR51D1
4145 KRT19
4146 PKD1L3
4147 PTGDR
4148 MATK
4149 OR4C15
4150 CNTN4
4151 AADAC
4152 HSD11B1L
4153 TPTE2
4154 MAPKAPK3
4155 OR2Z1
4156 FCAR
4157 HCN3

4158 GRM3
4159 PDGFRA
4160 PRRT3
4161 OBSCN
4162 ITGA8
4163 USP8
4164 CASR
4165 PRADC1
4166 OR10C1
4167 ADAMTS1
4168 H4C6
4169 SOAT1
4170 CD276
4171 MMP10
4172 CHST1
4173 CST9L
4174 CGB5
4175 SLK
4176 OPCML
4177 TLR4
4178 PYGM
4179 IL36B
4180 DEFB107B
4181 ACP3
4182 RIPK3
4183 P2RX2
4184 GRIN2C
4185 P2RY14
4186 TXNRD2
4187 KCND3
4188 TRIM49
4189 PDE8B
4190 SRC
4191 TXN
4192 SUPT16H
4193 SLC25A48
4194 ATP6AP2
4195 H4C3
4196 FMOD
4197 ITGAX
4198 CD40LG
4199 TRIM69
4200 STK31
4201 NEK5
4202 IL31RA
4203 TNFRSF14
4204 CSHL1
4205 CGB8
4206 AKR1B10
4207 RHCG
4208 CCS
4209 ADAMTS3

4210 IL2
4211 OR6F1
4212 TRIM65
4213 CDK6
4214 MARCO
4215 BPIFB6
4216 CEACAM5
4217 KLK14
4218 CAD
4219 TLR9
4220 IFNAR1
4221 LPO
4222 LIF
4223 TBCK
4224 IGLV1-51
4225 MTAP
4226 YBX1
4227 OR2AK2
4228 UGT2B11
4229 COL1A2
4230 FKBP9P1
4231 DUSP8
4232 SLC22A31
4233 C8ORF44-SGK3
4234 SPAG11B
4235 DUSP14
4236 TRIM51
4237 PTPN5
4238 PTGS2
4239 CER1
4240 PROM1
4241 STK10
4242 OR10G6
4243 LRRC17
4244 UGT1A4
4245 NT5C1B
4246 PLP1
4247 RELA
4248 ARSA
4249 TSSK6
4250 HDAC5
4251 OR4D11
4252 CPA5
4253 PRKG1
4254 CAMK1
4255 DUSP6
4256 CX3CR1
4257 OR5P2
4258 TUBA3FP
4259 IGLL5
4260 IGLV1-44
4261 PDK4

4262 TESK2
4263 IL1B
4264 OR1L8
4265 ADAM5
4266 NUCB2
4267 BHMT
4268 CYP26B1
4269 ITIH5
4270 LRRC4
4271 CRLF1
4272 SIRT1
4273 PTPRH
4274 NPEPPS
4275 SPINT3
4276 RNASE13
4277 GALK1
4278 L1CAM
4279 F2RL1
4280 LIPK
4281 HLCS
4282 GSTM2
4283 MARK2
4284 SLC9A6
4285 DUSP28
4286 PRR29
4287 HTR6
4288 RSPO3
4289 RBCK1
4290 HCAR2
4291 DEFB108B
4292 RDH13
4293 CNGB1
4294 NEK1
4295 PDE8A
4296 OR1N2
4297 SMOC1
4298 IDO1
4299 AXIN2
4300 CARD17
4301 OR2F1
4302 COL5A2
4303 IGF1
4304 PRKCG
4305 RPS6KA1
4306 SOST
4307 OR52A1
4308 POLB
4309 TPSG1
4310 MAN2A2
4311 PTAR1
4312 OR4B1
4313 IL20

4314 CCL4L1
4315 CCNT1
4316 ALDH1A1
4317 OR5AU1
4318 LYZL4
4319 ADGRG7
4320 PDE2A
4321 NRBP2
4322 CACNG1
4323 CXCL17
4324 CYP2F1
4325 ADGRB2
4326 KSR1
4327 RNASE7
4328 SLC22A16
4329 CYB5D2
4330 HSD3B7
4331 NPR2
4332 TXNDC5
4333 GHRL
4334 ATP13A5
4335 GPX6
4336 CRYZL1
4337 BST1
4338 GGT2P
4339 EFEMP2
4340 LGALS7B
4341 OR1A2
4342 OR6C6
4343 MMP2
4344 KCNH5
4345 IL1R2
4346 MEP1A
4347 GPR20
4348 TNFRSF11B
4349 GLRA1
4350 SLC25A1
4351 SYNJ2
4352 MAP4K5
4353 APH1B
4354 SLC25A18
4355 SV2B
4356 NR2E1
4357 MELTF
4358 TNR
4359 CD180
4360 PDE6G
4361 TRPC1
4362 KCNJ1
4363 PTAFR
4364 OR1J1
4365 SPINT1

4366 CNTNAP3B
4367 OR2D3
4368 INHBA
4369 WNK3
4370 PRAP1
4371 WEE2
4372 PKM
4373 UBE2N
4374 BTN1A1
4375 GZMA
4376 GDF1
4377 HSD17B12
4378 TMPRSS11B
4379 APOO
4380 NECTIN4
4381 PGD
4382 OR10G8
4383 CLU
4384 DRD5P2
4385 PPID
4386 CCN4
4387 SERPINB2
4388 CEACAM6
4389 RANBP10
4390 EXOG
4391 MMP17
4392 PPIAL4C
4393 PIP4K2C
4394 GKN1
4395 KLK2
4396 KCNH8
4397 SLC46A2
4398 IL7R
4399 SLC43A3
4400 TOP2B
4401 LTF
4402 ICOS
4403 OPN1LW
4404 PIGF
4405 INSL5
4406 SCUBE2
4407 SLC2A5
4408 UBA6
4409 C1QB
4410 HPGD
4411 MMP20
4412 TERT
4413 AMD1
4414 RNASEH1
4415 ADCYAP1R1
4416 MAG
4417 SLC6A7

4418 NT5E
4419 LILRA5
4420 CDC7
4421 AZIN1
4422 ENPP2
4423 CD1C
4424 POMC
4425 OR9K2
4426 ADAM11
4427 HS3ST1
4428 LY6D
4429 GGH
4430 PTGER4P2
4431 PNLIPRP3
4432 ABHD16A
4433 SLC1A2
4434 OR10J6P
4435 SFTPD
4436 SLIT3
4437 TK1
4438 MORC3
4439 ESRRA
4440 ACAN
4441 ITGA2
4442 IGHG3
4443 IGSF8
4444 H3-3B
4445 VKORC1L1
4446 CHRNB4
4447 FURIN
4448 CD96
4449 CCL22
4450 F3
4451 ITGA7
4452 TAOK3
4453 LAMC2
4454 LATS2
4455 TNFRSF13B
4456 SLC1A7
4457 ABCA6
4458 TRIM15
4459 PRAME
4460 OR3A2
4461 CYTL1
4462 NOX4
4463 OR2V2
4464 MTRNR2L8
4465 HDGF
4466 ITGB6
4467 PIGK
4468 ASIC3
4469 APOL1

4470 PARP15
4471 CYB5R2
4472 CLCN4
4473 CR1L
4474 OLFM4
4475 ABCD4
4476 OR10Q1
4477 BTN3A3
4478 BMPR2
4479 SPARC
4480 OR52I1
4481 CYP4Z2P
4482 HSPA1A
4483 PRSS57
4484 RS1
4485 OR8S1
4486 PCYOX1
4487 KCNJ9
4488 RETNLB
4489 ALDH4A1
4490 OLFML2A
4491 KITLG
4492 OR5V1
4493 OR2J3
4494 ATM
4495 JMJD7-PLA2G4B
4496 CYP26A1
4497 PPIC
4498 CAMKK1
4499 MUC13
4500 ELANE
4501 GNLY
4502 CFHR3
4503 SMO
4504 DEFB118
4505 KLHL15
4506 IAPP
4507 ZRANB3
4508 PTGER1
4509 LALBA
4510 FOLR1
4511 MEP1B
4512 ANGPT4
4513 PRSS54
4514 IGFL3
4515 HTR7P1
4516 GLO1
4517 LCTL
4518 STK40
4519 FIBIN
4520 CALR
4521 KCNA2

4522 GAPDH
4523 TULP2
4524 ADAM28
4525 UCP3
4526 PLCG2
4527 CD300LF
4528 FGF2
4529 STK39
4530 DMPK
4531 UGGT1
4532 OR2A3P
4533 MORC2
4534 CPA6
4535 TLR10
4536 PCSK9
4537 CALCRL
4538 DUSP1
4539 IL6R
4540 NRG2
4541 WNT10B
4542 PLA2G7
4543 CXCL16
4544 SPX
4545 CD52
4546 C5AR2
4547 PRSS50
4548 CLCA2
4549 GPR31
4550 RXRB
4551 CCL23
4552 SLC22A11
4553 TRIM25
4554 OR52L2P
4555 CLCN7
4556 BMP15
4557 ADORA1
4558 TRPM5
4559 PREPL
4560 ACLY
4561 CETP
4562 INSRR
4563 OR4D2
4564 CXADR
4565 LNPEP
4566 DCXR
4567 UGT2B7
4568 ADGRD2
4569 PRSS55
4570 CDKL3
4571 AKR1C2
4572 TRIML1
4573 OR52L1

4574 KCNQ3
4575 CACNB3
4576 TUBA3D
4577 CHRNA5
4578 NPY1R
4579 COL9A1
4580 SLC6A1
4581 BMPER
4582 SLC12A8
4583 OR1P1
4584 CRHBP
4585 OR1L3
4586 KCNH7
4587 WARS1
4588 OR10J3
4589 OR56A1
4590 NCAM1
4591 OR10J1
4592 ERCC5
4593 BDKRB1
4594 VN1R2
4595 SLC7A2
4596 HABP2
4597 TNF
4598 LIPA
4599 SMG1
4600 CHL1
4601 REN
4602 ITGAM
4603 NRG1
4604 CAPN3
4605 KCNG1
4606 GALNT2
4607 KLK11
4608 EDNRA
4609 BTN2A2
4610 MAT1A
4611 ATR
4612 CCNB2
4613 CST4
4614 SCGB3A1
4615 HGF
4616 PIK3C2B
4617 ABCF2
4618 PLA2G5
4619 SLC5A9
4620 MYH7
4621 QRFP
4622 KCNT2
4623 HHIPL1
4624 CD8B
4625 SLC11A2

4626 SLC6A15
4627 OR7E22P
4628 CHUK
4629 IFNA21
4630 PRSS2
4631 OR51I1
4632 OR4K13
4633 GNS
4634 MAST3
4635 NOTUM
4636 GZMH
4637 IL13
4638 SIDT2
4639 GPER1
4640 ITM2B
4641 SSTR5
4642 GRK3
4643 AATK
4644 KCNG2
4645 CCL3
4646 TNKS2
4647 GPR35
4648 PRSS36
4649 PTPN21
4650 OR4F17
4651 LDHA
4652 ENAM
4653 BDKRB2
4654 SMN2
4655 GPR87
4656 NR2F6
4657 OR52E5
4658 SLC12A5
4659 OR4M2
4660 CD163
4661 GPR149
4662 EDN3
4663 PTPRK
4664 ANGPTL5
4665 ISLR2
4666 SOAT2
4667 KLHL25
4668 TRIM14
4669 NOS1
4670 CLCN1
4671 OR7E129P
4672 SLIT2
4673 CAMK1G
4674 PDE3B
4675 AMELX
4676 CYP2J2
4677 NCR2

4678 LRRC4C
4679 GUSBP12
4680 C1QTNF6
4681 OR7G1
4682 CLSTN1
4683 UTS2R
4684 OR8J1
4685 ABCD3
4686 GNRH2
4687 TGM3
4688 SLC39A6
4689 HSD11B1
4690 GRIK4
4691 LPAR3
4692 IGKV2-40
4693 IGLV3-12
4694 CMA1
4695 MST1R
4696 ADAMTSL3
4697 TYMS
4698 TSSK4
4699 TTBK1
4700 OR2L3
4701 ITIH2
4702 SCARB1
4703 ZP2
4704 AGBL3
4705 EGFL8
4706 ABCB5
4707 SLC17A6
4708 OR1L4
4709 NPNT
4710 MUCL1
4711 REV3L
4712 OPN4
4713 STK26
4714 CP
4715 KLHL18
4716 CTSW
4717 FGF23
4718 ABCG1
4719 TRIB2
4720 TRBV7-1
4721 OR6N1
4722 HSP90AA1
4723 CACNA1F
4724 CXCR3
4725 MFAP2
4726 MORC4
4727 TNFSF10
4728 IRAK3
4729 POSTN

4730 SLC5A3
4731 CAPN2
4732 IL1RAPL1
4733 TYSND1
4734 ARSF
4735 ANGEL2
4736 TGM4
4737 OR11L1
4738 SCN2B
4739 CD8A
4740 OR8A1
4741 PNP
4742 LYZL2
4743 OLFML2B
4744 TUBA4B
4745 OR2B2
4746 ITIH1
4747 PARP12
4748 COL6A3
4749 IL21R
4750 SLC22A15
4751 FZD9
4752 PI4K2B
4753 CCL11
4754 IL9
4755 DSTYK
4756 FAAH
4757 OR11N1P
4758 GPX8
4759 PIK3R1
4760 AZIN2
4761 AADAT
4762 MAP3K8
4763 KBTBD8
4764 OR10AG1
4765 HEXA
4766 OR5M9
4767 STK25
4768 COL25A1
4769 GPR37
4770 ZAR1L
4771 FGB
4772 CD101
4773 ADAM12
4774 TMPRSS11F
4775 ADAM18
4776 CLK3
4777 POLD1
4778 OR51Q1
4779 CAPN1
4780 PRKCSH
4781 CELSR3

4782 ARSI
4783 GSTA3
4784 CXCL9
4785 CTHRC1
4786 SEMA3E
4787 UGT2B15
4788 CAPN10
4789 KCNS1
4790 CCL28
4791 EGFL6
4792 GABRB2
4793 TRIM49B
4794 CD6
4795 SERPINB10
4796 TYRP1
4797 KLHL2
4798 PTGES
4799 DUSP21
4800 LMAN1
4801 PRSS22
4802 CYP3A4
4803 PSMA2
4804 TPSB2
4805 GPR146
4806 IL1RAP
4807 PCK1
4808 CAPN7
4809 LAMP3
4810 COL11A2
4811 ADRA1D
4812 H4C8
4813 OR5P3
4814 OR56A5
4815 PLCB2
4816 FGF12
4817 SFTPC
4818 TP63
4819 MOS
4820 BOK
4821 CHST5
4822 SIRPA
4823 HMMR
4824 RPS6KA2
4825 DEFB103B
4826 CYP4A22
4827 SLC7A14
4828 MAPK3
4829 MGAT4A
4830 IFNA1
4831 XCL1
4832 GP1BA
4833 SERPINB13

4834 PCSK1
4835 GUCA2A
4836 INPP5K
4837 F2RL2
4838 TXNDC12
4839 SVOP
4840 HLA-C
4841 HAO2
4842 ANXA1
4843 OTC
4844 ADAMTSL1
4845 PPIL3
4846 CSF2
4847 RIOK1
4848 MAN1B1
4849 PRSS33
4850 SIGLEC11
4851 TXNDC16
4852 TRIM43
4853 GDF6
4854 MMP27
4855 GSTK1
4856 KLHL5
4857 OR4F29
4858 SFRP5
4859 SLC25A46
4860 NAE1
4861 ADGRG6
4862 COL19A1
4863 OR10AB1P
4864 MPIG6B
4865 OR10T2
4866 PTGER2
4867 WNT2
4868 NRP1
4869 PLCL2
4870 IL37
4871 VAMP1
4872 OR5AS1
4873 ORM1
4874 DUSP5
4875 BIRC3
4876 CHST7
4877 MGAM
4878 CAPN15
4879 LTBP2
4880 IGLV4-3
4881 NUP210
4882 PSAP
4883 P2RY11
4884 PINK1
4885 SCGB1D4

4886 ADAM32
4887 CRTAM
4888 C9ORF47
4889 ADORA2B
4890 VSTM1
4891 CD302
4892 OR2J2
4893 P2RY12
4894 STKLD1
4895 DCT
4896 CREG1
4897 SORD
4898 OR10X1
4899 LRRTM3
4900 SLC9B1
4901 OR7E102P
4902 HEPHL1
4903 ITGAE
4904 EFTUD2
4905 CPN1
4906 NR0B2
4907 ME1
4908 VWA1
4909 CD300A
4910 PNOC
4911 COL8A2
4912 HGFAC
4913 S1PR1
4914 EFNA1
4915 OR51G2
4916 SLC16A6
4917 MAGEA3
4918 P2RX1
4919 NAGLU
4920 FLT3
4921 MUC5B
4922 OR2T4
4923 DNAJA1
4924 DMKN
4925 DEFB1
4926 SUV39H1
4927 OR8D1
4928 TSHB
4929 PTPRR
4930 LATS1
4931 MAP3K19
4932 VRK1
4933 PARP16
4934 LGALS3
4935 VEGFC
4936 MOK
4937 LUM

4938 PARP9
4939 APOA1
4940 ABCD2
4941 OR2Y1
4942 OR2T35
4943 NKTR
4944 SSH3
4945 GANAB
4946 RNASE8
4947 CYP2U1
4948 CASP2
4949 PRG2
4950 C9
4951 COL6A6
4952 MTRNR2L1
4953 TUBB4B
4954 OR5D17P
4955 SERPINA7
4956 PDGFB
4957 AMY2B
4958 OR4K14
4959 FCRL2
4960 JAM3
4961 SLC25A20
4962 TUBA3E
4963 GLRX3
4964 MMP26
4965 FLT3LG
4966 PSPN
4967 CCL16
4968 OR4K1
4969 APOA4
4970 CASP8
4971 TRIM39
4972 SULT2B1
4973 CPA4
4974 TMED9
4975 TSPAN7
4976 HSD17B4
4977 SSH1
4978 CFD
4979 FGL2
4980 CAMKK2
4981 OR4X2
4982 PPWD1
4983 OR13C2
4984 SLCO1A2
4985 MUC17
4986 SLC6A2
4987 HYOU1
4988 CALM2
4989 FGL1

4990 HAMP
4991 PIK3CB
4992 CYP2D6
4993 IL2RB
4994 HSPA1B
4995 A2M
4996 SMPD2
4997 ST3GAL4
4998 TGM5
4999 GPR39
5000 GRID2
5001 GPR141
5002 DRD3
5003 LTBP1
5004 BPIFB4
5005 IFNAR2
5006 CALM1
5007 THNSL2
5008 CLEC4C
5009 PPM1B
5010 SLC7A8
5011 STYXL1
5012 CD3D
5013 TRIM9
5014 PRKACB
5015 GLI2
5016 TRHDE
5017 PRSS38
5018 KCNN1
5019 IL10RB
5020 ADAMTS16
5021 FGFR1
5022 CHRNA9
5023 UCN
5024 SRD5A1
5025 KBTBD7
5026 MAPK12
5027 SCN2A
5028 PRKD1
5029 FGF21
5030 ATP13A4
5031 GABRA4
5032 PDE1C
5033 VCL
5034 GABBR2
5035 HNF4A
5036 GPD2
5037 XPNPEP1
5038 GZMB
5039 OR5I1
5040 GSTT1
5041 SHARPIN

5042 IL32
5043 ADH1B
5044 OR9G1
5045 IGFBP2
5046 PZP
5047 COL15A1
5048 AZGP1
5049 BLM
5050 PLA2G4A
5051 APOA5
5052 OR2AP1
5053 CAPN6
5054 TNK1
5055 GUCY1A2
5056 NLK
5057 KIR2DL1
5058 TNFRSF17
5059 AVP
5060 PON3
5061 OR6B2
5062 RIOK2
5063 ESR1
5064 CALU
5065 PLD1
5066 CTSH
5067 BMP10
5068 KLHL21
5069 EBP
5070 HTT
5071 SLC25A4
5072 TGM7
5073 ADRA2A
5074 PDPK1
5075 SLC6A13
5076 TPT1
5077 TAFA3
5078 PRB3
5079 MFAP5
5080 CHST3
5081 BUB1
5082 FSHB
5083 ARSG
5084 RNASE3
5085 AGTR2
5086 SPINK6
5087 KCNA7
5088 OR13F1
5089 TNFRSF21
5090 OR52B2
5091 TLK2
5092 ODAM
5093 LRRC26

5094 OR12D3
5095 OR4L1
5096 SPN
5097 RPS6KC1
5098 MTM1
5099 MAN2C1
5100 CASP14
5101 CYP4V2
5102 SERPIND1
5103 CD63
5104 TNKS
5105 SLC5A12
5106 DNASE1L2
5107 CYP2A7P1
5108 CEL
5109 GPHA2
5110 OR14J1
5111 RFPL4B
5112 CSF2RA
5113 SPOCK1
5114 TNFAIP6
5115 SLCO2B1
5116 DHRS4
5117 PRIMA1
5118 PCCB
5119 NR2F2
5120 RPS6KA4
5121 SLC12A3
5122 SRPK3
5123 XDH
5124 NMS
5125 TPH2
5126 CISD1
5127 CCR1
5128 IRAK4
5129 ADM
5130 OR5K2
5131 C6ORF120
5132 FEN1
5133 HDAC3
5134 CYP8B1
5135 OR4A5
5136 SLC16A9
5137 CYP27B1
5138 CDK15
5139 HIF1A
5140 PSMB7
5141 OR5J2
5142 SCN4A
5143 SSTR4
5144 OR6C74
5145 MMP28

5146 KCNC1
5147 IL36RN
5148 SLC25A27
5149 RECQL
5150 A2ML1
5151 SLC10A4
5152 CCL4
5153 FRRS1
5154 HTR1A
5155 SGK1
5156 OR1F1
5157 CRYZ
5158 SMPD1
5159 BSPH1
5160 DHRS3
5161 OR2T6
5162 PDYN
5163 TXN2
5164 ENPP3
5165 PLCG1
5166 CSNK1A1L
5167 F10
5168 CBLIF
5169 OR11H4
5170 KLK5
5171 PATE4
5172 PF4
5173 TRPV2
5174 VCAM1
5175 TUBA3C
5176 PELO
5177 ASGR1
5178 KIR3DL1
5179 CEACAM3
5180 CYP3A5
5181 IGLV8-61
5182 OR52B1P
5183 TMPRSS7
5184 OR5K1
5185 SLC9A5
5186 DUSP12
5187 TECTB
5188 OR6M1
5189 KDM1A
5190 PNLIPRP1
5191 SPINK4
5192 SF3B3
5193 FBP1
5194 MTTP
5195 STS
5196 TDP2
5197 KLHL29

5198 PARM1
5199 FCN1
5200 CYB5R1
5201 CHMP1A
5202 WNK2
5203 IL5
5204 BCAT1
5205 PRSS12
5206 MBL2
5207 ABCA7
5208 ACE2
5209 ADAM19
5210 PDE9A
5211 CYP19A1
5212 CST2
5213 NR4A2
5214 CFHR4
5215 AKR1C8
5216 RDH14
5217 CNGA2
5218 SLCO4C1
5219 OR2A4
5220 MAP2K2
5221 CCL13
5222 TCN1
5223 CTSB
5224 CSN1S1
5225 PDK3
5226 RANBP9
5227 CHI3L2
5228 SERPINB11
5229 CST9
5230 SIAE
5231 MUC3A
5232 REL
5233 TRPV1
5234 IL11
5235 SLC2A7
5236 KCNJ3
5237 UMPS
5238 PPAT
5239 TLR3
5240 SULT1A1
5241 OR52R1
5242 LILRA3
5243 CHRM3
5244 KCNF1
5245 SRPX2
5246 OR1Q1
5247 SLC7A6
5248 OR2B11
5249 GUCY2F

5250 PRKCQ
5251 ADRA1B
5252 CILP
5253 BMP5
5254 OTOG
5255 MGST1
5256 OR5J1P
5257 HAGH
5258 TLR7
5259 FKBP4
5260 MUC5AC
5261 CST3
5262 CARM1
5263 KDSR
5264 CAPN8
5265 COL16A1
5266 GDF2
5267 MATN2
5268 CTBS
5269 NPW
5270 AOAH
5271 LTA
5272 WNT7B
5273 EPHB1
5274 OR4K15
5275 VEGFB
5276 SLC25A45
5277 CYP3A7
5278 FOS
5279 NR2E3
5280 HSD17B10
5281 SERPINI2
5282 OR1N1
5283 FSTL5
5284 GSTM1
5285 CTRB2
5286 IFITM1
5287 OR10H4
5288 NFKB1
5289 TULP1
5290 PSMA4
5291 WNT8A
5292 ADRA1A
5293 TINAGL1
5294 SFRP4
5295 KISS1R
5296 SPINK2
5297 CHST2
5298 LYG2
5299 SCGB1D2
5300 GPRC5A
5301 EMILIN3

5302 CALCR
5303 SLC25A29
5304 ATRN
5305 BCL2L1
5306 SBSN
5307 RGR
5308 OR2K2
5309 IGLV5-37
5310 CCL19
5311 ADCY2
5312 KCNA4
5313 IGF2
5314 ALDH18A1
5315 CD164L2
5316 RYBP
5317 CYP2B6
5318 SLC2A6
5319 OR5AC2
5320 CDK16
5321 KCNC4
5322 SERPINA1
5323 TLK1
5324 UGT1A9
5325 HCRTR1
5326 GALNS
5327 SLC29A3
5328 CHRNA4
5329 OR5H6
5330 STK11
5331 OR4C16
5332 FLRT1
5333 PCOLCE2
5334 SERPINC1
5335 KISS1
5336 CHI3L1
5337 NPTX2
5338 IGFBP3
5339 ADAM23
5340 IFNG
5341 AGT
5342 SCN10A
5343 TSLP
5344 F8
5345 ITFG2
5346 TNFSF4
5347 CD300C
5348 C17ORF99
5349 CDK12
5350 CLEC4M
5351 KBTBD2
5352 OR1B1
5353 PLA2G4B

5354 CHIT1
5355 OTOR
5356 SBK1
5357 GPR55
5358 CDK2
5359 GABRR2
5360 FREM1
5361 CYBB
5362 SLC17A9
5363 DKK4
5364 KLK15
5365 KCNJ11
5366 OR5AR1
5367 SFTA2
5368 RAF1
5369 CD28
5370 SLC10A1
5371 DAGLA
5372 HLA-DRB5
5373 PDE7A
5374 PTK2
5375 OR52B4
5376 LECT2
5377 IFNW1
5378 LOXL3
5379 KCNG4
5380 VEGFA
5381 BPIFA3
5382 RIPK2
5383 SLC5A11
5384 MUC1
5385 AKR1E2
5386 ABCC8
5387 CXCL8
5388 FMO4
5389 EMID1
5390 TUBA4A
5391 OVCH2
5392 GYPE
5393 CNR2
5394 KLHL3
5395 TNFRSF6B
5396 TPH1
5397 WFIKKN1
5398 SMCHD1
5399 PRSS37
5400 TMED1
5401 CELSR2
5402 MFSD6L
5403 OR11H1
5404 MR1
5405 NCEH1

5406 ALDOA
5407 SLC44A1
5408 POLR2C
5409 CHST9
5410 FFAR4
5411 SLC9B2
5412 TPMT
5413 OR1D5
5414 CYSLTR2
5415 PAOX
5416 CHST13
5417 AADACL2
5418 SELPLG
5419 KLK7
5420 EPM2A
5421 TSHR
5422 MMP7
5423 PTPN4
5424 LCN6
5425 HTR3B
5426 ZAR1
5427 OSTN
5428 P2RY10
5429 GYPB
5430 OR6C70
5431 ITGAL
5432 FSTL3
5433 OR13C4
5434 GUCY1A1
5435 ANGPTL2
5436 ADCY3
5437 F2
5438 GDNF
5439 HBEGF
5440 EIF2AK2
5441 FLVCR2
5442 FAT1
5443 MRGPRX3
5444 TPO
5445 FKBP10
5446 FLT4
5447 COL11A1
5448 NR1I3
5449 HAS3
5450 OCLN
5451 CLEC3A
5452 GLRB
5453 OR1J2
5454 KLRK1
5455 MID2
5456 LIPE
5457 HTR1D

5458 CDC14A
5459 RYR2
5460 PRLR
5461 OR5AK2
5462 CACNB4
5463 CBLN1
5464 SCG5
5465 TRIM22
5466 PSENEN
5467 PIK3CD
5468 FDPS
5469 LIFR
5470 INHBB
5471 AOC1
5472 KLRC2
5473 MLH3
5474 CAMK2G
5475 GPI
5476 DZANK1
5477 SLC6A10P
5478 FNTA
5479 OR4Q3
5480 SELL
5481 LYG1
5482 C1QTNF5
5483 GDF15
5484 ICMT
5485 EIF4H
5486 IFNA8
5487 FJX1
5488 VCP
5489 GBA2
5490 PPIAL4G
5491 AKR1C4
5492 CACNB2
5493 KIR2DS4
5494 FKBP9
5495 SLC6A4
5496 TRIM6
5497 PIN1
5498 NPFFR1
5499 RCE1
5500 SLC5A5
5501 VDR
5502 TSSK3
5503 EIF2AK1
5504 RAMP3
5505 KCNQ1
5506 GNRHR2
5507 OR5M10
5508 ATP1B1
5509 OR52N2

5510 CD160
5511 OR8U3
5512 OXT
5513 IGFBP7
5514 NEK3
5515 TMEM9B
5516 SLC22A6
5517 SERPINA2
5518 CST1
5519 CAPN9
5520 H1-5
5521 CADM3
5522 SERPINA9
5523 CA5B
5524 ADCY5
5525 SLC25A34
5526 HSPD1
5527 GGTLC3
5528 ADAMTS5
5529 CCL4L2
5530 AFP
5531 TST
5532 PTX3
5533 OR5D16
5534 MLN
5535 HSP90AA5P
5536 IL22
5537 PIP4K2B
5538 MASP2
5539 CACNA1D
5540 PDE6D
5541 OR9G4
5542 CAPNS1
5543 ECE2
5544 CD177
5545 OR2L2
5546 PAK5
5547 RBBP9
5548 KCNH1
5549 TACR3
5550 PRLHR
5551 XIAP
5552 SLCO1B3
5553 MET
5554 TMPRSS4
5555 CSF2RB
5556 GNRHR
5557 NPVF
5558 ENC1
5559 ALOXE3
5560 GZMK
5561 PAK4

5562 COL5A3
5563 TRIM16
5564 DEFB110
5565 VAT1L
5566 OR6C65
5567 PMPCB
5568 CNTFR
5569 AIPL1
5570 LCN1
5571 HRG
5572 LRRN1
5573 KCNA10
5574 MFSD1
5575 TUBB
5576 ABCB11
5577 SRPK1
5578 GGPS1
5579 TAAR5
5580 SERPINB5
5581 IGSF11
5582 PTHLH
5583 LGALS3BP
5584 ANKK1
5585 IGFL1
5586 SCN11A
5587 GABRA3
5588 PKLR
5589 CD40
5590 C4B
5591 CYP11A1
5592 CASP3
5593 RPS6KB1
5594 OR7G2
5595 CREBBP
5596 MECR
5597 PNLIPRP2
5598 MAST2
5599 FKBP3
5600 SCG3
5601 PI3
5602 LYZL6
5603 OR5M13P
5604 RARS1
5605 CPT2
5606 ERO1A
5607 GPR6
5608 SYCN
5609 ADRB2
5610 SPINK1
5611 BMPR1A
5612 RRM2B
5613 NAT2

5614 MANBA
5615 OR4F4
5616 PGGT1B
5617 APOA2
5618 KCNH4
5619 OPRD1
5620 PMS1
5621 IGF2R
5622 PSMA5
5623 TRPV5
5624 EPHX2
5625 CDA
5626 HDAC6
5627 NOS3
5628 PTPRO
5629 SLC10A2
5630 EHMT2
5631 WNT9A
5632 MVD
5633 FCER1A
5634 FKBP2
5635 GLA
5636 TMSB4X
5637 MAMDC2
5638 ARSH
5639 NTRK2
5640 COL23A1
5641 PDE6B
5642 ADCY10P1
5643 PPIL1
5644 TTBK2
5645 PRKAA1
5646 PNLIP
5647 CD247
5648 TNN
5649 TLL1
5650 TRIM46
5651 MSMB
5652 CPA1
5653 NDUFAF6
5654 SDR9C7
5655 CYP11B2
5656 CD83
5657 MPI
5658 FZD4
5659 OR13J1
5660 ULK4
5661 PRKX
5662 MRC1
5663 YAF2
5664 GSK3B
5665 WIF1

5666 GABRB1
5667 NENF
5668 CST7
5669 F11
5670 KDM4E
5671 PTGR1
5672 STK17B
5673 CHGA
5674 KIR2DL3
5675 KCNAB3
5676 SLAMF1
5677 CNOT6
5678 RORB
5679 CRAT
5680 CRB1
5681 SLC2A14
5682 ANTXR1
5683 TP73
5684 EBPL
5685 VIT
5686 CSF1R
5687 GPR160
5688 SPARCL1
5689 SCGB1D1
5690 HFE
5691 ALB
5692 PIK3C2G
5693 TNFRSF9
5694 OR5AZ1P
5695 BDH2
5696 CXCR4
5697 WDR1
5698 SLC49A3
5699 CACNB1
5700 ZP4
5701 TUBD1
5702 SLC46A3
5703 TMPRSS11E
5704 RHAG
5705 P2RX5
5706 PHEX
5707 NMB
5708 BCHE
5709 IL17RA
5710 CACNA1A
5711 PRPS1
5712 EPHA8
5713 ADH1C
5714 PLXNB1
5715 CCL3L1
5716 SCTR
5717 BCL2L2

5718 YARS1
5719 CTF1
5720 SLC22A12
5721 EBI3
5722 SFTPA1
5723 OR4F6
5724 COCH
5725 BCAN
5726 AURKC
5727 OR8B3
5728 LPAR6
5729 CHRNA1
5730 LHB
5731 IGLV3-27
5732 GABRQ
5733 TFF1
5734 KCNQ4
5735 PTPRE
5736 PLSCR4
5737 TRIM49C
5738 PSMB5
5739 CYB5RL
5740 ADGRG5
5741 AEBP1
5742 PLA2G3
5743 PHKG2
5744 LYZL1
5745 C4BPB
5746 NDNF
5747 FAAH2
5748 CDC25B
5749 KCNA1
5750 CACNG7
5751 SLC25A21
5752 HSD17B6
5753 MERTK
5754 SIGMAR1
5755 ZRANB1
5756 PRB2
5757 SLC41A2
5758 RBP3
5759 HLA-B
5760 CYP2E1
5761 CCND2
5762 CES2
5763 ABCC9
5764 HMGCR
5765 OR10J5
5766 KLHL22
5767 SLC4A4
5768 CYP4F12
5769 OR51F2

5770 HTRA3
5771 LRIT2
5772 WNT10A
5773 EPCAM
5774 SEMA3C
5775 PSMA8
5776 OR52I2
5777 GUCY1B3
5778 PAK7
5779 ICK
5780 CTGF
5781 HIST1H1A
5782 PVRL4
5783 HIST1H1E
5784 IL8
5785 ADRBK1
5786 HIST1H1B
5787 HIST1H1C
5788 H1F0
5789 IARS
5790 C8orf44-SGK3
5791 ACPP
5792 KARS
5793 TARS
5794 ADCK4
5795 G6PC
5796 LARS
5797 YARS
5798 ADC
5799 ADCK3
5800 ALPPL2
5801 GUCY1A3
5802 MARS
5803 GSG2
5804 DHFRL1
5805 MGEA5
5806 AREGB
5807 PPIAL4B
5808 WISP2
5809 LPHN1
5810 WISP1
5811 C10orf54
5812 HIST1H4B
5813 HIST1H3B
5814 PVRL2
5815 VIMP
5816 GIF
5817 NOV
5818 HIST1H4H
5819 AMICA1
5820 FAM19A3
5821 HIST1H4D

5822 HIST1H4A
5823 HIST1H3E
5824 HIST1H4C
5825 HIST1H3D
5826 HIST1H4F
5827 HIST1H4I
5828 HIST1H3A
5829 HIST1H4E
5830 C6orf25
5831 C6orf15
5832 DARC
5833 FAM19A5
5834 KAL1
5835 C19orf10
5836 CECR1
5837 ADRBK2
5838 CGB
5839 PVRL1
5840 WISP3
5841 ERBB2IP
5842 LEPRE1
5843 C2orf40
5844 CD97
5845 EMR2
5846 EMR3
5847 IGJ
5848 H3F3B
5849 GGT2
5850 TMEM66
5851 C12orf39
5852 LECT1
5853 EFTUD1
5854 CYR61
5855 C16orf89
5856 H3F3A
5857 FAM19A4
5858 APOA1BP
5859 MFI2
5860 FIGF
5861 PROL1
5862 PVRL3
5863 HIST2H4B
5864 HIST1H3I
5865 GPR97
5866 UTP11L
5867 HIST2H3D
5868 FAM19A1
5869 GPR1
5870 HIST2H4A
5871 C6orf120
5872 TMEM256-PLSCR3
5873 C17orf99

5874	HIST1H3J
5875	HIST1H4J
5876	HIST4H4
5877	HIST1H4K
5878	ERO1L
5879	HIST1H4L
5880	C9orf96
5881	HIST2H3C
5882	HIST1H3H
5883	HIST2H3A
5884	C11orf83
5885	GPR56
5886	NOTCH2NL
5887	C17orf72
5888	SEPP1

Table S5. MR results of the association between blood eQTL and ischemic stroke (IVW or Wald ratio methods).

Druggable gene	Method	Nsnp	beta	se	P value	OR	LL	LR	FDR
CALCRL	Inverse variance weighted	3	0.197494	0.048766	5.12492E-05	1.21834582	1.1072874	1.3405431	0.061775
NMB	Inverse variance weighted	3	-0.15479	0.038337	5.40183E-05	0.85659763	0.794591	0.9234429	0.061775
KCNJ11	Inverse variance weighted	2	0.248616	0.061785	5.72516E-05	1.2822497	1.1360037	1.447323	0.061775
GGT1	Inverse variance weighted	2	-0.18646	0.050479	0.000220959	0.82989501	0.7517179	0.9162024	0.173738
IL6R	Wald ratio	1	0.384598	0.105541	0.000268363	1.46902391	1.1945123	1.8066211	0.173738
MPO	Inverse variance weighted	2	0.118618	0.033271	0.000363487	1.12594022	1.0548601	1.20181	0.174535
HNRNPA1	Wald ratio	1	0.429788	0.122039	0.000428726	1.53693135	1.2099661	1.9522514	0.174535
ATP1A1	Wald ratio	1	0.969136	0.280027	0.000538434	2.6356664	1.5223933	4.5630373	0.174535
ROCK1	Wald ratio	1	0.450264	0.130113	0.000539026	1.56872703	1.2156071	2.0244242	0.174535
NAPSB	Inverse variance weighted	3	-0.06919	0.020139	0.000591265	0.93315135	0.8970358	0.9707209	0.174535
KCNH2	Inverse variance weighted	3	0.219154	0.063805	0.000593107	1.2450227	1.0986654	1.4108769	0.174535
FES	Inverse variance weighted	4	-0.1047	0.031385	0.000850093	0.90059454	0.8468637	0.9577345	0.229313
OLFML1	Wald ratio	1	0.446831	0.138112	0.001215175	1.56335062	1.1925964	2.0493648	0.272515
HLA-DOB	Inverse variance weighted	5	0.07274	0.022493	0.001220852	1.07545105	1.0290692	1.1239234	0.272515
PIP4K2B	Wald ratio	1	-0.39007	0.120979	0.001262814	0.67700813	0.5340902	0.8581697	0.272515
MECR	Inverse variance weighted	2	-0.3379	0.105618	0.001377771	0.71326648	0.5798931	0.8773152	0.27874
PDIK1L	Wald ratio	1	0.788643	0.250932	0.001673075	2.20040746	1.3455682	3.598326	0.318573
TOP2A	Wald ratio	1	-0.76683	0.247697	0.001962674	0.46448434	0.2858425	0.7547713	0.347212
LARS	Wald ratio	1	0.181842	0.059521	0.002249719	1.19942504	1.0673533	1.347839	0.347212
SCD	Inverse variance weighted	3	0.228282	0.074827	0.002282243	1.25644015	1.0850455	1.4549085	0.347212
IGFBP3	Inverse variance weighted	6	-0.10786	0.035438	0.002337322	0.89775327	0.8375133	0.9623261	0.347212
SOD1	Inverse variance weighted	2	-0.23518	0.077343	0.002359795	0.79042753	0.6792451	0.9198089	0.347212
KLHL35	Inverse variance weighted	6	-0.08379	0.027774	0.002555921	0.9196289	0.8709048	0.971079	0.359718
PTPN11	Wald ratio	1	-0.21648	0.073091	0.003058974	0.80535063	0.6978604	0.9293973	0.412579
SLC27A4	Wald ratio	1	0.47932	0.162606	0.003201111	1.61497547	1.174229	2.2211559	0.41448
COL17A1	Wald ratio	1	0.193346	0.066214	0.003500314	1.21330257	1.0656296	1.3814397	0.435789
F7	Wald ratio	1	0.518689	0.1793	0.003817549	1.6798243	1.1820631	2.3871905	0.442929
ULK4	Inverse variance weighted	4	-0.0887	0.030841	0.004024764	0.91511637	0.8614386	0.9721389	0.442929
SLC16A1	Wald ratio	1	-0.23367	0.081344	0.004071749	0.79162671	0.6749612	0.9284576	0.442929
CD8B	Wald ratio	1	0.544558	0.190095	0.00417455	1.72384543	1.187644	2.5021328	0.442929
GPM6A	Inverse variance weighted	5	-0.10116	0.035375	0.004241832	0.90379023	0.8432491	0.9686779	0.442929
SCT	Wald ratio	1	0.665194	0.233806	0.004440161	1.94486727	1.229902	3.0754553	0.44915
RIOK1	Inverse variance weighted	3	-0.11494	0.04171	0.005856512	0.89141846	0.8214424	0.9673555	0.572436
IGLV5-45	Wald ratio	1	-0.16124	0.058731	0.006044264	0.85108928	0.7585467	0.9549221	0.572436
SLC5A6	Inverse variance weighted	4	0.108296	0.039564	0.006195544	1.11437701	1.0312285	1.2042299	0.572436
FCRL2	Inverse variance weighted	2	-0.23352	0.085764	0.006471698	0.79173901	0.6692349	0.9366677	0.572436
MAPKAPK5	Inverse variance weighted	3	-0.12854	0.047271	0.006543137	0.87937745	0.8015628	0.9647463	0.572436
PLCE1	Wald ratio	1	-0.70047	0.259853	0.007025098	0.49635002	0.298261	0.8259991	0.598427
PPP5C	Inverse variance weighted	3	0.065766	0.02464	0.007607361	1.0679765	1.017624	1.1208205	0.625717
OLFML2A	Wald ratio	1	0.39204	0.147187	0.00773206	1.47999649	1.1091051	1.9749161	0.625717
CAMK4	Inverse variance weighted	2	0.131303	0.049795	0.008367647	1.1403138	1.0342791	1.2572192	0.63538
GDF7	Wald ratio	1	0.250623	0.095092	0.008399035	1.28482557	1.0663518	1.5480602	0.63538
CAPZA1	Wald ratio	1	-0.30553	0.115996	0.008440324	0.73673589	0.5869131	0.9248043	0.63538
ABCA5	Wald ratio	1	0.135468	0.051607	0.008664897	1.14507237	1.0349142	1.266956	0.635854
HSD17B13	Inverse variance weighted	3	0.083409	0.031857	0.008839491	1.08698601	1.0211899	1.1570215	0.635854
VRK2	Inverse variance weighted	2	0.094834	0.036524	0.009418779	1.09947683	1.0235189	1.1810718	0.662795
GRIN2D	Wald ratio	1	-0.43877	0.170727	0.010169851	0.64483016	0.4614446	0.901096	0.675317
ACTN4	Inverse variance weighted	3	-0.1623	0.063344	0.01040036	0.85018439	0.7509197	0.962571	0.675317
GNRH1	Wald ratio	1	-0.30871	0.120951	0.010698947	0.73439158	0.5793916	0.9308575	0.675317
SLCO5A1	Wald ratio	1	0.53127	0.209117	0.011068126	1.70109187	1.1290775	2.5629007	0.675317
ADCY6	Wald ratio	1	-0.42105	0.166515	0.011450725	0.6563541	0.4735851	0.9096585	0.675317
CCR9	Wald ratio	1	0.225124	0.089078	0.01149522	1.25247797	1.0518299	1.4914018	0.675317
HSPG2	Inverse variance weighted	3	-0.06816	0.027013	0.011626864	0.93410885	0.8859376	0.9848993	0.675317
HILPDA	Wald ratio	1	0.299848	0.119024	0.011761155	1.34965381	1.0688269	1.704266	0.675317
INPP5J	Wald ratio	1	0.552261	0.219629	0.011919359	1.7371769	1.1295159	2.6717496	0.675317
CHST12	Inverse variance weighted	2	-0.27728	0.110274	0.011922714	0.7578458	0.6105395	0.940693	0.675317
IL18RAP	Inverse variance weighted	8	0.04908	0.019537	0.012000084	1.05030433	1.0108456	1.0913033	0.675317
CSK	Inverse variance weighted	4	0.084034	0.03359	0.0123591	1.08766593	1.0183633	1.1616848	0.675317
PROS1	Wald ratio	1	-0.12557	0.05062	0.013115405	0.88199509	0.7986889	0.9739905	0.675317
SIAE	Wald ratio	1	0.245701	0.099748	0.013769722	1.2785178	1.051476	1.5545841	0.675317

SREBF2	Wald ratio	1	-0.56276	0.229696	0.014285621	0.5696374	0.3631427	0.8935517	0.675317
DDX1	Wald ratio	1	-0.14447	0.059077	0.014463358	0.86547659	0.7708469	0.9717231	0.675317
WNT10A	Wald ratio	1	-0.56365	0.230742	0.014575907	0.56913057	0.3620763	0.8945894	0.675317
SLC2A4	Wald ratio	1	-0.52229	0.21395	0.014639499	0.59316123	0.3899914	0.9021743	0.675317
LPAR2	Inverse variance weighted	7	-0.06489	0.0266	0.014703082	0.93716749	0.8895595	0.9873235	0.675317
ADCK4	Inverse variance weighted	2	-0.23254	0.095774	0.015183764	0.79252193	0.6568817	0.9561707	0.675317
MFSD3	Inverse variance weighted	2	-0.22495	0.092866	0.015421967	0.79855567	0.6656656	0.9579752	0.675317
IRAK3	Inverse variance weighted	3	-0.08154	0.033798	0.015840972	0.92169542	0.8626164	0.9848206	0.675317
LNPEP	Inverse variance weighted	3	-0.07883	0.032812	0.016282498	0.92419563	0.8666301	0.9855849	0.675317
GPR161	Wald ratio	1	0.275977	0.115334	0.016717749	1.3178173	1.0511901	1.6520726	0.675317
PNP	Inverse variance weighted	4	-0.09545	0.039925	0.016816053	0.90896473	0.8405473	0.9829511	0.675317
CD70	Inverse variance weighted	2	-0.24461	0.102379	0.016883458	0.78301233	0.6406519	0.957007	0.675317
ANGPT1	Inverse variance weighted	4	-0.06309	0.026427	0.016971654	0.93885956	0.8914678	0.9887708	0.675317
MLKL	Inverse variance weighted	5	0.088405	0.037038	0.016992106	1.09243098	1.0159364	1.1746852	0.675317
MFSD8	Inverse variance weighted	2	0.404175	0.169424	0.017051936	1.49806644	1.0747676	2.0880822	0.675317
CCL3L3	Wald ratio	1	-0.05415	0.02272	0.017161304	0.94729242	0.9060335	0.9904302	0.675317
CCL4L1	Wald ratio	1	-0.06447	0.027049	0.017161304	0.93756891	0.8891567	0.9886171	0.675317
MAP4K4	Inverse variance weighted	2	0.325098	0.137132	0.017754938	1.38416691	1.057935	1.8109979	0.675317
USP7	Wald ratio	1	0.38106	0.161275	0.01813791	1.46383504	1.0671165	2.0080403	0.675317
TGFBI	Inverse variance weighted	9	-0.05739	0.024338	0.018372953	0.944226	0.9002412	0.9903598	0.675317
SMN2	Inverse variance weighted	5	-0.05218	0.022151	0.018490714	0.94915707	0.9088294	0.9912742	0.675317
GPR65	Inverse variance weighted	4	0.094609	0.040225	0.018673917	1.09922863	1.0158923	1.1894012	0.675317
LIMK2	Wald ratio	1	-0.42164	0.179317	0.018705362	0.65597161	0.4615802	0.9322297	0.675317
ENPP3	Wald ratio	1	-0.14286	0.060768	0.018729214	0.86687789	0.7695405	0.9765272	0.675317
KCNK7	Inverse variance weighted	2	0.132621	0.056426	0.018755935	1.14181727	1.0222701	1.2753446	0.675317
COL6A1	Inverse variance weighted	5	-0.09415	0.040108	0.018899165	0.91014279	0.8413351	0.9845778	0.675317
ESAM	Wald ratio	1	-0.38972	0.166018	0.018902023	0.6772463	0.4891356	0.9377002	0.675317
PRSS22	Inverse variance weighted	2	-0.13132	0.056035	0.019097563	0.87693291	0.7857211	0.9787332	0.675317
SLC4A8	Inverse variance weighted	2	-0.14773	0.063058	0.019141941	0.86266421	0.7623697	0.9761531	0.675317
MYLK	Inverse variance weighted	2	0.255718	0.109243	0.019242142	1.29138788	1.042478	1.5997294	0.675317
MAN2C1	Inverse variance weighted	6	0.06717	0.02883	0.019813496	1.06947706	1.0107201	1.1316497	0.675317
MAP3K1	Inverse variance weighted	4	-0.10988	0.047308	0.020195119	0.89593871	0.8165986	0.9829875	0.675317
CDKL3	Wald ratio	1	-0.42436	0.183036	0.020425651	0.65418998	0.4569834	0.9364991	0.675317
NEK3	Inverse variance weighted	3	-0.14714	0.063602	0.020701475	0.8631759	0.7620086	0.9777746	0.675317
MAP2K3	Wald ratio	1	0.399993	0.17297	0.020750145	1.49181361	1.062869	2.0938685	0.675317
SLC43A3	Inverse variance weighted	2	-0.21821	0.094363	0.020750175	0.80395278	0.6682008	0.9672842	0.675317
SIGLEC5	Inverse variance weighted	7	0.056596	0.024528	0.021033312	1.05822789	1.0085571	1.1103449	0.675317
GSTM4	Inverse variance weighted	4	-0.05052	0.021916	0.021152502	0.95073374	0.9107598	0.9924621	0.675317
NDUFAF6	Inverse variance weighted	3	0.126269	0.054808	0.021230303	1.13458789	1.0190251	1.2632561	0.675317
COMT	Inverse variance weighted	3	-0.08578	0.037257	0.021305511	0.91779161	0.8531598	0.9873196	0.675317
BMPR2	Inverse variance weighted	3	0.083837	0.036416	0.021323766	1.08745127	1.0125396	1.1679053	0.675317
KCNJ2	Inverse variance weighted	2	-0.12814	0.055747	0.021529384	0.87973246	0.7886751	0.9813029	0.675317
RNPEP	Inverse variance weighted	6	-0.05075	0.022117	0.021757418	0.95051648	0.9101921	0.9926274	0.675317
CDK3	Wald ratio	1	-0.37388	0.162941	0.02175757	0.68805919	0.4999511	0.9469435	0.675317
TGFB2	Wald ratio	1	0.424462	0.185397	0.022051845	1.52876742	1.0629876	2.1986425	0.675317
SLC25A40	Wald ratio	1	-0.27603	0.12062	0.022114165	0.7587924	0.5990308	0.9611625	0.675317
CAMK1	Inverse variance weighted	2	-0.16905	0.074212	0.022729078	0.84446525	0.7301487	0.9766799	0.681066
KLHL21	Inverse variance weighted	2	0.238355	0.104838	0.02299256	1.26915949	1.033419	1.5586764	0.681066
WDR5	Inverse variance weighted	2	0.124324	0.054813	0.023321037	1.13238247	1.0170333	1.2608143	0.681066
LOXL4	Wald ratio	1	-0.4114	0.181447	0.023370274	0.66272251	0.4643882	0.9457629	0.681066
CYP1B1	Inverse variance weighted	5	-0.06381	0.02817	0.0235071	0.93818463	0.8877878	0.9914423	0.681066
DAG1	Wald ratio	1	-0.28185	0.124483	0.023564833	0.75438747	0.5910607	0.9628461	0.681066
KDM4C	Inverse variance weighted	3	-0.16173	0.07168	0.024058182	0.85067481	0.7391761	0.9789921	0.689171
PI4K2A	Wald ratio	1	0.241786	0.107574	0.024600492	1.27352203	1.0314241	1.5724457	0.691493
ST6GAL1	Inverse variance weighted	3	0.097955	0.04366	0.024859951	1.10291317	1.0124578	1.20145	0.691493
FOS	Inverse variance weighted	2	0.264643	0.11827	0.02524605	1.30296605	1.0333788	1.6428831	0.691493
ITGA3	Inverse variance weighted	2	0.224547	0.100378	0.025284998	1.25175591	1.0281969	1.5239229	0.691493
SIGMAR1	Inverse variance weighted	4	-0.07297	0.03262	0.025294433	0.92963122	0.8720549	0.9910089	0.691493
SLC16A10	Wald ratio	1	0.374831	0.167851	0.025541331	1.45474608	1.0469098	2.0214599	0.691493
TYSND1	Inverse variance weighted	4	-0.07986	0.0358	0.0257029	0.92324675	0.8606844	0.9903567	0.691493
CAPN1	Inverse variance weighted	4	-0.09107	0.040866	0.025848204	0.91295365	0.8426797	0.989088	0.691493
SLC22A1	Inverse variance weighted	3	-0.09973	0.0449	0.026340437	0.90508151	0.8288346	0.9883426	0.698828
EEF2K	Wald ratio	1	-0.31672	0.142793	0.026554186	0.72853692	0.5506852	0.9638284	0.698828

RYBP	Inverse variance weighted	4	0.134508	0.060774	0.026880458	1.14397356	1.01551	1.288688	0.70171
NR2C1	Wald ratio	1	0.145093	0.065684	0.027177755	1.15614726	1.0164868	1.3149964	0.703795
SIRT1	Inverse variance weighted	5	0.048898	0.022261	0.028051804	1.05011332	1.00528	1.0969461	0.704973
MFSD6	Inverse variance weighted	7	-0.05749	0.026178	0.028077721	0.94412999	0.89691	0.9938359	0.704973
BPNT1	Wald ratio	1	0.293973	0.13391	0.028141189	1.3417482	1.0320123	1.7444445	0.704973
RTN4	Inverse variance weighted	4	-0.09911	0.045227	0.028424414	0.90564392	0.8288189	0.98959	0.704973
SLC2A1	Wald ratio	1	-0.26509	0.121047	0.028524237	0.7671346	0.6051099	0.9725432	0.704973
LARS2	Inverse variance weighted	5	0.084149	0.038425	0.028529968	1.08779061	1.008874	1.1728803	0.704973
SLC22A15	Inverse variance weighted	5	-0.0529	0.024245	0.029110588	0.94847311	0.904456	0.9946325	0.713871
TLR10	Inverse variance weighted	2	0.153339	0.070544	0.029729711	1.16571953	1.0151874	1.3385726	0.722596
EPHB6	Inverse variance weighted	2	0.185796	0.085573	0.029915372	1.2041769	1.0182381	1.4240698	0.722596
GRK6	Inverse variance weighted	3	0.284571	0.131412	0.030350456	1.32919155	1.0273713	1.7196803	0.722596
PRKG1	Inverse variance weighted	3	-0.1351	0.062449	0.030517038	0.87363188	0.772985	0.9873836	0.722596
FGL2	Inverse variance weighted	4	-0.12773	0.059149	0.030816319	0.88009224	0.7837536	0.9882728	0.722596
NR1H2	Wald ratio	1	0.304826	0.141261	0.030936017	1.35638878	1.0283492	1.7890718	0.722596
ERP44	Wald ratio	1	-0.22735	0.10548	0.031135253	0.79664551	0.6478562	0.9796064	0.722596
TMPRSS6	Wald ratio	1	0.337317	0.156612	0.031252239	1.40118338	1.0308241	1.9046071	0.722596
CNDP2	Inverse variance weighted	4	0.062742	0.029219	0.031770251	1.06475182	1.0054874	1.1275093	0.729364
BCAM	Wald ratio	1	0.325961	0.152191	0.0322103	1.38536086	1.0280532	1.8668535	0.734259
GUCY1A3	Inverse variance weighted	2	-0.1943	0.091146	0.033029221	0.82341233	0.6887037	0.9844696	0.747661
SLCO4C1	Inverse variance weighted	3	0.061515	0.028915	0.033381821	1.0634465	1.0048538	1.1254557	0.750396
MAP4K5	Inverse variance weighted	3	-0.20228	0.095403	0.033986848	0.81686852	0.6775527	0.98483	0.751177
SLC16A11	Wald ratio	1	-0.22561	0.106588	0.034288148	0.79802871	0.6475737	0.9834399	0.751177
CTRC	Wald ratio	1	0.067276	0.031786	0.034301893	1.06959093	1.0049871	1.1383477	0.751177
CCR10	Wald ratio	1	-0.39779	0.187993	0.034344808	0.67180111	0.4647484	0.971099	0.751177
PDF	Wald ratio	1	0.119026	0.056325	0.034583008	1.12639913	1.0086667	1.2578734	0.75131
TTK	Wald ratio	1	0.402392	0.190931	0.035071934	1.49539782	1.0285679	2.174105	0.754469
PTK2	Inverse variance weighted	4	-0.11729	0.055721	0.035294329	0.88932618	0.7973155	0.9919549	0.754469
PKN3	Inverse variance weighted	3	0.065653	0.031234	0.035554758	1.06785618	1.0044445	1.1352711	0.754469
KIR2DL3	Wald ratio	1	-0.32728	0.155791	0.035660705	0.72088107	0.5311925	0.9783073	0.754469
NME8	Inverse variance weighted	3	0.062031	0.02964	0.036367656	1.06399577	1.0039438	1.1276398	0.762019
TBK1	Inverse variance weighted	3	0.107137	0.051226	0.03648842	1.11308691	1.006756	1.2306481	0.762019
STK4	Wald ratio	1	0.125191	0.06011	0.03727796	1.13336524	1.0074032	1.2750772	0.773518
BCL2A1	Inverse variance weighted	2	-0.08167	0.039285	0.037615606	0.92157252	0.8532762	0.9953353	0.775552
HRH4	Inverse variance weighted	2	-0.35513	0.171623	0.038524032	0.70108413	0.5008205	0.9814274	0.783501
PTGDR	Inverse variance weighted	6	0.061835	0.029931	0.038834456	1.0637869	1.0031758	1.1280601	0.783501
ABCB9	Inverse variance weighted	3	-0.07913	0.038313	0.038885226	0.92391825	0.857079	0.99597	0.783501
SDC2	Inverse variance weighted	3	0.170776	0.08272	0.038969308	1.18622518	1.0086826	1.3950178	0.783501
IGLV3-12	Wald ratio	1	-0.26769	0.130251	0.03985827	0.76514277	0.5927492	0.9876748	0.786876
PSMB8	Inverse variance weighted	3	0.071432	0.034783	0.040009211	1.0740448	1.0032628	1.1498205	0.786876
TTN	Inverse variance weighted	2	-0.32146	0.156904	0.040482051	0.72508619	0.5331263	0.9861641	0.786876
HSP90B1	Wald ratio	1	0.223268	0.109003	0.040532977	1.25015523	1.0096688	1.5479216	0.786876
EMILIN2	Inverse variance weighted	3	0.103134	0.050364	0.040580578	1.10864046	1.0044312	1.2236613	0.786876
WDR1	Inverse variance weighted	3	-0.10921	0.05338	0.040765469	0.89654074	0.8074797	0.9954247	0.786876
ZC3HAV1	Inverse variance weighted	2	-0.11788	0.057698	0.041049337	0.88880335	0.793765	0.9952207	0.786876
BAD	Wald ratio	1	-0.29188	0.143189	0.041503797	0.74685462	0.5640938	0.9888281	0.786876
COLQ	Inverse variance weighted	5	0.07041	0.034605	0.04188031	1.07294812	1.0025886	1.1482454	0.786876
SPARC	Inverse variance weighted	2	0.126841	0.062406	0.042102064	1.13523619	1.0045353	1.2829427	0.786876
TRPC1	Wald ratio	1	-0.18836	0.0928	0.042386741	0.82831902	0.6905649	0.9935524	0.786876
TRGV3	Inverse variance weighted	4	-0.02893	0.014269	0.04258598	0.97148124	0.9446886	0.9990338	0.786876
PTPN22	Inverse variance weighted	3	0.107288	0.052924	0.042642686	1.11325448	1.0035625	1.2349361	0.786876
CD40	Inverse variance weighted	3	-0.08404	0.041518	0.042957352	0.91939706	0.8475444	0.9973412	0.786876
CHSY1	Inverse variance weighted	5	-0.05035	0.024906	0.043206134	0.95089389	0.90559	0.9984642	0.786876
KLHL28	Wald ratio	1	0.234564	0.11616	0.043454666	1.26435689	1.0069136	1.5876221	0.786876
ADAM12	Inverse variance weighted	3	0.179586	0.089308	0.044339176	1.19672177	1.0045534	1.4256514	0.786876
CALU	Inverse variance weighted	4	0.095788	0.04766	0.044449681	1.1005253	1.0023775	1.2082833	0.786876
P2RX6	Inverse variance weighted	2	-0.08638	0.043002	0.044566765	0.91724609	0.8431049	0.9979071	0.786876
SLC7A4	Wald ratio	1	-0.27187	0.135405	0.04466322	0.76195475	0.5843459	0.9935469	0.786876
FARS2	Inverse variance weighted	2	0.053587	0.026705	0.044790355	1.05504854	1.0012457	1.1117425	0.786876
UGT2B11	Inverse variance weighted	2	-0.0567	0.028279	0.044973694	0.94488053	0.8939336	0.9987311	0.786876
MLYCD	Inverse variance weighted	2	0.173558	0.086577	0.044998213	1.18952943	1.003875	1.4095184	0.786876
CD96	Inverse variance weighted	4	0.064067	0.031987	0.045187844	1.066164	1.0013732	1.1351468	0.786876
CACNB3	Inverse variance weighted	5	0.068982	0.034446	0.0452144	1.07141739	1.0014703	1.1462498	0.786876

PDE8A	Inverse variance weighted	2	-0.15376	0.077056	0.045992744	0.85747639	0.7372775	0.9972713	0.79339
RHD	Inverse variance weighted	6	0.053893	0.027088	0.046639661	1.05537189	1.000801	1.1129184	0.79339
IL1RN	Inverse variance weighted	3	-0.15915	0.080045	0.046778591	0.85286515	0.7290285	0.9977373	0.79339
ABCC6	Inverse variance weighted	2	-0.12165	0.061244	0.047001724	0.88545929	0.7853013	0.9983915	0.79339
MAPK3	Inverse variance weighted	5	0.044858	0.022597	0.047135488	1.04587895	1.0005669	1.093243	0.79339
PIP5K1C	Inverse variance weighted	2	0.102671	0.051808	0.047506472	1.1081268	1.0011278	1.2265617	0.79339
FSTL4	Inverse variance weighted	2	0.203653	0.102803	0.047591985	1.22587256	1.0021606	1.4995237	0.79339
PI4K2B	Inverse variance weighted	2	0.198152	0.100092	0.047737882	1.21914749	1.0019732	1.4833936	0.79339
ADAMTS17	Inverse variance weighted	5	0.08226	0.041587	0.047928828	1.08573766	1.0007488	1.1779442	0.79339
SLC5A2	Wald ratio	1	0.394313	0.199809	0.048444165	1.48336436	1.0026913	2.1944638	0.79339
FKBP7	Wald ratio	1	-0.42431	0.215063	0.048498642	0.65421884	0.4291981	0.9972139	0.79339
DNMT3B	Wald ratio	1	0.268603	0.136368	0.04887311	1.30813535	1.0013232	1.7089567	0.79339
MLH1	Inverse variance weighted	2	-0.05816	0.029529	0.048878585	0.94349654	0.8904394	0.9997151	0.79339
HSD17B4	Inverse variance weighted	4	0.079635	0.040468	0.049085967	1.08289183	1.0003175	1.1722825	0.79339
LRPAP1	Inverse variance weighted	3	-0.08002	0.040718	0.049381351	0.923096	0.8522896	0.9997849	0.79339
MGLL	Inverse variance weighted	3	0.163623	0.083329	0.049579276	1.17776974	1.000298	1.3867283	0.79339
PRSS35	Inverse variance weighted	2	-0.27786	0.141769	0.049998009	0.75739929	0.5736521	1.0000027	0.79339
RFPL2	Inverse variance weighted	2	-0.11865	0.060535	0.050000482	0.88812268	0.78876	1.0000024	0.79339
ENPEP	Wald ratio	1	-0.51021	0.260755	0.050385256	0.60036717	0.3601287	1.0008664	0.795595
TNFSF13	Inverse variance weighted	4	-0.06002	0.030813	0.051426571	0.94174499	0.8865531	1.0003728	0.795776
NAPSA	Wald ratio	1	-0.09408	0.048382	0.051841879	0.91021299	0.8278644	1.0007529	0.795776
EPHB2	Inverse variance weighted	3	-0.08112	0.041762	0.052094404	0.92208553	0.8496153	1.0007373	0.795776
KLHL29	Inverse variance weighted	2	-0.19533	0.100578	0.052124331	0.8225605	0.6753891	1.0018014	0.795776
CDK9	Inverse variance weighted	2	-0.18351	0.094517	0.052189204	0.83234287	0.6915889	1.0017435	0.795776
MAPK1	Inverse variance weighted	3	0.159847	0.08237	0.052305401	1.1733317	0.9984043	1.3789077	0.795776
LACTB	Inverse variance weighted	2	-0.09774	0.050369	0.052310152	0.90688037	0.8216276	1.000979	0.795776
RNGTT	Inverse variance weighted	6	-0.05781	0.029796	0.052363396	0.94383051	0.8902889	1.0005921	0.795776
HCN2	Wald ratio	1	0.346012	0.178812	0.052982328	1.4134201	0.9955512	2.0066836	0.80142
KBTBD6	Wald ratio	1	-0.51563	0.267245	0.053679764	0.59712682	0.3536576	1.0082081	0.803032
CD58	Inverse variance weighted	2	0.276782	0.143532	0.053810529	1.31887875	0.9954687	1.7473589	0.803032
NEK8	Wald ratio	1	-0.44483	0.230701	0.053833139	0.64093233	0.4077894	1.0073686	0.803032
TBXAS1	Inverse variance weighted	4	-0.05832	0.030308	0.054343639	0.94335262	0.8889461	1.001089	0.805315
TAP2	Inverse variance weighted	3	-0.03408	0.017734	0.054644856	0.96649573	0.9334796	1.0006796	0.805315
BIRC5	Inverse variance weighted	2	0.104848	0.054631	0.054958427	1.11054177	0.9977738	1.2360547	0.805315
PLCG1	Inverse variance weighted	2	-0.27139	0.14142	0.05498137	0.76232046	0.5777746	1.0058117	0.805315
CA14	Wald ratio	1	0.115124	0.060098	0.055418015	1.12201221	0.9973343	1.2622763	0.806833
HS3ST1	Inverse variance weighted	3	-0.11882	0.06207	0.055592145	0.88797167	0.7862563	1.0028456	0.806833
PPOX	Inverse variance weighted	4	0.094264	0.049378	0.056257269	1.09884962	0.9974867	1.2105128	0.806833
KBTBD7	Wald ratio	1	0.103731	0.054372	0.056416887	1.10930156	0.9971659	1.2340474	0.806833
HDC	Wald ratio	1	0.469645	0.246222	0.056467851	1.59942607	0.9871341	2.5915059	0.806833
CBR4	Inverse variance weighted	4	-0.13156	0.069003	0.056580472	0.87673028	0.7658256	1.0036958	0.806833
CCR6	Inverse variance weighted	2	-0.10857	0.057026	0.056935147	0.89711983	0.8022492	1.0032095	0.808329
CD69	Wald ratio	1	-0.40361	0.21253	0.057553621	0.66790281	0.4403559	1.013031	0.813542
APEH	Inverse variance weighted	4	-0.0719	0.037981	0.0583488	0.93062393	0.8638621	1.0025453	0.813833
GPR84	Inverse variance weighted	3	-0.08368	0.044256	0.058644073	0.91972466	0.8433088	1.0030649	0.813833
MATN2	Inverse variance weighted	5	0.074523	0.039536	0.059439806	1.07737021	0.9970363	1.1641769	0.813833
TNFRSF8	Wald ratio	1	0.271831	0.144323	0.059633297	1.31236498	0.9890194	1.7414238	0.813833
CNTNAP1	Inverse variance weighted	3	-0.1758	0.093452	0.059949909	0.83878732	0.6983996	1.0073948	0.813833
SLC17A3	Inverse variance weighted	4	0.074637	0.039699	0.060098262	1.07749332	0.996832	1.1646816	0.813833
ALDH3A2	Inverse variance weighted	2	0.112355	0.059908	0.060729272	1.11891022	0.9949483	1.2583168	0.813833
NPPA	Wald ratio	1	-0.08271	0.044111	0.060792724	0.92062032	0.8443699	1.0037564	0.813833
MPI	Inverse variance weighted	4	-0.07632	0.040733	0.060981631	0.92652105	0.8554264	1.0035244	0.813833
ABCG2	Inverse variance weighted	3	-0.06497	0.034716	0.06127685	0.93709463	0.8754523	1.0030774	0.813833
CD47	Inverse variance weighted	4	0.057548	0.030754	0.06131091	1.05923613	0.9972742	1.1250478	0.813833
TSSK6	Wald ratio	1	0.125576	0.067109	0.061315685	1.13380121	0.9940592	1.2931877	0.813833
LTK	Wald ratio	1	-0.44658	0.238815	0.061483818	0.63981013	0.4006524	1.021726	0.813833
FKBP4	Wald ratio	1	0.278713	0.149051	0.061495109	1.32142748	0.9866631	1.7697739	0.813833
CCS	Inverse variance weighted	4	0.058319	0.031204	0.061631265	1.06005284	0.9971626	1.1269096	0.813833
CCR1	Inverse variance weighted	4	0.060734	0.032519	0.061809033	1.06261646	0.9970018	1.1325493	0.813833
FKBP9	Inverse variance weighted	3	-0.07322	0.039212	0.061848329	0.92939231	0.8606387	1.0036384	0.813833
CLEC10A	Inverse variance weighted	5	0.074145	0.039829	0.06266131	1.07696309	0.996088	1.1644047	0.820411
UCN	Wald ratio	1	0.523534	0.281437	0.062855106	1.68798196	0.9723075	2.9304342	0.820411
TMX1	Inverse variance weighted	3	0.083591	0.045044	0.063485843	1.08718406	0.9953163	1.1875313	0.824609

PRKAA1	Inverse variance weighted	2	0.1992	0.107472	0.063810083	1.22042571	0.98862	1.5065839	0.824609
ADAM17	Inverse variance weighted	2	-0.07335	0.039591	0.06394092	0.92927963	0.8598962	1.0042614	0.824609
ASPRV1	Inverse variance weighted	3	-0.07481	0.040457	0.064435593	0.92791843	0.85718	1.0044946	0.827691
SLC25A45	Wald ratio	1	0.137096	0.074297	0.065002911	1.14693837	0.9915098	1.3267319	0.828893
SEMA6B	Wald ratio	1	0.290606	0.157617	0.065219583	1.33723732	0.9818439	1.8212708	0.828893
LILRB2	Inverse variance weighted	3	0.082887	0.044969	0.065297426	1.08641878	0.9947622	1.1865206	0.828893
TUB	Inverse variance weighted	2	-0.20061	0.109132	0.066032852	0.81823384	0.6606662	1.013381	0.832473
LTBP3	Inverse variance weighted	5	0.086623	0.047134	0.066093774	1.09048496	0.9942562	1.1960272	0.832473
SOAT1	Inverse variance weighted	7	-0.04957	0.027004	0.06641407	0.95163971	0.9025815	1.0033644	0.833265
CNOT6	Inverse variance weighted	5	-0.0645	0.0352	0.06690447	0.93753853	0.8750368	1.0045046	0.835232
ATP2A3	Inverse variance weighted	4	-0.11859	0.064765	0.067086915	0.88817118	0.7822895	1.0083837	0.835232
GPX4	Inverse variance weighted	5	-0.04739	0.025945	0.067748202	0.95371301	0.9064277	1.003465	0.835626
PTPN13	Inverse variance weighted	2	-0.18878	0.103373	0.067821382	0.82796919	0.6761158	1.0139283	0.835626
NODAL	Wald ratio	1	0.340664	0.186869	0.068302051	1.40588142	0.9747254	2.0277531	0.835626
CFD	Inverse variance weighted	6	-0.03642	0.020008	0.06871993	0.9642349	0.927153	1.0027999	0.835626
TLN1	Wald ratio	1	0.418114	0.229733	0.068759005	1.51909326	0.9683491	2.3830706	0.835626
KCNC3	Wald ratio	1	0.205408	0.112873	0.068786448	1.2280266	0.984302	1.5321003	0.835626
CD53	Inverse variance weighted	2	0.083878	0.046152	0.06915308	1.08749647	0.9934411	1.1904566	0.835626
HSD17B11	Inverse variance weighted	4	0.06319	0.034773	0.069188654	1.06522879	0.9950462	1.1403615	0.835626
KLK14	Wald ratio	1	-0.32989	0.181705	0.069441889	0.71900162	0.5035695	1.0265978	0.835626
QDPR	Inverse variance weighted	5	-0.03673	0.020283	0.070143291	0.96393388	0.9263643	1.0030271	0.83804
CEACAM1	Wald ratio	1	0.493234	0.272374	0.070160249	1.63760441	0.9601961	2.7929173	0.83804
METAP1	Inverse variance weighted	2	0.218245	0.120902	0.0710535	1.2438913	0.9814507	1.5765086	0.84314
CD101	Inverse variance weighted	7	-0.05653	0.031351	0.071341537	0.945034	0.8887125	1.0049248	0.84314
VEGFA	Inverse variance weighted	2	-0.32648	0.181061	0.071368677	0.72146222	0.5059312	1.0288113	0.84314
PHOSPHO1	Inverse variance weighted	3	-0.10285	0.057154	0.071942482	0.90226495	0.8066476	1.0092164	0.845257
RHCE	Wald ratio	1	-0.21492	0.119519	0.072150842	0.80660959	0.6381554	1.0195307	0.845257
APH1A	Wald ratio	1	0.214595	0.11956	0.072674129	1.2393603	0.9804508	1.5666405	0.845257
RELA	Wald ratio	1	-0.35679	0.19891	0.072853081	0.6999167	0.4739485	1.0336216	0.845257
PVRL2	Inverse variance weighted	5	0.022719	0.012666	0.072868106	1.02297935	0.9978952	1.048694	0.845257
HIPK2	Inverse variance weighted	3	-0.24162	0.134821	0.073114597	0.78535816	0.6029835	1.0228926	0.845257
LIPE	Wald ratio	1	0.235841	0.132048	0.074095016	1.26597328	0.977289	1.6399329	0.853543
ALOX5AP	Inverse variance weighted	4	-0.06118	0.034291	0.074400168	0.94065415	0.8795106	1.0060484	0.854019
PROCA1	Inverse variance weighted	3	0.073586	0.041321	0.074940727	1.07636157	0.9926236	1.1671637	0.857184
CCND2	Inverse variance weighted	3	0.143851	0.080979	0.075668477	1.15471206	0.9852415	1.3533332	0.860413
CAPN13	Inverse variance weighted	2	0.229618	0.129299	0.075754645	1.25811952	0.9764731	1.6210019	0.860413
AIP	Wald ratio	1	-0.14356	0.081003	0.076348549	0.86626957	0.7390983	1.0153222	0.864127
KDM1A	Wald ratio	1	-0.12055	0.068461	0.078260482	0.88643267	0.7751236	1.0137259	0.870228
CXCR2	Inverse variance weighted	3	-0.06556	0.037241	0.07833386	0.9365426	0.8706176	1.0074596	0.870228
ZRANB1	Inverse variance weighted	3	0.043576	0.0248	0.078898493	1.04453981	0.994981	1.096567	0.870228
KAT2B	Inverse variance weighted	3	0.073962	0.042103	0.078971228	1.07676632	0.9914764	1.1693932	0.870228
GIPR	Wald ratio	1	-0.3023	0.172338	0.079413655	0.73911734	0.5272492	1.0361219	0.870228
CASP4	Inverse variance weighted	4	0.120483	0.068717	0.079546414	1.12804208	0.9858982	1.2906799	0.870228
FGFBP2	Inverse variance weighted	4	-0.06071	0.034625	0.079552343	0.94109855	0.8793501	1.007183	0.870228
UGT2B17	Inverse variance weighted	3	0.081151	0.046363	0.08005946	1.08453442	0.9903267	1.1877039	0.870228
KLHL22	Wald ratio	1	-0.19432	0.111038	0.080118314	0.82339671	0.6623558	1.0235921	0.870228
NR1I2	Wald ratio	1	0.365655	0.208946	0.080118314	1.4414578	0.9570701	2.1710014	0.870228
CD109	Inverse variance weighted	3	0.124973	0.071422	0.080154714	1.13311765	0.9850985	1.3033778	0.870228
LSS	Inverse variance weighted	4	0.049047	0.028048	0.080349692	1.0502693	0.9940899	1.1096236	0.870228
FNTB	Inverse variance weighted	7	0.058283	0.033373	0.080735775	1.06001489	0.9928981	1.1316686	0.870228
LAMC1	Inverse variance weighted	4	-0.03732	0.021379	0.080830242	0.9633634	0.9238306	1.0045879	0.870228
ATRAID	Inverse variance weighted	4	-0.0806	0.046179	0.080920179	0.92256339	0.8427292	1.0099606	0.870228
MAPK11	Wald ratio	1	0.279906	0.161158	0.082415045	1.32300523	0.9646754	1.8144372	0.881327
RDH10	Inverse variance weighted	2	0.170903	0.098458	0.082599444	1.18637558	0.9781675	1.4389019	0.881327
LEFTY1	Inverse variance weighted	2	0.255961	0.147542	0.08276909	1.29170283	0.9673253	1.7248553	0.881327
DKK2	Wald ratio	1	-0.20894	0.120777	0.083630275	0.81144096	0.6403977	1.0281681	0.881413
NOD1	Inverse variance weighted	3	0.121134	0.070073	0.083866291	1.12877632	0.9839214	1.2949571	0.881413
KCNK6	Inverse variance weighted	4	-0.07839	0.04538	0.084077172	0.92459951	0.845912	1.0106066	0.881413
BRAF	Wald ratio	1	-0.23665	0.137007	0.084118695	0.78926815	0.6033947	1.0323991	0.881413
THBS3	Inverse variance weighted	10	-0.05647	0.032698	0.084138556	0.94509064	0.886422	1.0076423	0.881413
STK35	Wald ratio	1	0.160854	0.093369	0.084929714	1.1745132	0.9780938	1.4103773	0.883084
PLA2G4A	Inverse variance weighted	2	0.107648	0.062501	0.085008978	1.11365566	0.9852554	1.2587893	0.883084
C1R	Wald ratio	1	-0.49968	0.290218	0.085116785	0.60672535	0.3435215	1.0715943	0.883084

MAPT	Wald ratio	1	0.350837	0.204011	0.085487539	1.42025628	0.9521578	2.1184807	0.883084
MAP3K4	Inverse variance weighted	2	0.254942	0.148331	0.085662171	1.29038729	0.964846	1.7257669	0.883084
MTMR2	Wald ratio	1	-0.18618	0.108518	0.086219478	0.83012244	0.6710736	1.0268669	0.883449
CRYZL1	Wald ratio	1	0.306419	0.178612	0.086243419	1.35855122	0.9572787	1.9280294	0.883449
ODC1	Wald ratio	1	0.161716	0.094476	0.086949809	1.17552604	0.9768157	1.4146594	0.887875
MOXD1	Inverse variance weighted	2	0.197122	0.115542	0.087995471	1.217893	0.9710864	1.5274267	0.892574
SRPK2	Wald ratio	1	-0.13837	0.081116	0.088029986	0.87077238	0.7427748	1.020827	0.892574
PIK3CA	Inverse variance weighted	2	-0.15949	0.093553	0.088237172	0.85258175	0.7097448	1.0241648	0.892574
PTP4A3	Inverse variance weighted	5	0.081583	0.048001	0.089206036	1.08500301	0.9875783	1.1920386	0.899564
FAT4	Wald ratio	1	0.27926	0.164616	0.089804747	1.32215112	0.9575396	1.8255992	0.901777
COL4A2	Wald ratio	1	-0.32688	0.192895	0.090149281	0.72116832	0.4941294	1.0525254	0.901777
TRIM7	Inverse variance weighted	2	0.079245	0.046781	0.090271127	1.08246978	0.987632	1.1864144	0.901777
AKT1	Inverse variance weighted	3	-0.05967	0.035257	0.090539861	0.94207171	0.8791701	1.0094737	0.901777
PTGER2	Inverse variance weighted	3	0.064754	0.03832	0.091068037	1.06689608	0.9896989	1.1501147	0.901908
SLC25A29	Inverse variance weighted	5	0.05543	0.032808	0.091115665	1.05699477	0.9911662	1.1271954	0.901908
ABCB4	Inverse variance weighted	3	0.086764	0.051431	0.091604657	1.09063929	0.9860571	1.2063136	0.901908
MST1R	Wald ratio	1	-0.40273	0.238773	0.091667465	0.66849249	0.418648	1.0674414	0.901908
PTPMT1	Inverse variance weighted	2	-0.15158	0.090398	0.093570444	0.85934523	0.7198124	1.0259259	0.915807
F10	Wald ratio	1	0.313153	0.186793	0.093646053	1.36773104	0.9484167	1.9724329	0.915807
PTP4A1	Inverse variance weighted	3	-0.0873	0.052152	0.09415706	0.9164066	0.8273622	1.0150343	0.916211
APLP1	Wald ratio	1	-0.45375	0.271681	0.094891887	0.63524469	0.3729765	1.0819335	0.916211
DLK1	Wald ratio	1	0.119301	0.071442	0.094938574	1.1267086	0.9794883	1.2960566	0.916211
PVRL1	Wald ratio	1	-0.33115	0.198338	0.094990046	0.71809494	0.486803	1.0592794	0.916211
PRPF4	Inverse variance weighted	2	0.122775	0.073663	0.095573187	1.13062967	0.9786266	1.3062422	0.916211
CYP11A1	Wald ratio	1	0.284303	0.170582	0.095580705	1.32883574	0.9511939	1.8564085	0.916211
RBM5	Wald ratio	1	-0.40673	0.244506	0.096220084	0.66582691	0.4123197	1.0751985	0.916211
AMY2B	Inverse variance weighted	5	-0.06424	0.038619	0.096245165	0.93778273	0.8694183	1.0115229	0.916211
SNAP23	Inverse variance weighted	2	0.106178	0.063977	0.096992873	1.11201926	0.9809653	1.2605816	0.916211
CNR1	Inverse variance weighted	4	-0.06055	0.036498	0.097139931	0.94124989	0.8762677	1.011051	0.916211
EPHX2	Inverse variance weighted	4	0.040341	0.024332	0.097322637	1.04116584	0.9926782	1.0920219	0.916211
SLC1A3	Inverse variance weighted	2	-0.09276	0.055967	0.097422525	0.91140869	0.8167201	1.0170752	0.916211
EIF4H	Wald ratio	1	-0.10734	0.06484	0.097830781	0.89822006	0.7910243	1.0199425	0.916211
ATIC	Inverse variance weighted	2	-0.06066	0.036674	0.098108077	0.94114027	0.8758635	1.011282	0.916211
PTPRJ	Wald ratio	1	0.085752	0.05186	0.09822034	1.08953617	0.9842329	1.2061059	0.916211
ACP5	Inverse variance weighted	5	0.032553	0.019693	0.098325384	1.03308888	0.993973	1.0737441	0.916211
TIMP3	Wald ratio	1	0.223172	0.135078	0.098499103	1.25003555	0.9592721	1.6289318	0.916211
GHRL	Inverse variance weighted	3	0.075183	0.045719	0.100084441	1.07808104	0.9856766	1.1791481	0.927449
MAP3K8	Inverse variance weighted	2	-0.07554	0.045965	0.100280241	0.92723974	0.8473561	1.0146544	0.927449
PRSS3	Wald ratio	1	-0.15468	0.094261	0.100792093	0.85668575	0.7121726	1.0305233	0.928103
HSD17B7P2	Inverse variance weighted	2	0.028223	0.01722	0.101225865	1.0286253	0.9944865	1.0639361	0.928103
LTF	Wald ratio	1	0.290915	0.177797	0.101794528	1.3376506	0.9440576	1.8953389	0.928103
RANBP2	Wald ratio	1	0.152431	0.093234	0.102065686	1.16466207	0.9701461	1.3981788	0.928103
LMCD1	Inverse variance weighted	3	-0.09166	0.056108	0.102351666	0.91241854	0.8173981	1.0184849	0.928103
NGFR	Wald ratio	1	-0.27859	0.171113	0.103503859	0.75685072	0.5411975	1.0584363	0.928103
HAPLN3	Inverse variance weighted	3	0.163637	0.100567	0.103707988	1.17778653	0.967079	1.4344032	0.928103
GPR19	Inverse variance weighted	2	0.047074	0.028992	0.104441376	1.04819979	0.9902971	1.109488	0.928103
SSH2	Inverse variance weighted	3	-0.04557	0.028091	0.104792324	0.95545671	0.904272	1.0095387	0.928103
IKBKE	Inverse variance weighted	2	0.043316	0.026708	0.104846729	1.04426757	0.9910078	1.1003897	0.928103
ADAL	Inverse variance weighted	3	-0.05467	0.033739	0.105154714	0.94679742	0.8862116	1.0115251	0.928103
PTH2R	Inverse variance weighted	2	-0.18239	0.112661	0.105455083	0.83327279	0.6681718	1.0391692	0.928103
SLC12A3	Inverse variance weighted	2	-0.15838	0.097894	0.105689119	0.85352446	0.7045091	1.0340591	0.928103
PIK3IP1	Inverse variance weighted	4	-0.04473	0.027674	0.106051308	0.95625897	0.9057723	1.0095597	0.928103
ALCAM	Inverse variance weighted	4	0.046964	0.029076	0.106267384	1.04808466	0.9900245	1.1095498	0.928103
VCL	Inverse variance weighted	5	-0.04619	0.0286	0.106328054	0.95486404	0.9028112	1.0099181	0.928103
RAF1	Wald ratio	1	0.106517	0.066002	0.106557496	1.11239738	0.9774133	1.2660233	0.928103
SLC4A2	Inverse variance weighted	2	-0.06705	0.04162	0.107192745	0.9351513	0.8618951	1.0146338	0.928103
STK16	Wald ratio	1	-0.37077	0.230204	0.107262688	0.69020143	0.4395637	1.0837519	0.928103
PTGER4	Inverse variance weighted	3	-0.05967	0.037051	0.107266971	0.94207197	0.8760845	1.0130296	0.928103
NETO2	Inverse variance weighted	2	0.25484	0.158233	0.10728079	1.2902548	0.9462045	1.7594056	0.928103
GPX1	Wald ratio	1	0.161706	0.100617	0.108023034	1.17551483	0.9651194	1.4317764	0.928103
CASP7	Inverse variance weighted	5	0.046094	0.028706	0.108340318	1.04717248	0.9898808	1.10778	0.928103
NR6A1	Inverse variance weighted	2	0.169354	0.105482	0.108377948	1.18453909	0.9632999	1.4565898	0.928103
LOXL1	Wald ratio	1	-0.45534	0.283721	0.108520645	0.63423367	0.3636981	1.1060063	0.928103

PDGFRB	Wald ratio	1	-0.45134	0.281423	0.108763969	0.63677517	0.366804	1.1054476	0.928103
TLR9	Wald ratio	1	0.329328	0.205414	0.108881928	1.39003302	0.9293372	2.0791075	0.928103
TRPV6	Inverse variance weighted	2	-0.17874	0.111514	0.108963867	0.83632176	0.6721264	1.0406288	0.928103
PLSCR3	Inverse variance weighted	2	0.050888	0.031756	0.10905534	1.05220483	0.9887099	1.1197774	0.928103
PDE4B	Inverse variance weighted	2	0.141659	0.088447	0.10923708	1.15218393	0.9688005	1.3702798	0.928103
PTGDS	Inverse variance weighted	2	0.054222	0.0339	0.109712395	1.05571929	0.9878531	1.128248	0.928103
LGMN	Inverse variance weighted	2	0.082295	0.051461	0.109781976	1.08577561	0.981603	1.2010036	0.928103
UCP2	Inverse variance weighted	6	-0.05761	0.03603	0.109812669	0.9440152	0.8796497	1.0130904	0.928103
VARS2	Inverse variance weighted	4	0.070895	0.044429	0.110556022	1.07346884	0.9839447	1.1711383	0.929407
CELA1	Inverse variance weighted	3	-0.03467	0.021731	0.110626933	0.96592491	0.9256474	1.007955	0.929407
MAP3K9	Inverse variance weighted	2	-0.06753	0.042353	0.110828315	0.93469867	0.8602407	1.0156013	0.929407
RAB35	Inverse variance weighted	2	-0.50506	0.317107	0.111221439	0.60346662	0.3241354	1.1235179	0.930294
BTLA	Inverse variance weighted	2	0.191278	0.12022	0.11159323	1.21079617	0.9566169	1.5325125	0.930998
DUSP28	Inverse variance weighted	2	-0.06222	0.039166	0.112162201	0.93967833	0.8702422	1.0146547	0.933339
KLHL24	Inverse variance weighted	2	0.036191	0.02282	0.112755734	1.03685387	0.9915	1.0842824	0.934874
FASLG	Wald ratio	1	0.107495	0.067812	0.112924265	1.11348561	0.9749032	1.2717675	0.934874
HCAR1	Wald ratio	1	0.081362	0.051413	0.113529936	1.0847634	0.98078	1.1997712	0.937491
HSD17B12	Inverse variance weighted	4	-0.04924	0.031146	0.113857921	0.95194827	0.8955737	1.0118715	0.937807
DKK3	Inverse variance weighted	6	0.040441	0.025691	0.115465483	1.04126965	0.9901345	1.0950456	0.948155
CD300C	Inverse variance weighted	5	0.041688	0.026519	0.115942191	1.0425692	0.9897645	1.098191	0.948155
ALDH1B1	Inverse variance weighted	2	-0.15076	0.095918	0.11599299	0.86004994	0.7126512	1.0379354	0.948155
MAPK13	Inverse variance weighted	4	-0.10724	0.068332	0.116558227	0.89831042	0.7857074	1.027051	0.949958
P2RY1	Wald ratio	1	0.267365	0.170476	0.116800564	1.3065168	0.9354126	1.8248484	0.949958
PTPRB	Inverse variance weighted	2	0.089474	0.057157	0.117485617	1.09359869	0.9776993	1.2232371	0.952811
SLC47A1	Inverse variance weighted	3	-0.06943	0.044394	0.11783732	0.93292614	0.8551807	1.0177395	0.952811
PSEN1	Inverse variance weighted	5	0.049619	0.031744	0.118034367	1.05087036	0.987479	1.1183311	0.952811
HTR6	Inverse variance weighted	2	-0.06374	0.040883	0.118972588	0.93824799	0.8659986	1.0165251	0.957831
LRRN1	Inverse variance weighted	4	-0.06025	0.038671	0.119248075	0.94153139	0.8728047	1.0156698	0.957831
CDH1	Inverse variance weighted	4	-0.06274	0.040417	0.120589454	0.93918782	0.8676581	1.0166144	0.96082
PROZ	Wald ratio	1	0.101699	0.065528	0.120662224	1.10705059	0.9736188	1.2587688	0.96082
MR1	Inverse variance weighted	3	-0.06383	0.041166	0.120999055	0.93816246	0.8654393	1.0169965	0.96082
PTPN9	Wald ratio	1	0.314	0.202972	0.121861185	1.36888907	0.9195915	2.0377062	0.96082
CST3	Inverse variance weighted	4	-0.03349	0.021661	0.122044372	0.96706154	0.9268639	1.0090025	0.96082
PPID	Inverse variance weighted	2	-0.19104	0.123612	0.122228841	0.82609961	0.6483539	1.0525742	0.96082
DUSP18	Inverse variance weighted	8	-0.03679	0.02385	0.122922559	0.96387757	0.9198577	1.010004	0.96082
PRSS21	Inverse variance weighted	3	-0.15624	0.101292	0.122955765	0.85535224	0.7013313	1.0431981	0.96082
SIGLEC9	Inverse variance weighted	3	0.073899	0.047941	0.123204864	1.07669831	0.9801347	1.1827754	0.96082
HMGCS1	Wald ratio	1	0.322972	0.209722	0.123560353	1.3812268	0.9156846	2.0834548	0.96082
BCKDK	Inverse variance weighted	3	0.062142	0.040368	0.123709221	1.06411301	0.9831643	1.1517267	0.96082
LILRA5	Wald ratio	1	0.066972	0.043543	0.12403124	1.06926594	0.9817956	1.1645292	0.96082
KARS	Inverse variance weighted	2	0.175882	0.114419	0.124251146	1.1922979	0.9527719	1.4920405	0.96082
WARS2	Inverse variance weighted	5	-0.0524	0.034094	0.124319854	0.94895089	0.8876109	1.0145299	0.96082
FBLN7	Inverse variance weighted	2	-0.14223	0.09259	0.124508219	0.8674222	0.7234632	1.0400271	0.96082
CTSS	Inverse variance weighted	5	-0.05382	0.035074	0.124893768	0.94759955	0.884645	1.0150341	0.96082
CAMKK2	Inverse variance weighted	5	0.040749	0.026599	0.125533136	1.0415906	0.9886789	1.097334	0.96082
MFSD11	Inverse variance weighted	2	-0.14244	0.092998	0.125609693	0.86723951	0.7227324	1.0406401	0.96082
AKT3	Inverse variance weighted	2	0.142437	0.093084	0.125964641	1.1530809	0.9607834	1.3838661	0.96082
L3MBTL1	Wald ratio	1	0.28692	0.187738	0.126437602	1.33231751	0.9221513	1.9249228	0.96082
CTSW	Inverse variance weighted	8	-0.03194	0.020906	0.126529546	0.96856182	0.9296763	1.0090738	0.96082
KDM2A	Wald ratio	1	-0.37018	0.242433	0.126774413	0.69060886	0.4294072	1.1106955	0.96082
EGF	Inverse variance weighted	2	-0.09257	0.060675	0.12707372	0.9115817	0.8093721	1.0266986	0.96082
SMN1	Wald ratio	1	-0.14936	0.097969	0.127361352	0.86125705	0.710788	1.0435794	0.96082
LIPC	Inverse variance weighted	4	0.068147	0.044723	0.12756391	1.07052302	0.9806801	1.1685967	0.96082
ADAM28	Inverse variance weighted	2	-0.07867	0.051668	0.127876209	0.92434883	0.8353248	1.0228605	0.96082
NLN	Inverse variance weighted	3	0.050321	0.033061	0.127995445	1.05160894	0.9856252	1.12201	0.96082
FSTL1	Inverse variance weighted	3	-0.0579	0.038061	0.128183668	0.94374176	0.8759005	1.0168375	0.96082
IL4	Wald ratio	1	0.126144	0.082928	0.12822803	1.13444541	0.9642595	1.3346681	0.96082
EEPD1	Inverse variance weighted	3	0.072503	0.047709	0.128584251	1.07519652	0.9792133	1.1805881	0.961264
FKBP10	Wald ratio	1	-0.29053	0.19133	0.12889871	0.74786959	0.5139993	1.0881511	0.961344
SPP1	Inverse variance weighted	4	0.033544	0.022123	0.129467046	1.03411252	0.9902298	1.0799399	0.961344
GALNT2	Inverse variance weighted	2	0.24276	0.160118	0.129485981	1.27476282	0.9313955	1.7447156	0.961344
CAPN12	Inverse variance weighted	6	-0.0361	0.023873	0.130519299	0.9645461	0.9204532	1.0107512	0.966799
OPRL1	Inverse variance weighted	6	0.047543	0.031514	0.131396283	1.04869119	0.9858758	1.1155089	0.966941

CAPN7	Wald ratio	1	0.280103	0.185742	0.131549053	1.32326634	0.9194759	1.9043826	0.966941
GYPB	Inverse variance weighted	2	0.061377	0.040712	0.131656	1.06330019	0.9817506	1.1516237	0.966941
KLHL33	Wald ratio	1	0.268287	0.178292	0.132385301	1.30772252	0.9220412	1.8547307	0.966941
MMP15	Inverse variance weighted	2	0.219893	0.146166	0.132476726	1.24594285	0.9355767	1.6592691	0.966941
ITGAM	Wald ratio	1	-0.09682	0.064374	0.132564621	0.90771743	0.8001193	1.0297852	0.966941
KLHL7	Inverse variance weighted	3	-0.08399	0.055868	0.132762844	0.91944353	0.8240792	1.0258436	0.966941
SRM	Inverse variance weighted	2	0.260422	0.173747	0.13391042	1.29747787	0.9230037	1.8238809	0.966941
ALDH1L2	Wald ratio	1	0.294239	0.19672	0.134724943	1.34210456	0.9127148	1.9735021	0.966941
OPN3	Inverse variance weighted	5	0.042594	0.028483	0.134804224	1.04351424	0.9868546	1.1034269	0.966941
PPME1	Wald ratio	1	0.235468	0.157469	0.134829095	1.26550054	0.9294413	1.7230691	0.966941
BCL2L15	Wald ratio	1	0.153109	0.102695	0.135985364	1.16545158	0.9529687	1.4253116	0.966941
SLC11A2	Wald ratio	1	0.094131	0.063218	0.136490849	1.09870353	0.9706624	1.2436347	0.966941
DUSP2	Wald ratio	1	0.191458	0.128615	0.13659073	1.21101396	0.9411729	1.5582203	0.966941
COL5A1	Wald ratio	1	-0.1613	0.108484	0.137056518	0.85103764	0.6880264	1.0526704	0.966941
ESR2	Inverse variance weighted	3	0.077935	0.052448	0.137290511	1.08105268	0.975444	1.1980954	0.966941
TOP1MT	Inverse variance weighted	2	-0.11843	0.079736	0.13746823	0.88831371	0.7597906	1.0385772	0.966941
ERAP2	Inverse variance weighted	9	-0.03419	0.023022	0.137469091	0.96638427	0.9237477	1.0109888	0.966941
TPMT	Inverse variance weighted	5	0.046841	0.031548	0.13760309	1.04795558	0.9851196	1.1147995	0.966941
TFPI	Wald ratio	1	0.301674	0.203241	0.137723899	1.35212053	0.9078489	2.0138042	0.966941
PPP3CA	Inverse variance weighted	3	-0.07723	0.052043	0.137795831	0.92567352	0.8359077	1.025079	0.966941
CHIT1	Inverse variance weighted	2	0.09175	0.061851	0.137966552	1.09609129	0.9709523	1.2373585	0.966941
KLHL18	Wald ratio	1	0.181264	0.122325	0.13838868	1.19873128	0.9431842	1.5235165	0.966941
CD38	Inverse variance weighted	2	0.178332	0.120351	0.138404661	1.19522184	0.944068	1.5131911	0.966941
CFLAR	Wald ratio	1	-0.27842	0.187935	0.138478316	0.75697668	0.5237314	1.0940983	0.966941
POLD1	Wald ratio	1	0.23467	0.158561	0.138873247	1.26449122	0.9267151	1.7253826	0.966941
CHST10	Inverse variance weighted	3	-0.13161	0.089089	0.139586984	0.87667877	0.7362173	1.0439387	0.966941
PKD2	Inverse variance weighted	6	-0.0388	0.026273	0.139747516	0.9619445	0.9136625	1.012778	0.966941
BICD1	Inverse variance weighted	4	0.148279	0.100485	0.140041298	1.1598367	0.9524943	1.4123141	0.966941
CAT	Inverse variance weighted	4	-0.02605	0.017659	0.140242488	0.97429085	0.9411455	1.0086035	0.966941
FIBCD1	Wald ratio	1	0.161483	0.109578	0.140566636	1.17525266	0.9481056	1.4568197	0.966941
PTPRN2	Inverse variance weighted	5	-0.03973	0.027037	0.141683724	0.96104669	0.9114442	1.0133486	0.966941
COPA	Inverse variance weighted	2	-0.25898	0.176237	0.141698604	0.77183848	0.5463993	1.0902918	0.966941
PAN3	Wald ratio	1	0.099059	0.067457	0.141977015	1.10413135	0.9673863	1.260206	0.966941
IL6	Wald ratio	1	-0.19033	0.129734	0.142347423	0.82668335	0.6410727	1.066034	0.966941
SLAMF8	Inverse variance weighted	3	-0.05217	0.035613	0.142953934	0.94916814	0.8851735	1.0177893	0.966941
FGFR4	Inverse variance weighted	2	-0.15463	0.105564	0.142973316	0.8567312	0.6966051	1.0536649	0.966941
AZIN1	Inverse variance weighted	3	0.055982	0.038219	0.142991623	1.05757829	0.9812495	1.1398445	0.966941
NR4A2	Wald ratio	1	0.184537	0.126025	0.143115899	1.20266121	0.9394389	1.539636	0.966941
ULK3	Inverse variance weighted	6	0.065061	0.04447	0.143456875	1.06722378	0.9781427	1.1644176	0.966941
AGBL5	Wald ratio	1	-0.32709	0.223583	0.143477514	0.72101619	0.4651867	1.1175391	0.966941
ERBB2IP	Inverse variance weighted	2	-0.21884	0.14973	0.143855551	0.80344749	0.599107	1.0774835	0.966941
MAP2K2	Inverse variance weighted	2	0.094786	0.064871	0.143978442	1.09942314	0.968156	1.248488	0.966941
MAPKAPK2	Inverse variance weighted	3	-0.09468	0.064818	0.14411371	0.90966729	0.8011394	1.0328971	0.966941
PDE12	Wald ratio	1	0.120898	0.082807	0.144290074	1.12851034	0.9594418	1.3273713	0.966941
IL10RA	Inverse variance weighted	3	-0.07447	0.051024	0.144434194	0.92823756	0.839899	1.0258674	0.966941
PDE6H	Wald ratio	1	0.072637	0.049808	0.144748687	1.07534021	0.9753224	1.1856146	0.966941
ACPP	Inverse variance weighted	5	-0.0509	0.034911	0.14487693	0.9503773	0.8875215	1.0176847	0.966941
FNDC5	Wald ratio	1	-0.22952	0.157695	0.145545955	0.7949176	0.583565	1.0828168	0.967417
BAK1P1	Wald ratio	1	-0.09629	0.066156	0.145545955	0.90820326	0.7977551	1.0339429	0.967417
HIST1H4D	Wald ratio	1	-0.34408	0.237179	0.146862238	0.70887434	0.4453261	1.1283929	0.968437
ADRB1	Inverse variance weighted	3	0.075127	0.051796	0.146937162	1.07802128	0.9739517	1.193211	0.968437
NCOA3	Inverse variance weighted	4	0.059184	0.040819	0.147083996	1.06097034	0.9793938	1.1493416	0.968437
C1QTNF6	Inverse variance weighted	4	-0.04125	0.028519	0.148077802	0.9595904	0.9074233	1.0147565	0.968437
SLC12A2	Wald ratio	1	0.094213	0.065141	0.148098405	1.09879326	0.9670894	1.2484333	0.968437
LPAR1	Inverse variance weighted	8	0.026743	0.018507	0.148467261	1.02710335	0.9905134	1.065045	0.968437
HIST1H3D	Wald ratio	1	-0.11361	0.078737	0.149060718	0.89260948	0.764961	1.0415586	0.968437
FAS	Inverse variance weighted	4	0.042744	0.029628	0.149113226	1.04367045	0.9847893	1.1060721	0.968437
ADCY9	Inverse variance weighted	5	0.046721	0.032421	0.149570346	1.04782912	0.9833158	1.116575	0.968437
RIPK3	Inverse variance weighted	3	-0.13918	0.096622	0.149743634	0.87007347	0.7199624	1.0514825	0.968437
IL27	Wald ratio	1	-0.14095	0.097905	0.149964982	0.86853382	0.7168837	1.0522641	0.968437
COL27A1	Inverse variance weighted	2	-0.14231	0.098967	0.150437623	0.86734938	0.7144167	1.0530198	0.968437
TRIM38	Inverse variance weighted	4	0.05681	0.039513	0.150509476	1.0584542	0.9795751	1.143685	0.968437
TRPV2	Inverse variance weighted	2	-0.08546	0.05946	0.150625183	0.91808708	0.8170916	1.031566	0.968437

KLHL8	Inverse variance weighted	3	0.137617	0.095756	0.15067141	1.14753556	0.951168	1.3844429	0.968437
PARP10	Inverse variance weighted	2	-0.06931	0.048246	0.150833254	0.9330368	0.8488492	1.025574	0.968437
ITGB2	Inverse variance weighted	5	0.035089	0.024445	0.151166989	1.03571179	0.9872585	1.0865431	0.968437
RRM2B	Inverse variance weighted	2	-0.08827	0.061501	0.15119898	0.91551041	0.8115443	1.0327955	0.968437
DBH	Inverse variance weighted	3	-0.07936	0.055314	0.151383682	0.92371077	0.8288045	1.0294847	0.968437
DECR2	Inverse variance weighted	4	0.057076	0.039826	0.151818937	1.05873591	0.9792359	1.1446902	0.969124
ENGASE	Inverse variance weighted	6	0.039379	0.027496	0.15208984	1.04016438	0.9855924	1.097758	0.969124
COL1A1	Wald ratio	1	0.258189	0.180851	0.153397929	1.29458383	0.90821	1.8453301	0.974416
LAIR1	Inverse variance weighted	2	-0.06048	0.042402	0.153792099	0.94131655	0.8662491	1.0228893	0.974416
TAOK3	Wald ratio	1	0.156348	0.109983	0.155151518	1.16923299	0.9425011	1.4505084	0.974416
CD52	Inverse variance weighted	4	-0.06398	0.045014	0.155207297	0.93802264	0.8588099	1.0245417	0.974416
HIST1H3E	Inverse variance weighted	2	0.045617	0.032095	0.155222074	1.04667344	0.98286	1.11463	0.974416
UQCRC1	Inverse variance weighted	2	-0.06553	0.046108	0.15527336	0.93657421	0.8556461	1.0251566	0.974416
S100A13	Inverse variance weighted	3	-0.05415	0.038115	0.155414928	0.94729165	0.8791029	1.0207695	0.974416
GPR75	Wald ratio	1	-0.21138	0.148873	0.155652133	0.80946841	0.6046113	1.083736	0.974416
SLC16A5	Inverse variance weighted	2	-0.06976	0.049184	0.156092666	0.93261808	0.8469105	1.0269993	0.974416
CXCR6	Inverse variance weighted	5	-0.07234	0.051046	0.156414944	0.93021118	0.8416481	1.0280933	0.974416
HDAC4	Inverse variance weighted	2	0.053176	0.037523	0.156441309	1.05461516	0.9798362	1.1351011	0.974416
STK33	Inverse variance weighted	3	0.06782	0.04787	0.15655362	1.07017278	0.9743303	1.1754431	0.974416
TEX14	Wald ratio	1	0.127633	0.090255	0.157320385	1.13613561	0.9519277	1.3559897	0.974416
ALDH1A1	Inverse variance weighted	5	-0.0459	0.032478	0.157620535	0.95514191	0.8962353	1.0179203	0.974416
NPM1	Wald ratio	1	-0.24675	0.174909	0.158328877	0.78133902	0.5545673	1.1008414	0.974416
HSPA1A	Wald ratio	1	0.239249	0.169608	0.158363764	1.2702946	0.9110263	1.7712423	0.974416
PIM3	Inverse variance weighted	4	0.041423	0.02938	0.158574988	1.04229239	0.9839673	1.1040747	0.974416
BLM	Inverse variance weighted	5	0.044132	0.031358	0.159328554	1.04512007	0.9828186	1.1113709	0.974416
LAMA2	Inverse variance weighted	2	-0.12922	0.091841	0.159426425	0.87877981	0.7340121	1.0520997	0.974416
SNCA	Inverse variance weighted	3	0.058925	0.041908	0.159711573	1.0606958	0.9770519	1.1515004	0.974416
USP8	Inverse variance weighted	2	-0.04201	0.029884	0.159748686	0.95885544	0.9043052	1.0166963	0.974416
TRIM65	Inverse variance weighted	3	-0.07363	0.052391	0.159917747	0.92901718	0.8383538	1.0294853	0.974416
BTN3A3	Inverse variance weighted	6	-0.08159	0.058075	0.160049634	0.92165047	0.822493	1.0327622	0.974416
SERPINB6	Inverse variance weighted	5	0.023581	0.016788	0.160145035	1.02386077	0.9907189	1.0581114	0.974416
TTL	Inverse variance weighted	2	0.121776	0.086864	0.160936738	1.1295015	0.9526801	1.3391417	0.974467
TRIM47	Inverse variance weighted	2	-0.07035	0.050283	0.161812237	0.93207137	0.844593	1.0286102	0.974467
UGT8	Wald ratio	1	0.083381	0.059679	0.162364524	1.08695611	0.9669683	1.2218327	0.974467
TPT1	Wald ratio	1	0.3412	0.244601	0.163037692	1.40663487	0.8709096	2.2719025	0.974467
FGFRL1	Inverse variance weighted	4	-0.07178	0.051475	0.163158053	0.93073286	0.8414123	1.0295353	0.974467
NCEH1	Inverse variance weighted	3	0.071672	0.051401	0.163204073	1.07430256	0.9713452	1.1881728	0.974467
F2RL1	Inverse variance weighted	5	-0.02883	0.020684	0.163357317	0.97158141	0.9329813	1.0117785	0.974467
ALDH2	Inverse variance weighted	4	-0.11679	0.083869	0.163760435	0.8897717	0.7548974	1.0487434	0.974467
EMC10	Wald ratio	1	-0.12483	0.089772	0.164384324	0.88265095	0.740242	1.0524567	0.974467
CHST2	Inverse variance weighted	3	0.101032	0.072715	0.164705959	1.10631164	0.9593589	1.2757743	0.974467
HIST1H3B	Inverse variance weighted	2	0.095626	0.068874	0.165009353	1.10034775	0.9613977	1.2593802	0.974467
BLMH	Inverse variance weighted	4	-0.05192	0.037412	0.165172057	0.94940139	0.8822752	1.0216347	0.974467
AGTPBP1	Inverse variance weighted	4	0.064603	0.046596	0.165611668	1.06673527	0.9736284	1.1687458	0.974467
FMOD	Wald ratio	1	0.39071	0.281882	0.165723713	1.47803043	0.8506291	2.5681863	0.974467
ATP1B2	Inverse variance weighted	2	0.087697	0.063291	0.165862889	1.09165727	0.9642999	1.235835	0.974467
CLCN7	Inverse variance weighted	3	-0.03351	0.024205	0.166187322	0.96704241	0.9222359	1.0140259	0.974467
NMT2	Inverse variance weighted	6	-0.03753	0.027119	0.16639613	0.96316675	0.9133089	1.0157463	0.974467
DUOX1	Wald ratio	1	-0.12438	0.08993	0.16662903	0.88304059	0.7403387	1.0532486	0.974467
ADRBK1	Wald ratio	1	-0.35397	0.255988	0.16673383	0.70189323	0.4249811	1.1592378	0.974467
LOXL2	Inverse variance weighted	4	-0.03712	0.026858	0.166929657	0.96355894	0.9141473	1.0156414	0.974467
SCGB3A2	Inverse variance weighted	4	0.038897	0.028145	0.166961097	1.03966389	0.9838647	1.0986277	0.974467
LCN2	Wald ratio	1	0.084916	0.061453	0.167027535	1.08862608	0.965093	1.2279716	0.974467
MUC1	Inverse variance weighted	4	-0.07288	0.052749	0.167077206	0.92971155	0.8383926	1.0309771	0.974467
MLH3	Inverse variance weighted	5	-0.0431	0.031256	0.167954916	0.95781983	0.9009037	1.0183317	0.977156
LY9	Inverse variance weighted	2	-0.065	0.04716	0.168142066	0.93707142	0.8543372	1.0278176	0.977156
MIF	Inverse variance weighted	2	0.066092	0.048028	0.168784415	1.0683252	0.972347	1.1737772	0.977786
PGF	Wald ratio	1	-0.10011	0.072954	0.16998165	0.90473586	0.7841912	1.0438104	0.977786
VWF	Inverse variance weighted	4	-0.04616	0.033644	0.170104269	0.9548933	0.8939561	1.0199844	0.977786
CDK10	Inverse variance weighted	6	0.040229	0.029342	0.170356601	1.04104942	0.9828679	1.102675	0.977786
CASP6	Wald ratio	1	-0.14932	0.10908	0.17102052	0.86129029	0.6955021	1.0665978	0.977786
GUCY1B2	Inverse variance weighted	4	0.044856	0.032776	0.171135935	1.04587713	0.9808018	1.1152702	0.977786
IL7R	Inverse variance weighted	4	0.054835	0.040089	0.171364252	1.05636623	0.9765403	1.1427175	0.977786

KCNJ15	Inverse variance weighted	5	-0.04567	0.033429	0.171841187	0.95535357	0.8947654	1.0200444	0.977786
PLCL1	Inverse variance weighted	3	-0.04201	0.030751	0.17191266	0.95886116	0.9027752	1.0184316	0.977786
CXCR1	Inverse variance weighted	3	-0.04365	0.031993	0.172454413	0.95728947	0.8991054	1.0192388	0.977786
LOX	Wald ratio	1	0.245865	0.180301	0.172682041	1.27872664	0.8980542	1.8207607	0.977786
TNNC1	Wald ratio	1	-0.12998	0.09553	0.173622508	0.8781102	0.7281694	1.0589261	0.977786
EMR3	Inverse variance weighted	6	0.049598	0.036481	0.173965188	1.05084892	0.9783341	1.1287386	0.977786
NPLOC4	Inverse variance weighted	2	0.07274	0.053647	0.175131959	1.07545089	0.9681111	1.194692	0.977786
SIRPG	Inverse variance weighted	2	0.044226	0.032621	0.175170912	1.04521888	0.9804827	1.1142292	0.977786
CASP3	Inverse variance weighted	3	0.062239	0.045915	0.175247513	1.06421632	0.9726278	1.1644293	0.977786
LAMB1	Wald ratio	1	0.348	0.257845	0.177128166	1.41623233	0.8543833	2.3475576	0.977786
SERPINB10	Inverse variance weighted	4	0.040007	0.029696	0.177910161	1.04081838	0.9819672	1.1031966	0.977786
FCRL5	Inverse variance weighted	12	0.030371	0.022557	0.178170031	1.03083737	0.9862544	1.0774357	0.977786
IGFALS	Inverse variance weighted	2	-0.17553	0.130532	0.178712371	0.83901198	0.6496166	1.0836255	0.977786
IFITM1	Inverse variance weighted	5	-0.04516	0.03362	0.179224157	0.95584753	0.8948918	1.0209552	0.977786
MANBA	Inverse variance weighted	3	0.058609	0.043683	0.17969668	1.0603604	0.9733519	1.1551466	0.977786
RDH5	Inverse variance weighted	3	-0.02598	0.019397	0.180431882	0.9743536	0.9380056	1.0121101	0.977786
PLA2G2D	Wald ratio	1	0.327174	0.24429	0.180477678	1.38704342	0.8593023	2.238897	0.977786
PLCXD2	Wald ratio	1	-0.37639	0.281099	0.180576191	0.68633684	0.3956043	1.1907308	0.977786
JAK3	Wald ratio	1	0.285529	0.213339	0.180771345	1.33046616	0.8758023	2.0211642	0.977786
SMPDL3A	Inverse variance weighted	4	0.056116	0.041937	0.180854288	1.05772088	0.974258	1.1483338	0.977786
GZMA	Inverse variance weighted	4	0.094265	0.070478	0.181053401	1.09885133	0.9570773	1.2616267	0.977786
GPX3	Inverse variance weighted	2	-0.05453	0.040834	0.181724552	0.94692788	0.8740943	1.0258303	0.977786
MMEL1	Inverse variance weighted	2	-0.03347	0.025076	0.181961748	0.96708355	0.9207009	1.0158028	0.977786
HDAC1	Inverse variance weighted	2	0.153872	0.115392	0.182378956	1.16634111	0.9302544	1.4623436	0.977786
IGLC3	Wald ratio	1	-0.15809	0.118571	0.182422439	0.85376933	0.6767234	1.0771345	0.977786
PGD	Wald ratio	1	0.142694	0.10702	0.182422439	1.15337644	0.9351335	1.4225533	0.977786
EFNA1	Inverse variance weighted	2	-0.17952	0.134721	0.182675374	0.83566721	0.6417356	1.0882047	0.977786
MINK1	Inverse variance weighted	5	0.050246	0.037788	0.183621871	1.05153011	0.976463	1.1323681	0.977786
PDZD2	Inverse variance weighted	2	-0.21129	0.159147	0.184291391	0.80953705	0.5926087	1.1058735	0.977786
SACS	Wald ratio	1	0.14553	0.10962	0.184314844	1.15665194	0.9330234	1.4338801	0.977786
ITGA2	Inverse variance weighted	4	-0.05639	0.042549	0.185041588	0.94516667	0.8695409	1.0273698	0.977786
OXTR	Inverse variance weighted	3	0.228502	0.172635	0.185631633	1.2567159	0.8959572	1.7627348	0.977786
TMPRSS5	Wald ratio	1	0.340346	0.257441	0.186156863	1.40543414	0.8485393	2.3278181	0.977786
CHI3L2	Inverse variance weighted	6	0.035999	0.027246	0.186414167	1.03665529	0.9827473	1.0935204	0.977786
PDIA5	Inverse variance weighted	3	0.063429	0.048013	0.186475756	1.06548396	0.969789	1.1706217	0.977786
TLR3	Inverse variance weighted	2	-0.14251	0.107894	0.186561033	0.86718056	0.7018894	1.0713969	0.977786
PTAR1	Inverse variance weighted	2	-0.04564	0.034559	0.186653366	0.95538871	0.8928175	1.0223451	0.977786
IMPA1	Inverse variance weighted	5	0.042433	0.032158	0.186994655	1.04334613	0.9796143	1.1112243	0.977786
NID1	Inverse variance weighted	4	-0.09203	0.069748	0.186998159	0.91207464	0.7955355	1.0456857	0.977786
PRB3	Inverse variance weighted	2	-0.14264	0.108138	0.18714695	0.86706478	0.7014591	1.0717678	0.977786
ARSK	Wald ratio	1	-0.28772	0.21895	0.188811691	0.74996947	0.4882807	1.1519075	0.977786
MTCH2	Inverse variance weighted	2	-0.12328	0.093828	0.188871666	0.88401392	0.7355147	1.0624949	0.977786
IGHG1	Inverse variance weighted	2	0.103228	0.078743	0.189872517	1.10874416	0.9501767	1.2937737	0.977786
FCAR	Inverse variance weighted	3	-0.0396	0.030227	0.190208405	0.96117786	0.9058876	1.0198428	0.977786
NOTCH2	Inverse variance weighted	4	-0.09976	0.076207	0.190520089	0.90505639	0.7794837	1.0508585	0.977786
PRTFDC1	Inverse variance weighted	2	0.207535	0.158654	0.190839304	1.23064097	0.9017424	1.6795009	0.977786
SLC25A51	Inverse variance weighted	3	0.073018	0.055908	0.191542284	1.07575001	0.9640979	1.2003325	0.977786
PRKCB	Inverse variance weighted	4	-0.03939	0.030158	0.191565533	0.96137984	0.9061992	1.0199206	0.977786
PRKG2	Inverse variance weighted	2	0.05879	0.045048	0.191875821	1.06055231	0.9709268	1.158451	0.977786
UTP11L	Inverse variance weighted	2	0.100265	0.076842	0.191951984	1.10546383	0.9509017	1.285149	0.977786
NCR1	Inverse variance weighted	2	0.146275	0.112242	0.192502705	1.15751449	0.9289324	1.4423437	0.977786
BSPRY	Inverse variance weighted	3	0.040998	0.031488	0.192908731	1.04184973	0.9794949	1.108174	0.977786
PDE6D	Wald ratio	1	0.079284	0.0609	0.192955088	1.08251204	0.9607137	1.2197518	0.977786
BMPR1A	Wald ratio	1	-0.08733	0.067125	0.193268425	0.91637665	0.8034077	1.0452305	0.977786
SYT15	Wald ratio	1	-0.22711	0.174585	0.193316693	0.79683668	0.5659258	1.1219646	0.977786
HSD17B8	Inverse variance weighted	2	0.042417	0.032668	0.194146299	1.04332917	0.978619	1.1123183	0.977786
ULK1	Wald ratio	1	0.165341	0.127405	0.194372291	1.1797951	0.9190875	1.5144547	0.977786
CD3G	Wald ratio	1	0.123714	0.095363	0.194528914	1.13169216	0.9387583	1.3642779	0.977786
IL1B	Inverse variance weighted	2	0.158863	0.122458	0.194532766	1.17217722	0.9220512	1.4901553	0.977786
CCNA2	Wald ratio	1	-0.13604	0.104885	0.194620967	0.87280884	0.7106232	1.0720101	0.977786
ABCA1	Inverse variance weighted	4	0.080992	0.06247	0.194804054	1.08436191	0.9593985	1.225602	0.977786
HIST1H1C	Wald ratio	1	0.084928	0.065569	0.195238107	1.08863864	0.9573483	1.2379341	0.977786
NRG1	Inverse variance weighted	9	-0.02668	0.020608	0.195434274	0.97367256	0.9351284	1.0138054	0.977786

PRKACB	Wald ratio	1	-0.34161	0.26418	0.195975283	0.71062372	0.4234138	1.1926537	0.977786
GPR171	Wald ratio	1	0.187689	0.145166	0.196035515	1.20645836	0.907705	1.6035405	0.977786
SMOX	Inverse variance weighted	3	-0.04735	0.036633	0.196132474	0.95374985	0.8876703	1.0247485	0.977786
TXNDC15	Inverse variance weighted	3	-0.05134	0.039717	0.196151281	0.94995736	0.8788124	1.0268619	0.977786
ADH5	Inverse variance weighted	3	-0.06736	0.052116	0.196168992	0.93485624	0.844078	1.0353974	0.977786
SLC29A2	Wald ratio	1	-0.24977	0.193315	0.196344886	0.77898002	0.533302	1.1378353	0.977786
KLHL25	Wald ratio	1	-0.23367	0.180959	0.196612796	0.7916267	0.5552456	1.1286408	0.977786
PHKG2	Wald ratio	1	0.196284	0.152066	0.196779926	1.21687186	0.9032414	1.6394036	0.977786
KLHL12	Inverse variance weighted	6	-0.08266	0.064062	0.196919712	0.92066077	0.8120248	1.0438305	0.977786
HLA-A	Inverse variance weighted	6	-0.03297	0.025609	0.197949823	0.96756795	0.9202006	1.0173736	0.977786
MDM2	Inverse variance weighted	4	-0.04756	0.036969	0.198272844	0.95355354	0.8869043	1.0252114	0.977786
FOLR2	Inverse variance weighted	2	0.163593	0.127179	0.198330306	1.17773526	0.9178901	1.5111398	0.977786
SLC9A1	Inverse variance weighted	2	-0.13331	0.103682	0.198540007	0.87519719	0.7142498	1.072412	0.977786
CP	Wald ratio	1	-0.29095	0.226292	0.198542794	0.7475553	0.4797551	1.1648421	0.977786
AHCY	Inverse variance weighted	2	0.194187	0.151157	0.198907067	1.21432346	0.9029568	1.6330588	0.977786
CDK18	Wald ratio	1	0.225484	0.175526	0.19892672	1.25292842	0.8882095	1.7674092	0.977786
RPS6KA2	Inverse variance weighted	6	-0.04185	0.032591	0.199073485	0.95901096	0.8996671	1.0222692	0.977786
SLC25A19	Inverse variance weighted	2	0.132332	0.103179	0.199652784	1.14148669	0.9324874	1.3973291	0.977786
HSD17B14	Inverse variance weighted	3	-0.03839	0.029961	0.200023983	0.9623333	0.9074491	1.020537	0.977786
SCN5A	Inverse variance weighted	2	-0.12955	0.101149	0.200261938	0.87848924	0.7205051	1.0711143	0.977786
CHRNB1	Inverse variance weighted	3	0.047647	0.037241	0.200747876	1.04880005	0.9749733	1.1282171	0.977786
CNTNAP2	Inverse variance weighted	4	-0.04685	0.036634	0.200968499	0.95423355	0.8881199	1.0252688	0.977786
P2RY12	Inverse variance weighted	3	0.068337	0.053528	0.201728645	1.0707257	0.9640819	1.1891662	0.977786
SLC2A9	Inverse variance weighted	3	0.073502	0.057594	0.201879608	1.07627081	0.9613836	1.2048873	0.977786
DUSP23	Inverse variance weighted	3	-0.05404	0.042386	0.202329838	0.947395	0.87187	1.0294623	0.977786
PMS2P4	Inverse variance weighted	2	-0.04134	0.032452	0.202736448	0.95950587	0.9003765	1.0225184	0.977786
BST1	Inverse variance weighted	7	-0.04114	0.032306	0.202865217	0.95969563	0.9008123	1.0224279	0.977786
VIMP	Wald ratio	1	-0.09963	0.078283	0.203114836	0.90516967	0.7764155	1.0552754	0.977786
TNFRSF1B	Inverse variance weighted	2	0.082948	0.065178	0.203147337	1.08648522	0.9561877	1.2345381	0.977786
RTN3	Inverse variance weighted	2	0.072657	0.057168	0.203746332	1.07536196	0.9613744	1.2028648	0.977786
IFNL1	Inverse variance weighted	2	-0.05542	0.043609	0.203776503	0.94608701	0.8685816	1.0305084	0.977786
EPM2A	Inverse variance weighted	2	-0.06796	0.053484	0.203844122	0.93429687	0.8413142	1.037556	0.977786
TK2	Inverse variance weighted	3	-0.0492	0.038725	0.203882157	0.95198801	0.8824052	1.0270579	0.977786
KCNJ10	Wald ratio	1	-0.08656	0.06816	0.20408463	0.91707742	0.8023926	1.048154	0.977786
SCP2	Inverse variance weighted	7	0.040988	0.032284	0.204215117	1.04184003	0.977959	1.1098938	0.977786
CD200	Inverse variance weighted	2	-0.14766	0.1164	0.204590007	0.86272201	0.6867353	1.0838081	0.977786
SULT1B1	Inverse variance weighted	2	0.115473	0.091115	0.205035537	1.12240388	0.9388384	1.3418608	0.977786
ELOVL6	Inverse variance weighted	2	-0.08337	0.0658	0.205135899	0.92000897	0.8086902	1.0466511	0.977786
TPBG	Inverse variance weighted	3	0.071788	0.056698	0.205458344	1.07442801	0.961424	1.2007143	0.977786
CNR2	Wald ratio	1	-0.15734	0.124479	0.206230027	0.85441217	0.6694354	1.0905012	0.977786
GPR160	Inverse variance weighted	4	0.033349	0.026408	0.206646904	1.03391127	0.9817579	1.0888352	0.977786
RPS6KB2	Inverse variance weighted	4	0.041781	0.033115	0.207055451	1.0426658	0.9771416	1.1125839	0.977786
TAB2	Wald ratio	1	-0.10262	0.081366	0.207238949	0.90247144	0.7694376	1.0585066	0.977786
DUSP7	Wald ratio	1	-0.23565	0.186927	0.207439194	0.79005901	0.5477014	1.1396598	0.977786
CBR1	Inverse variance weighted	3	-0.0407	0.032288	0.207501408	0.96011924	0.9012414	1.0228435	0.977786
CD177	Inverse variance weighted	5	0.053316	0.042367	0.208239028	1.05476281	0.9707135	1.1460896	0.977786
SLC6A20	Inverse variance weighted	2	-0.24424	0.194104	0.208291758	0.78330202	0.535432	1.1459197	0.977786
SLC46A1	Wald ratio	1	-0.21227	0.168712	0.208333812	0.80874925	0.5810367	1.125704	0.977786
MFI2	Wald ratio	1	0.12198	0.096996	0.208545225	1.12973189	0.9341366	1.3662822	0.977786
GSTO1	Inverse variance weighted	4	0.069994	0.055748	0.209277282	1.07250182	0.9614901	1.1963308	0.977786
SOD2	Inverse variance weighted	4	0.041961	0.033447	0.209639814	1.04285407	0.9766812	1.1135104	0.977786
SHARPIN	Inverse variance weighted	3	0.097271	0.077541	0.209680714	1.10215862	0.9467602	1.2830636	0.977786
C17orf99	Wald ratio	1	-0.10497	0.083747	0.210051203	0.9003509	0.7640553	1.0609595	0.977786
NENF	Inverse variance weighted	4	0.062196	0.049628	0.210117679	1.06417084	0.965533	1.1728854	0.977786
TNFSF14	Inverse variance weighted	2	-0.05436	0.043389	0.210291381	0.94709494	0.8698818	1.0311618	0.977786
ADAM22	Wald ratio	1	0.211249	0.169	0.211299547	1.23522048	0.8869296	1.7202828	0.977786
AGPAT2	Wald ratio	1	-0.11033	0.088268	0.211299547	0.89553442	0.7532642	1.0646755	0.977786
NCAM1	Inverse variance weighted	3	0.051501	0.041207	0.211365939	1.05285007	0.9711598	1.1414118	0.977786
MFGE8	Inverse variance weighted	5	0.028967	0.023203	0.211886041	1.0293902	0.9836242	1.0772856	0.977786
TRPV3	Inverse variance weighted	2	0.203828	0.163298	0.211958617	1.2260871	0.8902654	1.6885859	0.977786
REL	Wald ratio	1	-0.09679	0.077546	0.211987005	0.9077492	0.7797531	1.0567558	0.977786
FBN2	Inverse variance weighted	7	0.028302	0.022702	0.212520085	1.02870659	0.983936	1.0755143	0.977786
CA1	Wald ratio	1	-0.30909	0.24801	0.212665781	0.73411713	0.4514974	1.1936457	0.977786

ACE	Wald ratio	1	0.169665	0.136266	0.213095715	1.18490782	0.9071776	1.5476645	0.977786
IL6ST	Inverse variance weighted	3	-0.1248	0.100246	0.213137746	0.88266974	0.7252161	1.0743086	0.977786
TAF1A	Inverse variance weighted	3	0.066215	0.053346	0.214515218	1.06845665	0.9623832	1.1862214	0.977786
CDK11B	Inverse variance weighted	3	0.043215	0.034825	0.214636856	1.04416271	0.9752686	1.1179236	0.977786
DHRS13	Inverse variance weighted	2	0.070853	0.05712	0.214820664	1.07342366	0.9597305	1.2005854	0.977786
ACVR1	Inverse variance weighted	3	-0.10838	0.087456	0.21526822	0.89729012	0.7559429	1.0650667	0.977786
SLC25A38	Wald ratio	1	0.056637	0.045707	0.215297198	1.05827146	0.9675881	1.1574537	0.977786
HDAC11	Inverse variance weighted	2	0.231675	0.187079	0.215574418	1.26070976	0.8737165	1.819113	0.977786
HSD17B3	Inverse variance weighted	2	0.104369	0.084292	0.215649494	1.11000945	0.9409704	1.3094153	0.977786
NAMPT	Inverse variance weighted	2	-0.12983	0.104863	0.21568421	0.87824478	0.715079	1.0786415	0.977786
SLC9B2	Wald ratio	1	-0.16408	0.132576	0.215855599	0.84867451	0.6544703	1.1005059	0.977786
KLHL9	Inverse variance weighted	6	0.039612	0.03201	0.215902308	1.0404069	0.9771383	1.107772	0.977786
WNT6	Wald ratio	1	0.31127	0.25161	0.216044985	1.36515751	0.8336969	2.2354107	0.977786
DOT1L	Wald ratio	1	0.130634	0.105643	0.216250797	1.1395509	0.9264208	1.4017132	0.977786
GNLY	Inverse variance weighted	8	-0.0276	0.022349	0.216790469	0.97277436	0.9310831	1.0163324	0.977786
SLC7A1	Inverse variance weighted	6	-0.04267	0.034555	0.216872276	0.95822557	0.8954752	1.0253731	0.977786
PSMA1	Wald ratio	1	0.259257	0.209994	0.216982222	1.29596638	0.858704	1.9558881	0.977786
PMS1	Inverse variance weighted	2	0.033064	0.02683	0.217823611	1.0336166	0.9806659	1.0894263	0.977786
ALDH6A1	Wald ratio	1	0.193731	0.157406	0.218409187	1.21376968	0.8915573	1.652431	0.977786
LTBR	Inverse variance weighted	3	0.046269	0.037603	0.21852129	1.04735588	0.9729407	1.1274627	0.977786
DUSP14	Inverse variance weighted	2	0.122732	0.099757	0.218580271	1.13058154	0.9297947	1.3747277	0.977786
PDCD1	Inverse variance weighted	3	0.04962	0.040346	0.21874705	1.05087187	0.9709717	1.1373469	0.977786
CAPN2	Inverse variance weighted	2	-0.07608	0.061874	0.218847826	0.92674206	0.8209009	1.0462296	0.977786
CD200R1	Inverse variance weighted	5	-0.0408	0.033185	0.218861024	0.96001788	0.8995627	1.0245359	0.977786
GPRC5C	Inverse variance weighted	6	0.040994	0.033359	0.219123418	1.04184597	0.9759047	1.1122428	0.977786
CYP2C8	Inverse variance weighted	2	-0.12738	0.103716	0.219384299	0.88039893	0.7184474	1.0788574	0.977786
MMP19	Wald ratio	1	-0.23496	0.191628	0.220142831	0.79059928	0.5430497	1.1509945	0.977786
CLCN3	Wald ratio	1	0.165799	0.135322	0.220490373	1.18033621	0.9053526	1.5388409	0.977786
POMC	Inverse variance weighted	5	0.042475	0.034724	0.221238046	1.04339046	0.9747416	1.1168741	0.977786
PRSS30P	Inverse variance weighted	7	-0.04571	0.037384	0.221417197	0.95531709	0.8878215	1.027944	0.977786
TTBK2	Wald ratio	1	-0.12601	0.103103	0.221623602	0.88160206	0.7202939	1.0790348	0.977786
PSMB4	Wald ratio	1	0.154855	0.126788	0.221944429	1.16748874	0.9106027	1.4968438	0.977786
MOCS3	Wald ratio	1	-0.143	0.117122	0.222112437	0.8667564	0.6889713	1.0904179	0.977786
SLC3A1	Inverse variance weighted	2	-0.1807	0.148096	0.22241309	0.83468796	0.6243992	1.1157991	0.977786
RABGGTB	Wald ratio	1	0.098592	0.08086	0.222731014	1.10361607	0.9418654	1.2931449	0.977786
HTR3B	Wald ratio	1	0.185114	0.15182	0.222731014	1.20335508	0.8936392	1.6204117	0.977786
PDIA6	Inverse variance weighted	3	-0.03504	0.028745	0.222861387	0.96556759	0.9126709	1.02153	0.977786
C4A	Inverse variance weighted	5	-0.03088	0.025353	0.223144353	0.96958732	0.922585	1.0189842	0.977786
ADAM19	Inverse variance weighted	3	-0.03911	0.032126	0.223508653	0.96164906	0.9029635	1.0241487	0.977786
PLCD4	Wald ratio	1	-0.30356	0.249438	0.223611395	0.73818483	0.4527302	1.2036239	0.977786
PMPCB	Wald ratio	1	-0.22517	0.185176	0.223984922	0.7983777	0.5553717	1.1477123	0.977786
PSMB1	Inverse variance weighted	3	-0.06687	0.055008	0.224151326	0.9353204	0.8397236	1.0418003	0.977786
LILRA3	Inverse variance weighted	2	-0.16315	0.134281	0.224381657	0.84946712	0.6528963	1.1052205	0.977786
PLCB2	Inverse variance weighted	3	-0.05199	0.042798	0.224450379	0.94933809	0.8729521	1.0324081	0.977786
CBR3	Inverse variance weighted	4	0.035456	0.029192	0.224519417	1.03609247	0.9784754	1.0971023	0.977786
SLC15A4	Inverse variance weighted	3	0.061761	0.050876	0.224768884	1.06370795	0.9627549	1.1752468	0.977786
CD8A	Inverse variance weighted	3	-0.06721	0.055386	0.224937658	0.93499777	0.8388127	1.0422122	0.977786
CD37	Inverse variance weighted	4	-0.04588	0.037883	0.225878306	0.95515853	0.8868064	1.028779	0.977786
ALDH9A1	Inverse variance weighted	5	-0.05394	0.0446	0.226488368	0.94748749	0.868179	1.0340408	0.977786
HTT	Wald ratio	1	-0.13858	0.114798	0.227352356	0.87058938	0.695177	1.0902631	0.977786
KLKB1	Inverse variance weighted	4	0.051272	0.042476	0.227399798	1.05260874	0.9685254	1.1439918	0.977786
NR2C2	Inverse variance weighted	2	0.103773	0.086032	0.227738393	1.10934837	0.9372073	1.3131074	0.977786
ABCB8	Wald ratio	1	0.118098	0.097935	0.227862856	1.12535401	0.9288071	1.3634927	0.977786
IFNAR2	Inverse variance weighted	7	0.036935	0.030756	0.229792921	1.03762535	0.9769232	1.1020993	0.977786
TLR5	Inverse variance weighted	5	-0.05797	0.04827	0.22979613	0.94368191	0.858495	1.0373218	0.977786
EHMT2	Wald ratio	1	-0.1496	0.124668	0.23013934	0.8610512	0.6743877	1.0993812	0.977786
SSH1	Inverse variance weighted	3	0.070072	0.058471	0.230756165	1.0725859	0.9564462	1.2028283	0.977786
OBSCN	Inverse variance weighted	3	0.073161	0.061053	0.230790812	1.07590359	0.9545624	1.2126694	0.977786
WNT3	Wald ratio	1	0.099822	0.083413	0.231415198	1.10497384	0.9383173	1.3012306	0.977786
EPX	Wald ratio	1	0.332504	0.278266	0.232120142	1.39445608	0.8082392	2.4058566	0.977786
HTRA1	Inverse variance weighted	5	-0.05269	0.044147	0.232677842	0.94867507	0.8700392	1.0344182	0.977786
B4GALT1	Inverse variance weighted	2	-0.04162	0.034879	0.232747735	0.95923249	0.8958474	1.0271023	0.977786
TAC4	Wald ratio	1	-0.09521	0.079852	0.233140619	0.90918404	0.7774647	1.0632194	0.977786

TNK1	Wald ratio	1	-0.11715	0.098297	0.233347198	0.88945357	0.7335869	1.0784376	0.977786
WWOX	Inverse variance weighted	5	-0.04829	0.040521	0.233379902	0.95285841	0.8801082	1.0316222	0.977786
RET	Inverse variance weighted	2	0.103296	0.086684	0.233403002	1.10881937	0.9355651	1.314158	0.977786
TUBE1	Wald ratio	1	0.082229	0.069047	0.233689275	1.08570423	0.9482819	1.2430415	0.977786
LAIR2	Inverse variance weighted	2	-0.03558	0.029882	0.233728346	0.9650414	0.9101428	1.0232515	0.977786
ADM2	Wald ratio	1	0.127617	0.107282	0.234226264	1.1361178	0.9206673	1.4019871	0.977786
HERC1	Inverse variance weighted	2	0.08582	0.07215	0.234251512	1.08961067	0.9459242	1.2551232	0.977786
SFRP2	Wald ratio	1	0.080254	0.067487	0.234365228	1.08356266	0.9493104	1.2368009	0.977786
RCE1	Inverse variance weighted	2	0.066452	0.055915	0.23465262	1.06870998	0.9577768	1.1924918	0.977786
FLT1	Inverse variance weighted	3	-0.04511	0.037964	0.234763277	0.95589475	0.8873498	1.0297346	0.977786
LILRA6	Inverse variance weighted	2	0.051113	0.043025	0.23483427	1.05244206	0.9673304	1.1450423	0.977786
CDC14B	Wald ratio	1	0.19506	0.164261	0.235030457	1.21538446	0.8808288	1.6770107	0.977786
MAP3K10	Wald ratio	1	-0.07596	0.063963	0.235030457	0.92685645	0.8176467	1.0506529	0.977786
ST3GAL4	Inverse variance weighted	2	0.092062	0.077557	0.235220252	1.09643229	0.9418116	1.2764377	0.977786
NMUR1	Inverse variance weighted	3	-0.07751	0.065297	0.235241962	0.92542216	0.8142503	1.0517727	0.977786
ME1	Inverse variance weighted	2	-0.18535	0.156296	0.235664165	0.83081323	0.6115919	1.1286131	0.977786
SEMA4D	Inverse variance weighted	6	0.034135	0.028786	0.235684479	1.03472445	0.9779619	1.0947816	0.977786
BTD	Wald ratio	1	0.150461	0.126881	0.235685212	1.16236969	0.9064439	1.4905537	0.977786
ATP2C2	Inverse variance weighted	3	0.048512	0.040917	0.235765992	1.04970833	0.9688123	1.1373592	0.977786
CX3CR1	Inverse variance weighted	3	0.050474	0.042584	0.235913636	1.05176926	0.9675465	1.1433234	0.977786
ATP13A3	Inverse variance weighted	2	-0.09942	0.08388	0.235914767	0.90536237	0.7681076	1.0671435	0.977786
PLEK	Inverse variance weighted	4	0.045266	0.038218	0.236251101	1.04630647	0.9707931	1.1276936	0.977786
ANXA1	Inverse variance weighted	4	-0.04415	0.037297	0.236527274	0.95681157	0.8893621	1.0293764	0.977786
TEC	Inverse variance weighted	3	-0.13594	0.114886	0.236689679	0.87289077	0.6968943	1.0933341	0.977786
REV3L	Inverse variance weighted	2	0.136908	0.115784	0.237032592	1.14672241	0.9139039	1.4388518	0.977786
GRIN3B	Inverse variance weighted	2	0.076347	0.064631	0.237495449	1.07933664	0.9509155	1.225101	0.977786
TMX2	Wald ratio	1	0.038791	0.032847	0.237621641	1.03955286	0.9747353	1.1086806	0.977786
PRKD2	Inverse variance weighted	4	0.040667	0.034447	0.237772495	1.04150527	0.9735084	1.1142516	0.977786
TYK2	Inverse variance weighted	2	-0.12176	0.103176	0.237961858	0.88536331	0.7232637	1.0837931	0.977786
ADAM10	Inverse variance weighted	3	0.046308	0.039262	0.238218938	1.04739681	0.9698187	1.1311806	0.977786
VN1R1	Inverse variance weighted	2	-0.08425	0.071522	0.238811919	0.91920103	0.7989683	1.057527	0.977786
CAMK2G	Wald ratio	1	-0.17989	0.152844	0.23922621	0.83536588	0.619118	1.1271457	0.977786
AKR1C2	Inverse variance weighted	6	-0.0374	0.031778	0.239235295	0.96329074	0.9051219	1.0251979	0.977786
WNT7A	Inverse variance weighted	2	-0.08957	0.076198	0.239797983	0.91432453	0.7874802	1.0616005	0.977786
KDSR	Inverse variance weighted	4	-0.05042	0.042914	0.240016225	0.95082797	0.8741228	1.0342641	0.977786
TXNRD2	Inverse variance weighted	3	-0.05357	0.045616	0.240261895	0.94784158	0.8667762	1.0364887	0.977786
YARS	Wald ratio	1	-0.14407	0.122687	0.240270108	0.86582477	0.6807643	1.1011925	0.977786
PARP9	Inverse variance weighted	4	0.10872	0.092589	0.240304294	1.11485016	0.9298298	1.3366864	0.977786
CAMK2D	Inverse variance weighted	5	-0.04473	0.038107	0.240471847	0.9562552	0.8874346	1.0304128	0.977786
JAK1	Inverse variance weighted	2	-0.16854	0.143629	0.24062768	0.84490024	0.6375969	1.1196046	0.977786
RXRB	Wald ratio	1	-0.18157	0.154766	0.240721884	0.8339603	0.6157517	1.1294972	0.977786
CD59	Inverse variance weighted	3	0.03413	0.029133	0.241386689	1.03471943	0.9772911	1.0955224	0.977786
UTS2	Inverse variance weighted	3	0.06802	0.058128	0.241936169	1.07038639	0.9551259	1.1995561	0.977786
TUBD1	Wald ratio	1	-0.11238	0.096053	0.242027272	0.89370851	0.7403448	1.0788418	0.977786
PRKCE	Inverse variance weighted	3	0.137448	0.117727	0.243003824	1.14734217	0.9109224	1.4451221	0.977786
ADCY4	Inverse variance weighted	2	-0.15962	0.136752	0.243110883	0.85246531	0.6520358	1.1145049	0.977786
NOTCH3	Wald ratio	1	0.341338	0.292576	0.243345009	1.40682899	0.7928589	2.4962421	0.977786
PRF1	Wald ratio	1	-0.09965	0.085443	0.243514655	0.90515615	0.765584	1.0701735	0.977786
ENPP1	Inverse variance weighted	2	-0.2588	0.221982	0.243672474	0.77197786	0.4996315	1.1927788	0.977786
CORIN	Inverse variance weighted	4	-0.06171	0.052947	0.243853469	0.94015977	0.8474847	1.0429691	0.977786
AKR1B1	Inverse variance weighted	4	0.042386	0.036372	0.243871922	1.04329718	0.9715115	1.1203871	0.977786
HMGCR	Wald ratio	1	-0.0994	0.085314	0.243963293	0.90537798	0.7659652	1.0701652	0.977786
MEFV	Inverse variance weighted	3	0.073207	0.062864	0.244212724	1.07595294	0.9512226	1.2170387	0.977786
SLC12A1	Inverse variance weighted	4	-0.01439	0.012372	0.244831342	0.98571495	0.9621005	1.009909	0.977786
ABCA10	Inverse variance weighted	2	0.114152	0.098161	0.24486364	1.12092301	0.9247404	1.3587255	0.977786
PCYOX1	Inverse variance weighted	2	0.085125	0.073236	0.245094634	1.08885344	0.9432568	1.2569237	0.977786
KLHL20	Inverse variance weighted	2	-0.07431	0.063953	0.245257564	0.92838386	0.8190108	1.0523629	0.977786
TPO	Inverse variance weighted	4	0.09253	0.079676	0.245507951	1.0969464	0.9383474	1.2823517	0.977786
GPR15	Wald ratio	1	0.22628	0.194852	0.245523854	1.25392656	0.8558756	1.8371033	0.977786
PMPCA	Wald ratio	1	-0.23168	0.199724	0.246048806	0.79319969	0.5362579	1.1732521	0.977786
CD300E	Inverse variance weighted	5	0.03804	0.03283	0.246567895	1.03877321	0.9740375	1.1078113	0.977786
CCL25	Wald ratio	1	-0.21715	0.187452	0.246694377	0.80481111	0.5573543	1.1621349	0.977786
MARCO	Inverse variance weighted	7	0.037503	0.032378	0.24674687	1.03821537	0.974376	1.1062374	0.977786

ITK	Inverse variance weighted	3	0.055427	0.047861	0.246824826	1.05699205	0.9623476	1.1609446	0.977786
GFM2	Inverse variance weighted	2	0.058759	0.050785	0.247268784	1.06051957	0.9600402	1.1715152	0.977786
PDGFB	Inverse variance weighted	5	0.041843	0.036244	0.248311882	1.04273046	0.9712257	1.1194996	0.977786
RBBP9	Inverse variance weighted	2	0.087041	0.075452	0.248665021	1.09094173	0.9409694	1.2648168	0.977786
SRD5A1	Inverse variance weighted	2	0.043559	0.037797	0.249134385	1.04452136	0.9699387	1.124839	0.977786
CD209	Inverse variance weighted	3	0.060928	0.052924	0.249634803	1.06282283	0.9580999	1.1789923	0.977786
CRTAM	Inverse variance weighted	3	-0.05015	0.04372	0.251382636	0.95108955	0.8729833	1.036184	0.977786
SAE1	Inverse variance weighted	2	-0.06841	0.05966	0.251528178	0.93387849	0.8308195	1.0497214	0.977786
FGFR1	Inverse variance weighted	2	0.150253	0.131209	0.252151021	1.16212815	0.8986002	1.5029396	0.977786
GCK	Wald ratio	1	-0.11622	0.101568	0.252527946	0.89028196	0.7295772	1.0863853	0.977786
GLO1	Inverse variance weighted	3	-0.04073	0.035644	0.253156353	0.96008767	0.8953036	1.0295595	0.977786
PXDN	Inverse variance weighted	4	-0.02576	0.022543	0.25318568	0.97457078	0.9324484	1.018596	0.977786
NOTCH4	Inverse variance weighted	3	-0.06858	0.060055	0.253483572	0.93372013	0.8300359	1.0503561	0.977786
PARP11	Inverse variance weighted	2	-0.09976	0.087513	0.254318912	0.90505606	0.7623995	1.0744058	0.977786
TRIM69	Inverse variance weighted	4	-0.03885	0.034109	0.254724538	0.96189614	0.8996917	1.0284014	0.977786
SLC41A2	Inverse variance weighted	2	-0.07221	0.063435	0.25501389	0.93033951	0.8215692	1.0535103	0.977786
SLC40A1	Inverse variance weighted	5	-0.0424	0.037269	0.255222429	0.95848291	0.8909636	1.031119	0.977786
CLCN2	Wald ratio	1	-0.07186	0.063216	0.255641917	0.93066056	0.8222066	1.0534203	0.977786
BAK1	Inverse variance weighted	5	-0.03565	0.031424	0.25665418	0.96498246	0.9073415	1.0262852	0.977786
AGER	Inverse variance weighted	4	0.063572	0.056061	0.256805875	1.06563571	0.954748	1.1894022	0.977786
PSMB11	Wald ratio	1	0.060943	0.053802	0.257322042	1.06283869	0.9564683	1.1810388	0.977786
PF4V1	Inverse variance weighted	6	-0.03692	0.032601	0.257381232	0.96374938	0.904094	1.0273411	0.977786
C9orf96	Wald ratio	1	-0.14417	0.12752	0.258224744	0.86573738	0.674278	1.1115611	0.977786
CNGA1	Inverse variance weighted	2	-0.05592	0.049468	0.258267779	0.94561218	0.8582335	1.0418871	0.977786
HSDL1	Inverse variance weighted	3	-0.04447	0.039351	0.258454121	0.95650504	0.8855042	1.0331988	0.977786
PARP2	Inverse variance weighted	2	0.046354	0.041043	0.258718036	1.04744566	0.9664856	1.1351875	0.977786
IRAK4	Inverse variance weighted	4	-0.0627	0.055612	0.259551822	0.93922572	0.8422332	1.047388	0.977786
LYG1	Inverse variance weighted	3	0.030111	0.026724	0.259839825	1.03056918	0.9779792	1.0859871	0.977786
NOD2	Inverse variance weighted	6	-0.02946	0.026169	0.260286404	0.97097109	0.9224248	1.0220723	0.977786
INPP5A	Inverse variance weighted	2	0.112551	0.100041	0.260566658	1.11912926	0.9198648	1.3615591	0.977786
SLC12A8	Inverse variance weighted	2	0.205183	0.182388	0.260594853	1.22775028	0.858734	1.7553407	0.977786
PLCL2	Inverse variance weighted	3	-0.10803	0.09604	0.260650117	0.89759928	0.7435862	1.0835119	0.977786
CEP41	Wald ratio	1	0.062935	0.055991	0.260999872	1.06495789	0.9542721	1.1884821	0.977786
PDE3B	Wald ratio	1	0.089421	0.079564	0.26105808	1.09354091	0.9356409	1.2780884	0.977786
IL34	Wald ratio	1	-0.17723	0.157715	0.261113847	0.83758356	0.614863	1.1409797	0.977786
CDC25B	Inverse variance weighted	5	0.048813	0.04357	0.262566837	1.05002419	0.9640775	1.143633	0.977786
C1RL	Inverse variance weighted	3	0.136518	0.12197	0.263023965	1.1462756	0.9025385	1.4558356	0.977786
RDH14	Inverse variance weighted	2	-0.20521	0.18336	0.263066144	0.81447482	0.5685889	1.1666939	0.977786
MAP3K6	Inverse variance weighted	6	-0.04133	0.036937	0.263133504	0.9595094	0.892499	1.031551	0.977786
CREBBP	Wald ratio	1	0.37205	0.332519	0.263190859	1.45070514	0.7560193	2.7837192	0.977786
CRISPLD2	Inverse variance weighted	6	0.031866	0.028483	0.263240466	1.0323791	0.9763238	1.0916528	0.977786
ADAM29	Wald ratio	1	0.139522	0.124724	0.263292016	1.14972385	0.900381	1.4681173	0.977786
SLC22A18	Inverse variance weighted	3	0.026706	0.023874	0.263309507	1.02706542	0.9801128	1.0762673	0.977786
PGLYRP1	Inverse variance weighted	4	0.044528	0.039819	0.263458896	1.04553384	0.9670381	1.1304012	0.977786
LIF	Wald ratio	1	0.316923	0.283562	0.263717762	1.37289647	0.7875254	2.3933764	0.977786
HFE	Wald ratio	1	0.235342	0.210785	0.264206093	1.26534141	0.8371134	1.9126308	0.977786
LEPR	Inverse variance weighted	3	0.029331	0.026276	0.264298697	1.02976569	0.9780748	1.0841884	0.977786
TSPO2	Wald ratio	1	-0.25684	0.230341	0.264831461	0.77349203	0.4924771	1.2148585	0.977786
HIST1H4H	Inverse variance weighted	6	-0.02884	0.025864	0.264881168	0.97157575	0.9235516	1.0220972	0.977786
SLC46A3	Inverse variance weighted	3	0.041489	0.037236	0.265184159	1.04236147	0.9689976	1.1212798	0.977786
CCDC3	Inverse variance weighted	3	0.034758	0.031203	0.265311577	1.03536888	0.9739454	1.1006662	0.977786
PIK3R4	Wald ratio	1	0.116497	0.104764	0.26613815	1.12355449	0.9149923	1.3796562	0.977786
PRSS57	Inverse variance weighted	2	0.031636	0.028461	0.266328935	1.03214173	0.9761417	1.0913545	0.977786
CLCF1	Inverse variance weighted	3	0.117399	0.10566	0.266525715	1.12456791	0.91421	1.3833288	0.977786
TF	Inverse variance weighted	2	-0.08308	0.074823	0.26684348	0.92027657	0.7947442	1.0656372	0.977786
OR52N4	Inverse variance weighted	3	-0.06988	0.062957	0.267021547	0.93250643	0.8242549	1.0549749	0.977786
FCN1	Inverse variance weighted	4	-0.03088	0.027822	0.267051471	0.96959261	0.918135	1.0239342	0.977786
PLCD1	Inverse variance weighted	2	-0.04893	0.044086	0.267060777	0.95224864	0.873421	1.0381906	0.977786
MPZ	Inverse variance weighted	3	-0.0913	0.082295	0.267244201	0.91274323	0.7767794	1.0725056	0.977786
DPP4	Inverse variance weighted	2	-0.08277	0.074661	0.26762526	0.92056705	0.795248	1.0656345	0.977786
PPAT	Inverse variance weighted	2	-0.05579	0.050365	0.267986942	0.94573781	0.8568388	1.0438603	0.977786
QSOX2	Wald ratio	1	0.154817	0.139771	0.268013552	1.16744403	0.8876891	1.5353636	0.977786
IMPDH1	Inverse variance weighted	2	0.078922	0.07127	0.268134744	1.08211972	0.9410426	1.2443465	0.977786

TRIM46	Wald ratio	1	0.157253	0.14208	0.268382088	1.17029204	0.8858364	1.5460907	0.977786
IL17D	Wald ratio	1	-0.15597	0.14099	0.268618327	0.85558461	0.6490076	1.1279144	0.977786
TRAV8-4	Inverse variance weighted	2	0.034553	0.031246	0.268795079	1.0351573	0.9736638	1.1005346	0.977786
PIK3R1	Inverse variance weighted	3	0.102327	0.092546	0.268858592	1.10774603	0.9239824	1.328057	0.977786
PDE8B	Inverse variance weighted	2	-0.04418	0.039987	0.269268516	0.95678573	0.884661	1.0347906	0.977786
ITPR3	Inverse variance weighted	6	0.034993	0.031691	0.26950729	1.0356125	0.9732433	1.1019785	0.977786
PLD2	Inverse variance weighted	5	0.03729	0.033813	0.270101247	1.03799396	0.9714325	1.1091161	0.977786
HCAR2	Wald ratio	1	0.095383	0.086491	0.270112404	1.10007994	0.9285417	1.303308	0.977786
SLC16A3	Inverse variance weighted	2	0.043644	0.039593	0.270323456	1.04461086	0.9666116	1.1289041	0.977786
STK40	Wald ratio	1	-0.19588	0.177774	0.270526084	0.82211082	0.5802384	1.1648077	0.977786
IGKV3-15	Wald ratio	1	-0.13957	0.126797	0.27101879	0.86973434	0.6783521	1.1151109	0.977786
CACNA2D3	Inverse variance weighted	2	-0.05468	0.049696	0.27117227	0.94678483	0.8589137	1.0436456	0.977786
HYOU1	Wald ratio	1	-0.33332	0.303017	0.271332122	0.71654167	0.3956471	1.2977017	0.977786
SLC22A16	Inverse variance weighted	4	-0.02915	0.026544	0.272117736	0.97127068	0.9220319	1.0231389	0.977786
TAS1R3	Wald ratio	1	-0.06738	0.061358	0.272155059	0.93484146	0.8289126	1.0543072	0.977786
SCARB1	Inverse variance weighted	5	-0.03831	0.034908	0.272473446	0.96241695	0.8987708	1.0305702	0.977786
COL7A1	Inverse variance weighted	2	-0.22437	0.204477	0.272522157	0.79902175	0.5351854	1.1929246	0.977786
NUCB2	Inverse variance weighted	3	-0.0457	0.041669	0.272750782	0.95532816	0.8804066	1.0366255	0.977786
PVR	Inverse variance weighted	3	0.077252	0.07046	0.272908504	1.08031398	0.9409642	1.2403004	0.977786
ERP29	Inverse variance weighted	2	0.190295	0.173597	0.272995424	1.2096066	0.8607471	1.6998584	0.977786
SCN1B	Wald ratio	1	-0.34582	0.315819	0.273516636	0.70763888	0.3810496	1.3141408	0.977786
CYP24A1	Inverse variance weighted	2	0.134787	0.123136	0.27368343	1.14429258	0.8989219	1.45664	0.977786
CTLA4	Inverse variance weighted	2	0.073064	0.066818	0.274187283	1.07579885	0.9437445	1.226331	0.977786
BIRC3	Inverse variance weighted	3	-0.05674	0.051927	0.274544163	0.94484128	0.8534101	1.046068	0.977786
UPP1	Inverse variance weighted	5	0.103798	0.095009	0.27461436	1.10937596	0.9208842	1.3364492	0.977786
C6orf25	Wald ratio	1	-0.23318	0.213595	0.274963641	0.79200949	0.5210927	1.2037763	0.977786
ROCK2	Wald ratio	1	0.067414	0.061796	0.275312887	1.06973781	0.9477105	1.2074773	0.977786
QPCT	Inverse variance weighted	3	0.06882	0.063153	0.275824534	1.07124376	0.9465242	1.2123971	0.977786
RDH11	Wald ratio	1	-0.14112	0.129577	0.276105861	0.8683825	0.6736173	1.1194607	0.977786
KLHL26	Wald ratio	1	0.12999	0.119355	0.276105861	1.13881705	0.9012751	1.4389661	0.977786
EGLN3	Inverse variance weighted	2	0.062189	0.057134	0.276381108	1.06416397	0.9514262	1.1902604	0.977786
TUBA1C	Inverse variance weighted	2	-0.03488	0.03207	0.276752235	0.96572098	0.9068876	1.0283711	0.977786
ALDH7A1	Inverse variance weighted	5	0.034159	0.031407	0.276764276	1.03474928	0.9729727	1.1004482	0.977786
GAS6	Inverse variance weighted	3	0.082051	0.075488	0.277062609	1.08551104	0.9362195	1.2586089	0.977786
MGST3	Inverse variance weighted	7	-0.02525	0.023232	0.27709628	0.97506555	0.931661	1.0204922	0.977786
DUSP6	Inverse variance weighted	2	0.052495	0.048321	0.277306664	1.05389725	0.9586652	1.1585894	0.977786
TRIM41	Inverse variance weighted	2	0.082599	0.076066	0.27753179	1.08610591	0.9356715	1.2607268	0.977786
ASGR1	Inverse variance weighted	3	0.073626	0.067857	0.277917751	1.07640384	0.9423538	1.2295225	0.977786
NFATC4	Wald ratio	1	-0.14914	0.13759	0.278378338	0.86144603	0.6578237	1.1280976	0.977786
OR2W3	Inverse variance weighted	5	-0.02871	0.026494	0.278469738	0.97169556	0.9225257	1.0234862	0.977786
PZP	Inverse variance weighted	2	-0.09004	0.083103	0.278605732	0.91389567	0.7765289	1.0755624	0.977786
CILP	Wald ratio	1	0.102996	0.095073	0.278660495	1.10848699	0.9200313	1.3355451	0.977786
FBP1	Inverse variance weighted	4	0.066043	0.06099	0.278875076	1.06827266	0.9479084	1.2039206	0.977786
MBOAT2	Wald ratio	1	-0.08452	0.07812	0.279267097	0.9189502	0.7884875	1.0709991	0.977786
PTPRG	Inverse variance weighted	2	0.110696	0.102338	0.279399874	1.11705472	0.914035	1.365168	0.977786
TLR6	Inverse variance weighted	2	0.08199	0.075866	0.279819417	1.08544533	0.9354691	1.259466	0.977786
SERPINF1	Wald ratio	1	-0.09244	0.085566	0.279983173	0.91170265	0.770936	1.0781721	0.977786
CELSR2	Inverse variance weighted	2	-0.05152	0.047692	0.280014604	0.94978402	0.8650255	1.0428475	0.977786
KLRB1	Wald ratio	1	0.210182	0.194896	0.280841293	1.23390245	0.8421357	1.8079215	0.978257
ANG	Inverse variance weighted	7	-0.02712	0.025153	0.280946342	0.9732442	0.9264262	1.0224282	0.978257
TIPARP	Inverse variance weighted	2	-0.141	0.130801	0.281056271	0.86849229	0.672088	1.1222918	0.978257
TXNDC9	Wald ratio	1	0.284903	0.265026	0.282374728	1.32963272	0.7909284	2.2352506	0.979709
HNMT	Inverse variance weighted	4	0.043278	0.04027	0.282512974	1.04422786	0.9649767	1.1299877	0.979709
PCSK6	Inverse variance weighted	3	0.139732	0.130027	0.282536638	1.14996523	0.8912586	1.4837669	0.979709
GSTP1	Inverse variance weighted	3	0.087356	0.081534	0.283983459	1.09128534	0.9301126	1.2803866	0.979709
HIF1A	Inverse variance weighted	4	0.075842	0.070797	0.284054867	1.07879214	0.9390178	1.2393721	0.979709
HSD17B7	Inverse variance weighted	2	-0.05199	0.048612	0.284824717	0.94933591	0.8630592	1.0442373	0.979709
SFTPB	Wald ratio	1	-0.09066	0.084889	0.28553803	0.91333032	0.7733379	1.0786647	0.979709
TSTD2	Wald ratio	1	-0.31136	0.291652	0.28571562	0.73245166	0.4135424	1.2972926	0.979709
MFSD2A	Wald ratio	1	0.19217	0.180073	0.285891238	1.21187601	0.8514856	1.7248013	0.979709
NRBP1	Inverse variance weighted	2	0.064962	0.06092	0.286260158	1.06711876	0.9470153	1.2024541	0.979709
FFAR2	Inverse variance weighted	2	-0.1043	0.097838	0.286384674	0.90095169	0.7437384	1.0913971	0.979709
SCUBE3	Wald ratio	1	-0.26876	0.252107	0.286406584	0.7643298	0.4663186	1.2527917	0.979709

AATK	Inverse variance weighted	4	0.025455	0.023896	0.286767308	1.02578221	0.9788454	1.0749697	0.979709
RARS2	Inverse variance weighted	3	0.075767	0.071153	0.286944724	1.07871155	0.9382931	1.240144	0.979709
STK31	Inverse variance weighted	2	0.150625	0.141695	0.287774736	1.16256004	0.8806469	1.5347194	0.979709
STYX	Wald ratio	1	-0.13169	0.123943	0.288008758	0.87661354	0.6875528	1.1176615	0.979709
LAG3	Wald ratio	1	0.164209	0.154825	0.288868967	1.17846001	0.8700115	1.5962639	0.979709
ACACB	Inverse variance weighted	3	0.025526	0.024074	0.289001248	1.02585439	0.978574	1.0754191	0.979709
PSMG3	Inverse variance weighted	4	-0.06562	0.06194	0.289427073	0.93648832	0.8294262	1.05737	0.979709
STAT5B	Wald ratio	1	-0.13345	0.125997	0.289536123	0.87507251	0.683586	1.1201983	0.979709
SIK2	Inverse variance weighted	3	0.050236	0.047457	0.289797834	1.0515196	0.9581227	1.1540207	0.979709
ICAM3	Inverse variance weighted	3	0.03099	0.029296	0.290143916	1.03147518	0.9739151	1.0924371	0.979709
CYP17A1	Wald ratio	1	0.14187	0.134131	0.290195743	1.15242647	0.8860094	1.4989534	0.979709
TNFRSF10C	Inverse variance weighted	2	0.043419	0.041061	0.290315862	1.0443757	0.9636177	1.1319018	0.979709
NOG	Wald ratio	1	-0.0843	0.079844	0.291053222	0.91915537	0.7860037	1.0748634	0.979709
TRIM6	Inverse variance weighted	5	0.042351	0.040135	0.291331454	1.04326066	0.9643372	1.1286434	0.979709
ABCD2	Inverse variance weighted	2	-0.06593	0.062647	0.292604295	0.93619523	0.8280189	1.0585042	0.979709
FLT3	Inverse variance weighted	2	-0.2513	0.238852	0.29275144	0.77779124	0.4870217	1.2421607	0.979709
LRRK1	Inverse variance weighted	3	-0.03585	0.034138	0.293640522	0.964784	0.9023415	1.0315475	0.979709
FPR1	Inverse variance weighted	5	0.023899	0.022777	0.294055289	1.02418652	0.9794703	1.0709442	0.979709
NFAT5	Wald ratio	1	-0.14632	0.13948	0.294169094	0.86388392	0.657246	1.1354888	0.979709
SENP8	Wald ratio	1	0.307228	0.292938	0.29427916	1.35965077	0.7657259	2.4142454	0.979709
SCN8A	Wald ratio	1	0.0693	0.06615	0.294814158	1.0717573	0.9414315	1.2201246	0.979709
EPDR1	Inverse variance weighted	8	-0.03526	0.033671	0.29500727	0.96535438	0.9037029	1.0312118	0.979709
EIF2AK1	Inverse variance weighted	3	0.048539	0.046377	0.295276672	1.04973672	0.9585242	1.1496289	0.979709
COL23A1	Inverse variance weighted	4	0.040234	0.038458	0.295477699	1.04105479	0.9654663	1.1225612	0.979709
TUBA1B	Inverse variance weighted	2	0.111762	0.106849	0.295571326	1.11824683	0.9069547	1.3787634	0.979709
IGKV1D-16	Wald ratio	1	0.048202	0.046099	0.295733105	1.049383	0.9587244	1.1486145	0.979709
ENDOG	Inverse variance weighted	6	0.031938	0.030568	0.296102979	1.03245372	0.9724127	1.0962019	0.979709
PPARA	Wald ratio	1	-0.28049	0.268541	0.296258038	0.75541472	0.4462714	1.2787093	0.979709
TRIM11	Inverse variance weighted	2	-0.11559	0.110718	0.296495819	0.89084329	0.7170617	1.1067412	0.979709
HDAC7	Inverse variance weighted	3	0.09131	0.087486	0.296619279	1.09560891	0.9229662	1.3005448	0.979709
ZADH2	Inverse variance weighted	4	0.028321	0.027146	0.2968201	1.02872613	0.9754213	1.0849439	0.979709
CDK5R1	Inverse variance weighted	6	0.021564	0.020689	0.297278952	1.02179835	0.9811923	1.0640849	0.979709
ADC	Inverse variance weighted	3	0.089572	0.085942	0.297298021	1.09370638	0.9241569	1.294362	0.979709
C4B	Inverse variance weighted	2	0.015988	0.015343	0.297402717	1.01611655	0.9860138	1.0471383	0.979709
CYP4F22	Inverse variance weighted	5	0.04228	0.04059	0.297582699	1.04318603	0.9634099	1.1295681	0.979709
SLC9A8	Inverse variance weighted	3	0.070695	0.06793	0.298010727	1.07325394	0.9394626	1.2260988	0.979709
TNIK	Inverse variance weighted	3	0.065021	0.062543	0.298520726	1.06718109	0.9440613	1.2063575	0.979709
RDH13	Inverse variance weighted	5	0.031089	0.02991	0.298618322	1.03157722	0.9728401	1.0938607	0.979709
FREM1	Wald ratio	1	-0.25477	0.245121	0.298632654	0.77509372	0.4794056	1.2531566	0.979709
THSD1	Wald ratio	1	0.144121	0.138671	0.298664626	1.15502422	0.8801398	1.5157603	0.979709
TRRAP	Inverse variance weighted	2	-0.08904	0.085762	0.299160495	0.91480765	0.7732634	1.0822613	0.979709
DST	Inverse variance weighted	2	-0.09444	0.090973	0.299208449	0.9098804	0.7612836	1.0874822	0.979709
C6orf120	Inverse variance weighted	2	0.041303	0.039791	0.299266916	1.04216772	0.9639782	1.1266994	0.979709
SERPINB1	Inverse variance weighted	4	-0.033	0.0318	0.29942886	0.96754121	0.9090777	1.0297646	0.979709
IDE	Inverse variance weighted	2	-0.09037	0.087126	0.299609515	0.91359037	0.7701736	1.0837133	0.979709
UGCG	Inverse variance weighted	2	-0.0898	0.086619	0.299856976	0.91411262	0.7713794	1.0832567	0.979709
STK17A	Wald ratio	1	-0.13551	0.130711	0.299876975	0.87327254	0.6759063	1.1282702	0.979709
HP	Inverse variance weighted	4	-0.02618	0.025263	0.300017533	0.97415702	0.9270954	1.0236076	0.979709
SPINK2	Inverse variance weighted	6	0.052628	0.050844	0.300621265	1.05403799	0.9540633	1.1644889	0.979709
LPL	Inverse variance weighted	5	-0.03413	0.032978	0.300736341	0.96644861	0.905957	1.0309793	0.979709
NAALAD2	Wald ratio	1	-0.11742	0.113607	0.301343288	0.88921198	0.7117063	1.1109891	0.979709
PRIM1	Inverse variance weighted	3	0.048671	0.047094	0.30138193	1.0498744	0.9573044	1.1513958	0.979709
SRPK1	Inverse variance weighted	4	0.039949	0.03868	0.301686467	1.04075821	0.9647724	1.1227287	0.979709
COL8A2	Wald ratio	1	-0.17994	0.174316	0.301951241	0.83532146	0.5935718	1.1755309	0.979709
QSOX1	Inverse variance weighted	4	-0.02875	0.027881	0.302456696	0.97165904	0.9199858	1.0262347	0.979709
CCR2	Inverse variance weighted	4	-0.07081	0.068685	0.302557068	0.9316372	0.8142936	1.0658906	0.979709
INSR	Inverse variance weighted	3	0.154745	0.150166	0.30277707	1.16736068	0.8697237	1.5668551	0.979709
RSPRY1	Wald ratio	1	0.069851	0.067816	0.303010006	1.07234806	0.9388786	1.2247913	0.979709
MAP3K3	Wald ratio	1	-0.14398	0.139941	0.303551975	0.86590684	0.6581893	1.1391778	0.979709
HSDL2	Inverse variance weighted	3	0.025864	0.025143	0.303644591	1.02620108	0.9768548	1.0780402	0.979709
SLC15A2	Inverse variance weighted	2	-0.08594	0.083562	0.303708208	0.91764496	0.7790139	1.0809463	0.979709
GPR183	Wald ratio	1	0.166355	0.161798	0.303870331	1.1809927	0.8600471	1.6217062	0.979709
ITGA9	Inverse variance weighted	3	-0.07003	0.068246	0.304809422	0.93236396	0.8156306	1.0658042	0.981163

MAP3K5	Inverse variance weighted	3	-0.02811	0.027402	0.304927388	0.97227936	0.9214382	1.0259257	0.981163
ESM1	Inverse variance weighted	2	0.058674	0.057273	0.305613789	1.06042996	0.9478295	1.1864072	0.982025
ITGA1	Inverse variance weighted	5	0.040855	0.039909	0.305976887	1.04170133	0.9633224	1.1264574	0.982025
JAG1	Inverse variance weighted	3	0.110007	0.107582	0.306524939	1.1162859	0.9040653	1.3783233	0.982025
GFM1	Inverse variance weighted	2	-0.04418	0.043214	0.306588808	0.9567796	0.879078	1.0413492	0.982025
PLCB1	Inverse variance weighted	4	0.027842	0.027239	0.306712008	1.02823345	0.9747773	1.0846211	0.982025
CCL3L1	Inverse variance weighted	3	0.026167	0.02564	0.307464238	1.02651201	0.9762009	1.0794161	0.982594
ADAMTS8	Wald ratio	1	-0.16331	0.160197	0.308004813	0.84932965	0.6204598	1.1626231	0.982594
BPI	Inverse variance weighted	6	-0.02619	0.025725	0.308600891	0.97414807	0.9262487	1.0245244	0.982594
GPR56	Inverse variance weighted	3	-0.04738	0.046567	0.308924528	0.95372407	0.870531	1.0448675	0.982594
GPR35	Inverse variance weighted	3	-0.03382	0.033245	0.309060487	0.96674828	0.9057624	1.0318404	0.982594
AKR7A2	Inverse variance weighted	5	0.058998	0.058043	0.309418199	1.06077277	0.9467056	1.1885837	0.982594
PCOLCE	Wald ratio	1	0.148022	0.145709	0.30968986	1.15953831	0.8714752	1.5428197	0.982594
NRXN1	Inverse variance weighted	2	-0.05418	0.053367	0.30999138	0.9472611	0.8531839	1.0517119	0.982594
CD5	Inverse variance weighted	2	-0.03895	0.038396	0.3103831	0.96179956	0.8920754	1.0369733	0.982594
DUSP3	Inverse variance weighted	4	0.039872	0.039313	0.310482816	1.04067727	0.9635008	1.1240356	0.982594
MICAL3	Inverse variance weighted	3	0.098673	0.097342	0.310736576	1.10370566	0.9119986	1.3357106	0.982594
ENG	Inverse variance weighted	3	-0.04179	0.041233	0.310779623	0.95906836	0.8846095	1.0397945	0.982594
TGFA	Inverse variance weighted	4	-0.06498	0.064115	0.310836159	0.93708722	0.8264259	1.0625664	0.982594
PKN2	Inverse variance weighted	2	-0.2036	0.201153	0.311462375	0.81578968	0.5499881	1.2100495	0.983248
LGALS3	Wald ratio	1	0.166991	0.165049	0.311650569	1.18174338	0.855127	1.6331112	0.983248
FKRP	Inverse variance weighted	5	0.033713	0.033384	0.312559295	1.03428809	0.9687785	1.1042275	0.984432
IL17RB	Inverse variance weighted	3	-0.07689	0.076187	0.312875097	0.92599351	0.7975476	1.0751257	0.984432
FBLN2	Inverse variance weighted	2	-0.16036	0.158917	0.312938035	0.8518374	0.6238549	1.1631341	0.984432
CD72	Inverse variance weighted	2	0.072923	0.072385	0.313729702	1.07564769	0.9333711	1.2396119	0.985964
SLC9A9	Inverse variance weighted	3	0.077839	0.077389	0.314499486	1.08094912	0.9288182	1.2579975	0.986721
P4HA1	Wald ratio	1	-0.1118	0.111258	0.31494406	0.89421975	0.7190171	1.1121139	0.986721
GGT5	Wald ratio	1	-0.12456	0.123977	0.315033149	0.88288343	0.6924234	1.1257319	0.986721
PLOD1	Inverse variance weighted	4	0.048509	0.048303	0.315245258	1.04970527	0.9548852	1.153941	0.986721
IGKV2D-29	Wald ratio	1	0.032244	0.032123	0.315494599	1.03276911	0.9697498	1.0998838	0.986721
GALE	Wald ratio	1	0.156679	0.156679	0.317310508	1.16961955	0.860354	1.5900547	0.990486
TP73	Wald ratio	1	-0.19107	0.191066	0.317310508	0.82607771	0.5680439	1.2013234	0.990486
LTA	Inverse variance weighted	2	-0.05716	0.057201	0.317688189	0.94444657	0.8442812	1.0564956	0.99071
SLC2A5	Inverse variance weighted	2	0.043478	0.043573	0.318362064	1.04443722	0.9589422	1.1375545	0.990752
HHAT	Inverse variance weighted	3	0.091563	0.091831	0.318725421	1.09588604	0.9153707	1.3119998	0.990752
LMNA	Inverse variance weighted	2	0.036175	0.036312	0.319137847	1.03683717	0.9656092	1.1133193	0.990752
HEXA	Inverse variance weighted	2	-0.11995	0.120406	0.31914868	0.88696553	0.7005117	1.1230474	0.990752
MGST2	Inverse variance weighted	5	0.020453	0.020535	0.319232059	1.02066388	0.9804	1.0625814	0.990752
PARM1	Inverse variance weighted	2	0.044437	0.044783	0.321065313	1.04543939	0.9575875	1.1413511	0.992342
CPT2	Inverse variance weighted	2	-0.04427	0.044616	0.321082657	0.95669602	0.8765891	1.0441235	0.992342
ERBB2	Wald ratio	1	0.154374	0.15583	0.321854459	1.16692672	0.8598021	1.5837575	0.992342
IMPG2	Wald ratio	1	0.052522	0.053022	0.321897528	1.05392539	0.9498975	1.1693459	0.992342
IL2RB	Wald ratio	1	0.147988	0.149453	0.322078289	1.15949866	0.8650741	1.5541294	0.992342
LILRA1	Inverse variance weighted	3	-0.02641	0.026675	0.322134482	0.97393511	0.9243227	1.0262104	0.992342
PIK3CB	Wald ratio	1	0.210275	0.212399	0.322174119	1.23401778	0.8138108	1.8711964	0.992342
CAPZA2	Inverse variance weighted	3	-0.05007	0.050605	0.32243016	0.9511603	0.8613461	1.0503396	0.992342
PSMB3	Inverse variance weighted	4	0.032334	0.032682	0.322503396	1.03286208	0.9687739	1.1011899	0.992342
TNXB	Inverse variance weighted	3	0.022425	0.02271	0.323410848	1.02267848	0.9781564	1.0692271	0.992571
ULK2	Inverse variance weighted	2	0.055343	0.056054	0.32349275	1.05690263	0.9469362	1.1796393	0.992571
ALDH16A1	Inverse variance weighted	3	-0.02153	0.02181	0.323497906	0.97869696	0.937741	1.0214416	0.992571
GSTM3	Inverse variance weighted	5	-0.02388	0.024247	0.324648184	0.97640093	0.9310839	1.0239236	0.992869
NFATC3	Inverse variance weighted	3	0.04775	0.048495	0.324802502	1.04890807	0.9538012	1.1534983	0.992869
SGK2	Wald ratio	1	0.234889	0.238588	0.324871636	1.26476794	0.792357	2.0188349	0.992869
BRD2	Inverse variance weighted	3	0.052803	0.053712	0.325575253	1.05422162	0.9488796	1.1712585	0.992869
CTSF	Inverse variance weighted	3	-0.03063	0.031213	0.326357742	0.96983013	0.9122777	1.0310134	0.992869
SCYL3	Inverse variance weighted	3	-0.0488	0.049724	0.326380453	0.95237078	0.8639328	1.0498618	0.992869
COL24A1	Inverse variance weighted	2	0.141414	0.144156	0.326601923	1.15190188	0.8683752	1.5280008	0.992869
TNFSF4	Inverse variance weighted	3	-0.03448	0.035222	0.327555337	0.96610358	0.9016583	1.035155	0.992869
IL1RAP	Inverse variance weighted	2	0.050597	0.051718	0.32791195	1.05189927	0.950497	1.1641195	0.992869
LIG1	Inverse variance weighted	2	0.042039	0.042971	0.327922438	1.04293506	0.958693	1.1345796	0.992869
CRISP2	Inverse variance weighted	2	0.068015	0.069531	0.327977452	1.07038114	0.9340123	1.2266603	0.992869
TXNDC12	Inverse variance weighted	4	0.041066	0.042119	0.329562981	1.04192104	0.959361	1.1315859	0.992869
ROR2	Wald ratio	1	0.057419	0.058903	0.329649483	1.05909996	0.943622	1.1887099	0.992869

DYRK1A	Wald ratio	1	0.115279	0.118273	0.329717258	1.12218669	0.8899974	1.4149513	0.992869
ISG15	Inverse variance weighted	2	0.05931	0.060867	0.329841744	1.06110449	0.9417758	1.1955529	0.992869
FMO5	Inverse variance weighted	3	-0.04883	0.050215	0.330798672	0.95233879	0.8630731	1.050837	0.992869
PLK2	Wald ratio	1	0.157021	0.16155	0.331069147	1.17001968	0.8524698	1.6058587	0.992869
CWC27	Wald ratio	1	-0.12623	0.129941	0.331334907	0.88141339	0.6832372	1.1370715	0.992869
PRSS33	Wald ratio	1	-0.15353	0.158081	0.331437232	0.8576735	0.6291596	1.1691848	0.992869
CRB2	Inverse variance weighted	2	-0.16153	0.166443	0.331806783	0.85084084	0.6140006	1.1790381	0.992869
MPL	Wald ratio	1	-0.04909	0.050611	0.332046492	0.95209244	0.8621796	1.0513819	0.992869
PTGES	Inverse variance weighted	5	0.032289	0.033293	0.332115935	1.03281645	0.9675728	1.1024595	0.992869
IGHE	Wald ratio	1	0.187362	0.193279	0.332351759	1.20606388	0.8257491	1.76154	0.992869
HPSE	Inverse variance weighted	4	-0.0379	0.039116	0.332542313	0.96280566	0.8917485	1.0395249	0.992869
P2RY2	Wald ratio	1	0.039508	0.040836	0.333304297	1.0402986	0.96028	1.126985	0.992869
PTPRU	Wald ratio	1	0.112794	0.116772	0.334078277	1.11940158	0.8904042	1.4072933	0.992869
MAP3K13	Inverse variance weighted	3	-0.06399	0.066261	0.334184791	0.93801525	0.8237734	1.0681002	0.992869
LYN	Inverse variance weighted	2	0.058827	0.060921	0.334233648	1.0605916	0.9412196	1.1951032	0.992869
SMPD1	Wald ratio	1	0.125757	0.130248	0.334285804	1.1340061	0.8785089	1.4638097	0.992869
CNDP1	Wald ratio	1	-0.1436	0.148778	0.334435938	0.86623117	0.6471294	1.1595154	0.992869
CDK15	Wald ratio	1	-0.10957	0.113569	0.334673256	0.89622353	0.7173714	1.1196664	0.992869
LYZ	Inverse variance weighted	8	0.019821	0.020561	0.335043554	1.02001904	0.9797292	1.0619657	0.992869
PTPN23	Wald ratio	1	0.175934	0.182636	0.335395612	1.19235941	0.8335738	1.7055731	0.992869
MMP9	Inverse variance weighted	3	-0.05379	0.055836	0.335401677	0.94763498	0.8494012	1.0572295	0.992869
INPPL1	Inverse variance weighted	3	0.061868	0.064256	0.335622345	1.06382241	0.9379372	1.2066033	0.992869
COL11A2	Inverse variance weighted	3	-0.05304	0.055096	0.335662532	0.94833753	0.8512641	1.0564807	0.992869
LGALS3BP	Inverse variance weighted	3	-0.06348	0.065955	0.335825245	0.93849469	0.8246884	1.0680062	0.992869
MMP14	Inverse variance weighted	2	-0.12086	0.12565	0.336099494	0.88615569	0.6927153	1.1336142	0.992869
LDLR	Inverse variance weighted	2	-0.16291	0.169502	0.33650604	0.84967046	0.6094916	1.1844952	0.992869
TPH1	Inverse variance weighted	2	0.065014	0.067738	0.337165006	1.06717376	0.9344914	1.2186949	0.992869
CSF1	Wald ratio	1	-0.10682	0.111354	0.337419713	0.89868818	0.7224747	1.1178806	0.992869
MMRN1	Wald ratio	1	0.068324	0.071231	0.337466224	1.07071192	0.9311924	1.2311354	0.992869
GUSBP1	Inverse variance weighted	2	-0.04406	0.04596	0.337753806	0.9568986	0.8744681	1.0470993	0.992869
ORM1	Inverse variance weighted	4	-0.02151	0.022444	0.337773856	0.97871602	0.9365962	1.02273	0.992869
TULP2	Wald ratio	1	0.189593	0.197888	0.338020487	1.20875778	0.8201508	1.7814961	0.992869
PTX3	Wald ratio	1	-0.20161	0.210771	0.338808707	0.81741632	0.5407938	1.2355347	0.992869
NEK11	Inverse variance weighted	3	0.039665	0.041478	0.338933249	1.04046193	0.9592218	1.1285826	0.992869
UGT2B28	Inverse variance weighted	2	-0.05147	0.053841	0.339046183	0.94982775	0.8547018	1.055541	0.992869
NEGR1	Inverse variance weighted	3	-0.03942	0.041305	0.339921868	0.96134823	0.886586	1.0424149	0.992869
KLF5	Inverse variance weighted	3	-0.06747	0.070714	0.340004936	0.93475377	0.8137749	1.0737179	0.992869
PSMA7	Wald ratio	1	0.138169	0.145078	0.340903816	1.14816992	0.8639995	1.5258044	0.992869
TSHR	Inverse variance weighted	2	-0.08958	0.094082	0.341018784	0.91431423	0.7603454	1.0994616	0.992869
TULP3	Wald ratio	1	-0.14088	0.147986	0.341096982	0.8685912	0.649901	1.1608702	0.992869
S1PR5	Inverse variance weighted	2	-0.08142	0.085642	0.341737009	0.92180358	0.7793607	1.0902806	0.992869
CD244	Inverse variance weighted	5	-0.03436	0.036221	0.342855468	0.96622675	0.90001	1.0373152	0.992869
C5	Inverse variance weighted	8	-0.02997	0.031597	0.342861456	0.97047415	0.9121959	1.0324756	0.992869
AHR	Inverse variance weighted	4	0.035137	0.037064	0.343126033	1.03576168	0.963186	1.1138059	0.992869
ADORA3	Inverse variance weighted	4	0.036382	0.038433	0.343836272	1.0370515	0.9618007	1.1181899	0.992869
MADCAM1	Inverse variance weighted	2	-0.11994	0.126756	0.344030537	0.88697334	0.691853	1.1371226	0.992869
PRLR	Inverse variance weighted	2	-0.05761	0.060899	0.344191848	0.94402254	0.8378073	1.0637035	0.992869
SLC6A9	Wald ratio	1	-0.10965	0.116042	0.344682295	0.89614396	0.7138402	1.1250053	0.992869
SLK	Inverse variance weighted	3	0.054026	0.057183	0.344763472	1.05551237	0.9436004	1.1806973	0.992869
NUP62	Inverse variance weighted	3	-0.04443	0.047041	0.344895373	0.95654035	0.87229	1.0489281	0.992869
APP	Inverse variance weighted	3	0.043171	0.045722	0.345063224	1.04411621	0.9546183	1.1420048	0.992869
NOS2	Inverse variance weighted	2	0.076533	0.081104	0.345353391	1.07953756	0.9208754	1.2655364	0.992869
AAK1	Wald ratio	1	0.247641	0.2625	0.345478273	1.28100047	0.7657815	2.1428595	0.992869
GSTM1	Inverse variance weighted	8	-0.01419	0.015048	0.34567131	0.98590975	0.9572561	1.0154211	0.992869
CRHR2	Wald ratio	1	0.085084	0.09024	0.345753979	1.08880807	0.9122993	1.2994672	0.992869
THRB	Wald ratio	1	0.177183	0.188003	0.345964324	1.1938495	0.8258819	1.7257632	0.992869
PTPRF	Wald ratio	1	-0.14912	0.158357	0.346363326	0.86146634	0.6316003	1.1749904	0.992869
GSTCD	Wald ratio	1	0.057223	0.060768	0.346363326	1.05889193	0.9399939	1.1928291	0.992869
PMP22	Inverse variance weighted	4	-0.033	0.035056	0.346554875	0.96754052	0.9032935	1.0363571	0.992869
DEFA4	Inverse variance weighted	2	0.109307	0.116274	0.347174954	1.11550522	0.8881718	1.401026	0.992869
FCGR3B	Inverse variance weighted	5	0.046125	0.04907	0.347223277	1.04720568	0.9511799	1.1529257	0.992869
MMP17	Inverse variance weighted	2	-0.08897	0.094733	0.347651179	0.91487369	0.7598406	1.1015387	0.992869
TPP1	Inverse variance weighted	3	0.032481	0.034587	0.347673997	1.03301414	0.9653068	1.1054706	0.992869

COL9A2	Inverse variance weighted	3	-0.07304	0.077787	0.347743287	0.9295636	0.7981151	1.0826615	0.992869
CACNA1C	Wald ratio	1	-0.19089	0.203343	0.347846015	0.82622035	0.554634	1.2307937	0.992869
PCSK7	Inverse variance weighted	4	-0.03622	0.038605	0.348101385	0.96442615	0.894145	1.0402315	0.992869
ADHFE1	Inverse variance weighted	5	-0.02192	0.023365	0.348132775	0.97831742	0.934526	1.0241608	0.992869
UGGT1	Inverse variance weighted	2	-0.10587	0.113087	0.349156783	0.89953714	0.720705	1.1227437	0.993059
FYN	Inverse variance weighted	2	0.08255	0.08826	0.34963462	1.08605244	0.9135289	1.2911577	0.993059
BDH1	Inverse variance weighted	3	-0.0696	0.074485	0.350104854	0.93276892	0.8060669	1.0793866	0.993059
IK	Inverse variance weighted	3	-0.04	0.043026	0.352578562	0.96079238	0.8830902	1.0453315	0.993059
FST	Inverse variance weighted	4	0.053411	0.057478	0.352762264	1.05486355	0.9424748	1.1806545	0.993059
LTBP2	Inverse variance weighted	2	-0.0684	0.073656	0.353069909	0.93388635	0.8083454	1.0789246	0.993059
SLC25A23	Wald ratio	1	0.148735	0.160401	0.353784951	1.16036579	0.8473425	1.5890254	0.993059
IL17RC	Inverse variance weighted	3	-0.04435	0.047849	0.353992981	0.95661946	0.8709828	1.050676	0.993059
MRC2	Wald ratio	1	-0.25895	0.279526	0.354248343	0.77186361	0.4462757	1.3349897	0.993059
LRRC17	Inverse variance weighted	2	0.039378	0.042535	0.354565586	1.04016335	0.9569624	1.130598	0.993059
GAPDH	Inverse variance weighted	3	0.033138	0.035826	0.354979758	1.03369316	0.9635983	1.1088869	0.993059
CACNG8	Wald ratio	1	-0.10913	0.11813	0.355585496	0.89661449	0.7112983	1.1302115	0.993059
GPR153	Inverse variance weighted	2	-0.1603	0.173851	0.356512469	0.85189157	0.6058977	1.1977586	0.993059
DDOST	Inverse variance weighted	2	-0.04474	0.048554	0.356859392	0.9562503	0.8694447	1.0517226	0.993059
TNFRSF4	Inverse variance weighted	3	-0.04117	0.044682	0.356898008	0.9596702	0.8791997	1.0475059	0.993059
DHFRL1	Wald ratio	1	0.075481	0.081951	0.357022949	1.07840289	0.9183812	1.2663073	0.993059
PI3	Inverse variance weighted	3	-0.02881	0.031306	0.357395861	0.97159864	0.9137731	1.0330835	0.993059
SF3B3	Wald ratio	1	-0.05022	0.054586	0.357572759	0.95102058	0.8545253	1.0584123	0.993059
FBXO45	Wald ratio	1	-0.14979	0.162819	0.357572759	0.86088603	0.6256791	1.1845126	0.993059
SCN2B	Wald ratio	1	-0.08047	0.087462	0.357572759	0.92268718	0.7773299	1.0952257	0.993059
PI4KAP2	Inverse variance weighted	2	0.020044	0.021811	0.358111498	1.0202462	0.9775496	1.0648077	0.993059
CA11	Wald ratio	1	-0.05185	0.056475	0.358601701	0.94947519	0.8499848	1.0606109	0.993059
MYO3B	Inverse variance weighted	2	-0.06227	0.067912	0.359148308	0.93962485	0.8225203	1.0734019	0.993059
CNOT6L	Inverse variance weighted	2	-0.05857	0.063878	0.359158326	0.94310818	0.8321227	1.0688964	0.993059
SLC22A17	Inverse variance weighted	4	-0.06807	0.074298	0.359582612	0.93419646	0.8075973	1.0806414	0.993059
GP5	Inverse variance weighted	2	-0.03692	0.040331	0.359928675	0.96375054	0.8905011	1.0430252	0.993059
SIDT2	Inverse variance weighted	7	0.024454	0.026716	0.360012388	1.02475557	0.9724769	1.0798446	0.993059
TSPEAR	Inverse variance weighted	2	-0.07738	0.084781	0.361420671	0.92554145	0.7838425	1.092856	0.993059
ADCY3	Inverse variance weighted	3	0.067832	0.074333	0.361488621	1.07018516	0.9250922	1.2380348	0.993059
DHRS3	Inverse variance weighted	3	0.051401	0.056452	0.362546795	1.05274501	0.9424755	1.175916	0.993059
LMTK3	Inverse variance weighted	2	-0.08984	0.098665	0.362552855	0.91408159	0.7533552	1.1090986	0.993059
AMH	Wald ratio	1	-0.03609	0.039647	0.362640684	0.96455134	0.8924366	1.0424935	0.993059
TMIGD2	Wald ratio	1	0.070473	0.07752	0.363302141	1.07301522	0.9217637	1.2490855	0.993059
PROCR	Wald ratio	1	-0.07664	0.084306	0.363302141	0.92622188	0.78515	1.0926408	0.993059
MORC3	Inverse variance weighted	4	0.037443	0.041199	0.363435176	1.03815289	0.9576178	1.125461	0.993059
CDKN1A	Inverse variance weighted	3	0.082309	0.090617	0.363713293	1.08579084	0.9090994	1.2968239	0.993059
AGA	Inverse variance weighted	5	-0.02411	0.026553	0.363845538	0.9761765	0.9266717	1.028326	0.993059
TRPM4	Inverse variance weighted	3	0.03291	0.036249	0.363931337	1.03345799	0.9625805	1.1095544	0.993059
SMAD3	Inverse variance weighted	3	-0.05036	0.05548	0.363999724	0.95088446	0.8529087	1.0601149	0.993059
SLC22A14	Wald ratio	1	0.154017	0.169732	0.364191394	1.16651014	0.8363909	1.6269257	0.993059
MMP23A	Inverse variance weighted	2	-0.03972	0.043815	0.36465878	0.96105885	0.8819696	1.0472403	0.993059
CATSPER1	Inverse variance weighted	2	0.082935	0.091618	0.365347991	1.08647094	0.9078856	1.3001849	0.993059
MGST1	Inverse variance weighted	2	0.085201	0.094202	0.365760142	1.08893553	0.9053481	1.309751	0.993059
FRRS1	Wald ratio	1	0.125235	0.138482	0.365810994	1.13341528	0.863995	1.4868491	0.993059
LCN8	Inverse variance weighted	3	0.020151	0.022319	0.366604052	1.02035559	0.9766812	1.065983	0.993059
SLC5A5	Wald ratio	1	-0.16481	0.182744	0.367126376	0.84805418	0.5927467	1.2133275	0.993059
KSR1	Inverse variance weighted	3	0.031512	0.034982	0.367693311	1.03201328	0.9636256	1.1052544	0.993059
DDO	Inverse variance weighted	3	-0.04443	0.049358	0.368061665	0.9565446	0.8683416	1.0537069	0.993059
TECTA	Wald ratio	1	0.153152	0.170169	0.368120251	1.16550189	0.8349539	1.6269098	0.993059
CXCL10	Wald ratio	1	-0.04862	0.054024	0.368120251	0.95254178	0.8568369	1.0589365	0.993059
ALDH8A1	Inverse variance weighted	3	0.073963	0.082204	0.368252618	1.07676709	0.9165335	1.2650136	0.993059
CDK13	Inverse variance weighted	2	0.047364	0.052678	0.368580545	1.048504	0.9456489	1.1625463	0.993059
MPST	Inverse variance weighted	3	0.13013	0.145002	0.369486239	1.13897602	0.8572091	1.5133605	0.993059
PSMB5	Inverse variance weighted	3	-0.04576	0.051008	0.369622748	0.95526786	0.8643829	1.0557089	0.993059
SLC16A13	Wald ratio	1	0.247516	0.276297	0.370341784	1.28083993	0.7452571	2.2013222	0.993059
CDC42	Inverse variance weighted	5	-0.04373	0.048815	0.370347633	0.95721247	0.8698731	1.0533212	0.993059
CDC42BPB	Inverse variance weighted	4	0.048092	0.053709	0.370562029	1.04926762	0.9444262	1.1657476	0.993059
SEMG1	Inverse variance weighted	2	-0.03639	0.040642	0.370608003	0.96426599	0.8904341	1.0442198	0.993059
STRADB	Wald ratio	1	-0.0632	0.070674	0.371198433	0.93875704	0.8173246	1.0782311	0.993059

PCSK5	Inverse variance weighted	6	0.031219	0.034911	0.371199867	1.03171115	0.9634761	1.1047787	0.993059
PXK	Inverse variance weighted	4	-0.02894	0.032406	0.371907486	0.97147912	0.9116937	1.0351851	0.993059
HSP90AB1	Wald ratio	1	0.216842	0.243394	0.372977943	1.24214769	0.7708895	2.0014943	0.993059
PDE4A	Inverse variance weighted	3	-0.07737	0.08688	0.373176775	0.92554768	0.7806303	1.0973678	0.993059
PDE2A	Inverse variance weighted	2	0.145348	0.163224	0.373206681	1.15644239	0.8398171	1.5924408	0.993059
SPINT1	Inverse variance weighted	2	-0.09948	0.111722	0.373231874	0.90530681	0.7272706	1.1269264	0.993059
FCRL6	Inverse variance weighted	3	0.021211	0.023821	0.373245856	1.0214373	0.9748428	1.0702589	0.993059
TRIM14	Inverse variance weighted	3	0.043741	0.04914	0.373393212	1.04471225	0.9487846	1.1503387	0.993059
KDM1B	Inverse variance weighted	4	-0.03438	0.038634	0.373563241	0.96620732	0.8957458	1.0422115	0.993059
CLK2	Wald ratio	1	-0.23415	0.263159	0.373592739	0.79124374	0.4723946	1.3253044	0.993059
ULBP2	Wald ratio	1	-0.13259	0.149032	0.373651071	0.87582695	0.6539733	1.1729421	0.993059
KIR2DS4	Inverse variance weighted	4	-0.01509	0.01696	0.373706742	0.98502654	0.9528211	1.0183205	0.993059
NAT1	Inverse variance weighted	2	-0.07609	0.085551	0.373787996	0.92673357	0.7836684	1.0959165	0.993059
NRP1	Inverse variance weighted	5	-0.02772	0.031188	0.374164455	0.97266416	0.9149882	1.0339757	0.993059
XYLT1	Inverse variance weighted	6	-0.03067	0.034551	0.374691109	0.96979426	0.9062948	1.0377428	0.993059
SLC25A22	Wald ratio	1	0.116016	0.130827	0.375190652	1.12301428	0.8690066	1.4512675	0.993059
MMP11	Inverse variance weighted	3	-0.07244	0.081695	0.375251607	0.93012401	0.7925024	1.0916443	0.993059
SBK1	Inverse variance weighted	2	0.036138	0.040764	0.375335478	1.03679917	0.9571844	1.1230359	0.993059
GZMH	Inverse variance weighted	2	-0.06434	0.072582	0.375345832	0.93768205	0.8133406	1.0810325	0.993059
ATP2A2	Inverse variance weighted	2	-0.09057	0.102304	0.375976456	0.91340751	0.7474493	1.1162139	0.993059
METAP1D	Inverse variance weighted	2	-0.07433	0.084002	0.376232276	0.92836505	0.7874348	1.0945182	0.993059
SCUBE2	Wald ratio	1	0.245091	0.277275	0.376734773	1.27773788	0.7420285	2.200204	0.993059
NR3C2	Wald ratio	1	-0.11885	0.134527	0.376968804	0.88793773	0.6821362	1.1558299	0.993059
SEMA4B	Inverse variance weighted	2	0.041558	0.047073	0.377314039	1.04243401	0.95056	1.1431879	0.993059
SLC2A10	Wald ratio	1	-0.22239	0.251899	0.377319922	0.80060463	0.4886491	1.3117138	0.993059
PLAU	Inverse variance weighted	2	-0.05923	0.067107	0.377427898	0.94248803	0.8263287	1.0749762	0.993059
IGLL5	Wald ratio	1	0.055841	0.063358	0.378125201	1.05742979	0.9339419	1.1972456	0.993059
FAAH	Inverse variance weighted	4	-0.02745	0.031195	0.37891505	0.97292517	0.915221	1.0342675	0.993059
CRAT	Inverse variance weighted	4	-0.03109	0.035329	0.378915964	0.96939214	0.9045376	1.0388967	0.993059
CD55	Inverse variance weighted	10	0.022888	0.026078	0.380127022	1.023152	0.9721689	1.0768087	0.993059
MVD	Inverse variance weighted	3	0.047959	0.054717	0.380762156	1.04912808	0.9424365	1.167898	0.993059
IGLV3-27	Wald ratio	1	-0.02917	0.0333	0.381012063	0.97124978	0.9098833	1.0367551	0.993059
FGF9	Wald ratio	1	0.218281	0.249253	0.381171588	1.24393645	0.7631846	2.0275277	0.993059
KCNN4	Inverse variance weighted	4	0.023952	0.027367	0.381450422	1.02424127	0.9707496	1.0806805	0.993059
CYB5RL	Wald ratio	1	0.181504	0.207867	0.382570025	1.19901883	0.797785	1.8020472	0.993059
ATP2B1	Wald ratio	1	-0.20637	0.236571	0.38302269	0.81353185	0.5116836	1.293444	0.993059
SERPINC1	Inverse variance weighted	2	-0.03698	0.042403	0.383158982	0.96369599	0.886841	1.0472114	0.993059
KCNS1	Wald ratio	1	-0.10473	0.120169	0.38344866	0.90056334	0.7115805	1.1397366	0.993059
FAM3B	Inverse variance weighted	6	0.016154	0.018543	0.383679736	1.01628506	0.9800112	1.0539016	0.993059
KLHL36	Inverse variance weighted	2	-0.03566	0.040934	0.383707231	0.96497159	0.8905765	1.0455814	0.993059
TRPV5	Inverse variance weighted	3	0.018572	0.021351	0.384396501	1.01874552	0.976992	1.0622834	0.993059
ERAP1	Inverse variance weighted	4	0.054414	0.062566	0.384462334	1.05592123	0.9340595	1.1936816	0.993059
RPS6KL1	Inverse variance weighted	3	0.054798	0.063054	0.384814265	1.0563269	0.9335245	1.1952836	0.993059
IGKV3D-15	Wald ratio	1	0.068376	0.078684	0.384849519	1.07076754	0.9177372	1.2493153	0.993059
SSH3	Inverse variance weighted	4	-0.03354	0.038665	0.385736507	0.96701917	0.8964434	1.0431513	0.993059
SLC16A6	Inverse variance weighted	3	0.039289	0.045362	0.386422874	1.04007064	0.951591	1.1367771	0.993059
GGCX	Inverse variance weighted	4	-0.02956	0.034126	0.386425559	0.97087577	0.9080618	1.0380348	0.993059
GDF15	Inverse variance weighted	2	-0.06467	0.074796	0.387250008	0.93737667	0.8095547	1.0853807	0.993059
ACP2	Inverse variance weighted	7	-0.02976	0.034425	0.387326216	0.97067855	0.907344	1.038434	0.993059
SIRPB1	Inverse variance weighted	7	-0.02352	0.027224	0.387658685	0.976756	0.9260025	1.0302912	0.993059
DHRS4	Inverse variance weighted	3	-0.07565	0.087685	0.388244006	0.92713617	0.7807372	1.1009869	0.993059
YARS2	Wald ratio	1	0.106973	0.124088	0.388649568	1.11290384	0.8726328	1.4193312	0.993059
IGSF8	Inverse variance weighted	2	-0.05062	0.058759	0.388993173	0.95064177	0.8472272	1.0666794	0.993059
CR1L	Wald ratio	1	0.033681	0.039129	0.389370519	1.03425455	0.9578994	1.1166961	0.993059
BDH2	Inverse variance weighted	2	0.018764	0.021811	0.389610142	1.01894158	0.9763005	1.0634451	0.993059
SHPK	Inverse variance weighted	5	0.024425	0.028397	0.389729896	1.02472537	0.9692489	1.0833772	0.993059
CMKLR1	Inverse variance weighted	6	-0.02633	0.030611	0.389755722	0.97401644	0.9172968	1.0342432	0.993059
NOS3	Wald ratio	1	0.043839	0.050976	0.389789043	1.04481412	0.94547	1.1545967	0.993059
RECQL	Inverse variance weighted	3	-0.05367	0.062433	0.389976571	0.94774404	0.8385855	1.0711117	0.993059
CMYA5	Inverse variance weighted	2	0.133253	0.155036	0.39006632	1.14253879	0.8431442	1.5482463	0.993059
MARS	Inverse variance weighted	2	0.053274	0.062005	0.390233731	1.05471876	0.934022	1.1910123	0.993059
TNFRSF17	Wald ratio	1	-0.11538	0.134401	0.390621638	0.89102582	0.6846773	1.1595637	0.993059
IL2RA	Wald ratio	1	0.064305	0.075023	0.391365938	1.06641812	0.9205907	1.2353455	0.993059

CALM3	Inverse variance weighted	4	-0.04978	0.058091	0.391444835	0.95143463	0.8490448	1.0661721	0.993059
PPIE	Inverse variance weighted	5	-0.01731	0.020221	0.391949165	0.98283829	0.9446477	1.0225728	0.993059
CDC25A	Wald ratio	1	-0.10966	0.128304	0.392716777	0.89613708	0.6968825	1.1523632	0.993059
CD83	Inverse variance weighted	2	0.035401	0.041447	0.393025293	1.03603537	0.9552006	1.1237108	0.993059
WNK1	Inverse variance weighted	2	0.121542	0.142344	0.393180859	1.12923707	0.8543179	1.4926251	0.993059
TDP1	Inverse variance weighted	3	0.085164	0.099768	0.393317468	1.08889553	0.895492	1.3240694	0.993059
CAPN14	Wald ratio	1	-0.19603	0.229761	0.393542299	0.82198368	0.523946	1.2895549	0.993059
TGFBR3	Inverse variance weighted	3	-0.07314	0.085745	0.393648222	0.92946851	0.7856822	1.0995689	0.993059
RARG	Inverse variance weighted	2	-0.15279	0.179156	0.393760787	0.85831202	0.6041492	1.2193999	0.993059
FKBP11	Inverse variance weighted	4	0.033996	0.039865	0.393779035	1.03458096	0.9568205	1.118661	0.993059
SAMD1	Wald ratio	1	0.136684	0.160509	0.39445696	1.14646531	0.8370141	1.5703232	0.993059
TREM1	Inverse variance weighted	3	0.021222	0.024936	0.39474563	1.02144872	0.9727255	1.0726125	0.993059
SCN4A	Wald ratio	1	-0.17462	0.205367	0.395158649	0.83977287	0.5614997	1.2559552	0.993059
RNASEH1	Inverse variance weighted	2	0.179552	0.21119	0.395217748	1.19668171	0.7910608	1.8102869	0.993059
LPAR5	Inverse variance weighted	4	-0.03275	0.038527	0.395326715	0.96778228	0.8973927	1.0436931	0.993059
TGM1	Inverse variance weighted	4	0.033291	0.039167	0.395340675	1.03385158	0.9574546	1.1163444	0.993059
ANGPT4	Wald ratio	1	-0.20776	0.244503	0.395485791	0.81240424	0.5030912	1.3118908	0.993059
CCL23	Inverse variance weighted	3	-0.09318	0.109659	0.395496311	0.91103304	0.7348366	1.1294772	0.993059
KDM4A	Wald ratio	1	-0.17838	0.209963	0.395571137	0.83662821	0.5543813	1.2625729	0.993059
PIK3C2A	Inverse variance weighted	3	-0.04152	0.04889	0.395777687	0.95933316	0.8716724	1.0558096	0.993059
DUSP11	Wald ratio	1	0.144033	0.169753	0.396168007	1.15492235	0.8280486	1.6108301	0.993059
EIF4A1	Wald ratio	1	-0.16948	0.199749	0.396168007	0.84410012	0.5706424	1.2486017	0.993059
NKTR	Inverse variance weighted	2	0.0343	0.040435	0.396283978	1.03489538	0.9560425	1.1202519	0.993059
SNRK	Wald ratio	1	-0.06947	0.081901	0.396318643	0.9328885	0.7945372	1.0953307	0.993059
SULT1A2	Inverse variance weighted	3	0.027851	0.032842	0.39641156	1.02824263	0.9641405	1.0966066	0.993059
SLC43A2	Wald ratio	1	0.094464	0.111396	0.396437971	1.09906926	0.8834927	1.3672475	0.993059
DCLK2	Inverse variance weighted	4	-0.03493	0.041198	0.396477068	0.96566982	0.8907584	1.0468812	0.993059
AKR1C4	Wald ratio	1	-0.15086	0.178171	0.397153813	0.85996789	0.6064848	1.2193954	0.993059
UHMK1	Inverse variance weighted	3	-0.03674	0.043398	0.39726271	0.96392912	0.885327	1.0495097	0.993059
SLC39A6	Wald ratio	1	-0.1186	0.140159	0.397466925	0.8881662	0.6748204	1.1689618	0.993059
DUSP8	Inverse variance weighted	3	0.055195	0.065242	0.397550008	1.05674669	0.9298991	1.2008976	0.993059
CYB5R2	Inverse variance weighted	5	-0.04641	0.054876	0.397731788	0.95465288	0.8573025	1.0630578	0.993059
CD274	Inverse variance weighted	2	0.052153	0.061749	0.398342948	1.05353649	0.9334422	1.1890818	0.993059
ADAMTS5	Inverse variance weighted	3	-0.07332	0.086825	0.398411907	0.92930346	0.7838826	1.1017019	0.993059
KL	Inverse variance weighted	5	0.038564	0.045676	0.398505498	1.03931749	0.9503153	1.1366552	0.993059
GPLD1	Inverse variance weighted	2	-0.09977	0.118201	0.398612897	0.90504208	0.7178832	1.140995	0.993059
IL23A	Wald ratio	1	0.120195	0.142453	0.398809121	1.12771672	0.8529848	1.4909351	0.993059
PIGT	Inverse variance weighted	4	0.046634	0.055288	0.398964364	1.04773824	0.9401363	1.1676556	0.993059
EPHA4	Inverse variance weighted	5	-0.02864	0.033961	0.398973106	0.97176202	0.9091845	1.0386466	0.993059
CYP26B1	Inverse variance weighted	6	0.014587	0.017297	0.399057147	1.01469361	0.9808699	1.0496837	0.993059
CTBS	Inverse variance weighted	4	0.026787	0.031772	0.399173722	1.02714911	0.9651358	1.093147	0.993059
NEK1	Inverse variance weighted	4	-0.03491	0.041413	0.399207285	0.96568979	0.8904023	1.0473432	0.993059
CPQ	Inverse variance weighted	5	0.021761	0.025866	0.400176612	1.02199958	0.9714787	1.0751478	0.993059
PIN1	Wald ratio	1	-0.11161	0.132817	0.400719947	0.89439229	0.6894012	1.1603367	0.993059
DRD4	Inverse variance weighted	2	-0.05441	0.064752	0.400743138	0.94704315	0.8341649	1.075196	0.993059
ACVRL1	Inverse variance weighted	4	-0.03211	0.038269	0.401472919	0.96840245	0.8984224	1.0438334	0.993059
GPR20	Inverse variance weighted	2	0.089267	0.106495	0.401899129	1.09337303	0.8873977	1.3471576	0.993059
METRN	Inverse variance weighted	2	0.040317	0.048152	0.402431821	1.04114086	0.9473743	1.144188	0.993059
CTSZ	Inverse variance weighted	3	0.046193	0.055191	0.402610719	1.04727697	0.9399	1.1669209	0.993059
TRIB3	Inverse variance weighted	6	0.037744	0.045113	0.402785053	1.03846518	0.9505857	1.1344689	0.993059
ITGAL	Inverse variance weighted	2	0.154661	0.185022	0.403208304	1.16726162	0.8122218	1.6774971	0.993059
SCGB3A1	Inverse variance weighted	6	-0.0312	0.037354	0.403511575	0.96927742	0.9008481	1.0429047	0.993059
ERBB3	Wald ratio	1	0.051213	0.061336	0.403745041	1.05254663	0.9333211	1.1870024	0.993059
XBP1	Inverse variance weighted	14	0.023911	0.028643	0.403817774	1.02419964	0.9682858	1.0833422	0.993059
TRIM27	Inverse variance weighted	6	-0.02534	0.030374	0.404120839	0.97497812	0.9186293	1.0347834	0.993059
KCNA6	Wald ratio	1	0.083511	0.100213	0.404656762	1.08709668	0.8932344	1.3230337	0.993059
IDO1	Inverse variance weighted	3	0.029749	0.035707	0.404754637	1.03019618	0.9605631	1.104877	0.993059
ANKK1	Inverse variance weighted	3	0.037993	0.045603	0.404767328	1.03872412	0.9499102	1.1358418	0.993059
ADAM8	Inverse variance weighted	3	-0.05261	0.063224	0.405312397	0.94874655	0.8381706	1.0739102	0.993059
NFATC1	Inverse variance weighted	3	-0.04475	0.053785	0.405399087	0.95623684	0.860564	1.0625461	0.993059
IL12RB1	Inverse variance weighted	2	-0.09868	0.118755	0.405993891	0.90603156	0.7178886	1.1434828	0.993059
ADAM23	Inverse variance weighted	2	-0.07269	0.087494	0.406109227	0.9298921	0.7833502	1.1038477	0.993059
LTB4R2	Wald ratio	1	-0.17516	0.210878	0.406174114	0.83931897	0.555168	1.2689067	0.993059

LRP8	Wald ratio	1	-0.09541	0.114877	0.406251358	0.90900361	0.725739	1.1385465	0.993059
PLGLB2	Inverse variance weighted	2	-0.01775	0.021373	0.406258613	0.98240625	0.9421019	1.0244349	0.993059
TRIM25	Wald ratio	1	0.188467	0.227016	0.406432115	1.20739666	0.773766	1.8840408	0.993059
PRKCSH	Inverse variance weighted	2	-0.10172	0.122523	0.406432926	0.90328547	0.7104471	1.1484664	0.993059
MGMT	Inverse variance weighted	4	-0.01637	0.019716	0.406488343	0.98376746	0.9464772	1.0225269	0.993059
NBL1	Inverse variance weighted	7	0.020688	0.025027	0.408460782	1.02090311	0.9720328	1.0722304	0.995185
SLC4A11	Wald ratio	1	0.091904	0.111366	0.409233805	1.09625959	0.8812857	1.3636725	0.995185
GALNT1	Inverse variance weighted	2	0.03972	0.048132	0.409250637	1.04051892	0.9468448	1.1434605	0.995185
ACVR1C	Inverse variance weighted	2	0.162995	0.197527	0.40927046	1.17703097	0.7991887	1.7335104	0.995185
ADAMTS14	Wald ratio	1	-0.22984	0.278536	0.409272344	0.79466029	0.460349	1.3717526	0.995185
STK39	Inverse variance weighted	3	0.030727	0.037299	0.410052024	1.03120384	0.9585065	1.1094149	0.995185
DEAF1	Inverse variance weighted	2	-0.03347	0.040639	0.410148828	0.96708239	0.8930399	1.0472638	0.995185
CDC25C	Wald ratio	1	0.267162	0.324493	0.410325159	1.30625193	0.6915333	2.467407	0.995185
SLC12A4	Inverse variance weighted	2	0.110001	0.133777	0.410921752	1.11627912	0.8588153	1.450928	0.995185
THNSL2	Inverse variance weighted	4	-0.01414	0.017218	0.411480969	0.98595873	0.9532409	1.0197995	0.995185
SMC3	Wald ratio	1	0.082368	0.100328	0.4116533	1.08585568	0.8920124	1.321823	0.995185
PLSCR2	Wald ratio	1	-0.24704	0.301082	0.411923824	0.78110843	0.4329378	1.4092796	0.995185
PLA2G7	Inverse variance weighted	4	-0.02007	0.024514	0.413022053	0.98013321	0.9341539	1.0283756	0.995185
RNF39	Inverse variance weighted	4	0.022329	0.0273	0.413424733	1.02257969	0.9693007	1.0787872	0.995185
RNASET2	Inverse variance weighted	6	0.026851	0.03284	0.413566476	1.02721514	0.9631793	1.0955084	0.995185
PMEL	Wald ratio	1	-0.08963	0.109839	0.414500136	0.91427096	0.7371883	1.1338913	0.995185
SLC25A36	Wald ratio	1	-0.10754	0.131801	0.414557571	0.89804378	0.6935953	1.1627568	0.995185
KLHL2	Inverse variance weighted	2	0.097286	0.119457	0.415413366	1.10217559	0.8721019	1.3929463	0.995185
SLC37A1	Inverse variance weighted	4	0.049625	0.060962	0.415631949	1.05087679	0.9325228	1.184252	0.995185
FCGRT	Inverse variance weighted	3	0.031748	0.039002	0.415640472	1.03225724	0.9562882	1.1142614	0.995185
IGHA2	Wald ratio	1	0.092489	0.113629	0.415671597	1.0969011	0.8778983	1.3705369	0.995185
ITPR1	Inverse variance weighted	3	-0.03183	0.039119	0.415867954	0.96867316	0.897177	1.0458669	0.995185
KLHL3	Inverse variance weighted	3	0.035306	0.043408	0.416011009	1.03593665	0.9514456	1.1279307	0.995185
VKORC1	Wald ratio	1	0.073266	0.090109	0.416169835	1.07601665	0.9018139	1.28387	0.995185
PPWD1	Inverse variance weighted	4	-0.02854	0.035123	0.416439607	0.97186196	0.9072087	1.0411228	0.995185
BIRC2	Wald ratio	1	0.166508	0.205012	0.416686781	1.18117241	0.7903205	1.7653196	0.995185
TRIO	Inverse variance weighted	2	0.0737	0.090751	0.416724802	1.076484	0.9010709	1.286045	0.995185
CAD	Inverse variance weighted	2	0.066983	0.082518	0.416941243	1.06927706	0.9095987	1.2569866	0.995185
ZAP70	Inverse variance weighted	2	0.035311	0.04353	0.417250307	1.03594235	0.9512227	1.1282075	0.995185
ST3GAL2	Inverse variance weighted	2	-0.08398	0.103532	0.417286425	0.91945074	0.7505855	1.1263069	0.995185
PPIA	Wald ratio	1	0.037641	0.046435	0.417591935	1.03835801	0.948027	1.1372961	0.995185
GGPS1	Inverse variance weighted	4	-0.03761	0.046454	0.418158306	0.96308857	0.8792736	1.0548931	0.995185
DUOX2	Wald ratio	1	0.194642	0.24044	0.418213913	1.21487579	0.7583423	1.9462493	0.995185
TDP2	Inverse variance weighted	2	0.157391	0.194426	0.418216682	1.17045363	0.7995684	1.7133765	0.995185
RNF135	Wald ratio	1	-0.09027	0.111583	0.418512792	0.91368368	0.7342006	1.1370432	0.995185
SSR2	Wald ratio	1	-0.2282	0.282185	0.41869023	0.79596377	0.4578179	1.3838654	0.995185
DHRS12	Inverse variance weighted	3	-0.03058	0.037836	0.418972815	0.96988348	0.9005603	1.044543	0.995185
AGBL2	Wald ratio	1	0.112488	0.139366	0.419584183	1.11905935	0.8515736	1.4705645	0.995185
IL16	Inverse variance weighted	6	-0.03164	0.039207	0.419628655	0.96885269	0.8971891	1.0462404	0.995185
FBN1	Wald ratio	1	-0.04275	0.052979	0.419656376	0.95814623	0.863645	1.0629879	0.995185
IARS2	Inverse variance weighted	2	-0.11745	0.145936	0.420922776	0.88918294	0.667987	1.1836253	0.995761
ENDOD1	Inverse variance weighted	4	-0.04177	0.051912	0.421041038	0.95909118	0.8663066	1.0618133	0.995761
CISD1	Inverse variance weighted	6	-0.02369	0.029457	0.421317288	0.97659107	0.9218042	1.0346342	0.995761
GYPE	Inverse variance weighted	3	-0.01238	0.015402	0.421579739	0.98769786	0.9583266	1.0179693	0.995761
ABCC10	Inverse variance weighted	2	0.084524	0.10528	0.422064398	1.08819853	0.8853029	1.3375942	0.995761
JUN	Inverse variance weighted	4	-0.04494	0.055998	0.422279062	0.956058	0.8566788	1.0669657	0.995761
C19orf10	Inverse variance weighted	2	0.09005	0.112262	0.422471057	1.09422942	0.8781094	1.3635408	0.995761
ICOS	Inverse variance weighted	3	-0.14348	0.178911	0.422571621	0.86633774	0.6100923	1.2302091	0.995761
TRPV1	Wald ratio	1	0.108731	0.135608	0.422667616	1.11486219	0.8546519	1.454297	0.995761
SLC25A25	Wald ratio	1	-0.07042	0.088184	0.424515943	0.93199824	0.7840644	1.1078436	0.995904
FKBP1B	Wald ratio	1	-0.04596	0.057552	0.424515943	0.95507866	0.8531983	1.0691245	0.995904
GGH	Inverse variance weighted	4	0.035151	0.044018	0.424547195	1.03577583	0.9501607	1.1291054	0.995904
CHID1	Wald ratio	1	-0.13852	0.173573	0.424825852	0.87064193	0.6195714	1.2234544	0.995904
CCR3	Inverse variance weighted	5	-0.0394	0.04938	0.424893328	0.96136277	0.872678	1.05906	0.995904
SCNN1D	Wald ratio	1	0.087651	0.110126	0.426079485	1.09160751	0.8796811	1.3545896	0.995904
RXRA	Inverse variance weighted	2	-0.09904	0.124486	0.426268892	0.90570637	0.7096151	1.1559845	0.995904
GYPC	Inverse variance weighted	3	-0.04101	0.051553	0.426297447	0.95981725	0.867573	1.0618693	0.995904
RAMP1	Inverse variance weighted	3	-0.0348	0.043748	0.426324817	0.96579708	0.8864345	1.0522649	0.995904

SLC10A1	Wald ratio	1	-0.21109	0.265419	0.42642643	0.80969862	0.4812763	1.3622358	0.995904
FAM20A	Inverse variance weighted	2	0.101975	0.12829	0.426681242	1.10735634	0.861162	1.4239342	0.995904
ACACA	Wald ratio	1	0.086584	0.108946	0.426766597	1.09044262	0.8807767	1.3500188	0.995904
CSNK2A1	Wald ratio	1	0.143046	0.180131	0.427126945	1.15378236	0.8105745	1.642309	0.995904
CD207	Inverse variance weighted	2	0.098643	0.124442	0.427959034	1.10367271	0.8647957	1.4085332	0.995904
HDAC3	Wald ratio	1	-0.06794	0.085731	0.428096685	0.93431846	0.7898035	1.1052762	0.995904
GNS	Inverse variance weighted	2	0.043621	0.055056	0.42818137	1.04458638	0.9377348	1.1636133	0.995904
TNFRSF9	Inverse variance weighted	4	-0.06565	0.082881	0.428282848	0.93645604	0.7960453	1.1016332	0.995904
MICAL2	Inverse variance weighted	3	-0.05152	0.065066	0.428501788	0.94978814	0.8360681	1.0789761	0.995904
PLXNB1	Inverse variance weighted	2	0.110098	0.13911	0.428687251	1.11638692	0.8499661	1.466317	0.995904
PRKD3	Inverse variance weighted	4	0.026203	0.033122	0.42888186	1.02654923	0.962024	1.0954024	0.995904
PTPRS	Wald ratio	1	-0.08765	0.110927	0.429455672	0.91608503	0.7370768	1.1385676	0.996522
CCL8	Wald ratio	1	-0.17738	0.224825	0.430117369	0.83745755	0.5389991	1.3011805	0.997342
VASH1	Inverse variance weighted	7	0.011931	0.015139	0.43064655	1.01200254	0.9824145	1.0424817	0.997347
ALOX5	Inverse variance weighted	4	-0.03089	0.039211	0.430783352	0.9695798	0.8978555	1.0470337	0.997347
CTSG	Inverse variance weighted	5	0.025431	0.032297	0.431047538	1.02575715	0.9628362	1.09279	0.997347
MAP3K7	Wald ratio	1	0.052154	0.066377	0.432034892	1.05353762	0.9250144	1.1999182	0.997347
TRIM16L	Inverse variance weighted	2	0.023405	0.029801	0.432240187	1.02368067	0.9656003	1.0852546	0.997347
ITGA6	Inverse variance weighted	3	-0.11917	0.15182	0.432490548	0.88765746	0.6591942	1.1953015	0.997347
DPYD	Inverse variance weighted	4	0.019154	0.024409	0.432617871	1.01933894	0.97172	1.0692914	0.997347
CCR8	Inverse variance weighted	3	0.063797	0.081322	0.432742433	1.06587644	0.9088339	1.2500553	0.997347
MBTPS1	Inverse variance weighted	5	-0.02554	0.032591	0.433293257	0.97478629	0.9144662	1.0390852	0.997347
UQCRC2	Wald ratio	1	-0.08027	0.102521	0.433658978	0.92286849	0.7548697	1.1282559	0.997347
HSD3B7	Inverse variance weighted	3	0.039609	0.050635	0.434065657	1.04040441	0.942108	1.1489567	0.997347
GRN	Inverse variance weighted	3	-0.03834	0.049166	0.435455383	0.96238184	0.8739702	1.0597374	0.997347
OR52K2	Inverse variance weighted	3	-0.02469	0.031661	0.43549601	0.97561258	0.9169111	1.0380722	0.997347
RIOK3	Inverse variance weighted	2	-0.1865	0.239402	0.43596204	0.8298572	0.5190629	1.3267429	0.997347
CYP4F12	Inverse variance weighted	4	0.029284	0.037626	0.436395431	1.02971723	0.956511	1.1085263	0.997347
SLC12A7	Inverse variance weighted	5	-0.02246	0.028894	0.43697604	0.97779082	0.9239553	1.0347631	0.997347
GNPTG	Inverse variance weighted	2	0.037758	0.048636	0.437551612	1.0384796	0.9440571	1.1423461	0.997347
GALNS	Inverse variance weighted	4	0.032928	0.042436	0.437776979	1.03347606	0.9509958	1.1231099	0.997347
TRIM36	Inverse variance weighted	3	-0.03298	0.042505	0.437818997	0.9675587	0.8902172	1.0516196	0.997347
LTB4R	Inverse variance weighted	2	-0.05411	0.069752	0.437909765	0.94732946	0.8262799	1.0861127	0.997347
ITGB5	Inverse variance weighted	3	0.05103	0.065845	0.438336288	1.05235456	0.9249406	1.1973202	0.997347
BMP3	Wald ratio	1	-0.21534	0.278008	0.438595874	0.80627079	0.4675583	1.3903561	0.997347
KLHL23	Wald ratio	1	0.080956	0.104526	0.438629373	1.08432357	0.8834554	1.3308624	0.997347
CYP4B1	Wald ratio	1	-0.10806	0.139572	0.438816343	0.89757748	0.6827564	1.1799895	0.997347
FASN	Inverse variance weighted	3	-0.02221	0.028713	0.439201391	0.97803442	0.924514	1.0346532	0.997347
IMPDH2	Wald ratio	1	0.152719	0.197465	0.439286273	1.16499809	0.7911146	1.7155803	0.997347
STK32B	Inverse variance weighted	3	-0.05111	0.06615	0.439750656	0.95017539	0.8346329	1.0817131	0.997347
FUCA2	Inverse variance weighted	2	-0.05029	0.06512	0.43998733	0.95095702	0.8370085	1.0804183	0.997347
CYP2J2	Wald ratio	1	-0.05169	0.066975	0.44026895	0.94962558	0.8328021	1.0828367	0.997347
ANPEP	Inverse variance weighted	8	-0.0123	0.015945	0.440367502	0.98777269	0.9573801	1.0191301	0.997347
GDF10	Inverse variance weighted	2	0.07182	0.093085	0.44038008	1.07446176	0.8952729	1.2895152	0.997347
PDXDC1	Inverse variance weighted	2	-0.09096	0.117947	0.440571929	0.91305049	0.7245964	1.150518	0.997347
SPOCK2	Inverse variance weighted	3	0.038793	0.050301	0.440583896	1.03955504	0.9419549	1.1472679	0.997347
GPR3	Wald ratio	1	-0.17879	0.231946	0.440805727	0.8362799	0.5307806	1.3176143	0.997347
CR2	Wald ratio	1	-0.07143	0.092711	0.441008215	0.93105844	0.7763536	1.1165915	0.997347
PARP4	Inverse variance weighted	4	-0.02455	0.031916	0.441787901	0.97574951	0.9165804	1.0387383	0.997347
MAN2B1	Inverse variance weighted	5	0.019814	0.025772	0.442009017	1.02001154	0.9697665	1.0728598	0.997347
SPSB2	Inverse variance weighted	4	-0.04902	0.063781	0.442168118	0.95216405	0.8402731	1.0789545	0.997347
SERPINA1	Inverse variance weighted	5	-0.02401	0.031284	0.442727283	0.97627253	0.918209	1.0380077	0.997347
COL13A1	Wald ratio	1	-0.08427	0.109876	0.443087288	0.91917978	0.7410925	1.1400621	0.997347
TGM3	Inverse variance weighted	3	-0.02656	0.034634	0.44317842	0.97379069	0.9098803	1.0421901	0.997347
RIPK4	Inverse variance weighted	2	0.069134	0.090165	0.443230644	1.07157992	0.8979959	1.278718	0.997347
CHRFAM7A	Inverse variance weighted	3	0.044793	0.058469	0.44361312	1.04581183	0.9325749	1.1727984	0.997347
IL18R1	Inverse variance weighted	4	0.029992	0.039179	0.443967302	1.03044595	0.9542797	1.1126914	0.997347
DHRS4L2	Inverse variance weighted	2	-0.04384	0.057276	0.443984437	0.95710363	0.8554701	1.0708117	0.997347
SLC25A26	Inverse variance weighted	2	0.070985	0.092839	0.444506257	1.0735652	0.8949576	1.2878177	0.997827
PYROXD1	Inverse variance weighted	3	0.030248	0.039635	0.445365235	1.03071	0.9536712	1.1139721	0.998617
GNRHR2	Wald ratio	1	-0.04298	0.056393	0.445937827	0.95792782	0.8576899	1.0698806	0.998617
AIFM2	Inverse variance weighted	3	0.035081	0.046051	0.446183032	1.03570387	0.9463163	1.1335349	0.998617
MSLN	Inverse variance weighted	3	-0.04378	0.057487	0.446321276	0.95716426	0.8551695	1.0713238	0.998617

NTNG1	Wald ratio	1	-0.03703	0.048663	0.446683244	0.96364665	0.8759813	1.0600853	0.998617
SPRYD7	Wald ratio	1	-0.08119	0.106705	0.446734979	0.92202001	0.748017	1.1364994	0.998617
IL12A	Wald ratio	1	-0.12702	0.167131	0.447254585	0.88071634	0.6347039	1.2220837	0.998617
RIPK1	Inverse variance weighted	3	0.059526	0.078395	0.447670065	1.06133316	0.9101657	1.2376077	0.998617
CAMK1D	Inverse variance weighted	8	0.023424	0.030854	0.447726665	1.02370087	0.9636292	1.0875173	0.998617
DUT	Inverse variance weighted	4	-0.03048	0.040181	0.448127177	0.96998078	0.8965206	1.0494602	0.998617
NEIL3	Wald ratio	1	0.213603	0.281674	0.448251457	1.23813049	0.7128545	2.1504628	0.998617
OR10AA1P	Inverse variance weighted	3	-0.02998	0.039688	0.449970162	0.97046182	0.8978318	1.0489673	0.99882
ANGPT2	Inverse variance weighted	3	-0.09199	0.12177	0.450001115	0.91211716	0.7184519	1.1579866	0.99882
BCL2L2	Inverse variance weighted	2	0.105559	0.139748	0.450038887	1.11133151	0.84506	1.4615031	0.99882
CSNK1D	Inverse variance weighted	3	0.044505	0.058923	0.450062587	1.04551034	0.9314769	1.1735039	0.99882
CACNA2D4	Wald ratio	1	-0.09338	0.123732	0.450418815	0.91084518	0.7146972	1.1608257	0.99882
BRSK1	Inverse variance weighted	2	0.119378	0.15819	0.450456613	1.12679619	0.8264027	1.5363814	0.99882
EDN3	Inverse variance weighted	2	-0.0323	0.042805	0.450524911	0.96821787	0.8903007	1.0529542	0.99882
JAK2	Inverse variance weighted	2	-0.03379	0.044862	0.451332228	0.96677451	0.8853964	1.0556322	0.99882
PSAP	Inverse variance weighted	2	-0.06078	0.080731	0.451502767	0.94102734	0.8033095	1.1023552	0.99882
IGLV3-16	Wald ratio	1	-0.04545	0.060438	0.4520113	0.95556438	0.8488176	1.0757356	0.99882
IGLV4-3	Wald ratio	1	-0.03467	0.046097	0.4520113	0.96592578	0.88248	1.057262	0.99882
SNAP29	Inverse variance weighted	4	-0.0171	0.022744	0.452045669	0.983042	0.9401828	1.027855	0.99882
AKR7A3	Inverse variance weighted	3	0.080254	0.106848	0.452589354	1.08356223	0.8788265	1.3359942	0.999221
DPEP3	Inverse variance weighted	4	-0.02923	0.038982	0.453315511	0.97119059	0.899751	1.0483024	0.999221
TPSB2	Inverse variance weighted	4	0.012932	0.017266	0.45385779	1.01301645	0.9793075	1.0478856	0.999221
NR4A1	Wald ratio	1	-0.18042	0.2412	0.45444116	0.8349154	0.5203904	1.33954	0.999221
GPR83	Inverse variance weighted	2	-0.0291	0.039	0.45561713	0.97132215	0.8998414	1.0484811	0.999221
PDGFD	Inverse variance weighted	3	-0.03607	0.048384	0.455950337	0.96457134	0.8773026	1.060521	0.999221
LMAN1	Inverse variance weighted	2	0.02106	0.028248	0.455951663	1.02128333	0.966275	1.0794232	0.999221
TBCK	Inverse variance weighted	3	0.024654	0.033166	0.457263243	1.02496053	0.9604525	1.0938012	0.999221
FKBP15	Inverse variance weighted	4	0.035893	0.048377	0.458126387	1.03654465	0.9427761	1.1396394	0.999221
FCGBP	Inverse variance weighted	6	0.016197	0.021844	0.45838486	1.01632911	0.9737349	1.0607866	0.999221
TFRC	Inverse variance weighted	4	0.026404	0.035649	0.458891396	1.02675566	0.9574639	1.1010621	0.999221
PI16	Inverse variance weighted	7	-0.02049	0.027668	0.45905302	0.97972256	0.9280072	1.0343199	0.999221
TXNDC16	Inverse variance weighted	2	0.04787	0.064658	0.459081992	1.04903464	0.9241694	1.1907705	0.999221
LY75	Inverse variance weighted	5	-0.0303	0.040933	0.45909139	0.97014987	0.8953559	1.0511918	0.999221
CAPNS1	Inverse variance weighted	4	-0.03584	0.048501	0.459903941	0.96479236	0.877302	1.0610079	0.999221
TRIM9	Inverse variance weighted	3	0.03034	0.041188	0.461344764	1.0308054	0.9508605	1.1174717	0.999221
RFPL4A	Inverse variance weighted	4	0.020251	0.027496	0.461430615	1.02045709	0.966918	1.0769607	0.999221
POLR1C	Wald ratio	1	-0.12089	0.164271	0.461793118	0.88613454	0.6421988	1.2227279	0.999221
MGEA5	Wald ratio	1	0.15174	0.20621	0.461822563	1.16385728	0.7769085	1.7435305	0.999221
SLC25A17	Wald ratio	1	0.056866	0.077338	0.46216039	1.05851388	0.9096313	1.2317646	0.999221
HSPB1	Wald ratio	1	-0.06918	0.094123	0.462313468	0.93315445	0.775951	1.1222065	0.999221
C1QB	Inverse variance weighted	4	0.025514	0.03472	0.462436076	1.02584183	0.9583547	1.0980814	0.999221
PDK2	Inverse variance weighted	2	0.04416	0.060129	0.462696918	1.04514922	0.9289563	1.1758754	0.999221
IL4R	Inverse variance weighted	3	-0.03116	0.042449	0.462969896	0.96932406	0.8919396	1.0534224	0.999221
GNAS	Inverse variance weighted	3	-0.06108	0.083291	0.463388712	0.94075242	0.7990553	1.1075768	0.999221
ADAM15	Inverse variance weighted	6	0.020132	0.027455	0.463393087	1.02033562	0.9668816	1.0767449	0.999221
GPR135	Wald ratio	1	0.181166	0.247331	0.463873158	1.19861439	0.7381538	1.9463104	0.999221
MTR	Inverse variance weighted	2	0.065477	0.089428	0.464057461	1.06766847	0.8960126	1.2722097	0.999221
ALDH18A1	Inverse variance weighted	2	-0.05944	0.081208	0.464174334	0.94228846	0.8036331	1.1048668	0.999221
DPP9	Inverse variance weighted	2	-0.03052	0.041705	0.464267931	0.96993945	0.8938079	1.0525556	0.999221
F5	Inverse variance weighted	4	0.014111	0.019284	0.464303172	1.01421138	0.9765938	1.053278	0.999221
PDK4	Inverse variance weighted	3	-0.0416	0.056845	0.464334314	0.9592579	0.8581194	1.0723167	0.999221
COL9A3	Inverse variance weighted	3	0.01996	0.02729	0.464535473	1.0201606	0.967027	1.0762136	0.999221
SUMF1	Inverse variance weighted	7	-0.01979	0.027064	0.464712993	0.98040745	0.9297558	1.0338185	0.999221
TEK	Inverse variance weighted	4	-0.01425	0.019507	0.464967891	0.98584757	0.9488666	1.0242699	0.999221
NR1H3	Inverse variance weighted	8	-0.02675	0.036724	0.466420672	0.97360792	0.9059914	1.0462709	0.999221
ITLN1	Inverse variance weighted	3	0.018633	0.025602	0.466734372	1.01880792	0.9689456	1.0712361	0.999221
IGF2	Inverse variance weighted	2	0.07449	0.102391	0.466919477	1.07733437	0.8814408	1.3167638	0.999221
TMX3	Inverse variance weighted	2	0.037877	0.052081	0.467062373	1.03860311	0.9378158	1.1502221	0.999221
EMID1	Inverse variance weighted	2	-0.0894	0.122967	0.467209663	0.91447964	0.7186256	1.1637118	0.999221
INPP5K	Inverse variance weighted	4	-0.02269	0.031242	0.467638305	0.97756378	0.9194996	1.0392945	0.999221
CSF3R	Inverse variance weighted	3	0.0716	0.098623	0.467841121	1.07422583	0.8854124	1.3033035	0.999221
SLC25A24	Inverse variance weighted	4	-0.01917	0.026412	0.468052645	0.98101651	0.9315236	1.033139	0.999221
MRGPRE	Inverse variance weighted	2	-0.04144	0.057104	0.468062794	0.95941029	0.857821	1.0730305	0.999221

SLC7A11	Inverse variance weighted	2	0.110459	0.152246	0.46812723	1.1167909	0.8286613	1.5051045	0.999221
PARP8	Inverse variance weighted	2	0.090685	0.125218	0.468933555	1.09492405	0.8566354	1.399497	0.999221
ICAM2	Inverse variance weighted	3	-0.03508	0.048505	0.469549547	0.96552906	0.8779655	1.0618258	0.999221
GLRX3	Inverse variance weighted	2	0.082239	0.113805	0.469905158	1.08571556	0.868647	1.357028	0.999221
PTPN18	Inverse variance weighted	2	-0.17702	0.245076	0.470104881	0.83776257	0.5182126	1.3543595	0.999221
HPD	Inverse variance weighted	3	-0.02093	0.02898	0.470259028	0.9792919	0.9252169	1.0365274	0.999221
PDCD4	Inverse variance weighted	3	0.034648	0.048053	0.470880256	1.03525548	0.9422026	1.1374983	0.999221
SMO	Inverse variance weighted	3	-0.07971	0.110791	0.471861051	0.9233849	0.7431483	1.1473345	0.999221
IFI30	Inverse variance weighted	2	-0.02088	0.029055	0.472415608	0.97933905	0.9251267	1.0367282	0.999221
MAPK12	Inverse variance weighted	2	0.118748	0.165327	0.472597289	1.12608563	0.8144086	1.5570425	0.999221
C4BPA	Inverse variance weighted	4	-0.007	0.009746	0.472792143	0.99302769	0.9742397	1.012178	0.999221
LCAT	Inverse variance weighted	2	0.03336	0.04656	0.473687457	1.03392263	0.9437464	1.1327154	0.999221
MAN2A1	Inverse variance weighted	3	-0.04992	0.069669	0.473706749	0.95130991	0.8298856	1.0905003	0.999221
PSMA2	Inverse variance weighted	3	0.026505	0.036997	0.473732406	1.02685951	0.955034	1.1040868	0.999221
CRHBP	Wald ratio	1	0.190936	0.266787	0.474185088	1.21038198	0.7175109	2.0418149	0.999221
NCOR2	Inverse variance weighted	3	-0.05409	0.075676	0.474734431	0.94734379	0.8167528	1.0988151	0.999221
CYP21A2	Inverse variance weighted	4	0.030745	0.043018	0.474804403	1.03122201	0.9478382	1.1219413	0.999221
CGREF1	Wald ratio	1	-0.1121	0.156935	0.475050524	0.89395775	0.6572504	1.2159148	0.999221
VIPR2	Inverse variance weighted	2	0.083453	0.116863	0.475158311	1.08703409	0.8645053	1.3668432	0.999221
PLA2G12A	Inverse variance weighted	2	-0.0454	0.0636	0.475301713	0.9556127	0.8436158	1.0824781	0.999221
SCUBE1	Wald ratio	1	0.175048	0.245553	0.475925408	1.19130338	0.7362123	1.9277098	0.999221
HTR3A	Wald ratio	1	0.075097	0.105424	0.476261214	1.07798857	0.8767485	1.3254192	0.999221
LAMC3	Inverse variance weighted	2	-0.10419	0.146322	0.476418495	0.90105203	0.6763912	1.200333	0.999221
ALOX15	Inverse variance weighted	3	0.017757	0.024944	0.476546221	1.0179151	0.9693468	1.0689168	0.999221
CHRNE	Wald ratio	1	0.03514	0.04937	0.476610492	1.03576463	0.9402349	1.1410004	0.999221
FSD1	Wald ratio	1	0.050017	0.070294	0.476750626	1.05128869	0.9159815	1.2065832	0.999221
PIK3CG	Wald ratio	1	-0.07101	0.099798	0.476750626	0.93145216	0.7659676	1.1326891	0.999221
ADORA2A	Wald ratio	1	-0.05296	0.074424	0.476750626	0.94842238	0.8196927	1.0973686	0.999221
PCYOX1L	Inverse variance weighted	5	-0.02356	0.033134	0.47696892	0.97671086	0.915296	1.0422465	0.999221
CACNA2D2	Wald ratio	1	0.079316	0.111605	0.477275227	1.08254684	0.8698552	1.3472445	0.999221
PIGF	Inverse variance weighted	2	0.051834	0.072981	0.477554817	1.0532009	0.9128273	1.2151609	0.999221
AKR1C3	Inverse variance weighted	4	-0.04348	0.061312	0.478177208	0.95744699	0.8490333	1.0797041	0.999221
ALB	Wald ratio	1	-0.09071	0.127899	0.478188052	0.91328399	0.7107816	1.1734796	0.999221
HCN3	Wald ratio	1	-0.06294	0.088898	0.47896724	0.93900322	0.7888527	1.1177335	0.999221
ZP3	Inverse variance weighted	4	0.059501	0.084174	0.479635458	1.06130733	0.8998934	1.2516741	0.999221
XCR1	Wald ratio	1	0.053419	0.075627	0.479971	1.05487144	0.9095461	1.2234165	0.999221
SELPLG	Inverse variance weighted	3	-0.05659	0.080164	0.480245853	0.94498295	0.8075826	1.1057603	0.999221
CD34	Inverse variance weighted	2	-0.07593	0.107605	0.480416435	0.92688174	0.7506362	1.1445088	0.999221
CACNA1E	Inverse variance weighted	3	-0.03848	0.054589	0.480815369	0.96224617	0.8646073	1.0709113	0.999221
HLA-C	Inverse variance weighted	8	-0.0164	0.023264	0.480863702	0.98373468	0.9398864	1.0296286	0.999221
KCNMA1	Inverse variance weighted	3	-0.01991	0.028339	0.482289761	0.98028537	0.9273209	1.036275	0.999221
AGBL3	Wald ratio	1	0.17396	0.247607	0.48232744	1.1900078	0.7324574	1.93338	0.999221
CD82	Inverse variance weighted	4	-0.05853	0.083318	0.482386945	0.94315186	0.8010512	1.1104601	0.999221
A1BG	Wald ratio	1	0.192461	0.274111	0.482599554	1.21222912	0.7083644	2.0744965	0.999221
CHEK1	Wald ratio	1	0.095157	0.13565	0.48299569	1.09983194	0.8430606	1.4348082	0.999221
PROC	Wald ratio	1	-0.0427	0.060914	0.483343843	0.95820221	0.8503671	1.0797119	0.999221
CNP	Inverse variance weighted	2	0.025572	0.036495	0.483486631	1.02590222	0.9550818	1.101974	0.999221
GPR162	Inverse variance weighted	4	0.019203	0.027411	0.483582707	1.01938825	0.9660666	1.0756529	0.999221
TRIM26	Inverse variance weighted	4	0.057402	0.081945	0.483617396	1.0590817	0.9019375	1.2436051	0.999221
CDKL1	Inverse variance weighted	2	0.015589	0.022275	0.484035373	1.01571076	0.97232	1.0610379	0.999221
BCAT1	Inverse variance weighted	6	-0.01755	0.02511	0.484614133	0.98260381	0.9354154	1.0321727	0.999221
SDC4	Wald ratio	1	-0.07948	0.113848	0.485106399	0.92359773	0.7388795	1.154495	0.999221
APH1B	Inverse variance weighted	4	-0.03946	0.056589	0.485622043	0.96130919	0.8603857	1.074071	0.999221
PSMA5	Wald ratio	1	-0.13606	0.195222	0.485821758	0.87278714	0.5952951	1.2796299	0.999221
LAD1	Wald ratio	1	0.101598	0.145849	0.48605194	1.10693881	0.8317156	1.4732363	0.999221
C1QC	Inverse variance weighted	3	0.04183	0.060104	0.486447542	1.04271756	0.9268414	1.1730808	0.999221
CXCR5	Wald ratio	1	0.135447	0.1948	0.486859482	1.14504862	0.7816396	1.6774181	0.999221
SLC7A9	Inverse variance weighted	4	-0.02526	0.036341	0.48692189	0.97505196	0.9080169	1.0470359	0.999221
CRELD2	Inverse variance weighted	5	-0.02248	0.032348	0.487034348	0.977768	0.9177	1.0417677	0.999221
FGR	Wald ratio	1	0.071376	0.1027	0.487055302	1.07398537	0.8781698	1.3134641	0.999221
TRIM4	Inverse variance weighted	4	-0.0202	0.029069	0.487066199	0.98000034	0.9257257	1.0374571	0.999221
SLC16A14	Wald ratio	1	0.189164	0.272487	0.487547465	1.20823933	0.7082845	2.0610958	0.999221
VAMP8	Inverse variance weighted	4	0.022173	0.031951	0.487693399	1.0224208	0.9603571	1.0884954	0.999221

SIK3	Inverse variance weighted	2	-0.06938	0.100019	0.48790967	0.93297513	0.7668888	1.135031	0.999221
BLK	Inverse variance weighted	4	-0.02743	0.039661	0.489155067	0.97294095	0.9001732	1.051591	0.999221
RXFP2	Inverse variance weighted	3	-0.04701	0.067997	0.489354037	0.95407924	0.8350348	1.090095	0.999221
SFRP5	Wald ratio	1	-0.04826	0.069953	0.490242938	0.95288405	0.8307971	1.0929118	0.999221
EGFL8	Inverse variance weighted	2	-0.05593	0.081101	0.490421819	0.94560488	0.8066311	1.1085223	0.999221
TSPO	Inverse variance weighted	4	-0.02433	0.035279	0.490477246	0.97596702	0.9107627	1.0458395	0.999221
PTGR2	Inverse variance weighted	4	-0.02609	0.037883	0.491050671	0.97424953	0.9045309	1.0493419	0.999221
TMPRSS11E	Wald ratio	1	-0.15029	0.218243	0.491060698	0.86046098	0.5609951	1.3197852	0.999221
TLR4	Inverse variance weighted	6	-0.02367	0.034422	0.491650674	0.97660624	0.9128904	1.0447692	0.999221
TRPC6	Inverse variance weighted	3	0.052775	0.076746	0.491669521	1.05419246	0.9069684	1.2253147	0.999221
CD80	Inverse variance weighted	2	0.127457	0.185359	0.491691677	1.1359357	0.7899015	1.633558	0.999221
PI4KA	Wald ratio	1	-0.0705	0.102736	0.49254002	0.93192318	0.7619557	1.1398049	0.999221
ITGB8	Wald ratio	1	0.190035	0.276908	0.49254002	1.20929208	0.7027843	2.0808479	0.999221
MALT1	Inverse variance weighted	2	0.032265	0.047216	0.494388596	1.03279111	0.9415021	1.1329316	0.999221
VIPR1	Inverse variance weighted	8	-0.04206	0.061581	0.494647627	0.95881619	0.8497996	1.081818	0.999221
TNFRSF10A	Inverse variance weighted	4	0.026709	0.039125	0.494821313	1.02706894	0.9512524	1.1089282	0.999221
SYK	Inverse variance weighted	5	-0.02251	0.032985	0.494887165	0.97773737	0.9165259	1.0430369	0.999221
AZU1	Wald ratio	1	-0.17586	0.25765	0.494897759	0.8387387	0.5061869	1.3897684	0.999221
DPP8	Inverse variance weighted	2	0.04726	0.069343	0.495524249	1.04839501	0.9151647	1.2010211	0.999221
COL10A1	Wald ratio	1	0.064257	0.094434	0.496225413	1.06636638	0.8861808	1.2831888	0.999221
LTBP4	Inverse variance weighted	3	-0.02818	0.041506	0.497242595	0.97221725	0.8962566	1.0546158	0.999221
ITIH1	Wald ratio	1	0.18342	0.270217	0.497272275	1.20131911	0.707367	2.0401964	0.999221
TMED1	Inverse variance weighted	3	-0.02815	0.04149	0.497490092	0.97224368	0.8963094	1.0546111	0.999221
PLXNC1	Inverse variance weighted	3	0.042398	0.062573	0.498036146	1.04331001	0.9228906	1.1794419	0.999221
EPHA10	Wald ratio	1	-0.14763	0.218016	0.498301047	0.86274798	0.5627367	1.3227039	0.999221
TUBB4A	Inverse variance weighted	2	-0.01491	0.022014	0.498304069	0.98520327	0.9435976	1.0286435	0.999221
CR1	Inverse variance weighted	5	0.039766	0.058733	0.498368541	1.04056694	0.9274179	1.1675206	0.999221
AGGF1	Inverse variance weighted	2	0.078237	0.115696	0.498897944	1.08137851	0.861976	1.3566265	0.999221
EFNA5	Inverse variance weighted	2	0.087676	0.129654	0.498898081	1.09163388	0.8466675	1.4074764	0.999221
SORD	Inverse variance weighted	2	0.071143	0.105298	0.499269384	1.07373521	0.8735059	1.3198621	0.999221
AHCYL2	Inverse variance weighted	2	-0.07832	0.116003	0.499566644	0.9246666	0.7366163	1.1607242	0.999221
LRRN3	Inverse variance weighted	3	0.063423	0.094032	0.500007066	1.065477	0.8861401	1.281108	0.999221
RPN1	Inverse variance weighted	3	0.058735	0.0871	0.500096297	1.06049419	0.8940608	1.2579098	0.999221
PSPN	Wald ratio	1	-0.13254	0.196644	0.500290239	0.87586364	0.5957299	1.2877264	0.999221
CACNB4	Inverse variance weighted	3	-0.07557	0.112205	0.50065015	0.92721792	0.7441674	1.1552953	0.999221
PECR	Inverse variance weighted	2	0.04844	0.071993	0.501048035	1.04963228	0.9114975	1.2087009	0.999221
BMP2	Inverse variance weighted	2	-0.03499	0.05207	0.501557646	0.96561223	0.8719271	1.0693635	0.999221
PASK	Inverse variance weighted	5	-0.01446	0.021623	0.503707395	0.98564529	0.944745	1.0283162	0.999221
CD14	Inverse variance weighted	4	-0.02458	0.036756	0.503725218	0.97572317	0.9079031	1.0486094	0.999221
STK32C	Inverse variance weighted	5	-0.01853	0.027773	0.504761858	0.98164542	0.9296376	1.0365628	0.999221
SSC5D	Inverse variance weighted	2	-0.03818	0.057247	0.504809077	0.96253925	0.860377	1.0768323	0.999221
STK3	Wald ratio	1	0.136887	0.205331	0.504985075	1.14669909	0.7667752	1.7148688	0.999221
SLC22A4	Inverse variance weighted	5	-0.02468	0.037031	0.505104632	0.97562187	0.9073198	1.0490657	0.999221
KIR3DL2	Inverse variance weighted	2	-0.03419	0.051344	0.505505559	0.96639052	0.8738725	1.0687035	0.999221
HPGD	Inverse variance weighted	4	0.021069	0.031663	0.505796548	1.02129219	0.9598374	1.0866817	0.999221
EDN1	Inverse variance weighted	2	0.059071	0.088844	0.506122944	1.06085077	0.89131	1.2626408	0.999221
PRSS50	Wald ratio	1	0.079533	0.119685	0.506360029	1.08278084	0.8563724	1.3690473	0.999221
RYR1	Inverse variance weighted	2	0.030449	0.045835	0.506484704	1.03091774	0.9423411	1.1278202	0.999221
STAT6	Inverse variance weighted	4	-0.0259	0.03903	0.506933351	0.97443159	0.902669	1.0518993	0.999221
NEK2	Wald ratio	1	0.177701	0.267878	0.50709596	1.19446871	0.706565	2.0192842	0.999221
SLC9A3	Inverse variance weighted	8	-0.01046	0.015787	0.507783662	0.98959898	0.9594475	1.020698	0.999221
GUCY2C	Inverse variance weighted	2	-0.03091	0.046681	0.50789687	0.96956429	0.8847908	1.0624601	0.999221
SLC25A12	Inverse variance weighted	3	-0.03241	0.049098	0.509243624	0.96811378	0.8792919	1.065908	0.999221
ADAMDEC1	Wald ratio	1	-0.03128	0.0474	0.509253829	0.96920005	0.883213	1.0635586	0.999221
METAP2	Inverse variance weighted	4	0.043133	0.065436	0.509793776	1.04407627	0.9184007	1.1869495	0.999221
CD22	Wald ratio	1	0.045228	0.068746	0.510605773	1.04626597	0.914375	1.1971811	0.999221
CDNF	Wald ratio	1	0.162663	0.247248	0.510605773	1.17663992	0.72474	1.9103148	0.999221
NAGLU	Wald ratio	1	0.039751	0.060443	0.510758136	1.04055176	0.9243005	1.1714242	0.999221
TNFRSF19	Wald ratio	1	0.146638	0.22324	0.511269175	1.15793443	0.7475816	1.7935328	0.999221
FEN1	Wald ratio	1	0.155493	0.236941	0.511663283	1.16823332	0.7342454	1.8587371	0.999221
ENDOU	Wald ratio	1	0.044845	0.068353	0.511769656	1.0458661	0.9147299	1.1958021	0.999221
SCG5	Inverse variance weighted	2	0.109572	0.167104	0.512009746	1.11580036	0.8041643	1.5482041	0.999221
DYRK4	Inverse variance weighted	4	-0.02974	0.045438	0.512749996	0.97069566	0.8879839	1.0611117	0.999221

SIRPA	Inverse variance weighted	5	0.03388	0.051861	0.513566915	1.03446067	0.9344786	1.1451401	0.999221
NEK10	Wald ratio	1	-0.11234	0.172357	0.514539419	0.89374075	0.6375272	1.2529231	0.999221
SDF2	Wald ratio	1	-0.21675	0.332698	0.514733361	0.80513296	0.4194395	1.545489	0.999221
STAT1	Inverse variance weighted	2	0.128549	0.197468	0.515055421	1.13717704	0.7722181	1.6746197	0.999221
PLTP	Inverse variance weighted	6	-0.01953	0.03001	0.515292114	0.98066423	0.9246454	1.0400769	0.999221
ZRANB2	Wald ratio	1	0.083299	0.128057	0.515378706	1.08686709	0.8456136	1.3969501	0.999221
TXNRD3	Wald ratio	1	-0.08387	0.128985	0.515533135	0.9195491	0.714136	1.184047	0.999221
GZMK	Inverse variance weighted	3	0.036331	0.055886	0.515626668	1.03699946	0.9294106	1.1570428	0.999221
KCNQ4	Wald ratio	1	0.122499	0.189154	0.517231374	1.1303185	0.7801715	1.6376142	0.999221
P2RX5	Wald ratio	1	-0.08972	0.138653	0.517593873	0.91419017	0.6966469	1.1996661	0.999221
GSS	Inverse variance weighted	2	0.085949	0.132833	0.517601044	1.08975123	0.8399577	1.4138303	0.999221
IL17B	Wald ratio	1	-0.20156	0.311656	0.517800793	0.81745463	0.4437895	1.505741	0.999221
MFNG	Inverse variance weighted	3	0.035011	0.054171	0.51807978	1.03563127	0.9313091	1.1516392	0.999221
EIF4E	Inverse variance weighted	2	0.024531	0.037967	0.518207554	1.02483432	0.9513392	1.1040072	0.999221
LAMP3	Inverse variance weighted	2	-0.06919	0.10712	0.518347214	0.93315107	0.7564317	1.151156	0.999221
MTAP	Inverse variance weighted	3	0.031866	0.049444	0.519261539	1.03237879	0.9370263	1.1374344	0.999221
MYLK4	Inverse variance weighted	4	-0.01375	0.021344	0.519376503	0.98634205	0.9459304	1.0284802	0.999221
SMPD3	Wald ratio	1	-0.05556	0.086243	0.519425801	0.94595487	0.7988383	1.1201649	0.999221
GANC	Inverse variance weighted	3	-0.06699	0.104014	0.519548124	0.93520494	0.7627251	1.1466887	0.999221
POLB	Inverse variance weighted	2	-0.03914	0.060801	0.519759357	0.96161717	0.8535854	1.0833216	0.999221
CA8	Inverse variance weighted	3	0.034704	0.053914	0.519775928	1.03531348	0.9314916	1.1507071	0.999221
MAST4	Inverse variance weighted	3	-0.01752	0.027226	0.519819807	0.98262931	0.9315677	1.0364897	0.999221
KISS1R	Inverse variance weighted	3	-0.02047	0.031821	0.519979488	0.97973544	0.9204972	1.0427859	0.999221
DBF4	Wald ratio	1	0.157626	0.2451	0.520153024	1.17072819	0.7241408	1.892732	0.999221
ACVR1B	Wald ratio	1	-0.04396	0.068377	0.520316801	0.95699535	0.8369625	1.0942427	0.999221
SLC12A6	Inverse variance weighted	3	-0.0262	0.040864	0.521358951	0.97413625	0.8991572	1.0553677	0.999221
CA13	Inverse variance weighted	2	0.031658	0.049401	0.521622571	1.0321647	0.9369109	1.1371028	0.999221
STAT5A	Wald ratio	1	0.0498	0.077737	0.521766353	1.051061	0.9025204	1.224049	0.999221
RLN2	Inverse variance weighted	2	-0.03839	0.059967	0.522033546	0.96233575	0.855621	1.0823601	0.999221
ABL1	Wald ratio	1	0.122604	0.191569	0.522172599	1.13043655	0.7765684	1.645556	0.999221
OPN1SW	Inverse variance weighted	2	0.099289	0.155173	0.522260797	1.1043859	0.8147704	1.4969471	0.999221
SLC7A10	Inverse variance weighted	3	-0.07213	0.112732	0.522275465	0.93040963	0.7459594	1.1604681	0.999221
P4HB	Wald ratio	1	-0.12742	0.199436	0.522895217	0.88036613	0.5955251	1.3014474	0.999221
HCK	Inverse variance weighted	2	0.031673	0.049585	0.522977771	1.03217995	0.9365863	1.1375305	0.999221
TMEM9B	Inverse variance weighted	3	0.021981	0.034479	0.523794965	1.0222242	0.9554252	1.0936934	0.999221
PSMA8	Wald ratio	1	0.163311	0.256272	0.523958821	1.17740262	0.7124945	1.9456668	0.999221
EPHA1	Inverse variance weighted	4	-0.01573	0.024695	0.524082937	0.98439088	0.9378791	1.0332093	0.999221
GSK3B	Inverse variance weighted	4	-0.02372	0.037345	0.525408705	0.97656398	0.9076371	1.0507252	0.999221
TRPM6	Inverse variance weighted	5	0.020536	0.032349	0.52553878	1.02074851	0.9580378	1.087564	0.999221
FGFBP3	Wald ratio	1	-0.1089	0.171728	0.525985322	0.89681922	0.6405111	1.255692	0.999221
LINGO2	Inverse variance weighted	6	0.020604	0.032572	0.527008378	1.02081804	0.9576848	1.0881132	0.999221
HLA-B	Inverse variance weighted	7	-0.00974	0.0154	0.527071463	0.99030711	0.9608625	1.020654	0.999221
SCPEP1	Inverse variance weighted	2	-0.04142	0.065562	0.527578427	0.95943013	0.8437351	1.0909896	0.999221
PPIG	Wald ratio	1	-0.03759	0.059524	0.527662047	0.96310373	0.857048	1.0822834	0.999221
NDFIP1	Inverse variance weighted	3	0.025409	0.040253	0.527896752	1.02573421	0.9479176	1.109939	0.999221
TNFRSF25	Inverse variance weighted	2	0.043416	0.068784	0.527918433	1.04437185	0.9126515	1.195103	0.999221
LIPN	Inverse variance weighted	9	-0.0158	0.025047	0.528097314	0.9843218	0.9371666	1.0338498	0.999221
CD81	Inverse variance weighted	3	-0.02746	0.04352	0.528120452	0.97291744	0.8933686	1.0595496	0.999221
IGLV1-36	Wald ratio	1	-0.07704	0.12213	0.528144413	0.92584878	0.7287545	1.1762479	0.999221
TRIM58	Inverse variance weighted	3	-0.02017	0.031989	0.528446111	0.98003691	0.9204773	1.0434503	0.999221
PAK6	Wald ratio	1	-0.12076	0.191795	0.528936933	0.88624705	0.6085496	1.2906652	0.999221
RETN	Inverse variance weighted	3	-0.08427	0.133959	0.529283516	0.91917984	0.7069236	1.1951668	0.999221
EFEMP2	Inverse variance weighted	4	0.022702	0.036141	0.529907562	1.02296126	0.9530061	1.0980515	0.999221
PDE9A	Inverse variance weighted	6	-0.01476	0.023509	0.530213004	0.98535228	0.94098	1.031817	0.999221
TNFRSF10B	Inverse variance weighted	5	-0.02558	0.040788	0.530546331	0.97474355	0.8998523	1.0558677	0.999221
CXCL5	Inverse variance weighted	4	0.014701	0.023476	0.531178939	1.01480922	0.9691735	1.0625939	0.999221
TIE1	Wald ratio	1	0.134151	0.214241	0.531204607	1.14356514	0.7514422	1.7403085	0.999221
SGPL1	Inverse variance weighted	3	0.024576	0.039256	0.531288158	1.02488046	0.9489813	1.10685	0.999221
CYP2R1	Inverse variance weighted	2	-0.0834	0.133359	0.531701303	0.9199791	0.7083702	1.1948011	0.999221
CELSR1	Inverse variance weighted	2	0.049347	0.078954	0.531964209	1.0505849	0.899962	1.2264169	0.999221
DCK	Inverse variance weighted	4	-0.05143	0.082395	0.532537082	0.94987433	0.8082216	1.1163538	0.999221
SLC6A12	Inverse variance weighted	4	-0.02926	0.046881	0.53256883	0.97116592	0.8859057	1.0646316	0.999221
NOTCH1	Inverse variance weighted	4	0.034294	0.054962	0.532654339	1.03488867	0.9292002	1.1525983	0.999221

P2RY14	Inverse variance weighted	3	-0.03444	0.055201	0.532711136	0.96614796	0.8670732	1.0765434	0.999221
PTPN12	Inverse variance weighted	4	-0.01873	0.030041	0.533028033	0.981447	0.9253274	1.0409701	0.999221
CLSTN1	Inverse variance weighted	2	0.025912	0.041612	0.533483305	1.02625014	0.9458725	1.113458	0.999221
ANGEL1	Inverse variance weighted	3	-0.02421	0.038973	0.534499701	0.9760823	0.9042982	1.0535647	0.999221
TUBB2A	Inverse variance weighted	5	0.012622	0.020329	0.534690303	1.0127016	0.9731437	1.0538675	0.999221
ATRN	Inverse variance weighted	2	0.068671	0.110615	0.53472277	1.071084	0.8623154	1.330396	0.999221
APOA1BP	Inverse variance weighted	3	0.026293	0.042365	0.534844477	1.0266414	0.9448378	1.1155276	0.999221
CD226	Inverse variance weighted	3	-0.0279	0.044954	0.534848141	0.97248596	0.8904663	1.0620604	0.999221
LEAP2	Inverse variance weighted	2	-0.05813	0.093668	0.534896672	0.94353137	0.7852797	1.1336744	0.999221
PLIN2	Inverse variance weighted	5	0.017322	0.027918	0.534954637	1.01747296	0.9632933	1.0746999	0.999221
KBTBD8	Inverse variance weighted	3	-0.06396	0.103127	0.535098239	0.93803873	0.7663671	1.148166	0.999221
TG	Inverse variance weighted	5	0.027374	0.044137	0.535118483	1.02775223	0.9425806	1.12062	0.999221
GPRC5D	Inverse variance weighted	2	-0.11288	0.182209	0.535567644	0.89325457	0.6249943	1.2766576	0.999221
IMPA2	Inverse variance weighted	4	-0.02266	0.036577	0.535622383	0.97759723	0.9099655	1.0502556	0.999221
MAPK14	Inverse variance weighted	3	-0.03178	0.051346	0.536002147	0.96872293	0.8759771	1.0712884	0.999221
FZD4	Wald ratio	1	0.180346	0.291544	0.53618666	1.19763168	0.6763261	2.1207544	0.999221
XPNPEP1	Inverse variance weighted	4	0.023223	0.037546	0.536236328	1.02349459	0.9508798	1.1016547	0.999221
PPM1B	Wald ratio	1	-0.08052	0.130254	0.536455494	0.92263568	0.7147523	1.1909813	0.999221
STK38L	Inverse variance weighted	2	-0.06616	0.107018	0.536465162	0.93598565	0.7588806	1.1544229	0.999221
SLC37A4	Inverse variance weighted	2	0.039251	0.063503	0.536507896	1.04003164	0.9183157	1.1778802	0.999221
STIM2	Inverse variance weighted	3	0.027064	0.043786	0.536515719	1.02743325	0.942936	1.1195024	0.999221
ITGA7	Inverse variance weighted	2	0.064375	0.104365	0.537348567	1.06649276	0.8692011	1.3085658	0.999221
FXYD2	Inverse variance weighted	3	-0.05158	0.083655	0.537542252	0.94973131	0.806106	1.1189466	0.999221
ITGAX	Inverse variance weighted	4	-0.01748	0.028359	0.537655334	0.98267226	0.9295414	1.03884	0.999221
COL6A2	Inverse variance weighted	5	-0.02912	0.047277	0.537901861	0.97129795	0.885339	1.0656028	0.999221
PDXK	Inverse variance weighted	5	0.020559	0.033382	0.537988231	1.02077138	0.9561216	1.0897926	0.999221
PYGL	Inverse variance weighted	2	-0.02763	0.044872	0.538000673	0.9727444	0.8908466	1.0621713	0.999221
ADAMTS4	Wald ratio	1	-0.10278	0.166941	0.538120023	0.90232698	0.650521	1.2516029	0.999221
PIGK	Inverse variance weighted	2	0.047768	0.0776	0.538181224	1.04892732	0.9009294	1.2212373	0.999221
KIT	Wald ratio	1	0.167712	0.272667	0.538502309	1.18259547	0.6930072	2.0180627	0.999221
HBEGF	Inverse variance weighted	2	-0.04234	0.068856	0.538629341	0.95854486	0.8375306	1.0970444	0.999221
LILRB1	Inverse variance weighted	3	-0.01699	0.027629	0.538657163	0.98315599	0.9313314	1.0378644	0.999221
SLC25A30	Inverse variance weighted	2	-0.04422	0.07202	0.539229848	0.95674455	0.8307897	1.1017952	0.999221
PLK3	Wald ratio	1	0.121556	0.198091	0.539455648	1.12925217	0.7659013	1.6649803	0.999221
ADK	Inverse variance weighted	2	0.048678	0.079435	0.540003855	1.04988243	0.8985131	1.2267524	0.999221
SERPINH1	Wald ratio	1	-0.02464	0.040288	0.540769028	0.97565902	0.9015801	1.0558247	0.999221
CD93	Inverse variance weighted	5	-0.01689	0.027621	0.540850766	0.98325089	0.9314358	1.0379484	0.999221
CAMKK1	Inverse variance weighted	2	-0.05256	0.086073	0.541459592	0.94880015	0.8015074	1.1231609	0.999221
HNRNPUL2	Inverse variance weighted	3	0.038094	0.062394	0.541504245	1.0388288	0.9192492	1.1739638	0.999221
LMLN	Inverse variance weighted	2	-0.06495	0.10643	0.541705166	0.93711641	0.7606731	1.1544869	0.999221
KIF11	Wald ratio	1	0.079572	0.130445	0.541861808	1.08282314	0.8385331	1.3982822	0.999221
PTPRN	Inverse variance weighted	2	0.023671	0.038852	0.542353645	1.0239533	0.9488741	1.1049731	0.999221
SRC	Inverse variance weighted	3	-0.06913	0.113519	0.542555712	0.93320765	0.7470492	1.1657552	0.999221
ATP13A2	Inverse variance weighted	2	0.034237	0.056251	0.54275202	1.03483015	0.9268033	1.1554484	0.999221
PELO	Inverse variance weighted	4	-0.01703	0.027986	0.542835551	0.98311394	0.9306402	1.0385464	0.999221
KLHL5	Inverse variance weighted	4	-0.02326	0.038237	0.542920682	0.97700475	0.9064593	1.0530405	0.999221
ICAM1	Inverse variance weighted	2	-0.03732	0.061402	0.543272008	0.96336322	0.8541288	1.0865676	0.999221
TOR2A	Inverse variance weighted	2	-0.07572	0.12468	0.543635379	0.92707474	0.7260813	1.1837071	0.999221
KLHL6	Inverse variance weighted	3	0.033082	0.054525	0.544033536	1.03363501	0.9288691	1.1502173	0.999221
KCNK13	Inverse variance weighted	2	0.031326	0.051643	0.544122436	1.03182226	0.932492	1.1417333	0.999221
PKD2L2	Wald ratio	1	-0.05937	0.097958	0.544474509	0.94235973	0.7777385	1.1418258	0.999221
PTPRM	Inverse variance weighted	6	-0.01664	0.027471	0.544587229	0.98349329	0.9319392	1.0378993	0.999221
NEK6	Inverse variance weighted	5	-0.03156	0.052097	0.544675814	0.96893471	0.8748806	1.0731001	0.999221
KCNH8	Inverse variance weighted	2	-0.08287	0.136813	0.544726846	0.92047522	0.7039701	1.2035662	0.999221
H3F3A	Inverse variance weighted	2	-0.01402	0.02317	0.545139594	0.98607871	0.9422997	1.0318917	0.999221
TYMP	Inverse variance weighted	5	0.014047	0.023222	0.545241614	1.01414597	0.9690226	1.0613706	0.999221
C10orf54	Inverse variance weighted	3	0.087689	0.145226	0.545969798	1.09164858	0.8212283	1.4511149	0.999221
CSF1R	Inverse variance weighted	3	-0.02896	0.04799	0.546213169	0.97145606	0.8842465	1.0672668	0.999221
KCNE1	Inverse variance weighted	2	-0.02376	0.039382	0.546238967	0.97651661	0.9039756	1.0548788	0.999221
CCRL2	Inverse variance weighted	3	-0.0879	0.145699	0.54629585	0.9158494	0.6883388	1.2185571	0.999221
RIOK2	Inverse variance weighted	2	0.046402	0.076942	0.54645724	1.04749552	0.9008607	1.2179983	0.999221
RAMP3	Inverse variance weighted	3	0.023657	0.039268	0.546867743	1.02393934	0.9480883	1.1058587	0.999221
FGF22	Wald ratio	1	-0.18919	0.31412	0.546980234	0.82762777	0.4471474	1.5318613	0.999221

ITGB3	Inverse variance weighted	2	-0.03711	0.061644	0.547219096	0.96357425	0.8539107	1.0873213	0.999221
SLC7A7	Inverse variance weighted	4	-0.02443	0.040583	0.547269384	0.97587047	0.9012533	1.0566654	0.999221
DERL1	Inverse variance weighted	3	-0.05994	0.099809	0.548126854	0.94181914	0.7744773	1.1453187	0.999221
ST3GAL1	Inverse variance weighted	4	-0.02295	0.038289	0.548963134	0.97731387	0.9066537	1.053481	0.999221
OR52K1	Inverse variance weighted	2	-0.05989	0.100006	0.549273569	0.94186944	0.7742188	1.1458234	0.999221
CDA	Inverse variance weighted	10	0.010221	0.017073	0.549384452	1.01027347	0.9770268	1.0446515	0.999221
CHRNA2	Inverse variance weighted	2	0.054288	0.09069	0.549434464	1.05578815	0.8838533	1.2611693	0.999221
CSNK1A1	Wald ratio	1	-0.13045	0.218068	0.549696961	0.87769908	0.5724299	1.3457643	0.999221
PRR4	Inverse variance weighted	5	-0.02163	0.03618	0.549923907	0.97860115	0.911609	1.0505164	0.999221
LGALS9	Inverse variance weighted	8	-0.01601	0.026789	0.550161692	0.98412047	0.9337808	1.037174	0.999221
CHI3L1	Inverse variance weighted	5	0.013278	0.022241	0.550497249	1.01336701	0.9701402	1.0585199	0.999221
CHST13	Inverse variance weighted	5	-0.01738	0.029148	0.550879352	0.98276533	0.9281942	1.0405449	0.999221
AOC2	Wald ratio	1	-0.15286	0.256336	0.55095461	0.85824884	0.5192963	1.4184409	0.999221
CHST11	Inverse variance weighted	4	0.04043	0.06785	0.551265953	1.04125795	0.9115973	1.1893609	0.999221
FBLN5	Inverse variance weighted	5	0.013963	0.02344	0.551388053	1.0140608	0.9685261	1.0617363	0.999221
ABCD3	Inverse variance weighted	2	0.131767	0.221773	0.552409662	1.14084241	0.7386674	1.7619857	0.999221
EXTL2	Inverse variance weighted	4	0.019177	0.032285	0.552511806	1.01936247	0.9568568	1.0859512	0.999221
ICK	Wald ratio	1	-0.11115	0.187153	0.552573227	0.89480305	0.6200401	1.2913236	0.999221
INHBB	Inverse variance weighted	2	0.024969	0.042077	0.552903446	1.02528348	0.9441202	1.1134242	0.999221
MDM4	Wald ratio	1	0.040426	0.068147	0.553033703	1.04125422	0.9110646	1.1900477	0.999221
SLC6A6	Inverse variance weighted	2	0.023314	0.039309	0.553120904	1.02358743	0.9476867	1.1055671	0.999221
WNT5B	Inverse variance weighted	2	-0.02568	0.043331	0.553423915	0.97464738	0.8952895	1.0610395	0.999221
PODN	Inverse variance weighted	3	0.030171	0.050969	0.553887727	1.03063044	0.9326473	1.1389076	0.999221
PTGR1	Inverse variance weighted	3	0.050607	0.085604	0.554400686	1.05190959	0.8894283	1.2440731	0.999221
GPR146	Inverse variance weighted	2	0.023821	0.040309	0.554543055	1.02410739	0.9463103	1.1083002	0.999221
KCNAB2	Wald ratio	1	0.135331	0.22919	0.554871444	1.14491601	0.7306058	1.7941723	0.999221
HLCS	Inverse variance weighted	4	-0.02311	0.03915	0.555026265	0.97715692	0.9049806	1.0550896	0.999221
NTSR1	Inverse variance weighted	6	-0.01858	0.031535	0.555747066	0.98159184	0.9227573	1.0441776	0.999221
PTGS2	Inverse variance weighted	3	0.028604	0.048569	0.555906143	1.02901717	0.9355769	1.1317897	0.999221
CTSB	Inverse variance weighted	4	0.031368	0.053321	0.556334797	1.03186546	0.9294703	1.1455409	0.999221
CTDSP1	Wald ratio	1	0.085559	0.14545	0.55637437	1.08932572	0.8191211	1.4486631	0.999221
DAGLA	Wald ratio	1	0.047492	0.080819	0.556781431	1.04863751	0.8950157	1.2286271	0.999221
ARSA	Inverse variance weighted	4	-0.0201	0.034218	0.556926712	0.98010067	0.9165241	1.0480874	0.999221
DAPK1	Inverse variance weighted	5	0.011139	0.018965	0.556998217	1.01120078	0.9743022	1.0494968	0.999221
ROR1	Wald ratio	1	-0.09751	0.166185	0.55735358	0.9070903	0.6549247	1.2563473	0.999221
DUSP13	Wald ratio	1	0.12974	0.221196	0.557513723	1.13853198	0.7380058	1.7564294	0.999221
PI4KB	Wald ratio	1	0.140071	0.239035	0.557884075	1.15035603	0.7200482	1.8378201	0.999221
ANGEL2	Inverse variance weighted	4	0.023634	0.040349	0.558055067	1.02391531	0.9460588	1.108179	0.999221
H3F3B	Wald ratio	1	-0.05311	0.090704	0.558154037	0.94827103	0.7938231	1.1327687	0.999221
RARA	Inverse variance weighted	2	0.097084	0.166353	0.559488642	1.10195298	0.7953535	1.526743	0.999221
MFAP1	Wald ratio	1	0.062736	0.107636	0.559989473	1.06474601	0.8622329	1.3148235	0.999221
C17orf72	Inverse variance weighted	3	0.029097	0.049954	0.560243175	1.02952484	0.9335012	1.1354258	0.999221
FFAR3	Inverse variance weighted	2	0.01863	0.032002	0.560472078	1.01880445	0.9568634	1.0847552	0.999221
TRIB2	Inverse variance weighted	5	-0.02773	0.047738	0.561268637	0.97264707	0.8857671	1.0680485	0.999221
TNF	Inverse variance weighted	3	-0.02412	0.04154	0.561401392	0.97616374	0.8998351	1.058967	0.999221
CCND3	Wald ratio	1	0.039833	0.068602	0.561479628	1.04063711	0.9097133	1.1904032	0.999221
PTGFRN	Inverse variance weighted	3	-0.05861	0.100939	0.561508575	0.94307887	0.773797	1.1493941	0.999221
PLA2G15	Inverse variance weighted	2	0.067982	0.117363	0.562423902	1.07034623	0.8503986	1.3471813	0.999221
SERPINE2	Inverse variance weighted	5	-0.0177	0.030584	0.562743601	0.98245465	0.9252923	1.0431484	0.999221
HPGDS	Wald ratio	1	-0.11803	0.20399	0.562862265	0.88867158	0.5958016	1.3255036	0.999221
PSMB9	Inverse variance weighted	4	-0.02275	0.039388	0.563614914	0.97751092	0.904886	1.0559647	0.999221
PDE6B	Wald ratio	1	-0.03246	0.056282	0.564150837	0.96806372	0.8669526	1.0809672	0.999221
TUBBP5	Wald ratio	1	-0.08602	0.149175	0.564164654	0.91757215	0.6849521	1.2291935	0.999221
SPNS3	Wald ratio	1	-0.10813	0.187975	0.56514168	0.89751348	0.620917	1.2973239	0.999221
JAM2	Wald ratio	1	-0.05387	0.093653	0.565180794	0.94755925	0.7886554	1.1384802	0.999221
EDEM2	Inverse variance weighted	2	-0.05191	0.090268	0.56522091	0.94941075	0.7954559	1.1331625	0.999221
TXNRD1	Inverse variance weighted	2	-0.10203	0.17743	0.565265459	0.90300311	0.6377602	1.2785599	0.999221
HYAL3	Inverse variance weighted	3	0.020749	0.036086	0.565304219	1.02096568	0.9512483	1.0957927	0.999221
GBA	Inverse variance weighted	2	-0.0247	0.042965	0.565426705	0.97560594	0.8968122	1.0613225	0.999221
NUCB1	Inverse variance weighted	4	-0.0191	0.033327	0.566535528	0.98107953	0.9190427	1.047304	0.999221
C1QA	Inverse variance weighted	4	0.055188	0.096443	0.567165073	1.05673918	0.8747291	1.2766211	0.999221
MUC16	Inverse variance weighted	2	0.062482	0.109279	0.567482376	1.06447518	0.8592413	1.3187302	0.999221
DLD	Inverse variance weighted	2	0.061812	0.108167	0.567696601	1.06376222	0.8605393	1.3149778	0.999221

PSMB6	Wald ratio	1	0.071738	0.125706	0.568216152	1.07437399	0.8397541	1.3745447	0.999221
PDE7B	Wald ratio	1	-0.08148	0.142927	0.568614312	0.92174958	0.6965481	1.2197611	0.999221
KCNA5	Wald ratio	1	-0.13426	0.235644	0.568835441	0.87436065	0.5509425	1.387634	0.999221
DNMT3A	Wald ratio	1	-0.05665	0.099563	0.569379541	0.94492664	0.7774066	1.1485448	0.999221
GABBR1	Inverse variance weighted	3	0.026176	0.046007	0.569390976	1.02652121	0.9380068	1.1233883	0.999221
INSL3	Inverse variance weighted	5	0.025848	0.045461	0.56965115	1.02618472	0.9387026	1.1218197	0.999221
PTGIR	Inverse variance weighted	2	0.027678	0.048707	0.56986888	1.02806418	0.9344581	1.1310469	0.999221
DHRS1	Inverse variance weighted	8	0.012981	0.02285	0.569973695	1.01306575	0.9686947	1.0594692	0.999221
TRIM62	Wald ratio	1	0.14437	0.254316	0.57025447	1.15531097	0.7018113	1.9018551	0.999221
SLC25A27	Inverse variance weighted	2	-0.04328	0.076276	0.570411768	0.9576409	0.8246607	1.1120648	0.999221
SPINT2	Inverse variance weighted	4	-0.02992	0.05273	0.570443221	0.97052399	0.8752281	1.0761958	0.999221
HPX	Inverse variance weighted	2	-0.14201	0.250407	0.570639999	0.86761446	0.5311002	1.41735	0.999221
ITGAV	Inverse variance weighted	3	0.037736	0.066612	0.571052994	1.03845681	0.9113542	1.1832859	0.999221
WEE1	Inverse variance weighted	2	-0.02895	0.051193	0.571742623	0.97146603	0.8787215	1.0739993	0.999221
ECM1	Inverse variance weighted	2	-0.04208	0.074479	0.572088331	0.95879425	0.8285677	1.1094885	0.999221
ALOX12	Inverse variance weighted	2	0.049283	0.087301	0.572401518	1.05051735	0.8853021	1.2465651	0.999221
GUCY1B3	Inverse variance weighted	2	-0.16816	0.297966	0.572501912	0.84521569	0.4713392	1.5156593	0.999221
LEP	Wald ratio	1	0.087825	0.15562	0.572511581	1.09179708	0.8047772	1.4811811	0.999221
GLB1	Inverse variance weighted	3	0.027153	0.048119	0.57255726	1.02752503	0.9350454	1.1291512	0.999221
MASP2	Wald ratio	1	0.072614	0.12877	0.572815538	1.07531584	0.8354591	1.3840345	0.999221
RPS6KB1	Wald ratio	1	-0.0614	0.108929	0.573001634	0.9404503	0.7596498	1.1642823	0.999221
CD63	Inverse variance weighted	2	-0.04219	0.075105	0.574289227	0.95868789	0.8274598	1.1107277	0.999221
PXDNL	Wald ratio	1	0.125787	0.223963	0.574358708	1.13404085	0.7311186	1.759015	0.999221
SIRT3	Inverse variance weighted	2	-0.03153	0.05629	0.575328655	0.96895706	0.8677394	1.0819813	0.999221
ABCD4	Wald ratio	1	0.097795	0.174635	0.575479438	1.10273715	0.783105	1.5528304	0.999221
GMPR	Inverse variance weighted	2	-0.06688	0.119467	0.575612509	0.93530908	0.7400529	1.1820818	0.999221
CES2	Inverse variance weighted	2	0.08596	0.153609	0.575748897	1.08976298	0.8064504	1.4726056	0.999221
IGKV3-11	Wald ratio	1	-0.07017	0.125462	0.57594196	0.93223176	0.7290012	1.1921188	0.999221
CXCR4	Wald ratio	1	-0.10741	0.192213	0.576282163	0.89815436	0.6162199	1.3090802	0.999221
SCGB1C1	Inverse variance weighted	2	-0.01215	0.02175	0.576519232	0.98792648	0.9466958	1.0309529	0.999221
LPAR3	Inverse variance weighted	3	0.027494	0.049239	0.576581127	1.02787575	0.9333137	1.1320187	0.999221
F2R	Inverse variance weighted	4	-0.02521	0.04514	0.576584646	0.97510951	0.892543	1.065314	0.999221
HAGH	Inverse variance weighted	4	0.020734	0.037156	0.576836747	1.02095013	0.9492407	1.0980768	0.999221
TPSD1	Inverse variance weighted	2	0.022068	0.039552	0.5768803	1.02231345	0.946055	1.1047188	0.999221
SLC18B1	Inverse variance weighted	6	-0.01418	0.025425	0.576961532	0.9859173	0.9379901	1.0362933	0.999221
BTN3A1	Inverse variance weighted	3	0.017278	0.03102	0.577526389	1.01742839	0.9574123	1.0812067	0.999221
CDK19	Inverse variance weighted	2	-0.037	0.066455	0.577668374	0.96367428	0.8459843	1.0977368	0.999221
IFNGR2	Inverse variance weighted	8	-0.01542	0.027715	0.577852872	0.98469433	0.9326314	1.0396636	0.999221
PSMA6	Wald ratio	1	0.049468	0.088925	0.578011672	1.05071227	0.8826514	1.2507728	0.999221
TACR2	Wald ratio	1	-0.05119	0.092145	0.578514722	0.95009678	0.7931089	1.1381589	0.999221
MORC2	Inverse variance weighted	3	-0.04888	0.088072	0.578856942	0.95229082	0.8013112	1.1317174	0.999221
RNASEL	Inverse variance weighted	4	-0.02381	0.042894	0.578867513	0.97647307	0.8977342	1.0621179	0.999221
RNLS	Inverse variance weighted	2	-0.03682	0.066484	0.579734214	0.96385245	0.8460932	1.0980014	0.999221
CCNH	Wald ratio	1	0.073966	0.13358	0.579771164	1.07676978	0.8287385	1.3990337	0.999221
FDPS	Wald ratio	1	-0.07201	0.130046	0.579771164	0.93052283	0.7211574	1.200671	0.999221
HAS3	Wald ratio	1	0.15533	0.281626	0.581259174	1.16804316	0.6725645	2.0285412	0.999221
SLC25A34	Inverse variance weighted	5	0.019677	0.035684	0.581336606	1.01987237	0.9509787	1.0937571	0.999221
CARD17	Inverse variance weighted	2	-0.04389	0.079766	0.582130109	0.95705613	0.8185389	1.1190139	0.999221
CDK17	Wald ratio	1	0.080211	0.145838	0.582319374	1.08351584	0.8141324	1.4420339	0.999221
MCHR1	Inverse variance weighted	2	0.042913	0.078064	0.582516664	1.04384658	0.8957516	1.2164261	0.999221
C11orf83	Wald ratio	1	-0.05053	0.091955	0.58262837	0.95072194	0.7939262	1.1384839	0.999221
EPOR	Inverse variance weighted	4	-0.02435	0.044348	0.582985922	0.97594593	0.8946977	1.0645724	0.999221
CD68	Inverse variance weighted	3	0.029553	0.053872	0.583296417	1.02999384	0.9267828	1.144699	0.999221
IGSF11	Inverse variance weighted	2	-0.02835	0.051708	0.583521017	0.9720493	0.8783623	1.075729	0.999221
RANBP9	Wald ratio	1	0.1299	0.23701	0.583639085	1.13871416	0.7155958	1.8120145	0.999221
CRISP3	Inverse variance weighted	3	0.035181	0.064213	0.583777433	1.03580702	0.9133126	1.1747305	0.999221
HIST1H4K	Inverse variance weighted	2	0.016708	0.030514	0.584014251	1.0168479	0.957815	1.0795191	0.999221
STK11	Inverse variance weighted	2	0.021544	0.039406	0.584566752	1.02177814	0.9458307	1.1038239	0.999221
CPD	Inverse variance weighted	4	-0.0202	0.03695	0.5846582	0.98000592	0.9115409	1.0536133	0.999221
PTGDR2	Wald ratio	1	-0.05274	0.096684	0.585440935	0.94862983	0.7848702	1.1465571	0.999221
CES1	Inverse variance weighted	4	-0.01113	0.020437	0.585913786	0.98892841	0.9500985	1.0293453	0.999221
ADRA2A	Wald ratio	1	-0.10763	0.198848	0.588311569	0.89795661	0.6081241	1.3259236	0.999221
GUSB	Wald ratio	1	0.025017	0.046222	0.58834416	1.02533251	0.9365262	1.1225599	0.999221

PSENEN	Wald ratio	1	-0.06849	0.126579	0.588441491	0.93380174	0.728633	1.196742	0.999221
PRKD1	Inverse variance weighted	3	-0.01741	0.032215	0.588991105	0.98274479	0.922611	1.046798	0.999221
IGLC7	Inverse variance weighted	3	0.016029	0.029674	0.589072819	1.01615824	0.958744	1.0770107	0.999221
PIK3C3	Wald ratio	1	-0.01785	0.033063	0.589197032	0.98230448	0.9206667	1.0480689	0.999221
PCOLCE2	Wald ratio	1	-0.08407	0.156126	0.590258451	0.91936901	0.6770067	1.2484949	0.999221
HIST1H4E	Inverse variance weighted	3	-0.03253	0.06047	0.59056474	0.96798962	0.8598004	1.0897924	0.999221
LGR4	Inverse variance weighted	2	-0.03976	0.073978	0.590933514	0.96101792	0.8313038	1.1109723	0.999221
KLRC1	Inverse variance weighted	5	0.019165	0.035705	0.591436542	1.01934963	0.9504527	1.0932407	0.999221
ITFG2	Inverse variance weighted	4	0.025236	0.047032	0.591564706	1.02555697	0.9352453	1.1245896	0.999221
PRKCI	Wald ratio	1	-0.05644	0.105425	0.592405414	0.94512374	0.7686869	1.1620582	0.999221
RPS6KA4	Inverse variance weighted	5	-0.01989	0.03718	0.592604756	0.98030308	0.9114073	1.0544069	0.999221
PRPSAP1	Inverse variance weighted	3	0.034159	0.063863	0.592732326	1.03474932	0.9130061	1.1727261	0.999221
SLC25A4	Inverse variance weighted	3	0.042889	0.080368	0.59357578	1.04382243	0.8916941	1.2219048	0.999221
CDC42BPA	Inverse variance weighted	2	-0.04201	0.078746	0.593657694	0.95885611	0.8217197	1.1188792	0.999221
IGFBP4	Inverse variance weighted	3	0.02345	0.043983	0.593927921	1.02372689	0.9391713	1.1158952	0.999221
PRG4	Inverse variance weighted	2	0.028848	0.054213	0.594636155	1.0292685	0.9255111	1.144658	0.999221
TSG101	Wald ratio	1	-0.07882	0.148124	0.594649748	0.92420773	0.6913273	1.2355363	0.999221
GSTO2	Inverse variance weighted	3	-0.02518	0.047417	0.595384052	0.97513335	0.8885901	1.0701054	0.999221
CLEC3B	Inverse variance weighted	4	0.019695	0.037107	0.595595524	1.01988973	0.9483461	1.0968307	0.999221
PLAUR	Inverse variance weighted	3	0.043289	0.081597	0.59574632	1.04423986	0.8899054	1.2253402	0.999221
CACNA1I	Inverse variance weighted	2	-0.05792	0.109324	0.596276846	0.94372941	0.7617087	1.1692465	0.999221
CREG1	Inverse variance weighted	2	-0.06191	0.116865	0.596280785	0.93996734	0.747541	1.1819266	0.999221
ICOSLG	Inverse variance weighted	4	0.025837	0.04881	0.596569599	1.0261739	0.9325515	1.1291954	0.999221
CHGB	Inverse variance weighted	4	-0.01969	0.037249	0.597118232	0.98050455	0.9114703	1.0547674	0.999221
STX1A	Wald ratio	1	-0.03486	0.066115	0.598004211	0.96574021	0.8483644	1.0993556	0.999221
SLAMF6	Inverse variance weighted	2	-0.03128	0.059376	0.598353589	0.96920674	0.8627298	1.088825	0.999221
HDAC9	Wald ratio	1	-0.12768	0.242584	0.598668814	0.88013876	0.5470911	1.4159329	0.999221
KBTBD2	Inverse variance weighted	5	-0.01365	0.025939	0.598687483	0.98644136	0.9375438	1.0378892	0.999221
CATSPER2	Inverse variance weighted	2	-0.02444	0.046533	0.59941818	0.97585542	0.890791	1.0690429	0.999221
KCNG1	Wald ratio	1	0.087027	0.16571	0.599458167	1.09092651	0.7883891	1.5095599	0.999221
NLK	Wald ratio	1	0.13121	0.2504	0.60027854	1.14020691	0.697973	1.862639	0.999221
MCCC2	Wald ratio	1	0.095695	0.182921	0.600871162	1.10042317	0.7688723	1.5749445	0.999221
NELL2	Inverse variance weighted	3	0.012777	0.024426	0.600926912	1.01285864	0.9655097	1.0625296	0.999221
SPON2	Inverse variance weighted	4	-0.0294	0.056313	0.601557796	0.97102379	0.869552	1.0843367	0.999221
PARP3	Wald ratio	1	-0.10516	0.201908	0.60248288	0.90018004	0.6059848	1.3372021	0.999221
FSTL3	Inverse variance weighted	2	0.035819	0.068776	0.602506441	1.03646796	0.905758	1.1860407	0.999221
MMP24	Wald ratio	1	0.031352	0.060215	0.602602838	1.03184839	0.9169795	1.1611068	0.999221
HCAR3	Inverse variance weighted	5	0.014168	0.027226	0.602799609	1.01426887	0.9615625	1.0698642	0.999221
STK25	Wald ratio	1	0.055663	0.106977	0.602836932	1.05724138	0.8572611	1.3038726	0.999221
MC1R	Inverse variance weighted	5	-0.01791	0.034488	0.603599329	0.98225215	0.9180498	1.0509444	0.999221
PLK4	Wald ratio	1	0.062233	0.120152	0.60449405	1.06420975	0.8409146	1.3467983	0.999221
LILRA2	Inverse variance weighted	5	-0.01174	0.022707	0.605061404	0.98832604	0.9453042	1.0333058	0.999221
GP9	Wald ratio	1	-0.11921	0.231058	0.605907794	0.88762277	0.5643488	1.3960766	0.999221
TNFRSF14	Wald ratio	1	0.014832	0.028824	0.606858351	1.01494237	0.9591925	1.0739325	0.999221
ALDH3B1	Inverse variance weighted	3	0.027432	0.053376	0.607290791	1.02781209	0.9257188	1.1411648	0.999221
PRNP	Inverse variance weighted	2	-0.02791	0.054312	0.607314584	0.97247442	0.8742726	1.0817067	0.999221
RNASE6	Inverse variance weighted	4	-0.01126	0.021934	0.607626878	0.98880107	0.9471934	1.0322364	0.999221
SIGLEC7	Inverse variance weighted	2	0.070786	0.13793	0.607809355	1.07335148	0.819094	1.4065338	0.999221
IGKV3-7	Inverse variance weighted	2	-0.03817	0.074653	0.609157409	0.9625511	0.8315306	1.1142159	0.999221
NFATC2	Wald ratio	1	-0.11723	0.229511	0.609497155	0.88937878	0.5671832	1.3946017	0.999221
GRIK1	Inverse variance weighted	2	0.057272	0.112155	0.609595468	1.05894403	0.8499717	1.3192938	0.999221
FUCA1	Inverse variance weighted	4	-0.01648	0.032269	0.609624163	0.98365798	0.9233704	1.0478818	0.999221
GAD1	Inverse variance weighted	3	0.022468	0.044014	0.609728519	1.0227218	0.9381924	1.1148671	0.999221
PKDREJ	Inverse variance weighted	2	-0.04553	0.089329	0.610232802	0.95548638	0.8020214	1.1383165	0.999221
PEBP4	Wald ratio	1	0.125493	0.246338	0.610448076	1.13370717	0.6995419	1.8373336	0.999221
MSR1	Inverse variance weighted	5	0.018484	0.036353	0.611125119	1.01865602	0.9486011	1.0938846	0.999221
GPI	Inverse variance weighted	2	0.043998	0.086945	0.612827857	1.04497994	0.8812496	1.2391302	0.999221
FZD3	Inverse variance weighted	2	-0.038	0.075112	0.612874994	0.96270853	0.8309185	1.1154015	0.999221
CYP2E1	Inverse variance weighted	2	0.035693	0.070604	0.613177602	1.03633813	0.9024059	1.1901481	0.999221
PARP14	Inverse variance weighted	4	-0.02376	0.047011	0.6133415	0.97652486	0.8905674	1.0707789	0.999221
FPGS	Inverse variance weighted	2	-0.07854	0.155546	0.613618421	0.92446732	0.6815349	1.2539928	0.999221
RNF31	Wald ratio	1	-0.0946	0.187381	0.613661126	0.90973675	0.6301065	1.3134619	0.999221
ABCA6	Wald ratio	1	0.093709	0.185714	0.613848833	1.09823982	0.7631581	1.5804467	0.999221

PAK2	Inverse variance weighted	2	-0.04007	0.079475	0.614112229	0.96071978	0.8221401	1.1226584	0.999221
TARS	Inverse variance weighted	3	-0.04548	0.090222	0.614216289	0.95554103	0.8006653	1.140375	0.999221
SLC9A5	Wald ratio	1	-0.11596	0.230366	0.61469827	0.89050961	0.5669528	1.3987185	0.999221
SMPD2	Inverse variance weighted	2	0.01522	0.030255	0.614927241	1.01533625	0.9568775	1.0773664	0.999221
CD86	Inverse variance weighted	3	0.023482	0.046727	0.615290009	1.02375971	0.9341649	1.1219475	0.999221
IGKV2D-40	Wald ratio	1	-0.03247	0.064718	0.615865751	0.96805109	0.8527247	1.0989748	0.999221
ANTXR2	Inverse variance weighted	3	0.033579	0.067117	0.616862481	1.03414901	0.9066753	1.1795448	0.999221
FAM19A1	Inverse variance weighted	4	-0.02491	0.049803	0.616942703	0.97539671	0.8846834	1.0754116	0.999221
ITGAE	Wald ratio	1	-0.03116	0.062321	0.617075077	0.96932013	0.8578647	1.0952561	0.999221
PAM	Inverse variance weighted	5	-0.01461	0.029242	0.617301579	0.98549486	0.9306009	1.0436269	0.999221
NOV	Inverse variance weighted	3	-0.04861	0.097317	0.617415427	0.95255093	0.7871362	1.1527271	0.999221
CYTL1	Inverse variance weighted	3	-0.01402	0.028096	0.617706356	0.986075	0.9332415	1.0418995	0.999221
SLC6A16	Inverse variance weighted	2	0.032151	0.06446	0.617940449	1.03267335	0.9101088	1.1717437	0.999221
PON2	Inverse variance weighted	4	0.016603	0.033364	0.618737958	1.01674169	0.9523814	1.0854514	0.999221
SLC28A3	Inverse variance weighted	2	-0.01745	0.035109	0.619190948	0.98270226	0.9173531	1.0527066	0.999221
MPZL3	Inverse variance weighted	3	0.01793	0.036095	0.619363537	1.01809213	0.9485543	1.0927278	0.999221
ABCC3	Inverse variance weighted	4	0.012975	0.026124	0.61942237	1.01305971	0.9624929	1.0662832	0.999221
TMCO3	Inverse variance weighted	4	0.014449	0.029115	0.619694143	1.01455407	0.9582796	1.0741333	0.999221
MS4A1	Inverse variance weighted	2	0.100302	0.202107	0.619694708	1.10550493	0.743916	1.6428483	0.999221
PREPL	Wald ratio	1	-0.03967	0.080023	0.620087391	0.96110722	0.8215902	1.1243161	0.999221
DNMT1	Wald ratio	1	-0.1098	0.221532	0.620139835	0.89601079	0.5804187	1.3832003	0.999221
FGF17	Wald ratio	1	0.104205	0.210473	0.620530918	1.1098276	0.7346787	1.6765388	0.999221
SLC44A1	Inverse variance weighted	3	0.064731	0.130916	0.620990507	1.06687189	0.8254192	1.3789547	0.999221
GZMM	Inverse variance weighted	2	-0.06404	0.129561	0.621097274	0.93796626	0.7276172	1.2091258	0.999221
SYNJ1	Inverse variance weighted	2	-0.05557	0.112447	0.621194504	0.94594922	0.758842	1.1791913	0.999221
FLT3LG	Wald ratio	1	-0.09416	0.190593	0.62127255	0.9101353	0.6264262	1.3223365	0.999221
P2RY6	Wald ratio	1	-0.10833	0.219765	0.622042443	0.89732726	0.5832886	1.3804422	0.999221
SCD5	Inverse variance weighted	4	-0.01486	0.030161	0.622216367	0.98524918	0.9286937	1.0452488	0.999221
CLK4	Inverse variance weighted	7	-0.01531	0.031091	0.622506455	0.98481038	0.9265895	1.0466895	0.999221
EPHX1	Inverse variance weighted	3	-0.04472	0.091003	0.62309758	0.95626053	0.800042	1.1429828	0.999221
GABRD	Inverse variance weighted	2	-0.07469	0.151984	0.623129722	0.92803286	0.6889562	1.2500723	0.999221
CACHD1	Wald ratio	1	0.036071	0.073511	0.623650509	1.03672914	0.8976175	1.1974001	0.999221
PRKCZ	Inverse variance weighted	2	-0.03142	0.064051	0.623763722	0.96906991	0.8547395	1.0986933	0.999221
SMCHD1	Inverse variance weighted	2	0.057232	0.11698	0.624667432	1.05890149	0.841938	1.3317754	0.999221
KCNK5	Inverse variance weighted	2	-0.20388	0.416894	0.624805332	0.81555813	0.3602364	1.8463849	0.999221
PLA2G6	Inverse variance weighted	2	0.028234	0.057734	0.624815825	1.02863664	0.9185809	1.1518782	0.999221
SLC25A41	Inverse variance weighted	2	-0.02119	0.043345	0.624944562	0.97903367	0.8992944	1.0658433	0.999221
CPAMD8	Inverse variance weighted	4	0.015734	0.032206	0.625164242	1.01585825	0.9537164	1.0820491	0.999221
EBPL	Inverse variance weighted	5	0.01173	0.024015	0.625245651	1.0117989	0.9652768	1.0605632	0.999221
SLC5A3	Inverse variance weighted	3	0.052077	0.106741	0.625631944	1.05345697	0.8545889	1.2986028	0.999221
PDK1	Inverse variance weighted	4	0.038825	0.079637	0.625888821	1.03958852	0.88935	1.215207	0.999221
CTSE	Wald ratio	1	0.028544	0.058656	0.62651984	1.02895491	0.9172077	1.1543168	0.999221
EPHB3	Inverse variance weighted	3	-0.02039	0.041991	0.627265339	0.97981654	0.9024048	1.063869	0.999221
SGK3	Inverse variance weighted	2	-0.06675	0.137721	0.627920221	0.93543132	0.7141362	1.2253009	0.999221
CA6	Inverse variance weighted	3	0.016327	0.033696	0.628002491	1.01646091	0.9514991	1.0858579	0.999221
CFH	Inverse variance weighted	2	0.044527	0.092069	0.628649046	1.04553371	0.8729049	1.2523022	0.999221
TRBV7-1	Inverse variance weighted	4	0.007185	0.014863	0.628792431	1.00721121	0.9782923	1.036985	0.999221
ST14	Inverse variance weighted	3	0.016674	0.034494	0.628821439	1.01681382	0.9503408	1.0879364	0.999221
DUSP4	Wald ratio	1	0.083117	0.172257	0.629438382	1.08666892	0.7752989	1.5230891	0.999221
FCGR3A	Inverse variance weighted	3	0.040941	0.084918	0.629716391	1.04179072	0.8820577	1.23045	0.999221
CYB5R4	Wald ratio	1	0.073976	0.153479	0.629809882	1.07678082	0.7970464	1.4546918	0.999221
TNFRSF1A	Inverse variance weighted	2	-0.0188	0.039003	0.629851101	0.98137855	0.9091519	1.0593432	0.999221
FPR2	Inverse variance weighted	7	-0.01648	0.034222	0.630123211	0.98365514	0.9198398	1.0518978	0.999221
EMR2	Inverse variance weighted	6	-0.01148	0.023878	0.630809067	0.98859023	0.9433898	1.0359563	0.999221
TNFRSF10D	Inverse variance weighted	2	-0.04207	0.087594	0.631011724	0.95880101	0.8075453	1.1383874	0.999221
SUCNR1	Inverse variance weighted	2	0.085862	0.179202	0.63184177	1.08965596	0.7669196	1.5482067	0.999221
PTGFR	Inverse variance weighted	3	-0.0272	0.056784	0.631909366	0.97316482	0.8706648	1.0877318	0.999221
TXNL1	Inverse variance weighted	2	-0.01976	0.041266	0.632050434	0.98043395	0.904257	1.0630282	0.999221
TRIM5	Inverse variance weighted	4	-0.01703	0.035573	0.632136229	0.98311478	0.9169047	1.0541059	0.999221
TGM2	Inverse variance weighted	4	-0.05301	0.111029	0.633030757	0.94836794	0.7628984	1.1789274	0.999221
EFTUD1	Inverse variance weighted	2	-0.0692	0.14508	0.633389347	0.93314246	0.7021886	1.2400585	0.999221
SLC25A13	Inverse variance weighted	5	0.021878	0.045875	0.63342724	1.02211914	0.9342264	1.1182809	0.999221
PRKCD	Inverse variance weighted	2	0.030529	0.064101	0.633883532	1.03099993	0.9092744	1.169021	0.999221

KCNQ1	Inverse variance weighted	2	0.061616	0.12944	0.634056794	1.06355439	0.8252364	1.3706956	0.999221
LILRB4	Inverse variance weighted	3	0.014137	0.029708	0.634178787	1.01423716	0.9568665	1.0750476	0.999221
OVCH1	Wald ratio	1	-0.06059	0.127513	0.634660602	0.941208	0.7330686	1.2084441	0.999221
CYP20A1	Wald ratio	1	-0.13166	0.277098	0.634698324	0.87664251	0.5092745	1.5090133	0.999221
INPP5E	Inverse variance weighted	2	0.023202	0.048934	0.635393	1.02347326	0.9298719	1.1264966	0.999221
THBD	Inverse variance weighted	4	0.017497	0.036962	0.635939553	1.01765125	0.9465338	1.094112	0.999221
FLT4	Inverse variance weighted	3	-0.02207	0.046667	0.636233247	0.97816958	0.8926687	1.0718598	0.999221
IL1RL1	Inverse variance weighted	2	0.023282	0.049268	0.636527044	1.02355531	0.9293374	1.1273252	0.999221
IL18	Inverse variance weighted	2	0.017305	0.036691	0.63717643	1.0174558	0.9468556	1.0933201	0.999221
PRSS53	Wald ratio	1	-0.02768	0.058763	0.63758762	0.97269793	0.866878	1.0914353	0.999221
VIT	Inverse variance weighted	2	0.018827	0.040032	0.638136887	1.0190056	0.942108	1.1021798	0.999221
CYP4F3	Inverse variance weighted	3	-0.01409	0.029976	0.638347135	0.98600983	0.9297482	1.045676	0.999221
MARK3	Inverse variance weighted	6	-0.01638	0.034911	0.638900427	0.98375199	0.9186898	1.0534219	0.999221
PTPN6	Wald ratio	1	-0.13634	0.290616	0.638972569	0.87254739	0.4936417	1.5422907	0.999221
IL15RA	Inverse variance weighted	4	0.019415	0.041393	0.6390425	1.01960466	0.9401502	1.105774	0.999221
TRPM2	Inverse variance weighted	2	0.041322	0.088119	0.639118006	1.04218753	0.8768744	1.2386665	0.999221
CLEC11A	Inverse variance weighted	4	-0.01647	0.035274	0.640480257	0.98366116	0.9179519	1.054074	0.999221
HSPH1	Wald ratio	1	0.025895	0.055489	0.640738382	1.02623283	0.9204773	1.1441388	0.999221
CTSC	Inverse variance weighted	6	-0.01313	0.02817	0.641061154	0.98695252	0.9339365	1.042978	0.999221
IGFBP7	Inverse variance weighted	3	-0.01774	0.038063	0.641190122	0.98241769	0.9117931	1.0585127	0.999221
CYP3A5	Inverse variance weighted	2	0.103637	0.222391	0.641207818	1.10919733	0.7173081	1.7151887	0.999221
CD300A	Inverse variance weighted	5	0.014162	0.030397	0.641299104	1.01426231	0.9555989	1.076527	0.999221
CHL1	Inverse variance weighted	2	0.043565	0.093519	0.641325992	1.04452811	0.869592	1.2546562	0.999221
CD1D	Inverse variance weighted	2	0.049866	0.107137	0.641616488	1.05113003	0.8520393	1.296741	0.999221
SLC5A9	Wald ratio	1	-0.08357	0.179563	0.64163873	0.91982642	0.6469319	1.3078356	0.999221
RTN4RL1	Wald ratio	1	0.0518	0.111447	0.642082758	1.05316458	0.8465064	1.3102743	0.999221
HDAC2	Inverse variance weighted	2	-0.1512	0.325573	0.642342082	0.85967177	0.4541503	1.6272928	0.999221
IFIT1	Inverse variance weighted	2	0.032605	0.070225	0.642433409	1.03314287	0.9002923	1.1855974	0.999221
FLVCR1	Inverse variance weighted	4	-0.0174	0.037487	0.642452966	0.98274641	0.9131284	1.0576721	0.999221
RYR2	Wald ratio	1	0.023623	0.050951	0.642908297	1.02390381	0.9265931	1.1314341	0.999221
CPM	Inverse variance weighted	2	-0.03316	0.071541	0.642984855	0.96738251	0.8408169	1.1129997	0.999221
PRKAB1	Inverse variance weighted	7	0.011258	0.024318	0.643389844	1.01132199	0.9642498	1.0606921	0.999221
GPR142	Wald ratio	1	0.124357	0.268611	0.643390915	1.13242011	0.6688999	1.9171409	0.999221
DUSP19	Wald ratio	1	0.027917	0.060322	0.643500622	1.02831073	0.9136454	1.1573669	0.999221
RAD51	Wald ratio	1	-0.07886	0.170399	0.643519795	0.9241715	0.6617688	1.2906213	0.999221
MCOLN1	Inverse variance weighted	2	0.05659	0.12261	0.644409195	1.05822155	0.8321641	1.3456876	0.999221
SERPINE1	Wald ratio	1	-0.06601	0.143012	0.644412334	0.93612587	0.7072946	1.238991	0.999221
CYP46A1	Wald ratio	1	0.040077	0.086833	0.644412334	1.04089057	0.877994	1.2340098	0.999221
NOTCH2NL	Wald ratio	1	0.023649	0.05124	0.644412334	1.02393093	0.926093	1.1321051	0.999221
KLRC2	Inverse variance weighted	4	-0.0112	0.024269	0.644459277	0.98886291	0.9429263	1.0370374	0.999221
MAK	Inverse variance weighted	5	0.019245	0.041708	0.644493228	1.01943152	0.9394107	1.1062687	0.999221
KCNQ5	Inverse variance weighted	3	0.047723	0.103632	0.64515264	1.04888023	0.8560767	1.2851064	0.999221
AMD1	Wald ratio	1	0.048414	0.10515	0.645206418	1.04960561	0.8541231	1.289828	0.999221
BTN3A2	Inverse variance weighted	8	0.009173	0.01995	0.645670637	1.00921512	0.9705135	1.0494601	0.999221
SLC25A10	Wald ratio	1	-0.13037	0.28374	0.645904258	0.87777309	0.5033356	1.5307592	0.999221
PGLYRP2	Inverse variance weighted	2	0.032207	0.07012	0.646011696	1.03273107	0.9001189	1.1848807	0.999221
BOK	Inverse variance weighted	3	-0.01963	0.042785	0.646406504	0.98056337	0.9016883	1.0663381	0.999221
B2M	Wald ratio	1	-0.05578	0.121745	0.646832229	0.94574736	0.7449777	1.2006239	0.999221
ALDH5A1	Inverse variance weighted	5	-0.01797	0.039222	0.646867055	0.98219215	0.9095154	1.0606763	0.999221
CPA5	Inverse variance weighted	7	0.016891	0.036883	0.646966575	1.01703494	0.9461082	1.0932788	0.999221
SDR42E1	Wald ratio	1	0.05478	0.119705	0.647220357	1.05630861	0.8354022	1.3356296	0.999221
NUAK1	Inverse variance weighted	4	-0.02792	0.061076	0.64762814	0.97247055	0.8627546	1.0961389	0.999221
RNF123	Wald ratio	1	-0.05983	0.13105	0.648014842	0.94192751	0.7285609	1.2177807	0.999221
S1PR4	Inverse variance weighted	2	-0.06424	0.140943	0.648550327	0.93778135	0.7114243	1.2361594	0.999221
PRKCH	Inverse variance weighted	4	-0.04212	0.092826	0.650010782	0.95875527	0.7992688	1.1500657	0.999221
ANGPTL6	Inverse variance weighted	2	-0.02409	0.053249	0.650965097	0.97619699	0.8794502	1.0835867	0.999221
MOK	Inverse variance weighted	2	-0.02426	0.05366	0.651148651	0.97602872	0.8785901	1.0842736	0.999221
NISCH	Wald ratio	1	0.060743	0.13441	0.651324399	1.06262557	0.8165232	1.382904	0.999221
ADCK3	Inverse variance weighted	4	0.012784	0.028341	0.65193561	1.01286618	0.9581364	1.0707222	0.999221
TRAC	Wald ratio	1	-0.03468	0.077222	0.65332162	0.96591035	0.8302416	1.1237485	0.999221
BMP6	Inverse variance weighted	2	0.028021	0.062388	0.653329849	1.02841738	0.9100464	1.1621851	0.999221
RPS6KA5	Inverse variance weighted	3	0.034644	0.077146	0.653381057	1.0352512	0.8899743	1.2042426	0.999221
STYK1	Inverse variance weighted	3	-0.03578	0.079696	0.653462765	0.96485247	0.8253199	1.1279751	0.999221

KCNN3	Inverse variance weighted	2	-0.06829	0.152478	0.654263187	0.93399242	0.6927099	1.2593178	0.999221
PKN1	Inverse variance weighted	3	-0.06767	0.15134	0.654777134	0.93456936	0.6946859	1.2572875	0.999221
MMRN2	Wald ratio	1	0.070326	0.157355	0.654927498	1.07285836	0.7881316	1.4604478	0.999221
SERPINB8	Inverse variance weighted	2	-0.02358	0.052773	0.654999065	0.97669538	0.88072	1.0831296	0.999221
SLC25A46	Inverse variance weighted	5	-0.02631	0.058897	0.655070728	0.97403216	0.8678391	1.0932195	0.999221
DUSP12	Inverse variance weighted	3	-0.07274	0.162837	0.655099118	0.92984478	0.6757729	1.2794406	0.999221
RYK	Inverse variance weighted	2	0.035972	0.080537	0.655121892	1.03662721	0.885255	1.213883	0.999221
BSG	Inverse variance weighted	4	-0.01724	0.038613	0.655246157	0.98290752	0.9112645	1.060183	0.999221
NR1D2	Inverse variance weighted	2	0.032215	0.07224	0.655638679	1.03273937	0.8963939	1.1898236	0.999221
TGFB1	Wald ratio	1	-0.09521	0.213647	0.655848517	0.90917998	0.5981222	1.3820055	0.999221
PPIH	Wald ratio	1	-0.03839	0.086159	0.655926269	0.96233972	0.8128085	1.13938	0.999221
PLAT	Wald ratio	1	0.06229	0.139807	0.655926269	1.06427113	0.8091821	1.3997752	0.999221
CD151	Inverse variance weighted	6	0.008209	0.018442	0.656237666	1.00824266	0.9724488	1.045354	0.999221
EFTUD2	Inverse variance weighted	3	0.015802	0.035632	0.657412206	1.01592781	0.947398	1.0894147	0.999221
TAF15	Wald ratio	1	0.057135	0.128858	0.657479165	1.05879892	0.8224837	1.363012	0.999221
PSMA4	Inverse variance weighted	3	-0.03006	0.067946	0.658180976	0.97038616	0.8493909	1.1086172	0.999221
CD164L2	Wald ratio	1	0.023364	0.052862	0.658497202	1.02363941	0.9228904	1.1353868	0.999221
MAST3	Inverse variance weighted	3	-0.02257	0.051149	0.659043663	0.97768417	0.8844226	1.0807801	0.999221
SIK1	Inverse variance weighted	2	-0.03751	0.08506	0.659206326	0.96318265	0.8152751	1.1379237	0.999221
POGLUT1	Inverse variance weighted	4	0.012657	0.028703	0.659232461	1.01273779	0.9573359	1.0713458	0.999221
MAP4K1	Inverse variance weighted	2	-0.06155	0.140021	0.660238163	0.940305	0.7146289	1.2372485	0.999221
IL1R1	Inverse variance weighted	2	-0.03073	0.069993	0.660601545	0.9697349	0.8454231	1.1123256	0.999221
MAN2B2	Inverse variance weighted	5	-0.01346	0.030678	0.660875914	0.98663179	0.9290559	1.0477758	0.999221
PRSS36	Wald ratio	1	-0.03597	0.082008	0.66095394	0.96467056	0.8214328	1.1328855	0.999221
BCL2L14	Inverse variance weighted	2	0.02207	0.050334	0.661039438	1.02231554	0.9262754	1.1283135	0.999221
SIGLEC6	Inverse variance weighted	2	0.033353	0.076078	0.661089842	1.03391568	0.8906895	1.2001732	0.999221
GPA33	Inverse variance weighted	3	0.054212	0.123735	0.661290736	1.05570883	0.8283587	1.3454571	0.999221
EARS2	Wald ratio	1	-0.03275	0.074771	0.661418031	0.96778404	0.8358576	1.1205329	0.999221
HNRNPUL1	Inverse variance weighted	2	-0.02324	0.0531	0.661623537	0.97702742	0.8804543	1.0841932	0.999221
MTMR7	Wald ratio	1	0.101181	0.23155	0.662129631	1.10647732	0.7028189	1.7419737	0.999221
ATG4B	Inverse variance weighted	2	-0.02825	0.064698	0.662383248	0.97214631	0.8563656	1.1035806	0.999221
TMPRSS9	Inverse variance weighted	4	0.014688	0.033642	0.662404806	1.01479654	0.9500401	1.0839669	0.999221
ATP1B3	Inverse variance weighted	2	0.050216	0.115031	0.662445818	1.05149787	0.8392506	1.3174227	0.999221
RIPK2	Inverse variance weighted	4	-0.01635	0.037466	0.662554559	0.98378305	0.9141288	1.0587448	0.999221
FGF2	Inverse variance weighted	2	0.032907	0.075693	0.663742369	1.03345494	0.8909652	1.1987327	0.999221
PM20D1	Inverse variance weighted	4	-0.01887	0.0435	0.664441678	0.98130696	0.9011076	1.0686441	0.999221
ABI3BP	Wald ratio	1	-0.05447	0.125605	0.664558367	0.94699072	0.7403352	1.2113316	0.999221
HMGB1	Wald ratio	1	0.069711	0.160762	0.664558367	1.07219827	0.782405	1.4693274	0.999221
ARSG	Inverse variance weighted	4	0.031183	0.071968	0.664800279	1.03167479	0.8959468	1.1879644	0.999221
FBLN1	Wald ratio	1	0.101043	0.233523	0.665238319	1.10632472	0.7000098	1.7484818	0.999221
SCNN1A	Inverse variance weighted	3	0.076302	0.17659	0.665678701	1.07928841	0.7635218	1.5256454	0.999221
ADAMTS1	Inverse variance weighted	2	-0.01786	0.041422	0.666391113	0.98230118	0.9057023	1.0653783	0.999221
FN1	Inverse variance weighted	4	-0.0254	0.058937	0.66652177	0.97492231	0.8685641	1.0943044	0.999221
CYP2U1	Inverse variance weighted	4	0.028198	0.065682	0.667696034	1.02859951	0.9043499	1.1699199	0.999221
SLC18A1	Inverse variance weighted	4	-0.01361	0.031737	0.66798768	0.98648005	0.9269869	1.0497914	0.999221
GFPT1	Inverse variance weighted	6	0.013332	0.031123	0.668386085	1.01342146	0.9534485	1.0771668	0.999221
PTPRK	Wald ratio	1	-0.15349	0.358396	0.668461204	0.85771199	0.4248824	1.7314668	0.999221
KHK	Inverse variance weighted	5	-0.02821	0.065953	0.668856317	0.9721848	0.854296	1.1063417	0.999221
ITGA4	Inverse variance weighted	3	0.013769	0.032238	0.669312417	1.01386384	0.9517832	1.0799937	0.999221
SLC4A4	Wald ratio	1	0.076939	0.180428	0.669797149	1.07997663	0.7582819	1.5381477	0.999221
SPRYD4	Inverse variance weighted	6	0.02141	0.050271	0.67018325	1.02164108	0.9257779	1.1274307	0.999221
OR2B6	Wald ratio	1	-0.08267	0.194225	0.670369441	0.92065472	0.6291714	1.3471769	0.999221
SLC25A32	Wald ratio	1	0.043718	0.102738	0.670448961	1.04468803	0.8541502	1.2777297	0.999221
CD84	Inverse variance weighted	3	-0.01673	0.039377	0.67093959	0.98340942	0.9103656	1.062314	0.999221
SPACA3	Wald ratio	1	-0.02711	0.063864	0.671230056	0.9732562	0.8587459	1.103036	0.999221
SPSB1	Inverse variance weighted	3	0.020593	0.048555	0.671484561	1.02080612	0.9281381	1.1227263	0.999221
FCER2	Inverse variance weighted	2	-0.02561	0.060522	0.672133471	0.97471102	0.8656822	1.0974715	0.999221
IFNAR1	Inverse variance weighted	3	0.015762	0.037245	0.672146783	1.01588698	0.9443697	1.0928203	0.999221
ITGA5	Inverse variance weighted	2	-0.11355	0.268555	0.672418826	0.89265659	0.5273339	1.511065	0.999221
P2RY13	Inverse variance weighted	4	0.014145	0.033479	0.672672177	1.01424504	0.9498271	1.0830318	0.999221
PLSCR1	Inverse variance weighted	2	0.01613	0.038366	0.674173682	1.0162609	0.9426433	1.0956278	0.999221
PTBP1	Inverse variance weighted	2	-0.05913	0.140792	0.674493891	0.94258314	0.7152779	1.2421227	0.999221
ASIC1	Inverse variance weighted	2	-0.05049	0.120233	0.674517571	0.95076087	0.7511499	1.2034165	0.999221

TCN2	Inverse variance weighted	4	0.01401	0.033468	0.675494024	1.01410906	0.9497208	1.0828626	0.999221
SLCO3A1	Inverse variance weighted	5	-0.01062	0.025389	0.67561432	0.98943215	0.9414009	1.039914	0.999221
CXCL9	Wald ratio	1	-0.03725	0.089067	0.675814188	0.96343891	0.8091123	1.1472011	0.999221
CD164	Inverse variance weighted	2	-0.01975	0.047319	0.676333162	0.98043935	0.893597	1.0757213	0.999221
EFNA4	Wald ratio	1	0.074472	0.178452	0.676442288	1.07731542	0.7593486	1.5284265	0.999221
GFER	Wald ratio	1	-0.06481	0.155532	0.676922239	0.93725008	0.6909772	1.2712977	0.999221
SLC7A5	Inverse variance weighted	4	0.022018	0.052845	0.676934345	1.02226218	0.9216786	1.1338226	0.999221
CBLN3	Inverse variance weighted	5	-0.01144	0.027466	0.676973294	0.98862291	0.9368089	1.0433027	0.999221
IGLV5-37	Wald ratio	1	-0.03635	0.087526	0.677955798	0.96430699	0.8122909	1.1447721	0.999221
CD3D	Inverse variance weighted	2	0.054821	0.132282	0.67856129	1.05635198	0.8150937	1.3690199	0.999221
AGRN	Inverse variance weighted	3	-0.08104	0.1956	0.678640896	0.92215617	0.6285016	1.3530148	0.999221
CDC42BPG	Wald ratio	1	-0.04549	0.109868	0.678864777	0.95553213	0.7704128	1.185133	0.999221
CCNB1	Wald ratio	1	0.03348	0.080975	0.679268496	1.03404666	0.8822936	1.211901	0.999221
IL20RB	Wald ratio	1	-0.0774	0.187311	0.679443136	0.92551838	0.6411249	1.3360647	0.999221
PLAA	Inverse variance weighted	2	-0.04352	0.105357	0.679521014	0.95740893	0.7787821	1.1770069	0.999221
TNFAIP6	Inverse variance weighted	4	-0.01414	0.034231	0.679604192	0.985962	0.9219816	1.0543822	0.999221
RARB	Wald ratio	1	0.06932	0.167908	0.679720885	1.07177873	0.7712219	1.4894672	0.999221
TLK1	Inverse variance weighted	2	-0.0474	0.11493	0.680040795	0.95370781	0.7613513	1.1946635	0.999221
THBS4	Wald ratio	1	-0.07494	0.181777	0.680133692	0.9277964	0.6497127	1.3249028	0.999221
CTSD	Inverse variance weighted	3	-0.02278	0.055266	0.680198614	0.97747719	0.8771281	1.0893068	0.999221
PGGT1B	Inverse variance weighted	2	0.016833	0.040854	0.680317664	1.01697566	0.938717	1.1017586	0.999221
SLC37A2	Inverse variance weighted	2	-0.01343	0.032633	0.680623051	0.98665747	0.9255253	1.0518275	0.999221
DAGLB	Inverse variance weighted	6	0.010721	0.026066	0.680838299	1.01077915	0.960436	1.0637612	0.999221
TSSK4	Inverse variance weighted	2	-0.06617	0.161289	0.681618146	0.93597207	0.6822935	1.2839691	0.999221
CROT	Inverse variance weighted	3	0.018409	0.044949	0.682138979	1.01857922	0.9326815	1.112388	0.999221
LAMP1	Inverse variance weighted	2	0.054028	0.132016	0.682355531	1.05551395	0.814872	1.3672205	0.999221
CCL2	Inverse variance weighted	2	-0.12495	0.305407	0.68245701	0.88254451	0.4850299	1.605849	0.999221
PTGS1	Inverse variance weighted	2	0.035349	0.086641	0.683275583	1.03598151	0.8741817	1.2277284	0.999221
GSN	Inverse variance weighted	5	-0.01108	0.027188	0.68358581	0.98898017	0.9376592	1.0431101	0.999221
DHRS7	Inverse variance weighted	2	0.032883	0.080699	0.683661587	1.03342926	0.8822429	1.2105237	0.999221
TRIM21	Inverse variance weighted	2	0.032404	0.079795	0.68467371	1.03293506	0.883385	1.2078028	0.999221
CRIM1	Inverse variance weighted	2	-0.0245	0.060512	0.685545157	0.97579609	0.8666636	1.0986709	0.999221
PARP12	Inverse variance weighted	2	-0.03673	0.090851	0.685980021	0.96393372	0.8067014	1.1518118	0.999221
ELAVL1	Wald ratio	1	0.095217	0.235536	0.686024978	1.09989706	0.6932022	1.7451957	0.999221
IGLV1-44	Wald ratio	1	-0.01841	0.045602	0.686496252	0.98176282	0.8978207	1.0735532	0.999221
TMED9	Inverse variance weighted	4	0.010405	0.025829	0.687068337	1.01045921	0.9605781	1.0629306	0.999221
MTOR	Inverse variance weighted	3	0.018085	0.044933	0.687330284	1.01824914	0.9324089	1.1119921	0.999221
PSMD14	Wald ratio	1	-0.0665	0.165468	0.687780321	0.93566627	0.6765064	1.2941065	0.999221
SLC2A6	Inverse variance weighted	2	0.034504	0.086116	0.688668973	1.03510574	0.8743412	1.22543	0.999221
FAM3C	Wald ratio	1	0.020972	0.052431	0.689156517	1.02119391	0.9214631	1.1317187	0.999221
TXN2	Wald ratio	1	-0.03786	0.094645	0.689156517	0.96284974	0.7998255	1.1591024	0.999221
MUC4	Inverse variance weighted	2	0.02056	0.051419	0.68926393	1.02077275	0.9229128	1.1290091	0.999221
PRPSAP2	Inverse variance weighted	3	0.016372	0.04097	0.689432418	1.01650724	0.9380727	1.1014999	0.999221
AIMP1	Inverse variance weighted	2	-0.04991	0.124995	0.689667942	0.95131392	0.7446052	1.2154068	0.999221
DUSP22	Wald ratio	1	0.07106	0.178166	0.690008205	1.0736459	0.7571876	1.5223644	0.999221
PRKCQ	Inverse variance weighted	4	-0.02441	0.061216	0.690057879	0.97588411	0.8655462	1.1002876	0.999221
ASH2L	Wald ratio	1	-0.20829	0.522343	0.690074553	0.81197458	0.2916858	2.2603186	0.999221
PIP5K1A	Wald ratio	1	-0.02825	0.07085	0.690135568	0.97214944	0.8461055	1.1169701	0.999221
CACNG6	Wald ratio	1	-0.0439	0.110253	0.690520985	0.95705236	0.7710564	1.1879147	0.999221
DCT	Wald ratio	1	-0.03285	0.082506	0.690520985	0.9676842	0.8231963	1.1375327	0.999221
PLCG2	Inverse variance weighted	4	-0.01966	0.049395	0.690656714	0.98053452	0.8900554	1.0802114	0.999221
HSD17B1	Inverse variance weighted	2	-0.02414	0.060673	0.690773407	0.97615288	0.8667063	1.0994203	0.999221
DNAJA1	Wald ratio	1	-0.08647	0.218164	0.691836788	0.91716096	0.5980542	1.406535	0.999221
SLC25A11	Inverse variance weighted	3	-0.01753	0.044233	0.691889077	0.98262346	0.9010214	1.0716159	0.999221
LRRC4B	Inverse variance weighted	2	0.046571	0.117876	0.692779992	1.04767271	0.8315477	1.3199701	0.999221
ELANE	Wald ratio	1	0.041843	0.105914	0.692797333	1.04273031	0.847259	1.2832989	0.999221
PPARG	Inverse variance weighted	4	0.016163	0.040916	0.692814097	1.01629483	0.9379747	1.1011546	0.999221
CD302	Inverse variance weighted	7	0.009056	0.022925	0.69282499	1.00909717	0.9647585	1.0554736	0.999221
H1F0	Inverse variance weighted	5	-0.00918	0.023233	0.69284241	0.99086475	0.9467551	1.0370295	0.999221
BGLAP	Wald ratio	1	0.053858	0.136441	0.69303711	1.05533503	0.8076988	1.3788952	0.999221
IFNG	Inverse variance weighted	2	0.035051	0.08894	0.693507101	1.03567271	0.8699924	1.232905	0.999221
DMPK	Inverse variance weighted	2	0.014243	0.036176	0.693796742	1.01434449	0.944914	1.0888766	0.999221
GAN	Wald ratio	1	0.076779	0.195286	0.694199544	1.07980356	0.7364002	1.5833452	0.999221

LY96	Inverse variance weighted	2	0.015346	0.039037	0.694230001	1.01546456	0.9406671	1.0962096	0.999221
MME	Inverse variance weighted	2	-0.02564	0.065215	0.694239414	0.97468918	0.8577364	1.1075885	0.999221
GIF	Inverse variance weighted	3	-0.01767	0.044982	0.694458032	0.98248587	0.8995747	1.0730387	0.999221
IL15	Inverse variance weighted	2	-0.02032	0.05174	0.694573441	0.97988887	0.8853901	1.0844736	0.999221
CTSO	Inverse variance weighted	4	-0.02615	0.066667	0.694869748	0.97418865	0.8548607	1.1101734	0.999221
MAP4K2	Inverse variance weighted	2	-0.05437	0.13881	0.695282189	0.94708024	0.7214883	1.2432094	0.999221
UBE2N	Wald ratio	1	-0.06299	0.161137	0.695864434	0.93895276	0.6846691	1.2876764	0.999221
TNFRSF13B	Wald ratio	1	0.081948	0.209788	0.696074444	1.08539972	0.7194735	1.6374371	0.999221
ACVR2A	Inverse variance weighted	2	-0.01728	0.04423	0.696080898	0.9828716	0.9012553	1.071879	0.999221
EPAS1	Inverse variance weighted	2	-0.04014	0.102831	0.696260201	0.96065267	0.7852993	1.1751615	0.999221
TKT	Inverse variance weighted	4	-0.01054	0.027065	0.696928092	0.98951453	0.9383922	1.0434219	0.999221
IGLV4-60	Inverse variance weighted	5	0.00488	0.012536	0.697085618	1.0048918	0.9805012	1.0298891	0.999221
BCL2L13	Inverse variance weighted	4	0.014407	0.03702	0.697145247	1.01451159	0.9435068	1.09086	0.999221
ADORA2B	Inverse variance weighted	4	-0.02409	0.061981	0.697509542	0.97619674	0.8645256	1.1022924	0.999221
DARC	Inverse variance weighted	2	0.016433	0.042333	0.697877329	1.01656885	0.9356261	1.1045141	0.999221
TPCN2	Inverse variance weighted	5	0.010168	0.026196	0.697905439	1.01021987	0.9596599	1.0634436	0.999221
TRPV4	Inverse variance weighted	2	0.03134	0.081154	0.699362399	1.03183633	0.8800987	1.2097351	0.999221
GMPR2	Inverse variance weighted	2	-0.07912	0.205321	0.699979197	0.92392882	0.6178251	1.3816928	0.999221
UCP3	Inverse variance weighted	2	0.026017	0.067573	0.700227474	1.02635807	0.8990405	1.1717058	0.999221
STK36	Wald ratio	1	-0.02368	0.061576	0.700522394	0.97659526	0.8655661	1.1018664	0.999221
MAP3K14	Wald ratio	1	-0.05648	0.146859	0.700522394	0.94508139	0.7086965	1.2603121	0.999221
SLC46A2	Inverse variance weighted	3	-0.01247	0.032448	0.700656645	0.98760342	0.9267497	1.0524531	0.999221
PDIA3	Wald ratio	1	-0.03666	0.095481	0.701031389	0.96400593	0.7994748	1.1623975	0.999221
TEX101	Inverse variance weighted	3	-0.01768	0.046077	0.70114472	0.98247216	0.8976326	1.0753303	0.999221
PTPRO	Inverse variance weighted	2	-0.0544	0.142232	0.702106251	0.9470524	0.7166439	1.2515397	0.999221
KLRK1	Inverse variance weighted	3	-0.01032	0.027126	0.703494542	0.98972873	0.9384821	1.0437737	0.999221
GSR	Inverse variance weighted	3	0.017144	0.045047	0.703509261	1.01729218	0.9313241	1.1111958	0.999221
SLC6A13	Inverse variance weighted	2	-0.06084	0.159879	0.703528358	0.94097016	0.6878348	1.2872639	0.999221
MANF	Inverse variance weighted	4	0.044527	0.117137	0.703848981	1.0455336	0.831053	1.3153679	0.999221
GAK	Wald ratio	1	0.061939	0.163518	0.704845389	1.06389706	0.7721653	1.4658481	0.999221
OLFM2	Wald ratio	1	-0.0991	0.261622	0.704845389	0.90565299	0.5423318	1.5123718	0.999221
PLD1	Inverse variance weighted	2	0.033756	0.08912	0.704853578	1.03433265	0.8685606	1.2317438	0.999221
EDAR	Inverse variance weighted	2	-0.055	0.145383	0.705180163	0.94648141	0.711802	1.258534	0.999221
TAPBPL	Inverse variance weighted	10	0.009312	0.024654	0.705657002	1.00935528	0.9617406	1.0593274	0.999221
LIMK1	Inverse variance weighted	6	0.007207	0.01911	0.706091464	1.00723264	0.9702038	1.0456748	0.999221
SPNS2	Inverse variance weighted	4	-0.01297	0.034392	0.706132765	0.98711602	0.9227692	1.0559499	0.999221
CD7	Inverse variance weighted	2	0.036342	0.096407	0.706199318	1.03701059	0.8584595	1.2526986	0.999221
HIST4H4	Wald ratio	1	-0.02267	0.060267	0.706740247	0.9775803	0.8686641	1.1001529	0.999221
BOLA3	Wald ratio	1	-0.07513	0.199686	0.706740247	0.92762358	0.6271854	1.3719795	0.999221
PODNL1	Wald ratio	1	0.069981	0.186176	0.707001259	1.07248806	0.7445877	1.5447888	0.999221
PHKG1	Inverse variance weighted	3	0.012368	0.032911	0.707058257	1.01244516	0.9491982	1.0799064	0.999221
COCH	Inverse variance weighted	2	0.014606	0.038872	0.707106348	1.01471306	0.9402751	1.0950439	0.999221
MAP3K2	Inverse variance weighted	4	-0.0173	0.046067	0.707263723	0.98284915	0.8979952	1.0757212	0.999221
LILRB5	Wald ratio	1	0.049068	0.130848	0.707660467	1.05029174	0.8126994	1.3573441	0.999221
BACE1	Inverse variance weighted	4	-0.01536	0.040983	0.707845265	0.98475873	0.9087491	1.0671259	0.999221
C4BPB	Inverse variance weighted	4	0.015629	0.041774	0.708309422	1.0157516	0.9358984	1.1024181	0.999221
IGFBP6	Wald ratio	1	-0.04507	0.120579	0.708563233	0.95592964	0.7547213	1.21078	0.999221
GRIN3A	Wald ratio	1	0.029417	0.078711	0.708599717	1.02985417	0.8826239	1.2016439	0.999221
PRADC1	Wald ratio	1	-0.01608	0.043021	0.708599717	0.98405005	0.904476	1.0706249	0.999221
HHIPL1	Wald ratio	1	0.064702	0.173519	0.709236749	1.06684124	0.7592714	1.4990031	0.999221
ATM	Inverse variance weighted	2	0.047208	0.12662	0.70927707	1.04833955	0.8179382	1.3436416	0.999221
ERCC5	Inverse variance weighted	2	0.018047	0.048494	0.709779679	1.01821081	0.9258895	1.1197376	0.999221
NR3C1	Wald ratio	1	0.063135	0.170191	0.710661558	1.06517107	0.7630443	1.4869246	0.999221
PMS2	Inverse variance weighted	3	0.028591	0.077149	0.71093696	1.02900395	0.8845989	1.1969821	0.999221
BMP1	Inverse variance weighted	3	0.047336	0.127883	0.711272613	1.04847379	0.816021	1.3471433	0.999221
SPNS1	Inverse variance weighted	5	-0.01239	0.033494	0.711405449	0.98768457	0.9249266	1.0547008	0.999221
HABP4	Inverse variance weighted	2	0.031234	0.084638	0.712108289	1.03172664	0.8740153	1.2178961	0.999221
CARM1	Inverse variance weighted	2	0.058948	0.160208	0.712911195	1.0607204	0.7748708	1.4520198	0.999221
CASP1	Inverse variance weighted	3	0.015028	0.040859	0.713020197	1.0151416	0.9370152	1.099782	0.999221
FDFT1	Inverse variance weighted	3	-0.01386	0.03772	0.713339169	0.986238	0.9159534	1.0619158	0.999221
CSNK1G2	Wald ratio	1	0.025735	0.070055	0.713360246	1.02606855	0.8944256	1.177087	0.999221
SLC29A1	Inverse variance weighted	2	-0.05669	0.154736	0.714112486	0.94489107	0.697699	1.2796623	0.999221
BRD3	Inverse variance weighted	2	-0.01335	0.036497	0.714553416	0.98674014	0.9186207	1.0599109	0.999221

PRMT3	Inverse variance weighted	3	-0.03711	0.101472	0.714610998	0.96357475	0.7897887	1.1756008	0.999221
DECR1	Inverse variance weighted	3	0.007179	0.019647	0.714832462	1.00720439	0.9691558	1.0467467	0.999221
IARS	Inverse variance weighted	2	-0.0146	0.04	0.715170304	0.98550908	0.9111958	1.065883	0.999221
CSNK1G3	Inverse variance weighted	3	-0.04039	0.110764	0.715342387	0.96041039	0.7729878	1.1932765	0.999221
TMEM123	Inverse variance weighted	2	-0.02994	0.082129	0.715481931	0.97050741	0.8262081	1.140009	0.999221
FMO4	Inverse variance weighted	3	0.01723	0.047402	0.716244323	1.01737897	0.9271151	1.1164309	0.999221
HIPK1	Wald ratio	1	-0.04835	0.133286	0.716795308	0.95280133	0.7337481	1.2372508	0.999221
NPFF	Wald ratio	1	-0.10004	0.275796	0.716795308	0.90479787	0.5269739	1.5535098	0.999221
CDKN3	Inverse variance weighted	2	0.032522	0.089707	0.716952634	1.03305652	0.8664907	1.2316414	0.999221
CD247	Inverse variance weighted	2	-0.01384	0.038216	0.717261603	0.98625633	0.9150804	1.0629683	0.999221
SLC25A35	Inverse variance weighted	2	-0.03894	0.107539	0.717304223	0.96181212	0.779024	1.1874891	0.999221
ADA	Inverse variance weighted	3	0.008909	0.024612	0.717363701	1.00894912	0.9614324	1.0588143	0.999221
MST1P2	Inverse variance weighted	4	0.007709	0.021315	0.71758143	1.00773913	0.9665065	1.0507308	0.999221
OXSR1	Inverse variance weighted	3	0.035061	0.096961	0.717651045	1.03568312	0.8564297	1.2524549	0.999221
ITPR2	Inverse variance weighted	5	-0.01023	0.028311	0.717879998	0.98982338	0.9363937	1.0463017	0.999221
IGKV5-2	Wald ratio	1	-0.09307	0.257927	0.718230465	0.91113287	0.5495783	1.5105456	0.999221
ATP1A4	Inverse variance weighted	3	-0.01191	0.033003	0.71824732	0.98816316	0.926267	1.0541955	0.999221
MFSD5	Inverse variance weighted	2	0.052403	0.145297	0.718354782	1.05380003	0.7926448	1.4009988	0.999221
PDCD1LG2	Inverse variance weighted	2	0.017338	0.048153	0.718799642	1.01748914	0.9258519	1.1181963	0.999221
DZANK1	Wald ratio	1	-0.07015	0.194869	0.718847134	0.93225141	0.6362934	1.3658678	0.999221
CDH13	Inverse variance weighted	2	0.025217	0.070117	0.719118981	1.02553721	0.8938543	1.1766196	0.999221
HLA-DRB1	Inverse variance weighted	5	0.003452	0.00963	0.719980577	1.00345824	0.9846954	1.0225785	0.999221
YES1	Wald ratio	1	0.026087	0.072911	0.720494364	1.02643054	0.8897475	1.1841108	0.999221
TNKS	Inverse variance weighted	2	0.043337	0.121175	0.720615265	1.04428952	0.8235203	1.3242425	0.999221
HLA-DQA2	Inverse variance weighted	3	0.005001	0.013985	0.720628563	1.00501372	0.9778404	1.0329422	0.999221
TXN	Inverse variance weighted	4	-0.01265	0.035387	0.720753234	0.98743065	0.9212658	1.0583474	0.999221
EIF2AK3	Wald ratio	1	0.044279	0.12398	0.720984862	1.04527356	0.8197774	1.332797	0.999221
VAT1	Inverse variance weighted	4	0.018737	0.052487	0.721100617	1.01891401	0.9193045	1.1293165	0.999221
DNASE1L3	Inverse variance weighted	2	0.009936	0.027854	0.721313938	1.00998518	0.9563241	1.0666573	0.999221
OLFM4	Inverse variance weighted	4	-0.01928	0.054086	0.721544429	0.98090847	0.8822451	1.0906055	0.999221
HEBP1	Inverse variance weighted	4	0.013142	0.036878	0.721566129	1.01322876	0.9425762	1.0891772	0.999221
PPIL1	Inverse variance weighted	2	-0.02253	0.0635	0.722769172	0.97772464	0.8633054	1.1073086	0.999221
MFSD1	Inverse variance weighted	5	-0.01485	0.041894	0.722907271	0.98525521	0.9075863	1.0695709	0.999221
DHODH	Inverse variance weighted	2	0.044048	0.124368	0.723207853	1.04503263	0.8189652	1.3335038	0.999221
ABCC1	Inverse variance weighted	4	0.01407	0.039762	0.723444485	1.01416972	0.9381326	1.0963697	0.999221
CTNNB1	Inverse variance weighted	4	0.013689	0.038783	0.724109251	1.01378357	0.9395766	1.0938513	0.999221
S1PR1	Inverse variance weighted	2	-0.04338	0.123573	0.725547682	0.95754667	0.7515753	1.2199651	0.999221
ADCY10P1	Inverse variance weighted	9	0.011488	0.032802	0.726185313	1.01155376	0.9485649	1.0787254	0.999221
GPR17	Wald ratio	1	-0.04307	0.123069	0.726338698	0.9578404	0.752549	1.2191342	0.999221
ENC1	Inverse variance weighted	7	-0.01757	0.050317	0.726886265	0.98257912	0.8903001	1.0844228	0.999221
DNAJC16	Inverse variance weighted	2	-0.04407	0.126655	0.727851451	0.95688309	0.7465311	1.2265064	0.999221
MBNL1	Inverse variance weighted	5	0.013349	0.038443	0.728404987	1.01343888	0.9398833	1.092751	0.999221
SLC1A7	Inverse variance weighted	4	0.012425	0.035788	0.728444393	1.01250299	0.9439149	1.0860749	0.999221
SCN9A	Inverse variance weighted	5	0.016517	0.047592	0.728546833	1.01665436	0.9261094	1.1160518	0.999221
DAPK3	Inverse variance weighted	2	-0.02359	0.067994	0.728627398	0.9766853	0.8548239	1.1159189	0.999221
CD1C	Inverse variance weighted	3	0.024797	0.071489	0.728694157	1.025107	0.8910792	1.179294	0.999221
XCL1	Inverse variance weighted	4	0.009227	0.026605	0.728723667	1.00927012	0.9579887	1.0632967	0.999221
KMO	Inverse variance weighted	3	-0.01442	0.041593	0.728883685	0.98568685	0.9085197	1.0694084	0.999221
TNFRSF13C	Inverse variance weighted	3	-0.01698	0.049197	0.729925177	0.98315931	0.892784	1.0826832	0.999221
KBTBD11	Inverse variance weighted	5	-0.01043	0.030235	0.730095506	0.98962314	0.9326811	1.0500416	0.999221
SUOX	Inverse variance weighted	7	-0.01534	0.044493	0.73018367	0.98477236	0.9025322	1.0745063	0.999221
AKR1E2	Inverse variance weighted	4	0.01592	0.046326	0.731105529	1.01604763	0.9278557	1.1126221	0.999221
JMJD7-PLA2G4I	Wald ratio	1	0.03281	0.095534	0.731271727	1.03335395	0.856897	1.2461478	0.999221
CDH2	Inverse variance weighted	5	-0.01244	0.03625	0.731546312	0.98764072	0.9199036	1.0603657	0.999221
CD1E	Inverse variance weighted	4	0.018266	0.053501	0.73279599	1.0184336	0.917047	1.1310293	0.999221
SENP6	Inverse variance weighted	6	0.012013	0.035197	0.732873814	1.01208526	0.9446199	1.0843691	0.999221
ITGB7	Inverse variance weighted	4	-0.013	0.038157	0.73326068	0.98708071	0.9159527	1.0637322	0.999221
KCNB1	Inverse variance weighted	2	0.038201	0.112241	0.733598092	1.03893965	0.8337742	1.2945899	0.999221
EPHB4	Inverse variance weighted	13	0.010445	0.030696	0.733666575	1.01049928	0.9514953	1.0731622	0.999221
AKR1A1	Wald ratio	1	-0.03503	0.10303	0.733856528	0.96557642	0.7890166	1.1816453	0.999221
DYRK2	Wald ratio	1	-0.07357	0.216507	0.734002761	0.92907076	0.6077909	1.4201799	0.999221
MASTL	Inverse variance weighted	8	0.010076	0.029657	0.734042485	1.01012713	0.9530839	1.0705845	0.999221
MAN2A2	Inverse variance weighted	4	0.015539	0.04575	0.734127532	1.01565995	0.9285494	1.1109427	0.999221

HMMR	Wald ratio	1	-0.0861	0.253637	0.734271103	0.91750519	0.558095	1.5083735	0.999221
GPRC5B	Wald ratio	1	0.057138	0.168627	0.734728025	1.05880182	0.7608112	1.4735079	0.999221
KCNJ12	Wald ratio	1	0.02242	0.066211	0.734892745	1.02267372	0.8982093	1.1643851	0.999221
CHST8	Inverse variance weighted	2	-0.02358	0.069667	0.735040367	0.97669862	0.8520384	1.1195976	0.999221
LTBP1	Inverse variance weighted	2	-0.06315	0.186969	0.735533731	0.93879978	0.650762	1.3543278	0.999221
HTRA2	Inverse variance weighted	2	0.052192	0.154545	0.735579204	1.05357801	0.7782428	1.4263243	0.999221
PTK2B	Inverse variance weighted	4	-0.01017	0.030141	0.735757613	0.98987974	0.9330954	1.0501197	0.999221
AURKC	Inverse variance weighted	2	-0.03412	0.101333	0.736340201	0.96645645	0.7923669	1.178795	0.999221
TMED10	Wald ratio	1	0.030261	0.090027	0.736769478	1.03072371	0.863992	1.229631	0.999221
PGBD1	Inverse variance weighted	3	-0.0231	0.068831	0.737190644	0.97716675	0.8538443	1.1183008	0.999221
TAC3	Inverse variance weighted	2	-0.04265	0.127125	0.73722656	0.95824293	0.7469042	1.2293807	0.999221
KRT18	Inverse variance weighted	2	0.03111	0.092923	0.737779451	1.03159909	0.8598318	1.2376801	0.999221
LIPA	Inverse variance weighted	7	0.009319	0.027883	0.738211471	1.00936282	0.95568	1.0660611	0.999221
CST7	Inverse variance weighted	4	0.009516	0.028474	0.73822012	1.00956186	0.954762	1.0675071	0.999221
NQO2	Inverse variance weighted	4	-0.00935	0.027971	0.738222388	0.99069538	0.9378441	1.046525	0.999221
LPHN1	Wald ratio	1	0.022716	0.068148	0.73888268	1.02297602	0.8950692	1.169161	0.999221
GPBAR1	Inverse variance weighted	3	0.013387	0.040229	0.739308346	1.01347687	0.9366353	1.0966225	0.999221
TSSK3	Wald ratio	1	-0.05857	0.176284	0.739710426	0.943114	0.6675873	1.3323562	0.999221
FCRL3	Inverse variance weighted	8	-0.00782	0.023545	0.739754991	0.99220941	0.9474614	1.0390709	0.999221
CTSH	Inverse variance weighted	5	0.011672	0.035143	0.739804972	1.01173994	0.944396	1.0838861	0.999221
BST2	Inverse variance weighted	2	0.045166	0.136017	0.739844417	1.04620108	0.8013745	1.3658243	0.999221
PLA2G4B	Inverse variance weighted	4	0.011877	0.035782	0.739944324	1.01194768	0.9434093	1.0854653	0.999221
MAG	Wald ratio	1	-0.04039	0.12213	0.740864559	0.96041541	0.7559624	1.2201636	0.999221
RLN1	Wald ratio	1	0.019527	0.059059	0.740912529	1.01971937	0.9082573	1.1448601	0.999221
TUBB	Inverse variance weighted	6	-0.01368	0.041419	0.741107465	0.98640899	0.9094958	1.0698265	0.999221
CEL	Wald ratio	1	-0.02471	0.074843	0.7412574	0.97559038	0.8424797	1.1297324	0.999221
ITFG1	Wald ratio	1	0.030051	0.091038	0.741326604	1.03050731	0.8621015	1.2318101	0.999221
IGLV4-69	Wald ratio	1	-0.01392	0.042281	0.74206958	0.98618125	0.9077511	1.0713878	0.999221
CAPN10	Inverse variance weighted	2	0.024573	0.07471	0.742219604	1.02487776	0.8852735	1.1864971	0.999221
ABCC2	Inverse variance weighted	2	0.033137	0.100814	0.74238282	1.03369251	0.8483541	1.2595215	0.999221
CD320	Inverse variance weighted	5	0.014638	0.044545	0.742443513	1.01474601	0.9299078	1.1073243	0.999221
LTA4H	Inverse variance weighted	8	-0.0091	0.027722	0.74260534	0.99093737	0.9385319	1.0462691	0.999221
LAP3	Inverse variance weighted	6	0.015838	0.048302	0.742995821	1.01596364	0.9241937	1.1168461	0.999221
INPP5B	Inverse variance weighted	11	-0.00976	0.029777	0.743070447	0.99028675	0.9341443	1.0498034	0.999221
PROK2	Inverse variance weighted	3	-0.01312	0.040048	0.743152439	0.9869629	0.9124555	1.0675542	0.999221
PSKH1	Wald ratio	1	-0.08383	0.255905	0.743224552	0.91958664	0.5568802	1.5185306	0.999221
TNFRSF18	Inverse variance weighted	4	0.012147	0.037115	0.743445177	1.01222156	0.9412019	1.0886001	0.999221
GZMB	Inverse variance weighted	5	0.011186	0.034201	0.743612683	1.01124887	0.9456838	1.0813596	0.999221
VCP	Inverse variance weighted	5	0.014284	0.043685	0.743687846	1.01438618	0.9311472	1.1050663	0.999221
MATK	Inverse variance weighted	3	-0.02152	0.065852	0.743785598	0.97870672	0.8601981	1.1135421	0.999221
IGKV3-20	Wald ratio	1	-0.03972	0.121759	0.74425828	0.96105819	0.7570175	1.2200945	0.999221
AURKB	Inverse variance weighted	2	0.014459	0.044362	0.744470366	1.01456429	0.9300752	1.1067285	0.999221
CIT	Wald ratio	1	-0.08243	0.252933	0.744496011	0.920874	0.5609184	1.5118223	0.999221
TUFT1	Inverse variance weighted	2	-0.03159	0.097014	0.744745013	0.96890813	0.8011291	1.1718248	0.999221
MAP2K5	Inverse variance weighted	3	0.009328	0.028687	0.745064352	1.00937134	0.9541839	1.0677506	0.999221
CD28	Wald ratio	1	0.031253	0.096164	0.745181072	1.03174697	0.8545084	1.2457476	0.999221
ABCC5	Inverse variance weighted	9	0.006903	0.021276	0.745586217	1.00692709	0.9658013	1.049804	0.999221
RBKS	Inverse variance weighted	2	-0.07317	0.225701	0.745792653	0.92944237	0.5971754	1.446582	0.999221
DCXR	Inverse variance weighted	4	0.009184	0.028339	0.745871774	1.00922676	0.9546974	1.0668706	0.999221
SMPDL3B	Wald ratio	1	-0.02178	0.067254	0.746082245	0.97845795	0.8576191	1.116323	0.999221
P2RY11	Inverse variance weighted	5	0.016444	0.050826	0.746291857	1.01657977	0.9201903	1.123066	0.999221
ABCA7	Inverse variance weighted	4	-0.01316	0.040707	0.746439828	0.98692413	0.9112409	1.0688932	0.999221
TGFB3	Inverse variance weighted	2	0.013162	0.040736	0.746616831	1.01324904	0.9354935	1.0974674	0.999221
PAK4	Inverse variance weighted	3	-0.01646	0.050991	0.746842773	0.98367461	0.8901171	1.0870656	0.999221
IL32	Inverse variance weighted	5	0.008883	0.027574	0.747343504	1.00892222	0.9558431	1.0649488	0.999221
PPIB	Wald ratio	1	-0.07151	0.222249	0.747650331	0.93099058	0.6022313	1.4392202	0.999221
GPC1	Wald ratio	1	0.039475	0.12307	0.748396278	1.04026464	0.8173064	1.324045	0.999221
CD27	Inverse variance weighted	3	0.028626	0.089256	0.748420943	1.02903999	0.8638855	1.2257682	0.999221
MKNK1	Inverse variance weighted	3	-0.02477	0.077285	0.748557814	0.97553122	0.8384066	1.1350831	0.999221
FLRT1	Wald ratio	1	0.05711	0.178469	0.748968331	1.05877239	0.7462539	1.5021682	0.999221
TK1	Wald ratio	1	0.063783	0.19955	0.749245264	1.06586119	0.7208423	1.5760175	0.999221
CPT1B	Inverse variance weighted	4	0.008962	0.02807	0.74951128	1.00900261	0.9549894	1.0660708	0.999221
NID2	Wald ratio	1	0.043579	0.136961	0.750347024	1.04454202	0.7986238	1.3661852	0.999221

BPHL	Inverse variance weighted	2	0.037832	0.118937	0.750420513	1.03855666	0.8226012	1.3112063	0.999221
CCNE1	Wald ratio	1	0.028614	0.090051	0.750669263	1.02902771	0.8625295	1.2276658	0.999221
HPN	Wald ratio	1	0.065375	0.205738	0.750669263	1.06755885	0.7132871	1.5977885	0.999221
MARS2	Inverse variance weighted	2	-0.01527	0.048077	0.75080938	0.98484801	0.896283	1.0821644	0.999221
CHRNA10	Inverse variance weighted	2	-0.03465	0.109519	0.751704214	0.96594235	0.7793392	1.1972253	0.999221
S100A12	Inverse variance weighted	4	-0.00934	0.029596	0.752235403	0.99070038	0.9348674	1.0498678	0.999221
GPNMB	Inverse variance weighted	5	0.009321	0.02955	0.752437261	1.00936447	0.9525647	1.0695511	0.999221
IL21R	Inverse variance weighted	3	0.011414	0.0362	0.752541366	1.01147903	0.9421986	1.0858537	0.999221
CDK14	Inverse variance weighted	2	0.042566	0.135126	0.752754218	1.04348483	0.8006908	1.3599014	0.999221
NQO1	Inverse variance weighted	2	-0.01765	0.056053	0.752836423	0.98250372	0.8802803	1.0965979	0.999221
KCNAB1	Wald ratio	1	-0.01459	0.046329	0.752902263	0.98552064	0.8999726	1.0792006	0.999221
KLHL17	Inverse variance weighted	2	0.048178	0.153117	0.753029728	1.04935714	0.7772983	1.4166382	0.999221
HIPK3	Inverse variance weighted	2	0.031042	0.098709	0.753154942	1.03152909	0.8500776	1.2517119	0.999221
PF4	Wald ratio	1	0.055701	0.177588	0.753784497	1.05728171	0.7464906	1.4974664	0.999221
MARK4	Inverse variance weighted	5	0.010441	0.033291	0.753812015	1.01049532	0.9466652	1.0786293	0.999221
DNAJB11	Wald ratio	1	0.046018	0.147817	0.755556791	1.04709376	0.7837206	1.3989748	0.999221
PARP15	Inverse variance weighted	5	-0.01197	0.038512	0.755982458	0.98810319	0.9162625	1.0655767	0.999221
SLC4A5	Wald ratio	1	-0.03331	0.107218	0.756024926	0.96723568	0.7839109	1.1934326	0.999221
HNRNPU	Wald ratio	1	-0.06007	0.193353	0.756044198	0.94169788	0.6446535	1.3756148	0.999221
VRK1	Wald ratio	1	-0.01272	0.041045	0.756560956	0.98735653	0.9110358	1.0700709	0.999221
HLA-G	Inverse variance weighted	10	0.008771	0.028353	0.757059885	1.0088093	0.9542778	1.066457	0.999221
IL11RA	Inverse variance weighted	10	0.007477	0.024188	0.75723023	1.00750517	0.9608545	1.0564208	0.999221
MIPEP	Wald ratio	1	-0.02757	0.089589	0.758316474	0.97281052	0.8161468	1.1595467	0.999221
MUC6	Wald ratio	1	-0.04888	0.159089	0.758669138	0.95229849	0.6971942	1.3007457	0.999221
SMG1	Wald ratio	1	-0.02333	0.076097	0.759171191	0.97694086	0.8415749	1.1340802	0.999221
STK24	Inverse variance weighted	2	-0.01902	0.062222	0.759832635	0.98115842	0.8685095	1.1084183	0.999221
TOP2B	Inverse variance weighted	2	-0.0242	0.079287	0.760201447	0.97609069	0.835602	1.1401996	0.999221
THBS1	Inverse variance weighted	6	-0.00789	0.025886	0.760493095	0.99214002	0.9430572	1.0437774	0.999221
LAMB3	Inverse variance weighted	2	0.026144	0.085805	0.760600711	1.02648893	0.8675917	1.2144878	0.999221
NPBWR1	Wald ratio	1	0.021887	0.071863	0.76070109	1.02212785	0.8878391	1.1767282	0.999221
CD6	Inverse variance weighted	3	-0.0277	0.091087	0.761019847	0.97267695	0.8136433	1.162795	0.999221
CD300LD	Inverse variance weighted	4	0.014657	0.048282	0.761456312	1.01476499	0.923138	1.1154865	0.999221
SERPINI1	Inverse variance weighted	3	0.023624	0.077831	0.761483723	1.02390552	0.8790404	1.1926443	0.999221
GALC	Inverse variance weighted	4	-0.01287	0.042578	0.762465595	0.9872135	0.9081718	1.0731345	0.999221
ACLY	Wald ratio	1	0.021492	0.071127	0.762531411	1.02172435	0.8887689	1.1745693	0.999221
TP63	Inverse variance weighted	2	-0.02672	0.088459	0.762626025	0.97363615	0.8186513	1.1579623	0.999221
THRA	Inverse variance weighted	4	-0.0174	0.057617	0.762672716	0.98275169	0.8778072	1.1002426	0.999221
KLK1	Inverse variance weighted	2	-0.01964	0.065168	0.763113961	0.98055034	0.8629739	1.1141461	0.999221
TMEM66	Inverse variance weighted	2	-0.01876	0.062379	0.763602181	0.98141409	0.8684683	1.1090486	0.999221
VIM	Inverse variance weighted	3	0.020157	0.067025	0.763616241	1.02036138	0.8947485	1.1636089	0.999221
PARP16	Inverse variance weighted	4	0.010029	0.03346	0.764387985	1.01007924	0.9459618	1.0785425	0.999221
KLHL32	Wald ratio	1	-0.05204	0.173966	0.764829184	0.94928976	0.6750185	1.3350019	0.999221
PNMT	Wald ratio	1	0.04895	0.163676	0.76489013	1.05016779	0.7619641	1.447381	0.999221
SLC3A2	Inverse variance weighted	2	0.047018	0.157484	0.765277297	1.04814084	0.7697806	1.4271589	0.999221
ATP7B	Inverse variance weighted	3	0.027022	0.090509	0.765278996	1.02739037	0.8603848	1.2268126	0.999221
ITGAD	Inverse variance weighted	3	-0.01022	0.034255	0.765437842	0.9898322	0.9255572	1.0585707	0.999221
TMPRSS3	Inverse variance weighted	3	0.0178	0.059704	0.765594818	1.01795967	0.9055437	1.1443312	0.999221
TWSG1	Inverse variance weighted	2	-0.01974	0.066232	0.765618338	0.98044925	0.8610881	1.1163558	0.999221
TRIM10	Inverse variance weighted	2	0.020056	0.067355	0.765881612	1.02025844	0.8940806	1.1642432	0.999221
CELSR3	Wald ratio	1	-0.01898	0.063922	0.766489644	0.98119627	0.8656548	1.1121594	0.999221
BAX	Inverse variance weighted	4	0.011788	0.039829	0.767262087	1.01185751	0.9358712	1.0940134	0.999221
CDK8	Inverse variance weighted	2	0.017206	0.058191	0.767475053	1.0173547	0.9076938	1.1402641	0.999221
IGLL1	Wald ratio	1	-0.04324	0.146464	0.767812003	0.95767978	0.7186997	1.2761249	0.999221
CA2	Inverse variance weighted	3	-0.01137	0.038512	0.767860876	0.98869663	0.9168135	1.0662158	0.999221
MLX	Inverse variance weighted	3	0.020853	0.070656	0.767888207	1.02107226	0.8890227	1.1727356	0.999221
CD33	Inverse variance weighted	4	0.015341	0.052122	0.768511504	1.015459	0.9168433	1.1246819	0.999221
LATS2	Inverse variance weighted	3	-0.01572	0.053422	0.768546176	0.98440199	0.8865404	1.0930661	0.999221
SLC25A37	Inverse variance weighted	5	-0.01623	0.055234	0.768926133	0.98390427	0.882951	1.0964001	0.999221
SLC4A10	Wald ratio	1	-0.1337	0.45511	0.768934772	0.87485523	0.3585413	2.1346822	0.999221
RNPEPL1	Wald ratio	1	-0.06424	0.218762	0.769024758	0.93778031	0.6107836	1.4398422	0.999221
GSTZ1	Inverse variance weighted	3	-0.01545	0.05265	0.769246155	0.98467333	0.8881288	1.0917128	0.999221
P2RX7	Inverse variance weighted	7	0.015266	0.052148	0.769719291	1.01538291	0.9167289	1.1246535	0.999221
DNPEP	Inverse variance weighted	2	0.017749	0.060785	0.770295839	1.01790701	0.90358	1.1466994	0.999221

DAPK2	Inverse variance weighted	3	0.016544	0.056658	0.770296337	1.01668114	0.9098214	1.1360917	0.999221
ALDH1A2	Inverse variance weighted	3	0.044489	0.152487	0.770471946	1.04549375	0.7753929	1.4096817	0.999221
ABCC4	Inverse variance weighted	4	0.008521	0.029233	0.770675459	1.00855755	0.9523951	1.0680319	0.999221
NR1D1	Wald ratio	1	-0.01448	0.049677	0.770752505	0.98562897	0.8941861	1.0864232	0.999221
SLPI	Inverse variance weighted	3	0.01867	0.064123	0.770930342	1.0188453	0.8985157	1.1552895	0.999221
ADAM11	Wald ratio	1	0.046866	0.161173	0.771219474	1.04798144	0.7641181	1.4372977	0.999221
ACP6	Inverse variance weighted	4	0.0191	0.065757	0.771458287	1.01928399	0.8960276	1.1594954	0.999221
PEPD	Inverse variance weighted	2	0.017089	0.05907	0.772350512	1.01723593	0.9060251	1.1420975	0.999221
FCGR2C	Inverse variance weighted	7	0.006125	0.021175	0.772399822	1.00614349	0.9652395	1.0487808	0.999221
HLA-DRA	Inverse variance weighted	3	-0.02007	0.069412	0.772513329	0.98013372	0.8554617	1.122975	0.999221
LIPH	Inverse variance weighted	3	-0.0265	0.091863	0.772993779	0.97384902	0.8133847	1.1659697	0.999221
SLC18A2	Wald ratio	1	0.03967	0.13759	0.773100905	1.04046753	0.7945284	1.3625349	0.999221
RBCK1	Inverse variance weighted	5	0.015623	0.05434	0.77372942	1.0157453	0.9131247	1.1298989	0.999221
LRG1	Wald ratio	1	-0.05942	0.206829	0.773872338	0.94230657	0.6282554	1.4133451	0.999221
CCL28	Wald ratio	1	-0.02214	0.077103	0.774013768	0.97810478	0.8409187	1.1376712	0.999221
CLCN6	Inverse variance weighted	3	-0.00965	0.033631	0.774166123	0.99039671	0.9272183	1.0578799	0.999221
TUSC3	Inverse variance weighted	2	-0.0207	0.072227	0.774375879	0.97950867	0.8502129	1.128467	0.999221
CSNK1G1	Inverse variance weighted	2	-0.01364	0.047619	0.774540125	0.98645269	0.8985506	1.0829539	0.999221
DNASE1	Inverse variance weighted	2	0.011383	0.039763	0.774668917	1.01144807	0.9356141	1.0934286	0.999221
BRPF3	Inverse variance weighted	2	-0.03664	0.128103	0.774854351	0.96402171	0.7499689	1.2391685	0.999221
XCL2	Inverse variance weighted	4	-0.0036	0.012617	0.775332791	0.9964056	0.972068	1.0213526	0.999221
USP47	Inverse variance weighted	2	-0.01341	0.047071	0.775682335	0.98667657	0.899719	1.0820386	0.999221
EMILIN1	Wald ratio	1	0.076168	0.267816	0.776101047	1.07914383	0.6384242	1.8241027	0.999221
CTSK	Inverse variance weighted	5	0.009247	0.032611	0.776757188	1.00928978	0.946796	1.0759085	0.999221
SGSH	Inverse variance weighted	2	0.010027	0.035397	0.776965016	1.01007751	0.9423763	1.0826424	0.999221
NDUFA4	Wald ratio	1	0.051804	0.183164	0.777308473	1.05316918	0.7355057	1.5080309	0.999221
ANXA2	Inverse variance weighted	3	-0.01089	0.03849	0.777312403	0.98917325	0.9172955	1.0666832	0.999221
APOD	Wald ratio	1	0.022675	0.080335	0.777745638	1.02293409	0.8739077	1.1973738	0.999221
COL4A3	Wald ratio	1	0.024292	0.086125	0.777904188	1.02458922	0.8654428	1.213001	0.999221
OR13A1	Inverse variance weighted	3	-0.01058	0.037608	0.778562896	0.98948061	0.9191675	1.0651725	0.999221
TST	Inverse variance weighted	3	-0.01386	0.049348	0.778850024	0.98623781	0.895315	1.0863941	0.999221
CDCP1	Inverse variance weighted	2	0.03258	0.116072	0.778951303	1.03311636	0.8228991	1.2970355	0.999221
CALM2	Inverse variance weighted	3	0.023213	0.082702	0.778957485	1.02348419	0.8703293	1.2035903	0.999221
ENPP5	Inverse variance weighted	2	-0.02721	0.096962	0.778970185	0.97315339	0.804721	1.1768396	0.999221
MAP3K11	Inverse variance weighted	3	0.008995	0.032068	0.779097113	1.00903552	0.9475663	1.0744923	0.999221
TAB1	Wald ratio	1	-0.05684	0.203003	0.779477505	0.9447445	0.6346218	1.4064159	0.999221
CD2	Inverse variance weighted	2	-0.04568	0.163585	0.780071339	0.95535025	0.6932919	1.3164645	0.999221
GPR42	Inverse variance weighted	2	-0.00939	0.033658	0.780295148	0.99065557	0.9274115	1.0582126	0.999221
SLC6A19	Wald ratio	1	0.033568	0.120999	0.781458122	1.03413725	0.8157961	1.3109156	0.999221
PARP1	Inverse variance weighted	3	0.008002	0.028866	0.781614789	1.00803407	0.9525864	1.0667092	0.999221
TGFBR2	Inverse variance weighted	3	0.010259	0.037035	0.781766245	1.01031224	0.9395731	1.0863772	0.999221
NT5E	Inverse variance weighted	9	-0.00608	0.022008	0.782192833	0.99393406	0.9519714	1.0377464	0.999221
MAPK7	Wald ratio	1	-0.0254	0.091895	0.782223374	0.974918	0.8142267	1.1673224	0.999221
PLK1	Inverse variance weighted	2	-0.01762	0.063777	0.782355846	0.9825358	0.8670817	1.1133628	0.999221
IFNGR1	Inverse variance weighted	2	-0.01949	0.070634	0.782571367	0.98069597	0.8539053	1.126313	0.999221
LRRK2	Inverse variance weighted	4	0.036445	0.132323	0.782989048	1.03711733	0.8001891	1.3441977	0.999221
MAPK8	Inverse variance weighted	2	-0.01425	0.051769	0.78304045	0.98584615	0.8907232	1.0911276	0.999221
MAP2K4	Wald ratio	1	0.021835	0.0794	0.783316238	1.02207514	0.8747746	1.1941792	0.999221
CACNB1	Inverse variance weighted	2	-0.04853	0.177266	0.784270201	0.95263062	0.6730274	1.3483925	0.999221
CACNA1D	Inverse variance weighted	2	-0.04531	0.165868	0.784719023	0.95570024	0.6904494	1.3228529	0.999221
BOLA1	Wald ratio	1	-0.03107	0.113928	0.785062869	0.96940658	0.7754054	1.2119456	0.999221
DHRS9	Inverse variance weighted	5	-0.01021	0.037504	0.785405305	0.98984028	0.919689	1.0653425	0.999221
HRH1	Wald ratio	1	-0.03182	0.116866	0.785408865	0.96868108	0.7703761	1.2180324	0.999221
AMICA1	Inverse variance weighted	4	-0.00848	0.031138	0.78547979	0.99156053	0.9328548	1.0539607	0.999221
EGLN1	Wald ratio	1	0.012134	0.044688	0.785988675	1.01220782	0.9273214	1.1048647	0.999221
PDE6C	Wald ratio	1	0.049353	0.182058	0.786326168	1.05059115	0.7352974	1.501082	0.999221
BMP8B	Wald ratio	1	-0.02617	0.09673	0.786707745	0.97416546	0.8059241	1.1775282	0.999221
ABCF1	Inverse variance weighted	2	0.019009	0.070337	0.786961663	1.01919087	0.8879403	1.1698422	0.999221
CDC7	Inverse variance weighted	6	0.007299	0.027016	0.787020619	1.00732594	0.9553742	1.0621028	0.999221
TNFRSF21	Wald ratio	1	0.050104	0.185942	0.78757489	1.05138065	0.7302687	1.5136911	0.999221
IGLV1-51	Inverse variance weighted	2	-0.01234	0.045803	0.787662826	0.98773878	0.9029287	1.0805149	0.999221
IGLV1-40	Wald ratio	1	0.044986	0.167389	0.788122072	1.04601296	0.7534471	1.452183	0.999221
GPR141	Inverse variance weighted	3	-0.02502	0.093136	0.788237875	0.97529399	0.8125619	1.1706165	0.999221

SIRT2	Wald ratio	1	0.038119	0.141959	0.788300222	1.03885435	0.7865331	1.3721206	0.999221
CD1B	Inverse variance weighted	3	0.018545	0.069333	0.789099273	1.01871835	0.8892756	1.1670027	0.999221
NPTX2	Inverse variance weighted	2	-0.0695	0.260741	0.789806873	0.93285678	0.5595867	1.5551151	0.999221
OMG	Inverse variance weighted	2	-0.03523	0.132352	0.79011062	0.96538547	0.7448009	1.2512996	0.999221
ADAM9	Wald ratio	1	-0.01722	0.065051	0.791236046	0.98292794	0.8652645	1.1165919	0.999221
LGALS8	Inverse variance weighted	4	0.014484	0.054999	0.792282291	1.01458927	0.9109073	1.1300726	0.999221
ITGB4	Wald ratio	1	0.064514	0.245571	0.792772671	1.06664096	0.6591496	1.7260467	0.999221
IL24	Wald ratio	1	-0.01793	0.068259	0.792838656	0.98223323	0.8592346	1.122839	0.999221
GRM2	Inverse variance weighted	4	0.013441	0.051218	0.792998173	1.01353137	0.9167257	1.1205596	0.999221
RNASE2	Inverse variance weighted	5	0.005062	0.019307	0.793169151	1.00507496	0.9677527	1.0438366	0.999221
SLC2A11	Inverse variance weighted	3	0.010583	0.040366	0.793195061	1.01063883	0.9337601	1.0938471	0.999221
DUSP5	Inverse variance weighted	3	0.015047	0.057426	0.79329632	1.01516126	0.9070956	1.1361012	0.999221
PDE5A	Inverse variance weighted	3	0.027152	0.104028	0.794086922	1.02752393	0.8379958	1.2599173	0.999221
MAPK6	Inverse variance weighted	2	-0.02775	0.106345	0.794136938	0.97263151	0.7896332	1.1980398	0.999221
KCNT1	Inverse variance weighted	2	-0.01242	0.047694	0.794620984	0.98766127	0.8995182	1.0844414	0.999221
CSNK1A1L	Wald ratio	1	0.028848	0.110953	0.794863774	1.02926783	0.8281014	1.2793026	0.999221
ASIC3	Wald ratio	1	-0.01558	0.059971	0.794985251	0.98453765	0.8753537	1.1073403	0.999221
CYP27B1	Wald ratio	1	-0.0713	0.274652	0.795160468	0.93117899	0.543557	1.5952226	0.999221
FNTA	Wald ratio	1	-0.04301	0.165755	0.795254365	0.95789949	0.6921915	1.3256034	0.999221
PRCP	Inverse variance weighted	3	0.015683	0.060468	0.795355377	1.01580672	0.9022769	1.1436215	0.999221
ITLN2	Wald ratio	1	-0.01751	0.067774	0.796149663	0.98264407	0.8604106	1.1222425	0.999221
CD9	Inverse variance weighted	4	-0.00587	0.022822	0.796965393	0.99414569	0.9506567	1.0396241	0.999221
TRIM16	Wald ratio	1	-0.01671	0.065095	0.79745888	0.98343309	0.8656359	1.1172603	0.999221
ERN1	Inverse variance weighted	4	-0.0103	0.040269	0.798204388	0.98975724	0.9146422	1.0710411	0.999221
FKBP2	Wald ratio	1	0.041868	0.163779	0.798229526	1.04275717	0.756435	1.4374566	0.999221
TNFSF10	Inverse variance weighted	2	0.013326	0.052181	0.798429279	1.01341511	0.914893	1.1225468	0.999221
KCNA2	Inverse variance weighted	2	-0.04394	0.172122	0.798502494	0.95701113	0.6829735	1.3410042	0.999221
VWA5A	Inverse variance weighted	4	0.009309	0.03647	0.79853	1.00935255	0.9397203	1.0841445	0.999221
CASP9	Inverse variance weighted	4	0.011051	0.043311	0.798613063	1.01111179	0.9288209	1.1006934	0.999221
SSR1	Inverse variance weighted	6	-0.01005	0.039503	0.799192233	0.99000111	0.9162421	1.0696978	0.999221
SQLE	Wald ratio	1	-0.01888	0.074256	0.799312245	0.98129833	0.8483844	1.1350354	0.999221
CAPN3	Inverse variance weighted	4	0.019001	0.075175	0.800451474	1.01918316	0.8795527	1.1809802	0.999221
DPP7	Inverse variance weighted	2	-0.01168	0.046238	0.800611721	0.98839016	0.9027541	1.0821497	0.999221
HGF	Wald ratio	1	0.04384	0.173898	0.80096312	1.04481496	0.743044	1.4691435	0.999221
PTPRC	Inverse variance weighted	3	-0.02813	0.111579	0.800973524	0.97226423	0.7812793	1.2099357	0.999221
PVRL3	Inverse variance weighted	3	-0.03597	0.142869	0.801201751	0.96466602	0.7290617	1.2764085	0.999221
DDR1	Inverse variance weighted	5	-0.0125	0.050103	0.803038578	0.98758124	0.8952087	1.0894853	0.999221
ENPP2	Inverse variance weighted	3	0.01333	0.053595	0.803577043	1.01341941	0.9123642	1.1256677	0.999221
AIFM3	Inverse variance weighted	2	0.010272	0.041352	0.803826032	1.01032469	0.9316687	1.0956211	0.999221
NDOR1	Wald ratio	1	0.017768	0.071721	0.804340116	1.01792658	0.8844354	1.1715661	0.999221
NUP210	Inverse variance weighted	2	0.009607	0.038793	0.804404524	1.00965336	0.9357315	1.089415	0.999221
ARNT	Inverse variance weighted	3	0.012786	0.051638	0.804438702	1.01286795	0.9153725	1.1207476	0.999221
CLCN1	Inverse variance weighted	4	0.0087	0.03528	0.805221491	1.00873772	0.9413426	1.080958	0.999221
VDR	Inverse variance weighted	3	-0.01322	0.053961	0.806496986	0.98686935	0.8878245	1.0969635	0.999221
SLC4A1	Inverse variance weighted	3	0.027996	0.1147	0.807167695	1.0283916	0.8213425	1.2876349	0.999221
ACRBP	Inverse variance weighted	2	0.029627	0.121477	0.807314231	1.03007051	0.8118276	1.3069835	0.999221
SLC5A11	Inverse variance weighted	3	-0.00782	0.032114	0.807538862	0.99220737	0.9316785	1.0566686	0.999221
PLA2G2C	Inverse variance weighted	2	0.028269	0.116171	0.807742042	1.0286723	0.8192014	1.2917053	0.999221
CETP	Inverse variance weighted	3	0.018487	0.076019	0.80785793	1.01865914	0.877647	1.1823278	0.999221
SERPINB2	Inverse variance weighted	2	0.012843	0.052937	0.808305812	1.0129259	0.9130974	1.1236686	0.999221
LTB	Inverse variance weighted	2	0.042726	0.176526	0.808750896	1.04365191	0.7384031	1.4750876	0.999221
PRRT3	Inverse variance weighted	4	0.016233	0.06721	0.80914746	1.01636537	0.8909222	1.1594711	0.999221
GRK7	Wald ratio	1	0.041026	0.170257	0.80958313	1.04187891	0.7462627	1.4545972	0.999221
HMOX1	Inverse variance weighted	2	0.014406	0.059804	0.80964611	1.01451009	0.9022978	1.1406775	0.999221
ATF1	Inverse variance weighted	4	0.009519	0.039556	0.809839669	1.00956395	0.9342497	1.0909496	0.999221
ITIH3	Wald ratio	1	0.021564	0.089706	0.810032104	1.02179805	0.8570497	1.2182155	0.999221
DYRK1B	Wald ratio	1	0.021247	0.088531	0.810330257	1.02147476	0.8587536	1.2150291	0.999221
LAMB2	Inverse variance weighted	3	-0.02324	0.09701	0.810680238	0.97702928	0.8078502	1.1816377	0.999221
FCER1A	Inverse variance weighted	3	-0.01003	0.041965	0.811062438	0.99001806	0.9118457	1.0748921	0.999221
TNFAIP2	Inverse variance weighted	3	0.022414	0.09379	0.811117275	1.02266753	0.8509394	1.2290521	0.999221
CSAD	Inverse variance weighted	2	0.015764	0.066165	0.811686291	1.01588876	0.89233	1.1565565	0.999221
GSTT1	Inverse variance weighted	4	0.006301	0.026475	0.811872156	1.00632115	0.9554347	1.0599179	0.999221
SLC17A5	Wald ratio	1	-0.02061	0.086835	0.812366862	0.97959861	0.8262903	1.1613515	0.999221

NLRP3	Inverse variance weighted	6	0.010822	0.045646	0.812596118	1.01088045	0.9243684	1.1054892	0.999221
SLC25A3	Inverse variance weighted	3	0.011046	0.046593	0.81260719	1.01110673	0.922861	1.1077907	0.999221
SEMA3C	Inverse variance weighted	4	-0.01429	0.060747	0.813981612	0.98580843	0.8751529	1.1104553	0.999221
ABCA13	Inverse variance weighted	2	-0.03063	0.131167	0.815353948	0.9698337	0.7499735	1.2541474	0.999221
AMPD3	Inverse variance weighted	4	0.011036	0.047322	0.815605062	1.01109669	0.9215339	1.109364	0.999221
KCNAB3	Wald ratio	1	0.014159	0.060767	0.815753867	1.01425993	0.9003755	1.1425491	0.999221
PMS2P1	Wald ratio	1	0.020941	0.090045	0.816104536	1.02116154	0.8559461	1.218267	0.999221
PIK3CD	Wald ratio	1	0.041252	0.178315	0.817048123	1.0421147	0.7347348	1.4780885	0.999221
CCL5	Inverse variance weighted	2	-0.02054	0.088798	0.817113323	0.97967394	0.8231799	1.1659189	0.999221
SPEG	Wald ratio	1	0.019048	0.082541	0.817494086	1.01923049	0.866986	1.1982094	0.999221
OVGP1	Inverse variance weighted	3	0.007402	0.03212	0.817740433	1.00742948	0.9459625	1.0728904	0.999221
IGLV8-61	Inverse variance weighted	4	-0.00549	0.023842	0.817858545	0.99452418	0.9491185	1.0421021	0.999221
ABCC13	Inverse variance weighted	3	-0.01715	0.07449	0.817881106	0.98299343	0.8494614	1.1375162	0.999221
GDF9	Wald ratio	1	0.031441	0.136649	0.818023004	1.03194091	0.7894719	1.348879	0.999221
NUP153	Wald ratio	1	0.030354	0.131923	0.818023004	1.03081924	0.7959536	1.3349877	0.999221
ATP2B2	Wald ratio	1	-0.03677	0.159866	0.81809177	0.96389853	0.7046126	1.3185975	0.999221
GUCY2D	Wald ratio	1	-0.03049	0.132583	0.81809177	0.96996621	0.747997	1.2578052	0.999221
TPSAB1	Inverse variance weighted	4	0.007446	0.032415	0.818315425	1.00747383	0.9454572	1.0735584	0.999221
CHEK2	Wald ratio	1	-0.03565	0.155824	0.81905688	0.96498192	0.7110162	1.3096609	0.999221
CD79B	Wald ratio	1	0.021972	0.096127	0.819202033	1.02221494	0.8466768	1.2341467	0.999221
ENHO	Wald ratio	1	-0.04567	0.199806	0.819202033	0.95535733	0.645785	1.4133306	0.999221
DPEP2	Inverse variance weighted	4	0.009043	0.039583	0.819294371	1.0090838	0.9337566	1.0904877	0.999221
ATP2A1	Wald ratio	1	0.025569	0.112283	0.819861768	1.02589916	0.8232415	1.2784452	0.999221
PPIL3	Inverse variance weighted	5	-0.00626	0.027506	0.820034363	0.99376195	0.9416055	1.0488074	0.999221
TESK1	Inverse variance weighted	2	-0.03133	0.13788	0.820232365	0.96915303	0.7396501	1.2698675	0.999221
TRIM17	Wald ratio	1	0.052775	0.233531	0.821210209	1.05419282	0.6670132	1.6661178	0.999221
BTNL8	Inverse variance weighted	4	0.008182	0.036209	0.821221727	1.00821589	0.9391429	1.0823691	0.999221
PYGB	Inverse variance weighted	5	0.005071	0.02246	0.821358749	1.00508419	0.9617994	1.0503169	0.999221
CCL3	Inverse variance weighted	2	0.009224	0.040884	0.82150737	1.00926644	0.9315463	1.0934709	0.999221
UMPS	Wald ratio	1	-0.01148	0.050933	0.821597951	0.9885808	0.8946581	1.0923637	0.999221
SGK1	Inverse variance weighted	4	-0.00838	0.037198	0.821826651	0.99165804	0.92193	1.0666597	0.999221
MFSD12	Inverse variance weighted	2	0.016372	0.072736	0.821909777	1.01650694	0.8814464	1.1722623	0.999221
EREG	Inverse variance weighted	2	-0.01533	0.068193	0.82209179	0.9847833	0.8615753	1.1256104	0.999221
LDHA	Inverse variance weighted	2	-0.01874	0.083407	0.822194207	0.981431	0.8334166	1.1557326	0.999221
TNK2	Inverse variance weighted	3	-0.01782	0.079333	0.822280674	0.98233857	0.8408748	1.1476013	0.999221
ENTPD6	Inverse variance weighted	3	0.013291	0.059173	0.82228614	1.0133793	0.902408	1.137997	0.999221
SLC12A9	Inverse variance weighted	2	-0.01396	0.062214	0.822401056	0.98613258	0.8729262	1.1140203	0.999221
CYB5R3	Inverse variance weighted	4	0.007136	0.031823	0.822559956	1.00716189	0.9462616	1.0719816	0.999221
TNKS2	Wald ratio	1	-0.02106	0.094052	0.822789725	0.9791565	0.8143164	1.1773647	0.999221
C2orf40	Inverse variance weighted	5	-0.00957	0.042757	0.822802948	0.99047071	0.9108491	1.0770524	0.999221
SLC25A16	Inverse variance weighted	6	0.007491	0.033483	0.82296948	1.00751912	0.9435225	1.0758565	0.999221
MGAM	Inverse variance weighted	3	0.012574	0.056213	0.82299906	1.01265361	0.9070089	1.1306034	0.999221
NCR3	Inverse variance weighted	5	-0.006	0.026896	0.823536968	0.99402015	0.9429761	1.0478272	0.999221
CDC14A	Inverse variance weighted	4	-0.00602	0.02706	0.8238928	0.99399604	0.9426498	1.0481392	0.999221
ALOX15B	Inverse variance weighted	2	-0.02239	0.100764	0.824173186	0.97786088	0.8026105	1.1913772	0.999221
AXIN2	Inverse variance weighted	4	0.008315	0.037525	0.824645339	1.00834923	0.9368479	1.0853077	0.999221
AURKA	Inverse variance weighted	2	0.032666	0.147458	0.824681472	1.03320555	0.7738692	1.3794498	0.999221
XPNPEP3	Wald ratio	1	0.016679	0.07542	0.824972645	1.01681925	0.877092	1.1788061	0.999221
WNK2	Inverse variance weighted	2	-0.02741	0.12398	0.825021956	0.97296151	0.7630657	1.2405932	0.999221
PAK1	Inverse variance weighted	3	0.011335	0.0517	0.826453305	1.01139975	0.913935	1.1192584	0.999221
KEAP1	Inverse variance weighted	3	-0.04301	0.196175	0.82647982	0.95790637	0.6521318	1.407054	0.999221
ORM2	Inverse variance weighted	5	-0.00692	0.031637	0.826975872	0.99310866	0.9333986	1.0566385	0.999221
LY86	Inverse variance weighted	7	0.006167	0.02824	0.827118353	1.00618655	0.9520077	1.0634487	0.999221
PLCB3	Inverse variance weighted	2	0.01342	0.061488	0.827232221	1.01351034	0.8984392	1.1433197	0.999221
CHAD	Wald ratio	1	-0.04736	0.217421	0.827567972	0.95374483	0.6228154	1.4605117	0.999221
TAP1	Wald ratio	1	0.01965	0.090561	0.828223049	1.01984436	0.8539782	1.2179263	0.999221
MCAM	Wald ratio	1	-0.05892	0.271538	0.828223049	0.94278359	0.5537001	1.6052749	0.999221
PARP6	Wald ratio	1	-0.02574	0.118734	0.828342017	0.97458371	0.7722375	1.2299498	0.999221
CCL4	Wald ratio	1	-0.00667	0.030784	0.828468127	0.99335233	0.9351891	1.0551329	0.999221
GSTK1	Inverse variance weighted	3	-0.01226	0.056762	0.829007993	0.98781567	0.88381	1.1040606	0.999221
ITGB1	Wald ratio	1	-0.03055	0.141632	0.829232301	0.96991373	0.7348069	1.2802447	0.999221
STAT3	Inverse variance weighted	3	-0.01683	0.078029	0.829276016	0.98331538	0.8438657	1.1458093	0.999221
HLA-DRB5	Inverse variance weighted	11	-0.00407	0.018897	0.829373532	0.9959358	0.9597223	1.0335158	0.999221

FCGR2A	Wald ratio	1	-0.02225	0.103323	0.829467464	0.97799159	0.7987019	1.1975276	0.999221
PTK6	Inverse variance weighted	2	0.05308	0.246651	0.829608268	1.05451437	0.6502774	1.7100402	0.999221
PTPN7	Inverse variance weighted	4	-0.00707	0.032879	0.829737656	0.99295467	0.9309838	1.0590506	0.999221
SCN3A	Wald ratio	1	0.046531	0.216702	0.829982567	1.04763067	0.6850901	1.6020228	0.999221
APRT	Inverse variance weighted	4	0.009779	0.045566	0.830067362	1.00982709	0.9235504	1.1041636	0.999221
UBA6	Wald ratio	1	0.023015	0.107404	0.830324258	1.02328194	0.8290325	1.2630457	0.999221
OAS1	Inverse variance weighted	10	-0.00518	0.024175	0.830343295	0.99483373	0.9487958	1.0431056	0.999221
CTGF	Inverse variance weighted	2	0.019897	0.092905	0.830415138	1.02009672	0.8502738	1.2238379	0.999221
TUBA1A	Inverse variance weighted	2	0.015025	0.070269	0.830690555	1.01513809	0.8845265	1.1650361	0.999221
DNASE1L2	Wald ratio	1	-0.0311	0.145792	0.831066976	0.9693764	0.7284366	1.2900102	0.999221
CPXM1	Inverse variance weighted	2	0.023388	0.109813	0.831341247	1.02366387	0.8254341	1.269499	0.999221
MGP	Inverse variance weighted	2	-0.01063	0.049951	0.831544548	0.98943058	0.8971519	1.0912008	0.999221
CAPN8	Inverse variance weighted	2	0.015138	0.071176	0.8315717	1.01525327	0.8830562	1.1672407	0.999221
SLC7A6	Inverse variance weighted	4	-0.00726	0.034136	0.831580128	0.99276646	0.9285177	1.061461	0.999221
TUBB1	Wald ratio	1	-0.05131	0.241533	0.831752696	0.94997975	0.5917219	1.5251446	0.999221
TRAP1	Inverse variance weighted	5	0.004526	0.021307	0.83177255	1.00453646	0.9634494	1.0473757	0.999221
ABAT	Inverse variance weighted	6	0.007875	0.037075	0.831782142	1.00790643	0.9372628	1.0838747	0.999221
IGHV1-2	Inverse variance weighted	2	0.017393	0.08198	0.831977768	1.01754549	0.8665049	1.194914	0.999221
CD97	Inverse variance weighted	2	-0.02971	0.140077	0.832057514	0.97073169	0.7376722	1.2774237	0.999221
COL6A3	Inverse variance weighted	2	0.010521	0.049652	0.832190559	1.01057648	0.916863	1.1138685	0.999221
RANBP10	Wald ratio	1	-0.03323	0.157023	0.832404865	0.96731715	0.7110637	1.3159194	0.999221
PPARD	Inverse variance weighted	2	0.024426	0.115565	0.832608654	1.02472626	0.8170278	1.2852242	0.999221
CDK20	Wald ratio	1	-0.04021	0.19099	0.833256915	0.9605892	0.6606382	1.3967276	0.999221
PCCB	Inverse variance weighted	4	0.007538	0.035855	0.833479049	1.00756667	0.9391901	1.0809213	0.999221
CPT1C	Inverse variance weighted	2	-0.01944	0.09253	0.833561889	0.98074398	0.8180739	1.1757602	0.999221
PRKCA	Inverse variance weighted	4	0.009354	0.044561	0.833728515	1.00939833	0.9249773	1.1015243	0.999221
ILK	Inverse variance weighted	2	-0.02333	0.111776	0.834677893	0.97694179	0.7847357	1.2162251	0.999221
PDE7A	Inverse variance weighted	2	0.013984	0.067188	0.835123562	1.01408247	0.8889584	1.1568182	0.999221
POR	Inverse variance weighted	4	0.006751	0.032449	0.835182029	1.00677426	0.9447364	1.0728859	0.999221
TBXA2R	Wald ratio	1	-0.0205	0.098575	0.835290814	0.97971279	0.8075881	1.1885231	0.999221
IL1R2	Inverse variance weighted	3	0.010235	0.049292	0.835509095	1.01028769	0.9172475	1.1127653	0.999221
MCOLN2	Inverse variance weighted	4	0.008353	0.04023	0.835516552	1.00838792	0.9319305	1.0911181	0.999221
ITIH4	Inverse variance weighted	4	0.005262	0.025408	0.835937533	1.00527565	0.9564396	1.0566053	0.999221
NUAK2	Inverse variance weighted	3	0.017775	0.08587	0.836005798	1.01793442	0.8602525	1.204519	0.999221
NEK7	Wald ratio	1	0.024275	0.11733	0.83609065	1.02457218	0.8140843	1.2894833	0.999221
KCNG2	Inverse variance weighted	2	0.023356	0.113192	0.836522319	1.02363135	0.8199601	1.2778929	0.999221
MARK2	Inverse variance weighted	3	0.023297	0.112902	0.836522837	1.02357008	0.8203767	1.277091	0.999221
SLC5A10	Inverse variance weighted	2	0.026336	0.128315	0.837377964	1.0266863	0.7983879	1.3202664	0.999221
OXNAD1	Inverse variance weighted	2	-0.0312	0.152179	0.83754545	0.9692799	0.7193032	1.3061301	0.999221
AXL	Inverse variance weighted	3	0.014938	0.072893	0.83762561	1.01505011	0.8799137	1.1709406	0.999221
CYSLTR2	Wald ratio	1	-0.02855	0.140135	0.838585048	0.97185764	0.7384441	1.2790505	0.999221
ATP2C1	Inverse variance weighted	2	-0.01661	0.081622	0.83878507	0.98353135	0.8381281	1.1541601	0.999221
CDK6	Wald ratio	1	-0.05004	0.24612	0.838903132	0.95119568	0.5871755	1.5408906	0.999221
TSTD1	Inverse variance weighted	3	-0.00583	0.028715	0.839118593	0.99418713	0.9397776	1.0517467	0.999221
TRIM68	Inverse variance weighted	3	0.006908	0.034263	0.840218447	1.00693177	0.9415318	1.0768745	0.999221
FLVCR2	Inverse variance weighted	4	0.011393	0.056577	0.840402238	1.01145847	0.905293	1.1300742	0.999221
ABCA3	Wald ratio	1	0.017226	0.085624	0.840555146	1.01737537	0.8601943	1.2032778	0.999221
TUBA4A	Wald ratio	1	-0.03294	0.16373	0.840555146	0.96759682	0.7019797	1.3337189	0.999221
CD160	Wald ratio	1	0.008917	0.044587	0.841480581	1.00895726	0.9245271	1.1010978	0.999221
COL19A1	Inverse variance weighted	2	0.019443	0.097651	0.842182322	1.01963274	0.8420191	1.2347118	0.999221
CCR7	Inverse variance weighted	2	0.021825	0.109656	0.842234834	1.02206533	0.8243996	1.2671252	0.999221
TRPC3	Inverse variance weighted	3	-0.00945	0.047495	0.842294903	0.99059492	0.9025419	1.0872385	0.999221
HVCN1	Wald ratio	1	0.01094	0.055011	0.842369415	1.01099965	0.907664	1.1260999	0.999221
HSPA1B	Inverse variance weighted	4	-0.00799	0.040292	0.842752474	0.99203905	0.9167097	1.0735584	0.999221
GART	Wald ratio	1	-0.01702	0.085834	0.842829236	0.9831252	0.8308938	1.1632476	0.999221
C5AR1	Inverse variance weighted	2	0.034875	0.176221	0.843117381	1.03549076	0.7330673	1.4626776	0.999221
TRIM34	Inverse variance weighted	2	-0.03057	0.155266	0.843917446	0.96989301	0.7154165	1.3148878	0.999221
MITF	Wald ratio	1	0.027848	0.141559	0.844045632	1.02823909	0.7791059	1.3570372	0.999221
MAP3K12	Wald ratio	1	-0.01537	0.0784	0.844548778	0.98474501	0.8444781	1.1483101	0.999221
F2RL3	Wald ratio	1	-0.01752	0.089333	0.844548778	0.98263622	0.8248042	1.1706705	0.999221
TFF3	Inverse variance weighted	3	0.00966	0.049292	0.844637894	1.00970635	0.9167198	1.1121249	0.999221
KYNU	Inverse variance weighted	6	0.005897	0.030158	0.844981716	1.00591413	0.9481775	1.0671664	0.999221
SERPINF2	Inverse variance weighted	4	-0.00728	0.037255	0.845032918	0.99274467	0.9228388	1.067946	0.999221

ACP1	Inverse variance weighted	2	0.01618	0.082931	0.845309783	1.01631206	0.8638421	1.1956933	0.999221
ALDH4A1	Inverse variance weighted	2	-0.04986	0.256253	0.845731669	0.95136409	0.5757314	1.5720761	0.999221
AGRP	Wald ratio	1	-0.01515	0.077927	0.845827892	0.9849617	0.8454469	1.1474991	0.999221
S100A9	Inverse variance weighted	3	0.006368	0.032764	0.845893014	1.00638831	0.9437926	1.0731355	0.999221
HTRA3	Wald ratio	1	-0.01316	0.067776	0.846039047	0.98692588	0.864157	1.1271363	0.999221
NEK4	Wald ratio	1	-0.03014	0.155435	0.846271762	0.97031413	0.7154894	1.3158959	0.999221
RORC	Inverse variance weighted	2	0.022843	0.118067	0.846588647	1.02310568	0.8117452	1.2894998	0.999221
PPP1CA	Wald ratio	1	0.068867	0.357047	0.84705425	1.07129332	0.5320885	2.1569145	0.999221
TUBB2B	Wald ratio	1	-0.01213	0.062954	0.847224821	0.98794445	0.8732624	1.1176872	0.999221
PAOX	Inverse variance weighted	5	0.010795	0.056056	0.847284601	1.01085389	0.9056762	1.1282461	0.999221
RELT	Inverse variance weighted	2	0.010182	0.052944	0.847500405	1.01023357	0.9106575	1.1206978	0.999221
APOBEC3A	Inverse variance weighted	4	0.005112	0.026623	0.847731712	1.00512515	0.9540207	1.0589671	0.999221
PIK3C2B	Inverse variance weighted	2	0.015919	0.083124	0.848130346	1.01604604	0.8632898	1.195832	0.999221
APEX1	Inverse variance weighted	2	0.045459	0.237504	0.848210843	1.0465077	0.6570149	1.6669004	0.999221
SLC2A12	Wald ratio	1	-0.04166	0.218722	0.848935999	0.95919462	0.6247799	1.4726055	0.999221
SELL	Inverse variance weighted	6	-0.00416	0.021884	0.849328185	0.99585125	0.9540401	1.0394947	0.999221
FFAR4	Inverse variance weighted	3	0.008191	0.043148	0.849428414	1.00822511	0.9264662	1.0971991	0.999221
NPTN	Wald ratio	1	0.034175	0.180156	0.849547708	1.03476547	0.7269256	1.4729699	0.999221
LIPM	Wald ratio	1	0.028202	0.148792	0.849667661	1.02860382	0.7684117	1.3768996	0.999221
TGFBR1	Inverse variance weighted	2	0.021017	0.111048	0.849892024	1.02123895	0.8214881	1.2695607	0.999221
GCLC	Inverse variance weighted	3	-0.01837	0.097086	0.849954409	0.98180121	0.811675	1.1875856	0.999221
INPP5D	Inverse variance weighted	3	0.009016	0.047714	0.850125415	1.00905682	0.9189677	1.1079777	0.999221
SPOCK1	Wald ratio	1	-0.03893	0.206523	0.850465441	0.96181353	0.6416451	1.4417398	0.999221
ATP13A4	Inverse variance weighted	3	-0.00695	0.036895	0.850508542	0.99307056	0.9237927	1.0675437	0.999221
PTPN4	Wald ratio	1	-0.01803	0.095821	0.850783515	0.98213572	0.8139671	1.1850486	0.999221
IL7	Wald ratio	1	0.030872	0.164652	0.851268624	1.03135368	0.7468842	1.4241704	0.999221
TNFSF8	Inverse variance weighted	3	-0.01538	0.082982	0.852984496	0.98474002	0.8369231	1.1586642	0.999221
MTCH1	Inverse variance weighted	3	-0.02086	0.112578	0.853027812	0.97936016	0.7854419	1.221155	0.999221
SLAMF7	Wald ratio	1	0.010887	0.058791	0.853083789	1.01094677	0.9009155	1.1344164	0.999221
ECE1	Inverse variance weighted	3	-0.01011	0.054656	0.853218585	0.98993894	0.8893743	1.1018747	0.999221
IGLC2	Inverse variance weighted	2	-0.00451	0.024489	0.853854118	0.99549929	0.9488463	1.0444461	0.999221
WNT11	Inverse variance weighted	2	0.014736	0.08009	0.854019633	1.01484496	0.8674133	1.1873351	0.999221
GPX2	Inverse variance weighted	2	-0.01609	0.087493	0.854117408	0.9840415	0.8289674	1.1681252	0.999221
IGHV3-23	Wald ratio	1	0.02292	0.124953	0.854459107	1.02318506	0.8009254	1.3071226	0.999221
CACNA1H	Inverse variance weighted	2	0.027362	0.149821	0.855085821	1.02774011	0.7662191	1.3785219	0.999221
ESR1	Inverse variance weighted	2	0.014002	0.076916	0.855550468	1.01410044	0.8721846	1.1791079	0.999221
AOAH	Inverse variance weighted	6	-0.00395	0.021762	0.855938848	0.99605693	0.9544644	1.039462	0.999221
SLC19A1	Wald ratio	1	-0.02496	0.138293	0.856769627	0.97534868	0.7437762	1.2790205	0.999221
CCNE2	Wald ratio	1	-0.02386	0.132546	0.857152568	0.97642412	0.7530318	1.2660875	0.999221
PDE4D	Inverse variance weighted	4	-0.00797	0.044316	0.857200228	0.99205749	0.909524	1.0820804	0.999221
ABHD12	Inverse variance weighted	2	0.010218	0.05691	0.857515851	1.01026991	0.9036377	1.1294851	0.999221
ATR	Wald ratio	1	-0.03447	0.192035	0.857555183	0.96611945	0.6630823	1.4076484	0.999221
NR1I3	Inverse variance weighted	2	-0.02688	0.149837	0.857616444	0.97347604	0.7257412	1.3057763	0.999221
DUSP16	Inverse variance weighted	3	0.009838	0.055136	0.858380497	1.00988685	0.9064416	1.1251375	0.999221
CCL4L2	Wald ratio	1	0.018325	0.103287	0.859179006	1.01849412	0.8318375	1.2470347	0.999221
GALK1	Inverse variance weighted	2	0.008327	0.047191	0.859937488	1.00836186	0.9192768	1.10608	0.999221
GGT7	Inverse variance weighted	2	-0.00846	0.048108	0.860392475	0.99157478	0.9023508	1.0896212	0.999221
IRAK2	Inverse variance weighted	2	-0.02244	0.12859	0.861469138	0.97781031	0.7599696	1.2580937	0.999221
PPIL4	Wald ratio	1	0.019841	0.113871	0.861674946	1.02003922	0.8159966	1.2751034	0.999221
PPBP	Wald ratio	1	-0.04539	0.261017	0.861933802	0.95562072	0.5729329	1.5939229	0.999221
BTNL9	Inverse variance weighted	3	-0.00762	0.043987	0.862459812	0.9924085	0.9104332	1.0817649	0.999221
APOA2	Wald ratio	1	0.0126	0.072799	0.862590964	1.01267948	0.8780202	1.167991	0.999221
EEF2	Inverse variance weighted	2	0.021544	0.124773	0.8629166	1.02177741	0.8001054	1.3048644	0.999221
SLC25A44	Inverse variance weighted	2	-0.04396	0.255114	0.863198707	0.95699503	0.5804334	1.5778546	0.999221
CASP10	Wald ratio	1	-0.01439	0.083773	0.863659885	0.98571777	0.8364575	1.1616126	0.999221
PYGM	Inverse variance weighted	2	-0.02777	0.161875	0.863810133	0.97261618	0.7081918	1.3357713	0.999221
CCNA1	Inverse variance weighted	2	-0.00465	0.027157	0.863936638	0.995357	0.9437613	1.0497735	0.999221
CYB5D2	Inverse variance weighted	3	-0.00601	0.035144	0.86417374	0.99400613	0.9278412	1.0648893	0.999221
SEMA3A	Inverse variance weighted	3	0.009803	0.057307	0.86417457	1.00985131	0.9025611	1.1298954	0.999221
PIM1	Inverse variance weighted	3	0.010986	0.064306	0.864349569	1.01104666	0.8913178	1.1468585	0.999221
NRBP2	Inverse variance weighted	3	0.007694	0.045081	0.864475507	1.00772408	0.9225035	1.1008173	0.999221
S1PR2	Wald ratio	1	-0.0078	0.045766	0.864718872	0.99223312	0.9071033	1.0853522	0.999221
GSTA4	Inverse variance weighted	2	0.009123	0.053861	0.865492726	1.00916505	0.9080606	1.1215266	0.999221

ADCY7	Inverse variance weighted	3	-0.00638	0.037762	0.865867454	0.99364184	0.9227537	1.0699758	0.999221
PRPF4B	Wald ratio	1	-0.03863	0.229492	0.866334033	0.96210913	0.6135878	1.5085927	0.999221
SERPINI2	Wald ratio	1	-0.02804	0.166817	0.866530392	0.97235297	0.7011756	1.3484073	0.999221
P2RX1	Inverse variance weighted	5	-0.00334	0.019958	0.867132434	0.99666655	0.9584319	1.0364265	0.999221
VCAN	Wald ratio	1	0.049453	0.296718	0.867632335	1.0506962	0.5873618	1.8795271	0.999221
CCND1	Inverse variance weighted	2	-0.01113	0.066904	0.867917972	0.98893533	0.8673977	1.1275025	0.999221
IL17RA	Inverse variance weighted	3	0.011165	0.067316	0.868262975	1.01122793	0.8862345	1.1538503	0.999221
KLHL30	Inverse variance weighted	2	0.007219	0.043643	0.868623216	1.00724495	0.9246677	1.0971967	0.999221
PTK7	Inverse variance weighted	3	-0.01063	0.064355	0.868760832	0.98942284	0.872172	1.1224363	0.999221
BTN2A2	Inverse variance weighted	4	0.006165	0.037395	0.869046359	1.0061844	0.9350742	1.0827024	0.999221
SLC22A5	Inverse variance weighted	5	-0.00448	0.027209	0.869195741	0.99552923	0.9438288	1.0500617	0.999221
HIST1H1E	Wald ratio	1	0.024271	0.14757	0.869358262	1.02456833	0.7672321	1.3682173	0.999221
CPA3	Inverse variance weighted	2	0.02215	0.134739	0.869423718	1.02239702	0.7851037	1.3314109	0.999221
LYPD5	Inverse variance weighted	2	-0.01322	0.080532	0.869588794	0.98686528	0.8427679	1.1556006	0.999221
SIGLEC12	Inverse variance weighted	4	-0.00549	0.033571	0.870045993	0.99452281	0.9311902	1.0621629	0.999221
ADM	Inverse variance weighted	3	0.00599	0.036647	0.870168308	1.00600773	0.9362822	1.0809258	0.999221
CNGA4	Inverse variance weighted	2	0.013413	0.082072	0.870179479	1.01350342	0.8629073	1.1903818	0.999221
CD48	Inverse variance weighted	5	0.005475	0.033607	0.870587729	1.00548992	0.9413937	1.0739503	0.999221
RPN2	Wald ratio	1	0.015401	0.094609	0.870683233	1.0155207	0.8436375	1.2224235	0.999221
GMNN	Wald ratio	1	-0.01682	0.103517	0.870912128	0.98331911	0.8027469	1.2045097	0.999221
ABL2	Inverse variance weighted	3	-0.01459	0.089826	0.870960611	0.98551474	0.8264217	1.1752345	0.999221
DRAXIN	Inverse variance weighted	2	-0.00577	0.035556	0.871114813	0.99424783	0.9273173	1.0660092	0.999221
IL17RE	Wald ratio	1	-0.02074	0.12786	0.871130861	0.97947186	0.7623513	1.2584292	0.999221
PTPRA	Inverse variance weighted	3	0.010355	0.063915	0.871296888	1.01040871	0.8914393	1.1452555	0.999221
KRT8	Wald ratio	1	-0.03064	0.189229	0.871380853	0.96982742	0.6692974	1.4053023	0.999221
FKBP1A	Inverse variance weighted	5	0.00569	0.035231	0.871699198	1.00570597	0.9386035	1.0776057	0.999221
P4HTM	Inverse variance weighted	2	-0.00618	0.038458	0.87228342	0.99383654	0.9216764	1.0716463	0.999221
PRTN3	Inverse variance weighted	2	0.008701	0.054234	0.872544164	1.0087386	0.9070134	1.1218727	0.999221
RPS6KC1	Inverse variance weighted	2	0.015554	0.097009	0.872614558	1.01567586	0.8398068	1.2283747	0.999221
ACTN1	Inverse variance weighted	2	-0.01635	0.102117	0.872818656	0.98378613	0.8053362	1.2017777	0.999221
IKBKB	Inverse variance weighted	2	-0.01919	0.119936	0.872863341	0.98099054	0.7754846	1.2409562	0.999221
GPR132	Wald ratio	1	-0.02763	0.172683	0.872881074	0.97274894	0.693442	1.364556	0.999221
IGF1R	Inverse variance weighted	4	-0.00767	0.048002	0.873032955	0.99235832	0.9032512	1.0902559	0.999221
MBOAT4	Wald ratio	1	0.010712	0.067155	0.873267709	1.0107695	0.8861112	1.1529648	0.999221
PSEN2	Inverse variance weighted	2	0.010199	0.063988	0.873356189	1.01025165	0.891173	1.1452415	0.999221
RHAG	Wald ratio	1	0.046489	0.293808	0.874277261	1.04758609	0.5889733	1.8633047	0.999221
GBA2	Inverse variance weighted	6	0.005183	0.032798	0.874445825	1.00519601	0.9426108	1.0719366	0.999221
ATG4C	Inverse variance weighted	2	0.006941	0.043993	0.874628189	1.00696551	0.9237763	1.0976462	0.999221
MGAT4A	Inverse variance weighted	2	-0.01319	0.083588	0.874652346	0.98690039	0.8377641	1.1625855	0.999221
MFSD10	Inverse variance weighted	4	-0.00922	0.058539	0.874797741	0.99081856	0.8834148	1.1112803	0.999221
DHFR	Inverse variance weighted	3	-0.00495	0.0315	0.875243811	0.99506669	0.9354896	1.058438	0.999221
STK38	Inverse variance weighted	3	0.011095	0.070845	0.875557559	1.01115636	0.8800628	1.1617775	0.999221
GPR97	Inverse variance weighted	2	0.006031	0.038588	0.875805273	1.00604916	0.9327647	1.0850913	0.999221
EXOG	Inverse variance weighted	2	0.018805	0.120375	0.875862205	1.01898253	0.8048248	1.290126	0.999221
CD3E	Wald ratio	1	-0.01073	0.069059	0.876553436	0.98932975	0.8640861	1.1327267	0.999221
HSPA5	Inverse variance weighted	3	-0.02089	0.134708	0.876738343	0.97932286	0.7520734	1.2752389	0.999221
AKT2	Inverse variance weighted	2	-0.02497	0.161444	0.877095823	0.97534157	0.7107767	1.3383826	0.999221
PRSS23	Inverse variance weighted	3	-0.02032	0.131752	0.87741979	0.97988352	0.7568761	1.2685983	0.999221
OLR1	Wald ratio	1	-0.02772	0.180157	0.877731042	0.97266418	0.6832986	1.3845712	0.999221
SIGLEC11	Inverse variance weighted	4	-0.00385	0.025068	0.877833666	0.99615404	0.9483924	1.046321	0.999221
MAPK9	Inverse variance weighted	2	-0.00576	0.03752	0.877945096	0.99425439	0.9237608	1.0701275	0.999221
CD1A	Inverse variance weighted	3	-0.00595	0.038817	0.878073126	0.99406272	0.9212389	1.0726432	0.999221
STK10	Inverse variance weighted	3	0.007246	0.047257	0.878142357	1.00727195	0.9181653	1.1050262	0.999221
UPK3A	Inverse variance weighted	3	-0.00369	0.024131	0.878370447	0.9963139	0.9502882	1.0445687	0.999221
CLU	Wald ratio	1	-0.02321	0.152123	0.878759173	0.97706197	0.7251577	1.3164725	0.999221
BACE2	Inverse variance weighted	3	-0.0072	0.047432	0.879340911	0.99282547	0.9046864	1.0895515	0.999221
NEK9	Wald ratio	1	-0.02899	0.191478	0.879664589	0.97142741	0.6674532	1.4138388	0.999221
WFIKKN1	Inverse variance weighted	2	-0.01104	0.073491	0.880614867	0.98902308	0.8563466	1.1422555	0.999221
GABRR2	Inverse variance weighted	2	-0.01112	0.074505	0.881402323	0.988946	0.8545797	1.1444389	0.999221
PLGLB1	Inverse variance weighted	4	-0.0032	0.021582	0.882307911	0.99681003	0.9555239	1.0398801	0.999221
SECTM1	Inverse variance weighted	2	0.021143	0.143223	0.882641529	1.02136788	0.7713799	1.3523718	0.999221
MAST2	Wald ratio	1	-0.01094	0.074522	0.883298575	0.98912065	0.8547028	1.1446781	0.999221
RABGGTA	Wald ratio	1	0.013527	0.092658	0.883932913	1.01361868	0.845283	1.2154779	0.999221

TRIB1	Inverse variance weighted	2	0.019527	0.133764	0.883933575	1.01971936	0.7845464	1.325387	0.999221
CAPN11	Inverse variance weighted	4	0.005186	0.035526	0.883946249	1.0051992	0.9375868	1.0776873	0.999221
MFSD9	Inverse variance weighted	6	-0.00309	0.021169	0.883986464	0.99691591	0.9563999	1.0391483	0.999221
RGS12	Inverse variance weighted	6	-0.00714	0.04909	0.884305967	0.99288217	0.9018019	1.0931614	0.999221
RB1	Wald ratio	1	-0.00987	0.067868	0.884352001	0.9901769	0.8668474	1.131053	0.999221
GREM2	Inverse variance weighted	3	-0.01024	0.070426	0.884381563	0.98981109	0.8621927	1.1363191	0.999221
FCGR2B	Inverse variance weighted	7	-0.00361	0.024903	0.884875003	0.99640075	0.9489345	1.0462413	0.999221
CALM1	Inverse variance weighted	2	-0.01377	0.095132	0.884915367	0.98632492	0.8185446	1.1884958	0.999221
CASP5	Inverse variance weighted	4	0.005193	0.03599	0.885260681	1.00520701	0.9367423	1.0786756	0.999221
GLP1R	Inverse variance weighted	2	-0.01583	0.109687	0.885272505	0.98429799	0.7938878	1.2203771	0.999221
NTN4	Wald ratio	1	-0.04633	0.32268	0.885834641	0.95472751	0.5072348	1.7970074	0.999221
CTSA	Inverse variance weighted	3	-0.01129	0.078759	0.885997277	0.98877175	0.8473345	1.1538177	0.999221
JAM3	Inverse variance weighted	2	-0.01125	0.078775	0.886416816	0.98881094	0.8473426	1.1538982	0.999221
LGR6	Inverse variance weighted	6	0.004982	0.035038	0.886924029	1.0049947	0.9382943	1.0764366	0.999221
TLR2	Inverse variance weighted	2	-0.01051	0.073969	0.887065159	0.98954998	0.8560004	1.1439355	0.999221
PREP	Inverse variance weighted	3	-0.01368	0.096441	0.887216068	0.98641516	0.8165218	1.1916581	0.999221
TIMP2	Inverse variance weighted	3	-0.00509	0.036023	0.887644085	0.99492338	0.9270991	1.0677095	0.999221
TM2D1	Inverse variance weighted	4	-0.00693	0.049024	0.887653177	0.99309809	0.9021153	1.0932569	0.999221
VSIG2	Wald ratio	1	0.01571	0.111935	0.888382768	1.0158342	0.8157217	1.2650381	0.999221
HDAC10	Inverse variance weighted	2	0.007954	0.056735	0.88851268	1.00798521	0.9019038	1.1265439	0.999221
PINK1	Inverse variance weighted	2	0.006104	0.043543	0.88851492	1.00612267	0.9238181	1.0957599	0.999221
EPHB1	Inverse variance weighted	5	-0.00375	0.026842	0.888914586	0.99625774	0.9451985	1.0500752	0.999221
TLR1	Inverse variance weighted	2	-0.00579	0.041477	0.888962708	0.99422581	0.916598	1.0784281	0.999221
SYNJ2	Inverse variance weighted	7	0.003998	0.02866	0.889055994	1.00400605	0.9491621	1.0620189	0.999221
AURKAIP1	Wald ratio	1	-0.02823	0.203491	0.889664835	0.97216474	0.652416	1.4486223	0.999221
ABHD16A	Inverse variance weighted	2	0.014878	0.108146	0.890579848	1.01498888	0.8211185	1.2546331	0.999221
SFRP1	Inverse variance weighted	2	-0.01816	0.132224	0.890763888	0.98200448	0.7578124	1.2725218	0.999221
SLC16A7	Inverse variance weighted	2	0.009283	0.067602	0.890775389	1.00932653	0.8840723	1.1523266	0.999221
SFTPD	Inverse variance weighted	3	-0.00433	0.031677	0.891158554	0.99567479	0.9357369	1.059452	0.999221
STK17B	Inverse variance weighted	3	0.006464	0.047313	0.891329221	1.00648496	0.917347	1.1042844	0.999221
GPX7	Inverse variance weighted	5	0.002765	0.02026	0.891455641	1.00276862	0.9637284	1.0433903	0.999221
SV2B	Wald ratio	1	0.031411	0.230347	0.891533819	1.03190953	0.657001	1.6207545	0.999221
CD300LB	Inverse variance weighted	6	-0.00456	0.033455	0.891606143	0.99545144	0.9322726	1.0629118	0.999221
GPR88	Wald ratio	1	-0.03935	0.289468	0.891882682	0.96141878	0.5451454	1.6955589	0.999221
HRAS	Inverse variance weighted	3	0.005489	0.040723	0.89277351	1.00550441	0.9283669	1.0890512	0.999221
ADAMTS2	Wald ratio	1	0.008632	0.064122	0.892915977	1.00866912	0.8895437	1.1437475	0.999221
ITGA2B	Wald ratio	1	0.038072	0.283158	0.893043756	1.03880566	0.5963558	1.8095191	0.999221
S100A8	Inverse variance weighted	2	-0.0052	0.038726	0.893282727	0.99481826	0.9221024	1.0732684	0.999221
GLUL	Inverse variance weighted	3	-0.01068	0.079745	0.893437535	0.98937454	0.8462143	1.1567543	0.999221
CACNB2	Inverse variance weighted	2	0.009837	0.073668	0.893769359	1.00988595	0.8741068	1.1667564	0.999221
CA4	Inverse variance weighted	2	-0.01701	0.127881	0.894193728	0.98313565	0.7651712	1.2631889	0.999221
IGKV1-5	Inverse variance weighted	2	-0.0183	0.137927	0.894450471	0.98186691	0.749284	1.2866452	0.999221
TP53	Inverse variance weighted	2	-0.03359	0.25495	0.895191196	0.96697108	0.5866726	1.5937903	0.999221
PDGFC	Inverse variance weighted	3	0.011994	0.091513	0.895728211	1.01206586	0.845885	1.2108943	0.999221
GRK5	Inverse variance weighted	3	-0.02228	0.171437	0.896607225	0.9779684	0.6988678	1.3685309	0.999221
TM4SF1	Inverse variance weighted	2	0.014475	0.111571	0.896769975	1.01458075	0.8152969	1.2625758	0.999221
PTPN1	Inverse variance weighted	2	0.005399	0.041811	0.897257625	1.00541355	0.9263054	1.0912776	0.999221
PKD2L1	Inverse variance weighted	3	0.007907	0.061362	0.897473978	1.007938	0.8937202	1.1367529	0.999221
RTN4R	Inverse variance weighted	2	-0.01883	0.146446	0.897667923	0.98134219	0.7364843	1.3076077	0.999221
EPB42	Wald ratio	1	0.033312	0.259485	0.897849541	1.03387329	0.6217123	1.7192742	0.999221
KCNA3	Inverse variance weighted	2	-0.01608	0.125488	0.898030944	0.98404749	0.7694828	1.258442	0.999221
OCLN	Inverse variance weighted	2	-0.01002	0.078612	0.898601319	0.99003263	0.8486602	1.1549554	0.999221
VEGFB	Wald ratio	1	-0.01262	0.099163	0.898724545	0.98745861	0.8130364	1.1993	0.999221
HKDC1	Inverse variance weighted	6	-0.00439	0.034573	0.899068112	0.99562446	0.9303937	1.0654286	0.999221
LRRC4	Wald ratio	1	-0.00993	0.078631	0.899481958	0.99011683	0.8487004	1.1550971	0.999221
CDK12	Inverse variance weighted	3	-0.00464	0.036779	0.899540523	0.99536771	0.9261398	1.0697703	0.999221
APAF1	Inverse variance weighted	3	-0.00491	0.039167	0.900212524	0.99510081	0.9215687	1.0745001	0.999221
NRG4	Wald ratio	1	-0.01854	0.147893	0.900228156	0.98162908	0.7346131	1.3117049	0.999221
VMO1	Inverse variance weighted	4	-0.00572	0.045811	0.900571217	0.99429273	0.9089064	1.0877006	0.999221
VAMP1	Inverse variance weighted	4	0.003432	0.027474	0.900595175	1.00343772	0.9508318	1.0589542	0.999221
BCR	Inverse variance weighted	4	-0.00531	0.042576	0.900786028	0.99470613	0.9150672	1.081276	0.999221
FPR3	Inverse variance weighted	4	0.00577	0.046472	0.90119323	1.00578631	0.9182234	1.1016993	0.999221
CCR5	Inverse variance weighted	2	0.005764	0.046466	0.901279207	1.00578057	0.9182278	1.1016815	0.999221

PDE6G	Inverse variance weighted	3	0.020491	0.166386	0.901986903	1.02070208	0.7366626	1.4142605	0.999221
TXNDC11	Wald ratio	1	-0.00646	0.05278	0.90254345	0.99355794	0.8959127	1.1018455	0.999221
UCHL3	Wald ratio	1	-0.01457	0.119258	0.902790199	0.98553969	0.7801166	1.2450555	0.999221
WISP3	Wald ratio	1	-0.03285	0.269755	0.903061475	0.9676791	0.5703105	1.6419175	0.999221
SRGN	Inverse variance weighted	3	-0.00637	0.05229	0.903096882	0.99365391	0.8968608	1.1008934	0.999221
CD300LF	Inverse variance weighted	5	-0.00586	0.04823	0.903233103	0.99415345	0.9044807	1.0927166	0.999221
KLRD1	Inverse variance weighted	5	0.004913	0.040649	0.903805911	1.00492472	0.9279668	1.0882649	0.999221
SRD5A3	Inverse variance weighted	10	-0.0046	0.038211	0.904138383	0.99540864	0.9235824	1.0728207	0.999221
GPR27	Inverse variance weighted	3	-0.00597	0.049634	0.904190886	0.99404338	0.9018945	1.0956074	0.999221
COL4A1	Inverse variance weighted	2	-0.0064	0.053534	0.904827839	0.99361968	0.8946461	1.1035426	0.999221
SERPING1	Inverse variance weighted	3	-0.00431	0.036025	0.904843505	0.99570265	0.9278219	1.0685497	0.999221
THBS2	Inverse variance weighted	2	-0.0076	0.063672	0.905046076	0.9924334	0.8759974	1.1243459	0.999221
DUSP1	Wald ratio	1	-0.01577	0.132669	0.905369541	0.98435191	0.7589628	1.2766749	0.999221
KCNK17	Inverse variance weighted	5	0.004162	0.035014	0.905371047	1.00417117	0.9375681	1.0755056	0.999221
MST1L	Inverse variance weighted	4	-0.00186	0.015694	0.905636569	0.99814134	0.9679064	1.0293207	0.999221
MAN1B1	Inverse variance weighted	3	0.004413	0.037238	0.905656034	1.00442323	0.9337246	1.0804749	0.999221
CPT1A	Inverse variance weighted	3	-0.00977	0.082475	0.905723075	0.99027967	0.8424691	1.1640235	0.999221
ABCG1	Inverse variance weighted	4	-0.00929	0.07875	0.906047483	0.99074857	0.849044	1.1561035	0.999221
PPIL2	Inverse variance weighted	2	0.007087	0.0604	0.906588321	1.00711263	0.8946742	1.1336817	0.999221
FGFR2	Inverse variance weighted	3	0.004418	0.037902	0.907202915	1.00442791	0.9325152	1.0818863	0.999221
PTPDC1	Wald ratio	1	-0.02108	0.180976	0.907252439	0.97913618	0.6867419	1.3960233	0.999221
NMT1	Inverse variance weighted	2	-0.0115	0.098742	0.90730294	0.98856826	0.8146219	1.1996573	0.999221
TMX4	Inverse variance weighted	4	0.00281	0.024232	0.907676438	1.00281408	0.9562999	1.0515908	0.999221
TNFRSF12A	Wald ratio	1	-0.02654	0.2293	0.907839942	0.97380466	0.6212809	1.5263555	0.999221
NFKBIA	Inverse variance weighted	3	-0.00591	0.051213	0.908080936	0.99410448	0.8991643	1.0990691	0.999221
PTPRE	Inverse variance weighted	4	-0.00523	0.045354	0.908154917	0.9947814	0.9101687	1.08726	0.999221
PSMB10	Wald ratio	1	0.010875	0.094651	0.908529444	1.01093416	0.8397581	1.2170027	0.999221
PSMB7	Inverse variance weighted	2	-0.02537	0.220868	0.908544894	0.97494715	0.6323746	1.5030994	0.999221
NR2F6	Inverse variance weighted	5	-0.00234	0.020387	0.908617608	0.99766272	0.9585845	1.038334	0.999221
PLCH1	Wald ratio	1	-0.00932	0.081521	0.909011307	0.99072656	0.844426	1.1623743	0.999221
FKBP5	Wald ratio	1	0.026361	0.232177	0.909604737	1.02671127	0.6513507	1.6183846	0.999221
EIF2AK2	Inverse variance weighted	2	0.006667	0.058826	0.909760357	1.0066897	0.8970604	1.1297168	0.999221
CYP51A1	Wald ratio	1	-0.00257	0.022731	0.909866012	0.99742995	0.9539665	1.0428736	0.999221
BCL2L1	Wald ratio	1	-0.01895	0.167848	0.910107279	0.98122788	0.7061468	1.3634674	0.999221
CXCL16	Inverse variance weighted	4	-0.00366	0.032468	0.910269979	0.99634756	0.9349173	1.0618142	0.999221
CDK2	Wald ratio	1	0.021078	0.187066	0.910287385	1.02130152	0.7078162	1.4736265	0.999221
SV2A	Wald ratio	1	0.00757	0.067667	0.910930919	1.0075983	0.8824451	1.1505014	0.999221
IL18BP	Inverse variance weighted	3	0.010192	0.091288	0.911105971	1.0102438	0.8447351	1.2081806	0.999221
HTRA4	Wald ratio	1	-0.02321	0.208908	0.911528238	0.97705536	0.6487738	1.4714483	0.999221
CD44	Inverse variance weighted	3	-0.00739	0.066921	0.912107852	0.99264043	0.8706175	1.1317657	0.999221
PMS2P3	Wald ratio	1	0.014269	0.129848	0.912496526	1.01437132	0.7864443	1.308356	0.999221
ARSB	Inverse variance weighted	5	0.003262	0.029725	0.912608854	1.00326764	0.9464858	1.063456	0.999221
CSF2RB	Inverse variance weighted	2	0.008189	0.074617	0.912615104	1.00822216	0.8710454	1.1670023	0.999221
CYP2S1	Inverse variance weighted	4	0.003931	0.035843	0.912668516	1.00393874	0.9358305	1.0770038	0.999221
BCL2	Inverse variance weighted	3	0.015631	0.142619	0.912724846	1.01575415	0.7680491	1.3433471	0.999221
GLIPR1L1	Wald ratio	1	0.013667	0.125278	0.913130385	1.01376048	0.7930431	1.2959073	0.999221
WNT16	Wald ratio	1	0.017786	0.165483	0.91441086	1.01794471	0.735973	1.4079477	0.999221
FOLR3	Inverse variance weighted	9	0.001566	0.014574	0.914419125	1.00156747	0.9733618	1.0305904	0.999221
DUSP10	Inverse variance weighted	4	0.013182	0.122842	0.914542083	1.01326964	0.7964517	1.2891118	0.999221
OR7D2	Inverse variance weighted	6	-0.00226	0.021131	0.914773599	0.99774111	0.9572616	1.0399324	0.999221
RRM1	Inverse variance weighted	3	-0.00391	0.036708	0.915109853	0.99609474	0.9269454	1.0704025	0.999221
GRIK4	Inverse variance weighted	5	-0.00359	0.033727	0.915146911	0.9964129	0.9326755	1.064506	0.999221
NTN1	Wald ratio	1	0.010815	0.101839	0.915427884	1.01087343	0.8279609	1.2341948	0.999221
NPC1	Inverse variance weighted	3	-0.0073	0.068893	0.915587217	0.99272432	0.8673321	1.1362448	0.999221
RASGRP3	Inverse variance weighted	3	0.003611	0.034253	0.916039609	1.00361762	0.9384507	1.0733098	0.999221
TRPM7	Inverse variance weighted	2	0.008483	0.080519	0.916090559	1.00851955	0.8612812	1.1809287	0.999221
IPP	Inverse variance weighted	2	-0.00358	0.03403	0.916126789	0.99642261	0.9321308	1.0651488	0.999221
VAMP2	Inverse variance weighted	2	0.009728	0.093189	0.916861922	1.0097752	0.841203	1.2121283	0.999221
ACVR2B	Inverse variance weighted	2	0.002794	0.026856	0.917139714	1.00279788	0.9513789	1.0569959	0.999221
SSTR3	Inverse variance weighted	2	0.00618	0.060935	0.919217178	1.00619911	0.8929259	1.1338418	0.999221
MERTK	Inverse variance weighted	7	-0.00225	0.022442	0.920161661	0.99775318	0.9548172	1.0426199	0.999221
VSTM1	Inverse variance weighted	4	0.001949	0.019451	0.920205391	1.00195041	0.964471	1.0408863	0.999221
FCRLA	Inverse variance weighted	3	0.007066	0.070535	0.920206085	1.00709078	0.8770578	1.1564024	0.999221

TUBB6	Inverse variance weighted	7	-0.00279	0.027859	0.920329767	0.99721751	0.9442266	1.0531824	0.999221
SLC16A4	Inverse variance weighted	3	0.009379	0.094192	0.92068056	1.00942342	0.8392583	1.2140906	0.999221
SLC17A9	Inverse variance weighted	4	0.007246	0.073503	0.921465815	1.00727277	0.8721281	1.1633593	0.999221
CHMP1A	Inverse variance weighted	3	0.006728	0.068619	0.921898549	1.00675029	0.880059	1.1516797	0.999221
COL15A1	Wald ratio	1	0.012073	0.124995	0.923051276	1.01214659	0.7922191	1.2931281	0.999221
IL4I1	Wald ratio	1	0.018179	0.1884	0.92313044	1.01834516	0.7039243	1.473208	0.999221
EHMT1	Inverse variance weighted	2	0.004539	0.047079	0.923193039	1.00454929	0.9160032	1.1016548	0.999221
PIP4K2C	Wald ratio	1	-0.00821	0.085335	0.923398386	0.9918283	0.8390696	1.1723978	0.999221
CES4A	Inverse variance weighted	2	0.009817	0.103298	0.92428973	1.00986495	0.8247721	1.2364957	0.999221
SLC27A1	Inverse variance weighted	3	0.005785	0.060984	0.924425078	1.00580182	0.892486	1.133505	0.999221
BTN2A1	Inverse variance weighted	4	-0.00305	0.032356	0.924939596	0.99695623	0.9356937	1.0622298	0.999221
RNASE3	Inverse variance weighted	5	0.002194	0.023348	0.92513852	1.00219624	0.9573676	1.0491239	0.999221
ATP1B1	Inverse variance weighted	4	0.00406	0.04347	0.92558261	1.00406854	0.9220635	1.0933668	0.999221
FURIN	Inverse variance weighted	2	-0.01083	0.11599	0.925628783	0.98923125	0.7880711	1.2417387	0.999221
MCL1	Inverse variance weighted	2	-0.00673	0.072303	0.925831121	0.99329179	0.862047	1.1445184	0.999221
PLBD1	Inverse variance weighted	3	-0.00382	0.041298	0.926206637	0.99618237	0.918725	1.0801702	0.999221
NFKB1	Inverse variance weighted	3	0.004762	0.051419	0.92621876	1.00477288	0.9084468	1.1113127	0.999221
MAST1	Wald ratio	1	-0.02319	0.250667	0.926285152	0.9770752	0.5977997	1.596983	0.999221
CNTNAP3	Wald ratio	1	0.005178	0.05599	0.926312272	1.0051917	0.9007187	1.1217823	0.999221
KCNJ14	Wald ratio	1	0.022868	0.248953	0.926810832	1.02313183	0.628085	1.6666514	0.999221
NLGN2	Inverse variance weighted	2	0.017052	0.18705	0.927362041	1.0171985	0.7049942	1.4676614	0.999221
BRSK2	Wald ratio	1	-0.01595	0.175425	0.927564827	0.98417873	0.6978287	1.3880308	0.999221
TXK	Inverse variance weighted	3	-0.00417	0.046003	0.927727561	0.99583598	0.9099737	1.0897999	0.999221
PLCH2	Inverse variance weighted	4	0.003086	0.034121	0.927935153	1.00309081	0.9382003	1.0724694	0.999221
SLC22A31	Inverse variance weighted	3	0.001571	0.017388	0.928029049	1.0015718	0.9680129	1.0362941	0.999221
CRTAP	Wald ratio	1	0.007752	0.085923	0.92810797	1.00778258	0.8515843	1.1926308	0.999221
GPR157	Wald ratio	1	0.023939	0.265718	0.928215624	1.02422736	0.6084331	1.7241693	0.999221
PRL	Wald ratio	1	0.025376	0.281671	0.928215624	1.02570047	0.5905509	1.7814916	0.999221
KALRN	Inverse variance weighted	2	-0.00549	0.061524	0.928835168	0.99452035	0.8815432	1.1219765	0.999221
TNFRSF11A	Wald ratio	1	-0.01119	0.125382	0.928854847	0.98886756	0.7734111	1.2643458	0.999221
OLFML2B	Inverse variance weighted	3	-0.00862	0.096641	0.928907992	0.99141486	0.8203381	1.1981687	0.999221
CD180	Wald ratio	1	-0.00557	0.062743	0.929312641	0.99444959	0.8793772	1.12458	0.999221
S1PR3	Inverse variance weighted	4	0.003337	0.037971	0.929979611	1.00334214	0.9313805	1.0808638	0.999221
CDK7	Wald ratio	1	0.006074	0.069243	0.930099781	1.00609247	0.8784088	1.1523359	0.999221
IGFBP2	Inverse variance weighted	3	-0.00526	0.059999	0.930163104	0.99475549	0.8843903	1.1188934	0.999221
MAP2K1	Inverse variance weighted	2	-0.00472	0.053918	0.930299948	0.99529506	0.8954806	1.1062353	0.999221
IGLV7-46	Inverse variance weighted	3	-0.00387	0.044439	0.930636884	0.99613937	0.9130469	1.0867937	0.999221
ABCB1	Inverse variance weighted	2	-0.01252	0.14411	0.930779114	0.98755994	0.7445507	1.3098834	0.999221
CDK11A	Inverse variance weighted	2	-0.00589	0.067839	0.930809059	0.99412706	0.8703546	1.1355011	0.999221
SLC37A3	Inverse variance weighted	3	0.002899	0.033444	0.930931296	1.00290292	0.9392703	1.0708464	0.999221
RORA	Inverse variance weighted	3	0.007371	0.085073	0.930956277	1.00739808	0.8526794	1.1901905	0.999221
DNAJC10	Wald ratio	1	-0.00477	0.055152	0.931038383	0.99523858	0.8932657	1.1088524	0.999221
IL10RB	Inverse variance weighted	6	0.003961	0.045951	0.931302633	1.00396907	0.9175007	1.0985865	0.999221
KCNH3	Inverse variance weighted	3	-0.00525	0.061602	0.932057334	0.99476179	0.8816221	1.1224208	0.999221
H6PD	Inverse variance weighted	5	0.002569	0.030259	0.932332827	1.00257264	0.9448405	1.0638324	0.999221
FKTN	Inverse variance weighted	2	0.012296	0.145422	0.932613668	1.01237235	0.7612976	1.3462511	0.999221
THOP1	Wald ratio	1	0.004545	0.054087	0.933029707	1.00455545	0.9035129	1.1168979	0.999221
BRCA1	Wald ratio	1	-0.00338	0.040973	0.934194405	0.99662261	0.9197159	1.0799603	0.999221
NAAA	Inverse variance weighted	6	0.003185	0.038591	0.934221289	1.00319021	0.9301088	1.0820139	0.999221
MMP23B	Inverse variance weighted	2	-0.01188	0.144384	0.934444189	0.98819397	0.7446289	1.3114282	0.999221
BAMBI	Inverse variance weighted	4	-0.00346	0.042183	0.934595203	0.99654425	0.9174654	1.0824391	0.999221
GSTM5	Inverse variance weighted	3	-0.00555	0.068355	0.935284424	0.99446511	0.8697709	1.137036	0.999221
FER	Inverse variance weighted	2	-0.02285	0.281687	0.935342027	0.97740712	0.5627287	1.6976649	0.999221
ADAMTS6	Inverse variance weighted	2	-0.00406	0.050252	0.935551493	0.99594474	0.9025259	1.0990331	0.999221
CECR1	Inverse variance weighted	2	0.002829	0.035246	0.936028265	1.00283291	0.9358947	1.0745588	0.999221
ADAMTSL4	Inverse variance weighted	3	0.002362	0.029482	0.936135049	1.00236515	0.9460853	1.0619929	0.999221
GFOD2	Wald ratio	1	0.008784	0.11014	0.936432432	1.00882284	0.8129467	1.2518945	0.999221
AQP1	Wald ratio	1	-0.00976	0.122474	0.936467791	0.9902851	0.7789481	1.2589601	0.999221
ADORA1	Inverse variance weighted	4	-0.00274	0.034432	0.936653833	0.99726724	0.9321864	1.0668917	0.999221
SERPINB9	Inverse variance weighted	5	-0.00223	0.02816	0.936742842	0.99776762	0.94419	1.0543854	0.999221
CCNT1	Inverse variance weighted	2	0.008883	0.112641	0.937142718	1.00892259	0.8090515	1.2581706	0.999221
SULF2	Inverse variance weighted	6	0.001475	0.018704	0.9371506	1.00147596	0.9654262	1.0388719	0.999221
DGAT1	Inverse variance weighted	3	0.004178	0.053196	0.937401929	1.00418649	0.90476	1.1145392	0.999221

ABCF3	Inverse variance weighted	3	0.005897	0.075477	0.937729749	1.00591395	0.867588	1.1662943	0.999221
ICMT	Inverse variance weighted	3	-0.00334	0.042843	0.937923009	0.99666895	0.9163948	1.083975	0.999221
PPIF	Inverse variance weighted	3	-0.00541	0.069922	0.938276529	0.99460013	0.8672208	1.1406893	0.999221
GPD2	Inverse variance weighted	3	0.002392	0.031094	0.938675932	1.00239505	0.9431293	1.065385	0.999221
PTAFR	Wald ratio	1	-0.01555	0.20306	0.938960393	0.98457062	0.6613001	1.465869	0.999221
CXCL6	Inverse variance weighted	2	-0.00774	0.101196	0.939025843	0.99228898	0.813764	1.209979	0.999221
SIGLEC10	Inverse variance weighted	2	0.008885	0.116969	0.9394522	1.00892437	0.8022183	1.2688921	0.999221
OXER1	Inverse variance weighted	3	-0.00562	0.074546	0.939939143	0.99439899	0.8592231	1.1508412	0.999221
DTX2P1	Inverse variance weighted	2	0.007979	0.107	0.940559678	1.00801052	0.8173064	1.2432122	0.999221
COQ6	Inverse variance weighted	3	0.002743	0.037437	0.941590156	1.00274684	0.9318031	1.0790919	0.999221
SPHK1	Inverse variance weighted	7	-0.00296	0.040539	0.941788664	0.99704411	0.9208873	1.0794991	0.999221
CHUK	Inverse variance weighted	2	-0.01033	0.141577	0.941859352	0.98972748	0.7498993	1.3062561	0.999221
ITIH2	Inverse variance weighted	2	0.003581	0.04929	0.942081102	1.00358759	0.9111678	1.1053815	0.999221
PDE1B	Inverse variance weighted	6	0.002767	0.038295	0.942389039	1.00277131	0.9302606	1.080934	0.999221
IL10	Wald ratio	1	0.004526	0.062863	0.942601908	1.00453638	0.8880874	1.1362545	0.999221
SLC15A3	Inverse variance weighted	3	-0.00995	0.138365	0.942655401	0.9900964	0.7549166	1.2985419	0.999221
TCN1	Inverse variance weighted	3	-0.00314	0.043669	0.942674321	0.99686472	0.915091	1.0859458	0.999221
OAT	Inverse variance weighted	4	-0.0029	0.040835	0.943331684	0.99710154	0.9204072	1.0801865	0.999221
MST1	Inverse variance weighted	4	0.00286	0.040254	0.943358093	1.00286412	0.9267815	1.0851926	0.999221
TRIM22	Inverse variance weighted	2	-0.00426	0.060089	0.943432577	0.99574539	0.8851148	1.1202037	0.999221
HIST1H3H	Wald ratio	1	0.017833	0.252638	0.943725474	1.01799321	0.6204337	1.6702997	0.999221
ABCF2	Inverse variance weighted	2	-0.00419	0.05947	0.943825967	0.99581838	0.8862539	1.118928	0.999221
MMP25	Inverse variance weighted	4	-0.0028	0.040018	0.944240635	0.99720503	0.9219782	1.0785698	0.999221
LCK	Wald ratio	1	0.019445	0.280875	0.944805932	1.01963549	0.5879755	1.7681971	0.999221
APOM	Wald ratio	1	-0.00607	0.08946	0.945947542	0.99395326	0.8340958	1.184448	0.999221
NEU1	Wald ratio	1	-0.00622	0.091813	0.945947542	0.99379474	0.8301262	1.1897323	0.999221
GAA	Inverse variance weighted	5	-0.00188	0.02785	0.946230151	0.99812349	0.9450995	1.0541224	0.999221
CCR4	Inverse variance weighted	3	-0.00435	0.064619	0.946320524	0.99565877	0.8772142	1.1300961	0.999221
C8G	Wald ratio	1	-0.01071	0.159309	0.946401078	0.98934732	0.7240065	1.3519327	0.999221
IL5RA	Wald ratio	1	-0.0153	0.229529	0.946847071	0.98481454	0.6280228	1.5443066	0.999221
APOBEC3G	Inverse variance weighted	5	-0.00178	0.027039	0.947406531	0.99821796	0.9466925	1.0525478	0.999221
NAGA	Inverse variance weighted	4	-0.00251	0.038397	0.947853096	0.99749188	0.925178	1.075458	0.999221
SLC29A3	Inverse variance weighted	3	0.004578	0.070842	0.948479743	1.00458805	0.8743507	1.1542247	0.999221
P2RX4	Inverse variance weighted	5	-0.00165	0.025485	0.948531016	0.99835623	0.949712	1.049492	0.999221
HSD17B1P1	Inverse variance weighted	2	0.002051	0.031918	0.948762944	1.00205314	0.9412868	1.0667423	0.999221
SLC25A42	Wald ratio	1	-0.00957	0.150006	0.94910576	0.99047085	0.7381661	1.3290131	0.999221
CNTF	Wald ratio	1	-0.01372	0.214971	0.94910576	0.98637217	0.647223	1.5032379	0.999221
MMP28	Inverse variance weighted	2	-0.00628	0.099167	0.949474703	0.99373587	0.8181985	1.2069333	0.999221
ADRBK2	Inverse variance weighted	3	0.004823	0.076648	0.949825471	1.00483481	0.8646701	1.1677204	0.999221
APOL1	Wald ratio	1	0.005143	0.082284	0.950164662	1.00515602	0.855444	1.1810693	0.999221
CYP2D6	Inverse variance weighted	4	0.002152	0.034542	0.950317789	1.00215454	0.9365523	1.0723519	0.999221
IGLV10-54	Inverse variance weighted	3	0.002283	0.036684	0.950376598	1.00228558	0.9327509	1.0770039	0.999221
COL18A1	Inverse variance weighted	6	0.001123	0.018077	0.950442817	1.00112413	0.9662744	1.0372308	0.999221
SLC2A8	Inverse variance weighted	3	0.002839	0.045713	0.950479089	1.00284303	0.9168991	1.0968428	0.999221
SLC25A15	Wald ratio	1	0.006763	0.109051	0.950550485	1.00678574	0.813039	1.2467023	0.999221
LILRA4	Inverse variance weighted	2	0.009269	0.152799	0.951629479	1.00931198	0.7481009	1.361729	0.999221
TUBG2	Wald ratio	1	-0.00497	0.081979	0.951672947	0.9950439	0.8473451	1.1684877	0.999221
SLC7A8	Inverse variance weighted	4	-0.00176	0.029111	0.951674772	0.9982373	0.9428743	1.0568511	0.999221
SCARA5	Inverse variance weighted	2	-0.00331	0.054671	0.951719814	0.99669527	0.8954165	1.1094294	0.999221
TPCN1	Wald ratio	1	-0.00571	0.095338	0.952251018	0.99430738	0.8248349	1.1986	0.999221
CKLF	Inverse variance weighted	2	-0.00528	0.088418	0.952425606	0.99473879	0.8364621	1.1829648	0.999221
CSNK1E	Inverse variance weighted	4	-0.00328	0.055112	0.952497163	0.9967223	0.8946682	1.1104176	0.999221
SLC22A3	Inverse variance weighted	2	-0.0064	0.109837	0.953523604	0.99361885	0.8011694	1.2322967	0.999221
F13A1	Inverse variance weighted	3	0.002106	0.036166	0.953557553	1.00210855	0.9335322	1.0757224	0.999221
CSNK2A2	Inverse variance weighted	2	0.015124	0.259857	0.953588338	1.01523892	0.6100618	1.6895174	0.999221
LOXL3	Inverse variance weighted	3	0.003918	0.067335	0.953599466	1.0039257	0.8798026	1.1455602	0.999221
MTRR	Inverse variance weighted	4	0.001722	0.029614	0.95363167	1.00172346	0.9452348	1.061588	0.999221
HLA-DPB1	Inverse variance weighted	5	0.002234	0.038698	0.953965801	1.0022364	0.9290309	1.0812103	0.999221
RSPO4	Inverse variance weighted	3	-0.0029	0.050446	0.954227266	0.99710861	0.9032369	1.1007363	0.999221
GPR55	Inverse variance weighted	4	-0.00444	0.07903	0.95522261	0.9955723	0.8527088	1.1623712	0.999221
EVI2B	Wald ratio	1	-0.0051	0.091	0.955282246	0.99491017	0.832382	1.189173	0.999221
GBE1	Inverse variance weighted	2	0.003547	0.063565	0.955506236	1.00355282	0.8859977	1.1367052	0.999221
CACNA1A	Wald ratio	1	0.007346	0.132224	0.955695871	1.00737284	0.7773896	1.3053945	0.999221

CHRNB2	Wald ratio	1	-0.00382	0.068803	0.955695871	0.9961849	0.8705094	1.1400041	0.999221
GRIN2C	Wald ratio	1	0.015493	0.281456	0.956101919	1.01561362	0.5849901	1.7632283	0.999221
MAPKAPK3	Inverse variance weighted	3	-0.00213	0.039231	0.956707464	0.9978726	0.9240199	1.077628	0.999221
ERO1L	Inverse variance weighted	5	0.001351	0.025008	0.956914106	1.00135198	0.9534544	1.0516557	0.999221
ZRANB3	Wald ratio	1	0.006869	0.127772	0.957123637	1.00689309	0.7838302	1.2934353	0.999221
MINPP1	Inverse variance weighted	2	-0.00851	0.158713	0.957252262	0.99152876	0.7264509	1.3533321	0.999221
VPREB1	Inverse variance weighted	2	-0.00431	0.080578	0.957390752	0.99570413	0.8502392	1.1660563	0.999221
SCYL2	Wald ratio	1	-0.00547	0.103079	0.957688355	0.9945461	0.81261	1.2172161	0.999221
MAP4K3	Wald ratio	1	-0.00977	0.184689	0.957803575	0.9902757	0.6895186	1.4222184	0.999221
MMP1	Inverse variance weighted	2	-0.00854	0.161707	0.95790554	0.99150107	0.7221794	1.3612605	0.999221
ERMAP	Inverse variance weighted	7	-0.00113	0.021959	0.959032377	0.99887266	0.9567941	1.0428018	0.999221
CD46	Wald ratio	1	0.008044	0.15954	0.959787565	1.00807645	0.7373789	1.3781492	0.999221
CHADL	Wald ratio	1	-0.00336	0.067275	0.960217031	0.99664985	0.8735278	1.1371257	0.999221
WNT10B	Wald ratio	1	0.004339	0.087639	0.960516892	1.00434798	0.8458328	1.1925701	0.999221
NPEPPS	Wald ratio	1	0.009553	0.194239	0.960775588	1.0095985	0.6899369	1.4773657	0.999221
BTNL3	Inverse variance weighted	7	0.0014	0.028642	0.961002762	1.00140144	0.9467331	1.0592265	0.999221
RELB	Inverse variance weighted	2	0.008783	0.180159	0.961119264	1.00882128	0.7086953	1.4360478	0.999221
CA3	Wald ratio	1	0.010917	0.2249	0.961282946	1.01097727	0.6505835	1.5710129	0.999221
GH1	Wald ratio	1	0.00607	0.125045	0.961282946	1.00608863	0.7874001	1.2855146	0.999221
COMTD1	Inverse variance weighted	2	0.003292	0.068384	0.961609799	1.00329696	0.8774458	1.1471989	0.999221
ATP2B4	Wald ratio	1	0.006948	0.144753	0.961716243	1.00697233	0.7582308	1.3373148	0.999221
C2	Inverse variance weighted	3	-0.00322	0.067152	0.961741456	0.99678399	0.8738557	1.137005	0.999221
KIR2DL4	Wald ratio	1	-0.00462	0.098446	0.962576334	0.9953915	0.8207206	1.207237	0.999221
SOAT2	Inverse variance weighted	2	0.002887	0.061656	0.962647876	1.00289161	0.8887324	1.1317147	0.999221
CYP4F2	Wald ratio	1	-0.00539	0.115967	0.962902466	0.9946207	0.7923995	1.2484489	0.999221
STYXL1	Inverse variance weighted	13	0.000794	0.017154	0.963093803	1.00079405	0.9677052	1.0350143	0.999221
FCGR1A	Inverse variance weighted	2	-0.00164	0.035515	0.963180268	0.99836187	0.9312304	1.0703328	0.999221
IGF2R	Inverse variance weighted	2	-0.00543	0.117926	0.963284103	0.99458622	0.789335	1.253209	0.999221
YWHAG	Inverse variance weighted	3	-0.00197	0.042892	0.963456579	0.99803678	0.9175637	1.0855676	0.999221
VKORC1L1	Inverse variance weighted	3	0.001859	0.0407	0.963559235	1.00186123	0.925045	1.0850563	0.999221
GPR25	Wald ratio	1	-0.00351	0.077163	0.963745005	0.99649876	0.8566328	1.1592012	0.999221
CYB5R1	Inverse variance weighted	3	-0.0028	0.062527	0.964230889	0.99719991	0.882182	1.1272137	0.999221
ADRB2	Inverse variance weighted	3	-0.00317	0.071145	0.964412813	0.99683078	0.8670848	1.1459912	0.999221
KIR3DL1	Inverse variance weighted	2	-0.00667	0.152423	0.965102054	0.99335339	0.7368161	1.3392093	0.999221
BBS2	Inverse variance weighted	8	0.001089	0.025105	0.965413878	1.00108919	0.9530212	1.0515816	0.999221
TERT	Inverse variance weighted	2	0.015339	0.356825	0.965711694	1.01545718	0.5045753	2.0436064	0.999221
SLCO4A1	Inverse variance weighted	2	-0.0059	0.138499	0.966034294	0.99411973	0.7577855	1.3041606	0.999221
SLC25A28	Inverse variance weighted	2	-0.00308	0.073099	0.966357838	0.99692167	0.8638492	1.1504935	0.999221
LPAR6	Inverse variance weighted	3	0.003837	0.09127	0.966468979	1.00384411	0.8394131	1.2004851	0.999221
S100B	Inverse variance weighted	4	0.001121	0.026775	0.966608624	1.00112147	0.9499392	1.0550614	0.999221
DGCR6	Inverse variance weighted	3	-0.00143	0.034418	0.966899261	0.99857278	0.9334325	1.0682589	0.999221
GBAP1	Inverse variance weighted	7	0.001477	0.035793	0.967081315	1.00147825	0.9336277	1.0742597	0.999221
GRK4	Inverse variance weighted	3	0.001254	0.0304	0.967108047	1.00125435	0.9433384	1.0627261	0.999221
GPR68	Inverse variance weighted	2	0.002343	0.057637	0.967579624	1.00234535	0.8952738	1.1222223	0.999221
LRP1	Wald ratio	1	0.008084	0.199396	0.967662187	1.00811638	0.6819953	1.4901843	0.999221
TNFSF12	Inverse variance weighted	5	-0.0021	0.052906	0.968334454	0.99790198	0.8996082	1.1069356	0.999221
DEFA3	Inverse variance weighted	4	-0.00079	0.020042	0.968526527	0.99920952	0.9607189	1.0392422	0.999221
DSTYK	Inverse variance weighted	7	0.001857	0.047293	0.96867244	1.00185907	0.9131671	1.0991653	0.999221
PTP4A2	Wald ratio	1	-0.00839	0.213897	0.968718427	0.99164697	0.6520553	1.5080987	0.999221
ADAMTS13	Wald ratio	1	-0.00791	0.203752	0.969021979	0.99211853	0.6654668	1.4791108	0.999221
PDIA4	Inverse variance weighted	3	0.00345	0.089141	0.969126397	1.00345609	0.8425965	1.1950253	0.999221
KCNC4	Wald ratio	1	0.008284	0.215372	0.969319697	1.00831793	0.6611036	1.5378907	0.999221
TNFSF13B	Inverse variance weighted	4	-0.0042	0.109746	0.969452344	0.9958061	0.8030776	1.234787	0.999221
CD36	Inverse variance weighted	5	0.001222	0.032927	0.970397991	1.00122263	0.9386484	1.0679683	0.999221
COL5A3	Inverse variance weighted	3	0.002155	0.05814	0.970431469	1.00215739	0.8942243	1.123118	0.999221
EGLN2	Inverse variance weighted	3	0.002022	0.055343	0.970859285	1.00202377	0.899019	1.1168303	0.999221
TP53I3	Inverse variance weighted	6	-0.00131	0.035958	0.970892168	0.99868877	0.9307265	1.0716138	0.999221
LAMA5	Inverse variance weighted	2	-0.00156	0.042891	0.971050461	0.99844466	0.9179405	1.0860091	0.999221
AHCYL1	Inverse variance weighted	2	-0.00347	0.096123	0.971170441	0.9965321	0.8254096	1.2031314	0.999221
SLAMF1	Inverse variance weighted	3	-0.00197	0.054549	0.971252415	0.99803612	0.8968366	1.110655	0.999221
PLBD2	Inverse variance weighted	3	-0.00295	0.08485	0.972238236	0.99705147	0.8442903	1.1774524	0.999221
DMKN	Wald ratio	1	-0.00315	0.091425	0.972492191	0.99685239	0.8333139	1.1924854	0.999221
M6PR	Wald ratio	1	-0.00938	0.274443	0.972727208	0.9906612	0.5785144	1.6964308	0.999221

SEPP1	Wald ratio	1	0.005046	0.147589	0.972727208	1.00505853	0.7525945	1.3422138	0.999221
EBI3	Wald ratio	1	0.008971	0.262407	0.972727208	1.00901154	0.6032966	1.6875683	0.999221
MTMR4	Inverse variance weighted	2	0.006875	0.204726	0.973211856	1.00689844	0.6740922	1.5040145	0.999221
BMP2K	Inverse variance weighted	2	0.002482	0.07506	0.973625781	1.00248464	0.8653378	1.1613678	0.999221
GP1BA	Wald ratio	1	0.004115	0.12655	0.974057107	1.00412394	0.7835479	1.2867942	0.999221
GPR18	Wald ratio	1	-0.00503	0.156562	0.974348968	0.99497849	0.7320572	1.352329	0.999221
ITM2B	Inverse variance weighted	3	-0.00137	0.042692	0.974400533	0.99863096	0.9184696	1.0857886	0.999221
LGALS1	Inverse variance weighted	2	0.001104	0.035164	0.974963538	1.00110419	0.9344305	1.0725352	0.999221
CXCL1	Inverse variance weighted	3	-0.00134	0.042874	0.975063124	0.9986607	0.9181692	1.0862086	0.999221
PPT1	Inverse variance weighted	5	-0.00075	0.024166	0.975285773	0.99925162	0.9530249	1.0477206	0.999221
POLR2C	Inverse variance weighted	2	-0.00282	0.094092	0.976115368	0.9971869	0.8292466	1.1991387	0.999221
CASP2	Inverse variance weighted	4	-0.00275	0.09285	0.976387233	0.99725555	0.8313253	1.1963051	0.999221
COL26A1	Inverse variance weighted	4	-0.00079	0.026971	0.976598645	0.99920917	0.9477606	1.0534507	0.999221
SLC24A1	Inverse variance weighted	3	-0.00258	0.088603	0.976742762	0.99742032	0.8384129	1.1865839	0.999221
GPR85	Wald ratio	1	0.003436	0.121393	0.977421376	1.00344155	0.7909712	1.2729857	0.999221
NCSTN	Inverse variance weighted	5	0.000851	0.030299	0.977601881	1.00085102	0.9431454	1.0620873	0.999221
HSP90AA1	Inverse variance weighted	3	-0.00129	0.046218	0.977727773	0.99871053	0.9122168	1.0934053	0.999221
CDK4	Wald ratio	1	0.003988	0.144223	0.977941486	1.0039957	0.7567747	1.3319781	0.999221
FAM20C	Inverse variance weighted	4	-0.00139	0.050752	0.978227889	0.99861589	0.9040598	1.1030617	0.999221
UBA2	Wald ratio	1	0.002675	0.100324	0.9787256	1.0026789	0.8236906	1.2205614	0.999221
TRPM5	Wald ratio	1	0.006554	0.252346	0.979278056	1.00657598	0.6138256	1.6506239	0.999221
CYP27A1	Inverse variance weighted	4	-0.00097	0.037926	0.979648458	0.99903298	0.9274623	1.0761267	0.999221
DHCR7	Wald ratio	1	0.005799	0.230021	0.979887394	1.0058157	0.6407971	1.5787606	0.999221
SERPIND1	Wald ratio	1	-0.0066	0.262546	0.979954758	0.99342507	0.5938149	1.6619545	0.999221
RPS6KA1	Inverse variance weighted	2	-0.00121	0.048736	0.980159147	0.99878869	0.9077963	1.0989016	0.999221
SHISA2	Inverse variance weighted	3	0.002148	0.089236	0.980797425	1.00215014	0.8413443	1.1936907	0.999221
ADCY5	Wald ratio	1	0.005734	0.242736	0.981154086	1.0057504	0.6249842	1.6184951	0.999221
RNASE1	Inverse variance weighted	4	-0.00118	0.05005	0.981227361	0.998823	0.9054923	1.1017735	0.999221
GSTM2	Inverse variance weighted	6	-0.00062	0.026718	0.981590933	0.99938369	0.9483952	1.0531135	0.999221
UCHL1	Inverse variance weighted	2	0.002522	0.122356	0.983555931	1.00252507	0.7887575	1.2742275	0.999221
TMPRSS13	Wald ratio	1	0.002397	0.117437	0.983717772	1.00239954	0.7963	1.2618421	0.999221
C12orf39	Inverse variance weighted	4	0.001018	0.05008	0.983783283	1.00101844	0.9074304	1.1042586	0.999221
MKNK2	Inverse variance weighted	3	-0.00076	0.038129	0.984021	0.99923664	0.9272831	1.0767735	0.999221
BMP8A	Inverse variance weighted	4	-0.00053	0.026556	0.984053472	0.99946936	0.9487777	1.0528694	0.999221
TRIM39	Wald ratio	1	-0.00286	0.145127	0.984268084	0.99714243	0.7502784	1.3252321	0.999221
SLC25A1	Inverse variance weighted	2	-0.00081	0.041231	0.984287639	0.99918833	0.9216172	1.0832885	0.999221
CRYZ	Inverse variance weighted	7	0.00025	0.012768	0.9843826	1.00024995	0.9755299	1.0255965	0.999221
DHRS7B	Wald ratio	1	-0.00152	0.078099	0.984508069	0.99848466	0.856766	1.1636452	0.999221
TRIM35	Inverse variance weighted	3	-0.00056	0.030463	0.985286034	0.99943835	0.9415108	1.06093	0.999221
HDGF	Wald ratio	1	-0.00169	0.091555	0.985293283	0.99831378	0.8343226	1.1945385	0.999221
SPRYD3	Inverse variance weighted	3	0.001371	0.075376	0.9854919	1.00137159	0.8638415	1.1607974	0.999221
GYPA	Wald ratio	1	0.001334	0.073386	0.985493807	1.00133518	0.8671855	1.1562372	0.999221
GPC2	Wald ratio	1	0.00196	0.109766	0.985752819	1.00196203	0.80801	1.2424696	0.999221
LDHB	Wald ratio	1	0.002629	0.147552	0.985784546	1.00263246	0.7508317	1.3388778	0.999221
SLC25A39	Inverse variance weighted	2	-0.00149	0.083422	0.985797086	0.99851605	0.8478998	1.1758869	0.999221
PPIL6	Wald ratio	1	0.003332	0.193285	0.986244051	1.00333805	0.6869421	1.4654616	0.999221
CD4	Inverse variance weighted	2	-0.00139	0.083317	0.986642249	0.99860606	0.8481512	1.1757503	0.999221
F12	Wald ratio	1	-0.00201	0.120857	0.98670254	0.99798774	0.7874983	1.2647386	0.999221
SENP7	Inverse variance weighted	8	0.000922	0.057023	0.987101544	1.00092229	0.8950787	1.119282	0.999221
SELP	Inverse variance weighted	3	-0.00111	0.069308	0.987253219	0.99889332	0.8720126	1.1442356	0.999221
REG4	Wald ratio	1	0.004064	0.264185	0.987725337	1.00407266	0.598255	1.6851709	0.999221
CASP8	Inverse variance weighted	4	0.000633	0.0418	0.987921366	1.000633	0.921922	1.0860641	0.999221
LPCAT3	Inverse variance weighted	2	-0.00266	0.185558	0.988545582	0.99733958	0.6932545	1.4348067	0.999221
MBOAT1	Inverse variance weighted	4	-0.00066	0.047466	0.988986056	0.99934498	0.9105668	1.0967788	0.999221
F11R	Inverse variance weighted	2	-0.00079	0.064528	0.990262859	0.99921281	0.8805019	1.1339285	0.999221
ABCA2	Inverse variance weighted	2	-0.00045	0.036851	0.990339454	0.9995539	0.9299032	1.0744215	0.999221
POLE	Wald ratio	1	0.001925	0.165579	0.990722482	1.00192719	0.7242569	1.3860526	0.999221
C3	Inverse variance weighted	2	-0.0014	0.126706	0.991191677	0.99860217	0.7790017	1.2801079	0.999221
SLC45A3	Wald ratio	1	0.000866	0.085727	0.991940697	1.00086631	0.8460645	1.1839917	0.999221
DCPS	Inverse variance weighted	4	0.000351	0.035319	0.992075484	1.00035085	0.9334436	1.0720539	0.999221
SLC4A7	Inverse variance weighted	2	0.000546	0.055273	0.992112757	1.00054654	0.897817	1.1150305	0.999221
CRY2	Inverse variance weighted	3	0.000393	0.041164	0.992373584	1.00039354	0.9228514	1.0844511	0.999221
ENTPD1	Inverse variance weighted	5	0.000276	0.029484	0.992535079	1.00027589	0.9441107	1.0597824	0.999221

GDF11	Wald ratio	1	0.001482	0.160094	0.992612286	1.00148345	0.7317602	1.3706254	0.999221
YBX1	Wald ratio	1	0.001223	0.132062	0.992612286	1.00122354	0.7728903	1.2970128	0.999221
PEAK1	Inverse variance weighted	2	-0.00079	0.089688	0.992950182	0.99920785	0.8381312	1.1912412	0.999221
ALPL	Wald ratio	1	-0.00075	0.086278	0.993061961	0.99925004	0.8437867	1.1833567	0.999221
AKR1C1	Inverse variance weighted	3	0.000435	0.052189	0.99335593	1.00043468	0.9031598	1.1081865	0.999221
LEPRE1	Inverse variance weighted	3	0.000368	0.047448	0.993809438	1.00036821	0.9115308	1.0978637	0.999221
MCOLN3	Inverse variance weighted	3	0.000608	0.081909	0.994078963	1.00060803	0.8522008	1.1748598	0.999221
OR6N1	Wald ratio	1	-0.00085	0.118715	0.994300873	0.99915239	0.7917339	1.2609104	0.999221
SULT2B1	Inverse variance weighted	2	0.00062	0.087	0.99431378	1.00062021	0.8437494	1.1866566	0.999221
GANAB	Wald ratio	1	0.000347	0.055569	0.995013254	1.00034737	0.8971177	1.1154554	0.999221
AEBP1	Inverse variance weighted	3	0.000329	0.059995	0.995625584	1.00032898	0.889352	1.1251542	0.999221
HLA-DPA1	Inverse variance weighted	7	-0.00015	0.028805	0.995800523	0.9998484	0.9449639	1.0579206	0.999221
SULT1A1	Inverse variance weighted	3	0.000202	0.040683	0.996031832	1.00020235	0.9235438	1.0832239	0.999221
TXNDC5	Inverse variance weighted	2	0.000662	0.136258	0.996121024	1.00066265	0.7661306	1.3069908	0.999221
COL5A2	Wald ratio	1	0.000306	0.071089	0.996560853	1.00030647	0.8702031	1.1498615	0.999221
TESK2	Inverse variance weighted	3	0.00016	0.037978	0.996644633	1.00015972	0.9284148	1.0774489	0.999221
HSD11B2	Wald ratio	1	0.000994	0.245484	0.996769707	1.00099436	0.6186873	1.6195413	0.999221
ADCY10	Inverse variance weighted	3	0.000621	0.156634	0.996835116	1.0006215	0.7361053	1.3601903	0.999221
IVNS1ABP	Inverse variance weighted	4	0.000149	0.040707	0.997077003	1.00014914	0.9234525	1.0832158	0.999221
EP300	Inverse variance weighted	2	0.000176	0.051522	0.997274962	1.00017598	0.9041073	1.1064527	0.999221
IL8	Inverse variance weighted	2	0.000123	0.037241	0.997368685	1.00012282	0.9297221	1.0758544	0.999221
GRIK3	Inverse variance weighted	2	0.000894	0.359868	0.998018445	1.00089414	0.4943821	2.0263457	0.999262
VCAM1	Inverse variance weighted	2	-9.2E-05	0.041385	0.998227158	0.99990805	0.9220024	1.0843964	0.999262
PTPN2	Inverse variance weighted	2	0.000191	0.091749	0.998335687	1.0001914	0.8355739	1.1972404	0.999262
LMTK2	Inverse variance weighted	3	8.68E-05	0.061085	0.998865701	1.00008684	0.8872406	1.1272858	0.999342
CYP4V2	Inverse variance weighted	6	2.66E-05	0.021954	0.999032802	1.00002661	0.957908	1.0439972	0.999342
IL12RB2	Inverse variance weighted	4	-2.9E-06	0.027807	0.999916104	0.99999708	0.9469538	1.0560116	0.999916

Table S6. MR results of the association between brain eQTL and ischemic stroke (IVW or Wald ratio methods).

Druggable gene	Method	Nsnp	beta	se	P value	OR	LL	LR	FDR
MMP3	Wald ratio	1	0.4791311	0.099286	1.39E-06	1.614671	1.329137	1.961544	0.00488292
MAPKAPK5	Wald ratio	1	0.5450574	0.122495	8.6E-06	1.724707	1.356579	2.192733	0.01003916
DNMT1	Wald ratio	1	-0.879285	0.194294	6.02E-06	0.415079	0.283625	0.60746	0.01003916
ALDH2	Wald ratio	1	0.2366382	0.055216	1.82E-05	1.266983	1.137026	1.411793	0.01594294
HSD17B12	Inverse variance weighted	3	-0.082515	0.020159	4.26E-05	0.920798	0.885124	0.957909	0.02980458
SIAE	Inverse variance weighted	2	0.5129059	0.132485	0.000108	1.670137	1.288185	2.16534	0.06313822
NEK3	Wald ratio	1	-0.369533	0.096604	0.000131	0.691057	0.571851	0.835112	0.06381582
THSD1	Wald ratio	1	-0.282747	0.074445	0.000146	0.753711	0.651382	0.872114	0.06381582
NMB	Wald ratio	1	-0.407517	0.110087	0.000214	0.6653	0.536178	0.825517	0.08329028
NPC1	Wald ratio	1	-0.269858	0.076571	0.000425	0.763488	0.657087	0.887117	0.14866938
FURIN	Wald ratio	1	0.695381	0.200398	0.00052	2.004473	1.353374	2.96881	0.1556742
FGF2	Wald ratio	1	-0.859739	0.248243	0.000534	0.423272	0.260203	0.688539	0.1556742
CHST10	Inverse variance weighted	2	0.4145943	0.121632	0.000653	1.513756	1.19267	1.921284	0.17586006
PROCR	Wald ratio	1	0.2902972	0.086428	0.000783	1.336825	1.12851	1.583593	0.1957435
ADAMTS19	Wald ratio	1	0.9000966	0.271727	0.000925	2.459841	1.444136	4.189921	0.19704958
CD5	Inverse variance weighted	2	0.2248184	0.067832	0.000919	1.252095	1.09622	1.430135	0.19704958
MIPEP	Inverse variance weighted	2	-0.24818	0.075139	0.000957	0.78022	0.673375	0.904018	0.19704958
COL19A1	Wald ratio	1	0.4257227	0.129619	0.001022	1.530696	1.187284	1.973437	0.19877594
ADAM15	Wald ratio	1	-0.785959	0.249151	0.001607	0.455683	0.279628	0.742582	0.20656905
SLC18A2	Wald ratio	1	-0.326653	0.102018	0.001365	0.721334	0.590605	0.880999	0.20656905
GUCY1A2	Wald ratio	1	-1.317962	0.405853	0.001165	0.26768	0.120822	0.593041	0.20656905
SERPINA3	Wald ratio	1	0.6592154	0.208765	0.00159	1.933275	1.284072	2.910703	0.20656905
CARM1	Wald ratio	1	0.6332377	0.198009	0.001384	1.8837	1.2778	2.776902	0.20656905
SSC5D	Wald ratio	1	-0.397477	0.126322	0.001652	0.672013	0.524627	0.860805	0.20656905
CCR2	Wald ratio	1	-0.291393	0.09132	0.001418	0.747222	0.624765	0.893681	0.20656905
HLA-DPA1	Wald ratio	1	0.414232	0.130439	0.001495	1.513208	1.171836	1.954027	0.20656905
RIOK1	Wald ratio	1	-0.300466	0.095475	0.001649	0.740473	0.6141	0.892852	0.20656905
IMPDH1	Wald ratio	1	-0.523988	0.164776	0.001473	0.592154	0.428721	0.81789	0.20656905
GRK5	Wald ratio	1	0.4426628	0.142038	0.00183	1.556847	1.17853	2.056607	0.22093564
F11	Wald ratio	1	-0.106061	0.034213	0.001935	0.89937	0.841038	0.961748	0.22583859
PDE3A	Inverse variance weighted	2	0.143746	0.046852	0.002154	1.154591	1.053288	1.265637	0.24328926
CDK1	Wald ratio	1	-0.202246	0.066273	0.002275	0.816894	0.717386	0.930204	0.24893325
HTR1B	Wald ratio	1	-0.40539	0.134293	0.002539	0.666717	0.512423	0.867469	0.26933174
PTGDR	Inverse variance weighted	2	0.1178648	0.039222	0.002655	1.125092	1.041842	1.214994	0.2733736
CYB5R1	Wald ratio	1	-0.673794	0.226547	0.002938	0.509771	0.32699	0.794722	0.29384365
SLC7A11	Wald ratio	1	0.255739	0.088186	0.003732	1.291416	1.086427	1.535081	0.35309256
PARP4	Inverse variance weighted	2	0.2547142	0.087786	0.003713	1.290093	1.086166	1.532308	0.35309256
VEGFB	Wald ratio	1	-0.569223	0.197342	0.003921	0.565965	0.384422	0.833241	0.36088268
TSHR	Wald ratio	1	0.3644954	0.126889	0.004072	1.439787	1.122763	1.846327	0.36088268
GPR151	Wald ratio	1	0.3274295	0.114455	0.004226	1.387397	1.108599	1.73631	0.36088268
FAM234A	Inverse variance weighted	2	0.3098856	0.108311	0.004222	1.363269	1.102518	1.685689	0.36088268
INPP5K	Inverse variance weighted	2	-0.308151	0.108399	0.004473	0.734804	0.594156	0.908747	0.37283966
KLHL35	Wald ratio	1	-0.333933	0.119014	0.005019	0.716101	0.56711	0.904236	0.37384649
CDKN1A	Wald ratio	1	0.4564764	0.161754	0.004772	1.578502	1.149627	2.167371	0.37384649
SHARPIN	Wald ratio	1	0.6293118	0.223684	0.004902	1.876319	1.210328	2.908776	0.37384649
PTPN3	Wald ratio	1	-0.533974	0.190156	0.004984	0.586271	0.403863	0.851064	0.37384649
PI4K2B	Inverse variance weighted	2	-0.090557	0.032085	0.004766	0.913422	0.857749	0.972709	0.37384649
ERP44	Wald ratio	1	0.4581769	0.163753	0.005142	1.581189	1.147082	2.17958	0.3750689
ERP29	Wald ratio	1	-0.690125	0.247249	0.005251	0.501514	0.308901	0.814227	0.37518993
ADRB1	Wald ratio	1	-0.288969	0.106539	0.006681	0.749036	0.607875	0.922976	0.40329281
MAT1A	Wald ratio	1	0.1543711	0.056874	0.006642	1.166924	1.043832	1.304531	0.40329281
SLC16A12	Wald ratio	1	0.4280381	0.156551	0.006254	1.534245	1.128849	2.085228	0.40329281
DYRK1A	Wald ratio	1	-0.649006	0.238702	0.00655	0.522565	0.327305	0.83431	0.40329281
CYP4V2	Wald ratio	1	-0.09619	0.035133	0.006184	0.908292	0.847851	0.973041	0.40329281
RARS1	Wald ratio	1	-0.321632	0.118052	0.00644	0.724965	0.575213	0.913703	0.40329281
MSMP	Wald ratio	1	0.1830482	0.066563	0.00596	1.200872	1.053992	1.368221	0.40329281
VSIG2	Inverse variance weighted	2	-0.242618	0.089072	0.006453	0.784571	0.65889	0.934225	0.40329281
SLC4A8	Inverse variance weighted	2	0.2833717	0.104142	0.006508	1.327598	1.082478	1.628225	0.40329281
MMP15	Wald ratio	1	0.5547443	0.205385	0.006913	1.741496	1.164381	2.604652	0.41022243
OR5L2	Wald ratio	1	-0.35208	0.130819	0.007116	0.703224	0.544174	0.908759	0.41522899
PMS2P3	Wald ratio	1	-0.164436	0.061738	0.007734	0.848372	0.751683	0.9575	0.44388808
KCNH1	Wald ratio	1	-0.478843	0.180937	0.008134	0.6195	0.434535	0.883197	0.45929736

TLR1	Wald ratio	1	-0.151332	0.057889	0.008944	0.859562	0.767363	0.962839	0.49705128
MASP2	Wald ratio	1	0.6615972	0.257054	0.01006	1.937885	1.170899	3.207278	0.5418308
EMILIN2	Inverse variance weighted	2	-0.197931	0.076851	0.010009	0.820426	0.705704	0.953798	0.5418308
LAMC1	Wald ratio	1	-0.169584	0.066504	0.010772	0.844016	0.740869	0.961523	0.55461462
PSKH1	Wald ratio	1	-0.357424	0.13978	0.010556	0.699476	0.53185	0.919932	0.55461462
ERAP2	Inverse variance weighted	4	-0.029695	0.01163	0.01067	0.970742	0.948864	0.993124	0.55461462
C5AR1	Wald ratio	1	-0.381578	0.150196	0.011068	0.682783	0.508667	0.916499	0.55470134
CNTNAP3	Wald ratio	1	-0.568487	0.223829	0.011091	0.566382	0.365243	0.878287	0.55470134
PMS2	Wald ratio	1	-0.152865	0.060447	0.011441	0.858245	0.762356	0.966195	0.5641653
CYP2C18	Wald ratio	1	0.3321693	0.135216	0.014027	1.393989	1.069452	1.817011	0.58460673
ESRRB	Wald ratio	1	0.1685738	0.067685	0.012754	1.183616	1.036564	1.351529	0.58460673
FSD2	Wald ratio	1	0.2709265	0.108746	0.012725	1.311179	1.059487	1.622662	0.58460673
ABCA5	Wald ratio	1	0.5640593	0.22846	0.01355	1.757794	1.123308	2.750659	0.58460673
MMP9	Wald ratio	1	-0.247047	0.10039	0.01386	0.781104	0.641586	0.950961	0.58460673
ACP3	Wald ratio	1	0.0626662	0.025311	0.013294	1.064671	1.013141	1.118822	0.58460673
PROS1	Wald ratio	1	-0.213444	0.085883	0.012944	0.807797	0.682649	0.955889	0.58460673
HSD17B11	Wald ratio	1	0.218025	0.088474	0.013729	1.243618	1.045626	1.4791	0.58460673
BMP1	Wald ratio	1	0.3906068	0.157503	0.013138	1.477877	1.085349	2.012368	0.58460673
POMK	Wald ratio	1	0.5868268	0.233722	0.012046	1.798273	1.137385	2.843176	0.58460673
NAGLU	Inverse variance weighted	2	0.3061278	0.123208	0.012968	1.358156	1.066776	1.729123	0.58460673
PDYN	Inverse variance weighted	2	-0.180544	0.07327	0.013737	0.834816	0.723139	0.96374	0.58460673
RRM2B	Inverse variance weighted	2	0.4528823	0.182997	0.013331	1.572839	1.098787	2.251412	0.58460673
RHD	Wald ratio	1	0.0552692	0.02257	0.014333	1.056825	1.011094	1.104625	0.58503489
MBNL1	Wald ratio	1	0.8134159	0.332298	0.014371	2.2556	1.175992	4.326329	0.58503489
CTSW	Wald ratio	1	-0.114539	0.047408	0.015692	0.891777	0.812646	0.978614	0.60746338
NEK6	Wald ratio	1	-0.300256	0.124073	0.015521	0.740629	0.580748	0.944525	0.60746338
OGA	Inverse variance weighted	2	-0.691233	0.284668	0.015174	0.500958	0.286739	0.875217	0.60746338
OR3A1	Inverse variance weighted	2	0.1394758	0.057536	0.015344	1.149671	1.027066	1.286912	0.60746338
FRK	Inverse variance weighted	2	0.1120176	0.04642	0.015817	1.118533	1.021256	1.225074	0.60746338
HILPDA	Inverse variance weighted	2	-0.255392	0.105982	0.015963	0.774613	0.629319	0.953452	0.60746338
RARG	Wald ratio	1	0.2736564	0.114024	0.016395	1.314763	1.05145	1.644017	0.61062922
GGT1	Wald ratio	1	-0.413073	0.172072	0.016369	0.661614	0.472209	0.92699	0.61062922
CDK18	Wald ratio	1	0.6310853	0.26451	0.017039	1.87965	1.119236	3.15669	0.62138349
PDGFRL	Wald ratio	1	0.1657663	0.069437	0.016973	1.180297	1.030114	1.352376	0.62138349
MATN2	Wald ratio	1	0.195221	0.081993	0.017268	1.21558	1.035118	1.427503	0.62324816
OR51Q1	Inverse variance weighted	3	-0.124811	0.052688	0.017842	0.882664	0.796061	0.978688	0.62466559
MLYCD	Inverse variance weighted	2	0.1499061	0.063198	0.017691	1.161725	1.026381	1.314917	0.62466559
THBD	Inverse variance weighted	2	-0.180709	0.076104	0.017573	0.834678	0.719015	0.968948	0.62466559
GPI	Wald ratio	1	0.6152412	0.261455	0.018615	1.850103	1.108259	3.088521	0.64526829
PTPRZ1	Wald ratio	1	-0.549091	0.234477	0.019192	0.577475	0.364705	0.914373	0.6523562
PXDNL	Wald ratio	1	-0.193529	0.082622	0.019163	0.824046	0.700846	0.968904	0.6523562
F7	Wald ratio	1	0.1177331	0.050638	0.020072	1.124944	1.018655	1.242323	0.66294276
PDPK1	Wald ratio	1	0.1049752	0.045048	0.019792	1.110683	1.01682	1.21321	0.66294276
WNT2	Inverse variance weighted	2	-0.150662	0.064722	0.019922	0.860138	0.757662	0.976475	0.66294276
COQ8B	Wald ratio	1	-0.149406	0.064539	0.020615	0.861219	0.758886	0.977351	0.6682724
IMPG1	Wald ratio	1	-0.142509	0.061549	0.020592	0.86718	0.768631	0.978364	0.6682724
KAT2B	Inverse variance weighted	2	-0.268711	0.116442	0.021017	0.764364	0.608392	0.960324	0.67504287
MUC1	Wald ratio	1	-0.275147	0.120017	0.021872	0.75946	0.600268	0.960871	0.67862395
ATP4B	Wald ratio	1	0.1965995	0.085419	0.021358	1.217256	1.029608	1.439104	0.67862395
CACNB4	Wald ratio	1	0.5724086	0.249738	0.021904	1.772531	1.086457	2.891847	0.67862395
CARTPT	Wald ratio	1	-0.239191	0.104152	0.021644	0.787264	0.641895	0.965555	0.67862395
IGLV5-45	Wald ratio	1	0.3876649	0.170176	0.022725	1.473536	1.055612	2.056919	0.69182727
GSTM1	Inverse variance weighted	3	-0.050357	0.022088	0.022615	0.95089	0.910602	0.99296	0.69182727
IAPP	Wald ratio	1	0.1525845	0.067116	0.023	1.164841	1.02126	1.328609	0.69415862
ALDH18A1	Wald ratio	1	0.2892611	0.127884	0.023703	1.33544	1.039364	1.715858	0.70326451
CSNK1G3	Inverse variance weighted	3	-0.199253	0.088062	0.023657	0.819343	0.689455	0.9737	0.70326451
UBA2	Wald ratio	1	0.7673727	0.340086	0.024045	2.154099	1.106061	4.195198	0.70740647
PDE8A	Wald ratio	1	0.6247271	0.280776	0.026081	1.867736	1.077244	3.2383	0.71336262
TEX14	Wald ratio	1	0.1298679	0.058158	0.025548	1.138678	1.016004	1.276163	0.71336262
CD226	Wald ratio	1	0.2480675	0.111023	0.025458	1.281546	1.030931	1.593086	0.71336262
HAPLN4	Wald ratio	1	0.3063092	0.136248	0.024565	1.358402	1.040044	1.77421	0.71336262
CTSZ	Wald ratio	1	0.2334769	0.10491	0.026048	1.262984	1.028244	1.551312	0.71336262
LIMK2	Wald ratio	1	-0.194311	0.08716	0.02579	0.823401	0.694096	0.976796	0.71336262
TRPC6	Inverse variance weighted	2	-0.158713	0.070668	0.024711	0.853242	0.742879	0.979999	0.71336262
WNT7B	Inverse variance weighted	2	0.3389235	0.151888	0.025654	1.403436	1.042085	1.890088	0.71336262

OR7E22P	Inverse variance weighted	2	-0.13958	0.062663	0.025914	0.869723	0.769204	0.983378	0.71336262
SIRT1	Wald ratio	1	0.3124443	0.140801	0.026483	1.366762	1.037148	1.80113	0.71451623
CYP26C1	Wald ratio	1	0.0813415	0.036766	0.02694	1.084741	1.009323	1.165795	0.71451623
TPO	Wald ratio	1	-0.215646	0.097351	0.02675	0.806021	0.666008	0.975468	0.71451623
RIPK4	Wald ratio	1	0.2262161	0.102162	0.026809	1.253847	1.026319	1.531816	0.71451623
OR8B8	Wald ratio	1	-0.196514	0.089192	0.027577	0.82159	0.689816	0.978537	0.72050031
PDE11A	Wald ratio	1	-0.124387	0.056449	0.027557	0.883038	0.790549	0.986347	0.72050031
SPSB1	Wald ratio	1	-0.602527	0.275024	0.028465	0.547427	0.319316	0.938494	0.72340717
LGALS9	Wald ratio	1	0.3416976	0.156246	0.028749	1.407335	1.036091	1.9116	0.72340717
CRTAP	Wald ratio	1	-0.296299	0.135224	0.028439	0.743565	0.570445	0.969223	0.72340717
IL6	Wald ratio	1	0.225783	0.103358	0.028928	1.253304	1.023472	1.534747	0.72340717
CYP7A1	Wald ratio	1	0.180303	0.082106	0.028094	1.19758	1.019563	1.406679	0.72340717
SULF1	Wald ratio	1	-0.189485	0.086601	0.028667	0.827385	0.698219	0.980446	0.72340717
GIPR	Inverse variance weighted	2	-0.167431	0.076803	0.029256	0.845835	0.727629	0.983244	0.72642807
ALOX12	Wald ratio	1	0.2440968	0.112202	0.029592	1.276468	1.024475	1.590443	0.72958632
ADAM23	Wald ratio	1	-0.33712	0.155594	0.03026	0.713823	0.526195	0.968355	0.74084783
DAO	Wald ratio	1	0.2892525	0.133897	0.030752	1.335429	1.027178	1.736185	0.74743175
IFNA17	Wald ratio	1	0.1676189	0.077686	0.030956	1.182486	1.015472	1.376968	0.74743175
PDF	Wald ratio	1	0.0884057	0.041101	0.031481	1.092431	1.007879	1.184077	0.74975386
FLRT2	Inverse variance weighted	2	-0.452466	0.210275	0.031415	0.636058	0.421218	0.960476	0.74975386
OR2T5	Wald ratio	1	-0.129617	0.061089	0.033855	0.878431	0.779306	0.990165	0.7597836
OR2T29	Wald ratio	1	-0.158685	0.074788	0.033855	0.853265	0.736924	0.987973	0.7597836
COL9A2	Wald ratio	1	-0.581573	0.272879	0.033069	0.559018	0.327452	0.954345	0.7597836
SLC47A2	Wald ratio	1	0.2422096	0.114045	0.033686	1.274061	1.018857	1.593189	0.7597836
OR7E91P	Wald ratio	1	0.1378245	0.064446	0.032467	1.147774	1.011577	1.302308	0.7597836
CACNA2D3	Wald ratio	1	-0.278253	0.130705	0.033265	0.757105	0.586	0.97817	0.7597836
ARSJ	Wald ratio	1	0.4079468	0.191886	0.033505	1.503727	1.032363	2.190311	0.7597836
STYK1	Inverse variance weighted	2	0.2169966	0.101798	0.033036	1.24234	1.017627	1.516674	0.7597836
ADAMTS2	Inverse variance weighted	2	-0.147029	0.069179	0.033558	0.863269	0.753806	0.988627	0.7597836
FFAR4	Wald ratio	1	0.2430439	0.114917	0.034435	1.275125	1.017965	1.597248	0.76260011
GPHA2	Wald ratio	1	-0.236113	0.111763	0.034634	0.789692	0.634341	0.983088	0.76260011
VIPR1	Wald ratio	1	0.2378952	0.112339	0.034205	1.268576	1.017868	1.581036	0.76260011
RPS6KA4	Wald ratio	1	-0.383356	0.181871	0.035045	0.68157	0.477198	0.97347	0.76681878
CA6	Wald ratio	1	0.2035238	0.097305	0.036474	1.225714	1.012888	1.483259	0.77363688
CTSC	Wald ratio	1	0.2191278	0.104882	0.036682	1.24499	1.013652	1.529125	0.77363688
GPR75	Wald ratio	1	0.4768581	0.226984	0.035655	1.611005	1.032485	2.513679	0.77363688
NUCB2	Inverse variance weighted	2	-0.166256	0.079478	0.036453	0.84683	0.724674	0.989577	0.77363688
OXTR	Inverse variance weighted	2	-0.056607	0.02698	0.035892	0.944965	0.896293	0.99628	0.77363688
MAP3K5	Inverse variance weighted	2	0.3369266	0.16108	0.036468	1.400636	1.021435	1.920613	0.77363688
ITM2B	Wald ratio	1	0.5903889	0.282967	0.036941	1.80469	1.03642	3.142457	0.77442575
INHBB	Wald ratio	1	0.4084334	0.196001	0.037175	1.504459	1.024568	2.209123	0.77470922
NRG3	Wald ratio	1	-0.435895	0.209481	0.037449	0.646686	0.428923	0.975004	0.77578653
BCAN	Wald ratio	1	-0.326234	0.158457	0.039511	0.721636	0.528978	0.984463	0.77873482
KLHL12	Wald ratio	1	-0.452999	0.219125	0.038705	0.635719	0.413754	0.97676	0.77873482
PIK3C2A	Wald ratio	1	1.3921815	0.670909	0.03798	4.023618	1.080258	14.9867	0.77873482
IRAK3	Wald ratio	1	0.2888595	0.140303	0.039511	1.334904	1.013962	1.757432	0.77873482
MFSD11	Wald ratio	1	0.3712816	0.180311	0.039483	1.449591	1.018032	2.064095	0.77873482
PTPN2	Wald ratio	1	-0.289369	0.139884	0.038581	0.748736	0.569189	0.98492	0.77873482
EPHX2	Wald ratio	1	0.1195815	0.058106	0.039593	1.127025	1.005709	1.262975	0.77873482
ADAM32	Wald ratio	1	-0.419736	0.203244	0.038906	0.657221	0.441272	0.97885	0.77873482
KAZALD1	Inverse variance weighted	2	0.2549731	0.123562	0.039062	1.290427	1.012874	1.644036	0.77873482
KCNJ10	Wald ratio	1	-0.412517	0.201178	0.040315	0.661982	0.446272	0.981957	0.78007787
PIK3CD	Wald ratio	1	0.1069857	0.052237	0.040552	1.112918	1.004611	1.232902	0.78007787
DHODH	Wald ratio	1	0.0950557	0.04638	0.040413	1.09972	1.00416	1.204374	0.78007787
PROC	Wald ratio	1	0.2864116	0.13947	0.040017	1.33164	1.013136	1.750275	0.78007787
BRCA1	Wald ratio	1	-0.273757	0.134104	0.041213	0.760517	0.584732	0.989146	0.78462365
CDKL3	Wald ratio	1	-0.46307	0.227119	0.041461	0.629349	0.40324	0.982242	0.78462365
LEP	Wald ratio	1	0.17683	0.086664	0.04131	1.193428	1.006992	1.414381	0.78462365
IFNA8	Wald ratio	1	0.1861887	0.091705	0.042326	1.20465	1.006468	1.441855	0.79347489
WNT2B	Inverse variance weighted	2	0.0352169	0.01735	0.042382	1.035844	1.001211	1.071676	0.79347489
GRM4	Wald ratio	1	0.2158917	0.106676	0.04299	1.240968	1.00683	1.529554	0.79633401
TRAV8-4	Inverse variance weighted	3	0.280182	0.138292	0.042762	1.323371	1.009172	1.735392	0.79633401
NCAM1	Wald ratio	1	0.6359613	0.316459	0.044472	1.888837	1.015825	3.512126	0.8035215
AVPR1A	Wald ratio	1	-0.173462	0.086731	0.0455	0.840749	0.709316	0.996537	0.8035215
LIPC	Wald ratio	1	0.2440513	0.121141	0.043947	1.27641	1.006636	1.618481	0.8035215

NFAT5	Wald ratio	1	0.5691706	0.285495	0.046192	1.766801	1.009648	3.091758	0.8035215
IL17C	Wald ratio	1	-0.207503	0.103752	0.0455	0.81261	0.663082	0.995859	0.8035215
CYP2G1P	Wald ratio	1	0.2436985	0.12096	0.043935	1.27596	1.006639	1.617335	0.8035215
NAPSB	Wald ratio	1	-0.034846	0.0175	0.046464	0.965754	0.93319	0.999455	0.8035215
BCHE	Wald ratio	1	0.1174569	0.058959	0.04635	1.124633	1.0019	1.262402	0.8035215
TINAG	Wald ratio	1	0.1476292	0.073815	0.0455	1.159083	1.002957	1.339513	0.8035215
ZC3HAV1	Wald ratio	1	0.2578475	0.12945	0.046385	1.294141	1.004134	1.667907	0.8035215
ANXA1	Wald ratio	1	-0.376674	0.189284	0.046591	0.68614	0.473468	0.994338	0.8035215
SLC15A5	Inverse variance weighted	2	-0.137272	0.068368	0.044661	0.871733	0.762407	0.996735	0.8035215
NME9	Inverse variance weighted	2	-0.076997	0.038611	0.046135	0.925893	0.858409	0.998682	0.8035215
DHFR2	Inverse variance weighted	2	0.2780851	0.138267	0.044302	1.320599	1.007107	1.731673	0.8035215
BMPR1A	Wald ratio	1	-0.215167	0.108589	0.047537	0.806406	0.65181	0.99767	0.80554358
POSTN	Inverse variance weighted	2	-0.124058	0.062634	0.047629	0.883329	0.781281	0.998706	0.80554358
ADGRG1	Inverse variance weighted	2	0.4462463	0.224968	0.047301	1.562436	1.005323	2.428281	0.80554358
SMN2	Inverse variance weighted	2	-0.131267	0.066095	0.04703	0.876984	0.770425	0.998281	0.80554358
F2	Wald ratio	1	-0.150214	0.076185	0.048644	0.860524	0.741162	0.999109	0.80709792
CLCN3	Wald ratio	1	-0.257472	0.130716	0.048873	0.773004	0.598292	0.998733	0.80709792
SMR3A	Wald ratio	1	-0.403836	0.204618	0.048426	0.667754	0.447139	0.99722	0.80709792
GRM1	Wald ratio	1	0.5325569	0.270293	0.048804	1.703282	1.002787	2.893106	0.80709792
ADAMTSL1	Inverse variance weighted	2	0.210709	0.106634	0.048155	1.234553	1.001707	1.521524	0.80709792
MARK3	Wald ratio	1	-0.293243	0.149554	0.049904	0.745841	0.556344	0.999883	0.80976213
TNFSF12	Wald ratio	1	-0.438637	0.223973	0.050178	0.644915	0.41577	1.000349	0.80976213
GAD1	Wald ratio	1	0.3241679	0.165533	0.050191	1.382879	0.999724	1.912883	0.80976213
COL5A3	Inverse variance weighted	2	-0.217046	0.110732	0.049985	0.804893	0.647859	0.999989	0.80976213
CGREF1	Inverse variance weighted	3	-0.234984	0.11981	0.049843	0.790584	0.625121	0.999843	0.80976213
IGLV3-25	Inverse variance weighted	4	-0.16729	0.085632	0.050749	0.845954	0.715246	1.000548	0.81500833
SLC4A10	Wald ratio	1	0.4477425	0.229553	0.051117	1.564776	0.99782	2.453871	0.81539975
LGR5	Inverse variance weighted	2	-0.352807	0.180976	0.051239	0.702713	0.492865	1.001907	0.81539975
ANGPTL7	Wald ratio	1	0.0831415	0.043763	0.057461	1.086696	0.997369	1.184023	0.81776133
CD58	Wald ratio	1	-0.361411	0.18952	0.056524	0.696693	0.480528	1.0101	0.81776133
NODAL	Wald ratio	1	0.096193	0.050263	0.055647	1.100972	0.99768	1.214957	0.81776133
PLAU	Wald ratio	1	-0.274911	0.144632	0.057333	0.75964	0.572129	1.008604	0.81776133
OR52E2	Wald ratio	1	-0.144635	0.074811	0.053195	0.865338	0.747317	1.001997	0.81776133
HPX	Wald ratio	1	-0.306169	0.158712	0.053721	0.736262	0.539428	1.00492	0.81776133
COL2A1	Wald ratio	1	-0.112383	0.058404	0.054324	0.893702	0.797036	1.002091	0.81776133
ALOX5AP	Wald ratio	1	-0.253886	0.133624	0.057433	0.77578	0.597029	1.00805	0.81776133
FLVCR2	Wald ratio	1	0.1847172	0.095976	0.054277	1.202878	0.99661	1.451837	0.81776133
TDP1	Wald ratio	1	0.4209347	0.21679	0.052177	1.523385	0.996033	2.329943	0.81776133
TUBB3	Wald ratio	1	0.6764004	0.35541	0.057021	1.966785	0.979999	3.947193	0.81776133
CD300A	Wald ratio	1	0.3185606	0.164059	0.052168	1.375147	0.99701	1.8967	0.81776133
NLGN2	Wald ratio	1	-0.602163	0.311202	0.052995	0.547626	0.297566	1.007823	0.81776133
CYP4F2	Wald ratio	1	-0.086484	0.045063	0.05496	0.91715	0.839619	1.001841	0.81776133
TGFB1	Wald ratio	1	-0.365059	0.191477	0.05658	0.694156	0.476945	1.010289	0.81776133
CD177	Wald ratio	1	-0.106876	0.055739	0.055183	0.898637	0.805635	1.002376	0.81776133
CD93	Wald ratio	1	-0.251694	0.131738	0.05606	0.777483	0.600556	1.006533	0.81776133
LARS2	Wald ratio	1	0.0406499	0.021071	0.053706	1.041487	0.999351	1.0854	0.81776133
PDE4D	Wald ratio	1	-0.279567	0.144152	0.052453	0.756111	0.570008	1.002975	0.81776133
ENPP3	Wald ratio	1	0.2021269	0.104402	0.052862	1.224003	0.997502	1.501935	0.81776133
PTK2	Wald ratio	1	-0.485335	0.25544	0.057433	0.615491	0.373067	1.015444	0.81776133
INPP5E	Wald ratio	1	0.1646059	0.085781	0.054995	1.178928	0.996482	1.394779	0.81776133
SLC16A9	Inverse variance weighted	2	0.3011483	0.157078	0.055213	1.35141	0.993299	1.83863	0.81776133
LARS1	Inverse variance weighted	2	-0.246291	0.128678	0.05562	0.781695	0.607441	1.005936	0.81776133
NPR3	Inverse variance weighted	2	-0.222523	0.114611	0.052192	0.800497	0.639441	1.002117	0.81776133
COL12A1	Inverse variance weighted	3	-0.144266	0.075798	0.057003	0.865657	0.746149	1.004308	0.81776133
MAP2K6	Wald ratio	1	0.3311223	0.175197	0.058758	1.39253	0.987811	1.963068	0.83284056
ADAMTS16	Wald ratio	1	0.2041275	0.108118	0.059026	1.226455	0.992246	1.515946	0.83326553
CAPZA1	Wald ratio	1	-0.411051	0.22384	0.066304	0.662953	0.42751	1.028061	0.83815846
NTRK1	Wald ratio	1	-0.188954	0.103307	0.067391	0.827824	0.676086	1.013619	0.83815846
CD1E	Wald ratio	1	-0.224912	0.123269	0.068067	0.798587	0.627182	1.016836	0.83815846
TNR	Wald ratio	1	0.2425118	0.137423	0.077613	1.274446	0.973519	1.668394	0.83815846
REN	Wald ratio	1	0.178884	0.102877	0.082069	1.195882	0.977501	1.463051	0.83815846
WNT4	Wald ratio	1	-0.136925	0.0741	0.064628	0.872036	0.754152	1.008347	0.83815846
CDC42	Wald ratio	1	0.4369147	0.249976	0.080494	1.547924	0.948344	2.526583	0.83815846
EPHB2	Wald ratio	1	0.3292296	0.177917	0.064246	1.389897	0.980701	1.969829	0.83815846
ADGRL4	Wald ratio	1	-0.227726	0.128878	0.07723	0.796342	0.618581	1.025187	0.83815846

TMPRSS4	Wald ratio	1	-0.158484	0.089385	0.07622	0.853437	0.716285	1.016851	0.83815846
ST3GAL4	Wald ratio	1	-0.180329	0.102321	0.078006	0.834995	0.683261	1.020427	0.83815846
NR1H3	Wald ratio	1	0.2162835	0.120025	0.071549	1.241454	0.981212	1.570719	0.83815846
OR4C12	Wald ratio	1	0.2721628	0.155912	0.080878	1.312801	0.967127	1.782026	0.83815846
OR56A3	Wald ratio	1	0.1484961	0.083079	0.073873	1.160088	0.985763	1.365242	0.83815846
MRGPRD	Wald ratio	1	-0.150012	0.08534	0.078779	0.860698	0.728128	1.017404	0.83815846
PLBD2	Wald ratio	1	-0.463927	0.261287	0.075808	0.628809	0.376796	1.049376	0.83815846
WNT5B	Wald ratio	1	0.278495	0.155897	0.074033	1.32114	0.9733	1.793291	0.83815846
BCAT1	Wald ratio	1	0.1380394	0.07517	0.066304	1.148021	0.990749	1.330258	0.83815846
OR10AD1	Wald ratio	1	0.1686556	0.091165	0.064314	1.183712	0.990022	1.415297	0.83815846
GAPDH	Wald ratio	1	-0.383952	0.216443	0.076078	0.681164	0.445668	1.041099	0.83815846
GLIPR1L1	Wald ratio	1	0.2222339	0.120877	0.065987	1.248863	0.985422	1.582733	0.83815846
DUSP6	Wald ratio	1	-0.311701	0.18093	0.08493	0.732201	0.513594	1.043856	0.83815846
LTB4R2	Wald ratio	1	0.152215	0.085014	0.073377	1.164411	0.985692	1.375534	0.83815846
PTPN21	Wald ratio	1	-0.249754	0.139415	0.073222	0.778992	0.592735	1.023778	0.83815846
OTOA	Wald ratio	1	-0.17667	0.099685	0.076349	0.838057	0.689318	1.018889	0.83815846
TAOK2	Wald ratio	1	0.5614104	0.325027	0.084119	1.753143	0.927147	3.31502	0.83815846
CNGB1	Wald ratio	1	0.1819607	0.102644	0.076274	1.199567	0.980961	1.466889	0.83815846
NGFR	Wald ratio	1	-0.182575	0.098711	0.064372	0.833122	0.686568	1.010958	0.83815846
OR4D1	Wald ratio	1	0.1977452	0.107184	0.065051	1.218652	0.98774	1.503546	0.83815846
CLEC10A	Wald ratio	1	0.1367452	0.079064	0.083709	1.146536	0.981946	1.338714	0.83815846
SLC16A5	Wald ratio	1	0.1654168	0.094717	0.080735	1.179885	0.979975	1.420575	0.83815846
CDK3	Wald ratio	1	0.1496592	0.086726	0.084408	1.161438	0.979882	1.376634	0.83815846
MC4R	Wald ratio	1	-0.293355	0.158537	0.064257	0.745757	0.546573	1.017528	0.83815846
TXNDC2	Wald ratio	1	0.2172232	0.121228	0.073156	1.242621	0.979822	1.575906	0.83815846
CILP2	Wald ratio	1	0.2837429	0.157496	0.07161	1.328091	0.975359	1.808387	0.83815846
ADAMTS10	Wald ratio	1	0.5561036	0.297851	0.061894	1.743864	0.972695	3.12643	0.83815846
FKBP7	Wald ratio	1	-0.20582	0.111824	0.065683	0.813979	0.653773	1.013444	0.83815846
SLC5A6	Wald ratio	1	0.1899385	0.108038	0.078737	1.209175	0.978419	1.494354	0.83815846
ATRAID	Wald ratio	1	-0.193153	0.109867	0.078737	0.824356	0.664652	1.022434	0.83815846
RTN4	Wald ratio	1	0.3637825	0.200918	0.070202	1.438761	0.97043	2.13311	0.83815846
VAMP8	Wald ratio	1	0.140634	0.07742	0.069292	1.151003	0.988953	1.339607	0.83815846
SIRPG	Wald ratio	1	-0.15951	0.090389	0.077613	0.852561	0.714142	1.017809	0.83815846
SSTR4	Wald ratio	1	-0.226218	0.129727	0.081194	0.797544	0.618485	1.028444	0.83815846
ATRN	Wald ratio	1	0.3605769	0.204506	0.077874	1.434157	0.960544	2.141292	0.83815846
MYT1	Wald ratio	1	-0.383462	0.210981	0.069138	0.681498	0.450686	1.030517	0.83815846
TIMP3	Wald ratio	1	0.3305771	0.191752	0.084711	1.391771	0.955752	2.026705	0.83815846
POGLUT1	Wald ratio	1	0.0532506	0.030105	0.07692	1.054694	0.994262	1.118799	0.83815846
ALDH1L1	Wald ratio	1	0.2486562	0.138955	0.073539	1.282301	0.976583	1.683724	0.83815846
ATR	Wald ratio	1	0.2750225	0.15915	0.083976	1.31656	0.963761	1.798507	0.83815846
P2RY12	Wald ratio	1	0.2461229	0.141624	0.082235	1.279057	0.96903	1.688272	0.83815846
ACKR2	Wald ratio	1	-0.086974	0.050327	0.08396	0.916701	0.830593	1.011736	0.83815846
DRD5	Wald ratio	1	-0.3003	0.166373	0.071078	0.740596	0.534517	1.026127	0.83815846
ADH1A	Wald ratio	1	0.1552758	0.086199	0.071645	1.16798	0.986419	1.382959	0.83815846
IL2	Wald ratio	1	0.2346154	0.13141	0.074202	1.264422	0.977313	1.635878	0.83815846
AMBN	Wald ratio	1	-0.259274	0.140074	0.064172	0.771612	0.586362	1.015388	0.83815846
ERAP1	Wald ratio	1	0.3060632	0.163123	0.060618	1.358068	0.986435	1.869712	0.83815846
GMPR	Wald ratio	1	0.1929213	0.111831	0.084506	1.212787	0.974075	1.51	0.83815846
KCNK17	Wald ratio	1	0.1271508	0.072115	0.077874	1.135588	0.985905	1.307997	0.83815846
RNGTT	Wald ratio	1	-0.575455	0.308427	0.062073	0.562449	0.307287	1.029489	0.83815846
LAMB4	Wald ratio	1	-0.208241	0.118844	0.079737	0.812012	0.64328	1.025002	0.83815846
LRRN3	Wald ratio	1	-0.713759	0.406031	0.078765	0.4898	0.221003	1.085522	0.83815846
CAPZA2	Wald ratio	1	-0.245462	0.139217	0.077874	0.782343	0.595516	1.027783	0.83815846
AKR1D1	Wald ratio	1	0.2205554	0.127761	0.084291	1.246769	0.970586	1.601541	0.83815846
CHST12	Wald ratio	1	-0.372565	0.211719	0.078456	0.688965	0.454966	1.043315	0.83815846
NME8	Wald ratio	1	0.1578002	0.091491	0.084569	1.170932	0.978709	1.400909	0.83815846
SFRP1	Wald ratio	1	-0.123385	0.069162	0.074424	0.883923	0.771867	1.012247	0.83815846
MOS	Wald ratio	1	0.183077	0.098508	0.063099	1.200907	0.99005	1.456671	0.83815846
IMPA1	Wald ratio	1	0.1966112	0.106704	0.065389	1.217271	0.98755	1.500428	0.83815846
WDR5	Wald ratio	1	0.2744194	0.154935	0.07653	1.315767	0.97117	1.782635	0.83815846
AGPAT2	Wald ratio	1	0.4463638	0.24677	0.070478	1.56262	0.963381	2.534594	0.83815846
LCN8	Wald ratio	1	-0.217771	0.119211	0.067734	0.80431	0.636721	1.016009	0.83815846
AGTPBP1	Wald ratio	1	0.6751734	0.381916	0.077085	1.964374	0.929245	4.152578	0.83815846
PTPDC1	Wald ratio	1	-0.583942	0.318015	0.066327	0.557696	0.299018	1.040153	0.83815846
GSTM3	Inverse variance weighted	3	-0.027525	0.015622	0.078071	0.97285	0.943514	1.003098	0.83815846

GSTM2	Inverse variance weighted	2	0.2743475	0.153344	0.073599	1.315672	0.974134	1.776956	0.83815846
GPR161	Inverse variance weighted	4	0.0557262	0.030814	0.070531	1.057308	0.995342	1.123132	0.83815846
TAF1A	Inverse variance weighted	2	0.139651	0.079292	0.0782	1.149872	0.984362	1.343211	0.83815846
CD52	Inverse variance weighted	2	-0.060014	0.034584	0.082683	0.941751	0.88003	1.0078	0.83815846
PTGFR	Inverse variance weighted	2	-0.197855	0.112909	0.079716	0.820489	0.657601	1.023724	0.83815846
SLC29A3	Inverse variance weighted	2	0.1477724	0.078649	0.06026	1.159249	0.993641	1.352459	0.83815846
HTR3A	Inverse variance weighted	3	-0.115296	0.066266	0.081879	0.891102	0.782565	1.014693	0.83815846
TPCN1	Inverse variance weighted	2	-0.334615	0.179891	0.062872	0.715614	0.502981	1.018135	0.83815846
PNP	Inverse variance weighted	2	-0.1464	0.081865	0.073723	0.863812	0.735757	1.014153	0.83815846
GABRB3	Inverse variance weighted	2	-0.351461	0.194714	0.071073	0.70366	0.480418	1.030638	0.83815846
MPI	Inverse variance weighted	3	-0.083913	0.046231	0.069512	0.919512	0.839856	1.006722	0.83815846
CHRNB1	Inverse variance weighted	2	0.1042745	0.056477	0.064844	1.109905	0.993601	1.239823	0.83815846
CLUL1	Inverse variance weighted	2	0.032613	0.018752	0.082006	1.033151	0.995867	1.07183	0.83815846
S1PR5	Inverse variance weighted	2	-0.310663	0.17552	0.076734	0.732961	0.519607	1.033918	0.83815846
SLC19A3	Inverse variance weighted	2	-0.12423	0.070546	0.078242	0.883177	0.769126	1.014139	0.83815846
LTBP1	Inverse variance weighted	3	-0.111807	0.064912	0.084989	0.894216	0.787387	1.01554	0.83815846
MORC3	Inverse variance weighted	2	-0.351527	0.187382	0.060656	0.703613	0.487338	1.015866	0.83815846
RFPL3	Inverse variance weighted	2	0.1287181	0.074473	0.083919	1.137369	0.982899	1.316116	0.83815846
PLCXD2	Inverse variance weighted	2	-0.224889	0.124935	0.071852	0.798605	0.625152	1.020184	0.83815846
ATP2C1	Inverse variance weighted	2	0.1537522	0.088507	0.082355	1.166202	0.980472	1.387114	0.83815846
HYAL3	Inverse variance weighted	2	-0.07353	0.042449	0.083236	0.929108	0.854935	1.009716	0.83815846
CAMK2D	Inverse variance weighted	2	0.3313992	0.192294	0.084817	1.392916	0.955522	2.030528	0.83815846
NPY1R	Inverse variance weighted	2	0.3630494	0.207351	0.079964	1.437707	0.957568	2.158594	0.83815846
CASP3	Inverse variance weighted	2	0.2451045	0.135999	0.071505	1.277755	0.978775	1.668062	0.83815846
LRPAP1	Inverse variance weighted	3	0.0634757	0.0345	0.065784	1.065534	0.995865	1.140076	0.83815846
CNGA1	Inverse variance weighted	3	0.0473238	0.02626	0.071521	1.048461	0.995864	1.103837	0.83815846
HTRA3	Inverse variance weighted	3	-0.135791	0.074491	0.068316	0.873025	0.75443	1.010264	0.83815846
MAP3K1	Inverse variance weighted	2	-0.306507	0.173212	0.076802	0.736014	0.524137	1.033539	0.83815846
ALDH5A1	Inverse variance weighted	2	-0.102976	0.056757	0.069626	0.902149	0.807172	1.008302	0.83815846
SDR16C5	Inverse variance weighted	3	0.1187074	0.065078	0.06814	1.12604	0.991194	1.279232	0.83815846
PSMB7	Inverse variance weighted	2	0.2352907	0.125428	0.060668	1.265277	0.989508	1.6179	0.83815846
LCN9	Inverse variance weighted	2	0.1123973	0.062933	0.074102	1.118957	0.989109	1.265853	0.83815846
PMPCA	Inverse variance weighted	2	-0.329258	0.183716	0.073099	0.719457	0.501907	1.031305	0.83815846
SPP1	Wald ratio	1	-0.532303	0.309948	0.085908	0.587251	0.319882	1.078096	0.84483948
PHKG2	Wald ratio	1	0.3892216	0.227046	0.086476	1.475832	0.945739	2.303044	0.8456799
ABCB6	Inverse variance weighted	2	-0.24204	0.141127	0.086336	0.785025	0.595324	1.035174	0.8456799
KITLG	Wald ratio	1	-0.472585	0.276591	0.087523	0.623389	0.36251	1.072007	0.84594544
ATP2C2	Wald ratio	1	0.1489625	0.087391	0.088279	1.160629	0.977923	1.377471	0.84594544
TSSK6	Wald ratio	1	0.1508766	0.088445	0.08803	1.162853	0.977775	1.382964	0.84594544
COL3A1	Wald ratio	1	-0.224248	0.131209	0.087434	0.799117	0.617907	1.033469	0.84594544
PLCB4	Wald ratio	1	-0.353812	0.207672	0.088436	0.702007	0.46727	1.054668	0.84594544
AURKA	Wald ratio	1	0.2072914	0.121553	0.088128	1.230341	0.969521	1.561326	0.84594544
ACKR1	Inverse variance weighted	2	0.1826823	0.107159	0.088235	1.200433	0.973022	1.480993	0.84594544
GALNS	Inverse variance weighted	2	-0.150529	0.088089	0.087483	0.860253	0.72384	1.022372	0.84594544
RHCE	Wald ratio	1	-0.039746	0.023367	0.088955	0.961034	0.918011	1.006072	0.84858967
HHATL	Inverse variance weighted	2	-0.07603	0.044744	0.089277	0.926788	0.848973	1.011736	0.84934295
SLC46A3	Wald ratio	1	0.1161138	0.068526	0.090181	1.123124	0.981967	1.284571	0.85021334
SCN1A	Wald ratio	1	0.5524685	0.325733	0.089871	1.737537	0.917624	3.290057	0.85021334
ST6GAL1	Wald ratio	1	0.1145835	0.067688	0.090492	1.121406	0.982077	1.280503	0.85021334
NELL1	Inverse variance weighted	3	0.0935398	0.055273	0.090583	1.098054	0.985314	1.223694	0.85021334
NTN4	Inverse variance weighted	2	0.0544897	0.032125	0.089853	1.056002	0.99156	1.124631	0.85021334
HTR1E	Wald ratio	1	0.2393232	0.141611	0.091028	1.270389	0.962487	1.67679	0.85210928
TAOK3	Inverse variance weighted	2	-0.268812	0.159191	0.091294	0.764287	0.559436	1.044149	0.85231736
OR8G2P	Wald ratio	1	-0.306798	0.182519	0.09278	0.735799	0.514513	1.052259	0.85557948
JAM3	Wald ratio	1	0.4721792	0.280609	0.092435	1.603485	0.925135	2.779229	0.85557948
SLC29A2	Wald ratio	1	-0.402861	0.239479	0.092522	0.668405	0.418014	1.068779	0.85557948
DKKL1	Wald ratio	1	-0.120149	0.071338	0.092141	0.886789	0.771073	1.019869	0.85557948
EDEM2	Wald ratio	1	-0.095349	0.056824	0.093354	0.909056	0.813244	1.016155	0.85557948
SULT2B1	Inverse variance weighted	4	0.0733931	0.043683	0.092931	1.076154	0.987849	1.172352	0.85557948
FCN2	Inverse variance weighted	2	-0.10504	0.062594	0.093325	0.900288	0.796343	1.017801	0.85557948
CTSA	Wald ratio	1	0.4877743	0.290953	0.093646	1.628687	0.920817	2.880726	0.85601783
AOAH	Wald ratio	1	-0.269978	0.161286	0.094148	0.763396	0.556495	1.047223	0.85836215
AKR7A3	Wald ratio	1	0.1141343	0.068481	0.095581	1.120903	0.980113	1.281916	0.85928795
PSEN2	Wald ratio	1	-0.173905	0.104343	0.095581	0.840376	0.684944	1.031081	0.85928795
CDKL1	Wald ratio	1	-0.21346	0.128454	0.09656	0.807784	0.62799	1.039053	0.85928795

CAMKV	Wald ratio	1	0.4005947	0.240848	0.096259	1.492712	0.931026	2.393263	0.85928795
OR1F1	Inverse variance weighted	2	0.1494512	0.089974	0.096704	1.161197	0.973461	1.385139	0.85928795
OLFM2	Inverse variance weighted	3	0.1868029	0.111764	0.094642	1.20539	0.96826	1.500594	0.85928795
TFF2	Inverse variance weighted	4	0.1842455	0.110911	0.096674	1.202311	0.967402	1.494261	0.85928795
RNF123	Inverse variance weighted	2	0.1911607	0.114949	0.096311	1.210654	0.966437	1.516584	0.85928795
SOD3	Inverse variance weighted	2	-0.223815	0.134002	0.094874	0.799463	0.6148	1.039594	0.85928795
CLPS	Inverse variance weighted	2	-0.080693	0.048571	0.096648	0.922477	0.838708	1.014613	0.85928795
HAO2	Wald ratio	1	-0.121053	0.074237	0.102968	0.885987	0.766012	1.024753	0.86377026
KCNJ9	Wald ratio	1	0.1701622	0.103878	0.101402	1.185497	0.967114	1.453193	0.86377026
MAPKAPK2	Wald ratio	1	0.3798978	0.229706	0.098159	1.462135	0.932091	2.293596	0.86377026
CYP26A1	Wald ratio	1	0.3223669	0.197771	0.103101	1.380391	0.93682	2.033988	0.86377026
TLL2	Wald ratio	1	-0.163007	0.100104	0.103445	0.849585	0.698227	1.033754	0.86377026
VAMP1	Wald ratio	1	0.1402248	0.08508	0.099322	1.150532	0.973816	1.359317	0.86377026
OR1P1	Wald ratio	1	0.1845233	0.111994	0.099431	1.202645	0.96562	1.497851	0.86377026
SLC25A19	Wald ratio	1	-0.221482	0.136211	0.103946	0.801331	0.613573	1.046543	0.86377026
KIR2DL4	Wald ratio	1	0.1714365	0.105636	0.10461	1.187009	0.965017	1.460067	0.86377026
MAP2K7	Wald ratio	1	-0.29818	0.182018	0.101382	0.742168	0.519476	1.060324	0.86377026
ITGB6	Wald ratio	1	-0.049995	0.030217	0.098017	0.951234	0.896532	1.009273	0.86377026
PTPN1	Wald ratio	1	0.3470257	0.210694	0.099546	1.414853	0.936192	2.138247	0.86377026
KALRN	Wald ratio	1	0.4234576	0.258547	0.101456	1.527233	0.920081	2.535039	0.86377026
CP	Wald ratio	1	0.1557739	0.094942	0.100856	1.168562	0.970141	1.407565	0.86377026
CXCL9	Wald ratio	1	0.1410811	0.08672	0.103766	1.151518	0.971524	1.36486	0.86377026
IL31RA	Wald ratio	1	0.1426102	0.087295	0.10233	1.15328	0.971914	1.36849	0.86377026
NQO2	Wald ratio	1	0.0936381	0.056515	0.097547	1.098162	0.983014	1.226799	0.86377026
RARS2	Wald ratio	1	0.2245621	0.136733	0.10052	1.251774	0.957495	1.636499	0.86377026
PRPS1L1	Wald ratio	1	0.1512345	0.092604	0.10244	1.163269	0.970185	1.394782	0.86377026
TRGV3	Wald ratio	1	-0.091084	0.055527	0.100932	0.912941	0.818799	1.017908	0.86377026
CPA6	Wald ratio	1	-0.071969	0.04362	0.098962	0.93056	0.854308	1.013618	0.86377026
SPINK4	Wald ratio	1	0.2046017	0.124357	0.099911	1.227036	0.961619	1.565712	0.86377026
OR2AK2	Inverse variance weighted	2	-0.132306	0.080515	0.100334	0.876073	0.748177	1.025832	0.86377026
MECR	Inverse variance weighted	2	-0.120209	0.072897	0.099142	0.886735	0.768674	1.022928	0.86377026
FAAH	Inverse variance weighted	2	0.1127365	0.068381	0.099219	1.119337	0.978935	1.279876	0.86377026
CD6	Inverse variance weighted	2	0.0549951	0.03377	0.103417	1.056535	0.988868	1.128833	0.86377026
MAP4K2	Inverse variance weighted	2	-0.267628	0.164741	0.104261	0.765193	0.554039	1.05682	0.86377026
HPR	Inverse variance weighted	4	0.02332	0.014317	0.103337	1.023594	0.995271	1.052723	0.86377026
SEMA3F	Inverse variance weighted	2	0.1743253	0.107363	0.104439	1.190443	0.964538	1.469256	0.86377026
SLC25A46	Inverse variance weighted	2	-0.522995	0.320774	0.103014	0.592743	0.316095	1.111513	0.86377026
KLHL22	Inverse variance weighted	2	0.2122155	0.130862	0.104872	1.236414	0.956693	1.597922	0.86389877
LRRC4B	Wald ratio	1	0.3962575	0.244547	0.105153	1.486252	0.9203	2.400245	0.86417781
OPRL1	Inverse variance weighted	2	-0.17706	0.109366	0.105453	0.837729	0.676098	1.038001	0.86461771
LILRA4	Wald ratio	1	-0.141419	0.087628	0.106557	0.868125	0.731125	1.030796	0.8646257
SLC25A4	Wald ratio	1	-0.473975	0.293413	0.106227	0.622523	0.350265	1.106404	0.8646257
LPAR1	Wald ratio	1	0.2282917	0.141323	0.106227	1.256452	0.952465	1.657459	0.8646257
PLK5	Inverse variance weighted	2	0.141287	0.087351	0.10578	1.151755	0.970522	1.366832	0.8646257
GPR27	Inverse variance weighted	2	0.1039362	0.064429	0.106703	1.10953	0.977903	1.258873	0.8646257
AZIN1	Inverse variance weighted	2	-0.297906	0.184791	0.106936	0.742371	0.516802	1.066397	0.8646257
NEK1	Inverse variance weighted	2	-0.101982	0.063329	0.107324	0.903046	0.797633	1.022391	0.86576417
GPC5	Inverse variance weighted	2	-0.084481	0.052514	0.107672	0.918989	0.829105	1.018617	0.86657395
CD248	Wald ratio	1	0.3727199	0.232014	0.108175	1.451678	0.921246	2.28752	0.86862314
TESK1	Inverse variance weighted	2	0.4743791	0.295787	0.108761	1.607016	0.899998	2.869453	0.87133287
BLMH	Wald ratio	1	-0.116409	0.072756	0.109599	0.890111	0.771815	1.026538	0.87603799
SLC4A3	Wald ratio	1	0.7532607	0.471252	0.109948	2.123914	0.843334	5.349025	0.87683207
TNFRSF4	Wald ratio	1	0.0670056	0.043154	0.120496	1.069301	0.982576	1.163681	0.87887095
INSL5	Wald ratio	1	-0.139882	0.088549	0.114173	0.869461	0.73093	1.034248	0.87887095
DAGLA	Wald ratio	1	0.529559	0.340001	0.119347	1.698183	0.872107	3.306734	0.87887095
FKBP2	Wald ratio	1	0.4584343	0.291996	0.116415	1.581596	0.892366	2.80316	0.87887095
TNFSF13B	Wald ratio	1	0.1864888	0.117166	0.11146	1.205011	0.957762	1.516089	0.87887095
FLT1	Wald ratio	1	0.2526816	0.161477	0.117626	1.287473	0.93818	1.766811	0.87887095
MAN2C1	Wald ratio	1	0.1538948	0.098322	0.117532	1.166368	0.961928	1.414258	0.87887095
KLHL25	Wald ratio	1	-0.20772	0.13271	0.117532	0.812435	0.626359	1.053788	0.87887095
SPNS3	Wald ratio	1	-0.065165	0.041633	0.117532	0.936913	0.863496	1.016572	0.87887095
CD7	Wald ratio	1	-0.181347	0.113935	0.11146	0.834146	0.667204	1.042859	0.87887095
C5AR2	Wald ratio	1	-0.221171	0.141209	0.117287	0.801579	0.607781	1.057174	0.87887095
TUBA3E	Wald ratio	1	0.0898098	0.057597	0.118931	1.093966	0.977184	1.224705	0.87887095
CYP20A1	Wald ratio	1	-0.280497	0.179305	0.117734	0.755408	0.531562	1.073518	0.87887095

REG3A	Wald ratio	1	0.2419289	0.154884	0.118287	1.273704	0.940219	1.725472	0.87887095
BPIFB4	Wald ratio	1	-0.176804	0.112926	0.117429	0.837944	0.671569	1.045538	0.87887095
KCNK15	Wald ratio	1	0.156429	0.100561	0.119814	1.169328	0.960144	1.424085	0.87887095
CBLN4	Wald ratio	1	-0.306302	0.192576	0.111711	0.736164	0.50472	1.073739	0.87887095
KCNJ6	Wald ratio	1	0.3298494	0.20884	0.114235	1.390759	0.9236	2.094207	0.87887095
COMT	Wald ratio	1	0.2682284	0.170846	0.116415	1.307646	0.935541	1.827752	0.87887095
EWSR1	Wald ratio	1	0.4156725	0.267218	0.119814	1.515389	0.89756	2.558497	0.87887095
HDAC10	Wald ratio	1	-0.275713	0.177001	0.119306	0.759031	0.536529	1.073805	0.87887095
SLC25A20	Wald ratio	1	0.3071094	0.192886	0.111345	1.35949	0.931511	1.984102	0.87887095
HPSE	Wald ratio	1	0.1768793	0.111479	0.112588	1.193487	0.959235	1.484944	0.87887095
IBSP	Wald ratio	1	0.0375659	0.024069	0.118583	1.03828	0.990436	1.088436	0.87887095
HPGDS	Wald ratio	1	0.1329326	0.083957	0.113346	1.142173	0.968871	1.346474	0.87887095
RSPO3	Wald ratio	1	-0.156656	0.100497	0.119038	0.854998	0.702135	1.041141	0.87887095
CCR6	Wald ratio	1	0.20121	0.128043	0.116083	1.222882	0.951464	1.571725	0.87887095
SERPINE1	Wald ratio	1	0.1199372	0.076324	0.116083	1.127426	0.970778	1.309351	0.87887095
CPA1	Wald ratio	1	-0.151901	0.096793	0.116568	0.859073	0.710622	1.038537	0.87887095
CAMK2B	Wald ratio	1	-0.348623	0.223032	0.118027	0.705659	0.455771	1.092556	0.87887095
IGFBP3	Wald ratio	1	0.1514651	0.096122	0.115082	1.163538	0.96374	1.404757	0.87887095
SLC7A2	Wald ratio	1	0.2122835	0.135331	0.116735	1.236498	0.948413	1.61209	0.87887095
SEMA4D	Wald ratio	1	0.1623639	0.104445	0.120054	1.176288	0.958537	1.443507	0.87887095
OR10A5	Inverse variance weighted	2	0.1597994	0.100613	0.112229	1.173276	0.963288	1.429038	0.87887095
STAT6	Inverse variance weighted	2	0.1483412	0.093602	0.11301	1.159909	0.965491	1.393476	0.87887095
WARS1	Inverse variance weighted	2	0.2323304	0.148558	0.117839	1.261537	0.942855	1.687931	0.87887095
MAP4K5	Inverse variance weighted	2	-0.187174	0.120389	0.120006	0.8293	0.65499	1.049997	0.87887095
ANGPTL4	Inverse variance weighted	2	-0.177938	0.112522	0.113795	0.836994	0.671339	1.043525	0.87887095
RNASEH1	Inverse variance weighted	2	0.2315557	0.14656	0.114121	1.26056	0.945821	1.680032	0.87887095
PI15	Inverse variance weighted	2	0.0511004	0.032114	0.111559	1.052429	0.988227	1.120801	0.87887095
OR1J4	Inverse variance weighted	2	-0.080205	0.051633	0.120337	0.922927	0.834097	1.021218	0.87887095
SLC12A5	Wald ratio	1	0.1902411	0.122814	0.121377	1.209541	0.950779	1.538728	0.88345301
PRSS22	Wald ratio	1	0.4403713	0.285108	0.122448	1.553284	0.888305	2.71606	0.88846514
GLRA3	Inverse variance weighted	4	0.164505	0.10654	0.122573	1.178809	0.956653	1.452555	0.88846514
CPAMD8	Inverse variance weighted	2	-0.093706	0.060958	0.124235	0.91055	0.808008	1.026105	0.89864716
FAT3	Wald ratio	1	0.124827	0.081335	0.124852	1.132952	0.966001	1.328757	0.90099232
CDC42BPA	Inverse variance weighted	2	-0.163252	0.106435	0.125073	0.849377	0.689448	1.046405	0.90099232
IGLV2-23	Wald ratio	1	0.1367131	0.089197	0.125347	1.146499	0.962604	1.365525	0.90111035
OR52E6	Inverse variance weighted	2	-0.078626	0.051334	0.125613	0.924386	0.835904	1.022233	0.9011675
CDC14A	Wald ratio	1	0.2805596	0.184813	0.128996	1.32387	0.921572	1.901785	0.90530852
SCYL3	Wald ratio	1	-0.188026	0.123902	0.129129	0.828593	0.649941	1.056352	0.90530852
SLC25A33	Wald ratio	1	-0.232682	0.152821	0.127864	0.792406	0.587305	1.069132	0.90530852
HIF1A	Wald ratio	1	-0.341047	0.223286	0.126662	0.711026	0.459008	1.101413	0.90530852
USP8	Wald ratio	1	-0.307322	0.201619	0.127441	0.735413	0.495347	1.091826	0.90530852
DNASE1	Wald ratio	1	0.1702076	0.111793	0.127878	1.185551	0.95227	1.47598	0.90530852
CDH13	Wald ratio	1	0.261509	0.17263	0.12981	1.298889	0.926032	1.821871	0.90530852
JAG1	Wald ratio	1	0.1699777	0.112208	0.12981	1.185278	0.951278	1.47684	0.90530852
DSCAM	Wald ratio	1	-0.304748	0.201174	0.12981	0.737309	0.497058	1.093684	0.90530852
TM4SF1	Wald ratio	1	0.2394127	0.157367	0.128168	1.270503	0.933302	1.729533	0.90530852
FGL2	Wald ratio	1	-0.068736	0.045057	0.127123	0.933573	0.854664	1.019768	0.90530852
SSTR2	Inverse variance weighted	3	-0.137678	0.09034	0.127508	0.871379	0.729976	1.040174	0.90530852
KLHL24	Inverse variance weighted	3	-0.067038	0.044089	0.128377	0.935159	0.857742	1.019564	0.90530852
OBP2A	Inverse variance weighted	3	-0.05169	0.034062	0.129132	0.949623	0.888295	1.015185	0.90530852
OR1D5	Wald ratio	1	0.0989761	0.065395	0.130149	1.10404	0.971224	1.255018	0.90586998
CAMK1D	Wald ratio	1	0.4133447	0.274066	0.131504	1.511866	0.883536	2.587037	0.9080792
CDK4	Wald ratio	1	0.2056423	0.136205	0.131093	1.228314	0.940523	1.604165	0.9080792
KSR1	Wald ratio	1	0.3367342	0.223032	0.131093	1.400367	0.904468	2.168155	0.9080792
CALU	Wald ratio	1	-0.208396	0.138123	0.131357	0.811886	0.619331	1.064308	0.9080792
SCGB1A1	Inverse variance weighted	2	-0.102324	0.067945	0.13207	0.902737	0.790179	1.031328	0.90840353
BMP4	Inverse variance weighted	2	-0.076342	0.050665	0.131857	0.926499	0.838916	1.023226	0.90840353
TMPRSS11D	Wald ratio	1	0.1385322	0.092177	0.132867	1.148587	0.95874	1.376026	0.91031048
DEFA6	Inverse variance weighted	2	0.2522692	0.167786	0.132705	1.286942	0.926269	1.788057	0.91031048
MFSD5	Wald ratio	1	0.1852314	0.123488	0.133614	1.203497	0.944779	1.533062	0.91093159
CLEC14A	Wald ratio	1	0.1883975	0.125598	0.133614	1.207313	0.943862	1.544299	0.91093159
TREM1	Inverse variance weighted	2	0.0969237	0.064636	0.133739	1.101776	0.970675	1.250585	0.91093159
SLAMF8	Wald ratio	1	-0.132744	0.088919	0.135475	0.875689	0.735632	1.042413	0.91154986
PI4K2A	Wald ratio	1	-0.10835	0.07263	0.135751	0.897313	0.778252	1.03459	0.91154986
OR51J1	Wald ratio	1	-0.144283	0.096821	0.136173	0.865643	0.716016	1.046537	0.91154986

NXPH3	Wald ratio	1	0.3729833	0.250172	0.135985	1.45206	0.889271	2.371019	0.91154986
PPID	Wald ratio	1	0.2305323	0.154625	0.135985	1.25927	0.930035	1.705056	0.91154986
ARSK	Wald ratio	1	-0.231835	0.154886	0.134441	0.793077	0.585428	1.074377	0.91154986
POR	Wald ratio	1	-0.148461	0.099245	0.134679	0.862034	0.709652	1.047137	0.91154986
H1-0	Inverse variance weighted	3	-0.095196	0.063766	0.135461	0.909195	0.802377	1.030232	0.91154986
COL15A1	Inverse variance weighted	2	-0.151859	0.101492	0.134584	0.85911	0.704137	1.04819	0.91154986
TXNDC15	Wald ratio	1	0.551105	0.370286	0.136666	1.735169	0.839746	3.585383	0.91310494
PROM1	Wald ratio	1	-0.091794	0.061724	0.136967	0.912293	0.808339	1.029614	0.91337697
IL1RL1	Inverse variance weighted	3	-0.026801	0.018048	0.137547	0.973554	0.939717	1.00861	0.91375796
MGAT4A	Inverse variance weighted	2	0.3056488	0.205755	0.137411	1.357505	0.906984	2.031812	0.91375796
C1QTNF5	Wald ratio	1	-0.167702	0.11359	0.139842	0.845606	0.676828	1.056472	0.91683229
SLCO1C1	Wald ratio	1	0.173845	0.117432	0.138769	1.189871	0.945236	1.497821	0.91683229
S1PR4	Wald ratio	1	0.3057847	0.206985	0.139588	1.35769	0.904922	2.036995	0.91683229
LTF	Wald ratio	1	-0.042398	0.028632	0.138668	0.958489	0.906181	1.013816	0.91683229
FZD3	Wald ratio	1	0.1076318	0.072711	0.138803	1.113638	0.965719	1.284213	0.91683229
HS3ST1	Inverse variance weighted	2	0.1568201	0.106193	0.139744	1.169785	0.949976	1.440455	0.91683229
COL25A1	Inverse variance weighted	2	0.1884625	0.127522	0.139438	1.207392	0.940372	1.550232	0.91683229
WNT3	Wald ratio	1	0.1282244	0.086914	0.14013	1.136808	0.958749	1.347937	0.91699752
SLC16A11	Wald ratio	1	-0.272968	0.185287	0.140693	0.761118	0.529337	1.094388	0.9189685
PADI1	Wald ratio	1	-0.096329	0.065815	0.143293	0.908165	0.798255	1.033208	0.91984785
HSD11B1	Wald ratio	1	-0.251417	0.172111	0.144075	0.777698	0.555018	1.08972	0.91984785
RNLS	Wald ratio	1	-0.136122	0.093848	0.146933	0.872736	0.726102	1.048982	0.91984785
NRXN3	Wald ratio	1	-0.429586	0.292826	0.142367	0.650779	0.366585	1.155293	0.91984785
SDR42E2	Wald ratio	1	-0.166631	0.113331	0.141481	0.846512	0.677897	1.057068	0.91984785
OR3A4P	Wald ratio	1	0.0882971	0.060853	0.146785	1.092313	0.969499	1.230683	0.91984785
SECTM1	Wald ratio	1	0.1648366	0.112896	0.144269	1.1792	0.945124	1.47125	0.91984785
PDE4C	Wald ratio	1	-0.08988	0.061967	0.146933	0.914041	0.809503	1.032079	0.91984785
UGT1A6	Wald ratio	1	0.2402237	0.165809	0.147395	1.271534	0.91873	1.759817	0.91984785
COL20A1	Wald ratio	1	0.195808	0.134696	0.146028	1.216293	0.934077	1.583776	0.91984785
SLC25A17	Wald ratio	1	-0.192134	0.131395	0.143669	0.825196	0.63784	1.067586	0.91984785
ITGB5	Wald ratio	1	0.2004122	0.137657	0.145425	1.221906	0.932958	1.600346	0.91984785
SLC10A6	Wald ratio	1	-0.153057	0.104777	0.144075	0.858081	0.698779	1.053699	0.91984785
CD74	Wald ratio	1	0.4770888	0.329279	0.147369	1.611376	0.845102	3.072451	0.91984785
GABRB2	Wald ratio	1	-0.372866	0.255888	0.145077	0.688757	0.417109	1.137321	0.91984785
GRM6	Wald ratio	1	-0.209313	0.144354	0.147059	0.811141	0.611251	1.076398	0.91984785
F2RL2	Wald ratio	1	0.156525	0.107915	0.146933	1.16944	0.946497	1.444897	0.91984785
RFPL4B	Wald ratio	1	-0.125159	0.085734	0.14433	0.882357	0.745875	1.043812	0.91984785
ABCC10	Wald ratio	1	-0.40677	0.280765	0.147396	0.665797	0.384017	1.154342	0.91984785
PMS2P4	Wald ratio	1	-0.084326	0.057878	0.145129	0.919131	0.82056	1.029543	0.91984785
SCARA5	Wald ratio	1	0.1488383	0.101481	0.142467	1.160485	0.951169	1.415865	0.91984785
ERMAP	Inverse variance weighted	3	0.0945165	0.064934	0.145509	1.099127	0.967777	1.248305	0.91984785
PTH2R	Inverse variance weighted	2	0.1553334	0.106326	0.144037	1.168047	0.948318	1.438688	0.91984785
GHR	Inverse variance weighted	2	-0.043428	0.029926	0.146733	0.957502	0.902955	1.015344	0.91984785
FBP1	Inverse variance weighted	2	-0.071293	0.049087	0.146396	0.931189	0.845773	1.025231	0.91984785
CHSY1	Wald ratio	1	0.1725217	0.119355	0.14833	1.188298	0.940434	1.501488	0.92402885
MGST3	Wald ratio	1	0.1943986	0.134584	0.148614	1.21458	0.932967	1.581198	0.92405775
LAMP1	Wald ratio	1	0.4103635	0.284457	0.149127	1.507366	0.863146	2.632406	0.92405775
IL13	Wald ratio	1	-0.135445	0.093838	0.148909	0.873327	0.726609	1.049671	0.92405775
RYR3	Inverse variance weighted	2	0.145529	0.101021	0.149704	1.156651	0.94888	1.409916	0.92599365
FCRL5	Wald ratio	1	0.1202363	0.085783	0.161023	1.127763	0.953231	1.334251	0.9283787
ECE1	Wald ratio	1	-0.239959	0.168651	0.154789	0.78666	0.565234	1.094827	0.9283787
OR2M7	Wald ratio	1	-0.080088	0.056393	0.155559	0.923036	0.826448	1.030911	0.9283787
MUC5B	Wald ratio	1	0.0258486	0.018077	0.152744	1.026186	0.990463	1.063196	0.9283787
OR52B6	Wald ratio	1	0.1485062	0.103324	0.150636	1.1601	0.947424	1.420517	0.9283787
OR5P3	Wald ratio	1	-0.202511	0.142557	0.155444	0.816677	0.617594	1.079935	0.9283787
KLRK1	Wald ratio	1	0.0790182	0.056144	0.159307	1.082224	0.969451	1.208115	0.9283787
ITPR2	Wald ratio	1	-0.225447	0.158134	0.153962	0.798159	0.585442	1.088167	0.9283787
CPM	Wald ratio	1	0.0760379	0.05408	0.15972	1.079003	0.970485	1.199657	0.9283787
SLC28A2	Wald ratio	1	-0.041505	0.029566	0.160365	0.959344	0.905332	1.016579	0.9283787
MEFV	Wald ratio	1	0.1632703	0.115354	0.156956	1.177355	0.939109	1.476042	0.9283787
NUCB1	Wald ratio	1	0.2124632	0.150935	0.159237	1.236721	0.920011	1.662457	0.9283787
SBK3	Wald ratio	1	-0.270651	0.191537	0.157643	0.762883	0.524105	1.110447	0.9283787
CPS1	Wald ratio	1	-0.055823	0.039764	0.160365	0.945707	0.8748	1.022361	0.9283787
OTOS	Wald ratio	1	-0.075512	0.053107	0.155063	0.927269	0.835603	1.028991	0.9283787
PLTP	Wald ratio	1	0.1515511	0.107654	0.1592	1.163638	0.942283	1.436992	0.9283787

HRH3	Wald ratio	1	0.5738974	0.406059	0.157558	1.775172	0.800932	3.934461	0.9283787
HTR3D	Wald ratio	1	-0.139258	0.09905	0.159742	0.870004	0.716487	1.056413	0.9283787
IL17RE	Wald ratio	1	-0.11515	0.081544	0.157914	0.891232	0.75959	1.045689	0.9283787
STIM2	Wald ratio	1	0.0919426	0.064831	0.156138	1.096302	0.965483	1.244846	0.9283787
HTN3	Wald ratio	1	-0.586102	0.410199	0.153055	0.556492	0.249052	1.243449	0.9283787
SPINK5	Wald ratio	1	0.1291273	0.091844	0.159742	1.137835	0.950386	1.362255	0.9283787
DUSP1	Wald ratio	1	0.1850229	0.129841	0.154157	1.203246	0.932893	1.551947	0.9283787
SLC22A16	Wald ratio	1	0.2086408	0.146599	0.154676	1.232002	0.924324	1.642098	0.9283787
MAS1	Wald ratio	1	0.1559563	0.111277	0.16106	1.168775	0.939745	1.453623	0.9283787
MET	Wald ratio	1	-0.112556	0.080058	0.159742	0.893547	0.763785	1.045355	0.9283787
ADCYAP1R1	Wald ratio	1	0.145682	0.103926	0.160979	1.156828	0.943638	1.418184	0.9283787
TRPA1	Wald ratio	1	0.0946682	0.06728	0.159404	1.099294	0.963483	1.254249	0.9283787
PTGS1	Wald ratio	1	0.3131607	0.221776	0.157932	1.367741	0.885574	2.112434	0.9283787
IFNA14	Wald ratio	1	-0.097438	0.068089	0.15242	0.907159	0.793825	1.036673	0.9283787
PROK1	Inverse variance weighted	3	-0.09262	0.064787	0.152832	0.91154	0.802837	1.034961	0.9283787
NAXE	Inverse variance weighted	3	0.0400622	0.02853	0.160255	1.040876	0.984269	1.100738	0.9283787
CAPN8	Inverse variance weighted	2	-0.070837	0.050033	0.156829	0.931613	0.844592	1.027601	0.9283787
MGMT	Inverse variance weighted	2	-0.034623	0.024346	0.154995	0.96597	0.920957	1.013182	0.9283787
CXCL12	Inverse variance weighted	2	0.1604811	0.114551	0.161227	1.174076	0.937969	1.469616	0.9283787
EIF2AK4	Inverse variance weighted	2	0.142731	0.099916	0.153144	1.15342	0.948282	1.402934	0.9283787
DECR2	Inverse variance weighted	3	0.0860806	0.061328	0.160436	1.089894	0.966453	1.229102	0.9283787
MALT1	Inverse variance weighted	2	0.2143311	0.151595	0.15741	1.239033	0.920539	1.667721	0.9283787
QPCT	Inverse variance weighted	2	0.0593275	0.042297	0.160729	1.061123	0.9767	1.152842	0.9283787
PARP3	Inverse variance weighted	3	-0.128112	0.090226	0.155635	0.879755	0.737157	1.049937	0.9283787
CXCL14	Inverse variance weighted	2	-0.189679	0.132158	0.151218	0.827224	0.638451	1.071812	0.9283787
FGF10	Inverse variance weighted	2	0.2994886	0.208764	0.151406	1.349169	0.896114	2.031278	0.9283787
CA7	Wald ratio	1	0.3288708	0.234908	0.161513	1.389398	0.876737	2.201833	0.92850267
SPOCK3	Wald ratio	1	0.450904	0.322806	0.162466	1.569731	0.833771	2.955312	0.93092268
PPAT	Wald ratio	1	-0.312566	0.223641	0.162225	0.731567	0.47194	1.134021	0.93092268
APH1B	Wald ratio	1	-0.214905	0.154054	0.163016	0.806618	0.596396	1.090941	0.93227725
TRH	Inverse variance weighted	2	0.1763745	0.126499	0.163235	1.192885	0.930937	1.52854	0.93227725
MAPK9	Wald ratio	1	-0.13519	0.097259	0.164529	0.87355	0.721936	1.057003	0.93642184
LATS1	Wald ratio	1	0.2016331	0.145232	0.165031	1.223399	0.920331	1.626269	0.93642184
SLC22A2	Wald ratio	1	0.1373178	0.098907	0.165031	1.147193	0.945028	1.392605	0.93642184
EPOR	Inverse variance weighted	2	0.3943938	0.283858	0.16471	1.483485	0.850468	2.587666	0.93642184
NTNG1	Wald ratio	1	0.2439335	0.175981	0.165705	1.276259	0.903943	1.801925	0.93658934
SLC25A47	Wald ratio	1	-0.123865	0.089356	0.165684	0.883499	0.741558	1.052609	0.93658934
CX3CL1	Inverse variance weighted	2	-0.420951	0.3038	0.165863	0.656422	0.361896	1.190646	0.93658934
TMEM9B	Wald ratio	1	-0.178133	0.128708	0.166358	0.836832	0.650248	1.076953	0.93664666
GPR6	Wald ratio	1	0.1187206	0.085791	0.166408	1.126055	0.951772	1.332252	0.93664666
GGCX	Inverse variance weighted	2	-0.053158	0.038442	0.166722	0.94823	0.879409	1.022436	0.93690845
AGBL2	Wald ratio	1	0.1197443	0.087271	0.170034	1.127209	0.949987	1.337492	0.94298171
HNRNPUL2	Wald ratio	1	-0.356622	0.260356	0.170766	0.700037	0.420244	1.166113	0.94298171
ACE	Wald ratio	1	0.100853	0.073539	0.170241	1.106114	0.957641	1.277607	0.94298171
FPR1	Wald ratio	1	0.086056	0.062798	0.170571	1.089867	0.96365	1.232617	0.94298171
SIRPB1	Wald ratio	1	-0.095013	0.069101	0.169131	0.909361	0.794175	1.041252	0.94298171
HSP90AA5P	Wald ratio	1	0.1429543	0.10416	0.169924	1.153677	0.940636	1.414969	0.94298171
PCDHA2	Wald ratio	1	-0.062296	0.045265	0.168748	0.939605	0.859834	1.026777	0.94298171
GPR146	Wald ratio	1	-0.215798	0.156575	0.16813	0.805898	0.592926	1.095367	0.94298171
UHMK1	Inverse variance weighted	2	0.3960815	0.288718	0.170106	1.48599	0.843829	2.616841	0.94298171
CRYZ	Inverse variance weighted	2	0.1287704	0.093878	0.170165	1.137429	0.946267	1.367209	0.94298171
ITGA7	Inverse variance weighted	2	0.1018642	0.074356	0.170704	1.107233	0.957074	1.280951	0.94298171
PRKAA2	Wald ratio	1	0.2631625	0.192462	0.171517	1.301038	0.892201	1.897218	0.94486629
PARP15	Wald ratio	1	-0.12904	0.094446	0.171851	0.878939	0.730406	1.057677	0.94486629
AKR1B15	Wald ratio	1	-0.126229	0.092403	0.171917	0.881413	0.735401	1.056415	0.94486629
PLCH2	Wald ratio	1	-0.338497	0.248103	0.17246	0.712841	0.438332	1.159263	0.94636881
KCNA2	Wald ratio	1	0.6743523	0.498248	0.175912	1.962761	0.739181	5.211759	0.94836393
PTAFR	Wald ratio	1	0.1994988	0.147456	0.176074	1.220791	0.914375	1.629889	0.94836393
PSMB2	Wald ratio	1	0.3652882	0.268594	0.17383	1.440929	0.851159	2.439353	0.94836393
SMPD1	Wald ratio	1	-0.310054	0.227829	0.173545	0.733408	0.46926	1.146246	0.94836393
ABCC6	Wald ratio	1	-0.097123	0.07166	0.175307	0.907444	0.788537	1.044282	0.94836393
FCAR	Wald ratio	1	-0.164982	0.121848	0.175735	0.847909	0.667775	1.076633	0.94836393
FSHR	Wald ratio	1	-0.051735	0.038062	0.174076	0.949581	0.881319	1.02313	0.94836393
COL9A3	Wald ratio	1	0.4396856	0.324753	0.175765	1.552219	0.821331	2.933513	0.94836393
GRIA1	Wald ratio	1	-0.334981	0.247594	0.176074	0.715352	0.440315	1.162188	0.94836393

OR2W6P	Wald ratio	1	0.1196928	0.088117	0.174358	1.12715	0.948364	1.339643	0.94836393
OGN	Wald ratio	1	-0.123529	0.091304	0.176074	0.883796	0.73898	1.056992	0.94836393
UCN3	Inverse variance weighted	2	0.0806439	0.059491	0.175237	1.083985	0.964681	1.218044	0.94836393
RPS27	Wald ratio	1	-0.350643	0.267596	0.190079	0.704235	0.416807	1.189871	0.95207514
EGLN1	Wald ratio	1	-0.138468	0.103628	0.181482	0.870691	0.710647	1.066777	0.95207514
PPT1	Wald ratio	1	0.2075886	0.157378	0.187153	1.230707	0.904049	1.675395	0.95207514
P4HA1	Wald ratio	1	0.2736891	0.204058	0.179845	1.314806	0.881382	1.961369	0.95207514
ADM	Wald ratio	1	0.1620998	0.123092	0.187872	1.175978	0.923892	1.496845	0.95207514
SIK2	Wald ratio	1	-0.247278	0.183297	0.177319	0.780924	0.545234	1.118495	0.95207514
SAA2	Wald ratio	1	0.1169669	0.087212	0.179863	1.124082	0.947462	1.333627	0.95207514
SLC1A2	Wald ratio	1	0.3581413	0.26727	0.180245	1.430668	0.847294	2.415702	0.95207514
APLNR	Wald ratio	1	0.1501482	0.113431	0.185603	1.162006	0.930367	1.451318	0.95207514
TUBA1A	Wald ratio	1	-0.251689	0.192263	0.190504	0.777486	0.533378	1.133313	0.95207514
GPR182	Wald ratio	1	-0.165167	0.123875	0.182422	0.847752	0.665004	1.080721	0.95207514
TPTE2	Wald ratio	1	0.0442956	0.033796	0.189966	1.045291	0.978295	1.116876	0.95207514
SPRYD7	Wald ratio	1	-0.20851	0.155046	0.178681	0.811793	0.599056	1.100077	0.95207514
RNASE7	Wald ratio	1	0.1107744	0.084167	0.188131	1.117143	0.94725	1.317507	0.95207514
LGALS3	Wald ratio	1	-0.150797	0.115112	0.190196	0.860022	0.686316	1.077693	0.95207514
ADAM20	Wald ratio	1	0.269418	0.204236	0.187119	1.309202	0.877319	1.953692	0.95207514
RPS6KL1	Wald ratio	1	-0.183802	0.136539	0.178253	0.832101	0.636725	1.087426	0.95207514
CSNK1G1	Wald ratio	1	0.2431368	0.185982	0.191105	1.275243	0.885691	1.836131	0.95207514
CCL2	Wald ratio	1	-0.144745	0.108063	0.180424	0.865243	0.700088	1.069358	0.95207514
CCR10	Wald ratio	1	-0.273942	0.205046	0.181549	0.760376	0.508733	1.136495	0.95207514
ABCA9	Wald ratio	1	-0.154308	0.114911	0.179318	0.857008	0.684181	1.073491	0.95207514
NPLOC4	Wald ratio	1	0.1724692	0.130691	0.186945	1.188235	0.919721	1.535143	0.95207514
UTS2R	Wald ratio	1	0.100341	0.075256	0.182422	1.105548	0.953934	1.281258	0.95207514
IL36RN	Wald ratio	1	-0.28646	0.216479	0.185745	0.750917	0.491272	1.14779	0.95207514
INPP5D	Wald ratio	1	-0.236673	0.177839	0.183246	0.789249	0.556973	1.118392	0.95207514
CNGA3	Wald ratio	1	0.1230816	0.094211	0.191399	1.130977	0.940286	1.36034	0.95207514
EPHB3	Wald ratio	1	-0.22596	0.172976	0.191448	0.79775	0.568364	1.119714	0.95207514
TAFA4	Wald ratio	1	0.1304344	0.099744	0.190978	1.139323	0.937008	1.385322	0.95207514
KLHL8	Wald ratio	1	0.4041929	0.304255	0.184024	1.498093	0.825185	2.719731	0.95207514
SPARC	Wald ratio	1	-0.166718	0.125039	0.182422	0.846438	0.662461	1.081509	0.95207514
GLRA1	Wald ratio	1	0.2824384	0.209991	0.178625	1.32636	0.878847	2.001749	0.95207514
LTC4S	Wald ratio	1	-0.218282	0.166452	0.18973	0.803899	0.580115	1.114009	0.95207514
SLC6A3	Wald ratio	1	-0.119254	0.089613	0.183265	0.887582	0.744609	1.058007	0.95207514
TRBV7-1	Wald ratio	1	0.2331961	0.173993	0.180161	1.262629	0.89778	1.77575	0.95207514
STEAP1	Wald ratio	1	0.0539832	0.040796	0.185758	1.055467	0.974357	1.143329	0.95207514
PRPF4	Wald ratio	1	0.1195873	0.090826	0.18795	1.127032	0.943243	1.346631	0.95207514
XCL2	Inverse variance weighted	2	-0.085341	0.064888	0.188438	0.918199	0.808543	1.042726	0.95207514
MDM4	Inverse variance weighted	2	-0.314886	0.239274	0.188172	0.729872	0.456639	1.166597	0.95207514
LGALS8	Inverse variance weighted	2	-0.131123	0.100212	0.190718	0.87711	0.720696	1.067471	0.95207514
INPP5B	Inverse variance weighted	2	-0.102974	0.077871	0.186047	0.90215	0.77445	1.050907	0.95207514
TUBAL3	Inverse variance weighted	2	-0.068081	0.051245	0.184003	0.934185	0.844913	1.032889	0.95207514
OR51V1	Inverse variance weighted	2	-0.068149	0.051714	0.187574	0.934121	0.844079	1.033769	0.95207514
C1QTNF9	Inverse variance weighted	2	-0.051062	0.038317	0.182654	0.95022	0.881471	1.02433	0.95207514
KBTBD7	Inverse variance weighted	2	0.217777	0.164473	0.185474	1.24331	0.900693	1.716256	0.95207514
HSP90AA1	Inverse variance weighted	2	-0.452884	0.336867	0.17882	0.635792	0.328525	1.230445	0.95207514
MAPK4	Inverse variance weighted	2	0.2351242	0.17751	0.185315	1.265066	0.893333	1.791484	0.95207514
PIN1	Inverse variance weighted	2	-0.34053	0.255211	0.182102	0.711393	0.43139	1.173138	0.95207514
CCNE1	Inverse variance weighted	2	0.1724465	0.131272	0.188963	1.188208	0.918652	1.536858	0.95207514
IL1RL2	Inverse variance weighted	2	0.0723163	0.054927	0.187975	1.074995	0.965277	1.197185	0.95207514
KLHL6	Inverse variance weighted	2	0.135956	0.103896	0.190678	1.145631	0.934559	1.404375	0.95207514
PDGFC	Inverse variance weighted	2	-0.163612	0.122665	0.182264	0.849071	0.66762	1.079838	0.95207514
PHYKPL	Inverse variance weighted	2	0.2095265	0.159392	0.188667	1.233094	0.902233	1.685286	0.95207514
SEMA3D	Inverse variance weighted	3	-0.079977	0.061033	0.190064	0.923137	0.819057	1.040444	0.95207514
CER1	Inverse variance weighted	2	0.0481745	0.036058	0.181543	1.049354	0.977752	1.126199	0.95207514
OR10D4P	Wald ratio	1	0.1138559	0.087276	0.192044	1.120591	0.944401	1.32965	0.9520824
OR52L1	Wald ratio	1	-0.136916	0.105003	0.19226	0.872043	0.709835	1.071319	0.9520824
CAPN12	Inverse variance weighted	2	0.1064765	0.08166	0.192266	1.112352	0.947833	1.305426	0.9520824
CAT	Wald ratio	1	-0.060325	0.046314	0.192738	0.941459	0.859762	1.030919	0.95307476
OVCH2	Wald ratio	1	-0.135164	0.105026	0.198109	0.873573	0.711049	1.073245	0.95383859
ADCY6	Wald ratio	1	0.2792159	0.217071	0.198342	1.322093	0.863948	2.023188	0.95383859
GNS	Wald ratio	1	-0.311615	0.242101	0.198049	0.732263	0.455603	1.176922	0.95383859
GPR162	Wald ratio	1	0.4015034	0.310729	0.19631	1.494069	0.812593	2.747061	0.95383859

METAP2	Wald ratio	1	0.1817662	0.14022	0.194873	1.199334	0.911135	1.578692	0.95383859
SLC25A39	Wald ratio	1	0.1238065	0.096195	0.198081	1.131797	0.937315	1.366631	0.95383859
LCT	Wald ratio	1	-0.153717	0.119209	0.197233	0.857515	0.678842	1.083214	0.95383859
SPR	Wald ratio	1	-0.339801	0.261557	0.193894	0.711912	0.426368	1.188689	0.95383859
UGT2B7	Wald ratio	1	0.0733536	0.056512	0.194283	1.076111	0.963281	1.202157	0.95383859
FDCSP	Wald ratio	1	-0.470056	0.363225	0.195625	0.624967	0.306671	1.273623	0.95383859
MEPE	Wald ratio	1	-0.055318	0.042924	0.197491	0.946184	0.869837	1.029233	0.95383859
GSTK1	Wald ratio	1	0.2642855	0.204447	0.196121	1.3025	0.872466	1.944495	0.95383859
NDUFAF6	Wald ratio	1	0.2490965	0.191613	0.193601	1.282866	0.881206	1.867606	0.95383859
TNFRSF12A	Inverse variance weighted	2	0.1113085	0.08581	0.194582	1.11774	0.944707	1.322465	0.95383859
PRKD2	Inverse variance weighted	4	0.0292897	0.022673	0.196407	1.029723	0.984966	1.076514	0.95383859
IL18R1	Inverse variance weighted	2	-0.020739	0.016067	0.196771	0.979475	0.949111	1.01081	0.95383859
TMPRSS7	Inverse variance weighted	2	-0.037176	0.028746	0.195917	0.963507	0.910722	1.01935	0.95383859
SLC4A7	Inverse variance weighted	2	-0.137818	0.10714	0.198327	0.871258	0.706232	1.074845	0.95383859
ACHE	Inverse variance weighted	2	-0.210282	0.162086	0.194511	0.810356	0.589801	1.113387	0.95383859
FGL1	Inverse variance weighted	2	-0.047721	0.03688	0.195678	0.9534	0.886916	1.024867	0.95383859
RPN2	Inverse variance weighted	2	-0.139616	0.108736	0.199144	0.869692	0.70276	1.076275	0.9550736
BOC	Inverse variance weighted	2	-0.055402	0.043123	0.198876	0.946104	0.869425	1.029546	0.9550736
LAMC2	Wald ratio	1	0.0211655	0.016497	0.199485	1.021391	0.988894	1.054956	0.9552568
PTPRE	Inverse variance weighted	2	-0.087384	0.068145	0.199728	0.916326	0.801759	1.047263	0.9552568
HHIP	Wald ratio	1	-0.265613	0.207543	0.200618	0.766736	0.510483	1.151623	0.95689883
GRM8	Wald ratio	1	-0.058816	0.04595	0.200545	0.94288	0.861674	1.031739	0.95689883
SPON2	Inverse variance weighted	2	-0.039129	0.030597	0.200939	0.961626	0.905653	1.021059	0.95712808
PRKD1	Wald ratio	1	0.203941	0.159675	0.201522	1.226226	0.896711	1.676827	0.95859788
APOA5	Wald ratio	1	-0.094883	0.0745	0.202811	0.90948	0.785917	1.052468	0.95947759
LTBR	Wald ratio	1	0.0905797	0.070995	0.202004	1.094809	0.95259	1.25826	0.95947759
ANTXR1	Wald ratio	1	-0.399759	0.313672	0.202505	0.670482	0.362564	1.23991	0.95947759
OR2L2	Inverse variance weighted	3	-0.072095	0.056642	0.203077	0.930442	0.832674	1.03969	0.95947759
KDR	Inverse variance weighted	2	-0.120093	0.094281	0.202743	0.886838	0.737209	1.066837	0.95947759
CD209	Wald ratio	1	-0.128902	0.101386	0.203587	0.87906	0.720637	1.072309	0.9605896
COL13A1	Wald ratio	1	0.0865879	0.068629	0.207063	1.090447	0.953205	1.247449	0.9614612
F10	Wald ratio	1	0.1780379	0.140693	0.205716	1.194871	0.906902	1.574278	0.9614612
CDKN3	Wald ratio	1	0.158115	0.125212	0.206671	1.171301	0.916401	1.497102	0.9614612
CDH5	Wald ratio	1	0.3003549	0.237542	0.206076	1.350338	0.847701	2.151009	0.9614612
SREBF2	Wald ratio	1	-0.229271	0.181765	0.207178	0.795113	0.556811	1.135403	0.9614612
DUSP22	Wald ratio	1	0.2534883	0.200398	0.205898	1.288512	0.869974	1.908405	0.9614612
ENPP2	Wald ratio	1	-0.554664	0.439465	0.2069	0.574265	0.242679	1.358915	0.9614612
HSD17B7	Inverse variance weighted	2	0.1003143	0.078994	0.204122	1.105518	0.946945	1.290646	0.9614612
FXYD2	Inverse variance weighted	2	0.0528636	0.041849	0.206521	1.054286	0.97126	1.144409	0.9614612
NUAK1	Inverse variance weighted	2	-0.169711	0.134043	0.205477	0.843908	0.648927	1.097475	0.9614612
OR6J1	Inverse variance weighted	2	0.0885211	0.070007	0.206067	1.092557	0.952473	1.253244	0.9614612
SLC16A3	Inverse variance weighted	2	0.1407444	0.111621	0.207342	1.15113	0.924933	1.432645	0.9614612
PTPN18	Inverse variance weighted	2	-0.147579	0.116295	0.204437	0.862794	0.686934	1.083676	0.9614612
TUBB6	Inverse variance weighted	2	0.1664304	0.132089	0.207674	1.181081	0.911682	1.530088	0.96172947
KLHL21	Wald ratio	1	0.1754701	0.139578	0.208702	1.191806	0.906555	1.566813	0.96360884
CST3	Wald ratio	1	-0.130208	0.103551	0.2086	0.877913	0.71665	1.075464	0.96360884
SLC25A31	Wald ratio	1	0.1543081	0.122829	0.209014	1.16685	0.917193	1.484464	0.96360884
PSMB5	Inverse variance weighted	4	-0.177637	0.141451	0.209181	0.837246	0.634524	1.104736	0.96360884
CFH	Wald ratio	1	0.1343551	0.10809	0.213871	1.143799	0.925425	1.413702	0.96391639
SCD	Wald ratio	1	-0.249115	0.199798	0.212458	0.77949	0.526914	1.15314	0.96391639
LRIT2	Wald ratio	1	-0.087904	0.070059	0.209582	0.915849	0.798341	1.050653	0.96391639
NOS1	Wald ratio	1	-0.109743	0.088673	0.215856	0.896064	0.753111	1.066151	0.96391639
CELA1	Wald ratio	1	0.1199187	0.095834	0.210817	1.127405	0.93434	1.360364	0.96391639
SERPINA5	Wald ratio	1	0.0810535	0.065116	0.213222	1.084429	0.954494	1.232052	0.96391639
GRIN2A	Wald ratio	1	0.2642079	0.212039	0.212753	1.302399	0.859513	1.973493	0.96391639
NPW	Wald ratio	1	0.164952	0.131962	0.2113	1.179337	0.910562	1.527446	0.96391639
ATP1B2	Wald ratio	1	-0.367205	0.294936	0.21312	0.692668	0.388571	1.234751	0.96391639
DDX1	Wald ratio	1	-0.221767	0.177106	0.210506	0.801102	0.566151	1.133557	0.96391639
FKBP1B	Wald ratio	1	0.4047533	0.326952	0.215731	1.498933	0.789724	2.845045	0.96391639
CD207	Wald ratio	1	0.0858706	0.068938	0.212906	1.089665	0.951944	1.247311	0.96391639
BPIFC	Wald ratio	1	-0.130257	0.103902	0.209966	0.87787	0.716122	1.07615	0.96391639
QDPR	Wald ratio	1	-0.219908	0.177686	0.215856	0.802593	0.56656	1.136957	0.96391639
SCGB3A2	Wald ratio	1	0.157057	0.126495	0.21438	1.170062	0.913134	1.499282	0.96391639
DLD	Wald ratio	1	-0.094856	0.076035	0.212205	0.909504	0.783578	1.055668	0.96391639
EPHX1	Inverse variance weighted	2	0.150698	0.121462	0.214717	1.162645	0.91634	1.475156	0.96391639

TUB	Inverse variance weighted	2	-0.218468	0.175003	0.211896	0.803749	0.570368	1.132625	0.96391639
KCNH5	Inverse variance weighted	4	-0.119505	0.09582	0.212331	0.88736	0.735421	1.070689	0.96391639
ESR2	Inverse variance weighted	2	0.0738243	0.059636	0.215744	1.076618	0.957853	1.210108	0.96391639
CHL1	Inverse variance weighted	2	0.1935221	0.155602	0.21361	1.213516	0.894529	1.646253	0.96391639
TMPRSS11A	Inverse variance weighted	2	-0.142646	0.115145	0.215407	0.867061	0.691889	1.086584	0.96391639
C6orf120	Inverse variance weighted	2	0.2113642	0.170461	0.214991	1.235362	0.884495	1.725414	0.96391639
KCNQ5	Inverse variance weighted	2	-0.144642	0.116862	0.215821	0.865332	0.68819	1.088072	0.96391639
PARP11	Wald ratio	1	-0.103094	0.083491	0.216911	0.902043	0.765875	1.06242	0.96739731
ROR1	Wald ratio	1	-0.07442	0.06141	0.225564	0.928282	0.823013	1.047015	0.96749443
CPT1A	Wald ratio	1	-0.128676	0.106344	0.226279	0.879259	0.71383	1.083025	0.96749443
KCNA5	Wald ratio	1	0.0637258	0.052546	0.225219	1.0658	0.961497	1.181418	0.96749443
OR4N5	Wald ratio	1	0.178746	0.145121	0.218058	1.195717	0.899703	1.589123	0.96749443
OR4F6	Wald ratio	1	0.1497119	0.122599	0.222028	1.1615	0.9134	1.476989	0.96749443
PAK6	Wald ratio	1	0.251988	0.205262	0.219581	1.286581	0.860428	1.923797	0.96749443
MAN2A2	Wald ratio	1	-0.318904	0.2604	0.2207	0.726945	0.43636	1.211041	0.96749443
TRPV3	Wald ratio	1	0.0736428	0.060534	0.223771	1.076422	0.955995	1.21202	0.96749443
SLC25A10	Wald ratio	1	0.2209145	0.182072	0.225002	1.247217	0.872889	1.782072	0.96749443
GALNT1	Wald ratio	1	0.3566016	0.293334	0.224104	1.428467	0.803858	2.538404	0.96749443
IFNL2	Wald ratio	1	-0.146354	0.120923	0.22616	0.863851	0.681565	1.094891	0.96749443
DYRK1B	Wald ratio	1	0.2647248	0.216289	0.220976	1.303072	0.852824	1.99103	0.96749443
LAIR1	Wald ratio	1	-0.294699	0.239645	0.218798	0.744756	0.465612	1.191253	0.96749443
DPP4	Wald ratio	1	-0.069213	0.057101	0.225466	0.933128	0.834327	1.043629	0.96749443
PLEK	Wald ratio	1	-0.194237	0.157818	0.218409	0.823463	0.604375	1.121969	0.96749443
NCOA3	Wald ratio	1	-0.31994	0.262052	0.222123	0.726192	0.434499	1.21371	0.96749443
CACNA1I	Wald ratio	1	0.3205204	0.263539	0.223903	1.377845	0.821999	2.30956	0.96749443
EGF	Wald ratio	1	-0.155694	0.128509	0.225689	0.855821	0.665263	1.100962	0.96749443
ENC1	Wald ratio	1	0.2005903	0.163271	0.219233	1.222124	0.887434	1.68304	0.96749443
EPHB4	Wald ratio	1	-0.15325	0.126244	0.224779	0.857915	0.669859	1.098767	0.96749443
FKBP14	Wald ratio	1	-0.221987	0.181141	0.220391	0.800926	0.561568	1.142307	0.96749443
ADCY1	Wald ratio	1	-0.385864	0.312979	0.217622	0.679863	0.368136	1.255551	0.96749443
CADM3	Inverse variance weighted	2	-0.310981	0.256709	0.225737	0.732728	0.443024	1.211875	0.96749443
TRIM58	Inverse variance weighted	2	-0.049398	0.040742	0.225336	0.951802	0.878753	1.030925	0.96749443
LEPR	Inverse variance weighted	2	-0.087175	0.071881	0.225221	0.916517	0.796074	1.055181	0.96749443
ADAMTS14	Inverse variance weighted	2	-0.039851	0.032921	0.226077	0.960932	0.900887	1.02498	0.96749443
DRD4	Inverse variance weighted	2	-0.115598	0.094509	0.221278	0.890833	0.740199	1.072123	0.96749443
P2RX7	Inverse variance weighted	2	0.0929399	0.07672	0.225739	1.097396	0.944186	1.275466	0.96749443
P3H3	Inverse variance weighted	2	0.0293552	0.024278	0.226605	1.02979	0.981936	1.079976	0.96749443
ADAMTS1	Inverse variance weighted	3	-0.096795	0.079053	0.220791	0.907742	0.777447	1.059874	0.96749443
SNRK	Inverse variance weighted	2	0.1251172	0.101716	0.218672	1.133281	0.928444	1.383311	0.96749443
HSD17B13	Inverse variance weighted	2	0.0342961	0.027886	0.218751	1.034891	0.979845	1.093029	0.96749443
SLIT3	Inverse variance weighted	2	0.4080449	0.332804	0.220167	1.503875	0.783292	2.887352	0.96749443
RTN4IP1	Inverse variance weighted	2	-0.080886	0.066095	0.221036	0.922299	0.810233	1.049864	0.96749443
SLC25A27	Inverse variance weighted	2	0.0272833	0.022556	0.226436	1.027659	0.983216	1.07411	0.96749443
IL3	Wald ratio	1	0.1063484	0.088207	0.227943	1.112209	0.935629	1.322116	0.97083883
ABCB8	Wald ratio	1	0.2067036	0.171413	0.227863	1.229618	0.878741	1.720599	0.97083883
DDI1	Wald ratio	1	-0.236432	0.196289	0.228393	0.78944	0.537321	1.159855	0.971572
SLC12A3	Inverse variance weighted	2	0.0915401	0.076159	0.229376	1.095861	0.943904	1.272281	0.9745711
NDUFS3	Wald ratio	1	0.202748	0.168957	0.230139	1.224764	0.879495	1.705576	0.97662767
PLA2G2C	Wald ratio	1	0.1183693	0.098936	0.231529	1.12566	0.927239	1.366541	0.9813369
HDC	Wald ratio	1	0.1456528	0.121858	0.231983	1.156794	0.911021	1.468872	0.98207077
MST1P2	Wald ratio	1	0.1451974	0.121778	0.233141	1.156268	0.910749	1.467974	0.98222059
OR2M5	Wald ratio	1	-0.135659	0.113715	0.232876	0.87314	0.698696	1.091138	0.98222059
NOS2	Wald ratio	1	-0.076608	0.064199	0.232761	0.926253	0.816737	1.050455	0.98222059
CYP4F12	Wald ratio	1	0.078731	0.065995	0.232876	1.081913	0.95064	1.231314	0.98222059
ADCY2	Inverse variance weighted	2	0.1247629	0.104868	0.234158	1.13288	0.922398	1.391391	0.98532101
KISS1	Wald ratio	1	-0.120046	0.101055	0.234865	0.88688	0.72752	1.081147	0.98592669
MUCL1	Wald ratio	1	0.098668	0.083046	0.234787	1.1037	0.93791	1.298795	0.98592669
AMPD1	Wald ratio	1	-0.298967	0.274537	0.276161	0.741584	0.432982	1.270139	0.98631075
MMP23B	Wald ratio	1	-0.103462	0.093577	0.268885	0.901711	0.750608	1.083232	0.98631075
PLOD1	Wald ratio	1	0.0995543	0.088902	0.262792	1.104678	0.928027	1.314956	0.98631075
BGLAP	Wald ratio	1	-0.060493	0.051627	0.241311	0.941301	0.850712	1.041536	0.98631075
APCS	Wald ratio	1	-0.214319	0.194755	0.271133	0.807091	0.55099	1.182227	0.98631075
MST1L	Wald ratio	1	-0.102405	0.093653	0.274193	0.902664	0.751289	1.084538	0.98631075
PLA2G4A	Wald ratio	1	0.1466617	0.124803	0.239938	1.157962	0.906691	1.478867	0.98631075
CHIT1	Wald ratio	1	0.0313141	0.027372	0.252624	1.031809	0.977912	1.088678	0.98631075

OR2M4	Wald ratio	1	-0.111575	0.094473	0.237589	0.894424	0.743236	1.076366	0.98631075
GRIK3	Wald ratio	1	0.2845932	0.259448	0.272678	1.329221	0.799375	2.210264	0.98631075
MKNK1	Wald ratio	1	-0.118259	0.100995	0.241623	0.888466	0.728906	1.082953	0.98631075
CPT2	Wald ratio	1	-0.156524	0.133376	0.240574	0.855111	0.658401	1.110592	0.98631075
CHUK	Wald ratio	1	-0.320201	0.277137	0.247931	0.726003	0.42173	1.249805	0.98631075
PNLIPRP1	Wald ratio	1	-0.161066	0.148829	0.279154	0.851236	0.635863	1.139556	0.98631075
DMBT1	Wald ratio	1	0.1056384	0.091199	0.246728	1.11142	0.929498	1.328948	0.98631075
GLRX3	Wald ratio	1	-0.094947	0.083926	0.257923	0.909421	0.771482	1.072024	0.98631075
ADAM8	Wald ratio	1	-0.120658	0.102782	0.24043	0.886337	0.724618	1.08415	0.98631075
SGPL1	Wald ratio	1	-0.125746	0.109302	0.249962	0.881839	0.711786	1.09252	0.98631075
FGFBP3	Wald ratio	1	0.1716827	0.147727	0.24517	1.187301	0.888819	1.58602	0.98631075
MUC2	Wald ratio	1	-0.177672	0.154597	0.250451	0.837217	0.618361	1.133533	0.98631075
MTCH2	Wald ratio	1	0.0760526	0.069496	0.273806	1.079019	0.941614	1.236476	0.98631075
OR9I1	Wald ratio	1	0.0975629	0.090253	0.279698	1.102481	0.923733	1.315818	0.98631075
NOX4	Wald ratio	1	0.1367967	0.115447	0.236045	1.146595	0.914407	1.437741	0.98631075
RECQL	Wald ratio	1	0.308179	0.267428	0.249165	1.360945	0.805751	2.298687	0.98631075
FKBP4	Wald ratio	1	-0.197271	0.169435	0.244308	0.820968	0.58898	1.144333	0.98631075
C1S	Wald ratio	1	0.20914	0.181476	0.249141	1.232618	0.86368	1.759154	0.98631075
NTS	Wald ratio	1	0.0930678	0.082677	0.2603	1.097536	0.933347	1.290609	0.98631075
GAS6	Wald ratio	1	-0.316135	0.288553	0.273259	0.728961	0.414079	1.283292	0.98631075
FGF9	Wald ratio	1	0.2134157	0.193171	0.269245	1.237899	0.847725	1.807655	0.98631075
TRPC4	Wald ratio	1	0.1884301	0.166116	0.256657	1.207353	0.871833	1.671995	0.98631075
HTR2A	Wald ratio	1	-0.364537	0.313779	0.245331	0.694518	0.375482	1.284628	0.98631075
SLC25A29	Wald ratio	1	0.1633537	0.148503	0.271332	1.177453	0.880106	1.57526	0.98631075
RNASE6	Wald ratio	1	-0.154036	0.134494	0.252085	0.857241	0.658596	1.115801	0.98631075
RDH11	Wald ratio	1	-0.082451	0.073891	0.264485	0.920856	0.7967	1.064361	0.98631075
SCG5	Wald ratio	1	0.2321241	0.210166	0.269386	1.261276	0.835436	1.904178	0.98631075
ADAL	Wald ratio	1	-0.040825	0.036316	0.260935	0.959997	0.894041	1.030819	0.98631075
TRIM69	Wald ratio	1	-0.02767	0.025614	0.280032	0.97271	0.925082	1.02279	0.98631075
DAPK2	Wald ratio	1	-0.156997	0.138278	0.25622	0.854706	0.651797	1.120784	0.98631075
SEMA4B	Wald ratio	1	-0.207223	0.191749	0.27983	0.812838	0.558193	1.183652	0.98631075
GNPTG	Wald ratio	1	0.198506	0.17485	0.256252	1.219579	0.865715	1.718087	0.98631075
CAPN15	Wald ratio	1	-0.342211	0.316078	0.278952	0.710198	0.382233	1.319566	0.98631075
PRSS54	Wald ratio	1	-0.114768	0.102094	0.260955	0.891573	0.729882	1.089083	0.98631075
IL34	Wald ratio	1	0.1195353	0.109574	0.275313	1.126973	0.909164	1.396963	0.98631075
CHST6	Wald ratio	1	-0.187454	0.16572	0.257993	0.829067	0.599136	1.147238	0.98631075
LAMA3	Wald ratio	1	0.1498344	0.129421	0.246975	1.161642	0.901378	1.497055	0.98631075
APLP1	Wald ratio	1	0.2705828	0.231267	0.242001	1.310728	0.833017	2.062393	0.98631075
CAPNS1	Wald ratio	1	-0.25389	0.218381	0.244991	0.775777	0.505646	1.190219	0.98631075
TMIGD2	Wald ratio	1	-0.1448	0.125789	0.249677	0.865195	0.676145	1.107103	0.98631075
LTBP4	Wald ratio	1	-0.209593	0.191797	0.274489	0.810914	0.556819	1.180963	0.98631075
PPP5C	Wald ratio	1	0.2532777	0.214582	0.23787	1.288241	0.845942	1.961794	0.98631075
FKRP	Wald ratio	1	0.0817867	0.07235	0.258293	1.085224	0.941747	1.250561	0.98631075
MUC16	Wald ratio	1	-0.111372	0.099307	0.262077	0.894606	0.736377	1.086834	0.98631075
GPR39	Wald ratio	1	-0.082475	0.072079	0.252528	0.920835	0.799516	1.060563	0.98631075
KCNS3	Wald ratio	1	0.2633709	0.239563	0.271603	1.301309	0.813692	2.081139	0.98631075
CALCRL	Wald ratio	1	0.280581	0.238842	0.240092	1.323899	0.828989	2.114272	0.98631075
SLC40A1	Wald ratio	1	-0.064808	0.056334	0.249962	0.937247	0.83927	1.046661	0.98631075
CLK1	Wald ratio	1	-0.161973	0.145552	0.265786	0.850464	0.639381	1.131233	0.98631075
ERBB4	Wald ratio	1	0.1976044	0.17964	0.271332	1.21848	0.856851	1.732732	0.98631075
SCG2	Wald ratio	1	0.1793179	0.158222	0.257074	1.196401	0.877396	1.63139	0.98631075
NMUR1	Wald ratio	1	-0.05068	0.045775	0.268232	0.950583	0.869011	1.039812	0.98631075
CHRND	Wald ratio	1	0.0923461	0.080706	0.252528	1.096744	0.936283	1.284706	0.98631075
EFEMP1	Wald ratio	1	0.0509476	0.045853	0.266521	1.052268	0.961824	1.151217	0.98631075
AAK1	Wald ratio	1	-0.234757	0.213608	0.271764	0.790763	0.520259	1.201913	0.98631075
IGKV2D-40	Wald ratio	1	0.260562	0.226041	0.249025	1.297659	0.833203	2.02102	0.98631075
L3MBTL1	Wald ratio	1	-0.238483	0.218078	0.274145	0.787822	0.513803	1.20798	0.98631075
LIPI	Wald ratio	1	-0.094494	0.084171	0.26159	0.909833	0.771461	1.073025	0.98631075
ITGB2	Wald ratio	1	0.1817592	0.157416	0.248238	1.199325	0.88093	1.632798	0.98631075
RTN4R	Wald ratio	1	0.4117065	0.359825	0.252546	1.509391	0.745612	3.055561	0.98631075
IGLC7	Wald ratio	1	0.302372	0.260446	0.245652	1.353064	0.812123	2.254317	0.98631075
ATP2B2	Wald ratio	1	-0.466886	0.426867	0.274065	0.626951	0.271567	1.447407	0.98631075
CHST2	Wald ratio	1	-0.194769	0.171513	0.256127	0.823025	0.588056	1.151881	0.98631075
CLCN2	Wald ratio	1	0.1474817	0.12974	0.255642	1.158912	0.898698	1.494469	0.98631075
ITPR1	Wald ratio	1	-0.428501	0.368279	0.244618	0.651485	0.316533	1.34088	0.98631075

QARS1	Wald ratio	1	-0.184531	0.162846	0.257147	0.831494	0.604285	1.144134	0.98631075
COL8A1	Wald ratio	1	-0.105095	0.089256	0.239013	0.900239	0.755756	1.072344	0.98631075
SLC2A9	Wald ratio	1	0.0544743	0.049696	0.273011	1.055985	0.957979	1.164018	0.98631075
KLHL2	Wald ratio	1	-0.205646	0.178588	0.24952	0.814121	0.573683	1.155329	0.98631075
GNRHR	Wald ratio	1	0.0595072	0.05493	0.27866	1.061313	0.952986	1.181955	0.98631075
SMR3B	Wald ratio	1	0.2774418	0.256356	0.279141	1.319749	0.798503	2.181254	0.98631075
OR7E85P	Wald ratio	1	0.2437756	0.217228	0.261772	1.276058	0.833609	1.953342	0.98631075
EIF4E	Wald ratio	1	0.5524527	0.465568	0.235378	1.737509	0.697633	4.3274	0.98631075
IK	Wald ratio	1	-0.113758	0.098333	0.247328	0.892474	0.736025	1.082177	0.98631075
IL6ST	Wald ratio	1	0.3961195	0.360109	0.271332	1.486047	0.733672	3.009977	0.98631075
ADAMTS6	Wald ratio	1	-0.144342	0.130038	0.266999	0.865591	0.670845	1.116873	0.98631075
GFM2	Wald ratio	1	0.0491676	0.041519	0.23633	1.050396	0.968303	1.13945	0.98631075
SLC2A12	Wald ratio	1	-0.060539	0.053636	0.259019	0.941257	0.84733	1.045595	0.98631075
FUCA2	Wald ratio	1	-0.201384	0.185344	0.277241	0.817599	0.568554	1.175732	0.98631075
VIP	Wald ratio	1	0.2461573	0.226893	0.277963	1.279101	0.819917	1.995445	0.98631075
SLC17A3	Wald ratio	1	0.1107004	0.09569	0.247328	1.11706	0.926027	1.347503	0.98631075
SLC22A7	Wald ratio	1	-0.126261	0.107048	0.238204	0.881384	0.714569	1.087142	0.98631075
CYP3A43	Wald ratio	1	-0.089992	0.076794	0.241247	0.913938	0.786229	1.062392	0.98631075
PIK3CG	Wald ratio	1	0.1021778	0.089771	0.255032	1.10758	0.928883	1.320655	0.98631075
CFTR	Wald ratio	1	-0.08351	0.074538	0.262556	0.919881	0.794847	1.064584	0.98631075
PSMG3	Wald ratio	1	-0.189401	0.173736	0.275641	0.827454	0.588649	1.163139	0.98631075
ABCA13	Wald ratio	1	-0.075832	0.064264	0.238	0.926972	0.817267	1.051404	0.98631075
CDK6	Wald ratio	1	-0.202816	0.18	0.259844	0.816428	0.573719	1.161814	0.98631075
CALCR	Wald ratio	1	0.0831754	0.076744	0.278454	1.086732	0.934968	1.263132	0.98631075
ANGPT1	Wald ratio	1	0.1886107	0.167654	0.260589	1.207571	0.869366	1.677345	0.98631075
NAT2	Wald ratio	1	-0.081879	0.069625	0.239595	0.921384	0.80385	1.056103	0.98631075
TNFRSF10A	Wald ratio	1	0.0856466	0.074851	0.252528	1.089421	0.940766	1.261567	0.98631075
CRH	Wald ratio	1	0.2607323	0.234894	0.266999	1.29788	0.819009	2.056745	0.98631075
SIGMAR1	Wald ratio	1	-0.199766	0.184314	0.278439	0.818923	0.570626	1.175261	0.98631075
F11R	Inverse variance weighted	2	-0.095573	0.083695	0.253491	0.908852	0.771348	1.070869	0.98631075
TNFSF4	Inverse variance weighted	2	-0.087932	0.080044	0.271961	0.915823	0.782847	1.071386	0.98631075
LEFTY1	Inverse variance weighted	2	0.0380992	0.033203	0.25119	1.038834	0.973382	1.108688	0.98631075
OBSCN	Inverse variance weighted	2	-0.042799	0.039402	0.277386	0.958104	0.886896	1.035029	0.98631075
AGT	Inverse variance weighted	2	-0.045578	0.039339	0.246623	0.955446	0.884545	1.032029	0.98631075
RYR2	Inverse variance weighted	2	-0.25408	0.232539	0.274554	0.77563	0.491715	1.223476	0.98631075
ADGRA1	Inverse variance weighted	2	-0.145778	0.134851	0.279686	0.86435	0.663593	1.125842	0.98631075
PAOX	Inverse variance weighted	2	0.0846576	0.074017	0.252725	1.088344	0.941373	1.258262	0.98631075
FAS	Inverse variance weighted	3	-0.022967	0.020408	0.26041	0.977294	0.938975	1.017178	0.98631075
HTR3B	Inverse variance weighted	2	0.0562659	0.052045	0.279648	1.057879	0.955289	1.171486	0.98631075
OR8A1	Inverse variance weighted	2	-0.075258	0.067299	0.263453	0.927504	0.812887	1.058283	0.98631075
BBOX1	Inverse variance weighted	2	0.1193466	0.10507	0.256008	1.12676	0.917052	1.384423	0.98631075
OR52J3	Inverse variance weighted	2	-0.070469	0.064514	0.274696	0.931956	0.821259	1.057574	0.98631075
NAALAD2	Inverse variance weighted	2	0.0986946	0.085485	0.248287	1.103729	0.93346	1.305056	0.98631075
KLRC2	Inverse variance weighted	2	0.0170272	0.014443	0.238441	1.017173	0.988782	1.04638	0.98631075
SVOP	Inverse variance weighted	2	-0.11094	0.102317	0.278245	0.894993	0.732361	1.093739	0.98631075
GPR12	Inverse variance weighted	2	0.1321399	0.113852	0.245791	1.141268	0.913009	1.426593	0.98631075
TRPM7	Inverse variance weighted	2	0.2260156	0.196672	0.250473	1.253595	0.852603	1.84318	0.98631075
ADAM10	Inverse variance weighted	2	0.1880594	0.1619	0.245408	1.206905	0.878741	1.657622	0.98631075
PRKCB	Inverse variance weighted	2	0.0917091	0.083768	0.273602	1.096046	0.930089	1.291615	0.98631075
PLA2G15	Inverse variance weighted	2	-0.114873	0.09695	0.236069	0.89148	0.737202	1.078044	0.98631075
TRPV2	Inverse variance weighted	2	0.0208848	0.018898	0.269095	1.021104	0.983975	1.059635	0.98631075
RIOK3	Inverse variance weighted	2	-0.098546	0.088111	0.263385	0.906154	0.76243	1.076971	0.98631075
LIPG	Inverse variance weighted	2	-0.064488	0.057743	0.264072	0.937547	0.837224	1.049892	0.98631075
CNDP1	Inverse variance weighted	2	0.1155115	0.104465	0.268838	1.122447	0.914626	1.37749	0.98631075
MAP2K2	Inverse variance weighted	2	-0.278714	0.256648	0.277488	0.756756	0.457608	1.251465	0.98631075
RDH14	Inverse variance weighted	2	-0.170569	0.153557	0.266661	0.843185	0.62404	1.139287	0.98631075
CTDSP1	Inverse variance weighted	2	0.1654277	0.153027	0.279681	1.179898	0.874148	1.592589	0.98631075
SLC16A14	Inverse variance weighted	2	-0.233554	0.207598	0.260575	0.791714	0.527057	1.189267	0.98631075
VIT	Inverse variance weighted	2	-0.147805	0.134033	0.270136	0.862599	0.663312	1.121761	0.98631075
PRKCE	Inverse variance weighted	2	-0.185861	0.170417	0.275438	0.830389	0.594593	1.159694	0.98631075
EMILIN3	Inverse variance weighted	2	-0.103993	0.095838	0.277878	0.901231	0.746892	1.087464	0.98631075
ADA	Inverse variance weighted	2	-0.065755	0.060044	0.273471	0.936361	0.832401	1.053304	0.98631075
GSTT2B	Inverse variance weighted	3	-0.017347	0.014696	0.237855	0.982803	0.954898	1.011523	0.98631075
ADORA2A	Inverse variance weighted	2	0.1227117	0.113462	0.279465	1.130558	0.905132	1.412128	0.98631075
SLC5A1	Inverse variance weighted	2	-0.070724	0.063483	0.26525	0.931719	0.822711	1.05517	0.98631075

ARSA	Inverse variance weighted	2	-0.147195	0.124386	0.236662	0.863126	0.676386	1.101422	0.98631075
SLC6A1	Inverse variance weighted	2	-0.208342	0.182196	0.252829	0.811929	0.568106	1.160397	0.98631075
PARP9	Inverse variance weighted	2	-0.12989	0.114574	0.256929	0.878192	0.701555	1.099301	0.98631075
PLCH1	Inverse variance weighted	3	-0.113818	0.098372	0.247267	0.89242	0.735925	1.082195	0.98631075
LAMB2	Inverse variance weighted	2	0.2228126	0.203026	0.272441	1.249586	0.839358	1.860311	0.98631075
TKT	Inverse variance weighted	2	-0.073014	0.063423	0.249636	0.929588	0.820926	1.052632	0.98631075
KLKB1	Inverse variance weighted	2	-0.03291	0.030082	0.273948	0.967625	0.912222	1.026393	0.98631075
SPINK2	Inverse variance weighted	2	0.0695419	0.062928	0.269115	1.072017	0.947625	1.212738	0.98631075
ADRA1B	Inverse variance weighted	2	0.1529581	0.141564	0.279925	1.165276	0.882932	1.537909	0.98631075
PELO	Inverse variance weighted	2	0.1129765	0.104414	0.27925	1.119606	0.912402	1.373865	0.98631075
THBS4	Inverse variance weighted	2	-0.053346	0.049221	0.278448	0.948052	0.860864	1.04407	0.98631075
DHFR	Inverse variance weighted	3	0.017704	0.015452	0.251897	1.017862	0.987497	1.04916	0.98631075
DDO	Inverse variance weighted	2	-0.053503	0.046444	0.249323	0.947903	0.865427	1.03824	0.98631075
ULBP1	Inverse variance weighted	2	-0.041682	0.037481	0.266094	0.959174	0.891237	1.03229	0.98631075
MAP3K4	Inverse variance weighted	2	-0.177503	0.159964	0.267154	0.837359	0.611994	1.145713	0.98631075
RPS6KA2	Inverse variance weighted	2	0.1598543	0.135841	0.239287	1.17334	0.89907	1.531279	0.98631075
HLA-DPB1	Inverse variance weighted	5	-0.039533	0.035232	0.261833	0.961238	0.8971	1.029962	0.98631075
NOS3	Inverse variance weighted	2	0.107679	0.098595	0.274774	1.11369	0.917992	1.351108	0.98631075
PSCA	Inverse variance weighted	2	0.0333594	0.029272	0.25444	1.033922	0.976272	1.094976	0.98631075
CA8	Inverse variance weighted	2	0.0886145	0.080588	0.271508	1.092659	0.933011	1.279625	0.98631075
RDH10	Inverse variance weighted	2	-0.052272	0.04706	0.266679	0.949071	0.865447	1.040776	0.98631075
ALDH1B1	Inverse variance weighted	2	0.0748434	0.064487	0.245805	1.077715	0.949755	1.222915	0.98631075
PPM1B	Wald ratio	1	-0.267341	0.248246	0.281515	0.765412	0.470527	1.245105	0.9865693
MORC1	Wald ratio	1	-0.082597	0.076697	0.281515	0.920722	0.792214	1.070076	0.9865693
PLCL2	Wald ratio	1	0.2744914	0.254885	0.281515	1.315861	0.79845	2.168565	0.9865693
OR7E38P	Wald ratio	1	0.0973845	0.090428	0.281515	1.102284	0.92325	1.316036	0.9865693
LACTB	Inverse variance weighted	2	0.1100396	0.102099	0.281133	1.116322	0.913864	1.363634	0.9865693
ESAM	Wald ratio	1	0.2175383	0.202938	0.283746	1.243013	0.835086	1.850207	0.98658634
SUPT16H	Wald ratio	1	-0.278624	0.258898	0.281842	0.756825	0.455635	1.257111	0.98658634
TRPM2	Wald ratio	1	-0.101426	0.094614	0.28372	0.903548	0.75061	1.087647	0.98658634
CCNA2	Wald ratio	1	-0.140674	0.131217	0.283688	0.868772	0.671756	1.12357	0.98658634
MFSD4B	Wald ratio	1	-0.215745	0.201476	0.28425	0.805941	0.543004	1.196198	0.98658634
TAAR6	Wald ratio	1	-0.094914	0.088704	0.284619	0.909451	0.764316	1.082147	0.98658634
TNFRSF10D	Wald ratio	1	-0.216023	0.201192	0.282949	0.805717	0.543156	1.195199	0.98658634
CD48	Inverse variance weighted	2	0.093729	0.08754	0.284302	1.098262	0.925105	1.30383	0.98658634
OR2T33	Inverse variance weighted	2	-0.056067	0.052338	0.284059	0.945476	0.853296	1.047614	0.98658634
BRDT	Inverse variance weighted	2	-0.132553	0.123836	0.284444	0.875856	0.687102	1.116463	0.98658634
HSD17B14	Inverse variance weighted	2	-0.091096	0.084825	0.282856	0.91293	0.773094	1.078058	0.98658634
GPR61	Wald ratio	1	-0.15892	0.150747	0.291784	0.853064	0.634838	1.146306	0.98664924
COPA	Wald ratio	1	0.0886027	0.084004	0.291542	1.092647	0.926774	1.288206	0.98664924
RNPEP	Wald ratio	1	0.0853933	0.081681	0.295813	1.089145	0.928021	1.278244	0.98664924
NID1	Wald ratio	1	-0.151782	0.143191	0.289145	0.859175	0.648926	1.137544	0.98664924
OR2T4	Wald ratio	1	0.1243799	0.118628	0.294415	1.132446	0.89751	1.42888	0.98664924
PDE4B	Wald ratio	1	-0.156844	0.148267	0.290123	0.854837	0.639258	1.143117	0.98664924
HTRA1	Wald ratio	1	0.1951569	0.187699	0.298463	1.215502	0.841363	1.756014	0.98664924
VIM	Wald ratio	1	0.1469801	0.141731	0.299719	1.158331	0.877382	1.529242	0.98664924
BIRC2	Wald ratio	1	-0.256393	0.24562	0.29655	0.773838	0.47816	1.252351	0.98664924
PTPMT1	Wald ratio	1	0.1776589	0.166928	0.287199	1.194418	0.861122	1.656716	0.98664924
OR5B3	Wald ratio	1	-0.085555	0.082019	0.2969	0.918003	0.781678	1.078103	0.98664924
CATSPER1	Wald ratio	1	0.1113836	0.104586	0.286876	1.117824	0.910643	1.37214	0.98664924
KL	Wald ratio	1	0.1026744	0.0988	0.298704	1.108131	0.913042	1.344903	0.98664924
MAP2K5	Wald ratio	1	0.049787	0.046976	0.289222	1.051047	0.958595	1.152416	0.98664924
PDIA2	Wald ratio	1	0.140417	0.135031	0.298393	1.150754	0.883164	1.49942	0.98664924
ABAT	Wald ratio	1	0.1501565	0.145151	0.300911	1.162016	0.874293	1.544427	0.98664924
OR1A1	Wald ratio	1	0.1035668	0.09916	0.29628	1.10912	0.913213	1.347054	0.98664924
DHRS13	Wald ratio	1	-0.11212	0.108105	0.299669	0.893937	0.723246	1.104912	0.98664924
CA10	Wald ratio	1	-0.12964	0.123747	0.294814	0.878411	0.689226	1.119525	0.98664924
ABCA8	Wald ratio	1	0.1477751	0.139818	0.290552	1.159252	0.881378	1.524732	0.98664924
SHBG	Wald ratio	1	-0.105839	0.100457	0.292079	0.899569	0.738794	1.095332	0.98664924
DCXR	Wald ratio	1	0.0992274	0.094065	0.291481	1.104317	0.918383	1.327896	0.98664924
NR1H2	Wald ratio	1	0.3150707	0.304376	0.300606	1.370356	0.754646	2.488421	0.98664924
C3	Wald ratio	1	-0.252395	0.241205	0.29538	0.776938	0.484249	1.246534	0.98664924
KISS1R	Wald ratio	1	-0.083048	0.0781	0.287624	0.920307	0.789683	1.072539	0.98664924
TTL	Wald ratio	1	0.1110674	0.107569	0.301828	1.11747	0.905047	1.379751	0.98664924
TUBA3D	Wald ratio	1	-0.059098	0.055639	0.288156	0.942614	0.845226	1.051223	0.98664924

SCN2A	Wald ratio	1	0.3275756	0.311827	0.293486	1.3876	0.753064	2.556799	0.98664924
CTLA4	Wald ratio	1	0.1203945	0.116546	0.301595	1.127942	0.897595	1.417401	0.98664924
CD40	Wald ratio	1	-0.103797	0.097154	0.285351	0.901408	0.745113	1.090488	0.98664924
BCR	Wald ratio	1	-0.18786	0.175906	0.285538	0.82873	0.587056	1.169896	0.98664924
PTPN23	Wald ratio	1	0.1625026	0.153719	0.290446	1.176451	0.870414	1.590091	0.98664924
ADH7	Wald ratio	1	-0.122971	0.119023	0.301522	0.884289	0.700293	1.116628	0.98664924
FGF5	Wald ratio	1	0.0779592	0.073628	0.28968	1.081079	0.935801	1.248909	0.98664924
HMGCR	Wald ratio	1	0.1768747	0.169461	0.296601	1.193482	0.856185	1.663657	0.98664924
PDE10A	Wald ratio	1	-0.126406	0.121689	0.298917	0.881257	0.694255	1.11863	0.98664924
H1-4	Wald ratio	1	-0.187313	0.180741	0.300033	0.829184	0.581837	1.181682	0.98664924
SENP6	Wald ratio	1	-0.45225	0.427356	0.28994	0.636195	0.275307	1.470154	0.98664924
CRHR2	Wald ratio	1	0.1164429	0.112529	0.30077	1.123493	0.901123	1.400738	0.98664924
DTX2P1	Wald ratio	1	0.1249863	0.119497	0.29559	1.133133	0.896526	1.432184	0.98664924
ROR2	Wald ratio	1	0.0503952	0.048729	0.301048	1.051687	0.955888	1.157086	0.98664924
H3-3A	Inverse variance weighted	4	-0.026562	0.025205	0.291953	0.973788	0.926851	1.023102	0.98664924
JAK1	Inverse variance weighted	3	-0.052067	0.049883	0.296588	0.949265	0.860848	1.046764	0.98664924
RGR	Inverse variance weighted	2	0.0527719	0.050076	0.29196	1.054189	0.955637	1.162905	0.98664924
MCAM	Inverse variance weighted	2	0.275835	0.2622	0.292798	1.31763	0.788143	2.202837	0.98664924
OR52Z1P	Inverse variance weighted	2	-0.05113	0.049252	0.299209	0.950155	0.862722	1.04645	0.98664924
ABCD2	Inverse variance weighted	2	0.2233581	0.21496	0.298773	1.250268	0.8204	1.905376	0.98664924
PDXDC1	Inverse variance weighted	2	0.0796517	0.075224	0.289661	1.08291	0.93446	1.254943	0.98664924
KCNG4	Inverse variance weighted	3	0.0417412	0.039766	0.293869	1.042625	0.964447	1.127139	0.98664924
GLP2R	Inverse variance weighted	3	-0.031815	0.030386	0.295076	0.968685	0.912678	1.028129	0.98664924
POMC	Inverse variance weighted	2	0.0334988	0.032386	0.300972	1.034066	0.970467	1.101834	0.98664924
PRNP	Inverse variance weighted	2	-0.239504	0.224647	0.286362	0.787018	0.506712	1.222385	0.98664924
CBR3	Inverse variance weighted	3	0.0261608	0.024584	0.28726	1.026506	0.978217	1.077178	0.98664924
IL17RC	Inverse variance weighted	3	-0.052336	0.050192	0.297082	0.94901	0.860095	1.047117	0.98664924
IRAK2	Inverse variance weighted	2	0.0777744	0.073754	0.291652	1.080879	0.935397	1.248987	0.98664924
GSK3B	Inverse variance weighted	2	-0.169598	0.158744	0.285354	0.844004	0.618328	1.152048	0.98664924
PPP3CA	Inverse variance weighted	2	-0.210021	0.202271	0.299123	0.810567	0.545271	1.20494	0.98664924
PRKG2	Inverse variance weighted	2	-0.158465	0.152764	0.299587	0.853453	0.632622	1.151369	0.98664924
IFNGR1	Inverse variance weighted	2	0.1491851	0.141899	0.2931	1.160888	0.879029	1.533124	0.98664924
TAB2	Inverse variance weighted	2	-0.126432	0.121857	0.299484	0.881234	0.694008	1.11897	0.98664924
TRIM35	Inverse variance weighted	3	0.0513773	0.048943	0.293842	1.05272	0.956426	1.158709	0.98664924
CHST3	Wald ratio	1	-0.05962	0.057917	0.303286	0.942122	0.841022	1.055375	0.98772564
VKORC1	Wald ratio	1	0.2091996	0.203222	0.303286	1.232691	0.82769	1.835865	0.98772564
TNK1	Wald ratio	1	0.0674024	0.065432	0.302953	1.069726	0.94097	1.216099	0.98772564
PSMA7	Wald ratio	1	0.2655317	0.257872	0.303149	1.304124	0.786708	2.161843	0.98772564
CHI3L2	Wald ratio	1	0.1866833	0.182603	0.306617	1.205245	0.842638	1.723892	0.98799695
CYP4A22	Wald ratio	1	-0.064129	0.062423	0.304269	0.937884	0.829877	1.059949	0.98799695
MFAP1	Wald ratio	1	-0.328206	0.320953	0.306498	0.720215	0.383938	1.351022	0.98799695
SLC25A48	Wald ratio	1	0.0540128	0.052776	0.306101	1.055498	0.951774	1.170526	0.98799695
HLA-B	Wald ratio	1	-0.09324	0.09072	0.304054	0.910974	0.762576	1.088251	0.98799695
KCNK5	Wald ratio	1	-0.099459	0.096864	0.304521	0.905327	0.748778	1.094606	0.98799695
PTGFRN	Inverse variance weighted	2	0.1133622	0.11039	0.304456	1.120038	0.902125	1.390588	0.98799695
OR2W3	Inverse variance weighted	3	-0.041582	0.040697	0.306895	0.959271	0.885726	1.038922	0.98799695
PKD2L1	Inverse variance weighted	2	0.0302785	0.029563	0.305738	1.030742	0.972714	1.09223	0.98799695
SPRYD3	Inverse variance weighted	2	-0.224767	0.220046	0.307038	0.798702	0.518893	1.229395	0.98799695
CHRNA7	Inverse variance weighted	2	0.0709291	0.069209	0.305432	1.073505	0.937329	1.229465	0.98799695
STK39	Inverse variance weighted	2	0.1267878	0.124084	0.30688	1.135176	0.890104	1.447723	0.98799695
MMRN1	Inverse variance weighted	2	0.0355293	0.034658	0.305296	1.036168	0.968119	1.109	0.98799695
YES1	Wald ratio	1	0.1112653	0.109153	0.308035	1.117691	0.902421	1.384314	0.98847747
SRPK2	Wald ratio	1	-0.251491	0.246654	0.307914	0.777641	0.479537	1.261059	0.98847747
MMRN2	Inverse variance weighted	2	-0.090333	0.088587	0.307863	0.913627	0.768002	1.086864	0.98847747
AGRN	Wald ratio	1	-0.17676	0.215262	0.411568	0.837981	0.54954	1.277818	0.98895853
COL11A1	Wald ratio	1	-0.097778	0.109122	0.370232	0.906851	0.732233	1.12311	0.98895853
AMY2A	Wald ratio	1	0.0591836	0.063285	0.349693	1.06097	0.937203	1.201082	0.98895853
AMY1B	Wald ratio	1	-0.062717	0.067064	0.349693	0.939209	0.823525	1.071144	0.98895853
NPPA	Wald ratio	1	0.0837612	0.082986	0.312809	1.087369	0.924141	1.279428	0.98895853
TAFA3	Wald ratio	1	0.1176603	0.130177	0.366077	1.124862	0.871545	1.451805	0.98895853
PTPN22	Wald ratio	1	0.0881769	0.108217	0.415178	1.092181	0.883443	1.35024	0.98895853
ATP1A1	Wald ratio	1	-0.355596	0.362181	0.326189	0.700756	0.344565	1.425153	0.98895853
AURKAIP1	Wald ratio	1	0.2573275	0.256054	0.314909	1.293469	0.783067	2.13655	0.98895853
CLCN6	Wald ratio	1	0.1537549	0.193999	0.428038	1.166205	0.797332	1.705731	0.98895853
CA14	Wald ratio	1	0.1521407	0.187426	0.416943	1.164324	0.806368	1.681181	0.98895853

LMNA	Wald ratio	1	-0.186599	0.206569	0.366354	0.829777	0.553511	1.243931	0.98895853
CD1D	Wald ratio	1	0.0798353	0.096115	0.406185	1.083109	0.897135	1.307635	0.98895853
NECTIN4	Wald ratio	1	-0.105439	0.119776	0.378697	0.899929	0.711628	1.138057	0.98895853
RXRG	Wald ratio	1	-0.094158	0.113162	0.405374	0.910139	0.729092	1.136144	0.98895853
STYXL2	Wald ratio	1	-0.152203	0.157737	0.334589	0.858814	0.630421	1.169952	0.98895853
SERPINC1	Wald ratio	1	-0.080229	0.091828	0.38229	0.922905	0.770888	1.1049	0.98895853
ANGPTL1	Wald ratio	1	-0.07851	0.080019	0.326528	0.924493	0.790296	1.081478	0.98895853
MR1	Wald ratio	1	0.0798627	0.083595	0.339397	1.083138	0.919447	1.275972	0.98895853
LAD1	Wald ratio	1	0.0946261	0.103806	0.361998	1.099248	0.896879	1.347278	0.98895853
CNTN2	Wald ratio	1	-0.339812	0.365762	0.352861	0.711904	0.347599	1.458022	0.98895853
PLA2G2F	Wald ratio	1	-0.095662	0.103561	0.355628	0.908771	0.741826	1.113287	0.98895853
IL20	Wald ratio	1	0.0850267	0.093583	0.363576	1.088746	0.90629	1.307935	0.98895853
CR1	Wald ratio	1	0.0239225	0.026785	0.371788	1.024211	0.971828	1.079417	0.98895853
DDOST	Wald ratio	1	0.1432506	0.169003	0.39665	1.154019	0.828618	1.607206	0.98895853
KCNK2	Wald ratio	1	0.1446498	0.182104	0.427006	1.155635	0.808743	1.651317	0.98895853
EPRS1	Wald ratio	1	-0.136966	0.166315	0.410207	0.872	0.629428	1.208056	0.98895853
GGPS1	Wald ratio	1	-0.042274	0.051121	0.408281	0.958608	0.867212	1.059635	0.98895853
OR2T2	Wald ratio	1	-0.104304	0.11558	0.366824	0.900952	0.71832	1.130017	0.98895853
SLC9A1	Wald ratio	1	0.2720445	0.280028	0.331304	1.312645	0.758199	2.272542	0.98895853
NRDC	Wald ratio	1	0.1056162	0.134334	0.431737	1.111395	0.854125	1.446157	0.98895853
COL24A1	Wald ratio	1	-0.135707	0.172074	0.430311	0.873098	0.623148	1.223306	0.98895853
CRTAC1	Wald ratio	1	-0.120468	0.124385	0.332793	0.886506	0.694708	1.131255	0.98895853
VWA2	Wald ratio	1	0.1431262	0.177582	0.42026	1.153875	0.8147	1.634256	0.98895853
MMP21	Wald ratio	1	0.0590838	0.068381	0.387569	1.060864	0.927796	1.213017	0.98895853
PRAP1	Wald ratio	1	-0.069769	0.086958	0.422365	0.93261	0.786466	1.10591	0.98895853
SYT15	Wald ratio	1	-0.155003	0.158963	0.329516	0.856413	0.62715	1.169486	0.98895853
PRKG1	Wald ratio	1	-0.040527	0.0513	0.429528	0.960283	0.868425	1.061858	0.98895853
AIFM2	Wald ratio	1	0.0299343	0.031156	0.336661	1.030387	0.969348	1.095269	0.98895853
NPFFR1	Wald ratio	1	-0.077864	0.081644	0.340234	0.92509	0.788293	1.085627	0.98895853
ITIH5	Wald ratio	1	0.1516749	0.185982	0.414767	1.163782	0.808277	1.675649	0.98895853
MMP7	Wald ratio	1	-0.085198	0.106375	0.423175	0.91833	0.745506	1.131219	0.98895853
MICAL2	Wald ratio	1	-0.222898	0.261329	0.393692	0.800197	0.479457	1.3355	0.98895853
SIDT2	Wald ratio	1	0.0755893	0.096415	0.43304	1.07852	0.892808	1.302861	0.98895853
APOA1	Wald ratio	1	0.1044719	0.111859	0.350323	1.110124	0.89157	1.382254	0.98895853
IL10RA	Wald ratio	1	-0.106923	0.125972	0.396003	0.898595	0.701995	1.150254	0.98895853
THY1	Wald ratio	1	0.2706416	0.335179	0.419405	1.310805	0.67956	2.528416	0.98895853
VWA5A	Wald ratio	1	-0.050984	0.058122	0.380382	0.950294	0.847977	1.064957	0.98895853
PATE2	Wald ratio	1	0.0514912	0.058449	0.378344	1.05284	0.938878	1.180635	0.98895853
ST14	Wald ratio	1	-0.193506	0.241882	0.423711	0.824065	0.512941	1.323902	0.98895853
TSG101	Wald ratio	1	0.1474662	0.167707	0.379233	1.158894	0.834236	1.609899	0.98895853
BDNF	Wald ratio	1	0.2152765	0.225431	0.3396	1.240205	0.797265	1.929231	0.98895853
CD59	Wald ratio	1	-0.064155	0.069502	0.355967	0.937859	0.818421	1.074728	0.98895853
OR52P2P	Wald ratio	1	-0.074528	0.075503	0.323595	0.928181	0.800504	1.076222	0.98895853
OR52A5	Wald ratio	1	-0.062921	0.069603	0.365995	0.939018	0.81927	1.076269	0.98895853
OR52A1	Wald ratio	1	-0.082015	0.089471	0.359317	0.921258	0.773075	1.097844	0.98895853
OR52B2	Wald ratio	1	0.1065747	0.10762	0.322032	1.112461	0.900901	1.373702	0.98895853
OR52N2	Wald ratio	1	0.0660134	0.079766	0.407905	1.068241	0.913631	1.249015	0.98895853
LRRC55	Wald ratio	1	0.2027127	0.227348	0.372585	1.224721	0.784358	1.912315	0.98895853
OR51B4	Wald ratio	1	-0.072526	0.081592	0.374063	0.930041	0.792592	1.091327	0.98895853
FEN1	Wald ratio	1	0.1659449	0.207721	0.424358	1.180508	0.785693	1.773719	0.98895853
NRXN2	Wald ratio	1	-0.308572	0.351847	0.380484	0.734495	0.368545	1.463818	0.98895853
SCT	Wald ratio	1	0.0974256	0.115384	0.398469	1.102329	0.879213	1.382065	0.98895853
FLRT1	Wald ratio	1	-0.272306	0.270228	0.313602	0.761621	0.448453	1.293485	0.98895853
CST6	Wald ratio	1	0.0707231	0.071225	0.320731	1.073284	0.933441	1.234077	0.98895853
OR5P2	Wald ratio	1	-0.10615	0.10528	0.313328	0.89929	0.731617	1.10539	0.98895853
OR10A2	Wald ratio	1	0.1145285	0.133407	0.390622	1.121345	0.863338	1.456456	0.98895853
PPME1	Wald ratio	1	0.2743174	0.289708	0.343702	1.315632	0.745641	2.321342	0.98895853
OR2AT4	Wald ratio	1	0.0764861	0.077681	0.324813	1.079487	0.92703	1.257017	0.98895853
PRCP	Wald ratio	1	0.2856542	0.292937	0.329492	1.330632	0.749385	2.362714	0.98895853
ENDOD1	Wald ratio	1	0.1090132	0.119233	0.360567	1.115177	0.882776	1.40876	0.98895853
MTMR2	Wald ratio	1	-0.097958	0.121035	0.41832	0.906687	0.715205	1.149434	0.98895853
TXNRD1	Wald ratio	1	0.11959	0.132878	0.36812	1.127035	0.868619	1.462329	0.98895853
KLRB1	Wald ratio	1	0.0735193	0.090818	0.418214	1.076289	0.900789	1.285982	0.98895853
SELPLG	Wald ratio	1	-0.161844	0.188034	0.389395	0.850574	0.588375	1.229618	0.98895853
PLA2G1B	Wald ratio	1	0.0624533	0.075997	0.411201	1.064445	0.917134	1.235416	0.98895853

PLCZ1	Wald ratio	1	-0.144732	0.156031	0.353622	0.865254	0.637276	1.174788	0.98895853
NTF3	Wald ratio	1	-0.091457	0.110379	0.407347	0.912601	0.735063	1.133019	0.98895853
FKBP11	Wald ratio	1	-0.106794	0.126086	0.396998	0.898711	0.70193	1.150658	0.98895853
KCNA6	Wald ratio	1	-0.243702	0.258934	0.346614	0.783721	0.471794	1.301878	0.98895853
CACNB3	Wald ratio	1	0.1926426	0.228391	0.398961	1.212449	0.774914	1.897028	0.98895853
TUBA1C	Wald ratio	1	0.0415648	0.04195	0.321771	1.042441	0.960159	1.131774	0.98895853
DCD	Wald ratio	1	0.4976083	0.633086	0.431865	1.644783	0.47557	5.68856	0.98895853
ERBB3	Wald ratio	1	0.150788	0.15688	0.336469	1.16275	0.854963	1.581342	0.98895853
IFNG	Wald ratio	1	-0.066393	0.08346	0.426319	0.935763	0.794554	1.102068	0.98895853
SACS	Wald ratio	1	-0.166063	0.16734	0.321019	0.846993	0.61015	1.175771	0.98895853
CSNK1A1L	Wald ratio	1	0.136308	0.139369	0.328056	1.146035	0.872096	1.506021	0.98895853
KLHL1	Wald ratio	1	-0.155015	0.152222	0.308512	0.856402	0.635482	1.154123	0.98895853
PARP2	Wald ratio	1	0.1525194	0.167327	0.36203	1.164765	0.839086	1.616851	0.98895853
ANG	Wald ratio	1	-0.050088	0.062829	0.425333	0.951146	0.840941	1.075793	0.98895853
SLC22A17	Wald ratio	1	0.207026	0.246591	0.401159	1.230015	0.758591	1.994402	0.98895853
BCL2L2	Wald ratio	1	0.1768362	0.182089	0.331472	1.193436	0.835222	1.705281	0.98895853
NID2	Wald ratio	1	0.1123777	0.138103	0.415802	1.118935	0.853591	1.466764	0.98895853
RDH12	Wald ratio	1	0.1183223	0.139203	0.395325	1.125607	0.856831	1.478694	0.98895853
MAP3K9	Wald ratio	1	-0.141192	0.175656	0.421514	0.868323	0.615403	1.225187	0.98895853
PSEN1	Wald ratio	1	0.1986106	0.247207	0.421733	1.219707	0.751327	1.980077	0.98895853
MLH3	Wald ratio	1	0.057372	0.066604	0.389025	1.05905	0.929441	1.206732	0.98895853
VRK1	Wald ratio	1	-0.055298	0.06431	0.389859	0.946203	0.834147	1.073312	0.98895853
ALDH1A3	Wald ratio	1	-0.149825	0.157242	0.340676	0.860859	0.632536	1.171599	0.98895853
GREM1	Wald ratio	1	0.1439711	0.15962	0.367078	1.154851	0.844607	1.579055	0.98895853
TGM7	Wald ratio	1	-0.116903	0.139385	0.401632	0.889671	0.676991	1.169166	0.98895853
EPB42	Wald ratio	1	-0.174436	0.211815	0.410207	0.839931	0.554553	1.272166	0.98895853
BCL2L10	Wald ratio	1	-0.122733	0.133199	0.356827	0.8845	0.681266	1.148362	0.98895853
CILP	Wald ratio	1	0.0878493	0.090472	0.331541	1.091824	0.914411	1.303657	0.98895853
PSMA4	Wald ratio	1	0.2236449	0.237623	0.346614	1.250627	0.784981	1.99249	0.98895853
FES	Wald ratio	1	0.1429588	0.15057	0.342392	1.153682	0.858852	1.549723	0.98895853
SSTR5	Wald ratio	1	0.0858824	0.085882	0.317311	1.089678	0.92086	1.289445	0.98895853
TPSD1	Wald ratio	1	-0.027808	0.033662	0.408755	0.972575	0.910478	1.038908	0.98895853
PRSS21	Wald ratio	1	0.0998183	0.105984	0.346281	1.10497	0.897709	1.360083	0.98895853
SEZ6L2	Wald ratio	1	-0.346842	0.365291	0.342369	0.706917	0.345483	1.446473	0.98895853
PRSS36	Wald ratio	1	-0.050257	0.06095	0.409621	0.950985	0.843903	1.071655	0.98895853
TRIM72	Wald ratio	1	-0.029094	0.035754	0.415802	0.971326	0.905588	1.041835	0.98895853
OR2C1	Wald ratio	1	0.0397686	0.046755	0.395006	1.04057	0.949451	1.140433	0.98895853
ADGRG3	Wald ratio	1	0.0958549	0.099741	0.336533	1.100599	0.905166	1.338229	0.98895853
RSPRY1	Wald ratio	1	-0.189368	0.189945	0.318784	0.827482	0.570262	1.200724	0.98895853
CSNK2A2	Wald ratio	1	0.2427037	0.258983	0.348685	1.274691	0.767281	2.117656	0.98895853
NAE1	Wald ratio	1	-0.141925	0.170648	0.405588	0.867687	0.621019	1.212331	0.98895853
NQO1	Wald ratio	1	-0.148689	0.147154	0.312287	0.861837	0.6459	1.149966	0.98895853
OR1D4	Wald ratio	1	-0.075946	0.087504	0.385436	0.926866	0.780787	1.100275	0.98895853
CDK12	Wald ratio	1	0.3341236	0.359297	0.352404	1.396716	0.690666	2.824543	0.98895853
ERBB2	Wald ratio	1	-0.10451	0.125224	0.403951	0.900766	0.704725	1.151342	0.98895853
CNTNAP1	Wald ratio	1	0.2370786	0.239403	0.322032	1.267541	0.792826	2.026496	0.98895853
CD300LG	Wald ratio	1	-0.075008	0.086774	0.387364	0.927736	0.782639	1.099734	0.98895853
ADAM11	Wald ratio	1	0.1801664	0.218319	0.409234	1.197417	0.780563	1.836887	0.98895853
CHRNE	Wald ratio	1	0.0233171	0.026626	0.381172	1.023591	0.971544	1.078427	0.98895853
PSMB6	Wald ratio	1	0.1184451	0.120659	0.326272	1.125745	0.888655	1.426091	0.98895853
SLC25A11	Wald ratio	1	-0.318374	0.331331	0.336606	0.727331	0.379924	1.392409	0.98895853
AIPL1	Wald ratio	1	-0.175193	0.203077	0.388306	0.839295	0.563705	1.249617	0.98895853
ERN1	Wald ratio	1	-0.107325	0.110825	0.332834	0.898234	0.722859	1.116157	0.98895853
EFNB3	Wald ratio	1	0.3104206	0.390765	0.426967	1.363999	0.634145	2.933862	0.98895853
GALK1	Wald ratio	1	-0.136938	0.14902	0.358138	0.872025	0.651148	1.167824	0.98895853
GALR2	Wald ratio	1	-0.076156	0.092936	0.412533	0.926671	0.772355	1.111821	0.98895853
TK1	Wald ratio	1	0.1378204	0.142595	0.333785	1.147769	0.867912	1.517867	0.98895853
TIMP2	Wald ratio	1	0.2404558	0.303183	0.427718	1.271829	0.702027	2.304112	0.98895853
PDE6G	Wald ratio	1	-0.096805	0.116468	0.405878	0.907733	0.722468	1.140507	0.98895853
MC2R	Wald ratio	1	-0.076342	0.078886	0.333173	0.9265	0.793772	1.081421	0.98895853
HRH4	Wald ratio	1	0.0818733	0.088768	0.356356	1.085318	0.912003	1.29157	0.98895853
TWSG1	Wald ratio	1	0.0761962	0.0964	0.429283	1.079174	0.893376	1.303613	0.98895853
S1PR2	Wald ratio	1	-0.083342	0.105816	0.430925	0.920037	0.747709	1.132081	0.98895853
MAST1	Wald ratio	1	0.2772763	0.325498	0.394296	1.319531	0.697188	2.497407	0.98895853
OR7C2	Wald ratio	1	0.1947922	0.21538	0.365777	1.215059	0.79664	1.853243	0.98895853

OR10H5	Wald ratio	1	0.041721	0.052538	0.427127	1.042604	0.940586	1.155686	0.98895853
GDF15	Wald ratio	1	-0.067232	0.085325	0.430721	0.934978	0.790991	1.105175	0.98895853
IL12RB1	Wald ratio	1	-0.054561	0.061381	0.374063	0.946901	0.839568	1.067955	0.98895853
SLC7A10	Wald ratio	1	0.3013783	0.372124	0.418006	1.351721	0.651821	2.803145	0.98895853
ATP4A	Wald ratio	1	0.073281	0.091912	0.425278	1.076033	0.898646	1.288435	0.98895853
SPINT2	Wald ratio	1	-0.161657	0.197755	0.413665	0.850733	0.577379	1.253504	0.98895853
IFNL1	Wald ratio	1	-0.083673	0.084174	0.3202	0.919732	0.779849	1.084705	0.98895853
MAP3K10	Wald ratio	1	-0.235259	0.289113	0.415802	0.790366	0.448467	1.392921	0.98895853
LMTK3	Wald ratio	1	0.3674976	0.413028	0.373593	1.444116	0.642725	3.244733	0.98895853
LIG1	Wald ratio	1	0.0513469	0.065467	0.432856	1.052688	0.925919	1.196814	0.98895853
CA11	Wald ratio	1	-0.176458	0.192213	0.358602	0.838234	0.575108	1.221745	0.98895853
CD37	Wald ratio	1	-0.087405	0.100618	0.385018	0.916306	0.752303	1.116061	0.98895853
SIGLEC5	Wald ratio	1	-0.065205	0.074905	0.384025	0.936876	0.80895	1.085031	0.98895853
MBOAT7	Wald ratio	1	-0.339379	0.423566	0.422991	0.712212	0.310501	1.63364	0.98895853
LILRA5	Wald ratio	1	0.0893452	0.107889	0.4076	1.093458	0.885045	1.350948	0.98895853
A1BG	Wald ratio	1	0.1229792	0.142083	0.386739	1.130861	0.855984	1.494006	0.98895853
CD70	Wald ratio	1	-0.101779	0.125043	0.415672	0.903229	0.706902	1.154081	0.98895853
PSPN	Wald ratio	1	-0.206562	0.205027	0.313699	0.813376	0.544213	1.215663	0.98895853
ELAVL1	Wald ratio	1	-0.178156	0.196135	0.363702	0.836812	0.569737	1.229084	0.98895853
GYPC	Wald ratio	1	0.0810787	0.088376	0.358917	1.084456	0.911979	1.289552	0.98895853
OR7E90P	Wald ratio	1	-0.061875	0.076607	0.419268	0.94	0.808944	1.092289	0.98895853
CD302	Wald ratio	1	0.1058852	0.112147	0.345087	1.111694	0.892326	1.384991	0.98895853
DHRS9	Wald ratio	1	-0.11406	0.123862	0.357122	0.892205	0.699893	1.137359	0.98895853
MYO3B	Wald ratio	1	0.0928706	0.109813	0.397712	1.09732	0.884827	1.360843	0.98895853
ITGAV	Wald ratio	1	0.064331	0.074228	0.386125	1.066445	0.92205	1.233453	0.98895853
STK17B	Wald ratio	1	0.3022379	0.341163	0.375668	1.352883	0.693197	2.640363	0.98895853
CFLAR	Wald ratio	1	-0.179577	0.176771	0.30969	0.835623	0.590935	1.18163	0.98895853
BMPR2	Wald ratio	1	-0.211414	0.254658	0.406432	0.809439	0.491376	1.333378	0.98895853
CPO	Wald ratio	1	-0.071422	0.084408	0.397467	0.931069	0.789101	1.098579	0.98895853
FN1	Wald ratio	1	-0.108473	0.134067	0.418463	0.897204	0.689875	1.16684	0.98895853
EPHA4	Wald ratio	1	-0.222368	0.273487	0.41617	0.800621	0.468415	1.368433	0.98895853
PDE6D	Wald ratio	1	0.1074414	0.119746	0.369589	1.113426	0.880504	1.407963	0.98895853
ATG4B	Wald ratio	1	0.1704671	0.169214	0.313739	1.185859	0.851129	1.65223	0.98895853
GFPT1	Wald ratio	1	0.1318759	0.137371	0.337055	1.140967	0.871647	1.4935	0.98895853
DUSP11	Wald ratio	1	-0.113507	0.133056	0.393615	0.892698	0.687773	1.158681	0.98895853
EIF2AK3	Wald ratio	1	-0.099151	0.103873	0.339808	0.905606	0.73879	1.110088	0.98895853
DEFB127	Wald ratio	1	0.1846117	0.221707	0.405023	1.202751	0.778853	1.857361	0.98895853
PTPRA	Wald ratio	1	0.1325185	0.166632	0.426453	1.1417	0.823592	1.582676	0.98895853
CDC25B	Wald ratio	1	0.086823	0.104188	0.404657	1.090704	0.889243	1.337806	0.98895853
ASIP	Wald ratio	1	0.0704065	0.086109	0.413559	1.072944	0.906317	1.270206	0.98895853
GNRH2	Wald ratio	1	0.0965982	0.105099	0.358034	1.101418	0.896376	1.353362	0.98895853
HNF4A	Wald ratio	1	0.0339986	0.033668	0.312589	1.034583	0.968515	1.105159	0.98895853
SEMG1	Wald ratio	1	-0.157202	0.167726	0.348627	0.854532	0.615116	1.187132	0.98895853
SDC4	Wald ratio	1	0.1097141	0.12164	0.367078	1.115959	0.879238	1.416413	0.98895853
PIGT	Wald ratio	1	-0.26212	0.279741	0.348755	0.769419	0.444675	1.331323	0.98895853
KCNB1	Wald ratio	1	0.2632957	0.331862	0.427552	1.301211	0.678986	2.493646	0.98895853
LAMA5	Wald ratio	1	-0.150393	0.158827	0.343688	0.86037	0.630216	1.174575	0.98895853
SYNJ1	Wald ratio	1	-0.133398	0.140419	0.342112	0.875117	0.664567	1.152373	0.98895853
CBR1	Wald ratio	1	0.0767653	0.095209	0.42008	1.079789	0.895974	1.301314	0.98895853
TSPEAR	Wald ratio	1	-0.092701	0.101714	0.362089	0.911466	0.746724	1.112553	0.98895853
SLC19A1	Wald ratio	1	0.121788	0.126759	0.336661	1.129515	0.881034	1.448076	0.98895853
S100B	Wald ratio	1	0.0741319	0.078068	0.342326	1.076949	0.924149	1.255012	0.98895853
P2RX6	Wald ratio	1	-0.056193	0.062178	0.366132	0.945356	0.83689	1.067881	0.98895853
SNAP29	Wald ratio	1	-0.072983	0.079239	0.357023	0.929616	0.795892	1.085809	0.98895853
IGLV7-46	Wald ratio	1	-0.158688	0.196353	0.418989	0.853263	0.580689	1.253782	0.98895853
IGLV1-40	Wald ratio	1	-0.109011	0.131282	0.406337	0.89672	0.693278	1.159863	0.98895853
IGLV2-11	Wald ratio	1	-0.113451	0.113451	0.317311	0.892748	0.714755	1.115065	0.98895853
IGLV7-43	Wald ratio	1	-0.201212	0.199491	0.313153	0.817739	0.553102	1.208995	0.98895853
DUSP18	Wald ratio	1	0.0474144	0.058093	0.414399	1.048556	0.935711	1.175011	0.98895853
KCNJ4	Wald ratio	1	0.1380545	0.155868	0.375772	1.148038	0.845822	1.558238	0.98895853
ALCAM	Wald ratio	1	-0.174811	0.213865	0.413705	0.839616	0.552122	1.276809	0.98895853
MYLK	Wald ratio	1	0.1955679	0.236969	0.409208	1.216001	0.764226	1.934846	0.98895853
MUC13	Wald ratio	1	0.0579482	0.062727	0.355585	1.05966	0.93707	1.198288	0.98895853
SLC25A36	Wald ratio	1	0.1699675	0.197064	0.388412	1.185266	0.805512	1.744054	0.98895853
PLSCR1	Wald ratio	1	0.0960115	0.104813	0.359651	1.100772	0.896353	1.35181	0.98895853

AGTR1	Wald ratio	1	-0.102925	0.125519	0.412216	0.902194	0.705434	1.153835	0.98895853
CAPN7	Wald ratio	1	-0.189389	0.198322	0.3396	0.827465	0.560963	1.220576	0.98895853
VN2R1P	Wald ratio	1	-0.094844	0.103132	0.357759	0.909515	0.743057	1.113261	0.98895853
TFRC	Wald ratio	1	0.1287357	0.132449	0.331069	1.137389	0.877336	1.474525	0.98895853
THRB	Wald ratio	1	0.1997603	0.204805	0.329377	1.22111	0.817375	1.824266	0.98895853
NR1D2	Wald ratio	1	0.1517338	0.189667	0.423711	1.16385	0.802508	1.687894	0.98895853
SLC22A14	Wald ratio	1	0.0976338	0.117858	0.407443	1.102559	0.875143	1.389071	0.98895853
NKTR	Wald ratio	1	0.1671821	0.204155	0.412845	1.18197	0.792184	1.763545	0.98895853
CDCP1	Wald ratio	1	-0.125488	0.156678	0.423175	0.882067	0.648835	1.199137	0.98895853
PTH1R	Wald ratio	1	0.0538741	0.058546	0.357466	1.055352	0.94094	1.183675	0.98895853
CDC25A	Wald ratio	1	-0.053145	0.058766	0.365811	0.948243	0.845079	1.064001	0.98895853
PXK	Wald ratio	1	-0.080506	0.082084	0.326707	0.92265	0.785534	1.083698	0.98895853
ADH1B	Wald ratio	1	0.1074043	0.107875	0.319428	1.113384	0.901197	1.375531	0.98895853
CYP2U1	Wald ratio	1	0.0956073	0.103017	0.353369	1.100327	0.89915	1.346516	0.98895853
VEGFC	Wald ratio	1	-0.092883	0.096525	0.335917	0.9113	0.75422	1.101097	0.98895853
ENPP6	Wald ratio	1	-0.170224	0.170224	0.317311	0.843476	0.604192	1.177525	0.98895853
MFSD10	Wald ratio	1	0.2506604	0.308736	0.416853	1.284874	0.701551	2.353214	0.98895853
TLR10	Wald ratio	1	-0.165964	0.170449	0.330213	0.847077	0.606503	1.183075	0.98895853
KLHL5	Wald ratio	1	-0.185381	0.187234	0.322126	0.830788	0.57559	1.199133	0.98895853
UCHL1	Wald ratio	1	-0.104194	0.126876	0.411516	0.90105	0.702668	1.155441	0.98895853
PF4V1	Wald ratio	1	0.1332848	0.162526	0.412168	1.142575	0.830883	1.571194	0.98895853
PF4	Wald ratio	1	0.1069799	0.117633	0.363121	1.112912	0.883749	1.401498	0.98895853
GPR78	Wald ratio	1	0.1088198	0.116044	0.348376	1.114961	0.888139	1.399712	0.98895853
SLC12A7	Wald ratio	1	-0.168245	0.199334	0.398649	0.845147	0.571815	1.249133	0.98895853
HBEGF	Wald ratio	1	0.0791856	0.085277	0.353111	1.082405	0.9158	1.279319	0.98895853
NDFIP1	Wald ratio	1	-0.211884	0.247577	0.392093	0.809059	0.49801	1.314384	0.98895853
PCYOX1L	Wald ratio	1	-0.14029	0.163671	0.391366	0.869107	0.630599	1.197823	0.98895853
SLC6A7	Wald ratio	1	-0.104706	0.130559	0.422564	0.90059	0.697257	1.163217	0.98895853
TRIM41	Wald ratio	1	0.2648317	0.284861	0.352533	1.303212	0.745652	2.277684	0.98895853
RXFP3	Wald ratio	1	-0.145771	0.173488	0.400776	0.864356	0.6152	1.214419	0.98895853
ITGA2	Wald ratio	1	-0.095182	0.111223	0.392125	0.909208	0.731119	1.130677	0.98895853
AGGF1	Wald ratio	1	-0.143516	0.158551	0.365374	0.866307	0.634908	1.182043	0.98895853
CCN6	Wald ratio	1	0.181148	0.205073	0.377056	1.198593	0.801881	1.791568	0.98895853
SERPINB6	Wald ratio	1	-0.133754	0.164038	0.414852	0.874805	0.634278	1.206544	0.98895853
H3C4	Wald ratio	1	-0.153467	0.184526	0.405588	0.857729	0.597418	1.231464	0.98895853
GPX5	Wald ratio	1	0.0873807	0.098665	0.375817	1.091312	0.899422	1.324141	0.98895853
AGER	Wald ratio	1	0.1636613	0.175404	0.350792	1.177815	0.835161	1.661055	0.98895853
MAPK13	Wald ratio	1	-0.0549	0.062743	0.381574	0.94658	0.837046	1.070447	0.98895853
MAPK14	Wald ratio	1	-0.151355	0.154229	0.326412	0.859542	0.635308	1.16292	0.98895853
GLP1R	Wald ratio	1	0.1386759	0.137368	0.312723	1.148752	0.8776	1.503682	0.98895853
ADGRF1	Wald ratio	1	-0.187271	0.191988	0.329347	0.829219	0.569174	1.208072	0.98895853
GCLC	Wald ratio	1	-0.058086	0.061716	0.346614	0.943569	0.836065	1.064897	0.98895853
HCRTR2	Wald ratio	1	-0.075524	0.085375	0.376364	0.927258	0.784383	1.096157	0.98895853
TPBG	Wald ratio	1	0.1201568	0.122536	0.326799	1.127674	0.886908	1.433799	0.98895853
MAP3K7	Wald ratio	1	0.0750466	0.089381	0.40112	1.077934	0.90471	1.284326	0.98895853
TAS2R16	Wald ratio	1	0.1136587	0.142714	0.425795	1.12037	0.846994	1.48198	0.98895853
SMO	Wald ratio	1	-0.103651	0.130349	0.426508	0.90154	0.698279	1.163966	0.98895853
AGBL3	Wald ratio	1	-0.12303	0.147993	0.40579	0.884237	0.661598	1.181797	0.98895853
SVOPL	Wald ratio	1	-0.083492	0.099651	0.402122	0.919899	0.756685	1.118317	0.98895853
MGAM2	Wald ratio	1	-0.08993	0.112877	0.425623	0.913995	0.73259	1.140321	0.98895853
CNTNAP2	Wald ratio	1	-0.177874	0.22584	0.430925	0.837048	0.537664	1.303135	0.98895853
AGR2	Wald ratio	1	-0.052231	0.05389	0.33243	0.949109	0.853974	1.054843	0.98895853
STK31	Wald ratio	1	0.0717167	0.081414	0.378376	1.074351	0.915895	1.260221	0.98895853
NPSR1	Wald ratio	1	-0.017717	0.018958	0.350005	0.982439	0.946604	1.01963	0.98895853
EGFR	Wald ratio	1	0.0437726	0.047627	0.358056	1.044745	0.951633	1.146967	0.98895853
GUSBP12	Wald ratio	1	0.086258	0.094345	0.360567	1.090088	0.906053	1.311503	0.98895853
LIMK1	Wald ratio	1	-0.128216	0.163091	0.431772	0.879663	0.638985	1.210995	0.98895853
TNFRSF11B	Wald ratio	1	-0.096335	0.121037	0.426079	0.90816	0.716364	1.151305	0.98895853
COL14A1	Wald ratio	1	-0.086878	0.097497	0.372881	0.916789	0.757318	1.10984	0.98895853
DGAT1	Wald ratio	1	-0.216711	0.228037	0.341944	0.805163	0.514961	1.258905	0.98895853
FGF20	Wald ratio	1	0.0768601	0.093107	0.409086	1.079891	0.899758	1.296087	0.98895853
SFTPC	Wald ratio	1	0.1486986	0.173412	0.391175	1.160323	0.825977	1.630009	0.98895853
GNRH1	Wald ratio	1	-0.085439	0.096831	0.377586	0.918109	0.759399	1.109989	0.98895853
IDO1	Wald ratio	1	-0.063433	0.073356	0.387185	0.938537	0.812849	1.08366	0.98895853
ADAM18	Wald ratio	1	0.0541004	0.054664	0.322325	1.055591	0.948341	1.174969	0.98895853

POLB	Wald ratio	1	-0.192396	0.197933	0.331037	0.82498	0.559706	1.215982	0.98895853
LYN	Wald ratio	1	0.1308757	0.147763	0.375772	1.139826	0.853219	1.522709	0.98895853
DEFA4	Wald ratio	1	0.1126882	0.143421	0.432035	1.119283	0.845001	1.482595	0.98895853
SGK3	Wald ratio	1	-0.149905	0.148724	0.313485	0.86079	0.643133	1.15211	0.98895853
PDE7A	Wald ratio	1	-0.146045	0.18207	0.422473	0.864119	0.604773	1.234679	0.98895853
ANGPT2	Wald ratio	1	-0.121759	0.128316	0.342669	0.885361	0.688488	1.138531	0.98895853
PTPRD	Wald ratio	1	0.327778	0.358248	0.360219	1.387881	0.687711	2.800907	0.98895853
OR13C8	Wald ratio	1	0.0584463	0.067155	0.384127	1.060188	0.929436	1.209335	0.98895853
ABCA1	Wald ratio	1	-0.091876	0.115221	0.425227	0.912218	0.727814	1.143345	0.98895853
TNFSF15	Wald ratio	1	-0.092503	0.091886	0.314073	0.911647	0.761397	1.091545	0.98895853
OR1L3	Wald ratio	1	-0.043833	0.043434	0.312891	0.957114	0.879006	1.042163	0.98895853
OR1Q1	Wald ratio	1	0.0775848	0.089257	0.384721	1.080674	0.907231	1.287276	0.98895853
OR1J2	Wald ratio	1	-0.075181	0.091786	0.412732	0.927575	0.774853	1.110398	0.98895853
OR1N2	Wald ratio	1	-0.082866	0.082866	0.317311	0.920475	0.782483	1.082801	0.98895853
OR5C1	Wald ratio	1	0.0774811	0.090764	0.393294	1.080562	0.904462	1.290949	0.98895853
OLFML2A	Wald ratio	1	0.1082266	0.116286	0.352012	1.1143	0.887192	1.399545	0.98895853
ANGPTL2	Wald ratio	1	0.1197595	0.128872	0.352737	1.127226	0.875615	1.451138	0.98895853
FIBCD1	Wald ratio	1	0.1156828	0.132086	0.38113	1.12264	0.866576	1.454367	0.98895853
KLHL9	Wald ratio	1	0.0845218	0.10432	0.417814	1.088197	0.886969	1.335076	0.98895853
IFNA10	Wald ratio	1	0.071169	0.075147	0.343609	1.073763	0.926705	1.244157	0.98895853
GBA2	Wald ratio	1	-0.239009	0.246212	0.331675	0.787408	0.485982	1.275791	0.98895853
KDM4C	Wald ratio	1	0.4234298	0.467537	0.365116	1.527191	0.610826	3.818289	0.98895853
RORB	Wald ratio	1	0.2488232	0.259031	0.336758	1.282515	0.771917	2.130858	0.98895853
OR7E31P	Wald ratio	1	0.078227	0.094192	0.406251	1.081368	0.899075	1.300622	0.98895853
ASPN	Wald ratio	1	-0.134869	0.143834	0.348414	0.87383	0.659163	1.158408	0.98895853
HSD17B3	Wald ratio	1	0.1086125	0.113138	0.337055	1.11473	0.893027	1.391473	0.98895853
HABP4	Wald ratio	1	0.2410668	0.241067	0.317311	1.272606	0.793403	2.04124	0.98895853
S100A13	Inverse variance weighted	2	0.0271128	0.031434	0.388401	1.027484	0.96609	1.092779	0.98895853
NR1I3	Inverse variance weighted	2	-0.166263	0.163909	0.310411	0.846824	0.614145	1.167657	0.98895853
PAPPA2	Inverse variance weighted	2	-0.064356	0.077747	0.407806	0.937672	0.80514	1.092018	0.98895853
PLA2G2A	Inverse variance weighted	2	0.1072995	0.122252	0.380111	1.113268	0.876066	1.414694	0.98895853
NBL1	Inverse variance weighted	2	0.1103269	0.117763	0.348831	1.116643	0.886488	1.406552	0.98895853
ADORA1	Inverse variance weighted	3	-0.135393	0.17167	0.430299	0.873373	0.623837	1.222724	0.98895853
HHAT	Inverse variance weighted	2	-0.033626	0.034335	0.327413	0.966933	0.904003	1.034244	0.98895853
DUSP10	Inverse variance weighted	2	-0.117163	0.133651	0.380685	0.88944	0.684464	1.1558	0.98895853
MAP3K21	Inverse variance weighted	4	-0.03468	0.038753	0.370844	0.965914	0.895264	1.04214	0.98895853
OR2T34	Inverse variance weighted	2	-0.08904	0.0928	0.337314	0.914809	0.762671	1.097295	0.98895853
COL16A1	Inverse variance weighted	2	0.0444626	0.054948	0.41841	1.045466	0.938723	1.164346	0.98895853
RSPO1	Inverse variance weighted	2	0.0478264	0.052071	0.358368	1.048989	0.947211	1.161702	0.98895853
GUCA2A	Inverse variance weighted	2	-0.124876	0.157334	0.427367	0.882606	0.648398	1.201412	0.98895853
CYP2J2	Inverse variance weighted	2	0.1022804	0.112041	0.361302	1.107694	0.889301	1.379719	0.98895853
CACHD1	Inverse variance weighted	2	-0.082096	0.087194	0.346432	0.921183	0.776471	1.092867	0.98895853
TGFBR3	Inverse variance weighted	2	-0.119974	0.143777	0.404032	0.886944	0.66913	1.17566	0.98895853
ITGA8	Inverse variance weighted	3	-0.043001	0.051697	0.405531	0.957911	0.865605	1.06006	0.98895853
AKR1E2	Inverse variance weighted	2	0.0921659	0.117272	0.431918	1.096547	0.871371	1.379912	0.98895853
SRGN	Inverse variance weighted	3	0.1431576	0.142841	0.31624	1.153912	0.872135	1.526727	0.98895853
TYSND1	Inverse variance weighted	2	0.1290007	0.132543	0.330418	1.137691	0.877407	1.475188	0.98895853
LIPJ	Inverse variance weighted	2	0.0719698	0.080166	0.369314	1.074623	0.918369	1.257462	0.98895853
LIPN	Inverse variance weighted	2	0.0687046	0.072822	0.345445	1.07112	0.928647	1.23545	0.98895853
PLCE1	Inverse variance weighted	2	-0.041073	0.048257	0.394687	0.959759	0.873143	1.054967	0.98895853
MTRNR2L8	Inverse variance weighted	2	0.0838174	0.099978	0.401831	1.08743	0.893919	1.322832	0.98895853
FIBIN	Inverse variance weighted	2	0.1004661	0.105439	0.340671	1.105686	0.899251	1.359512	0.98895853
TRIM5	Inverse variance weighted	2	-0.046645	0.046458	0.315357	0.954426	0.871358	1.045412	0.98895853
CCKBR	Inverse variance weighted	2	0.1717189	0.17001	0.31247	1.187344	0.850866	1.656884	0.98895853
GSTP1	Inverse variance weighted	2	-0.037958	0.037977	0.317562	0.962754	0.893693	1.037151	0.98895853
MRGPRF	Inverse variance weighted	2	-0.036009	0.043185	0.40437	0.964631	0.886343	1.049835	0.98895853
P2RY2	Inverse variance weighted	3	-0.05163	0.064248	0.421631	0.949681	0.837314	1.077127	0.98895853
PAK1	Inverse variance weighted	2	0.0689846	0.085552	0.42004	1.07142	0.906018	1.267017	0.98895853
SLC5A8	Inverse variance weighted	2	0.0760866	0.091284	0.404556	1.079056	0.90228	1.290467	0.98895853
SLC15A4	Inverse variance weighted	2	-0.091303	0.104565	0.38257	0.912741	0.743601	1.120353	0.98895853
P2RX2	Inverse variance weighted	2	0.0284674	0.028662	0.320613	1.028876	0.97267	1.088331	0.98895853
ABCC9	Inverse variance weighted	3	0.0379322	0.045087	0.400172	1.038661	0.950813	1.134625	0.98895853
LRRK2	Inverse variance weighted	2	-0.128706	0.163509	0.431195	0.879233	0.638149	1.211394	0.98895853
SOAT2	Inverse variance weighted	2	-0.063419	0.072998	0.384966	0.93855	0.81343	1.082915	0.98895853
ACRBP	Inverse variance weighted	2	-0.084481	0.085157	0.32117	0.918989	0.777719	1.08592	0.98895853

ERCC5	Inverse variance weighted	2	0.0339577	0.043245	0.432315	1.034541	0.950466	1.126052	0.98895853
OLFM4	Inverse variance weighted	2	0.1324117	0.155383	0.394124	1.141578	0.841862	1.547998	0.98895853
STK24	Inverse variance weighted	2	-0.124636	0.123945	0.314619	0.882818	0.692416	1.125577	0.98895853
STYX	Inverse variance weighted	2	-0.231884	0.274608	0.398435	0.793038	0.46296	1.358453	0.98895853
MAP2K1	Inverse variance weighted	2	-0.250308	0.277087	0.366336	0.778561	0.452305	1.34015	0.98895853
CHRNA5	Inverse variance weighted	2	0.0119262	0.013976	0.39347	1.011998	0.984652	1.040102	0.98895853
ADAMTSL3	Inverse variance weighted	2	-0.101823	0.100904	0.312926	0.90319	0.741118	1.100704	0.98895853
SV2B	Inverse variance weighted	2	0.175233	0.189572	0.355299	1.191524	0.821742	1.727706	0.98895853
TPSB2	Inverse variance weighted	3	0.0139769	0.017167	0.415535	1.014075	0.980523	1.048776	0.98895853
PRSS30P	Inverse variance weighted	2	-0.034347	0.04066	0.398264	0.966236	0.892221	1.046391	0.98895853
ADCY9	Inverse variance weighted	2	-0.112202	0.12388	0.36508	0.893864	0.701168	1.139516	0.98895853
VASN	Inverse variance weighted	2	0.0973553	0.10081	0.33418	1.102252	0.904627	1.34305	0.98895853
CBLN1	Inverse variance weighted	2	0.135001	0.139826	0.334296	1.144538	0.870178	1.505401	0.98895853
APRT	Inverse variance weighted	6	-0.062074	0.075272	0.40956	0.939813	0.810903	1.089216	0.98895853
SPNS2	Inverse variance weighted	2	0.0638093	0.07785	0.412418	1.065889	0.91505	1.241593	0.98895853
PNMT	Inverse variance weighted	2	-0.10654	0.109349	0.329904	0.898939	0.725521	1.113808	0.98895853
PHOSPHO1	Inverse variance weighted	2	-0.134046	0.140568	0.340284	0.87455	0.663943	1.151962	0.98895853
MRC2	Inverse variance weighted	2	-0.087487	0.093058	0.347145	0.91623	0.763471	1.099555	0.98895853
ARSG	Inverse variance weighted	2	0.0835518	0.103214	0.418228	1.087142	0.888032	1.330895	0.98895853
CD300LF	Inverse variance weighted	2	-0.049539	0.053903	0.358072	0.951668	0.856253	1.057715	0.98895853
ITGB4	Inverse variance weighted	2	0.1033402	0.130714	0.429189	1.108869	0.85825	1.432671	0.98895853
SMCHD1	Inverse variance weighted	3	0.1129357	0.143917	0.432614	1.11956	0.844389	1.484404	0.98895853
SERPINB2	Inverse variance weighted	2	0.0536954	0.06537	0.411415	1.055163	0.928272	1.199399	0.98895853
TMX3	Inverse variance weighted	2	-0.275524	0.290645	0.343142	0.759174	0.429476	1.341972	0.98895853
GALR1	Inverse variance weighted	2	0.070352	0.080108	0.379824	1.072886	0.91699	1.255285	0.98895853
ACP5	Inverse variance weighted	2	0.1369573	0.154582	0.375627	1.146779	0.847026	1.552613	0.98895853
RLN3	Inverse variance weighted	2	-0.107059	0.125263	0.39273	0.898472	0.702876	1.148499	0.98895853
OR7A5	Inverse variance weighted	2	0.0292531	0.02992	0.328215	1.029685	0.971038	1.091875	0.98895853
MAST3	Inverse variance weighted	2	0.1886866	0.216682	0.383864	1.207662	0.789772	1.84667	0.98895853
MFSD12	Inverse variance weighted	3	-0.077011	0.09501	0.41762	0.92588	0.768566	1.115394	0.98895853
SLC7A9	Inverse variance weighted	3	-0.021821	0.026092	0.402967	0.978415	0.929638	1.029752	0.98895853
FFAR1	Inverse variance weighted	2	0.0550779	0.066442	0.407129	1.056623	0.927605	1.203585	0.98895853
PVR	Inverse variance weighted	3	-0.029865	0.035062	0.394332	0.970577	0.906118	1.03962	0.98895853
APOC2	Inverse variance weighted	2	0.0753751	0.08404	0.369772	1.078289	0.914532	1.271367	0.98895853
PGLYRP1	Inverse variance weighted	2	0.0708308	0.081022	0.381998	1.0734	0.915787	1.258138	0.98895853
KCNJ14	Inverse variance weighted	2	-0.034171	0.040222	0.395582	0.966407	0.893145	1.045678	0.98895853
CGB5	Inverse variance weighted	2	-0.140131	0.154184	0.363427	0.869245	0.642537	1.175942	0.98895853
CD33	Inverse variance weighted	2	-0.11346	0.116508	0.33014	0.89274	0.710479	1.121758	0.98895853
KLK8	Inverse variance weighted	2	-0.05113	0.054298	0.346366	0.950155	0.854231	1.056851	0.98895853
IL1R1	Inverse variance weighted	2	0.0680304	0.067699	0.314948	1.070398	0.937386	1.222283	0.98895853
MERTK	Inverse variance weighted	2	-0.036467	0.04135	0.377829	0.96419	0.889129	1.045588	0.98895853
IL37	Inverse variance weighted	2	0.0783444	0.085673	0.360475	1.081495	0.91432	1.279236	0.98895853
ABCB11	Inverse variance weighted	2	-0.055488	0.067099	0.408258	0.946023	0.829442	1.07899	0.98895853
KLHL23	Inverse variance weighted	2	-0.095453	0.113042	0.398446	0.908961	0.72832	1.134407	0.98895853
RESP18	Inverse variance weighted	3	-0.017017	0.020903	0.415598	0.983127	0.943663	1.024242	0.98895853
LHCGR	Inverse variance weighted	4	0.0398206	0.043406	0.358932	1.040624	0.955754	1.133031	0.98895853
CD8A	Inverse variance weighted	2	-0.087961	0.096643	0.362735	0.915796	0.757765	1.106784	0.98895853
RBCK1	Inverse variance weighted	3	0.0807781	0.095017	0.395243	1.08413	0.899916	1.306054	0.98895853
TRIB3	Inverse variance weighted	2	-0.048464	0.053092	0.361335	0.952692	0.858538	1.057171	0.98895853
LBP	Inverse variance weighted	2	-0.082529	0.08429	0.327524	0.920784	0.780565	1.086193	0.98895853
PROKR2	Inverse variance weighted	2	-0.073869	0.076755	0.335847	0.928793	0.799068	1.079578	0.98895853
CHRNA4	Inverse variance weighted	2	0.1189065	0.143014	0.40573	1.126265	0.850951	1.490653	0.98895853
CXADR	Inverse variance weighted	2	-0.239945	0.260233	0.356509	0.786671	0.472365	1.310112	0.98895853
BACE2	Inverse variance weighted	2	-0.094017	0.095808	0.326439	0.910267	0.754424	1.098303	0.98895853
SIK1	Inverse variance weighted	2	0.097322	0.102087	0.340424	1.102215	0.902336	1.34637	0.98895853
COL6A1	Inverse variance weighted	2	0.1392199	0.167669	0.406353	1.149377	0.827447	1.596558	0.98895853
ADA2	Inverse variance weighted	2	0.0500999	0.049915	0.315521	1.051376	0.953388	1.159436	0.98895853
PI4KAP2	Inverse variance weighted	3	0.0242175	0.030783	0.431443	1.024513	0.964528	1.088229	0.98895853
IGLV4-60	Inverse variance weighted	2	0.1267779	0.132699	0.339384	1.135165	0.875193	1.472361	0.98895853
RAF1	Inverse variance weighted	2	-0.152774	0.172492	0.375787	0.858324	0.6121	1.203593	0.98895853
MGLL	Inverse variance weighted	2	0.1390811	0.164059	0.396577	1.149217	0.833206	1.585083	0.98895853
SUCNR1	Inverse variance weighted	2	-0.068504	0.086408	0.427898	0.93379	0.788309	1.106118	0.98895853
DNAJB11	Inverse variance weighted	2	-0.094329	0.112873	0.403318	0.909983	0.72938	1.135306	0.98895853
SUMF1	Inverse variance weighted	3	-0.081889	0.092011	0.373471	0.921374	0.769333	1.103463	0.98895853
ULK4	Inverse variance weighted	3	-0.059472	0.063933	0.352258	0.942262	0.831287	1.068053	0.98895853

GPX1	Inverse variance weighted	2	-0.03769	0.045498	0.407447	0.963011	0.880851	1.052834	0.98895853
PRKCD	Inverse variance weighted	2	0.0744382	0.080986	0.358017	1.077279	0.919161	1.262597	0.98895853
ADH5	Inverse variance weighted	3	-0.106656	0.120184	0.374844	0.898835	0.710194	1.137583	0.98895853
MTTP	Inverse variance weighted	3	0.0544838	0.061042	0.372092	1.055995	0.936919	1.190206	0.98895853
FREM3	Inverse variance weighted	2	-0.053092	0.062427	0.395063	0.948293	0.83908	1.071719	0.98895853
SFRP2	Inverse variance weighted	2	0.0545099	0.066603	0.413111	1.056023	0.926787	1.203281	0.98895853
TDO2	Inverse variance weighted	2	-0.039784	0.046952	0.396807	0.960997	0.876508	1.05363	0.98895853
CBR4	Inverse variance weighted	2	-0.052967	0.056765	0.350773	0.948412	0.84855	1.060025	0.98895853
AGA	Inverse variance weighted	2	0.026438	0.028009	0.345223	1.026791	0.971941	1.084736	0.98895853
TLR6	Inverse variance weighted	2	-0.065055	0.075826	0.39092	0.937016	0.807612	1.087155	0.98895853
STK32B	Inverse variance weighted	3	-0.079071	0.084917	0.351773	0.923974	0.782307	1.091296	0.98895853
EPHA5	Inverse variance weighted	2	-0.146909	0.162903	0.367153	0.863372	0.627382	1.18813	0.98895853
PARM1	Inverse variance weighted	2	-0.129793	0.152929	0.396039	0.878277	0.650813	1.185242	0.98895853
SLC9A3	Inverse variance weighted	2	-0.036025	0.044541	0.418637	0.964617	0.883976	1.052614	0.98895853
PPWD1	Inverse variance weighted	2	0.0345409	0.038605	0.370932	1.035144	0.95971	1.116508	0.98895853
MCHR2	Inverse variance weighted	2	0.0406687	0.040628	0.316823	1.041507	0.961788	1.127834	0.98895853
ROS1	Inverse variance weighted	2	-0.034904	0.044445	0.432259	0.965698	0.885133	1.053595	0.98895853
SMPDL3A	Inverse variance weighted	2	0.0509134	0.051997	0.327502	1.052232	0.950278	1.165124	0.98895853
MOXD1	Inverse variance weighted	2	0.0797607	0.101488	0.431917	1.083028	0.88767	1.32138	0.98895853
IGF2R	Inverse variance weighted	2	-0.140656	0.161788	0.384636	0.868788	0.632699	1.192973	0.98895853
TPMT	Inverse variance weighted	3	0.0492732	0.057585	0.392186	1.050507	0.938386	1.176025	0.98895853
GMNN	Inverse variance weighted	2	-0.13374	0.131732	0.309992	0.874818	0.675748	1.132532	0.98895853
SERPINB1	Inverse variance weighted	3	-0.069376	0.072798	0.340593	0.932976	0.808916	1.076062	0.98895853
BAK1	Inverse variance weighted	2	-0.079939	0.080572	0.321125	0.923172	0.788313	1.081103	0.98895853
ME1	Inverse variance weighted	2	0.0924216	0.105288	0.380053	1.096827	0.892309	1.348221	0.98895853
GPR63	Inverse variance weighted	2	0.0626168	0.077527	0.419275	1.064619	0.914539	1.239328	0.98895853
OR2A42	Inverse variance weighted	2	-0.062014	0.069743	0.373908	0.93987	0.819787	1.077542	0.98895853
SEMA3C	Inverse variance weighted	3	-0.06295	0.068352	0.357068	0.93899	0.821256	1.073603	0.98895853
TFPI2	Inverse variance weighted	2	0.0624187	0.071663	0.383754	1.064408	0.924926	1.224924	0.98895853
PRSS55	Inverse variance weighted	2	0.0393612	0.044712	0.37868	1.040146	0.952873	1.135413	0.98895853
CTSB	Inverse variance weighted	2	-0.040354	0.044695	0.366593	0.96045	0.879893	1.048382	0.98895853
FGFR1	Inverse variance weighted	2	-0.123273	0.131596	0.348887	0.884023	0.683041	1.144143	0.98895853
CPQ	Inverse variance weighted	2	0.1419704	0.147654	0.336297	1.152542	0.862921	1.539369	0.98895853
TLR4	Inverse variance weighted	3	-0.103143	0.106464	0.332642	0.901998	0.732118	1.111296	0.98895853
GSN	Inverse variance weighted	2	-0.129312	0.164884	0.432887	0.8787	0.636046	1.213927	0.98895853
ABL1	Inverse variance weighted	2	-0.124497	0.125494	0.321169	0.882941	0.690413	1.129156	0.98895853
BRD3	Inverse variance weighted	2	0.0639784	0.069742	0.358957	1.066069	0.929864	1.222225	0.98895853
TUBBP5	Inverse variance weighted	2	0.0335686	0.035743	0.34764	1.034138	0.964171	1.109183	0.98895853
PRSS3	Inverse variance weighted	2	0.0625179	0.077576	0.420304	1.064514	0.91436	1.239325	0.98895853
IL33	Inverse variance weighted	2	0.0701051	0.071696	0.328168	1.072621	0.932003	1.234455	0.98895853
OMD	Inverse variance weighted	2	-0.099917	0.103833	0.33591	0.904913	0.738282	1.109153	0.98895853
EXTL2	Wald ratio	1	-0.106628	0.136953	0.436232	0.89886	0.687251	1.175625	0.9894838
AMY1A	Wald ratio	1	-0.063464	0.081681	0.437172	0.938507	0.799667	1.101453	0.9894838
CELSR2	Wald ratio	1	-0.196029	0.255582	0.443087	0.821988	0.498092	1.356506	0.9894838
CD244	Wald ratio	1	0.0817	0.107184	0.445918	1.08513	0.879518	1.33881	0.9894838
SLC9C2	Wald ratio	1	-0.025551	0.033735	0.448812	0.974773	0.912406	1.041404	0.9894838
SLC45A3	Wald ratio	1	-0.143922	0.190077	0.448945	0.865956	0.596621	1.256877	0.9894838
OR6F1	Wald ratio	1	0.0442946	0.058339	0.447697	1.04529	0.932347	1.171916	0.9894838
TIE1	Wald ratio	1	-0.05355	0.069117	0.438473	0.947859	0.827771	1.085368	0.9894838
AGBL4	Wald ratio	1	-0.199592	0.257701	0.438629	0.819065	0.494264	1.357306	0.9894838
TNNI3K	Wald ratio	1	0.0287519	0.037599	0.444447	1.029169	0.956053	1.107877	0.9894838
SFRP5	Wald ratio	1	0.0492793	0.065349	0.45079	1.050514	0.924221	1.194064	0.9894838
FSHB	Wald ratio	1	-0.01489	0.019472	0.444447	0.98522	0.948328	1.023547	0.9894838
OR5M8	Wald ratio	1	-0.207487	0.274088	0.449044	0.812623	0.474876	1.390587	0.9894838
LTBP3	Wald ratio	1	-0.29734	0.382062	0.436422	0.742792	0.351276	1.570671	0.9894838
DUSP8	Wald ratio	1	0.3202033	0.424053	0.450188	1.377408	0.599931	3.162451	0.9894838
LRIG3	Wald ratio	1	-0.105683	0.137388	0.441756	0.899709	0.687314	1.177741	0.9894838
EGLN3	Wald ratio	1	-0.11836	0.154688	0.444181	0.888376	0.65603	1.203013	0.9894838
PSMA6	Wald ratio	1	0.2171406	0.282001	0.4413	1.242519	0.714923	2.159468	0.9894838
SLCO3A1	Wald ratio	1	0.1846963	0.243365	0.447895	1.202853	0.746546	1.938066	0.9894838
CDH3	Wald ratio	1	-0.05606	0.071672	0.43411	0.945482	0.821571	1.088082	0.9894838
PRKCSH	Wald ratio	1	-0.388842	0.515863	0.450988	0.677841	0.246613	1.863116	0.9894838
ADGRL1	Wald ratio	1	0.2928398	0.388678	0.451195	1.340228	0.625647	2.870968	0.9894838
OR1I1	Wald ratio	1	0.0941141	0.122466	0.442197	1.098685	0.864227	1.39675	0.9894838
EMC10	Wald ratio	1	-0.304216	0.402973	0.450291	0.737702	0.33486	1.625167	0.9894838

PDIA6	Wald ratio	1	0.1968136	0.253397	0.437336	1.217517	0.740933	2.000651	0.9894838
NTSR2	Wald ratio	1	-0.055835	0.072258	0.439684	0.945695	0.820813	1.089577	0.9894838
GLI2	Wald ratio	1	0.1194553	0.154525	0.439493	1.126883	0.832424	1.525503	0.9894838
KCNK3	Wald ratio	1	-0.246536	0.321699	0.443465	0.781503	0.416002	1.468137	0.9894838
OXER1	Wald ratio	1	0.0861961	0.11403	0.449706	1.09002	0.871706	1.36301	0.9894838
PYGB	Wald ratio	1	-0.050538	0.065549	0.440711	0.950718	0.836095	1.081056	0.9894838
CYP2D6	Wald ratio	1	0.1840546	0.244037	0.450724	1.202081	0.745085	1.939377	0.9894838
UPK3A	Wald ratio	1	-0.050185	0.066178	0.448251	0.951053	0.835358	1.082772	0.9894838
PLA1A	Wald ratio	1	-0.095432	0.124971	0.445084	0.90898	0.711503	1.161266	0.9894838
SCN5A	Wald ratio	1	-0.09388	0.124545	0.450977	0.910392	0.713204	1.162098	0.9894838
LYZL4	Wald ratio	1	-0.031301	0.041515	0.450868	0.969184	0.893445	1.051343	0.9894838
SLC6A20	Wald ratio	1	-0.077994	0.100981	0.439903	0.92497	0.758876	1.127417	0.9894838
CFI	Wald ratio	1	-0.105941	0.137851	0.442179	0.899477	0.686513	1.178506	0.9894838
UGT2B17	Wald ratio	1	0.0330223	0.043357	0.446282	1.033574	0.949368	1.125247	0.9894838
ODAM	Wald ratio	1	0.1727681	0.223257	0.439019	1.18859	0.767347	1.841081	0.9894838
ERBIN	Wald ratio	1	-0.266984	0.345509	0.439684	0.765685	0.388998	1.507139	0.9894838
MBOAT1	Wald ratio	1	-0.099521	0.130799	0.446735	0.905271	0.700551	1.169815	0.9894838
COL11A2	Wald ratio	1	-0.173793	0.224876	0.439617	0.840471	0.540884	1.305994	0.9894838
COL21A1	Wald ratio	1	0.096476	0.126161	0.444447	1.101283	0.86002	1.410228	0.9894838
EEPD1	Wald ratio	1	0.0771546	0.100015	0.440453	1.080209	0.887919	1.314143	0.9894838
LY96	Wald ratio	1	0.108307	0.141401	0.443702	1.11439	0.844645	1.470279	0.9894838
CDK9	Wald ratio	1	0.0932344	0.122677	0.447255	1.097719	0.863111	1.396098	0.9894838
FCRL2	Inverse variance weighted	2	0.0571758	0.073788	0.438419	1.058842	0.916266	1.223604	0.9894838
ATF1	Inverse variance weighted	2	0.2060082	0.267374	0.44101	1.228763	0.727571	2.075207	0.9894838
DUOX2	Inverse variance weighted	2	-0.045634	0.060307	0.449231	0.955392	0.848882	1.075265	0.9894838
EEF2K	Inverse variance weighted	2	0.0427545	0.055095	0.437743	1.043682	0.93685	1.162696	0.9894838
SLC1A6	Inverse variance weighted	2	0.0455502	0.060554	0.451914	1.046604	0.929475	1.178492	0.9894838
LYPD5	Inverse variance weighted	2	0.0264394	0.034662	0.445601	1.026792	0.95935	1.098975	0.9894838
LILRB2	Inverse variance weighted	2	0.1027792	0.131447	0.434269	1.108247	0.856538	1.433924	0.9894838
SLC25A23	Inverse variance weighted	2	0.0323515	0.042381	0.445251	1.032881	0.95055	1.122342	0.9894838
TOP1	Inverse variance weighted	2	-0.425872	0.548005	0.437081	0.6532	0.223139	1.91213	0.9894838
SLC13A3	Inverse variance weighted	2	-0.064837	0.083636	0.438205	0.93722	0.795516	1.104166	0.9894838
ADAMTS5	Inverse variance weighted	2	-0.079732	0.105962	0.451777	0.923364	0.750198	1.136501	0.9894838
NUP210	Inverse variance weighted	2	-0.131993	0.170687	0.439342	0.876347	0.62717	1.224525	0.9894838
WNT5A	Inverse variance weighted	2	-0.116124	0.153734	0.450033	0.890364	0.658729	1.203452	0.9894838
CTSO	Inverse variance weighted	2	0.1043979	0.133333	0.433635	1.110042	0.85476	1.441567	0.9894838
TEC	Inverse variance weighted	2	0.0435614	0.057842	0.451383	1.044524	0.932572	1.169915	0.9894838
COL23A1	Inverse variance weighted	3	-0.06686	0.087697	0.445822	0.935326	0.787614	1.11074	0.9894838
GUSBP1	Inverse variance weighted	2	-0.036426	0.047909	0.447065	0.964229	0.877807	1.05916	0.9894838
TNKS	Inverse variance weighted	2	-0.0898	0.119382	0.451924	0.914114	0.723404	1.155101	0.9894838
SLC10A5	Inverse variance weighted	2	0.0442314	0.058041	0.44602	1.045224	0.932832	1.171158	0.9894838
MUSK	Inverse variance weighted	2	0.0782428	0.102053	0.443266	1.081385	0.885343	1.320838	0.9894838
CTSH	Wald ratio	1	-0.051056	0.068074	0.453255	0.950226	0.831536	1.085857	0.98992185
OR1E1	Wald ratio	1	-0.043242	0.057656	0.453255	0.95768	0.855348	1.072254	0.98992185
FGFR3	Wald ratio	1	0.1674876	0.223317	0.453255	1.182331	0.763217	1.831597	0.98992185
TNFRSF25	Inverse variance weighted	2	-0.238906	0.318249	0.45284	0.787489	0.422032	1.469412	0.98992185
DUSP5	Wald ratio	1	-0.139086	0.185881	0.454308	0.870153	0.604464	1.252625	0.99020447
TECTA	Wald ratio	1	-0.117957	0.158311	0.456212	0.888734	0.65165	1.212074	0.99020447
OR2AG2	Wald ratio	1	0.0579071	0.07762	0.455648	1.059617	0.910075	1.233731	0.99020447
EPYC	Wald ratio	1	-0.072053	0.096197	0.45385	0.930482	0.77059	1.12355	0.99020447
TRIM25	Wald ratio	1	-0.086744	0.116339	0.455901	0.916912	0.729959	1.151747	0.99020447
ROCK1	Wald ratio	1	-0.161184	0.216326	0.456212	0.851135	0.557003	1.300586	0.99020447
COL27A1	Wald ratio	1	0.1012035	0.135376	0.454718	1.106502	0.848629	1.442735	0.99020447
MTAP	Wald ratio	1	0.0843919	0.112903	0.454777	1.088055	0.87206	1.35755	0.99020447
OR51E1	Inverse variance weighted	3	0.0436132	0.058487	0.455853	1.044578	0.931442	1.171456	0.99020447
ADH1C	Inverse variance weighted	2	0.025662	0.034383	0.455449	1.025994	0.95913	1.097519	0.99020447
KCNC4	Wald ratio	1	0.090693	0.161403	0.574181	1.094933	0.797992	1.502368	0.99261813
SCNN1D	Wald ratio	1	-0.072019	0.118124	0.542069	0.930513	0.738199	1.17293	0.99261813
ACP6	Wald ratio	1	-0.01755	0.024436	0.472645	0.982604	0.936651	1.03081	0.99261813
DNAJC16	Wald ratio	1	-0.221031	0.423983	0.602144	0.801692	0.349225	1.840388	0.99261813
SLC25A34	Wald ratio	1	0.0228395	0.043141	0.59652	1.023102	0.940149	1.113375	0.99261813
PGLYRP4	Wald ratio	1	-0.084041	0.124568	0.49989	0.919393	0.720223	1.173642	0.99261813
CASP9	Wald ratio	1	-0.048446	0.074871	0.517594	0.952709	0.822676	1.103295	0.99261813
EFNA1	Wald ratio	1	0.0744608	0.105309	0.479523	1.077303	0.87639	1.324276	0.99261813
CLCNKA	Wald ratio	1	0.0212064	0.045064	0.637935	1.021433	0.935085	1.115755	0.99261813

HDGF	Wald ratio	1	0.083713	0.128603	0.515083	1.087317	0.84506	1.399023	0.99261813
ATP1A4	Wald ratio	1	-0.059426	0.125155	0.634915	0.942305	0.737322	1.204276	0.99261813
FCGR2B	Wald ratio	1	0.0716545	0.104423	0.492592	1.074284	0.875452	1.318275	0.99261813
FCGR2A	Wald ratio	1	0.0497393	0.092965	0.592628	1.050997	0.875927	1.261058	0.99261813
FMO3	Wald ratio	1	0.0349468	0.048202	0.468452	1.035565	0.942207	1.138172	0.99261813
FMO4	Wald ratio	1	0.0577697	0.110472	0.601019	1.059471	0.853205	1.315602	0.99261813
GPR52	Wald ratio	1	-0.088336	0.151682	0.560314	0.915454	0.680021	1.232396	0.99261813
CACNA1E	Wald ratio	1	0.236751	0.382444	0.535885	1.267126	0.598793	2.681406	0.99261813
PRG4	Wald ratio	1	-0.035518	0.075476	0.637935	0.965105	0.832393	1.118976	0.99261813
PTPRC	Wald ratio	1	0.1941954	0.323659	0.548506	1.214334	0.643923	2.290035	0.99261813
LGR6	Wald ratio	1	0.0469594	0.074842	0.530364	1.048079	0.905082	1.21367	0.99261813
GPR37L1	Wald ratio	1	0.1499178	0.294481	0.610689	1.161739	0.65229	2.069074	0.99261813
PLA2G5	Wald ratio	1	0.0257417	0.050111	0.607463	1.026076	0.930089	1.131969	0.99261813
PRELP	Wald ratio	1	0.1090278	0.149264	0.465124	1.115193	0.832327	1.494192	0.99261813
CD34	Wald ratio	1	0.1002285	0.169617	0.554581	1.105424	0.79277	1.541381	0.99261813
ALPL	Wald ratio	1	-0.055891	0.122028	0.646941	0.945642	0.744482	1.201157	0.99261813
BPNT1	Wald ratio	1	-0.076107	0.106209	0.473635	0.926717	0.752559	1.14118	0.99261813
MARK1	Wald ratio	1	-0.109488	0.181339	0.545994	0.896293	0.62819	1.27882	0.99261813
CAPN2	Wald ratio	1	-0.086061	0.140636	0.540577	0.917538	0.696486	1.208749	0.99261813
MTR	Wald ratio	1	-0.056919	0.118485	0.630949	0.94467	0.7489	1.191617	0.99261813
OR14A2	Wald ratio	1	0.0280419	0.055574	0.613849	1.028439	0.922302	1.14679	0.99261813
OR2T1	Wald ratio	1	-0.049577	0.074366	0.504985	0.951632	0.822559	1.100957	0.99261813
SMPDL3B	Wald ratio	1	0.0383311	0.053988	0.477704	1.039075	0.934742	1.155054	0.99261813
MATN1	Wald ratio	1	0.0918694	0.125617	0.464569	1.096222	0.85698	1.402252	0.99261813
MMEL1	Wald ratio	1	0.0530261	0.077213	0.492242	1.054457	0.906366	1.226745	0.99261813
COL8A2	Wald ratio	1	-0.077628	0.159677	0.626858	0.925309	0.676655	1.265338	0.99261813
MPL	Wald ratio	1	0.0825738	0.159106	0.60377	1.086079	0.795111	1.483525	0.99261813
AKR1A1	Wald ratio	1	0.1260943	0.197751	0.523708	1.134389	0.769897	1.671443	0.99261813
GPX7	Wald ratio	1	0.0946363	0.141377	0.503248	1.099259	0.833215	1.45025	0.99261813
CDCP2	Wald ratio	1	0.0633183	0.104803	0.545733	1.065366	0.867539	1.308304	0.99261813
TAS1R1	Wald ratio	1	0.0497324	0.096074	0.604705	1.05099	0.8706	1.268757	0.99261813
ZRANB2	Wald ratio	1	-0.156044	0.236233	0.508901	0.855521	0.538449	1.359305	0.99261813
PIGK	Wald ratio	1	0.1579239	0.258252	0.540861	1.171077	0.705922	1.942737	0.99261813
CLCA2	Wald ratio	1	0.0547335	0.101257	0.588824	1.056259	0.866121	1.288137	0.99261813
ABCD3	Wald ratio	1	-0.142967	0.261703	0.584862	0.866782	0.518972	1.447692	0.99261813
FGF8	Wald ratio	1	0.0893554	0.153342	0.560081	1.093469	0.809617	1.476841	0.99261813
SLK	Wald ratio	1	-0.132981	0.238498	0.577134	0.875482	0.548572	1.397207	0.99261813
PDCD4	Wald ratio	1	-0.132502	0.205607	0.519287	0.875901	0.585381	1.310603	0.99261813
ADRA2A	Wald ratio	1	0.1206531	0.264614	0.64842	1.128233	0.671668	1.895148	0.99261813
DMBT1L1	Wald ratio	1	0.1470289	0.2002	0.462698	1.158387	0.782421	1.715013	0.99261813
CYP2E1	Wald ratio	1	0.0785962	0.131216	0.549183	1.081767	0.836452	1.39903	0.99261813
PRTFDC1	Wald ratio	1	-0.160907	0.262677	0.540164	0.851372	0.508773	1.424669	0.99261813
HSD17B7P2	Wald ratio	1	-0.041584	0.05747	0.469325	0.959269	0.857079	1.073643	0.99261813
OR13A1	Wald ratio	1	0.036355	0.0557	0.513953	1.037024	0.929771	1.156649	0.99261813
ALOX5	Wald ratio	1	0.025433	0.036666	0.487907	1.025759	0.954629	1.102189	0.99261813
AKR1C1	Wald ratio	1	-0.018289	0.037449	0.625286	0.981877	0.912389	1.056658	0.99261813
CISD1	Wald ratio	1	0.1203127	0.193382	0.533843	1.127849	0.772042	1.647636	0.99261813
ITIH2	Wald ratio	1	-0.031998	0.061265	0.601466	0.968508	0.858922	1.092076	0.99261813
ADK	Wald ratio	1	-0.122456	0.266657	0.646071	0.884745	0.524608	1.492111	0.99261813
RBP4	Wald ratio	1	0.1709634	0.259958	0.510758	1.186447	0.7128	1.974828	0.99261813
CYP2C19	Wald ratio	1	-0.025297	0.051326	0.622114	0.975021	0.881706	1.078211	0.99261813
PDGFD	Wald ratio	1	-0.077108	0.122891	0.530364	0.92579	0.727621	1.17793	0.99261813
CASP4	Wald ratio	1	-0.072221	0.145767	0.620279	0.930325	0.699126	1.237981	0.99261813
ATM	Wald ratio	1	0.1217224	0.19321	0.528695	1.129441	0.773392	1.649405	0.99261813
MUC5AC	Wald ratio	1	0.0742501	0.09999	0.457739	1.077076	0.885387	1.310267	0.99261813
SCN3B	Wald ratio	1	0.1813927	0.263241	0.490776	1.198886	0.715654	2.008412	0.99261813
SLC37A2	Wald ratio	1	0.0432378	0.087234	0.62014	1.044186	0.880081	1.238891	0.99261813
KCNJ1	Wald ratio	1	-0.040538	0.056935	0.476465	0.960273	0.858877	1.07364	0.99261813
ABCC8	Wald ratio	1	-0.084228	0.117703	0.474241	0.919222	0.729844	1.15774	0.99261813
OTOG	Wald ratio	1	-0.065136	0.097704	0.504985	0.93694	0.77365	1.134695	0.99261813
MUC15	Wald ratio	1	0.0969491	0.150271	0.518823	1.101804	0.820713	1.479169	0.99261813
SLC5A12	Wald ratio	1	0.0786439	0.106875	0.461823	1.081819	0.877366	1.333916	0.99261813
PAMR1	Wald ratio	1	0.0990518	0.136342	0.467535	1.104123	0.845203	1.442362	0.99261813
OR52M1	Wald ratio	1	0.0666365	0.114452	0.560417	1.068907	0.854115	1.337714	0.99261813
TRIM21	Wald ratio	1	0.0743431	0.116559	0.523595	1.077176	0.857175	1.353643	0.99261813

TRIM68	Wald ratio	1	0.0392626	0.055229	0.477146	1.040044	0.933339	1.158947	0.99261813
CRY2	Wald ratio	1	-0.075675	0.158825	0.633739	0.927117	0.67911	1.265695	0.99261813
FOLH1	Wald ratio	1	0.0555188	0.08594	0.518268	1.057089	0.893219	1.251023	0.99261813
OR4C13	Wald ratio	1	-0.14053	0.19751	0.47677	0.868898	0.58999	1.279654	0.99261813
OR5B2	Wald ratio	1	0.0641454	0.112855	0.569771	1.066247	0.854661	1.330215	0.99261813
OR9Q1	Wald ratio	1	-0.064054	0.093337	0.49254	0.937954	0.781145	1.12624	0.99261813
PTGDR2	Wald ratio	1	-0.029215	0.059927	0.625904	0.971208	0.863577	1.092254	0.99261813
SLC22A6	Wald ratio	1	-0.0836	0.120755	0.488744	0.9198	0.725946	1.165418	0.99261813
SLC22A9	Wald ratio	1	0.0524236	0.083071	0.527996	1.053822	0.895479	1.240163	0.99261813
PYGM	Wald ratio	1	-0.041882	0.091186	0.646017	0.958983	0.802033	1.146647	0.99261813
SLC25A22	Wald ratio	1	-0.101557	0.194153	0.600921	0.903429	0.617487	1.321786	0.99261813
CAPN1	Wald ratio	1	-0.109683	0.225599	0.626834	0.896118	0.57588	1.394436	0.99261813
ALDH3B1	Wald ratio	1	-0.072418	0.127824	0.571022	0.930142	0.724007	1.194965	0.99261813
AIP	Wald ratio	1	-0.183024	0.373467	0.624087	0.832748	0.400509	1.731473	0.99261813
P2RY6	Wald ratio	1	0.0757728	0.136939	0.580035	1.078717	0.824789	1.410822	0.99261813
UCP2	Wald ratio	1	-0.129121	0.174922	0.460416	0.878868	0.623773	1.238284	0.99261813
SLCO2B1	Wald ratio	1	0.0906978	0.179825	0.614004	1.094938	0.769696	1.557614	0.99261813
SERPINH1	Wald ratio	1	-0.128099	0.184614	0.487759	0.879766	0.612662	1.263321	0.99261813
WNT11	Wald ratio	1	0.0642726	0.095852	0.502512	1.066383	0.883736	1.286778	0.99261813
PRB4	Wald ratio	1	-0.063863	0.090983	0.482727	0.938133	0.784907	1.121272	0.99261813
PRKAB1	Wald ratio	1	-0.103013	0.151736	0.497202	0.902115	0.670041	1.214569	0.99261813
GRIN2B	Wald ratio	1	-0.171185	0.367219	0.641097	0.842666	0.410273	1.730766	0.99261813
MGP	Wald ratio	1	0.0685518	0.138209	0.619894	1.070956	0.816818	1.404164	0.99261813
PIK3C2G	Wald ratio	1	0.0512295	0.10187	0.615041	1.052564	0.862055	1.285175	0.99261813
PTHLH	Wald ratio	1	-0.127672	0.173029	0.460597	0.880142	0.627001	1.235486	0.99261813
YAF2	Wald ratio	1	-0.093131	0.167088	0.57727	0.911074	0.656637	1.264101	0.99261813
FGF6	Wald ratio	1	-0.047062	0.08857	0.595174	0.954028	0.80199	1.134889	0.99261813
IRAK4	Wald ratio	1	0.0682862	0.127855	0.593279	1.070672	0.833343	1.375589	0.99261813
TUBA1B	Wald ratio	1	0.1489186	0.23827	0.531971	1.160578	0.727537	1.851372	0.99261813
ITGB7	Wald ratio	1	0.0774053	0.105737	0.464134	1.08048	0.878237	1.329295	0.99261813
SDR9C7	Wald ratio	1	-0.040394	0.060289	0.502858	0.960411	0.853371	1.080877	0.99261813
RDH16	Wald ratio	1	0.0909177	0.122862	0.4593	1.095179	0.860802	1.393372	0.99261813
CYP27B1	Wald ratio	1	0.0566504	0.098305	0.564432	1.058286	0.872818	1.283164	0.99261813
PIP4K2C	Wald ratio	1	-0.107136	0.1883	0.56938	0.898403	0.621137	1.299437	0.99261813
TRHDE	Wald ratio	1	0.1801264	0.263757	0.494653	1.197369	0.714026	2.0079	0.99261813
SLC6A15	Wald ratio	1	0.2263691	0.314254	0.471316	1.254038	0.67735	2.321715	0.99261813
UBE2N	Wald ratio	1	0.1188659	0.257822	0.644771	1.126219	0.679455	1.866745	0.99261813
NR2C1	Wald ratio	1	-0.153027	0.213432	0.473386	0.858107	0.56476	1.303822	0.99261813
SLC25A15	Wald ratio	1	0.082046	0.147683	0.578515	1.085506	0.812685	1.449914	0.99261813
SLC25A30	Wald ratio	1	-0.056571	0.081121	0.485572	0.944999	0.806083	1.107855	0.99261813
SERPINE3	Wald ratio	1	0.0800824	0.175218	0.647639	1.083376	0.768477	1.527312	0.99261813
IGHV1-46	Wald ratio	1	-0.095984	0.200693	0.632465	0.908479	0.61303	1.346319	0.99261813
MYH6	Wald ratio	1	0.0364846	0.079179	0.644953	1.037158	0.888068	1.211278	0.99261813
COCH	Wald ratio	1	-0.030954	0.042459	0.465982	0.96952	0.892103	1.053656	0.99261813
NFKBIA	Wald ratio	1	0.1015365	0.212592	0.632926	1.10687	0.729684	1.679031	0.99261813
KLHL28	Wald ratio	1	-0.264503	0.456681	0.562464	0.767587	0.313612	1.878723	0.99261813
TXNDC16	Wald ratio	1	0.0511718	0.08588	0.551272	1.052504	0.88945	1.245449	0.99261813
FNTB	Wald ratio	1	0.0897014	0.138045	0.515823	1.093848	0.834546	1.433716	0.99261813
ADAM21	Wald ratio	1	0.0523942	0.071511	0.463758	1.053791	0.915974	1.212344	0.99261813
COQ6	Wald ratio	1	0.0496549	0.084161	0.555191	1.050908	0.891098	1.239379	0.99261813
ABCD4	Wald ratio	1	0.1086025	0.164549	0.509254	1.114719	0.807418	1.538979	0.99261813
TGFB3	Wald ratio	1	0.0530504	0.105693	0.615716	1.054483	0.85718	1.2972	0.99261813
ANGEL1	Wald ratio	1	0.0462592	0.073427	0.528695	1.047346	0.906959	1.209464	0.99261813
GPR68	Wald ratio	1	0.0941374	0.206442	0.64839	1.098711	0.733089	1.646683	0.99261813
PRIMA1	Wald ratio	1	-0.114197	0.159157	0.473057	0.892082	0.653022	1.218658	0.99261813
OR4F15	Wald ratio	1	0.0973846	0.184866	0.598342	1.102284	0.767243	1.583633	0.99261813
SLC12A6	Wald ratio	1	-0.211051	0.301502	0.483927	0.809732	0.448433	1.462127	0.99261813
PLCB2	Wald ratio	1	-0.075766	0.140353	0.589319	0.927033	0.704083	1.22058	0.99261813
PLA2G4B	Wald ratio	1	0.0782484	0.129993	0.547213	1.081391	0.838166	1.395197	0.99261813
GANC	Wald ratio	1	-0.118881	0.189704	0.530878	0.887913	0.612197	1.287805	0.99261813
FBN1	Wald ratio	1	0.1245404	0.242661	0.607792	1.132628	0.703931	1.822403	0.99261813
RORA	Wald ratio	1	0.080866	0.175631	0.645206	1.084226	0.768457	1.529747	0.99261813
HEXA	Wald ratio	1	0.1014582	0.189925	0.593202	1.106784	0.762773	1.605942	0.99261813
SEMA7A	Wald ratio	1	0.1177558	0.220683	0.59362	1.124969	0.729947	1.733764	0.99261813
IL16	Wald ratio	1	-0.082432	0.1273	0.517282	0.920874	0.71753	1.181845	0.99261813

NTRK3	Wald ratio	1	-0.114256	0.217404	0.599203	0.892029	0.582533	1.365958	0.99261813
TPSAB1	Wald ratio	1	-0.026904	0.04588	0.557615	0.973455	0.889737	1.06505	0.99261813
CACNA1H	Wald ratio	1	-0.149415	0.205446	0.467059	0.861212	0.575746	1.288216	0.99261813
HAGH	Wald ratio	1	0.0448013	0.08178	0.583811	1.04582	0.890932	1.227636	0.99261813
XYLT1	Wald ratio	1	0.1350473	0.200746	0.501121	1.144591	0.772275	1.696402	0.99261813
GPRC5B	Wald ratio	1	0.0837418	0.150956	0.579069	1.087348	0.808859	1.461721	0.99261813
ABCA3	Wald ratio	1	0.2001714	0.312806	0.522223	1.221612	0.66171	2.255274	0.99261813
IL21R	Wald ratio	1	0.0342544	0.060604	0.571926	1.034848	0.918945	1.165369	0.99261813
NTN3	Wald ratio	1	0.0781622	0.150934	0.604558	1.081298	0.804392	1.453526	0.99261813
NOD2	Wald ratio	1	0.0547568	0.095352	0.565794	1.056284	0.876224	1.273345	0.99261813
CETP	Wald ratio	1	-0.068382	0.101367	0.499928	0.933903	0.765626	1.139166	0.99261813
TK2	Wald ratio	1	0.0634076	0.129302	0.623862	1.065461	0.826939	1.372782	0.99261813
NFATC3	Wald ratio	1	-0.096123	0.210097	0.6473	0.908352	0.60175	1.371174	0.99261813
SLC7A6	Wald ratio	1	0.0951	0.165174	0.564779	1.099769	0.795615	1.520198	0.99261813
CHST5	Wald ratio	1	0.0946005	0.128566	0.461845	1.09922	0.854372	1.414237	0.99261813
CLEC3A	Wald ratio	1	-0.044506	0.067064	0.506923	0.95647	0.838659	1.09083	0.99261813
HSD17B2	Wald ratio	1	-0.053792	0.104982	0.608373	0.947629	0.771393	1.164128	0.99261813
ADORA2B	Wald ratio	1	0.0262919	0.041492	0.526301	1.026641	0.946455	1.11362	0.99261813
SLC5A10	Wald ratio	1	0.0455005	0.08089	0.573775	1.046552	0.893112	1.226353	0.99261813
NLK	Wald ratio	1	-0.12051	0.211864	0.569487	0.886468	0.585222	1.342783	0.99261813
SHPK	Wald ratio	1	0.0109761	0.015013	0.464723	1.011037	0.981719	1.04123	0.99261813
CCL8	Wald ratio	1	0.0610229	0.089048	0.493168	1.062923	0.892694	1.265614	0.99261813
CACNB1	Wald ratio	1	-0.211106	0.351267	0.54785	0.809688	0.406737	1.611841	0.99261813
P2RX1	Wald ratio	1	-0.04014	0.086301	0.641848	0.960655	0.811159	1.137702	0.99261813
WNK4	Wald ratio	1	0.1001531	0.151394	0.508267	1.10534	0.821536	1.487186	0.99261813
CRHR1	Wald ratio	1	-0.03032	0.043388	0.48468	0.970135	0.891045	1.056247	0.99261813
CXCL16	Wald ratio	1	-0.091226	0.159259	0.56677	0.912812	0.668063	1.247225	0.99261813
PLD2	Wald ratio	1	0.0953354	0.185039	0.606401	1.100028	0.765412	1.580928	0.99261813
WFIKKN2	Wald ratio	1	0.0652654	0.137282	0.634495	1.067442	0.815619	1.397017	0.99261813
CACNG5	Wald ratio	1	0.0585342	0.102267	0.567072	1.060281	0.867701	1.295604	0.99261813
PLSCR3	Wald ratio	1	0.1114338	0.221179	0.61439	1.11788	0.724642	1.724513	0.99261813
EIF4A1	Wald ratio	1	0.068255	0.137687	0.620087	1.070638	0.817413	1.402311	0.99261813
H3-3B	Wald ratio	1	-0.194428	0.289381	0.501663	0.823306	0.466912	1.451734	0.99261813
SGSH	Wald ratio	1	0.0808552	0.115507	0.483927	1.084214	0.864556	1.359681	0.99261813
GCGR	Wald ratio	1	-0.058582	0.117794	0.618959	0.943101	0.748669	1.188027	0.99261813
AATK	Wald ratio	1	-0.214418	0.388633	0.581137	0.807011	0.376763	1.728584	0.99261813
P4HB	Wald ratio	1	0.1486896	0.231679	0.521009	1.160313	0.736827	1.827193	0.99261813
AQP4	Wald ratio	1	-0.281315	0.413617	0.496419	0.75479	0.335543	1.69787	0.99261813
KDSR	Wald ratio	1	-0.079444	0.151919	0.601019	0.92363	0.685775	1.243982	0.99261813
SERPINB11	Wald ratio	1	-0.046549	0.073246	0.525093	0.954518	0.826867	1.101875	0.99261813
CNDP2	Wald ratio	1	0.1926817	0.320268	0.547423	1.212497	0.647236	2.271425	0.99261813
PTPRM	Wald ratio	1	0.0411643	0.060059	0.493096	1.042023	0.926305	1.172198	0.99261813
ANGPTL6	Wald ratio	1	0.0586365	0.107339	0.584877	1.06039	0.859205	1.308682	0.99261813
PTGER1	Wald ratio	1	-0.074949	0.101931	0.46216	0.927791	0.759775	1.132962	0.99261813
NCAN	Wald ratio	1	0.1706688	0.260146	0.511791	1.186098	0.712328	1.974972	0.99261813
CSNK1G2	Wald ratio	1	-0.170669	0.242571	0.481692	0.843101	0.524082	1.356313	0.99261813
PEPD	Wald ratio	1	0.1083724	0.190473	0.56938	1.114463	0.767241	1.618822	0.99261813
SCN1B	Wald ratio	1	-0.113169	0.234142	0.628859	0.893	0.564346	1.413049	0.99261813
HAMP	Wald ratio	1	-0.092939	0.155852	0.550955	0.911249	0.671387	1.236804	0.99261813
DAPK3	Wald ratio	1	0.1425878	0.215664	0.508512	1.153254	0.755698	1.759957	0.99261813
HIPK4	Wald ratio	1	-0.333891	0.498171	0.502709	0.716132	0.269738	1.901273	0.99261813
CYP2F1	Wald ratio	1	-0.038325	0.072875	0.59896	0.962401	0.834303	1.110166	0.99261813
AXL	Wald ratio	1	0.0827945	0.176953	0.639863	1.086319	0.767948	1.536677	0.99261813
CYP2S1	Wald ratio	1	0.0444599	0.096004	0.64329	1.045463	0.866141	1.261912	0.99261813
EBI3	Wald ratio	1	0.0699251	0.127444	0.58323	1.072428	0.835383	1.376736	0.99261813
PSG1	Wald ratio	1	0.0701811	0.140362	0.617075	1.072702	0.814705	1.412401	0.99261813
KCNN4	Wald ratio	1	0.0180275	0.030771	0.557971	1.018191	0.958598	1.081489	0.99261813
MADCAM1	Wald ratio	1	-0.127746	0.211974	0.546742	0.880077	0.580878	1.333388	0.99261813
MYDGF	Wald ratio	1	0.1388274	0.200857	0.489455	1.148926	0.775032	1.703196	0.99261813
GPR4	Wald ratio	1	-0.164617	0.242352	0.496982	0.848219	0.527489	1.363962	0.99261813
CGB2	Wald ratio	1	0.0470478	0.075524	0.533317	1.048172	0.903952	1.215402	0.99261813
FUT1	Wald ratio	1	0.0334816	0.04639	0.470455	1.034048	0.944176	1.132476	0.99261813
BAX	Wald ratio	1	0.161415	0.225208	0.473536	1.175173	0.75579	1.827269	0.99261813
TRPM4	Wald ratio	1	-0.07049	0.148961	0.63606	0.931937	0.695967	1.247913	0.99261813
CLEC11A	Wald ratio	1	0.0664465	0.119009	0.576617	1.068704	0.84636	1.349459	0.99261813

KLK6	Wald ratio	1	-0.167378	0.343126	0.625688	0.845879	0.431751	1.657231	0.99261813
KLK10	Wald ratio	1	-0.023801	0.044907	0.596112	0.97648	0.894206	1.066324	0.99261813
KLK14	Wald ratio	1	-0.088323	0.145306	0.543292	0.915465	0.688581	1.217106	0.99261813
KLK3	Wald ratio	1	-0.059444	0.103577	0.566027	0.942288	0.76916	1.154385	0.99261813
PRKCG	Wald ratio	1	-0.210055	0.324884	0.517922	0.81054	0.428773	1.532222	0.99261813
CACNG6	Wald ratio	1	0.0631783	0.121178	0.602111	1.065217	0.840019	1.350787	0.99261813
KIR2DS4	Wald ratio	1	-0.139586	0.197527	0.479774	0.869719	0.590528	1.280905	0.99261813
FCER2	Wald ratio	1	-0.062517	0.100473	0.533796	0.939397	0.77148	1.143863	0.99261813
FBN3	Wald ratio	1	0.0690757	0.097569	0.478967	1.071517	0.885006	1.297334	0.99261813
IL1A	Wald ratio	1	-0.056852	0.110712	0.607592	0.944734	0.760448	1.173679	0.99261813
IL36G	Wald ratio	1	-0.051086	0.102172	0.617075	0.950197	0.777756	1.160871	0.99261813
DPP10	Wald ratio	1	-0.165531	0.257824	0.520852	0.847443	0.511265	1.404672	0.99261813
C1QL2	Wald ratio	1	0.1274912	0.219677	0.561674	1.135975	0.738543	1.747277	0.99261813
TRIB2	Wald ratio	1	-0.075221	0.150441	0.617075	0.927539	0.690675	1.245633	0.99261813
UGGT1	Wald ratio	1	-0.239368	0.352198	0.496732	0.787125	0.394682	1.569786	0.99261813
KYNU	Wald ratio	1	0.0407035	0.087778	0.642856	1.041543	0.876918	1.237073	0.99261813
ACVR2A	Wald ratio	1	-0.13359	0.19853	0.501013	0.874948	0.592912	1.291144	0.99261813
PPIG	Wald ratio	1	0.1750369	0.236059	0.458392	1.19129	0.750033	1.892147	0.99261813
NT5C1B	Wald ratio	1	0.0633165	0.086559	0.464485	1.065364	0.899119	1.262347	0.99261813
DNAJC10	Wald ratio	1	-0.067405	0.136106	0.620432	0.934817	0.71593	1.220625	0.99261813
DUSP19	Wald ratio	1	0.0786445	0.141098	0.57727	1.08182	0.820446	1.42646	0.99261813
PMS1	Wald ratio	1	0.1775872	0.295611	0.548009	1.194332	0.669108	2.131838	0.99261813
ABCA12	Wald ratio	1	-0.057497	0.114294	0.614917	0.944124	0.754641	1.181185	0.99261813
WNT10A	Wald ratio	1	0.0774179	0.16966	0.648167	1.080493	0.774826	1.506746	0.99261813
ASIC4	Wald ratio	1	0.0966777	0.1493	0.517282	1.101505	0.822053	1.475955	0.99261813
COL4A4	Wald ratio	1	-0.109736	0.181017	0.54437	0.896071	0.628432	1.277694	0.99261813
ECEL1	Wald ratio	1	-0.075695	0.115389	0.511824	0.927099	0.739443	1.162378	0.99261813
TP53I3	Wald ratio	1	0.0208934	0.034076	0.539785	1.021113	0.955141	1.091642	0.99261813
GPR35	Wald ratio	1	-0.044925	0.097479	0.644892	0.956069	0.789793	1.157351	0.99261813
PASK	Wald ratio	1	0.0864787	0.121438	0.47639	1.090328	0.859383	1.383335	0.99261813
CAPN13	Wald ratio	1	-0.081103	0.157137	0.605764	0.922099	0.677672	1.254686	0.99261813
PRKD3	Wald ratio	1	0.0817239	0.122586	0.504985	1.085156	0.853385	1.379874	0.99261813
MAP4K3	Wald ratio	1	-0.132857	0.264425	0.615359	0.87559	0.521456	1.470224	0.99261813
EPCAM	Wald ratio	1	0.1019443	0.137814	0.459466	1.107322	0.84521	1.450718	0.99261813
NRXN1	Wald ratio	1	0.2017391	0.434783	0.642648	1.223529	0.521819	2.868855	0.99261813
PRADC1	Wald ratio	1	-0.043726	0.082594	0.59652	0.957216	0.814151	1.125422	0.99261813
BOLA3	Wald ratio	1	-0.09826	0.182228	0.589738	0.906413	0.634177	1.295513	0.99261813
TACR1	Wald ratio	1	0.0746211	0.144147	0.604687	1.077476	0.812282	1.42925	0.99261813
SFTPB	Wald ratio	1	0.0469551	0.08436	0.577798	1.048075	0.888349	1.23652	0.99261813
FABP1	Wald ratio	1	0.0122131	0.023252	0.599408	1.012288	0.96719	1.059489	0.99261813
IGKV1-39	Wald ratio	1	0.1421258	0.279945	0.611669	1.152722	0.665933	1.995346	0.99261813
PAK5	Wald ratio	1	0.1266167	0.196256	0.518823	1.134982	0.772561	1.667421	0.99261813
PCSK2	Wald ratio	1	0.1245979	0.265809	0.639248	1.132693	0.672746	1.907099	0.99261813
SIRPA	Wald ratio	1	-0.053257	0.08987	0.553454	0.948137	0.795008	1.130759	0.99261813
DUSP15	Wald ratio	1	-0.08282	0.153543	0.589615	0.920517	0.681292	1.243741	0.99261813
MMP24	Wald ratio	1	0.1580703	0.272671	0.56211	1.171249	0.686352	1.998718	0.99261813
SLPI	Wald ratio	1	-0.056064	0.092662	0.545151	0.945478	0.788454	1.133775	0.99261813
EDN3	Wald ratio	1	-0.109417	0.162049	0.49954	0.896356	0.652442	1.231457	0.99261813
ABCC13	Wald ratio	1	-0.00966	0.017623	0.58359	0.990387	0.956762	1.025193	0.99261813
IFNAR1	Wald ratio	1	0.067568	0.121193	0.577171	1.069903	0.843689	1.35677	0.99261813
ERG	Wald ratio	1	-0.073938	0.140901	0.599755	0.928729	0.704614	1.224128	0.99261813
PDE9A	Wald ratio	1	-0.117734	0.182863	0.519682	0.888933	0.621174	1.272111	0.99261813
SLC37A1	Wald ratio	1	-0.105266	0.149924	0.4826	0.900085	0.670913	1.20754	0.99261813
COL6A2	Wald ratio	1	-0.108187	0.164632	0.511089	0.89746	0.649946	1.239232	0.99261813
PI4KA	Wald ratio	1	-0.193794	0.273895	0.479227	0.823828	0.481606	1.409227	0.99261813
SERPIND1	Wald ratio	1	0.0496263	0.080929	0.539739	1.050878	0.896735	1.231517	0.99261813
IGLC2	Wald ratio	1	-0.046721	0.071489	0.513406	0.954354	0.829577	1.097898	0.99261813
IL2RB	Wald ratio	1	0.0573213	0.095536	0.548506	1.058996	0.878158	1.277073	0.99261813
PDGFB	Wald ratio	1	-0.063356	0.115973	0.584862	0.93861	0.747768	1.178157	0.99261813
TAB1	Wald ratio	1	-0.070736	0.147131	0.630681	0.931708	0.698295	1.243141	0.99261813
TSPO	Wald ratio	1	0.0540958	0.104757	0.60558	1.055586	0.859652	1.296177	0.99261813
FBLN1	Wald ratio	1	-0.03944	0.079538	0.619988	0.961327	0.82256	1.123506	0.99261813
CRELD2	Wald ratio	1	-0.077638	0.136119	0.568427	0.925299	0.708623	1.208228	0.99261813
MAPK11	Wald ratio	1	-0.096953	0.208951	0.642648	0.907598	0.602602	1.366962	0.99261813
TBC1D23	Wald ratio	1	0.1055748	0.22492	0.638792	1.111349	0.715146	1.727056	0.99261813

BTLA	Wald ratio	1	-0.043004	0.094242	0.648167	0.957908	0.796349	1.152243	0.99261813
TIMP4	Wald ratio	1	-0.025425	0.050544	0.614943	0.974895	0.882945	1.076421	0.99261813
TXNRD3	Wald ratio	1	-0.083208	0.133657	0.533581	0.92016	0.708097	1.195733	0.99261813
RHO	Wald ratio	1	0.0764481	0.150348	0.611121	1.079446	0.803938	1.449371	0.99261813
NEK11	Wald ratio	1	0.0538186	0.089137	0.545994	1.055293	0.886131	1.256748	0.99261813
EPHB1	Wald ratio	1	0.0547778	0.116403	0.637935	1.056306	0.840825	1.327008	0.99261813
FBLN2	Wald ratio	1	0.1608151	0.243122	0.508318	1.174468	0.729275	1.891432	0.99261813
SPSB4	Wald ratio	1	-0.079866	0.118291	0.499575	0.92324	0.732189	1.164144	0.99261813
CPB1	Wald ratio	1	0.0461107	0.099315	0.642443	1.04719	0.861959	1.272227	0.99261813
BTD	Wald ratio	1	0.0733115	0.121772	0.547147	1.076066	0.847588	1.366133	0.99261813
OTOL1	Wald ratio	1	-0.045049	0.083047	0.587507	0.955951	0.812353	1.124932	0.99261813
HTR3C	Wald ratio	1	0.0653863	0.100519	0.515379	1.067571	0.876664	1.300051	0.99261813
ADIPOQ	Wald ratio	1	0.0718597	0.104831	0.493039	1.074505	0.874933	1.319599	0.99261813
OSTN	Wald ratio	1	0.0873668	0.121854	0.473386	1.091297	0.859447	1.385692	0.99261813
UTS2B	Wald ratio	1	0.0300664	0.041443	0.468151	1.030523	0.950125	1.117724	0.99261813
GP5	Wald ratio	1	-0.067582	0.094615	0.475051	0.934651	0.776446	1.125091	0.99261813
MUC4	Wald ratio	1	0.030686	0.045204	0.497244	1.031162	0.943731	1.126692	0.99261813
TOP2B	Wald ratio	1	-0.083426	0.142466	0.558154	0.919959	0.695824	1.216292	0.99261813
NEK10	Wald ratio	1	-0.080994	0.127722	0.525985	0.922199	0.717969	1.184523	0.99261813
CCR8	Wald ratio	1	0.0274694	0.044032	0.532724	1.02785	0.942864	1.120496	0.99261813
CCK	Wald ratio	1	0.1247325	0.258218	0.62906	1.132845	0.682922	1.879188	0.99261813
PRSS45P	Wald ratio	1	-0.042257	0.071548	0.554786	0.958624	0.833192	1.102938	0.99261813
ITIH4	Wald ratio	1	-0.045592	0.099935	0.648231	0.955431	0.785476	1.16216	0.99261813
IL17RB	Wald ratio	1	-0.067892	0.124212	0.584665	0.934361	0.732459	1.191918	0.99261813
DNASE1L3	Wald ratio	1	0.0365672	0.079229	0.644412	1.037244	0.888055	1.211496	0.99261813
LMCD1	Wald ratio	1	-0.068057	0.13701	0.619378	0.934207	0.714197	1.221991	0.99261813
OR5K1	Wald ratio	1	0.0521537	0.082403	0.526792	1.053538	0.896411	1.238206	0.99261813
FGFRL1	Wald ratio	1	0.076235	0.118695	0.520694	1.079216	0.855211	1.361895	0.99261813
GAK	Wald ratio	1	0.1350789	0.208247	0.516566	1.144627	0.761029	1.721579	0.99261813
GSTCD	Wald ratio	1	0.1090662	0.228919	0.633761	1.115236	0.712044	1.746734	0.99261813
DKK2	Wald ratio	1	-0.094823	0.154191	0.538572	0.909533	0.672308	1.230464	0.99261813
PRSS12	Wald ratio	1	0.0473679	0.072112	0.511269	1.048508	0.910308	1.207688	0.99261813
UCP1	Wald ratio	1	-0.057708	0.083471	0.489341	0.943925	0.801468	1.111704	0.99261813
TLR2	Wald ratio	1	-0.075139	0.148776	0.613523	0.927614	0.69299	1.241675	0.99261813
GUCY1A1	Wald ratio	1	0.1082917	0.229431	0.636927	1.114373	0.710779	1.747135	0.99261813
GLRB	Wald ratio	1	-0.06565	0.107757	0.542363	0.936459	0.758166	1.156679	0.99261813
CPE	Wald ratio	1	0.1082449	0.157979	0.493227	1.114321	0.81759	1.518744	0.99261813
TLL1	Wald ratio	1	0.0424466	0.079234	0.592156	1.04336	0.893283	1.218651	0.99261813
AADAT	Wald ratio	1	-0.026442	0.047821	0.580304	0.973904	0.886769	1.069602	0.99261813
HPGD	Wald ratio	1	-0.0262	0.035853	0.46492	0.97414	0.908036	1.045057	0.99261813
GPM6A	Wald ratio	1	0.2937168	0.448254	0.512309	1.341404	0.557183	3.229394	0.99261813
MTNR1A	Wald ratio	1	0.0356186	0.057138	0.533037	1.036261	0.926471	1.15906	0.99261813
GRK4	Wald ratio	1	0.0382357	0.071692	0.593803	1.038976	0.902776	1.195724	0.99261813
ADRA2C	Wald ratio	1	0.1033335	0.221697	0.641143	1.108861	0.718066	1.712339	0.99261813
GABRG1	Wald ratio	1	0.197123	0.283466	0.486803	1.217894	0.698744	2.122759	0.99261813
GABRB1	Wald ratio	1	-0.091957	0.129466	0.47753	0.912144	0.707717	1.175621	0.99261813
SRD5A3	Wald ratio	1	-0.063939	0.097214	0.510719	0.938062	0.77532	1.134963	0.99261813
MAN2B2	Wald ratio	1	-0.058598	0.090928	0.519287	0.943086	0.789136	1.12707	0.99261813
TMPRSS11F	Wald ratio	1	0.0225677	0.045467	0.619647	1.022824	0.935618	1.118159	0.99261813
STATH	Wald ratio	1	-0.209234	0.30147	0.487654	0.811206	0.449278	1.464695	0.99261813
DCK	Wald ratio	1	0.2584254	0.494049	0.600921	1.29489	0.491689	3.410165	0.99261813
ADAMTS3	Wald ratio	1	-0.129104	0.17605	0.463355	0.878883	0.622406	1.241047	0.99261813
CXCL6	Wald ratio	1	-0.067579	0.097439	0.487965	0.934654	0.772163	1.131339	0.99261813
BMP3	Wald ratio	1	-0.052159	0.073519	0.478039	0.949178	0.821802	1.096297	0.99261813
FBN2	Wald ratio	1	0.0238574	0.042754	0.576837	1.024144	0.94182	1.113665	0.99261813
FSTL4	Wald ratio	1	0.0969914	0.193983	0.617075	1.101851	0.753358	1.611552	0.99261813
CATSPER3	Wald ratio	1	0.077944	0.123411	0.527662	1.081062	0.848791	1.376894	0.99261813
WNT8A	Wald ratio	1	-0.035331	0.062381	0.571141	0.965286	0.854194	1.090827	0.99261813
GFRA3	Wald ratio	1	0.0798703	0.136387	0.558133	1.083147	0.829072	1.415083	0.99261813
CAMK2A	Wald ratio	1	0.1313276	0.27122	0.628236	1.140341	0.670143	1.940448	0.99261813
ARSI	Wald ratio	1	0.0232913	0.047994	0.627468	1.023565	0.931669	1.124524	0.99261813
GABRA1	Wald ratio	1	-0.111615	0.155355	0.472482	0.894389	0.659607	1.21274	0.99261813
CNOT6	Wald ratio	1	-0.095412	0.185619	0.607239	0.908999	0.631773	1.307873	0.99261813
FLT4	Wald ratio	1	0.0862774	0.132015	0.513406	1.090109	0.841582	1.412027	0.99261813
LIFR	Wald ratio	1	-0.082219	0.166082	0.620565	0.92107	0.665152	1.275453	0.99261813

C9	Wald ratio	1	0.0409022	0.083986	0.626249	1.04175	0.883636	1.228157	0.99261813
C7	Wald ratio	1	-0.054428	0.099933	0.585999	0.947027	0.77857	1.151933	0.99261813
PRKAA1	Wald ratio	1	0.1170625	0.255029	0.646222	1.12419	0.681953	1.85321	0.99261813
BHMT2	Wald ratio	1	-0.041084	0.060884	0.499805	0.959748	0.851788	1.081391	0.99261813
VCAN	Wald ratio	1	-0.13668	0.243967	0.575315	0.872249	0.540719	1.40705	0.99261813
LNPEP	Wald ratio	1	-0.219275	0.304756	0.471825	0.8031	0.441933	1.45943	0.99261813
CD164	Wald ratio	1	0.0452341	0.08628	0.600089	1.046273	0.883491	1.239047	0.99261813
PPIL6	Wald ratio	1	-0.055448	0.089433	0.535258	0.946061	0.793949	1.127316	0.99261813
EDN1	Wald ratio	1	-0.075218	0.12317	0.541407	0.927541	0.728599	1.180803	0.99261813
LAMA2	Wald ratio	1	-0.033267	0.070116	0.635177	0.967281	0.84308	1.109778	0.99261813
TAAR9	Wald ratio	1	0.0594764	0.129505	0.646048	1.061281	0.823367	1.367941	0.99261813
TAAR3P	Wald ratio	1	0.0539185	0.111125	0.627529	1.055399	0.848839	1.312223	0.99261813
PDE7B	Wald ratio	1	-0.059272	0.092004	0.519426	0.942451	0.786944	1.128688	0.99261813
SLC22A1	Wald ratio	1	-0.032819	0.063167	0.603375	0.967714	0.855023	1.095257	0.99261813
SLC22A3	Wald ratio	1	-0.030573	0.051777	0.554871	0.969889	0.876291	1.073485	0.99261813
H4C3	Wald ratio	1	0.0493913	0.078266	0.527996	1.050631	0.901216	1.224819	0.99261813
BTN2A1	Wald ratio	1	-0.094402	0.171498	0.582005	0.909917	0.650159	1.273454	0.99261813
MYLK4	Wald ratio	1	0.014567	0.024421	0.550847	1.014674	0.96725	1.064422	0.99261813
OR1F12P	Wald ratio	1	0.0579642	0.078784	0.461892	1.059677	0.908053	1.236619	0.99261813
OR2B2	Wald ratio	1	0.0797044	0.118081	0.499676	1.082967	0.859217	1.364984	0.99261813
TUBB2B	Wald ratio	1	-0.040241	0.084207	0.632738	0.960558	0.814414	1.132928	0.99261813
ITPR3	Wald ratio	1	0.097729	0.14773	0.508267	1.102664	0.825454	1.472969	0.99261813
BRPF3	Wald ratio	1	-0.140856	0.213138	0.508696	0.868614	0.572006	1.319026	0.99261813
PI16	Wald ratio	1	0.0557947	0.10576	0.597805	1.057381	0.859422	1.300937	0.99261813
GLO1	Wald ratio	1	-0.047604	0.099348	0.63182	0.953511	0.7848	1.158491	0.99261813
TTBK1	Wald ratio	1	-0.219497	0.387347	0.570941	0.802923	0.3758	1.715499	0.99261813
CAPN11	Wald ratio	1	0.0215688	0.038464	0.57497	1.021803	0.947601	1.101815	0.99261813
HSP90AB1	Wald ratio	1	0.1359902	0.207661	0.512553	1.145671	0.762598	1.721171	0.99261813
MEP1A	Wald ratio	1	0.0693517	0.149491	0.642707	1.071813	0.799594	1.436709	0.99261813
TNFRSF21	Wald ratio	1	0.1782832	0.247468	0.47126	1.195164	0.735832	1.941227	0.99261813
TXNDC5	Wald ratio	1	-0.087327	0.145265	0.547736	0.916378	0.689323	1.218221	0.99261813
NT5E	Wald ratio	1	-0.030802	0.052182	0.555004	0.969668	0.875396	1.074091	0.99261813
GABRR1	Wald ratio	1	-0.052276	0.079893	0.512905	0.949067	0.811504	1.10995	0.99261813
GABRR2	Wald ratio	1	0.0356693	0.073717	0.628477	1.036313	0.896896	1.197401	0.99261813
PMS2P1	Wald ratio	1	0.0657035	0.091679	0.47358	1.06791	0.892269	1.278126	0.99261813
GPC2	Wald ratio	1	0.0639158	0.122877	0.602951	1.066003	0.837844	1.356292	0.99261813
MUC3A	Wald ratio	1	0.0931432	0.133599	0.485688	1.097619	0.844753	1.426177	0.99261813
LRRC17	Wald ratio	1	0.0294405	0.058056	0.612078	1.029878	0.919111	1.153994	0.99261813
NAMPT	Wald ratio	1	-0.07871	0.155906	0.613661	0.924308	0.680938	1.254661	0.99261813
NDUFA4	Wald ratio	1	0.1663386	0.233336	0.475925	1.180973	0.747516	1.865777	0.99261813
WNT16	Wald ratio	1	0.0938973	0.167255	0.574522	1.098447	0.791424	1.524576	0.99261813
AHCYL2	Wald ratio	1	0.1062846	0.214695	0.620565	1.112138	0.730141	1.69399	0.99261813
MGAM	Wald ratio	1	-0.016894	0.029047	0.560814	0.983248	0.928834	1.040849	0.99261813
PRSS37	Wald ratio	1	-0.022989	0.039928	0.564779	0.977273	0.903709	1.056826	0.99261813
OR9A4	Wald ratio	1	-0.028235	0.049411	0.567709	0.97216	0.882426	1.071019	0.99261813
EPHA1	Wald ratio	1	-0.06128	0.123357	0.619348	0.94056	0.738555	1.197814	0.99261813
OR2A7	Wald ratio	1	-0.035992	0.069268	0.603337	0.964648	0.842183	1.10492	0.99261813
CDK5	Wald ratio	1	0.2029917	0.393606	0.606048	1.225062	0.566388	2.649733	0.99261813
SHH	Wald ratio	1	0.0892063	0.146354	0.542177	1.093306	0.820659	1.456535	0.99261813
AQP1	Wald ratio	1	-0.068753	0.141062	0.625978	0.933557	0.708054	1.230879	0.99261813
INHBA	Wald ratio	1	-0.108491	0.159187	0.495537	0.897187	0.65672	1.225705	0.99261813
UPP1	Wald ratio	1	0.0941294	0.202583	0.642185	1.098702	0.738649	1.634263	0.99261813
EIF2AK1	Wald ratio	1	-0.058963	0.10845	0.586655	0.942742	0.762216	1.166023	0.99261813
VKORC1L1	Wald ratio	1	0.0581523	0.093396	0.53352	1.059876	0.882581	1.272787	0.99261813
FZD9	Wald ratio	1	-0.095251	0.183286	0.603283	0.909145	0.634771	1.302114	0.99261813
STX1A	Wald ratio	1	0.0539074	0.102238	0.598004	1.055387	0.863744	1.289551	0.99261813
SSC4D	Wald ratio	1	0.0941692	0.132633	0.477704	1.098746	0.847223	1.424939	0.99261813
CDK14	Wald ratio	1	0.1641338	0.271763	0.54587	1.178372	0.691757	2.007296	0.99261813
CYP3A4	Wald ratio	1	-0.060052	0.112145	0.592315	0.941716	0.755893	1.17322	0.99261813
SLC25A32	Wald ratio	1	0.043476	0.067771	0.521192	1.044435	0.91452	1.192806	0.99261813
COLEC10	Wald ratio	1	0.0483697	0.103378	0.639863	1.049559	0.857056	1.285299	0.99261813
DERL1	Wald ratio	1	-0.082851	0.174272	0.634495	0.920489	0.654147	1.295274	0.99261813
KCNQ3	Wald ratio	1	0.1049396	0.224354	0.639969	1.110644	0.715486	1.724044	0.99261813
ST3GAL1	Wald ratio	1	-0.052565	0.100656	0.601516	0.948793	0.778917	1.155717	0.99261813
CYP11B1	Wald ratio	1	0.0484483	0.083683	0.562625	1.049641	0.890857	1.236726	0.99261813

NRBP2	Wald ratio	1	0.4550683	0.728952	0.532445	1.576281	0.377691	6.57855	0.99261813
MAPK15	Wald ratio	1	0.1351648	0.250846	0.590001	1.144725	0.700127	1.871656	0.99261813
PARP10	Wald ratio	1	0.0561769	0.110193	0.610189	1.057785	0.852313	1.312791	0.99261813
MSR1	Wald ratio	1	0.0592297	0.098223	0.546499	1.061019	0.875214	1.286269	0.99261813
TNFRSF10C	Wald ratio	1	0.0564039	0.099665	0.571439	1.058025	0.87028	1.286271	0.99261813
GSR	Wald ratio	1	-0.057869	0.097933	0.554581	0.943773	0.778943	1.143483	0.99261813
SARAF	Wald ratio	1	0.1380506	0.239671	0.564615	1.148034	0.717699	1.836398	0.99261813
NRG1	Wald ratio	1	0.1247259	0.252519	0.621358	1.132838	0.690589	1.858301	0.99261813
PLAT	Wald ratio	1	0.1359288	0.232605	0.558968	1.1456	0.726165	1.807303	0.99261813
CA2	Wald ratio	1	-0.096255	0.140258	0.49254	0.908232	0.689933	1.195602	0.99261813
CCNE2	Wald ratio	1	-0.154242	0.267895	0.564779	0.857064	0.506964	1.448938	0.99261813
CTSV	Wald ratio	1	-0.076153	0.153548	0.619925	0.926674	0.685843	1.252073	0.99261813
TGFBR1	Wald ratio	1	-0.13764	0.238756	0.564286	0.871412	0.545746	1.391417	0.99261813
OR13C4	Wald ratio	1	0.0458247	0.069356	0.508796	1.046891	0.913827	1.19933	0.99261813
OR13C9	Wald ratio	1	-0.06016	0.105916	0.570035	0.941614	0.765095	1.158858	0.99261813
OR13D1	Wald ratio	1	-0.111892	0.164108	0.495354	0.894141	0.648208	1.233382	0.99261813
OR13F1	Wald ratio	1	-0.108894	0.193907	0.574405	0.896826	0.613269	1.311491	0.99261813
FKBP15	Wald ratio	1	-0.037368	0.070585	0.59652	0.963321	0.838857	1.106252	0.99261813
TYRP1	Wald ratio	1	-0.050138	0.070061	0.474214	0.951098	0.829064	1.091094	0.99261813
OR1L4	Wald ratio	1	0.0675036	0.126533	0.593696	1.069834	0.834852	1.370955	0.99261813
OR1L1	Wald ratio	1	-0.056026	0.076777	0.465555	0.945514	0.813419	1.099061	0.99261813
TOR2A	Wald ratio	1	0.0901791	0.172423	0.600966	1.09437	0.78054	1.534381	0.99261813
SLC27A4	Wald ratio	1	0.2070671	0.360582	0.565794	1.230065	0.606728	2.493802	0.99261813
SLC25A25	Wald ratio	1	0.1371021	0.240628	0.568835	1.146945	0.715675	1.838101	0.99261813
PTGES	Wald ratio	1	-0.105312	0.163523	0.519561	0.900043	0.653236	1.240101	0.99261813
OBP2B	Wald ratio	1	0.0709239	0.117748	0.546948	1.0735	0.852262	1.352168	0.99261813
LCN1	Wald ratio	1	-0.078309	0.145431	0.590258	0.924679	0.69534	1.229658	0.99261813
IFNW1	Wald ratio	1	-0.06576	0.096505	0.495608	0.936355	0.774986	1.131326	0.99261813
IFNK	Wald ratio	1	0.0781513	0.148705	0.599203	1.081286	0.807906	1.447173	0.99261813
MYORG	Wald ratio	1	0.1130896	0.164042	0.490575	1.119732	0.811856	1.544364	0.99261813
IL11RA	Wald ratio	1	0.1136058	0.157671	0.471202	1.12031	0.822481	1.525987	0.99261813
TLN1	Wald ratio	1	-0.126381	0.236454	0.593008	0.881279	0.554422	1.400836	0.99261813
INSL6	Wald ratio	1	0.0717826	0.104223	0.490986	1.074422	0.875908	1.317926	0.99261813
ALDH1A1	Wald ratio	1	-0.095344	0.194554	0.624087	0.90906	0.620848	1.331067	0.99261813
NTRK2	Wald ratio	1	0.2337864	0.325984	0.473268	1.263375	0.666882	2.3934	0.99261813
IARS1	Wald ratio	1	-0.102496	0.145401	0.480861	0.902582	0.678764	1.200202	0.99261813
PGD	Inverse variance weighted	2	0.0780061	0.131801	0.553953	1.081129	0.834999	1.39981	0.99261813
AMY2B	Inverse variance weighted	2	-0.054063	0.116959	0.64391	0.947372	0.753292	1.191457	0.99261813
DRAXIN	Inverse variance weighted	2	0.0417529	0.079659	0.600176	1.042637	0.89192	1.218821	0.99261813
SLC25A24	Inverse variance weighted	2	0.015618	0.028909	0.589022	1.015741	0.959788	1.074955	0.99261813
OLFML3	Inverse variance weighted	2	-0.076049	0.128263	0.55324	0.926771	0.720763	1.19166	0.99261813
NPPB	Inverse variance weighted	2	0.0299295	0.065292	0.646668	1.030382	0.90661	1.171051	0.99261813
CASQ2	Inverse variance weighted	2	-0.041418	0.082248	0.614563	0.959428	0.816586	1.127258	0.99261813
DHRS3	Inverse variance weighted	2	-0.063788	0.128909	0.620721	0.938204	0.728732	1.207888	0.99261813
CHRNB2	Inverse variance weighted	2	-0.040643	0.075335	0.589544	0.960172	0.828367	1.112949	0.99261813
SLC25A44	Inverse variance weighted	2	0.4356718	0.629837	0.489112	1.546001	0.449864	5.312979	0.99261813
DUSP23	Inverse variance weighted	3	-0.049916	0.070454	0.478642	0.951309	0.828609	1.092179	0.99261813
CD84	Inverse variance weighted	2	-0.186262	0.263293	0.479297	0.830056	0.495437	1.390678	0.99261813
TSTD1	Inverse variance weighted	2	-0.039933	0.07391	0.588998	0.960854	0.831273	1.110634	0.99261813
ATP1B1	Inverse variance weighted	2	-0.10719	0.225208	0.634102	0.898355	0.577759	1.396847	0.99261813
RNASEL	Inverse variance weighted	2	-0.011018	0.022744	0.628095	0.989043	0.945921	1.034131	0.99261813
AKR7L	Inverse variance weighted	2	-0.058483	0.08941	0.513046	0.943194	0.791579	1.12385	0.99261813
LAMB3	Inverse variance weighted	2	0.0279561	0.060476	0.643887	1.028351	0.913405	1.157762	0.99261813
CELA3B	Inverse variance weighted	4	0.0850569	0.1376	0.536479	1.088779	0.831404	1.425828	0.99261813
IARS2	Inverse variance weighted	3	-0.063791	0.127159	0.615905	0.938201	0.731234	1.203749	0.99261813
GALNT2	Inverse variance weighted	4	-0.057198	0.088211	0.516712	0.944407	0.79446	1.122655	0.99261813
KDM1A	Inverse variance weighted	2	-0.063012	0.129916	0.62766	0.938932	0.727859	1.211214	0.99261813
OR2L13	Inverse variance weighted	3	0.0205436	0.044623	0.64524	1.020756	0.935273	1.114052	0.99261813
OR2L3	Inverse variance weighted	3	0.0273668	0.059547	0.645815	1.027745	0.91453	1.154975	0.99261813
OR2T8	Inverse variance weighted	2	0.0330728	0.05632	0.557046	1.033626	0.9256	1.15426	0.99261813
PTPRU	Inverse variance weighted	2	-0.045022	0.085877	0.6001	0.955977	0.80788	1.131221	0.99261813
BMP8B	Inverse variance weighted	2	-0.033708	0.053426	0.528087	0.966854	0.870731	1.073588	0.99261813
SLC1A7	Inverse variance weighted	2	0.0094534	0.01708	0.579941	1.009498	0.976263	1.043865	0.99261813
ICMT	Inverse variance weighted	2	0.1053049	0.185583	0.570424	1.111049	0.772257	1.598472	0.99261813
UTS2	Inverse variance weighted	4	-0.028823	0.052038	0.579663	0.971589	0.877378	1.075916	0.99261813

ADGRL2	Inverse variance weighted	2	0.1243281	0.235292	0.597222	1.132387	0.71402	1.795888	0.99261813
SLC2A5	Inverse variance weighted	2	-0.058353	0.112867	0.605152	0.943317	0.756107	1.17688	0.99261813
LPAR3	Inverse variance weighted	3	0.0385354	0.072672	0.595927	1.039288	0.901314	1.198382	0.99261813
MCOLN2	Inverse variance weighted	2	0.0109832	0.022761	0.629419	1.011044	0.966931	1.057169	0.99261813
CDC7	Inverse variance weighted	2	0.0283812	0.049736	0.568248	1.028788	0.933232	1.134128	0.99261813
WNT8B	Inverse variance weighted	2	0.0687081	0.108139	0.525187	1.071123	0.866543	1.324003	0.99261813
GPR26	Inverse variance weighted	3	0.0627887	0.110113	0.568527	1.064802	0.858102	1.321292	0.99261813
NPS	Inverse variance weighted	3	-0.065727	0.115021	0.567703	0.936386	0.74739	1.173175	0.99261813
STK32C	Inverse variance weighted	2	0.0688884	0.099086	0.486907	1.071317	0.882214	1.300954	0.99261813
ITGB1	Inverse variance weighted	2	0.1110864	0.15924	0.485425	1.117491	0.817893	1.526834	0.99261813
IL2RA	Inverse variance weighted	2	0.0597118	0.082475	0.469064	1.061531	0.903085	1.247775	0.99261813
SFTPD	Inverse variance weighted	3	-0.029817	0.055615	0.591872	0.970623	0.870382	1.082409	0.99261813
OPN4	Inverse variance weighted	3	0.0131555	0.023736	0.579417	1.013242	0.967183	1.061495	0.99261813
AMPD3	Inverse variance weighted	2	-0.022199	0.031786	0.484949	0.978046	0.918972	1.040918	0.99261813
CD151	Inverse variance weighted	2	0.0622413	0.103965	0.549389	1.064219	0.868029	1.304751	0.99261813
TMPRSS5	Inverse variance weighted	2	0.0118643	0.019214	0.536918	1.011935	0.974535	1.050771	0.99261813
NXPE1	Inverse variance weighted	3	0.0373646	0.079668	0.639066	1.038071	0.887999	1.213506	0.99261813
TMEM25	Inverse variance weighted	2	0.0634712	0.137785	0.645047	1.065529	0.813355	1.395887	0.99261813
ABCG4	Inverse variance weighted	2	0.1188468	0.251292	0.636254	1.126197	0.688194	1.84297	0.99261813
CALCB	Inverse variance weighted	2	0.0533434	0.088087	0.544794	1.054792	0.887536	1.253567	0.99261813
CTSD	Inverse variance weighted	2	0.1424395	0.239762	0.552453	1.153083	0.720728	1.844803	0.99261813
IGF2	Inverse variance weighted	2	-0.041984	0.081923	0.608311	0.958885	0.816644	1.125902	0.99261813
CD81	Inverse variance weighted	2	-0.120595	0.174181	0.488715	0.886393	0.630029	1.247073	0.99261813
WT1	Inverse variance weighted	2	0.0360479	0.052824	0.494974	1.036705	0.93474	1.149793	0.99261813
MRGPRE	Inverse variance weighted	2	-0.051282	0.071767	0.474886	0.950011	0.825352	1.093499	0.99261813
TRIM6	Inverse variance weighted	2	-0.051022	0.090593	0.573296	0.950257	0.795659	1.134895	0.99261813
OR52E4	Inverse variance weighted	2	-0.038771	0.060112	0.518948	0.961971	0.855054	1.082258	0.99261813
GANAB	Inverse variance weighted	2	-0.038372	0.074011	0.60413	0.962355	0.832408	1.112588	0.99261813
CTSF	Inverse variance weighted	2	0.0574488	0.086099	0.504617	1.059131	0.894666	1.253829	0.99261813
OR2D2	Inverse variance weighted	2	0.0596151	0.090453	0.509848	1.061428	0.888987	1.267317	0.99261813
GPR83	Inverse variance weighted	2	0.0325361	0.054075	0.547383	1.033071	0.929182	1.148576	0.99261813
P2RX4	Inverse variance weighted	2	0.0204653	0.03132	0.513485	1.020676	0.959904	1.085296	0.99261813
NCOR2	Inverse variance weighted	2	0.1914399	0.307983	0.53421	1.210992	0.662187	2.214633	0.99261813
MMP17	Inverse variance weighted	2	0.1152828	0.196568	0.557554	1.122191	0.763387	1.649637	0.99261813
SLCO1A2	Inverse variance weighted	2	0.0910207	0.125232	0.467337	1.095292	0.856901	1.400003	0.99261813
SPX	Inverse variance weighted	3	-0.062243	0.086696	0.472788	0.939654	0.792814	1.113692	0.99261813
SLC2A13	Inverse variance weighted	2	0.1959419	0.309176	0.526241	1.216456	0.663621	2.229834	0.99261813
IL23A	Inverse variance weighted	2	0.0342204	0.061264	0.57645	1.034813	0.917726	1.166837	0.99261813
NALCN	Inverse variance weighted	2	-0.093858	0.131344	0.474857	0.910412	0.703778	1.177715	0.99261813
COL4A2	Inverse variance weighted	2	0.0793662	0.108437	0.464224	1.082601	0.875316	1.338973	0.99261813
EBPL	Inverse variance weighted	2	-0.016672	0.031641	0.598251	0.983466	0.924328	1.046388	0.99261813
UCHL3	Inverse variance weighted	3	0.0223334	0.043409	0.606908	1.022585	0.939181	1.113396	0.99261813
EDNRB	Inverse variance weighted	2	-0.079009	0.162907	0.627678	0.924031	0.671456	1.271615	0.99261813
GPC6	Inverse variance weighted	2	0.1173622	0.190114	0.537021	1.124527	0.774714	1.632293	0.99261813
RNASE2	Inverse variance weighted	2	0.0777009	0.110813	0.483186	1.080799	0.869799	1.342986	0.99261813
SLC7A7	Inverse variance weighted	2	0.0417902	0.088064	0.635114	1.042676	0.877379	1.239114	0.99261813
CBLN3	Inverse variance weighted	2	0.0373632	0.061632	0.54436	1.03807	0.919951	1.171356	0.99261813
SSTR1	Inverse variance weighted	2	0.1228281	0.175588	0.484223	1.13069	0.801457	1.595169	0.99261813
PYGL	Inverse variance weighted	2	0.0360864	0.056932	0.52618	1.036745	0.927279	1.159135	0.99261813
CHGA	Inverse variance weighted	2	-0.097847	0.166159	0.555947	0.906788	0.654739	1.255866	0.99261813
LRRK1	Inverse variance weighted	2	-0.1235	0.200069	0.537047	0.883822	0.597121	1.308179	0.99261813
SLC12A1	Inverse variance weighted	3	0.0397748	0.056179	0.478945	1.040576	0.93208	1.161702	0.99261813
NOX5	Inverse variance weighted	2	-0.083131	0.135897	0.540725	0.920231	0.705048	1.201088	0.99261813
CHRNB4	Inverse variance weighted	2	-0.077005	0.138912	0.579342	0.925885	0.705201	1.21563	0.99261813
SULT1A3	Inverse variance weighted	2	-0.055706	0.081645	0.495054	0.945817	0.805952	1.109954	0.99261813
ITGAD	Inverse variance weighted	2	-0.033215	0.055071	0.546421	0.96733	0.868355	1.077587	0.99261813
CES2	Inverse variance weighted	2	0.1122047	0.180877	0.535037	1.118742	0.784809	1.594762	0.99261813
RANBP10	Inverse variance weighted	2	-0.118586	0.195017	0.543133	0.888175	0.606034	1.301668	0.99261813
CDH1	Inverse variance weighted	2	-0.03518	0.063611	0.580236	0.965432	0.852265	1.093626	0.99261813
MC1R	Inverse variance weighted	2	0.0312268	0.067076	0.641545	1.031719	0.904618	1.176679	0.99261813
SERPINF2	Inverse variance weighted	2	0.0393508	0.059439	0.50795	1.040135	0.925751	1.168653	0.99261813
TRIM16L	Inverse variance weighted	3	-0.020248	0.031618	0.521916	0.979956	0.921071	1.042605	0.99261813
CAMKK1	Inverse variance weighted	2	0.1887471	0.411719	0.646639	1.207735	0.538901	2.706664	0.99261813
KRT19	Inverse variance weighted	2	-0.035643	0.075144	0.635263	0.964985	0.832831	1.118109	0.99261813
ABCC3	Inverse variance weighted	2	-0.05272	0.114877	0.646289	0.948646	0.757389	1.1882	0.99261813

CA4	Inverse variance weighted	2	0.032354	0.055436	0.559469	1.032883	0.926538	1.151434	0.99261813
SLC16A6	Inverse variance weighted	2	-0.045	0.083753	0.591067	0.955998	0.811269	1.126546	0.99261813
ABCA10	Inverse variance weighted	2	0.1069725	0.186117	0.565455	1.112904	0.772736	1.602817	0.99261813
TRIM65	Inverse variance weighted	2	-0.021965	0.036653	0.548995	0.978275	0.910461	1.051139	0.99261813
NOTUM	Inverse variance weighted	2	-0.059168	0.100939	0.557756	0.942548	0.773361	1.148748	0.99261813
SERPINB8	Inverse variance weighted	2	-0.071395	0.137138	0.602639	0.931094	0.711639	1.218225	0.99261813
OR7D2	Inverse variance weighted	2	0.0091483	0.018741	0.625452	1.00919	0.972793	1.04695	0.99261813
TYK2	Inverse variance weighted	2	-0.08529	0.138178	0.537072	0.918246	0.70039	1.203867	0.99261813
ADGRE5	Inverse variance weighted	2	-0.059889	0.123778	0.628497	0.941869	0.738972	1.200474	0.99261813
OR7C1	Inverse variance weighted	2	0.0294525	0.041343	0.476223	1.02989	0.949728	1.11682	0.99261813
CYP4F3	Inverse variance weighted	2	-0.016128	0.03124	0.605673	0.984001	0.925558	1.046135	0.99261813
KLHL26	Inverse variance weighted	2	0.080787	0.127989	0.527908	1.08414	0.843605	1.393258	0.99261813
FSD1	Inverse variance weighted	3	-0.080298	0.143405	0.575522	0.922842	0.69672	1.222351	0.99261813
SEMA6B	Inverse variance weighted	2	0.1623075	0.314569	0.605877	1.176222	0.634926	2.178992	0.99261813
APOC1	Inverse variance weighted	3	0.0643647	0.116528	0.580705	1.066481	0.848717	1.34012	0.99261813
DMPK	Inverse variance weighted	2	0.159677	0.255649	0.532237	1.173132	0.710778	1.936241	0.99261813
KLK13	Inverse variance weighted	2	-0.145789	0.26561	0.583086	0.86434	0.513562	1.454711	0.99261813
KLK5	Inverse variance weighted	2	-0.017705	0.036279	0.625535	0.982451	0.915019	1.054853	0.99261813
KLK2	Inverse variance weighted	2	0.0789749	0.127135	0.534474	1.082177	0.843488	1.38841	0.99261813
FPR2	Inverse variance weighted	2	-0.037359	0.076228	0.624069	0.963331	0.829638	1.118567	0.99261813
NRTN	Inverse variance weighted	2	-0.047313	0.076928	0.538536	0.953789	0.820294	1.109009	0.99261813
AURKC	Inverse variance weighted	2	-0.036759	0.059527	0.536893	0.963909	0.85776	1.083193	0.99261813
TUBB4A	Inverse variance weighted	2	0.1346179	0.19514	0.490287	1.144099	0.780472	1.677145	0.99261813
SLC25A41	Inverse variance weighted	3	0.00979	0.01959	0.617249	1.009838	0.9718	1.049365	0.99261813
MCOLN1	Inverse variance weighted	3	-0.136321	0.220854	0.537074	0.872562	0.56598	1.345214	0.99261813
NMS	Inverse variance weighted	2	0.0851494	0.139717	0.542232	1.08888	0.828038	1.43189	0.99261813
FBLN7	Inverse variance weighted	2	-0.032594	0.045039	0.469254	0.967931	0.886149	1.057261	0.99261813
SCTR	Inverse variance weighted	2	0.0480455	0.070804	0.49741	1.049218	0.913264	1.205412	0.99261813
CYP27C1	Inverse variance weighted	2	0.0564923	0.084197	0.502249	1.058118	0.897149	1.24797	0.99261813
KCNJ3	Inverse variance weighted	2	-0.125944	0.189218	0.505663	0.881664	0.608468	1.277522	0.99261813
PLA2R1	Inverse variance weighted	2	-0.035506	0.076354	0.64192	0.965117	0.830971	1.120918	0.99261813
SLC25A12	Inverse variance weighted	2	-0.040316	0.087833	0.646227	0.960486	0.808586	1.140921	0.99261813
ITGA4	Inverse variance weighted	2	-0.032866	0.064747	0.611723	0.967668	0.85234	1.0986	0.99261813
MFSD6	Inverse variance weighted	3	-0.074596	0.147493	0.613022	0.928118	0.695112	1.239228	0.99261813
MARS2	Inverse variance weighted	2	-0.055561	0.077041	0.470799	0.945955	0.813376	1.100143	0.99261813
ATIC	Inverse variance weighted	2	0.0346115	0.057751	0.548955	1.035217	0.924428	1.159284	0.99261813
IGFBP5	Inverse variance weighted	2	0.0577798	0.116807	0.620839	1.059482	0.842685	1.332053	0.99261813
SERPINE2	Inverse variance weighted	2	0.0895648	0.128303	0.485134	1.093698	0.850518	1.406409	0.99261813
KLHL29	Inverse variance weighted	2	0.0744459	0.145393	0.608629	1.077287	0.810159	1.432494	0.99261813
SPP2	Inverse variance weighted	2	0.0522824	0.088278	0.553685	1.053673	0.886262	1.252708	0.99261813
PDCD1	Inverse variance weighted	2	-0.03328	0.064426	0.605464	0.967268	0.852522	1.097457	0.99261813
ADCY3	Inverse variance weighted	2	0.0402627	0.079658	0.613247	1.041084	0.890593	1.217005	0.99261813
SLC4A5	Inverse variance weighted	2	-0.053099	0.075795	0.483577	0.948286	0.817376	1.100163	0.99261813
GNLY	Inverse variance weighted	2	-0.01446	0.020611	0.482932	0.985644	0.94662	1.026276	0.99261813
ENTPD6	Inverse variance weighted	2	0.0475453	0.0953	0.617849	1.048694	0.870017	1.264065	0.99261813
KCNG1	Inverse variance weighted	2	-0.226808	0.479727	0.636366	0.797074	0.311277	2.041034	0.99261813
MC3R	Inverse variance weighted	2	-0.031292	0.068024	0.645502	0.969192	0.848217	1.107421	0.99261813
NTSR1	Inverse variance weighted	3	0.0482749	0.08258	0.558829	1.049459	0.892631	1.233841	0.99261813
IFNGR2	Inverse variance weighted	2	0.0716202	0.125085	0.566934	1.074247	0.840678	1.37271	0.99261813
IL10RB	Inverse variance weighted	3	0.019529	0.033897	0.564535	1.019721	0.954173	1.089772	0.99261813
TFF1	Inverse variance weighted	2	-0.247051	0.403005	0.539863	0.781101	0.354537	1.720887	0.99261813
MICAL3	Inverse variance weighted	2	0.1335105	0.291368	0.646794	1.142833	0.645603	2.023019	0.99261813
TUBA8	Inverse variance weighted	3	0.0528719	0.111053	0.634006	1.054295	0.84807	1.310667	0.99261813
SLC25A1	Inverse variance weighted	3	0.0237104	0.038509	0.538085	1.023994	0.94955	1.104274	0.99261813
GRK3	Inverse variance weighted	2	-0.095225	0.144478	0.509834	0.909169	0.684956	1.206775	0.99261813
EMID1	Inverse variance weighted	3	0.0821503	0.138761	0.553833	1.085619	0.827107	1.424929	0.99261813
XBP1	Inverse variance weighted	2	-0.066315	0.100259	0.508334	0.935836	0.768878	1.139048	0.99261813
SLC5A4	Inverse variance weighted	2	0.012468	0.018234	0.494114	1.012546	0.976998	1.049387	0.99261813
TXN2	Inverse variance weighted	2	-0.066988	0.145168	0.644475	0.935207	0.703621	1.243016	0.99261813
CSNK1E	Inverse variance weighted	2	0.2226022	0.377601	0.555515	1.249323	0.596011	2.61876	0.99261813
CYB5R3	Inverse variance weighted	2	0.3037435	0.471231	0.519203	1.354921	0.538015	3.412197	0.99261813
CELSR1	Inverse variance weighted	2	-0.072015	0.107018	0.500992	0.930517	0.754447	1.147676	0.99261813
TAFA5	Inverse variance weighted	2	0.1047805	0.152446	0.491875	1.110467	0.823647	1.497166	0.99261813
CD80	Inverse variance weighted	2	0.0418811	0.076167	0.582414	1.04277	0.898161	1.210663	0.99261813
GPR156	Inverse variance weighted	2	-0.038451	0.071782	0.592191	0.962279	0.835986	1.107651	0.99261813

IL20RB	Inverse variance weighted	2	-0.043075	0.068486	0.529379	0.95784	0.837522	1.095442	0.99261813
GPR160	Inverse variance weighted	2	0.0624719	0.111044	0.573717	1.064465	0.856266	1.323286	0.99261813
NCEH1	Inverse variance weighted	2	-0.054018	0.087078	0.535032	0.947415	0.798763	1.123731	0.99261813
ATP13A5	Inverse variance weighted	2	-0.026292	0.057177	0.645642	0.974051	0.870786	1.089562	0.99261813
TNK2	Inverse variance weighted	3	-0.165971	0.257653	0.519469	0.847071	0.511212	1.403584	0.99261813
PAK2	Inverse variance weighted	2	-0.068746	0.123971	0.579217	0.933564	0.73218	1.190339	0.99261813
GLB1	Inverse variance weighted	2	0.0222233	0.031684	0.483049	1.022472	0.960908	1.087981	0.99261813
DCLK3	Inverse variance weighted	2	-0.139724	0.202449	0.490089	0.869598	0.584777	1.293145	0.99261813
XCR1	Inverse variance weighted	2	0.0798126	0.133319	0.549401	1.083084	0.834024	1.406519	0.99261813
ADAMTS9	Inverse variance weighted	3	0.052131	0.106491	0.624463	1.053514	0.855054	1.298037	0.99261813
MITF	Inverse variance weighted	2	0.089885	0.185012	0.627086	1.094048	0.761291	1.572253	0.99261813
WDR1	Inverse variance weighted	2	0.0616131	0.112135	0.582692	1.063551	0.853704	1.324979	0.99261813
PDE6B	Inverse variance weighted	2	0.0736246	0.109021	0.499468	1.076403	0.86931	1.332831	0.99261813
NDNF	Inverse variance weighted	2	-0.214748	0.314848	0.495196	0.806745	0.435243	1.495342	0.99261813
MGST2	Inverse variance weighted	3	-0.117958	0.160723	0.462995	0.888733	0.648576	1.217815	0.99261813
GYPE	Inverse variance weighted	2	0.0390709	0.062448	0.531544	1.039844	0.920049	1.175237	0.99261813
PRIMPOL	Inverse variance weighted	4	-0.027243	0.038	0.473426	0.973125	0.903281	1.04837	0.99261813
FAT1	Inverse variance weighted	2	-0.04118	0.089423	0.645155	0.959657	0.805373	1.143496	0.99261813
TRIML2	Inverse variance weighted	3	-0.032929	0.053888	0.541158	0.967607	0.87062	1.075399	0.99261813
SLIT2	Inverse variance weighted	2	0.0529051	0.109579	0.629234	1.05433	0.850553	1.306928	0.99261813
IGFBP7	Inverse variance weighted	2	-0.023475	0.049667	0.636468	0.976798	0.886191	1.07667	0.99261813
SPARCL1	Inverse variance weighted	2	-0.091147	0.13279	0.492462	0.912884	0.703692	1.184264	0.99261813
MAN2A1	Inverse variance weighted	2	-0.080033	0.15139	0.597045	0.923086	0.686082	1.241961	0.99261813
ALDH7A1	Inverse variance weighted	4	-0.049595	0.094176	0.598462	0.951615	0.791219	1.144527	0.99261813
CSF1R	Inverse variance weighted	2	-0.176955	0.294674	0.548165	0.837817	0.470238	1.492729	0.99261813
GPX3	Inverse variance weighted	3	-0.029713	0.06146	0.628773	0.970724	0.860558	1.094993	0.99261813
HMMR	Inverse variance weighted	2	-0.040198	0.084137	0.632816	0.960599	0.81456	1.132821	0.99261813
BTNL8	Inverse variance weighted	2	-0.024296	0.046252	0.599379	0.975997	0.891411	1.068609	0.99261813
BTNL3	Inverse variance weighted	2	-0.013165	0.022306	0.555035	0.986921	0.944703	1.031025	0.99261813
PDZD2	Inverse variance weighted	2	0.0730381	0.120776	0.545352	1.075772	0.849011	1.363097	0.99261813
PLK2	Inverse variance weighted	2	0.0598899	0.127394	0.638273	1.06172	0.827122	1.362857	0.99261813
MTRR	Inverse variance weighted	4	0.0237439	0.039999	0.552774	1.024028	0.946812	1.107541	0.99261813
F2RL1	Inverse variance weighted	2	-0.026302	0.038966	0.499668	0.974041	0.90242	1.051345	0.99261813
REV3L	Inverse variance weighted	2	0.0316933	0.053855	0.556205	1.032201	0.928799	1.147115	0.99261813
HDAC2	Inverse variance weighted	2	-0.100571	0.167339	0.547838	0.904321	0.651449	1.255349	0.99261813
IL20RA	Inverse variance weighted	2	-0.034584	0.063408	0.585468	0.966008	0.853113	1.093841	0.99261813
ULBP2	Inverse variance weighted	2	-0.054761	0.103227	0.595771	0.946711	0.773301	1.159008	0.99261813
LPA	Inverse variance weighted	2	0.0629907	0.101774	0.535965	1.065017	0.872418	1.300135	0.99261813
RNASET2	Inverse variance weighted	2	0.0410919	0.083267	0.621662	1.041948	0.88505	1.22666	0.99261813
THBS2	Inverse variance weighted	3	-0.024171	0.048444	0.617818	0.976119	0.8877	1.073345	0.99261813
RIPK1	Inverse variance weighted	2	0.0994521	0.169224	0.556737	1.104566	0.792766	1.538997	0.99261813
BPHL	Inverse variance weighted	3	-0.035629	0.049838	0.474667	0.964998	0.875192	1.064019	0.99261813
GPLD1	Inverse variance weighted	2	-0.061431	0.096726	0.525357	0.940417	0.778012	1.136725	0.99261813
BTN3A2	Inverse variance weighted	2	-0.01136	0.016211	0.483444	0.988704	0.957784	1.020623	0.99261813
BTN2A2	Inverse variance weighted	2	-0.051239	0.08854	0.562784	0.950052	0.798695	1.130092	0.99261813
MLN	Inverse variance weighted	2	-0.087086	0.152072	0.566872	0.916598	0.680351	1.234881	0.99261813
PPARD	Inverse variance weighted	2	0.2434109	0.483611	0.614739	1.275593	0.494372	3.291317	0.99261813
ADCY10P1	Inverse variance weighted	3	-0.048868	0.066288	0.460994	0.952307	0.83628	1.084432	0.99261813
SLC29A1	Inverse variance weighted	2	-0.128508	0.178236	0.470908	0.879406	0.620115	1.247116	0.99261813
ENPP5	Inverse variance weighted	2	0.0281481	0.059144	0.634129	1.028548	0.915967	1.154966	0.99261813
ADGRF4	Inverse variance weighted	2	0.0222033	0.039523	0.574267	1.022452	0.946237	1.104805	0.99261813
FARS2	Inverse variance weighted	2	-0.065112	0.111029	0.557581	0.936963	0.753724	1.164749	0.99261813
GSTA3	Inverse variance weighted	2	-0.147586	0.22219	0.50654	0.862788	0.558178	1.333632	0.99261813
SLC17A5	Inverse variance weighted	2	-0.0668	0.130314	0.608226	0.935382	0.724542	1.207576	0.99261813
PRSS35	Inverse variance weighted	2	-0.100647	0.143961	0.484474	0.904252	0.681943	1.199034	0.99261813
AZGP1	Inverse variance weighted	2	0.03044	0.06214	0.624234	1.030908	0.912693	1.164434	0.99261813
PMPCB	Inverse variance weighted	2	0.1337965	0.218184	0.539726	1.14316	0.745393	1.75319	0.99261813
CPA2	Inverse variance weighted	2	-0.05347	0.086661	0.537232	0.947934	0.799855	1.123429	0.99261813
FAM20C	Inverse variance weighted	2	-0.095526	0.197377	0.628401	0.908894	0.617309	1.33821	0.99261813
TRPV5	Inverse variance weighted	2	-0.103812	0.180147	0.564439	0.901395	0.633244	1.283097	0.99261813
KEL	Inverse variance weighted	2	0.0610019	0.087491	0.485656	1.062901	0.895404	1.261731	0.99261813
PDIA4	Inverse variance weighted	2	0.0388439	0.060184	0.518656	1.039608	0.923932	1.169767	0.99261813
ASIC3	Inverse variance weighted	2	-0.06691	0.118597	0.57263	0.935279	0.741292	1.18003	0.99261813
ABCF2	Inverse variance weighted	2	0.0697688	0.122562	0.569185	1.07226	0.843283	1.363413	0.99261813
EPDR1	Inverse variance weighted	2	-0.031972	0.054167	0.555024	0.968534	0.870977	1.077017	0.99261813

GCK	Inverse variance weighted	2	0.0774371	0.109166	0.478105	1.080514	0.872382	1.338302	0.99261813
TAC1	Inverse variance weighted	2	-0.077619	0.116301	0.50452	0.925317	0.736705	1.162219	0.99261813
LMTK2	Inverse variance weighted	2	0.1639386	0.32594	0.614984	1.178142	0.621945	2.231739	0.99261813
OR4F21	Inverse variance weighted	2	0.0191284	0.033773	0.571132	1.019312	0.954024	1.089069	0.99261813
TRHR	Inverse variance weighted	2	-0.057337	0.081077	0.479446	0.944276	0.805536	1.106911	0.99261813
MTMR7	Inverse variance weighted	2	-0.032072	0.067271	0.633528	0.968436	0.848807	1.104926	0.99261813
NAT1	Inverse variance weighted	2	-0.053029	0.085682	0.535981	0.948353	0.801744	1.12177	0.99261813
OPRK1	Inverse variance weighted	2	0.0887283	0.160649	0.580734	1.092784	0.797604	1.497204	0.99261813
CA13	Inverse variance weighted	2	-0.047641	0.08363	0.568906	0.953476	0.809324	1.123304	0.99261813
FKTN	Inverse variance weighted	2	-0.035986	0.074612	0.629582	0.964653	0.833414	1.11656	0.99261813
TXN	Inverse variance weighted	2	0.0371066	0.066418	0.576379	1.037804	0.911127	1.182092	0.99261813
PTGR1	Inverse variance weighted	3	0.0342497	0.062594	0.584262	1.034843	0.915363	1.169919	0.99261813
SLC46A2	Inverse variance weighted	2	-0.013905	0.029843	0.641263	0.986191	0.930161	1.045597	0.99261813
COL5A1	Inverse variance weighted	2	-0.054265	0.080325	0.499318	0.947181	0.809205	1.108683	0.99261813
S1PR3	Inverse variance weighted	3	-0.047393	0.0945	0.616008	0.953712	0.79246	1.147777	0.99261813
CD109	Inverse variance weighted	2	-0.040249	0.088442	0.649047	0.96055	0.807675	1.142361	0.99314328
SLC22A13	Wald ratio	1	-0.04159	0.091498	0.649436	0.959263	0.801775	1.147685	0.99330556
ASTL	Wald ratio	1	-0.059283	0.130568	0.649801	0.94244	0.729646	1.217294	0.99331934
PRRT3	Wald ratio	1	-0.14193	0.312797	0.650013	0.867682	0.470005	1.601838	0.99331934
CALR	Wald ratio	1	0.0653848	0.144391	0.650671	1.06757	0.804429	1.416787	0.99345803
PCOLCE2	Wald ratio	1	-0.016885	0.037288	0.650671	0.983257	0.91396	1.057808	0.99345803
MFAP2	Wald ratio	1	-0.040198	0.092549	0.664039	0.960599	0.80124	1.151653	0.99357155
ANGEL2	Wald ratio	1	-0.068168	0.154118	0.658267	0.934104	0.690569	1.263523	0.99357155
CAPN9	Wald ratio	1	-0.032054	0.07285	0.659937	0.968454	0.839591	1.117096	0.99357155
DPYD	Wald ratio	1	0.1227539	0.274472	0.654705	1.130606	0.660201	1.936185	0.99357155
PSAP	Wald ratio	1	-0.105276	0.236334	0.655991	0.900076	0.566379	1.430379	0.99357155
KIF11	Wald ratio	1	-0.140103	0.324258	0.66569	0.869269	0.460405	1.641225	0.99357155
SCN2B	Wald ratio	1	-0.12913	0.295564	0.662189	0.87886	0.492414	1.568588	0.99357155
DCPS	Wald ratio	1	-0.05863	0.135059	0.66421	0.943055	0.723724	1.228858	0.99357155
SIRT3	Wald ratio	1	-0.074783	0.170505	0.660954	0.927945	0.664332	1.296162	0.99357155
SLC15A3	Wald ratio	1	-0.049423	0.112456	0.660306	0.951778	0.763504	1.18648	0.99357155
TPCN2	Wald ratio	1	0.0300332	0.069873	0.667323	1.030489	0.898599	1.181736	0.99357155
ABCC4	Wald ratio	1	0.0718205	0.160587	0.654705	1.074462	0.784326	1.471926	0.99357155
CYP46A1	Wald ratio	1	0.1631508	0.361015	0.651324	1.177214	0.580168	2.388676	0.99357155
HERC1	Wald ratio	1	-0.128264	0.288594	0.656721	0.879621	0.49962	1.548644	0.99357155
NR2F2	Wald ratio	1	-0.110607	0.248465	0.656203	0.89529	0.550131	1.457007	0.99357155
PRPSAP2	Wald ratio	1	-0.022401	0.052095	0.667196	0.977848	0.882932	1.082968	0.99357155
OMG	Wald ratio	1	0.0550451	0.121946	0.651709	1.056588	0.831961	1.341864	0.99357155
VAMP2	Wald ratio	1	-0.103382	0.239846	0.666443	0.901783	0.563561	1.442987	0.99357155
CYP2A7	Wald ratio	1	0.0191137	0.042908	0.655991	1.019298	0.93708	1.108728	0.99357155
CFC1	Wald ratio	1	-0.063176	0.145522	0.664189	0.938778	0.705817	1.248629	0.99357155
MPST	Wald ratio	1	-0.096172	0.218091	0.659233	0.908308	0.592366	1.392758	0.99357155
PIK3CB	Wald ratio	1	0.1062764	0.236746	0.6535	1.112129	0.699251	1.768794	0.99357155
MASP1	Wald ratio	1	-0.06778	0.154449	0.660771	0.934466	0.69039	1.264833	0.99357155
SLC49A3	Wald ratio	1	-0.060014	0.138033	0.66372	0.941751	0.718523	1.234332	0.99357155
UBA6	Wald ratio	1	0.086375	0.198663	0.66372	1.090215	0.738596	1.609227	0.99357155
CXCL13	Wald ratio	1	0.044259	0.098792	0.654153	1.045253	0.861247	1.268572	0.99357155
BMPR1B	Wald ratio	1	0.0395869	0.09149	0.665238	1.040381	0.86959	1.244715	0.99357155
GPX8	Wald ratio	1	-0.049052	0.113034	0.664316	0.952131	0.762923	1.188265	0.99357155
PCSK1	Wald ratio	1	0.0747536	0.169008	0.658267	1.077619	0.773753	1.500817	0.99357155
GPR85	Wald ratio	1	0.0759224	0.171651	0.658267	1.078879	0.770656	1.510374	0.99357155
CEP41	Wald ratio	1	0.0674182	0.153343	0.660187	1.069743	0.792047	1.444801	0.99357155
FKBP9	Wald ratio	1	0.0276891	0.062147	0.655926	1.028076	0.910175	1.161249	0.99357155
PSMA2	Wald ratio	1	-0.072565	0.168045	0.665874	0.930005	0.669026	1.29279	0.99357155
AOPEP	Wald ratio	1	0.0646647	0.150071	0.666545	1.066801	0.794951	1.431616	0.99357155
TRIM17	Inverse variance weighted	2	-0.099248	0.224369	0.65824	0.905518	0.583325	1.405671	0.99357155
IL15RA	Inverse variance weighted	2	0.0348045	0.08109	0.667773	1.035417	0.883263	1.213782	0.99357155
ANGPTL5	Inverse variance weighted	3	-0.023568	0.052537	0.653714	0.976707	0.881138	1.082641	0.99357155
BACE1	Inverse variance weighted	3	0.0948925	0.220427	0.666837	1.099541	0.713805	1.693725	0.99357155
OR52N5	Inverse variance weighted	2	0.0203412	0.045514	0.65493	1.02055	0.933452	1.115774	0.99357155
GPRC5D	Inverse variance weighted	2	-0.024835	0.056251	0.658849	0.975471	0.873641	1.08917	0.99357155
POLE	Inverse variance weighted	2	0.0199943	0.04466	0.654367	1.020196	0.934691	1.113521	0.99357155
IL32	Inverse variance weighted	2	0.0332694	0.074386	0.654694	1.033829	0.893572	1.196101	0.99357155
PLAUR	Inverse variance weighted	2	0.160818	0.371712	0.665275	1.174471	0.566807	2.433604	0.99357155
IGFL1	Inverse variance weighted	2	0.0312132	0.070826	0.659428	1.031705	0.897982	1.185343	0.99357155

IGFBP2	Inverse variance weighted	3	0.0368198	0.085129	0.665363	1.037506	0.878067	1.225896	0.99357155
CYP1B1	Inverse variance weighted	2	0.0274891	0.060821	0.651294	1.02787	0.91236	1.158005	0.99357155
CCN5	Inverse variance weighted	3	0.0421935	0.097643	0.665655	1.043096	0.861408	1.263107	0.99357155
BCL2L13	Inverse variance weighted	2	-0.026953	0.0627	0.667291	0.973407	0.860841	1.100692	0.99357155
APOL1	Inverse variance weighted	2	0.0368068	0.083537	0.659497	1.037493	0.8808	1.222061	0.99357155
APOBEC3G	Inverse variance weighted	2	0.0121406	0.02731	0.656641	1.012215	0.959459	1.067871	0.99357155
PLD1	Inverse variance weighted	2	0.0804588	0.180996	0.656656	1.083784	0.76011	1.545288	0.99357155
QRFPR	Inverse variance weighted	3	-0.025715	0.059874	0.667565	0.974612	0.866695	1.095967	0.99357155
CPA5	Inverse variance weighted	2	-0.02992	0.06871	0.663238	0.970524	0.84824	1.110435	0.99357155
PDE1C	Inverse variance weighted	3	-0.120912	0.272474	0.657219	0.886112	0.519462	1.511553	0.99357155
DAGLB	Inverse variance weighted	2	0.0419489	0.096584	0.664052	1.042841	0.862987	1.260179	0.99357155
LPL	Inverse variance weighted	2	0.0362961	0.080629	0.652593	1.036963	0.885382	1.214495	0.99357155
PEBP4	Inverse variance weighted	4	-0.010577	0.024522	0.666213	0.989478	0.943047	1.038196	0.99357155
PENK	Inverse variance weighted	2	0.0450835	0.103636	0.663551	1.046115	0.853812	1.28173	0.99357155
LAMC3	Inverse variance weighted	2	0.0270133	0.061795	0.662006	1.027381	0.910188	1.159665	0.99357155
ECM2	Inverse variance weighted	2	-0.04825	0.110091	0.661188	0.952895	0.767951	1.18238	0.99357155
BICD1	Inverse variance weighted	2	0.1761479	0.410807	0.66808	1.192614	0.533107	2.668001	0.99360528
CR2	Wald ratio	1	0.0268691	0.063192	0.670693	1.027233	0.907567	1.162678	0.99399033
OR2T10	Wald ratio	1	-0.025563	0.060126	0.670728	0.974761	0.866398	1.096677	0.99399033
HSP90B1	Wald ratio	1	0.0385865	0.090633	0.670295	1.039341	0.87018	1.241385	0.99399033
SCARB1	Wald ratio	1	0.0973623	0.22859	0.670162	1.10226	0.704213	1.725296	0.99399033
TGM5	Wald ratio	1	-0.015199	0.035763	0.670837	0.984916	0.918242	1.05643	0.99399033
CPA4	Wald ratio	1	0.0307809	0.072299	0.670295	1.03126	0.895005	1.188257	0.99399033
ALDH4A1	Inverse variance weighted	2	0.0212811	0.050083	0.670894	1.021509	0.926	1.126869	0.99399033
EPHA3	Inverse variance weighted	2	0.0333989	0.078333	0.669836	1.033963	0.886802	1.205544	0.99399033
CCND3	Inverse variance weighted	2	-0.064264	0.150724	0.669841	0.937758	0.697897	1.260056	0.99399033
VCAM1	Wald ratio	1	-0.007823	0.093872	0.933586	0.992208	0.825461	1.192638	0.99410703
CORT	Wald ratio	1	-0.033488	0.196985	0.86501	0.967067	0.657324	1.422767	0.99410703
GSTM4	Wald ratio	1	0.0104638	0.057275	0.855039	1.010519	0.903214	1.130572	0.99410703
GSTM5	Wald ratio	1	0.0093812	0.086441	0.913578	1.009425	0.852107	1.195788	0.99410703
AMPD2	Wald ratio	1	0.08578	0.282569	0.761454	1.089567	0.626219	1.895752	0.99410703
SLC6A17	Wald ratio	1	0.1198339	0.356078	0.736465	1.12731	0.560975	2.265389	0.99410703
SLC16A4	Wald ratio	1	0.0106531	0.113456	0.925191	1.01071	0.809191	1.262415	0.99410703
KCNA10	Wald ratio	1	-0.047668	0.119171	0.689157	0.95345	0.754844	1.20431	0.99410703
KCND3	Wald ratio	1	0.1030536	0.282961	0.715711	1.108551	0.636641	1.930264	0.99410703
HIPK1	Wald ratio	1	0.0906532	0.230754	0.694425	1.094889	0.696544	1.721043	0.99410703
SLC22A15	Wald ratio	1	0.049867	0.199468	0.802587	1.051131	0.710995	1.553988	0.99410703
BOLA1	Wald ratio	1	-0.03126	0.196607	0.873672	0.969224	0.659278	1.424885	0.99410703
H3C14	Wald ratio	1	0.0260608	0.188289	0.889918	1.026403	0.709648	1.484543	0.99410703
H3C15	Wald ratio	1	0.0246541	0.178126	0.889918	1.024961	0.722908	1.45322	0.99410703
ARNT	Wald ratio	1	0.0236787	0.060715	0.696537	1.023961	0.90908	1.15336	0.99410703
PIP5K1A	Wald ratio	1	-0.063902	0.160288	0.690136	0.938097	0.685185	1.284362	0.99410703
APH1A	Wald ratio	1	-0.15098	0.373536	0.686072	0.859865	0.413495	1.788094	0.99410703
MCL1	Wald ratio	1	0.0121885	0.224726	0.956746	1.012263	0.651633	1.572476	0.99410703
CTRC	Wald ratio	1	0.0265452	0.093738	0.777034	1.026901	0.854549	1.234013	0.99410703
S100A9	Wald ratio	1	0.0368514	0.11209	0.742332	1.037539	0.832898	1.292459	0.99410703
S100A8	Wald ratio	1	0.0400929	0.121949	0.742332	1.040907	0.819609	1.321957	0.99410703
S100A12	Wald ratio	1	0.0203788	0.058339	0.726853	1.020588	0.910313	1.144221	0.99410703
HCN3	Wald ratio	1	-0.052667	0.180346	0.77026	0.948695	0.666213	1.350954	0.99410703
FCRL6	Wald ratio	1	-0.028031	0.088171	0.750546	0.972358	0.818038	1.155789	0.99410703
ITLN1	Wald ratio	1	0.0137229	0.136315	0.919812	1.013818	0.776116	1.32432	0.99410703
FCGR2C	Wald ratio	1	-0.051963	0.123273	0.673372	0.949364	0.745592	1.208829	0.99410703
ITLN2	Wald ratio	1	0.0341368	0.094938	0.719168	1.034726	0.859039	1.246344	0.99410703
MPZ	Wald ratio	1	-0.011896	0.13156	0.927949	0.988174	0.763568	1.278849	0.99410703
RGS4	Wald ratio	1	0.0828328	0.252048	0.742429	1.08636	0.662866	1.780417	0.99410703
CDK11A	Wald ratio	1	0.0480229	0.129865	0.711538	1.049195	0.813417	1.353316	0.99410703
CD247	Wald ratio	1	-0.027967	0.096135	0.771121	0.972421	0.80542	1.174049	0.99410703
DPT	Wald ratio	1	-0.042995	0.149767	0.77405	0.957916	0.714238	1.28473	0.99410703
F5	Wald ratio	1	-0.008701	0.0266	0.74359	0.991337	0.940978	1.044391	0.99410703
SELL	Wald ratio	1	-0.011127	0.042283	0.792429	0.988934	0.910281	1.074385	0.99410703
FASLG	Wald ratio	1	-0.023141	0.071327	0.745614	0.977125	0.849641	1.123738	0.99410703
TNN	Wald ratio	1	-0.005707	0.102212	0.955471	0.994309	0.813798	1.21486	0.99410703
ABL2	Wald ratio	1	-0.034923	0.137365	0.799312	0.96568	0.737744	1.264038	0.99410703
SOAT1	Wald ratio	1	0.0139257	0.210875	0.947348	1.014023	0.670729	1.533022	0.99410703
ODR4	Wald ratio	1	-0.081318	0.275371	0.767763	0.921901	0.537383	1.581557	0.99410703

CFHR1	Wald ratio	1	0.0021109	0.033511	0.949773	1.002113	0.938409	1.070142	0.99410703
CRB1	Wald ratio	1	-0.010861	0.128158	0.932464	0.989198	0.769472	1.271668	0.99410703
CACNA1S	Wald ratio	1	-0.122506	0.299835	0.682849	0.884701	0.491555	1.592286	0.99410703
PTPN7	Wald ratio	1	0.0389835	0.098903	0.693462	1.039753	0.85653	1.26217	0.99410703
DSTYK	Wald ratio	1	-0.035178	0.296084	0.905424	0.965433	0.540369	1.724862	0.99410703
PM20D1	Wald ratio	1	-0.005744	0.05687	0.919542	0.994272	0.889398	1.111512	0.99410703
AVPR1B	Wald ratio	1	-0.009362	0.074152	0.899532	0.990682	0.856672	1.145655	0.99410703
CD55	Wald ratio	1	-0.00945	0.040378	0.814952	0.990594	0.915219	1.072177	0.99410703
NEK2	Wald ratio	1	0.0175125	0.119085	0.883086	1.017667	0.805821	1.285206	0.99410703
RPS6KC1	Wald ratio	1	0.0372944	0.206101	0.856405	1.037999	0.693043	1.554652	0.99410703
HSPG2	Wald ratio	1	0.0163466	0.150004	0.913223	1.016481	0.757553	1.363909	0.99410703
TNFRSF14	Wald ratio	1	-0.193249	0.605449	0.749588	0.824277	0.251596	2.700485	0.99410703
LEFTY2	Wald ratio	1	-0.016447	0.052995	0.756299	0.983688	0.886638	1.09136	0.99410703
PARP1	Wald ratio	1	0.0589413	0.164545	0.720187	1.060713	0.768307	1.464405	0.99410703
COQ8A	Wald ratio	1	-0.009655	0.036765	0.792839	0.990391	0.921534	1.064392	0.99410703
WNT9A	Wald ratio	1	0.0444571	0.130855	0.73405	1.04546	0.80895	1.351118	0.99410703
HTR1D	Wald ratio	1	-0.016784	0.045361	0.711382	0.983356	0.899702	1.074789	0.99410703
FUCA1	Wald ratio	1	-0.040236	0.219063	0.85427	0.960563	0.625252	1.475694	0.99410703
OPN3	Wald ratio	1	0.0335622	0.143199	0.814694	1.034132	0.781057	1.369207	0.99410703
AKT3	Wald ratio	1	0.0651743	0.238223	0.784404	1.067345	0.669153	1.702489	0.99410703
OR14K1	Wald ratio	1	0.0159486	0.11802	0.892505	1.016076	0.806243	1.280521	0.99410703
OR2T3	Wald ratio	1	-0.040542	0.102129	0.69139	0.960269	0.786065	1.173078	0.99410703
FGR	Wald ratio	1	0.0184351	0.120267	0.878174	1.018606	0.804698	1.289377	0.99410703
GPR3	Wald ratio	1	-0.020458	0.313331	0.947941	0.97975	0.530154	1.810623	0.99410703
TINAGL1	Wald ratio	1	-0.062518	0.172429	0.716924	0.939396	0.67	1.317113	0.99410703
AZIN2	Wald ratio	1	0.0194238	0.085465	0.820212	1.019614	0.862356	1.205549	0.99410703
TP73	Wald ratio	1	0.043514	0.113592	0.701665	1.044475	0.836001	1.304936	0.99410703
PTPRF	Wald ratio	1	-0.017683	0.267767	0.947348	0.982473	0.581289	1.660537	0.99410703
TESK2	Wald ratio	1	-0.011359	0.037646	0.762862	0.988705	0.918379	1.064418	0.99410703
CYP4Z1	Wald ratio	1	-0.031776	0.121429	0.793566	0.968724	0.763549	1.229031	0.99410703
CYP4X1	Wald ratio	1	0.0188529	0.164963	0.909011	1.019032	0.737511	1.408015	0.99410703
TXNDC12	Wald ratio	1	-0.010435	0.096286	0.913699	0.989619	0.819423	1.195166	0.99410703
LRP8	Wald ratio	1	0.0047259	0.078764	0.952156	1.004737	0.861007	1.17246	0.99410703
PCSK9	Wald ratio	1	-0.005199	0.063682	0.934939	0.994815	0.878082	1.127067	0.99410703
C8B	Wald ratio	1	0.0327786	0.117551	0.780363	1.033322	0.820681	1.301058	0.99410703
CLCA4	Wald ratio	1	-0.024634	0.108176	0.819862	0.975667	0.78926	1.206099	0.99410703
TNFRSF9	Wald ratio	1	0.0243612	0.091181	0.789334	1.02466	0.85697	1.225164	0.99410703
ABCA4	Wald ratio	1	-0.017974	0.095339	0.850465	0.982187	0.814779	1.183991	0.99410703
F3	Wald ratio	1	0.0638592	0.153087	0.676574	1.065942	0.789629	1.438945	0.99410703
HPSE2	Wald ratio	1	0.0289717	0.076466	0.704776	1.029396	0.88612	1.195836	0.99410703
ABCC2	Wald ratio	1	-0.026686	0.111192	0.81033	0.973667	0.783	1.210763	0.99410703
KCNK18	Wald ratio	1	0.0300081	0.184304	0.870661	1.030463	0.718042	1.478818	0.99410703
PRLHR	Wald ratio	1	-0.017586	0.080161	0.826348	0.982567	0.839708	1.149732	0.99410703
OAT	Wald ratio	1	0.0496149	0.142229	0.727212	1.050866	0.795205	1.388723	0.99410703
CDNF	Wald ratio	1	-0.042097	0.134184	0.75373	0.958777	0.737051	1.247203	0.99410703
CACNB2	Wald ratio	1	0.0316715	0.077802	0.683949	1.032178	0.886194	1.202212	0.99410703
PTPN20	Wald ratio	1	-0.045973	0.137919	0.738883	0.955068	0.728846	1.251506	0.99410703
SLC18A3	Wald ratio	1	0.0432276	0.123433	0.72618	1.044176	0.819795	1.32997	0.99410703
SLC25A16	Wald ratio	1	0.0356055	0.116707	0.760302	1.036247	0.824367	1.302585	0.99410703
HKDC1	Wald ratio	1	-0.029027	0.08645	0.737047	0.97139	0.819985	1.150751	0.99410703
SPOCK2	Wald ratio	1	0.0783377	0.189157	0.678771	1.081488	0.746463	1.566878	0.99410703
VSIR	Wald ratio	1	-0.047767	0.208316	0.818636	0.953356	0.633772	1.434093	0.99410703
COMTD1	Wald ratio	1	-0.030232	0.088853	0.733669	0.97022	0.815149	1.154792	0.99410703
PPIF	Wald ratio	1	0.0120316	0.117308	0.918309	1.012104	0.804212	1.273738	0.99410703
GRID1	Wald ratio	1	-0.014656	0.110966	0.894925	0.985451	0.792827	1.224874	0.99410703
LIPF	Wald ratio	1	0.0316771	0.101568	0.755131	1.032184	0.845865	1.259544	0.99410703
LGI1	Wald ratio	1	0.0213576	0.158758	0.892984	1.021587	0.748407	1.394484	0.99410703
PDE6C	Wald ratio	1	-0.015042	0.134378	0.910871	0.98507	0.756977	1.281893	0.99410703
CYP2C8	Wald ratio	1	-0.022871	0.083317	0.783693	0.977388	0.83013	1.150769	0.99410703
MUC6	Wald ratio	1	-0.00915	0.099435	0.926679	0.990891	0.815428	1.204111	0.99410703
PGR	Wald ratio	1	-0.021449	0.134416	0.873216	0.978779	0.752085	1.273802	0.99410703
BIRC3	Wald ratio	1	0.0647246	0.212823	0.761034	1.066865	0.702992	1.619081	0.99410703
CASP12	Wald ratio	1	0.0389215	0.108867	0.720708	1.039689	0.839912	1.286984	0.99410703
CASP1	Wald ratio	1	0.0113882	0.165671	0.945197	1.011453	0.731011	1.399483	0.99410703
CASP5	Wald ratio	1	-0.033571	0.096983	0.729227	0.966986	0.799589	1.169429	0.99410703

WEE1	Wald ratio	1	0.0453789	0.115608	0.694672	1.046424	0.834258	1.312548	0.99410703
SCGB1C1	Wald ratio	1	0.0252917	0.181403	0.889116	1.025614	0.718739	1.463515	0.99410703
DRD2	Wald ratio	1	0.0123922	0.17349	0.943057	1.012469	0.720616	1.422525	0.99410703
SIK3	Wald ratio	1	-0.045964	0.343634	0.893594	0.955077	0.487002	1.873034	0.99410703
PCSK7	Wald ratio	1	-0.052295	0.240876	0.828126	0.949048	0.591903	1.52169	0.99410703
MPZL3	Wald ratio	1	-0.025957	0.072546	0.720494	0.974377	0.845229	1.123258	0.99410703
CXCR5	Wald ratio	1	0.04282	0.107599	0.69066	1.04375	0.845291	1.288803	0.99410703
OR10G6	Wald ratio	1	-0.008075	0.066533	0.903396	0.991957	0.87068	1.130127	0.99410703
OR8G1	Wald ratio	1	0.0250327	0.119677	0.834316	1.025349	0.810962	1.296411	0.99410703
ADAMTS8	Wald ratio	1	-0.06986	0.347118	0.840497	0.932524	0.472267	1.841334	0.99410703
BRSK2	Wald ratio	1	-0.067231	0.414004	0.870996	0.934979	0.415331	2.104791	0.99410703
SAA4	Wald ratio	1	0.0228641	0.087011	0.792725	1.023127	0.86271	1.213374	0.99410703
KCNC1	Wald ratio	1	-0.057166	0.328081	0.861675	0.944438	0.496484	1.796558	0.99410703
SAA1	Wald ratio	1	0.0255154	0.066907	0.702939	1.025844	0.899764	1.16959	0.99410703
PRMT3	Wald ratio	1	0.0177744	0.133901	0.894396	1.017933	0.782962	1.323421	0.99410703
SLC22A18	Wald ratio	1	-0.010558	0.055672	0.849579	0.989497	0.887209	1.103579	0.99410703
CD82	Wald ratio	1	-0.020226	0.153141	0.894925	0.979977	0.725872	1.323037	0.99410703
OR52I2	Wald ratio	1	0.0223039	0.064681	0.730224	1.022554	0.900801	1.160765	0.99410703
OR51A6P	Wald ratio	1	0.0533703	0.142586	0.70818	1.05482	0.797639	1.394923	0.99410703
TRIM49B	Wald ratio	1	0.0442567	0.123388	0.719834	1.045251	0.820712	1.331221	0.99410703
OR56A4	Wald ratio	1	-0.054345	0.141701	0.701337	0.947106	0.717432	1.250306	0.99410703
OR52H1	Wald ratio	1	-0.010822	0.075034	0.885318	0.989236	0.853945	1.145962	0.99410703
OR5D17P	Wald ratio	1	0.0394758	0.111342	0.72293	1.040265	0.836311	1.293959	0.99410703
OR4P1P	Wald ratio	1	-0.004276	0.066577	0.948795	0.995734	0.873921	1.134526	0.99410703
OR4C6	Wald ratio	1	0.0194706	0.094187	0.836226	1.019661	0.847779	1.226392	0.99410703
OR10Q1	Wald ratio	1	0.01135	0.109916	0.917756	1.011415	0.815393	1.254561	0.99410703
OR56B3P	Wald ratio	1	-0.013028	0.079587	0.869975	0.987057	0.844494	1.153686	0.99410703
OR52D1	Wald ratio	1	-0.012753	0.066316	0.847501	0.987328	0.866985	1.124375	0.99410703
OR52E8	Wald ratio	1	-0.005365	0.036123	0.881936	0.99465	0.926662	1.067625	0.99410703
OR51I2	Wald ratio	1	-0.019431	0.051992	0.7086	0.980756	0.885737	1.085969	0.99410703
CBLIF	Wald ratio	1	-0.005379	0.096442	0.95552	0.994635	0.823324	1.201592	0.99410703
SCGB1D4	Wald ratio	1	0.04148	0.113379	0.714474	1.042352	0.83465	1.301741	0.99410703
SCGB1D2	Wald ratio	1	0.0281732	0.08452	0.738883	1.028574	0.871547	1.213892	0.99410703
RTN3	Wald ratio	1	-0.111848	0.345983	0.746486	0.89418	0.453857	1.761698	0.99410703
PLCB3	Wald ratio	1	0.0264012	0.114121	0.817048	1.026753	0.820964	1.284127	0.99410703
CDC42BPG	Wald ratio	1	-0.033444	0.08078	0.678865	0.967109	0.825494	1.133019	0.99410703
MAP3K11	Wald ratio	1	0.0483215	0.261953	0.853648	1.049508	0.628068	1.753738	0.99410703
RELA	Wald ratio	1	0.101973	0.275603	0.711382	1.107354	0.645191	1.900571	0.99410703
OR2D3	Wald ratio	1	0.0876987	0.228319	0.7009	1.091659	0.697811	1.707797	0.99410703
DEFB108B	Wald ratio	1	0.0326827	0.108079	0.762351	1.033223	0.835978	1.277006	0.99410703
FOLR1	Wald ratio	1	-0.012781	0.142721	0.928643	0.9873	0.746385	1.305978	0.99410703
PDE2A	Wald ratio	1	-0.041591	0.121653	0.732441	0.959262	0.75576	1.217561	0.99410703
RELT	Wald ratio	1	0.0533656	0.220348	0.808635	1.054815	0.684877	1.624578	0.99410703
CHRDL2	Wald ratio	1	-0.028854	0.10099	0.775097	0.971558	0.797085	1.184222	0.99410703
CAPN5	Wald ratio	1	0.0560612	0.150408	0.709351	1.057662	0.787621	1.42029	0.99410703
FZD4	Wald ratio	1	-0.015446	0.114741	0.892916	0.984673	0.786361	1.232996	0.99410703
GRM5	Wald ratio	1	-0.108174	0.305291	0.723091	0.897472	0.493346	1.632636	0.99410703
OLR1	Wald ratio	1	0.0295325	0.099426	0.766444	1.029973	0.847603	1.251581	0.99410703
KLRD1	Wald ratio	1	-0.003357	0.060432	0.955696	0.996648	0.885322	1.121973	0.99410703
CMKLR1	Wald ratio	1	0.0183649	0.061019	0.763437	1.018535	0.903723	1.147932	0.99410703
PRB3	Wald ratio	1	-0.005979	0.100941	0.952766	0.994039	0.815606	1.211508	0.99410703
CIT	Wald ratio	1	0.0926564	0.308855	0.764177	1.097085	0.598877	2.009752	0.99410703
CAMKK2	Wald ratio	1	-0.03811	0.128304	0.766444	0.962607	0.748574	1.237837	0.99410703
ABCB9	Wald ratio	1	0.0102575	0.173352	0.952815	1.01031	0.719274	1.419106	0.99410703
ADGRD1	Wald ratio	1	0.0383704	0.116998	0.742944	1.039116	0.826177	1.306938	0.99410703
GUCY2C	Wald ratio	1	-0.035888	0.092166	0.696995	0.964749	0.805306	1.155759	0.99410703
TULP3	Wald ratio	1	0.0153573	0.036931	0.677526	1.015476	0.944569	1.091706	0.99410703
STK38L	Wald ratio	1	-0.047351	0.201898	0.814575	0.953753	0.642062	1.416755	0.99410703
FGF23	Wald ratio	1	0.0081444	0.082123	0.921	1.008178	0.858287	1.184245	0.99410703
NELL2	Wald ratio	1	-0.014461	0.234594	0.950846	0.985643	0.622342	1.561025	0.99410703
HDAC7	Wald ratio	1	-0.060332	0.178124	0.734828	0.941452	0.664012	1.334812	0.99410703
ACVR1B	Wald ratio	1	-0.040532	0.227749	0.85875	0.960279	0.614516	1.500588	0.99410703
IGFBP6	Wald ratio	1	-0.082951	0.219291	0.70523	0.920396	0.598839	1.414619	0.99410703
KRT18	Wald ratio	1	-0.026628	0.123582	0.829401	0.973723	0.764258	1.240598	0.99410703
PDE1B	Wald ratio	1	-0.022001	0.228007	0.92313	0.97824	0.625694	1.529426	0.99410703

SUOX	Wald ratio	1	0.0638225	0.191021	0.738295	1.065903	0.733022	1.549952	0.99410703
OR6C76	Wald ratio	1	-0.021865	0.137708	0.873847	0.978373	0.746938	1.281516	0.99410703
SPRYD4	Wald ratio	1	-0.013217	0.105735	0.900524	0.98687	0.802151	1.214126	0.99410703
HSD17B6	Wald ratio	1	-0.003483	0.038722	0.928329	0.996523	0.923691	1.075098	0.99410703
VWF	Wald ratio	1	-0.068992	0.17164	0.687713	0.933334	0.666706	1.306591	0.99410703
TAPBPL	Wald ratio	1	0.0027475	0.028225	0.922452	1.002751	0.948785	1.059787	0.99410703
LYZ	Wald ratio	1	0.0140786	0.044961	0.754181	1.014178	0.928631	1.107606	0.99410703
KCNC2	Wald ratio	1	-0.051709	0.122353	0.672573	0.949606	0.747127	1.206957	0.99410703
PTPRQ	Wald ratio	1	0.0366799	0.105548	0.728202	1.037361	0.8435	1.275776	0.99410703
KERA	Wald ratio	1	-0.037112	0.10189	0.71568	0.963568	0.789136	1.176557	0.99410703
CDK17	Wald ratio	1	-0.08838	0.265139	0.738883	0.915413	0.544411	1.539245	0.99410703
SLC25A3	Wald ratio	1	-0.015544	0.110365	0.887992	0.984576	0.793057	1.222345	0.99410703
LATS2	Wald ratio	1	0.0301201	0.202235	0.881604	1.030578	0.693322	1.531888	0.99410703
RXFP2	Wald ratio	1	-0.016781	0.042884	0.695572	0.983359	0.904083	1.069587	0.99410703
CCNA1	Wald ratio	1	-0.015	0.248332	0.951835	0.985112	0.605482	1.602765	0.99410703
CPB2	Wald ratio	1	-0.007859	0.119538	0.947582	0.992172	0.784936	1.254121	0.99410703
RB1	Wald ratio	1	-0.065875	0.206775	0.750042	0.936248	0.624282	1.404109	0.99410703
OXGR1	Wald ratio	1	0.0029292	0.033198	0.92969	1.002934	0.939752	1.070363	0.99410703
HHIPL1	Wald ratio	1	-0.029184	0.078336	0.709484	0.971238	0.833	1.132417	0.99410703
MOK	Wald ratio	1	-0.028383	0.143096	0.842773	0.972016	0.734291	1.286706	0.99410703
AKT1	Wald ratio	1	-0.020953	0.200795	0.916893	0.979265	0.660663	1.451512	0.99410703
IGHA2	Wald ratio	1	0.015613	0.184383	0.932518	1.015736	0.707671	1.457908	0.99410703
RNASE4	Wald ratio	1	-0.017071	0.060358	0.777308	0.983074	0.873389	1.106533	0.99410703
OR11H4	Wald ratio	1	-0.010143	0.06897	0.883086	0.989909	0.864743	1.133192	0.99410703
SLC7A8	Wald ratio	1	-0.073394	0.228522	0.748083	0.929235	0.59375	1.454278	0.99410703
DHRS1	Wald ratio	1	-0.008947	0.041754	0.830324	0.991093	0.913213	1.075613	0.99410703
ADCY4	Wald ratio	1	-0.025612	0.143709	0.858552	0.974714	0.735443	1.291828	0.99410703
RIPK3	Wald ratio	1	0.0077568	0.116352	0.946847	1.007787	0.802284	1.265929	0.99410703
GZMH	Wald ratio	1	-0.034678	0.087418	0.691593	0.965916	0.813818	1.14644	0.99410703
CTSG	Wald ratio	1	0.0404123	0.148888	0.786061	1.04124	0.777706	1.394076	0.99410703
RABGGTA	Wald ratio	1	-0.039478	0.27059	0.884003	0.961291	0.565619	1.633751	0.99410703
TMX1	Wald ratio	1	-0.066005	0.178392	0.711382	0.936126	0.659909	1.327959	0.99410703
PTGER2	Wald ratio	1	0.0155126	0.078756	0.843851	1.015634	0.870359	1.185156	0.99410703
GPX2	Wald ratio	1	-0.007772	0.078366	0.921	0.992258	0.850977	1.156995	0.99410703
SLC10A1	Wald ratio	1	0.0445396	0.135963	0.743225	1.045546	0.800957	1.364826	0.99410703
GALC	Wald ratio	1	-0.02095	0.066261	0.751869	0.979268	0.860001	1.115075	0.99410703
RPS6KA5	Wald ratio	1	0.0544639	0.167581	0.745181	1.055974	0.760335	1.466566	0.99410703
FBLN5	Wald ratio	1	0.0418357	0.15069	0.781298	1.042723	0.776067	1.401002	0.99410703
LGMN	Wald ratio	1	-0.014751	0.083591	0.859924	0.985357	0.836449	1.160774	0.99410703
SERPINA1	Wald ratio	1	-0.029957	0.164764	0.855725	0.970487	0.702652	1.340416	0.99410703
SERPINA6	Wald ratio	1	-0.027078	0.093926	0.773126	0.973286	0.809634	1.170016	0.99410703
SERPINA9	Wald ratio	1	0.0335251	0.2682	0.900524	1.034093	0.611312	1.749269	0.99410703
BDKRB2	Wald ratio	1	-0.018404	0.051787	0.722311	0.981765	0.887004	1.086649	0.99410703
DUOX1	Wald ratio	1	-0.016384	0.08028	0.83829	0.98375	0.840521	1.151385	0.99410703
DUT	Wald ratio	1	0.06201	0.219693	0.777746	1.063973	0.691711	1.636578	0.99410703
FGF7	Wald ratio	1	-0.032919	0.085763	0.701098	0.967617	0.8179	1.144739	0.99410703
ALDH1A2	Wald ratio	1	-0.018635	0.073873	0.800846	0.981538	0.84923	1.13446	0.99410703
CCNB2	Wald ratio	1	0.0151445	0.141103	0.914527	1.01526	0.76996	1.338709	0.99410703
PPIB	Wald ratio	1	0.0298736	0.414285	0.942515	1.030324	0.457433	2.320709	0.99410703
SMAD3	Wald ratio	1	0.0436547	0.210232	0.835502	1.044622	0.69184	1.577292	0.99410703
LCTL	Wald ratio	1	-0.017464	0.063764	0.784173	0.982687	0.867238	1.113506	0.99410703
SENP8	Wald ratio	1	0.0233889	0.207274	0.910157	1.023665	0.681903	1.536714	0.99410703
CD276	Wald ratio	1	-0.048879	0.134746	0.716795	0.952297	0.731263	1.24014	0.99410703
ISLR2	Wald ratio	1	-0.076739	0.219426	0.726544	0.926131	0.602412	1.423809	0.99410703
CYP11A1	Wald ratio	1	-0.040514	0.136734	0.767004	0.960296	0.734539	1.255438	0.99410703
LINGO1	Wald ratio	1	-0.084614	0.315115	0.7883	0.918867	0.495474	1.704056	0.99410703
ADAMTS7	Wald ratio	1	-0.025243	0.151459	0.867632	0.975073	0.724623	1.312084	0.99410703
RHCG	Wald ratio	1	-0.024791	0.073702	0.736597	0.975514	0.8443	1.12712	0.99410703
METRN	Wald ratio	1	0.0896496	0.300131	0.765168	1.093791	0.607376	1.96975	0.99410703
TNFRSF17	Wald ratio	1	0.0061125	0.022267	0.783693	1.006131	0.963165	1.051014	0.99410703
WFIKKN1	Wald ratio	1	-0.009573	0.02921	0.743115	0.990472	0.935358	1.048834	0.99410703
ABCC1	Wald ratio	1	-0.030365	0.156163	0.845828	0.970091	0.714306	1.317471	0.99410703
SMG1	Wald ratio	1	-0.107424	0.45381	0.812878	0.898145	0.369025	2.185936	0.99410703
VWA3A	Wald ratio	1	0.0443241	0.146212	0.761776	1.045321	0.784859	1.39222	0.99410703
ERN2	Wald ratio	1	0.0275973	0.117942	0.814993	1.027982	0.815813	1.295329	0.99410703

SCNN1G	Wald ratio	1	-0.029135	0.099979	0.770737	0.971285	0.798442	1.181545	0.99410703
SLC5A11	Wald ratio	1	-0.070898	0.210609	0.736393	0.931557	0.616503	1.407613	0.99410703
PLK1	Wald ratio	1	-0.016818	0.134017	0.900135	0.983323	0.756169	1.278714	0.99410703
IL4R	Wald ratio	1	-0.042695	0.116343	0.71364	0.958204	0.762825	1.203624	0.99410703
SBK1	Wald ratio	1	-0.146759	0.376623	0.69678	0.863502	0.412739	1.806556	0.99410703
CD19	Wald ratio	1	-0.029731	0.103641	0.774217	0.970707	0.792259	1.189348	0.99410703
SPN	Wald ratio	1	0.0213136	0.119593	0.858552	1.021542	0.808084	1.291386	0.99410703
HSD3B7	Wald ratio	1	-0.013837	0.07107	0.845635	0.986259	0.858015	1.133671	0.99410703
PRSS53	Wald ratio	1	-0.030221	0.128598	0.814209	0.970232	0.754068	1.248361	0.99410703
PYDC1	Wald ratio	1	0.0295788	0.086201	0.731495	1.030021	0.869901	1.219612	0.99410703
TRAP1	Wald ratio	1	0.0370079	0.103622	0.720985	1.037701	0.846969	1.271386	0.99410703
MYLK3	Wald ratio	1	0.007032	0.074339	0.924637	1.007057	0.870514	1.165017	0.99410703
NETO2	Wald ratio	1	-0.041546	0.190965	0.827774	0.959305	0.659788	1.394792	0.99410703
ITFG1	Wald ratio	1	-0.055515	0.362237	0.878196	0.945998	0.465102	1.924121	0.99410703
ABCC12	Wald ratio	1	-0.039892	0.173954	0.818618	0.960893	0.683286	1.351288	0.99410703
ADCY7	Wald ratio	1	-0.03838	0.10645	0.71844	0.962347	0.781124	1.185616	0.99410703
SLC6A2	Wald ratio	1	0.057471	0.145627	0.693105	1.059155	0.796158	1.409028	0.99410703
CCL17	Wald ratio	1	-0.036149	0.107384	0.736393	0.964496	0.781436	1.190441	0.99410703
CCL22	Wald ratio	1	-0.021477	0.090462	0.812339	0.978752	0.819728	1.168627	0.99410703
SLC9A5	Wald ratio	1	-0.040284	0.130007	0.756668	0.960517	0.744459	1.239279	0.99410703
CES4A	Wald ratio	1	0.0391331	0.320891	0.902938	1.039909	0.554431	1.950488	0.99410703
AGRP	Wald ratio	1	0.0324566	0.085412	0.703945	1.032989	0.873759	1.221237	0.99410703
GFOD2	Wald ratio	1	-0.028123	0.233259	0.904034	0.972268	0.615506	1.535819	0.99410703
HSD11B2	Wald ratio	1	0.0395053	0.190689	0.835876	1.040296	0.715878	1.511731	0.99410703
ST3GAL2	Wald ratio	1	-0.019766	0.16117	0.902391	0.980428	0.714867	1.344641	0.99410703
HP	Wald ratio	1	-0.062832	0.208525	0.763172	0.939101	0.62404	1.413228	0.99410703
ADAMTS18	Wald ratio	1	-0.018119	0.081537	0.824141	0.982044	0.836999	1.152223	0.99410703
WWOX	Wald ratio	1	-0.012558	0.194074	0.948408	0.987521	0.675067	1.444593	0.99410703
MBTPS1	Wald ratio	1	-0.023196	0.078867	0.768668	0.977071	0.837131	1.140404	0.99410703
CRISPLD2	Wald ratio	1	0.0478171	0.150669	0.750966	1.048979	0.780755	1.40935	0.99410703
KLHL36	Wald ratio	1	-0.014561	0.132266	0.912337	0.985544	0.760483	1.277211	0.99410703
CA5A	Wald ratio	1	-0.017946	0.051703	0.728511	0.982214	0.887556	1.086967	0.99410703
SLC7A5	Wald ratio	1	-0.019167	0.268902	0.943176	0.981015	0.579138	1.661766	0.99410703
MAP2K4	Wald ratio	1	-0.070707	0.292928	0.809261	0.931735	0.524744	1.654389	0.99410703
PMP22	Wald ratio	1	-0.040693	0.181555	0.82265	0.960124	0.672643	1.370471	0.99410703
ULK2	Wald ratio	1	0.0337891	0.117333	0.773366	1.034366	0.821861	1.301819	0.99410703
DHRS7B	Wald ratio	1	0.0352645	0.2385	0.882453	1.035894	0.649083	1.653218	0.99410703
SLC43A2	Wald ratio	1	0.0583476	0.191767	0.760927	1.060083	0.727956	1.543743	0.99410703
OR1E2	Wald ratio	1	-0.019708	0.092999	0.832169	0.980485	0.817106	1.176531	0.99410703
OR1D2	Wald ratio	1	-0.030339	0.081265	0.7089	0.970117	0.827274	1.137623	0.99410703
HASPIN	Wald ratio	1	-0.032951	0.090616	0.71613	0.967586	0.810132	1.155642	0.99410703
OR3A3	Wald ratio	1	-0.015289	0.098684	0.876877	0.984827	0.811631	1.194982	0.99410703
TRPV1	Wald ratio	1	0.0148108	0.037184	0.690405	1.014921	0.943583	1.091652	0.99410703
ALOX15	Wald ratio	1	-0.011404	0.094193	0.903631	0.98866	0.821994	1.189119	0.99410703
ACLY	Wald ratio	1	-0.032699	0.128617	0.799312	0.96783	0.752173	1.245317	0.99410703
STAT5B	Wald ratio	1	0.0127588	0.236746	0.957021	1.01284	0.636823	1.61088	0.99410703
TUBG2	Wald ratio	1	0.0185914	0.123942	0.880765	1.018765	0.799047	1.298901	0.99410703
PPY	Wald ratio	1	-0.090252	0.255394	0.723801	0.913701	0.55387	1.507301	0.99410703
SOST	Wald ratio	1	-0.012588	0.03147	0.689157	0.987491	0.928422	1.050318	0.99410703
PYY	Wald ratio	1	0.0294888	0.108882	0.786519	1.029928	0.832003	1.274938	0.99410703
GRN	Wald ratio	1	0.0119541	0.114107	0.916565	1.012026	0.809211	1.265672	0.99410703
ITGB3	Wald ratio	1	0.019206	0.056338	0.733172	1.019392	0.912821	1.138404	0.99410703
PDK2	Wald ratio	1	0.0403025	0.141059	0.775097	1.041126	0.789644	1.372698	0.99410703
TLK2	Wald ratio	1	-0.06531	0.171438	0.703239	0.936777	0.66943	1.310893	0.99410703
CSH2	Wald ratio	1	-0.009023	0.076698	0.906347	0.991017	0.852697	1.151776	0.99410703
ICAM2	Wald ratio	1	-0.057488	0.159198	0.718016	0.944133	0.691069	1.289868	0.99410703
CSH1	Wald ratio	1	0.024155	0.127855	0.850152	1.024449	0.797367	1.316202	0.99410703
CACNG4	Wald ratio	1	0.0920184	0.266369	0.729753	1.096385	0.650467	1.847996	0.99410703
KCNJ16	Wald ratio	1	0.0319361	0.087824	0.71613	1.032452	0.869185	1.226386	0.99410703
GPRC5C	Wald ratio	1	-0.063194	0.149445	0.672399	0.938761	0.700398	1.258245	0.99410703
TMEM94	Wald ratio	1	-0.017467	0.254525	0.945286	0.982684	0.596702	1.618342	0.99410703
ZACN	Wald ratio	1	-0.038057	0.259264	0.883299	0.962658	0.579138	1.600155	0.99410703
SPHK1	Wald ratio	1	0.0289287	0.073286	0.693037	1.029351	0.891623	1.188354	0.99410703
KCNAB3	Wald ratio	1	-0.018571	0.142855	0.896566	0.9816	0.74188	1.29878	0.99410703
ALOX15B	Wald ratio	1	0.0071793	0.075382	0.924126	1.007205	0.868863	1.167575	0.99410703

CSNK1D	Wald ratio	1	-0.034767	0.31473	0.912041	0.965831	0.521192	1.789801	0.99410703
SLC25A35	Wald ratio	1	-0.010635	0.184885	0.954129	0.989421	0.688659	1.421538	0.99410703
GRP	Wald ratio	1	0.0085301	0.134045	0.94926	1.008567	0.775538	1.311614	0.99410703
LMAN1	Wald ratio	1	0.017663	0.240658	0.941492	1.01782	0.635065	1.631261	0.99410703
TNFRSF11A	Wald ratio	1	0.0175358	0.052146	0.736657	1.01769	0.918815	1.127206	0.99410703
BCL2	Wald ratio	1	-0.031308	0.10895	0.77384	0.969177	0.782822	1.199896	0.99410703
SERPINB5	Wald ratio	1	-0.01643	0.104057	0.87454	0.983704	0.802212	1.206257	0.99410703
SERPINB10	Wald ratio	1	-0.010751	0.081185	0.894652	0.989307	0.843771	1.159945	0.99410703
KCNG2	Wald ratio	1	-0.010681	0.159401	0.946573	0.989375	0.723897	1.352214	0.99410703
NFATC1	Wald ratio	1	0.0330126	0.117142	0.778083	1.033564	0.821531	1.30032	0.99410703
ICAM3	Wald ratio	1	0.0063129	0.039207	0.87208	1.006333	0.931898	1.086714	0.99410703
ABCA7	Wald ratio	1	0.0525548	0.241027	0.827394	1.05396	0.65714	1.690405	0.99410703
GPX4	Wald ratio	1	-0.007565	0.085742	0.92969	0.992463	0.838937	1.174084	0.99410703
MAN2B1	Wald ratio	1	0.0536684	0.134795	0.690521	1.055135	0.810155	1.374193	0.99410703
OR10H1	Wald ratio	1	-0.024879	0.132285	0.850819	0.975428	0.752648	1.264149	0.99410703
BST2	Wald ratio	1	-0.045656	0.128912	0.723214	0.95537	0.742061	1.229997	0.99410703
JAK3	Wald ratio	1	0.0317974	0.153899	0.836313	1.032308	0.763498	1.395761	0.99410703
CRLF1	Wald ratio	1	-0.015258	0.060446	0.800711	0.984858	0.874823	1.108731	0.99410703
IFI30	Wald ratio	1	0.0309601	0.117848	0.792773	1.031444	0.818713	1.299451	0.99410703
CHST8	Wald ratio	1	0.009118	0.105272	0.930978	1.00916	0.821014	1.240421	0.99410703
HPN	Wald ratio	1	0.0294965	0.247161	0.905005	1.029936	0.634487	1.671851	0.99410703
SBSN	Wald ratio	1	0.0286335	0.111084	0.796589	1.029047	0.827711	1.279358	0.99410703
DMKN	Wald ratio	1	0.039533	0.129451	0.76007	1.040325	0.807194	1.340788	0.99410703
GAPDHS	Wald ratio	1	0.0181943	0.081175	0.82265	1.018361	0.868569	1.193985	0.99410703
CD22	Wald ratio	1	-0.060489	0.203598	0.766389	0.941304	0.631573	1.402931	0.99410703
MATK	Wald ratio	1	-0.090295	0.357706	0.800711	0.913662	0.453211	1.841921	0.99410703
TBXA2R	Wald ratio	1	0.0076258	0.13004	0.953237	1.007655	0.780944	1.300181	0.99410703
LGALS7B	Wald ratio	1	0.0216165	0.085701	0.800862	1.021852	0.863849	1.208754	0.99410703
LGALS7	Wald ratio	1	0.0089595	0.029708	0.762967	1.009	0.951926	1.069495	0.99410703
MIA	Wald ratio	1	-0.014158	0.080498	0.860389	0.985942	0.842035	1.154443	0.99410703
CYP2A6	Wald ratio	1	-0.006662	0.091602	0.942023	0.99336	0.830106	1.188721	0.99410703
LIPE	Wald ratio	1	-0.112967	0.310262	0.715781	0.89318	0.486227	1.640737	0.99410703
NECTIN2	Wald ratio	1	-0.00811	0.05587	0.884584	0.991923	0.889037	1.106715	0.99410703
CALM3	Wald ratio	1	0.1142582	0.273067	0.675636	1.121041	0.656421	1.914523	0.99410703
HSD11B1L	Wald ratio	1	-0.15086	0.426717	0.723687	0.859968	0.372609	1.984777	0.99410703
TULP2	Wald ratio	1	-0.01457	0.050265	0.771927	0.985536	0.893071	1.087574	0.99410703
FLT3LG	Wald ratio	1	-0.035389	0.110537	0.748854	0.96523	0.777212	1.198732	0.99410703
SIGLEC11	Wald ratio	1	0.0038179	0.03207	0.905238	1.003825	0.94267	1.068948	0.99410703
NAPSA	Wald ratio	1	-0.042122	0.100698	0.675727	0.958753	0.78703	1.167945	0.99410703
KLK15	Wald ratio	1	-0.019969	0.105406	0.849741	0.980229	0.797267	1.205178	0.99410703
SIGLEC10	Wald ratio	1	0.0082932	0.113618	0.941812	1.008328	0.807028	1.259839	0.99410703
FPR3	Wald ratio	1	-0.025355	0.080123	0.751657	0.974964	0.833272	1.140749	0.99410703
PTPRS	Wald ratio	1	0.0804799	0.340492	0.813151	1.083807	0.556057	2.112441	0.99410703
CACNG7	Wald ratio	1	-0.060026	0.25761	0.815754	0.94174	0.568394	1.560318	0.99410703
LILRA6	Wald ratio	1	0.0218662	0.113134	0.846742	1.022107	0.818832	1.275845	0.99410703
LILRB4	Wald ratio	1	-0.053865	0.164357	0.743115	0.94756	0.686599	1.307707	0.99410703
INSR	Wald ratio	1	0.0769668	0.224265	0.731452	1.080006	0.69587	1.676195	0.99410703
RETN	Wald ratio	1	-0.010048	0.105502	0.924126	0.990003	0.805065	1.217423	0.99410703
CD320	Wald ratio	1	-0.019533	0.176431	0.911843	0.980656	0.693961	1.385792	0.99410703
LYG2	Wald ratio	1	0.023986	0.071958	0.738883	1.024276	0.889539	1.179421	0.99410703
SLC9A2	Wald ratio	1	-0.027007	0.072992	0.711382	0.973354	0.843604	1.12306	0.99410703
MFSD9	Wald ratio	1	0.0362567	0.103734	0.726703	1.036922	0.846147	1.27071	0.99410703
ADAM17	Wald ratio	1	0.1099733	0.259792	0.672067	1.116248	0.670844	1.857378	0.99410703
SLC5A7	Wald ratio	1	0.0052066	0.019564	0.79014	1.00522	0.967404	1.044514	0.99410703
MARCO	Wald ratio	1	-0.021802	0.094883	0.818268	0.978434	0.812391	1.178414	0.99410703
PXDN	Wald ratio	1	-0.009637	0.116713	0.934194	0.990409	0.787892	1.244981	0.99410703
MAP3K2	Wald ratio	1	0.036024	0.184819	0.845459	1.036681	0.721646	1.489244	0.99410703
LY75	Wald ratio	1	0.0483828	0.127005	0.703239	1.049572	0.818283	1.346236	0.99410703
PSMD14	Wald ratio	1	0.050064	0.329454	0.879218	1.051338	0.551196	2.005299	0.99410703
FAP	Wald ratio	1	0.0543103	0.210658	0.796552	1.055812	0.698668	1.59552	0.99410703
TLK1	Wald ratio	1	0.0337028	0.176539	0.848597	1.034277	0.731753	1.461873	0.99410703
METAP1D	Wald ratio	1	0.0063573	0.085823	0.940951	1.006378	0.850563	1.190735	0.99410703
ITGA6	Wald ratio	1	0.0463878	0.168639	0.783261	1.047481	0.752658	1.457788	0.99410703
TTN	Wald ratio	1	-0.006488	0.06206	0.916736	0.993533	0.879743	1.122041	0.99410703
COL5A2	Wald ratio	1	-0.002655	0.043625	0.951463	0.997348	0.915614	1.086379	0.99410703

HSPD1	Wald ratio	1	-0.047549	0.147176	0.746637	0.953564	0.714614	1.272413	0.99410703
ACP1	Wald ratio	1	-0.024928	0.186963	0.89393	0.97538	0.676125	1.407085	0.99410703
MATN3	Wald ratio	1	0.0490869	0.217865	0.821739	1.050312	0.68528	1.609787	0.99410703
CASP8	Wald ratio	1	-0.017217	0.078192	0.825728	0.982931	0.843265	1.145728	0.99410703
SDC1	Wald ratio	1	-0.033657	0.152131	0.824906	0.966903	0.717606	1.302805	0.99410703
GDF7	Wald ratio	1	-0.02085	0.052357	0.690461	0.979366	0.883848	1.085206	0.99410703
GPBAR1	Wald ratio	1	0.0258788	0.094047	0.783187	1.026217	0.853462	1.233939	0.99410703
WNT6	Wald ratio	1	0.0468848	0.154862	0.762079	1.048001	0.773643	1.419655	0.99410703
PTPRN	Wald ratio	1	-0.026325	0.293626	0.928561	0.974018	0.547807	1.731836	0.99410703
SPEG	Wald ratio	1	0.0829239	0.308003	0.787752	1.086459	0.594068	1.986968	0.99410703
INHA	Wald ratio	1	-0.040161	0.11704	0.731495	0.960635	0.763717	1.208327	0.99410703
HTR2B	Wald ratio	1	0.0422506	0.108267	0.696356	1.043156	0.843704	1.289758	0.99410703
GPC1	Wald ratio	1	0.0495493	0.297296	0.867632	1.050797	0.586753	1.881839	0.99410703
CAPN10	Wald ratio	1	0.0511901	0.158332	0.746462	1.052523	0.771714	1.435511	0.99410703
DNMT3A	Wald ratio	1	0.0938396	0.302968	0.756763	1.098384	0.606544	1.989048	0.99410703
AGBL5	Wald ratio	1	-0.086856	0.417457	0.835181	0.916809	0.404513	2.077902	0.99410703
KCNG3	Wald ratio	1	0.0267652	0.266244	0.919924	1.027127	0.609527	1.730833	0.99410703
PREPL	Wald ratio	1	-0.062132	0.434924	0.886403	0.939759	0.400683	2.204101	0.99410703
TGFA	Wald ratio	1	0.0457662	0.114974	0.690587	1.04683	0.835619	1.311425	0.99410703
PLGLB2	Wald ratio	1	-0.002553	0.02371	0.91424	0.99745	0.952158	1.044896	0.99410703
ZAP70	Wald ratio	1	0.0178266	0.059962	0.76624	1.017986	0.90511	1.14494	0.99410703
ODC1	Wald ratio	1	-0.03628	0.21466	0.865786	0.96437	0.633172	1.468809	0.99410703
TXNDC9	Wald ratio	1	-0.055886	0.308408	0.856204	0.945647	0.516662	1.730819	0.99410703
STK35	Wald ratio	1	0.0290742	0.1606	0.856341	1.029501	0.751486	1.410368	0.99410703
CPXM1	Wald ratio	1	-0.034515	0.120804	0.775097	0.966073	0.762394	1.224167	0.99410703
MYLK2	Wald ratio	1	-0.017337	0.105757	0.869783	0.982812	0.798819	1.209185	0.99410703
DNMT3B	Wald ratio	1	-0.036142	0.093969	0.700522	0.964503	0.802261	1.159556	0.99410703
BAK1P1	Wald ratio	1	0.0189958	0.098978	0.847806	1.019177	0.839456	1.237376	0.99410703
SLC4A11	Wald ratio	1	-0.00474	0.082471	0.95417	0.995271	0.846722	1.169883	0.99410703
GFRA4	Wald ratio	1	0.0173396	0.048892	0.722851	1.017491	0.924513	1.11982	0.99410703
ADRA1D	Wald ratio	1	0.040969	0.197163	0.835391	1.04182	0.707887	1.53328	0.99410703
SRC	Wald ratio	1	-0.073612	0.240913	0.759943	0.929032	0.579376	1.489705	0.99410703
SGK2	Wald ratio	1	-0.006687	0.079255	0.932758	0.993335	0.850418	1.16027	0.99410703
STK4	Wald ratio	1	0.0103419	0.102479	0.919616	1.010396	0.826531	1.235161	0.99410703
MATN4	Wald ratio	1	0.0258525	0.10341	0.802587	1.02619	0.837921	1.256759	0.99410703
PTGIS	Wald ratio	1	-0.006759	0.078856	0.931694	0.993264	0.851023	1.159279	0.99410703
PCK1	Wald ratio	1	-0.015734	0.074925	0.833668	0.984389	0.849941	1.140104	0.99410703
TUBB1	Wald ratio	1	0.0046737	0.045179	0.917607	1.004685	0.919544	1.097709	0.99410703
GNAS	Wald ratio	1	-0.06317	0.351948	0.857555	0.938784	0.470957	1.871327	0.99410703
KCNQ2	Wald ratio	1	0.0306676	0.209562	0.883652	1.031143	0.683811	1.554897	0.99410703
PTK6	Wald ratio	1	-0.022531	0.14609	0.877429	0.977721	0.734277	1.301875	0.99410703
BMP2	Wald ratio	1	-0.040049	0.175424	0.819416	0.960743	0.681214	1.354974	0.99410703
HUNK	Wald ratio	1	0.0585417	0.173999	0.736533	1.060289	0.753899	1.491198	0.99410703
IFNAR2	Wald ratio	1	0.0202049	0.054062	0.7086	1.02041	0.917818	1.13447	0.99410703
GART	Wald ratio	1	0.0293083	0.182858	0.872662	1.029742	0.719575	1.473604	0.99410703
HLCS	Wald ratio	1	0.0116216	0.048208	0.8095	1.011689	0.920474	1.111944	0.99410703
COL18A1	Wald ratio	1	-0.037917	0.2002	0.849784	0.962793	0.650308	1.425433	0.99410703
DGCR6	Wald ratio	1	-0.008078	0.028904	0.77988	0.991954	0.93732	1.049774	0.99410703
PPIL2	Wald ratio	1	-0.078332	0.21232	0.712178	0.924658	0.609889	1.401882	0.99410703
IGLV1-36	Wald ratio	1	0.0345663	0.134689	0.797459	1.035171	0.794991	1.347913	0.99410703
MMP11	Wald ratio	1	0.0335647	0.096885	0.729014	1.034134	0.855276	1.250396	0.99410703
LIF	Wald ratio	1	0.0145048	0.105563	0.890711	1.01461	0.824979	1.247832	0.99410703
PLA2G3	Wald ratio	1	0.026712	0.100514	0.790429	1.027072	0.843415	1.250721	0.99410703
TCN2	Wald ratio	1	-0.026568	0.06642	0.689157	0.973782	0.854917	1.109173	0.99410703
OSM	Wald ratio	1	0.0161864	0.102784	0.874866	1.016318	0.83088	1.243142	0.99410703
HMOX1	Wald ratio	1	0.0069957	0.096773	0.942372	1.00702	0.833034	1.217344	0.99410703
CSF2RB	Wald ratio	1	-0.023503	0.126611	0.852736	0.976771	0.762113	1.251891	0.99410703
SLC16A8	Wald ratio	1	0.0106271	0.133193	0.936406	1.010684	0.778465	1.312174	0.99410703
TMPRSS6	Wald ratio	1	-0.007236	0.105436	0.945286	0.99279	0.807436	1.220694	0.99410703
GALR3	Wald ratio	1	-0.033368	0.083863	0.690713	0.967182	0.820583	1.139972	0.99410703
APOBEC3A	Wald ratio	1	-0.01794	0.086438	0.835583	0.98222	0.829146	1.163554	0.99410703
XPNPEP3	Wald ratio	1	0.0053006	0.023968	0.824973	1.005315	0.95918	1.053668	0.99410703
NAGA	Wald ratio	1	0.0267794	0.127433	0.833555	1.027141	0.800124	1.31857	0.99410703
CPT1B	Wald ratio	1	0.0162822	0.073896	0.825607	1.016415	0.879366	1.174824	0.99410703
TYMP	Wald ratio	1	0.0162674	0.132308	0.902146	1.0164	0.784227	1.31731	0.99410703

ADGRG7	Wald ratio	1	0.0103828	0.041656	0.803168	1.010437	0.931217	1.096396	0.99410703
GHRL	Wald ratio	1	0.0195927	0.085323	0.81838	1.019786	0.862741	1.205418	0.99410703
HRH1	Wald ratio	1	0.0394891	0.132042	0.76489	1.040279	0.803071	1.347554	0.99410703
NECTIN3	Wald ratio	1	0.0144378	0.23293	0.950576	1.014543	0.642683	1.601563	0.99410703
CD200	Wald ratio	1	0.0171253	0.195989	0.930371	1.017273	0.6928	1.493712	0.99410703
CD200R1	Wald ratio	1	-0.023826	0.080413	0.767004	0.976455	0.834072	1.143146	0.99410703
NR1I2	Wald ratio	1	0.0065418	0.083408	0.937485	1.006563	0.854758	1.185329	0.99410703
CASR	Wald ratio	1	0.040509	0.109613	0.711706	1.041341	0.840018	1.290913	0.99410703
UMPS	Wald ratio	1	-0.012144	0.171754	0.943631	0.987929	0.705547	1.38333	0.99410703
HDAC11	Wald ratio	1	0.0359997	0.261598	0.890545	1.036656	0.620809	1.731056	0.99410703
RPN1	Wald ratio	1	0.0273644	0.091803	0.765644	1.027742	0.858499	1.23035	0.99410703
RYK	Wald ratio	1	-0.098723	0.473488	0.834837	0.905993	0.358165	2.291746	0.99410703
WNT7A	Wald ratio	1	-0.02315	0.075423	0.758896	0.977116	0.84284	1.132785	0.99410703
CLDN18	Wald ratio	1	-0.049361	0.152195	0.745693	0.951838	0.706337	1.282668	0.99410703
SLC9A9	Wald ratio	1	0.0836316	0.39725	0.833257	1.087228	0.499085	2.368465	0.99410703
PLSCR2	Wald ratio	1	-0.00623	0.069483	0.928561	0.99379	0.86726	1.13878	0.99410703
NR2C2	Wald ratio	1	-0.065911	0.252657	0.794193	0.936215	0.57057	1.536178	0.99410703
AADACL2	Wald ratio	1	0.0312136	0.075529	0.679412	1.031706	0.889742	1.196321	0.99410703
P2RY1	Wald ratio	1	0.0410103	0.174739	0.814446	1.041863	0.739723	1.467411	0.99410703
GPR149	Wald ratio	1	-0.055815	0.159552	0.726471	0.945714	0.691746	1.292924	0.99410703
PTX3	Wald ratio	1	-0.03008	0.072633	0.678771	0.970367	0.841607	1.118827	0.99410703
MFSD1	Wald ratio	1	0.0074773	0.089728	0.933586	1.007505	0.845025	1.201228	0.99410703
GFM1	Wald ratio	1	-0.031524	0.108234	0.770851	0.968967	0.783752	1.197952	0.99410703
COLQ	Wald ratio	1	0.0355321	0.156933	0.820879	1.036171	0.761811	1.40934	0.99410703
TNFSF10	Wald ratio	1	0.0096199	0.141893	0.945948	1.009666	0.764533	1.333398	0.99410703
HTR3E	Wald ratio	1	0.0256016	0.074529	0.731214	1.025932	0.886499	1.187297	0.99410703
ABCF3	Wald ratio	1	-0.053091	0.132913	0.689568	0.948294	0.730811	1.230498	0.99410703
CHRD	Wald ratio	1	-0.016659	0.078629	0.832213	0.983479	0.843013	1.14735	0.99410703
KNG1	Wald ratio	1	-0.011629	0.106845	0.913327	0.988438	0.801681	1.218702	0.99410703
ATP13A3	Wald ratio	1	-0.075062	0.193215	0.697654	0.927686	0.635232	1.354782	0.99410703
RARB	Wald ratio	1	0.0987387	0.252099	0.695305	1.103778	0.673428	1.809141	0.99410703
CCR4	Wald ratio	1	0.0296807	0.077459	0.701588	1.030126	0.885025	1.199016	0.99410703
LRRN1	Wald ratio	1	-0.073852	0.182273	0.685351	0.928809	0.649789	1.327641	0.99410703
PLCD1	Wald ratio	1	-0.043748	0.170689	0.797717	0.957195	0.685026	1.3375	0.99410703
EXOG	Wald ratio	1	-0.025966	0.186955	0.889538	0.974368	0.675435	1.405602	0.99410703
CX3CR1	Wald ratio	1	0.0914997	0.236374	0.698685	1.095816	0.689496	1.741581	0.99410703
SCN11A	Wald ratio	1	0.0133647	0.118055	0.909866	1.013454	0.804107	1.277305	0.99410703
SLC25A38	Wald ratio	1	-0.01598	0.119316	0.893459	0.984147	0.778927	1.243436	0.99410703
CYP8B1	Wald ratio	1	-0.026749	0.079971	0.73802	0.973606	0.832358	1.138823	0.99410703
CLEC3B	Wald ratio	1	-0.020672	0.229146	0.928117	0.97954	0.625129	1.534882	0.99410703
CXCR6	Wald ratio	1	0.0249892	0.141605	0.859924	1.025304	0.776812	1.353286	0.99410703
CCRL2	Wald ratio	1	-0.014627	0.10755	0.891821	0.98548	0.798177	1.216735	0.99410703
P4HTM	Wald ratio	1	-0.006104	0.095053	0.948795	0.993914	0.82497	1.197456	0.99410703
PLXNB1	Wald ratio	1	0.0439967	0.256647	0.863887	1.044979	0.631895	1.728104	0.99410703
COL7A1	Wald ratio	1	0.0233715	0.166405	0.888305	1.023647	0.73876	1.418394	0.99410703
HYAL1	Wald ratio	1	0.0284028	0.09941	0.775097	1.02881	0.846673	1.250128	0.99410703
CACNA2D2	Wald ratio	1	-0.08651	0.222455	0.697358	0.917126	0.593023	1.41836	0.99410703
NEK4	Wald ratio	1	-0.008579	0.062693	0.891156	0.991458	0.876817	1.121087	0.99410703
NISCH	Wald ratio	1	0.0721145	0.197027	0.714355	1.074778	0.730476	1.581364	0.99410703
TNNC1	Wald ratio	1	-0.026673	0.093934	0.776448	0.97368	0.80995	1.170508	0.99410703
GRM7	Wald ratio	1	-0.04638	0.260298	0.85858	0.954679	0.573174	1.590112	0.99410703
GBE1	Wald ratio	1	-0.014697	0.169828	0.931038	0.985411	0.70641	1.374605	0.99410703
EPHA6	Wald ratio	1	0.0144642	0.235947	0.951118	1.014569	0.63891	1.611105	0.99410703
ADH6	Wald ratio	1	0.0052314	0.060048	0.930575	1.005245	0.893631	1.130799	0.99410703
NPNT	Wald ratio	1	-0.019427	0.100695	0.847017	0.980761	0.805101	1.194747	0.99410703
CASP6	Wald ratio	1	-0.01305	0.058439	0.823301	0.987035	0.880214	1.106821	0.99410703
ENPEP	Wald ratio	1	0.0321466	0.083489	0.700207	1.032669	0.876787	1.216265	0.99410703
RRH	Wald ratio	1	0.0164244	0.044102	0.709585	1.01656	0.932379	1.108342	0.99410703
PDE5A	Wald ratio	1	0.0333299	0.184108	0.856341	1.033892	0.720707	1.483171	0.99410703
TRPC3	Wald ratio	1	-0.044704	0.171938	0.794864	0.956281	0.682698	1.339498	0.99410703
NOCT	Wald ratio	1	-0.095221	0.35077	0.786036	0.909172	0.457156	1.808122	0.99410703
IL15	Wald ratio	1	0.011838	0.086196	0.890763	1.011908	0.854614	1.198154	0.99410703
NR3C2	Wald ratio	1	0.0426178	0.225874	0.850344	1.043539	0.670256	1.624713	0.99410703
DCLK2	Wald ratio	1	-0.016909	0.070141	0.8095	0.983233	0.856942	1.128136	0.99410703
CD38	Wald ratio	1	-0.00892	0.055192	0.871608	0.99112	0.8895	1.104349	0.99410703

BST1	Wald ratio	1	-0.010253	0.114647	0.928739	0.989799	0.790602	1.239186	0.99410703
RXFP1	Wald ratio	1	-0.03432	0.125842	0.785063	0.966262	0.755051	1.236555	0.99410703
FSTL5	Wald ratio	1	0.0506727	0.177354	0.775097	1.051979	0.743087	1.489272	0.99410703
SCRG1	Wald ratio	1	-0.028857	0.190145	0.879375	0.971556	0.669287	1.410336	0.99410703
NEIL3	Wald ratio	1	-0.006333	0.110829	0.954431	0.993687	0.799669	1.234778	0.99410703
TLR3	Wald ratio	1	-0.040589	0.117378	0.729497	0.960224	0.762883	1.208612	0.99410703
TRIML1	Wald ratio	1	0.0320176	0.099102	0.746637	1.032536	0.850252	1.253898	0.99410703
RGS12	Wald ratio	1	0.0182566	0.14214	0.8978	1.018424	0.77079	1.345616	0.99410703
HGFAC	Wald ratio	1	0.0108452	0.068899	0.874924	1.010904	0.883207	1.157065	0.99410703
CCKAR	Wald ratio	1	0.0273887	0.131074	0.834483	1.027767	0.794917	1.328824	0.99410703
GABRA4	Wald ratio	1	-0.068429	0.255468	0.788809	0.93386	0.566008	1.540779	0.99410703
TXK	Wald ratio	1	0.0167966	0.060703	0.782011	1.016938	0.902865	1.145424	0.99410703
SLC10A4	Wald ratio	1	-0.024375	0.099375	0.806237	0.97592	0.803201	1.185779	0.99410703
ADGRL3	Wald ratio	1	-0.062486	0.286396	0.827287	0.939426	0.535892	1.646827	0.99410703
HTN1	Wald ratio	1	0.0700528	0.172178	0.684109	1.072565	0.765355	1.503088	0.99410703
SULT1B1	Wald ratio	1	-0.014851	0.109526	0.892143	0.985259	0.794914	1.221183	0.99410703
JCHAIN	Wald ratio	1	0.0120701	0.07557	0.8731	1.012143	0.872802	1.17373	0.99410703
MUC7	Wald ratio	1	0.0649066	0.202267	0.74829	1.067059	0.71782	1.586215	0.99410703
ENAM	Wald ratio	1	0.0396896	0.098147	0.685927	1.040488	0.858405	1.261193	0.99410703
SLC4A4	Wald ratio	1	-0.033637	0.139932	0.810032	0.966922	0.734986	1.272049	0.99410703
CXCL5	Wald ratio	1	0.0100661	0.105359	0.923885	1.010117	0.821653	1.241809	0.99410703
CXCL11	Wald ratio	1	-0.006215	0.098305	0.949593	0.993805	0.819639	1.204979	0.99410703
FER	Wald ratio	1	0.0349479	0.389722	0.928546	1.035566	0.482436	2.22288	0.99410703
TRIM36	Wald ratio	1	0.0240289	0.140741	0.864435	1.02432	0.777382	1.349698	0.99410703
LVRN	Wald ratio	1	-0.014375	0.097753	0.883086	0.985727	0.813857	1.193893	0.99410703
TERT	Wald ratio	1	0.0169727	0.107494	0.87454	1.017118	0.823893	1.255659	0.99410703
GDF9	Wald ratio	1	-0.010146	0.055801	0.855725	0.989906	0.887351	1.104313	0.99410703
LEAP2	Wald ratio	1	0.0137776	0.110221	0.900524	1.013873	0.816887	1.258361	0.99410703
KLHL3	Wald ratio	1	0.049637	0.188621	0.792429	1.05089	0.726106	1.520947	0.99410703
SLC4A9	Wald ratio	1	0.0113951	0.077486	0.883086	1.01146	0.868943	1.177353	0.99410703
SPINK1	Wald ratio	1	-0.047176	0.142956	0.7414	0.95392	0.720817	1.262405	0.99410703
STK32A	Wald ratio	1	-0.075939	0.194296	0.695912	0.926872	0.633333	1.356462	0.99410703
SPINK6	Wald ratio	1	0.0267611	0.067308	0.690932	1.027122	0.900178	1.171969	0.99410703
HTR4	Wald ratio	1	0.0128008	0.126941	0.919677	1.012883	0.789778	1.299013	0.99410703
ADAM19	Wald ratio	1	-0.024635	0.100509	0.806381	0.975666	0.801209	1.18811	0.99410703
GABRA6	Wald ratio	1	0.0174738	0.046444	0.70674	1.017627	0.929084	1.114609	0.99410703
GABRG2	Wald ratio	1	0.0153736	0.106711	0.885447	1.015492	0.823839	1.251731	0.99410703
NPM1	Wald ratio	1	0.0224712	0.081645	0.78314	1.022726	0.871487	1.20021	0.99410703
CLK4	Wald ratio	1	-0.038088	0.126549	0.763437	0.962629	0.751169	1.233615	0.99410703
TRIM7	Wald ratio	1	-0.006477	0.064766	0.920344	0.993544	0.875099	1.128021	0.99410703
TARS1	Wald ratio	1	0.0624775	0.34683	0.857044	1.06447	0.539394	2.100687	0.99410703
PTGER4	Wald ratio	1	-0.035015	0.107047	0.74359	0.965591	0.78284	1.191004	0.99410703
SELENOP	Wald ratio	1	-0.048643	0.186185	0.793891	0.952521	0.661289	1.372013	0.99410703
FST	Wald ratio	1	-0.017747	0.110917	0.872881	0.98241	0.790457	1.220976	0.99410703
CD180	Wald ratio	1	-0.014449	0.137675	0.916415	0.985655	0.752547	1.29097	0.99410703
CDK7	Wald ratio	1	-0.023191	0.093623	0.804362	0.977076	0.813271	1.173874	0.99410703
SMN1	Wald ratio	1	0.0052899	0.079569	0.946994	1.005304	0.860136	1.174972	0.99410703
F2R	Wald ratio	1	-0.037367	0.10154	0.712873	0.963323	0.789476	1.175451	0.99410703
ARSB	Wald ratio	1	-0.001722	0.029845	0.953994	0.99828	0.941559	1.058418	0.99410703
CMYA5	Wald ratio	1	-0.014695	0.153482	0.923723	0.985412	0.729409	1.331266	0.99410703
HAPLN1	Wald ratio	1	-0.081433	0.240012	0.734395	0.921795	0.575879	1.475492	0.99410703
MAK	Wald ratio	1	-0.040219	0.153881	0.793812	0.960579	0.710472	1.298732	0.99410703
PREP	Wald ratio	1	0.039794	0.238764	0.867632	1.040596	0.651692	1.661585	0.99410703
SMPD2	Wald ratio	1	0.0142937	0.101943	0.888493	1.014396	0.830676	1.23875	0.99410703
SLC16A10	Wald ratio	1	0.0448265	0.237207	0.850111	1.045846	0.656981	1.664879	0.99410703
ENPP1	Wald ratio	1	-0.006013	0.09879	0.951463	0.994005	0.819025	1.206368	0.99410703
TAAR8	Wald ratio	1	0.0906901	0.252891	0.719884	1.09493	0.666992	1.797428	0.99410703
ALDH8A1	Wald ratio	1	0.0170618	0.110902	0.877731	1.017208	0.818481	1.264187	0.99410703
ADGRG6	Wald ratio	1	-0.010838	0.132469	0.934791	0.98922	0.763015	1.282487	0.99410703
PPIL4	Wald ratio	1	-0.115328	0.301026	0.701633	0.891074	0.493941	1.607505	0.99410703
ESR1	Wald ratio	1	0.0085318	0.087878	0.922657	1.008568	0.848989	1.198143	0.99410703
OPRM1	Wald ratio	1	0.0548069	0.344662	0.873656	1.056337	0.537552	2.075795	0.99410703
SYNJ2	Wald ratio	1	0.0169014	0.118309	0.886403	1.017045	0.806553	1.28247	0.99410703
PLG	Wald ratio	1	-0.019322	0.072994	0.791236	0.980864	0.85011	1.131728	0.99410703
SMOC2	Wald ratio	1	-0.024355	0.066181	0.712873	0.97594	0.857213	1.111111	0.99410703

TUBB2A	Wald ratio	1	0.1248733	0.299696	0.676922	1.133005	0.629688	2.038628	0.99410703
HFE	Wald ratio	1	0.0332816	0.083631	0.69066	1.033842	0.877539	1.217985	0.99410703
BTN3A3	Wald ratio	1	0.0233909	0.057117	0.682155	1.023667	0.915249	1.144927	0.99410703
BTN3A1	Wald ratio	1	0.0365876	0.089342	0.682155	1.037265	0.870644	1.235774	0.99410703
H4C11	Wald ratio	1	0.028137	0.13968	0.840355	1.028537	0.782207	1.35244	0.99410703
PGBD1	Wald ratio	1	0.0425211	0.126275	0.736317	1.043438	0.814666	1.336454	0.99410703
HLA-DRB5	Wald ratio	1	-0.014666	0.063657	0.817787	0.985441	0.869852	1.116391	0.99410703
HLA-DRB1	Wald ratio	1	0.0283517	0.072714	0.696604	1.028757	0.892109	1.186337	0.99410703
PSMB8	Wald ratio	1	0.0506859	0.219998	0.817787	1.051992	0.683512	1.61912	0.99410703
SRPK1	Wald ratio	1	0.0235615	0.105045	0.822525	1.023841	0.833329	1.257908	0.99410703
MTCH1	Wald ratio	1	-0.049375	0.173169	0.775549	0.951825	0.67788	1.336476	0.99410703
KCNK16	Wald ratio	1	0.0457494	0.132959	0.730782	1.046812	0.806662	1.358457	0.99410703
PGC	Wald ratio	1	-0.01862	0.109857	0.86541	0.981552	0.791409	1.217379	0.99410703
PTK7	Wald ratio	1	0.0472874	0.133598	0.723375	1.048423	0.806892	1.362253	0.99410703
VEGFA	Wald ratio	1	-0.029743	0.121521	0.806646	0.970695	0.764966	1.231753	0.99410703
CRISP3	Wald ratio	1	0.0068278	0.084034	0.935243	1.006851	0.853953	1.187125	0.99410703
GSTA4	Wald ratio	1	0.0318393	0.251531	0.899271	1.032352	0.630551	1.690187	0.99410703
BMP5	Wald ratio	1	0.019123	0.149609	0.898292	1.019307	0.760247	1.366643	0.99410703
EYS	Wald ratio	1	-0.015445	0.165685	0.925729	0.984673	0.711637	1.362467	0.99410703
CNR1	Wald ratio	1	-0.0484	0.210884	0.818474	0.952753	0.630191	1.440419	0.99410703
COL26A1	Wald ratio	1	0.0768049	0.395682	0.846092	1.079831	0.497215	2.345134	0.99410703
VGF	Wald ratio	1	-0.119773	0.307297	0.696711	0.887122	0.485743	1.620166	0.99410703
SLC12A9	Wald ratio	1	-0.009138	0.103566	0.92969	0.990903	0.808862	1.213915	0.99410703
TRIM4	Wald ratio	1	0.0202547	0.144676	0.88866	1.020461	0.768503	1.355025	0.99410703
RELN	Wald ratio	1	-0.014881	0.157493	0.924721	0.985229	0.723563	1.341522	0.99410703
GPR37	Wald ratio	1	-0.042704	0.177935	0.81033	0.958195	0.676071	1.358048	0.99410703
OPN1SW	Wald ratio	1	-0.013352	0.059845	0.823452	0.986737	0.877527	1.109538	0.99410703
PTN	Wald ratio	1	0.0709002	0.16806	0.673116	1.073474	0.772211	1.492268	0.99410703
CHRM2	Wald ratio	1	0.0648196	0.196423	0.7414	1.066967	0.726026	1.568013	0.99410703
PARP12	Wald ratio	1	-0.040943	0.125104	0.743462	0.959884	0.751151	1.226619	0.99410703
BRAF	Wald ratio	1	0.0277959	0.286066	0.922595	1.028186	0.586904	1.801259	0.99410703
OR2A14	Wald ratio	1	-0.005383	0.071931	0.94035	0.994632	0.863841	1.145226	0.99410703
OR2A5	Wald ratio	1	-0.002739	0.036598	0.94035	0.997265	0.928235	1.071429	0.99410703
OR2F1	Wald ratio	1	0.0251644	0.065646	0.701473	1.025484	0.901674	1.166294	0.99410703
OR6B1	Wald ratio	1	0.0098704	0.05064	0.845459	1.009919	0.914495	1.115301	0.99410703
OR2A25	Wald ratio	1	0.0302882	0.07862	0.700055	1.030752	0.88355	1.202478	0.99410703
OR2A12	Wald ratio	1	0.0309113	0.083804	0.712238	1.031394	0.875164	1.215514	0.99410703
SLC4A2	Wald ratio	1	-0.035892	0.197407	0.855725	0.964744	0.655203	1.420525	0.99410703
HTR5A	Wald ratio	1	-0.055843	0.156073	0.720494	0.945688	0.696459	1.284104	0.99410703
SOSTDC1	Wald ratio	1	0.0539895	0.144458	0.7086	1.055474	0.79521	1.400919	0.99410703
HDAC9	Wald ratio	1	0.0600544	0.219627	0.784517	1.061894	0.690447	1.633172	0.99410703
KLHL7	Wald ratio	1	-0.012259	0.144274	0.932287	0.987816	0.744505	1.310645	0.99410703
NOD1	Wald ratio	1	0.0240449	0.078514	0.759414	1.024336	0.878234	1.194745	0.99410703
GHRHR	Wald ratio	1	0.0115566	0.065651	0.86027	1.011624	0.889478	1.150542	0.99410703
KBTBD2	Wald ratio	1	0.0646008	0.204832	0.75247	1.066733	0.714002	1.59372	0.99410703
SFRP4	Wald ratio	1	0.0486935	0.124168	0.694942	1.049898	0.823101	1.339188	0.99410703
AEBP1	Wald ratio	1	-0.015088	0.111003	0.891883	0.985025	0.792427	1.224434	0.99410703
RAMP3	Wald ratio	1	0.0107141	0.092645	0.907933	1.010772	0.842931	1.212032	0.99410703
DDC	Wald ratio	1	0.0274077	0.078416	0.726703	1.027787	0.881361	1.19854	0.99410703
FKBP9P1	Wald ratio	1	-0.021345	0.116834	0.855039	0.978882	0.778537	1.230782	0.99410703
COL28A1	Wald ratio	1	-0.004968	0.027078	0.854417	0.995044	0.943611	1.04928	0.99410703
EIF4H	Wald ratio	1	0.0707764	0.245248	0.772895	1.073341	0.66371	1.735791	0.99410703
HSPB1	Wald ratio	1	-0.106422	0.391946	0.785989	0.899045	0.417013	1.938263	0.99410703
PTPN12	Wald ratio	1	-0.060064	0.158629	0.704955	0.941705	0.69006	1.285117	0.99410703
CACNA2D1	Wald ratio	1	0.1107297	0.398028	0.780862	1.117093	0.512013	2.437238	0.99410703
SEMA3A	Wald ratio	1	0.0127324	0.19038	0.946678	1.012814	0.697388	1.470905	0.99410703
PON3	Wald ratio	1	0.0265588	0.06878	0.699394	1.026915	0.897402	1.175118	0.99410703
SLC25A13	Wald ratio	1	-0.013051	0.13051	0.920344	0.987034	0.764257	1.274748	0.99410703
TRRAP	Wald ratio	1	-0.073821	0.295285	0.802587	0.928838	0.520701	1.65688	0.99410703
BLK	Wald ratio	1	0.0241626	0.080794	0.76489	1.024457	0.874421	1.200236	0.99410703
FDFT1	Wald ratio	1	0.0069574	0.089181	0.937817	1.006982	0.845492	1.199316	0.99410703
CCN3	Wald ratio	1	-0.008177	0.097309	0.93303	0.991856	0.81963	1.200272	0.99410703
LY6D	Wald ratio	1	0.0100464	0.031357	0.748674	1.010097	0.949886	1.074125	0.99410703
MFSD3	Wald ratio	1	0.1050851	0.262713	0.689157	1.110805	0.663762	1.858932	0.99410703
TNFRSF10B	Wald ratio	1	0.0231118	0.158114	0.883786	1.023381	0.750668	1.395169	0.99410703

LOXL2	Wald ratio	1	0.0175948	0.114366	0.877731	1.01775	0.813375	1.273479	0.99410703
ADAM7	Wald ratio	1	0.0672595	0.165879	0.68513	1.069573	0.7727	1.480504	0.99410703
PBK	Wald ratio	1	-0.009266	0.131117	0.943659	0.990777	0.766244	1.281105	0.99410703
DUSP4	Wald ratio	1	-0.071475	0.202512	0.724133	0.93102	0.626004	1.384652	0.99410703
MBOAT4	Wald ratio	1	0.0152972	0.087959	0.861934	1.015415	0.854617	1.206467	0.99410703
ADGRA2	Wald ratio	1	0.0158762	0.293709	0.956892	1.016003	0.571327	1.806779	0.99410703
ADAM3A	Wald ratio	1	0.0471263	0.126497	0.709484	1.048254	0.81807	1.343208	0.99410703
DKK4	Wald ratio	1	0.0309608	0.092882	0.738883	1.031445	0.859772	1.237397	0.99410703
FNTA	Wald ratio	1	-0.018726	0.182576	0.918309	0.981449	0.686208	1.403716	0.99410703
PRKDC	Wald ratio	1	-0.034219	0.201711	0.865291	0.96636	0.650787	1.434957	0.99410703
DEFA1	Wald ratio	1	0.0257978	0.065327	0.692913	1.026133	0.90281	1.166302	0.99410703
DEFA1B	Wald ratio	1	-0.023786	0.11621	0.837823	0.976495	0.777589	1.226281	0.99410703
HNF4G	Wald ratio	1	-0.033555	0.086038	0.696537	0.967002	0.816941	1.144628	0.99410703
IL7	Wald ratio	1	-0.011246	0.089965	0.900524	0.988817	0.828965	1.179494	0.99410703
CNGB3	Wald ratio	1	0.0320783	0.11813	0.785967	1.032598	0.819177	1.301623	0.99410703
GABBR2	Wald ratio	1	0.0899774	0.397217	0.820798	1.09415	0.502295	2.383389	0.99410703
OR13C3	Wald ratio	1	0.0430297	0.108052	0.690461	1.043969	0.844718	1.290219	0.99410703
BSPRY	Wald ratio	1	-0.001393	0.023222	0.952156	0.998608	0.954175	1.045109	0.99410703
OR1L6	Wald ratio	1	0.0350789	0.114828	0.759994	1.035701	0.826972	1.297115	0.99410703
OR1J1	Wald ratio	1	0.0159137	0.088056	0.856585	1.016041	0.854981	1.207441	0.99410703
HSPA5	Wald ratio	1	-0.011432	0.20993	0.956572	0.988633	0.655148	1.491871	0.99410703
SLC2A8	Wald ratio	1	-0.025816	0.163499	0.87454	0.974515	0.70732	1.342645	0.99410703
STKLD1	Wald ratio	1	-0.015839	0.105955	0.881171	0.984286	0.799707	1.211468	0.99410703
PTGDS	Wald ratio	1	0.0141301	0.170473	0.933941	1.01423	0.726152	1.416596	0.99410703
NDOR1	Wald ratio	1	-0.10545	0.511431	0.836646	0.89992	0.330267	2.452124	0.99410703
ABCA2	Wald ratio	1	-0.04909	0.378692	0.896859	0.952096	0.453243	2	0.99410703
EHMT1	Wald ratio	1	-0.02695	0.201223	0.893459	0.97341	0.656162	1.444044	0.99410703
PLIN2	Wald ratio	1	0.0257225	0.159158	0.871608	1.026056	0.751092	1.401681	0.99410703
PLAA	Wald ratio	1	-0.042458	0.187399	0.820764	0.958431	0.66381	1.383816	0.99410703
B4GALT1	Wald ratio	1	0.0147231	0.176678	0.933586	1.014832	0.717799	1.43478	0.99410703
CD72	Wald ratio	1	-0.018801	0.084602	0.824141	0.981375	0.831419	1.158377	0.99410703
SLC25A51	Wald ratio	1	-0.056832	0.157867	0.718847	0.944753	0.693329	1.287351	0.99410703
JAK2	Wald ratio	1	0.0696164	0.276725	0.801372	1.072097	0.623276	1.844112	0.99410703
RLN1	Wald ratio	1	0.0166927	0.092969	0.857505	1.016833	0.847448	1.220074	0.99410703
PDCD1LG2	Wald ratio	1	0.0096014	0.065929	0.884213	1.009648	0.887257	1.14892	0.99410703
PTAR1	Wald ratio	1	-0.098811	0.286932	0.730567	0.905914	0.516233	1.589747	0.99410703
TRPM6	Wald ratio	1	0.0946921	0.296128	0.749145	1.09932	0.615255	1.964235	0.99410703
PCSK5	Wald ratio	1	0.0227844	0.285475	0.936387	1.023046	0.584648	1.790177	0.99410703
CDK20	Wald ratio	1	-0.032652	0.256189	0.898583	0.967876	0.585797	1.59916	0.99410703
DAPK1	Wald ratio	1	-0.06021	0.264401	0.819862	0.941567	0.560774	1.580935	0.99410703
WNK2	Wald ratio	1	0.0794558	0.271246	0.769576	1.082698	0.636236	1.842451	0.99410703
OLFM3	Inverse variance weighted	2	0.0460616	0.306941	0.880712	1.047139	0.573761	1.911073	0.99410703
SRM	Inverse variance weighted	2	0.0670725	0.24863	0.787339	1.069373	0.656887	1.740875	0.99410703
CD53	Inverse variance weighted	2	-0.004859	0.035564	0.891316	0.995152	0.928148	1.066994	0.99410703
OVGP1	Inverse variance weighted	2	-0.008356	0.103045	0.935373	0.991679	0.810322	1.213626	0.99410703
BCL2L15	Inverse variance weighted	2	0.0198747	0.128542	0.877124	1.020073	0.792893	1.312346	0.99410703
CTSS	Inverse variance weighted	2	-0.00648	0.06946	0.92567	0.993541	0.867082	1.138443	0.99410703
KCNN3	Inverse variance weighted	2	-0.011315	0.052024	0.827824	0.988749	0.8929	1.094887	0.99410703
SLAMF7	Inverse variance weighted	2	0.0349447	0.096572	0.717463	1.035562	0.856984	1.251354	0.99410703
FCGR3A	Inverse variance weighted	2	0.0301384	0.118366	0.799017	1.030597	0.817209	1.299704	0.99410703
ALDH9A1	Inverse variance weighted	2	-0.011998	0.075586	0.873881	0.988074	0.85202	1.145854	0.99410703
FMO2	Inverse variance weighted	2	0.0183016	0.061453	0.765846	1.01847	0.902897	1.148836	0.99410703
ATP13A2	Inverse variance weighted	2	0.0110701	0.192321	0.954098	1.011132	0.693587	1.474058	0.99410703
AKR7A2	Inverse variance weighted	2	0.0101578	0.117127	0.93089	1.01021	0.802992	1.270901	0.99410703
CFHR4	Inverse variance weighted	2	-0.006271	0.026106	0.810152	0.993748	0.944179	1.045919	0.99410703
CFHR3	Inverse variance weighted	2	0.0191408	0.062022	0.757615	1.019325	0.902649	1.151083	0.99410703
NR5A2	Inverse variance weighted	2	0.0207265	0.059676	0.728352	1.020943	0.908248	1.147621	0.99410703
TNNT2	Inverse variance weighted	2	0.027845	0.124614	0.823185	1.028236	0.805414	1.312703	0.99410703
CDA	Inverse variance weighted	2	0.0089399	0.053259	0.866694	1.00898	0.908967	1.119998	0.99410703
FMOD	Inverse variance weighted	3	0.0296879	0.111244	0.789567	1.030133	0.828325	1.281108	0.99410703
PINK1	Inverse variance weighted	2	-0.035363	0.145082	0.80743	0.965255	0.726349	1.28274	0.99410703
MFSD4A	Inverse variance weighted	2	0.0157965	0.163922	0.92323	1.015922	0.736761	1.400857	0.99410703
CD46	Inverse variance weighted	2	0.0066551	0.058249	0.909037	1.006677	0.898066	1.128424	0.99410703
FLVCR1	Inverse variance weighted	3	-0.00385	0.067097	0.954242	0.996157	0.873401	1.136167	0.99410703
PTPN14	Inverse variance weighted	3	0.0059828	0.061635	0.922673	1.006001	0.891524	1.135176	0.99410703

EPHA8	Inverse variance weighted	3	-0.017935	0.102567	0.861192	0.982225	0.80335	1.20093	0.99410703
TLR5	Inverse variance weighted	2	-0.037726	0.136185	0.781765	0.962977	0.737383	1.257589	0.99410703
ACTN2	Inverse variance weighted	2	0.0124042	0.229709	0.956935	1.012481	0.645438	1.588252	0.99410703
GREM2	Inverse variance weighted	3	-0.019686	0.066236	0.766303	0.980506	0.86113	1.116431	0.99410703
KMO	Inverse variance weighted	3	0.0090816	0.076116	0.905027	1.009123	0.869267	1.171481	0.99410703
OR1C1	Inverse variance weighted	2	0.0091083	0.041325	0.825554	1.00915	0.930634	1.09429	0.99410703
OR14A16	Inverse variance weighted	2	-0.019374	0.062634	0.757073	0.980812	0.867503	1.108922	0.99410703
MAP3K6	Inverse variance weighted	2	0.0255173	0.086263	0.767376	1.025846	0.866271	1.214816	0.99410703
EPHA10	Inverse variance weighted	3	-0.016423	0.100032	0.869593	0.983711	0.808571	1.196787	0.99410703
PPIE	Inverse variance weighted	2	0.0552129	0.173328	0.750071	1.056766	0.752383	1.484289	0.99410703
CYP4Z2P	Inverse variance weighted	2	0.0092366	0.09029	0.918519	1.009279	0.84558	1.204669	0.99410703
SLC5A9	Inverse variance weighted	2	-0.015743	0.051595	0.760264	0.98438	0.889702	1.089133	0.99410703
PODN	Inverse variance weighted	2	0.048771	0.195108	0.802611	1.04998	0.716311	1.539076	0.99410703
TM2D1	Inverse variance weighted	2	0.0262015	0.104012	0.801112	1.026548	0.837225	1.258682	0.99410703
GPR153	Inverse variance weighted	2	-0.025924	0.154367	0.86663	0.974409	0.720015	1.318685	0.99410703
PTGER3	Inverse variance weighted	3	0.0017118	0.02124	0.935766	1.001713	0.960868	1.044295	0.99410703
NEGR1	Inverse variance weighted	2	-0.041887	0.355692	0.906257	0.958978	0.477571	1.925662	0.99410703
MCOLN3	Inverse variance weighted	3	0.004057	0.024001	0.865771	1.004065	0.957926	1.052427	0.99410703
CTBS	Inverse variance weighted	2	0.0287847	0.083615	0.730657	1.029203	0.873627	1.212483	0.99410703
H6PD	Inverse variance weighted	2	-0.015291	0.115617	0.894782	0.984825	0.785135	1.235305	0.99410703
GSTO1	Inverse variance weighted	3	0.0224407	0.071889	0.754922	1.022694	0.888285	1.177441	0.99410703
GSTO2	Inverse variance weighted	3	0.0044279	0.018609	0.811928	1.004438	0.968462	1.04175	0.99410703
CASP7	Inverse variance weighted	3	0.0117775	0.033252	0.723195	1.011847	0.948005	1.079989	0.99410703
CCDC3	Inverse variance weighted	3	0.0196235	0.072729	0.787302	1.019817	0.88433	1.176063	0.99410703
NMT2	Inverse variance weighted	2	-0.019912	0.067455	0.76785	0.980285	0.858882	1.118848	0.99410703
BAMBI	Inverse variance weighted	2	0.0128885	0.052338	0.805485	1.012972	0.914211	1.122402	0.99410703
AKR1C2	Inverse variance weighted	2	-0.055325	0.165883	0.738745	0.946178	0.683551	1.30971	0.99410703
DKK1	Inverse variance weighted	2	-0.023571	0.076771	0.758818	0.976704	0.84026	1.135304	0.99410703
KCNMA1	Inverse variance weighted	2	0.0444847	0.271533	0.869867	1.045489	0.614025	1.780134	0.99410703
SFTPA2	Inverse variance weighted	3	-0.007574	0.051515	0.883115	0.992455	0.89714	1.097896	0.99410703
MINPP1	Inverse variance weighted	2	-0.01693	0.205895	0.934468	0.983213	0.656729	1.472003	0.99410703
LIPA	Inverse variance weighted	2	-0.025034	0.26373	0.924376	0.975277	0.581616	1.635382	0.99410703
DKK3	Inverse variance weighted	2	0.031013	0.087353	0.722566	1.031499	0.869186	1.224122	0.99410703
SLC37A4	Inverse variance weighted	2	-0.013316	0.122794	0.913646	0.986772	0.775698	1.255282	0.99410703
GRIK4	Inverse variance weighted	2	-0.036348	0.296235	0.902345	0.964305	0.539578	1.723355	0.99410703
NTM	Inverse variance weighted	2	-0.033306	0.165873	0.84086	0.967242	0.698781	1.338843	0.99410703
DEAF1	Inverse variance weighted	2	0.0348972	0.192611	0.856227	1.035513	0.709907	1.510462	0.99410703
INS-IGF2	Inverse variance weighted	2	0.0143106	0.079334	0.856852	1.014413	0.868329	1.185075	0.99410703
CHID1	Inverse variance weighted	3	0.0098779	0.177898	0.95572	1.009927	0.712623	1.431264	0.99410703
SLC6A5	Inverse variance weighted	2	0.0480585	0.132411	0.716642	1.049232	0.809396	1.360135	0.99410703
SLC17A6	Inverse variance weighted	2	-0.016517	0.08738	0.850072	0.983618	0.828795	1.167364	0.99410703
TH	Inverse variance weighted	2	-0.032053	0.216014	0.882039	0.968455	0.634169	1.478952	0.99410703
CHRNA10	Inverse variance weighted	2	-0.038353	0.102326	0.707801	0.962373	0.787484	1.176103	0.99410703
HRAS	Inverse variance weighted	2	0.022854	0.122783	0.85234	1.023117	0.804286	1.301488	0.99410703
OR52K2	Inverse variance weighted	2	0.0087258	0.065223	0.893573	1.008764	0.887709	1.146327	0.99410703
FJX1	Inverse variance weighted	2	-0.054669	0.28666	0.848752	0.946798	0.539818	1.660609	0.99410703
OR52B4	Inverse variance weighted	2	0.0172185	0.12021	0.886103	1.017368	0.80381	1.287664	0.99410703
OR51C1P	Inverse variance weighted	2	0.024171	0.077137	0.754012	1.024466	0.880719	1.191674	0.99410703
OR52I1	Inverse variance weighted	2	-0.005885	0.072798	0.935571	0.994132	0.86194	1.146599	0.99410703
OR51E2	Inverse variance weighted	2	-0.002314	0.024741	0.925475	0.997688	0.950462	1.047262	0.99410703
TRIM22	Inverse variance weighted	2	-0.019569	0.140533	0.889252	0.980621	0.744521	1.291592	0.99410703
OR56B4	Inverse variance weighted	2	-0.011809	0.065136	0.856132	0.98826	0.869814	1.122836	0.99410703
OR51B5	Inverse variance weighted	2	0.0034385	0.053208	0.948473	1.003444	0.904069	1.113743	0.99410703
SERPING1	Inverse variance weighted	2	0.0202103	0.112585	0.857536	1.020416	0.818357	1.272364	0.99410703
OR52L2P	Inverse variance weighted	2	-0.017259	0.087547	0.843715	0.982889	0.827909	1.16688	0.99410703
PGA3	Inverse variance weighted	2	-0.012823	0.070639	0.855951	0.987259	0.859611	1.133862	0.99410703
KCNK4	Inverse variance weighted	2	0.0425915	0.110322	0.699448	1.043511	0.8406	1.295403	0.99410703
GPR152	Inverse variance weighted	2	0.039065	0.171199	0.819503	1.039838	0.743427	1.454431	0.99410703
GAL	Inverse variance weighted	2	0.0073958	0.035187	0.833525	1.007423	0.940286	1.079354	0.99410703
DHCR7	Inverse variance weighted	2	0.026274	0.093475	0.778648	1.026622	0.854758	1.233043	0.99410703
HEPHL1	Inverse variance weighted	3	-0.0103	0.039137	0.792424	0.989753	0.916669	1.068665	0.99410703
ALDH1L2	Inverse variance weighted	3	0.0200657	0.062546	0.748351	1.020268	0.902556	1.153332	0.99410703
HCAR3	Inverse variance weighted	2	0.0265757	0.08025	0.740523	1.026932	0.877468	1.201854	0.99410703
HTR7P1	Inverse variance weighted	2	0.0650384	0.338859	0.847794	1.0672	0.549292	2.073427	0.99410703
DUSP16	Inverse variance weighted	2	-0.053351	0.139503	0.70214	0.948048	0.721245	1.24617	0.99410703

HEBP1	Inverse variance weighted	2	0.0186847	0.114106	0.869929	1.01886	0.814677	1.274219	0.99410703
FZD10	Inverse variance weighted	2	0.0143059	0.110067	0.896587	1.014409	0.817565	1.258647	0.99410703
PLBD1	Inverse variance weighted	2	0.0036681	0.063441	0.953893	1.003675	0.88632	1.136568	0.99410703
PYROXD1	Inverse variance weighted	3	-0.017725	0.063598	0.780474	0.982431	0.867295	1.112853	0.99410703
ADAMTS20	Inverse variance weighted	2	-0.006001	0.043973	0.891447	0.994017	0.911933	1.083489	0.99410703
KCNA1	Inverse variance weighted	2	0.0261694	0.189794	0.890333	1.026515	0.707635	1.489092	0.99410703
KRT8	Inverse variance weighted	2	-0.019774	0.120995	0.870182	0.98042	0.773428	1.242811	0.99410703
OR6C3	Inverse variance weighted	2	-0.057757	0.165297	0.726779	0.943879	0.682674	1.305028	0.99410703
PRIM1	Inverse variance weighted	2	-0.023199	0.05851	0.691735	0.977068	0.871204	1.095795	0.99410703
CD4	Inverse variance weighted	2	0.0048007	0.089439	0.957194	1.004812	0.843243	1.197339	0.99410703
TPH2	Inverse variance weighted	3	-0.042799	0.120976	0.723502	0.958104	0.75585	1.214478	0.99410703
DCN	Inverse variance weighted	2	-0.019465	0.116432	0.867227	0.980723	0.780617	1.232126	0.99410703
LTA4H	Inverse variance weighted	2	0.0490622	0.123565	0.691325	1.050286	0.824379	1.338098	0.99410703
APAF1	Inverse variance weighted	2	-0.050565	0.301478	0.866802	0.950693	0.526522	1.716578	0.99410703
TMCO3	Inverse variance weighted	2	-0.006425	0.034691	0.853057	0.993595	0.928283	1.063503	0.99410703
C1QTNF9B	Inverse variance weighted	3	0.0063405	0.0495	0.898078	1.006361	0.91331	1.108892	0.99410703
SHISA2	Inverse variance weighted	2	0.0182454	0.077079	0.812882	1.018413	0.875614	1.1845	0.99410703
FLT3	Inverse variance weighted	3	-0.003546	0.028883	0.902279	0.99646	0.941616	1.054498	0.99410703
HMGB1	Inverse variance weighted	3	0.0141562	0.093535	0.879702	1.014257	0.844364	1.218334	0.99410703
DHRS12	Inverse variance weighted	2	-0.027018	0.088809	0.760959	0.973344	0.817844	1.15841	0.99410703
GUCY1B2	Inverse variance weighted	2	0.0048847	0.020609	0.812643	1.004897	0.965114	1.046319	0.99410703
DCT	Inverse variance weighted	2	-0.004753	0.022788	0.834763	0.995258	0.951783	1.040718	0.99410703
DIO3	Inverse variance weighted	2	-0.044135	0.110628	0.689927	0.956824	0.770307	1.188504	0.99410703
OR4H12P	Inverse variance weighted	2	-0.012084	0.18532	0.948008	0.987988	0.687075	1.42069	0.99410703
OR11H7	Inverse variance weighted	2	0.0215333	0.075178	0.774548	1.021767	0.881777	1.183981	0.99410703
TRAV29DV5	Inverse variance weighted	3	-0.030566	0.107935	0.777033	0.969896	0.784962	1.1984	0.99410703
DHRS4	Inverse variance weighted	5	-0.00544	0.047983	0.909736	0.994575	0.905302	1.092651	0.99410703
DHRS4L2	Inverse variance weighted	5	0.0034817	0.038761	0.928427	1.003488	0.930075	1.082695	0.99410703
DHRS7	Inverse variance weighted	2	0.0089266	0.165421	0.956965	1.008967	0.729571	1.395358	0.99410703
PTGR2	Inverse variance weighted	2	0.0077663	0.034977	0.824282	1.007796	0.941023	1.079309	0.99410703
GSTZ1	Inverse variance weighted	3	-0.01791	0.048376	0.711221	0.98225	0.893394	1.079943	0.99410703
VASH1	Inverse variance weighted	2	-0.026107	0.163651	0.873252	0.974231	0.706903	1.342654	0.99410703
GPR65	Inverse variance weighted	2	-0.023946	0.093526	0.797922	0.976338	0.81281	1.172766	0.99410703
SERPINA12	Inverse variance weighted	2	0.0329651	0.20481	0.872129	1.033514	0.691797	1.544025	0.99410703
PCSK6	Inverse variance weighted	2	-0.01442	0.095582	0.880084	0.985684	0.817291	1.188772	0.99410703
GABRG3	Inverse variance weighted	2	-0.010378	0.130999	0.936855	0.989675	0.765568	1.279387	0.99410703
CHRFAM7A	Inverse variance weighted	2	-0.009251	0.032923	0.778729	0.990792	0.928876	1.056835	0.99410703
CHRM5	Inverse variance weighted	2	0.0057661	0.016543	0.727423	1.005783	0.973694	1.038929	0.99410703
GPR176	Inverse variance weighted	2	0.039403	0.124288	0.751221	1.04019	0.815298	1.327115	0.99410703
LTK	Inverse variance weighted	2	-0.0083	0.148602	0.95546	0.991735	0.741144	1.327053	0.99410703
SORD	Inverse variance weighted	3	-0.028735	0.099916	0.773663	0.971674	0.79886	1.181874	0.99410703
ANXA2	Inverse variance weighted	2	0.0158434	0.207286	0.939075	1.01597	0.676761	1.525196	0.99410703
THSD4	Inverse variance weighted	4	0.0100743	0.048097	0.834092	1.010125	0.919251	1.109983	0.99410703
CYP1A1	Inverse variance weighted	2	-0.032065	0.083353	0.700462	0.968443	0.822475	1.140316	0.99410703
PEAK1	Inverse variance weighted	2	0.033859	0.153581	0.82551	1.034439	0.76555	1.39777	0.99410703
MFGE8	Inverse variance weighted	3	0.0070011	0.077418	0.927944	1.007026	0.865248	1.172034	0.99410703
BLM	Inverse variance weighted	2	0.0324629	0.12272	0.791373	1.032996	0.812152	1.313893	0.99410703
IGFALS	Inverse variance weighted	2	-0.0193	0.076774	0.80152	0.980885	0.843853	1.140171	0.99410703
TXNDC11	Inverse variance weighted	2	-0.006989	0.03994	0.861081	0.993035	0.918262	1.073896	0.99410703
EARS2	Inverse variance weighted	3	-0.018355	0.07408	0.804308	0.981812	0.849123	1.135237	0.99410703
SULT1A1	Inverse variance weighted	2	-0.009436	0.027959	0.735738	0.990608	0.937784	1.046408	0.99410703
C16orf89	Inverse variance weighted	2	-0.047149	0.194046	0.808023	0.953945	0.652151	1.395401	0.99410703
CES1	Inverse variance weighted	4	0.0055772	0.036499	0.87855	1.005593	0.936168	1.080166	0.99410703
BBS2	Inverse variance weighted	3	0.0419541	0.16239	0.796134	1.042847	0.758562	1.433671	0.99410703
KARS1	Inverse variance weighted	3	0.0273265	0.131273	0.8351	1.027703	0.794558	1.329259	0.99410703
VAT1L	Inverse variance weighted	2	0.0279146	0.137749	0.83941	1.028308	0.784998	1.347032	0.99410703
PLCG2	Inverse variance weighted	2	0.0128946	0.084721	0.879029	1.012978	0.857994	1.195958	0.99410703
HSDL1	Inverse variance weighted	2	-0.01642	0.065319	0.801517	0.983714	0.865503	1.118071	0.99410703
SLC22A31	Inverse variance weighted	2	0.0148125	0.055682	0.790225	1.014923	0.909987	1.131959	0.99410703
CDK10	Inverse variance weighted	2	-0.008394	0.065818	0.898518	0.991641	0.871624	1.128184	0.99410703
CHMP1A	Inverse variance weighted	2	-0.061783	0.168708	0.714208	0.940087	0.6754	1.308504	0.99410703
SERPINF1	Inverse variance weighted	2	0.0088314	0.042169	0.834114	1.008871	0.928838	1.095799	0.99410703
KCNJ12	Inverse variance weighted	2	-0.018718	0.232725	0.935894	0.981456	0.621972	1.548711	0.99410703
P2RX5	Inverse variance weighted	3	0.0370254	0.114129	0.745622	1.037719	0.82972	1.297861	0.99410703
SLC6A4	Inverse variance weighted	2	-0.047877	0.136874	0.726498	0.953251	0.72895	1.24657	0.99410703

CCL13	Inverse variance weighted	2	0.0150402	0.082791	0.855846	1.015154	0.863095	1.194002	0.99410703
CYB5D2	Inverse variance weighted	2	-0.050969	0.177792	0.774361	0.950308	0.670694	1.346494	0.99410703
ATP2A3	Inverse variance weighted	2	0.0166898	0.077192	0.828823	1.01683	0.874059	1.182921	0.99410703
IGFBP4	Inverse variance weighted	2	-0.06746	0.214505	0.753149	0.934766	0.613921	1.423289	0.99410703
DUSP3	Inverse variance weighted	2	0.046688	0.131262	0.722076	1.047795	0.81011	1.355216	0.99410703
MAPT	Inverse variance weighted	2	-0.014079	0.039395	0.720809	0.98602	0.91275	1.065171	0.99410703
NPEPPS	Inverse variance weighted	2	-0.020432	0.341668	0.952315	0.979775	0.501526	1.914079	0.99410703
SCPEP1	Inverse variance weighted	2	0.0801227	0.218393	0.713713	1.08342	0.706151	1.66225	0.99410703
OR4D2	Inverse variance weighted	2	0.0354175	0.09647	0.713517	1.036052	0.857561	1.251694	0.99410703
EPX	Inverse variance weighted	2	0.0205411	0.129856	0.874312	1.020754	0.79138	1.316609	0.99410703
PRKCA	Inverse variance weighted	2	0.0134007	0.18431	0.942039	1.013491	0.706206	1.454481	0.99410703
ABCA6	Inverse variance weighted	2	0.0233122	0.118106	0.843528	1.023586	0.812064	1.290204	0.99410703
KCNJ2	Inverse variance weighted	2	-0.068978	0.175855	0.694877	0.933347	0.66123	1.317449	0.99410703
TNFSF13	Inverse variance weighted	3	0.0512308	0.151828	0.735795	1.052566	0.781647	1.417385	0.99410703
CD300C	Inverse variance weighted	2	0.0571976	0.221739	0.796446	1.058865	0.685634	1.635268	0.99410703
GUCY2D	Inverse variance weighted	2	0.0034332	0.060141	0.954478	1.003439	0.891862	1.128975	0.99410703
PRPSAP1	Inverse variance weighted	4	0.0027338	0.032235	0.932415	1.002738	0.941343	1.068136	0.99410703
MC5R	Inverse variance weighted	2	-0.033075	0.099959	0.740729	0.967466	0.795333	1.176853	0.99410703
HMSD	Inverse variance weighted	2	-0.011322	0.073315	0.877271	0.988742	0.856399	1.141537	0.99410703
P2RY11	Inverse variance weighted	2	0.0520109	0.137232	0.704687	1.053387	0.80496	1.378484	0.99410703
ADGRE2	Inverse variance weighted	3	-0.034641	0.100383	0.730029	0.965952	0.793429	1.175989	0.99410703
NOTCH3	Inverse variance weighted	2	0.0314292	0.183142	0.863743	1.031928	0.720702	1.477554	0.99410703
CYP4F8	Inverse variance weighted	2	0.0102793	0.086503	0.905409	1.010332	0.852768	1.197009	0.99410703
AMH	Inverse variance weighted	3	-0.008189	0.04938	0.868288	0.991845	0.900349	1.092639	0.99410703
GDF1	Inverse variance weighted	2	-0.014582	0.152907	0.924025	0.985524	0.730314	1.329916	0.99410703
AKT2	Inverse variance weighted	3	0.0335584	0.167529	0.841235	1.034128	0.744682	1.436076	0.99410703
CEACAM8	Inverse variance weighted	2	0.0242609	0.064779	0.708019	1.024558	0.902392	1.163262	0.99410703
ELSPBP1	Inverse variance weighted	2	-0.073615	0.366098	0.840636	0.929029	0.453315	1.903962	0.99410703
PTH2	Inverse variance weighted	2	0.0168647	0.108596	0.876586	1.017008	0.822027	1.258238	0.99410703
KLK7	Inverse variance weighted	3	-0.005973	0.057319	0.917005	0.994045	0.888413	1.112236	0.99410703
SIGLEC6	Inverse variance weighted	2	-0.006615	0.020272	0.744201	0.993407	0.95471	1.033673	0.99410703
SIGLEC12	Inverse variance weighted	3	-0.005313	0.01951	0.785367	0.994701	0.957383	1.033474	0.99410703
PTPRH	Inverse variance weighted	2	0.0114317	0.075391	0.879477	1.011497	0.872551	1.17257	0.99410703
GALP	Inverse variance weighted	2	0.0219276	0.105578	0.83547	1.02217	0.8311	1.257166	0.99410703
ADGRE4P	Inverse variance weighted	3	0.0009086	0.015958	0.954594	1.000909	0.970087	1.03271	0.99410703
CLEC4M	Inverse variance weighted	2	-0.006651	0.029689	0.822737	0.993371	0.937216	1.052891	0.99410703
KCNF1	Inverse variance weighted	2	-0.058212	0.384849	0.87977	0.94345	0.44374	2.005896	0.99410703
SULT1C2	Inverse variance weighted	3	-0.006402	0.019184	0.738588	0.993618	0.956951	1.03169	0.99410703
GPD2	Inverse variance weighted	2	0.0233988	0.154148	0.879349	1.023675	0.756743	1.384763	0.99410703
MAP3K20	Inverse variance weighted	3	-0.032289	0.101001	0.749203	0.968227	0.794335	1.180186	0.99410703
FRZB	Inverse variance weighted	2	0.0600548	0.164317	0.714753	1.061895	0.769505	1.465383	0.99410703
PPIL3	Inverse variance weighted	2	-0.008925	0.022466	0.691176	0.991115	0.948421	1.035731	0.99410703
APOB	Inverse variance weighted	2	-0.021535	0.092964	0.816809	0.978695	0.815671	1.174301	0.99410703
PECR	Inverse variance weighted	2	0.0230994	0.088471	0.794019	1.023368	0.860446	1.217139	0.99410703
COL4A3	Inverse variance weighted	2	-0.020499	0.075508	0.78602	0.97971	0.844937	1.13598	0.99410703
RNPEPL1	Inverse variance weighted	2	0.0267903	0.142774	0.851159	1.027152	0.776431	1.358836	0.99410703
OR6B3	Inverse variance weighted	2	0.0160857	0.146123	0.912343	1.016216	0.763139	1.353219	0.99410703
BOK	Inverse variance weighted	2	-0.029297	0.195099	0.880636	0.971128	0.66253	1.423469	0.99410703
RASGRP3	Inverse variance weighted	2	-0.026813	0.113336	0.812982	0.973543	0.779618	1.215707	0.99410703
CDKL4	Inverse variance weighted	2	-0.022831	0.080355	0.776315	0.977428	0.834998	1.144153	0.99410703
CYP26B1	Inverse variance weighted	2	0.0255734	0.142695	0.857767	1.025903	0.775607	1.356973	0.99410703
PLGLB1	Inverse variance weighted	3	-0.015467	0.044051	0.725504	0.984652	0.903205	1.073445	0.99410703
THNSL2	Inverse variance weighted	3	0.0011858	0.011018	0.91429	1.001187	0.979798	1.023042	0.99410703
RSPO4	Inverse variance weighted	3	-0.020033	0.104626	0.848158	0.980167	0.798437	1.20326	0.99410703
FKBP1A	Inverse variance weighted	2	-0.030161	0.11094	0.785723	0.970289	0.780669	1.205967	0.99410703
ABHD12	Inverse variance weighted	2	0.0110871	0.053047	0.834444	1.011149	0.911298	1.12194	0.99410703
AVP	Inverse variance weighted	2	-0.027003	0.100338	0.78784	0.973359	0.799583	1.184901	0.99410703
ADAM33	Inverse variance weighted	2	-0.051232	0.143391	0.720874	0.950058	0.717288	1.258365	0.99410703
PTPRT	Inverse variance weighted	2	0.0763574	0.349848	0.827228	1.079348	0.543707	2.142686	0.99410703
SLC9A8	Inverse variance weighted	2	-0.024447	0.067253	0.716227	0.97585	0.855335	1.113344	0.99410703
CHGB	Inverse variance weighted	2	0.0353416	0.118854	0.766198	1.035974	0.820687	1.307735	0.99410703
TMX4	Inverse variance weighted	2	-0.011021	0.080124	0.8906	0.98904	0.8453	1.157222	0.99410703
PLCB1	Inverse variance weighted	2	0.0135416	0.113262	0.904832	1.013634	0.81184	1.265587	0.99410703
ANGPT4	Inverse variance weighted	2	-0.00427	0.075998	0.955198	0.995739	0.857937	1.155676	0.99410703
SOD1	Inverse variance weighted	2	-0.066635	0.223752	0.765849	0.935536	0.603392	1.450515	0.99410703

KCNE1	Inverse variance weighted	2	-0.036512	0.118697	0.758379	0.964146	0.764022	1.216689	0.99410703
TMPRSS3	Inverse variance weighted	3	0.0058204	0.097286	0.952293	1.005837	0.83122	1.217137	0.99410703
ICOSLG	Inverse variance weighted	2	0.0124576	0.093705	0.894237	1.012535	0.84265	1.216671	0.99410703
LSS	Inverse variance weighted	2	0.0478717	0.118529	0.6863	1.049036	0.831566	1.323379	0.99410703
TXNRD2	Inverse variance weighted	2	0.0112044	0.050637	0.824882	1.011267	0.915721	1.116783	0.99410703
IGLV2-18	Inverse variance weighted	2	0.0181231	0.179412	0.919539	1.018288	0.716395	1.447402	0.99410703
GGT5	Inverse variance weighted	2	0.0153665	0.113451	0.892259	1.015485	0.813022	1.268367	0.99410703
PIK3IP1	Inverse variance weighted	2	-0.021736	0.11703	0.852658	0.978499	0.777934	1.230773	0.99410703
RFPL2	Inverse variance weighted	3	-0.022822	0.069711	0.743377	0.977436	0.852608	1.12054	0.99410703
CACNG2	Inverse variance weighted	2	-0.008842	0.10613	0.933604	0.991197	0.805046	1.220392	0.99410703
C1QTNF6	Inverse variance weighted	2	-0.012233	0.033227	0.712751	0.987842	0.92556	1.054315	0.99410703
MCHR1	Inverse variance weighted	3	0.0439068	0.126597	0.728725	1.044885	0.81528	1.339153	0.99410703
SCUBE1	Inverse variance weighted	2	-0.0253	0.092629	0.784749	0.975017	0.813138	1.169122	0.99410703
SULT4A1	Inverse variance weighted	3	0.0284178	0.101578	0.779659	1.028825	0.843095	1.255471	0.99410703
PKDREJ	Inverse variance weighted	2	0.0259527	0.090753	0.7749	1.026292	0.859055	1.226088	0.99410703
MAPK12	Inverse variance weighted	3	0.0118043	0.036721	0.74786	1.011874	0.941606	1.087386	0.99410703
ACR	Inverse variance weighted	2	0.0191926	0.153532	0.900518	1.019378	0.754477	1.377288	0.99410703
SLC9C1	Inverse variance weighted	2	-0.044799	0.151928	0.768092	0.956189	0.709938	1.287857	0.99410703
SLC12A8	Inverse variance weighted	3	0.0103257	0.043469	0.812234	1.010379	0.927862	1.100235	0.99410703
KBTBD12	Inverse variance weighted	3	0.0348773	0.098658	0.723701	1.035493	0.853429	1.256396	0.99410703
ATP1B3	Inverse variance weighted	2	0.0215625	0.088518	0.807546	1.021797	0.859045	1.215382	0.99410703
GRK7	Inverse variance weighted	2	-0.013609	0.063366	0.829942	0.986483	0.871267	1.116934	0.99410703
IL12A	Inverse variance weighted	2	0.0210356	0.080312	0.793382	1.021258	0.872514	1.195361	0.99410703
OXNAD1	Inverse variance weighted	2	0.0327217	0.176358	0.852805	1.033263	0.731293	1.459924	0.99410703
SLC7A14	Inverse variance weighted	2	-0.051132	0.155798	0.742765	0.950153	0.700124	1.289472	0.99410703
TNIK	Inverse variance weighted	2	0.022323	0.15351	0.884381	1.022574	0.756876	1.381544	0.99410703
THPO	Inverse variance weighted	2	0.0587827	0.153033	0.70089	1.060545	0.785715	1.431506	0.99410703
ABCC5	Inverse variance weighted	2	0.0572117	0.214345	0.789535	1.05888	0.695654	1.61176	0.99410703
ECE2	Inverse variance weighted	2	-0.011582	0.035895	0.746944	0.988484	0.92133	1.060533	0.99410703
BDH1	Inverse variance weighted	3	-0.037998	0.106285	0.720711	0.962715	0.781675	1.185685	0.99410703
CNTN4	Inverse variance weighted	2	0.0767148	0.274098	0.779569	1.079734	0.630957	1.847709	0.99410703
ITGA9	Inverse variance weighted	2	0.0085148	0.076445	0.911311	1.008551	0.868215	1.171572	0.99410703
CSPG5	Inverse variance weighted	2	-0.034326	0.087192	0.693816	0.966257	0.814467	1.146335	0.99410703
SLC25A26	Inverse variance weighted	4	-0.005845	0.036872	0.874055	0.994172	0.924859	1.068681	0.99410703
HTR1F	Inverse variance weighted	2	0.0157273	0.058225	0.787075	1.015852	0.906291	1.138656	0.99410703
BDH2	Inverse variance weighted	2	0.018056	0.104121	0.862327	1.01822	0.830255	1.248739	0.99410703
SLC9B1	Inverse variance weighted	2	-0.00989	0.038618	0.797867	0.990158	0.917978	1.068014	0.99410703
TACR3	Inverse variance weighted	2	-0.021593	0.065033	0.73986	0.978638	0.86152	1.111678	0.99410703
PLA2G12A	Inverse variance weighted	2	-0.025134	0.069913	0.719221	0.97518	0.850303	1.118396	0.99410703
MFSD8	Inverse variance weighted	2	0.0243139	0.329274	0.941137	1.024612	0.537373	1.953632	0.99410703
NPY2R	Inverse variance weighted	2	-0.020299	0.173293	0.90675	0.979905	0.697708	1.376241	0.99410703
GUCY1B1	Inverse variance weighted	2	-0.021469	0.149276	0.885644	0.97876	0.730483	1.311423	0.99410703
NPY5R	Inverse variance weighted	2	0.0355298	0.133472	0.790088	1.036169	0.797658	1.345998	0.99410703
ADGRA3	Inverse variance weighted	2	-0.020729	0.067088	0.757333	0.979484	0.858798	1.117131	0.99410703
PDGFRA	Inverse variance weighted	3	0.0209072	0.10733	0.845554	1.021127	0.827407	1.260204	0.99410703
UGT2B4	Inverse variance weighted	2	-0.019088	0.083155	0.818441	0.981093	0.833542	1.154763	0.99410703
CDKL2	Inverse variance weighted	2	0.0453094	0.129482	0.726393	1.046352	0.811821	1.348636	0.99410703
NAAA	Inverse variance weighted	3	-0.003538	0.018305	0.846753	0.996469	0.961352	1.032868	0.99410703
BMP2K	Inverse variance weighted	2	-0.021541	0.128483	0.866853	0.978689	0.760814	1.258959	0.99410703
GRID2	Inverse variance weighted	2	0.0179663	0.142887	0.89994	1.018129	0.76944	1.347196	0.99410703
SPOCK1	Inverse variance weighted	2	-0.037873	0.122483	0.757162	0.962835	0.757342	1.224085	0.99410703
ITK	Inverse variance weighted	2	0.0232019	0.083668	0.781543	1.023473	0.868674	1.205858	0.99410703
DRD1	Inverse variance weighted	3	0.0090656	0.077045	0.906332	1.009107	0.867671	1.173597	0.99410703
FGFR4	Inverse variance weighted	2	0.0181602	0.080441	0.821389	1.018326	0.86979	1.192228	0.99410703
SCGB3A1	Inverse variance weighted	2	0.0063751	0.025212	0.800378	1.006396	0.957872	1.057377	0.99410703
PRLR	Inverse variance weighted	2	0.0226065	0.056693	0.690073	1.022864	0.915293	1.143077	0.99410703
OCLN	Inverse variance weighted	2	0.0140584	0.0451	0.755254	1.014158	0.928359	1.107886	0.99410703
SV2C	Inverse variance weighted	2	0.0202266	0.072842	0.78126	1.020433	0.884667	1.177033	0.99410703
CDK19	Inverse variance weighted	2	0.0116857	0.09403	0.901096	1.011754	0.841464	1.216507	0.99410703
CCN2	Inverse variance weighted	2	0.0077668	0.076687	0.919329	1.007797	0.867153	1.171251	0.99410703
SGK1	Inverse variance weighted	3	-0.017917	0.101993	0.860553	0.982242	0.804267	1.199602	0.99410703
CD83	Inverse variance weighted	2	0.0113572	0.041673	0.785215	1.011422	0.932093	1.097503	0.99410703
NMBR	Inverse variance weighted	2	-0.013803	0.120234	0.908604	0.986292	0.779221	1.248391	0.99410703
H4C8	Inverse variance weighted	2	-0.023344	0.06879	0.734342	0.976926	0.853702	1.117937	0.99410703
RXRB	Inverse variance weighted	2	-0.01888	0.136797	0.890228	0.981297	0.75051	1.283052	0.99410703

HSD17B8	Inverse variance weighted	2	0.0099898	0.098698	0.919379	1.01004	0.832387	1.225608	0.99410703
PPIL1	Inverse variance weighted	3	-0.021146	0.055029	0.70077	0.979076	0.878972	1.09058	0.99410703
GSTA1	Inverse variance weighted	3	0.0044412	0.011845	0.707696	1.004451	0.981401	1.028043	0.99410703
LY86	Inverse variance weighted	2	-0.02873	0.081064	0.72303	0.971679	0.828933	1.139006	0.99410703
COL9A1	Inverse variance weighted	2	-0.004934	0.065968	0.940374	0.995078	0.874388	1.132426	0.99410703
EPHA7	Inverse variance weighted	3	-0.043835	0.103504	0.67192	0.957112	0.781373	1.172375	0.99410703
KLHL32	Inverse variance weighted	2	-0.032569	0.137916	0.813316	0.967956	0.738685	1.268387	0.99410703
EPHB6	Inverse variance weighted	2	0.0113751	0.14599	0.937894	1.01144	0.75975	1.34651	0.99410703
TRPV6	Inverse variance weighted	2	0.0393804	0.131086	0.763859	1.040166	0.80449	1.344884	0.99410703
OR2A3P	Inverse variance weighted	2	0.0065994	0.031851	0.835857	1.006621	0.945701	1.071466	0.99410703
OR2A2	Inverse variance weighted	2	-0.004255	0.039928	0.915124	0.995754	0.920798	1.076811	0.99410703
PTPRN2	Inverse variance weighted	2	0.0185402	0.068421	0.786413	1.018713	0.890862	1.164913	0.99410703
VIPR2	Inverse variance weighted	2	0.0063151	0.027419	0.817845	1.006335	0.953681	1.061896	0.99410703
GPNMB	Inverse variance weighted	3	0.0018419	0.018298	0.919818	1.001844	0.966551	1.038425	0.99410703
NPY	Inverse variance weighted	2	-0.072498	0.195669	0.711	0.930068	0.633808	1.364807	0.99410703
STK17A	Inverse variance weighted	2	-0.065847	0.200906	0.743101	0.936274	0.631521	1.388091	0.99410703
VWC2	Inverse variance weighted	2	0.0609347	0.15911	0.701741	1.06283	0.778084	1.45178	0.99410703
STYXL1	Inverse variance weighted	3	0.0054802	0.022542	0.807918	1.005495	0.962038	1.050916	0.99410703
CCL24	Inverse variance weighted	2	-0.041603	0.171517	0.808348	0.959251	0.685384	1.34255	0.99410703
CD36	Inverse variance weighted	2	0.0046361	0.043443	0.915015	1.004647	0.922643	1.093939	0.99410703
HGF	Inverse variance weighted	2	-0.042941	0.119904	0.720247	0.957968	0.757332	1.211757	0.99410703
SLC25A40	Inverse variance weighted	2	-0.021407	0.104004	0.836925	0.978821	0.798313	1.200143	0.99410703
CYP3A5	Inverse variance weighted	2	-0.028547	0.238394	0.904684	0.971857	0.609084	1.550699	0.99410703
CTHRC1	Inverse variance weighted	2	0.0107407	0.14677	0.941663	1.010799	0.758108	1.347715	0.99410703
RSPO2	Inverse variance weighted	2	0.0150685	0.079794	0.850217	1.015183	0.868204	1.187043	0.99410703
SQLE	Inverse variance weighted	2	0.0401821	0.133754	0.763859	1.041	0.800934	1.353022	0.99410703
ADCY8	Inverse variance weighted	2	0.0180907	0.073132	0.804621	1.018255	0.882279	1.175189	0.99410703
CCN4	Inverse variance weighted	2	0.012821	0.069088	0.852778	1.012904	0.884625	1.159783	0.99410703
COL22A1	Inverse variance weighted	2	-0.005955	0.082711	0.942606	0.994063	0.845296	1.169012	0.99410703
TOP1MT	Inverse variance weighted	2	0.0211126	0.081305	0.795117	1.021337	0.870885	1.19778	0.99410703
KBTBD11	Inverse variance weighted	2	0.0375579	0.105234	0.721168	1.038272	0.844762	1.27611	0.99410703
SLC18A1	Inverse variance weighted	2	-0.005849	0.017006	0.730914	0.994169	0.961577	1.027864	0.99410703
SLC25A37	Inverse variance weighted	2	0.0401716	0.131779	0.760487	1.040989	0.804033	1.347779	0.99410703
ADRA1A	Inverse variance weighted	2	-0.017063	0.137929	0.901545	0.983082	0.750209	1.288241	0.99410703
DUSP26	Inverse variance weighted	2	-0.069516	0.173004	0.68782	0.932846	0.664578	1.309404	0.99410703
ADAM9	Inverse variance weighted	2	-0.099735	0.344368	0.772109	0.905077	0.460844	1.777533	0.99410703
DEFB1	Inverse variance weighted	2	-0.024536	0.059277	0.678934	0.975763	0.868733	1.095979	0.99410703
GGH	Inverse variance weighted	2	-0.02636	0.066902	0.693571	0.973984	0.854287	1.110453	0.99410703
ADHFE1	Inverse variance weighted	2	-0.012663	0.059374	0.831115	0.987417	0.878942	1.109279	0.99410703
KCNB2	Inverse variance weighted	2	0.0450753	0.164425	0.783978	1.046107	0.757905	1.4439	0.99410703
RIPK2	Inverse variance weighted	2	0.0222463	0.092353	0.809645	1.022496	0.853197	1.225388	0.99410703
GDF6	Inverse variance weighted	2	-0.066958	0.230538	0.771477	0.935234	0.595226	1.469463	0.99410703
TSTD2	Inverse variance weighted	2	0.021234	0.108914	0.845423	1.021461	0.825111	1.264536	0.99410703
HSDL2	Inverse variance weighted	2	0.0694205	0.387847	0.857946	1.071887	0.501195	2.292405	0.99410703
OR1N1	Inverse variance weighted	2	-0.00771	0.082822	0.925832	0.99232	0.84363	1.167216	0.99410703
NR6A1	Inverse variance weighted	2	0.0295099	0.141432	0.834722	1.02995	0.780596	1.358957	0.99410703
ENDOG	Inverse variance weighted	2	-0.050325	0.154757	0.74504	0.950921	0.702122	1.287881	0.99410703
CRAT	Inverse variance weighted	2	-0.005888	0.087796	0.946532	0.994129	0.83697	1.180799	0.99410703
CEL	Inverse variance weighted	2	-0.037699	0.126744	0.766128	0.963003	0.751175	1.234565	0.99410703
KCNT1	Inverse variance weighted	2	0.0201431	0.092303	0.827252	1.020347	0.851487	1.222695	0.99410703
FREM1	Inverse variance weighted	2	-0.011315	0.040238	0.778556	0.988749	0.913765	1.069886	0.99410703
IFNA21	Inverse variance weighted	2	-0.010176	0.095269	0.914939	0.989876	0.82127	1.193096	0.99410703
TEK	Inverse variance weighted	2	-0.008672	0.059841	0.884771	0.991365	0.881649	1.114735	0.99410703
LINGO2	Inverse variance weighted	2	0.043069	0.181238	0.812162	1.04401	0.731867	1.489283	0.99410703
DNAJA1	Inverse variance weighted	2	0.0397711	0.182547	0.827533	1.040573	0.727587	1.488195	0.99410703
SLC1A1	Inverse variance weighted	2	0.0095887	0.059671	0.872335	1.009635	0.898197	1.134899	0.99410703
MAMDC2	Inverse variance weighted	2	0.0061342	0.064278	0.923971	1.006153	0.887053	1.141245	0.99410703
TRPM3	Inverse variance weighted	2	0.0185917	0.060008	0.756698	1.018766	0.905721	1.14592	0.99410703
MFSD14B	Inverse variance weighted	2	-0.024309	0.063736	0.702901	0.975984	0.86137	1.105848	0.99410703
WIF1	Wald ratio	1	-0.006414	0.132954	0.96152	0.993606	0.76567	1.289398	0.99417698
TSSK4	Wald ratio	1	0.0113589	0.216576	0.958172	1.011424	0.661576	1.546275	0.99417698
TGM1	Wald ratio	1	0.0035356	0.070712	0.960122	1.003542	0.873664	1.152727	0.99417698
SNAP23	Wald ratio	1	0.0109569	0.219137	0.960122	1.011017	0.657999	1.553431	0.99417698
RAMP2	Wald ratio	1	-0.010865	0.21886	0.960405	0.989193	0.644146	1.519072	0.99417698
THOP1	Wald ratio	1	-0.010208	0.20246	0.959788	0.989844	0.665624	1.471988	0.99417698

PLCD4	Wald ratio	1	-0.005604	0.112587	0.960303	0.994412	0.797499	1.239945	0.99417698
CAMK4	Wald ratio	1	0.0138279	0.262731	0.958025	1.013924	0.605849	1.696861	0.99417698
PTP4A3	Wald ratio	1	0.0120024	0.24805	0.961408	1.012075	0.622398	1.645723	0.99417698
SLIT1	Inverse variance weighted	2	-0.022176	0.425553	0.958441	0.978068	0.424748	2.252201	0.99417698
OR10G7	Inverse variance weighted	2	0.0032116	0.066281	0.961355	1.003217	0.880999	1.142389	0.99417698
CNTNAP4	Inverse variance weighted	2	-0.003848	0.076122	0.959683	0.996159	0.858089	1.156445	0.99417698
CCL25	Inverse variance weighted	2	-0.002775	0.057034	0.961197	0.997229	0.891757	1.115176	0.99417698
ABCG2	Inverse variance weighted	2	0.0051732	0.100146	0.958803	1.005187	0.826039	1.223187	0.99417698
SRD5A1	Inverse variance weighted	2	-0.003293	0.063939	0.958925	0.996712	0.879314	1.129784	0.99417698
IL5	Wald ratio	1	0.0051642	0.108449	0.96202	1.005178	0.812698	1.243244	0.99439962
ABCC11	Wald ratio	1	0.0051603	0.111232	0.962998	1.005174	0.808274	1.25004	0.99482326
QSOX2	Wald ratio	1	0.0017258	0.036933	0.962729	1.001727	0.931776	1.07693	0.99482326
ADAMTS13	Inverse variance weighted	2	0.0032412	0.070773	0.963472	1.003247	0.873302	1.152527	0.99501885
GPR148	Wald ratio	1	-0.005886	0.135378	0.96532	0.994131	0.762443	1.296224	0.99663413
HSD3BP1	Wald ratio	1	0.0036212	0.159334	0.981868	1.003628	0.73442	1.371516	0.9967489
TUFT1	Wald ratio	1	0.0066723	0.178485	0.970179	1.006695	0.709526	1.428326	0.9967489
S100A7	Wald ratio	1	0.002828	0.079892	0.971762	1.002832	0.857478	1.172826	0.9967489
FCER1A	Wald ratio	1	-0.001152	0.027418	0.966485	0.998849	0.946588	1.053995	0.9967489
NEK7	Wald ratio	1	-0.012812	0.318167	0.967879	0.98727	0.529183	1.841897	0.9967489
C1QB	Wald ratio	1	0.0099923	0.380883	0.97907	1.010042	0.478768	2.130854	0.9967489
C1QA	Wald ratio	1	0.0023606	0.106229	0.982271	1.002363	0.813957	1.23438	0.9967489
NLRP3	Wald ratio	1	0.0030297	0.111087	0.978242	1.003034	0.806782	1.247025	0.9967489
OR2T12	Wald ratio	1	0.0014884	0.081118	0.985361	1.00149	0.854274	1.174074	0.9967489
SCP2	Wald ratio	1	-0.005999	0.20797	0.976987	0.994019	0.661252	1.494247	0.9967489
FGFR2	Wald ratio	1	0.0049423	0.252056	0.984356	1.004955	0.613186	1.647027	0.9967489
ADAM12	Wald ratio	1	0.0042169	0.105424	0.968093	1.004226	0.816757	1.234724	0.9967489
USP47	Wald ratio	1	-0.00523	0.240602	0.982656	0.994783	0.620761	1.594163	0.9967489
KCNJ5	Wald ratio	1	0.0035863	0.097547	0.970673	1.003593	0.828941	1.215042	0.9967489
OR51M1	Wald ratio	1	-0.002774	0.07144	0.969022	0.997229	0.86693	1.147113	0.9967489
SLC22A10	Wald ratio	1	-0.003792	0.094789	0.968093	0.996216	0.827309	1.199607	0.9967489
CD9	Wald ratio	1	0.0084623	0.211559	0.968093	1.008498	0.666182	1.526714	0.9967489
PDIA3	Wald ratio	1	0.0048662	0.125549	0.969082	1.004878	0.785677	1.285235	0.9967489
SF3B3	Wald ratio	1	0.0088971	0.250899	0.971712	1.008937	0.617013	1.649809	0.9967489
TRIM16	Wald ratio	1	-0.002912	0.074733	0.968921	0.997093	0.861234	1.154383	0.9967489
GP1BA	Wald ratio	1	0.0050038	0.153866	0.974057	1.005016	0.74336	1.358773	0.9967489
LAMA1	Wald ratio	1	-0.002653	0.124679	0.983025	0.997351	0.781122	1.273436	0.9967489
PKN1	Wald ratio	1	-0.005254	0.217145	0.980698	0.99476	0.649951	1.522497	0.9967489
BRD4	Wald ratio	1	0.011288	0.318886	0.971762	1.011352	0.541329	1.889484	0.9967489
SLC27A1	Wald ratio	1	-0.003705	0.125963	0.976536	0.996302	0.77834	1.275301	0.9967489
RYR1	Wald ratio	1	-0.004643	0.127293	0.970904	0.995368	0.775585	1.277432	0.9967489
VN1R1	Wald ratio	1	0.0026186	0.140359	0.985115	1.002622	0.761485	1.320119	0.9967489
DZANK1	Wald ratio	1	-0.002115	0.105756	0.984043	0.997887	0.811074	1.227729	0.9967489
GHRH	Wald ratio	1	0.0022552	0.072729	0.975263	1.002258	0.869102	1.155814	0.9967489
GP1BB	Wald ratio	1	0.0082824	0.238533	0.972301	1.008317	0.631763	1.609311	0.9967489
SLC2A11	Wald ratio	1	-0.001678	0.052571	0.97454	0.998324	0.900579	1.106676	0.9967489
SENP7	Wald ratio	1	0.0034998	0.127159	0.978043	1.003506	0.782132	1.287536	0.9967489
KCNAB1	Wald ratio	1	-0.003814	0.194513	0.984356	0.996193	0.68041	1.458533	0.9967489
LAP3	Wald ratio	1	0.0025589	0.133065	0.984657	1.002562	0.772403	1.301304	0.9967489
ADRB2	Wald ratio	1	0.0034101	0.1185	0.977042	1.003416	0.795449	1.265756	0.9967489
AMD1	Wald ratio	1	0.0053528	0.272995	0.984356	1.005367	0.588771	1.716733	0.9967489
FKBP5	Wald ratio	1	0.0044117	0.222791	0.984201	1.004421	0.649041	1.554389	0.9967489
AKR1B1	Wald ratio	1	0.0041421	0.215388	0.984657	1.004151	0.65835	1.531583	0.9967489
AGR3	Wald ratio	1	-0.002152	0.076925	0.977684	0.997851	0.858195	1.160233	0.9967489
CYP51A1	Wald ratio	1	-0.002245	0.076319	0.976536	0.997758	0.859134	1.158749	0.9967489
NPTX2	Wald ratio	1	0.0082559	0.266941	0.975327	1.00829	0.597532	1.701414	0.9967489
TNC	Wald ratio	1	-0.002164	0.112535	0.984657	0.997838	0.800328	1.244091	0.9967489
IFNB1	Wald ratio	1	-0.00429	0.119052	0.971254	0.995719	0.788493	1.257406	0.9967489
KCNV2	Wald ratio	1	-0.005998	0.155947	0.96932	0.99402	0.732235	1.349397	0.9967489
PRKCQ	Inverse variance weighted	2	0.0067702	0.198094	0.972736	1.006793	0.682841	1.484435	0.9967489
PTPN5	Inverse variance weighted	2	-0.016519	0.514135	0.974369	0.983617	0.359075	2.69443	0.9967489
PRR4	Inverse variance weighted	2	0.0010038	0.03711	0.978421	1.001004	0.93078	1.076527	0.9967489
PTPRR	Inverse variance weighted	2	0.0053175	0.245207	0.982699	1.005332	0.621706	1.625675	0.9967489
TNFRSF19	Inverse variance weighted	2	0.0023484	0.091401	0.979502	1.002351	0.837949	1.199008	0.9967489
ATP12A	Inverse variance weighted	2	-0.001314	0.044441	0.976409	0.998687	0.915379	1.089577	0.9967489
OR4Q3	Inverse variance weighted	2	0.0030839	0.148984	0.983485	1.003089	0.749068	1.343252	0.9967489

FKBP3	Inverse variance weighted	2	-0.007394	0.207857	0.971623	0.992633	0.660476	1.491834	0.9967489
NRG4	Inverse variance weighted	3	-0.002019	0.060903	0.97356	0.997983	0.88569	1.124514	0.9967489
MMP25	Inverse variance weighted	2	0.0013057	0.057157	0.981774	1.001307	0.895188	1.120004	0.9967489
ENGASE	Inverse variance weighted	2	-0.002866	0.102216	0.977633	0.997138	0.816107	1.218327	0.9967489
INSL3	Inverse variance weighted	2	0.0020221	0.080153	0.979873	1.002024	0.856349	1.17248	0.9967489
TNFRSF13C	Inverse variance weighted	2	0.0056301	0.150347	0.970128	1.005646	0.748975	1.350277	0.9967489
CAMK1	Inverse variance weighted	2	-0.002412	0.127201	0.984872	0.997591	0.777457	1.280055	0.9967489
SLCO2A1	Inverse variance weighted	2	0.0021947	0.067527	0.974073	1.002197	0.877956	1.14402	0.9967489
ATP13A4	Inverse variance weighted	2	-0.00227	0.120742	0.985001	0.997733	0.787474	1.264131	0.9967489
MELTF	Inverse variance weighted	2	0.0025074	0.070939	0.971804	1.002511	0.872377	1.152056	0.9967489
OR5K2	Inverse variance weighted	2	-0.001408	0.06808	0.983496	0.998593	0.873852	1.14114	0.9967489
NPFFR2	Inverse variance weighted	2	-0.002236	0.083459	0.978629	0.997767	0.847202	1.17509	0.9967489
HSD17B4	Inverse variance weighted	2	0.0036689	0.08932	0.967235	1.003676	0.842486	1.195706	0.9967489
TMED9	Inverse variance weighted	2	-0.003849	0.157987	0.980563	0.996158	0.730881	1.357719	0.9967489
FAM3C	Inverse variance weighted	2	-0.002928	0.110417	0.978841	0.997076	0.803044	1.23799	0.9967489
FGF17	Inverse variance weighted	2	0.0082043	0.288098	0.977281	1.008238	0.57323	1.77336	0.9967489
GRIN3A	Inverse variance weighted	2	0.0044682	0.178177	0.979993	1.004478	0.708391	1.424321	0.9967489
DPP7	Inverse variance weighted	2	0.010083	0.234972	0.965772	1.010134	0.637333	1.601001	0.9967489
OR13J1	Inverse variance weighted	2	-0.004438	0.173882	0.979639	0.995572	0.708046	1.399859	0.9967489
PPOX	Wald ratio	1	0.001036	0.096867	0.991467	1.001037	0.827933	1.210332	0.99695149
MMP23A	Wald ratio	1	0.00182	0.107985	0.986553	1.001822	0.810721	1.237968	0.99695149
SELP	Wald ratio	1	0.0007755	0.084524	0.99268	1.000776	0.847985	1.181096	0.99695149
FMO6P	Wald ratio	1	0.0006654	0.068535	0.992254	1.000666	0.874885	1.144529	0.99695149
GPR25	Wald ratio	1	-0.001074	0.078131	0.989033	0.998927	0.857092	1.164233	0.99695149
C1QC	Wald ratio	1	-0.001945	0.198354	0.992178	0.998057	0.676571	1.472305	0.99695149
IPP	Wald ratio	1	-0.000705	0.076109	0.992612	0.999296	0.860812	1.160058	0.99695149
NTN1	Wald ratio	1	-0.001786	0.180414	0.9921	0.998215	0.700895	1.42166	0.99695149
FCGRT	Wald ratio	1	0.0017428	0.111538	0.987534	1.001744	0.805033	1.246522	0.99695149
ABCG5	Wald ratio	1	0.0012183	0.087717	0.988919	1.001219	0.843068	1.189037	0.99695149
MFNG	Wald ratio	1	0.0013248	0.143078	0.992612	1.001326	0.756459	1.325457	0.99695149
LGALS1	Wald ratio	1	0.0009705	0.098993	0.992178	1.000971	0.824437	1.215306	0.99695149
MANF	Wald ratio	1	0.0015387	0.163102	0.992473	1.00154	0.7275	1.378808	0.99695149
SCD5	Wald ratio	1	0.0019915	0.205129	0.992254	1.001994	0.670279	1.497869	0.99695149
PKD2	Wald ratio	1	-0.001254	0.124153	0.991941	0.998747	0.783022	1.273904	0.99695149
CLPSL2	Wald ratio	1	-0.001558	0.092696	0.986591	0.998443	0.832567	1.197368	0.99695149
PRH2	Inverse variance weighted	2	0.000419	0.040214	0.991687	1.000419	0.924594	1.082462	0.99695149
WNT10B	Inverse variance weighted	2	0.0008508	0.08749	0.992241	1.000851	0.843134	1.188071	0.99695149
ADAMTS17	Inverse variance weighted	2	0.0023602	0.175924	0.989296	1.002363	0.710029	1.415058	0.99695149
OR4F17	Inverse variance weighted	2	0.0009498	0.077691	0.990246	1.00095	0.859569	1.165585	0.99695149
IHH	Inverse variance weighted	2	0.0008176	0.079333	0.991777	1.000818	0.856693	1.169189	0.99695149
APOD	Inverse variance weighted	2	0.0064687	0.376773	0.986302	1.00649	0.480943	2.106324	0.99695149
PPIC	Inverse variance weighted	2	-0.000819	0.074644	0.991243	0.999181	0.863189	1.156598	0.99695149
SLC22A5	Inverse variance weighted	2	-0.00081	0.050127	0.987113	0.999191	0.905689	1.102345	0.99695149
AKR1B10	Inverse variance weighted	2	-0.000824	0.065758	0.989999	0.999176	0.878351	1.136622	0.99695149
SLC25A18	Inverse variance weighted	2	-0.001683	0.194493	0.993094	0.998318	0.681889	1.461586	0.99708142
RET	Inverse variance weighted	2	-0.002918	0.355022	0.993442	0.997086	0.497201	1.999556	0.99714446
C1R	Wald ratio	1	-0.000772	0.145999	0.995778	0.999228	0.750564	1.330275	0.99723065
LTBP2	Wald ratio	1	0.0006852	0.093186	0.994133	1.000685	0.833636	1.20121	0.99723065
DOT1L	Wald ratio	1	0.0006286	0.112521	0.995543	1.000629	0.802589	1.247535	0.99723065
SLC7A4	Wald ratio	1	-0.001403	0.247008	0.995467	0.998598	0.615365	1.620497	0.99723065
SLURP1	Wald ratio	1	-0.000741	0.107382	0.994497	0.99926	0.809605	1.233343	0.99723065
KCNT2	Inverse variance weighted	2	-0.000846	0.186206	0.996376	0.999155	0.693635	1.439243	0.99723065
CPD	Inverse variance weighted	2	-0.001464	0.319816	0.996348	0.998537	0.533496	1.868948	0.99723065
ITGA3	Inverse variance weighted	2	-0.001161	0.165384	0.994398	0.998839	0.722301	1.381253	0.99723065
ICAM1	Inverse variance weighted	2	0.0003627	0.070851	0.995915	1.000363	0.870659	1.149388	0.99723065
FGFBP1	Inverse variance weighted	2	0.0016746	0.306919	0.995647	1.001676	0.548874	1.828024	0.99723065
ATP7B	Inverse variance weighted	2	-0.000155	0.039454	0.996872	0.999845	0.925441	1.080232	0.99744164
SERPINB9	Inverse variance weighted	2	9.287E-05	0.047755	0.998448	1.000093	0.910731	1.098223	0.99873359
PSMA3	Inverse variance weighted	2	0.0001302	0.173259	0.999401	1.00013	0.712157	1.404551	0.9994006

Table S7. MR results of the association between blood pQTL and ischemic stroke (IVW or Wald ratio methods).

Druggable gene	Method	Nsnp	beta	se	P value	OR	LL	LR	FDR
MMP12	Inverse variance weighted	2	-0.09717	0.019199	4.17E-07	0.907403	0.873891	0.9422	0.000169713
LILRB2	Wald ratio	1	0.275225	0.078479	0.000453	1.316827	1.129084	1.535788	0.092223021
VEGFC	Wald ratio	1	0.082051	0.028078	0.003475	1.085511	1.027387	1.146924	0.282838475
SCARA5	Wald ratio	1	-0.13165	0.043358	0.002395	0.876646	0.805225	0.954403	0.282838475
QDPR	Inverse variance weighted	4	-0.05381	0.018303	0.00328	0.947609	0.914218	0.98222	0.282838475
SMOC1	Inverse variance weighted	2	-0.09256	0.032339	0.004208	0.911594	0.855606	0.971246	0.285417609
OAS1	Inverse variance weighted	2	-0.08335	0.030706	0.006638	0.920028	0.866291	0.9771	0.352168259
MLN	Inverse variance weighted	3	0.051054	0.018905	0.006922	1.05238	1.014099	1.092106	0.352168259
PTGR1	Inverse variance weighted	2	0.0709	0.02781	0.010789	1.073474	1.016528	1.13361	0.487886662
PDGFRB	Wald ratio	1	-0.02688	0.010881	0.013511	0.973481	0.952939	0.994466	0.499918293
SEMA4D	Wald ratio	1	0.035152	0.014141	0.012929	1.035777	1.007462	1.064887	0.499918293
PLAU	Inverse variance weighted	2	-0.08799	0.036568	0.016125	0.915774	0.852435	0.98382	0.534482799
APCS	Inverse variance weighted	2	-0.07391	0.030987	0.017072	0.928757	0.874029	0.986912	0.534482799
CEL	Wald ratio	1	0.097899	0.042165	0.020244	1.102851	1.015372	1.197867	0.549280269
MAPK13	Inverse variance weighted	2	-0.04406	0.018861	0.019492	0.956897	0.922168	0.992933	0.549280269
SIRPG	Wald ratio	1	-0.0917	0.040335	0.022991	0.912375	0.843023	0.987431	0.58483244
ACP5	Inverse variance weighted	2	-0.06067	0.027728	0.028665	0.941133	0.89135	0.993696	0.686274042
BCAN	Wald ratio	1	-0.13192	0.063735	0.038474	0.876414	0.773494	0.993028	0.745665236
LILRB5	Inverse variance weighted	2	0.035181	0.01658	0.033851	1.035807	1.002687	1.070021	0.745665236
TXNDC5	Inverse variance weighted	3	0.080621	0.038881	0.038126	1.08396	1.004423	1.169795	0.745665236
ESAM	Inverse variance weighted	3	-0.08562	0.040573	0.034833	0.917943	0.847772	0.993921	0.745665236
EPHB2	Wald ratio	1	-0.07853	0.04014	0.050432	0.924478	0.854532	1.000149	0.789449915
EPHB2	Wald ratio	1	-0.05848	0.029892	0.050432	0.943199	0.889526	1.000111	0.789449915
EPHB2	Wald ratio	1	-0.08809	0.04503	0.050432	0.915678	0.838326	1.000167	0.789449915
DLK1	Wald ratio	1	0.038462	0.019417	0.047617	1.039211	1.000403	1.079524	0.789449915
LAMB1	Inverse variance weighted	2	-0.05076	0.02583	0.049402	0.950508	0.903584	0.999868	0.789449915
KYNU	Wald ratio	1	0.049515	0.025748	0.05447	1.050761	0.99905	1.105149	0.821090694
QSOX1	Wald ratio	1	0.13251	0.075239	0.078206	1.14169	0.985153	1.3231	0.852941757
TNFRSF6B	Wald ratio	1	0.133333	0.075706	0.078206	1.142631	0.985062	1.325404	0.852941757
PTGDS	Wald ratio	1	-0.09651	0.053562	0.071563	0.907998	0.817507	1.008505	0.852941757
POGLUT1	Wald ratio	1	0.09352	0.050318	0.063086	1.098032	0.99491	1.211843	0.852941757
SCG3	Wald ratio	1	0.042798	0.024691	0.083036	1.043727	0.994419	1.095481	0.852941757
CBR3	Inverse variance weighted	2	0.027751	0.015055	0.065288	1.028139	0.998244	1.058929	0.852941757
PLG	Inverse variance weighted	2	0.066463	0.038442	0.083827	1.068721	0.991155	1.152358	0.852941757
HSP90B1	Inverse variance weighted	2	-0.02181	0.011935	0.067626	0.978425	0.955803	1.001583	0.852941757
FCGR2B	Inverse variance weighted	3	-0.02291	0.012915	0.076104	0.977353	0.952924	1.002408	0.852941757
IL16	Inverse variance weighted	2	-0.03367	0.018959	0.075735	0.96689	0.93162	1.003495	0.852941757
PDGFD	Inverse variance weighted	2	0.050028	0.028192	0.075974	1.051301	0.994785	1.111028	0.852941757
RETN	Inverse variance weighted	3	0.071105	0.040676	0.080448	1.073694	0.991418	1.162798	0.852941757
SERPINA10	Inverse variance weighted	2	0.02489	0.013807	0.071428	1.025202	0.997831	1.053324	0.852941757
FAS	Wald ratio	1	0.129418	0.076967	0.092669	1.138166	0.978791	1.323492	0.876283058
HP	Inverse variance weighted	6	-0.02269	0.01352	0.093263	0.977563	0.951999	1.003814	0.876283058
GZMB	Inverse variance weighted	3	0.020171	0.012072	0.094733	1.020376	0.996517	1.044807	0.876283058
IL6ST	Inverse variance weighted	3	0.050624	0.029717	0.088469	1.051928	0.992407	1.115018	0.876283058
GSTP1	Wald ratio	1	0.088372	0.063049	0.161023	1.092395	0.965408	1.236084	0.927690438
BPI	Wald ratio	1	-0.12959	0.082249	0.115132	0.878459	0.747671	1.032127	0.927690438
TNFRSF1B	Wald ratio	1	-0.11444	0.078812	0.146486	0.891865	0.76421	1.040844	0.927690438
PTN	Wald ratio	1	-0.0724	0.046448	0.119038	0.930155	0.849215	1.018809	0.927690438
CTSS	Wald ratio	1	-0.03196	0.022429	0.154195	0.968547	0.926892	1.012075	0.927690438
COL15A1	Wald ratio	1	0.068527	0.046779	0.142945	1.070929	0.977107	1.173761	0.927690438
XCL1	Wald ratio	1	-0.04517	0.029336	0.123638	0.955836	0.902426	1.012407	0.927690438
ROR1	Wald ratio	1	-0.04773	0.030227	0.114348	0.953394	0.89855	1.011585	0.927690438
IGFBP7	Wald ratio	1	-0.04716	0.033094	0.154157	0.953935	0.894023	1.017863	0.927690438
ASPN	Wald ratio	1	0.031753	0.022227	0.153127	1.032262	0.988257	1.078226	0.927690438
RSPO3	Wald ratio	1	0.053333	0.036667	0.145795	1.054781	0.981637	1.133375	0.927690438
PCOLCE2	Wald ratio	1	-0.0455	0.031754	0.151905	0.955522	0.897866	1.01688	0.927690438
ICOSLG	Inverse variance weighted	2	-0.04828	0.032741	0.140333	0.952869	0.893642	1.016021	0.927690438
MPO	Inverse variance weighted	4	0.058698	0.041793	0.160169	1.060455	0.977052	1.150978	0.927690438
PYGL	Inverse variance weighted	3	-0.03773	0.027408	0.168671	0.962977	0.912611	1.016121	0.927690438
IL6R	Inverse variance weighted	2	-0.01718	0.01174	0.143469	0.982971	0.960612	1.005852	0.927690438
IL6R	Inverse variance weighted	2	-0.01723	0.011786	0.143663	0.982913	0.960468	1.005883	0.927690438

MMP9	Inverse variance weighted	3	-0.04172	0.030111	0.165889	0.959139	0.904171	1.017448	0.927690438
IDO1	Inverse variance weighted	2	-0.03532	0.023636	0.13508	0.965296	0.921597	1.011067	0.927690438
NQO2	Inverse variance weighted	2	-0.02507	0.017005	0.140397	0.975241	0.943272	1.008293	0.927690438
IL7R	Inverse variance weighted	2	0.045984	0.028966	0.112387	1.047058	0.98927	1.108222	0.927690438
CA6	Inverse variance weighted	2	0.021352	0.014981	0.154086	1.021581	0.992021	1.052023	0.927690438
CRHBP	Inverse variance weighted	2	0.022114	0.015513	0.153994	1.022361	0.991744	1.053923	0.927690438
ACP1	Inverse variance weighted	2	0.020922	0.014596	0.151731	1.021143	0.992344	1.050777	0.927690438
IL12B	Inverse variance weighted	2	-0.03942	0.028559	0.1675	0.961348	0.909014	1.016694	0.927690438
LRPAP1	Inverse variance weighted	4	-0.03698	0.024782	0.135694	0.9637	0.918008	1.011665	0.927690438
GPC5	Inverse variance weighted	2	-0.02009	0.014034	0.152233	0.980108	0.953515	1.007442	0.927690438
NELL1	Inverse variance weighted	3	0.029014	0.018262	0.112103	1.029439	0.993244	1.066953	0.927690438
FCRL3	Inverse variance weighted	2	0.032048	0.023091	0.165168	1.032567	0.986876	1.080374	0.927690438
C1QTNF5	Inverse variance weighted	3	-0.03895	0.027929	0.163103	0.961796	0.910562	1.015913	0.927690438
ATP1B2	Wald ratio	1	0.046165	0.035751	0.196613	1.047247	0.976375	1.123263	0.964113347
PDIA5	Wald ratio	1	0.028183	0.021281	0.185395	1.028583	0.986563	1.072393	0.964113347
LRIG3	Wald ratio	1	0.075476	0.056012	0.177822	1.078397	0.966274	1.203531	0.964113347
NTN4	Wald ratio	1	-0.06369	0.049298	0.196367	0.938294	0.851874	1.033481	0.964113347
LY9	Wald ratio	1	0.066002	0.050228	0.18883	1.068229	0.968076	1.178745	0.964113347
F10	Inverse variance weighted	5	0.031122	0.02395	0.193797	1.031611	0.984304	1.081192	0.964113347
THBS2	Inverse variance weighted	2	0.026455	0.020242	0.191238	1.026808	0.986867	1.068365	0.964113347
ISLR2	Inverse variance weighted	3	0.055944	0.042297	0.185954	1.057538	0.973402	1.148947	0.964113347
CRISPLD2	Inverse variance weighted	3	0.045001	0.034354	0.19023	1.046028	0.977914	1.118887	0.964113347
RPN1	Wald ratio	1	0.037424	0.029301	0.201522	1.038133	0.980193	1.099498	0.967034812
EMILIN3	Wald ratio	1	-0.05193	0.0407	0.201961	0.949393	0.876599	1.028231	0.967034812
LILRA5	Wald ratio	1	-0.01084	0.082656	0.895659	0.989218	0.841268	1.163189	0.977376677
AGRP	Wald ratio	1	-0.00489	0.053382	0.926989	0.99512	0.896265	1.10488	0.977376677
FLRT2	Wald ratio	1	-0.04717	0.047174	0.317311	0.953922	0.869676	1.046328	0.977376677
TNFSF12	Wald ratio	1	-0.01704	0.028223	0.546107	0.983109	0.930204	1.039023	0.977376677
GNRH2	Wald ratio	1	0.036205	0.056596	0.522364	1.036868	0.928	1.158508	0.977376677
HPGDS	Wald ratio	1	0.015469	0.023203	0.504985	1.015589	0.970437	1.062842	0.977376677
MATN4	Wald ratio	1	0.023499	0.050479	0.641561	1.023777	0.927336	1.130248	0.977376677
EGF	Wald ratio	1	-0.04333	0.03994	0.277963	0.957595	0.885491	1.035569	0.977376677
CGA	Wald ratio	1	-0.01331	0.027059	0.622858	0.98678	0.93581	1.040528	0.977376677
LHB	Wald ratio	1	-0.02027	0.042146	0.630606	0.979937	0.90224	1.064324	0.977376677
APOE	Wald ratio	1	0.032362	0.089401	0.717359	1.032892	0.866872	1.230707	0.977376677
CAT	Wald ratio	1	-0.0396	0.053854	0.46216	0.961175	0.86489	1.068179	0.977376677
SERPING1	Wald ratio	1	0.001495	0.018774	0.936519	1.001496	0.965314	1.039035	0.977376677
FABP1	Wald ratio	1	0.055118	0.056168	0.326441	1.056665	0.946512	1.179638	0.977376677
CTSB	Wald ratio	1	-0.02708	0.02732	0.3215	0.973279	0.922534	1.026816	0.977376677
RET	Wald ratio	1	-0.0065	0.047688	0.891534	0.993518	0.904864	1.090859	0.977376677
CD55	Wald ratio	1	-0.02245	0.022069	0.308967	0.977798	0.936405	1.02102	0.977376677
SOD3	Wald ratio	1	-0.01576	0.057193	0.782924	0.984366	0.879981	1.101134	0.977376677
SERPINF2	Wald ratio	1	-0.0159	0.065048	0.806887	0.984225	0.866413	1.118058	0.977376677
CTSH	Wald ratio	1	0.01645	0.043723	0.70674	1.016586	0.933096	1.107546	0.977376677
PATE4	Wald ratio	1	0.011413	0.022411	0.610571	1.011479	0.96801	1.056899	0.977376677
GZMB	Wald ratio	1	-0.02078	0.017793	0.24278	0.979431	0.945863	1.014191	0.977376677
CCL3	Wald ratio	1	-0.02116	0.068367	0.756923	0.979061	0.856278	1.11945	0.977376677
RNASE2	Wald ratio	1	0.010989	0.042688	0.79685	1.01105	0.929899	1.099282	0.977376677
PF4V1	Wald ratio	1	-0.00446	0.022483	0.84289	0.995554	0.952635	1.040406	0.977376677
MBL2	Wald ratio	1	0.018795	0.076627	0.806237	1.018973	0.876873	1.1841	0.977376677
COL6A1	Wald ratio	1	-0.00425	0.05196	0.934791	0.995758	0.899341	1.102512	0.977376677
HGF	Wald ratio	1	0.034501	0.064367	0.591959	1.035103	0.912417	1.174285	0.977376677
CPM	Wald ratio	1	0.019365	0.062399	0.756299	1.019554	0.902184	1.152194	0.977376677
NQO1	Wald ratio	1	0.014137	0.017071	0.4076	1.014238	0.980863	1.048748	0.977376677
VEGFA	Wald ratio	1	0.007383	0.024291	0.761188	1.00741	0.96057	1.056534	0.977376677
IFNAR1	Wald ratio	1	-0.02485	0.051433	0.628938	0.975453	0.881914	1.078913	0.977376677
CAPN2	Wald ratio	1	-0.003	0.074139	0.967771	0.997009	0.862166	1.152942	0.977376677
IGFBP3	Wald ratio	1	0.025075	0.047536	0.59785	1.025392	0.934171	1.125521	0.977376677
PI3	Wald ratio	1	0.015708	0.032748	0.631459	1.015832	0.952679	1.083172	0.977376677
EPHA1	Wald ratio	1	-0.00727	0.021823	0.738883	0.992752	0.951184	1.036137	0.977376677
FAS	Wald ratio	1	-0.00533	0.034461	0.87718	0.994688	0.929722	1.064194	0.977376677
CNTFR	Wald ratio	1	0.050172	0.053602	0.349273	1.051451	0.946591	1.167928	0.977376677
CSF2RB	Wald ratio	1	0.006016	0.031982	0.850784	1.006035	0.944907	1.071117	0.977376677
RNASE4	Wald ratio	1	-0.0109	0.027147	0.687949	0.989156	0.9379	1.043213	0.977376677

SERPINF1	Wald ratio	1	0.011438	0.02521	0.650042	1.011504	0.962738	1.062739	0.977376677
SERPINF1	Wald ratio	1	0.011014	0.024275	0.650042	1.011075	0.964095	1.060344	0.977376677
SNCA	Wald ratio	1	0.058636	0.048265	0.224413	1.060389	0.964676	1.165599	0.977376677
COL18A1	Wald ratio	1	-0.00196	0.047414	0.967039	0.998043	0.909473	1.095238	0.977376677
ASIP	Wald ratio	1	0.01453	0.019765	0.462257	1.014636	0.976081	1.054714	0.977376677
CCN3	Wald ratio	1	-0.00432	0.057074	0.939713	0.995693	0.890313	1.113546	0.977376677
SPINT3	Wald ratio	1	-0.01455	0.018229	0.424882	0.985559	0.950968	1.021408	0.977376677
ENTPD1	Wald ratio	1	-0.0318	0.052827	0.54717	0.968698	0.873416	1.074374	0.977376677
GZMM	Wald ratio	1	-0.03718	0.063212	0.556374	0.963499	0.851225	1.090582	0.977376677
CCNH	Wald ratio	1	-0.02808	0.050712	0.579771	0.97231	0.880314	1.073921	0.977376677
LYZ	Wald ratio	1	0.006547	0.020908	0.754181	1.006568	0.966153	1.048674	0.977376677
CCL8	Wald ratio	1	0.034647	0.060199	0.564926	1.035254	0.920035	1.164903	0.977376677
CCL7	Wald ratio	1	0.01361	0.023647	0.564926	1.013703	0.967791	1.061793	0.977376677
MFGE8	Wald ratio	1	-0.00266	0.047492	0.955282	0.99734	0.908693	1.094636	0.977376677
IL15RA	Wald ratio	1	-0.00776	0.03705	0.834039	0.992267	0.922765	1.067005	0.977376677
IL18R1	Wald ratio	1	-0.00444	0.01822	0.807306	0.995566	0.960641	1.03176	0.977376677
APOF	Wald ratio	1	-0.04212	0.052106	0.418924	0.958758	0.865676	1.061848	0.977376677
EBI3	Wald ratio	1	0.004975	0.064124	0.938157	1.004988	0.886293	1.139578	0.977376677
IL11RA	Wald ratio	1	0.022705	0.040474	0.574814	1.022965	0.944949	1.107421	0.977376677
GPNMB	Wald ratio	1	0.043501	0.05334	0.414767	1.044461	0.94078	1.159568	0.977376677
POSTN	Wald ratio	1	0.048471	0.060589	0.423711	1.049665	0.93213	1.18202	0.977376677
PCOLCE	Wald ratio	1	0.024136	0.049692	0.62717	1.02443	0.929358	1.129227	0.977376677
CST6	Wald ratio	1	0.055556	0.056429	0.32486	1.057128	0.946442	1.180758	0.977376677
PCSK7	Wald ratio	1	0.032612	0.040508	0.420771	1.03315	0.954294	1.118522	0.977376677
CPZ	Wald ratio	1	0.006157	0.051308	0.904483	1.006176	0.909912	1.112624	0.977376677
MYORG	Wald ratio	1	-0.02661	0.033095	0.421442	0.973745	0.912586	1.039002	0.977376677
APOA5	Wald ratio	1	0.068952	0.057905	0.233736	1.071385	0.956436	1.200149	0.977376677
QSOX2	Wald ratio	1	-0.02827	0.037049	0.445369	0.972122	0.904032	1.04534	0.977376677
CHST9	Wald ratio	1	0.010808	0.031822	0.734141	1.010866	0.949743	1.075923	0.977376677
LILRB2	Wald ratio	1	0.031671	0.083558	0.704664	1.032178	0.876251	1.215852	0.977376677
SFRP1	Wald ratio	1	-0.01108	0.055409	0.841481	0.988979	0.887201	1.102433	0.977376677
CD200R1	Wald ratio	1	-0.0398	0.057987	0.49254	0.960986	0.857743	1.076656	0.977376677
BPIFB1	Wald ratio	1	0.005726	0.023362	0.806381	1.005742	0.960728	1.052866	0.977376677
WFIKKN2	Wald ratio	1	0.009796	0.016531	0.553454	1.009845	0.977648	1.043101	0.977376677
WFIKKN2	Wald ratio	1	0.001839	0.017884	0.918119	1.00184	0.967331	1.03758	0.977376677
DEFB104A	Wald ratio	1	0.037128	0.089018	0.676616	1.037826	0.871667	1.235658	0.977376677
SPOCK2	Wald ratio	1	-0.00872	0.04681	0.852229	0.991318	0.904414	1.086573	0.977376677
RELT	Wald ratio	1	0.006545	0.029818	0.826251	1.006567	0.949426	1.067147	0.977376677
FCRL4	Wald ratio	1	0.00971	0.012534	0.438548	1.009757	0.985252	1.034871	0.977376677
GPX7	Wald ratio	1	0.029292	0.024321	0.228443	1.029725	0.98179	1.08	0.977376677
SEMA3C	Wald ratio	1	0.036551	0.058824	0.534363	1.037227	0.924277	1.163979	0.977376677
THSD1	Wald ratio	1	0.014682	0.036538	0.687822	1.01479	0.944657	1.09013	0.977376677
DKK3	Wald ratio	1	0.010702	0.03478	0.758316	1.010759	0.944153	1.082064	0.977376677
CTSZ	Wald ratio	1	-0.02456	0.030077	0.414206	0.975741	0.919882	1.034991	0.977376677
KLK11	Wald ratio	1	0.005176	0.016529	0.754181	1.005189	0.973146	1.038287	0.977376677
CNTNAP2	Wald ratio	1	-0.00139	0.025854	0.957277	0.998616	0.949273	1.050524	0.977376677
LGALS9	Inverse variance weighted	2	0.005585	0.043912	0.898792	1.005601	0.922672	1.095983	0.977376677
TLR4	Inverse variance weighted	3	-0.02091	0.021319	0.326638	0.979305	0.939228	1.021093	0.977376677
SIRPB1	Inverse variance weighted	4	-0.00072	0.015135	0.962024	0.99928	0.970073	1.029366	0.977376677
MANBA	Inverse variance weighted	3	0.023143	0.028665	0.419452	1.023413	0.9675	1.082558	0.977376677
CHL1	Inverse variance weighted	3	-0.05031	0.068608	0.463367	0.950934	0.831286	1.087803	0.977376677
FCN1	Inverse variance weighted	2	-0.00658	0.018541	0.722793	0.993445	0.957991	1.03021	0.977376677
CCL22	Inverse variance weighted	4	0.013424	0.048608	0.782412	1.013515	0.921413	1.114823	0.977376677
CXCL11	Inverse variance weighted	2	0.041119	0.042075	0.328425	1.041977	0.959496	1.131548	0.977376677
NRP1	Inverse variance weighted	2	-0.03014	0.030349	0.320594	0.970306	0.914271	1.029775	0.977376677
APOL1	Inverse variance weighted	4	0.002428	0.017978	0.892581	1.002431	0.967724	1.038383	0.977376677
NCR3	Inverse variance weighted	2	-0.02545	0.030951	0.410899	0.97487	0.917488	1.03584	0.977376677
SEMA3E	Inverse variance weighted	2	0.009921	0.012376	0.422752	1.00997	0.985767	1.034768	0.977376677
CCL25	Inverse variance weighted	3	-0.03492	0.051382	0.496719	0.96568	0.873165	1.067998	0.977376677
CCL25	Inverse variance weighted	4	-0.01587	0.032852	0.629144	0.98426	0.922882	1.049721	0.977376677
CCL16	Inverse variance weighted	2	0.005221	0.01258	0.678102	1.005235	0.980752	1.03033	0.977376677
SPINT2	Inverse variance weighted	2	0.006791	0.013376	0.611692	1.006814	0.98076	1.033559	0.977376677
COCH	Inverse variance weighted	3	-0.01305	0.065557	0.842191	0.987033	0.868016	1.122368	0.977376677
KLK8	Inverse variance weighted	2	-0.03956	0.040825	0.332525	0.961211	0.887295	1.041285	0.977376677

KLK8	Inverse variance weighted	2	-0.07718	0.097068	0.426528	0.92572	0.76534	1.11971	0.977376677
PLXNC1	Inverse variance weighted	5	-0.00399	0.022428	0.858901	0.996021	0.953184	1.040782	0.977376677
FCGR3B	Inverse variance weighted	3	0.046029	0.118608	0.697958	1.047105	0.829906	1.321149	0.977376677
ADAM23	Inverse variance weighted	4	-0.01059	0.027659	0.701912	0.98947	0.937256	1.044591	0.977376677
PGLYRP1	Inverse variance weighted	2	-0.01599	0.06161	0.795232	0.984138	0.872192	1.110452	0.977376677
CREG1	Inverse variance weighted	3	-0.0252	0.02386	0.290989	0.975119	0.930567	1.021805	0.977376677
CST7	Inverse variance weighted	4	0.005703	0.022517	0.800043	1.00572	0.962299	1.051099	0.977376677
DKK1	Inverse variance weighted	3	0.021969	0.04154	0.596901	1.022212	0.942283	1.108922	0.977376677
CCN4	Inverse variance weighted	2	-0.0142	0.016753	0.396672	0.985901	0.954054	1.018811	0.977376677
APOM	Inverse variance weighted	2	0.009382	0.042897	0.826878	1.009426	0.928026	1.097967	0.977376677
H6PD	Inverse variance weighted	5	-0.00953	0.013476	0.479562	0.990517	0.964696	1.017029	0.977376677
ANGPTL1	Inverse variance weighted	2	0.027318	0.030857	0.375989	1.027695	0.967382	1.091768	0.977376677
F10	Inverse variance weighted	3	0.006829	0.025896	0.792013	1.006852	0.957024	1.059275	0.977376677
CFB	Inverse variance weighted	4	0.017526	0.024635	0.476829	1.01768	0.969709	1.068025	0.977376677
PENK	Inverse variance weighted	3	0.012712	0.019494	0.51433	1.012794	0.974826	1.05224	0.977376677
CGA	Inverse variance weighted	3	0.007311	0.015995	0.647607	1.007338	0.976248	1.039417	0.977376677
HLA-DQA2	Inverse variance weighted	3	0.009367	0.02672	0.72591	1.009411	0.957908	1.063684	0.977376677
CRP	Inverse variance weighted	4	-0.00244	0.032099	0.939328	0.99756	0.936733	1.062336	0.977376677
C1QC	Inverse variance weighted	4	-0.01023	0.033435	0.759659	0.989823	0.927036	1.056863	0.977376677
AHSG	Inverse variance weighted	2	-0.00617	0.017319	0.721696	0.99385	0.960681	1.028165	0.977376677
LTF	Inverse variance weighted	2	0.039364	0.03263	0.227673	1.040149	0.975709	1.108844	0.977376677
LTF	Inverse variance weighted	2	0.032513	0.060624	0.591751	1.033047	0.917309	1.163387	0.977376677
CLPS	Inverse variance weighted	3	-0.00125	0.023467	0.957545	0.998752	0.953854	1.045762	0.977376677
TFF1	Inverse variance weighted	4	-0.00426	0.034657	0.902279	0.995754	0.93036	1.065744	0.977376677
ISG15	Inverse variance weighted	4	0.002805	0.064963	0.96556	1.002809	0.882919	1.138978	0.977376677
ICAM1	Inverse variance weighted	3	-0.00315	0.010748	0.769672	0.996858	0.976077	1.018081	0.977376677
FCER2	Inverse variance weighted	3	-0.0048	0.025939	0.853128	0.99521	0.945878	1.047114	0.977376677
SERPINE2	Inverse variance weighted	2	-0.0293	0.072607	0.686587	0.971129	0.842311	1.119647	0.977376677
GP1BA	Inverse variance weighted	2	0.180953	0.250134	0.469419	1.198358	0.733954	1.956612	0.977376677
CA3	Inverse variance weighted	2	-0.03206	0.046016	0.485969	0.968448	0.884925	1.059853	0.977376677
PRSS2	Inverse variance weighted	3	0.005372	0.0358	0.880715	1.005387	0.937259	1.078466	0.977376677
RNASE1	Inverse variance weighted	2	-0.03748	0.040068	0.349612	0.963216	0.890466	1.04191	0.977376677
MSMB	Inverse variance weighted	4	0.008698	0.014233	0.541151	1.008736	0.980983	1.037273	0.977376677
GSTA1	Inverse variance weighted	2	0.001897	0.027604	0.945213	1.001899	0.949132	1.057599	0.977376677
CFH	Inverse variance weighted	2	-0.0235	0.024574	0.338877	0.976772	0.930842	1.024969	0.977376677
CD48	Inverse variance weighted	2	0.00294	0.051073	0.954098	1.002944	0.907409	1.108538	0.977376677
CXCL1	Inverse variance weighted	2	-0.01233	0.015334	0.421395	0.987747	0.958501	1.017885	0.977376677
LCT	Inverse variance weighted	2	0.005856	0.018983	0.75772	1.005873	0.969135	1.044004	0.977376677
SAA1	Inverse variance weighted	2	-0.01109	0.013262	0.403091	0.988973	0.963598	1.015016	0.977376677
AMY1A	Inverse variance weighted	4	-0.01812	0.026983	0.501978	0.982047	0.931458	1.035383	0.977376677
IGF2R	Inverse variance weighted	7	0.014958	0.02058	0.467334	1.015071	0.97494	1.056853	0.977376677
PTHLH	Inverse variance weighted	4	-0.04768	0.039875	0.231781	0.953438	0.88176	1.030942	0.977376677
FCGR2A	Inverse variance weighted	3	-0.00783	0.0182	0.667072	0.992201	0.957431	1.028234	0.977376677
NCAM1	Inverse variance weighted	3	-0.02701	0.042282	0.523004	0.973355	0.895941	1.057457	0.977376677
COL11A2	Inverse variance weighted	2	0.019887	0.042236	0.637745	1.020086	0.939041	1.108125	0.977376677
CD59	Inverse variance weighted	2	-0.02012	0.02907	0.488799	0.980078	0.925796	1.037543	0.977376677
AKR1A1	Inverse variance weighted	3	0.002575	0.02137	0.904102	1.002578	0.961452	1.045463	0.977376677
PLA2G2A	Inverse variance weighted	2	-0.01184	0.012145	0.329754	0.988234	0.964988	1.012039	0.977376677
CPB1	Inverse variance weighted	2	-0.01883	0.034004	0.579795	0.981349	0.918076	1.048982	0.977376677
AKR1B1	Inverse variance weighted	2	-0.02914	0.048721	0.549737	0.971278	0.882818	1.068601	0.977376677
B4GALT1	Inverse variance weighted	4	-0.00746	0.03663	0.838654	0.992569	0.923805	1.066451	0.977376677
VEGFA	Inverse variance weighted	3	0.014556	0.013998	0.298401	1.014663	0.987202	1.042887	0.977376677
IGLL1	Inverse variance weighted	7	-0.02831	0.026972	0.293851	0.972085	0.92203	1.024856	0.977376677
SELP	Inverse variance weighted	5	0.006544	0.018873	0.728771	1.006566	0.970012	1.044498	0.977376677
CBR1	Inverse variance weighted	3	-0.00148	0.030185	0.960958	0.998523	0.941161	1.059382	0.977376677
CRISP2	Inverse variance weighted	2	-0.00809	0.016463	0.622965	0.991939	0.960443	1.024468	0.977376677
CCL3L1	Inverse variance weighted	2	-0.02674	0.02807	0.340775	0.973614	0.921495	1.02868	0.977376677
NPPB	Inverse variance weighted	2	-0.02731	0.032999	0.407935	0.973062	0.912117	1.038078	0.977376677
CTRB1	Inverse variance weighted	2	-0.00232	0.025535	0.927747	0.997687	0.948983	1.048891	0.977376677
IL1RN	Inverse variance weighted	2	0.008357	0.044481	0.850974	1.008392	0.9242	1.100253	0.977376677
PAM	Inverse variance weighted	6	-0.02069	0.025868	0.423773	0.979521	0.931096	1.030465	0.977376677
UGT1A6	Inverse variance weighted	2	-0.00911	0.033409	0.78519	0.990935	0.928125	1.057996	0.977376677
ITIH1	Inverse variance weighted	3	0.003255	0.013745	0.812822	1.00326	0.976593	1.030655	0.977376677
TCN1	Inverse variance weighted	2	0.053964	0.046782	0.248696	1.055447	0.962974	1.156799	0.977376677

TCN2	Inverse variance weighted	4	-0.01071	0.023971	0.654931	0.989344	0.943937	1.036935	0.977376677
CD33	Inverse variance weighted	2	0.013333	0.011306	0.238305	1.013422	0.991211	1.036131	0.977376677
SPINK2	Inverse variance weighted	3	0.004815	0.029875	0.871952	1.004827	0.947679	1.065421	0.977376677
TPSB2	Inverse variance weighted	2	-0.00475	0.012452	0.703156	0.995266	0.971269	1.019856	0.977376677
FGF7	Inverse variance weighted	2	-0.01061	0.0749	0.887361	0.989447	0.854351	1.145905	0.977376677
LPO	Inverse variance weighted	2	0.005451	0.035997	0.879637	1.005466	0.936971	1.078968	0.977376677
TNXB	Inverse variance weighted	3	-0.00166	0.021121	0.937283	0.998339	0.957854	1.040536	0.977376677
GNLY	Inverse variance weighted	4	0.005529	0.014719	0.707208	1.005544	0.976949	1.034976	0.977376677
CBLN1	Inverse variance weighted	2	-0.01538	0.017232	0.372089	0.984737	0.952033	1.018564	0.977376677
PRTN3	Inverse variance weighted	3	0.020048	0.026317	0.4462	1.02025	0.968958	1.074257	0.977376677
PRTN3	Inverse variance weighted	2	0.014184	0.028311	0.616359	1.014286	0.959536	1.072159	0.977376677
TNC	Inverse variance weighted	4	0.019883	0.025571	0.436825	1.020082	0.970217	1.07251	0.977376677
IL1R2	Inverse variance weighted	2	0.016507	0.0239	0.489785	1.016644	0.970118	1.065401	0.977376677
GRN	Inverse variance weighted	4	0.013937	0.013061	0.285931	1.014035	0.988405	1.040329	0.977376677
PCSK1	Inverse variance weighted	3	-0.0096	0.017381	0.580745	0.990446	0.957272	1.02477	0.977376677
SERPINA4	Inverse variance weighted	2	0.005133	0.0233	0.825644	1.005146	0.960276	1.052112	0.977376677
SERPINA4	Inverse variance weighted	2	0.004183	0.018028	0.816534	1.004191	0.969328	1.040309	0.977376677
ALDH3A1	Inverse variance weighted	2	0.023535	0.037445	0.529662	1.023814	0.951365	1.101781	0.977376677
GPC1	Inverse variance weighted	2	0.001907	0.033669	0.954827	1.001909	0.937925	1.070258	0.977376677
TIE1	Inverse variance weighted	3	-0.01389	0.014546	0.339686	0.986207	0.958487	1.01473	0.977376677
FLT4	Inverse variance weighted	2	0.002216	0.014253	0.876435	1.002219	0.974609	1.030611	0.977376677
KDR	Inverse variance weighted	3	0.004339	0.023874	0.855785	1.004348	0.958435	1.052461	0.977376677
CHI3L1	Inverse variance weighted	2	-0.01045	0.009954	0.293551	0.9896	0.970481	1.009095	0.977376677
PRCP	Inverse variance weighted	3	-0.01083	0.031366	0.729862	0.989227	0.930244	1.051951	0.977376677
LEPR	Inverse variance weighted	3	-0.00156	0.014209	0.912548	0.998441	0.971019	1.026637	0.977376677
KLK7	Inverse variance weighted	2	-0.01006	0.040098	0.801992	0.989995	0.915169	1.07094	0.977376677
GZMK	Inverse variance weighted	2	0.046353	0.046475	0.31858	1.047444	0.956248	1.147337	0.977376677
BCAM	Inverse variance weighted	2	-0.01647	0.041102	0.688648	0.983666	0.907531	1.066188	0.977376677
PPT1	Inverse variance weighted	4	-0.01186	0.023433	0.612661	0.988207	0.943847	1.034651	0.977376677
MFAP2	Inverse variance weighted	2	0.049053	0.042207	0.245154	1.050276	0.966888	1.140857	0.977376677
CCL23	Inverse variance weighted	3	-0.0161	0.02034	0.428642	0.984029	0.94557	1.024052	0.977376677
FAM3B	Inverse variance weighted	2	0.020562	0.036948	0.577863	1.020774	0.949466	1.097439	0.977376677
FAM3B	Inverse variance weighted	3	0.018259	0.028765	0.525576	1.018427	0.962597	1.077495	0.977376677
LILRA4	Inverse variance weighted	3	0.044037	0.041332	0.286669	1.045021	0.963702	1.133203	0.977376677
DEFB1	Inverse variance weighted	3	-0.02208	0.041045	0.590578	0.97816	0.902551	1.060103	0.977376677
SIRPA	Inverse variance weighted	4	-0.01674	0.014177	0.237735	0.983401	0.956451	1.01111	0.977376677
GSTO1	Inverse variance weighted	3	-0.01929	0.023189	0.405507	0.980895	0.937311	1.026507	0.977376677
CX3CL1	Inverse variance weighted	2	-0.02101	0.055807	0.706536	0.979207	0.877751	1.092391	0.977376677
TNFAIP6	Inverse variance weighted	3	-0.00278	0.022801	0.902793	0.997219	0.953635	1.042796	0.977376677
IL5RA	Inverse variance weighted	2	0.012513	0.045556	0.783565	1.012592	0.926097	1.107164	0.977376677
IL5RA	Inverse variance weighted	3	0.02757	0.042748	0.518962	1.027954	0.945335	1.117792	0.977376677
IL1RL1	Inverse variance weighted	4	-0.0126	0.011118	0.257192	0.987481	0.966195	1.009237	0.977376677
NAAA	Inverse variance weighted	2	-0.01104	0.017423	0.526486	0.989025	0.955821	1.023383	0.977376677
CNTN2	Inverse variance weighted	4	-0.00797	0.021959	0.716708	0.992064	0.950272	1.035693	0.977376677
TEK	Inverse variance weighted	3	-0.01921	0.030277	0.525692	0.98097	0.92445	1.040945	0.977376677
AKR1C1	Inverse variance weighted	2	-0.00292	0.062859	0.9629	0.99708	0.881503	1.127811	0.977376677
DPT	Inverse variance weighted	2	-0.00792	0.022081	0.719983	0.992115	0.950093	1.035997	0.977376677
BST1	Inverse variance weighted	2	0.02102	0.024682	0.394424	1.021242	0.973014	1.07186	0.977376677
ST3GAL1	Inverse variance weighted	2	0.004637	0.034014	0.891567	1.004648	0.939855	1.073907	0.977376677
FSTL1	Inverse variance weighted	2	-0.01515	0.094225	0.872256	0.984963	0.818868	1.184748	0.977376677
PLA2R1	Inverse variance weighted	2	-0.00175	0.027548	0.949307	0.99825	0.945781	1.053631	0.977376677
CHIT1	Inverse variance weighted	3	0.011892	0.013919	0.39288	1.011963	0.984729	1.039951	0.977376677
NOG	Inverse variance weighted	3	0.012813	0.034915	0.713637	1.012895	0.945899	1.084637	0.977376677
IL15RA	Inverse variance weighted	2	-0.00821	0.023864	0.730679	0.991819	0.946496	1.039312	0.977376677
IL18R1	Inverse variance weighted	2	-0.01042	0.012512	0.405131	0.989638	0.965663	1.014208	0.977376677
ALCAM	Inverse variance weighted	2	0.023725	0.063902	0.710432	1.024009	0.903461	1.160641	0.977376677
LAMC2	Inverse variance weighted	2	0.009691	0.01792	0.588639	1.009738	0.974889	1.045833	0.977376677
NID2	Inverse variance weighted	2	0.007072	0.024352	0.771495	1.007097	0.960157	1.056332	0.977376677
SPARCL1	Inverse variance weighted	2	-0.01231	0.046789	0.792464	0.987765	0.901209	1.082633	0.977376677
GPNMB	Inverse variance weighted	2	0.020979	0.039489	0.595234	1.021201	0.945143	1.103378	0.977376677
GPNMB	Inverse variance weighted	2	0.022343	0.04206	0.595269	1.022594	0.941675	1.110467	0.977376677
GPNMB	Inverse variance weighted	3	-0.03161	0.05154	0.539615	0.96888	0.875787	1.071869	0.977376677
AGER	Inverse variance weighted	6	-0.01433	0.033629	0.670037	0.985773	0.922893	1.052937	0.977376677
FCN2	Inverse variance weighted	2	0.001274	0.019738	0.948538	1.001275	0.963279	1.040769	0.977376677

TGFBI	Inverse variance weighted	2	0.008951	0.02831	0.751874	1.008991	0.954529	1.06656	0.977376677
CST6	Inverse variance weighted	2	0.054111	0.043834	0.217038	1.055601	0.968696	1.150303	0.977376677
CCL14	Inverse variance weighted	3	-0.00701	0.018428	0.703633	0.993014	0.957788	1.029536	0.977376677
MIA	Inverse variance weighted	2	-0.00246	0.021387	0.908543	0.997546	0.956595	1.040251	0.977376677
VWC2	Inverse variance weighted	3	-0.02961	0.036077	0.411847	0.970827	0.904549	1.041962	0.977376677
CDNF	Inverse variance weighted	2	-0.00804	0.044679	0.857275	0.991997	0.908821	1.082785	0.977376677
LIPN	Inverse variance weighted	3	0.000789	0.012524	0.949762	1.000789	0.976523	1.025659	0.977376677
FCRL6	Inverse variance weighted	2	-0.01307	0.019266	0.497396	0.987011	0.950434	1.024995	0.977376677
ERAP2	Inverse variance weighted	2	0.020566	0.040019	0.607308	1.020779	0.943772	1.10407	0.977376677
LILRA6	Inverse variance weighted	2	-0.00736	0.022286	0.741304	0.99267	0.950244	1.03699	0.977376677
SPINK6	Inverse variance weighted	2	-0.00444	0.023159	0.847971	0.99557	0.95139	1.041802	0.977376677
ENPP7	Inverse variance weighted	2	-0.0167	0.015477	0.280473	0.983435	0.954051	1.013725	0.977376677
VIT	Inverse variance weighted	2	0.001775	0.025001	0.943406	1.001776	0.95387	1.052089	0.977376677
LRRN1	Inverse variance weighted	2	0.005377	0.014218	0.705329	1.005391	0.977759	1.033804	0.977376677
CHRDL2	Inverse variance weighted	2	0.014767	0.036343	0.684498	1.014877	0.9451	1.089805	0.977376677
CD109	Inverse variance weighted	2	0.003068	0.023619	0.896643	1.003073	0.957696	1.0506	0.977376677
ADAMTS13	Inverse variance weighted	3	-0.0066	0.018471	0.720709	0.993418	0.958098	1.030041	0.977376677
ITIH5	Inverse variance weighted	3	0.017627	0.026903	0.512322	1.017784	0.965507	1.07289	0.977376677
SERPINA12	Inverse variance weighted	3	0.009276	0.029733	0.755061	1.009319	0.95218	1.069887	0.977376677
CNTN4	Inverse variance weighted	3	0.031403	0.030695	0.306276	1.031901	0.971651	1.095888	0.977376677
ADGRF5	Inverse variance weighted	4	-0.01423	0.014062	0.311511	0.985869	0.959068	1.013419	0.977376677
CACNA2D3	Inverse variance weighted	2	-0.04861	0.044133	0.270741	0.952556	0.873623	1.038621	0.977376677
CD177	Inverse variance weighted	5	-0.00249	0.018371	0.892179	0.997513	0.962234	1.034085	0.977376677
NPW	Inverse variance weighted	3	-0.02661	0.047335	0.574014	0.973741	0.887465	1.068406	0.977376677
LILRB1	Inverse variance weighted	3	0.005476	0.013114	0.676286	1.005491	0.979975	1.03167	0.977376677
UCMA	Inverse variance weighted	2	0.017087	0.027244	0.530537	1.017234	0.96434	1.073028	0.977376677
SECTM1	Inverse variance weighted	2	0.009869	0.069038	0.886324	1.009918	0.882105	1.156252	0.977376677
SMPDL3A	Inverse variance weighted	2	-0.01223	0.012131	0.313232	0.987841	0.964629	1.01161	0.977376677
CCL17	Inverse variance weighted	6	0.010829	0.048076	0.821795	1.010887	0.919982	1.110775	0.977376677
FRZB	Inverse variance weighted	2	0.012377	0.021575	0.566194	1.012454	0.970533	1.056185	0.977376677
GGH	Inverse variance weighted	3	-0.00604	0.021286	0.77671	0.993981	0.953366	1.036327	0.977376677
ART4	Inverse variance weighted	3	-0.01411	0.017238	0.413117	0.985991	0.953234	1.019874	0.977376677
RNASE6	Inverse variance weighted	3	0.011305	0.011335	0.31859	1.011369	0.989148	1.034089	0.977376677
RELT	Inverse variance weighted	2	-0.06641	0.105442	0.528788	0.935744	0.761033	1.150564	0.977376677
ESAM	Inverse variance weighted	3	-0.04939	0.061999	0.42564	0.951807	0.842897	1.07479	0.977376677
SLAMF6	Inverse variance weighted	2	-0.06796	0.084197	0.419607	0.934302	0.792169	1.101937	0.977376677
IL17RA	Inverse variance weighted	3	0.01175	0.012982	0.365394	1.01182	0.986399	1.037895	0.977376677
FAM20A	Inverse variance weighted	4	-0.05472	0.054619	0.316385	0.946746	0.85063	1.053723	0.977376677
SIGLEC12	Inverse variance weighted	2	-0.00485	0.012581	0.699688	0.995159	0.97092	1.020003	0.977376677
CPXM1	Inverse variance weighted	3	0.018005	0.0295	0.541642	1.018168	0.960967	1.078773	0.977376677
IL12RB2	Inverse variance weighted	2	-0.0327	0.035453	0.356324	0.967827	0.902858	1.037472	0.977376677
TIMP4	Inverse variance weighted	2	-0.0078	0.029065	0.788432	0.992231	0.937286	1.050397	0.977376677
GDF15	Inverse variance weighted	2	-0.01585	0.019988	0.427657	0.98427	0.946455	1.023596	0.977376677
SPOCK3	Inverse variance weighted	4	0.012824	0.025575	0.616057	1.012907	0.963385	1.064975	0.977376677
PDCD1LG2	Inverse variance weighted	3	-0.00883	0.023322	0.704995	0.99121	0.946921	1.03757	0.977376677
TAPBPL	Inverse variance weighted	3	-0.00183	0.008606	0.831175	0.998167	0.98147	1.015147	0.977376677
CFHR5	Inverse variance weighted	3	0.006798	0.022059	0.757965	1.006821	0.964218	1.051306	0.977376677
CFHR5	Inverse variance weighted	2	0.020272	0.027483	0.460755	1.020478	0.966963	1.076956	0.977376677
REG4	Inverse variance weighted	2	0.003645	0.053052	0.945228	1.003651	0.904532	1.113632	0.977376677
RTN4R	Inverse variance weighted	3	0.017067	0.026304	0.51645	1.017213	0.966098	1.071033	0.977376677
PRSS22	Inverse variance weighted	2	-0.04645	0.050029	0.353192	0.954615	0.865451	1.052964	0.977376677
CXCL16	Inverse variance weighted	5	0.02331	0.041888	0.577871	1.023584	0.942905	1.111166	0.977376677
VSIR	Inverse variance weighted	2	-0.01053	0.106532	0.921261	0.989525	0.803054	1.219295	0.977376677
IL1RL2	Inverse variance weighted	2	0.012908	0.063794	0.839652	1.012992	0.89393	1.147911	0.977376677
TREM1	Inverse variance weighted	2	0.013182	0.020636	0.522971	1.013269	0.973103	1.055093	0.977376677
CHST11	Inverse variance weighted	3	0.036314	0.032247	0.260111	1.036981	0.973469	1.104637	0.977376677
IL1RAP	Inverse variance weighted	2	-0.01481	0.017628	0.40091	0.985301	0.951839	1.01994	0.977376677
IL1RAP	Inverse variance weighted	2	-0.01412	0.016525	0.392941	0.985982	0.95456	1.018439	0.977376677
OBP2B	Inverse variance weighted	3	-0.00726	0.014591	0.618628	0.992763	0.964775	1.021564	0.977376677
SLAMF7	Inverse variance weighted	2	0.009428	0.015289	0.537481	1.009472	0.97967	1.040181	0.977376677
IL17RB	Inverse variance weighted	2	-0.00554	0.054442	0.918904	0.994472	0.893821	1.106457	0.977376677
CA10	Inverse variance weighted	3	0.008973	0.051818	0.862522	1.009013	0.911568	1.116876	0.977376677
SEMA3G	Inverse variance weighted	2	0.03854	0.038456	0.316249	1.039292	0.963837	1.120655	0.977376677
CBLN4	Inverse variance weighted	3	0.007885	0.105639	0.940497	1.007917	0.819413	1.239784	0.977376677

ERAP1	Inverse variance weighted	3	0.000518	0.012158	0.96601	1.000518	0.976958	1.024647	0.977376677
ADA2	Inverse variance weighted	2	-0.00233	0.012362	0.850749	0.997677	0.973794	1.022145	0.977376677
KLK14	Inverse variance weighted	2	-0.04563	0.036944	0.216806	0.955397	0.888661	1.027144	0.977376677
PTGFRN	Inverse variance weighted	2	0.00698	0.012163	0.566061	1.007004	0.983282	1.031298	0.977376677
GALP	Inverse variance weighted	3	-0.00939	0.053197	0.859881	0.990653	0.892564	1.099523	0.977376677
MRC2	Inverse variance weighted	3	-0.00418	0.048615	0.931458	0.995827	0.90532	1.095382	0.977376677
CD300A	Inverse variance weighted	2	0.022258	0.02504	0.374065	1.022507	0.973536	1.073942	0.977376677
DPP7	Inverse variance weighted	2	0.009669	0.034532	0.77948	1.009716	0.943636	1.080423	0.977376677
CPA4	Inverse variance weighted	3	0.000749	0.013562	0.955948	1.000749	0.974498	1.027708	0.977376677
ENPP5	Inverse variance weighted	2	-0.00713	0.020871	0.732712	0.992897	0.9531	1.034356	0.977376677
SERPINA10	Inverse variance weighted	5	0.012035	0.022615	0.59462	1.012107	0.968225	1.057978	0.977376677
KLK12	Inverse variance weighted	3	0.003939	0.02903	0.892055	1.003947	0.948419	1.062726	0.977376677
ADAMTS5	Inverse variance weighted	2	-0.03053	0.031881	0.338215	0.969929	0.911177	1.03247	0.977376677
EDAR	Inverse variance weighted	4	-0.01888	0.026549	0.47706	0.981299	0.931542	1.033715	0.977376677
PPIE	Inverse variance weighted	2	-0.01195	0.038595	0.756841	0.988121	0.91613	1.065769	0.977376677
CLEC11A	Inverse variance weighted	2	0.006008	0.029513	0.838692	1.006026	0.949483	1.065936	0.977376677
SIGLEC7	Inverse variance weighted	3	0.020255	0.03446	0.556685	1.020461	0.953813	1.091766	0.977376677
MAN2B2	Inverse variance weighted	3	-0.01025	0.026601	0.699899	0.989799	0.939516	1.042773	0.977376677
NTNG1	Inverse variance weighted	2	-0.01314	0.022919	0.566511	0.986949	0.943595	1.032294	0.977376677
SIGLEC9	Inverse variance weighted	2	0.010142	0.012925	0.43263	1.010194	0.984924	1.036112	0.977376677
PPIL1	Inverse variance weighted	2	0.002668	0.044429	0.952106	1.002672	0.919053	1.093899	0.977376677
FKBP7	Inverse variance weighted	2	0.004486	0.021965	0.838159	1.004496	0.962169	1.048686	0.977376677
CFI	Wald ratio	1	0.000963	0.032734	0.976536	1.000963	0.938759	1.067289	0.982248148
RRM2B	Wald ratio	1	0.001296	0.045808	0.977421	1.001297	0.915315	1.095357	0.982248148
CD209	Inverse variance weighted	2	0.000552	0.030369	0.985509	1.000552	0.942734	1.061915	0.987936083
SELL	Inverse variance weighted	3	0.000768	0.067546	0.990927	1.000768	0.876673	1.14243	0.990926897

Table S8. MR results of the association between brain pQTL and ischemic stroke (IVW or Wald ratio methods).

Druggable gene	Method	Nsnp	beta	se	P value	OR	LL	LR	FDR
ALDH2	Wald ratio	1	0.621041	0.136047	5E-06	1.860863	1.425308	2.429519	0.001334378
HSD17B12	Wald ratio	1	-0.41807	0.110228	0.000149	0.658314	0.530401	0.817074	0.019886487
LARS	Wald ratio	1	0.965784	0.306291	0.001615	2.626845	1.441166	4.78801	0.143750168
LNPEP	Wald ratio	1	0.847697	0.282566	0.0027	2.334265	1.341607	4.061392	0.180211387
NUCB2	Wald ratio	1	-0.83077	0.307692	0.006934	0.435714	0.23839	0.796369	0.370272802
PTPN18	Wald ratio	1	-0.15291	0.06009	0.010935	0.858203	0.762852	0.965471	0.437543638
ABCC8	Wald ratio	1	0.509498	0.204154	0.012573	1.664455	1.115559	2.483427	0.437543638
ULK3	Wald ratio	1	0.241799	0.09747	0.01311	1.273539	1.052069	1.541629	0.437543638
AKR7A3	Wald ratio	1	0.289086	0.135693	0.033136	1.335206	1.023396	1.742018	0.441225559
MECR	Wald ratio	1	-0.51183	0.228385	0.025021	0.599399	0.383099	0.937824	0.441225559
ATG4B	Wald ratio	1	-0.76966	0.369437	0.037221	0.46317	0.224527	0.955458	0.441225559
SLC7A11	Wald ratio	1	0.339332	0.148243	0.022078	1.404009	1.049984	1.877401	0.441225559
ERAP2	Wald ratio	1	-0.05303	0.022247	0.017133	0.948348	0.907884	0.990615	0.441225559
ALDH5A1	Wald ratio	1	-0.28483	0.137285	0.038008	0.752139	0.574696	0.984368	0.441225559
ME1	Wald ratio	1	-0.3876	0.159599	0.015158	0.678686	0.496382	0.927944	0.441225559
IMPA1	Wald ratio	1	0.446783	0.214456	0.037221	1.563275	1.026802	2.380039	0.441225559
CD274	Wald ratio	1	-0.22622	0.106765	0.034106	0.797546	0.646957	0.983187	0.441225559
ALDH18A1	Wald ratio	1	0.289696	0.130912	0.026904	1.336021	1.033662	1.726824	0.441225559
CPT1A	Wald ratio	1	0.465737	0.215429	0.030626	1.593187	1.044456	2.430208	0.441225559
BCAT1	Wald ratio	1	0.414141	0.191919	0.030936	1.513071	1.03871	2.204064	0.441225559
MIPEP	Wald ratio	1	0.222375	0.098833	0.024449	1.249039	1.029076	1.516019	0.441225559
MPI	Wald ratio	1	-0.26953	0.122185	0.027392	0.763742	0.601091	0.970404	0.441225559
PDF	Wald ratio	1	-0.37585	0.161509	0.01996	0.686706	0.50037	0.942433	0.441225559
MLYCD	Wald ratio	1	0.391061	0.192039	0.041714	1.478549	1.014773	2.154283	0.464066627
SCRG1	Wald ratio	1	0.135962	0.067981	0.0455	1.145638	1.002723	1.308923	0.46725271
INPP5E	Wald ratio	1	0.312192	0.156096	0.0455	1.366418	1.006263	1.855475	0.46725271
CALU	Wald ratio	1	-0.42257	0.2145	0.048838	0.655363	0.430423	0.997857	0.48295722
LARS2	Wald ratio	1	0.177419	0.091129	0.051546	1.194132	0.998807	1.427654	0.491531803
DHODH	Wald ratio	1	0.189725	0.098706	0.054589	1.208917	0.996269	1.466953	0.502597648
UCHL3	Wald ratio	1	-0.2347	0.124109	0.058612	0.790807	0.62005	1.008589	0.504823361
CBR1	Wald ratio	1	0.211971	0.112026	0.05847	1.236113	0.992429	1.539632	0.504823361
PLD2	Wald ratio	1	-0.30021	0.160677	0.061703	0.740662	0.540567	1.014823	0.514837784
BCAN	Wald ratio	1	-0.38716	0.22474	0.084945	0.678984	0.437076	1.054781	0.527447739
HNMT	Wald ratio	1	-0.10588	0.061381	0.084527	0.89953	0.797567	1.014529	0.527447739
ATIC	Wald ratio	1	0.28852	0.166081	0.082347	1.334451	0.963678	1.847878	0.527447739
HHATL	Wald ratio	1	-0.17829	0.102623	0.082321	0.836697	0.684249	1.023109	0.527447739
GPX1	Wald ratio	1	0.168333	0.095	0.076406	1.183331	0.982292	1.425515	0.527447739
AKR1B1	Wald ratio	1	-0.78391	0.448534	0.080512	0.456616	0.189562	1.099895	0.527447739
CA8	Wald ratio	1	0.322281	0.182832	0.077949	1.380272	0.964572	1.975126	0.527447739
EFTUD1	Wald ratio	1	0.545181	0.313253	0.081792	1.72492	0.933518	3.187244	0.527447739
PRKCB	Wald ratio	1	-0.63508	0.345508	0.066048	0.529895	0.269207	1.043019	0.527447739
S100B	Wald ratio	1	0.182805	0.104072	0.078999	1.200581	0.979046	1.472243	0.527447739
SLC2A11	Wald ratio	1	-0.14116	0.079302	0.075076	0.868352	0.743349	1.014377	0.527447739
STAT6	Wald ratio	1	0.348403	0.207383	0.092957	1.416803	0.943587	2.127342	0.564081893
UMPS	Wald ratio	1	0.195402	0.117816	0.097209	1.2158	0.965106	1.531614	0.576774643
LRPAP1	Wald ratio	1	0.354251	0.220648	0.108383	1.425113	0.924762	2.196183	0.597200444
ARSB	Wald ratio	1	-0.10795	0.066429	0.104163	0.897674	0.788085	1.022503	0.597200444
TPMT	Wald ratio	1	0.074624	0.046205	0.106294	1.077479	0.984189	1.179612	0.597200444
GBA2	Wald ratio	1	0.220751	0.137969	0.109599	1.247012	0.951544	1.634227	0.597200444
S100A13	Wald ratio	1	0.058993	0.03741	0.114813	1.060768	0.985771	1.14147	0.601081006
CYP4F11	Wald ratio	1	0.18249	0.115316	0.11353	1.200202	0.957405	1.504572	0.601081006
GSTM3	Wald ratio	1	-0.04698	0.030453	0.122919	0.954108	0.898825	1.012791	0.607767161
KCNJ9	Wald ratio	1	0.122846	0.079401	0.121822	1.130711	0.967752	1.321109	0.607767161
MGMT	Inverse variance weighted	2	-0.07154	0.046325	0.122497	0.930957	0.850153	1.01944	0.607767161
DPP10	Wald ratio	1	-0.29557	0.195455	0.130484	0.74411	0.507297	1.09147	0.62324278
PYGB	Wald ratio	1	-0.25586	0.169297	0.130718	0.774254	0.555616	1.078927	0.62324278
APOH	Wald ratio	1	-0.10757	0.072096	0.135683	0.898012	0.779673	1.034312	0.635565401
P2RX7	Wald ratio	1	0.04125	0.027976	0.14036	1.042112	0.986508	1.100851	0.646139002
ABCA5	Wald ratio	1	-0.1883	0.128499	0.142826	0.82837	0.643938	1.065626	0.646349964
CBR3	Wald ratio	1	0.079213	0.054758	0.148008	1.082435	0.972279	1.205072	0.658637567
CAT	Wald ratio	1	-0.1295	0.091188	0.155559	0.878533	0.734746	1.050458	0.663673268
ABCB9	Wald ratio	1	-0.23524	0.166317	0.15724	0.79038	0.570511	1.094984	0.663673268
GALC	Wald ratio	1	0.057119	0.040563	0.159083	1.058782	0.977865	1.146395	0.663673268

SENP8	Wald ratio	1	0.394647	0.278975	0.157176	1.483861	0.858865	2.563665	0.663673268
CDC42BPA	Wald ratio	1	-0.74374	0.547206	0.174097	0.475334	0.162633	1.389281	0.685178439
LMCD1	Wald ratio	1	-0.2963	0.215686	0.169523	0.743567	0.487219	1.134791	0.685178439
CBR4	Wald ratio	1	0.20232	0.150773	0.179635	1.224239	0.911015	1.645156	0.685178439
HLA-B	Wald ratio	1	-0.09917	0.073576	0.177714	0.90559	0.783975	1.046071	0.685178439
TRAP1	Wald ratio	1	0.163913	0.119565	0.170403	1.178112	0.931989	1.489232	0.685178439
GPX4	Wald ratio	1	-0.29911	0.221811	0.177502	0.741478	0.480052	1.145271	0.685178439
SOD3	Wald ratio	1	-0.125	0.096014	0.192955	0.882497	0.731112	1.065228	0.705739843
PON1	Wald ratio	1	-0.05912	0.04506	0.189545	0.942598	0.86292	1.029633	0.705739843
ZADH2	Wald ratio	1	0.217899	0.166537	0.190734	1.243461	0.897167	1.723421	0.705739843
TKT	Wald ratio	1	-0.2431	0.19019	0.201181	0.784193	0.540169	1.138456	0.725881016
TRPV2	Wald ratio	1	0.044574	0.03553	0.209644	1.045582	0.975247	1.12099	0.746331982
GSTZ1	Wald ratio	1	-0.03533	0.028427	0.213871	0.965282	0.91297	1.020592	0.751363567
RNPEP	Wald ratio	1	0.197535	0.161889	0.222395	1.218395	0.887126	1.673366	0.755222579
PPID	Wald ratio	1	-0.07334	0.060241	0.223455	0.929288	0.825795	1.045751	0.755222579
FBP1	Wald ratio	1	-0.16216	0.132498	0.220997	0.850303	0.655827	1.10245	0.755222579
GGH	Wald ratio	1	-0.20402	0.169001	0.227352	0.815446	0.585516	1.13567	0.758788487
CRTAC1	Wald ratio	1	-0.26079	0.218502	0.232655	0.77044	0.502049	1.182311	0.766898739
RRM2B	Wald ratio	1	0.191235	0.161753	0.2371	1.210744	0.88179	1.662414	0.772021279
SLC7A8	Wald ratio	1	-0.12963	0.111111	0.243345	0.878421	0.706517	1.09215	0.782808644
AKR7A2	Wald ratio	1	-0.34913	0.302098	0.247809	0.7053	0.390142	1.275045	0.787677156
BBOX1	Wald ratio	1	0.0936	0.0816	0.251357	1.09812	0.935816	1.288574	0.789557669
ALDH16A1	Wald ratio	1	-0.08829	0.078261	0.259233	0.915491	0.785303	1.067263	0.798097901
ARSA	Wald ratio	1	-0.09996	0.088749	0.260054	0.904878	0.760405	1.0768	0.798097901
SLC6A17	Wald ratio	1	0.13607	0.122232	0.265619	1.145762	0.901671	1.455931	0.799957937
GFM2	Wald ratio	1	0.045223	0.041482	0.275641	1.046261	0.96456	1.134882	0.799957937
ERAP1	Wald ratio	1	-0.0575	0.052385	0.272396	0.944126	0.851999	1.046215	0.799957937
SHPK	Wald ratio	1	0.07151	0.065217	0.272864	1.074129	0.945241	1.220592	0.799957937
PLCG1	Wald ratio	1	-0.4304	0.387747	0.266999	0.650249	0.304105	1.390391	0.799957937
EIF2AK4	Wald ratio	1	0.233463	0.215953	0.279661	1.262966	0.82712	1.928479	0.80236155
CAMKK1	Wald ratio	1	0.282833	0.263158	0.282479	1.326884	0.792188	2.222478	0.80236155
AKR1A1	Wald ratio	1	0.069917	0.066544	0.2934	1.072419	0.941285	1.221822	0.815511968
PPOX	Wald ratio	1	-0.12581	0.121105	0.298882	0.881784	0.695465	1.118018	0.815511968
COQ6	Wald ratio	1	-0.12344	0.118648	0.298152	0.883873	0.700478	1.115283	0.815511968
MIF	Wald ratio	1	-0.23711	0.228453	0.299326	0.788907	0.504154	1.234494	0.815511968
CTSB	Wald ratio	1	-0.21948	0.21404	0.305163	0.802935	0.527821	1.221447	0.820442207
LGALS3	Wald ratio	1	0.099898	0.097848	0.307282	1.105058	0.91221	1.338675	0.820442207
PPAT	Wald ratio	1	-0.21415	0.211439	0.311146	0.807227	0.533353	1.221734	0.822534213
ITGA6	Wald ratio	1	-0.22149	0.221488	0.317311	0.801325	0.519128	1.236925	0.822542773
IGFBP7	Wald ratio	1	0.113649	0.113649	0.317311	1.120359	0.896638	1.399899	0.822542773
ECM1	Wald ratio	1	-0.17816	0.17982	0.321812	0.836813	0.58825	1.190404	0.826190916
MTAP	Wald ratio	1	0.135098	0.137855	0.327086	1.144649	0.87363	1.499743	0.831733273
DPP7	Wald ratio	1	-0.03897	0.041379	0.346363	0.961784	0.886859	1.043038	0.848431267
NTM	Wald ratio	1	-0.26493	0.28013	0.344283	0.76726	0.443089	1.3286	0.848431267
YARS2	Wald ratio	1	-0.20879	0.21978	0.342112	0.811565	0.527524	1.248544	0.848431267
GSTT2B	Wald ratio	1	-0.01488	0.015531	0.338071	0.985232	0.955692	1.015684	0.848431267
ALCAM	Wald ratio	1	-0.22723	0.245556	0.354772	0.796737	0.492372	1.289248	0.855575827
EARS2	Wald ratio	1	-0.05208	0.056382	0.355689	0.949258	0.849945	1.060175	0.855575827
GANAB	Wald ratio	1	-0.36808	0.403416	0.361554	0.692062	0.31387	1.525946	0.86191791
ENPP6	Wald ratio	1	0.053208	0.058946	0.366709	1.054649	0.939576	1.183816	0.866471937
SLC16A7	Wald ratio	1	-0.08826	0.1015	0.384538	0.915522	0.75036	1.117037	0.870098815
PSEN1	Wald ratio	1	-0.20305	0.23225	0.381963	0.816235	0.517751	1.286798	0.870098815
ACE	Wald ratio	1	0.115438	0.129392	0.372309	1.122365	0.87095	1.446356	0.870098815
GAA	Wald ratio	1	-0.10855	0.124058	0.381574	0.897133	0.703487	1.144083	0.870098815
LMAN1	Wald ratio	1	-0.32141	0.365474	0.379167	0.725126	0.354254	1.484265	0.870098815
SLC25A12	Wald ratio	1	0.190106	0.224857	0.397857	1.209378	0.778323	1.879162	0.870720049
MFI2	Wald ratio	1	-0.0961	0.113379	0.39665	0.908371	0.727367	1.134419	0.870720049
BHMT	Wald ratio	1	-0.03396	0.039848	0.394118	0.966613	0.893992	1.045133	0.870720049
FAS	Wald ratio	1	-0.02034	0.023883	0.3945	0.97987	0.935059	1.026828	0.870720049
PTGR1	Wald ratio	1	-0.04407	0.052991	0.405588	0.956886	0.862489	1.061614	0.875540996
LACTB	Wald ratio	1	-0.07511	0.091139	0.409897	0.927646	0.775895	1.109076	0.875540996
PEPD	Wald ratio	1	0.079796	0.096774	0.409621	1.083066	0.895941	1.309275	0.875540996
CDA	Wald ratio	1	0.075697	0.101594	0.456212	1.078636	0.883887	1.316295	0.905108742
CPT2	Wald ratio	1	-0.04394	0.057943	0.448251	0.957011	0.85427	1.072109	0.905108742
ACP6	Wald ratio	1	-0.02365	0.032924	0.472645	0.976632	0.915599	1.041733	0.905108742
PSMB4	Wald ratio	1	0.032407	0.045304	0.474406	1.032938	0.945171	1.128855	0.905108742

FDPS	Wald ratio	1	-0.07442	0.105116	0.478967	0.928283	0.755446	1.140663	0.905108742
APOA1BP	Wald ratio	1	0.077838	0.125653	0.535607	1.080948	0.84498	1.382812	0.905108742
MPZ	Wald ratio	1	-0.01195	0.019011	0.52963	0.988121	0.951979	1.025635	0.905108742
KHK	Wald ratio	1	0.023641	0.033097	0.475051	1.023922	0.959609	1.092546	0.905108742
NEK4	Wald ratio	1	-0.0442	0.063421	0.485822	0.95676	0.844925	1.083398	0.905108742
PRKCD	Wald ratio	1	0.078257	0.126054	0.534719	1.0814	0.84467	1.384478	0.905108742
ATP1B3	Wald ratio	1	0.059951	0.084672	0.478927	1.061784	0.899418	1.253461	0.905108742
PLSCR1	Wald ratio	1	0.078567	0.11554	0.496504	1.081736	0.862525	1.35666	0.905108742
BDH1	Wald ratio	1	-0.07036	0.104156	0.49937	0.932062	0.759951	1.143154	0.905108742
BDH2	Wald ratio	1	0.069444	0.109127	0.524539	1.071913	0.865503	1.327548	0.905108742
BTN3A3	Wald ratio	1	0.123419	0.158902	0.437336	1.131358	0.828591	1.544756	0.905108742
BTN2A1	Wald ratio	1	-0.03586	0.057489	0.532724	0.964771	0.861962	1.079842	0.905108742
GPNMB	Wald ratio	1	0.023569	0.034007	0.488266	1.023849	0.957831	1.094417	0.905108742
HSDL2	Wald ratio	1	-0.13265	0.19596	0.498455	0.875772	0.596467	1.285865	0.905108742
GRID1	Wald ratio	1	0.167933	0.268237	0.531274	1.182858	0.699205	2.001062	0.905108742
CD82	Wald ratio	1	-0.05581	0.075415	0.4593	0.945722	0.815772	1.096371	0.905108742
FOLH1	Wald ratio	1	0.056233	0.075881	0.45865	1.057844	0.911655	1.227475	0.905108742
GSTP1	Wald ratio	1	-0.06825	0.10771	0.526301	0.934025	0.756264	1.153569	0.905108742
HEBP1	Wald ratio	1	0.038773	0.049056	0.429307	1.039534	0.944238	1.144448	0.905108742
RECQL	Wald ratio	1	-0.13779	0.214947	0.521506	0.871285	0.571733	1.327782	0.905108742
DHRS7	Wald ratio	1	-0.17909	0.241088	0.457568	0.836027	0.521197	1.341031	0.905108742
MAN2C1	Wald ratio	1	0.101477	0.149004	0.49585	1.106805	0.826487	1.482198	0.905108742
DECR2	Wald ratio	1	0.100134	0.138852	0.470815	1.105318	0.841966	1.451044	0.905108742
CES1	Wald ratio	1	-0.03541	0.051382	0.490776	0.965214	0.872743	1.067482	0.905108742
WWOX	Wald ratio	1	0.065096	0.10436	0.532784	1.067262	0.869836	1.309496	0.905108742
SIRPA	Wald ratio	1	-0.03308	0.045434	0.466519	0.967458	0.885029	1.057564	0.905108742
CYB5R3	Wald ratio	1	-0.11648	0.17608	0.508267	0.890045	0.630274	1.256881	0.905108742
SERPINB9	Inverse variance weighted	2	-0.05864	0.08993	0.514334	0.943043	0.790645	1.124815	0.905108742
EPHX2	Inverse variance weighted	2	0.02826	0.037346	0.449232	1.028663	0.956056	1.106783	0.905108742
CRYZ	Wald ratio	1	-0.03282	0.060009	0.584465	0.967715	0.860333	1.0885	0.908106875
GSTM2	Wald ratio	1	0.026942	0.048047	0.57497	1.027308	0.934981	1.128753	0.908106875
GSTM1	Wald ratio	1	0.008139	0.014514	0.57497	1.008172	0.979896	1.037265	0.908106875
GSTM5	Wald ratio	1	0.007051	0.012575	0.57497	1.007076	0.982558	1.032206	0.908106875
TSTD1	Wald ratio	1	-0.09282	0.159119	0.559669	0.911358	0.667182	1.244898	0.908106875
OXSR1	Wald ratio	1	0.156028	0.285714	0.584998	1.168859	0.667663	2.04629	0.908106875
DAGLB	Wald ratio	1	0.090221	0.161108	0.575479	1.094416	0.798076	1.500792	0.908106875
SLC25A13	Wald ratio	1	-0.12326	0.208123	0.553695	0.884036	0.587912	1.329315	0.908106875
SLC25A32	Wald ratio	1	0.12091	0.211869	0.568216	1.128523	0.745013	1.709452	0.908106875
TXN	Wald ratio	1	0.108363	0.179034	0.545004	1.114452	0.784629	1.582918	0.908106875
SPRYD4	Wald ratio	1	-0.10703	0.192363	0.577944	0.898499	0.616275	1.309968	0.908106875
TXNRD1	Wald ratio	1	0.197304	0.328839	0.548506	1.218114	0.639402	2.320606	0.908106875
DHRS1	Wald ratio	1	-0.03013	0.054755	0.582155	0.970321	0.871579	1.080249	0.908106875
LSS	Wald ratio	1	0.036851	0.064768	0.56938	1.037538	0.913844	1.177975	0.908106875
SLC25A24	Wald ratio	1	0.031123	0.082544	0.706137	1.031613	0.877513	1.212773	0.910661415
GSTM4	Wald ratio	1	0.029083	0.058166	0.617075	1.02951	0.918584	1.15383	0.910661415
NENF	Wald ratio	1	0.089658	0.218932	0.682155	1.0938	0.712162	1.679953	0.910661415
CAPN2	Wald ratio	1	0.085794	0.225459	0.703551	1.089582	0.700399	1.695018	0.910661415
EPHX1	Wald ratio	1	0.069052	0.145591	0.635296	1.071491	0.805489	1.425338	0.910661415
TP53I3	Wald ratio	1	0.035577	0.077723	0.647135	1.036218	0.889799	1.20673	0.910661415
QPCT	Wald ratio	1	0.060592	0.125845	0.630174	1.062465	0.830221	1.359678	0.910661415
PCYOX1	Wald ratio	1	-0.12018	0.346661	0.728842	0.886765	0.449495	1.749411	0.910661415
DUSP19	Wald ratio	1	-0.0515	0.113019	0.648608	0.949802	0.761079	1.185322	0.910661415
PPIL3	Wald ratio	1	-0.01175	0.03334	0.724415	0.988314	0.925795	1.055055	0.910661415
OXNAD1	Wald ratio	1	0.027039	0.060086	0.65271	1.027407	0.913265	1.155816	0.910661415
EXOG	Wald ratio	1	0.059587	0.133739	0.655926	1.061398	0.816652	1.379493	0.910661415
POGLUT1	Wald ratio	1	0.066445	0.197381	0.736393	1.068702	0.725843	1.573516	0.910661415
GFM1	Wald ratio	1	-0.07729	0.226202	0.732602	0.925625	0.59414	1.442056	0.910661415
MAN2B2	Wald ratio	1	-0.02204	0.064861	0.734003	0.978201	0.861424	1.110809	0.910661415
ABCG2	Wald ratio	1	-0.03494	0.093178	0.70766	0.965662	0.804471	1.15915	0.910661415
ADH5	Wald ratio	1	-0.08283	0.199088	0.677388	0.920511	0.623105	1.359866	0.910661415
MTRR	Wald ratio	1	0.058612	0.124402	0.637531	1.060364	0.830925	1.353157	0.910661415
THBS4	Wald ratio	1	-0.0413	0.114118	0.717423	0.959541	0.767228	1.20006	0.910661415
PCYOX1L	Wald ratio	1	0.08729	0.215031	0.684786	1.091213	0.715932	1.663211	0.910661415
NQO2	Wald ratio	1	-0.02027	0.055311	0.714009	0.979934	0.879255	1.092141	0.910661415
PON2	Wald ratio	1	0.024731	0.073557	0.736715	1.025039	0.887415	1.184005	0.910661415
PDIA4	Wald ratio	1	0.064364	0.153484	0.674957	1.066481	0.789414	1.440793	0.910661415

ADHFE1	Wald ratio	1	-0.02188	0.043077	0.611498	0.978357	0.899145	1.064548	0.910661415
DECR1	Wald ratio	1	0.075502	0.185542	0.684062	1.078425	0.749641	1.551411	0.910661415
ALDH1B1	Wald ratio	1	-0.01345	0.031588	0.670249	0.98664	0.927408	1.049655	0.910661415
PRTFDC1	Wald ratio	1	-0.06201	0.143195	0.664963	0.93987	0.709868	1.244395	0.910661415
PRKG1	Wald ratio	1	-0.05906	0.111561	0.59652	0.942649	0.757508	1.173039	0.910661415
COMTD1	Wald ratio	1	-0.04291	0.124932	0.731239	0.957996	0.749928	1.223793	0.910661415
AMPD3	Wald ratio	1	-0.05195	0.109416	0.634967	0.949381	0.766132	1.176461	0.910661415
PYGM	Wald ratio	1	-0.05585	0.115074	0.627407	0.945677	0.754727	1.184938	0.910661415
VWA5A	Wald ratio	1	0.092484	0.181456	0.610277	1.096896	0.768611	1.565396	0.910661415
DCPS	Wald ratio	1	0.107633	0.210318	0.608816	1.113639	0.737426	1.681786	0.910661415
SLC25A15	Wald ratio	1	-0.04276	0.084642	0.61345	0.958144	0.811674	1.131044	0.910661415
PTGR2	Wald ratio	1	-0.04212	0.104155	0.685939	0.958757	0.781718	1.175891	0.910661415
CTSH	Wald ratio	1	-0.01654	0.044018	0.707037	0.983593	0.902291	1.07222	0.910661415
ALDH1A3	Wald ratio	1	-0.03607	0.080622	0.654609	0.964575	0.823586	1.1297	0.910661415
NQO1	Wald ratio	1	0.007966	0.016715	0.633674	1.007997	0.97551	1.041567	0.910661415
APRT	Wald ratio	1	-0.07996	0.200351	0.689803	0.923149	0.623346	1.367144	0.910661415
CA4	Wald ratio	1	0.038729	0.082572	0.639051	1.039488	0.884164	1.222099	0.910661415
TRIM65	Wald ratio	1	-0.03348	0.086608	0.699086	0.967075	0.816089	1.145995	0.910661415
TYK2	Wald ratio	1	0.046232	0.085992	0.590829	1.047317	0.884873	1.239584	0.910661415
GSS	Wald ratio	1	0.069767	0.178891	0.696537	1.072259	0.755135	1.522561	0.910661415
JAM2	Wald ratio	1	-0.05923	0.141724	0.676013	0.942492	0.713904	1.244273	0.910661415
GLB1	Wald ratio	1	-0.06177	0.192587	0.748396	0.940096	0.644523	1.371216	0.918275048
GART	Wald ratio	1	-0.02144	0.067207	0.749753	0.978791	0.85799	1.116601	0.918275048
BPHL	Wald ratio	1	-0.01602	0.051813	0.757252	0.984112	0.889079	1.089304	0.923225497
CEP41	Wald ratio	1	0.086627	0.286952	0.762738	1.09049	0.621389	1.913727	0.925687015
GLRX3	Wald ratio	1	0.080053	0.271848	0.768392	1.083345	0.635865	1.845731	0.926393254
PDE1B	Wald ratio	1	0.09116	0.312155	0.77026	1.095445	0.594127	2.01977	0.926393254
ALDH4A1	Wald ratio	1	0.032233	0.12	0.788231	1.032758	0.816305	1.306606	0.935367497
PI16	Wald ratio	1	-0.01014	0.03762	0.787542	0.989912	0.919547	1.065662	0.935367497
COMT	Wald ratio	1	-0.01781	0.066158	0.787752	0.982346	0.862879	1.118353	0.935367497
PPIL1	Wald ratio	1	-0.02443	0.096544	0.800201	0.975862	0.807624	1.179147	0.945370719
CAMKK2	Wald ratio	1	-0.05022	0.204752	0.806237	0.951018	0.63665	1.420617	0.948305546
CD200	Wald ratio	1	0.003782	0.017267	0.826613	1.003789	0.970386	1.038342	0.95958982
PLSCR4	Wald ratio	1	0.015344	0.068712	0.823301	1.015462	0.887512	1.161857	0.95958982
ADA	Wald ratio	1	0.021138	0.092683	0.819592	1.021363	0.851701	1.224823	0.95958982
DCK	Wald ratio	1	0.035052	0.170427	0.837046	1.035674	0.741571	1.446417	0.963324213
ABCB8	Wald ratio	1	0.024857	0.118547	0.833919	1.025168	0.812617	1.293315	0.963324213
CASP9	Wald ratio	1	-0.02158	0.112462	0.847806	0.978648	0.78505	1.219988	0.971378357
BOLA1	Wald ratio	1	-0.01558	0.085895	0.856075	0.984542	0.831992	1.165063	0.971378357
CHL1	Wald ratio	1	-0.0415	0.232963	0.858597	0.959345	0.607677	1.514526	0.971378357
NT5E	Wald ratio	1	-0.01753	0.095078	0.853702	0.982621	0.815557	1.183907	0.971378357
FAAH	Wald ratio	1	-0.00991	0.082563	0.904483	0.990141	0.842206	1.164062	0.972170273
WARS2	Wald ratio	1	0.012587	0.116084	0.913652	1.012667	0.806592	1.271391	0.972170273
DUSP23	Wald ratio	1	0.006229	0.050396	0.901636	1.006248	0.911605	1.110717	0.972170273
FABP1	Wald ratio	1	0.003926	0.028957	0.892143	1.003934	0.948542	1.062561	0.972170273
THNSL2	Wald ratio	1	-0.00308	0.034472	0.928855	0.996927	0.931794	1.066612	0.972170273
SLC15A2	Wald ratio	1	-0.00925	0.083263	0.911528	0.990791	0.841603	1.166426	0.972170273
CD38	Wald ratio	1	-0.0098	0.069979	0.88866	0.990251	0.863332	1.135828	0.972170273
NAAA	Wald ratio	1	-0.00685	0.06225	0.912337	0.99317	0.879094	1.12205	0.972170273
SV2C	Wald ratio	1	0.009025	0.084838	0.915278	1.009066	0.854485	1.191612	0.972170273
GLO1	Wald ratio	1	-0.02432	0.275601	0.92969	0.975976	0.568647	1.675079	0.972170273
FUCA2	Wald ratio	1	-0.00471	0.059193	0.936519	0.995297	0.88627	1.117735	0.972170273
SOD2	Wald ratio	1	0.018102	0.134631	0.893044	1.018266	0.782099	1.325749	0.972170273
ADK	Wald ratio	1	0.025504	0.283091	0.928216	1.025832	0.588985	1.786685	0.972170273
GSTO1	Wald ratio	1	0.003926	0.047418	0.934009	1.003934	0.914834	1.101712	0.972170273
DHRS4	Wald ratio	1	-0.00335	0.044061	0.9394	0.996656	0.914197	1.086552	0.972170273
HAGH	Wald ratio	1	0.030939	0.19505	0.873968	1.031423	0.703731	1.511702	0.972170273
SULT1A1	Wald ratio	1	-0.00419	0.040746	0.918119	0.99582	0.919384	1.078611	0.972170273
TRIM25	Wald ratio	1	-0.03195	0.190044	0.866472	0.968551	0.66735	1.405694	0.972170273
PRKCA	Wald ratio	1	-0.03593	0.279726	0.8978	0.96471	0.557556	1.669185	0.972170273
SERPINB8	Wald ratio	1	-0.02046	0.122769	0.867632	0.979746	0.770212	1.246284	0.972170273
BCL2L13	Wald ratio	1	-0.01365	0.120858	0.910107	0.986447	0.778391	1.250115	0.972170273
MGST1	Inverse variance weighted	2	-0.01302	0.083741	0.876412	0.987061	0.83765	1.163123	0.972170273
CDH13	Wald ratio	1	0.012163	0.180712	0.946337	1.012238	0.710325	1.442474	0.9733579
PDE4C	Wald ratio	1	-0.00653	0.099813	0.947839	0.993491	0.816961	1.208167	0.9733579
HDGF	Wald ratio	1	0.006741	0.176946	0.969612	1.006764	0.711718	1.424122	0.974375751

CFHR1	Wald ratio	1	0.001877	0.033789	0.955696	1.001879	0.937677	1.070477	0.974375751
ACP1	Wald ratio	1	0.002418	0.064087	0.969898	1.002421	0.884094	1.136586	0.974375751
HSD17B4	Wald ratio	1	0.006656	0.164725	0.967771	1.006678	0.728909	1.390297	0.974375751
MVD	Wald ratio	1	0.008003	0.163252	0.960904	1.008035	0.732002	1.388157	0.974375751
ABHD12	Wald ratio	1	0.008047	0.219294	0.970726	1.00808	0.655886	1.549393	0.974375751
ALDH9A1	Wald ratio	1	0.002412	0.138671	0.986124	1.002415	0.76385	1.315487	0.986124446

Table S9. Detailed MR results of the significant druggable genes (FDR < 0.1) and ischemic stroke.

Druggable gene	Method	Nsnp	beta	se	OR (95% CI)	P value	F-statistic	FDR	$P_{\text{for heterogeneity}}$	$P_{\text{for pleiotropy}}$	$P_{\text{for Steiger directionality test}}$
**Blood eQTL**											
CALCRL	Inverse variance weighted	3	0.197	0.049	1.218 (1.107–1.341)	5.12E-05	93.14	0.062	0.477	0.881	<0.001
NMB	Inverse variance weighted	3	-0.155	0.038	0.857 (0.795–0.923)	5.40E-05	167.42	0.062	0.966	0.853	<0.001
KCNJ11	Inverse variance weighted	2	0.249	0.062	1.282 (1.136–1.447)	5.73E-05	97.01	0.062	0.624	NA	<0.001
**Brain eQTL**											
MMP3	Wald ratio	1	0.479	0.099	1.615 (1.329–1.962)	1.39E-06	4.06	0.005	NA	NA	<0.001
MAPKAPK5	Wald ratio	1	0.545	0.122	1.725 (1.357–2.193)	8.60E-06	8.39	0.01	NA	NA	<0.001
DNMT1	Wald ratio	1	-0.879	0.194	0.415 (0.284–0.607)	6.02E-06	3.94	0.01	NA	NA	<0.001
ALDH2	Wald ratio	1	0.237	0.055	1.267 (1.137–1.412)	1.82E-05	63.68	0.016	NA	NA	<0.001
HSD17B12	Inverse variance weighted	3	-0.083	0.020	0.921 (0.885–0.958)	4.26E-05	193.16	0.03	0.841	0.801	<0.001
SIAE	Inverse variance weighted	2	0.513	0.132	1.670 (1.288–2.165)	1.08E-04	4.09	0.063	0.431	NA	<0.001
NEK3	Wald ratio	1	-0.370	0.097	0.691 (0.572–0.835)	1.31E-04	22.79	0.064	NA	NA	<0.001
THSD1	Wald ratio	1	-0.283	0.074	0.754 (0.651–0.872)	1.46E-04	26.54	0.064	NA	NA	<0.001
NMB	Wald ratio	1	-0.408	0.110	0.665 (0.536–0.826)	2.14E-04	8.14	0.083	NA	NA	<0.001
**Blood pQTL**											
MMP12	Inverse variance weighted	2	-0.097	0.019	0.907 (0.874–0.942)	4.17E-07	57.31	1.70E-04	0.529	NA	<0.001
LILRB2	Wald ratio	1	0.275	0.078	1.317 (1.129–1.536)	4.53E-04	1.66	0.092	NA	NA	<0.001
**Brain pQTL**											
ALDH2	Wald ratio	1	0.621	0.136	1.861 (1.425–2.430)	5.00E-06	19.41	0.005	NA	NA	<0.001
HSD17B12	Wald ratio	1	-0.418	0.110	0.658 (0.530–0.817)	1.49E-04	48.70	0.02	NA	NA	<0.001

Table S10. The results of the colocalization analysis.

Druggable gene	Nsnp	PP.H0.abf	PP.H1.abf	PP.H2.abf	PP.H3.abf	PP.H4.abf
**Blood_eQTL**						
CALCRL	1413	2.77E-133	7.92E-02	3.29E-133	9.32E-02	8.28E-01
NMB	1636	5.72E-293	4.34E-01	2.54E-293	1.92E-01	3.73E-01
KCNJ11	445	2.86E-32	2.21E-01	2.39E-33	1.77E-02	7.61E-01
**Brain_eQTL**						
MMP3	4411	1.15E-03	5.04E-04	5.29E-01	2.32E-01	2.37E-01
MAPKAPK5	1493	3.49E-11	2.65E-08	1.31E-03	9.99E-01	2.13E-05
DNMT1	3005	3.46E-02	7.73E-03	7.72E-01	1.72E-01	1.35E-02
ALDH2	1592	2.19E-50	2.65E-08	8.23E-43	1.00E+00	3.66E-06
HSD17B12	3393	1.69E-209	1.33E-01	1.95E-209	1.54E-01	7.13E-01
SIAE	3568	4.07E-02	2.22E-01	3.45E-02	1.88E-01	5.15E-01
NEK3	2432	4.01E-08	1.32E-01	3.20E-08	1.05E-01	7.63E-01
THSD1	2587	1.47E-09	5.00E-02	1.19E-09	3.95E-02	9.10E-01
NMB	3214	2.78E-02	3.27E-01	1.40E-02	1.64E-01	4.67E-01
**Blood_pQTL**						
MMP12	6992	7.08E-104	2.38E-04	4.28E-101	1.43E-01	8.57E-01
LILRB2	7450	1.75E-124	8.34E-01	3.32E-125	1.58E-01	7.81E-03
**Brain_pQTL**						
ALDH2	61	1.03E-14	8.51E-04	9.99E-12	8.21E-01	1.78E-01
HSD17B12	227	1.01E-18	1.50E-01	3.35E-19	4.89E-02	8.01E-01

Table S11. The detail of the 783 phenotypes in the phenome-wide MR analysis.

Category	Phenostring	Phenocode	Number of case	Number of control
hematopoietic	Disorders of iron metabolism	275.1	669	406834
digestive	Intestinal malabsorption (non-celiac)	557	2103	334783
digestive	Celiac disease	557.1	1855	334783
dermatologic	Diseases of hair and hair follicles	704	5344	402357
dermatologic	Diseases of sebaceous glands	706	8948	399255
dermatologic	Sebaceous cyst	706.2	8876	399255
musculoskeletal	Contracture of palmar fascia [Dupuytren's disease]	728.71	3503	378711
musculoskeletal	Fasciitis	728.7	3843	378711
musculoskeletal	Disorders of muscle, ligament, and fascia	728	4488	378711
endocrine/metabolic	Disorders of mineral metabolism	275	2127	406834
musculoskeletal	Ankylosing spondylitis	715.2	620	365085
digestive	Cholelithiasis and cholecystitis	574	16225	391307
digestive	Cholelithiasis	574.1	13777	391307
circulatory system	Atrial fibrillation and flutter	427.2	14820	380919
dermatologic	Psoriasis	696.4	2237	398199
dermatologic	Psoriasis and related disorders	696	2293	398199
endocrine/metabolic	Type 1 diabetes	250.1	2660	388756
hematopoietic	Coagulation defects	286	941	406281
dermatologic	Psoriasis vulgaris	696.41	1684	398199
endocrine/metabolic	Type 2 diabetes	250.2	18945	388756
endocrine/metabolic	Diabetes mellitus	250	20203	388756
musculoskeletal	Rheumatoid arthritis	714.1	4412	365085
musculoskeletal	Other inflammatory spondylopathies	715	1671	365085
musculoskeletal	Rheumatoid arthritis and other inflammatory polyarthropathies	714	4879	365085
circulatory system	Phlebitis and thrombophlebitis of lower extremities	451.2	3587	369592
circulatory system	Phlebitis and thrombophlebitis	451	3900	369592
endocrine/metabolic	Hypothyroidism NOS	244.4	14171	391429
endocrine/metabolic	Hypothyroidism	244	14871	391429
neoplasms	Other non-epithelial cancer of skin	172.2	11149	395071
circulatory system	Coronary atherosclerosis	411.4	20023	377103
digestive	Cholelithiasis with other cholecystitis	574.12	5472	391307
neoplasms	Skin cancer	172	13752	395071
circulatory system	Cardiac dysrhythmias	427	24681	380919
mental disorders	Dementias	290.1	956	402383
neurological	Multiple sclerosis	335	1356	395209
circulatory system	Varicose veins of lower extremity	454.1	11697	369592
circulatory system	Varicose veins	454	12172	369592
endocrine/metabolic	Disorders of lipoid metabolism	272	35927	373034
endocrine/metabolic	Hyperlipidemia	272.1	35844	373034
circulatory system	Ischemic Heart Disease	411	31355	377103
neoplasms	Breast cancer	174	12898	388549
neoplasms	Breast cancer [female]	174.1	12671	388549
neoplasms	Malignant neoplasm of female breast	174.11	11874	388549
endocrine/metabolic	Hypercholesterolemia	272.11	33242	373034
circulatory system	Circulatory disease NEC	459.9	16366	387905
respiratory	Asthma	495	26332	375505
endocrine/metabolic	Gout	274.1	3195	405198
endocrine/metabolic	Other disorders of metabolism	277	1424	407537
circulatory system	Other disorders of circulatory system	459	16544	387905
mental disorders	Delirium dementia and amnestic and other cognitive disorders	290	1970	402383
endocrine/metabolic	Gout and other crystal arthropathies	274	3763	405198
circulatory system	Myocardial infarction	411.2	11703	377103
digestive	Calculus of bile duct	574.2	2634	391307
circulatory system	Hypertension	401	77977	330366
circulatory system	Essential hypertension	401.1	77723	330366
circulatory system	Angina pectoris	411.3	16175	377103
circulatory system	Other chronic ischemic heart disease, unspecified	411.8	14921	377103
digestive	Inguinal hernia	550.1	15995	361617
digestive	Inflammatory bowel disease and other gastroenteritis and colitis	555	4528	334783
endocrine/metabolic	Type 2 diabetes with ophthalmic manifestations	250.23	1298	388756
endocrine/metabolic	Diabetic retinopathy	250.7	1339	396859
digestive	Diverticulosis and diverticulitis	562	27311	334783
digestive	Diverticulosis	562.1	27268	334783
respiratory	Nasal polyps	471	3311	390045
endocrine/metabolic	Thyrotoxicosis with or without goiter	242	1860	391429
genitourinary	Hematuria	593	16409	379936
circulatory system	Pulmonary heart disease	415	4257	402375
digestive	Ulcerative colitis	555.2	3195	334783
sense organs	Cataract	366	20352	388609
digestive	Other biliary tract disease	575	3892	391307
neoplasms	Benign neoplasm of skin	216	7722	400618
dermatologic	Psoriatic arthropathy	696.42	708	398199
neoplasms	Benign neoplasm of colon	208	20204	386011
endocrine/metabolic	Hypoglycemia	251.1	939	386319
endocrine/metabolic	Other disorders of pancreatic internal secretion	251	943	405386
digestive	Cholecystitis without cholelithiasis	574.3	2761	391307
injuries & poisonings	Fracture of radius and ulna	803.2	5246	387765
neoplasms	Melanomas of skin, dx or hx	172.1	2691	395071
neoplasms	Melanomas of skin	172.11	2691	395071
digestive	Abdominal hernia	550	47344	361617
neoplasms	Benign neoplasm of unspecified sites	229	2402	406559
sense organs	Corneal opacity and other disorders of cornea	364	732	397761
endocrine/metabolic	Overweight, obesity and other hyperalimentation	278	10968	397993
musculoskeletal	Polymyalgia Rheumatica	717	1152	407809
endocrine/metabolic	Obesity	278.1	10799	397993
digestive	Ileostomy status	559	1660	334783
injuries & poisonings	Allergy/adverse effect of penicillin	960.2	16090	381797

digestive	Cholelithiasis with acute cholecystitis	574.11	1513	391307
digestive	Regional enteritis	555.1	1743	334783
digestive	Chronic liver disease and cirrhosis	571	2895	400055
respiratory	Postinflammatory pulmonary fibrosis	502	887	397411
injuries & poisonings	Poisoning by antibiotics	960	18430	381797
endocrine/metabolic	Nontoxic multinodular goiter	241.2	680	391429
digestive	Umbilical hernia	550.4	3727	361617
circulatory system	Unstable angina (intermediate coronary syndrome)	411.1	5181	377103
injuries & poisonings	Fracture of upper limb	803	8521	387765
neoplasms	Uterine leiomyoma	218.1	10345	391653
dermatologic	Actinic keratosis	702.1	2594	403439
neoplasms	Benign neoplasm of uterus	218	10610	391653
sense organs	Senile cataract	366.2	8369	388609
genitourinary	Calculus of kidney	594.1	3191	401005
respiratory	Emphysema	496.1	1727	375505
digestive	Other chronic nonalcoholic liver disease	571.5	1664	400055
sense organs	Macular degeneration (senile) of retina NOS	362.29	2188	396859
sense organs	Degeneration of macula and posterior pole of retina	362.2	2191	396859
neoplasms	Benign neoplasm of other parts of digestive system	211	5280	395301
digestive	Appendiceal conditions	540	3409	405552
digestive	Anal and rectal polyp	565.1	7408	387338
digestive	Appendicitis	540.1	3217	405552
symptoms	Swelling of limb	771.1	4663	403468
neoplasms	Myeloproliferative disease	200	995	404466
digestive	Diaphragmatic hernia	550.2	27126	361617
sense organs	Glaucoma	365	4462	397761
digestive	Other disorders of gallbladder	575.7	1412	391307
symptoms	Musculoskeletal symptoms referable to limbs	771	5493	403468
musculoskeletal	Unspecified monoarthritis	716.2	15790	365819
genitourinary	Urinary calculus	594	6643	401005
genitourinary	Genital prolapse	618	11966	396730
sense organs	Retinal detachments and defects	361	3263	397761
endocrine/metabolic	Nontoxic nodular goiter	241	1143	391429
genitourinary	Polyp of corpus uteri	622.1	7910	396384
circulatory system	Peripheral vascular disease, unspecified	443.9	2566	400595
injuries & poisonings	Internal derangement of knee	835	15430	391457
dermatologic	Seborrheic keratosis	702.2	3092	403439
neoplasms	Non-Hodgkins lymphoma	202.2	1793	404466
mental disorders	Alcohol-related disorders	317	12922	379355
neoplasms	Lipoma	214	6271	401613
dermatologic	Unspecified diffuse connective tissue disease	709.7	2720	399404
symptoms	Syncope and collapse	788	9163	399798
neoplasms	Malignant neoplasm of testis	187.2	2981	401788
musculoskeletal	Other disorders of soft tissues	729	6170	378711
neurological	Other peripheral nerve disorders	351	12592	394067
neoplasms	Acquired absence of breast	175	2415	387566
congenital anomalies	Congenital anomalies of great vessels	747.13	1799	406165
neoplasms	Colorectal cancer	153	4562	382756
digestive	Acute appendicitis	540.11	2608	405552
dermatologic	Sarcoidosis	697	548	402672
neoplasms	Lipoma of skin and subcutaneous tissue	214.1	4611	401613
dermatologic	Sicca syndrome	709.2	513	399404
hematopoietic	Other anemias	285	12256	390026
dermatologic	Degenerative skin conditions and other dermatoses	702	5522	398746
respiratory	Chronic airway obstruction	496	10502	375505
neoplasms	Malignant neoplasm of bladder	189.21	2146	404796
circulatory system	Hemorrhoids	455	23896	369592
dermatologic	Diffuse diseases of connective tissue	709	3463	399404
dermatologic	Erythematous conditions	695	2420	402672
musculoskeletal	Hallux valgus (Bunion)	735.3	6699	394914
injuries & poisonings	Fracture of hand or wrist	804	3357	387765
neoplasms	Cancer of bladder	189.2	2427	404796
musculoskeletal	Ganglion and cyst of synovium, tendon, and bursa	727.4	3185	378711
musculoskeletal	Osteoarthrosis	740	28439	380522
genitourinary	Irregular menstrual cycle/bleeding	626.1	15880	377857
mental disorders	Tobacco use disorder	318	19780	379355
musculoskeletal	Osteoporosis NOS	743.11	5622	401279
neoplasms	Cancer of urinary organs (incl. kidney and bladder)	189	4165	404796
digestive	Other disorders of biliary tract	575.8	1040	391307
digestive	Duodenal ulcer	531.3	3002	401525
neoplasms	Cancer of other lymphoid, histiocytic tissue	202	2270	404466
neoplasms	Carcinoma in situ of skin	172.3	667	395071
sense organs	Other retinal disorders	362	3867	396859
genitourinary	Polyp of female genital organs	622	10881	396384
genitourinary	Excessive or frequent menstruation	626.12	9820	377857
endocrine/metabolic	Simple and unspecified goiter	240	602	391429
musculoskeletal	Osteoarthritis; localized	740.1	17691	380522
genitourinary	Urinary incontinence	599.4	8856	384930
mental disorders	Neurological disorders	292	4655	402383
mental disorders	Altered mental status	292.4	2189	402383
respiratory	Chronic sinusitis	475	2602	390045
digestive	Femoral hernia	550.3	651	361617
digestive	Duodenitis	535.6	7655	378124
dermatologic	Cellulitis and abscess of arm/hand	681.3	5539	397635
dermatologic	Cellulitis and abscess of leg, except foot	681.5	5547	397635
digestive	Anal and rectal conditions	565	14997	387338
dermatologic	Superficial cellulitis and abscess	681	7451	397635
dermatologic	Cellulitis and abscess of foot, toe	681.6	5502	397635
circulatory system	Occlusion and stenosis of precerebral arteries	433.1	1185	399017
sense organs	Otitis media	381.1	1824	404888

neoplasms	Malignant neoplasm of other and ill-defined sites within the digestive orga	159	5584	393372
mental disorders	Alcoholic liver damage	317.11	802	379355
genitourinary	Benign neoplasm of breast	610.4	1553	401746
respiratory	Obstructive chronic bronchitis	496.21	2698	375505
genitourinary	Uterine/Uterovaginal prolapse	618.2	5428	396730
circulatory system	Peripheral vascular disease	443	3927	400595
genitourinary	Disorders of menstruation and other abnormal bleeding from female genit	626	18580	377857
congenital anomalies	Cardiac congenital anomalies	747.1	2618	406165
sense organs	Inflammation of the eye	371	3174	399306
symptoms	Other abnormal blood chemistry	790.6	6413	402342
sense organs	Otitis media and Eustachian tube disorders	381	2259	404888
circulatory system	Cerebrovascular disease	433	8742	399017
digestive	Gastric ulcer	531.2	4109	401525
musculoskeletal	Osteoporosis	743.1	6484	401279
respiratory	Chronic bronchitis	496.2	2934	375505
digestive	Acute pancreatitis	577.1	1986	406271
musculoskeletal	Other arthropathies	716	38715	365819
injuries & poisonings	Fracture of lower limb	800	7251	387765
endocrine/metabolic	Hyperparathyroidism	252.1	781	405386
symptoms	Nonspecific findings on examination of blood	790	6619	402342
infectious diseases	Viral warts & HPV	78	1007	403316
hematopoietic	Iron deficiency anemias, unspecified or not due to blood loss	280.1	7414	390026
neoplasms	Cancer of bronchus; lung	165.1	2101	406226
digestive	Esophagitis, GERD and related diseases	530.1	32108	369275
genitourinary	Postmenopausal atrophic vaginitis	627.3	1115	377857
injuries & poisonings	Fracture of pelvis	802	722	387765
dermatologic	Atopic/contact dermatitis due to other or unspecified	939	2110	404817
musculoskeletal	Enthesopathy	726.1	9668	378711
genitourinary	Bladder neck obstruction	596.1	1980	394699
musculoskeletal	Arthropathy NOS	716.9	37043	365819
circulatory system	Atherosclerosis of the extremities	440.2	811	400595
congenital anomalies	Cardiac and circulatory congenital anomalies	747	2796	406165
genitourinary	stress incontinence, female	624.9	5924	399509
endocrine/metabolic	Type 2 diabetes with neurological manifestations	250.24	575	388756
digestive	Noninfectious gastroenteritis	558	15747	334783
digestive	Obstruction of bile duct	575.2	777	391307
dermatologic	Prurigo and Lichen	695.7	779	402672
genitourinary	Calculus of ureter	594.3	2417	401005
symptoms	Pain in limb	773	6327	402634
circulatory system	Atherosclerosis	440	1324	400595
genitourinary	Prolapse of vaginal walls	618.1	7462	396730
neoplasms	Colon cancer	153.2	3051	382756
circulatory system	Other disorders of arteries and arterioles	447	1333	400595
genitourinary	Endometriosis	615	4053	399757
digestive	Portal hypertension	571.81	529	400055
sense organs	Myopia	367.1	1257	406530
digestive	Ventral hernia	550.5	3448	361617
sense organs	Inflammation of eyelids	371.3	2396	399306
digestive	Diseases of esophagus	530	35852	369275
musculoskeletal	Osteoarthrosis NOS	740.9	12436	380522
neoplasms	Cancer within the respiratory system	165	2700	406226
mental disorders	Mood disorders	296	12560	365476
circulatory system	Abdominal aortic aneurysm	442.11	903	400595
genitourinary	Other nonmalignant breast conditions	613	1983	406978
mental disorders	Psychogenic and somatoform disorders	303	529	365476
digestive	Diseases of pancreas	577	2690	406271
neoplasms	Manlignant and unknown neoplasms of brain and nervous system	191	655	407239
endocrine/metabolic	Disorders of parathyroid gland	252	877	405386
digestive	Diseases of pulp and periapical tissues	522	1795	398136
genitourinary	Chronic cystitis	592.12	892	379936
genitourinary	Pelvic inflammatory disease (PID)	614.3	936	399757
injuries & poisonings	Dislocation	830	2094	391457
circulatory system	Other aneurysm	442	1808	400595
genitourinary	Other disorders of bladder	596	9933	394699
pregnancy complications	Early or threatened labor; hemorrhage in early pregnancy	636	2558	400946
digestive	Peptic ulcer (excl. esophageal)	531	7436	401525
symptoms	Other tests	1010	5972	402989
sense organs	Primary open angle glaucoma	365.11	1037	397761
circulatory system	Polyarteritis nodosa and allied conditions	446	828	400595
injuries & poisonings	Superficial injury without mention of infection	915	4289	403595
neoplasms	Lymphoid leukemia, chronic	204.12	506	404466
mental disorders	Depression	296.2	11901	365476
endocrine/metabolic	Abnormal glucose	250.4	685	388756
sense organs	Aphakia and other disorders of lens	379.3	1825	401245
digestive	Jaundice (not of newborn)	573.5	897	400055
sense organs	Open-angle glaucoma	365.1	1043	397761
sense organs	Other disorders of tympanic membrane	384	1364	404888
respiratory	Bronchiectasis	496.3	1882	375505
musculoskeletal	Intervertebral disc disorders	722	9241	391917
musculoskeletal	Osteoporosis, osteopenia and pathological fracture	743	7682	401279
neoplasms	Cancer of brain and nervous system	191.1	531	407239
digestive	Peritonitis and retroperitoneal infections	567	887	387338
hematopoietic	Iron deficiency anemias	280	7787	390026
circulatory system	Tachycardia NOS	427.7	2193	380919
musculoskeletal	Hammer toe (acquired)	735.21	1939	394914
neoplasms	Neoplasm of uncertain behavior	199	1412	370604
circulatory system	Heart valve disorders	395	4239	402421
pregnancy complications	Umbilical cord complications during labor and delivery	663	533	401037
digestive	Liver abscess and sequelae of chronic liver disease	571.8	942	400055
circulatory system	Congestive heart failure; nonhypertensive	428	5415	402834

neurological	Hemiplegia	342	1500	395209
musculoskeletal	Peripheral enthesopathies and allied syndromes	726	14983	378711
respiratory	Hemoptysis	516.1	2073	406794
digestive	Personal history of diseases of digestive system	564.9	15392	334783
musculoskeletal	Acquired toe deformities	735.2	5144	394914
circulatory system	Cardiomegaly	416	2573	402375
respiratory	Chronic tonsillitis and adenoiditis	474.2	1126	390045
musculoskeletal	Kyphoscoliosis and scoliosis	737.3	1063	394914
infectious diseases	Septicemia	38	4005	393897
endocrine/metabolic	Secondary hypothyroidism	244.1	1117	391429
congenital anomalies	Cardiac shunt/ heart septal defect	747.11	586	406165
injuries & poisonings	Fracture of ribs	807	775	387765
digestive	Abnormal results of function study of liver	573.7	3479	400055
sense organs	Visual disturbances	368	3307	405654
sense organs	Other disorders of eye	379	3910	401245
injuries & poisonings	Injury, NOS	1009	8440	400521
hematopoietic	Other diseases of blood and blood-forming organs	289	4177	401375
neurological	Abnormal involuntary movements	350.1	888	406217
digestive	Irritable Bowel Syndrome	564.1	5548	334783
injuries & poisonings	Complication of internal orthopedic device	858	3167	394929
endocrine/metabolic	Hypopotassemia	276.14	1430	401506
circulatory system	Congestive heart failure (CHF) NOS	428.1	2031	402834
musculoskeletal	Joint effusions	741.4	1425	402633
neoplasms	Malignant neoplasm of rectum, rectosigmoid junction, and anus	153.3	2095	382756
musculoskeletal	Pathologic fracture	743.2	514	401279
genitourinary	Postmenopausal bleeding	627.1	9109	377857
genitourinary	Prolapse of vaginal vault after hysterectomy	618.5	513	396730
musculoskeletal	Other and unspecified disc disorder	722.9	4434	391917
circulatory system	Cardiac pacemaker/device in situ	426.9	2487	380919
digestive	Acute periodontitis	523.31	668	398136
neoplasms	Benign neoplasm of ovary	220	1482	380325
circulatory system	Other venous embolism and thrombosis	452	558	369592
injuries & poisonings	Complication due to other implant and internal device	859	3265	394929
sense organs	Disorders of vitreous body	379.2	1372	401245
genitourinary	Benign mammary dysplasias	610	3379	401746
sense organs	Labyrinthitis	386.3	767	402827
sense organs	Dizziness and giddiness (Light-headedness and vertigo)	386.9	4611	402827
respiratory	Other symptoms of respiratory system	512	9128	399833
neoplasms	Vascular hamartomas and non-neoplastic nevi	217	649	400618
digestive	Gastritis and duodenitis	535	28941	378124
genitourinary	Calculus of lower urinary tract	594.2	778	401005
neoplasms	Benign neoplasm of brain and other parts of nervous system	225	828	407239
mental disorders	Delirium due to conditions classified elsewhere	290.2	654	402383
neurological	Disorders of other cranial nerves	352	1393	394067
digestive	Paralytic ileus	560.1	522	334783
genitourinary	Pyelonephritis	590	1324	379936
musculoskeletal	Osteomyelitis	710.1	565	365819
symptoms	Edema	782.3	1648	407145
injuries & poisonings	Anaphylactic shock NOS	946	548	404817
neoplasms	Multiple myeloma	204.4	552	404466
endocrine/metabolic	Hypovolemia	276.5	2834	401506
circulatory system	Occlusion of cerebral arteries	433.2	4134	399017
respiratory	Respiratory failure	509.1	2018	397411
respiratory	Respiratory abnormalities	513	611	408350
digestive	Ascites (non malignant)	572	1547	400055
genitourinary	Lump or mass in breast	611.3	1525	401746
injuries & poisonings	Mechanical complication of unspecified genitourinary device, implant, and	857	1241	394929
hematopoietic	Other deficiency anemia	281	1133	390026
respiratory	Pneumonitis due to inhalation of food or vomitus	501	586	397411
genitourinary	Mucous polyp of cervix	622.2	3450	396384
pregnancy complications	Fetal distress and abnormal forces of labor	661	2272	401037
musculoskeletal	Spondylosis without myelopathy	721.1	5077	391917
musculoskeletal	Rheumatism, unspecified and fibrositis	729.1	1010	378711
musculoskeletal	Malunion and nonunion of fracture	733.8	1268	391041
neoplasms	Cancer of larynx, pharynx, nasal cavities	149	628	406821
neurological	Degenerative disease of the spinal cord	334	1789	395209
symptoms	Pain	338	771	408190
respiratory	Bronchitis	497	631	375505
respiratory	Abnormal sputum	516	2167	406794
digestive	Peritoneal or intestinal adhesions	560.3	832	334783
digestive	Other disorders of liver	573	5847	400055
neoplasms	Secondary malignancy of bone	198.6	2151	370604
mental disorders	Alcoholism	317.1	8968	379355
genitourinary	Other disorders of urethra and urinary tract	597	4069	394699
musculoskeletal	Curvature of spine	737	1134	394914
musculoskeletal	Other derangement of joint	742.9	2000	402633
neoplasms	Neoplasm of unspecified nature of digestive system	158	1056	393372
genitourinary	Other signs and symptoms in breast	613.7	704	406978
genitourinary	Symptoms involving female genital tract	624	6688	399509
dermatologic	Carbuncle and furuncle	686.1	2302	397635
endocrine/metabolic	Crystal arthropathies	274.2	616	405198
mental disorders	Other mental disorder	306	28791	365476
circulatory system	Paroxysmal tachycardia, unspecified	427.1	3225	380919
symptoms	Neuralgia, neuritis, and radiculitis NOS	766	1181	405481
injuries & poisonings	Fracture of neck of femur	800.1	1814	387765
neoplasms	Malignant neoplasm of ovary	184.11	2103	389695
neoplasms	Lymphoid leukemia	204.1	578	404466
hematopoietic	Megaloblastic anemia	281.1	1076	390026
hematopoietic	Lymphadenitis	289.4	2622	401375
genitourinary	Abnormal findings on mammogram or breast exam	611	1580	401746

dermatologic	Disorders of sweat glands	705	817	399255
injuries & poisonings	Opiates and related narcotics causing adverse effects in therapeutic use	965.1	1150	381797
neurological	Sleep disorders	327	5238	403723
circulatory system	Hypertensive chronic kidney disease	401.22	1548	330366
circulatory system	Paroxysmal ventricular tachycardia	427.12	938	380919
pregnancy complications	Known or suspected fetal abnormality affecting management of mother	655	4325	404636
dermatologic	Decubitus ulcer	707.1	913	406973
musculoskeletal	Other disorders of synovium, tendon, and bursa	727	7629	378711
infectious diseases	Bacterial enteritis	8.5	2737	399970
neoplasms	Malignant neoplasm of uterus	182	1284	381967
mental disorders	Symptoms involving head and neck	293	2986	405975
genitourinary	Inflammatory diseases of female pelvic organs	614	6204	399757
pregnancy complications	Hypertension complicating pregnancy, childbirth, and the puerperium	642	1114	407847
dermatologic	Other local infections of skin and subcutaneous tissue	686	4520	397635
musculoskeletal	Other unspecified back disorders	724.9	1617	391917
injuries & poisonings	Contusion	916	1486	407475
infectious diseases	Gram negative septicemia	38.1	832	393897
symptoms	Other ill-defined and unknown causes of morbidity and mortality	1019	16398	392563
hematopoietic	Thrombocytopenia	287.3	1563	406281
genitourinary	Chronic glomerulonephritis, NOS	580.14	845	397602
pregnancy complications	Early onset of delivery	636.2	617	400946
infectious diseases	Streptococcus infection	41.2	1644	393897
infectious diseases	Viral hepatitis	70	1215	403316
circulatory system	Rheumatic disease of the heart valves	394	4895	402421
circulatory system	Pericarditis	420.2	1273	405779
circulatory system	Arterial embolism and thrombosis of lower extremity artery	444.1	557	400595
circulatory system	Hypotension	458	5827	387905
respiratory	Diseases of the larynx and vocal cords	473	2630	390045
digestive	Ulceration of the lower GI tract	556	1063	334783
neurological	Sleep apnea	327.3	4471	403723
sense organs	Otitis externa	380.1	524	407510
circulatory system	Arrhythmia (cardiac) NOS	427.5	922	380919
digestive	Ulcer of esophagus	530.12	5243	369275
digestive	Malposition and malpresentation of fetus or obstruction	652	1720	405994
musculoskeletal	Pain in joint	745	8037	400924
injuries & poisonings	Hemorrhage or hematoma complicating a procedure	850	5329	394929
genitourinary	Endometrial hyperplasia	621	1133	396384
neoplasms	Malignant neoplasm of ovary and other uterine adnexa	184.1	2127	389695
mental disorders	Anxiety disorder	300.1	6375	365476
circulatory system	Varicose veins of lower extremity, symptomtic	454.11	633	369592
respiratory	Respiratory failure, insufficiency, arrest	509	2565	397411
genitourinary	Hypertrophy of female genital organs	623	1275	396384
musculoskeletal	Osteomyelitis, periostitis, and other infections involving bone	710	612	365819
neurological	Other paralytic syndromes	344	660	395209
digestive	Other diseases of the teeth and supporting structures	525	2689	398136
pregnancy complications	Hemorrhage during pregnancy; childbirth and postpartum	635	2005	400946
hematopoietic	Pernicious anemia	281.11	754	390026
sense organs	Epiphora	375.2	899	401245
genitourinary	Noninflammatory disorders of vagina	619.4	1776	399629
injuries & poisonings	Complication of colostomy or enterostomy	853	502	394929
neoplasms	Benign neoplasm of lip, oral cavity, and pharynx	210	984	406821
sense organs	Retinal detachment with retinal defect	361.1	1392	397761
circulatory system	Abnormal heart sounds	396	1049	402421
circulatory system	Chronic pulmonary heart disease	415.2	590	402375
circulatory system	Left bundle branch block	426.32	1757	380919
genitourinary	Retention of urine	599.2	6755	384930
neoplasms	Secondary malignancy of lymph nodes	198.1	5379	370604
endocrine/metabolic	Disorders of fluid, electrolyte, and acid-base balance	276	7455	401506
circulatory system	Cardiac conduction disorders	426	6959	380919
circulatory system	Arterial embolism and thrombosis	444	921	400595
circulatory system	Orthostatic hypotension	458.1	1347	387905
respiratory	Allergic rhinitis	476	1060	390045
genitourinary	Other specified benign mammary dysplasias	610.8	696	401746
genitourinary	Inflammatory disease of cervix, vagina, and vulva	614.5	2864	399757
endocrine/metabolic	Hyposmolality and/or hyponatremia	276.12	1826	401506
sense organs	Disorders of external ear	380	1451	407510
circulatory system	Heart valve replaced	395.6	1499	402421
circulatory system	Nonspecific chest pain	418	31429	377532
genitourinary	Inflammatory disease of breast	613.1	723	406978
genitourinary	Irregular menstrual cycle	626.13	1989	377857
musculoskeletal	Other acquired musculoskeletal deformity	738	1921	394914
hematopoietic	Decreased white blood cell count	288.1	3184	401375
hematopoietic	Neutropenia	288.11	3184	401375
neurological	Other headache syndromes	339	7891	398780
circulatory system	Cardiac pacemaker in situ	426.91	2283	380919
circulatory system	Transient cerebral ischemia	433.31	2146	399017
respiratory	Acute tonsillitis	474.1	614	390045
respiratory	Other dyspnea	512.9	912	399833
digestive	Disorders of esophageal motility	530.5	639	369275
dermatologic	Cellulitis and abscess of fingers/toes	681.1	566	397635
musculoskeletal	Osteopenia or other disorder of bone and cartilage	743.9	820	401279
neurological	Convulsions	345.3	2232	395209
sense organs	Infection of the eye	369	560	399306
respiratory	Acute pharyngitis	465.2	870	406447
digestive	Chronic periodontitis	523.32	561	398136
digestive	Other disorders of peritoneum	568	3308	387338
symptoms	Symptoms involving skin and other integumentary tissue	782	1816	407145
symptoms	Malaise and fatigue	798	3429	405532
neoplasms	Secondary malignant neoplasm of liver	198.4	2638	370604
sense organs	Perforation of tympanic membrane	384.4	1044	404888

circulatory system	Carditis	420	2020	405779
injuries & poisonings	Open wounds of head; neck; and trunk	870	3837	400426
injuries & poisonings	Adverse effects of sedatives or other central nervous system depressants a	967	588	381797
neoplasms	Cancer of stomach	151	554	393372
neoplasms	Other benign neoplasm of connective and other soft tissue	215	1110	401613
neurological	Other conditions of brain	348	1426	395209
circulatory system	Aortic valve disease	394.3	1260	402421
digestive	Diseases of the salivary glands	527	710	403323
musculoskeletal	Other and unspecified disorders of back	724	2077	391917
symptoms	Gangrene	791	550	408411
infectious diseases	Postoperative infection	80	4489	402343
neoplasms	Secondary malignant neoplasm	198	9483	370604
neurological	Extrapyramidal disease and abnormal movement disorders	333	891	395209
circulatory system	Atrioventricular block, complete	426.24	587	380919
musculoskeletal	Bursitis	726.3	858	378711
injuries & poisonings	Fracture of vertebral column without mention of spinal cord injury	805	1352	387765
neoplasms	Malignant neoplasm of kidney, except pelvis	189.11	1002	404796
neoplasms	Secondary malignancy of brain/spine	198.5	806	370604
respiratory	Other diseases of respiratory system, NEC	519.8	8844	399525
digestive	Reflux esophagitis	530.14	10551	369275
symptoms	Back pain	760	11274	397687
symptoms	Symptoms of the muscles	772	698	408263
infectious diseases	Intestinal infection due to C. difficile	8.52	650	399970
neurological	Epilepsy, recurrent seizures, convulsions	345	5087	395209
neurological	Inflammatory and toxic neuropathy	357	1368	406852
digestive	Diseases of hard tissues of teeth	521	3091	398136
digestive	Flatulence	561.2	1689	334783
digestive	Gastrointestinal hemorrhage	578	21137	385157
pregnancy complications	Complications of labor and delivery NEC	669	2559	401037
injuries & poisonings	Skull and face fracture and other intercranial injury	819	2957	405554
circulatory system	Hypertensive heart and/or renal disease	401.2	1719	330366
genitourinary	Chronic renal failure [CKD]	585.3	2629	397602
musculoskeletal	Other disorders of bone and cartilage	733	3773	391041
symptoms	Chronic fatigue syndrome	798.1	593	405532
digestive	Peritoneal adhesions (postoperative) (postinfection)	568.1	3108	387338
mental disorders	Schizophrenia and other psychotic disorders	295	850	365476
sense organs	Suppurative and unspecified otitis media	381.11	856	404888
pregnancy complications	Problems associated with amniotic cavity and membranes	653	1404	405994
injuries & poisonings	Poisoning by analgesics, antipyretics, and antirheumatics	965	6910	381797
injuries & poisonings	Foreign body injury	1001	1210	407751
dermatologic	Disorder of skin and subcutaneous tissue NOS	689	5782	403179
endocrine/metabolic	Vitamin deficiency	261	1208	406492
sense organs	Diplopia and disorders of binocular vision	368.2	732	405654
sense organs	Disorders of conjunctiva	372	765	399306
musculoskeletal	Rupture of synovium	727.5	593	378711
neoplasms	Cervical cancer and dysplasia	180	3653	381902
endocrine/metabolic	Hyperpotassemia	276.13	975	401506
respiratory	Pulmonary collapse; interstitial and compensatory emphysema	508	2005	397411
genitourinary	Cystitis	592.1	2948	379936
injuries & poisonings	Poisoning by psychotropic agents	969	1940	381797
endocrine/metabolic	Chondrocalcinosis	274.21	560	405198
respiratory	Cough	512.8	2884	399833
musculoskeletal	Other specified osteoporosis	743.13	532	401279
injuries & poisonings	Open wounds of extremities	871	4219	400426
neoplasms	Large cell lymphoma	202.24	573	404466
circulatory system	Stricture of artery	447.1	873	400595
dermatologic	Pruritus and related conditions	698	783	408178
circulatory system	Nonspecific abnormal findings on radiological and other examination of o	793.2	567	408263
endocrine/metabolic	Anorexia	260.6	835	406492
endocrine/metabolic	Vitamin B-complex deficiencies	261.2	754	406492
sense organs	Vertiginous syndromes and other disorders of vestibular system	386	6134	402827
circulatory system	First degree AV block	426.21	988	380919
genitourinary	Dysmenorrhea	626.2	1787	377857
pregnancy complications	Hemorrhage in early pregnancy	636.3	1083	400946
circulatory system	Precordial pain	418.1	3582	377532
digestive	Intestinal obstruction without mention of hernia	560	3994	334783
digestive	Functional digestive disorders	564	22138	334783
genitourinary	Cystitis and urethritis	592	3088	379936
mental disorders	Schizophrenia	295.1	571	365476
sense organs	Disorders of lacrimal system	375	2218	401245
congenital anomalies	Digestive congenital anomalies	750	703	406730
symptoms	Nausea and vomiting	789	11706	397255
neurological	Migraine	340	2870	398780
circulatory system	Heart failure NOS	428.2	4269	402834
respiratory	Acute and chronic tonsillitis	474	2013	390045
genitourinary	Disorders of uterus, NEC	619.2	3336	399629
dermatologic	Scar conditions and fibrosis of skin	701.2	2356	403875
musculoskeletal	Spondylosis and allied disorders	721	7930	391917
genitourinary	Cyst of kidney, acquired	586.2	1260	397602
musculoskeletal	Acquired foot deformities	735	9865	394914
mental disorders	Anxiety disorders	300	6939	365476
sense organs	Blindness and low vision	367.9	723	406530
sense organs	Cholesteatoma	385.3	608	404888
digestive	Stricture and stenosis of esophagus	530.3	1874	369275
circulatory system	Primary/intrinsic cardiomyopathies	425.1	1208	405779
digestive	Periapical abscess	522.5	1177	398136
genitourinary	Noninflammatory disorders of cervix	619.3	2884	399629
injuries & poisonings	Fracture of foot	801.1	1842	387765
neoplasms	Cervical cancer	180.1	1659	381902
mental disorders	Agorophobia, social phobia, and panic disorder	300.12	709	365476

genitourinary	Glomerulonephritis	580.1	1033	397602
musculoskeletal	Stiffness of joint	741.2	615	402633
dermatologic	Hyperhidrosis	705.8	592	399255
circulatory system	Mitral valve disease	394.2	2985	402421
circulatory system	Premature beats	427.6	536	380919
dermatologic	Ingrowing nail	703.1	970	402357
musculoskeletal	Unspecified polyarthropathy or polyarthritis	716.1	3535	365819
musculoskeletal	Other disorders of cervical region	723	521	391917
sense organs	Strabismus (not specified as paralytic)	378.1	918	401245
respiratory	Voice disturbance	473.4	1086	390045
genitourinary	Acute renal failure	585.1	4521	397602
genitourinary	Cystic mastopathy	610.1	928	401746
neoplasms	Cervical intraepithelial neoplasia [CIN] [Cervical dysplasia]	180.3	2090	381902
endocrine/metabolic	Electrolyte imbalance	276.1	4123	401506
mental disorders	Memory loss	292.3	700	402383
digestive	Dental caries	521.1	3051	398136
digestive	Other specified gastritis	535.8	8147	378124
genitourinary	Infertility, female	626.8	1370	377857
musculoskeletal	Osteoarthrosis, localized, primary	740.11	9069	380522
neoplasms	Secondary malignant neoplasm of digestive systems	198.3	1519	370604
neurological	Parkinson's disease	332	1127	395209
digestive	Ulceration of intestine	556.1	674	334783
digestive	Other intestinal obstruction	560.4	3346	334783
pregnancy complications	Placenta previa and abruptio placenta	635.3	1274	400946
injuries & poisonings	Nonspecific abnormal findings on radiological and other examination of n	793	698	408263
circulatory system	Cardiac arrest and ventricular fibrillation	427.4	1137	380919
genitourinary	Renal colic	594.8	2055	401005
musculoskeletal	Other acquired deformities of limbs	736	1649	394914
endocrine/metabolic	Disorders of adrenal glands	255	642	405386
hematopoietic	Diseases of spleen	289.5	515	401375
sense organs	Hearing loss	389	4256	404562
genitourinary	Frequency of urination and polyuria	599.5	4037	384930
genitourinary	Other abnormality of urination	599.9	1926	384930
genitourinary	Pain and other symptoms associated with female genital organs	625	3003	399509
genitourinary	Other disorders of the kidney and ureters	586	3362	397602
injuries & poisonings	Traumatic arthropathy	836	528	391457
circulatory system	Nonrheumatic mitral valve disorders	395.1	2892	402421
genitourinary	Dyspareunia	625.1	1192	399509
dermatologic	Symptoms affecting skin	687	5554	403407
circulatory system	Atrioventricular [AV] block	426.2	2125	380919
digestive	Gingival and periodontal diseases	523	1742	398136
symptoms	Myalgia and myositis unspecified	770	642	408319
dermatologic	Diseases of nail, NOS	703	1287	402357
circulatory system	Paroxysmal supraventricular tachycardia	427.11	2359	380919
circulatory system	Palpitations	427.9	3832	380919
respiratory	Shortness of breath	512.7	5884	399833
dermatologic	Pilonidal cyst	686.3	587	397635
musculoskeletal	Derangement of joint, non-traumatic	742	2913	402633
neoplasms	Benign neoplasm of brain, cranial nerves, meninges	225.1	774	407239
injuries & poisonings	Injuries to the nervous system	907	1315	407496
infectious diseases	Sepsis and SIRS	994	2811	406150
infectious diseases	Sepsis	994.2	2811	406150
injuries & poisonings	Complications of surgical and medical procedures	1011	9140	399821
hematopoietic	Purpura and other hemorrhagic conditions	287	1791	406281
circulatory system	Noninfectious disorders of lymphatic channels	450	705	408256
respiratory	Other diseases of respiratory system, not elsewhere classified	519	9436	399525
digestive	Diseases of the oral soft tissues, excluding lesions specific for gingiva and	528	3939	403323
congenital anomalies	Other congenital musculoskeletal anomalies	756	594	407831
endocrine/metabolic	Protein-calorie malnutrition	260	1057	406492
sense organs	Amblyopia	368.1	538	405654
sense organs	Strabismus and other disorders of binocular eye movements	378	1442	401245
digestive	Constipation	563	10442	334783
dermatologic	Other hypertrophic and atrophic conditions of skin	701	4804	403875
neoplasms	Cancer of kidney and renal pelvis	189.1	1045	404796
neurological	Facial nerve disorders [CN7]	352.2	880	394067
circulatory system	Aneurysm and dissection of heart	411.41	698	377103
circulatory system	Cardiomyopathy	425	1247	405779
digestive	Nonspecific abnormal findings in stool contents	579.8	1659	385157
infectious diseases	Intestinal infection	8	8991	399970
circulatory system	Vascular insufficiency of intestine	441	576	400595
genitourinary	Noninflammatory disorders of vulva and perineum	619.5	1355	399629
circulatory system	Intracerebral hemorrhage	430.2	700	399017
injuries & poisonings	Fracture of clavicle or scapula	803.3	1521	387765
injuries & poisonings	Fracture of unspecified bones	809	1304	387765
neurological	Abnormality of gait	350.2	1601	406217
circulatory system	Late effects of cerebrovascular disease	433.8	1263	399017
respiratory	Other upper respiratory disease	479	4101	390045
musculoskeletal	Synovitis and tenosynovitis	727.1	2754	378711
infectious diseases	Staphylococcus infections	41.1	3149	393897
genitourinary	Dysuria	599.3	1199	384930
neoplasms	Benign neoplasm of other endocrine glands and related structures	227	876	407399
dermatologic	Rash and other nonspecific skin eruption	687.1	2157	403407
digestive	Periodontitis (acute or chronic)	523.3	1222	398136
injuries & poisonings	Fracture of tibia and fibula	800.3	2162	387765
injuries & poisonings	Complications of cardiac/vascular device, implant, and graft	854	1789	394929
genitourinary	Nephritis; nephrosis; renal sclerosis	580	1522	397602
sense organs	Ectropion or entropion	374.1	1068	399306
circulatory system	Disease of tricuspid valve	394.7	1058	402421
digestive	Dysphagia	532	6482	369275
genitourinary	Vaginal enterocele, congenital or acquired	618.6	681	396730

musculoskeletal	Symptoms and disorders of the joints	741	3634	402633
circulatory system	Other acute and subacute forms of ischemic heart disease	411.9	1169	377103
digestive	Diseases of the jaws	526	960	398136
genitourinary	Functional disorders of bladder	596.5	1664	394699
injuries & poisonings	Burns	1000	579	408382
sense organs	Meniere's disease	386.1	578	402827
digestive	GERD	530.11	14223	369275
symptoms	Effects of other external causes	1015	4230	404731
circulatory system	Cerebral artery occlusion, with cerebral infarction	433.21	1501	399017
infectious diseases	Viral Enteritis	8.6	862	399970
circulatory system	Other forms of chronic heart disease	414	1796	377103
dermatologic	Dyschromia and Vitiligo	694	987	402672
endocrine/metabolic	Acidosis	276.41	1055	401506
genitourinary	Renal failure	585	6985	397602
pregnancy complications	Missed abortion/Hydatidiform mole	634.1	1251	400946
injuries & poisonings	Fracture of ankle and foot	801	2339	387765
digestive	Other disorders of stomach and duodenum	537	3404	378124
genitourinary	Pelvic peritoneal adhesions, female (postoperative) (postinfection)	614.1	2546	399757
infectious diseases	E. coli	41.4	2744	393897
circulatory system	Bundle branch block	426.3	3353	380919
dermatologic	Cellulitis and abscess of trunk	681.7	602	397635
symptoms	Symptoms involving nervous and musculoskeletal systems	781	1886	407075
injuries & poisonings	Other open wound of head and face	870.3	2919	400426
symptoms	Symptoms concerning nutrition, metabolism, and development	1002	5512	403449
neurological	Epilepsy	345.1	901	395209
circulatory system	Raynaud's syndrome	443.1	1148	400595
injuries & poisonings	Poisoning by other anti-infectives	961	1256	381797
neoplasms	Malignant neoplasm, other	195.1	15979	370604
hematopoietic	Anemia of chronic disease	285.2	702	390026
symptoms	Other symptoms	1005	1222	407739
digestive	Hemorrhage of rectum and anus	578.8	13222	385157
circulatory system	Other specified cardiac dysrhythmias	427.3	3236	380919
respiratory	Chronic pharyngitis and nasopharyngitis	472	966	390045
respiratory	Other diseases of lung	510	781	408180
sense organs	Other disorders of middle ear and mastoid	385	828	404888
musculoskeletal	Degeneration of intervertebral disc	722.6	2846	391917
hematopoietic	Diseases of white blood cells	288	3788	401375
respiratory	Bacterial pneumonia	480.1	6710	398538
injuries & poisonings	Adverse drug events and drug allergies	979	695	381797
pregnancy complications	Antepartum hemorrhage, abruptio placentae, and placenta previa	635.2	866	400946
symptoms	Fever of unknown origin	783	3940	405021
respiratory	Septal Deviations/Turbinate Hypertrophy	470	4939	390045
sense organs	Disorders of refraction and accommodation; blindness and low vision	367	2431	406530
circulatory system	Elevated blood pressure reading without diagnosis of hypertension	402	1487	330366
respiratory	Acute upper respiratory infections of multiple or unspecified sites	465	2335	406447
digestive	Other symptoms involving abdomen and pelvis	579	3195	385157
genitourinary	Noninflammatory disorders of ovary, fallopian tube, and broad ligament	619.1	730	399629
mental disorders	Bipolar	296.1	1064	365476
sense organs	Primary angle-closure glaucoma	365.2	705	397761
sense organs	Ptosis of eyelid	374.3	1834	399306
circulatory system	Endocarditis	420.3	686	405779
circulatory system	Intracranial hemorrhage	430	1796	399017
respiratory	Abnormal findings examination of lungs	514	2500	406461
digestive	Diseases and other conditions of the tongue	529	1236	403323
genitourinary	Renal failure NOS	585.2	1412	397602
injuries & poisonings	Fracture of patella	800.4	768	387765
neoplasms	Cancer of esophagus	150	720	393372
neoplasms	Leukemia	204	1661	404466
mental disorders	Other specified nonpsychotic and/or transient mental disorders	291	641	402383
neurological	Nerve root and plexus disorders	353	1139	394067
sense organs	Tinnitus	389.4	515	404562
circulatory system	Cardiac arrest	427.42	927	380919
circulatory system	Ill-defined descriptions and complications of heart disease	429	796	402834
circulatory system	Subarachnoid hemorrhage	430.1	812	399017
digestive	Disturbances in tooth eruption	520.2	2364	398136
digestive	Abnormal findings on exam of gastrointestinal tract/ abdominal area	564.8	1650	334783
genitourinary	Hypertrophy of breast (Gynecomastia)	612.2	854	401746
genitourinary	Irregular menstrual bleeding	626.14	3817	377857
genitourinary	Ovarian cyst	628	4777	377857
dermatologic	Chronic ulcer of skin	707	1988	406973
musculoskeletal	Displacement of intervertebral disc	722.1	513	391917
musculoskeletal	Hallux rigidus	735.23	1561	394914
symptoms	Sciatica	764	2383	405481
injuries & poisonings	Intracranial hemorrhage (injury)	818	508	405554
injuries & poisonings	Poisoning by anticonvulsants and anti-Parkinsonism drugs	966	509	381797
circulatory system	Aortic aneurysm	442.1	1374	400595
respiratory	Respiratory insufficiency	509.2	1749	397411
injuries & poisonings	Poisoning/allergy of sulfonamides	961.1	874	381797
injuries & poisonings	Crushing or internal injury to organs	1008	1007	407954
neoplasms	Hemangioma and lymphangioma, any site	228	1603	407358
endocrine/metabolic	Acid-base balance disorder	276.4	1161	401506
digestive	Disorders of tooth development	520	2449	398136
digestive	Esophageal bleeding (varices/hemorrhage)	530.2	1672	369275
digestive	Ulcerative colitis (chronic)	555.21	539	334783
pregnancy complications	Obstetrical/birth trauma	665	5335	401037
dermatologic	Other dyschromia	694.2	783	402672
musculoskeletal	Osteoarthrosis, generalized	740.2	599	380522
injuries & poisonings	Fracture of humerus	803.1	1692	387765
endocrine/metabolic	Disorders of the pituitary gland and its hypothalamic control	253	693	405386
circulatory system	Cerebral ischemia	433.3	2920	399017

circulatory system	Hypotension NOS	458.9	3518	387905
digestive	Diseases of lips	528.5	684	403323
genitourinary	Urethral stricture (not specified as infectious)	597.1	3331	394699
congenital anomalies	Genitourinary congenital anomalies	751	1596	406730
symptoms	Cervicalgia	761	1796	407165
neoplasms	Pancreatic cancer	157	589	393372
neoplasms	Nevus, non-neoplastic	217.1	597	400618
respiratory	Pneumococcal pneumonia	480.11	5951	398538
digestive	Acute gastritis	535.1	1184	378124
pregnancy complications	Late pregnancy and failed induction	645	1256	407705
infectious diseases	Viral infection	79	2806	403316
infectious diseases	Candidiasis	112	2134	406301
neoplasms	Secondary malignancy of respiratory organs	198.2	2211	370604
respiratory	Pneumonia	480	10059	398538
genitourinary	Cervicitis and endocervicitis	614.51	1323	399757
pregnancy complications	Other complications of pregnancy NEC	646	2235	406726
musculoskeletal	Spinal stenosis	720	3733	391917
mental disorders	Swelling, mass, or lump in head and neck [Space-occupying lesion, intrac	293.1	907	405975
sense organs	Other disorders of eyelids	374	5726	399306
digestive	Chronic pancreatitis	577.2	514	406271
genitourinary	Urinary tract infection	591	12491	379936
genitourinary	Breast conditions, congenital or relating to hormones	612	942	401746
musculoskeletal	Aseptic necrosis of bone	733.4	542	391041
mental disorders	Phobia	300.13	503	365476
respiratory	Empyema and pneumothorax	506	1174	397411
digestive	Hemorrhage from gastrointestinal ulcer	531.1	617	401525
dermatologic	Disturbance of skin sensation	687.4	2900	403407
digestive	Blood in stool	578.2	2639	385157
genitourinary	Stricture/obstruction of ureter	586.4	913	397602
genitourinary	Abnormal findings on examination of urine	598	3547	405414
genitourinary	Noninflammatory female genital disorders	619	9332	399629
pregnancy complications	Miscarriage; stillbirth	634	5463	400946
neurological	Abnormal movement	350	2744	406217
circulatory system	Abnormal function study of cardiovascular system	429.2	524	402834
respiratory	Epistaxis or throat hemorrhage	477	2456	390045
genitourinary	Other disorders of male genital organs	608	2833	389094
pregnancy complications	Normal delivery	650	1928	407033
neoplasms	Cancer, suspected or other	195	16725	370604
sense organs	Retinal vascular changes and abnomalities	362.4	849	396859
genitourinary	Other symptoms/disorders or the urinary system	599	24031	384930
neoplasms	Chemotherapy	197	21798	370604
injuries & poisonings	Effects radiation NOS	990	3130	403295
genitourinary	Hydronephrosis	595	1951	401005
genitourinary	Menopausal and postmenopausal disorders	627	10699	377857
endocrine/metabolic	Disorders of calcium/phosphorus metabolism	275.5	1204	406834
mental disorders	Aphasia/speech disturbance	292.1	1514	402383
sense organs	Subjective visual disturbances	368.9	615	405654
digestive	Other disorders of intestine	569	4200	387338
digestive	Hematemesis	578.1	1961	385157
congenital anomalies	Congenital anomalies of urinary system	751.2	855	406730
digestive	Heartburn	530.9	2100	369275
digestive	Hemorrhage of gastrointestinal tract	578.9	5229	385157
infectious diseases	Infection/inflammation of internal prosthetic device; implant; and graft	81	2485	402343
musculoskeletal	Acquired spondylolisthesis	738.4	1521	394914
dermatologic	Cellulitis and abscess of face/neck	681.2	524	397635
neoplasms	Cancer of other female genital organs	184	2463	389695
genitourinary	Cyst or abscess of Bartholin's gland	614.53	758	399757
neoplasms	Cancer of mouth	145	643	406821
neurological	Other cerebral degenerations	331	1112	395209
digestive	Symptoms involving digestive system	561	15977	334783
circulatory system	Right bundle branch block	426.31	1498	380919
symptoms	Abdominal pain	785	41316	367645
congenital anomalies	Congenital anomalies of genital organs	751.1	732	406730
injuries & poisonings	Torus fracture	823	1674	385157
infectious diseases	Bacterial infection NOS	41	12187	393897
respiratory	Pleurisy; pleural effusion	507	6448	397411

Table S12. The phenome-wide MR analysis results of CALCRL (IVW or Wald ratio methods).

Category	Phenostring	Method	Nsnp	beta	se	P value	OR	LL	LR	FDR
genitourinary	Benign neoplasm of breast	Inverse var	3	-0.79743	0.185281	1.6785E-05	0.450487	0.313306	0.647735	0.013142
mental disorders	Symptoms involving head and neck	Inverse var	3	0.500264	0.130595	0.0001278	1.649157	1.276725	2.13023	0.050034
genitourinary	Urinary calculus	Inverse var	3	-0.27945	0.085879	0.00113786	0.756198	0.639048	0.894823	0.296982
genitourinary	Excessive or frequent menstruation	Inverse var	3	-0.26857	0.092473	0.00368101	0.764473	0.637745	0.916383	0.349705
genitourinary	Other disorders of bladder	Inverse var	3	0.208397	0.072172	0.00388318	1.231703	1.069231	1.418862	0.349705
genitourinary	Benign mammary dysplasias	Inverse var	3	-0.3698	0.12904	0.00416035	0.690875	0.536486	0.889695	0.349705
endocrine/metabolic	Hyperlipidemia	Inverse var	3	0.118598	0.041501	0.00426752	1.125917	1.037958	1.22133	0.349705
digestive	Acute gastritis	Inverse var	3	0.571848	0.203372	0.00492599	1.771537	1.18915	2.639148	0.349705
genitourinary	Endometrial hyperplasia	Inverse var	3	-0.58198	0.207731	0.0050852	0.558793	0.371901	0.839605	0.349705
neurological	Hemiplegia	Inverse var	3	0.498927	0.180382	0.00567573	1.646954	1.156478	2.345447	0.349705
endocrine/metabolic	Disorders of lipoid metabolism	Inverse var	3	0.114473	0.041501	0.00581038	1.121283	1.033685	1.216303	0.349705
genitourinary	Calculus of ureter	Inverse var	3	-0.39257	0.144313	0.00652326	0.675321	0.508943	0.896091	0.349705
endocrine/metabolic	Hypercholesterolemia	Inverse var	3	0.11492	0.042809	0.00726409	1.121784	1.031501	1.21997	0.349705
circulatory system	Essential hypertension	Inverse var	3	0.118554	0.044196	0.00730818	1.125867	1.032445	1.227743	0.349705
genitourinary	Irregular menstrual cycle/bleeding	Inverse var	3	-0.15627	0.058691	0.00775481	0.855329	0.762384	0.959604	0.349705
circulatory system	Other acute and subacute forms of ischemic heart disease	Inverse var	3	0.548165	0.207629	0.00828782	1.730076	1.151668	2.59898	0.349705
symptoms	Syncope and collapse	Inverse var	3	0.222475	0.084843	0.00873674	1.249165	1.05779	1.475162	0.349705
neoplasms	Benign neoplasm of brain, cranial nerves, meninges	Inverse var	3	0.659158	0.252033	0.00891321	1.933163	1.179598	3.168131	0.349705
genitourinary	Disorders of menstruation and other abnormal bleeding from female genital tract	Inverse var	3	-0.15132	0.057936	0.00900629	0.859573	0.767302	0.96294	0.349705
circulatory system	Phlebitis and thrombophlebitis of lower extremities	Inverse var	3	0.305933	0.117383	0.00915311	1.357891	1.078814	1.709161	0.349705
congenital anomalies	Congenital anomalies of urinary system	Inverse var	3	-0.61897	0.238961	0.00958992	0.538497	0.337113	0.860182	0.349705
endocrine/metabolic	Disorders of mineral metabolism	Inverse var	3	0.392768	0.153322	0.01041552	1.481074	1.096646	2.000263	0.349705
genitourinary	Other signs and symptoms in breast	Inverse var	3	-0.74995	0.294852	0.01097523	0.47239	0.265044	0.841944	0.349705
circulatory system	Hypertension	Inverse var	3	0.117534	0.04638	0.01127118	1.12472	1.026988	1.231753	0.349705
circulatory system	Myocardial infarction	Inverse var	3	0.171114	0.067701	0.01148816	1.186626	1.039167	1.355009	0.349705
injuries & poisonings	Allergy/adverse effect of penicillin	Inverse var	3	-0.14765	0.058523	0.01163906	0.862735	0.769239	0.967594	0.349705
dermatologic	Sicca syndrome	Inverse var	3	-0.77752	0.31084	0.01237219	0.459545	0.249882	0.845124	0.349705
mental disorders	Swelling, mass, or lump in head and neck [Space-occupying lesion, intracranial NOS]	Inverse var	3	0.58389	0.233785	0.0125054	1.793	1.13391	2.835189	0.349705
digestive	Gastrointestinal hemorrhage	Inverse var	3	-0.12222	0.049514	0.01357188	0.884953	0.803107	0.975141	0.362204
digestive	Ulcerative colitis (chronic)	Inverse var	3	0.744426	0.302561	0.01387755	2.105233	1.16347	3.809301	0.362204
sense organs	Retinal detachments and defects	Inverse var	3	-0.33979	0.140921	0.0158986	0.711918	0.540102	0.938391	0.399607
musculoskeletal	Spondylosis and allied disorders	Inverse var	3	0.194708	0.08108	0.01633131	1.214956	1.036439	1.424221	0.399607
symptoms	Nonspecific findings on examination of blood	Inverse var	3	0.212133	0.090019	0.01844621	1.236312	1.036341	1.474871	0.43163
circulatory system	Cerebrovascular disease	Inverse var	3	0.179968	0.076711	0.01897279	1.197179	1.030058	1.391414	0.43163
circulatory system	Circulatory disease NEC	Inverse var	3	0.136167	0.058523	0.0199797	1.145874	1.021694	1.285146	0.43163
neoplasms	Benign neoplasm of other parts of digestive system	Inverse var	3	-0.22831	0.099098	0.02122862	0.795877	0.655378	0.966495	0.43163
symptoms	Pain in limb	Inverse var	3	0.207661	0.090191	0.02130914	1.230796	1.031369	1.468785	0.43163
circulatory system	Varicose veins of lower extremity	Inverse var	3	-0.1558	0.067701	0.0213719	0.855727	0.749388	0.977155	0.43163
circulatory system	Varicose veins	Inverse var	3	-0.15443	0.067532	0.0222103	0.856904	0.750668	0.978175	0.43163
mental disorders	Tobacco use disorder	Inverse var	3	0.122777	0.054053	0.0231225	1.130633	1.016975	1.256993	0.43163
neoplasms	Malignant neoplasm of rectum, rectosigmoid junction, and anus	Inverse var	3	0.391879	0.1736	0.02398518	1.479759	1.052979	2.079516	0.43163
circulatory system	Other disorders of circulatory system	Inverse var	3	0.132	0.058523	0.02410034	1.141109	1.017446	1.279802	0.43163
musculoskeletal	Spondylosis without myelopathy	Inverse var	3	0.225522	0.100045	0.02418296	1.252977	1.029872	1.524415	0.43163
symptoms	Other abnormal blood chemistry	Inverse var	3	0.203206	0.090191	0.02425507	1.225325	1.026784	1.462256	0.43163
genitourinary	Other disorders of the kidney and ureters	Inverse var	3	0.273098	0.121757	0.02489815	1.314029	1.035055	1.668194	0.433228
injuries & poisonings	Torus fracture	Inverse var	3	-0.37762	0.17144	0.02762156	0.685493	0.489858	0.959258	0.454905
digestive	Other biliary tract disease	Inverse var	3	0.247347	0.112813	0.02834059	1.280623	1.026581	1.597532	0.454905
digestive	Hemorrhage of gastrointestinal tract	Inverse var	3	-0.21703	0.099028	0.02841012	0.804908	0.662906	0.977328	0.454905
genitourinary	Noninflammatory disorders of vagina	Inverse var	3	-0.36546	0.167032	0.02867448	0.69388	0.500154	0.962643	0.454905
injuries & poisonings	Fracture of tibia and fibula	Inverse var	3	-0.33235	0.153255	0.03011242	0.717236	0.53114	0.968535	0.454905
endocrine/metabolic	Electrolyte imbalance	Inverse var	3	0.295703	0.136576	0.03037827	1.344071	1.028411	1.756621	0.454905
digestive	Diverticulosis	Inverse var	3	-0.12055	0.055793	0.03071727	0.88643	0.794607	0.988864	0.454905
injuries & poisonings	Poisoning by antibiotics	Inverse var	3	-0.11653	0.054053	0.0311034	0.890007	0.800539	0.989475	0.454905
digestive	Diverticulosis and diverticulitis	Inverse var	3	-0.1211	0.05637	0.03168628	0.885944	0.793274	0.989439	0.454905
neoplasms	Benign neoplasm of brain and other parts of nervous system	Inverse var	3	0.522884	0.24377	0.0319537	1.686886	1.046127	2.720112	0.454905
injuries & poisonings	Adverse drug events and drug allergies	Inverse var	3	-0.56277	0.266135	0.03446334	0.569628	0.338105	0.959688	0.481871
neoplasms	Malignant neoplasm of female breast	Inverse var	3	-0.1402	0.068821	0.04163055	0.869184	0.759504	0.994702	0.563156
digestive	Inguinal hernia	Inverse var	3	-0.11952	0.058691	0.04171526	0.88735	0.790926	0.995529	0.563156
endocrine/metabolic	Abnormal glucose	Inverse var	3	0.537412	0.266135	0.0434542	1.711571	1.015912	2.883593	0.576689
circulatory system	Nonspecific chest pain	Inverse var	3	0.085344	0.042826	0.04628065	1.089092	1.001407	1.184455	0.595139
circulatory system	Other disorders of arteries and arterioles	Inverse var	3	0.475496	0.238696	0.04636457	1.608812	1.007682	2.568544	0.595139
sense organs	Amblyopia	Inverse var	3	-0.59994	0.302561	0.04738031	0.548843	0.303321	0.9931	0.598367
digestive	Constipation	Inverse var	3	0.142249	0.072001	0.04819363	1.152863	1.001128	1.327596	0.598978
circulatory system	Phlebitis and thrombophlebitis	Inverse var	3	0.218435	0.112813	0.05283629	1.244128	0.997325	1.552006	0.646419
neoplasms	Breast cancer	Inverse var	3	-0.14977	0.07818	0.05539542	0.860902	0.738594	1.003465	0.657274
mental disorders	Agorophobia, social phobia, and panic disorder	Inverse var	3	-0.50868	0.266135	0.0559601	0.601291	0.356899	1.013033	0.657274
injuries & poisonings	Complication of internal orthopedic device	Inverse var	3	0.284114	0.149538	0.05744028	1.328584	0.99106	1.781059	0.657274
genitourinary	Bladder neck obstruction	Inverse var	3	0.297627	0.157959	0.05953687	1.346659	0.988099	1.835333	0.657274
respiratory	Other upper respiratory disease	Inverse var	3	0.204182	0.108373	0.05955549	1.226522	0.991805	1.516785	0.657274
neoplasms	Malignant neoplasm of other and ill-defined sites within the digestive organs and peritoneum	Inverse var	3	0.177857	0.094729	0.0604456	1.194654	0.992218	1.438393	0.657274
musculoskeletal	Spinal stenosis	Inverse var	3	0.219258	0.117116	0.06118588	1.245153	0.989764	1.56644	0.657274
genitourinary	Pelvic peritoneal adhesions, female (postoperative) (postinfection)	Inverse var	3	-0.2612	0.139775	0.06166138	0.770126	0.585576	1.01284	0.657274
musculoskeletal	Bursitis	Inverse var	3	-0.44615	0.238961	0.06189737	0.64009	0.400713	1.022465	0.657274
circulatory system	Chronic pulmonary heart disease	Inverse var	3	-0.53695	0.288492	0.06271088	0.584528	0.332075	1.028902	0.657274
endocrine/metabolic	Type 2 diabetes with ophthalmic manifestations	Inverse var	3	0.360855	0.194061	0.06295733	1.434555	0.980684	2.098483	0.657274
digestive	Femoral hernia	Inverse var	3	-0.50689	0.274382	0.06469259	0.602368	0.351806	1.031384	0.659759
sense organs	Other disorders of middle ear and mastoid	Inverse var	3	0.449754	0.24377	0.06503859	1.567926	0.972354	2.52829	0.659759
neoplasms	Breast cancer [female]	Inverse var	3	-0.15227	0.082742	0.06572308	0.858756	0.730194	1.009953	0.659759
neurological	Inflammatory and toxic neuropathy	Inverse var	3	-0.34697	0.189711	0.06740845	0.706827	0.487336	1.025175	0.661044
genitourinary	Renal colic	Inverse var	3	-0.28814	0.157622	0.06753961	0.749655	0.550416	1.021013	0.661044
genitourinary	Other nonmalignant breast conditions	Inverse var	3	-0.28541	0.157959	0.07078561	0.751708	0.551559	1.024486	0.680802
circulatory system	Cardiac dysrhythmias	Inverse var	3	0.088892	0.049401	0.07195719	1.092963	0.992097	1.204084	0.680802
digestive	Diseases and other conditions of the tongue	Inverse var	3	0.356455	0.1984	0.07239173	1.428258	0.96811	2.107116	0.680802
dermatologic	Other dyschromia	Inverse var	3	0.450332	0.252033	0.07396972	1.568833	0.957287	2.571056	0.680802
endocrine/metabolic	Diabetic retinopathy	Inverse var	3	0.345108	0.19396	0.07519522	1.412142	0.965553	2.065289	0.680802
circulatory system	Bundle branch block	Inverse var	3	0.21603	0.121757	0.07601754	1.24114	0.977641	1.575659	0.680802
digestive	Personal history of diseases of digestive system	Inverse var	3	0.103348	0.058591	0.0777526	1.108877	0.988575	1.243819	0.680802
circulatory system	Other venous embolism and thrombosis	Inverse var	3	0.52363	0.297403	0.07829375	1.688145	0.942442	3.023881	0.680802
genitourinary	Ovarian cyst	Inverse var	3	-0.18153	0.103739	0.08014381	0.833996	0.68055	1.02204	0.680802
digestive	Regional enteritis	Inverse var	3	0.375316	0.214856	0.08066787	1.455451	0.955231	2.217619	0.680802
sense organs	Visual disturbances	Inverse var	3	-0.21103	0.12192	0.08346971	0.809749	0.637631	1.028326	0.680802
sense organs	Disorders of lacrimal system	Inverse var	3	-0.25635	0.148949	0.08524261	0.773872	0.577938	1.036233	0.680802
endocrine/metabolic	Disorders of calcium/phosphorus metabolism	Inverse var	3	0.348621	0.202628	0.08534226	1.417112	0.952629	2.108069	0.680802
dermatologic	Cellulitis and abscess of fingers/toes	Inverse var	3	-0.50409	0.293268	0.08563555	0.604053	0.33997	1.073272	0.680802
genitourinary	Nephritis; nephrosis; renal sclerosis	Inverse var	3	0.308984	0.180382	0.08672269	1.362041	0.956414	1.939699	0.680802
endocrine/metabolic	Hypopotassemia	Inverse var	3	0.451965	0.263895	0.08677393	1.571397	0.936814	2.635835	0.680802
endocrine/metabolic	Type 2 diabetes with neurological manifestations	Inverse var	3	0.498705	0.292615	0.08832487	1.646587	0.927909	2.921891	0.680802
neoplasms	Cancer of brain and nervous system	Inverse var	3	-0.52157	0.306708	0.08902839	0.593587	0.325394	1.082829	0.680802

digestive	Cholelithiasis with other cholecystitis	Inverse var	3	0.160978	0.09489	0.08979711	1.174659	0.975304	1.414762	0.680802
respiratory	Bacterial pneumonia	Inverse var	3	0.145281	0.085784	0.09034909	1.156364	0.977403	1.368093	0.680802
endocrine/metabolic	Simple and unspecified goiter	Inverse var	3	-0.48034	0.284351	0.0911751	0.618576	0.354281	1.080035	0.680802
pregnancy complications	Placenta previa and abruptio placenta	Inverse var	3	0.489108	0.289779	0.09143669	1.63086	0.924171	2.877937	0.680802
respiratory	Pneumonia	Inverse var	3	0.121459	0.072071	0.09193745	1.129143	0.980394	1.300461	0.680802
genitourinary	Dysmenorrhea	Inverse var	3	-0.28102	0.166968	0.09236348	0.755015	0.544289	1.047326	0.680802
genitourinary	Calculus of kidney	Inverse var	3	-0.21222	0.126228	0.09271683	0.808788	0.631521	1.035814	0.680802
sense organs	Meniere's disease	Inverse var	3	-0.49154	0.292615	0.09299553	0.611686	0.344706	1.085445	0.680802
genitourinary	Chronic cystitis	Inverse var	3	-0.3944	0.234816	0.09303421	0.674085	0.425437	1.068057	0.680802
neoplasms	Chemotherapy	Inverse var	3	0.082874	0.049514	0.09418205	1.086404	0.985926	1.197123	0.68282
genitourinary	Abnormal findings on examination of urine	Inverse var	3	0.194806	0.117285	0.09672152	1.215075	0.965535	1.529109	0.694798
circulatory system	Orthostatic hypotension	Inverse var	3	0.31784	0.19396	0.10127839	1.374157	0.93958	2.009734	0.714634
digestive	Diseases of pancreas	Inverse var	3	0.221862	0.135402	0.10130833	1.2484	0.957408	1.627834	0.714634
digestive	Other diseases of the teeth and supporting structures	Inverse var	3	0.2209	0.135237	0.10237736	1.247199	0.956798	1.625742	0.715727
circulatory system	Abnormal heart sounds	Inverse var	3	0.506028	0.312372	0.10524171	1.65869	0.899227	3.059577	0.7247
digestive	Noninfectious gastroenteritis	Inverse var	3	0.149278	0.092221	0.10551187	1.160996	0.969015	1.391011	0.7247
circulatory system	Cardiac conduction disorders	Inverse var	3	0.143971	0.089482	0.10762776	1.154851	0.969076	1.37624	0.732805
digestive	Chronic pancreatitis	Inverse var	3	0.61175	0.381729	0.10902855	1.843655	0.872459	3.895957	0.735943
circulatory system	Pulmonary heart disease	Inverse var	3	0.171698	0.108276	0.11279785	1.187319	0.960287	1.468026	0.747455
infectious diseases	Viral infection	Inverse var	3	0.207375	0.13093	0.11322539	1.230444	0.951945	1.590419	0.747455
genitourinary	Postmenopausal bleeding	Inverse var	3	0.183014	0.115867	0.11421696	1.200831	0.956872	1.506989	0.747455
circulatory system	Ischemic Heart Disease	Inverse var	3	0.125436	0.079488	0.1145525	1.133643	0.970096	1.324761	0.747455
injuries & poisonings	Fracture of humerus	Inverse var	3	0.315636	0.201213	0.11672638	1.37113	0.924278	2.034018	0.755345
endocrine/metabolic	Other disorders of pancreatic internal secretion	Inverse var	3	0.358437	0.229675	0.11861128	1.431092	0.912356	2.244764	0.757765
sense organs	Disorders of conjunctiva	Inverse var	3	-0.45977	0.295349	0.1195431	0.63143	0.353931	1.126501	0.757765
neoplasms	Manlignant and unknown neoplasms of brain and nervous system	Inverse var	3	-0.42556	0.274382	0.12090855	0.653405	0.381614	1.11877	0.757765
digestive	Hemorrhage of rectum and anus	Inverse var	3	-0.11529	0.074349	0.12097147	0.891105	0.770268	1.030898	0.757765
endocrine/metabolic	Hypoglycemia	Inverse var	3	0.354211	0.229675	0.12301787	1.425057	0.908508	2.235298	0.764468
circulatory system	Late effects of cerebrovascular disease	Inverse var	3	0.299769	0.1983	0.13061132	1.349547	0.914938	1.990601	0.805265
digestive	Calculus of bile duct	Inverse var	3	0.207542	0.139604	0.13710791	1.230649	0.936054	1.617958	0.838715
neoplasms	Neoplasm of uncertain behavior	Inverse var	3	-0.27322	0.18471	0.13909698	0.760929	0.529805	1.092879	0.838983
neoplasms	Other non-epithelial cancer of skin	Inverse var	3	-0.10018	0.067764	0.13929472	0.90467	0.792151	1.033172	0.838983
digestive	Chronic periodontitis	Inverse var	3	0.437869	0.297403	0.14093791	1.549402	0.864986	2.775359	0.8424
sense organs	Glaucoma	Inverse var	3	0.158454	0.108107	0.14272677	1.171698	0.947967	1.448231	0.846255
digestive	Dental caries	Inverse var	3	0.239335	0.163705	0.14374446	1.270404	0.921708	1.751018	0.846255
sense organs	Epiphora	Inverse var	3	-0.34179	0.234816	0.14551032	0.710496	0.448417	1.125748	0.850258
digestive	Other disorders of gallbladder	Inverse var	3	0.271369	0.188925	0.15089266	1.311759	0.905812	1.899636	0.864211
genitourinary	Hematuria	Inverse var	3	0.08403	0.058523	0.15104535	1.087662	0.969791	1.219859	0.864211
injuries & poisonings	Fracture of patella	Inverse var	3	0.36134	0.253015	0.15325235	1.435251	0.874092	2.356668	0.864211
dermatologic	Ingrowing nail	Inverse var	3	0.321407	0.225544	0.15414916	1.379067	0.886335	2.145717	0.864211
respiratory	Chronic pharyngitis and nasopharyngitis	Inverse var	3	0.378695	0.26587	0.15434165	1.460377	0.867266	2.45911	0.864211
neoplasms	Colorectal cancer	Inverse var	3	0.155777	0.10955	0.15503453	1.168565	0.942762	1.448451	0.864211
pregnancy complications	Other complications of pregnancy NEC	Inverse var	3	-0.22251	0.157514	0.15775305	0.800503	0.587874	1.090038	0.864211
endocrine/metabolic	Hypothyroidism NOS	Inverse var	3	0.105021	0.074375	0.15793329	1.110734	0.960066	1.285048	0.864211
digestive	Inflammatory bowel disease and other gastroenteritis and colitis	Inverse var	3	0.229675	0.162822	0.15836599	1.258191	0.914428	1.731186	0.864211
pregnancy complications	Late pregnancy and failed induction	Inverse var	3	0.286134	0.20327	0.15923334	1.331271	0.893798	1.982868	0.864211
endocrine/metabolic	Secondary hypothyroidism	Inverse var	3	0.297619	0.211979	0.1603169	1.346649	0.888822	2.040301	0.864211
neurological	Epilepsy	Inverse var	3	0.329034	0.234816	0.16114274	1.389625	0.877037	2.201796	0.864211
genitourinary	Menopausal and postmenopausal disorders	Inverse var	3	0.124687	0.089649	0.16427601	1.132794	0.950255	1.350398	0.869607
endocrine/metabolic	Gout	Inverse var	3	0.175522	0.126228	0.16437009	1.191869	0.930639	1.526425	0.869607
digestive	Functional digestive disorders	Inverse var	3	0.071175	0.051416	0.16626956	1.073769	0.970833	1.187618	0.871116
musculoskeletal	Other disorders of synovium, tendon, and bursa	Inverse var	3	0.112286	0.081248	0.16696782	1.118832	0.954125	1.311972	0.871116
digestive	Other disorders of biliary tract	Inverse var	3	0.304201	0.220647	0.16799308	1.355542	0.879619	2.088965	0.871116
digestive	Diseases of hard tissues of teeth	Inverse var	3	0.243406	0.17841	0.17247035	1.275586	0.899176	1.809569	0.888449
sense organs	Ptosis of eyelid	Inverse var	3	-0.25628	0.188856	0.17478016	0.773927	0.534494	1.120617	0.891731
hematopoietic	Diseases of white blood cells	Inverse var	3	0.233542	0.172343	0.17538509	1.263066	0.901	1.770627	0.891731
sense organs	Primary open angle glaucoma	Inverse var	3	0.392722	0.295627	0.18403376	1.481006	0.829687	2.643623	0.92414
endocrine/metabolic	Hypothyroidism	Inverse var	3	0.106626	0.080373	0.18463057	1.112518	0.950368	1.302333	0.92414
sense organs	Open-angle glaucoma	Inverse var	3	0.389379	0.294631	0.1863066	1.476064	0.828534	2.629662	0.92414
circulatory system	Right bundle branch block	Inverse var	3	0.237713	0.180479	0.18779659	1.268345	0.890453	1.806608	0.92414
pregnancy complications	Hemorrhage during pregnancy; childbirth and postpartum	Inverse var	3	0.219278	0.16663	0.18818894	1.245178	0.898241	1.726116	0.92414
pregnancy complications	Fetal distress and abnormal forces of labor	Inverse var	3	0.230269	0.175465	0.18940634	1.258938	0.892578	1.775672	0.92414
mental disorders	Other mental disorder	Inverse var	3	0.05834	0.044632	0.19116335	1.060075	0.971282	1.156986	0.92414
genitourinary	Frequency of urination and polyuria	Inverse var	3	0.146664	0.112577	0.1926463	1.157965	0.928684	1.443852	0.92414
mental disorders	Other specified nonpsychotic and/or transient mental disorders	Inverse var	3	-0.36414	0.279509	0.1926494	0.694794	0.401729	1.201654	0.92414
neoplasms	Secondary malignant neoplasm of digestive systems	Inverse var	3	0.233251	0.180382	0.19597847	1.262698	0.886656	1.798224	0.92414
genitourinary	Stricture/obstruction of ureter	Inverse var	3	0.296964	0.229675	0.19602015	1.345766	0.857959	2.110926	0.92414
endocrine/metabolic	Other disorders of metabolism	Inverse var	3	0.237014	0.18471	0.19943236	1.267459	0.882482	1.820379	0.92414
musculoskeletal	Ganglion and cyst of synovium, tendon, and bursa	Inverse var	3	0.161559	0.126228	0.20058073	1.175341	0.917734	1.505259	0.92414
musculoskeletal	Other and unspecified disorders of back	Inverse var	3	-0.1956	0.153421	0.20234615	0.822344	0.608778	1.110832	0.92414
circulatory system	Left bundle branch block	Inverse var	3	0.212817	0.166968	0.20245172	1.237158	0.891865	1.716135	0.92414
endocrine/metabolic	Obesity	Inverse var	3	0.086014	0.067857	0.20494505	1.089822	0.954102	1.244848	0.92414
circulatory system	Aortic valve disease	Inverse var	3	0.272969	0.215547	0.20536848	1.31386	0.861136	2.004592	0.92414
circulatory system	Unstable angina (intermediate coronary syndrome)	Inverse var	3	0.125321	0.099267	0.20678136	1.133512	0.933101	1.376968	0.92414
symptoms	Symptoms involving nervous and musculoskeletal systems	Inverse var	3	-0.20482	0.162264	0.20686308	0.814798	0.592827	1.11988	0.92414
infectious diseases	Viral warts & HPV	Inverse var	3	-0.27827	0.220748	0.20745404	0.75709	0.491183	1.166948	0.92414
circulatory system	Intracranial hemorrhage	Inverse var	3	0.209502	0.166802	0.20911926	1.233064	0.889203	1.709899	0.92414
genitourinary	Pain and other symptoms associated with female genital organs	Inverse var	3	-0.16371	0.130698	0.21036913	0.848992	0.65713	1.096871	0.92414
sense organs	Cataract	Inverse var	3	-0.06756	0.053982	0.21076193	0.934674	0.840834	1.038989	0.92414
circulatory system	Atrial fibrillation and flutter	Inverse var	3	0.106684	0.085268	0.21087428	1.112582	0.94135	1.314963	0.92414
circulatory system	Cerebral ischemia	Inverse var	3	0.163446	0.130766	0.21133245	1.177561	0.911325	1.521576	0.92414
injuries & poisonings	Hemorrhage or hematoma complicating a procedure	Inverse var	3	-0.22067	0.177199	0.21300228	0.801978	0.566667	1.135002	0.92414
dermatologic	Dyschromia and Vitiligo	Inverse var	3	0.309277	0.249011	0.2142287	1.362439	0.836286	2.219624	0.92414
genitourinary	Hydronephrosis	Inverse var	3	0.195609	0.157959	0.21558366	1.216052	0.892267	1.657331	0.92414
circulatory system	Other chronic ischemic heart disease, unspecified	Inverse var	3	0.077838	0.062991	0.21657392	1.080947	0.9554	1.222993	0.92414
musculoskeletal	Other derangement of joint	Inverse var	3	-0.19425	0.157793	0.21829922	0.823449	0.604395	1.121897	0.92414
neurological	Parkinson's disease	Inverse var	3	0.354658	0.288686	0.21925068	1.425692	0.809639	2.510501	0.92414
circulatory system	Paroxysmal supraventricular tachycardia	Inverse var	3	-0.17731	0.144412	0.21952756	0.837523	0.631061	1.111533	0.92414
neurological	Other conditions of brain	Inverse var	3	0.223927	0.18471	0.22538972	1.25098	0.871008	1.796712	0.929188
digestive	Intestinal malabsorption (non-celiac)	Inverse var	3	0.185958	0.153421	0.22548474	1.204371	0.891591	1.626878	0.929188
dermatologic	Diseases of nail, NOS	Inverse var	3	0.245426	0.203098	0.22688875	1.278165	0.858434	1.903125	0.929188
hematopoietic	Decreased white blood cell count	Inverse var	3	0.240594	0.199424	0.22764573	1.272004	0.86047	1.880361	0.929188
hematopoietic	Neutropenia	Inverse var	3	0.240594	0.199424	0.22764573	1.272004	0.86047	1.880361	0.929188
musculoskeletal	Other inflammatory spondylopathies	Inverse var	3	0.206501	0.17144	0.22839391	1.229368	0.878515	1.720342	0.929188
dermatologic	Superficial cellulitis and abscess	Inverse var	3	-0.09752	0.081248	0.23004243	0.907086	0.773551	1.063673	0.929188
symptoms	Myalgia and myositis unspecified	Inverse var	3	-0.3292	0.274382	0.23022034	0.719499	0.420215	1.231936	0.929188
circulatory system	Cardiac arrest and ventricular fibrillation	Inverse var	3	0.26704	0.223242	0.23162145	1.306093	0.843231	2.023027	0.930049
digestive	Periodontitis (acute or chronic)	Inverse var	3	0.277475	0.233963	0.23563063	1.319793	0.834358	2.087657	0.939279
digestive	Acute pancreatitis	Inverse var	3	0.186131	0.157793	0.23816392	1.20458	0.884137	1.641164	0.939279
musculoskeletal	Rheumatism, unspecified and fibrositis	Inverse var	3	0.259911	0.220748	0.23903132	1.296814	0.841344	1.998858	0.939279
circulatory system	Elevated blood pressure reading without diagnosis of hypertension	Inverse var	3	0.237725	0.201961	0.23916195	1.26836	0.853749	1.884321	0.939279

infectious diseases	Gram negative septicemia	Inverse var	3	0.29134	0.248799	0.24160496	1.338219	0.821761	2.179261	0.939279
circulatory system	Coronary atherosclerosis	Inverse var	3	0.100208	0.085617	0.2418296	1.105401	0.934634	1.307369	0.939279
genitourinary	Functional disorders of bladder	Inverse var	3	0.281308	0.241492	0.24406959	1.324862	0.825293	2.12683	0.939279
musculoskeletal	Contracture of palmar fascia [Dupuytren's disease]	Inverse var	3	-0.19614	0.169731	0.24785308	0.821899	0.589306	1.146295	0.939279
respiratory	Obstructive chronic bronchitis	Inverse var	3	0.156332	0.135467	0.24848909	1.169215	0.896567	1.524775	0.939279
neoplasms	Melanomas of skin, dx or hx	Inverse var	3	0.156113	0.135402	0.24892773	1.168958	0.896484	1.524248	0.939279
neoplasms	Melanomas of skin	Inverse var	3	0.156113	0.135402	0.24892773	1.168958	0.896484	1.524248	0.939279
respiratory	Chronic tonsillitis and adenoiditis	Inverse var	3	-0.23939	0.207829	0.24936843	0.787104	0.523751	1.182877	0.939279
neoplasms	Malignant neoplasm of kidney, except pelvis	Inverse var	3	-0.25383	0.220748	0.25019288	0.775821	0.503335	1.19582	0.939279
endocrine/metabolic	Hyperpotassemia	Inverse var	3	0.495987	0.431824	0.25072706	1.642118	0.704414	3.828073	0.939279
neoplasms	Carcinoma in situ of skin	Inverse var	3	-0.3084	0.270267	0.25382635	0.734619	0.43252	1.247723	0.939279
injuries & poisonings	Injury, NOS	Inverse var	3	0.087275	0.076711	0.25523656	1.091197	0.938871	1.268237	0.939279
endocrine/metabolic	Overweight, obesity and other hyperalimentation	Inverse var	3	0.076822	0.067764	0.25693789	1.07985	0.945542	1.233234	0.939279
endocrine/metabolic	Acidosis	Inverse var	3	-0.25336	0.223577	0.25713064	0.776191	0.500791	1.203042	0.939279
neoplasms	Lymphoid leukemia, chronic	Inverse var	3	0.351382	0.31084	0.25829637	1.42103	0.7727	2.613338	0.939279
mental disorders	Alcohol-related disorders	Inverse var	3	0.071351	0.063228	0.2591262	1.073958	0.948781	1.215649	0.939279
musculoskeletal	Other acquired musculoskeletal deformity	Inverse var	3	0.178183	0.157959	0.25930538	1.195044	0.876853	1.6287	0.939279
congenital anomalies	Genitourinary congenital anomalies	Inverse var	3	-0.2926	0.260692	0.26169555	0.746322	0.447734	1.244032	0.939279
dermatologic	Chronic ulcer of skin	Inverse var	3	0.226666	0.202632	0.26330542	1.254411	0.84325	1.86605	0.939279
neoplasms	Benign neoplasm of ovary	Inverse var	3	0.299238	0.268227	0.26458663	1.34883	0.79733	2.281794	0.939279
respiratory	Chronic bronchitis	Inverse var	3	0.145888	0.130832	0.2648149	1.157067	0.895349	1.495287	0.939279
symptoms	Neuralgia, neuritis, and radiculitis NOS	Inverse var	3	0.22592	0.202729	0.26510933	1.253475	0.842461	1.865013	0.939279
sense organs	Labyrinthitis	Inverse var	3	0.280929	0.253015	0.26685852	1.324359	0.806558	2.174585	0.939452
injuries & poisonings	Skull and face fracture and other intercranial injury	Inverse var	3	0.173589	0.15763	0.27079057	1.189566	0.873397	1.620188	0.939452
injuries & poisonings	Fracture of clavicle or scapula	Inverse var	3	-0.23365	0.212357	0.27121098	0.791638	0.522114	1.200295	0.939452
endocrine/metabolic	Gout and other crystal arthropathies	Inverse var	3	0.128852	0.117116	0.27124113	1.137522	0.904209	1.431036	0.939452
sense organs	Inflammation of eyelids	Inverse var	3	-0.15847	0.144246	0.27193712	0.853448	0.643269	1.132301	0.939452
circulatory system	First degree AV block	Inverse var	3	0.246819	0.224862	0.27235709	1.279948	0.823733	1.988833	0.939452
circulatory system	Other specified cardiac dysrhythmias	Inverse var	3	0.224103	0.205462	0.27539334	1.2512	0.836438	1.871627	0.945759
sense organs	Retinal detachment with retinal defect	Inverse var	3	-0.20355	0.188925	0.28129719	0.81583	0.563357	1.181451	0.961815
respiratory	Pneumonitis due to inhalation of food or vomitus	Inverse var	3	0.309461	0.288492	0.28341156	1.362691	0.774156	2.398646	0.962252
endocrine/metabolic	Hyposmolality and/or hyponatremia	Inverse var	3	0.178678	0.166734	0.28388276	1.195636	0.862327	1.657776	0.962252
digestive	Other symptoms involving abdomen and pelvis	Inverse var	3	0.134449	0.126125	0.28642222	1.143907	0.893369	1.464705	0.966675
sense organs	Cholesteatoma	Inverse var	3	0.358285	0.337805	0.28885924	1.430874	0.737999	2.774258	0.970716
injuries & poisonings	Intracranial hemorrhage (injury)	Inverse var	3	0.536038	0.512345	0.29544892	1.709221	0.626154	4.665685	0.977583
neoplasms	Cancer of kidney and renal pelvis	Inverse var	3	-0.2478	0.23705	0.29585632	0.780514	0.490455	1.242115	0.977583
sense organs	Otitis media	Inverse var	3	0.174267	0.166734	0.29593888	1.190374	0.858532	1.65048	0.977583
genitourinary	Other abnormality of urination	Inverse var	3	-0.16909	0.162264	0.29737312	0.844431	0.614387	1.160609	0.977583
endocrine/metabolic	Disorders of fluid, electrolyte, and acid-base balance	Inverse var	3	0.144551	0.138848	0.29783897	1.155521	0.880214	1.516936	0.977583
neoplasms	Malignant neoplasm of testis	Inverse var	3	-0.11709	0.112748	0.29904332	0.889508	0.713144	1.109488	0.977583
endocrine/metabolic	Disorders of the pituitary gland and its hypothalamic control	Inverse var	3	-0.27604	0.266135	0.29964219	0.758786	0.450381	1.278375	0.977583
circulatory system	Stricture of artery	Inverse var	3	0.244629	0.238961	0.30596793	1.277147	0.799528	2.040085	0.992603
genitourinary	Vaginal enterocele, congenital or acquired	Inverse var	3	-0.27525	0.270267	0.30847615	0.759385	0.447101	1.289787	0.992603
respiratory	Asthma	Inverse var	3	0.05777	0.056746	0.30865256	1.059472	0.947952	1.184111	0.992603
digestive	Other disorders of stomach and duodenum	Inverse var	3	0.123506	0.121757	0.31040914	1.131457	0.891243	1.436413	0.992603
digestive	Ascites (non malignant)	Inverse var	3	0.182538	0.180282	0.3112908	1.20026	0.842978	1.70897	0.992603
neoplasms	Other benign neoplasm of connective and other soft tissue	Inverse var	3	0.21414	0.211979	0.3124003	1.238797	0.817636	1.876895	0.992603
musculoskeletal	Fasciitis	Inverse var	3	-0.17834	0.176801	0.31311998	0.836659	0.591634	1.183162	0.992603
sense organs	Ectropion or entropion	Inverse var	3	0.219851	0.220228	0.31813972	1.245891	0.80913	1.918413	0.995612
digestive	Umbilical hernia	Inverse var	3	0.133128	0.134396	0.32189636	1.142397	0.877843	1.486678	0.995612
dermatologic	Disorders of sweat glands	Inverse var	3	0.241397	0.24377	0.32204419	1.273026	0.789471	2.052762	0.995612
infectious diseases	Intestinal infection due to C. difficile	Inverse var	3	0.271627	0.274382	0.32219385	1.312097	0.766316	2.246593	0.995612
respiratory	Acute upper respiratory infections of multiple or unspecified sites	Inverse var	3	0.167563	0.1696	0.32315917	1.18242	0.848018	1.648688	0.995612
digestive	Ulcer of esophagus	Inverse var	3	-0.09788	0.0992	0.3237902	0.906757	0.746535	1.101366	0.995612
pregnancy complications	Miscarriage; stillbirth	Inverse var	3	0.10209	0.103636	0.32458587	1.107483	0.9039	1.356918	0.995612
genitourinary	Noninflammatory disorders of cervix	Inverse var	3	0.128352	0.130766	0.32632648	1.136953	0.879898	1.469105	0.995612
hematopoietic	Disorders of iron metabolism	Inverse var	3	0.264546	0.270267	0.32766404	1.302839	0.767069	2.212824	0.995612
genitourinary	Breast conditions, congenital or relating to hormones	Inverse var	3	0.224251	0.229675	0.328874	1.251385	0.797788	1.962882	0.995612
circulatory system	Ill-defined descriptions and complications of heart disease	Inverse var	3	0.24103	0.247911	0.33092882	1.272559	0.782802	2.06873	0.995612
genitourinary	Other symptoms/disorders or the urinary system	Inverse var	3	0.043857	0.045208	0.33198892	1.044833	0.956236	1.141638	0.995612
circulatory system	Cardiac arrest	Inverse var	3	0.222729	0.229675	0.33216728	1.249482	0.796575	1.959897	0.995612
respiratory	Nasal polyps	Inverse var	3	0.117691	0.12192	0.33439029	1.124896	0.885792	1.428541	0.995612
dermatologic	Cellulitis and abscess of face/neck	Inverse var	3	-0.29559	0.306708	0.3351656	0.74409	0.407897	1.357377	0.995612
neurological	Degenerative disease of the spinal cord	Inverse var	3	-0.16032	0.166802	0.33647528	0.851869	0.614311	1.181293	0.995612
hematopoietic	Other deficiency anemia	Inverse var	3	0.199657	0.207829	0.3367144	1.220983	0.81246	1.834921	0.995612
neoplasms	Skin cancer	Inverse var	3	-0.06039	0.063062	0.33827558	0.9414	0.831945	1.065256	0.995612
digestive	Celiac disease	Inverse var	3	0.155118	0.162496	0.33978102	1.167796	0.849274	1.60578	0.995612
circulatory system	Premature beats	Inverse var	3	0.286112	0.302561	0.34433447	1.331242	0.735719	2.408808	0.995612
digestive	Hematemesis	Inverse var	3	0.233191	0.246868	0.34486324	1.262623	0.778279	2.048387	0.995612
sense organs	Other disorders of eye	Inverse var	3	-0.10554	0.112748	0.3492505	0.899841	0.721428	1.122377	0.995612
mental disorders	Delirium dementia and amnestic and other cognitive disorders	Inverse var	3	-0.1477	0.157959	0.34975444	0.862688	0.63299	1.175739	0.995612
digestive	Jaundice (not of newborn)	Inverse var	3	0.264097	0.284452	0.35317902	1.302254	0.745702	2.274187	0.995612
genitourinary	Lump or mass in breast	Inverse var	3	-0.16724	0.180382	0.35385142	0.845996	0.594052	1.204794	0.995612
genitourinary	Cystitis and urethritis	Inverse var	3	0.116596	0.126294	0.35589826	1.123666	0.87727	1.439266	0.995612
neoplasms	Cancer of other lymphoid, histiocytic tissue	Inverse var	3	0.193837	0.210336	0.35675659	1.213899	0.803788	1.833259	0.995612
sense organs	Disorders of external ear	Inverse var	3	0.169935	0.18471	0.35756794	1.185227	0.825227	1.702275	0.995612
circulatory system	Congestive heart failure (CHF) NOS	Inverse var	3	-0.14476	0.157793	0.3589257	0.865228	0.63506	1.178819	0.995612
digestive	Ileostomy status	Inverse var	3	0.156739	0.171505	0.36076871	1.16969	0.835762	1.637039	0.995612
genitourinary	Acute renal failure	Inverse var	3	0.094727	0.1039	0.36192011	1.099359	0.896805	1.347662	0.995612
digestive	Cholelithiasis and cholecystitis	Inverse var	3	0.091518	0.100561	0.36278307	1.095836	0.899801	1.33458	0.995612
dermatologic	Psoriasis	Inverse var	3	-0.13539	0.148883	0.36315835	0.873376	0.652333	1.16932	0.995612
circulatory system	Occlusion of cerebral arteries	Inverse var	3	0.102348	0.112577	0.36327894	1.107768	0.888427	1.381263	0.995612
injuries & poisonings	Fracture of pelvis	Inverse var	3	0.236321	0.261986	0.3670377	1.266581	0.757924	2.116607	0.995612
symptoms	Abdominal pain	Inverse var	3	-0.03231	0.036036	0.36991695	0.968206	0.902181	1.039063	0.995612
hematopoietic	Megaloblastic anemia	Inverse var	3	0.193508	0.216318	0.3710265	1.213499	0.794156	1.854269	0.995612
circulatory system	Tachycardia NOS	Inverse var	3	-0.13248	0.148949	0.37376923	0.87592	0.654148	1.172877	0.995612
digestive	Diseases of the salivary glands	Inverse var	3	0.232507	0.261986	0.37482158	1.261759	0.755039	2.108549	0.995612
circulatory system	Hypertensive chronic kidney disease	Inverse var	3	0.236727	0.266903	0.37511198	1.267095	0.750959	2.137971	0.995612
endocrine/metabolic	Hypovolemia	Inverse var	3	0.135616	0.153025	0.37549254	1.145241	0.848476	1.545804	0.995612
injuries & poisonings	Internal derangement of knee	Inverse var	3	-0.05156	0.058591	0.37887785	0.949748	0.84671	1.065326	0.995612
pregnancy complications	Known or suspected fetal abnormality affecting management of mother	Inverse var	3	0.13532	0.154733	0.38182635	1.144903	0.84539	1.55053	0.995612
digestive	Paralytic ileus	Inverse var	3	0.324468	0.371029	0.38184168	1.383295	0.66848	2.862473	0.995612
genitourinary	Pelvic inflammatory disease (PID)	Inverse var	3	-0.20075	0.229675	0.38207601	0.818114	0.521567	1.283267	0.995612
digestive	Abnormal results of function study of liver	Inverse var	3	0.106244	0.121586	0.382219	1.112093	0.876284	1.411357	0.995612
respiratory	Pneumococcal pneumonia	Inverse var	3	0.082605	0.094559	0.38234344	1.086113	0.90237	1.30727	0.995612
neoplasms	Cervical cancer	Inverse var	3	-0.14954	0.171505	0.3832358	0.861101	0.61527	1.205153	0.995612
genitourinary	Abnormal findings on mammogram or breast exam	Inverse var	3	-0.15298	0.175978	0.38468002	0.858148	0.607809	1.211595	0.995612
digestive	Peritoneal or intestinal adhesions	Inverse var	3	-0.25321	0.291678	0.38532442	0.776302	0.438278	1.375029	0.995612
circulatory system	Hemorrhoids	Inverse var	3	-0.04283	0.049514	0.38705988	0.958076	0.869467	1.055717	0.995612
neoplasms	Benign neoplasm of lip, oral cavity, and pharynx	Inverse var	3	0.269183	0.313355	0.39032156	1.308894	0.708225	2.419011	0.995612
mental disorders	Depression	Inverse var	3	0.057924	0.067532	0.39104622	1.059634	0.928264	1.209596	0.995612

hematopoietic	Purpura and other hemorrhagic conditions	Inverse var	3	-0.14245	0.166802	0.39309834	0.86723	0.625388	1.202594	0.995612
sense organs	Otitis media and Eustachian tube disorders	Inverse var	3	0.150218	0.176247	0.39403814	1.162088	0.822649	1.641583	0.995612
circulatory system	Subarachnoid hemorrhage	Inverse var	3	0.211185	0.247911	0.39429352	1.23514	0.759784	2.0079	0.995612
musculoskeletal	Hammer toe (acquired)	Inverse var	3	-0.13808	0.162264	0.39477548	0.871025	0.633737	1.19716	0.995612
infectious diseases	Postoperative infection	Inverse var	3	0.110722	0.130815	0.39732898	1.117085	0.864438	1.443572	0.995612
digestive	Ulcerative colitis	Inverse var	3	0.105511	0.126228	0.40322362	1.111278	0.867712	1.423213	0.995612
genitourinary	Cyst of kidney, acquired	Inverse var	3	0.268271	0.321529	0.40407907	1.307701	0.696333	2.455838	0.995612
pregnancy complications	Hypertension complicating pregnancy, childbirth, and the puerperium	Inverse var	3	0.179095	0.216214	0.40748732	1.196134	0.782952	1.827363	0.995612
infectious diseases	E. coli	Inverse var	3	0.111483	0.135237	0.40973655	1.117935	0.857632	1.457244	0.995612
neoplasms	Non-Hodgkins lymphoma	Inverse var	3	0.238682	0.290739	0.41167513	1.269575	0.718086	2.244607	0.995612
neoplasms	Benign neoplasm of skin	Inverse var	3	-0.06653	0.081182	0.41248762	0.935635	0.798	1.097007	0.995612
musculoskeletal	Derangement of joint, non-traumatic	Inverse var	3	-0.16527	0.201902	0.41302509	0.847662	0.570638	1.259172	0.995612
sense organs	Inflammation of the eye	Inverse var	3	-0.10295	0.126125	0.41434303	0.90217	0.704578	1.155175	0.995612
circulatory system	Raynaud's syndrome	Inverse var	3	0.168439	0.207731	0.41745057	1.183456	0.787641	1.778181	0.995612
injuries & poisonings	Fracture of lower limb	Inverse var	3	-0.06918	0.08555	0.41869861	0.933156	0.789101	1.103509	0.995612
digestive	Flatulence	Inverse var	3	-0.13854	0.17134	0.41877495	0.870631	0.622281	1.218097	0.995612
circulatory system	Intracerebral hemorrhage	Inverse var	3	0.213913	0.266135	0.42152584	1.238515	0.735127	2.086606	0.995612
digestive	Reflux esophagitis	Inverse var	3	0.057815	0.072001	0.42198404	1.059519	0.92007	1.220105	0.995612
digestive	Cholelithiasis	Inverse var	3	0.097194	0.121781	0.42480807	1.102075	0.868058	1.399178	0.995612
injuries & poisonings	Burns	Inverse var	3	-0.23257	0.292615	0.42672221	0.792491	0.446596	1.406285	0.995612
genitourinary	Prolapse of vaginal vault after hysterectomy	Inverse var	3	-0.26038	0.328562	0.42808451	0.770761	0.404801	1.467566	0.995612
genitourinary	Hypertrophy of female genital organs	Inverse var	3	-0.20826	0.263366	0.42907707	0.811994	0.484586	1.360612	0.995612
digestive	Peritoneal adhesions (postoperative) (postinfection)	Inverse var	3	0.125871	0.159351	0.42958693	1.134136	0.829895	1.549913	0.995612
symptoms	Musculoskeletal symptoms referable to limbs	Inverse var	3	-0.07469	0.094729	0.43043989	0.928033	0.770776	1.117374	0.995612
circulatory system	Angina pectoris	Inverse var	3	0.063962	0.08123	0.43103323	1.066052	0.909147	1.250036	0.995612
circulatory system	Arrhythmia (cardiac) NOS	Inverse var	3	0.180355	0.229675	0.43229893	1.197643	0.763526	1.878584	0.995612
respiratory	Pulmonary collapse; interstitial and compensatory emphysema	Inverse var	3	-0.12353	0.157793	0.43371511	0.883797	0.648688	1.204117	0.995612
genitourinary	Polyp of corpus uteri	Inverse var	3	-0.0633	0.08108	0.4349862	0.938663	0.800743	1.10034	0.995612
respiratory	Other dyspnea	Inverse var	3	-0.1823	0.233785	0.43553444	0.833355	0.527022	1.317747	0.995612
genitourinary	Urinary tract infection	Inverse var	3	-0.04916	0.063163	0.43641255	0.952031	0.841174	1.077498	0.995612
digestive	Acute appendicitis	Inverse var	3	-0.16966	0.218195	0.43683763	0.843955	0.550285	1.294347	0.995612
neoplasms	Cancer of urinary organs (incl. kidney and bladder)	Inverse var	3	-0.11497	0.148029	0.43735126	0.891393	0.666906	1.191444	0.995612
circulatory system	Hypertensive heart and/or renal disease	Inverse var	3	0.235815	0.303684	0.43744504	1.26594	0.698091	2.295696	0.995612
dermatologic	Prurigo and Lichen	Inverse var	3	-0.27363	0.352434	0.43751402	0.760614	0.381212	1.517617	0.995612
circulatory system	Polyarteritis nodosa and allied conditions	Inverse var	3	0.341929	0.444551	0.44180023	1.40766	0.588964	3.364395	0.995612
injuries & poisonings	Fracture of foot	Inverse var	3	0.128333	0.16699	0.44218577	1.136932	0.819576	1.577173	0.995612
circulatory system	Vascular insufficiency of intestine	Inverse var	3	0.571354	0.743511	0.44221802	1.770662	0.412331	7.603708	0.995612
musculoskeletal	Disorders of muscle, ligament, and fascia	Inverse var	3	-0.10159	0.132225	0.44228669	0.903397	0.69715	1.17066	0.995612
genitourinary	Pyelonephritis	Inverse var	3	-0.14901	0.19396	0.44234469	0.861562	0.589094	1.260053	0.995612
dermatologic	Diffuse diseases of connective tissue	Inverse var	3	-0.11713	0.153309	0.4448475	0.889467	0.658614	1.201237	0.995612
circulatory system	Disease of tricuspid valve	Inverse var	3	-0.16439	0.216419	0.44750203	0.848412	0.555121	1.29666	0.995612
digestive	Other disorders of liver	Inverse var	3	0.07172	0.094559	0.44817286	1.074354	0.892601	1.293117	0.995612
circulatory system	Hypotension	Inverse var	3	0.071628	0.094487	0.44840541	1.074256	0.892645	1.292816	0.995612
genitourinary	Mucous polyp of cervix	Inverse var	3	0.091941	0.121757	0.45017546	1.096301	0.863551	1.391782	0.995612
circulatory system	Atrioventricular [AV] block	Inverse var	3	0.115604	0.153255	0.45065245	1.122552	0.831291	1.515861	0.995612
digestive	Diseases of pulp and periapical tissues	Inverse var	3	0.125501	0.166734	0.45163001	1.133716	0.817669	1.571922	0.995612
sense organs	Disorders of vitreous body	Inverse var	3	-0.14189	0.189711	0.45450098	0.867716	0.598264	1.258527	0.995612
dermatologic	Carbuncle and furuncle	Inverse var	3	-0.1108	0.148716	0.45625506	0.89512	0.668793	1.198039	0.995612
dermatologic	Psoriasis and related disorders	Inverse var	3	-0.11078	0.148716	0.45632623	0.895136	0.668805	1.19806	0.995612
endocrine/metabolic	Vitamin B-complex deficiencies	Inverse var	3	-0.37566	0.504299	0.45632759	0.686838	0.255615	1.845536	0.995612
digestive	Gingival and periodontal diseases	Inverse var	3	0.124	0.166968	0.45768918	1.132016	0.816068	1.570286	0.995612
circulatory system	Peripheral vascular disease	Inverse var	3	0.083506	0.112748	0.45890687	1.087092	0.871553	1.355936	0.995612
neoplasms	Secondary malignant neoplasm	Inverse var	3	0.052896	0.072238	0.46401956	1.05432	0.915128	1.214683	0.995612
endocrine/metabolic	Type 2 diabetes	Inverse var	3	-0.03934	0.054218	0.46809865	0.961424	0.864497	1.069219	0.995612
injuries & poisonings	Complications of surgical and medical procedures	Inverse var	3	-0.07283	0.100843	0.47015521	0.929758	0.763011	1.132945	0.995612
genitourinary	Other disorders of urethra and urinary tract	Inverse var	3	-0.081	0.112577	0.47181601	0.922192	0.739595	1.14987	0.995612
sense organs	Tinnitus	Inverse var	3	0.239493	0.332959	0.47196452	1.270605	0.661591	2.440233	0.995612
genitourinary	Endometriosis	Inverse var	3	-0.08103	0.112748	0.47233306	0.922166	0.739326	1.150222	0.995612
digestive	Other disorders of intestine	Inverse var	3	0.07747	0.108276	0.4743062	1.08055	0.873934	1.336015	0.995612
neurological	Sleep disorders	Inverse var	3	0.09977	0.139689	0.47508677	1.104917	0.840279	1.452899	0.995612
respiratory	Allergic rhinitis	Inverse var	3	-0.15372	0.216318	0.47730891	0.857509	0.561184	1.310304	0.995612
pregnancy complications	Missed abortion/Hydatidiform mole	Inverse var	3	0.143987	0.202628	0.47733494	1.154869	0.77634	1.717961	0.995612
respiratory	Acute pharyngitis	Inverse var	3	0.173132	0.24418	0.47830235	1.189023	0.736784	1.918847	0.995612
respiratory	Abnormal findings examination of lungs	Inverse var	3	0.099217	0.13994	0.47832669	1.104306	0.839403	1.452808	0.995612
genitourinary	Calculus of lower urinary tract	Inverse var	3	-0.17869	0.252033	0.47833969	0.836369	0.510344	1.370669	0.995612
respiratory	Respiratory failure	Inverse var	3	0.123689	0.174897	0.47943776	1.131663	0.803233	1.594384	0.995612
musculoskeletal	Rupture of synovium	Inverse var	3	-0.20246	0.288492	0.48282107	0.816723	0.463987	1.437619	0.995612
digestive	Other disorders of peritoneum	Inverse var	3	0.088891	0.126943	0.48377551	1.092962	0.852214	1.401721	0.995612
injuries & poisonings	Injuries to the nervous system	Inverse var	3	-0.15869	0.226937	0.48437204	0.853257	0.546899	1.331229	0.995612
genitourinary	Renal failure NOS	Inverse var	3	0.136261	0.195127	0.48497832	1.145981	0.781775	1.679858	0.995612
injuries & poisonings	Contusion	Inverse var	3	0.130201	0.186994	0.48625308	1.139057	0.789537	1.643305	0.995612
mental disorders	Delirium due to conditions classified elsewhere	Inverse var	3	-0.21466	0.308831	0.48701134	0.806816	0.440445	1.477941	0.995612
neurological	Epilepsy, recurrent seizures, convulsions	Inverse var	3	0.095468	0.137951	0.48891037	1.100173	0.839528	1.44174	0.995612
neoplasms	Cancer within the respiratory system	Inverse var	3	0.093702	0.135402	0.48892241	1.098232	0.842243	1.432025	0.995612
musculoskeletal	Stiffness of joint	Inverse var	3	0.196287	0.284351	0.49000788	1.216876	0.69695	2.124668	0.995612
infectious diseases	Candidiasis	Inverse var	3	0.152442	0.221349	0.49101301	1.164675	0.754726	1.7973	0.995612
neoplasms	Cancer of other female genital organs	Inverse var	3	-0.09905	0.144143	0.49196647	0.905695	0.682787	1.201375	0.995612
musculoskeletal	Osteomyelitis, periostitis, and other infections involving bone	Inverse var	3	0.342251	0.501975	0.49536076	1.408113	0.526438	3.76641	0.995612
symptoms	Swelling of limb	Inverse var	3	-0.07554	0.110805	0.49538942	0.92724	0.74623	1.152157	0.995612
endocrine/metabolic	Hyperparathyroidism	Inverse var	3	-0.17024	0.252033	0.49938999	0.843466	0.514675	1.3823	0.995612
congenital anomalies	Congenital anomalies of great vessels	Inverse var	3	-0.11245	0.166802	0.50020238	0.893639	0.644432	1.239216	0.995612
circulatory system	Arterial embolism and thrombosis of lower extremity artery	Inverse var	3	-0.19979	0.298399	0.50315671	0.818905	0.45628	1.469724	0.995612
dermatologic	Cellulitis and abscess of arm/hand	Inverse var	3	-0.06325	0.094729	0.50436206	0.938713	0.779647	1.130233	0.995612
injuries & poisonings	Complications of cardiac/vascular device, implant, and graft	Inverse var	3	-0.13308	0.200164	0.50614813	0.875396	0.591319	1.295949	0.995612
circulatory system	Varicose veins of lower extremity, symptomtic	Inverse var	3	-0.23983	0.361196	0.50670113	0.786763	0.387604	1.596982	0.995612
neoplasms	Benign neoplasm of other endocrine glands and related structures	Inverse var	3	-0.15758	0.238961	0.50962217	0.854211	0.534759	1.364497	0.995612
respiratory	Bronchitis	Inverse var	3	-0.23808	0.361309	0.50994264	0.788143	0.388197	1.600137	0.995612
neoplasms	Benign neoplasm of colon	Inverse var	3	-0.0355	0.053982	0.51083666	0.965128	0.868229	1.07284	0.995612
sense organs	Myopia	Inverse var	3	-0.12991	0.1984	0.51261268	0.878177	0.595251	1.295578	0.995612
digestive	Cholelithiasis with acute cholecystitis	Inverse var	3	0.12288	0.18929	0.51623245	1.130749	0.78026	1.638677	0.995612
genitourinary	Cystitis	Inverse var	3	0.084706	0.130766	0.51713381	1.088397	0.84232	1.406364	0.995612
pregnancy complications	Complications of labor and delivery NEC	Inverse var	3	0.096193	0.148746	0.51783186	1.100972	0.822547	1.473641	0.995612
circulatory system	Cardiac pacemaker in situ	Inverse var	3	0.096076	0.148784	0.51844668	1.100843	0.82239	1.473577	0.995612
endocrine/metabolic	Diabetes mellitus	Inverse var	3	-0.03488	0.054053	0.51878153	0.965725	0.868644	1.073654	0.995612
sense organs	Macular degeneration (senile) of retina NOS	Inverse var	3	-0.09866	0.153151	0.51943754	0.906049	0.671099	1.223254	0.995612
digestive	Cholecystitis without cholelithiasis	Inverse var	3	-0.15227	0.236723	0.52008161	0.858761	0.539969	1.365762	0.995612
respiratory	Chronic airway obstruction	Inverse var	3	0.04635	0.072071	0.52015492	1.04744	0.909455	1.206362	0.995612
circulatory system	Cardiomegaly	Inverse var	3	0.089246	0.139775	0.5231519	1.093349	0.831342	1.43793	0.995612
genitourinary	Hypertrophy of breast (Gynecomastia)	Inverse var	3	0.15213	0.238961	0.52436426	1.164312	0.72889	1.859845	0.995612
injuries & poisonings	Anaphylactic shock NOS	Inverse var	3	0.233971	0.368409	0.5253722	1.263608	0.613786	2.601406	0.995612
neoplasms	Benign neoplasm of unspecified sites	Inverse var	3	0.091256	0.144246	0.52696574	1.09555	0.825748	1.453505	0.995612

dermatologic	Cellulitis and abscess of foot, toe	Inverse var	3	-0.05981	0.094729	0.52777363	0.941941	0.782327	1.134119	0.995612
sense organs	Degeneration of macula and posterior pole of retina	Inverse var	3	-0.09557	0.153151	0.5325945	0.908851	0.673174	1.227037	0.995612
infectious diseases	Bacterial enteritis	Inverse var	3	0.083748	0.135237	0.5357407	1.087354	0.834171	1.417382	0.995612
circulatory system	Transient cerebral ischemia	Inverse var	3	0.093767	0.153255	0.54064707	1.098303	0.813335	1.483117	0.995612
musculoskeletal	Hallux rigidus	Inverse var	3	-0.16441	0.269773	0.54223216	0.848394	0.499991	1.439569	0.995612
digestive	Peptic ulcer (excl. esophageal)	Inverse var	3	0.051208	0.084077	0.54248682	1.052541	0.89263	1.2411	0.995612
respiratory	Other diseases of respiratory system, NEC	Inverse var	3	-0.04615	0.076541	0.54657225	0.954902	0.821875	1.10946	0.995612
genitourinary	Irregular menstrual bleeding	Inverse var	3	-0.07036	0.117116	0.54798969	0.932058	0.740887	1.172557	0.995612
endocrine/metabolic	Disorders of adrenal glands	Inverse var	3	-0.16455	0.274382	0.54868858	0.848272	0.495424	1.452424	0.995612
symptoms	Effects of other external causes	Inverse var	3	-0.06534	0.108967	0.54876862	0.936751	0.756605	1.159789	0.995612
circulatory system	Abnormal function study of cardiovascular system	Inverse var	3	0.191606	0.319629	0.54886275	1.211193	0.647352	2.266141	0.995612
genitourinary	Inflammatory diseases of female pelvic organs	Inverse var	3	-0.09385	0.156606	0.54897859	0.910417	0.669784	1.237502	0.995612
dermatologic	Hyperhidrosis	Inverse var	3	0.172801	0.288492	0.54918466	1.18863	0.67527	2.09226	0.995612
infectious diseases	Bacterial infection NOS	Inverse var	3	0.037858	0.063228	0.54933915	1.038584	0.91753	1.175608	0.995612
digestive	Other specified gastritis	Inverse var	3	0.048479	0.08101	0.54955	1.049673	0.895565	1.2303	0.995612
injuries & poisonings	Fracture of radius and ulna	Inverse var	3	-0.08667	0.144889	0.54970079	0.916976	0.690281	1.21812	0.995612
respiratory	Acute and chronic tonsillitis	Inverse var	3	-0.09423	0.157793	0.55037475	0.91007	0.667972	1.239912	0.995612
musculoskeletal	Hallux valgus (Bunion)	Inverse var	3	-0.05365	0.090019	0.5511887	0.947764	0.794465	1.130644	0.995612
digestive	Obstruction of bile duct	Inverse var	3	0.153097	0.257414	0.55201148	1.165438	0.703678	1.930208	0.995612
musculoskeletal	Osteomyelitis	Inverse var	3	0.313122	0.527348	0.55266762	1.367688	0.486519	3.844809	0.995612
genitourinary	Dysuria	Inverse var	3	-0.13404	0.225981	0.5530918	0.874558	0.561604	1.361906	0.995612
digestive	Abnormal findings on exam of gastrointestinal tract/ abdominal area	Inverse var	3	0.109071	0.184037	0.55341098	1.115241	0.777524	1.599646	0.995612
pregnancy complications	Obstetrical/birth trauma	Inverse var	3	0.064033	0.10834	0.55449684	1.066127	0.862161	1.318346	0.995612
musculoskeletal	Other disorders of soft tissues	Inverse var	3	-0.05325	0.090257	0.55520956	0.948144	0.794411	1.131626	0.995612
symptoms	Gangrene	Inverse var	3	-0.17583	0.298399	0.55570623	0.838764	0.467345	1.505366	0.995612
neoplasms	Secondary malignant neoplasm of liver	Inverse var	3	0.078871	0.135467	0.56041838	1.082065	0.82974	1.411124	0.995612
dermatologic	Cellulitis and abscess of leg, except foot	Inverse var	3	-0.055	0.094729	0.56149521	0.946483	0.7861	1.139589	0.995612
neoplasms	Vascular hamartomas and non-neoplastic nevi	Inverse var	3	-0.247	0.426442	0.56245112	0.781143	0.338638	1.801877	0.995612
circulatory system	Rheumatic disease of the heart valves	Inverse var	3	0.074561	0.12888	0.56290712	1.077411	0.836905	1.387032	0.995612
sense organs	Dizziness and giddiness (Light-headedness and vertigo)	Inverse var	3	0.089619	0.155113	0.56342044	1.093758	0.807024	1.482368	0.995612
sense organs	Other retinal disorders	Inverse var	3	-0.06499	0.112748	0.56435435	0.93708	0.751284	1.168825	0.995612
digestive	Intestinal obstruction without mention of hernia	Inverse var	3	0.063829	0.112577	0.57072404	1.065911	0.854857	1.329071	0.995612
respiratory	Empyema and pneumothorax	Inverse var	3	-0.11524	0.20347	0.57115045	0.891155	0.598076	1.327854	0.995612
mental disorders	Mood disorders	Inverse var	3	0.035695	0.063228	0.57238602	1.03634	0.915548	1.173068	0.995612
circulatory system	Paroxysmal tachycardia, unspecified	Inverse var	3	-0.0712	0.126228	0.57272564	0.931278	0.727164	1.192687	0.995612
circulatory system	Primary/intrinsic cardiomyopathies	Inverse var	3	0.161744	0.28768	0.57395589	1.175559	0.668908	2.065961	0.995612
genitourinary	Urinary incontinence	Inverse var	3	0.07425	0.133388	0.57776677	1.077077	0.829286	1.398907	0.995612
digestive	Esophageal bleeding (varices/hemorrhage)	Inverse var	3	0.152177	0.274983	0.57998588	1.164366	0.679234	1.995997	0.995612
neoplasms	Malignant neoplasm of ovary and other uterine adnexa	Inverse var	3	-0.08468	0.153255	0.58058158	0.918807	0.680411	1.240731	0.995612
neoplasms	Neoplasm of unspecified nature of digestive system	Inverse var	3	-0.11812	0.216419	0.58521208	0.888591	0.58141	1.358067	0.995612
mental disorders	Dementias	Inverse var	3	-0.12299	0.225544	0.58554703	0.884273	0.568328	1.375858	0.995612
sense organs	Senile cataract	Inverse var	3	-0.04398	0.08101	0.58716168	0.956969	0.816471	1.121643	0.995612
neurological	Other peripheral nerve disorders	Inverse var	3	-0.03428	0.063228	0.5876963	0.9663	0.853672	1.093788	0.995612
respiratory	Pleurisy; pleural effusion	Inverse var	3	-0.0464	0.085879	0.58896604	0.954657	0.806762	1.129663	0.995612
circulatory system	Other aneurysm	Inverse var	3	0.090129	0.166802	0.58896611	1.094315	0.789147	1.517496	0.995612
respiratory	Cough	Inverse var	3	-0.07041	0.130766	0.59028809	0.932015	0.721294	1.204295	0.995612
injuries & poisonings	Complication of colostomy or enterostomy	Inverse var	3	0.169598	0.316944	0.59257792	1.184829	0.636601	2.20518	0.995612
neoplasms	Lipoma of skin and subcutaneous tissue	Inverse var	3	0.104776	0.197702	0.59613486	1.110461	0.75373	1.636029	0.995612
circulatory system	Heart failure NOS	Inverse var	3	0.100055	0.190493	0.59941417	1.105232	0.760856	1.605479	0.995612
hematopoietic	Iron deficiency anemias	Inverse var	3	-0.06136	0.117129	0.60034485	0.94048	0.747563	1.183183	0.995612
circulatory system	Arterial embolism and thrombosis	Inverse var	3	0.119566	0.229675	0.6026539	1.127008	0.718495	1.767788	0.995612
endocrine/metabolic	Protein-calorie malnutrition	Inverse var	3	-0.11254	0.216419	0.60305772	0.893562	0.584663	1.365665	0.995612
dermatologic	Erythematous conditions	Inverse var	3	0.074805	0.144246	0.60404325	1.077674	0.812275	1.429789	0.995612
mental disorders	Alcoholism	Inverse var	3	0.039658	0.076541	0.60436997	1.040455	0.895509	1.208861	0.995612
circulatory system	Nonspecific abnormal findings on radiological and other examination of other intrathoracic organs (e	Inverse var	3	0.152876	0.297403	0.60722658	1.165181	0.650487	2.087124	0.995612
neoplasms	Multiple myeloma	Inverse var	3	-0.153	0.298399	0.60813007	0.858128	0.478134	1.540119	0.995612
neoplasms	Cancer of esophagus	Inverse var	3	-0.13758	0.269256	0.6093872	0.871468	0.51411	1.477226	0.995612
endocrine/metabolic	Vitamin deficiency	Inverse var	3	-0.14125	0.279004	0.61267283	0.868273	0.502532	1.5002	0.995612
digestive	Ulceration of the lower GI tract	Inverse var	3	-0.10949	0.216419	0.61289918	0.896287	0.586445	1.369829	0.995612
genitourinary	Urethral stricture (not specified as infectious)	Inverse var	3	0.061492	0.121757	0.61352961	1.063422	0.837653	1.350042	0.995612
neoplasms	Leukemia	Inverse var	3	0.086269	0.171505	0.61495748	1.090099	0.778893	1.525648	0.995612
circulatory system	Cardiac pacemaker/device in situ	Inverse var	3	0.07003	0.13994	0.61677046	1.072541	0.815258	1.411019	0.995612
digestive	Acute periodontitis	Inverse var	3	0.156458	0.314893	0.61928785	1.169361	0.630822	2.167657	0.995612
genitourinary	Dyspareunia	Inverse var	3	-0.09954	0.202729	0.62343852	0.905257	0.608423	1.346908	0.995612
circulatory system	Cardiomyopathy	Inverse var	3	0.155776	0.318559	0.62483963	1.168564	0.625879	2.181801	0.995612
neurological	Nerve root and plexus disorders	Inverse var	3	-0.13513	0.276928	0.62557859	0.873603	0.507678	1.503279	0.995612
dermatologic	Sarcoidosis	Inverse var	3	0.147059	0.302561	0.62693388	1.158422	0.640209	2.0961	0.995612
neoplasms	Cancer of bladder	Inverse var	3	-0.07001	0.144246	0.6274031	0.93238	0.702762	1.237022	0.995612
injuries & poisonings	Poisoning by anticonvulsants and anti-Parkinsonism drugs	Inverse var	3	0.150526	0.31084	0.62820349	1.162446	0.632092	2.13779	0.995612
neoplasms	Lymphoid leukemia	Inverse var	3	0.141397	0.292615	0.62894177	1.151882	0.649125	2.044029	0.995612
circulatory system	Precordial pain	Inverse var	3	0.072341	0.150786	0.63139583	1.075022	0.799956	1.44467	0.995612
digestive	Hemorrhage from gastrointestinal ulcer	Inverse var	3	0.13627	0.284351	0.63177418	1.145991	0.656352	2.000904	0.995612
digestive	Nonspecific abnormal findings in stool contents	Inverse var	3	0.082152	0.17144	0.63180432	1.085621	0.775792	1.519186	0.995612
digestive	Irritable Bowel Syndrome	Inverse var	3	0.045401	0.094794	0.63197759	1.046448	0.869015	1.260108	0.995612
dermatologic	Seborrheic keratosis	Inverse var	3	-0.06039	0.126294	0.63251687	0.941395	0.734967	1.205802	0.995612
symptoms	Other ill-defined and unknown causes of morbidity and mortality	Inverse var	3	0.032562	0.068175	0.63291585	1.033098	0.903878	1.180792	0.995612
circulatory system	Heart valve replaced	Inverse var	3	0.085871	0.180382	0.63403786	1.089666	0.765154	1.551806	0.995612
digestive	Gastritis and duodenitis	Inverse var	3	0.031221	0.065585	0.63404424	1.031714	0.90726	1.17324	0.995612
neurological	Abnormality of gait	Inverse var	3	0.083647	0.175811	0.63423371	1.087245	0.770325	1.534549	0.995612
sense organs	Other disorders of tympanic membrane	Inverse var	3	0.090213	0.189711	0.63440868	1.094408	0.754561	1.587318	0.995612
neoplasms	Malignant neoplasm of ovary	Inverse var	3	-0.07275	0.153322	0.63513689	0.929831	0.688484	1.255782	0.995612
neoplasms	Cervical cancer and dysplasia	Inverse var	3	-0.10184	0.215507	0.63653533	0.903176	0.59201	1.377895	0.995612
neurological	Abnormal movement	Inverse var	3	0.063697	0.135237	0.63763633	1.06577	0.817613	1.389246	0.995612
symptoms	Malaise and fatigue	Inverse var	3	0.056778	0.121757	0.64098296	1.058421	0.833714	1.343693	0.995612
genitourinary	Prolapse of vaginal walls	Inverse var	3	-0.03986	0.08555	0.64127292	0.960924	0.812583	1.136347	0.995612
genitourinary	Cyst or abscess of Bartholin's gland	Inverse var	3	0.270396	0.582656	0.64259388	1.310483	0.418278	4.105805	0.995612
sense organs	Corneal opacity and other disorders of cornea	Inverse var	3	-0.12114	0.261301	0.64293262	0.88591	0.530843	1.478473	0.995612
digestive	Disorders of tooth development	Inverse var	3	0.066786	0.144143	0.64312754	1.069066	0.80595	1.418082	0.995612
respiratory	Respiratory insufficiency	Inverse var	3	0.077282	0.166968	0.64346938	1.080346	0.77882	1.498612	0.995612
genitourinary	Inflammatory disease of breast	Inverse var	3	-0.1214	0.264468	0.64620919	0.88568	0.52742	1.487292	0.995612
neurological	Sleep apnea	Inverse var	3	0.07289	0.158791	0.64621169	1.075612	0.787935	1.468322	0.995612
musculoskeletal	Acquired foot deformities	Inverse var	3	-0.03314	0.072238	0.64640354	0.967403	0.839686	1.114545	0.995612
circulatory system	Mitral valve disease	Inverse var	3	0.094657	0.20906	0.65070935	1.099282	0.729716	1.656016	0.995612
musculoskeletal	Displacement of intervertebral disc	Inverse var	3	-0.1399	0.31084	0.65265026	0.869442	0.472768	1.598942	0.995612
circulatory system	Peripheral vascular disease, unspecified	Inverse var	3	0.062908	0.13994	0.65304649	1.064928	0.809471	1.401004	0.995612
neoplasms	Acquired absence of breast	Inverse var	3	-0.0706	0.158592	0.65617838	0.93183	0.682874	1.271549	0.995612
genitourinary	Genital prolapse	Inverse var	3	-0.03006	0.0676	0.65656393	0.970388	0.849969	1.107868	0.995612
sense organs	Suppurative and unspecified otitis media	Inverse var	3	-0.10607	0.238961	0.65713609	0.899364	0.563025	1.436623	0.995612
respiratory	Other symptoms of respiratory system	Inverse var	3	-0.04744	0.106973	0.65743125	0.953669	0.773286	1.17613	0.995612
digestive	Ventral hernia	Inverse var	3	0.053762	0.121586	0.65836573	1.055233	0.831481	1.339197	0.995612
sense organs	Strabismus (not specified as paralytic)	Inverse var	3	0.123632	0.281147	0.66012542	1.131599	0.652192	1.963403	0.995612

dermatologic	Rash and other nonspecific skin eruption	Inverse var	3	0.085279	0.194254	0.66065371	1.089021	0.744191	1.593634	0.995612
infectious diseases	Sepsis	Inverse var	3	-0.10747	0.246422	0.66274826	0.898103	0.554074	1.455744	0.995612
infectious diseases	Sepsis and SIRS	Inverse var	3	-0.10747	0.246422	0.66274826	0.898103	0.554074	1.455744	0.995612
musculoskeletal	Polymyalgia Rheumatica	Inverse var	3	-0.09051	0.207731	0.66303384	0.913461	0.607948	1.372506	0.995612
dermatologic	Diseases of hair and hair follicles	Inverse var	3	-0.04315	0.099028	0.66306402	0.957772	0.788802	1.162938	0.995612
sense organs	Otitis externa	Inverse var	3	0.133519	0.306708	0.66332351	1.142843	0.626486	2.084787	0.995612
injuries & poisonings	Poisoning/allergy of sulfonamides	Inverse var	3	-0.10365	0.238961	0.66447844	0.901544	0.56439	1.440106	0.995612
circulatory system	Carditis	Inverse var	3	0.077723	0.179499	0.66501701	1.080823	0.760259	1.536552	0.995612
congenital anomalies	Digestive congenital anomalies	Inverse var	3	0.114666	0.266135	0.66657302	1.121499	0.665671	1.889461	0.995612
neoplasms	Uterine leiomyoma	Inverse var	3	0.059802	0.139259	0.66761307	1.061626	0.808038	1.394798	0.995612
digestive	GERD	Inverse var	3	-0.02505	0.058754	0.66980612	0.975258	0.869174	1.094289	0.995612
endocrine/metabolic	Disorders of parathyroid gland	Inverse var	3	-0.10186	0.238961	0.66991695	0.903156	0.565399	1.442681	0.995612
sense organs	Aphakia and other disorders of lens	Inverse var	3	-0.07077	0.166802	0.67137538	0.931678	0.671864	1.291965	0.995612
neoplasms	Secondary malignancy of bone	Inverse var	3	0.06478	0.153151	0.67230841	1.066925	0.790257	1.440452	0.995612
endocrine/metabolic	Nontoxic nodular goiter	Inverse var	3	0.087792	0.207731	0.67257041	1.091761	0.726614	1.640406	0.995612
genitourinary	Glomerulonephritis	Inverse var	3	0.093025	0.220647	0.67331663	1.097489	0.712167	1.691291	0.995612
injuries & poisonings	Effects radiation NOS	Inverse var	3	0.053154	0.126228	0.67368561	1.054592	0.82345	1.350615	0.995612
genitourinary	Cystic mastopathy	Inverse var	3	-0.09649	0.229675	0.67441165	0.908022	0.578886	1.424294	0.995612
dermatologic	Cellulitis and abscess of trunk	Inverse var	3	0.120881	0.288492	0.67520735	1.128491	0.641105	1.986402	0.995612
musculoskeletal	Osteoarthritis; localized	Inverse var	3	-0.02271	0.054218	0.67538209	0.977551	0.878998	1.087153	0.995612
dermatologic	Symptoms affecting skin	Inverse var	3	0.039627	0.094729	0.67571263	1.040423	0.864121	1.252694	0.995612
symptoms	Back pain	Inverse var	3	-0.02815	0.067701	0.67753	0.97224	0.851423	1.110202	0.995612
neoplasms	Cancer of bronchus; lung	Inverse var	3	0.068429	0.164685	0.6777667	1.070824	0.775417	1.478771	0.995612
digestive	Diseases of lips	Inverse var	3	0.112095	0.270267	0.67832111	1.118619	0.658607	1.899933	0.995612
pregnancy complications	Normal delivery	Inverse var	3	-0.0815	0.197771	0.68027338	0.921733	0.625546	1.358162	0.995612
musculoskeletal	Osteoporosis NOS	Inverse var	3	0.039018	0.094794	0.68062771	1.039789	0.863485	1.25209	0.995612
injuries & poisonings	Opiates and related narcotics causing adverse effects in therapeutic use	Inverse var	3	0.085278	0.207731	0.6814216	1.08902	0.72479	1.636289	0.995612
genitourinary	Symptoms involving female genital tract	Inverse var	3	-0.05592	0.137242	0.68367575	0.945615	0.72259	1.237477	0.995612
musculoskeletal	Osteoarthrosis NOS	Inverse var	3	0.027441	0.067348	0.68367743	1.027821	0.900719	1.172858	0.995612
neurological	Other headache syndromes	Inverse var	3	0.032759	0.08108	0.68618998	1.033301	0.881475	1.211278	0.995612
respiratory	Postinflammatory pulmonary fibrosis	Inverse var	3	0.094741	0.234816	0.68660308	1.099374	0.693851	1.741908	0.995612
neoplasms	Cancer of mouth	Inverse var	3	0.119389	0.29896	0.68963564	1.126809	0.627149	2.024556	0.995612
musculoskeletal	Synovitis and tenosynovitis	Inverse var	3	0.104631	0.262005	0.68963677	1.110301	0.664382	1.855513	0.995612
symptoms	Fever of unknown origin	Inverse var	3	0.053841	0.134938	0.6898915	1.055317	0.810067	1.374816	0.995612
injuries & poisonings	Open wounds of extremities	Inverse var	3	-0.08071	0.202513	0.69023135	0.922461	0.620248	1.371927	0.995612
digestive	Periapical abscess	Inverse var	3	0.080734	0.202826	0.69059541	1.084083	0.728473	1.613286	0.995612
sense organs	Diplopia and disorders of binocular vision	Inverse var	3	-0.10398	0.261301	0.69068402	0.901245	0.540031	1.504064	0.995612
infectious diseases	Intestinal infection	Inverse var	3	0.030394	0.076541	0.69129564	1.030861	0.887252	1.197714	0.995612
endocrine/metabolic	Chondrocalcinosis	Inverse var	3	-0.11778	0.297403	0.6920741	0.888888	0.49624	1.592216	0.995612
neoplasms	Pancreatic cancer	Inverse var	3	-0.11388	0.288492	0.69302192	0.892361	0.506958	1.570759	0.995612
circulatory system	Congestive heart failure; nonhypertensive	Inverse var	3	0.061519	0.15611	0.69352425	1.063451	0.78313	1.444112	0.995612
sense organs	Vertiginous syndromes and other disorders of vestibular system	Inverse var	3	0.034865	0.090257	0.69928465	1.03548	0.867587	1.235863	0.995612
sense organs	Primary angle-closure glaucoma	Inverse var	3	-0.10216	0.266135	0.70106767	0.902882	0.53591	1.521142	0.995612
infectious diseases	Viral Enteritis	Inverse var	3	-0.10747	0.280188	0.70131109	0.898107	0.518593	1.555353	0.995612
mental disorders	Bipolar	Inverse var	3	-0.08158	0.216419	0.70622281	0.921663	0.603049	1.408613	0.995612
digestive	Diseases of the oral soft tissues, excluding lesions specific for gingiva and tongue	Inverse var	3	0.042189	0.112748	0.7082631	1.043092	0.836276	1.301054	0.995612
injuries & poisonings	Fracture of neck of femur	Inverse var	3	0.061436	0.166802	0.71263772	1.063362	0.766825	1.474573	0.995612
genitourinary	Renal failure	Inverse var	3	0.031463	0.08555	0.71304469	1.031963	0.872655	1.220354	0.995612
endocrine/metabolic	Acid-base balance disorder	Inverse var	3	-0.10187	0.278995	0.71502409	0.903151	0.522727	1.560435	0.995612
genitourinary	Infertility, female	Inverse var	3	0.070462	0.194061	0.71653459	1.073004	0.733522	1.569602	0.995612
circulatory system	Pericarditis	Inverse var	3	0.09419	0.260287	0.71744802	1.098769	0.659699	1.830066	0.995612
circulatory system	Cerebral artery occlusion, with cerebral infarction	Inverse var	3	0.073271	0.203251	0.71847796	1.076022	0.722454	1.602626	0.995612
musculoskeletal	Other unspecified back disorders	Inverse var	3	-0.06304	0.175811	0.71993354	0.938909	0.665227	1.325187	0.995612
injuries & poisonings	Fracture of hand or wrist	Inverse var	3	-0.07348	0.204965	0.71996038	0.929152	0.621752	1.388535	0.995612
neoplasms	Lipoma	Inverse var	3	0.060645	0.169556	0.72059169	1.062522	0.762094	1.481382	0.995612
digestive	Duodenitis	Inverse var	3	0.071808	0.201119	0.72105952	1.074449	0.724419	1.593609	0.995612
sense organs	Infection of the eye	Inverse var	3	-0.15284	0.428994	0.72164209	0.858271	0.370218	1.989717	0.995612
infectious diseases	Streptococcus infection	Inverse var	3	-0.06053	0.171505	0.72412238	0.941262	0.672547	1.317343	0.995612
neoplasms	Benign neoplasm of uterus	Inverse var	3	0.051393	0.146502	0.72573625	1.052737	0.789978	1.402893	0.995612
hematopoietic	Iron deficiency anemias, unspecified or not due to blood loss	Inverse var	3	-0.0387	0.110545	0.72629435	0.962042	0.774634	1.19479	0.995612
neurological	Extrapyramidal disease and abnormal movement disorders	Inverse var	3	0.102534	0.296224	0.72923933	1.107975	0.619982	1.980074	0.995612
dermatologic	Degenerative skin conditions and other dermatoses	Inverse var	3	-0.03253	0.094794	0.73148422	0.967995	0.803864	1.165637	0.995612
musculoskeletal	Intervertebral disc disorders	Inverse var	3	-0.03267	0.097046	0.73636455	0.967855	0.800208	1.170625	0.995612
genitourinary	Disorders of uterus, NEC	Inverse var	3	0.072145	0.215641	0.73795708	1.074811	0.704328	1.640171	0.995612
musculoskeletal	Other specified osteoporosis	Inverse var	3	0.102109	0.306708	0.73919511	1.107504	0.607114	2.020322	0.995612
dermatologic	Pruritus and related conditions	Inverse var	3	0.083791	0.252033	0.73954059	1.087402	0.663522	1.78207	0.995612
digestive	Appendicitis	Inverse var	3	-0.05611	0.170151	0.74156182	0.945432	0.677321	1.319671	0.995612
digestive	Dysphagia	Inverse var	3	0.029567	0.090089	0.74276627	1.030008	0.863287	1.228927	0.995612
injuries & poisonings	Complication due to other implant and internal device	Inverse var	3	0.041177	0.126055	0.74392895	1.042036	0.813921	1.334084	0.995612
circulatory system	Nonrheumatic mitral valve disorders	Inverse var	3	0.075825	0.233113	0.74497557	1.078774	0.683125	1.703572	0.995612
respiratory	Epistaxis or throat hemorrhage	Inverse var	3	0.051525	0.158675	0.74539454	1.052875	0.771454	1.436956	0.995612
neoplasms	Nevus, non-neoplastic	Inverse var	3	-0.1261	0.389462	0.74609915	0.881524	0.410883	1.891258	0.995612
neoplasms	Secondary malignancy of brain/spine	Inverse var	3	-0.08011	0.247911	0.74659152	0.923016	0.567784	1.500496	0.995612
musculoskeletal	Osteoarthrosis, generalized	Inverse var	3	-0.09285	0.288492	0.74757457	0.911332	0.517735	1.604152	0.995612
respiratory	Septal Deviations/Turbinate Hypertrophy	Inverse var	3	0.038904	0.121466	0.74875191	1.03967	0.819412	1.319135	0.995612
hematopoietic	Anemia of chronic disease	Inverse var	3	0.10654	0.334257	0.74992512	1.112423	0.577757	2.141879	0.995612
dermatologic	Atopic/contact dermatitis due to other or unspecified	Inverse var	3	0.04847	0.153322	0.75190074	1.049664	0.777213	1.417622	0.995612
musculoskeletal	Acquired spondylolisthesis	Inverse var	3	-0.05685	0.180282	0.75249422	0.944734	0.663514	1.345143	0.995612
musculoskeletal	Pathologic fracture	Inverse var	3	0.097969	0.31084	0.75262868	1.102929	0.599729	2.028335	0.995612
neurological	Multiple sclerosis	Inverse var	3	0.068456	0.21745	0.75290549	1.070853	0.69925	1.639938	0.995612
digestive	Malposition and malpresentation of fetus or obstruction	Inverse var	3	-0.05531	0.175942	0.75324995	0.946193	0.670217	1.335809	0.995612
injuries & poisonings	Foreign body injury	Inverse var	3	-0.06368	0.202628	0.75332668	0.938308	0.630761	1.395809	0.995612
digestive	Anal and rectal polyp	Inverse var	3	-0.02543	0.081302	0.75446712	0.974893	0.831288	1.143306	0.995612
respiratory	Voice disturbance	Inverse var	3	-0.06548	0.21208	0.75750657	0.936617	0.618068	1.419345	0.995612
neoplasms	Secondary malignancy of respiratory organs	Inverse var	3	-0.04534	0.148949	0.76082512	0.955673	0.713709	1.279668	0.995612
infectious diseases	Viral hepatitis	Inverse var	3	0.125701	0.414832	0.76187797	1.133943	0.502897	2.556838	0.995612
hematopoietic	Diseases of spleen	Inverse var	3	-0.16595	0.548523	0.76223886	0.847088	0.289079	2.482219	0.995612
injuries & poisonings	Crushing or internal injury to organs	Inverse var	3	-0.11231	0.372687	0.76315076	0.893769	0.430514	1.855509	0.995612
dermatologic	Other hypertrophic and atrophic conditions of skin	Inverse var	3	-0.04573	0.152547	0.76435328	0.955301	0.708418	1.288222	0.995612
digestive	Heartburn	Inverse var	3	-0.04594	0.153322	0.76447256	0.955102	0.707196	1.289912	0.995612
respiratory	Abnormal sputum	Inverse var	3	-0.0458	0.153082	0.76480972	0.955235	0.707627	1.289486	0.995612
musculoskeletal	Unspecified polyarthropathy or polyarthritis	Inverse var	3	0.036296	0.121586	0.76530512	1.036963	0.817085	1.31601	0.995612
respiratory	Emphysema	Inverse var	3	0.050853	0.171273	0.7665342	1.052168	0.752133	1.471891	0.995612
respiratory	Other diseases of lung	Inverse var	3	-0.07466	0.252033	0.7670631	0.928062	0.566294	1.520938	0.995612
hematopoietic	Lymphadenitis	Inverse var	3	0.040109	0.135467	0.76717115	1.040924	0.798192	1.357471	0.995612
musculoskeletal	Unspecified monoarthritis	Inverse var	3	-0.02905	0.098122	0.76717184	0.971366	0.80142	1.177351	0.995612
congenital anomalies	Other congenital musculoskeletal anomalies	Inverse var	3	-0.08528	0.288492	0.76752744	0.918254	0.521668	1.616337	0.995612
symptoms	Nausea and vomiting	Inverse var	3	0.029762	0.100749	0.76768487	1.030209	0.845602	1.255119	0.995612
digestive	Diaphragmatic hernia	Inverse var	3	0.013243	0.045208	0.76956997	1.013331	0.927406	1.107218	0.995612
musculoskeletal	Osteoporosis	Inverse var	3	0.026167	0.090089	0.77147048	1.026512	0.860357	1.224755	0.995612
neurological	Other paralytic syndromes	Inverse var	3	0.140728	0.485791	0.77205448	1.151112	0.444226	2.982845	0.995612

circulatory system	Other forms of chronic heart disease	Inverse var	3	0.04831	0.166802	0.772102	1.049496	0.756826	1.455344	0.995612
symptoms	Other symptoms	Inverse var	3	0.063194	0.220306	0.77423149	1.065233	0.691698	1.640487	0.995612
neoplasms	Malignant neoplasm of uterus	Inverse var	3	-0.05664	0.1983	0.77514971	0.944931	0.640625	1.393787	0.995612
neurological	Convulsions	Inverse var	3	0.064189	0.235984	0.78561661	1.066294	0.671434	1.693365	0.995612
respiratory	Other diseases of respiratory system, not elsewhere classified	Inverse var	3	-0.0215	0.080102	0.7883612	0.978727	0.836522	1.145106	0.995612
musculoskeletal	Rheumatoid arthritis and other inflammatory polyarthropathies	Inverse var	3	0.033973	0.127237	0.78946464	1.034557	0.80621	1.327579	0.995612
sense organs	Other disorders of eyelids	Inverse var	3	-0.02524	0.094559	0.78955018	0.975078	0.81012	1.173626	0.995612
neoplasms	Large cell lymphoma	Inverse var	3	0.150132	0.564446	0.79025393	1.161988	0.384357	3.512917	0.995612
musculoskeletal	Kyphoscoliosis and scoliosis	Inverse var	3	0.057493	0.216419	0.79050457	1.059178	0.693026	1.618781	0.995612
congenital anomalies	Cardiac congenital anomalies	Inverse var	3	-0.03705	0.139604	0.7906873	0.963625	0.732951	1.266896	0.995612
mental disorders	Anxiety disorders	Inverse var	3	0.022602	0.08572	0.79202791	1.02286	0.864669	1.209992	0.995612
neoplasms	Cancer of larynx, pharynx, nasal cavities	Inverse var	3	-0.08784	0.334049	0.79257717	0.915904	0.475885	1.762781	0.995612
circulatory system	Paroxysmal ventricular tachycardia	Inverse var	3	0.05981	0.229675	0.79454647	1.061635	0.676818	1.665246	0.995612
injuries & poisonings	Poisoning by analgesics, antipyretics, and antirheumatics	Inverse var	3	-0.03333	0.128037	0.79461071	0.967218	0.752553	1.243115	0.995612
digestive	Chronic liver disease and cirrhosis	Inverse var	3	0.05019	0.193719	0.79556852	1.051471	0.719283	1.537073	0.995612
neurological	Abnormal involuntary movements	Inverse var	3	0.060566	0.234816	0.79646249	1.062437	0.670538	1.683383	0.995612
sense organs	Disorders of refraction and accommodation; blindness and low vision	Inverse var	3	-0.03858	0.149596	0.79650024	0.962157	0.717641	1.289984	0.995612
genitourinary	Irregular menstrual cycle	Inverse var	3	-0.04131	0.160855	0.79733527	0.959534	0.700065	1.315173	0.995612
mental disorders	Phobia	Inverse var	3	0.079781	0.31084	0.79744023	1.083049	0.58892	1.991776	0.995612
hematopoietic	Coagulation defects	Inverse var	3	0.14762	0.575515	0.79756441	1.159072	0.375165	3.580954	0.995612
injuries & poisonings	Dislocation	Inverse var	3	-0.03914	0.153322	0.79850798	0.961616	0.712019	1.29871	0.995612
musculoskeletal	Symptoms and disorders of the joints	Inverse var	3	-0.02991	0.117219	0.79856813	0.970529	0.771312	1.2212	0.995612
injuries & poisonings	Fracture of ankle and foot	Inverse var	3	0.069971	0.274297	0.79865248	1.072477	0.626472	1.836006	0.995612
pregnancy complications	Antepartum hemorrhage, abruptio placentae, and placenta previa	Inverse var	3	-0.06213	0.24377	0.79883538	0.939765	0.582798	1.515375	0.995612
musculoskeletal	Aseptic necrosis of bone	Inverse var	3	0.117808	0.467018	0.80084318	1.125028	0.450433	2.809938	0.995612
digestive	Esophagitis, GERD and related diseases	Inverse var	3	-0.01093	0.043383	0.80109639	0.98913	0.908501	1.076915	0.995612
digestive	Duodenal ulcer	Inverse var	3	0.040508	0.161099	0.80146946	1.041339	0.759385	1.427982	0.995612
musculoskeletal	Joint effusions	Inverse var	3	-0.04602	0.18471	0.80323627	0.955021	0.664943	1.371642	0.995612
pregnancy complications	Early or threatened labor; hemorrhage in early pregnancy	Inverse var	3	0.035474	0.144377	0.80591463	1.03611	0.780745	1.374999	0.995612
circulatory system	Heart valve disorders	Inverse var	3	0.030368	0.124121	0.80671682	1.030834	0.808229	1.314749	0.995612
endocrine/metabolic	Thyrotoxicosis with or without goiter	Inverse var	3	0.065164	0.266488	0.80682149	1.067334	0.633084	1.799448	0.995612
infectious diseases	Septicemia	Inverse var	3	-0.03516	0.144686	0.80799878	0.965451	0.727062	1.282003	0.995612
dermatologic	Sebaceous cyst	Inverse var	3	-0.02189	0.090858	0.80960353	0.978347	0.818752	1.16905	0.995612
genitourinary	Other disorders of male genital organs	Inverse var	3	-0.04573	0.190163	0.8099675	0.955302	0.658067	1.38679	0.995612
musculoskeletal	Osteoporosis, osteopenia and pathological fracture	Inverse var	3	0.019185	0.081248	0.81333397	1.01937	0.869305	1.19534	0.996306
symptoms	Cervicalgia	Inverse var	3	-0.03918	0.166802	0.81430036	0.961579	0.693426	1.333429	0.996306
musculoskeletal	Osteoarthrosis, localized, primary	Inverse var	3	-0.01825	0.079201	0.81777988	0.981918	0.840733	1.146812	0.996306
digestive	Disorders of esophageal motility	Inverse var	3	-0.06575	0.285793	0.81805515	0.936369	0.53478	1.639528	0.996306
digestive	Symptoms involving digestive system	Inverse var	3	-0.0133	0.058523	0.82017634	0.986785	0.879846	1.106721	0.996306
digestive	Stricture and stenosis of esophagus	Inverse var	3	-0.04416	0.194466	0.82037075	0.956804	0.653567	1.400734	0.996306
pregnancy complications	Early onset of delivery	Inverse var	3	0.065455	0.288492	0.8205106	1.067645	0.606538	1.879299	0.996306
digestive	Other intestinal obstruction	Inverse var	3	0.027064	0.121757	0.8240978	1.027433	0.809305	1.304353	0.996306
symptoms	Pain	Inverse var	3	0.055735	0.252033	0.82498214	1.057317	0.645165	1.732766	0.996306
musculoskeletal	Other disorders of bone and cartilage	Inverse var	3	0.0313	0.141709	0.82518857	1.031795	0.781571	1.36213	0.996306
dermatologic	Decubitus ulcer	Inverse var	3	-0.09328	0.429694	0.82813977	0.910937	0.392397	2.114712	0.996306
digestive	Blood in stool	Inverse var	3	-0.04134	0.19309	0.8304522	0.959498	0.657177	1.400895	0.996306
digestive	Diseases of the jaws	Inverse var	3	-0.04797	0.225544	0.83158294	0.953165	0.612606	1.483049	0.996306
injuries & poisonings	Fracture of vertebral column without mention of spinal cord injury	Inverse var	3	0.040297	0.189711	0.83178616	1.04112	0.71782	1.51003	0.996306
injuries & poisonings	Adverse effects of sedatives or other central nervous system depressants and anesthetics	Inverse var	3	0.075136	0.359089	0.83425939	1.078031	0.533296	2.179185	0.996306
dermatologic	Unspecified diffuse connective tissue disease	Inverse var	3	0.028126	0.135304	0.83532894	1.028525	0.788937	1.340873	0.996306
neoplasms	Myeloproliferative disease	Inverse var	3	0.049856	0.242983	0.83742752	1.05112	0.652862	1.692324	0.996306
musculoskeletal	Acquired toe deformities	Inverse var	3	-0.02585	0.126447	0.83804437	0.974486	0.760574	1.24856	0.996306
genitourinary	Chronic renal failure [CKD]	Inverse var	3	0.027575	0.135467	0.83870042	1.027959	0.78825	1.340563	0.996306
neoplasms	Malignant neoplasm, other	Inverse var	3	0.011885	0.058412	0.83876782	1.011956	0.902486	1.134704	0.996306
mental disorders	Anxiety disorder	Inverse var	3	0.018133	0.090191	0.84065964	1.018298	0.853302	1.215198	0.996306
genitourinary	Noninflammatory female genital disorders	Inverse var	3	-0.02654	0.134628	0.84372454	0.97381	0.747957	1.267861	0.996306
musculoskeletal	Arthropathy NOS	Inverse var	3	-0.00821	0.042138	0.84558443	0.991827	0.913203	1.077221	0.996306
congenital anomalies	Cardiac and circulatory congenital anomalies	Inverse var	3	-0.02603	0.135134	0.84723037	0.974302	0.747594	1.26976	0.996306
dermatologic	Actinic keratosis	Inverse var	3	0.027815	0.149234	0.8521439	1.028205	0.767449	1.377559	0.996306
dermatologic	Diseases of sebaceous glands	Inverse var	3	-0.01661	0.089338	0.85251031	0.983528	0.825544	1.171745	0.996306
musculoskeletal	Osteoarthrosis	Inverse var	3	-0.00829	0.04557	0.85565852	0.991745	0.907005	1.084402	0.996306
circulatory system	Endocarditis	Inverse var	3	0.048349	0.266135	0.855841	1.049537	0.622958	1.768222	0.996306
injuries & poisonings	Open wounds of head; neck; and trunk	Inverse var	3	0.030525	0.168033	0.85584783	1.030996	0.741694	1.433142	0.996306
sense organs	Subjective visual disturbances	Inverse var	3	-0.08519	0.469561	0.856026	0.918333	0.365849	2.305147	0.996306
symptoms	Sciatica	Inverse var	3	-0.02585	0.144313	0.85784941	0.974483	0.7344	1.293051	0.996306
circulatory system	Atherosclerosis	Inverse var	3	0.034264	0.194061	0.85985344	1.034857	0.707445	1.5138	0.996306
mental disorders	Aphasia/speech disturbance	Inverse var	3	0.031679	0.180382	0.8605904	1.032186	0.724793	1.469949	0.996306
dermatologic	Scar conditions and fibrosis of skin	Inverse var	3	-0.04578	0.264408	0.86254758	0.955254	0.568918	1.60394	0.996306
hematopoietic	Thrombocytopenia	Inverse var	3	-0.03	0.175978	0.86462259	0.970443	0.687345	1.37014	0.996306
symptoms	Symptoms of the muscles	Inverse var	3	0.04509	0.266135	0.86546199	1.046122	0.620931	1.762469	0.996306
musculoskeletal	Osteopenia or other disorder of bone and cartilage	Inverse var	3	0.040975	0.24377	0.86651227	1.041826	0.646092	1.679951	0.996306
congenital anomalies	Congenital anomalies of genital organs	Inverse var	3	0.070672	0.422224	0.86706978	1.07323	0.469125	2.455255	0.996306
genitourinary	Inflammatory disease of cervix, vagina, and vulva	Inverse var	3	0.041328	0.247539	0.86740428	1.042194	0.641563	1.693005	0.996306
neoplasms	Hemangioma and lymphangioma, any site	Inverse var	3	-0.03049	0.18805	0.87118471	0.969967	0.670943	1.402261	0.996306
dermatologic	Psoriasis vulgaris	Inverse var	3	-0.02754	0.17144	0.87235485	0.972831	0.695192	1.361351	0.996306
mental disorders	Alcoholic liver damage	Inverse var	3	-0.03951	0.247911	0.87337823	0.961261	0.59131	1.56267	0.996306
digestive	Abdominal hernia	Inverse var	3	-0.00549	0.034727	0.87443015	0.994527	0.929087	1.064576	0.996306
musculoskeletal	Malunion and nonunion of fracture	Inverse var	3	0.031212	0.1983	0.87493139	1.031704	0.699454	1.521778	0.996306
dermatologic	Pilonidal cyst	Inverse var	3	0.045328	0.292615	0.8768944	1.046371	0.589667	1.8568	0.996306
respiratory	Chronic sinusitis	Inverse var	3	0.021306	0.139707	0.87879078	1.021534	0.776841	1.343302	0.996306
circulatory system	Noninfectious disorders of lymphatic channels	Inverse var	3	0.049679	0.330042	0.88035225	1.050934	0.550348	2.006841	0.996306
digestive	Other chronic nonalcoholic liver disease	Inverse var	3	0.025771	0.171505	0.88055478	1.026106	0.733169	1.436087	0.996306
symptoms	Symptoms concerning nutrition, metabolism, and development	Inverse var	3	-0.01413	0.094794	0.88147704	0.985966	0.818788	1.187277	0.996306
mental disorders	Neurological disorders	Inverse var	3	0.015467	0.103739	0.88148146	1.015587	0.82873	1.244575	0.996306
musculoskeletal	Curvature of spine	Inverse var	3	0.030233	0.207829	0.88434009	1.030694	0.685839	1.54895	0.996306
mental disorders	Altered mental status	Inverse var	3	-0.0347	0.240468	0.88526311	0.965896	0.602893	1.547463	0.996306
injuries & poisonings	Fracture of ribs	Inverse var	3	-0.03552	0.252033	0.88793591	0.965108	0.588899	1.58165	0.996306
hematopoietic	Pernicious anemia	Inverse var	3	-0.03583	0.257167	0.88919882	0.964806	0.582821	1.597147	0.996306
digestive	Portal hypertension	Inverse var	3	0.09344	0.671158	0.88927473	1.097945	0.294631	4.091493	0.996306
dermatologic	Disturbance of skin sensation	Inverse var	3	0.018065	0.130766	0.89012516	1.018229	0.788016	1.315696	0.996306
genitourinary	Other specified benign mammary dysplasias	Inverse var	3	-0.05164	0.380049	0.8919174	0.94967	0.450888	2.000215	0.996306
infectious diseases	Infection/inflammation of internal prosthetic device; implant; and graft	Inverse var	3	0.019	0.13994	0.89200142	1.019182	0.774698	1.34082	0.996306
infectious diseases	Staphylococcus infections	Inverse var	3	-0.01684	0.126228	0.89389457	0.983305	0.767788	1.259318	0.996306
musculoskeletal	Other acquired deformities of limbs	Inverse var	3	-0.02266	0.171505	0.89490403	0.977599	0.69851	1.368198	0.996306
hematopoietic	Other diseases of blood and blood-forming organs	Inverse var	3	-0.01406	0.108276	0.89671571	0.986043	0.797498	1.219164	0.996306
digestive	Appendiceal conditions	Inverse var	3	-0.02514	0.193679	0.8967366	0.975177	0.667145	1.425431	0.996306
mental disorders	Memory loss	Inverse var	3	0.034524	0.266135	0.89678406	1.035127	0.614405	1.743945	0.996306
genitourinary	Polyp of female genital organs	Inverse var	3	0.008886	0.069066	0.89763268	1.008925	0.881188	1.155179	0.996306
neoplasms	Colon cancer	Inverse var	3	-0.01673	0.130595	0.89803831	0.983405	0.761321	1.270272	0.996306
digestive	Ulceration of intestine	Inverse var	3	-0.03459	0.270267	0.89815998	0.966001	0.56875	1.640717	0.996306
neurological	Disorders of other cranial nerves	Inverse var	3	0.025317	0.197991	0.89825263	1.02564	0.695764	1.511918	0.996306

circulatory system	Occlusion and stenosis of precerebral arteries	Inverse var	3	0.025644	0.202729	0.89934002	1.025976	0.689558	1.526523	0.996306
mental disorders	Schizophrenia	Inverse var	3	0.036497	0.292615	0.90073948	1.037171	0.584482	1.840474	0.996306
symptoms	Other tests	Inverse var	3	-0.0124	0.100016	0.90134277	0.987678	0.811858	1.201574	0.996306
respiratory	Diseases of the larynx and vocal cords	Inverse var	3	-0.0167	0.135467	0.90189692	0.98344	0.754113	1.282507	0.996306
neurological	Facial nerve disorders [CN7]	Inverse var	3	-0.02938	0.238961	0.90214673	0.971047	0.607901	1.551129	0.996306
symptoms	Chronic fatigue syndrome	Inverse var	3	-0.03359	0.288492	0.90730458	0.966966	0.549341	1.702081	0.998055
mental disorders	Psychogenic and somatoform disorders	Inverse var	3	0.035282	0.306708	0.90841879	1.035911	0.567868	1.889721	0.998055
digestive	Diseases of esophagus	Inverse var	3	-0.00624	0.055043	0.90975684	0.993781	0.892148	1.106991	0.998055
sense organs	Retinal vascular changes and abnomalities	Inverse var	3	-0.02979	0.269807	0.91208735	0.970651	0.572003	1.647129	0.998055
genitourinary	Chronic glomerulonephritis, NOS	Inverse var	3	-0.02667	0.24377	0.91289285	0.973686	0.603834	1.570074	0.998055
sense organs	Hearing loss	Inverse var	3	-0.01175	0.108276	0.91359573	0.98832	0.79934	1.22198	0.998055
respiratory	Respiratory abnormalities	Inverse var	3	0.030224	0.284351	0.91535148	1.030685	0.590312	1.799579	0.998055
genitourinary	Noninflammatory disorders of ovary, fallopian tube, and broad ligament	Inverse var	3	-0.057	0.541924	0.9162348	0.944596	0.326551	2.732381	0.998055
congenital anomalies	Cardiac shunt/ heart septal defect	Inverse var	3	-0.03918	0.378678	0.91760198	0.961581	0.457772	2.019869	0.998055
hematopoietic	Other anemias	Inverse var	3	0.006298	0.063228	0.92065216	1.006318	0.889025	1.139086	0.998055
circulatory system	Palpitations	Inverse var	3	0.011163	0.112937	0.92126226	1.011226	0.810428	1.261775	0.998055
injuries & poisonings	Poisoning by other anti-infectives	Inverse var	3	-0.01862	0.1984	0.92522677	0.981552	0.665321	1.448089	0.998055
genitourinary	stress incontinence, female	Inverse var	3	-0.01492	0.159319	0.92540539	0.985194	0.720953	1.346284	0.998055
digestive	Gastric ulcer	Inverse var	3	0.010217	0.112577	0.92768381	1.01027	0.810233	1.259693	0.998055
neurological	Other cerebral degenerations	Inverse var	3	0.02677	0.297967	0.92841288	1.027131	0.572785	1.841876	0.998055
genitourinary	Uterine/Uterovaginal prolapse	Inverse var	3	0.008665	0.099028	0.93027506	1.008702	0.830747	1.224778	0.998055
neoplasms	Secondary malignancy of lymph nodes	Inverse var	3	0.008219	0.09489	0.93097532	1.008253	0.837139	1.214343	0.998055
injuries & poisonings	Poisoning by psychotropic agents	Inverse var	3	-0.01398	0.16216	0.93131881	0.986121	0.717624	1.355077	0.998055
genitourinary	Cervicitis and endocervicitis	Inverse var	3	-0.02085	0.244648	0.93207288	0.979363	0.606311	1.581947	0.998055
musculoskeletal	Other arthropathies	Inverse var	3	-0.00337	0.040095	0.93300219	0.996635	0.921312	1.078116	0.998055
digestive	Disturbances in tooth eruption	Inverse var	3	0.011493	0.144313	0.93652579	1.011559	0.762342	1.342248	0.998055
dermatologic	Disorder of skin and subcutaneous tissue NOS	Inverse var	3	0.007175	0.094559	0.93951896	1.0072	0.836808	1.212289	0.998055
circulatory system	Atrioventricular block, complete	Inverse var	3	0.026171	0.346783	0.93984272	1.026516	0.520209	2.025601	0.998055
symptoms	Symptoms involving skin and other integumentary tissue	Inverse var	3	0.012394	0.166734	0.94074263	1.012472	0.730224	1.403814	0.998055
genitourinary	Noninflammatory disorders of vulva and perineum	Inverse var	3	-0.01387	0.189711	0.94171902	0.986226	0.679973	1.430413	0.998055
neoplasms	Cancer of stomach	Inverse var	3	-0.02174	0.298399	0.94191751	0.978493	0.545199	1.756145	0.998055
musculoskeletal	Pain in joint	Inverse var	3	0.008178	0.116809	0.94418231	1.008212	0.801903	1.267598	0.998055
injuries & poisonings	Mechanical complication of unspecified genitourinary device, implant, and graft	Inverse var	3	-0.01954	0.287795	0.94588232	0.980654	0.557879	1.723821	0.998055
endocrine/metabolic	Crystal arthropathies	Inverse var	3	-0.01925	0.284351	0.94602792	0.980935	0.561817	1.712715	0.998055
digestive	Liver abscess and sequelae of chronic liver disease	Inverse var	3	0.041581	0.625871	0.94702962	1.042458	0.305707	3.554766	0.998055
musculoskeletal	Enthesopathy	Inverse var	3	-0.00462	0.072238	0.94895304	0.995386	0.863975	1.146785	0.998055
circulatory system	Aneurysm and dissection of heart	Inverse var	3	0.02443	0.395246	0.95071426	1.024731	0.472248	2.223565	0.998055
injuries & poisonings	Nonspecific abnormal findings on radiological and other examination of musculoskeletal system	Inverse var	3	-0.01607	0.266135	0.95184105	0.984055	0.584091	1.657901	0.998055
respiratory	Hemoptysis	Inverse var	3	0.00915	0.157579	0.95369611	1.009192	0.741037	1.374384	0.998055
endocrine/metabolic	Anorexia	Inverse var	3	-0.01384	0.24377	0.95470965	0.986251	0.611626	1.590335	0.998055
genitourinary	Retention of urine	Inverse var	3	0.004577	0.087673	0.95836821	1.004587	0.845977	1.192935	0.998055
respiratory	Shortness of breath	Inverse var	3	-0.00582	0.11229	0.95867015	0.994198	0.797792	1.238957	0.998055
circulatory system	Abdominal aortic aneurysm	Inverse var	3	-0.01188	0.234816	0.95965821	0.988193	0.62368	1.565746	0.998055
injuries & poisonings	Fracture of unspecified bones	Inverse var	3	0.009759	0.194061	0.95989318	1.009807	0.69032	1.477156	0.998055
digestive	Anal and rectal conditions	Inverse var	3	-0.00287	0.058523	0.9609299	0.997137	0.889076	1.118332	0.998055
injuries & poisonings	Superficial injury without mention of infection	Inverse var	3	0.009855	0.21066	0.96268578	1.009904	0.668287	1.52615	0.998055
mental disorders	Schizophrenia and other psychotic disorders	Inverse var	3	0.013659	0.296512	0.96325696	1.013753	0.566938	1.812711	0.998055
musculoskeletal	Other disorders of cervical region	Inverse var	3	0.013405	0.306708	0.9651385	1.013495	0.55558	1.848829	0.998055
sense organs	Perforation of tympanic membrane	Inverse var	3	0.009303	0.216517	0.96572798	1.009346	0.660294	1.542919	0.998055
digestive	Peritonitis and retroperitoneal infections	Inverse var	3	0.009415	0.234816	0.96801777	1.009459	0.637102	1.599442	0.998055
pregnancy complications	Hemorrhage in early pregnancy	Inverse var	3	0.009339	0.270712	0.97247894	1.009383	0.593774	1.715895	0.998055
neoplasms	Malignant neoplasm of bladder	Inverse var	3	-0.00501	0.153322	0.97390978	0.994998	0.736736	1.343793	0.998055
respiratory	Respiratory failure, insufficiency, arrest	Inverse var	3	-0.00453	0.150535	0.97600871	0.995483	0.741132	1.337126	0.998055
circulatory system	Hypotension NOS	Inverse var	3	-0.0035	0.117383	0.97621887	0.996507	0.791703	1.254292	0.998055
dermatologic	Other local infections of skin and subcutaneous tissue	Inverse var	3	0.003311	0.115798	0.97719229	1.003316	0.799592	1.258946	0.998055
respiratory	Acute tonsillitis	Inverse var	3	0.007796	0.284351	0.97812847	1.007826	0.577219	1.759667	0.998055
endocrine/metabolic	Nontoxic multinodular goiter	Inverse var	3	-0.00727	0.270267	0.97853136	0.992753	0.584501	1.686155	0.998055
musculoskeletal	Rheumatoid arthritis	Inverse var	3	-0.00365	0.1379	0.97890499	0.99636	0.760385	1.305568	0.998055
musculoskeletal	Peripheral enthesopathies and allied syndromes	Inverse var	3	0.001508	0.058691	0.97950286	1.001509	0.89268	1.123606	0.998055
pregnancy complications	Problems associated with amniotic cavity and membranes	Inverse var	3	0.0098	0.396785	0.98029444	1.009849	0.463987	2.197893	0.998055
genitourinary	Postmenopausal atrophic vaginitis	Inverse var	3	-0.00518	0.211979	0.98050499	0.994834	0.656615	1.507267	0.998055
respiratory	Bronchiectasis	Inverse var	3	0.003945	0.162431	0.98062311	1.003953	0.730213	1.380311	0.998055
neoplasms	Cancer, suspected or other	Inverse var	3	0.001302	0.054218	0.98083745	1.001303	0.900355	1.113569	0.998055
neurological	Migraine	Inverse var	3	0.006641	0.291509	0.98182491	1.006663	0.568521	1.782469	0.998055
injuries & poisonings	Traumatic arthropathy	Inverse var	3	-0.00973	0.557501	0.98608179	0.990322	0.332064	2.953455	0.998055
sense organs	Blindness and low vision	Inverse var	3	-0.00548	0.325882	0.98657268	0.994531	0.525076	1.883712	0.998055
endocrine/metabolic	Type 1 diabetes	Inverse var	3	-0.00214	0.145468	0.98828283	0.997866	0.750321	1.327081	0.998055
musculoskeletal	Degeneration of intervertebral disc	Inverse var	3	-0.00294	0.227881	0.98969192	0.99706	0.637889	1.558468	0.998055
circulatory system	Aortic aneurysm	Inverse var	3	0.002112	0.189711	0.99111589	1.002115	0.690928	1.453457	0.998055
dermatologic	Psoriatic arthropathy	Inverse var	3	0.002798	0.266135	0.99161123	1.002802	0.595218	1.689485	0.998055
symptoms	Edema	Inverse var	3	-0.00175	0.171505	0.99185153	0.99825	0.713265	1.3971	0.998055
circulatory system	Atherosclerosis of the extremities	Inverse var	3	0.001936	0.247911	0.99377067	1.001937	0.616332	1.628795	0.998055
neoplasms	Cervical intraepithelial neoplasia [CIN] [Cervical dysplasia]	Inverse var	3	-0.00214	0.336731	0.99493305	0.997864	0.515751	1.930647	0.998055
musculoskeletal	Ankylosing spondylitis	Inverse var	3	0.001738	0.284351	0.99512446	1.001739	0.573733	1.749039	0.998055
injuries & poisonings	Other open wound of head and face	Inverse var	3	0.000647	0.137444	0.99624261	1.000647	0.76434	1.310014	0.998055
pregnancy complications	Umbilical cord complications during labor and delivery	Inverse var	3	-0.00258	0.570286	0.99639669	0.997428	0.32617	3.050133	0.998055
injuries & poisonings	Fracture of upper limb	Inverse var	3	-0.00041	0.125245	0.99736735	0.999587	0.782005	1.277708	0.998055
musculoskeletal	Other and unspecified disc disorder	Inverse var	3	0.000284	0.108107	0.99790416	1.000284	0.809284	1.236362	0.998055
sense organs	Strabismus and other disorders of binocular eye movements	Inverse var	3	0.000732	0.300255	0.99805502	1.000732	0.555566	1.802602	0.998055

Table S13. The phenome-wide MR analysis results of KCNJ11 (IVW or Wald ratio methods).

Category	Phenostring	Method	Nsnp	beta	se	P value	OR	LL	LR	FDR
musculoskeletal	Osteoporosis, osteopenia and pathological fracture	Inverse variance weighted	2	-0.39391	0.104727	0.000169	0.674413	0.549263	0.828079	0.132348
genitourinary	Other disorders of urethra and urinary tract	Inverse variance weighted	2	-0.41626	0.137461	0.00246	0.659509	0.503747	0.863435	0.830404
sense organs	Epiphora	Inverse variance weighted	2	0.86354	0.292763	0.003182	2.371541	1.33606	4.209548	0.830404
genitourinary	Urethral stricture (not specified as infectious)	Inverse variance weighted	2	-0.41337	0.155302	0.007774	0.661418	0.487843	0.896751	0.992635
mental disorders	Depression	Inverse variance weighted	2	-0.2126	0.083926	0.011301	0.808476	0.685848	0.953029	0.992635
sense organs	Amblyopia	Inverse variance weighted	2	-0.93804	0.381964	0.014056	0.391394	0.185131	0.827464	0.992635
genitourinary	Hydronephrosis	Inverse variance weighted	2	-0.49396	0.202875	0.0149	0.610204	0.41	0.908168	0.992635
sense organs	Disorders of lacrimal system	Inverse variance weighted	2	0.460766	0.190982	0.015839	1.585288	1.090287	2.305024	0.992635
musculoskeletal	Osteoporosis NOS	Inverse variance weighted	2	-0.38189	0.158436	0.015936	0.68257	0.500361	0.931131	0.992635
respiratory	Chronic airway obstruction	Inverse variance weighted	2	-0.21643	0.089876	0.016035	0.805388	0.675308	0.960525	0.992635
digestive	Chronic liver disease and cirrhosis	Inverse variance weighted	2	-0.38931	0.167196	0.019887	0.677524	0.488208	0.940252	0.992635
musculoskeletal	Rheumatoid arthritis	Inverse variance weighted	2	-0.31912	0.137461	0.020257	0.726787	0.555135	0.951516	0.992635
neoplasms	Melanomas of skin, dx or hx	Inverse variance weighted	2	0.401319	0.173142	0.020457	1.493794	1.06392	2.097358	0.992635
neoplasms	Melanomas of skin	Inverse variance weighted	2	0.401319	0.173142	0.020457	1.493794	1.06392	2.097358	0.992635
genitourinary	Endometrial hyperplasia	Inverse variance weighted	2	0.605543	0.262337	0.020985	1.832248	1.095666	3.064011	0.992635
symptoms	Chronic fatigue syndrome	Inverse variance weighted	2	-0.8361	0.364125	0.021665	0.433397	0.212294	0.884781	0.992635
hematopoietic	Other anemias	Inverse variance weighted	2	-0.17665	0.077976	0.023486	0.838075	0.719297	0.976465	0.992635
mental disorders	Mood disorders	Inverse variance weighted	2	-0.18255	0.083239	0.028304	0.833146	0.707729	0.980788	0.992635
symptoms	Malaise and fatigue	Inverse variance weighted	2	-0.3253	0.149355	0.029405	0.722313	0.539004	0.967963	0.992635
genitourinary	Irregular menstrual cycle/bleeding	Inverse variance weighted	2	0.16774	0.077293	0.029993	1.182629	1.016378	1.376073	0.992635
digestive	Inflammatory bowel disease and other gastroenteritis and colitis	Inverse variance weighted	2	-0.28496	0.131513	0.030249	0.752041	0.58116	0.973169	0.992635
respiratory	Pulmonary collapse; interstitial and compensatory emphysema	Inverse variance weighted	2	-0.4265	0.196929	0.030329	0.652789	0.443755	0.960289	0.992635
circulatory system	Hypertensive chronic kidney disease	Inverse variance weighted	2	0.494161	0.228534	0.030595	1.639123	1.04732	2.565333	0.992635
respiratory	Pleurisy; pleural effusion	Inverse variance weighted	2	-0.2318	0.107722	0.031411	0.793106	0.64215	0.979548	0.992635
endocrine/metabolic	Thyrotoxicosis with or without goiter	Inverse variance weighted	2	-0.44761	0.208822	0.032073	0.639155	0.424477	0.962407	0.992635
genitourinary	Irregular menstrual bleeding	Inverse variance weighted	2	0.304995	0.143408	0.033439	1.356619	1.024204	1.796921	0.992635
infectious diseases	Infection/inflammation of internal prosthetic device; implant; and graft	Inverse variance weighted	2	-0.37355	0.179089	0.036994	0.688286	0.484535	0.977716	0.992635
digestive	Paralytic ileus	Inverse variance weighted	2	-0.80801	0.387911	0.037253	0.445745	0.208396	0.953416	0.992635
genitourinary	Calculus of kidney	Inverse variance weighted	2	-0.3233	0.155302	0.037364	0.723755	0.533821	0.981267	0.992635
digestive	Ulcerative colitis	Inverse variance weighted	2	-0.32039	0.155951	0.039933	0.725863	0.534695	0.985379	0.992635
neoplasms	Secondary malignancy of respiratory organs	Inverse variance weighted	2	-0.38534	0.190982	0.043627	0.680221	0.467824	0.989049	0.992635
genitourinary	Disorders of menstruation and other abnormal bleeding from female genital tract	Inverse variance weighted	2	0.142072	0.071347	0.046451	1.152659	1.002233	1.325663	0.992635
neoplasms	Malignant neoplasm of uterus	Inverse variance weighted	2	0.497318	0.250445	0.047063	1.644305	1.006466	2.68637	0.992635
injuries & poisonings	Fracture of humerus	Inverse variance weighted	2	-0.42573	0.214768	0.047449	0.653294	0.428839	0.995228	0.992635
hematopoietic	Decreased white blood cell count	Inverse variance weighted	2	-0.30535	0.155302	0.049278	0.736864	0.54349	0.999041	0.992635
hematopoietic	Neutropenia	Inverse variance weighted	2	-0.30535	0.155302	0.049278	0.736864	0.54349	0.999041	0.992635
circulatory system	Atrioventricular block, complete	Inverse variance weighted	2	-0.70893	0.364125	0.051541	0.492169	0.241082	1.004763	0.992635
injuries & poisonings	Fracture of upper limb	Inverse variance weighted	2	-0.28092	0.144847	0.05245	0.75509	0.568463	1.002985	0.992635
musculoskeletal	Osteoporosis	Inverse variance weighted	2	-0.34046	0.175766	0.052741	0.71144	0.504108	1.004044	0.992635
neurological	Other peripheral nerve disorders	Inverse variance weighted	2	-0.16076	0.083239	0.053444	0.851497	0.723317	1.002391	0.992635
injuries & poisonings	Fracture of patella	Inverse variance weighted	2	-0.6083	0.316551	0.054649	0.544275	0.292661	1.012213	0.992635
respiratory	Asthma	Inverse variance weighted	2	-0.11156	0.058414	0.056151	0.894436	0.797676	1.002933	0.992635
mental disorders	Other mental disorder	Inverse variance weighted	2	-0.10613	0.056174	0.058843	0.899305	0.805548	1.003976	0.992635
injuries & poisonings	Fracture of neck of femur	Inverse variance weighted	2	-0.57758	0.305793	0.058919	0.561254	0.308222	1.022012	0.992635
musculoskeletal	Rheumatoid arthritis and other inflammatory polyarthropathies	Inverse variance weighted	2	-0.23568	0.125566	0.060528	0.790035	0.617679	1.010486	0.992635
circulatory system	Essential hypertension	Inverse variance weighted	2	0.074944	0.03998	0.060857	1.077824	0.996589	1.165682	0.992635
circulatory system	Hypertension	Inverse variance weighted	2	0.074944	0.03998	0.060857	1.077824	0.996589	1.165682	0.992635
sense organs	Retinal vascular changes and abnomalities	Inverse variance weighted	2	-0.56386	0.304657	0.064196	0.569006	0.313175	1.033823	0.992635
circulatory system	Atrioventricular [AV] block	Inverse variance weighted	2	-0.35089	0.190982	0.066167	0.704062	0.484221	1.023713	0.992635
dermatologic	Diffuse diseases of connective tissue	Inverse variance weighted	2	0.271795	0.149355	0.06879	1.312319	0.979277	1.758623	0.992635
symptoms	Symptoms concerning nutrition, metabolism, and development	Inverse variance weighted	2	-0.21682	0.119618	0.069898	0.805078	0.636821	1.017792	0.992635
digestive	Duodenitis	Inverse variance weighted	2	-0.18442	0.101773	0.06998	0.831589	0.681204	1.015172	0.992635
respiratory	Other diseases of respiratory system, NEC	Inverse variance weighted	2	-0.17208	0.09513	0.07046	0.841908	0.698697	1.014474	0.992635
endocrine/metabolic	Hyposmolality and/or hyponatremia	Inverse variance weighted	2	0.375311	0.208822	0.072291	1.455445	0.966592	2.191534	0.992635
respiratory	Other diseases of respiratory system, not elsewhere classified	Inverse variance weighted	2	-0.16088	0.089876	0.073447	0.851393	0.713882	1.015392	0.992635
digestive	Gingival and periodontal diseases	Inverse variance weighted	2	0.653616	0.366436	0.074471	1.922479	0.937443	3.942563	0.992635
circulatory system	Abnormal function study of cardiovascular system	Inverse variance weighted	2	-0.68925	0.387911	0.075598	0.501954	0.234675	1.073644	0.992635
digestive	Ventral hernia	Inverse variance weighted	2	-0.26493	0.149355	0.076089	0.767258	0.572543	1.028193	0.992635
digestive	Blood in stool	Inverse variance weighted	2	-0.30558	0.173142	0.077582	0.736698	0.524696	1.034359	0.992635
circulatory system	Cardiac conduction disorders	Inverse variance weighted	2	-0.18992	0.107722	0.077888	0.827024	0.669612	1.02144	0.992635
digestive	Abdominal hernia	Inverse variance weighted	2	-0.07621	0.043617	0.080602	0.926624	0.850699	1.009325	0.992635
neoplasms	Cervical cancer and dysplasia	Inverse variance weighted	2	-0.26017	0.149355	0.08152	0.770923	0.575278	1.033105	0.992635
neoplasms	Cervical cancer	Inverse variance weighted	2	-0.41776	0.241099	0.083143	0.65852	0.410527	1.056322	0.992635
digestive	Ulceration of the lower GI tract	Inverse variance weighted	2	-0.48334	0.279708	0.083984	0.616718	0.356446	1.067037	0.992635
circulatory system	Varicose veins of lower extremity	Inverse variance weighted	2	-0.14479	0.083926	0.084491	0.865205	0.733972	1.019901	0.992635
genitourinary	Dyspareunia	Inverse variance weighted	2	-0.44112	0.257079	0.086182	0.643316	0.388682	1.064767	0.992635
digestive	Ulceration of intestine	Inverse variance weighted	2	-0.5786	0.340338	0.089117	0.560682	0.28775	1.092493	0.992635
genitourinary	Functional disorders of bladder	Inverse variance weighted	2	0.365367	0.215444	0.089909	1.441043	0.944686	2.198195	0.992635
hematopoietic	Diseases of white blood cells	Inverse variance weighted	2	-0.24567	0.145962	0.092359	0.782184	0.587575	1.041248	0.992635
circulatory system	Varicose veins	Inverse variance weighted	2	-0.14107	0.083926	0.092779	0.868426	0.736705	1.023699	0.992635
musculoskeletal	Unspecified polyarthropathy or polyarthritis	Inverse variance weighted	2	0.249763	0.149355	0.094469	1.283721	0.957938	1.7203	0.992635
genitourinary	Other disorders of bladder	Inverse variance weighted	2	0.149574	0.089876	0.096065	1.16134	0.973768	1.385041	0.992635
endocrine/metabolic	Diabetes mellitus	Inverse variance weighted	2	0.347095	0.208589	0.09611	1.41495	0.940127	2.12959	0.992635
genitourinary	Other disorders of the kidney and ureters	Inverse variance weighted	2	-0.28668	0.172473	0.096481	0.750754	0.535409	1.052711	0.992635
mental disorders	Aphasia/speech disturbance	Inverse variance weighted	2	0.449051	0.271176	0.097734	1.566825	0.920855	2.665936	0.992635
musculoskeletal	Polymyalgia Rheumatica	Inverse variance weighted	2	0.433368	0.262337	0.098545	1.542444	0.922366	2.579381	0.992635
genitourinary	Chronic glomerulonephritis, NOS	Inverse variance weighted	2	0.502185	0.304657	0.099278	1.652328	0.909425	3.002102	0.992635
musculoskeletal	Osteopenia or other disorder of bone and cartilage	Inverse variance weighted	2	-0.50659	0.310604	0.102895	0.602547	0.327793	1.107599	0.992635
injuries & poisonings	Fracture of lower limb	Inverse variance weighted	2	-0.24919	0.153253	0.103947	0.779431	0.577199	1.052518	0.992635
dermatologic	Disorders of sweat glands	Inverse variance weighted	2	-0.50145	0.310604	0.106431	0.60565	0.329481	1.113303	0.992635
circulatory system	Raynaud's syndrome	Inverse variance weighted	2	-0.42113	0.262337	0.108427	0.656304	0.392463	1.097517	0.992635
circulatory system	Other chronic ischemic heart disease, unspecified	Inverse variance weighted	2	-0.12408	0.077293	0.108436	0.883313	0.75914	1.027798	0.992635
circulatory system	Peripheral vascular disease	Inverse variance weighted	2	-0.22941	0.143408	0.109659	0.795	0.6002	1.053024	0.992635
mental disorders	Tobacco use disorder	Inverse variance weighted	2	-0.10553	0.066073	0.110228	0.899848	0.790545	1.024263	0.992635
digestive	Diseases of the jaws	Inverse variance weighted	2	0.457836	0.286816	0.110428	1.58065	0.900935	2.773182	0.992635
symptoms	Nausea and vomiting	Inverse variance weighted	2	-0.13391	0.083926	0.110572	0.874665	0.741998	1.031053	0.992635
endocrine/metabolic	Hypovolemia	Inverse variance weighted	2	-0.26657	0.167196	0.110858	0.766004	0.551964	1.063043	0.992635
neoplasms	Benign neoplasm of unspecified sites	Inverse variance weighted	2	0.283146	0.179089	0.113871	1.327298	0.934383	1.885438	0.992635
sense organs	Myopia	Inverse variance weighted	2	0.514261	0.327911	0.116812	1.672402	0.879463	3.18027	0.992635
respiratory	Abnormal findings examination of lungs	Inverse variance weighted	2	-0.27832	0.179089	0.120161	0.757053	0.532945	1.0754	0.992635
respiratory	Chronic pharyngitis and nasopharyngitis	Inverse variance weighted	2	-0.4436	0.286122	0.121046	0.641721	0.366264	1.12434	0.992635
digestive	Periodontitis (acute or chronic)	Inverse variance weighted	2	0.590028	0.380727	0.121204	1.804039	0.85539	3.804763	0.992635
circulatory system	Atherosclerosis of the extremities	Inverse variance weighted	2	-0.47868	0.310604	0.123282	0.619598	0.337069	1.138941	0.992635
circulatory system	Ill-defined descriptions and complications of heart disease	Inverse variance weighted	2	-0.47327	0.310604	0.127581	0.622962	0.338899	1.145125	0.992635
neoplasms	Nevus, non-neoplastic	Inverse variance weighted	2	0.553596	0.364125	0.128424	1.739497	0.852068	3.551182	0.992635
neoplasms	Malignant neoplasm of ovary and other uterine adnexa	Inverse variance weighted	2	-0.47536	0.314686	0.130891	0.621658	0.335494	1.151909	0.992635

injuries & poisonings	Poisoning by analgesics, antipyretics, and antirheumatics	Inverse variance weighted	2	-0.16259	0.107722	0.131198	0.849935	0.688163	1.049737	0.992635
neoplasms	Malignant neoplasm of ovary	Inverse variance weighted	2	-0.46783	0.3114	0.133012	0.626363	0.340218	1.153174	0.992635
musculoskeletal	Other acquired deformities of limbs	Inverse variance weighted	2	0.323584	0.215444	0.133113	1.382072	0.906027	2.10824	0.992635
neoplasms	Cancer of other female genital organs	Inverse variance weighted	2	-0.36269	0.241946	0.133864	0.695805	0.433051	1.117985	0.992635
injuries & poisonings	Complications of cardiac/vascular device, implant, and graft	Inverse variance weighted	2	-0.31285	0.208822	0.134095	0.731363	0.485714	1.101248	0.992635
circulatory system	Cardiac pacemaker/device in situ	Inverse variance weighted	2	-0.26771	0.179089	0.134954	0.765129	0.53863	1.086872	0.992635
respiratory	Respiratory failure, insufficiency, arrest	Inverse variance weighted	2	-0.26545	0.177626	0.135058	0.766859	0.541399	1.086209	0.992635
digestive	Other chronic nonalcoholic liver disease	Inverse variance weighted	2	-0.32051	0.215444	0.136835	0.725778	0.475789	1.107116	0.992635
digestive	Constipation	Inverse variance weighted	2	-0.13263	0.089184	0.136983	0.875791	0.735335	1.043075	0.992635
neoplasms	Benign neoplasm of lip, oral cavity, and pharynx	Inverse variance weighted	2	-0.41609	0.280869	0.138485	0.659618	0.380375	1.14386	0.992635
sense organs	Retinal detachment with retinal defect	Inverse variance weighted	2	0.494415	0.337744	0.143229	1.639538	0.845722	3.178451	0.992635
injuries & poisonings	Fracture of hand or wrist	Inverse variance weighted	2	-0.21861	0.149355	0.143282	0.803637	0.59969	1.076945	0.992635
musculoskeletal	Hammer toe (acquired)	Inverse variance weighted	2	-0.29651	0.202875	0.143871	0.74341	0.499503	1.106418	0.992635
dermatologic	Erythematous conditions	Inverse variance weighted	2	-0.26169	0.179089	0.143953	0.76975	0.541883	1.093436	0.992635
mental disorders	Altered mental status	Inverse variance weighted	2	-0.27807	0.190982	0.145396	0.757246	0.520798	1.101043	0.992635
injuries & poisonings	Fracture of clavicle or scapula	Inverse variance weighted	2	-0.32989	0.22666	0.145549	0.719003	0.461098	1.121161	0.992635
digestive	Regional enteritis	Inverse variance weighted	2	-0.34973	0.240946	0.146649	0.704882	0.439561	1.130351	0.992635
circulatory system	Right bundle branch block	Inverse variance weighted	2	0.373179	0.25789	0.147883	1.452345	0.876091	2.407632	0.992635
dermatologic	Unspecified diffuse connective tissue disease	Inverse variance weighted	2	0.250479	0.173142	0.147991	1.28464	0.914955	1.803696	0.992635
injuries & poisonings	Poisoning by other anti-infectives	Inverse variance weighted	2	0.369443	0.255425	0.148069	1.446929	0.877052	2.387092	0.992635
endocrine/metabolic	Type 2 diabetes with ophthalmic manifestations	Inverse variance weighted	2	0.353014	0.245184	0.149926	1.423351	0.880253	2.301531	0.992635
genitourinary	Other specified benign mammary dysplasias	Inverse variance weighted	2	0.481285	0.334391	0.150069	1.618153	0.840195	3.116444	0.992635
digestive	Nonspecific abnormal findings in stool contents	Inverse variance weighted	2	-0.3054	0.215444	0.156325	0.736828	0.483033	1.123973	0.992635
sense organs	Primary open angle glaucoma	Inverse variance weighted	2	0.388779	0.274921	0.157319	1.475178	0.86065	2.528496	0.992635
neoplasms	Carcinoma in situ of skin	Inverse variance weighted	2	-0.47997	0.340338	0.158455	0.618799	0.317576	1.205734	0.992635
endocrine/metabolic	Type 1 diabetes	Inverse variance weighted	2	0.242352	0.173142	0.161595	1.274243	0.90755	1.789098	0.992635
genitourinary	Noninflammatory disorders of vagina	Inverse variance weighted	2	0.298872	0.214768	0.164042	1.348337	0.885084	2.054057	0.992635
digestive	Other disorders of liver	Inverse variance weighted	2	-0.16537	0.118912	0.164312	0.847578	0.671366	1.070039	0.992635
musculoskeletal	Ganglion and cyst of synovium, tendon, and bursa	Inverse variance weighted	2	0.215758	0.155302	0.164747	1.240802	0.915181	1.68228	0.992635
endocrine/metabolic	Acidosis	Inverse variance weighted	2	0.379133	0.27423	0.166807	1.461017	0.853545	2.50083	0.992635
digestive	Abnormal results of function study of liver	Inverse variance weighted	2	-0.20645	0.149355	0.166892	0.813469	0.607026	1.090121	0.992635
endocrine/metabolic	Vitamin deficiency	Inverse variance weighted	2	0.3542	0.256391	0.167131	1.42504	0.862149	2.355439	0.992635
circulatory system	Premature beats	Inverse variance weighted	2	-0.5249	0.381964	0.169379	0.591617	0.279837	1.250763	0.992635
musculoskeletal	Stiffness of joint	Inverse variance weighted	2	0.49022	0.358178	0.17111	1.632675	0.809119	3.294482	0.992635
circulatory system	Aneurysm and dissection of heart	Inverse variance weighted	2	0.456702	0.334391	0.172011	1.578859	0.819792	3.040766	0.992635
neurological	Abnormality of gait	Inverse variance weighted	2	0.300319	0.220714	0.173619	1.350289	0.876095	2.081146	0.992635
endocrine/metabolic	Hypopotassemia	Inverse variance weighted	2	-0.31584	0.233288	0.175785	0.729178	0.461588	1.151894	0.992635
respiratory	Acute pharyngitis	Inverse variance weighted	2	-0.40334	0.29871	0.176933	0.668088	0.37202	1.199777	0.992635
musculoskeletal	Osteoarthrosis	Inverse variance weighted	2	-0.07581	0.056174	0.177128	0.926988	0.830344	1.03488	0.992635
genitourinary	Frequency of urination and polyuria	Inverse variance weighted	2	-0.22811	0.169535	0.178466	0.796038	0.570983	1.109801	0.992635
endocrine/metabolic	Type 2 diabetes	Inverse variance weighted	2	0.332106	0.247294	0.179284	1.393901	0.858482	2.26325	0.992635
injuries & poisonings	Superficial injury without mention of infection	Inverse variance weighted	2	-0.18333	0.136749	0.180028	0.832489	0.63676	1.088383	0.992635
respiratory	Emphysema	Inverse variance weighted	2	-0.28763	0.214768	0.180482	0.750037	0.492344	1.142607	0.992635
respiratory	Other diseases of lung	Inverse variance weighted	2	-0.42341	0.316551	0.181032	0.654808	0.352095	1.217776	0.992635
symptoms	Other symptoms	Inverse variance weighted	2	-0.33557	0.251131	0.181475	0.714931	0.437016	1.169585	0.992635
digestive	Liver abscess and sequelae of chronic liver disease	Inverse variance weighted	2	-0.59866	0.450849	0.184227	0.549547	0.227109	1.329766	0.992635
circulatory system	Vascular insufficiency of intestine	Inverse variance weighted	2	0.490033	0.370071	0.185451	1.63237	0.790328	3.371551	0.992635
musculoskeletal	Malunion and nonunion of fracture	Inverse variance weighted	2	-0.32758	0.250445	0.190871	0.720663	0.441112	1.177376	0.992635
musculoskeletal	Osteoarthrosis NOS	Inverse variance weighted	2	-0.11666	0.089192	0.190903	0.889892	0.747163	1.059886	0.992635
digestive	Dysphagia	Inverse variance weighted	2	0.147628	0.112967	0.19127	1.159082	0.92887	1.44635	0.992635
sense organs	Inflammation of eyelids	Inverse variance weighted	2	-0.23371	0.179089	0.191892	0.79159	0.557258	1.12446	0.992635
digestive	Acute periodontitis	Inverse variance weighted	2	0.974812	0.747815	0.192388	2.650668	0.612072	11.47911	0.992635
circulatory system	Tachycardia NOS	Inverse variance weighted	2	-0.34662	0.266872	0.194005	0.707074	0.419082	1.192974	0.992635
circulatory system	Cardiac dysrhythmias	Inverse variance weighted	2	-0.07699	0.059456	0.195371	0.925902	0.824053	1.040339	0.992635
digestive	Hematemesis	Inverse variance weighted	2	-0.26266	0.202875	0.195435	0.769006	0.516701	1.144513	0.992635
genitourinary	Mucous polyp of cervix	Inverse variance weighted	2	0.25747	0.199383	0.196587	1.293653	0.875185	1.912212	0.992635
neoplasms	Lipoma of skin and subcutaneous tissue	Inverse variance weighted	2	0.169773	0.131513	0.196733	1.185035	0.915767	1.533478	0.992635
digestive	Diseases of esophagus	Inverse variance weighted	2	-0.06505	0.050753	0.199983	0.937025	0.848299	1.035031	0.992635
digestive	Diseases of the oral soft tissues, excluding lesions specific for gingiva and tongue	Inverse variance weighted	2	-0.18255	0.143408	0.203044	0.833145	0.628998	1.103549	0.992635
digestive	Gastritis and duodenitis	Inverse variance weighted	2	-0.0704	0.055511	0.204702	0.932019	0.835936	1.039145	0.992635
sense organs	Open-angle glaucoma	Inverse variance weighted	2	0.34834	0.274921	0.205137	1.416713	0.826541	2.428285	0.992635
injuries & poisonings	Anaphylactic shock NOS	Inverse variance weighted	2	0.477166	0.376697	0.205259	1.611502	0.770158	3.371956	0.992635
neoplasms	Malignant neoplasm of other and ill-defined sites within the digestive organs and pe	Inverse variance weighted	2	-0.15108	0.119618	0.206575	0.859777	0.680088	1.086943	0.992635
circulatory system	Unstable angina (intermediate coronary syndrome)	Inverse variance weighted	2	-0.15654	0.125566	0.212502	0.855093	0.668544	1.093697	0.992635
digestive	Functional digestive disorders	Inverse variance weighted	2	-0.07472	0.06012	0.213945	0.928006	0.824852	1.044062	0.992635
symptoms	Nonspecific findings on examination of blood	Inverse variance weighted	2	-0.14021	0.112967	0.214552	0.869177	0.696545	1.084595	0.992635
endocrine/metabolic	Disorders of the pituitary gland and its hypothalamic control	Inverse variance weighted	2	-0.41292	0.334391	0.216886	0.661713	0.343582	1.274411	0.992635
endocrine/metabolic	Diabetic retinopathy	Inverse variance weighted	2	0.299774	0.244499	0.220171	1.349554	0.835734	2.179274	0.992635
genitourinary	Urinary calculus	Inverse variance weighted	2	-0.13175	0.107722	0.221295	0.876557	0.709717	1.082617	0.992635
neoplasms	Multiple myeloma	Inverse variance weighted	2	-0.45649	0.376018	0.224744	0.633503	0.303163	1.323799	0.992635
genitourinary	Endometriosis	Inverse variance weighted	2	-0.23956	0.197477	0.225082	0.786971	0.534396	1.158922	0.992635
infectious diseases	Sepsis	Inverse variance weighted	2	-0.20231	0.167196	0.226269	0.816841	0.588596	1.133593	0.992635
infectious diseases	Sepsis and SIRS	Inverse variance weighted	2	-0.20231	0.167196	0.226269	0.816841	0.588596	1.133593	0.992635
injuries & poisonings	Fracture of ribs	Inverse variance weighted	2	-0.3827	0.316551	0.22667	0.682015	0.366725	1.268374	0.992635
musculoskeletal	Pathologic fracture	Inverse variance weighted	2	-0.46581	0.388592	0.230643	0.627628	0.293039	1.344247	0.992635
respiratory	Cough	Inverse variance weighted	2	-0.20029	0.167196	0.23095	0.818496	0.589789	1.135891	0.992635
mental disorders	Schizophrenia and other psychotic disorders	Inverse variance weighted	2	-0.36464	0.304657	0.231347	0.694444	0.382215	1.261731	0.992635
sense organs	Cataract	Inverse variance weighted	2	0.07817	0.065402	0.231996	1.081307	0.951213	1.229193	0.992635
neoplasms	Pancreatic cancer	Inverse variance weighted	2	0.431552	0.364125	0.235948	1.539645	0.754173	3.143184	0.992635
circulatory system	Myocardial infarction	Inverse variance weighted	2	0.099273	0.083926	0.236865	1.104367	0.936859	1.301825	0.992635
neoplasms	Lymphoid leukemia	Inverse variance weighted	2	0.436812	0.370071	0.237863	1.547764	0.749366	3.196804	0.992635
genitourinary	Excessive or frequent menstruation	Inverse variance weighted	2	0.111865	0.09513	0.239629	1.118361	0.928124	1.347591	0.992635
respiratory	Acute tonsillitis	Inverse variance weighted	2	-0.41987	0.358178	0.241102	0.657132	0.325661	1.32599	0.992635
symptoms	Symptoms of the muscles	Inverse variance weighted	2	0.391789	0.334391	0.241338	1.479626	0.768267	2.84965	0.992635
endocrine/metabolic	Secondary hypothyroidism	Inverse variance weighted	2	0.308051	0.263027	0.241527	1.36077	0.812629	2.278649	0.992635
sense organs	Macular degeneration (senile) of retina NOS	Inverse variance weighted	2	0.223256	0.190982	0.242409	1.25014	0.859788	1.817716	0.992635
sense organs	Hearing loss	Inverse variance weighted	2	0.159994	0.137461	0.244453	1.173504	0.896346	1.536362	0.992635
digestive	Hemorrhage from gastrointestinal ulcer	Inverse variance weighted	2	0.416468	0.358178	0.244935	1.516595	0.751592	3.060251	0.992635
circulatory system	Cerebral artery occlusion, with cerebral infarction	Inverse variance weighted	2	-0.28651	0.246926	0.245922	0.750879	0.462789	1.218308	0.992635
digestive	Irritable Bowel Syndrome	Inverse variance weighted	2	-0.14821	0.128036	0.247055	0.862253	0.670885	1.108209	0.992635
circulatory system	Intracerebral hemorrhage	Inverse variance weighted	2	-0.38624	0.334391	0.248073	0.67961	0.352874	1.308879	0.992635
pregnancy complicatic	Hypertension complicating pregnancy, childbirth, and the puerperium	Inverse variance weighted	2	0.310596	0.268974	0.248196	1.364237	0.805257	2.311241	0.992635
circulatory system	Chronic pulmonary heart disease	Inverse variance weighted	2	-0.42038	0.364125	0.248296	0.656796	0.321723	1.340849	0.992635
endocrine/metabolic	Anorexia	Inverse variance weighted	2	0.391079	0.339146	0.248857	1.478575	0.760601	2.874287	0.992635
symptoms	Gangrene	Inverse variance weighted	2	0.432991	0.376018	0.249519	1.541863	0.737858	3.221948	0.992635
genitourinary	Prolapse of vaginal vault after hysterectomy	Inverse variance weighted	2	0.446016	0.388592	0.251062	1.562077	0.729333	3.345637	0.992635
circulatory system	Hypertensive heart and/or renal disease	Inverse variance weighted	2	0.307641	0.268688	0.252219	1.360213	0.803332	2.303132	0.992635
hematopoietic	Pernicious anemia	Inverse variance weighted	2	0.44969	0.393183	0.252741	1.567827	0.72546	3.388303	0.992635
neurological	Migraine	Inverse variance weighted	2	0.190743	0.167196	0.253937	1.210149	0.872005	1.679417	0.992635

neurological	Degenerative disease of the spinal cord	Inverse variance weighted	2	-0.23738	0.208822	0.255633	0.78869	0.523786	1.187568	0.992635
injuries & poisonings	Crushing or internal injury to organs	Inverse variance weighted	2	-0.31823	0.280176	0.256035	0.727438	0.420054	1.259755	0.992635
sense organs	Degeneration of macula and posterior pole of retina	Inverse variance weighted	2	0.215653	0.190982	0.258823	1.240671	0.853276	1.803948	0.992635
mental disorders	Alcoholic liver damage	Inverse variance weighted	2	-0.34804	0.310604	0.262493	0.706072	0.384112	1.297898	0.992635
symptoms	Back pain	Inverse variance weighted	2	-0.09362	0.083926	0.264614	0.910625	0.772504	1.073443	0.992635
digestive	Ulcerative colitis (chronic)	Inverse variance weighted	2	-0.42405	0.381964	0.266917	0.654389	0.309529	1.383473	0.992635
pregnancy complicatic	Fetal distress and abnormal forces of labor	Inverse variance weighted	2	-0.21849	0.196929	0.267224	0.803733	0.546365	1.182336	0.992635
neoplasms	Chemotherapy	Inverse variance weighted	2	-0.06664	0.06012	0.267634	0.935527	0.831537	1.052523	0.992635
dermatologic	Chronic ulcer of skin	Inverse variance weighted	2	-0.21769	0.196929	0.268976	0.804375	0.546801	1.18328	0.992635
sense organs	Glaucoma	Inverse variance weighted	2	0.151047	0.136749	0.269352	1.163051	0.889602	1.520554	0.992635
digestive	Inguinal hernia	Inverse variance weighted	2	-0.08518	0.077293	0.270449	0.918348	0.789249	1.068563	0.992635
neoplasms	Breast cancer	Inverse variance weighted	2	-0.09148	0.083239	0.27174	0.912575	0.775202	1.074293	0.992635
neoplasms	Breast cancer [female]	Inverse variance weighted	2	-0.09127	0.083239	0.27285	0.912768	0.775366	1.07452	0.992635
musculoskeletal	Kyphoscoliosis and scoliosis	Inverse variance weighted	2	0.352801	0.322158	0.273466	1.423048	0.75682	2.675755	0.992635
neurological	Abnormal involuntary movements	Inverse variance weighted	2	-0.32443	0.298014	0.276316	0.722941	0.403115	1.296514	0.992635
digestive	Diaphragmatic hernia	Inverse variance weighted	2	-0.06293	0.057889	0.277033	0.939013	0.838292	1.051836	0.992635
respiratory	Obstructive chronic bronchitis	Inverse variance weighted	2	-0.3897	0.358639	0.277211	0.67726	0.335333	1.367841	0.992635
neoplasms	Benign neoplasm of other endocrine glands and related structures	Inverse variance weighted	2	-0.32351	0.29871	0.278793	0.723603	0.402933	1.299473	0.992635
mental disorders	Agorophobia, social phobia, and panic disorder	Inverse variance weighted	2	-0.47495	0.438684	0.278956	0.621916	0.263218	1.469426	0.992635
symptoms	Other abnormal blood chemistry	Inverse variance weighted	2	-0.12287	0.11367	0.279707	0.884374	0.707747	1.105081	0.992635
musculoskeletal	Acquired toe deformities	Inverse variance weighted	2	-0.1353	0.125566	0.281252	0.873455	0.6829	1.117183	0.992635
circulatory system	Atherosclerosis	Inverse variance weighted	2	-0.26256	0.244499	0.282876	0.769078	0.476265	1.241916	0.992635
dermatologic	Prurigo and Lichen	Inverse variance weighted	2	-0.33934	0.316551	0.283731	0.712244	0.382979	1.324592	0.992635
endocrine/metabolic	Type 2 diabetes with neurological manifestations	Inverse variance weighted	2	0.394427	0.370071	0.286508	1.483534	0.718268	3.06414	0.992635
symptoms	Myalgia and myositis unspecified	Inverse variance weighted	2	-0.36628	0.346958	0.291109	0.693308	0.351228	1.368558	0.992635
sense organs	Disorders of conjunctiva	Inverse variance weighted	2	0.33995	0.322498	0.29183	1.404878	0.74666	2.643347	0.992635
symptoms	Other ill-defined and unknown causes of morbidity and mortality	Inverse variance weighted	2	0.07533	0.072025	0.295614	1.07824	0.936282	1.241721	0.992635
hematopoietic	Lymphadenitis	Inverse variance weighted	2	0.181068	0.173142	0.295666	1.198496	0.853601	1.682746	0.992635
neoplasms	Cancer of stomach	Inverse variance weighted	2	-0.39282	0.376018	0.296167	0.675149	0.323093	1.410824	0.992635
neoplasms	Lymphoid leukemia, chronic	Inverse variance weighted	2	0.436279	0.420526	0.299522	1.54694	0.678444	3.527221	0.992635
respiratory	Pneumococcal pneumonia	Inverse variance weighted	2	-0.11782	0.11367	0.29995	0.888853	0.711331	1.110677	0.992635
musculoskeletal	Osteoarthrosis, generalized	Inverse variance weighted	2	-0.37328	0.364125	0.30529	0.688469	0.337237	1.405509	0.992635
injuries & poisonings	Injury, NOS	Inverse variance weighted	2	0.09801	0.095825	0.306398	1.102974	0.914108	1.330862	0.992635
circulatory system	Cardiac pacemaker in situ	Inverse variance weighted	2	-0.21	0.206863	0.310031	0.810586	0.540398	1.215861	0.992635
neoplasms	Vascular hamartomas and non-neoplastic nevi	Inverse variance weighted	2	0.350064	0.346285	0.312058	1.419158	0.719892	2.797657	0.992635
digestive	Calculus of bile duct	Inverse variance weighted	2	-0.17483	0.173142	0.31263	0.839604	0.597988	1.178844	0.992635
symptoms	Musculoskeletal symptoms referable to limbs	Inverse variance weighted	2	0.120617	0.119618	0.313286	1.128193	0.892406	1.426278	0.992635
circulatory system	Paroxysmal ventricular tachycardia	Inverse variance weighted	2	0.294364	0.292068	0.31352	1.342273	0.75723	2.379325	0.992635
circulatory system	Paroxysmal tachycardia, unspecified	Inverse variance weighted	2	0.156241	0.155302	0.314393	1.169108	0.862301	1.585077	0.992635
digestive	Ulcer of esophagus	Inverse variance weighted	2	-0.12607	0.125566	0.315357	0.88155	0.689228	1.127536	0.992635
musculoskeletal	Other disorders of synovium, tendon, and bursa	Inverse variance weighted	2	0.10193	0.101773	0.316565	1.107306	0.907061	1.351758	0.992635
circulatory system	Transient cerebral ischemia	Inverse variance weighted	2	0.190577	0.190982	0.318339	1.209947	0.832145	1.759275	0.992635
symptoms	Edema	Inverse variance weighted	2	-0.21982	0.220714	0.319268	0.802661	0.520782	1.237108	0.992635
sense organs	Tinnitus	Inverse variance weighted	2	0.391055	0.392849	0.319526	1.478539	0.684594	3.193247	0.992635
musculoskeletal	Other disorders of bone and cartilage	Inverse variance weighted	2	-0.14237	0.143408	0.32083	0.867301	0.654785	1.148792	0.992635
genitourinary	Breast conditions, congenital or relating to hormones	Inverse variance weighted	2	-0.28444	0.286816	0.321341	0.752438	0.428872	1.320119	0.992635
digestive	Other symptoms involving abdomen and pelvis	Inverse variance weighted	2	-0.19035	0.192297	0.322246	0.826673	0.567084	1.205093	0.992635
respiratory	Other symptoms of respiratory system	Inverse variance weighted	2	-0.09387	0.09513	0.32377	0.910403	0.75554	1.097007	0.992635
injuries & poisonings	Fracture of unspecified bones	Inverse variance weighted	2	0.327942	0.333013	0.324737	1.388108	0.722698	2.666183	0.992635
digestive	Ascites (non malignant)	Inverse variance weighted	2	-0.22309	0.22666	0.324994	0.800043	0.513069	1.247529	0.992635
hematopoietic	Megaloblastic anemia	Inverse variance weighted	2	0.313227	0.318308	0.325098	1.367832	0.732965	2.552597	0.992635
endocrine/metabolic	Hypothyroidism	Inverse variance weighted	2	-0.07604	0.077293	0.325195	0.926776	0.796492	1.07837	0.992635
injuries & poisonings	Dislocation	Inverse variance weighted	2	0.18822	0.191649	0.326047	1.207099	0.829102	1.757431	0.992635
endocrine/metabolic	Hypoglycemia	Inverse variance weighted	2	0.412784	0.428308	0.33517	1.511018	0.652659	3.498269	0.992635
digestive	Anal and rectal conditions	Inverse variance weighted	2	-0.09305	0.097292	0.338874	0.911148	0.75296	1.10257	0.992635
digestive	Obstruction of bile duct	Inverse variance weighted	2	-0.30192	0.316551	0.34019	0.739395	0.397579	1.375087	0.992635
neoplasms	Cancer of brain and nervous system	Inverse variance weighted	2	0.364725	0.382645	0.340505	1.440117	0.680274	3.048679	0.992635
circulatory system	Primary/intrinsic cardiomyopathies	Inverse variance weighted	2	0.438968	0.461726	0.341752	1.551105	0.627498	3.834161	0.992635
pregnancy complicatic	Other complications of pregnancy NEC	Inverse variance weighted	2	0.312458	0.329136	0.342454	1.366781	0.717022	2.605344	0.992635
genitourinary	Irregular menstrual cycle	Inverse variance weighted	2	0.18701	0.197598	0.343937	1.205639	0.818499	1.775892	0.992635
mental disorders	Bipolar	Inverse variance weighted	2	-0.25406	0.268974	0.344894	0.775648	0.457836	1.314074	0.992635
endocrine/metabolic	Hypothyroidism NOS	Inverse variance weighted	2	-0.07362	0.077976	0.345117	0.929027	0.79736	1.082437	0.992635
endocrine/metabolic	Other disorders of pancreatic internal secretion	Inverse variance weighted	2	0.384804	0.409343	0.34719	1.469327	0.658686	3.277616	0.992635
dermatologic	Rash and other nonspecific skin eruption	Inverse variance weighted	2	0.179517	0.190982	0.347235	1.196639	0.822992	1.739925	0.992635
digestive	Anal and rectal polyp	Inverse variance weighted	2	-0.09537	0.101773	0.348711	0.909035	0.744645	1.109717	0.992635
infectious diseases	Gram negative septicemia	Inverse variance weighted	2	0.418123	0.446774	0.349339	1.519108	0.63283	3.646618	0.992635
infectious diseases	Viral infection	Inverse variance weighted	2	-0.15586	0.167196	0.351239	0.855681	0.616583	1.187494	0.992635
respiratory	Hemoptysis	Inverse variance weighted	2	-0.34154	0.367	0.352044	0.710674	0.346157	1.45904	0.992635
endocrine/metabolic	Acid-base balance disorder	Inverse variance weighted	2	0.238615	0.257079	0.353315	1.26949	0.767007	2.101161	0.992635
sense organs	Labyrinthitis	Inverse variance weighted	2	0.294217	0.317217	0.353671	1.342074	0.720702	2.49918	0.992635
digestive	Chronic pancreatitis	Inverse variance weighted	2	0.360043	0.388592	0.35417	1.433391	0.66925	3.07002	0.992635
hematopoietic	Iron deficiency anemias	Inverse variance weighted	2	-0.09398	0.101773	0.355772	0.910298	0.74568	1.111259	0.992635
respiratory	Acute and chronic tonsillitis	Inverse variance weighted	2	-0.25478	0.276348	0.356546	0.775084	0.450937	1.332236	0.992635
digestive	Esophagitis, GERD and related diseases	Inverse variance weighted	2	-0.04828	0.053132	0.363493	0.952864	0.858626	1.057446	0.992635
genitourinary	Other nonmalignant breast conditions	Inverse variance weighted	2	0.18418	0.202875	0.363958	1.202232	0.807788	1.789284	0.992635
respiratory	Nasal polyps	Inverse variance weighted	2	0.140669	0.155302	0.365052	1.151044	0.848978	1.560586	0.992635
infectious diseases	Intestinal infection	Inverse variance weighted	2	-0.08998	0.099345	0.365059	0.913946	0.75224	1.110414	0.992635
circulatory system	Cardiomyopathy	Inverse variance weighted	2	0.367493	0.4068	0.366326	1.44411	0.650617	3.205346	0.992635
neurological	Other cerebral degenerations	Inverse variance weighted	2	-0.37623	0.416908	0.366829	0.686445	0.303198	1.554121	0.992635
neoplasms	Benign neoplasm of skin	Inverse variance weighted	2	0.091792	0.101773	0.367096	1.096137	0.897912	1.338123	0.992635
mental disorders	Schizophrenia	Inverse variance weighted	2	-0.33362	0.370071	0.367328	0.716329	0.346818	1.47953	0.992635
neoplasms	Secondary malignancy of lymph nodes	Inverse variance weighted	2	-0.1076	0.119618	0.368391	0.897991	0.710315	1.135253	0.992635
sense organs	Disorders of external ear	Inverse variance weighted	2	0.209173	0.232607	0.368517	1.232658	0.781347	1.944649	0.992635
respiratory	Respiratory insufficiency	Inverse variance weighted	2	-0.23158	0.257909	0.369225	0.793277	0.478507	1.315108	0.992635
sense organs	Subjective visual disturbances	Inverse variance weighted	2	-0.32119	0.358178	0.369863	0.725286	0.359436	1.463513	0.992635
musculoskeletal	Spondylosis and allied disorders	Inverse variance weighted	2	-0.12714	0.141841	0.370075	0.880613	0.66688	1.162848	0.992635
hematopoietic	Anemia of chronic disease	Inverse variance weighted	2	-0.29941	0.334391	0.370583	0.741257	0.384883	1.427606	0.992635
neoplasms	Lipoma	Inverse variance weighted	2	0.10106	0.112967	0.371	1.106343	0.886606	1.38054	0.992635
musculoskeletal	Other and unspecified disc disorder	Inverse variance weighted	2	0.139537	0.15609	0.371347	1.149742	0.846708	1.56123	0.992635
digestive	Peritoneal or intestinal adhesions	Inverse variance weighted	2	-0.42248	0.479204	0.377978	0.65542	0.25622	1.676588	0.992635
symptoms	Symptoms involving skin and other integumentary tissue	Inverse variance weighted	2	-0.18237	0.208822	0.382475	0.83329	0.553406	1.254726	0.992635
dermatologic	Atopic/contact dermatitis due to other or unspecified	Inverse variance weighted	2	-0.18927	0.2175	0.38418	0.827561	0.540332	1.267475	0.992635
digestive	Ileostomy status	Inverse variance weighted	2	-0.18648	0.215444	0.386728	0.829874	0.54403	1.265907	0.992635
congenital anomalies	Congenital anomalies of genital organs	Inverse variance weighted	2	0.28397	0.328445	0.387264	1.328393	0.697829	2.528741	0.992635
infectious diseases	Streptococcus infection	Inverse variance weighted	2	0.1862	0.215444	0.387443	1.204663	0.789726	1.837618	0.992635
genitourinary	Polyp of female genital organs	Inverse variance weighted	2	0.076737	0.089184	0.389553	1.079758	0.906591	1.286001	0.992635
musculoskeletal	Osteomyelitis	Inverse variance weighted	2	0.317755	0.37075	0.391412	1.374039	0.664371	2.841761	0.992635
injuries & poisonings	Fracture of radius and ulna	Inverse variance weighted	2	-0.23302	0.273617	0.394423	0.792139	0.463334	1.35428	0.992635
sense organs	Other retinal disorders	Inverse variance weighted	2	0.122771	0.144463	0.39541	1.130626	0.851824	1.50068	0.992635

hematopoietic	Diseases of spleen	Inverse variance weighted	2	0.3298	0.388592	0.396045	1.39069	0.649313	2.978564	0.992635
respiratory	Septal Deviations/Turbinate Hypertrophy	Inverse variance weighted	2	0.111258	0.131516	0.397571	1.117683	0.863714	1.44633	0.992635
hematopoietic	Thrombocytopenia	Inverse variance weighted	2	-0.18729	0.221392	0.397583	0.829207	0.537292	1.279722	0.992635
genitourinary	Inflammatory disease of breast	Inverse variance weighted	2	0.274458	0.328445	0.403363	1.315817	0.691223	2.504801	0.992635
hematopoietic	Other diseases of blood and blood-forming organs	Inverse variance weighted	2	0.114758	0.137461	0.403805	1.121602	0.856702	1.468411	0.992635
pregnancy complicatic	Early onset of delivery	Inverse variance weighted	2	0.30322	0.364125	0.404993	1.354213	0.663342	2.764625	0.992635
symptoms	Other tests	Inverse variance weighted	2	-0.09373	0.11367	0.409604	0.910527	0.728677	1.137761	0.992635
genitourinary	Hypertrophy of breast (Gynecomastia)	Inverse variance weighted	2	-0.24972	0.304657	0.412401	0.779019	0.428764	1.415394	0.992635
sense organs	Strabismus and other disorders of binocular eye movements	Inverse variance weighted	2	0.190332	0.232607	0.413211	1.209651	0.766763	1.908354	0.992635
symptoms	Pain in limb	Inverse variance weighted	2	-0.09249	0.11367	0.415845	0.91166	0.729584	1.139176	0.992635
genitourinary	Noninflammatory disorders of cervix	Inverse variance weighted	2	0.135782	0.167196	0.416727	1.145432	0.825371	1.589605	0.992635
pregnancy complicatic	Hemorrhage during pregnancy; childbirth and postpartum	Inverse variance weighted	2	0.169169	0.208822	0.417875	1.18432	0.786533	1.783289	0.992635
circulatory system	Polyarteritis nodosa and allied conditions	Inverse variance weighted	2	-0.33863	0.420797	0.420975	0.712747	0.312425	1.626017	0.992635
digestive	Portal hypertension	Inverse variance weighted	2	-0.30786	0.382645	0.421076	0.735019	0.347204	1.556009	0.992635
respiratory	Respiratory failure	Inverse variance weighted	2	-0.21766	0.271832	0.423301	0.8044	0.472154	1.370442	0.992635
circulatory system	Cerebral ischemia	Inverse variance weighted	2	0.133555	0.167196	0.424411	1.142884	0.823535	1.586068	0.992635
symptoms	Swelling of limb	Inverse variance weighted	2	0.105021	0.131513	0.424548	1.110734	0.858348	1.437329	0.992635
injuries & poisonings	Effects radiation NOS	Inverse variance weighted	2	-0.1287	0.161249	0.424789	0.879238	0.640986	1.206048	0.992635
dermatologic	Other dyschromia	Inverse variance weighted	2	0.251517	0.316551	0.426872	1.285975	0.691479	2.391587	0.992635
dermatologic	Sicca syndrome	Inverse variance weighted	2	0.352217	0.443467	0.427059	1.422217	0.596319	3.391977	0.992635
circulatory system	Cardiac arrest	Inverse variance weighted	2	0.420044	0.529107	0.427268	1.522029	0.539558	4.293463	0.992635
neurological	Sleep apnea	Inverse variance weighted	2	-0.10423	0.131513	0.428058	0.901021	0.696287	1.165953	0.992635
symptoms	Sciatica	Inverse variance weighted	2	-0.14148	0.179751	0.431241	0.868075	0.61031	1.234709	0.992635
neoplasms	Malignant neoplasm of female breast	Inverse variance weighted	2	-0.06601	0.083926	0.43157	0.936123	0.794134	1.103499	0.992635
mental disorders	Alcoholism	Inverse variance weighted	2	-0.08073	0.102805	0.432303	0.922445	0.754104	1.128364	0.992635
genitourinary	Urinary tract infection	Inverse variance weighted	2	-0.06117	0.078923	0.438316	0.940665	0.80585	1.098033	0.992635
symptoms	Cervicalgia	Inverse variance weighted	2	-0.24087	0.311805	0.439823	0.785946	0.426559	1.448126	0.992635
mental disorders	Other specified nonpsychotic and/or transient mental disorders	Inverse variance weighted	2	0.271535	0.352232	0.440766	1.311977	0.65781	2.616687	0.992635
circulatory system	Other acute and subacute forms of ischemic heart disease	Inverse variance weighted	2	0.198086	0.257079	0.440989	1.219067	0.736542	2.017705	0.992635
infectious diseases	Candidiasis	Inverse variance weighted	2	-0.2322	0.301626	0.441403	0.792788	0.438942	1.43188	0.992635
dermatologic	Cellulitis and abscess of trunk	Inverse variance weighted	2	0.274718	0.358178	0.44309	1.316159	0.65226	2.655803	0.992635
circulatory system	Precordial pain	Inverse variance weighted	2	-0.1144	0.149355	0.443714	0.891904	0.665556	1.195231	0.992635
neoplasms	Secondary malignant neoplasm	Inverse variance weighted	2	-0.06851	0.089876	0.445892	0.933784	0.782966	1.113653	0.992635
injuries & poisonings	Poisoning/allergy of sulfonamides	Inverse variance weighted	2	0.355077	0.472524	0.452383	1.426291	0.564921	3.601045	0.992635
endocrine/metabolic	Obesity	Inverse variance weighted	2	-0.06684	0.089184	0.453565	0.935343	0.785336	1.114002	0.992635
dermatologic	Psoriatic arthropathy	Inverse variance weighted	2	-0.27318	0.364527	0.453611	0.760956	0.37245	1.554717	0.992635
endocrine/metabolic	Protein-calorie malnutrition	Inverse variance weighted	2	0.200597	0.268974	0.455797	1.222132	0.721378	2.070491	0.992635
respiratory	Chronic bronchitis	Inverse variance weighted	2	-0.28963	0.389001	0.456542	0.748539	0.349213	1.604498	0.992635
sense organs	Other disorders of middle ear and mastoid	Inverse variance weighted	2	-0.2641	0.356271	0.458521	0.767898	0.381979	1.543716	0.992635
pregnancy complicatic	Placenta previa and abruptio placenta	Inverse variance weighted	2	0.190001	0.256391	0.458657	1.209251	0.731597	1.998763	0.992635
sense organs	Corneal opacity and other disorders of cornea	Inverse variance weighted	2	-0.24331	0.328445	0.458825	0.784031	0.411866	1.492489	0.992635
circulatory system	Intracranial hemorrhage	Inverse variance weighted	2	-0.18222	0.246804	0.460324	0.833419	0.513783	1.351908	0.992635
sense organs	Dizziness and giddiness (Light-headedness and vertigo)	Inverse variance weighted	2	-0.09669	0.131513	0.462203	0.907836	0.701554	1.174772	0.992635
neoplasms	Benign neoplasm of ovary	Inverse variance weighted	2	-0.18876	0.258099	0.464561	0.827983	0.499256	1.373156	0.992635
digestive	Disorders of esophageal motility	Inverse variance weighted	2	0.424284	0.582174	0.466129	1.528495	0.488323	4.784328	0.992635
sense organs	Ectropion or entropion	Inverse variance weighted	2	0.356111	0.489163	0.466613	1.427766	0.54736	3.724268	0.992635
neoplasms	Cancer within the respiratory system	Inverse variance weighted	2	0.125593	0.173142	0.468223	1.13382	0.807537	1.591938	0.992635
circulatory system	Stricture of artery	Inverse variance weighted	2	0.453037	0.629406	0.471657	1.573083	0.458132	5.401477	0.992635
musculoskeletal	Enthesopathy	Inverse variance weighted	2	-0.08079	0.112329	0.472015	0.92239	0.740113	1.149558	0.992635
genitourinary	Vaginal enterocele, congenital or acquired	Inverse variance weighted	2	0.327599	0.458515	0.474931	1.387633	0.564909	3.408556	0.992635
genitourinary	Hematuria	Inverse variance weighted	2	0.050896	0.071347	0.475627	1.052213	0.914896	1.210141	0.992635
digestive	Intestinal obstruction without mention of hernia	Inverse variance weighted	2	-0.10163	0.142695	0.476322	0.903362	0.682964	1.194885	0.992635
genitourinary	Chronic cystitis	Inverse variance weighted	2	-0.45897	0.645304	0.476928	0.631932	0.178392	2.238535	0.992635
infectious diseases	Bacterial enteritis	Inverse variance weighted	2	-0.11878	0.167196	0.477422	0.887999	0.639871	1.232345	0.992635
injuries & poisonings	Traumatic arthropathy	Inverse variance weighted	2	-0.27125	0.382645	0.478397	0.762426	0.36015	1.614029	0.992635
injuries & poisonings	Fracture of vertebral column without mention of spinal cord injury	Inverse variance weighted	2	0.168612	0.239236	0.480939	1.183661	0.740603	1.891773	0.992635
infectious diseases	Postoperative infection	Inverse variance weighted	2	0.092586	0.131513	0.481431	1.097007	0.847741	1.419567	0.992635
endocrine/metabolic	Overweight, obesity and other hyperalimentation	Inverse variance weighted	2	-0.06272	0.089184	0.481897	0.939207	0.788581	1.118604	0.992635
circulatory system	Cerebrovascular disease	Inverse variance weighted	2	-0.06734	0.095825	0.4822	0.934875	0.774793	1.128031	0.992635
digestive	Other disorders of gallbladder	Inverse variance weighted	2	0.167398	0.238553	0.482852	1.182225	0.740696	1.88695	0.992635
musculoskeletal	Osteomyelitis, periostitis, and other infections involving bone	Inverse variance weighted	2	0.28306	0.405946	0.485625	1.327185	0.598939	2.940899	0.992635
digestive	Other disorders of intestine	Inverse variance weighted	2	-0.0956	0.137461	0.486742	0.908824	0.694178	1.18984	0.992635
injuries & poisonings	Torus fracture	Inverse variance weighted	2	-0.149	0.215444	0.489201	0.861572	0.56481	1.31426	0.992635
circulatory system	Aortic aneurysm	Inverse variance weighted	2	-0.29364	0.425588	0.490223	0.745548	0.323749	1.716893	0.992635
circulatory system	Circulatory disease NEC	Inverse variance weighted	2	-0.08004	0.116211	0.49098	0.923079	0.735052	1.159204	0.992635
digestive	Appendicitis	Inverse variance weighted	2	-0.10682	0.155302	0.491566	0.898688	0.662847	1.218441	0.992635
musculoskeletal	Rupture of synovium	Inverse variance weighted	2	-0.24904	0.364125	0.49402	0.779552	0.381853	1.591456	0.992635
neurological	Abnormal movement	Inverse variance weighted	2	0.114262	0.167196	0.494351	1.121046	0.8078	1.555763	0.992635
genitourinary	Urinary incontinence	Inverse variance weighted	2	-0.09172	0.134649	0.495762	0.912361	0.700731	1.187907	0.992635
hematopoietic	Purpura and other hemorrhagic conditions	Inverse variance weighted	2	-0.14166	0.208822	0.497522	0.867913	0.5764	1.306859	0.992635
neoplasms	Manlignant and unknown neoplasms of brain and nervous system	Inverse variance weighted	2	0.233338	0.346285	0.500417	1.262809	0.640581	2.489437	0.992635
genitourinary	Calculus of lower urinary tract	Inverse variance weighted	2	-0.21216	0.316551	0.502707	0.808832	0.434916	1.504222	0.992635
hematopoietic	Iron deficiency anemias, unspecified or not due to blood loss	Inverse variance weighted	2	-0.06809	0.101773	0.503464	0.934175	0.765239	1.140406	0.992635
genitourinary	Renal failure NOS	Inverse variance weighted	2	-0.18161	0.271509	0.503568	0.833928	0.489796	1.419847	0.992635
digestive	Heartburn	Inverse variance weighted	2	-0.12817	0.191649	0.503649	0.879706	0.60423	1.280775	0.992635
digestive	Diseases and other conditions of the tongue	Inverse variance weighted	2	-0.3417	0.512807	0.505201	0.710563	0.260071	1.941388	0.992635
dermatologic	Hyperhidrosis	Inverse variance weighted	2	-0.2424	0.364125	0.505597	0.784741	0.384395	1.602049	0.992635
neurological	Epilepsy	Inverse variance weighted	2	-0.19488	0.292763	0.505626	0.822932	0.463617	1.460726	0.992635
sense organs	Visual disturbances	Inverse variance weighted	2	-0.12272	0.185885	0.509144	0.884515	0.614436	1.273309	0.992635
musculoskeletal	Ankylosing spondylitis	Inverse variance weighted	2	-0.2321	0.352906	0.510751	0.792871	0.397011	1.583442	0.992635
digestive	Esophageal bleeding (varices/hemorrhage)	Inverse variance weighted	2	0.140616	0.214768	0.512638	1.150983	0.755535	1.753408	0.992635
musculoskeletal	Unspecified monoarthritis	Inverse variance weighted	2	-0.07438	0.114221	0.514935	0.928321	0.742115	1.161249	0.992635
dermatologic	Ingrowing nail	Inverse variance weighted	2	-0.1862	0.286122	0.515198	0.830109	0.473787	1.45441	0.992635
digestive	Duodenal ulcer	Inverse variance weighted	2	-0.1097	0.169094	0.516496	0.896103	0.643313	1.248226	0.992635
sense organs	Disorders of vitreous body	Inverse variance weighted	2	0.154965	0.239236	0.517147	1.167617	0.730565	1.866132	0.992635
musculoskeletal	Synovitis and tenosynovitis	Inverse variance weighted	2	0.107918	0.167196	0.518631	1.113956	0.802691	1.545923	0.992635
circulatory system	Atrial fibrillation and flutter	Inverse variance weighted	2	-0.08177	0.127917	0.522644	0.92148	0.717135	1.184053	0.992635
endocrine/metabolic	Crystal arthropathies	Inverse variance weighted	2	0.228679	0.358178	0.523181	1.256938	0.622912	2.536305	0.992635
neoplasms	Benign neoplasm of brain, cranial nerves, meninges	Inverse variance weighted	2	0.224836	0.352789	0.523923	1.252118	0.627112	2.500032	0.992635
neoplasms	Secondary malignant neoplasm of liver	Inverse variance weighted	2	0.109908	0.173142	0.52557	1.116175	0.79497	1.567163	0.992635
genitourinary	Ovarian cyst	Inverse variance weighted	2	-0.08233	0.131513	0.531314	0.92097	0.711704	1.191769	0.992635
circulatory system	Other specified cardiac dysrhythmias	Inverse variance weighted	2	-0.18836	0.301841	0.532599	0.828315	0.45842	1.496676	0.992635
endocrine/metabolic	Simple and unspecified goiter	Inverse variance weighted	2	0.223423	0.358178	0.532774	1.250349	0.619646	2.523009	0.992635
neurological	Sleep disorders	Inverse variance weighted	2	-0.08979	0.143952	0.532808	0.914126	0.6894	1.212107	0.992635
dermatologic	Actinic keratosis	Inverse variance weighted	2	0.107794	0.173142	0.533563	1.113819	0.793291	1.563855	0.992635
respiratory	Bacterial pneumonia	Inverse variance weighted	2	-0.07248	0.116544	0.533979	0.930081	0.740144	1.168759	0.992635
sense organs	Disorders of refraction and accommodation; blindness and low vision	Inverse variance weighted	2	0.110804	0.179089	0.536107	1.117176	0.786462	1.586958	0.992635
circulatory system	Occlusion of cerebral arteries	Inverse variance weighted	2	-0.08446	0.137461	0.538948	0.919012	0.70196	1.203178	0.992635
musculoskeletal	Curvature of spine	Inverse variance weighted	2	0.161057	0.263027	0.540325	1.174752	0.701542	1.967156	0.992635

digestive	Malposition and malpresentation of fetus or obstruction	Inverse variance weighted	2	0.134366	0.220714	0.542671	1.143811	0.742128	1.76291	0.992635
sense organs	Meniere's disease	Inverse variance weighted	2	-0.22523	0.370071	0.542781	0.798333	0.386521	1.648902	0.992635
neurological	Convulsions	Inverse variance weighted	2	-0.11605	0.190982	0.543423	0.890431	0.612397	1.294696	0.992635
musculoskeletal	Other inflammatory spondylopathies	Inverse variance weighted	2	-0.13497	0.222542	0.544201	0.873746	0.564877	1.351502	0.992635
neoplasms	Large cell lymphoma	Inverse variance weighted	2	-0.22364	0.370071	0.545636	0.799604	0.387136	1.651528	0.992635
respiratory	Other upper respiratory disease	Inverse variance weighted	2	0.083056	0.137461	0.5457	1.086603	0.829969	1.422589	0.992635
digestive	Disorders of tooth development	Inverse variance weighted	2	-0.10794	0.179089	0.546706	0.897684	0.631945	1.275167	0.992635
circulatory system	Peripheral vascular disease, unspecified	Inverse variance weighted	2	-0.10762	0.179089	0.547889	0.897969	0.632147	1.275573	0.992635
circulatory system	Cardiomegaly	Inverse variance weighted	2	-0.10318	0.173142	0.551209	0.901961	0.642401	1.266396	0.992635
sense organs	Inflammation of the eye	Inverse variance weighted	2	-0.09204	0.155302	0.553423	0.91207	0.672717	1.236585	0.992635
neoplasms	Secondary malignant neoplasm of digestive systems	Inverse variance weighted	2	-0.13428	0.22666	0.553578	0.874349	0.560722	1.363397	0.992635
mental disorders	Phobia	Inverse variance weighted	2	0.232868	0.393857	0.554353	1.262215	0.583277	2.731439	0.992635
digestive	Peritonitis and retroperitoneal infections	Inverse variance weighted	2	0.1757	0.298014	0.555478	1.192081	0.664709	2.137864	0.992635
digestive	Diseases of pulp and periapical tissues	Inverse variance weighted	2	-0.12278	0.208822	0.556554	0.884458	0.587387	1.331771	0.992635
circulatory system	Other disorders of arteries and arterioles	Inverse variance weighted	2	0.32926	0.560785	0.557109	1.389939	0.463069	4.172013	0.992635
musculoskeletal	Osteoarthrosis, localized, primary	Inverse variance weighted	2	0.089945	0.153391	0.557622	1.094114	0.810016	1.477853	0.992635
musculoskeletal	Other disorders of cervical region	Inverse variance weighted	2	-0.50715	0.866198	0.558218	0.602209	0.110262	3.289046	0.992635
dermatologic	Decubitus ulcer	Inverse variance weighted	2	-0.25431	0.435301	0.559068	0.775448	0.330383	1.820071	0.992635
musculoskeletal	Other acquired musculoskeletal deformity	Inverse variance weighted	2	-0.2532	0.433533	0.559188	0.776309	0.331898	1.81579	0.992635
mental disorders	Delirium due to conditions classified elsewhere	Inverse variance weighted	2	0.202239	0.346285	0.559203	1.224141	0.620966	2.413209	0.992635
neoplasms	Cervical intraepithelial neoplasia [CIN] [Cervical dysplasia]	Inverse variance weighted	2	-0.11492	0.196929	0.559502	0.891434	0.605983	1.311349	0.992635
pregnancy complicatic	Problems associated with amniotic cavity and membranes	Inverse variance weighted	2	0.14216	0.244499	0.560948	1.152761	0.713867	1.861492	0.992635
genitourinary	Bladder neck obstruction	Inverse variance weighted	2	0.150659	0.259471	0.561482	1.162601	0.699141	1.933288	0.992635
dermatologic	Symptoms affecting skin	Inverse variance weighted	2	0.06935	0.119618	0.562073	1.071812	0.847808	1.355	0.992635
digestive	Chronic periodontitis	Inverse variance weighted	2	0.213615	0.37075	0.564501	1.238145	0.598664	2.560708	0.992635
respiratory	Abnormal sputum	Inverse variance weighted	2	-0.22094	0.383836	0.56488	0.801765	0.37785	1.701278	0.992635
sense organs	Diplopia and disorders of binocular vision	Inverse variance weighted	2	0.188809	0.328445	0.565386	1.207811	0.634485	2.299199	0.992635
circulatory system	Other aneurysm	Inverse variance weighted	2	-0.2438	0.42422	0.5655	0.783647	0.341206	1.7998	0.992635
injuries & poisonings	Poisoning by anticonvulsants and anti-Parkinsonism drugs	Inverse variance weighted	2	-0.33286	0.579907	0.565975	0.71687	0.230045	2.233919	0.992635
injuries & poisonings	Hemorrhage or hematoma complicating a procedure	Inverse variance weighted	2	0.071545	0.124858	0.566639	1.074166	0.840989	1.371995	0.992635
neurological	Parkinson's disease	Inverse variance weighted	2	-0.14882	0.263027	0.571543	0.861728	0.514609	1.442989	0.992635
sense organs	Senile cataract	Inverse variance weighted	2	0.056986	0.101075	0.572889	1.058641	0.868384	1.290583	0.992635
sense organs	Infection of the eye	Inverse variance weighted	2	0.208408	0.37075	0.574031	1.231715	0.595555	2.547409	0.992635
musculoskeletal	Contracture of palmar fascia [Dupuytren's disease]	Inverse variance weighted	2	0.179592	0.319707	0.574294	1.196729	0.639522	2.239421	0.992635
genitourinary	Cyst or abscess of Bartholin's gland	Inverse variance weighted	2	0.273037	0.486702	0.574801	1.313949	0.506162	3.41089	0.992635
respiratory	Postinflammatory pulmonary fibrosis	Inverse variance weighted	2	-0.19843	0.354722	0.575886	0.820015	0.409144	1.643491	0.992635
injuries & poisonings	Complications of surgical and medical procedures	Inverse variance weighted	2	-0.05305	0.09513	0.577102	0.948336	0.787021	1.142716	0.992635
circulatory system	Other disorders of circulatory system	Inverse variance weighted	2	-0.07173	0.129095	0.578442	0.930779	0.722702	1.198766	0.992635
musculoskeletal	Osteoarthritis; localized	Inverse variance weighted	2	-0.04897	0.08862	0.580556	0.95221	0.800383	1.132839	0.992635
dermatologic	Other local infections of skin and subcutaneous tissue	Inverse variance weighted	2	0.078369	0.141851	0.580624	1.081521	0.81901	1.428174	0.992635
injuries & poisonings	Adverse drug events and drug allergies	Inverse variance weighted	2	-0.18389	0.334391	0.582372	0.832028	0.432014	1.602424	0.992635
circulatory system	Occlusion and stenosis of precerebral arteries	Inverse variance weighted	2	-0.14093	0.257079	0.583554	0.868549	0.524765	1.437556	0.992635
genitourinary	Inflammatory disease of cervix, vagina, and vulva	Inverse variance weighted	2	0.197968	0.361454	0.583899	1.218923	0.600206	2.475438	0.992635
injuries & poisonings	Mechanical complication of unspecified genitourinary device, implant, and graft	Inverse variance weighted	2	-0.13738	0.251131	0.584356	0.871641	0.532808	1.425953	0.992635
pregnancy complicatic	Missed abortion/Hydatidiform mole	Inverse variance weighted	2	0.136698	0.251131	0.586216	1.146482	0.700809	1.875575	0.992635
neoplasms	Benign neoplasm of other parts of digestive system	Inverse variance weighted	2	0.067613	0.124858	0.588151	1.069951	0.837689	1.366611	0.992635
digestive	Personal history of diseases of digestive system	Inverse variance weighted	2	-0.03893	0.072025	0.588813	0.961815	0.835185	1.107644	0.992635
musculoskeletal	Intervertebral disc disorders	Inverse variance weighted	2	0.142433	0.263872	0.589347	1.153076	0.687457	1.934061	0.992635
endocrine/metabolic	Disorders of fluid, electrolyte, and acid-base balance	Inverse variance weighted	2	-0.05758	0.107021	0.590531	0.944042	0.765409	1.164366	0.992635
neoplasms	Colon cancer	Inverse variance weighted	2	-0.08669	0.161249	0.590827	0.916958	0.668485	1.257787	0.992635
infectious diseases	E. coli	Inverse variance weighted	2	-0.08985	0.167196	0.590976	0.914064	0.658653	1.268518	0.992635
endocrine/metabolic	Electrolyte imbalance	Inverse variance weighted	2	0.07352	0.137461	0.592758	1.07629	0.822092	1.409088	0.992635
respiratory	Chronic sinusitis	Inverse variance weighted	2	-0.09235	0.173142	0.593788	0.911789	0.649401	1.280195	0.992635
musculoskeletal	Degeneration of intervertebral disc	Inverse variance weighted	2	0.22673	0.426491	0.594992	1.254491	0.543789	2.894038	0.992635
endocrine/metabolic	Nontoxic nodular goiter	Inverse variance weighted	2	0.139367	0.263027	0.596211	1.149545	0.686489	1.924947	0.992635
respiratory	Shortness of breath	Inverse variance weighted	2	-0.06017	0.11367	0.596544	0.9416	0.753544	1.176588	0.992635
genitourinary	Postmenopausal atrophic vaginitis	Inverse variance weighted	2	0.13853	0.263027	0.598418	1.148584	0.685915	1.923338	0.992635
genitourinary	Nephritis; nephrosis; renal sclerosis	Inverse variance weighted	2	0.188528	0.358275	0.598741	1.207471	0.598283	2.436952	0.992635
genitourinary	Menopausal and postmenopausal disorders	Inverse variance weighted	2	0.052969	0.100711	0.598925	1.054397	0.86552	1.284491	0.992635
digestive	Other specified gastritis	Inverse variance weighted	2	-0.05287	0.101075	0.600896	0.9485	0.778037	1.15631	0.992635
neoplasms	Acquired absence of breast	Inverse variance weighted	2	0.093647	0.179751	0.602381	1.098172	0.772081	1.561987	0.992635
sense organs	Aphakia and other disorders of lens	Inverse variance weighted	2	-0.10808	0.208822	0.604746	0.897553	0.596084	1.351489	0.992635
neoplasms	Non-Hodgkins lymphoma	Inverse variance weighted	2	-0.10773	0.208822	0.605913	0.897866	0.596292	1.351961	0.992635
neoplasms	Benign neoplasm of brain and other parts of nervous system	Inverse variance weighted	2	0.241617	0.468575	0.606105	1.273306	0.508245	3.19001	0.992635
genitourinary	Abnormal findings on examination of urine	Inverse variance weighted	2	-0.07698	0.149355	0.606276	0.925911	0.690933	1.240803	0.992635
digestive	Disturbances in tooth eruption	Inverse variance weighted	2	-0.09256	0.179751	0.606597	0.911594	0.640906	1.296608	0.992635
infectious diseases	Viral warts & HPV	Inverse variance weighted	2	0.226683	0.442401	0.608376	1.254432	0.52707	2.985563	0.992635
pregnancy complicatic	Umbilical cord complications during labor and delivery	Inverse variance weighted	2	-0.36728	0.728138	0.613972	0.692615	0.166222	2.885994	0.992635
sense organs	Otitis media and Eustachian tube disorders	Inverse variance weighted	2	-0.13483	0.268527	0.615592	0.873865	0.516261	1.479174	0.992635
respiratory	Acute upper respiratory infections of multiple or unspecified sites	Inverse variance weighted	2	-0.1462	0.291216	0.615652	0.863988	0.488224	1.528958	0.992635
circulatory system	Left bundle branch block	Inverse variance weighted	2	-0.16884	0.336386	0.61572	0.844643	0.436853	1.633094	0.992635
musculoskeletal	Other specified osteoporosis	Inverse variance weighted	2	-0.25502	0.508889	0.61628	0.774901	0.285806	2.100978	0.992635
musculoskeletal	Other unspecified back disorders	Inverse variance weighted	2	-0.11061	0.220714	0.616284	0.895292	0.580884	1.379877	0.992635
circulatory system	Cardiac arrest and ventricular fibrillation	Inverse variance weighted	2	0.211094	0.425286	0.619641	1.235029	0.53662	2.842416	0.992635
circulatory system	Rheumatic disease of the heart valves	Inverse variance weighted	2	-0.06201	0.125566	0.621429	0.939876	0.73483	1.202137	0.992635
digestive	Gastric ulcer	Inverse variance weighted	2	0.091294	0.185722	0.623029	1.095591	0.761305	1.576661	0.992635
circulatory system	Varicose veins of lower extremity, symptomtic	Inverse variance weighted	2	0.243686	0.495833	0.623095	1.275944	0.482803	3.37204	0.992635
hematopoietic	Other deficiency anemia	Inverse variance weighted	2	0.154723	0.314861	0.623143	1.167335	0.629767	2.163769	0.992635
neurological	Nerve root and plexus disorders	Inverse variance weighted	2	0.12842	0.262337	0.624471	1.137031	0.679933	1.901422	0.992635
neoplasms	Cancer of mouth	Inverse variance weighted	2	-0.28159	0.583295	0.629274	0.754587	0.240546	2.367119	0.992635
dermatologic	Dyschromia and Vitiligo	Inverse variance weighted	2	0.142371	0.295839	0.630342	1.153005	0.645666	2.058991	0.992635
circulatory system	Noninfectious disorders of lymphatic channels	Inverse variance weighted	2	0.160734	0.334391	0.630746	1.174373	0.60977	2.261756	0.992635
dermatologic	Cellulitis and abscess of fingers/toes	Inverse variance weighted	2	0.177632	0.370071	0.631232	1.194385	0.578274	2.466923	0.992635
circulatory system	Paroxysmal supraventricular tachycardia	Inverse variance weighted	2	0.088801	0.185036	0.631291	1.092863	0.760431	1.57062	0.992635
respiratory	Empyema and pneumothorax	Inverse variance weighted	2	-0.29301	0.611141	0.631622	0.746015	0.225182	2.471507	0.992635
neoplasms	Malignant neoplasm of rectum, rectosigmoid junction, and anus	Inverse variance weighted	2	0.100531	0.210034	0.632194	1.105758	0.732614	1.668954	0.992635
genitourinary	Polyp of corpus uteri	Inverse variance weighted	2	0.048586	0.101773	0.633083	1.049785	0.859942	1.281539	0.992635
digestive	Gastrointestinal hemorrhage	Inverse variance weighted	2	-0.06262	0.131557	0.634055	0.939296	0.725803	1.215588	0.992635
genitourinary	Pelvic inflammatory disease (PID)	Inverse variance weighted	2	-0.13598	0.286816	0.635416	0.872856	0.497508	1.531388	0.992635
musculoskeletal	Disorders of muscle, ligament, and fascia	Inverse variance weighted	2	0.140157	0.295824	0.635652	1.150455	0.644257	2.054377	0.992635
symptoms	Symptoms involving nervous and musculoskeletal systems	Inverse variance weighted	2	-0.09601	0.202875	0.636036	0.908455	0.610397	1.352054	0.992635
digestive	Acute appendicitis	Inverse variance weighted	2	-0.08179	0.173142	0.636664	0.921468	0.656294	1.293785	0.992635
circulatory system	First degree AV block	Inverse variance weighted	2	0.131686	0.280869	0.639174	1.14075	0.657825	1.978202	0.992635
dermatologic	Cellulitis and abscess of leg, except foot	Inverse variance weighted	2	0.055907	0.119618	0.640226	1.0575	0.836487	1.336907	0.992635
circulatory system	Aortic valve disease	Inverse variance weighted	2	-0.11681	0.250445	0.640912	0.889751	0.544609	1.453623	0.992635
musculoskeletal	Acquired foot deformities	Inverse variance weighted	2	-0.04317	0.092942	0.642273	0.957745	0.798245	1.149115	0.992635
genitourinary	Pain and other symptoms associated with female genital organs	Inverse variance weighted	2	-0.09421	0.204668	0.64529	0.91009	0.609351	1.359255	0.992635
genitourinary	Glomerulonephritis	Inverse variance weighted	2	0.187655	0.409873	0.64707	1.206417	0.540264	2.693943	0.992635
congenital anomalies	Cardiac congenital anomalies	Inverse variance weighted	2	-0.13714	0.300073	0.647661	0.87185	0.484189	1.569889	0.992635

circulatory system	Mitral valve disease	Inverse variance weighted	2	-0.07358	0.161249	0.648178	0.929064	0.677311	1.274394	0.992635
congenital anomalies	Other congenital musculoskeletal anomalies	Inverse variance weighted	2	0.165591	0.364125	0.649278	1.180091	0.578051	2.409155	0.992635
circulatory system	Angina pectoris	Inverse variance weighted	2	0.035146	0.077293	0.649314	1.035771	0.890166	1.205194	0.992635
endocrine/metabolic	Hyperparathyroidism	Inverse variance weighted	2	0.143182	0.316551	0.65104	1.153939	0.620482	2.146034	0.992635
sense organs	Strabismus (not specified as paralytic)	Inverse variance weighted	2	0.217763	0.483056	0.652132	1.243292	0.482378	3.204491	0.992635
neoplasms	Hemangioma and lymphangioma, any site	Inverse variance weighted	2	-0.099	0.220714	0.653758	0.905742	0.587664	1.395983	0.992635
dermatologic	Carbuncle and furuncle	Inverse variance weighted	2	0.082963	0.185036	0.65389	1.086502	0.756006	1.561479	0.992635
genitourinary	Cyst of kidney, acquired	Inverse variance weighted	2	-0.11225	0.250445	0.653996	0.893817	0.547098	1.460266	0.992635
dermatologic	Sebaceous cyst	Inverse variance weighted	2	0.042822	0.095825	0.654962	1.043752	0.865027	1.259404	0.992635
circulatory system	Heart valve replaced	Inverse variance weighted	2	-0.11826	0.265462	0.655955	0.888461	0.528047	1.494873	0.992635
circulatory system	Pericarditis	Inverse variance weighted	2	-0.20159	0.453224	0.656466	0.817427	0.336246	1.987199	0.992635
congenital anomalies	Cardiac shunt/ heart septal defect	Inverse variance weighted	2	-0.16194	0.364125	0.656516	0.850495	0.416603	1.736285	0.992635
dermatologic	Diseases of sebaceous glands	Inverse variance weighted	2	0.042258	0.095825	0.659216	1.043164	0.86454	1.258694	0.992635
circulatory system	Coronary atherosclerosis	Inverse variance weighted	2	0.029134	0.066073	0.65926	1.029562	0.904503	1.171912	0.992635
musculoskeletal	Acquired spondylolisthesis	Inverse variance weighted	2	-0.22406	0.510812	0.660929	0.799269	0.293685	2.175228	0.992635
pregnancy complicatic	Normal delivery	Inverse variance weighted	2	-0.09157	0.208822	0.661027	0.9125	0.606011	1.373995	0.992635
injuries & poisonings	Complication of internal orthopedic device	Inverse variance weighted	2	-0.12469	0.284628	0.661324	0.882769	0.50532	1.542153	0.992635
congenital anomalies	Cardiac and circulatory congenital anomalies	Inverse variance weighted	2	-0.13839	0.317683	0.663112	0.87076	0.467176	1.62299	0.992635
digestive	Dental caries	Inverse variance weighted	2	-0.07009	0.161249	0.66381	0.932312	0.679678	1.278848	0.992635
musculoskeletal	Fasciitis	Inverse variance weighted	2	0.139184	0.321685	0.665252	1.149336	0.61182	2.159089	0.992635
musculoskeletal	Arthropathy NOS	Inverse variance weighted	2	-0.02195	0.050753	0.665441	0.978293	0.885659	1.080615	0.992635
endocrine/metabolic	Disorders of lipoid metabolism	Inverse variance weighted	2	0.031618	0.073132	0.665492	1.032123	0.894294	1.191195	0.992635
circulatory system	Nonrheumatic mitral valve disorders	Inverse variance weighted	2	-0.07151	0.167196	0.668882	0.93099	0.67085	1.292007	0.992635
neurological	Other headache syndromes	Inverse variance weighted	2	0.043235	0.101773	0.670968	1.044184	0.855353	1.274701	0.992635
genitourinary	Pyelonephritis	Inverse variance weighted	2	0.103797	0.244499	0.671181	1.109375	0.687	1.791431	0.992635
digestive	Diseases of hard tissues of teeth	Inverse variance weighted	2	-0.06841	0.161249	0.6714	0.933881	0.680822	1.281001	0.992635
pregnancy complicatic	Hemorrhage in early pregnancy	Inverse variance weighted	2	0.139701	0.333993	0.675746	1.14993	0.597545	2.212951	0.992635
sense organs	Other disorders of eyelids	Inverse variance weighted	2	0.071937	0.173863	0.67905	1.074588	0.76427	1.510905	0.992635
endocrine/metabolic	Vitamin B-complex deficiencies	Inverse variance weighted	2	0.133413	0.322498	0.679103	1.142722	0.60733	2.150089	0.992635
digestive	Acute gastritis	Inverse variance weighted	2	-0.10621	0.257079	0.679505	0.899236	0.543305	1.488346	0.992635
digestive	Cholelithiasis with other cholecystitis	Inverse variance weighted	2	-0.04927	0.119618	0.68043	0.951926	0.752978	1.203439	0.992635
symptoms	Neuralgia, neuritis, and radiculitis NOS	Inverse variance weighted	2	0.173644	0.423163	0.681552	1.189631	0.519049	2.726569	0.992635
genitourinary	Pelvic peritoneal adhesions, female (postoperative) (postinfection)	Inverse variance weighted	2	-0.07114	0.173801	0.682324	0.931335	0.662466	1.309329	0.992635
endocrine/metabolic	Disorders of mineral metabolism	Inverse variance weighted	2	-0.11191	0.273611	0.682521	0.894121	0.522991	1.528617	0.992635
digestive	Reflux esophagitis	Inverse variance weighted	2	-0.03642	0.089184	0.682974	0.964232	0.809592	1.148409	0.992635
circulatory system	Abdominal aortic aneurysm	Inverse variance weighted	2	-0.27551	0.676631	0.683872	0.759182	0.201551	2.8596	0.992635
genitourinary	Uterine/Uterovaginal prolapse	Inverse variance weighted	2	-0.16118	0.396694	0.684508	0.851135	0.391134	1.852129	0.992635
digestive	Jaundice (not of newborn)	Inverse variance weighted	2	-0.17541	0.433451	0.685704	0.83911	0.358805	1.962363	0.992635
injuries & poisonings	Fracture of ankle and foot	Inverse variance weighted	2	-0.07442	0.185036	0.687552	0.928284	0.645915	1.334094	0.992635
musculoskeletal	Spinal stenosis	Inverse variance weighted	2	-0.0597	0.14864	0.68795	0.942048	0.70396	1.26066	0.992635
infectious diseases	Intestinal infection due to C. difficile	Inverse variance weighted	2	0.195056	0.489958	0.69055	1.21538	0.465213	3.17521	0.992635
dermatologic	Superficial cellulitis and abscess	Inverse variance weighted	2	0.040261	0.101773	0.692403	1.041083	0.852813	1.270915	0.992635
musculoskeletal	Symptoms and disorders of the joints	Inverse variance weighted	2	0.058754	0.14864	0.692638	1.060514	0.792486	1.419194	0.992635
neoplasms	Cancer of other lymphoid, histiocytic tissue	Inverse variance weighted	2	-0.09552	0.242112	0.693201	0.908903	0.565494	1.460856	0.992635
digestive	Peptic ulcer (excl. esophageal)	Inverse variance weighted	2	0.04208	0.107021	0.69418	1.042977	0.845623	1.286391	0.992635
injuries & poisonings	Skull and face fracture and other intercranial injury	Inverse variance weighted	2	0.063191	0.161249	0.695143	1.06523	0.776579	1.461172	0.992635
injuries & poisonings	Complication of colostomy or enterostomy	Inverse variance weighted	2	-0.15443	0.39454	0.695484	0.856901	0.39545	1.856821	0.992635
genitourinary	Benign mammary dysplasias	Inverse variance weighted	2	0.124105	0.322079	0.699996	1.132135	0.602198	2.12842	0.992635
respiratory	Other dyspnea	Inverse variance weighted	2	0.112373	0.292763	0.7011	1.11893	0.630374	1.98613	0.992635
circulatory system	Other venous embolism and thrombosis	Inverse variance weighted	2	0.141956	0.37075	0.701802	1.152526	0.557266	2.383631	0.992635
circulatory system	Phlebitis and thrombophlebitis of lower extremities	Inverse variance weighted	2	0.057172	0.149355	0.701872	1.058838	0.790126	1.418937	0.992635
circulatory system	Elevated blood pressure reading without diagnosis of hypertension	Inverse variance weighted	2	0.086945	0.22734	0.70213	1.090837	0.698625	1.70324	0.992635
mental disorders	Anxiety disorders	Inverse variance weighted	2	-0.04241	0.111726	0.704237	0.958475	0.769976	1.19312	0.992635
circulatory system	Hypotension	Inverse variance weighted	2	0.04244	0.11367	0.708877	1.043354	0.834976	1.303736	0.992635
endocrine/metabolic	Hyperlipidemia	Inverse variance weighted	2	0.029065	0.078342	0.710639	1.029491	0.882951	1.200353	0.992635
musculoskeletal	Derangement of joint, non-traumatic	Inverse variance weighted	2	0.061944	0.167196	0.711021	1.063902	0.766623	1.47646	0.992635
circulatory system	Ischemic Heart Disease	Inverse variance weighted	2	0.025296	0.068397	0.711501	1.025619	0.896944	1.172753	0.992635
endocrine/metabolic	Nontoxic multinodular goiter	Inverse variance weighted	2	0.124267	0.340338	0.715016	1.132318	0.581121	2.206328	0.992635
respiratory	Bronchiectasis	Inverse variance weighted	2	-0.13705	0.377323	0.71644	0.871925	0.416194	1.826682	0.992635
genitourinary	Hypertrophy of female genital organs	Inverse variance weighted	2	0.106811	0.294481	0.716822	1.112724	0.62477	1.981778	0.992635
digestive	Other intestinal obstruction	Inverse variance weighted	2	-0.05608	0.155302	0.718031	0.945465	0.697348	1.281862	0.992635
mental disorders	Anxiety disorder	Inverse variance weighted	2	-0.04051	0.112967	0.719903	0.960301	0.76957	1.198303	0.992635
neoplasms	Malignant neoplasm of testis	Inverse variance weighted	2	0.051091	0.143408	0.721646	1.052418	0.794542	1.39399	0.992635
genitourinary	Prolapse of vaginal walls	Inverse variance weighted	2	0.038044	0.107021	0.722232	1.038776	0.842217	1.28121	0.992635
dermatologic	Disorder of skin and subcutaneous tissue NOS	Inverse variance weighted	2	0.040317	0.11367	0.722827	1.041141	0.833204	1.30097	0.992635
digestive	Symptoms involving digestive system	Inverse variance weighted	2	-0.05216	0.147091	0.722886	0.949178	0.711445	1.266351	0.992635
neoplasms	Cancer of esophagus	Inverse variance weighted	2	-0.11574	0.328445	0.724549	0.890707	0.467905	1.695558	0.992635
mental disorders	Memory loss	Inverse variance weighted	2	-0.11781	0.334391	0.724612	0.888868	0.461527	1.711894	0.992635
neoplasms	Benign neoplasm of colon	Inverse variance weighted	2	0.03947	0.112212	0.725029	1.040259	0.834882	1.296158	0.992635
musculoskeletal	Other and unspecified disorders of back	Inverse variance weighted	2	-0.06916	0.196929	0.725455	0.93318	0.634361	1.37276	0.992635
genitourinary	Other signs and symptoms in breast	Inverse variance weighted	2	0.117251	0.334391	0.725858	1.124402	0.583824	2.165515	0.992635
neurological	Epilepsy, recurrent seizures, convulsions	Inverse variance weighted	2	-0.07971	0.232159	0.731333	0.923382	0.585819	1.455456	0.992635
musculoskeletal	Rheumatism, unspecified and fibrositis	Inverse variance weighted	2	-0.09639	0.280869	0.731457	0.908109	0.52367	1.574774	0.992635
injuries & poisonings	Intracranial hemorrhage (injury)	Inverse variance weighted	2	0.134851	0.393857	0.73206	1.144367	0.528819	2.476415	0.992635
sense organs	Otitis externa	Inverse variance weighted	2	0.131427	0.387911	0.734754	1.140455	0.53319	2.439353	0.992635
hematopoietic	Disorders of iron metabolism	Inverse variance weighted	2	0.115031	0.340338	0.73537	1.121908	0.575779	2.186045	0.992635
congenital anomalies	Congenital anomalies of urinary system	Inverse variance weighted	2	-0.18658	0.554289	0.73641	0.829792	0.279994	2.459176	0.992635
dermatologic	Psoriasis and related disorders	Inverse variance weighted	2	-0.062	0.185036	0.737585	0.939886	0.653988	1.350768	0.992635
neurological	Hemiplegia	Inverse variance weighted	2	-0.07614	0.22734	0.737679	0.926684	0.593493	1.44693	0.992635
pregnancy complicatic	Late pregnancy and failed induction	Inverse variance weighted	2	0.085346	0.256391	0.739229	1.089094	0.658902	1.800156	0.992635
musculoskeletal	Displacement of intervertebral disc	Inverse variance weighted	2	0.128185	0.388592	0.741498	1.136763	0.530754	2.434706	0.992635
genitourinary	Noninflammatory female genital disorders	Inverse variance weighted	2	0.029602	0.089876	0.74188	1.030044	0.863679	1.228456	0.992635
respiratory	Respiratory abnormalities	Inverse variance weighted	2	0.117584	0.358178	0.742698	1.124776	0.557415	2.269621	0.992635
endocrine/metabolic	Gout	Inverse variance weighted	2	-0.05103	0.155951	0.7435	0.950249	0.699986	1.289989	0.992635
digestive	Diverticulosis and diverticulitis	Inverse variance weighted	2	-0.01911	0.058414	0.743503	0.981068	0.874936	1.100073	0.992635
circulatory system	Pulmonary heart disease	Inverse variance weighted	2	-0.04488	0.137461	0.744048	0.956112	0.730297	1.25175	0.992635
neoplasms	Cancer, suspected or other	Inverse variance weighted	2	-0.02319	0.071347	0.745166	0.977078	0.849566	1.123728	0.992635
circulatory system	Carditis	Inverse variance weighted	2	-0.1254	0.385898	0.745217	0.882145	0.414054	1.879418	0.992635
mental disorders	Delirium dementia and amnestic and other cognitive disorders	Inverse variance weighted	2	-0.06591	0.202875	0.74528	0.936217	0.629051	1.393373	0.992635
digestive	Cholecystitis without cholelithiasis	Inverse variance weighted	2	-0.05419	0.167196	0.745869	0.947255	0.68257	1.31458	0.992635
genitourinary	Cervicitis and endocervicitis	Inverse variance weighted	2	0.130376	0.402436	0.745964	1.139256	0.517679	2.50716	0.992635
digestive	Periapical abscess	Inverse variance weighted	2	-0.08325	0.257079	0.746052	0.920117	0.555921	1.522905	0.992635
pregnancy complicatic	Known or suspected fetal abnormality affecting management of mother	Inverse variance weighted	2	0.122269	0.379698	0.74744	1.130058	0.536902	2.378518	0.992635
digestive	Hemorrhage of gastrointestinal tract	Inverse variance weighted	2	-0.05825	0.18122	0.747869	0.943411	0.661369	1.345731	0.992635
genitourinary	Stricture/obstruction of ureter	Inverse variance weighted	2	-0.09478	0.297492	0.750044	0.909577	0.507702	1.629559	0.992635
digestive	Appendiceal conditions	Inverse variance weighted	2	-0.04758	0.149355	0.750063	0.953536	0.711547	1.277823	0.992635
digestive	Diverticulosis	Inverse variance weighted	2	-0.01838	0.057889	0.750814	0.981784	0.876475	1.099746	0.992635
neoplasms	Cancer of bronchus; lung	Inverse variance weighted	2	0.064755	0.204539	0.751556	1.066897	0.714522	1.593052	0.992635
genitourinary	Cystitis	Inverse variance weighted	2	-0.07631	0.242722	0.753217	0.926527	0.575769	1.490965	0.992635

respiratory	Epistaxis or throat hemorrhage	Inverse variance weighted	2	-0.05565	0.179089	0.75601	0.945873	0.665869	1.34362	0.992635
musculoskeletal	Joint effusions	Inverse variance weighted	2	0.072113	0.233288	0.757232	1.074777	0.680361	1.697842	0.992635
dermatologic	Degenerative skin conditions and other dermatoses	Inverse variance weighted	2	0.036784	0.119618	0.758453	1.037469	0.820643	1.311584	0.992635
mental disorders	Swelling, mass, or lump in head and neck [Space-occupying lesion, intracranial NOS	Inverse variance weighted	2	0.098111	0.323162	0.761435	1.103085	0.585502	2.078211	0.992635
symptoms	Pain	Inverse variance weighted	2	-0.09609	0.316551	0.761463	0.90838	0.488443	1.689356	0.992635
circulatory system	Nonspecific chest pain	Inverse variance weighted	2	-0.02146	0.072103	0.765962	0.978766	0.849774	1.127339	0.992635
neoplasms	Benign neoplasm of uterus	Inverse variance weighted	2	0.026486	0.089184	0.766479	1.02684	0.86216	1.222976	0.992635
musculoskeletal	Other arthropathies	Inverse variance weighted	2	-0.01466	0.049632	0.767742	0.985449	0.894102	1.086129	0.992635
sense organs	Vertiginous syndromes and other disorders of vestibular system	Inverse variance weighted	2	-0.03339	0.11367	0.768923	0.967157	0.773997	1.208523	0.992635
circulatory system	Arrhythmia (cardiac) NOS	Inverse variance weighted	2	0.085073	0.292763	0.771368	1.088796	0.613397	1.932642	0.992635
neoplasms	Malignant neoplasm of kidney, except pelvis	Inverse variance weighted	2	-0.08008	0.280176	0.775025	0.923046	0.533007	1.598503	0.992635
neoplasms	Neoplasm of unspecified nature of digestive system	Inverse variance weighted	2	-0.08029	0.281384	0.775392	0.922851	0.531634	1.601956	0.992635
dermatologic	Disturbance of skin sensation	Inverse variance weighted	2	0.050754	0.177904	0.775425	1.052064	0.742347	1.490998	0.992635
circulatory system	Hypotension NOS	Inverse variance weighted	2	0.042459	0.149355	0.776193	1.043374	0.778585	1.398213	0.992635
genitourinary	Calculus of ureter	Inverse variance weighted	2	0.050265	0.179751	0.779754	1.05155	0.739304	1.495675	0.992635
circulatory system	Heart valve disorders	Inverse variance weighted	2	-0.03844	0.137461	0.779777	0.962294	0.73502	1.259843	0.992635
pregnancy complicatic	Miscarriage; stillbirth	Inverse variance weighted	2	0.05625	0.201251	0.779859	1.057862	0.713051	1.569414	0.992635
endocrine/metabolic	Disorders of parathyroid gland	Inverse variance weighted	2	0.083431	0.29871	0.78001	1.087011	0.605295	1.952095	0.992635
mental disorders	Dementias	Inverse variance weighted	2	-0.07953	0.286816	0.781563	0.923551	0.526403	1.62033	0.992635
musculoskeletal	Other disorders of soft tissues	Inverse variance weighted	2	0.031282	0.11367	0.783163	1.031776	0.82571	1.289269	0.992635
sense organs	Otitis media	Inverse variance weighted	2	-0.0917	0.337169	0.785645	0.912379	0.471163	1.766766	0.992635
digestive	Femoral hernia	Inverse variance weighted	2	-0.09224	0.346285	0.789944	0.911882	0.462567	1.797638	0.992635
injuries & poisonings	Burns	Inverse variance weighted	2	-0.09667	0.364802	0.791009	0.907853	0.444109	1.855843	0.992635
injuries & poisonings	Open wounds of head; neck; and trunk	Inverse variance weighted	2	0.037815	0.143408	0.792022	1.038539	0.784064	1.375605	0.992635
neurological	Multiple sclerosis	Inverse variance weighted	2	-0.06307	0.239236	0.792052	0.938874	0.587443	1.500546	0.992635
digestive	Diseases of the salivary glands	Inverse variance weighted	2	-0.08565	0.329114	0.794673	0.917914	0.481565	1.749644	0.992635
musculoskeletal	Peripheral enthesopathies and allied syndromes	Inverse variance weighted	2	0.019937	0.077293	0.796455	1.020137	0.876729	1.187002	0.992635
digestive	Other diseases of the teeth and supporting structures	Inverse variance weighted	2	-0.0441	0.173142	0.798972	0.956863	0.681503	1.343481	0.992635
sense organs	Retinal detachments and defects	Inverse variance weighted	2	0.062427	0.246669	0.800206	1.064417	0.656362	1.726158	0.992635
endocrine/metabolic	Disorders of calcium/phosphorus metabolism	Inverse variance weighted	2	-0.10164	0.410842	0.804612	0.903359	0.403779	2.021047	0.992635
circulatory system	Hemorrhoids	Inverse variance weighted	2	-0.01464	0.059456	0.805511	0.985467	0.877066	1.107267	0.992635
injuries & poisonings	Fracture of tibia and fibula	Inverse variance weighted	2	-0.04673	0.190982	0.806689	0.954342	0.656352	1.387623	0.992635
digestive	Diseases of lips	Inverse variance weighted	2	0.083194	0.340338	0.806886	1.086753	0.557736	2.117544	0.992635
injuries & poisonings	Complication due to other implant and internal device	Inverse variance weighted	2	0.037935	0.155302	0.807024	1.038664	0.766089	1.408221	0.992635
symptoms	Effects of other external causes	Inverse variance weighted	2	0.032998	0.137461	0.810287	1.033549	0.789445	1.353131	0.992635
respiratory	Pneumonitis due to inhalation of food or vomitus	Inverse variance weighted	2	-0.08638	0.364802	0.812815	0.917242	0.448702	1.875036	0.992635
dermatologic	Diseases of hair and hair follicles	Inverse variance weighted	2	-0.02952	0.124858	0.813093	0.97091	0.760148	1.24011	0.992635
digestive	Other disorders of stomach and duodenum	Inverse variance weighted	2	-0.08213	0.348668	0.813775	0.921151	0.465092	1.824411	0.992635
sense organs	Ptosis of eyelid	Inverse variance weighted	2	0.130155	0.55797	0.815555	1.139005	0.381568	3.400004	0.992635
digestive	Diseases of pancreas	Inverse variance weighted	2	0.063966	0.275677	0.816513	1.066056	0.621039	1.829958	0.992635
circulatory system	Subarachnoid hemorrhage	Inverse variance weighted	2	-0.07158	0.310604	0.817727	0.930917	0.50643	1.711207	0.992635
musculoskeletal	Hallux valgus (Bunion)	Inverse variance weighted	2	-0.03337	0.146302	0.819555	0.967177	0.726057	1.28837	0.992635
neoplasms	Other benign neoplasm of connective and other soft tissue	Inverse variance weighted	2	0.180908	0.796095	0.820234	1.198305	0.25172	5.704496	0.992635
pregnancy complicatic	Obstetrical/birth trauma	Inverse variance weighted	2	-0.0388	0.172669	0.822209	0.961944	0.685758	1.349362	0.992635
musculoskeletal	Other derangement of joint	Inverse variance weighted	2	-0.04424	0.196929	0.822238	0.956721	0.650364	1.40739	0.992635
respiratory	Pneumonia	Inverse variance weighted	2	-0.03182	0.14257	0.823361	0.968676	0.732522	1.280962	0.992635
mental disorders	Alcohol-related disorders	Inverse variance weighted	2	0.017264	0.077976	0.824778	1.017414	0.87322	1.185419	0.992635
neoplasms	Skin cancer	Inverse variance weighted	2	0.033876	0.153433	0.825258	1.034456	0.765786	1.397387	0.992635
musculoskeletal	Aseptic necrosis of bone	Inverse variance weighted	2	-0.08303	0.381964	0.82791	0.920321	0.435316	1.945692	0.992635
circulatory system	Abnormal heart sounds	Inverse variance weighted	2	0.059569	0.274921	0.82846	1.061379	0.619231	1.819233	0.992635
infectious diseases	Viral Enteritis	Inverse variance weighted	2	-0.09601	0.444596	0.829036	0.908459	0.380065	2.171468	0.992635
genitourinary	Other abnormality of urination	Inverse variance weighted	2	0.079711	0.369378	0.829144	1.082975	0.525046	2.233774	0.992635
injuries & poisonings	Fracture of foot	Inverse variance weighted	2	0.044346	0.208822	0.831822	1.045344	0.694236	1.574026	0.992635
dermatologic	Scar conditions and fibrosis of skin	Inverse variance weighted	2	0.039362	0.188736	0.834795	1.040147	0.718521	1.505739	0.992635
dermatologic	Pruritus and related conditions	Inverse variance weighted	2	-0.08985	0.431439	0.835027	0.914067	0.392401	2.12925	0.992635
circulatory system	Heart failure NOS	Inverse variance weighted	2	0.036837	0.17805	0.836096	1.037523	0.731878	1.470811	0.992635
injuries & poisonings	Adverse effects of sedatives or other central nervous system depressants and anesth	Inverse variance weighted	2	0.075291	0.364125	0.836188	1.078198	0.52814	2.20114	0.992635
injuries & poisonings	Poisoning by antibiotics	Inverse variance weighted	2	0.013661	0.066073	0.836201	1.013755	0.890616	1.153919	0.992635
circulatory system	Nonspecific abnormal findings on radiological and other examination of other intrat	Inverse variance weighted	2	0.107162	0.520232	0.836799	1.113115	0.401523	3.085816	0.992635
neoplasms	Leukemia	Inverse variance weighted	2	-0.04461	0.218964	0.838562	0.95637	0.622644	1.468968	0.992635
genitourinary	Inflammatory diseases of female pelvic organs	Inverse variance weighted	2	0.023013	0.11367	0.839564	1.02328	0.81891	1.278652	0.992635
sense organs	Suppurative and unspecified otitis media	Inverse variance weighted	2	-0.06072	0.30396	0.841653	0.941082	0.518671	1.70751	0.992635
genitourinary	Benign neoplasm of breast	Inverse variance weighted	2	-0.07339	0.368149	0.841983	0.929235	0.451597	1.912055	0.992635
infectious diseases	Septicemia	Inverse variance weighted	2	-0.02726	0.137461	0.8428	0.973108	0.74328	1.274001	0.992635
circulatory system	Congestive heart failure (CHF) NOS	Inverse variance weighted	2	-0.06637	0.339784	0.845127	0.935782	0.480777	1.821398	0.992635
digestive	Peritoneal adhesions (postoperative) (postinfection)	Inverse variance weighted	2	-0.03121	0.160283	0.845624	0.969274	0.707963	1.327035	0.992635
symptoms	Fever of unknown origin	Inverse variance weighted	2	-0.02746	0.143408	0.84814	0.972912	0.734518	1.288679	0.992635
neurological	Other paralytic syndromes	Inverse variance weighted	2	-0.10966	0.574624	0.848658	0.896142	0.290567	2.763803	0.992635
musculoskeletal	Bursitis	Inverse variance weighted	2	-0.06025	0.315748	0.848671	0.94153	0.507065	1.748253	0.992635
genitourinary	Renal colic	Inverse variance weighted	2	-0.06714	0.352065	0.848756	0.935064	0.468983	1.864339	0.992635
sense organs	Other disorders of eye	Inverse variance weighted	2	-0.02734	0.143408	0.848803	0.97303	0.734607	1.288836	0.992635
digestive	Celiac disease	Inverse variance weighted	2	0.039711	0.208822	0.849177	1.04051	0.691025	1.566747	0.992635
genitourinary	Other symptoms/disorders or the urinary system	Inverse variance weighted	2	-0.01105	0.058414	0.849971	0.989012	0.882021	1.108981	0.992635
dermatologic	Cellulitis and abscess of foot, toe	Inverse variance weighted	2	0.022512	0.119618	0.85072	1.022767	0.809014	1.292998	0.992635
neoplasms	Neoplasm of uncertain behavior	Inverse variance weighted	2	0.043437	0.233288	0.852292	1.044394	0.661128	1.649846	0.992635
respiratory	Voice disturbance	Inverse variance weighted	2	-0.05064	0.274022	0.853378	0.950619	0.55559	1.626517	0.992635
dermatologic	Diseases of nail, NOS	Inverse variance weighted	2	-0.04503	0.245184	0.854274	0.955967	0.591205	1.545779	0.992635
pregnancy complicatic	Early or threatened labor; hemorrhage in early pregnancy	Inverse variance weighted	2	0.032776	0.179751	0.855315	1.033319	0.726486	1.469744	0.992635
injuries & poisonings	Allergy/adverse effect of penicillin	Inverse variance weighted	2	0.013069	0.072025	0.856009	1.013155	0.879766	1.166768	0.992635
circulatory system	Other forms of chronic heart disease	Inverse variance weighted	2	0.045164	0.249244	0.856207	1.0462	0.64188	1.705201	0.992635
circulatory system	Congestive heart failure; nonhypertensive	Inverse variance weighted	2	-0.04092	0.22617	0.856409	0.959901	0.616179	1.495362	0.992635
neoplasms	Colorectal cancer	Inverse variance weighted	2	-0.02362	0.131513	0.857435	0.976652	0.754733	1.263823	0.992635
musculoskeletal	Pain in joint	Inverse variance weighted	2	0.018119	0.101075	0.857735	1.018284	0.835279	1.241383	0.992635
neoplasms	Secondary malignancy of brain/spine	Inverse variance weighted	2	0.066625	0.373615	0.858467	1.068895	0.513934	2.223116	0.992635
circulatory system	Orthostatic hypotension	Inverse variance weighted	2	-0.04351	0.244499	0.858746	0.957419	0.592899	1.546052	0.992635
endocrine/metabolic	Hypercholesterolemia	Inverse variance weighted	2	0.012764	0.072424	0.860111	1.012845	0.878809	1.167325	0.992635
infectious diseases	Staphylococcus infections	Inverse variance weighted	2	-0.0273	0.155951	0.861039	0.97307	0.716796	1.320968	0.992635
neoplasms	Cancer of bladder	Inverse variance weighted	2	-0.03112	0.179089	0.862061	0.969362	0.682405	1.376987	0.992635
injuries & poisonings	Nonspecific abnormal findings on radiological and other examination of musculosk	Inverse variance weighted	2	-0.08498	0.490872	0.862549	0.918526	0.350956	2.403977	0.992635
mental disorders	Neurological disorders	Inverse variance weighted	2	-0.02725	0.160463	0.865136	0.973115	0.710519	1.332762	0.992635
neurological	Other conditions of brain	Inverse variance weighted	2	-0.05611	0.330667	0.865248	0.945432	0.494493	1.80759	0.992635
digestive	Other disorders of peritoneum	Inverse variance weighted	2	-0.02623	0.155302	0.865862	0.974108	0.718475	1.320696	0.992635
neurological	Extrapyramidal disease and abnormal movement disorders	Inverse variance weighted	2	-0.08691	0.522666	0.867941	0.916763	0.329121	2.553639	0.992885
genitourinary	Noninflammatory disorders of ovary, fallopian tube, and broad ligament	Inverse variance weighted	2	-0.12374	0.752567	0.869401	0.883612	0.202145	3.862423	0.992885
genitourinary	Retention of urine	Inverse variance weighted	2	0.021536	0.134333	0.87263	1.02177	0.785247	1.329534	0.992885
neurological	Disorders of other cranial nerves	Inverse variance weighted	2	0.038074	0.238553	0.873194	1.038808	0.650841	1.658042	0.992885
sense organs	Other disorders of tympanic membrane	Inverse variance weighted	2	-0.06474	0.406708	0.873524	0.93731	0.422363	2.080081	0.992885
neoplasms	Cancer of kidney and renal pelvis	Inverse variance weighted	2	-0.04352	0.27423	0.873907	0.957414	0.559333	1.63881	0.992885
mental disorders	Symptoms involving head and neck	Inverse variance weighted	2	0.03017	0.195112	0.877114	1.03063	0.703104	1.510726	0.992885

neoplasms	Other non-epithelial cancer of skin	Inverse variance weighted	2	-0.03101	0.202657	0.878396	0.969469	0.651671	1.442245	0.992885
musculoskeletal	Spondylosis without myelopathy	Inverse variance weighted	2	-0.02261	0.147885	0.878503	0.977647	0.731644	1.306364	0.992885
dermatologic	Other hypertrophic and atrophic conditions of skin	Inverse variance weighted	2	0.019542	0.13257	0.882809	1.019734	0.786396	1.322308	0.992885
endocrine/metabolic	Hyperpotassemia	Inverse variance weighted	2	0.041564	0.286122	0.8845	1.04244	0.594976	1.826428	0.992885
endocrine/metabolic	Chondrocalcinosis	Inverse variance weighted	2	0.05433	0.376018	0.885115	1.055833	0.505269	2.206318	0.992885
genitourinary	Noninflammatory disorders of vulva and perineum	Inverse variance weighted	2	-0.03366	0.239236	0.888093	0.966896	0.604975	1.545331	0.992885
infectious diseases	Viral hepatitis	Inverse variance weighted	2	0.042144	0.3014	0.888798	1.043044	0.577757	1.883042	0.992885
neoplasms	Secondary malignancy of bone	Inverse variance weighted	2	0.026646	0.190982	0.889039	1.027004	0.706325	1.493274	0.992885
sense organs	Blindness and low vision	Inverse variance weighted	2	-0.0892	0.640484	0.889236	0.914662	0.260657	3.209603	0.992885
endocrine/metabolic	Other disorders of metabolism	Inverse variance weighted	2	-0.03244	0.233288	0.88942	0.968084	0.612822	1.529298	0.992885
digestive	GERD	Inverse variance weighted	2	-0.01128	0.084773	0.894185	0.988788	0.837419	1.167517	0.992885
genitourinary	Lump or mass in breast	Inverse variance weighted	2	-0.06144	0.463411	0.894518	0.940406	0.379186	2.332269	0.992885
symptoms	Syncope and collapse	Inverse variance weighted	2	-0.01518	0.115095	0.895045	0.984931	0.786023	1.234174	0.992885
injuries & poisonings	Other open wound of head and face	Inverse variance weighted	2	0.021442	0.167196	0.897954	1.021674	0.736194	1.417856	0.992885
musculoskeletal	Hallux rigidus	Inverse variance weighted	2	-0.02777	0.22666	0.902495	0.972614	0.623739	1.516623	0.992885
genitourinary	Dysmenorrhea	Inverse variance weighted	2	-0.0255	0.209495	0.903115	0.974821	0.646545	1.469776	0.992885
injuries & poisonings	Foreign body injury	Inverse variance weighted	2	-0.03089	0.256391	0.9041	0.969581	0.586597	1.602614	0.992885
respiratory	Allergic rhinitis	Inverse variance weighted	2	0.032379	0.268974	0.904181	1.032909	0.609687	1.749917	0.992885
digestive	Umbilical hernia	Inverse variance weighted	2	0.031945	0.267759	0.905033	1.032461	0.610875	1.744998	0.992885
dermatologic	Psoriasis	Inverse variance weighted	2	-0.02146	0.1857	0.907992	0.978767	0.680155	1.40848	0.992885
neoplasms	Malignant neoplasm of bladder	Inverse variance weighted	2	-0.02173	0.191649	0.909736	0.978507	0.672092	1.42462	0.992885
dermatologic	Pilonidal cyst	Inverse variance weighted	2	-0.04127	0.364125	0.909764	0.959571	0.470033	1.958965	0.992885
congenital anomalies	Congenital anomalies of great vessels	Inverse variance weighted	2	-0.05765	0.516767	0.911173	0.94398	0.342832	2.599226	0.992885
neoplasms	Malignant neoplasm, other	Inverse variance weighted	2	0.007682	0.071347	0.914256	1.007712	0.876202	1.15896	0.992885
hematopoietic	Coagulation defects	Inverse variance weighted	2	0.030233	0.286816	0.91605	1.030695	0.587473	1.808309	0.992885
digestive	Stricture and stenosis of esophagus	Inverse variance weighted	2	-0.02108	0.202875	0.917259	0.979144	0.657894	1.457262	0.992885
genitourinary	Abnormal findings on mammogram or breast exam	Inverse variance weighted	2	-0.0485	0.482254	0.919888	0.952655	0.370197	2.451538	0.992885
neoplasms	Cancer of larynx, pharynx, nasal cavities	Inverse variance weighted	2	-0.04039	0.408676	0.921276	0.960417	0.431109	2.139597	0.992885
respiratory	Diseases of the larynx and vocal cords	Inverse variance weighted	2	-0.01679	0.173142	0.92273	0.983346	0.700365	1.380665	0.992885
respiratory	Bronchitis	Inverse variance weighted	2	-0.03348	0.352232	0.924285	0.967079	0.484882	1.928801	0.992885
endocrine/metabolic	Disorders of adrenal glands	Inverse variance weighted	2	-0.03371	0.356785	0.924732	0.966855	0.480463	1.945641	0.992885
symptoms	Abdominal pain	Inverse variance weighted	2	0.009475	0.100574	0.924944	1.00952	0.828905	1.229491	0.992885
digestive	Abnormal findings on exam of gastrointestinal tract/ abdominal area	Inverse variance weighted	2	0.020222	0.215444	0.925219	1.020428	0.668949	1.556581	0.992885
genitourinary	Renal failure	Inverse variance weighted	2	0.010042	0.107021	0.925242	1.010093	0.818961	1.245831	0.992885
mental disorders	Psychogenic and somatoform disorders	Inverse variance weighted	2	0.035332	0.382645	0.926431	1.035963	0.489362	2.193099	0.992885
dermatologic	Seborrheic keratosis	Inverse variance weighted	2	0.014661	0.161249	0.927555	1.014769	0.739792	1.391955	0.992885
genitourinary	Infertility, female	Inverse variance weighted	2	-0.02176	0.244499	0.929092	0.978478	0.605939	1.580057	0.992885
sense organs	Perforation of tympanic membrane	Inverse variance weighted	2	0.036927	0.421952	0.930263	1.037617	0.4538	2.372519	0.992885
circulatory system	Late effects of cerebrovascular disease	Inverse variance weighted	2	0.021239	0.250445	0.932415	1.021466	0.625231	1.668812	0.992885
genitourinary	Dysuria	Inverse variance weighted	2	-0.04467	0.530499	0.9329	0.956316	0.338089	2.705026	0.992885
endocrine/metabolic	Abnormal glucose	Inverse variance weighted	2	-0.02832	0.340338	0.93368	0.972076	0.498883	1.894095	0.992885
genitourinary	Cystic mastopathy	Inverse variance weighted	2	-0.07458	0.944126	0.937035	0.928131	0.145866	5.905617	0.992885
dermatologic	Cellulitis and abscess of arm/hand	Inverse variance weighted	2	0.009363	0.119618	0.937609	1.009407	0.798446	1.276108	0.992885
infectious diseases	Bacterial infection NOS	Inverse variance weighted	2	-0.00607	0.077976	0.937921	0.993945	0.853077	1.158075	0.992885
injuries & poisonings	Opiates and related narcotics causing adverse effects in therapeutic use	Inverse variance weighted	2	0.020353	0.262337	0.938158	1.020562	0.610286	1.706654	0.992885
sense organs	Primary angle-closure glaucoma	Inverse variance weighted	2	-0.05838	0.75855	0.93865	0.943288	0.213282	4.171912	0.992885
digestive	Cholelithiasis	Inverse variance weighted	2	-0.0058	0.077976	0.940757	0.994222	0.853314	1.158397	0.992885
neoplasms	Uterine leiomyoma	Inverse variance weighted	2	0.006652	0.089876	0.941002	1.006674	0.844083	1.200584	0.992885
genitourinary	Postmenopausal bleeding	Inverse variance weighted	2	0.012283	0.169239	0.942141	1.012359	0.726566	1.410567	0.992885
circulatory system	Arterial embolism and thrombosis	Inverse variance weighted	2	-0.04411	0.627365	0.943946	0.956848	0.279782	3.272404	0.992885
genitourinary	Symptoms involving female genital tract	Inverse variance weighted	2	0.014235	0.204521	0.944512	1.014337	0.679345	1.514516	0.992885
neoplasms	Cancer of urinary organs (incl. kidney and bladder)	Inverse variance weighted	2	0.009511	0.137461	0.94484	1.009556	0.771119	1.32172	0.992885
genitourinary	stress incontinence, female	Inverse variance weighted	2	-0.01619	0.236107	0.945324	0.983938	0.619426	1.562954	0.992885
genitourinary	Disorders of uterus, NEC	Inverse variance weighted	2	0.010363	0.155302	0.9468	1.010417	0.745255	1.369923	0.992885
circulatory system	Phlebitis and thrombophlebitis	Inverse variance weighted	2	0.009329	0.143408	0.948132	1.009373	0.762044	1.336973	0.992885
dermatologic	Sarcoidosis	Inverse variance weighted	2	-0.02438	0.376697	0.9484	0.975916	0.466403	2.042038	0.992885
digestive	Other disorders of biliary tract	Inverse variance weighted	2	0.017338	0.274921	0.949715	1.017489	0.593625	1.744004	0.992885
neurological	Facial nerve disorders [CN7]	Inverse variance weighted	2	-0.02081	0.334943	0.950467	0.979408	0.507989	1.88831	0.992885
injuries & poisonings	Fracture of pelvis	Inverse variance weighted	2	0.052591	0.878719	0.952276	1.053998	0.188304	5.899567	0.992885
digestive	Acute pancreatitis	Inverse variance weighted	2	-0.0123	0.211524	0.953632	0.987776	0.652538	1.49524	0.992885
injuries & poisonings	Open wounds of extremities	Inverse variance weighted	2	-0.00781	0.137461	0.954664	0.992216	0.757874	1.299017	0.992885
digestive	Cholelithiasis with acute cholecystitis	Inverse variance weighted	2	-0.01265	0.22734	0.95564	0.987434	0.6324	1.541785	0.992885
dermatologic	Psoriasis vulgaris	Inverse variance weighted	2	-0.01119	0.214768	0.958433	0.988869	0.649119	1.506443	0.992885
genitourinary	Cystitis and urethritis	Inverse variance weighted	2	-0.01102	0.212621	0.958666	0.989041	0.65197	1.50038	0.992885
circulatory system	Palpitations	Inverse variance weighted	2	-0.01715	0.336064	0.959298	0.982995	0.508731	1.899392	0.992885
genitourinary	Acute renal failure	Inverse variance weighted	2	-0.00666	0.131513	0.959592	0.993359	0.767644	1.285442	0.992885
pregnancy complicatic	Antepartum hemorrhage, abruptio placentae, and placenta previa	Inverse variance weighted	2	0.014716	0.304657	0.961473	1.014825	0.55855	1.843829	0.992885
circulatory system	Endocarditis	Inverse variance weighted	2	0.016219	0.340338	0.961991	1.016351	0.521605	1.980366	0.992885
circulatory system	Bundle branch block	Inverse variance weighted	2	0.00709	0.155302	0.963588	1.007115	0.74282	1.365447	0.992885
circulatory system	Arterial embolism and thrombosis of lower extremity artery	Inverse variance weighted	2	0.033101	0.727714	0.96372	1.033655	0.248275	4.303469	0.992885
sense organs	Cholesteatoma	Inverse variance weighted	2	0.014571	0.358178	0.967551	1.014677	0.502852	2.04746	0.995522
digestive	Hemorrhage of rectum and anus	Inverse variance weighted	2	-0.00799	0.212982	0.970068	0.99204	0.653485	1.505993	0.996489
dermatologic	Cellulitis and abscess of face/neck	Inverse variance weighted	2	-0.01398	0.387911	0.971261	0.986122	0.461035	2.109245	0.996489
endocrine/metabolic	Gout and other crystal arthropathies	Inverse variance weighted	2	0.004759	0.143408	0.973525	1.004771	0.75857	1.330878	0.996489
injuries & poisonings	Injuries to the nervous system	Inverse variance weighted	2	0.008329	0.251509	0.973581	1.008364	0.615927	1.650843	0.996489
digestive	Noninfectious gastroenteritis	Inverse variance weighted	2	0.00582	0.197618	0.976503	1.005837	0.682829	1.481642	0.996952
genitourinary	Other disorders of male genital organs	Inverse variance weighted	2	0.004826	0.167196	0.976973	1.004838	0.724062	1.394491	0.996952
injuries & poisonings	Contusion	Inverse variance weighted	2	0.011523	0.418893	0.978054	1.01159	0.445077	2.299182	0.996952
genitourinary	Genital prolapse	Inverse variance weighted	2	-0.00354	0.135309	0.979127	0.996466	0.764337	1.299092	0.996952
congenital anomalies	Digestive congenital anomalies	Inverse variance weighted	2	-0.00705	0.334391	0.983182	0.992976	0.515584	1.912399	0.997753
digestive	Flatulence	Inverse variance weighted	2	-0.00432	0.214768	0.983955	0.99569	0.653597	1.516835	0.997753
pregnancy complicatic	Complications of labor and delivery NEC	Inverse variance weighted	2	0.008628	0.451109	0.98474	1.008666	0.416635	2.441962	0.997753
circulatory system	Disease of tricuspid valve	Inverse variance weighted	2	0.00496	0.268974	0.985287	1.004972	0.593197	1.702587	0.997753
respiratory	Chronic tonsillitis and adenoiditis	Inverse variance weighted	2	0.004299	0.263027	0.986958	1.004309	0.599756	1.681744	0.997753
genitourinary	Chronic renal failure [CKD]	Inverse variance weighted	2	0.002377	0.173142	0.989048	1.002379	0.713921	1.407388	0.997753
digestive	Other biliary tract disease	Inverse variance weighted	2	0.0012	0.143408	0.993326	1.0012	0.755875	1.326149	0.997753
injuries & poisonings	Poisoning by psychotropic agents	Inverse variance weighted	2	0.001613	0.202875	0.993655	1.001615	0.672992	1.490704	0.997753
neoplasms	Myeloproliferative disease	Inverse variance weighted	2	0.003005	0.409941	0.994152	1.003009	0.449114	2.240028	0.997753
congenital anomalies	Genitourinary congenital anomalies	Inverse variance weighted	2	-0.00302	0.420098	0.994259	0.996982	0.437615	2.271339	0.997753
neurological	Inflammatory and toxic neuropathy	Inverse variance weighted	2	-0.00308	0.454228	0.99459	0.996925	0.409275	2.42834	0.997753
digestive	Cholelithiasis and cholecystitis	Inverse variance weighted	2	0.000402	0.072025	0.995544	1.000402	0.868692	1.152082	0.997753
digestive	Intestinal malabsorption (non-celiac)	Inverse variance weighted	2	0.000704	0.196929	0.997147	1.000704	0.680263	1.472092	0.997753
injuries & poisonings	Internal derangement of knee	Inverse variance weighted	2	-0.00036	0.129322	0.997753	0.999636	0.77582	1.28802	0.997753

Table S14. The phenome-wide MR analysis results of NEK3 (IVW or Wald ratio methods).

Category	Phenostring	Method	Nsnp	beta	se	P value	OR	LL	LR	FDR
musculoskeletal	Ganglion and cyst of synovium, tendon, and bursa	Wald ratio	1	-0.89101	0.243854	0.000258	0.410243	0.254371	0.661628	0.202281
musculoskeletal	Other disorders of synovium, tendon, and bursa	Wald ratio	1	-0.47833	0.159443	0.0027	0.619818	0.453464	0.847198	0.802334
neoplasms	Cancer of stomach	Wald ratio	1	1.688223	0.590878	0.004275	5.409858	1.699106	17.22469	0.802334
genitourinary	Cyst of kidney, acquired	Wald ratio	1	1.125482	0.393919	0.004275	3.081701	1.423902	6.669617	0.802334
symptoms	Malaise and fatigue	Wald ratio	1	0.628394	0.234475	0.007362	1.874598	1.18391	2.96823	0.802334
genitourinary	Nephritis; nephrosis; renal sclerosis	Wald ratio	1	0.937902	0.356403	0.008499	2.554615	1.270426	5.136904	0.802334
injuries & poisoni	Fracture of tibia and fibula	Wald ratio	1	0.787837	0.300128	0.008665	2.198636	1.220897	3.959387	0.802334
neoplasms	Skin cancer	Wald ratio	1	0.309508	0.121927	0.011134	1.362754	1.073077	1.730629	0.802334
infectious diseases	Bacterial enteritis	Wald ratio	1	0.66591	0.262612	0.011222	1.946261	1.163217	3.256427	0.802334
circulatory system	Cerebral ischemia	Wald ratio	1	0.647152	0.262612	0.013729	1.910093	1.141601	3.195912	0.802334
sense organs	Cataract	Wald ratio	1	-0.25323	0.103169	0.014106	0.776287	0.634166	0.950258	0.802334
genitourinary	Pyelonephritis	Wald ratio	1	0.937902	0.38454	0.014727	2.554615	1.202261	5.428155	0.802334
infectious diseases	Intestinal infection	Wald ratio	1	0.365782	0.150064	0.014789	1.44164	1.074285	1.934613	0.802334
infectious diseases	Intestinal infection due to C. difficile	Wald ratio	1	1.313062	0.543983	0.015787	3.71754	1.279994	10.797	0.802334
neurological	Other paralytic syndromes	Wald ratio	1	1.313062	0.543983	0.015787	3.71754	1.279994	10.797	0.802334
neoplasms	Benign neoplasm of other endocrine glands and related structures	Wald ratio	1	1.125482	0.468951	0.016395	3.081701	1.229169	7.726263	0.802334
injuries & poisoni	Fracture of lower limb	Wald ratio	1	0.393919	0.168822	0.019631	1.48278	1.065056	2.064339	0.904165
injuries & poisoni	Torus fracture	Wald ratio	1	0.778458	0.337645	0.021135	2.178112	1.123754	4.221716	0.919393
sense organs	Hearing loss	Wald ratio	1	0.487709	0.215717	0.023767	1.628581	1.067055	2.485602	0.978809
digestive	Femoral hernia	Wald ratio	1	1.219272	0.543983	0.025002	3.384723	1.165401	9.830389	0.978809
neoplasms	Other non-epithelial cancer of skin	Wald ratio	1	0.309508	0.140685	0.027807	1.362754	1.034341	1.795441	0.989673
circulatory system	Precordial pain	Wald ratio	1	-0.51585	0.234475	0.027807	0.596995	0.377035	0.94528	0.989673
circulatory system	Transient cerebral ischemia	Wald ratio	1	0.628394	0.300128	0.036282	1.874598	1.040959	3.375846	0.998644
genitourinary	Noninflammatory disorders of cervix	Wald ratio	1	0.543983	0.262612	0.038319	1.722855	1.029695	2.882631	0.998644
dermatologic	Degenerative skin conditions and other dermatoses	Wald ratio	1	0.38454	0.18758	0.040364	1.468938	1.017026	2.121656	0.998644
mental disorders	Swelling, mass, or lump in head and neck [Space-occupying lesion, intracranial NC	Wald ratio	1	0.937902	0.459572	0.041269	2.554615	1.037839	6.28812	0.998644
hematopoietic	Diseases of spleen	Wald ratio	1	1.219272	0.609636	0.0455	3.384723	1.024685	11.18036	0.998644
respiratory	Acute tonsillitis	Wald ratio	1	-1.12548	0.562741	0.0455	0.324496	0.107695	0.977742	0.998644
respiratory	Chronic sinusitis	Wald ratio	1	0.543983	0.271991	0.0455	1.722855	1.010939	2.936112	0.998644
injuries & poisoni	Poisoning/allergy of sulfonamides	Wald ratio	1	0.937902	0.468951	0.0455	2.554615	1.018935	6.404783	0.998644
neoplasms	Cancer of kidney and renal pelvis	Wald ratio	1	0.834732	0.431435	0.053017	2.304197	0.989179	5.367404	0.998644
neoplasms	Carcinoma in situ of skin	Wald ratio	1	1.031692	0.534604	0.053629	2.805808	0.983998	8.00059	0.998644
digestive	Symptoms involving digestive system	Wald ratio	1	-0.21572	0.112548	0.05528	0.805963	0.646416	1.004889	0.998644
sense organs	Disorders of external ear	Wald ratio	1	0.694047	0.365782	0.05777	2.001801	0.977375	4.099968	0.998644
infectious diseases	Sepsis	Wald ratio	1	0.497088	0.262612	0.058377	1.643927	0.982522	2.75057	0.998644
infectious diseases	Sepsis and SIRS	Wald ratio	1	0.497088	0.262612	0.058377	1.643927	0.982522	2.75057	0.998644
injuries & poisoni	Complication due to other implant and internal device	Wald ratio	1	0.459572	0.243854	0.059482	1.583396	0.981785	2.553657	0.998644
circulatory system	Varicose veins of lower extremity	Wald ratio	1	-0.24385	0.131306	0.063291	0.783602	0.605794	1.013597	0.998644
injuries & poisoni	Skull and face fracture and other intercranial injury	Wald ratio	1	-0.46895	0.253233	0.064047	0.625658	0.380873	1.027765	0.998644
neoplasms	Malignant neoplasm of kidney, except pelvis	Wald ratio	1	0.815974	0.440814	0.06416	2.261378	0.953114	5.365391	0.998644
circulatory system	Atherosclerosis of the extremities	Wald ratio	1	0.891006	0.487709	0.067711	2.437582	0.937159	6.340234	0.998644
respiratory	Chronic airway obstruction	Wald ratio	1	0.253233	0.140685	0.071861	1.288184	0.977742	1.697194	0.998644
circulatory system	Varicose veins	Wald ratio	1	-0.23448	0.131306	0.074146	0.790986	0.611503	1.023149	0.998644
genitourinary	Chronic glomerulonephritis, NOS	Wald ratio	1	0.844111	0.47833	0.077613	2.32591	0.910815	5.939576	0.998644
neurological	Other headache syndromes	Wald ratio	1	0.28137	0.159443	0.077613	1.324944	0.969341	1.811	0.998644
musculoskeletal	Spondylosis without myelopathy	Wald ratio	1	0.347024	0.196959	0.078085	1.41485	0.961735	2.081448	0.998644
dermatologic	Actinic keratosis	Wald ratio	1	0.47833	0.271991	0.078642	1.613377	0.9467	2.749538	0.998644
respiratory	Other diseases of respiratory system, NEC	Wald ratio	1	0.262612	0.150064	0.080118	1.300323	0.968978	1.744972	0.998644
symptoms	Other abnormal blood chemistry	Wald ratio	1	0.309508	0.178201	0.082415	1.362754	0.961013	1.932437	0.998644
endocrine/metabol	Vitamin B-complex deficiencies	Wald ratio	1	0.872248	0.506467	0.085029	2.392284	0.886542	6.455442	0.998644
neoplasms	Cancer of brain and nervous system	Wald ratio	1	-1.03169	0.600257	0.08566	0.356404	0.109899	1.155822	0.998644
genitourinary	Pain and other symptoms associated with female genital organs	Wald ratio	1	0.431435	0.253233	0.088436	1.539465	0.937159	2.528869	0.998644
infectious diseases	Viral Enteritis	Wald ratio	1	0.797216	0.468951	0.089131	2.219354	0.885213	5.564237	0.998644
neurological	Facial nerve disorders [CN7]	Wald ratio	1	0.797216	0.468951	0.089131	2.219354	0.885213	5.564237	0.998644
dermatologic	Scar conditions and fibrosis of skin	Wald ratio	1	0.47833	0.28137	0.089131	1.613377	0.929456	2.80055	0.998644
neoplasms	Cancer of larynx, pharynx, nasal cavities	Wald ratio	1	-0.9379	0.553362	0.090091	0.391448	0.132325	1.157992	0.998644
sense organs	Otitis externa	Wald ratio	1	1.031692	0.609636	0.090587	2.805808	0.849426	9.268099	0.998644
dermatologic	Cellulitis and abscess of face/neck	Wald ratio	1	1.031692	0.609636	0.090587	2.805808	0.849426	9.268099	0.998644
symptoms	Nonspecific findings on examination of blood	Wald ratio	1	0.300128	0.178201	0.092141	1.350032	0.952042	1.914398	0.998644
neoplasms	Acquired absence of breast	Wald ratio	1	-0.46895	0.28137	0.095581	0.625658	0.360437	1.086037	0.998644
genitourinary	Other disorders of bladder	Wald ratio	1	0.234475	0.140685	0.095581	1.264245	0.959572	1.665655	0.998644
hematopoietic	Lymphadenitis	Wald ratio	1	0.450193	0.271991	0.09789	1.568614	0.920434	2.673253	0.998644
genitourinary	Other disorders of the kidney and ureters	Wald ratio	1	0.403298	0.243854	0.098159	1.496752	0.928062	2.413921	0.998644
digestive	Other disorders of intestine	Wald ratio	1	0.356403	0.215717	0.098499	1.428182	0.935753	2.179747	0.998644
musculoskeletal	Pain in joint	Wald ratio	1	0.262612	0.159443	0.099546	1.300323	0.951328	1.777346	0.998644
musculoskeletal	Stiffness of joint	Wald ratio	1	0.909764	0.553362	0.100162	2.483738	0.839604	7.347454	0.998644
symptoms	Other symptoms	Wald ratio	1	0.647152	0.393919	0.100412	1.910093	0.88256	4.133947	0.998644
symptoms	Nausea and vomiting	Wald ratio	1	0.215717	0.131306	0.100412	1.240752	0.959212	1.604926	0.998644
symptoms	Cervicalgia	Wald ratio	1	0.534604	0.328266	0.103404	1.706772	0.896913	3.247886	0.998644
genitourinary	Excessive or frequent menstruation	Wald ratio	1	-0.24385	0.150064	0.104163	0.783602	0.583926	1.051557	0.998644
injuries & poisoni	Fracture of patella	Wald ratio	1	0.806595	0.497088	0.104666	2.240268	0.84561	5.935121	0.998644
digestive	Calculus of bile duct	Wald ratio	1	-0.44081	0.271991	0.105084	0.643513	0.377601	1.096682	0.998644
pregnancy complic	Complications of labor and delivery NEC	Wald ratio	1	0.468951	0.290749	0.106766	1.598316	0.904007	2.82588	0.998644
mental disorders	Anxiety disorders	Wald ratio	1	0.271991	0.168822	0.107156	1.312576	0.942801	1.82738	0.998644
infectious diseases	Septicemia	Wald ratio	1	0.347024	0.215717	0.107683	1.41485	0.927018	2.159398	0.998644
musculoskeletal	Rheumatoid arthritis	Wald ratio	1	-0.34702	0.215717	0.107683	0.706789	0.463092	1.078728	0.998644
sense organs	Suppurative and unspecified otitis media	Wald ratio	1	0.769079	0.47833	0.10787	2.157779	0.844976	5.510226	0.998644
circulatory system	Aneurysm and dissection of heart	Wald ratio	1	0.844111	0.525225	0.108023	2.32591	0.830831	6.511385	0.998644
injuries & poisoni	Complications of surgical and medical procedures	Wald ratio	1	0.225096	0.140685	0.109599	1.252443	0.950614	1.650106	0.998644
respiratory	Abnormal findings examination of lungs	Wald ratio	1	0.450193	0.28137	0.109599	1.568614	0.903668	2.722849	0.998644
infectious diseases	Candidiasis	Wald ratio	1	0.47833	0.300128	0.110992	1.613377	0.895904	2.905431	0.998644
respiratory	Pneumonitis due to inhalation of food or vomitus	Wald ratio	1	-0.90976	0.57212	0.111798	0.402619	0.131189	1.235642	0.998644
dermatologic	Carbuncle and furuncle	Wald ratio	1	-0.45957	0.290749	0.113959	0.631554	0.357207	1.11661	0.998644
respiratory	Pneumococcal pneumonia	Wald ratio	1	0.28137	0.178201	0.114348	1.324944	0.93435	1.878822	0.998644
musculoskeletal	Rheumatoid arthritis and other inflammatory polyarthropathies	Wald ratio	1	-0.30951	0.196959	0.116083	0.733808	0.498801	1.079538	0.998644
musculoskeletal	Other acquired musculoskeletal deformity	Wald ratio	1	-0.49709	0.318887	0.119038	0.6083	0.325594	1.136473	0.998644
respiratory	Pleurisy; pleural effusion	Wald ratio	1	0.262612	0.168822	0.119814	1.300323	0.934	1.810321	0.998644
digestive	Hemorrhage from gastrointestinal ulcer	Wald ratio	1	-0.87225	0.562741	0.121142	0.418011	0.138731	1.259511	0.998644
injuries & poisoni	Poisoning by other anti-infectives	Wald ratio	1	0.609636	0.393919	0.121714	1.839762	0.850063	3.981731	0.998644
digestive	Cholelithiasis with acute cholecystitis	Wald ratio	1	-0.54398	0.356403	0.126931	0.580432	0.288652	1.167152	0.998644
sense organs	Disorders of vitreous body	Wald ratio	1	-0.57212	0.375161	0.127259	0.564328	0.270513	1.177266	0.998644
genitourinary	Glomerulonephritis	Wald ratio	1	0.656531	0.431435	0.128074	1.928092	0.827719	4.491304	0.998644
sense organs	Ptosis of eyelid	Wald ratio	1	-0.49709	0.328266	0.129953	0.6083	0.319663	1.157558	0.998644
neurological	Other conditions of brain	Wald ratio	1	-0.55336	0.365782	0.130325	0.575013	0.280749	1.177708	0.998644

genitourinary	Infertility, female	Wald ratio	1	0.581499	0.38454	0.130484	1.788718	0.841812	3.800743	0.998644
dermatologic	Psoriasis vulgaris	Wald ratio	1	-0.50647	0.337645	0.133614	0.602621	0.310911	1.168028	0.998644
respiratory	Other diseases of lung	Wald ratio	1	-0.74094	0.497088	0.136075	0.476665	0.179922	1.262823	0.998644
mental disorders	Bipolar	Wald ratio	1	0.628394	0.422056	0.136517	1.874598	0.819685	4.287154	0.998644
mental disorders	Symptoms involving head and neck	Wald ratio	1	0.375161	0.253233	0.138478	1.455225	0.885877	2.390489	0.998644
sense organs	Senile cataract	Wald ratio	1	-0.23448	0.159443	0.141403	0.790986	0.578692	1.081159	0.998644
musculoskeletal	Other specified osteoporosis	Wald ratio	1	0.881627	0.600257	0.141901	2.414827	0.744624	7.83132	0.998644
infectious diseases	Infection/inflammation of internal prosthetic device; implant; and graft	Wald ratio	1	0.412677	0.28137	0.142467	1.510856	0.870394	2.622591	0.998644
genitourinary	Polyp of female genital organs	Wald ratio	1	0.206338	0.140685	0.142467	1.229169	0.932949	1.619442	0.998644
infectious diseases	E. coli	Wald ratio	1	0.38454	0.262612	0.143116	1.468938	0.877937	2.457784	0.998644
musculoskeletal	Synovitis and tenosynovitis	Wald ratio	1	-0.38454	0.262612	0.143116	0.680764	0.406871	1.139034	0.998644
neoplasms	Benign neoplasm of colon	Wald ratio	1	0.150064	0.103169	0.145795	1.161909	0.949189	1.422301	0.998644
endocrine/metabol	Diabetes mellitus	Wald ratio	1	0.150064	0.103169	0.145795	1.161909	0.949189	1.422301	0.998644
genitourinary	Ovarian cyst	Wald ratio	1	-0.30013	0.206338	0.145795	0.740723	0.49433	1.109927	0.998644
respiratory	Pulmonary collapse; interstitial and compensatory emphysema	Wald ratio	1	0.450193	0.309508	0.145795	1.568614	0.855181	2.877228	0.998644
dermatologic	Psoriasis and related disorders	Wald ratio	1	-0.42206	0.290749	0.146609	0.655698	0.370862	1.159296	0.998644
neurological	Degenerative disease of the spinal cord	Wald ratio	1	0.468951	0.328266	0.153127	1.598316	0.839919	3.041501	0.998644
digestive	Other disorders of stomach and duodenum	Wald ratio	1	0.347024	0.243854	0.154714	1.41485	0.877278	2.281831	0.998644
digestive	Ulcerative colitis	Wald ratio	1	0.347024	0.243854	0.154714	1.41485	0.877278	2.281831	0.998644
mental disorders	Anxiety disorder	Wald ratio	1	0.253233	0.178201	0.155301	1.288184	0.908427	1.826694	0.998644
sense organs	Vertiginous syndromes and other disorders of vestibular system	Wald ratio	1	0.253233	0.178201	0.155301	1.288184	0.908427	1.826694	0.998644
digestive	Cholelithiasis and cholecystitis	Wald ratio	1	-0.15944	0.112548	0.15658	0.852618	0.683836	1.06306	0.998644
neoplasms	Manlignant and unknown neoplasms of brain and nervous system	Wald ratio	1	-0.76908	0.543983	0.157423	0.46344	0.159568	1.345987	0.998644
neoplasms	Neoplasm of unspecified nature of digestive system	Wald ratio	1	-0.59088	0.422056	0.161513	0.553841	0.242172	1.266619	0.998644
genitourinary	stress incontinence, female	Wald ratio	1	0.262612	0.18758	0.161513	1.300323	0.900284	1.878117	0.998644
endocrine/metabol	Hyperpotassemia	Wald ratio	1	-0.62839	0.450193	0.162765	0.533448	0.22074	1.289151	0.998644
circulatory system	Occlusion and stenosis of precerebral arteries	Wald ratio	1	0.562741	0.403298	0.162911	1.755478	0.796345	3.869806	0.998644
symptoms	Neuralgia, neuritis, and radiculitis NOS	Wald ratio	1	0.562741	0.403298	0.162911	1.755478	0.796345	3.869806	0.998644
respiratory	Other upper respiratory disease	Wald ratio	1	-0.30013	0.215717	0.164133	0.740723	0.485326	1.13052	0.998644
endocrine/metabol	Type 1 diabetes	Wald ratio	1	0.375161	0.271991	0.167799	1.455225	0.853899	2.480013	0.998644
circulatory system	Intracranial hemorrhage	Wald ratio	1	0.450193	0.328266	0.170241	1.568614	0.824311	2.98498	0.998644
circulatory system	Congestive heart failure (CHF) NOS	Wald ratio	1	-0.42206	0.309508	0.172682	0.655698	0.357475	1.202712	0.998644
dermatologic	Symptoms affecting skin	Wald ratio	1	0.253233	0.18758	0.177016	1.288184	0.89188	1.860585	0.998644
injuries & poisoni	Complication of internal orthopedic device	Wald ratio	1	0.328266	0.243854	0.178253	1.388558	0.860976	2.239427	0.998644
injuries & poisoni	Traumatic arthropathy	Wald ratio	1	0.806595	0.600257	0.179029	2.240268	0.690798	7.265223	0.998644
circulatory system	Congestive heart failure; nonhypertensive	Wald ratio	1	-0.26261	0.196959	0.182422	0.76904	0.52275	1.131368	0.998644
genitourinary	Calculus of ureter	Wald ratio	1	0.375161	0.28137	0.182422	1.455225	0.838345	2.526024	0.998644
genitourinary	Irregular menstrual cycle	Wald ratio	1	-0.41268	0.309508	0.182422	0.661876	0.360843	1.214045	0.998644
pregnancy complic	Other complications of pregnancy NEC	Wald ratio	1	-0.41268	0.309508	0.182422	0.661876	0.360843	1.214045	0.998644
genitourinary	Other symptoms/disorders or the urinary system	Wald ratio	1	0.121927	0.091914	0.184664	1.129672	0.943438	1.352669	0.998644
circulatory system	Atherosclerosis	Wald ratio	1	0.506467	0.38454	0.187814	1.659418	0.78096	3.526001	0.998644
circulatory system	Cardiomyopathy	Wald ratio	1	-0.51585	0.393919	0.190357	0.596995	0.275842	1.292056	0.998644
infectious diseases	Bacterial infection NOS	Wald ratio	1	0.159443	0.121927	0.190978	1.172858	0.923547	1.48947	0.998644
digestive	Appendiceal conditions	Wald ratio	1	-0.31889	0.243854	0.190978	0.726958	0.450751	1.172418	0.998644
injuries & poisoni	Fracture of humerus	Wald ratio	1	0.440814	0.337645	0.191704	1.553971	0.801741	3.011978	0.998644
hematopoietic	Other diseases of blood and blood-forming organs	Wald ratio	1	0.28137	0.215717	0.192115	1.324944	0.868111	2.022181	0.998644
mental disorders	Depression	Wald ratio	1	-0.16882	0.131306	0.198543	0.844659	0.652997	1.092576	0.998644
musculoskeletal	Degeneration of intervertebral disc	Wald ratio	1	-0.33764	0.262612	0.198543	0.713449	0.426405	1.193722	0.998644
circulatory system	Tachycardia NOS	Wald ratio	1	0.38454	0.300128	0.200106	1.468938	0.815697	2.645319	0.998644
musculoskeletal	Spinal stenosis	Wald ratio	1	-0.30013	0.234475	0.200545	0.740723	0.467807	1.172858	0.998644
genitourinary	Renal failure	Wald ratio	1	0.215717	0.168822	0.201328	1.240752	0.891211	1.727386	0.998644
genitourinary	Retention of urine	Wald ratio	1	0.215717	0.168822	0.201328	1.240752	0.891211	1.727386	0.998644
musculoskeletal	Polymyalgia Rheumatica	Wald ratio	1	0.525225	0.412677	0.203115	1.690839	0.753052	3.796468	0.998644
genitourinary	Prolapse of vaginal vault after hysterectomy	Wald ratio	1	0.769079	0.609636	0.207115	2.157779	0.653242	7.127538	0.998644
circulatory system	Arterial embolism and thrombosis of lower extremity artery	Wald ratio	1	0.740942	0.590878	0.209854	2.097911	0.658903	6.679633	0.998644
injuries & poisoni	Injury, NOS	Wald ratio	1	0.18758	0.150064	0.2113	1.206327	0.898934	1.618834	0.998644
circulatory system	Cerebrovascular disease	Wald ratio	1	0.18758	0.150064	0.2113	1.206327	0.898934	1.618834	0.998644
genitourinary	Hematuria	Wald ratio	1	-0.14069	0.112548	0.2113	0.868763	0.696784	1.083189	0.998644
circulatory system	Aortic valve disease	Wald ratio	1	0.487709	0.393919	0.215681	1.628581	0.752487	3.524679	0.998644
circulatory system	Rheumatic disease of the heart valves	Wald ratio	1	0.243854	0.196959	0.215681	1.276159	0.86746	1.877413	0.998644
digestive	Stricture and stenosis of esophagus	Wald ratio	1	0.393919	0.318887	0.216721	1.48278	0.793661	2.770246	0.998644
genitourinary	Vaginal enterocele, congenital or acquired	Wald ratio	1	0.656531	0.534604	0.219421	1.928092	0.676182	5.497837	0.998644
sense organs	Retinal detachment with retinal defect	Wald ratio	1	-0.45957	0.375161	0.220575	0.631554	0.302738	1.317509	0.998644
genitourinary	Stricture/obstruction of ureter	Wald ratio	1	0.562741	0.459572	0.220768	1.755478	0.713181	4.321064	0.998644
respiratory	Bacterial pneumonia	Wald ratio	1	0.206338	0.168822	0.221624	1.229169	0.882891	1.71126	0.998644
digestive	Duodenal ulcer	Wald ratio	1	-0.30951	0.253233	0.221624	0.733808	0.44671	1.205422	0.998644
endocrine/metabol	Disorders of mineral metabolism	Wald ratio	1	-0.36578	0.300128	0.222939	0.693654	0.385184	1.249159	0.998644
genitourinary	Other disorders of urethra and urinary tract	Wald ratio	1	0.262612	0.215717	0.223455	1.300323	0.851979	1.984602	0.998644
mental disorders	Delirium dementia and amnestic and other cognitive disorders	Wald ratio	1	-0.37516	0.309508	0.225466	0.687179	0.374638	1.260457	0.998644
musculoskeletal	Osteoporosis	Wald ratio	1	0.215717	0.178201	0.226077	1.240752	0.874977	1.759434	0.998644
genitourinary	Irregular menstrual bleeding	Wald ratio	1	0.271991	0.225096	0.226919	1.312576	0.844342	2.04047	0.998644
respiratory	Other diseases of respiratory system, not elsewhere classified	Wald ratio	1	0.168822	0.140685	0.230139	1.18391	0.898597	1.559812	0.998644
digestive	Constipation	Wald ratio	1	-0.16882	0.140685	0.230139	0.844659	0.641103	1.112846	0.998644
sense organs	Infection of the eye	Wald ratio	1	0.694047	0.581499	0.232655	2.001801	0.640382	6.257527	0.998644
genitourinary	Calculus of kidney	Wald ratio	1	-0.29075	0.243854	0.233141	0.747703	0.463613	1.205875	0.998644
dermatologic	Dyschromia and Vitiligo	Wald ratio	1	0.525225	0.440814	0.233462	1.690839	0.712646	4.011719	0.998644
injuries & poisoni	Contusion	Wald ratio	1	0.422056	0.356403	0.23633	1.525093	0.758439	3.066708	0.998644
musculoskeletal	Curvature of spine	Wald ratio	1	0.487709	0.412677	0.237278	1.628581	0.725324	3.656678	0.998644
musculoskeletal	Other disorders of cervical region	Wald ratio	1	0.712805	0.609636	0.242311	2.039705	0.617497	6.737519	0.998644
circulatory system	Peripheral vascular disease	Wald ratio	1	0.262612	0.225096	0.243345	1.300323	0.83646	2.021422	0.998644
injuries & poisoni	Internal derangement of knee	Wald ratio	1	-0.13131	0.112548	0.243345	0.876949	0.70335	1.093396	0.998644
neurological	Hemiplegia	Wald ratio	1	-0.41268	0.356403	0.246907	0.661876	0.329155	1.330923	0.998644
genitourinary	Symptoms involving female genital tract	Wald ratio	1	0.206338	0.178201	0.246907	1.229169	0.866809	1.743009	0.998644
dermatologic	Disorder of skin and subcutaneous tissue NOS	Wald ratio	1	0.206338	0.178201	0.246907	1.229169	0.866809	1.743009	0.998644
musculoskeletal	Acquired spondylolisthesis	Wald ratio	1	-0.41268	0.356403	0.246907	0.661876	0.329155	1.330923	0.998644
digestive	Cholelithiasis	Wald ratio	1	-0.14069	0.121927	0.248563	0.868763	0.684092	1.103285	0.998644
dermatologic	Seborrheic keratosis	Wald ratio	1	0.290749	0.253233	0.250907	1.337429	0.814168	2.196987	0.998644
endocrine/metabol	Hypovolemia	Wald ratio	1	0.300128	0.262612	0.253098	1.350032	0.806871	2.258834	0.998644
digestive	Inflammatory bowel disease and other gastroenteritis and colitis	Wald ratio	1	0.234475	0.206338	0.255804	1.264245	0.843709	1.894393	0.998644
mental disorders	Aphasia/speech disturbance	Wald ratio	1	0.403298	0.356403	0.257811	1.496752	0.744344	3.009719	0.998644
dermatologic	Psoriasis	Wald ratio	1	-0.32827	0.290749	0.258884	0.720172	0.407329	1.273289	0.998644
genitourinary	Noninflammatory disorders of ovary, fallopian tube, and broad ligament	Wald ratio	1	0.581499	0.515846	0.259627	1.788718	0.650796	4.916303	0.998644
genitourinary	Mucous polyp of cervix	Wald ratio	1	0.262612	0.234475	0.262714	1.300323	0.821224	2.058925	0.998644
musculoskeletal	Bursitis	Wald ratio	1	-0.5346	0.47833	0.263718	0.585901	0.229436	1.496191	0.998644
endocrine/metabol	Disorders of calcium/phosphorus metabolism	Wald ratio	1	-0.45019	0.403298	0.264303	0.637505	0.289194	1.405328	0.998644
neoplasms	Lymphoid leukemia	Wald ratio	1	0.637773	0.57212	0.264956	1.892262	0.616571	5.807372	0.998644
sense organs	Otitis media	Wald ratio	1	0.365782	0.328266	0.265157	1.44164	0.757586	2.743356	0.998644

infectious diseases	Viral infection	Wald ratio	1	0.290749	0.262612	0.268232	1.337429	0.799338	2.237748	0.998644
digestive	Gastrointestinal hemorrhage	Wald ratio	1	-0.10317	0.09379	0.271332	0.901974	0.750513	1.084002	0.998644
circulatory system	Coronary atherosclerosis	Wald ratio	1	0.112548	0.103169	0.275313	1.119126	0.914239	1.36993	0.998644
injuries & poisoni	Poisoning by anticonvulsants and anti-Parkinsonism drugs	Wald ratio	1	-0.67529	0.619015	0.275313	0.509009	0.15129	1.712545	0.998644
genitourinary	Other nonmalignant breast conditions	Wald ratio	1	-0.34702	0.318887	0.276491	0.706789	0.37831	1.320478	0.998644
endocrine/metabol	Type 2 diabetes	Wald ratio	1	0.121927	0.112548	0.279087	1.129672	0.906044	1.408495	0.998644
circulatory system	Hypotension NOS	Wald ratio	1	0.253233	0.234475	0.280142	1.288184	0.813558	2.039705	0.998644
symptoms	Pain	Wald ratio	1	0.534604	0.497088	0.282164	1.706772	0.644237	4.521736	0.998644
respiratory	Pneumonia	Wald ratio	1	0.150064	0.140685	0.286122	1.161909	0.881898	1.530826	0.998644
pregnancy complic	Hemorrhage in early pregnancy	Wald ratio	1	0.459572	0.431435	0.286778	1.583396	0.679743	3.688367	0.998644
dermatologic	Decubitus ulcer	Wald ratio	1	0.487709	0.459572	0.288588	1.628581	0.661628	4.00871	0.998644
digestive	Other specified gastritis	Wald ratio	1	0.168822	0.159443	0.28968	1.18391	0.866159	1.618227	0.998644
circulatory system	Intracerebral hemorrhage	Wald ratio	1	0.553362	0.525225	0.292079	1.73909	0.621215	4.868582	0.998644
circulatory system	Unstable angina (intermediate coronary syndrome)	Wald ratio	1	0.206338	0.196959	0.294814	1.229169	0.835519	1.808285	0.998644
circulatory system	Primary/intrinsic cardiomyopathies	Wald ratio	1	-0.42206	0.403298	0.295325	0.655698	0.297447	1.445431	0.998644
dermatologic	Sicca syndrome	Wald ratio	1	-0.63777	0.609636	0.29549	0.528468	0.159987	1.745626	0.998644
sense organs	Dizziness and giddiness (Light-headedness and vertigo)	Wald ratio	1	0.215717	0.206338	0.295813	1.240752	0.82803	1.859189	0.998644
circulatory system	Palpitations	Wald ratio	1	0.234475	0.225096	0.297566	1.264245	0.813253	1.965338	0.998644
digestive	Diseases of the jaws	Wald ratio	1	-0.46895	0.450193	0.297566	0.625658	0.258896	1.511991	0.998644
genitourinary	Endometriosis	Wald ratio	1	0.234475	0.225096	0.297566	1.264245	0.813253	1.965338	0.998644
musculoskeletal	Osteopenia or other disorder of bone and cartilage	Wald ratio	1	-0.50647	0.487709	0.299055	0.602621	0.231685	1.567438	0.998644
neoplasms	Melanomas of skin, dx or hx	Wald ratio	1	0.28137	0.271991	0.300911	1.324944	0.777452	2.257987	0.998644
neoplasms	Melanomas of skin	Wald ratio	1	0.28137	0.271991	0.300911	1.324944	0.777452	2.257987	0.998644
neoplasms	Multiple myeloma	Wald ratio	1	0.609636	0.590878	0.302191	1.839762	0.577825	5.857699	0.998644
musculoskeletal	Displacement of intervertebral disc	Wald ratio	1	0.628394	0.609636	0.302649	1.874598	0.567512	6.192139	0.998644
infectious diseases	Viral hepatitis	Wald ratio	1	0.412677	0.403298	0.306187	1.510856	0.685377	3.330559	0.998644
circulatory system	Disease of tricuspid valve	Wald ratio	1	0.431435	0.422056	0.306676	1.539465	0.673145	3.520714	0.998644
mental disorders	Dementias	Wald ratio	1	-0.45957	0.450193	0.307333	0.631554	0.261336	1.526238	0.998644
sense organs	Disorders of conjunctiva	Wald ratio	1	-0.50647	0.497088	0.308266	0.602621	0.227465	1.596518	0.998644
congenital anomal	Digestive congenital anomalies	Wald ratio	1	0.534604	0.525225	0.308746	1.706772	0.60967	4.778108	0.998644
neoplasms	Cancer, suspected or other	Wald ratio	1	-0.11255	0.112548	0.317311	0.893554	0.716668	1.114099	0.998644
neoplasms	Leukemia	Wald ratio	1	0.337645	0.337645	0.317311	1.401642	0.72315	2.716727	0.998644
respiratory	Acute and chronic tonsillitis	Wald ratio	1	-0.30951	0.309508	0.317311	0.733808	0.400059	1.345987	0.998644
respiratory	Respiratory failure, insufficiency, arrest	Wald ratio	1	0.271991	0.271991	0.317311	1.312576	0.770195	2.236908	0.998644
digestive	Personal history of diseases of digestive system	Wald ratio	1	-0.11255	0.112548	0.317311	0.893554	0.716668	1.114099	0.998644
digestive	Anal and rectal conditions	Wald ratio	1	0.112548	0.112548	0.317311	1.119126	0.897586	1.395346	0.998644
digestive	Obstruction of bile duct	Wald ratio	1	0.497088	0.497088	0.317311	1.643927	0.620516	4.355241	0.998644
genitourinary	Cystitis	Wald ratio	1	-0.25323	0.253233	0.317311	0.776287	0.472569	1.275201	0.998644
musculoskeletal	Kyphoscoliosis and scoliosis	Wald ratio	1	0.422056	0.422056	0.317311	1.525093	0.666861	3.487848	0.998644
musculoskeletal	Osteoporosis NOS	Wald ratio	1	0.18758	0.18758	0.317311	1.206327	0.835206	1.742355	0.998644
symptoms	Myalgia and myositis unspecified	Wald ratio	1	0.543983	0.543983	0.317311	1.722855	0.5932	5.003759	0.998644
injuries & poisoni	Fracture of unspecified bones	Wald ratio	1	0.38454	0.38454	0.317311	1.468938	0.691316	3.121262	0.998644
pregnancy complic	Late pregnancy and failed induction	Wald ratio	1	0.393919	0.403298	0.328696	1.48278	0.67264	3.268666	0.998644
digestive	Gingival and periodontal diseases	Wald ratio	1	0.318887	0.328266	0.331335	1.375595	0.722879	2.617676	0.998644
dermatologic	Erythematous conditions	Wald ratio	1	0.271991	0.28137	0.333711	1.312576	0.756166	2.278409	0.998644
neoplasms	Cancer within the respiratory system	Wald ratio	1	-0.26261	0.271991	0.334286	0.76904	0.451258	1.310608	0.998644
digestive	Acute appendicitis	Wald ratio	1	-0.26261	0.271991	0.334286	0.76904	0.451258	1.310608	0.998644
digestive	Blood in stool	Wald ratio	1	-0.26261	0.271991	0.334286	0.76904	0.451258	1.310608	0.998644
digestive	Acute periodontitis	Wald ratio	1	-0.51585	0.534604	0.334589	0.596995	0.209366	1.702296	0.998644
digestive	Diseases of the salivary glands	Wald ratio	1	-0.49709	0.515846	0.335228	0.6083	0.22132	1.671916	0.998644
digestive	Appendicitis	Wald ratio	1	-0.23448	0.243854	0.336281	0.790986	0.490451	1.27568	0.998644
musculoskeletal	Contracture of palmar fascia [Dupuytren's disease]	Wald ratio	1	-0.2251	0.234475	0.337055	0.798439	0.504258	1.264245	0.998644
circulatory system	Abnormal heart sounds	Wald ratio	1	0.412677	0.431435	0.338809	1.510856	0.648602	3.519394	0.998644
genitourinary	Acute renal failure	Wald ratio	1	0.196959	0.206338	0.339808	1.217695	0.812643	1.82464	0.998644
endocrine/metabol	Vitamin deficiency	Wald ratio	1	0.38454	0.403298	0.340343	1.468938	0.666361	3.238153	0.998644
genitourinary	Dysuria	Wald ratio	1	0.38454	0.403298	0.340343	1.468938	0.666361	3.238153	0.998644
mental disorders	Delirium due to conditions classified elsewhere	Wald ratio	1	-0.51585	0.543983	0.342989	0.596995	0.205553	1.733878	0.998644
neoplasms	Hemangioma and lymphangioma, any site	Wald ratio	1	-0.32827	0.347024	0.344176	0.720172	0.364791	1.421767	0.998644
genitourinary	Cyst or abscess of Bartholin's gland	Wald ratio	1	0.47833	0.506467	0.344943	1.613377	0.597892	4.353607	0.998644
genitourinary	Polyp of corpus uteri	Wald ratio	1	0.150064	0.159443	0.346614	1.161909	0.850063	1.588155	0.998644
digestive	Diaphragmatic hernia	Wald ratio	1	0.084411	0.090039	0.348501	1.088076	0.912046	1.298081	0.998644
circulatory system	Cardiac pacemaker in situ	Wald ratio	1	-0.27199	0.290749	0.349539	0.761861	0.430908	1.346997	0.998644
endocrine/metabol	Hypercholesterolemia	Wald ratio	1	0.078784	0.084411	0.350648	1.08197	0.916987	1.276637	0.998644
endocrine/metabol	Obesity	Wald ratio	1	0.131306	0.140685	0.350648	1.140317	0.86551	1.502378	0.998644
sense organs	Disorders of refraction and accommodation; blindness and low vision	Wald ratio	1	-0.26261	0.28137	0.350648	0.76904	0.443039	1.334923	0.998644
mental disorders	Mood disorders	Wald ratio	1	-0.12193	0.131306	0.353111	0.885213	0.684349	1.145032	0.998644
digestive	Other intestinal obstruction	Wald ratio	1	0.225096	0.243854	0.355967	1.252443	0.776578	2.019906	0.998644
hematopoietic	Diseases of white blood cells	Wald ratio	1	0.206338	0.225096	0.359317	1.229169	0.790689	1.91081	0.998644
digestive	Diseases of the oral soft tissues, excluding lesions specific for gingiva and tongue	Wald ratio	1	0.206338	0.225096	0.359317	1.229169	0.790689	1.91081	0.998644
musculoskeletal	Unspecified monoarthritis	Wald ratio	1	-0.10317	0.112548	0.359317	0.901974	0.723421	1.124598	0.998644
symptoms	Symptoms involving skin and other integumentary tissue	Wald ratio	1	-0.30013	0.328266	0.360567	0.740723	0.389252	1.409552	0.998644
injuries & poisoni	Complications of cardiac/vascular device, implant, and graft	Wald ratio	1	0.300128	0.328266	0.360567	1.350032	0.709445	2.569031	0.998644
endocrine/metabol	Electrolyte imbalance	Wald ratio	1	-0.19696	0.215717	0.36122	0.821224	0.538071	1.253383	0.998644
digestive	Ulceration of intestine	Wald ratio	1	-0.48771	0.534604	0.361621	0.614032	0.215341	1.750873	0.998644
endocrine/metabol	Hyperlipidemia	Wald ratio	1	0.074094	0.081597	0.363854	1.076908	0.917744	1.263676	0.998644
genitourinary	Calculus of lower urinary tract	Wald ratio	1	0.450193	0.497088	0.365116	1.568614	0.592088	4.155717	0.998644
sense organs	Otitis media and Eustachian tube disorders	Wald ratio	1	0.262612	0.290749	0.366406	1.300323	0.735462	2.299016	0.998644
musculoskeletal	Other disorders of soft tissues	Wald ratio	1	0.159443	0.178201	0.370928	1.172858	0.827099	1.663157	0.998644
injuries & poisoni	Fracture of pelvis	Wald ratio	1	0.459572	0.515846	0.372978	1.583396	0.576093	4.351974	0.998644
digestive	Peptic ulcer (excl. esophageal)	Wald ratio	1	-0.15006	0.168822	0.374063	0.860653	0.618192	1.198208	0.998644
genitourinary	Cystitis and urethritis	Wald ratio	1	-0.2251	0.253233	0.374063	0.798439	0.486055	1.311591	0.998644
musculoskeletal	Spondylosis and allied disorders	Wald ratio	1	0.140685	0.159443	0.377586	1.151062	0.842128	1.573329	0.998644
musculoskeletal	Osteoporosis, osteopenia and pathological fracture	Wald ratio	1	0.140685	0.159443	0.377586	1.151062	0.842128	1.573329	0.998644
genitourinary	Hypertrophy of breast (Gynecomastia)	Wald ratio	1	0.422056	0.47833	0.377586	1.525093	0.597219	3.894565	0.998644
endocrine/metabol	Disorders of adrenal glands	Wald ratio	1	0.47833	0.543983	0.379233	1.613377	0.555506	4.685798	0.998644
respiratory	Respiratory failure	Wald ratio	1	0.271991	0.309508	0.379516	1.312576	0.715593	2.40759	0.998644
digestive	Jaundice (not of newborn)	Wald ratio	1	-0.4033	0.459572	0.380187	0.668113	0.271428	1.644544	0.998644
circulatory system	Paroxysmal ventricular tachycardia	Wald ratio	1	-0.39392	0.450193	0.381574	0.674409	0.279069	1.629803	0.998644
injuries & poisoni	Open wounds of extremities	Wald ratio	1	-0.18758	0.215717	0.384538	0.828963	0.543141	1.265194	0.998644
genitourinary	Noninflammatory female genital disorders	Wald ratio	1	0.121927	0.140685	0.386125	1.129672	0.85743	1.488353	0.998644
circulatory system	Subarachnoid hemorrhage	Wald ratio	1	0.422056	0.487709	0.386828	1.525093	0.586341	3.966821	0.998644
neoplasms	Lipoma of skin and subcutaneous tissue	Wald ratio	1	-0.1782	0.206338	0.387788	0.836774	0.558431	1.253854	0.998644
neurological	Other peripheral nerve disorders	Wald ratio	1	0.112548	0.131306	0.391366	1.119126	0.865185	1.447602	0.998644
injuries & poisoni	Fracture of neck of femur	Wald ratio	1	0.28137	0.328266	0.391366	1.324944	0.696261	2.521291	0.998644
endocrine/metabol	Hypoglycemia	Wald ratio	1	0.38454	0.450193	0.393013	1.468938	0.607843	3.549893	0.998644
genitourinary	Breast conditions, congenital or relating to hormones	Wald ratio	1	0.38454	0.450193	0.393013	1.468938	0.607843	3.549893	0.998644
neoplasms	Pancreatic cancer	Wald ratio	1	0.487709	0.57212	0.393959	1.628581	0.530653	4.99813	0.998644

musculoskeletal	Rupture of synovium	Wald ratio	1	-0.48771	0.57212	0.393959	0.614032	0.200075	1.884469	0.998644
neurological	Disorders of other cranial nerves	Wald ratio	1	0.318887	0.375161	0.395325	1.375595	0.659398	2.869682	0.998644
symptoms	Musculoskeletal symptoms referable to limbs	Wald ratio	1	0.159443	0.18758	0.395325	1.172858	0.812033	1.694014	0.998644
dermatologic	Prurigo and Lichen	Wald ratio	1	-0.42206	0.497088	0.39585	0.655698	0.247499	1.737134	0.998644
pregnancy complic	Fetal distress and abnormal forces of labor	Wald ratio	1	0.262612	0.309508	0.396168	1.300323	0.708913	2.385115	0.998644
dermatologic	Chronic ulcer of skin	Wald ratio	1	0.262612	0.309508	0.396168	1.300323	0.708913	2.385115	0.998644
digestive	Inguinal hernia	Wald ratio	1	-0.10317	0.121927	0.397467	0.901974	0.710244	1.145462	0.998644
musculoskeletal	Peripheral enthesopathies and allied syndromes	Wald ratio	1	-0.10317	0.121927	0.397467	0.901974	0.710244	1.145462	0.998644
genitourinary	Postmenopausal atrophic vaginitis	Wald ratio	1	0.347024	0.412677	0.400399	1.41485	0.630134	3.176786	0.998644
genitourinary	Abnormal findings on examination of urine	Wald ratio	1	0.196959	0.234475	0.400908	1.217695	0.76904	1.928092	0.998644
digestive	Malposition and malpresentation of fetus or obstruction	Wald ratio	1	-0.29075	0.347024	0.402122	0.747703	0.378736	1.47612	0.998644
congenital anomal	Genitourinary congenital anomalies	Wald ratio	1	0.290749	0.347024	0.402122	1.337429	0.677452	2.640361	0.998644
sense organs	Cholesteatoma	Wald ratio	1	0.468951	0.562741	0.404657	1.598316	0.530454	4.8159	0.998644
respiratory	Respiratory abnormalities	Wald ratio	1	-0.46895	0.562741	0.404657	0.625658	0.207646	1.885176	0.998644
symptoms	Edema	Wald ratio	1	-0.28137	0.337645	0.404657	0.754749	0.389398	1.462888	0.998644
circulatory system	Other disorders of circulatory system	Wald ratio	1	0.09379	0.112548	0.404657	1.098329	0.880906	1.369416	0.998644
injuries & poisoni	Fracture of foot	Wald ratio	1	0.271991	0.328266	0.407347	1.312576	0.689762	2.497754	0.998644
congenital anomal	Cardiac congenital anomalies	Wald ratio	1	0.225096	0.271991	0.407905	1.252443	0.73491	2.13443	0.998644
hematopoietic	Disorders of iron metabolism	Wald ratio	1	-0.44081	0.534604	0.409621	0.643513	0.22568	1.834937	0.998644
digestive	Hematemesis	Wald ratio	1	-0.26261	0.318887	0.410207	0.76904	0.41163	1.436781	0.998644
dermatologic	Superficial cellulitis and abscess	Wald ratio	1	0.131306	0.159443	0.410207	1.140317	0.834266	1.558642	0.998644
endocrine/metabol	Abnormal glucose	Wald ratio	1	0.431435	0.525225	0.411402	1.539465	0.549907	4.309731	0.998644
endocrine/metabol	Disorders of lipoid metabolism	Wald ratio	1	0.066591	0.081597	0.414448	1.068858	0.910884	1.25423	0.998644
neoplasms	Benign neoplasm of ovary	Wald ratio	1	-0.29075	0.356403	0.414621	0.747703	0.371837	1.503506	0.998644
dermatologic	Other hypertrophic and atrophic conditions of skin	Wald ratio	1	0.159443	0.196959	0.418214	1.172858	0.797242	1.725442	0.998644
injuries & poisoni	Crushing or internal injury to organs	Wald ratio	1	0.356403	0.440814	0.418797	1.428182	0.601943	3.388534	0.998644
neoplasms	Malignant neoplasm, other	Wald ratio	1	-0.09098	0.112548	0.418899	0.913039	0.732296	1.138393	0.998644
sense organs	Retinal detachments and defects	Wald ratio	1	-0.19696	0.243854	0.419268	0.821224	0.5092	1.324447	0.998644
genitourinary	Urethral stricture (not specified as infectious)	Wald ratio	1	0.196959	0.243854	0.419268	1.217695	0.755032	1.963864	0.998644
injuries & poisoni	Fracture of ankle and foot	Wald ratio	1	0.234475	0.290749	0.419982	1.264245	0.715057	2.235231	0.998644
digestive	Nonspecific abnormal findings in stool contents	Wald ratio	1	-0.27199	0.337645	0.420499	0.761861	0.393067	1.476673	0.998644
genitourinary	Cervicitis and endocervicitis	Wald ratio	1	-0.30951	0.38454	0.42089	0.733808	0.345347	1.559227	0.998644
injuries & poisoni	Burns	Wald ratio	1	-0.45957	0.57212	0.421814	0.631554	0.205784	1.938246	0.998644
neoplasms	Lymphoid leukemia, chronic	Wald ratio	1	0.497088	0.619015	0.421957	1.643927	0.488614	5.530937	0.998644
neoplasms	Benign neoplasm of unspecified sites	Wald ratio	1	0.225096	0.28137	0.423711	1.252443	0.721524	2.17403	0.998644
circulatory system	Circulatory disease NEC	Wald ratio	1	0.090039	0.112548	0.423711	1.094216	0.877607	1.364288	0.998644
musculoskeletal	Symptoms and disorders of the joints	Wald ratio	1	0.18758	0.234475	0.423711	1.206327	0.761861	1.910093	0.998644
congenital anomal	Congenital anomalies of great vessels	Wald ratio	1	0.262612	0.328266	0.423711	1.300323	0.683323	2.474437	0.998644
circulatory system	Nonspecific chest pain	Wald ratio	1	-0.06659	0.083473	0.425014	0.935578	0.794376	1.101879	0.998644
symptoms	Fever of unknown origin	Wald ratio	1	0.178201	0.225096	0.428555	1.195066	0.768751	1.857795	0.998644
neoplasms	Breast cancer [female]	Wald ratio	1	-0.10317	0.131306	0.432035	0.901974	0.697307	1.166714	0.998644
sense organs	Myopia	Wald ratio	1	-0.30951	0.393919	0.432035	0.733808	0.339057	1.588155	0.998644
injuries & poisoni	Other open wound of head and face	Wald ratio	1	0.206338	0.262612	0.432035	1.229169	0.734635	2.056609	0.998644
sense organs	Tinnitus	Wald ratio	1	-0.47833	0.609636	0.432679	0.619818	0.187643	2.047372	0.998644
endocrine/metabol	Secondary hypothyroidism	Wald ratio	1	-0.31889	0.412677	0.439684	0.726958	0.323766	1.632251	0.998644
mental disorders	Phobia	Wald ratio	1	-0.47833	0.619015	0.439684	0.619818	0.184225	2.085356	0.998644
injuries & poisoni	Intracranial hemorrhage (injury)	Wald ratio	1	0.47833	0.619015	0.439684	1.613377	0.479534	5.428155	0.998644
digestive	GERD	Wald ratio	1	-0.09379	0.121927	0.441756	0.910474	0.716937	1.156256	0.998644
dermatologic	Disorders of sweat glands	Wald ratio	1	-0.37516	0.487709	0.441756	0.687179	0.264194	1.787376	0.998644
digestive	Anal and rectal polyp	Wald ratio	1	0.121927	0.159443	0.444447	1.129672	0.826478	1.544092	0.998644
genitourinary	Other abnormality of urination	Wald ratio	1	0.243854	0.318887	0.444447	1.276159	0.683066	2.38422	0.998644
circulatory system	Hypertensive chronic kidney disease	Wald ratio	1	-0.27199	0.356403	0.445369	0.761861	0.378878	1.531975	0.998644
dermatologic	Diseases of hair and hair follicles	Wald ratio	1	-0.15006	0.196959	0.446117	0.860653	0.585023	1.266144	0.998644
circulatory system	Peripheral vascular disease, unspecified	Wald ratio	1	0.206338	0.271991	0.448079	1.229169	0.721253	2.094765	0.998644
circulatory system	Arrhythmia (cardiac) NOS	Wald ratio	1	0.347024	0.459572	0.450188	1.41485	0.574798	3.482618	0.998644
sense organs	Meniere's disease	Wald ratio	1	0.431435	0.57212	0.45079	1.539465	0.501616	4.724633	0.998644
congenital anomal	Cardiac shunt/ heart septal defect	Wald ratio	1	0.431435	0.57212	0.45079	1.539465	0.501616	4.724633	0.998644
hematopoietic	Other deficiency anemia	Wald ratio	1	0.309508	0.412677	0.453255	1.362754	0.606932	3.059813	0.998644
neoplasms	Malignant neoplasm of testis	Wald ratio	1	0.168822	0.225096	0.453255	1.18391	0.761575	1.840452	0.998644
musculoskeletal	Fasciitis	Wald ratio	1	-0.16882	0.225096	0.453255	0.844659	0.543345	1.313068	0.998644
injuries & poisoni	Open wounds of head; neck; and trunk	Wald ratio	1	0.168822	0.225096	0.453255	1.18391	0.761575	1.840452	0.998644
neoplasms	Malignant neoplasm of other and ill-defined sites within the digestive organs and pe	Wald ratio	1	-0.14069	0.18758	0.453255	0.868763	0.601492	1.254795	0.998644
sense organs	Other disorders of eyelids	Wald ratio	1	-0.14069	0.18758	0.453255	0.868763	0.601492	1.254795	0.998644
neoplasms	Cancer of urinary organs (incl. kidney and bladder)	Wald ratio	1	0.159443	0.215717	0.459828	1.172858	0.768463	1.79006	0.998644
circulatory system	Heart failure NOS	Wald ratio	1	-0.15944	0.215717	0.459828	0.852618	0.55864	1.301299	0.998644
musculoskeletal	Hallux rigidus	Wald ratio	1	-0.26261	0.356403	0.461218	0.76904	0.382448	1.546411	0.998644
endocrine/metabol	Protein-calorie malnutrition	Wald ratio	1	0.309508	0.422056	0.463355	1.362754	0.595877	3.116581	0.998644
endocrine/metabol	Overweight, obesity and other hyperalimentation	Wald ratio	1	0.103169	0.140685	0.463355	1.108679	0.841496	1.460695	0.998644
neurological	Abnormality of gait	Wald ratio	1	0.253233	0.347024	0.465555	1.288184	0.652507	2.54314	0.998644
endocrine/metabol	Other disorders of pancreatic internal secretion	Wald ratio	1	0.328266	0.450193	0.4659	1.388558	0.574582	3.355643	0.998644
endocrine/metabol	Hypothyroidism	Wald ratio	1	-0.08816	0.121927	0.469633	0.915612	0.720983	1.162781	0.998644
digestive	Diseases of lips	Wald ratio	1	0.38454	0.534604	0.471957	1.468938	0.515157	4.188586	0.998644
dermatologic	Cellulitis and abscess of trunk	Wald ratio	1	0.403298	0.562741	0.47358	1.496752	0.496747	4.509877	0.998644
neoplasms	Breast cancer	Wald ratio	1	-0.09379	0.131306	0.475051	0.910474	0.703878	1.177708	0.998644
neoplasms	Non-Hodgkins lymphoma	Wald ratio	1	0.234475	0.328266	0.475051	1.264245	0.664364	2.405784	0.998644
sense organs	Primary angle-closure glaucoma	Wald ratio	1	-0.37516	0.525225	0.475051	0.687179	0.245465	1.923757	0.998644
digestive	Chronic liver disease and cirrhosis	Wald ratio	1	0.18758	0.262612	0.475051	1.206327	0.720983	2.018391	0.998644
genitourinary	Noninflammatory disorders of vagina	Wald ratio	1	-0.23448	0.328266	0.475051	0.790986	0.415665	1.505199	0.998644
neurological	Epilepsy, recurrent seizures, convulsions	Wald ratio	1	0.140685	0.196959	0.475051	1.151062	0.782427	1.693378	0.998644
mental disorders	Schizophrenia	Wald ratio	1	0.412677	0.581499	0.477904	1.510856	0.483327	4.72286	0.998644
digestive	Intestinal obstruction without mention of hernia	Wald ratio	1	0.159443	0.225096	0.478738	1.172858	0.754466	1.823271	0.998644
circulatory system	Chronic pulmonary heart disease	Wald ratio	1	0.403298	0.57212	0.480861	1.496752	0.487699	4.593548	0.998644
neoplasms	Secondary malignancy of lymph nodes	Wald ratio	1	-0.13131	0.18758	0.483927	0.876949	0.60716	1.266619	0.998644
digestive	Acute pancreatitis	Wald ratio	1	0.215717	0.309508	0.485822	1.240752	0.676436	2.275846	0.998644
symptoms	Effects of other external causes	Wald ratio	1	0.150064	0.215717	0.486647	1.161909	0.761289	1.77335	0.998644
digestive	Disorders of esophageal motility	Wald ratio	1	-0.38454	0.553362	0.487108	0.680764	0.230126	2.013853	0.998644
circulatory system	Nonspecific abnormal findings on radiological and other examination of other intra	Wald ratio	1	0.403298	0.581499	0.487965	1.496752	0.478815	4.678772	0.998644
sense organs	Diplopia and disorders of binocular vision	Wald ratio	1	-0.3564	0.515846	0.489623	0.700191	0.254753	1.924479	0.998644
respiratory	Allergic rhinitis	Wald ratio	1	-0.29075	0.422056	0.490893	0.747703	0.32694	1.709977	0.998644
digestive	Heartburn	Wald ratio	1	0.206338	0.300128	0.491768	1.229169	0.682554	2.213534	0.998644
musculoskeletal	Other and unspecified disc disorder	Wald ratio	1	0.140685	0.206338	0.495354	1.151062	0.768175	1.724795	0.998644
symptoms	Swelling of limb	Wald ratio	1	0.140685	0.206338	0.495354	1.151062	0.768175	1.724795	0.998644
digestive	Ventral hernia	Wald ratio	1	-0.15944	0.234475	0.496504	0.852618	0.538475	1.350032	0.998644
congenital anomal	Cardiac and circulatory congenital anomalies	Wald ratio	1	0.178201	0.262612	0.497409	1.195066	0.714252	1.999549	0.998644
neoplasms	Cancer of other lymphoid, histiocytic tissue	Wald ratio	1	0.196959	0.290749	0.49814	1.217695	0.688727	2.152927	0.998644
endocrine/metabol	Thyrotoxicosis with or without goiter	Wald ratio	1	-0.21572	0.318887	0.498742	0.805963	0.431393	1.505764	0.998644
musculoskeletal	Other unspecified back disorders	Wald ratio	1	0.234475	0.347024	0.499247	1.264245	0.640382	2.495881	0.998644

circulatory system	Cardiac pacemaker/device in situ	Wald ratio	1	-0.18758	0.28137	0.504985	0.828963	0.47756	1.438939	0.998644
digestive	Hemorrhage of gastrointestinal tract	Wald ratio	1	-0.13131	0.196959	0.504985	0.876949	0.5961	1.290118	0.998644
mental disorders	Agorophobia, social phobia, and panic disorder	Wald ratio	1	0.347024	0.525225	0.508796	1.41485	0.505394	3.960872	0.998644
genitourinary	Benign neoplasm of breast	Wald ratio	1	0.234475	0.356403	0.510606	1.264245	0.628717	2.542186	0.998644
sense organs	Aphakia and other disorders of lens	Wald ratio	1	0.215717	0.328266	0.511089	1.240752	0.652018	2.361077	0.998644
neoplasms	Cancer of bronchus; lung	Wald ratio	1	-0.19696	0.300128	0.511663	0.821224	0.456023	1.478891	0.998644
circulatory system	Cardiomegaly	Wald ratio	1	0.178201	0.271991	0.512357	1.195066	0.701242	2.036646	0.998644
sense organs	Perforation of tympanic membrane	Wald ratio	1	-0.28137	0.431435	0.514289	0.754749	0.324009	1.758114	0.998644
neurological	Migraine	Wald ratio	1	0.168822	0.262612	0.520317	1.18391	0.707585	1.980883	0.998644
circulatory system	Late effects of cerebrovascular disease	Wald ratio	1	-0.25323	0.393919	0.520317	0.776287	0.358684	1.68009	0.998644
endocrine/metabol	Other disorders of metabolism	Wald ratio	1	0.234475	0.365782	0.521506	1.264245	0.617265	2.589351	0.998644
pregnancy complic	Early onset of delivery	Wald ratio	1	-0.36578	0.57212	0.522599	0.693654	0.226019	2.128832	0.998644
circulatory system	Hypertensive heart and/or renal disease	Wald ratio	1	-0.21572	0.337645	0.522895	0.805963	0.415821	1.562155	0.998644
mental disorders	Neurological disorders	Wald ratio	1	0.131306	0.206338	0.524539	1.140317	0.761004	1.708694	0.998644
neurological	Parkinson's disease	Wald ratio	1	0.262612	0.412677	0.524539	1.300323	0.579127	2.919635	0.998644
circulatory system	Other disorders of arteries and arterioles	Wald ratio	1	0.243854	0.38454	0.525985	1.276159	0.60059	2.711636	0.998644
circulatory system	Cerebral artery occlusion, with cerebral infarction	Wald ratio	1	0.225096	0.356403	0.527662	1.252443	0.622848	2.518455	0.998644
neoplasms	Large cell lymphoma	Wald ratio	1	-0.36578	0.581499	0.529328	0.693654	0.221902	2.168328	0.998644
injuries & poisoni	Foreign body injury	Wald ratio	1	0.253233	0.403298	0.530065	1.288184	0.584365	2.839696	0.998644
digestive	Other biliary tract disease	Wald ratio	1	0.140685	0.225096	0.531971	1.151062	0.740445	1.789389	0.998644
digestive	Ascites (non malignant)	Wald ratio	1	0.215717	0.347024	0.534191	1.240752	0.628481	2.449499	0.998644
genitourinary	Inflammatory disease of breast	Wald ratio	1	-0.31889	0.515846	0.536455	0.726958	0.264492	1.998049	0.998644
congenital anomal	Congenital anomalies of genital organs	Wald ratio	1	0.318887	0.515846	0.536455	1.375595	0.500488	3.780833	0.998644
neoplasms	Neoplasm of uncertain behavior	Wald ratio	1	-0.2251	0.365782	0.538301	0.798439	0.389836	1.635315	0.998644
circulatory system	Arterial embolism and thrombosis	Wald ratio	1	0.28137	0.459572	0.540376	1.324944	0.538273	3.261317	0.998644
respiratory	Emphysema	Wald ratio	1	0.206338	0.337645	0.541126	1.229169	0.634166	2.382432	0.998644
digestive	Functional digestive disorders	Wald ratio	1	-0.05721	0.09379	0.541862	0.944394	0.78581	1.134982	0.998644
symptoms	Other ill-defined and unknown causes of morbidity and mortality	Wald ratio	1	0.068467	0.112548	0.542966	1.070865	0.858879	1.335174	0.998644
mental disorders	Schizophrenia and other psychotic disorders	Wald ratio	1	0.290749	0.47833	0.543292	1.337429	0.523731	3.415336	0.998644
dermatologic	Disturbance of skin sensation	Wald ratio	1	0.159443	0.262612	0.543756	1.172858	0.700979	1.962391	0.998644
digestive	Periapical abscess	Wald ratio	1	-0.24385	0.403298	0.545411	0.783602	0.355469	1.727386	0.998644
genitourinary	Dysmenorrhea	Wald ratio	1	-0.19696	0.328266	0.548506	0.821224	0.431555	1.562741	0.998644
injuries & poisoni	Fracture of hand or wrist	Wald ratio	1	0.140685	0.234475	0.548506	1.151062	0.726958	1.822587	0.998644
neoplasms	Malignant neoplasm of bladder	Wald ratio	1	0.178201	0.300128	0.552679	1.195066	0.663617	2.152119	0.998644
neurological	Sleep apnea	Wald ratio	1	0.121927	0.206338	0.554581	1.129672	0.7539	1.692743	0.998644
circulatory system	Cardiac arrest and ventricular fibrillation	Wald ratio	1	0.243854	0.412677	0.554581	1.276159	0.568365	2.865379	0.998644
sense organs	Other disorders of middle ear and mastoid	Wald ratio	1	0.28137	0.47833	0.556374	1.324944	0.518842	3.383453	0.998644
digestive	Ileostomy status	Wald ratio	1	0.196959	0.337645	0.559669	1.217695	0.628246	2.360191	0.998644
dermatologic	Ingrowing nail	Wald ratio	1	-0.26261	0.450193	0.559669	0.76904	0.318227	1.858492	0.998644
digestive	Acute gastritis	Wald ratio	1	0.234475	0.403298	0.560974	1.264245	0.573505	2.786925	0.998644
symptoms	Back pain	Wald ratio	1	-0.07597	0.131306	0.562878	0.926844	0.716534	1.198883	0.998644
sense organs	Inflammation of the eye	Wald ratio	1	0.140685	0.243854	0.563991	1.151062	0.713717	1.856401	0.998644
endocrine/metabol	Disorders of the pituitary gland and its hypothalamic control	Wald ratio	1	-0.30013	0.525225	0.567709	0.740723	0.264591	2.073654	0.998644
neurological	Sleep disorders	Wald ratio	1	0.112548	0.196959	0.567709	1.119126	0.760718	1.646396	0.998644
digestive	Celiac disease	Wald ratio	1	-0.18758	0.328266	0.567709	0.828963	0.435622	1.577467	0.998644
pregnancy complic	Missed abortion/Hydatidiform mole	Wald ratio	1	-0.2251	0.393919	0.567709	0.798439	0.368919	1.728034	0.998644
musculoskeletal	Derangement of joint, non-traumatic	Wald ratio	1	0.150064	0.262612	0.567709	1.161909	0.694435	1.944072	0.998644
endocrine/metabol	Nontoxic nodular goiter	Wald ratio	1	0.234475	0.412677	0.569912	1.264245	0.563059	2.83863	0.998644
respiratory	Epistaxis or throat hemorrhage	Wald ratio	1	0.159443	0.28137	0.570941	1.172858	0.675675	2.035882	0.998644
dermatologic	Other dyschromia	Wald ratio	1	0.28137	0.497088	0.571368	1.324944	0.500113	3.510163	0.998644
circulatory system	Pulmonary heart disease	Wald ratio	1	-0.12193	0.215717	0.571926	0.885213	0.579996	1.351046	0.998644
musculoskeletal	Aseptic necrosis of bone	Wald ratio	1	0.328266	0.590878	0.578515	1.388558	0.436112	4.42109	0.998644
circulatory system	Hypertension	Wald ratio	1	-0.0347	0.062839	0.580785	0.965893	0.853963	1.092494	0.998644
symptoms	Symptoms concerning nutrition, metabolism, and development	Wald ratio	1	0.103169	0.18758	0.582319	1.108679	0.767599	1.601317	0.998644
injuries & poisoni	Fracture of vertebral column without mention of spinal cord injury	Wald ratio	1	-0.20634	0.375161	0.582319	0.813558	0.389983	1.697194	0.998644
digestive	Portal hypertension	Wald ratio	1	-0.32827	0.600257	0.584465	0.720172	0.222069	2.335528	0.998644
circulatory system	Atrioventricular block, complete	Wald ratio	1	-0.30951	0.57212	0.588519	0.733808	0.239103	2.252065	0.998644
dermatologic	Pilonidal cyst	Wald ratio	1	0.309508	0.57212	0.588519	1.362754	0.444037	4.182305	0.998644
neoplasms	Vascular hamartomas and non-neoplastic nevi	Wald ratio	1	0.290749	0.543983	0.593008	1.337429	0.460493	3.884351	0.998644
dermatologic	Cellulitis and abscess of fingers/toes	Wald ratio	1	0.309508	0.581499	0.594547	1.362754	0.435949	4.259899	0.998644
circulatory system	Cardiac arrest	Wald ratio	1	0.243854	0.459572	0.595688	1.276159	0.518453	3.141232	0.998644
injuries & poisoni	Poisoning by analgesics, antipyretics, and antirheumatics	Wald ratio	1	0.089101	0.168822	0.597654	1.093191	0.78522	1.52195	0.998644
digestive	Dysphagia	Wald ratio	1	0.09379	0.178201	0.598669	1.098329	0.774541	1.557473	0.998644
genitourinary	Urinary incontinence	Wald ratio	1	0.078784	0.150064	0.599583	1.08197	0.806265	1.451953	0.998644
respiratory	Septal Deviations/Turbinate Hypertrophy	Wald ratio	1	-0.10317	0.196959	0.600411	0.901974	0.613111	1.326934	0.998644
genitourinary	Irregular menstrual cycle/bleeding	Wald ratio	1	-0.06378	0.121927	0.600921	0.938214	0.73878	1.191484	0.998644
neurological	Other cerebral degenerations	Wald ratio	1	0.215717	0.412677	0.601164	1.240752	0.552596	2.78588	0.998644
respiratory	Asthma	Wald ratio	1	0.047833	0.091914	0.602779	1.048995	0.876061	1.256067	0.998644
circulatory system	Stricture of artery	Wald ratio	1	0.243854	0.468951	0.603064	1.276159	0.509009	3.199511	0.998644
genitourinary	Other specified benign mammary dysplasias	Wald ratio	1	-0.27199	0.525225	0.604558	0.761861	0.272142	2.132829	0.998644
endocrine/metabol	Simple and unspecified goiter	Wald ratio	1	-0.29075	0.562741	0.605389	0.747703	0.24815	2.25291	0.998644
circulatory system	Paroxysmal supraventricular tachycardia	Wald ratio	1	0.150064	0.290749	0.605764	1.161909	0.657175	2.054296	0.998644
genitourinary	Renal colic	Wald ratio	1	-0.15944	0.309508	0.606447	0.852618	0.464833	1.563914	0.998644
injuries & poisoni	Adverse effects of sedatives or other central nervous system depressants and anestl	Wald ratio	1	-0.29075	0.57212	0.611315	0.747703	0.24363	2.294708	0.998644
hematopoietic	Other anemias	Wald ratio	1	0.061902	0.121927	0.611669	1.063858	0.837716	1.351046	0.998644
circulatory system	Essential hypertension	Wald ratio	1	-0.03189	0.062839	0.61183	0.968614	0.856369	1.095572	0.998644
genitourinary	Functional disorders of bladder	Wald ratio	1	0.168822	0.337645	0.617075	1.18391	0.610815	2.294708	0.998644
neoplasms	Benign neoplasm of uterus	Wald ratio	1	0.069405	0.140685	0.621777	1.07187	0.813558	1.412199	0.998644
endocrine/metabol	Hypothyroidism NOS	Wald ratio	1	-0.06003	0.121927	0.622502	0.94174	0.741557	1.195963	0.998644
symptoms	Chronic fatigue syndrome	Wald ratio	1	-0.28137	0.57212	0.622858	0.754749	0.245926	2.316331	0.998644
sense organs	Retinal vascular changes and abnomalities	Wald ratio	1	0.234475	0.47833	0.623995	1.264245	0.495073	3.228449	0.998644
endocrine/metabol	Hypopotassemia	Wald ratio	1	-0.1782	0.365782	0.626131	0.836774	0.408553	1.71383	0.998644
pregnancy complic	Hemorrhage during pregnancy; childbirth and postpartum	Wald ratio	1	-0.15944	0.328266	0.62717	0.852618	0.448053	1.622482	0.998644
neoplasms	Malignant neoplasm of rectum, rectosigmoid junction, and anus	Wald ratio	1	0.150064	0.309508	0.627784	1.161909	0.633452	2.131229	0.998644
musculoskeletal	Other derangement of joint	Wald ratio	1	0.150064	0.309508	0.627784	1.161909	0.633452	2.131229	0.998644
circulatory system	Vascular insufficiency of intestine	Wald ratio	1	-0.28137	0.581499	0.628477	0.754749	0.241446	2.359306	0.998644
digestive	Gastritis and duodenitis	Wald ratio	1	0.042206	0.087225	0.628477	1.043109	0.879189	1.23759	0.998644
injuries & poisoni	Effects radiation NOS	Wald ratio	1	-0.12193	0.253233	0.630174	0.885213	0.538879	1.454134	0.998644
digestive	Hemorrhage of rectum and anus	Wald ratio	1	-0.05815	0.121927	0.633417	0.943509	0.742949	1.198208	0.998644
genitourinary	Chronic cystitis	Wald ratio	1	0.215717	0.459572	0.638792	1.240752	0.504068	3.054079	0.998644
neoplasms	Lipoma	Wald ratio	1	-0.08347	0.178201	0.639484	0.919916	0.648724	1.304476	0.998644
musculoskeletal	Rheumatism, unspecified and fibrositis	Wald ratio	1	0.206338	0.440814	0.639724	1.229169	0.518064	2.916351	0.998644
hematopoietic	Megaloblastic anemia	Wald ratio	1	0.196959	0.422056	0.640738	1.217695	0.532448	2.784835	0.998644
injuries & poisoni	Allergy/adverse effect of penicillin	Wald ratio	1	-0.05252	0.112548	0.640738	0.948833	0.761004	1.183022	0.998644
hematopoietic	Decreased white blood cell count	Wald ratio	1	0.112548	0.243854	0.644412	1.119126	0.693915	1.804896	0.998644
hematopoietic	Neutropenia	Wald ratio	1	0.112548	0.243854	0.644412	1.119126	0.693915	1.804896	0.998644
digestive	Other symptoms involving abdomen and pelvis	Wald ratio	1	-0.11255	0.243854	0.644412	0.893554	0.554049	1.4411	0.998644

dermatologic	Hyperhidrosis	Wald ratio	1	-0.26261	0.57212	0.646222	0.76904	0.250582	2.360191	0.998644
symptoms	Pain in limb	Wald ratio	1	0.081597	0.178201	0.647028	1.085019	0.765155	1.538599	0.998644
mental disorders	Alcoholism	Wald ratio	1	-0.06847	0.150064	0.64821	0.933824	0.69587	1.253148	0.998644
injuries & poisoni	Fracture of upper limb	Wald ratio	1	0.068467	0.150064	0.64821	1.070865	0.79799	1.43705	0.998644
endocrine/metabol	Anorexia	Wald ratio	1	0.215717	0.47833	0.652004	1.240752	0.485873	3.168454	0.998644
musculoskeletal	Disorders of muscle, ligament, and fascia	Wald ratio	1	-0.09285	0.206338	0.65271	0.911328	0.608185	1.365569	0.998644
endocrine/metabol	Crystal arthropathies	Wald ratio	1	-0.25323	0.562741	0.65271	0.776287	0.257636	2.339036	0.998644
musculoskeletal	Osteoarthrosis, localized, primary	Wald ratio	1	-0.06753	0.150064	0.65271	0.934701	0.696523	1.254324	0.998644
circulatory system	Mitral valve disease	Wald ratio	1	0.112548	0.253233	0.656721	1.119126	0.681275	1.838382	0.998644
digestive	Abnormal findings on exam of gastrointestinal tract/ abdominal area	Wald ratio	1	0.150064	0.337645	0.656721	1.161909	0.599464	2.252065	0.998644
neoplasms	Malignant neoplasm of female breast	Wald ratio	1	-0.05815	0.131306	0.657869	0.943509	0.729417	1.220439	0.998644
genitourinary	Hydronephrosis	Wald ratio	1	0.140685	0.318887	0.659085	1.151062	0.616108	2.150505	0.998644
endocrine/metabol	Disorders of parathyroid gland	Wald ratio	1	0.206338	0.468951	0.659937	1.229169	0.490267	3.081701	0.998644
musculoskeletal	Unspecified polyarthropathy or polyarthritis	Wald ratio	1	0.103169	0.234475	0.659937	1.108679	0.700191	1.755478	0.998644
respiratory	Shortness of breath	Wald ratio	1	0.077846	0.178201	0.662226	1.080956	0.76229	1.532837	0.998644
musculoskeletal	Osteoarthrosis, generalized	Wald ratio	1	0.243854	0.562741	0.664773	1.276159	0.423536	3.845204	0.998644
hematopoietic	Thrombocytopenia	Wald ratio	1	-0.15006	0.347024	0.665427	0.860653	0.435949	1.699106	0.998644
circulatory system	Raynaud's syndrome	Wald ratio	1	0.178201	0.412677	0.665874	1.195066	0.532248	2.683301	0.998644
respiratory	Chronic tonsillitis and adenoiditis	Wald ratio	1	0.178201	0.412677	0.665874	1.195066	0.532248	2.683301	0.998644
musculoskeletal	Pathologic fracture	Wald ratio	1	0.262612	0.609636	0.666636	1.300323	0.393657	4.295204	0.998644
circulatory system	Cardiac dysrhythmias	Wald ratio	1	0.04033	0.09379	0.667196	1.041154	0.866322	1.251269	0.998644
genitourinary	Cystic mastopathy	Wald ratio	1	-0.19696	0.459572	0.668235	0.821224	0.333631	2.021422	0.998644
circulatory system	Other forms of chronic heart disease	Wald ratio	1	0.140685	0.328266	0.668235	1.151062	0.604886	2.190403	0.998644
circulatory system	Other aneurysm	Wald ratio	1	-0.14069	0.328266	0.668235	0.868763	0.456537	1.653204	0.998644
respiratory	Respiratory insufficiency	Wald ratio	1	0.140685	0.328266	0.668235	1.151062	0.604886	2.190403	0.998644
genitourinary	Other disorders of male genital organs	Wald ratio	1	-0.11255	0.262612	0.668235	0.893554	0.534049	1.495069	0.998644
dermatologic	Psoriatic arthropathy	Wald ratio	1	0.225096	0.525225	0.668235	1.252443	0.447381	3.506215	0.998644
circulatory system	Aortic aneurysm	Wald ratio	1	-0.15944	0.375161	0.670837	0.852618	0.408706	1.77868	0.998644
musculoskeletal	Intervertebral disc disorders	Wald ratio	1	-0.06378	0.150064	0.670837	0.938214	0.699141	1.259039	0.998644
genitourinary	Frequency of urination and polyuria	Wald ratio	1	0.090976	0.215717	0.673215	1.095243	0.71761	1.671602	0.998644
endocrine/metabol	Nontoxic multinodular goiter	Wald ratio	1	0.225096	0.534604	0.673717	1.252443	0.439232	3.571265	0.998644
endocrine/metabol	Chondrocalcinosis	Wald ratio	1	-0.24385	0.581499	0.674957	0.783602	0.250676	2.449499	0.998644
digestive	Flatulence	Wald ratio	1	0.140685	0.337645	0.676922	1.151062	0.593868	2.231042	0.998644
musculoskeletal	Other inflammatory spondylopathies	Wald ratio	1	-0.14069	0.337645	0.676922	0.868763	0.448221	1.683876	0.998644
pregnancy complic	Problems associated with amniotic cavity and membranes	Wald ratio	1	0.159443	0.38454	0.67841	1.172858	0.551974	2.492138	0.998644
genitourinary	Chronic renal failure [CKD]	Wald ratio	1	0.112548	0.271991	0.679026	1.119126	0.656682	1.907229	0.998644
symptoms	Gangrene	Wald ratio	1	0.243854	0.590878	0.679828	1.276159	0.400811	4.063218	0.998644
neoplasms	Secondary malignancy of bone	Wald ratio	1	0.121927	0.300128	0.684559	1.129672	0.627304	2.034355	0.998644
neoplasms	Cancer of bladder	Wald ratio	1	0.112548	0.28137	0.689157	1.119126	0.644721	1.942614	0.998644
endocrine/metabol	Acidosis	Wald ratio	1	-0.16882	0.422056	0.689157	0.844659	0.369335	1.931712	0.998644
respiratory	Acute pharyngitis	Wald ratio	1	-0.18758	0.468951	0.689157	0.828963	0.33064	2.078327	0.998644
digestive	Disturbances in tooth eruption	Wald ratio	1	-0.11255	0.28137	0.689157	0.893554	0.51477	1.551059	0.998644
digestive	Disorders of tooth development	Wald ratio	1	-0.11255	0.28137	0.689157	0.893554	0.51477	1.551059	0.998644
digestive	Paralytic ileus	Wald ratio	1	-0.24385	0.609636	0.689157	0.783602	0.237226	2.58838	0.998644
genitourinary	Urinary calculus	Wald ratio	1	-0.06753	0.168822	0.689157	0.934701	0.67138	1.301299	0.998644
dermatologic	Pruritus and related conditions	Wald ratio	1	-0.19696	0.497088	0.691938	0.821224	0.309979	2.175662	0.998644
symptoms	Other tests	Wald ratio	1	-0.07034	0.178201	0.693037	0.932074	0.657298	1.321717	0.998644
circulatory system	Right bundle branch block	Wald ratio	1	-0.14069	0.356403	0.693037	0.868763	0.432041	1.746937	0.998644
neoplasms	Benign neoplasm of skin	Wald ratio	1	-0.06284	0.159443	0.693494	0.939094	0.68705	1.283601	0.998644
respiratory	Hemoptysis	Wald ratio	1	0.121927	0.309508	0.693626	1.129672	0.615877	2.072099	0.998644
respiratory	Cough	Wald ratio	1	-0.10317	0.262612	0.694425	0.901974	0.539081	1.509157	0.998644
injuries & poisoni	Adverse drug events and drug allergies	Wald ratio	1	-0.20634	0.525225	0.694425	0.813558	0.290608	2.277555	0.998644
circulatory system	Premature beats	Wald ratio	1	0.234475	0.600257	0.696074	1.264245	0.389836	4.099968	0.998644
neoplasms	Chemotherapy	Wald ratio	1	0.036578	0.09379	0.696537	1.037255	0.863078	1.246584	0.998644
sense organs	Strabismus (not specified as paralytic)	Wald ratio	1	0.178201	0.459572	0.698197	1.195066	0.485508	2.941624	0.998644
digestive	Chronic periodontitis	Wald ratio	1	0.225096	0.581499	0.698685	1.252443	0.40066	3.915074	0.998644
neoplasms	Secondary malignancy of brain/spine	Wald ratio	1	0.18758	0.487709	0.700522	1.206327	0.463787	3.137698	0.998644
mental disorders	Tobacco use disorder	Wald ratio	1	-0.03939	0.103169	0.702596	0.961374	0.785368	1.176824	0.998644
neoplasms	Uterine leiomyoma	Wald ratio	1	0.05346	0.140685	0.703945	1.054915	0.800689	1.389861	0.998644
respiratory	Postinflammatory pulmonary fibrosis	Wald ratio	1	-0.1782	0.468951	0.703945	0.836774	0.333756	2.097911	0.998644
dermatologic	Unspecified diffuse connective tissue disease	Wald ratio	1	0.103169	0.271991	0.704457	1.108679	0.650552	1.889425	0.998644
neoplasms	Benign neoplasm of other parts of digestive system	Wald ratio	1	0.074094	0.196959	0.706775	1.076908	0.732021	1.584287	0.998644
digestive	Gastric ulcer	Wald ratio	1	0.08066	0.215717	0.708469	1.084002	0.710244	1.654445	0.998644
digestive	Duodenitis	Wald ratio	1	0.059088	0.159443	0.710944	1.060868	0.776141	1.450048	0.998644
circulatory system	Hemorrhoids	Wald ratio	1	0.034702	0.09379	0.711382	1.035312	0.86146	1.244248	0.998644
sense organs	Inflammation of eyelids	Wald ratio	1	0.103169	0.28137	0.713868	1.108679	0.638702	1.924479	0.998644
endocrine/metabol	Type 2 diabetes with ophthalmic manifestations	Wald ratio	1	0.140685	0.38454	0.714474	1.151062	0.541717	2.445826	0.998644
injuries & poisoni	Injuries to the nervous system	Wald ratio	1	0.140685	0.38454	0.714474	1.151062	0.541717	2.445826	0.998644
respiratory	Nasal polyps	Wald ratio	1	-0.08816	0.243854	0.717697	0.915612	0.567725	1.476673	0.998644
digestive	Other chronic nonalcoholic liver disease	Wald ratio	1	0.121927	0.337645	0.718016	1.129672	0.582832	2.189582	0.998644
endocrine/metabol	Hyperparathyroidism	Wald ratio	1	0.178201	0.497088	0.719976	1.195066	0.451089	3.166077	0.998644
dermatologic	Sebaceous cyst	Wald ratio	1	-0.05346	0.150064	0.721653	0.947943	0.706391	1.272095	0.998644
congenital anomal	Congenital anomalies of urinary system	Wald ratio	1	0.168822	0.47833	0.724133	1.18391	0.463613	3.023299	0.998644
circulatory system	Orthostatic hypotension	Wald ratio	1	0.131306	0.375161	0.726339	1.140317	0.546616	2.378859	0.998644
pregnancy complic	Miscarriage; stillbirth	Wald ratio	1	0.072218	0.206338	0.726339	1.07489	0.71734	1.610656	0.998644
musculoskeletal	Osteoarthritis; localized	Wald ratio	1	0.039392	0.112548	0.726339	1.040178	0.834266	1.296912	0.998644
dermatologic	Atopic/contact dermatitis due to other or unspecified	Wald ratio	1	-0.10317	0.300128	0.731034	0.901974	0.500864	1.624309	0.998644
neoplasms	Cervical cancer	Wald ratio	1	-0.11255	0.337645	0.738883	0.893554	0.461012	1.731928	0.998644
digestive	Ulceration of the lower GI tract	Wald ratio	1	-0.14069	0.422056	0.738883	0.868763	0.379874	1.986837	0.998644
respiratory	Chronic pharyngitis and nasopharyngitis	Wald ratio	1	0.150064	0.450193	0.738883	1.161909	0.480795	2.807914	0.998644
musculoskeletal	Malunion and nonunion of fracture	Wald ratio	1	-0.13131	0.393919	0.738883	0.876949	0.405195	1.89795	0.998644
digestive	Dental caries	Wald ratio	1	-0.08254	0.253233	0.74448	0.920779	0.56053	1.512558	0.998644
endocrine/metabol	Acid-base balance disorder	Wald ratio	1	-0.13131	0.403298	0.744741	0.876949	0.397814	1.933162	0.998644
pregnancy complic	Placenta previa and abruptio placenta	Wald ratio	1	-0.13131	0.403298	0.744741	0.876949	0.397814	1.933162	0.998644
sense organs	Other disorders of tympanic membrane	Wald ratio	1	-0.12193	0.375161	0.745181	0.885213	0.424331	1.846677	0.998644
neoplasms	Cancer of other female genital organs	Wald ratio	1	0.090976	0.28137	0.746443	1.095243	0.630962	1.901157	0.998644
digestive	Chronic pancreatitis	Wald ratio	1	0.196959	0.609636	0.746637	1.217695	0.368643	4.022268	0.998644
sense organs	Labyrinthitis	Wald ratio	1	0.159443	0.497088	0.748396	1.172858	0.442706	3.107242	0.998644
neoplasms	Myeloproliferative disease	Wald ratio	1	0.140685	0.440814	0.749614	1.151062	0.485144	2.731034	0.998644
circulatory system	Elevated blood pressure reading without diagnosis of hypertension	Wald ratio	1	0.112548	0.356403	0.752162	1.119126	0.556549	2.250376	0.998644
digestive	Periodontitis (acute or chronic)	Wald ratio	1	-0.12193	0.393919	0.756923	0.885213	0.409013	1.915835	0.998644
circulatory system	Nonrheumatic mitral valve disorders	Wald ratio	1	0.08066	0.262612	0.758735	1.084002	0.647873	1.81372	0.998644
digestive	Regional enteritis	Wald ratio	1	0.103169	0.337645	0.759943	1.108679	0.572001	2.148892	0.998644
hematopoietic	Anemia of chronic disease	Wald ratio	1	-0.15944	0.525225	0.761454	0.852618	0.304561	2.386905	0.998644
genitourinary	Other signs and symptoms in breast	Wald ratio	1	-0.15944	0.525225	0.761454	0.852618	0.304561	2.386905	0.998644
symptoms	Symptoms of the muscles	Wald ratio	1	0.159443	0.525225	0.761454	1.172858	0.418953	3.283415	0.998644
digestive	Intestinal malabsorption (non-celiac)	Wald ratio	1	-0.09379	0.309508	0.761867	0.910474	0.496374	1.670035	0.998644

digestive	Other diseases of the teeth and supporting structures	Wald ratio	1	-0.0816	0.271991	0.764177	0.921643	0.540803	1.570676	0.998644
genitourinary	Abnormal findings on mammogram or breast exam	Wald ratio	1	-0.10317	0.347024	0.76624	0.901974	0.45688	1.780683	0.998644
infectious diseases	Postoperative infection	Wald ratio	1	-0.06096	0.206338	0.767647	0.940857	0.627892	1.409816	0.998644
neoplasms	Other benign neoplasm of connective and other soft tissue	Wald ratio	1	-0.12193	0.412677	0.767647	0.885213	0.394249	1.987583	0.998644
dermatologic	Diffuse diseases of connective tissue	Wald ratio	1	-0.06847	0.234475	0.770287	0.933824	0.589761	1.478614	0.998644
digestive	Other disorders of liver	Wald ratio	1	0.054398	0.18758	0.771816	1.055905	0.73106	1.525093	0.998644
dermatologic	Diseases of sebaceous glands	Wald ratio	1	-0.04314	0.150064	0.77373	0.957774	0.713717	1.285288	0.998644
symptoms	Syncope and collapse	Wald ratio	1	0.043143	0.150064	0.77373	1.044088	0.778036	1.401116	0.998644
sense organs	Glaucoma	Wald ratio	1	-0.05909	0.206338	0.7746	0.942624	0.629071	1.412464	0.998644
neurological	Extrapyramidal disease and abnormal movement disorders	Wald ratio	1	0.131306	0.459572	0.775097	1.140317	0.463266	2.806861	0.998644
neurological	Epilepsy	Wald ratio	1	0.131306	0.459572	0.775097	1.140317	0.463266	2.806861	0.998644
sense organs	Strabismus and other disorders of binocular eye movements	Wald ratio	1	0.103169	0.365782	0.777904	1.108679	0.54131	2.270729	0.998644
respiratory	Other symptoms of respiratory system	Wald ratio	1	0.042206	0.150064	0.778519	1.043109	0.777307	1.399803	0.998644
digestive	Peritonitis and retroperitoneal infections	Wald ratio	1	0.131306	0.468951	0.779478	1.140317	0.454827	2.858937	0.998644
musculoskeletal	Enthesopathy	Wald ratio	1	-0.03939	0.140685	0.779478	0.961374	0.72969	1.266619	0.998644
digestive	Diseases of hard tissues of teeth	Wald ratio	1	-0.07034	0.253233	0.781183	0.932074	0.567406	1.531113	0.998644
symptoms	Abdominal pain	Wald ratio	1	0.019696	0.071281	0.782306	1.019891	0.886908	1.172814	0.998644
digestive	Other disorders of gallbladder	Wald ratio	1	0.103169	0.375161	0.783316	1.108679	0.53145	2.312858	0.998644
neoplasms	Benign neoplasm of brain and other parts of nervous system	Wald ratio	1	-0.13131	0.47833	0.783693	0.876949	0.343409	2.239427	0.998644
musculoskeletal	Osteoarthrosis	Wald ratio	1	0.023448	0.088163	0.790272	1.023725	0.861266	1.216827	0.998644
injuries & poisoni	Hemorrhage or hematoma complicating a procedure	Wald ratio	1	-0.04877	0.18758	0.794864	0.952399	0.659398	1.375595	0.998644
neoplasms	Cancer of mouth	Wald ratio	1	-0.14069	0.543983	0.795928	0.868763	0.299126	2.523183	0.998644
circulatory system	Angina pectoris	Wald ratio	1	-0.02907	0.112548	0.79615	0.971344	0.779058	1.211088	0.998644
digestive	Cholecystitis without cholelithiasis	Wald ratio	1	0.067529	0.262612	0.797068	1.069861	0.639421	1.79006	0.998644
genitourinary	Dyspareunia	Wald ratio	1	-0.10317	0.403298	0.798094	0.901974	0.409167	1.988328	0.998644
genitourinary	Bladder neck obstruction	Wald ratio	1	-0.08066	0.318887	0.800314	0.922508	0.493774	1.723502	0.998644
musculoskeletal	Acquired toe deformities	Wald ratio	1	0.049709	0.196959	0.800747	1.050965	0.714386	1.546121	0.998644
endocrine/metabol	Hyposmolality and/or hyponatremia	Wald ratio	1	-0.08254	0.328266	0.801483	0.920779	0.483872	1.752188	0.998644
neurological	Nerve root and plexus disorders	Wald ratio	1	-0.10317	0.412677	0.802587	0.901974	0.401714	2.025218	0.998644
genitourinary	Pelvic inflammatory disease (PID)	Wald ratio	1	-0.11255	0.450193	0.802587	0.893554	0.369751	2.159398	0.998644
circulatory system	Occlusion of cerebral arteries	Wald ratio	1	0.05346	0.215717	0.804269	1.054915	0.691186	1.610052	0.998644
circulatory system	Abnormal function study of cardiovascular system	Wald ratio	1	-0.15006	0.609636	0.805563	0.860653	0.260553	2.842893	0.998644
neoplasms	Colorectal cancer	Wald ratio	1	0.049709	0.206338	0.809626	1.050965	0.701374	1.574806	0.998644
infectious diseases	Staphylococcus infections	Wald ratio	1	0.05815	0.243854	0.811523	1.059874	0.657175	1.709335	0.998644
circulatory system	Phlebitis and thrombophlebitis of lower extremities	Wald ratio	1	-0.05534	0.234475	0.813433	0.946167	0.597556	1.498157	0.998644
digestive	Esophagitis, GERD and related diseases	Wald ratio	1	-0.0197	0.083473	0.813468	0.980497	0.832515	1.154782	0.998644
neurological	Multiple sclerosis	Wald ratio	1	-0.08816	0.375161	0.814209	0.915612	0.438903	1.910093	0.998644
circulatory system	Left bundle branch block	Wald ratio	1	0.076908	0.328266	0.814763	1.079943	0.567512	2.055067	0.998644
digestive	Diseases and other conditions of the tongue	Wald ratio	1	-0.09191	0.393919	0.815503	0.912183	0.421475	1.974206	0.998644
circulatory system	Endocarditis	Wald ratio	1	-0.12193	0.525225	0.816427	0.885213	0.316204	2.478153	0.998644
hematopoietic	Iron deficiency anemias	Wald ratio	1	0.036578	0.159443	0.818549	1.037255	0.758866	1.417772	0.998644
respiratory	Acute upper respiratory infections of multiple or unspecified sites	Wald ratio	1	-0.06659	0.290749	0.818844	0.935578	0.529162	1.654135	0.998644
musculoskeletal	Other arthropathies	Wald ratio	1	-0.01782	0.077846	0.818934	0.982338	0.843329	1.144259	0.998644
digestive	Noninfectious gastroenteritis	Wald ratio	1	0.025323	0.112548	0.821979	1.025647	0.822612	1.278794	0.998644
respiratory	Diseases of the larynx and vocal cords	Wald ratio	1	0.060964	0.271991	0.82265	1.06286	0.623666	1.81134	0.998644
digestive	Ulcer of esophagus	Wald ratio	1	0.044081	0.196959	0.822906	1.045067	0.710377	1.537445	0.998644
genitourinary	Urinary tract infection	Wald ratio	1	0.027199	0.121927	0.823476	1.027572	0.809144	1.304965	0.998644
injuries & poisoni	Mechanical complication of unspecified genitourinary device, implant, and graft	Wald ratio	1	0.087225	0.393919	0.824759	1.091142	0.504163	2.36152	0.998644
digestive	Reflux esophagitis	Wald ratio	1	-0.03095	0.140685	0.825871	0.969523	0.735876	1.277356	0.998644
injuries & poisoni	Fracture of clavicle or scapula	Wald ratio	1	-0.07785	0.356403	0.827101	0.925107	0.460062	1.860236	0.998644
dermatologic	Other local infections of skin and subcutaneous tissue	Wald ratio	1	0.045019	0.206338	0.827287	1.046048	0.698092	1.567438	0.998644
digestive	Irritable Bowel Syndrome	Wald ratio	1	-0.04033	0.18758	0.829767	0.960473	0.664987	1.387256	0.998644
infectious diseases	Viral warts & HPV	Wald ratio	1	0.09379	0.440814	0.83151	1.098329	0.462918	2.605918	0.998644
circulatory system	Atrioventricular [AV] block	Wald ratio	1	-0.06378	0.300128	0.831717	0.938214	0.520988	1.689571	0.998644
neoplasms	Colon cancer	Wald ratio	1	0.05346	0.253233	0.832801	1.054915	0.642186	1.732903	0.998644
mental disorders	Alcohol-related disorders	Wald ratio	1	-0.02532	0.121927	0.835469	0.974995	0.767743	1.238194	0.998644
endocrine/metabol	Diabetic retinopathy	Wald ratio	1	-0.07878	0.38454	0.837667	0.92424	0.434969	1.963864	0.998644
sense organs	Subjective visual disturbances	Wald ratio	1	0.112548	0.553362	0.83883	1.119126	0.37831	3.310627	0.998644
musculoskeletal	Arthropathy NOS	Wald ratio	1	-0.01594	0.079722	0.841481	0.984182	0.841812	1.150631	0.998644
digestive	Liver abscess and sequelae of chronic liver disease	Wald ratio	1	0.089101	0.450193	0.84311	1.093191	0.45236	2.641847	0.998644
neurological	Convulsions	Wald ratio	1	0.059088	0.300128	0.843925	1.060868	0.589097	1.910452	0.998644
circulatory system	Paroxysmal tachycardia, unspecified	Wald ratio	1	0.047833	0.243854	0.84449	1.048995	0.65043	1.691791	0.998644
circulatory system	Hypotension	Wald ratio	1	0.034702	0.178201	0.845599	1.035312	0.730101	1.468112	0.998644
injuries & poisoni	Fracture of radius and ulna	Wald ratio	1	-0.03752	0.196959	0.848936	0.963179	0.654714	1.416975	0.998644
symptoms	Sciatica	Wald ratio	1	0.05346	0.28137	0.849309	1.054915	0.607729	1.831154	0.998644
circulatory system	Other acute and subacute forms of ischemic heart disease	Wald ratio	1	-0.07597	0.403298	0.850585	0.926844	0.420448	2.043151	0.998644
digestive	Cholelithiasis with other cholecystitis	Wald ratio	1	-0.0347	0.18758	0.853229	0.965893	0.66874	1.395085	0.998644
pregnancy complic	Umbilical cord complications during labor and delivery	Wald ratio	1	0.112548	0.609636	0.853531	1.119126	0.338802	3.696679	0.998644
sense organs	Blindness and low vision	Wald ratio	1	0.09379	0.515846	0.855725	1.098329	0.399609	3.018766	0.998644
neoplasms	Secondary malignancy of respiratory organs	Wald ratio	1	0.054398	0.300128	0.856171	1.055905	0.586341	1.901513	0.998644
digestive	Peritoneal or intestinal adhesions	Wald ratio	1	0.084411	0.47833	0.859924	1.088076	0.426086	2.778573	0.998644
dermatologic	Diseases of nail, NOS	Wald ratio	1	-0.06753	0.38454	0.860601	0.934701	0.439892	1.986092	0.998644
neurological	Abnormal involuntary movements	Wald ratio	1	-0.0816	0.468951	0.861865	0.921643	0.367607	2.31069	0.998644
neoplasms	Cervical intraepithelial neoplasia [CIN] [Cervical dysplasia]	Wald ratio	1	-0.05346	0.309508	0.862866	0.947943	0.516802	1.738764	0.998644
neoplasms	Benign neoplasm of lip, oral cavity, and pharynx	Wald ratio	1	-0.07503	0.440814	0.864843	0.927714	0.391008	2.201112	0.998644
dermatologic	Cellulitis and abscess of leg, except foot	Wald ratio	1	-0.03189	0.18758	0.86501	0.968614	0.670624	1.399015	0.998644
circulatory system	Heart valve disorders	Wald ratio	1	0.036578	0.215717	0.865352	1.037255	0.679616	1.583099	0.998644
neoplasms	Malignant neoplasm of ovary and other uterine adnexa	Wald ratio	1	0.050647	0.300128	0.865993	1.051951	0.584146	1.894393	0.998644
genitourinary	Inflammatory diseases of female pelvic organs	Wald ratio	1	0.030013	0.178201	0.866252	1.030468	0.726685	1.461243	0.998644
genitourinary	Disorders of uterus, NEC	Wald ratio	1	0.04033	0.243854	0.868641	1.041154	0.645568	1.679144	0.998644
neoplasms	Malignant neoplasm of ovary	Wald ratio	1	0.048771	0.300128	0.870912	1.04998	0.583051	1.890843	0.998644
neoplasms	Benign neoplasm of brain, cranial nerves, meninges	Wald ratio	1	-0.08066	0.497088	0.871098	0.922508	0.348209	2.443992	0.998644
mental disorders	Other mental disorder	Wald ratio	1	0.014069	0.088163	0.873216	1.014168	0.853226	1.205468	0.998644
genitourinary	Hypertrophy of female genital organs	Wald ratio	1	-0.06284	0.393919	0.873256	0.939094	0.433909	2.032448	0.998644
musculoskeletal	Hammer toe (acquired)	Wald ratio	1	-0.05065	0.318887	0.873808	0.950614	0.508818	1.776013	0.998644
dermatologic	Sarcoidosis	Wald ratio	1	-0.09379	0.590878	0.873881	0.910474	0.285958	2.898898	0.998644
endocrine/metabol	Gout	Wald ratio	1	0.038454	0.243854	0.874699	1.039203	0.644358	1.675998	0.998644
genitourinary	Pelvic peritoneal adhesions, female (postoperative) (postinfection)	Wald ratio	1	0.042206	0.271991	0.876685	1.043109	0.612077	1.777679	0.998644
neoplasms	Cancer of esophagus	Wald ratio	1	-0.07972	0.515846	0.87718	0.923373	0.335955	2.537898	0.998644
injuries & poisoni	Poisoning by antibiotics	Wald ratio	1	-0.01594	0.103169	0.87718	0.984182	0.804	1.204744	0.998644
musculoskeletal	Other disorders of bone and cartilage	Wald ratio	1	0.034702	0.225096	0.877478	1.035312	0.665986	1.609448	0.998644
respiratory	Empyema and pneumothorax	Wald ratio	1	-0.06096	0.403298	0.879847	0.940857	0.426805	2.074043	0.998644
digestive	Abdominal hernia	Wald ratio	1	-0.01032	0.068467	0.880224	0.989736	0.865445	1.131878	0.998644
injuries & poisoni	Nonspecific abnormal findings on radiological and other examination of musculosk	Wald ratio	1	0.078784	0.525225	0.880765	1.08197	0.386487	3.028976	0.998644
genitourinary	Menopausal and postmenopausal disorders	Wald ratio	1	0.020634	0.140685	0.883395	1.020848	0.774832	1.344977	0.998644
genitourinary	Inflammatory disease of cervix, vagina, and vulva	Wald ratio	1	-0.03845	0.262612	0.883583	0.962276	0.575121	1.610052	0.998644
respiratory	Obstructive chronic bronchitis	Wald ratio	1	0.039392	0.271991	0.884847	1.040178	0.610357	1.772685	0.998644

genitourinary	Lump or mass in breast	Wald ratio	1	-0.04971	0.356403	0.889076	0.951506	0.47319	1.913321	0.998644
congenital anomal	Other congenital musculoskeletal anomalies	Wald ratio	1	0.079722	0.57212	0.889178	1.082986	0.352878	3.323694	0.998644
mental disorders	Other specified nonpsychotic and/or transient mental disorders	Wald ratio	1	-0.07691	0.553362	0.889464	0.925975	0.313017	2.739242	0.998644
digestive	Umbilical hernia	Wald ratio	1	-0.03189	0.234475	0.891821	0.968614	0.611732	1.5337	0.998644
sense organs	Degeneration of macula and posterior pole of retina	Wald ratio	1	0.04033	0.300128	0.893106	1.041154	0.57815	1.874949	0.998644
genitourinary	Disorders of menstruation and other abnormal bleeding from female genital tract	Wald ratio	1	-0.01501	0.112548	0.89393	0.985106	0.790096	1.228247	0.998644
sense organs	Amblyopia	Wald ratio	1	-0.07972	0.600257	0.894342	0.923373	0.284727	2.994514	0.998644
respiratory	Bronchiectasis	Wald ratio	1	0.042206	0.318887	0.894705	1.043109	0.558326	1.948818	0.998644
circulatory system	Heart valve replaced	Wald ratio	1	0.046895	0.356403	0.895317	1.048012	0.521183	2.107377	0.998644
digestive	Other disorders of peritoneum	Wald ratio	1	0.031889	0.243854	0.895958	1.032403	0.640142	1.66503	0.998644
genitourinary	Benign mammary dysplasias	Wald ratio	1	-0.03189	0.243854	0.895958	0.968614	0.60059	1.562155	0.998644
digestive	Other disorders of biliary tract	Wald ratio	1	0.055336	0.431435	0.897943	1.056896	0.45372	2.461936	0.998644
sense organs	Macular degeneration (senile) of retina NOS	Wald ratio	1	0.038454	0.300128	0.89805	1.039203	0.577066	1.871436	0.998644
sense organs	Open-angle glaucoma	Wald ratio	1	0.05346	0.431435	0.901384	1.054915	0.452869	2.457323	0.998644
musculoskeletal	Other and unspecified disorders of back	Wald ratio	1	0.037516	0.309508	0.903523	1.038229	0.566024	1.904369	0.998644
neurological	Inflammatory and toxic neuropathy	Wald ratio	1	-0.04502	0.375161	0.904483	0.955979	0.458253	1.994305	0.998644
circulatory system	Noninfectious disorders of lymphatic channels	Wald ratio	1	0.060964	0.525225	0.907596	1.06286	0.379661	2.975477	0.998644
respiratory	Other dyspnea	Wald ratio	1	-0.05252	0.459572	0.909011	0.948833	0.385473	2.335528	0.998644
sense organs	Other retinal disorders	Wald ratio	1	0.025323	0.225096	0.910427	1.025647	0.659769	1.594423	0.998644
circulatory system	Varicose veins of lower extremity, symptomtic	Wald ratio	1	-0.0619	0.553362	0.910931	0.939975	0.31775	2.780659	0.998644
circulatory system	Other specified cardiac dysrhythmias	Wald ratio	1	0.027199	0.243854	0.911189	1.027572	0.637147	1.65724	0.998644
musculoskeletal	Hallux valgus (Bunion)	Wald ratio	1	0.018758	0.168822	0.911528	1.018935	0.731884	1.418571	0.998644
neurological	Abnormal movement	Wald ratio	1	0.029075	0.262612	0.911843	1.029502	0.6153	1.722532	0.998644
digestive	Ulcerative colitis (chronic)	Wald ratio	1	0.065653	0.600257	0.912905	1.067856	0.329279	3.463075	0.998644
musculoskeletal	Osteoarthrosis NOS	Wald ratio	1	0.014069	0.131306	0.914676	1.014168	0.784043	1.311837	0.998644
hematopoietic	Coagulation defects	Wald ratio	1	-0.0469	0.450193	0.917037	0.954188	0.394841	2.305927	0.998644
digestive	Esophageal bleeding (varices/hemorrhage)	Wald ratio	1	-0.0347	0.337645	0.918139	0.965893	0.498334	1.872138	0.998644
injuries & poisoni	Opiates and related narcotics causing adverse effects in therapeutic use	Wald ratio	1	-0.04221	0.412677	0.91854	0.958673	0.426966	2.152523	0.998644
pregnancy complic	Hypertension complicating pregnancy, childbirth, and the puerperium	Wald ratio	1	0.043143	0.422056	0.91858	1.044088	0.456537	2.3878	0.998644
hematopoietic	Purpura and other hemorrhagic conditions	Wald ratio	1	0.032827	0.328266	0.920344	1.033371	0.543039	1.966444	0.998644
injuries & poisoni	Complication of colostomy or enterostomy	Wald ratio	1	0.061902	0.619015	0.920344	1.063858	0.316204	3.579313	0.998644
injuries & poisoni	Fracture of ribs	Wald ratio	1	0.048771	0.497088	0.921842	1.04998	0.396325	2.781702	0.998644
genitourinary	Noninflammatory disorders of vulva and perineum	Wald ratio	1	0.036578	0.375161	0.922329	1.037255	0.497213	2.163858	0.998644
mental disorders	Altered mental status	Wald ratio	1	0.028137	0.300128	0.925308	1.028537	0.571143	1.852227	0.998644
injuries & poisoni	Dislocation	Wald ratio	1	-0.02814	0.300128	0.925308	0.972255	0.539891	1.750873	0.998644
neoplasms	Secondary malignant neoplasm	Wald ratio	1	0.013131	0.140685	0.925639	1.013217	0.76904	1.334923	0.998644
musculoskeletal	Acquired foot deformities	Wald ratio	1	0.013131	0.140685	0.925639	1.013217	0.76904	1.334923	0.998644
circulatory system	Atrial fibrillation and flutter	Wald ratio	1	0.011255	0.121927	0.926454	1.011318	0.796345	1.284324	0.998644
respiratory	Abnormal sputum	Wald ratio	1	0.027199	0.300128	0.927791	1.027572	0.570608	1.850491	0.998644
circulatory system	Other venous embolism and thrombosis	Wald ratio	1	-0.05252	0.581499	0.928031	0.948833	0.303534	2.966004	0.998644
neoplasms	Secondary malignant neoplasm of liver	Wald ratio	1	-0.02439	0.271991	0.928561	0.975909	0.572645	1.663157	0.998644
circulatory system	Ill-defined descriptions and complications of heart disease	Wald ratio	1	-0.04314	0.487709	0.92951	0.957774	0.368228	2.491203	0.998644
respiratory	Bronchitis	Wald ratio	1	0.048771	0.553362	0.929769	1.04998	0.354936	3.106076	0.998644
genitourinary	Postmenopausal bleeding	Wald ratio	1	0.013131	0.150064	0.930274	1.013217	0.755032	1.35969	0.998644
musculoskeletal	Osteomyelitis	Wald ratio	1	-0.04971	0.581499	0.931877	0.951506	0.30439	2.974361	0.998644
neoplasms	Secondary malignant neoplasm of digestive systems	Wald ratio	1	0.027199	0.356403	0.939168	1.027572	0.511018	2.066277	0.998644
neoplasms	Cervical cancer and dysplasia	Wald ratio	1	-0.01782	0.234475	0.939419	0.982338	0.620399	1.555429	0.998644
circulatory system	Phlebitis and thrombophlebitis	Wald ratio	1	-0.01688	0.225096	0.940215	0.983259	0.632502	1.52853	0.998644
genitourinary	Endometrial hyperplasia	Wald ratio	1	0.030951	0.412677	0.940215	1.031435	0.459372	2.315897	0.998644
neoplasms	Nevus, non-neoplastic	Wald ratio	1	0.042206	0.57212	0.941193	1.043109	0.339884	3.201312	0.998644
circulatory system	Polyarteritis nodosa and allied conditions	Wald ratio	1	-0.03376	0.47833	0.943725	0.966799	0.378594	2.468873	0.998644
dermatologic	Cellulitis and abscess of arm/hand	Wald ratio	1	0.013131	0.18758	0.944194	1.013217	0.701505	1.463437	0.998644
sense organs	Primary open angle glaucoma	Wald ratio	1	0.030013	0.431435	0.94454	1.030468	0.442374	2.400375	0.998644
dermatologic	Rash and other nonspecific skin eruption	Wald ratio	1	0.020634	0.300128	0.945189	1.020848	0.566874	1.838382	0.998644
mental disorders	Psychogenic and somatoform disorders	Wald ratio	1	0.04033	0.600257	0.946432	1.041154	0.321045	3.376479	0.998644
hematopoietic	Pernicious anemia	Wald ratio	1	-0.03376	0.506467	0.946847	0.966799	0.35828	2.608853	0.998644
musculoskeletal	Osteomyelitis, periostitis, and other infections involving bone	Wald ratio	1	0.037516	0.562741	0.946847	1.038229	0.344571	3.128295	0.998644
sense organs	Epiphora	Wald ratio	1	-0.03001	0.459572	0.94793	0.970433	0.394249	2.388696	0.998644
endocrine/metabol	Disorders of fluid, electrolyte, and acid-base balance	Wald ratio	1	0.010317	0.159443	0.948408	1.01037	0.739196	1.381025	0.998644
hematopoietic	Iron deficiency anemias, unspecified or not due to blood loss	Wald ratio	1	0.010317	0.159443	0.948408	1.01037	0.739196	1.381025	0.998644
sense organs	Ectropion or entropion	Wald ratio	1	-0.02439	0.422056	0.953926	0.975909	0.426725	2.231879	0.998644
circulatory system	Carditis	Wald ratio	1	-0.01688	0.309508	0.956501	0.983259	0.536056	1.803542	0.998644
digestive	Diseases of pulp and periapical tissues	Wald ratio	1	-0.01782	0.328266	0.956708	0.982338	0.516221	1.86933	0.998644
circulatory system	Abdominal aortic aneurysm	Wald ratio	1	-0.02532	0.468951	0.956935	0.974995	0.388887	2.44445	0.998644
sense organs	Visual disturbances	Wald ratio	1	-0.01313	0.243854	0.957058	0.986955	0.611962	1.591734	0.998644
digestive	Diverticulosis	Wald ratio	1	-0.00478	0.090976	0.958069	0.995228	0.832687	1.189497	0.998644
infectious diseases	Streptococcus infection	Wald ratio	1	-0.01688	0.337645	0.960122	0.983259	0.507293	1.905799	0.998644
sense organs	Other disorders of eye	Wald ratio	1	-0.01125	0.225096	0.960122	0.988808	0.636072	1.537156	0.998644
pregnancy complic	Obstetrical/birth trauma	Wald ratio	1	0.010317	0.215717	0.961855	1.01037	0.662	1.542066	0.998644
pregnancy complic	Normal delivery	Wald ratio	1	-0.01501	0.328266	0.963538	0.985106	0.517675	1.874598	0.998644
pregnancy complic	Early or threatened labor; hemorrhage in early pregnancy	Wald ratio	1	-0.01219	0.28137	0.965436	0.987881	0.569112	1.714795	0.998644
pregnancy complic	Antepartum hemorrhage, abruptio placentae, and placenta previa	Wald ratio	1	0.020634	0.47833	0.965592	1.020848	0.399759	2.606896	0.998644
genitourinary	Genital prolapse	Wald ratio	1	-0.00544	0.131306	0.966954	0.994575	0.768896	1.286494	0.998644
mental disorders	Alcoholic liver damage	Wald ratio	1	0.019696	0.487709	0.967786	1.019891	0.39211	2.652772	0.998644
sense organs	Corneal opacity and other disorders of cornea	Wald ratio	1	0.020634	0.515846	0.968093	1.020848	0.371419	2.805808	0.998644
endocrine/metabol	Gout and other crystal arthropathies	Wald ratio	1	0.008254	0.225096	0.970751	1.008288	0.648602	1.567438	0.998644
sense organs	Disorders of lacrimal system	Wald ratio	1	-0.01032	0.300128	0.972578	0.989736	0.549598	1.782354	0.998644
genitourinary	Prolapse of vaginal walls	Wald ratio	1	0.005627	0.168822	0.973409	1.005643	0.722336	1.400066	0.998644
digestive	Diseases of pancreas	Wald ratio	1	0.008816	0.271991	0.974142	1.008855	0.591977	1.719304	0.998644
injuries & poisoni	Anaphylactic shock NOS	Wald ratio	1	0.018758	0.590878	0.974675	1.018935	0.320023	3.244233	0.998644
musculoskeletal	Joint effusions	Wald ratio	1	0.010317	0.365782	0.977499	1.01037	0.493311	2.06938	0.998644
dermatologic	Cellulitis and abscess of foot, toe	Wald ratio	1	-0.00478	0.18758	0.979656	0.995228	0.689051	1.437455	0.998644
mental disorders	Memory loss	Wald ratio	1	-0.01313	0.525225	0.980055	0.986955	0.352547	2.762981	0.998644
symptoms	Symptoms involving nervous and musculoskeletal systems	Wald ratio	1	0.007972	0.318887	0.980055	1.008004	0.539536	1.883233	0.998644
respiratory	Chronic bronchitis	Wald ratio	1	0.006472	0.262612	0.98034	1.006493	0.601548	1.684034	0.998644
pregnancy complic	Known or suspected fetal abnormality affecting management of mother	Wald ratio	1	0.005721	0.234475	0.980534	1.005738	0.635178	1.592481	0.998644
endocrine/metabol	Type 2 diabetes with neurological manifestations	Wald ratio	1	0.014069	0.581499	0.980698	1.014168	0.324435	3.170237	0.998644
injuries & poisoni	Poisoning by psychotropic agents	Wald ratio	1	-0.00769	0.318887	0.980759	0.992339	0.531151	1.853965	0.998644
infectious diseases	Gram negative septicemia	Wald ratio	1	-0.01125	0.47833	0.981228	0.988808	0.387213	2.525077	0.998644
genitourinary	Renal failure NOS	Wald ratio	1	-0.00853	0.365782	0.981384	0.991501	0.484099	2.030733	0.998644
genitourinary	Uterine/Uterovaginal prolapse	Wald ratio	1	0.004596	0.196959	0.981384	1.004606	0.682874	1.47792	0.998644
musculoskeletal	Ankylosing spondylitis	Wald ratio	1	-0.01219	0.553362	0.982421	0.987881	0.333944	2.922375	0.998644
musculoskeletal	Other acquired deformities of limbs	Wald ratio	1	-0.00732	0.337645	0.982714	0.992711	0.51217	1.924118	0.998644
circulatory system	Myocardial infarction	Wald ratio	1	-0.00253	0.131306	0.984613	0.997471	0.771134	1.290239	0.998644
circulatory system	Bundle branch block	Wald ratio	1	-0.00469	0.243854	0.984657	0.995321	0.617149	1.605227	0.998644
digestive	Diverticulosis and diverticulitis	Wald ratio	1	-0.00159	0.090976	0.986017	0.998407	0.835347	1.193296	0.998644
digestive	Abnormal results of function study of liver	Wald ratio	1	-0.00385	0.234475	0.986915	0.996162	0.62913	1.577319	0.998644

injuries & poisoni	Superficial injury without mention of infection	Wald ratio	1	-0.00347	0.215717	0.987165	0.996536	0.652936	1.520951	0.998644
circulatory system	Cardiac conduction disorders	Wald ratio	1	-0.00216	0.168822	0.989805	0.997845	0.716735	1.389209	0.998752
digestive	Peritoneal adhesions (postoperative) (postinfection)	Wald ratio	1	-0.00281	0.243854	0.990794	0.99719	0.618308	1.608241	0.998752
digestive	Diseases of esophagus	Wald ratio	1	-0.00081	0.079722	0.991927	0.999194	0.854652	1.168181	0.998752
neoplasms	Malignant neoplasm of uterus	Wald ratio	1	-0.00356	0.38454	0.992605	0.996442	0.468949	2.117283	0.998752
circulatory system	Other chronic ischemic heart disease, unspecified	Wald ratio	1	-0.00094	0.121927	0.993862	0.999063	0.786695	1.268759	0.998752
circulatory system	Ischemic Heart Disease	Wald ratio	1	0.000544	0.087225	0.995024	1.000544	0.843313	1.18709	0.998752
circulatory system	Pericarditis	Wald ratio	1	-0.00188	0.393919	0.996201	0.998126	0.461185	2.160208	0.998752
circulatory system	First degree AV block	Wald ratio	1	-0.00063	0.440814	0.998863	0.999372	0.42121	2.37113	0.999592
respiratory	Voice disturbance	Wald ratio	1	-0.00022	0.422056	0.999592	0.999784	0.437165	2.28648	0.999592

Table S15. The phenome-wide MR analysis results of THSD1 (IVW or Wald ratio methods).

Category	Phenostring	Method	Nsnp	beta	se	P value	OR	LL	LR	FDR
neoplasms	Cancer of stomach	Wald ratio	1	1.431629	0.443805	0.001256	4.185512	1.753776	9.989023	0.510617
injuries & poisonings	Skull and face fracture and other intercranial injury	Wald ratio	1	-0.60844	0.19327	0.001643	0.544198	0.372599	0.794825	0.510617
pregnancy complications	Complications of labor and delivery NEC	Wald ratio	1	0.687182	0.221902	0.001956	1.988105	1.286922	3.07133	0.510617
dermatologic	Sicca syndrome	Wald ratio	1	-1.28847	0.465279	0.005619	0.275693	0.110757	0.686246	0.911909
circulatory system	Congestive heart failure (CHF) NOS	Wald ratio	1	-0.65139	0.236219	0.005823	0.52132	0.328119	0.828281	0.911909
genitourinary	Cystitis	Wald ratio	1	-0.50823	0.19327	0.008548	0.60156	0.411874	0.878606	0.922563
circulatory system	Subarachnoid hemorrhage	Wald ratio	1	0.930559	0.365065	0.010803	2.535926	1.2399	5.186645	0.922563
dermatologic	Actinic keratosis	Wald ratio	1	0.522545	0.207586	0.011828	1.686313	1.122632	2.533023	0.922563
sense organs	Otitis externa	Wald ratio	1	1.145303	0.458121	0.012419	3.143394	1.280673	7.71542	0.922563
genitourinary	Cystitis and urethritis	Wald ratio	1	-0.46528	0.186112	0.012419	0.62796	0.436024	0.904385	0.922563
respiratory	Pulmonary collapse; interstitial and compensatory emphysema	Wald ratio	1	0.586968	0.236219	0.012961	1.798527	1.131992	2.857527	0.922563
respiratory	Pleurisy; pleural effusion	Wald ratio	1	0.3078	0.128847	0.016899	1.360429	1.056816	1.751267	0.998587
genitourinary	Vaginal enterocele, congenital or acquired	Wald ratio	1	0.930559	0.400856	0.020264	2.535926	1.155902	5.563552	0.998587
dermatologic	Carbuncle and furuncle	Wald ratio	1	-0.48675	0.214744	0.023411	0.614618	0.40347	0.936267	0.998587
digestive	Hemorrhage from gastrointestinal ulcer	Wald ratio	1	-0.93056	0.422331	0.027567	0.394333	0.172333	0.902316	0.998587
genitourinary	Prolapse of vaginal vault after hysterectomy	Wald ratio	1	1.00214	0.458121	0.028706	2.724106	1.109847	6.686282	0.998587
respiratory	Pneumococcal pneumonia	Wald ratio	1	0.293484	0.136005	0.030936	1.341092	1.02728	1.750766	0.998587
genitourinary	Cyst of kidney, acquired	Wald ratio	1	0.629917	0.293484	0.031846	1.877454	1.056211	3.337244	0.998587
endocrine/metabolic	Obesity	Wald ratio	1	0.229061	0.107372	0.032897	1.257418	1.018785	1.551947	0.998587
sense organs	Disorders of external ear	Wald ratio	1	0.57981	0.27201	0.033041	1.785699	1.047777	3.043318	0.998587
circulatory system	Congestive heart failure; nonhypertensive	Wald ratio	1	-0.30064	0.143163	0.035729	0.740343	0.559204	0.980157	0.998587
endocrine/metabolic	Overweight, obesity and other hyperalimentation	Wald ratio	1	0.207586	0.100214	0.038319	1.230704	1.011229	1.497812	0.998587
respiratory	Acute tonsillitis	Wald ratio	1	-0.85898	0.422331	0.041962	0.423595	0.185121	0.969272	0.998587
digestive	Disturbances in tooth eruption	Wald ratio	1	-0.42949	0.214744	0.0455	0.650842	0.427249	0.991447	0.998587
digestive	Disorders of tooth development	Wald ratio	1	-0.41517	0.207586	0.0455	0.660226	0.439533	0.991731	0.998587
digestive	Diseases of the oral soft tissues, excluding lesions specific for gingiva and t	Wald ratio	1	0.329275	0.164637	0.0455	1.38996	1.006607	1.919306	0.998587
dermatologic	Degenerative skin conditions and other dermatoses	Wald ratio	1	0.286326	0.143163	0.0455	1.331526	1.005743	1.762838	0.998587
musculoskeletal	Other acquired musculoskeletal deformity	Wald ratio	1	-0.47244	0.236219	0.0455	0.623481	0.392419	0.990596	0.998587
congenital anomalies	Digestive congenital anomalies	Wald ratio	1	0.787396	0.393698	0.0455	2.197666	1.015873	4.754274	0.998587
congenital anomalies	Other congenital musculoskeletal anomalies	Wald ratio	1	-0.85898	0.429489	0.0455	0.423595	0.182542	0.982967	0.998587
symptoms	Chronic fatigue syndrome	Wald ratio	1	-0.85898	0.429489	0.0455	0.423595	0.182542	0.982967	0.998587
injuries & poisonings	Fracture of ankle and foot	Wald ratio	1	0.422331	0.214744	0.049222	1.525513	1.001433	2.32386	0.998587
infectious diseases	Viral infection	Wald ratio	1	0.393698	0.200428	0.049497	1.482453	1.000859	2.195779	0.998587
sense organs	Hearing loss	Wald ratio	1	0.3078	0.157479	0.050637	1.360429	0.999141	1.852358	0.998587
respiratory	Acute and chronic tonsillitis	Wald ratio	1	-0.45812	0.236219	0.052453	0.632471	0.398077	1.004879	0.998587
neurological	Facial nerve disorders [CN7]	Wald ratio	1	0.672866	0.350749	0.055064	1.959845	0.985503	3.897494	0.998587
neoplasms	Secondary malignancy of respiratory organs	Wald ratio	1	-0.42233	0.221902	0.057011	0.655517	0.424323	1.012678	0.998587
sense organs	Tinnitus	Wald ratio	1	-0.85898	0.458121	0.060793	0.423595	0.17258	1.039708	0.998587
circulatory system	Intracranial hemorrhage	Wald ratio	1	0.465279	0.250535	0.063291	1.592459	0.97456	2.602124	0.998587
musculoskeletal	Ganglion and cyst of synovium, tendon, and bursa	Wald ratio	1	-0.34359	0.186112	0.06487	0.709219	0.492446	1.021414	0.998587
circulatory system	Hypotension NOS	Wald ratio	1	0.329275	0.178954	0.065768	1.38996	0.978754	1.973925	0.998587
neurological	Abnormal involuntary movements	Wald ratio	1	-0.64423	0.350749	0.066249	0.525065	0.264028	1.044183	0.998587
digestive	Cholelithiasis and cholecystitis	Wald ratio	1	-0.15748	0.085898	0.066753	0.854295	0.721922	1.01094	0.998587
pregnancy complications	Problems associated with amniotic cavity and membranes	Wald ratio	1	0.522545	0.286326	0.068001	1.686313	0.962084	2.955723	0.998587
musculoskeletal	Other disorders of synovium, tendon, and bursa	Wald ratio	1	-0.2219	0.121688	0.068223	0.800993	0.631024	1.016746	0.998587
infectious diseases	Bacterial enteritis	Wald ratio	1	0.365065	0.200428	0.068542	1.440608	0.972609	2.1338	0.998587
digestive	Other disorders of stomach and duodenum	Wald ratio	1	0.322117	0.178954	0.071861	1.380046	0.971773	1.959845	0.998587
musculoskeletal	Fasciitis	Wald ratio	1	-0.3078	0.171795	0.073186	0.735062	0.524915	1.029341	0.998587
circulatory system	Endocarditis	Wald ratio	1	-0.71581	0.400856	0.074146	0.488794	0.222797	1.072362	0.998587
respiratory	Other upper respiratory disease	Wald ratio	1	-0.29348	0.164637	0.07465	0.745661	0.540007	1.029636	0.998587
digestive	Appendiceal conditions	Wald ratio	1	-0.31496	0.178954	0.078408	0.729819	0.51391	1.036439	0.998587
musculoskeletal	Other specified osteoporosis	Wald ratio	1	0.787396	0.450963	0.080805	2.197666	0.908017	5.318991	0.998587
genitourinary	Pain and other symptoms associated with female genital organs	Wald ratio	1	0.336433	0.19327	0.081729	1.399945	0.958509	2.044681	0.998587
respiratory	Bacterial pneumonia	Wald ratio	1	0.221902	0.128847	0.085029	1.24845	0.969828	1.607117	0.998587
genitourinary	Calculus of kidney	Wald ratio	1	-0.31496	0.186112	0.090587	0.729819	0.50675	1.051083	0.998587
circulatory system	Precordial pain	Wald ratio	1	-0.30064	0.178954	0.092957	0.740343	0.52132	1.051384	0.998587
musculoskeletal	Contracture of palmar fascia [Dupuytren's disease]	Wald ratio	1	-0.30064	0.178954	0.092957	0.740343	0.52132	1.051384	0.998587
symptoms	Edema	Wald ratio	1	-0.42949	0.257693	0.095581	0.650842	0.392756	1.07852	0.998587
respiratory	Pneumonia	Wald ratio	1	0.178954	0.107372	0.095581	1.195965	0.968995	1.476099	0.998587
neoplasms	Carcinoma in situ of skin	Wald ratio	1	0.680024	0.408014	0.095581	1.973925	0.887201	4.391767	0.998587
hematopoietic	Lymphadenitis	Wald ratio	1	0.343591	0.207586	0.09789	1.410002	0.938682	2.117974	0.998587
injuries & poisonings	Burns	Wald ratio	1	-0.70866	0.429489	0.098943	0.492305	0.212152	1.142412	0.998587
injuries & poisonings	Hemorrhage or hematoma complicating a procedure	Wald ratio	1	-0.23622	0.143163	0.098943	0.789608	0.596415	1.04538	0.998587
musculoskeletal	Acquired spondylolisthesis	Wald ratio	1	-0.43665	0.264851	0.09922	0.6462	0.384522	1.085957	0.998587
mental disorders	Depression	Wald ratio	1	-0.16464	0.100214	0.100412	0.848201	0.696939	1.032293	0.998587
digestive	Other diseases of the teeth and supporting structures	Wald ratio	1	-0.32927	0.200428	0.100412	0.719445	0.485725	1.065628	0.998587
sense organs	Infection of the eye	Wald ratio	1	0.715814	0.436647	0.101142	2.045852	0.869347	4.814549	0.998587
genitourinary	Acute renal failure	Wald ratio	1	0.257693	0.157479	0.101764	1.293942	0.950311	1.761829	0.998587
circulatory system	Other forms of chronic heart disease	Wald ratio	1	0.408014	0.250535	0.103404	1.503829	0.920319	2.4573	0.998587
digestive	Cholelithiasis	Wald ratio	1	-0.15032	0.093056	0.106227	0.860432	0.716978	1.032588	0.998587
neoplasms	Benign neoplasm of other endocrine glands and related structures	Wald ratio	1	0.565493	0.350749	0.106909	1.760316	0.885171	3.500696	0.998587
genitourinary	Hydronephrosis	Wald ratio	1	-0.37938	0.236219	0.108261	0.684284	0.430688	1.087202	0.998587
neoplasms	Secondary malignant neoplasm	Wald ratio	1	-0.1718	0.107372	0.109599	0.842151	0.682328	1.039411	0.998587
mental disorders	Bipolar	Wald ratio	1	0.515386	0.322117	0.109599	1.674285	0.890509	3.147898	0.998587
neurological	Multiple sclerosis	Wald ratio	1	-0.45812	0.286326	0.109599	0.632471	0.36084	1.108577	0.998587
digestive	Femoral hernia	Wald ratio	1	0.651391	0.408014	0.110379	1.918208	0.862158	4.267803	0.998587
neoplasms	Benign neoplasm of skin	Wald ratio	1	0.19327	0.121688	0.112233	1.21321	0.955769	1.539995	0.998587
neoplasms	Hemangioma and lymphangioma, any site	Wald ratio	1	-0.40801	0.257693	0.113346	0.664969	0.401281	1.101931	0.998587
digestive	Noninfectious gastroenteritis	Wald ratio	1	-0.136	0.085898	0.113346	0.872838	0.737592	1.032884	0.998587
sense organs	Disorders of refraction and accommodation; blindness and low vision	Wald ratio	1	-0.33643	0.214744	0.117193	0.714314	0.468916	1.088136	0.998587
injuries & poisonings	Open wounds of extremities	Wald ratio	1	-0.25769	0.164637	0.117532	0.772832	0.559684	1.067155	0.998587
genitourinary	Renal failure	Wald ratio	1	0.200428	0.128847	0.119814	1.221926	0.949223	1.572973	0.998587
genitourinary	Noninflammatory disorders of cervix	Wald ratio	1	0.300642	0.19327	0.119814	1.350726	0.92481	1.972795	0.998587
genitourinary	Infertility, female	Wald ratio	1	0.443805	0.286326	0.121142	1.558626	0.889235	2.731917	0.998587
mental disorders	Tobacco use disorder	Wald ratio	1	-0.12169	0.07874	0.122236	0.885424	0.758799	1.03318	0.998587
circulatory system	Pulmonary heart disease	Wald ratio	1	-0.24338	0.157479	0.122236	0.783976	0.575776	1.06746	0.998587
dermatologic	Dyschromia and Vitiligo	Wald ratio	1	0.508228	0.329275	0.122715	1.662343	0.871839	3.169604	0.998587
genitourinary	Lump or mass in breast	Wald ratio	1	-0.40801	0.264851	0.123429	0.664969	0.395691	1.1175	0.998587
hematopoietic	Other diseases of blood and blood-forming organs	Wald ratio	1	0.250535	0.164637	0.128074	1.284713	0.930388	1.773978	0.998587
infectious diseases	Gram negative septicemia	Wald ratio	1	-0.55118	0.365065	0.131093	0.576271	0.281758	1.178628	0.998587
circulatory system	Aneurysm and dissection of heart	Wald ratio	1	0.594126	0.393698	0.131276	1.811447	0.837343	3.918755	0.998587
digestive	Diseases of pulp and periapical tissues	Wald ratio	1	-0.36507	0.243377	0.133614	0.694151	0.430812	1.118461	0.998587
digestive	Periapical abscess	Wald ratio	1	-0.45812	0.3078	0.136653	0.632471	0.345968	1.156233	0.998587
genitourinary	Abnormal findings on mammogram or breast exam	Wald ratio	1	-0.3937	0.264851	0.13715	0.674558	0.401396	1.133614	0.998587
dermatologic	Chronic ulcer of skin	Wald ratio	1	-0.35075	0.236219	0.137584	0.70416	0.443198	1.118781	0.998587
endocrine/metabolic	Chondrocalcinosis	Wald ratio	1	0.658549	0.443805	0.137843	1.931988	0.809524	4.610826	0.998587
neoplasms	Secondary malignant neoplasm of liver	Wald ratio	1	-0.3078	0.207586	0.138139	0.735062	0.489354	1.104142	0.998587
pregnancy complications	Miscarriage; stillbirth	Wald ratio	1	0.221902	0.150321	0.139893	1.24845	0.929855	1.676204	0.998587

musculoskeletal	Spondylosis without myelopathy	Wald ratio	1	0.221902	0.150321	0.139893	1.24845	0.929855	1.676204	0.998587
sense organs	Vertiginous syndromes and other disorders of vestibular system	Wald ratio	1	0.200428	0.136005	0.140567	1.221926	0.935999	1.595197	0.998587
neoplasms	Malignant neoplasm of rectum, rectosigmoid junction, and anus	Wald ratio	1	-0.33643	0.229061	0.141901	0.714314	0.455941	1.119101	0.998587
digestive	Constipation	Wald ratio	1	-0.15748	0.107372	0.142467	0.854295	0.692167	1.054398	0.998587
digestive	Liver abscess and sequelae of chronic liver disease	Wald ratio	1	-0.50107	0.343591	0.144749	0.605882	0.308971	1.188115	0.998587
sense organs	Ptosis of eyelid	Wald ratio	1	-0.35075	0.243377	0.149535	0.70416	0.437024	1.134588	0.998587
infectious diseases	Viral Enteritis	Wald ratio	1	0.515386	0.357907	0.149867	1.674285	0.830181	3.376652	0.998587
digestive	Acute periodontitis	Wald ratio	1	-0.58697	0.408014	0.150265	0.556011	0.249905	1.237063	0.998587
symptoms	Symptoms of the muscles	Wald ratio	1	0.565493	0.393698	0.150899	1.760316	0.813707	3.808142	0.998587
sense organs	Disorders of vitreous body	Wald ratio	1	-0.40086	0.279168	0.151032	0.669746	0.387506	1.157558	0.998587
pregnancy complications	Hemorrhage in early pregnancy	Wald ratio	1	0.458121	0.322117	0.154962	1.581101	0.840947	2.972697	0.998587
symptoms	Other ill-defined and unknown causes of morbidity and mortality	Wald ratio	1	0.121688	0.085898	0.15658	1.129402	0.954401	1.336492	0.998587
digestive	Symptoms involving digestive system	Wald ratio	1	-0.12169	0.085898	0.15658	0.885424	0.748228	1.047777	0.998587
congenital anomalies	Genitourinary congenital anomalies	Wald ratio	1	0.365065	0.257693	0.15658	1.440608	0.869347	2.387255	0.998587
injuries & poisonings	Complications of surgical and medical procedures	Wald ratio	1	0.150321	0.107372	0.161513	1.162207	0.941644	1.434434	0.998587
respiratory	Chronic airway obstruction	Wald ratio	1	0.150321	0.107372	0.161513	1.162207	0.941644	1.434434	0.998587
musculoskeletal	Acquired foot deformities	Wald ratio	1	0.150321	0.107372	0.161513	1.162207	0.941644	1.434434	0.998587
symptoms	Sciatica	Wald ratio	1	-0.30064	0.214744	0.161513	0.740343	0.486003	1.127786	0.998587
injuries & poisonings	Adverse effects of sedatives or other central nervous system depressants anc	Wald ratio	1	-0.60128	0.429489	0.161513	0.548107	0.236199	1.271902	0.998587
infectious diseases	E. coli	Wald ratio	1	0.279168	0.200428	0.163663	1.322029	0.892551	1.958163	0.998587
infectious diseases	Sepsis	Wald ratio	1	0.279168	0.200428	0.163663	1.322029	0.892551	1.958163	0.998587
infectious diseases	Sepsis and SIRS	Wald ratio	1	0.279168	0.200428	0.163663	1.322029	0.892551	1.958163	0.998587
endocrine/metabolic	Crystal arthropathies	Wald ratio	1	0.586968	0.422331	0.16458	1.798527	0.785999	4.115399	0.998587
symptoms	Nonspecific findings on examination of blood	Wald ratio	1	0.178954	0.128847	0.164867	1.195965	0.929056	1.539554	0.998587
digestive	Portal hypertension	Wald ratio	1	-0.62276	0.450963	0.167294	0.536463	0.221652	1.298395	0.998587
neoplasms	Malignant neoplasm of kidney, except pelvis	Wald ratio	1	0.450963	0.329275	0.170823	1.569823	0.823316	2.993195	0.998587
dermatologic	Other dyschromia	Wald ratio	1	0.508228	0.372224	0.172132	1.662343	0.801452	3.447973	0.998587
circulatory system	Nonspecific chest pain	Wald ratio	1	-0.0859	0.062992	0.172682	0.917688	0.811102	1.03828	0.998587
digestive	Diseases of the jaws	Wald ratio	1	-0.45812	0.336433	0.173292	0.632471	0.327087	1.222976	0.998587
endocrine/metabolic	Vitamin B-complex deficiencies	Wald ratio	1	0.515386	0.379382	0.174308	1.674285	0.795964	3.521808	0.998587
genitourinary	Other symptoms/disorders or the urinary system	Wald ratio	1	0.093056	0.068718	0.175683	1.097523	0.959223	1.255763	0.998587
genitourinary	Endometrial hyperplasia	Wald ratio	1	-0.41517	0.3078	0.177389	0.660226	0.36115	1.206974	0.998587
circulatory system	Abdominal aortic aneurysm	Wald ratio	1	0.472438	0.350749	0.178	1.603899	0.806517	3.189633	0.998587
digestive	Diseases of the salivary glands	Wald ratio	1	-0.5297	0.393698	0.178478	0.58878	0.272164	1.273725	0.998587
neurological	Abnormality of gait	Wald ratio	1	0.343591	0.257693	0.182422	1.410002	0.850877	2.336536	0.998587
genitourinary	Retention of urine	Wald ratio	1	0.171795	0.128847	0.182422	1.187435	0.92243	1.528573	0.998587
neoplasms	Cancer of mouth	Wald ratio	1	-0.54402	0.408014	0.182422	0.580411	0.260872	1.291351	0.998587
neoplasms	Cancer of kidney and renal pelvis	Wald ratio	1	0.429489	0.322117	0.182422	1.536472	0.81721	2.888788	0.998587
injuries & poisonings	Internal derangement of knee	Wald ratio	1	-0.11453	0.085898	0.182422	0.891785	0.753603	1.055304	0.998587
sense organs	Diplopia and disorders of binocular vision	Wald ratio	1	-0.51539	0.38654	0.182422	0.59727	0.279989	1.274089	0.998587
sense organs	Meniere's disease	Wald ratio	1	0.57981	0.436647	0.184221	1.785699	0.758799	4.202323	0.998587
symptoms	Other abnormal blood chemistry	Wald ratio	1	0.178954	0.136005	0.188245	1.195965	0.916113	1.561306	0.998587
injuries & poisonings	Fracture of tibia and fibula	Wald ratio	1	0.300642	0.229061	0.189351	1.350726	0.862158	2.116155	0.998587
digestive	Hemorrhage of rectum and anus	Wald ratio	1	-0.12169	0.093056	0.190978	0.885424	0.737803	1.062581	0.998587
injuries & poisonings	Torus fracture	Wald ratio	1	0.336433	0.257693	0.191704	1.399945	0.844808	2.319871	0.998587
digestive	Gastrointestinal hemorrhage	Wald ratio	1	-0.09306	0.071581	0.193601	0.911143	0.791872	1.048378	0.998587
respiratory	Chronic pharyngitis and nasopharyngitis	Wald ratio	1	-0.43665	0.336433	0.194331	0.6462	0.334187	1.249522	0.998587
genitourinary	Inflammatory disease of cervix, vagina, and vulva	Wald ratio	1	-0.25054	0.19327	0.194873	0.778384	0.532941	1.136864	0.998587
symptoms	Symptoms involving skin and other integumentary tissue	Wald ratio	1	-0.31496	0.243377	0.195625	0.729819	0.452948	1.175931	0.998587
injuries & poisonings	Fracture of foot	Wald ratio	1	0.314958	0.243377	0.195625	1.370202	0.85039	2.207757	0.998587
hematopoietic	Diseases of white blood cells	Wald ratio	1	0.221902	0.171795	0.196473	1.24845	0.89153	1.748261	0.998587
genitourinary	Chronic cystitis	Wald ratio	1	-0.45096	0.350749	0.198543	0.637014	0.320321	1.266814	0.998587
musculoskeletal	Synovitis and tenosynovitis	Wald ratio	1	-0.25769	0.200428	0.198543	0.772832	0.521768	1.144704	0.998587
dermatologic	Pilonidal cyst	Wald ratio	1	0.551177	0.429489	0.199375	1.735295	0.747799	4.026812	0.998587
genitourinary	Pyelonephritis	Wald ratio	1	0.365065	0.286326	0.202309	1.440608	0.821903	2.525058	0.998587
neoplasms	Cancer, suspected or other	Wald ratio	1	-0.10021	0.07874	0.203115	0.904644	0.77527	1.055607	0.998587
sense organs	Retinal detachments and defects	Wald ratio	1	-0.23622	0.186112	0.204359	0.789608	0.548264	1.13719	0.998587
mental disorders	Symptoms involving head and neck	Wald ratio	1	0.243377	0.19327	0.207937	1.275549	0.873338	1.862996	0.998587
dermatologic	Disorders of sweat glands	Wald ratio	1	-0.45812	0.365065	0.209514	0.632471	0.309236	1.293571	0.998587
infectious diseases	Candidiasis	Wald ratio	1	0.286326	0.229061	0.2113	1.331526	0.849903	2.086076	0.998587
neoplasms	Malignant neoplasm, other	Wald ratio	1	-0.10737	0.085898	0.2113	0.898191	0.759017	1.062886	0.998587
digestive	Ulceration of the lower GI tract	Wald ratio	1	-0.40086	0.322117	0.213336	0.669746	0.356221	1.25922	0.998587
respiratory	Abnormal findings examination of lungs	Wald ratio	1	0.257693	0.207586	0.214466	1.293942	0.861418	1.943639	0.998587
sense organs	Suppurative and unspecified otitis media	Wald ratio	1	0.443805	0.357907	0.214975	1.558626	0.772832	3.143394	0.998587
neoplasms	Secondary malignancy of bone	Wald ratio	1	-0.27201	0.221902	0.220272	0.761847	0.493152	1.176942	0.998587
circulatory system	Arterial embolism and thrombosis of lower extremity artery	Wald ratio	1	0.544019	0.443805	0.220272	1.722917	0.721922	4.111865	0.998587
musculoskeletal	Other inflammatory spondylopathies	Wald ratio	1	-0.31496	0.257693	0.221624	0.729819	0.440415	1.209395	0.998587
digestive	Chronic liver disease and cirrhosis	Wald ratio	1	-0.23622	0.19327	0.221624	0.789608	0.540626	1.153257	0.998587
circulatory system	Heart failure NOS	Wald ratio	1	-0.20043	0.164637	0.223455	0.81838	0.59267	1.130049	0.998587
digestive	Other disorders of intestine	Wald ratio	1	0.200428	0.164637	0.223455	1.221926	0.884917	1.687279	0.998587
digestive	Diseases and other conditions of the tongue	Wald ratio	1	0.365065	0.300642	0.224639	1.440608	0.799161	2.596914	0.998587
mental disorders	Anxiety disorder	Wald ratio	1	0.164637	0.136005	0.226077	1.178965	0.903091	1.539114	0.998587
genitourinary	Inflammatory diseases of female pelvic organs	Wald ratio	1	-0.16464	0.136005	0.226077	0.848201	0.649725	1.107308	0.998587
infectious diseases	Intestinal infection	Wald ratio	1	0.128847	0.107372	0.230139	1.137516	0.921638	1.403959	0.998587
sense organs	Cataract	Wald ratio	1	-0.09306	0.07874	0.237278	0.911143	0.78084	1.06319	0.998587
musculoskeletal	Disorders of muscle, ligament, and fascia	Wald ratio	1	-0.18611	0.157479	0.237278	0.830181	0.609711	1.130373	0.998587
congenital anomalies	Congenital anomalies of urinary system	Wald ratio	1	0.422331	0.357907	0.238	1.525513	0.756413	3.076611	0.998587
genitourinary	Prolapse of vaginal walls	Wald ratio	1	-0.14316	0.121688	0.239407	0.866613	0.682719	1.10004	0.998587
genitourinary	Endometriosis	Wald ratio	1	0.19327	0.164637	0.24043	1.21321	0.878606	1.675244	0.998587
dermatologic	Cellulitis and abscess of trunk	Wald ratio	1	0.493912	0.422331	0.242206	1.638714	0.716157	3.749715	0.998587
genitourinary	Urinary calculus	Wald ratio	1	-0.15032	0.128847	0.243345	0.860432	0.668405	1.107625	0.998587
digestive	Other biliary tract disease	Wald ratio	1	-0.20043	0.171795	0.243345	0.81838	0.584413	1.146015	0.998587
dermatologic	Disorder of skin and subcutaneous tissue NOS	Wald ratio	1	0.157479	0.136005	0.246907	1.170556	0.89665	1.528136	0.998587
neoplasms	Skin cancer	Wald ratio	1	0.107372	0.093056	0.248563	1.113349	0.927727	1.336109	0.998587
mental disorders	Mood disorders	Wald ratio	1	-0.10737	0.093056	0.248563	0.898191	0.748442	1.077903	0.998587
musculoskeletal	Joint effusions	Wald ratio	1	0.322117	0.279168	0.248563	1.380046	0.798475	2.385205	0.998587
genitourinary	Other abnormality of urination	Wald ratio	1	0.27201	0.236219	0.24952	1.312599	0.82615	2.085478	0.998587
sense organs	Myopia	Wald ratio	1	-0.33643	0.293484	0.251654	0.714314	0.401856	1.269719	0.998587
neoplasms	Other non-epithelial cancer of skin	Wald ratio	1	0.11453	0.100214	0.253098	1.121347	0.921374	1.364721	0.998587
digestive	Periodontitis (acute or chronic)	Wald ratio	1	-0.34359	0.300642	0.253098	0.709219	0.393431	1.278474	0.998587
congenital anomalies	Cardiac congenital anomalies	Wald ratio	1	0.236219	0.207586	0.255149	1.266451	0.843116	1.902346	0.998587
musculoskeletal	Rheumatoid arthritis	Wald ratio	1	-0.17895	0.157479	0.255804	0.836145	0.614091	1.138493	0.998587
musculoskeletal	Osteoarthrosis, generalized	Wald ratio	1	-0.48675	0.429489	0.257074	0.614618	0.264861	1.426243	0.998587
mental disorders	Swelling, mass, or lump in head and neck [Space-occupying lesion, intracra	Wald ratio	1	0.38654	0.343591	0.260589	1.471879	0.750588	2.886308	0.998587
circulatory system	Cardiac arrest	Wald ratio	1	0.38654	0.343591	0.260589	1.471879	0.750588	2.886308	0.998587
mental disorders	Delirium due to conditions classified elsewhere	Wald ratio	1	-0.45812	0.408014	0.261519	0.632471	0.28427	1.407179	0.998587
injuries & poisonings	Mechanical complication of unspecified genitourinary device, implant, and g	Wald ratio	1	-0.33643	0.300642	0.26312	0.714314	0.396257	1.287659	0.998587
hematopoietic	Iron deficiency anemias, unspecified or not due to blood loss	Wald ratio	1	-0.136	0.121688	0.263718	0.872838	0.687623	1.107942	0.998587
mental disorders	Anxiety disorders	Wald ratio	1	0.143163	0.128847	0.266521	1.153918	0.896393	1.485427	0.998587
neoplasms	Neoplasm of unspecified nature of digestive system	Wald ratio	1	-0.35791	0.322117	0.266521	0.699138	0.371853	1.31448	0.998587
respiratory	Chronic bronchitis	Wald ratio	1	-0.21474	0.19327	0.266521	0.806748	0.552361	1.178291	0.998587

neurological	Other paralytic syndromes	Wald ratio	1	0.450963	0.408014	0.269046	1.569823	0.705573	3.492686	0.998587
mental disorders	Other specified nonpsychotic and/or transient mental disorders	Wald ratio	1	-0.45812	0.415172	0.269833	0.632471	0.28031	1.42706	0.998587
circulatory system	Cardiomyopathy	Wald ratio	1	-0.32927	0.300642	0.273412	0.719445	0.399104	1.296909	0.998587
musculoskeletal	Acquired toe deformities	Wald ratio	1	0.164637	0.150321	0.273412	1.178965	0.878103	1.582913	0.998587
injuries & poisonings	Allergy/adverse effect of penicillin	Wald ratio	1	-0.09306	0.085898	0.27866	0.911143	0.769961	1.078212	0.998587
digestive	Ventral hernia	Wald ratio	1	-0.19327	0.178954	0.280142	0.824259	0.580411	1.170556	0.998587
genitourinary	Other disorders of the kidney and ureters	Wald ratio	1	0.19327	0.178954	0.280142	1.21321	0.854295	1.722917	0.998587
digestive	Appendicitis	Wald ratio	1	-0.20043	0.186112	0.281515	0.81838	0.568242	1.178628	0.998587
circulatory system	Varicose veins	Wald ratio	1	-0.10737	0.100214	0.283977	0.898191	0.738015	1.093132	0.998587
neoplasms	Benign neoplasm of unspecified sites	Wald ratio	1	0.229061	0.214744	0.286122	1.257418	0.82544	1.915463	0.998587
dermatologic	Cellulitis and abscess of fingers/toes	Wald ratio	1	0.465279	0.436647	0.286616	1.592459	0.676686	3.747568	0.998587
circulatory system	Transient cerebral ischemia	Wald ratio	1	0.243377	0.229061	0.288009	1.275549	0.814173	1.998378	0.998587
injuries & poisonings	Poisoning by analgesics, antipyretics, and antirheumatics	Wald ratio	1	-0.136	0.128847	0.291171	0.872838	0.678043	1.123596	0.998587
sense organs	Strabismus and other disorders of binocular eye movements	Wald ratio	1	-0.28633	0.27201	0.29251	0.751018	0.440668	1.27994	0.998587
endocrine/metabolic	Other disorders of metabolism	Wald ratio	1	-0.29348	0.279168	0.293129	0.745661	0.431429	1.288765	0.998587
neoplasms	Colorectal cancer	Wald ratio	1	-0.16464	0.157479	0.295813	0.848201	0.622945	1.154909	0.998587
neurological	Other cerebral degenerations	Wald ratio	1	0.329275	0.314958	0.295813	1.38996	0.749729	2.576915	0.998587
digestive	Intestinal obstruction without mention of hernia	Wald ratio	1	-0.1718	0.164637	0.296727	0.842151	0.609885	1.162873	0.998587
injuries & poisonings	Fracture of hand or wrist	Wald ratio	1	0.186112	0.178954	0.29834	1.204557	0.848201	1.710629	0.998587
congenital anomalies	Cardiac and circulatory congenital anomalies	Wald ratio	1	0.207586	0.200428	0.300335	1.230704	0.830894	1.822894	0.998587
pregnancy complications	Early onset of delivery	Wald ratio	1	0.443805	0.429489	0.301448	1.558626	0.671667	3.616848	0.998587
dermatologic	Scar conditions and fibrosis of skin	Wald ratio	1	0.221902	0.214744	0.301448	1.24845	0.819553	1.901801	0.998587
digestive	Ileostomy status	Wald ratio	1	-0.26485	0.257693	0.304054	0.76732	0.463045	1.271538	0.998587
injuries & poisonings	Fracture of unspecified bones	Wald ratio	1	0.293484	0.286326	0.305363	1.341092	0.765126	2.350628	0.998587
sense organs	Perforation of tympanic membrane	Wald ratio	1	-0.32927	0.322117	0.306676	0.719445	0.382654	1.352661	0.998587
genitourinary	Cystic mastopathy	Wald ratio	1	0.350749	0.343591	0.307333	1.420131	0.724199	2.784832	0.998587
neoplasms	Secondary malignancy of brain/spine	Wald ratio	1	-0.37222	0.365065	0.307914	0.6892	0.336973	1.409598	0.998587
hematopoietic	Anemia of chronic disease	Wald ratio	1	-0.3937	0.393698	0.317311	0.674558	0.311815	1.45929	0.998587
neurological	Other peripheral nerve disorders	Wald ratio	1	0.093056	0.093056	0.317311	1.097523	0.91454	1.317117	0.998587
circulatory system	Myocardial infarction	Wald ratio	1	0.100214	0.100214	0.317311	1.105407	0.908277	1.345322	0.998587
circulatory system	Varicose veins of lower extremity	Wald ratio	1	-0.10021	0.100214	0.317311	0.904644	0.743316	1.100985	0.998587
respiratory	Diseases of the larynx and vocal cords	Wald ratio	1	0.200428	0.200428	0.317311	1.221926	0.824968	1.809892	0.998587
digestive	Stricture and stenosis of esophagus	Wald ratio	1	0.243377	0.243377	0.317311	1.275549	0.791645	2.055246	0.998587
genitourinary	Excessive or frequent menstruation	Wald ratio	1	-0.10737	0.107372	0.317311	0.898191	0.727733	1.108577	0.998587
dermatologic	Psoriasis and related disorders	Wald ratio	1	-0.2219	0.221902	0.317311	0.800993	0.518492	1.237417	0.998587
musculoskeletal	Spondylosis and allied disorders	Wald ratio	1	0.121688	0.121688	0.317311	1.129402	0.889745	1.433613	0.998587
injuries & poisonings	Effects radiation NOS	Wald ratio	1	-0.18611	0.186112	0.317311	0.830181	0.576436	1.195623	0.998587
musculoskeletal	Curvature of spine	Wald ratio	1	0.300642	0.3078	0.328696	1.350726	0.73886	2.46929	0.998587
dermatologic	Diseases of nail, NOS	Wald ratio	1	0.286326	0.293484	0.329258	1.331526	0.749085	2.366837	0.998587
musculoskeletal	Other acquired deformities of limbs	Wald ratio	1	0.250535	0.257693	0.33094	1.284713	0.77527	2.128918	0.998587
endocrine/metabolic	Type 1 diabetes	Wald ratio	1	0.200428	0.207586	0.334286	1.221926	0.813474	1.835463	0.998587
pregnancy complications	Antepartum hemorrhage, abruptio placentae, and placenta previa	Wald ratio	1	0.350749	0.365065	0.336661	1.420131	0.69435	2.904546	0.998587
symptoms	Effects of other external causes	Wald ratio	1	-0.15748	0.164637	0.338809	0.854295	0.618679	1.179641	0.998587
digestive	Chronic pancreatitis	Wald ratio	1	0.436647	0.458121	0.340527	1.547509	0.630482	3.798342	0.998587
symptoms	Other symptoms	Wald ratio	1	0.286326	0.300642	0.340904	1.331526	0.738649	2.400277	0.998587
circulatory system	Aortic valve disease	Wald ratio	1	0.279168	0.293484	0.341493	1.322029	0.743742	2.349955	0.998587
neoplasms	Malignant neoplasm of other and ill-defined sites within the digestive organs	Wald ratio	1	-0.136	0.143163	0.342112	0.872838	0.659282	1.155571	0.998587
neoplasms	Secondary malignant neoplasm of digestive systems	Wald ratio	1	-0.25054	0.264851	0.344176	0.778384	0.463178	1.308097	0.998587
musculoskeletal	Other unspecified back disorders	Wald ratio	1	0.243377	0.257693	0.344943	1.275549	0.769741	2.113733	0.998587
musculoskeletal	Hallux valgus (Bunion)	Wald ratio	1	0.121688	0.128847	0.344943	1.129402	0.877349	1.453868	0.998587
genitourinary	Noninflammatory disorders of ovary, fallopian tube, and broad ligament	Wald ratio	1	0.365065	0.38654	0.344943	1.440608	0.675331	3.07309	0.998587
digestive	Other specified gastritis	Wald ratio	1	0.11453	0.121688	0.346614	1.121347	0.883398	1.423388	0.998587
circulatory system	Cardiac pacemaker in situ	Wald ratio	1	-0.20759	0.221902	0.349539	0.812543	0.525968	1.25526	0.998587
circulatory system	Tachycardia NOS	Wald ratio	1	0.207586	0.221902	0.349539	1.230704	0.796648	1.901257	0.998587
respiratory	Chronic tonsillitis and adenoiditis	Wald ratio	1	-0.28633	0.3078	0.352251	0.751018	0.410814	1.372951	0.998587
respiratory	Obstructive chronic bronchitis	Wald ratio	1	-0.18611	0.200428	0.353111	0.830181	0.560486	1.229647	0.998587
circulatory system	Cerebral ischemia	Wald ratio	1	0.178954	0.19327	0.354484	1.195965	0.818849	1.74676	0.998587
injuries & poisonings	Other open wound of head and face	Wald ratio	1	0.178954	0.19327	0.354484	1.195965	0.818849	1.74676	0.998587
circulatory system	Heart valve replaced	Wald ratio	1	0.250535	0.27201	0.357023	1.284713	0.753819	2.189501	0.998587
musculoskeletal	Kyphoscoliosis and scoliosis	Wald ratio	1	0.293484	0.322117	0.362237	1.341092	0.713292	2.521445	0.998587
digestive	Functional digestive disorders	Wald ratio	1	-0.06514	0.071581	0.362823	0.936937	0.81429	1.078057	0.998587
circulatory system	Primary/intrinsic cardiomyopathies	Wald ratio	1	-0.27201	0.300642	0.365592	0.761847	0.422626	1.373344	0.998587
endocrine/metabolic	Type 2 diabetes	Wald ratio	1	0.070866	0.07874	0.36812	1.073437	0.919924	1.252567	0.998587
dermatologic	Diseases of hair and hair follicles	Wald ratio	1	-0.12885	0.143163	0.36812	0.879109	0.664018	1.163872	0.998587
musculoskeletal	Degeneration of intervertebral disc	Wald ratio	1	-0.17895	0.200428	0.371934	0.836145	0.564513	1.238481	0.998587
circulatory system	Ischemic Heart Disease	Wald ratio	1	0.058697	0.065855	0.372766	1.060454	0.932041	1.206559	0.998587
infectious diseases	Streptococcus infection	Wald ratio	1	-0.22906	0.257693	0.374063	0.79528	0.479918	1.317872	0.998587
neoplasms	Cancer of brain and nervous system	Wald ratio	1	-0.40086	0.450963	0.374063	0.669746	0.276721	1.620981	0.998587
infectious diseases	Staphylococcus infections	Wald ratio	1	-0.16464	0.186112	0.376364	0.848201	0.588949	1.221566	0.998587
digestive	Other symptoms involving abdomen and pelvis	Wald ratio	1	0.164637	0.186112	0.376364	1.178965	0.818615	1.697941	0.998587
circulatory system	Other acute and subacute forms of ischemic heart disease	Wald ratio	1	0.27201	0.3078	0.376847	1.312599	0.718005	2.39959	0.998587
circulatory system	Atherosclerosis of the extremities	Wald ratio	1	0.322117	0.365065	0.377586	1.380046	0.674751	2.822561	0.998587
hematopoietic	Iron deficiency anemias	Wald ratio	1	-0.10737	0.121688	0.377586	0.898191	0.707596	1.140124	0.998587
musculoskeletal	Pain in joint	Wald ratio	1	0.107372	0.121688	0.377586	1.113349	0.877097	1.413235	0.998587
injuries & poisonings	Fracture of lower limb	Wald ratio	1	0.107372	0.121688	0.377586	1.113349	0.877097	1.413235	0.998587
injuries & poisonings	Poisoning by antibiotics	Wald ratio	1	-0.06943	0.07874	0.377875	0.932922	0.799504	1.088603	0.998587
circulatory system	Occlusion and stenosis of precerebral arteries	Wald ratio	1	0.264851	0.300642	0.378344	1.303237	0.722956	2.349282	0.998587
neoplasms	Cancer of bronchus; lung	Wald ratio	1	-0.20043	0.229061	0.381574	0.81838	0.522366	1.28214	0.998587
musculoskeletal	Spinal stenosis	Wald ratio	1	-0.15032	0.171795	0.381574	0.860432	0.614442	1.204902	0.998587
injuries & poisonings	Injuries to the nervous system	Wald ratio	1	-0.25054	0.286326	0.381574	0.778384	0.444088	1.36433	0.998587
pregnancy complications	Early or threatened labor; hemorrhage in early pregnancy	Wald ratio	1	0.186112	0.214744	0.386125	1.204557	0.790739	1.834938	0.998587
mental disorders	Alcoholic liver damage	Wald ratio	1	0.314958	0.365065	0.388278	1.370202	0.669938	2.802429	0.998587
neoplasms	Cervical cancer	Wald ratio	1	-0.2219	0.257693	0.389177	0.800993	0.483366	1.327339	0.998587
hematopoietic	Diseases of spleen	Wald ratio	1	0.393698	0.458121	0.390134	1.482453	0.603977	3.638661	0.998587
pregnancy complications	Normal delivery	Wald ratio	1	-0.21474	0.250535	0.391366	0.806748	0.493717	1.318249	0.998587
pregnancy complications	Late pregnancy and failed induction	Wald ratio	1	0.257693	0.300642	0.391366	1.293942	0.717799	2.332526	0.998587
digestive	Dental caries	Wald ratio	1	-0.15748	0.186112	0.397467	0.854295	0.593179	1.230351	0.998587
digestive	Diseases of hard tissues of teeth	Wald ratio	1	-0.15748	0.186112	0.397467	0.854295	0.593179	1.230351	0.998587
genitourinary	Renal colic	Wald ratio	1	0.19327	0.229061	0.398809	1.21321	0.774383	1.900712	0.998587
neoplasms	Cancer of bladder	Wald ratio	1	-0.17895	0.214744	0.404657	0.836145	0.548893	1.273725	0.998587
digestive	Diverticulosis	Wald ratio	1	-0.05655	0.068718	0.410555	0.94502	0.825937	1.081272	0.998587
genitourinary	Irregular menstrual cycle	Wald ratio	1	-0.19327	0.236219	0.413253	0.824259	0.518789	1.309596	0.998587
digestive	Jaundice (not of newborn)	Wald ratio	1	-0.28633	0.350749	0.414313	0.751018	0.377648	1.49353	0.998587
digestive	Duodenal ulcer	Wald ratio	1	-0.15748	0.19327	0.415178	0.854295	0.584915	1.247735	0.998587
hematopoietic	Thrombocytopenia	Wald ratio	1	-0.21474	0.264851	0.417474	0.806748	0.480056	1.355763	0.998587
musculoskeletal	Rheumatoid arthritis and other inflammatory polyarthropathies	Wald ratio	1	-0.12169	0.150321	0.418214	0.885424	0.659471	1.188796	0.998587
endocrine/metabolic	Hypercholesterolemia	Wald ratio	1	0.051539	0.063707	0.418522	1.05289	0.929296	1.192921	0.998587
digestive	Flatulence	Wald ratio	1	-0.20759	0.257693	0.420499	0.812543	0.490336	1.346478	0.998587
respiratory	Asthma	Wald ratio	1	0.055834	0.069434	0.421326	1.057422	0.922879	1.211579	0.998587
digestive	Ulceration of intestine	Wald ratio	1	-0.32212	0.400856	0.421645	0.724614	0.330287	1.589726	0.998587
digestive	Other intestinal obstruction	Wald ratio	1	-0.14316	0.178954	0.423711	0.866613	0.610234	1.230704	0.998587

symptoms	Symptoms concerning nutrition, metabolism, and development	Wald ratio	1	0.11453	0.143163	0.423711	1.121347	0.846988	1.484577	0.998587
circulatory system	Orthostatic hypotension	Wald ratio	1	0.229061	0.286326	0.423711	1.257418	0.717388	2.203968	0.998587
respiratory	Postinflammatory pulmonary fibrosis	Wald ratio	1	-0.27917	0.350749	0.426079	0.756413	0.38036	1.504259	0.998587
digestive	Disorders of esophageal motility	Wald ratio	1	-0.32927	0.415172	0.427718	0.719445	0.318857	1.623303	0.998587
digestive	Diverticulosis and diverticulitis	Wald ratio	1	-0.0544	0.068718	0.428555	0.947051	0.827712	1.083597	0.998587
infectious diseases	Intestinal infection due to C. difficile	Wald ratio	1	0.322117	0.408014	0.429835	1.380046	0.620276	3.070451	0.998587
neoplasms	Benign neoplasm of brain, cranial nerves, meninges	Wald ratio	1	-0.29348	0.372224	0.430427	0.745661	0.3595	1.546624	0.998587
digestive	Obstruction of bile duct	Wald ratio	1	0.293484	0.372224	0.430427	1.341092	0.64657	2.781644	0.998587
injuries & poisonings	Poisoning by psychotropic agents	Wald ratio	1	-0.18611	0.236219	0.430768	0.830181	0.522516	1.319004	0.998587
genitourinary	Dysuria	Wald ratio	1	0.236219	0.300642	0.432035	1.266451	0.702549	2.28297	0.998587
neoplasms	Benign neoplasm of brain and other parts of nervous system	Wald ratio	1	-0.28633	0.365065	0.432856	0.751018	0.367198	1.536032	0.998587
musculoskeletal	Other and unspecified disorders of back	Wald ratio	1	0.178954	0.229061	0.434655	1.195965	0.763376	1.873695	0.998587
circulatory system	Paroxysmal tachycardia, unspecified	Wald ratio	1	-0.14316	0.186112	0.441756	0.866613	0.601733	1.248092	0.998587
dermatologic	Pruritus and related conditions	Wald ratio	1	-0.28633	0.372224	0.441756	0.751018	0.362082	1.557734	0.998587
injuries & poisonings	Complication due to other implant and internal device	Wald ratio	1	0.143163	0.186112	0.441756	1.153918	0.801223	1.661867	0.998587
neurological	Other conditions of brain	Wald ratio	1	-0.21474	0.279168	0.441756	0.806748	0.466773	1.394344	0.998587
dermatologic	Ingrowing nail	Wald ratio	1	0.257693	0.336433	0.443702	1.293942	0.669171	2.502028	0.998587
digestive	Cholelithiasis with acute cholecystitis	Wald ratio	1	-0.20759	0.27201	0.445369	0.812543	0.476768	1.384795	0.998587
digestive	Other disorders of peritoneum	Wald ratio	1	-0.136	0.178954	0.447255	0.872838	0.614618	1.239545	0.998587
genitourinary	Disorders of menstruation and other abnormal bleeding from female genital	Wald ratio	1	0.065139	0.085898	0.448251	1.067307	0.901928	1.263011	0.998587
endocrine/metabolic	Type 2 diabetes with neurological manifestations	Wald ratio	1	0.329275	0.436647	0.45079	1.38996	0.590637	3.271022	0.998587
hematopoietic	Other anemias	Wald ratio	1	-0.07015	0.093056	0.450942	0.932254	0.776826	1.118781	0.998587
neoplasms	Leukemia	Wald ratio	1	-0.19327	0.257693	0.453255	0.824259	0.497406	1.365893	0.998587
dermatologic	Psoriasis vulgaris	Wald ratio	1	-0.19327	0.257693	0.453255	0.824259	0.497406	1.365893	0.998587
genitourinary	Irregular menstrual bleeding	Wald ratio	1	0.128847	0.171795	0.453255	1.137516	0.812311	1.592915	0.998587
pregnancy complications	Known or suspected fetal abnormality affecting management of mother	Wald ratio	1	0.128847	0.171795	0.453255	1.137516	0.812311	1.592915	0.998587
injuries & poisonings	Open wounds of head; neck; and trunk	Wald ratio	1	0.128847	0.171795	0.453255	1.137516	0.812311	1.592915	0.998587
digestive	Acute gastritis	Wald ratio	1	-0.22906	0.3078	0.456764	0.79528	0.435026	1.453868	0.998587
genitourinary	Dyspareunia	Wald ratio	1	0.221902	0.300642	0.460457	1.24845	0.692563	2.250519	0.998587
sense organs	Aphakia and other disorders of lens	Wald ratio	1	-0.17895	0.243377	0.46216	0.836145	0.518937	1.34725	0.998587
digestive	Celiac disease	Wald ratio	1	-0.17895	0.243377	0.46216	0.836145	0.518937	1.34725	0.998587
symptoms	Symptoms involving nervous and musculoskeletal systems	Wald ratio	1	0.178954	0.243377	0.46216	1.195965	0.742253	1.927015	0.998587
respiratory	Epistaxis or throat hemorrhage	Wald ratio	1	0.157479	0.214744	0.463355	1.170556	0.768419	1.783144	0.998587
genitourinary	Noninflammatory female genital disorders	Wald ratio	1	0.07874	0.107372	0.463355	1.081923	0.876595	1.335344	0.998587
digestive	Peritoneal adhesions (postoperative) (postinfection)	Wald ratio	1	-0.136	0.186112	0.46492	0.872838	0.606055	1.257058	0.998587
musculoskeletal	Other and unspecified disc disorder	Wald ratio	1	0.11453	0.157479	0.467059	1.121347	0.823552	1.526824	0.998587
digestive	Acute appendicitis	Wald ratio	1	-0.15032	0.207586	0.468981	0.860432	0.572816	1.292461	0.998587
congenital anomalies	Congenital anomalies of great vessels	Wald ratio	1	0.178954	0.250535	0.475051	1.195965	0.731912	1.954242	0.998587
circulatory system	Rheumatic disease of the heart valves	Wald ratio	1	0.107372	0.150321	0.475051	1.113349	0.829231	1.494813	0.998587
dermatologic	Psoriasis	Wald ratio	1	-0.15748	0.221902	0.477904	0.854295	0.552994	1.31976	0.998587
endocrine/metabolic	Disorders of adrenal glands	Wald ratio	1	0.293484	0.415172	0.479631	1.341092	0.59437	3.02594	0.998587
neurological	Other headache syndromes	Wald ratio	1	0.085898	0.121688	0.480261	1.089695	0.858463	1.38321	0.998587
digestive	Duodenitis	Wald ratio	1	0.085898	0.121688	0.480261	1.089695	0.858463	1.38321	0.998587
digestive	Acute pancreatitis	Wald ratio	1	0.164637	0.236219	0.485822	1.178965	0.74204	1.873159	0.998587
circulatory system	Aortic aneurysm	Wald ratio	1	0.19327	0.279168	0.488744	1.21321	0.701946	2.096855	0.998587
respiratory	Nasal polyps	Wald ratio	1	-0.12885	0.186112	0.488744	0.879109	0.610409	1.266089	0.998587
infectious diseases	Infection/inflammation of internal prosthetic device; implant; and graft	Wald ratio	1	0.143163	0.207586	0.490411	1.153918	0.768199	1.733308	0.998587
circulatory system	Cardiac pacemaker/device in situ	Wald ratio	1	-0.14316	0.207586	0.490411	0.866613	0.576931	1.301746	0.998587
endocrine/metabolic	Other disorders of pancreatic internal secretion	Wald ratio	1	-0.23622	0.343591	0.491768	0.789608	0.402662	1.548396	0.998587
circulatory system	Paroxysmal ventricular tachycardia	Wald ratio	1	-0.23622	0.343591	0.491768	0.789608	0.402662	1.548396	0.998587
dermatologic	Cellulitis and abscess of face/neck	Wald ratio	1	0.314958	0.458121	0.491768	1.370202	0.558244	3.363143	0.998587
neurological	Degenerative disease of the spinal cord	Wald ratio	1	0.171795	0.250535	0.492893	1.187435	0.726691	1.940303	0.998587
injuries & poisonings	Fracture of humerus	Wald ratio	1	0.171795	0.250535	0.492893	1.187435	0.726691	1.940303	0.998587
musculoskeletal	Other disorders of soft tissues	Wald ratio	1	0.093056	0.136005	0.493842	1.097523	0.840706	1.432792	0.998587
musculoskeletal	Osteoporosis	Wald ratio	1	0.093056	0.136005	0.493842	1.097523	0.840706	1.432792	0.998587
dermatologic	Diffuse diseases of connective tissue	Wald ratio	1	-0.12169	0.178954	0.496504	0.885424	0.623481	1.257418	0.998587
sense organs	Disorders of lacrimal system	Wald ratio	1	0.150321	0.221902	0.49814	1.162207	0.752309	1.79544	0.998587
respiratory	Bronchiectasis	Wald ratio	1	0.164637	0.243377	0.498742	1.178965	0.731702	1.899624	0.998587
injuries & poisonings	Fracture of neck of femur	Wald ratio	1	-0.16464	0.243377	0.498742	0.848201	0.52642	1.366676	0.998587
musculoskeletal	Unspecified monoarthritis	Wald ratio	1	-0.05798	0.085898	0.499676	0.943668	0.797446	1.116701	0.998587
circulatory system	Cardiac arrest and ventricular fibrillation	Wald ratio	1	0.207586	0.3078	0.500045	1.230704	0.673207	2.249875	0.998587
musculoskeletal	Polymyalgia Rheumatica	Wald ratio	1	-0.20759	0.3078	0.500045	0.812543	0.444469	1.485427	0.998587
endocrine/metabolic	Gout and other crystal arthropathies	Wald ratio	1	0.11453	0.171795	0.504985	1.121347	0.800764	1.570273	0.998587
sense organs	Other retinal disorders	Wald ratio	1	0.11453	0.171795	0.504985	1.121347	0.800764	1.570273	0.998587
sense organs	Other disorders of eye	Wald ratio	1	-0.11453	0.171795	0.504985	0.891785	0.636832	1.248807	0.998587
digestive	Peritoneal or intestinal adhesions	Wald ratio	1	-0.24338	0.365065	0.504985	0.783976	0.383312	1.60344	0.998587
endocrine/metabolic	Secondary hypothyroidism	Wald ratio	1	-0.20759	0.314958	0.509837	0.812543	0.438277	1.506414	0.998587
neoplasms	Benign neoplasm of ovary	Wald ratio	1	-0.17895	0.27201	0.510606	0.836145	0.490617	1.425019	0.998587
circulatory system	Right bundle branch block	Wald ratio	1	-0.17895	0.27201	0.510606	0.836145	0.490617	1.425019	0.998587
circulatory system	Intracerebral hemorrhage	Wald ratio	1	0.257693	0.393698	0.51276	1.293942	0.598125	2.799221	0.998587
circulatory system	Other chronic ischemic heart disease, unspecified	Wald ratio	1	0.060844	0.093056	0.513211	1.062733	0.885551	1.275367	0.998587
circulatory system	Raynaud's syndrome	Wald ratio	1	-0.20043	0.3078	0.514941	0.81838	0.447662	1.496098	0.998587
digestive	Irritable Bowel Syndrome	Wald ratio	1	-0.09306	0.143163	0.515692	0.911143	0.688214	1.206283	0.998587
sense organs	Degeneration of macula and posterior pole of retina	Wald ratio	1	0.143163	0.221902	0.518823	1.153918	0.746943	1.782634	0.998587
sense organs	Macular degeneration (senile) of retina NOS	Wald ratio	1	0.143163	0.221902	0.518823	1.153918	0.746943	1.782634	0.998587
sense organs	Cholesteatoma	Wald ratio	1	0.27201	0.422331	0.519531	1.312599	0.573637	3.003497	0.998587
digestive	Diaphragmatic hernia	Wald ratio	1	0.043665	0.068002	0.520805	1.044632	0.914279	1.193571	0.998587
neurological	Disorders of other cranial nerves	Wald ratio	1	0.178954	0.279168	0.521506	1.195965	0.691968	2.067049	0.998587
sense organs	Dizziness and giddiness (Light-headedness and vertigo)	Wald ratio	1	0.100214	0.157479	0.524539	1.105407	0.811846	1.505121	0.998587
circulatory system	Carditis	Wald ratio	1	-0.15032	0.236219	0.524539	0.860432	0.541555	1.367067	0.998587
digestive	Ulcerative colitis (chronic)	Wald ratio	1	-0.28633	0.450963	0.52548	0.751018	0.310301	1.817682	0.998587
endocrine/metabolic	Type 2 diabetes with ophthalmic manifestations	Wald ratio	1	0.186112	0.293484	0.525985	1.204557	0.677655	2.141144	0.998587
sense organs	Inflammation of eyelids	Wald ratio	1	-0.136	0.214744	0.526516	0.872838	0.57298	1.329621	0.998587
mental disorders	Psychogenic and somatoform disorders	Wald ratio	1	0.279168	0.450963	0.535885	1.322029	0.546227	3.199695	0.998587
neoplasms	Pancreatic cancer	Wald ratio	1	0.264851	0.429489	0.537455	1.303237	0.561611	3.024208	0.998587
genitourinary	Hematuria	Wald ratio	1	-0.05297	0.085898	0.537455	0.948408	0.801452	1.12231	0.998587
congenital anomalies	Congenital anomalies of genital organs	Wald ratio	1	0.236219	0.38654	0.541126	1.266451	0.593689	2.70158	0.998587
musculoskeletal	Osteopenia or other disorder of bone and cartilage	Wald ratio	1	-0.2219	0.365065	0.543292	0.800993	0.391633	1.638245	0.998587
digestive	Hematemesis	Wald ratio	1	-0.14316	0.236219	0.544475	0.866613	0.545446	1.376888	0.998587
endocrine/metabolic	Hypothyroidism NOS	Wald ratio	1	0.055834	0.093056	0.548506	1.057422	0.881125	1.268992	0.998587
symptoms	Cervicalgia	Wald ratio	1	0.150321	0.250535	0.548506	1.162207	0.711253	1.89908	0.998587
neoplasms	Nevus, non-neoplastic	Wald ratio	1	-0.25769	0.429489	0.548506	0.772832	0.333041	1.793385	0.998587
circulatory system	Paroxysmal supraventricular tachycardia	Wald ratio	1	-0.12885	0.214744	0.548506	0.879109	0.577097	1.339173	0.998587
circulatory system	Circulatory disease NEC	Wald ratio	1	0.051539	0.085898	0.548506	1.05289	0.889745	1.24595	0.998587
respiratory	Pneumonitis due to inhalation of food or vomitus	Wald ratio	1	-0.25769	0.429489	0.548506	0.772832	0.333041	1.793385	0.998587
symptoms	Back pain	Wald ratio	1	-0.06013	0.100214	0.548506	0.941644	0.773718	1.146015	0.998587
dermatologic	Disturbance of skin sensation	Wald ratio	1	0.11453	0.19327	0.553454	1.121347	0.76776	1.637776	0.998587
circulatory system	Other disorders of circulatory system	Wald ratio	1	0.050823	0.085898	0.554074	1.052136	0.889108	1.245058	0.998587
neoplasms	Other benign neoplasm of connective and other soft tissue	Wald ratio	1	-0.18611	0.314958	0.554581	0.830181	0.44779	1.539114	0.998587
neoplasms	Large cell lymphoma	Wald ratio	1	-0.25769	0.436647	0.555081	0.772832	0.328401	1.818723	0.998587
dermatologic	Superficial cellulitis and abscess	Wald ratio	1	0.071581	0.121688	0.556374	1.074206	0.846261	1.363549	0.998587

genitourinary	Other disorders of bladder	Wald ratio	1	0.062992	0.107372	0.557428	1.065018	0.862899	1.31448	0.998587
sense organs	Senile cataract	Wald ratio	1	-0.07087	0.121688	0.560329	0.931587	0.733905	1.182515	0.998587
neoplasms	Benign neoplasm of colon	Wald ratio	1	0.045812	0.07874	0.560689	1.046878	0.897163	1.221576	0.998587
endocrine/metabolic	Diabetes mellitus	Wald ratio	1	0.045812	0.07874	0.560689	1.046878	0.897163	1.221576	0.998587
sense organs	Otitis media and Eustachian tube disorders	Wald ratio	1	0.128847	0.221902	0.56148	1.137516	0.736326	1.757295	0.998587
dermatologic	Rash and other nonspecific skin eruption	Wald ratio	1	-0.12885	0.221902	0.56148	0.879109	0.569057	1.358094	0.998587
circulatory system	Atrial fibrillation and flutter	Wald ratio	1	0.053686	0.093056	0.563991	1.055153	0.879235	1.26627	0.998587
digestive	Peptic ulcer (excl. esophageal)	Wald ratio	1	-0.07015	0.121688	0.564297	0.932254	0.734431	1.183362	0.998587
mental disorders	Delirium dementia and amnestic and other cognitive disorders	Wald ratio	1	-0.136	0.236219	0.564779	0.872838	0.549364	1.386779	0.998587
genitourinary	Cervicitis and endocervicitis	Wald ratio	1	-0.16464	0.286326	0.565291	0.848201	0.48392	1.486703	0.998587
infectious diseases	Viral hepatitis	Wald ratio	1	-0.1718	0.300642	0.567709	0.842151	0.467174	1.518105	0.998587
neurological	Abnormal movement	Wald ratio	1	-0.11453	0.200428	0.567709	0.891785	0.602077	1.320894	0.998587
digestive	Diseases of lips	Wald ratio	1	0.229061	0.400856	0.567709	1.257418	0.573145	2.758642	0.998587
endocrine/metabolic	Hyperlipidemia	Wald ratio	1	0.035075	0.06156	0.568835	1.035697	0.917977	1.168514	0.998587
digestive	Ascites (non malignant)	Wald ratio	1	0.150321	0.264851	0.570329	1.162207	0.691572	1.953123	0.998587
genitourinary	Calculus of ureter	Wald ratio	1	-0.12169	0.214744	0.570941	0.885424	0.581242	1.348793	0.998587
infectious diseases	Septicemia	Wald ratio	1	0.093056	0.164637	0.571926	1.097523	0.794825	1.515499	0.998587
digestive	Gastric ulcer	Wald ratio	1	0.093056	0.164637	0.571926	1.097523	0.794825	1.515499	0.998587
genitourinary	Frequency of urination and polyuria	Wald ratio	1	0.093056	0.164637	0.571926	1.097523	0.794825	1.515499	0.998587
genitourinary	Genital prolapse	Wald ratio	1	-0.05655	0.100214	0.57256	0.94502	0.776492	1.150124	0.998587
mental disorders	Agorophobia, social phobia, and panic disorder	Wald ratio	1	0.221902	0.393698	0.573002	1.24845	0.577097	2.700807	0.998587
neoplasms	Malignant neoplasm of uterus	Wald ratio	1	-0.16464	0.293484	0.574814	0.848201	0.477178	1.507709	0.998587
circulatory system	Phlebitis and thrombophlebitis of lower extremities	Wald ratio	1	-0.10021	0.178954	0.575479	0.904644	0.637014	1.284713	0.998587
genitourinary	Chronic glomerulonephritis, NOS	Wald ratio	1	0.200428	0.357907	0.575479	1.221926	0.605882	2.464346	0.998587
circulatory system	Abnormal heart sounds	Wald ratio	1	-0.17895	0.322117	0.578515	0.836145	0.444724	1.572072	0.998587
digestive	Other disorders of biliary tract	Wald ratio	1	-0.17895	0.322117	0.578515	0.836145	0.444724	1.572072	0.998587
injuries & poisonings	Traumatic arthropathy	Wald ratio	1	0.250535	0.450963	0.578515	1.284713	0.530809	3.109378	0.998587
genitourinary	Nephritis; nephrosis; renal sclerosis	Wald ratio	1	0.150321	0.27201	0.580516	1.162207	0.681937	1.980719	0.998587
neoplasms	Cancer of larynx, pharynx, nasal cavities	Wald ratio	1	-0.22906	0.415172	0.581137	0.79528	0.352467	1.794412	0.998587
genitourinary	Pelvic peritoneal adhesions, female (postoperative) (postinfection)	Wald ratio	1	-0.11453	0.207586	0.581137	0.891785	0.593689	1.339557	0.998587
digestive	Peritonitis and retroperitoneal infections	Wald ratio	1	0.19327	0.350749	0.58162	1.21321	0.61006	2.41268	0.998587
dermatologic	Hyperhidrosis	Wald ratio	1	-0.23622	0.429489	0.582319	0.789608	0.34027	1.832313	0.998587
sense organs	Labyrinthitis	Wald ratio	1	-0.20759	0.379382	0.584262	0.812543	0.386287	1.70916	0.998587
respiratory	Emphysema	Wald ratio	1	0.136005	0.250535	0.587228	1.145687	0.701143	1.872086	0.998587
respiratory	Respiratory abnormalities	Wald ratio	1	-0.22906	0.422331	0.587562	0.79528	0.347556	1.819765	0.998587
endocrine/metabolic	Hypoglycemia	Wald ratio	1	-0.18611	0.343591	0.588048	0.830181	0.423353	1.627958	0.998587
dermatologic	Seborrheic keratosis	Wald ratio	1	0.100214	0.186112	0.590258	1.105407	0.76754	1.592003	0.998587
sense organs	Retinal detachment with retinal defect	Wald ratio	1	-0.15032	0.279168	0.590258	0.860432	0.497833	1.487129	0.998587
sense organs	Corneal opacity and other disorders of cornea	Wald ratio	1	0.207586	0.38654	0.591242	1.230704	0.576931	2.625324	0.998587
genitourinary	Hypertrophy of female genital organs	Wald ratio	1	0.157479	0.293484	0.591554	1.170556	0.658527	2.080707	0.998587
musculoskeletal	Osteoarthritis; localized	Wald ratio	1	0.042233	0.07874	0.591707	1.043138	0.893958	1.217212	0.998587
circulatory system	Hemorrhoids	Wald ratio	1	0.037938	0.070866	0.592405	1.038667	0.903971	1.193434	0.998587
endocrine/metabolic	Disorders of lipoid metabolism	Wald ratio	1	0.032927	0.06156	0.59273	1.033476	0.916008	1.166007	0.998587
symptoms	Neuralgia, neuritis, and radiculitis NOS	Wald ratio	1	0.164637	0.3078	0.59273	1.178965	0.644906	2.155291	0.998587
endocrine/metabolic	Hyperpotassemia	Wald ratio	1	-0.17895	0.336433	0.594785	0.836145	0.432418	1.616809	0.998587
injuries & poisonings	Dislocation	Wald ratio	1	0.121688	0.229061	0.595246	1.129402	0.720889	1.769412	0.998587
endocrine/metabolic	Thyrotoxicosis with or without goiter	Wald ratio	1	-0.12885	0.243377	0.59652	0.879109	0.545602	1.416476	0.998587
hematopoietic	Purpura and other hemorrhagic conditions	Wald ratio	1	-0.12885	0.243377	0.59652	0.879109	0.545602	1.416476	0.998587
circulatory system	Heart valve disorders	Wald ratio	1	0.085898	0.164637	0.601852	1.089695	0.789156	1.50469	0.998587
pregnancy complications	Obstetrical/birth trauma	Wald ratio	1	0.085898	0.164637	0.601852	1.089695	0.789156	1.50469	0.998587
circulatory system	Bundle branch block	Wald ratio	1	-0.09306	0.178954	0.603064	0.911143	0.641591	1.293942	0.998587
genitourinary	Inflammatory disease of breast	Wald ratio	1	-0.20043	0.38654	0.604097	0.81838	0.383642	1.74576	0.998587
digestive	Calculus of bile duct	Wald ratio	1	-0.10737	0.207586	0.604988	0.898191	0.597954	1.34918	0.998587
digestive	Blood in stool	Wald ratio	1	-0.10737	0.207586	0.604988	0.898191	0.597954	1.34918	0.998587
congenital anomalies	Cardiac shunt/ heart septal defect	Wald ratio	1	0.221902	0.429489	0.605389	1.24845	0.538001	2.897071	0.998587
musculoskeletal	Osteoarthrosis NOS	Wald ratio	1	-0.05154	0.100214	0.607052	0.949767	0.780393	1.155902	0.998587
genitourinary	Benign neoplasm of breast	Wald ratio	1	0.136005	0.264851	0.607592	1.145687	0.681742	1.925361	0.998587
neurological	Nerve root and plexus disorders	Wald ratio	1	-0.15748	0.3078	0.608911	0.854295	0.467308	1.561754	0.998587
circulatory system	Hypotension	Wald ratio	1	0.069434	0.136005	0.609683	1.071901	0.82108	1.399344	0.998587
genitourinary	Other signs and symptoms in breast	Wald ratio	1	0.200428	0.393698	0.610689	1.221926	0.564836	2.643427	0.998587
sense organs	Otitis media	Wald ratio	1	0.121688	0.243377	0.617075	1.129402	0.700942	1.819765	0.998587
circulatory system	Palpitations	Wald ratio	1	0.085898	0.171795	0.617075	1.089695	0.778161	1.52595	0.998587
digestive	Abnormal findings on exam of gastrointestinal tract/ abdominal area	Wald ratio	1	-0.12885	0.257693	0.617075	0.879109	0.530505	1.456785	0.998587
musculoskeletal	Symptoms and disorders of the joints	Wald ratio	1	0.085898	0.171795	0.617075	1.089695	0.778161	1.52595	0.998587
dermatologic	Atopic/contact dermatitis due to other or unspecified	Wald ratio	1	-0.11453	0.229061	0.617075	0.891785	0.569219	1.397142	0.998587
neoplasms	Secondary malignancy of lymph nodes	Wald ratio	1	-0.07087	0.143163	0.6206	0.931587	0.703657	1.23335	0.998587
symptoms	Pain in limb	Wald ratio	1	-0.06585	0.136005	0.628236	0.936267	0.717183	1.222276	0.998587
injuries & poisonings	Anaphylactic shock NOS	Wald ratio	1	-0.21474	0.443805	0.628477	0.806748	0.338036	1.925361	0.998587
circulatory system	Mitral valve disease	Wald ratio	1	-0.09306	0.19327	0.630174	0.911143	0.623838	1.330764	0.998587
genitourinary	Hypertrophy of breast (Gynecomastia)	Wald ratio	1	0.171795	0.357907	0.631227	1.187435	0.58878	2.394786	0.998587
musculoskeletal	Unspecified polyarthropathy or polyarthritis	Wald ratio	1	0.085898	0.178954	0.631227	1.089695	0.76732	1.547509	0.998587
neoplasms	Benign neoplasm of lip, oral cavity, and pharynx	Wald ratio	1	0.157479	0.329275	0.632465	1.170556	0.613915	2.23191	0.998587
endocrine/metabolic	Electrolyte imbalance	Wald ratio	1	-0.07874	0.164637	0.632465	0.924281	0.669363	1.27628	0.998587
digestive	GERD	Wald ratio	1	-0.04438	0.093056	0.633417	0.95659	0.797104	1.147986	0.998587
injuries & poisonings	Injury, NOS	Wald ratio	1	0.054402	0.11453	0.634787	1.055909	0.843599	1.32165	0.998587
respiratory	Other diseases of respiratory system, NEC	Wald ratio	1	0.054402	0.11453	0.634787	1.055909	0.843599	1.32165	0.998587
musculoskeletal	Stiffness of joint	Wald ratio	1	0.200428	0.422331	0.635089	1.221926	0.53401	2.796017	0.998587
circulatory system	Polyarteritis nodosa and allied conditions	Wald ratio	1	-0.1718	0.365065	0.637935	0.842151	0.411756	1.722424	0.998587
musculoskeletal	Osteoporosis, osteopenia and pathological fracture	Wald ratio	1	0.057265	0.121688	0.637935	1.058937	0.834232	1.344167	0.998587
musculoskeletal	Displacement of intervertebral disc	Wald ratio	1	0.214744	0.458121	0.639248	1.239545	0.505012	3.042447	0.998587
neoplasms	Melanomas of skin, dx or hx	Wald ratio	1	0.093056	0.200428	0.642443	1.097523	0.740979	1.625629	0.998587
neoplasms	Melanomas of skin	Wald ratio	1	0.093056	0.200428	0.642443	1.097523	0.740979	1.625629	0.998587
digestive	Diseases of pancreas	Wald ratio	1	0.093056	0.200428	0.642443	1.097523	0.740979	1.625629	0.998587
musculoskeletal	Malunion and nonunion of fracture	Wald ratio	1	-0.136	0.293484	0.643067	0.872838	0.491038	1.551502	0.998587
injuries & poisonings	Poisoning by other anti-infectives	Wald ratio	1	-0.136	0.293484	0.643067	0.872838	0.491038	1.551502	0.998587
neoplasms	Lymphoid leukemia, chronic	Wald ratio	1	-0.21474	0.465279	0.644412	0.806748	0.324104	2.008129	0.998587
respiratory	Acute pharyngitis	Wald ratio	1	-0.16464	0.357907	0.645516	0.848201	0.420574	1.710629	0.998587
neoplasms	Malignant neoplasm of testis	Wald ratio	1	0.07874	0.171795	0.646713	1.081923	0.772611	1.515066	0.998587
digestive	Personal history of diseases of digestive system	Wald ratio	1	-0.03937	0.085898	0.646713	0.961395	0.812427	1.137678	0.998587
symptoms	Abdominal pain	Wald ratio	1	-0.02434	0.053686	0.650309	0.975956	0.87848	1.084248	0.998587
genitourinary	Functional disorders of bladder	Wald ratio	1	0.11453	0.257693	0.656721	1.121347	0.676686	1.858201	0.998587
symptoms	Musculoskeletal symptoms referable to limbs	Wald ratio	1	0.062992	0.143163	0.659937	1.065018	0.804441	1.410002	0.998587
sense organs	Visual disturbances	Wald ratio	1	-0.07874	0.178954	0.659937	0.924281	0.650842	1.312599	0.998587
neoplasms	Vascular hamartomas and non-neoplastic nevi	Wald ratio	1	-0.17895	0.408014	0.660954	0.836145	0.375814	1.860331	0.998587
neoplasms	Malignant neoplasm of ovary	Wald ratio	1	-0.10021	0.229061	0.661749	0.904644	0.577427	1.417287	0.998587
circulatory system	First degree AV block	Wald ratio	1	0.143163	0.329275	0.66372	1.153918	0.605188	2.200185	0.998587
digestive	Chronic periodontitis	Wald ratio	1	-0.18611	0.436647	0.669941	0.830181	0.35277	1.953682	0.998587
musculoskeletal	Osteomyelitis	Wald ratio	1	-0.18611	0.436647	0.669941	0.830181	0.35277	1.953682	0.998587
circulatory system	Essential hypertension	Wald ratio	1	-0.02004	0.047244	0.671389	0.980157	0.893472	1.075252	0.998587
endocrine/metabolic	Simple and unspecified goiter	Wald ratio	1	-0.17895	0.422331	0.671764	0.836145	0.365415	1.913271	0.998587
circulatory system	Other specified cardiac dysrhythmias	Wald ratio	1	0.07874	0.186112	0.672239	1.081923	0.751233	1.55818	0.998587

genitourinary	Symptoms involving female genital tract	Wald ratio	1	0.054402	0.128847	0.672863	1.055909	0.820257	1.359261	0.998587
musculoskeletal	Other disorders of cervical region	Wald ratio	1	0.19327	0.458121	0.673116	1.21321	0.494283	2.977808	0.998587
symptoms	Nausea and vomiting	Wald ratio	1	0.042233	0.100214	0.673442	1.043138	0.857112	1.269537	0.998587
neoplasms	Cancer of other lymphoid, histiocytic tissue	Wald ratio	1	0.093056	0.221902	0.674957	1.097523	0.710438	1.695512	0.998587
symptoms	Gangrene	Wald ratio	1	-0.18611	0.443805	0.674957	0.830181	0.347855	1.981286	0.998587
neoplasms	Cancer of urinary organs (incl. kidney and bladder)	Wald ratio	1	-0.06872	0.164637	0.676392	0.93359	0.676105	1.289134	0.998587
digestive	Reflux esophagitis	Wald ratio	1	0.043665	0.107372	0.684253	1.044632	0.846382	1.289319	0.998587
neoplasms	Malignant neoplasm of ovary and other uterine adnexa	Wald ratio	1	-0.09306	0.229061	0.684559	0.911143	0.581575	1.427469	0.998587
neoplasms	Malignant neoplasm of bladder	Wald ratio	1	-0.09306	0.229061	0.684559	0.911143	0.581575	1.427469	0.998587
endocrine/metabolic	Disorders of calcium/phosphorus metabolism	Wald ratio	1	0.121688	0.300642	0.685653	1.129402	0.626523	2.035918	0.998587
circulatory system	Phlebitis and thrombophlebitis	Wald ratio	1	-0.06943	0.171795	0.68609	0.932922	0.666208	1.306413	0.998587
circulatory system	Ill-defined descriptions and complications of heart disease	Wald ratio	1	-0.15032	0.372224	0.686326	0.860432	0.414833	1.784676	0.998587
genitourinary	Calculus of lower urinary tract	Wald ratio	1	0.150321	0.372224	0.686326	1.162207	0.560326	2.410608	0.998587
mental disorders	Memory loss	Wald ratio	1	0.157479	0.393698	0.689157	1.170556	0.54109	2.532298	0.998587
circulatory system	Coronary atherosclerosis	Wald ratio	1	0.031496	0.07874	0.689157	1.031997	0.884411	1.204212	0.998587
genitourinary	Noninflammatory disorders of vulva and perineum	Wald ratio	1	-0.11453	0.286326	0.689157	0.891785	0.508785	1.563096	0.998587
circulatory system	Arrhythmia (cardiac) NOS	Wald ratio	1	0.136005	0.343591	0.692228	1.145687	0.584246	2.246656	0.998587
respiratory	Other dyspnea	Wald ratio	1	-0.136	0.343591	0.692228	0.872838	0.445106	1.711608	0.998587
hematopoietic	Other deficiency anemia	Wald ratio	1	0.121688	0.3078	0.692585	1.129402	0.617794	2.064683	0.998587
circulatory system	Hypertension	Wald ratio	1	-0.01861	0.047244	0.693626	0.981561	0.894752	1.076792	0.998587
mental disorders	Alcoholism	Wald ratio	1	0.045096	0.11453	0.693766	1.046129	0.835786	1.309409	0.998587
neurological	Convulsions	Wald ratio	1	0.085898	0.221902	0.698685	1.089695	0.705371	1.683419	0.998587
digestive	Gastritis and duodenitis	Wald ratio	1	0.025054	0.065139	0.700522	1.02537	0.902471	1.165006	0.998587
circulatory system	Other aneurysm	Wald ratio	1	0.093056	0.243377	0.7022	1.097523	0.681157	1.768399	0.998587
pregnancy complications	Missed abortion/Hydatidiform mole	Wald ratio	1	0.11453	0.300642	0.703239	1.121347	0.622054	2.021397	0.998587
circulatory system	Hypertensive chronic kidney disease	Wald ratio	1	0.100214	0.264851	0.70515	1.105407	0.657773	1.857669	0.998587
injuries & poisonings	Fracture of clavicle or scapula	Wald ratio	1	-0.10021	0.264851	0.70515	0.904644	0.538309	1.52028	0.998587
circulatory system	Atherosclerosis	Wald ratio	1	0.107372	0.286326	0.70766	1.113349	0.635193	1.951446	0.998587
genitourinary	Pelvic inflammatory disease (PID)	Wald ratio	1	-0.12885	0.343591	0.70766	0.879109	0.448303	1.723904	0.998587
neoplasms	Lipoma	Wald ratio	1	0.04796	0.128847	0.709727	1.049128	0.81499	1.350532	0.998587
injuries & poisonings	Complications of cardiac/vascular device, implant, and graft	Wald ratio	1	0.093056	0.250535	0.710318	1.097523	0.671667	1.793385	0.998587
infectious diseases	Postoperative infection	Wald ratio	1	-0.05798	0.157479	0.712738	0.943668	0.693059	1.284897	0.998587
dermatologic	Erythematous conditions	Wald ratio	1	0.07874	0.214744	0.713868	1.081923	0.710235	1.648125	0.998587
dermatologic	Other local infections of skin and subcutaneous tissue	Wald ratio	1	-0.05727	0.157479	0.71613	0.944344	0.693555	1.285817	0.998587
dermatologic	Cellulitis and abscess of leg, except foot	Wald ratio	1	-0.05154	0.143163	0.718847	0.949767	0.717388	1.257418	0.998587
endocrine/metabolic	Hypothyroidism	Wald ratio	1	0.032927	0.093056	0.723454	1.033476	0.861171	1.240255	0.998587
digestive	Other disorders of liver	Wald ratio	1	0.04796	0.136005	0.724365	1.049128	0.803635	1.369614	0.998587
dermatologic	Symptoms affecting skin	Wald ratio	1	0.050107	0.143163	0.726339	1.051384	0.794143	1.391951	0.998587
injuries & poisonings	Crushing or internal injury to organs	Wald ratio	1	0.11453	0.329275	0.727971	1.121347	0.588106	2.138081	0.998587
symptoms	Fever of unknown origin	Wald ratio	1	-0.05727	0.164637	0.727971	0.944344	0.683893	1.303984	0.998587
neurological	Hemiplegia	Wald ratio	1	-0.09306	0.27201	0.732272	0.911143	0.534622	1.552836	0.998587
digestive	Esophageal bleeding (varices/hemorrhage)	Wald ratio	1	-0.0859	0.257693	0.738883	0.917688	0.553786	1.520716	0.998587
neoplasms	Neoplasm of uncertain behavior	Wald ratio	1	0.093056	0.279168	0.738883	1.097523	0.635011	1.896907	0.998587
sense organs	Blindness and low vision	Wald ratio	1	0.128847	0.38654	0.738883	1.137516	0.533247	2.426536	0.998587
sense organs	Ectropion or entropion	Wald ratio	1	0.107372	0.322117	0.738883	1.113349	0.592161	2.093256	0.998587
genitourinary	Renal failure NOS	Wald ratio	1	-0.09306	0.279168	0.738883	0.911143	0.527174	1.574775	0.998587
genitourinary	Bladder neck obstruction	Wald ratio	1	0.07874	0.236219	0.738883	1.081923	0.680962	1.718975	0.998587
genitourinary	Breast conditions, congenital or relating to hormones	Wald ratio	1	0.11453	0.343591	0.738883	1.121347	0.571833	2.198925	0.998587
genitourinary	stress incontinence, female	Wald ratio	1	0.045096	0.136005	0.740207	1.046129	0.801338	1.365698	0.998587
hematopoietic	Decreased white blood cell count	Wald ratio	1	0.06156	0.186112	0.740819	1.063494	0.738437	1.53164	0.998587
hematopoietic	Neutropenia	Wald ratio	1	0.06156	0.186112	0.740819	1.063494	0.738437	1.53164	0.998587
genitourinary	Urinary incontinence	Wald ratio	1	0.037222	0.11453	0.745181	1.037924	0.829231	1.299139	0.998587
dermatologic	Other hypertrophic and atrophic conditions of skin	Wald ratio	1	0.048675	0.150321	0.746082	1.049879	0.781958	1.409598	0.998587
endocrine/metabolic	Hyposmolality and/or hyponatremia	Wald ratio	1	0.07874	0.243377	0.746294	1.081923	0.671475	1.743263	0.998587
injuries & poisonings	Poisoning by anticonvulsants and anti-Parkinsonism drugs	Wald ratio	1	0.150321	0.465279	0.746637	1.162207	0.466906	2.892927	0.998587
mental disorders	Altered mental status	Wald ratio	1	-0.07158	0.221902	0.747013	0.93092	0.602595	1.438135	0.998587
symptoms	Other tests	Wald ratio	1	-0.04366	0.136005	0.748171	0.957275	0.733275	1.249701	0.998587
circulatory system	Chronic pulmonary heart disease	Wald ratio	1	-0.136	0.429489	0.751497	0.872838	0.376137	2.025452	0.998587
genitourinary	Abnormal findings on examination of urine	Wald ratio	1	-0.05655	0.178954	0.752003	0.94502	0.665446	1.342052	0.998587
injuries & poisonings	Contusion	Wald ratio	1	-0.0859	0.27201	0.752162	0.917688	0.538463	1.563991	0.998587
neoplasms	Malignant neoplasm of female breast	Wald ratio	1	0.031496	0.100214	0.753304	1.031997	0.847958	1.255979	0.998587
neoplasms	Cervical intraepithelial neoplasia [CIN] [Cervical dysplasia]	Wald ratio	1	0.071581	0.229061	0.754661	1.074206	0.685657	1.682937	0.998587
circulatory system	Arterial embolism and thrombosis	Wald ratio	1	0.107372	0.343591	0.754661	1.113349	0.567755	2.183241	0.998587
injuries & poisonings	Complication of internal orthopedic device	Wald ratio	1	-0.05798	0.186112	0.755391	0.943668	0.655236	1.359067	0.998587
circulatory system	Vascular insufficiency of intestine	Wald ratio	1	-0.136	0.436647	0.755439	0.872838	0.370896	2.05407	0.998587
endocrine/metabolic	Acidosis	Wald ratio	1	0.100214	0.322117	0.755716	1.105407	0.587938	2.078325	0.998587
genitourinary	Glomerulonephritis	Wald ratio	1	0.100214	0.322117	0.755716	1.105407	0.587938	2.078325	0.998587
respiratory	Bronchitis	Wald ratio	1	-0.12885	0.415172	0.756299	0.879109	0.38962	1.983556	0.998587
symptoms	Pain	Wald ratio	1	0.11453	0.372224	0.758316	1.121347	0.540626	2.325857	0.998587
respiratory	Cough	Wald ratio	1	-0.05941	0.19327	0.758533	0.942318	0.645183	1.376297	0.998587
digestive	Cholelithiasis with other cholecystitis	Wald ratio	1	-0.04366	0.143163	0.760366	0.957275	0.723059	1.267358	0.998587
genitourinary	Chronic renal failure [CKD]	Wald ratio	1	0.062992	0.207586	0.761548	1.065018	0.709016	1.599771	0.998587
pregnancy complications	Fetal distress and abnormal forces of labor	Wald ratio	1	0.071581	0.236219	0.761867	1.074206	0.676105	1.706715	0.998587
neoplasms	Cancer of other female genital organs	Wald ratio	1	-0.06442	0.214744	0.764177	0.937608	0.615499	1.428287	0.998587
circulatory system	Other disorders of arteries and arterioles	Wald ratio	1	0.085898	0.286326	0.764177	1.089695	0.621698	1.909987	0.998587
injuries & poisonings	Complication of colostomy or enterostomy	Wald ratio	1	-0.136	0.465279	0.770051	0.872838	0.350655	2.17264	0.998587
genitourinary	Irregular menstrual cycle/bleeding	Wald ratio	1	0.025054	0.085898	0.770541	1.02537	0.866489	1.213384	0.998587
mental disorders	Neurological disorders	Wald ratio	1	0.043665	0.150321	0.771452	1.044632	0.77805	1.402553	0.998587
circulatory system	Elevated blood pressure reading without diagnosis of hypertension	Wald ratio	1	0.07874	0.27201	0.772219	1.081923	0.634829	1.843891	0.998587
endocrine/metabolic	Disorders of mineral metabolism	Wald ratio	1	0.065855	0.229061	0.77373	1.068072	0.681742	1.673327	0.998587
musculoskeletal	Osteoarthrosis, localized, primary	Wald ratio	1	0.032927	0.11453	0.77373	1.033476	0.825677	1.293571	0.998587
endocrine/metabolic	Nontoxic multinodular goiter	Wald ratio	1	0.11453	0.400856	0.775097	1.121347	0.511122	2.460116	0.998587
neurological	Epilepsy	Wald ratio	1	-0.10021	0.350749	0.775097	0.904644	0.454898	1.799042	0.998587
neurological	Migraine	Wald ratio	1	-0.05583	0.200428	0.780574	0.945697	0.638475	1.400747	0.998587
mental disorders	Alcohol-related disorders	Wald ratio	1	-0.02577	0.093056	0.781839	0.97456	0.812078	1.169551	0.998587
neoplasms	Cervical cancer and dysplasia	Wald ratio	1	-0.04724	0.171795	0.783316	0.953855	0.681157	1.335727	0.998587
injuries & poisonings	Fracture of vertebral column without mention of spinal cord injury	Wald ratio	1	0.07874	0.286326	0.783316	1.081923	0.617264	1.896364	0.998587
dermatologic	Sarcoidosis	Wald ratio	1	-0.12169	0.443805	0.783936	0.885424	0.371003	2.113128	0.998587
sense organs	Primary angle-closure glaucoma	Wald ratio	1	0.107372	0.393698	0.785063	1.113349	0.514646	2.408539	0.998587
circulatory system	Left bundle branch block	Wald ratio	1	0.068002	0.250535	0.786061	1.070368	0.655048	1.749012	0.998587
musculoskeletal	Ankylosing spondylitis	Wald ratio	1	-0.11453	0.422331	0.786248	0.891785	0.389731	2.040587	0.998587
respiratory	Other diseases of lung	Wald ratio	1	0.100214	0.372224	0.787752	1.105407	0.532941	2.292796	0.998587
dermatologic	Prurigo and Lichen	Wald ratio	1	-0.10021	0.372224	0.787752	0.904644	0.436149	1.876379	0.998587
sense organs	Glaucoma	Wald ratio	1	-0.04223	0.157479	0.788559	0.958646	0.70406	1.305291	0.998587
endocrine/metabolic	Protein-calorie malnutrition	Wald ratio	1	0.085898	0.322117	0.789726	1.089695	0.579581	2.048783	0.998587
circulatory system	Atrioventricular block, complete	Wald ratio	1	-0.11453	0.429489	0.789726	0.891785	0.384301	2.069418	0.998587
genitourinary	Dysmenorrhea	Wald ratio	1	-0.06657	0.250535	0.790459	0.935597	0.57257	1.528792	0.998587
injuries & poisonings	Poisoning/allergy of sulfonamides	Wald ratio	1	0.093056	0.350749	0.790774	1.097523	0.551887	2.182616	0.998587
symptoms	Malaise and fatigue	Wald ratio	1	0.047244	0.178954	0.79178	1.048378	0.738226	1.488833	0.998587
circulatory system	Cardiac dysrhythmias	Wald ratio	1	0.018611	0.070866	0.792839	1.018785	0.886667	1.17059	0.998587
endocrine/metabolic	Hypopotassemia	Wald ratio	1	-0.07087	0.27201	0.794458	0.931587	0.546618	1.587679	0.998587

musculoskeletal	Bursitis	Wald ratio	1	-0.09306	0.357907	0.794864	0.911143	0.451783	1.837567	0.998587
genitourinary	Polyp of female genital organs	Wald ratio	1	0.025769	0.100214	0.797068	1.026104	0.843116	1.248807	0.998587
endocrine/metabolic	Disorders of the pituitary gland and its hypothalamic control	Wald ratio	1	-0.10021	0.393698	0.799074	0.904644	0.418172	1.957042	0.998587
dermatologic	Psoriatic arthropathy	Wald ratio	1	-0.10021	0.393698	0.799074	0.904644	0.418172	1.957042	0.998587
musculoskeletal	Other derangement of joint	Wald ratio	1	-0.06013	0.236219	0.799074	0.941644	0.59267	1.496098	0.998587
symptoms	Swelling of limb	Wald ratio	1	0.037938	0.150321	0.800747	1.038667	0.773607	1.394544	0.998587
digestive	Other chronic nonalcoholic liver disease	Wald ratio	1	0.064423	0.257693	0.802587	1.066544	0.643614	1.767387	0.998587
digestive	Cholecystitis without cholelithiasis	Wald ratio	1	-0.05011	0.200428	0.802587	0.951128	0.642142	1.408791	0.998587
genitourinary	Disorders of uterus, NEC	Wald ratio	1	-0.04438	0.178954	0.804134	0.95659	0.673593	1.358483	0.998587
neoplasms	Manlignant and unknown neoplasms of brain and nervous system	Wald ratio	1	-0.10021	0.408014	0.805981	0.904644	0.406601	2.012734	0.998587
musculoskeletal	Osteoporosis NOS	Wald ratio	1	0.035075	0.143163	0.806456	1.035697	0.782294	1.371183	0.998587
neurological	Extrapyramidal disease and abnormal movement disorders	Wald ratio	1	0.085898	0.350749	0.806535	1.089695	0.54795	2.167048	0.998587
neoplasms	Multiple myeloma	Wald ratio	1	0.107372	0.443805	0.80883	1.113349	0.466505	2.657086	0.998587
injuries & poisonings	Superficial injury without mention of infection	Wald ratio	1	0.037938	0.157479	0.809626	1.038667	0.762829	1.414247	0.998587
pregnancy complications	Placenta previa and abruptio placenta	Wald ratio	1	-0.07158	0.300642	0.811807	0.93092	0.516417	1.678125	0.998587
digestive	Intestinal malabsorption (non-celiac)	Wald ratio	1	-0.0544	0.229061	0.812269	0.947051	0.604496	1.483727	0.998587
injuries & poisonings	Adverse drug events and drug allergies	Wald ratio	1	0.093056	0.393698	0.813151	1.097523	0.507331	2.374303	0.998587
endocrine/metabolic	Disorders of fluid, electrolyte, and acid-base balance	Wald ratio	1	-0.02863	0.121688	0.81398	0.971773	0.765564	1.233526	0.998587
sense organs	Other disorders of middle ear and mastoid	Wald ratio	1	0.085898	0.365065	0.81398	1.089695	0.532789	2.228717	0.998587
genitourinary	Other nonmalignant breast conditions	Wald ratio	1	0.055118	0.236219	0.815503	1.056665	0.665065	1.678846	0.998587
digestive	Other disorders of gallbladder	Wald ratio	1	-0.06442	0.279168	0.817494	0.937608	0.542487	1.620517	0.998587
genitourinary	Urinary tract infection	Wald ratio	1	-0.02147	0.093056	0.817494	0.978754	0.815573	1.174585	0.998587
injuries & poisonings	Fracture of ribs	Wald ratio	1	-0.0859	0.372224	0.817494	0.917688	0.442438	1.903435	0.998587
genitourinary	Other disorders of urethra and urinary tract	Wald ratio	1	0.037938	0.164637	0.817754	1.038667	0.752202	1.434229	0.998587
dermatologic	Cellulitis and abscess of arm/hand	Wald ratio	1	-0.03293	0.143163	0.818092	0.967609	0.730865	1.281039	0.998587
genitourinary	Stricture/obstruction of ureter	Wald ratio	1	0.07874	0.343591	0.818739	1.081923	0.551729	2.121616	0.998587
dermatologic	Decubitus ulcer	Wald ratio	1	-0.07874	0.343591	0.818739	0.924281	0.471339	1.812485	0.998587
mental disorders	Other mental disorder	Wald ratio	1	0.015032	0.066571	0.821352	1.015146	0.890968	1.15663	0.998587
circulatory system	Stricture of artery	Wald ratio	1	-0.07874	0.350749	0.822376	0.924281	0.464772	1.838093	0.998587
respiratory	Abnormal sputum	Wald ratio	1	-0.04939	0.221902	0.823862	0.951809	0.616116	1.470405	0.998587
circulatory system	Cerebrovascular disease	Wald ratio	1	0.025054	0.11453	0.826845	1.02537	0.819201	1.283426	0.998587
circulatory system	Nonrheumatic mitral valve disorders	Wald ratio	1	-0.04223	0.19327	0.827025	0.958646	0.656362	1.400145	0.998587
injuries & poisonings	Nonspecific abnormal findings on radiological and other examination of mu	Wald ratio	1	-0.0859	0.393698	0.827287	0.917688	0.424202	1.985261	0.998587
infectious diseases	Viral warts & HPV	Wald ratio	1	-0.07158	0.329275	0.827903	0.93092	0.488234	1.774994	0.998587
respiratory	Acute upper respiratory infections of multiple or unspecified sites	Wald ratio	1	-0.04653	0.214744	0.828468	0.954538	0.626613	1.454076	0.998587
neurological	Parkinson's disease	Wald ratio	1	0.065855	0.3078	0.830583	1.068072	0.584246	1.952564	0.998587
circulatory system	Cerebral artery occlusion, with cerebral infarction	Wald ratio	1	-0.05798	0.27201	0.831204	0.943668	0.553707	1.608268	0.998587
circulatory system	Varicose veins of lower extremity, symptomtic	Wald ratio	1	-0.0859	0.415172	0.836091	0.917688	0.406718	2.070604	0.998587
respiratory	Voice disturbance	Wald ratio	1	0.063707	0.314958	0.839704	1.065781	0.57487	1.975904	0.998587
sense organs	Other disorders of eyelids	Wald ratio	1	-0.0272	0.136005	0.841481	0.973166	0.745448	1.270446	0.998587
musculoskeletal	Peripheral enthesopathies and allied syndromes	Wald ratio	1	0.01718	0.085898	0.841481	1.017328	0.859693	1.203867	0.998587
circulatory system	Hypertensive heart and/or renal disease	Wald ratio	1	0.049391	0.250535	0.843716	1.050631	0.64297	1.716762	0.998587
neoplasms	Lymphoid leukemia	Wald ratio	1	-0.0859	0.436647	0.844046	0.917688	0.389954	2.159615	0.998587
endocrine/metabolic	Disorders of parathyroid gland	Wald ratio	1	0.068718	0.350749	0.844674	1.071134	0.538617	2.130137	0.998587
endocrine/metabolic	Nontoxic nodular goiter	Wald ratio	1	-0.06013	0.3078	0.84512	0.941644	0.515088	1.721438	0.998587
respiratory	Septal Deviations/Turbinate Hypertrophy	Wald ratio	1	-0.02935	0.150321	0.845207	0.971078	0.723266	1.303797	0.998587
musculoskeletal	Intervertebral disc disorders	Wald ratio	1	0.02219	0.11453	0.846372	1.022438	0.816859	1.279756	0.998587
digestive	Esophagitis, GERD and related diseases	Wald ratio	1	-0.01217	0.062992	0.846817	0.987905	0.873163	1.117724	0.998587
sense organs	Amblyopia	Wald ratio	1	0.085898	0.450963	0.848936	1.089695	0.450233	2.637379	0.998587
digestive	Malposition and malpresentation of fetus or obstruction	Wald ratio	1	0.050107	0.264851	0.849945	1.051384	0.625627	1.766881	0.998587
genitourinary	Polyp of corpus uteri	Wald ratio	1	-0.02291	0.121688	0.850692	0.977354	0.769961	1.24061	0.998587
circulatory system	Occlusion of cerebral arteries	Wald ratio	1	-0.03078	0.164637	0.851695	0.969689	0.702248	1.338981	0.998587
neoplasms	Benign neoplasm of other parts of digestive system	Wald ratio	1	-0.02649	0.143163	0.853229	0.973863	0.735589	1.289319	0.998587
dermatologic	Cellulitis and abscess of foot, toe	Wald ratio	1	-0.02649	0.143163	0.853229	0.973863	0.735589	1.289319	0.998587
infectious diseases	Bacterial infection NOS	Wald ratio	1	0.01718	0.093056	0.853531	1.017328	0.847716	1.220877	0.998587
neoplasms	Breast cancer	Wald ratio	1	0.01718	0.093056	0.853531	1.017328	0.847716	1.220877	0.998587
musculoskeletal	Derangement of joint, non-traumatic	Wald ratio	1	-0.03507	0.19327	0.85599	0.965533	0.661078	1.410204	0.998587
circulatory system	Nonspecific abnormal findings on radiological and other examination of oth	Wald ratio	1	-0.07874	0.436647	0.856895	0.924281	0.392756	2.17513	0.998587
endocrine/metabolic	Hyperparathyroidism	Wald ratio	1	0.066571	0.372224	0.858059	1.068837	0.51531	2.216942	0.998587
neoplasms	Cancer within the respiratory system	Wald ratio	1	-0.03579	0.200428	0.858274	0.964842	0.651401	1.429105	0.998587
endocrine/metabolic	Gout	Wald ratio	1	0.032927	0.186112	0.859569	1.033476	0.717594	1.488407	0.998587
neoplasms	Uterine leiomyoma	Wald ratio	1	-0.01861	0.107372	0.862389	0.981561	0.79528	1.211475	0.998587
circulatory system	Peripheral vascular disease, unspecified	Wald ratio	1	0.035791	0.207586	0.863112	1.036439	0.68999	1.556842	0.998587
pregnancy complications	Umbilical cord complications during labor and delivery	Wald ratio	1	-0.07874	0.458121	0.863536	0.924281	0.376568	2.268634	0.998587
musculoskeletal	Pathologic fracture	Wald ratio	1	-0.07874	0.458121	0.863536	0.924281	0.376568	2.268634	0.998587
injuries & poisonings	Opiates and related narcotics causing adverse effects in therapeutic use	Wald ratio	1	0.052254	0.3078	0.865193	1.053644	0.576354	1.926188	0.998587
sense organs	Inflammation of the eye	Wald ratio	1	0.031496	0.186112	0.865615	1.031997	0.716567	1.486278	0.998587
hematopoietic	Disorders of iron metabolism	Wald ratio	1	0.066571	0.400856	0.868101	1.068837	0.487187	2.344914	0.998587
circulatory system	Noninfectious disorders of lymphatic channels	Wald ratio	1	-0.06442	0.393698	0.870017	0.937608	0.43341	2.028354	0.998587
sense organs	Strabismus (not specified as paralytic)	Wald ratio	1	-0.05583	0.343591	0.870912	0.945697	0.48226	1.854481	0.998587
mental disorders	Dementias	Wald ratio	1	-0.05369	0.336433	0.873216	0.94773	0.490125	1.832575	0.998587
respiratory	Allergic rhinitis	Wald ratio	1	-0.05082	0.322117	0.874632	0.950447	0.505518	1.786977	0.998587
dermatologic	Sebaceous cyst	Wald ratio	1	-0.0179	0.11453	0.875836	0.982264	0.784762	1.229471	0.998587
digestive	Hemorrhage of gastrointestinal tract	Wald ratio	1	-0.02219	0.143163	0.876821	0.978054	0.738755	1.294868	0.998587
hematopoietic	Megaloblastic anemia	Wald ratio	1	0.048675	0.314958	0.87718	1.049879	0.566293	1.946424	0.998587
hematopoietic	Pernicious anemia	Wald ratio	1	-0.05727	0.379382	0.88002	0.944344	0.448946	1.986398	0.998587
digestive	Anal and rectal conditions	Wald ratio	1	-0.01288	0.085898	0.880765	0.987198	0.834232	1.168213	0.998587
endocrine/metabolic	Acid-base balance disorder	Wald ratio	1	-0.0451	0.3078	0.883518	0.955905	0.52289	1.74751	0.998587
neoplasms	Lipoma of skin and subcutaneous tissue	Wald ratio	1	-0.02291	0.157479	0.884352	0.977354	0.717799	1.330764	0.998587
hematopoietic	Coagulation defects	Wald ratio	1	-0.04939	0.343591	0.885698	0.951809	0.485377	1.866467	0.998587
sense organs	Epiphora	Wald ratio	1	0.050107	0.350749	0.886403	1.051384	0.528686	2.09086	0.998587
genitourinary	Menopausal and postmenopausal disorders	Wald ratio	1	0.014316	0.100214	0.886403	1.014419	0.833515	1.234586	0.998587
sense organs	Open-angle glaucoma	Wald ratio	1	-0.04581	0.322117	0.886904	0.955221	0.508058	1.795954	0.998587
endocrine/metabolic	Vitamin deficiency	Wald ratio	1	0.041517	0.300642	0.890165	1.042391	0.578254	1.879068	0.998587
circulatory system	Unstable angina (intermediate coronary syndrome)	Wald ratio	1	0.020759	0.150321	0.890165	1.020976	0.76043	1.370791	0.998587
circulatory system	Disease of tricuspid valve	Wald ratio	1	0.04438	0.322117	0.890416	1.04538	0.556011	1.965465	0.998587
dermatologic	Diseases of sebaceous glands	Wald ratio	1	-0.01575	0.11453	0.890636	0.984375	0.786449	1.232114	0.998587
circulatory system	Atrioventricular [AV] block	Wald ratio	1	-0.03078	0.229061	0.893106	0.969689	0.618945	1.519192	0.998587
circulatory system	Other venous embolism and thrombosis	Wald ratio	1	-0.0587	0.443805	0.89478	0.942993	0.395124	2.250519	0.998587
sense organs	Retinal vascular changes and abnomalities	Wald ratio	1	-0.04653	0.357907	0.896566	0.954538	0.4733	1.925085	0.998587
sense organs	Other disorders of tympanic membrane	Wald ratio	1	0.037222	0.286326	0.896566	1.037924	0.592161	1.819244	0.998587
circulatory system	Abnormal function study of cardiovascular system	Wald ratio	1	-0.0587	0.458121	0.89805	0.942993	0.384191	2.314563	0.998587
circulatory system	Late effects of cerebrovascular disease	Wald ratio	1	0.037222	0.293484	0.899076	1.037924	0.583911	1.844948	0.998587
musculoskeletal	Rheumatism, unspecified and fibrositis	Wald ratio	1	-0.04152	0.329275	0.899663	0.959333	0.503136	1.829168	0.998587
genitourinary	Other specified benign mammary dysplasias	Wald ratio	1	-0.04939	0.393698	0.900164	0.951809	0.439974	2.059075	0.998587
digestive	Gingival and periodontal diseases	Wald ratio	1	-0.03078	0.250535	0.90222	0.969689	0.593434	1.5845	0.998587
digestive	Abdominal hernia	Wald ratio	1	0.006299	0.051539	0.902723	1.006319	0.909631	1.113285	0.998587
genitourinary	Other disorders of male genital organs	Wald ratio	1	-0.02434	0.200428	0.903352	0.975956	0.658904	1.445567	0.998587
sense organs	Disorders of conjunctiva	Wald ratio	1	0.045812	0.379382	0.903885	1.046878	0.497691	2.202075	0.998587
respiratory	Other symptoms of respiratory system	Wald ratio	1	-0.0136	0.11453	0.905473	0.986492	0.78814	1.234763	0.998587
genitourinary	Noninflammatory disorders of vagina	Wald ratio	1	-0.02935	0.250535	0.906747	0.971078	0.594284	1.58677	0.998587

neoplasms	Breast cancer [female]	Wald ratio	1	0.011453	0.100214	0.909011	1.011519	0.831132	1.231056	0.998587
injuries & poisonings	Fracture of radius and ulna	Wald ratio	1	-0.01718	0.150321	0.909011	0.982967	0.732122	1.31976	0.998587
neoplasms	Myeloproliferative disease	Wald ratio	1	0.037222	0.329275	0.909996	1.037924	0.544354	1.979018	0.998587
musculoskeletal	Hallux rigidus	Wald ratio	1	-0.02935	0.264851	0.911766	0.971078	0.577841	1.631925	0.998587
genitourinary	Urethral stricture (not specified as infectious)	Wald ratio	1	-0.01933	0.178954	0.913996	0.980859	0.690682	1.392948	0.998587
respiratory	Empyema and pneumothorax	Wald ratio	1	-0.03293	0.3078	0.914807	0.967609	0.529292	1.768905	0.998587
musculoskeletal	Arthropathy NOS	Wald ratio	1	0.006371	0.060128	0.91562	1.006391	0.894508	1.132268	0.998587
pregnancy complications	Hypertension complicating pregnancy, childbirth, and the puerperium	Wald ratio	1	0.033643	0.322117	0.916817	1.034216	0.550073	1.944474	0.998587
genitourinary	Mucous polyp of cervix	Wald ratio	1	0.018611	0.178954	0.917169	1.018785	0.717388	1.446809	0.998587
musculoskeletal	Enthesopathy	Wald ratio	1	-0.01074	0.107372	0.920344	0.98932	0.801567	1.221051	0.998587
mental disorders	Aphasia/speech disturbance	Wald ratio	1	-0.02649	0.27201	0.922434	0.973863	0.571424	1.659728	0.998587
musculoskeletal	Hammer toe (acquired)	Wald ratio	1	-0.02291	0.236219	0.92275	0.977354	0.615146	1.552836	0.998587
circulatory system	Pericarditis	Wald ratio	1	0.027917	0.293484	0.924218	1.02831	0.578503	1.827859	0.998587
genitourinary	Uterine/Uterovaginal prolapse	Wald ratio	1	-0.0136	0.143163	0.924315	0.986492	0.745128	1.306039	0.998587
injuries & poisonings	Intracranial hemorrhage (injury)	Wald ratio	1	0.043665	0.465279	0.925231	1.044632	0.419672	2.600262	0.998587
endocrine/metabolic	Diabetic retinopathy	Wald ratio	1	-0.02649	0.286326	0.926301	0.973863	0.555613	1.706959	0.998587
musculoskeletal	Other disorders of bone and cartilage	Wald ratio	1	0.015748	0.171795	0.926963	1.015873	0.725444	1.422573	0.998587
neoplasms	Cancer of esophagus	Wald ratio	1	0.035075	0.38654	0.927699	1.035697	0.485516	2.209338	0.998587
injuries & poisonings	Fracture of pelvis	Wald ratio	1	0.033643	0.38654	0.930642	1.034216	0.484821	2.206177	0.998587
genitourinary	Ovarian cyst	Wald ratio	1	-0.01288	0.150321	0.931694	0.987198	0.735273	1.32544	0.998587
respiratory	Shortness of breath	Wald ratio	1	0.011453	0.136005	0.932889	1.011519	0.774826	1.320516	0.998587
respiratory	Chronic sinusitis	Wald ratio	1	-0.01646	0.207586	0.936786	0.983671	0.654861	1.477579	0.998587
sense organs	Primary open angle glaucoma	Wald ratio	1	-0.02505	0.322117	0.938005	0.975258	0.518714	1.833625	0.998587
genitourinary	Cyst or abscess of Bartholin's gland	Wald ratio	1	0.029348	0.379382	0.938338	1.029783	0.489564	2.166118	0.998587
digestive	Anal and rectal polyp	Wald ratio	1	-0.00931	0.121688	0.939045	0.990738	0.780504	1.257598	0.998587
respiratory	Respiratory failure, insufficiency, arrest	Wald ratio	1	0.015748	0.207586	0.939529	1.015873	0.676298	1.52595	0.998587
pregnancy complications	Hemorrhage during pregnancy; childbirth and postpartum	Wald ratio	1	0.017895	0.243377	0.941385	1.018056	0.631837	1.640357	0.998587
digestive	Nonspecific abnormal findings in stool contents	Wald ratio	1	-0.01861	0.257693	0.942425	0.981561	0.592331	1.62656	0.998587
neoplasms	Chemotherapy	Wald ratio	1	0.005154	0.071581	0.942602	1.005167	0.873589	1.156564	0.998587
circulatory system	Angina pectoris	Wald ratio	1	0.00587	0.085898	0.94552	1.005887	0.850025	1.190328	0.998587
neoplasms	Colon cancer	Wald ratio	1	-0.01288	0.19327	0.946847	0.987198	0.675911	1.441846	0.998587
neurological	Sleep disorders	Wald ratio	1	-0.01002	0.150321	0.946847	0.990029	0.737381	1.329241	0.998587
neurological	Epilepsy, recurrent seizures, convulsions	Wald ratio	1	0.010021	0.150321	0.946847	1.010072	0.752309	1.356151	0.998587
circulatory system	Cardiac conduction disorders	Wald ratio	1	-0.00859	0.128847	0.946847	0.991447	0.770181	1.27628	0.998587
musculoskeletal	Aseptic necrosis of bone	Wald ratio	1	-0.02935	0.443805	0.947275	0.971078	0.406893	2.317547	0.998587
respiratory	Hemoptysis	Wald ratio	1	0.015032	0.229061	0.947676	1.015146	0.64796	1.590409	0.998587
endocrine/metabolic	Abnormal glucose	Wald ratio	1	-0.02505	0.400856	0.950165	0.975258	0.444533	2.139612	0.998587
digestive	Heartburn	Wald ratio	1	0.014316	0.229061	0.950165	1.014419	0.647496	1.589271	0.998587
injuries & poisonings	Fracture of patella	Wald ratio	1	-0.02362	0.379382	0.950352	0.976655	0.464307	2.054364	0.998587
symptoms	Syncope and collapse	Wald ratio	1	0.006585	0.107372	0.951094	1.006607	0.815573	1.242388	0.998587
digestive	Diseases of esophagus	Wald ratio	1	0.003651	0.060128	0.951587	1.003657	0.892079	1.129192	0.998587
injuries & poisonings	Foreign body injury	Wald ratio	1	-0.01718	0.300642	0.954431	0.982967	0.54529	1.771947	0.998587
endocrine/metabolic	Hypovolemia	Wald ratio	1	0.011453	0.200428	0.954431	1.011519	0.682914	1.498241	0.998587
mental disorders	Phobia	Wald ratio	1	0.026485	0.465279	0.954606	1.026839	0.412523	2.555973	0.998587
symptoms	Myalgia and myositis unspecified	Wald ratio	1	0.022906	0.415172	0.956001	1.02317	0.453467	2.308606	0.998587
neurological	Sleep apnea	Wald ratio	1	0.00859	0.157479	0.956501	1.008627	0.740767	1.373344	0.998587
digestive	Inflammatory bowel disease and other gastroenteritis and colitis	Wald ratio	1	-0.00787	0.157479	0.960122	0.992157	0.728671	1.350919	0.999088
pregnancy complications	Other complications of pregnancy NEC	Wald ratio	1	0.010737	0.229061	0.962613	1.010795	0.645183	1.583593	0.999088
circulatory system	Peripheral vascular disease	Wald ratio	1	0.007874	0.171795	0.963443	1.007905	0.719754	1.411415	0.999088
genitourinary	Postmenopausal atrophic vaginitis	Wald ratio	1	-0.01432	0.314958	0.963745	0.985786	0.531722	1.827597	0.999088
mental disorders	Schizophrenia	Wald ratio	1	-0.01933	0.436647	0.964695	0.980859	0.416797	2.308276	0.999088
digestive	Paralytic ileus	Wald ratio	1	0.020043	0.458121	0.965104	1.020245	0.415665	2.504178	0.999088
mental disorders	Schizophrenia and other psychotic disorders	Wald ratio	1	-0.01503	0.357907	0.966499	0.98508	0.488444	1.986682	0.999088
respiratory	Respiratory failure	Wald ratio	1	-0.00859	0.236219	0.970992	0.991447	0.624016	1.575226	0.999088
digestive	Dysphagia	Wald ratio	1	-0.00444	0.128847	0.972523	0.995572	0.773386	1.28159	0.999088
digestive	Ulcerative colitis	Wald ratio	1	-0.00637	0.186112	0.972693	0.99365	0.689941	1.43105	0.999088
neoplasms	Benign neoplasm of uterus	Wald ratio	1	-0.00358	0.107372	0.973409	0.996427	0.807325	1.229823	0.999088
respiratory	Other diseases of respiratory system, not elsewhere classified	Wald ratio	1	0.003436	0.107372	0.974472	1.003442	0.813009	1.238481	0.999088
musculoskeletal	Osteoarthrosis	Wald ratio	1	-0.002	0.066571	0.975981	0.997998	0.875918	1.137092	0.999088
genitourinary	Postmenopausal bleeding	Wald ratio	1	-0.00308	0.11453	0.978559	0.996927	0.796477	1.247824	0.999088
sense organs	Subjective visual disturbances	Wald ratio	1	-0.01074	0.422331	0.979717	0.98932	0.432356	2.263768	0.999088
dermatologic	Unspecified diffuse connective tissue disease	Wald ratio	1	0.004868	0.200428	0.980625	1.004879	0.678432	1.488407	0.999088
neurological	Inflammatory and toxic neuropathy	Wald ratio	1	-0.00637	0.279168	0.981793	0.99365	0.574911	1.717377	0.999088
injuries & poisonings	Fracture of upper limb	Wald ratio	1	0.002434	0.11453	0.983046	1.002437	0.800879	1.254721	0.999088
genitourinary	Benign mammary dysplasias	Wald ratio	1	0.003364	0.178954	0.985001	1.00337	0.706533	1.424917	0.999088
musculoskeletal	Other arthropathies	Wald ratio	1	-0.001	0.058697	0.986378	0.998998	0.890433	1.120801	0.999088
neoplasms	Acquired absence of breast	Wald ratio	1	-0.00301	0.214744	0.98883	0.996998	0.654486	1.518757	0.999088
musculoskeletal	Rupture of synovium	Wald ratio	1	0.006013	0.429489	0.98883	1.006031	0.433534	2.33453	0.999088
musculoskeletal	Osteomyelitis, periostitis, and other infections involving bone	Wald ratio	1	-0.00573	0.422331	0.989182	0.99429	0.434528	2.275139	0.999088
endocrine/metabolic	Anorexia	Wald ratio	1	-0.00372	0.357907	0.991702	0.996285	0.494	2.009279	0.999088
digestive	Abnormal results of function study of liver	Wald ratio	1	0.001432	0.178954	0.993617	1.001433	0.705169	1.422166	0.999088
circulatory system	Premature beats	Wald ratio	1	-0.00351	0.450963	0.993794	0.996499	0.411727	2.411817	0.999088
digestive	Umbilical hernia	Wald ratio	1	0.001074	0.171795	0.995013	1.001074	0.714877	1.40185	0.999088
digestive	Inguinal hernia	Wald ratio	1	-0.00052	0.085898	0.995146	0.999478	0.844608	1.182744	0.999088
respiratory	Respiratory insufficiency	Wald ratio	1	-0.00086	0.250535	0.997264	0.999141	0.611459	1.632626	0.999088
circulatory system	Cardiomegaly	Wald ratio	1	-0.0007	0.207586	0.997304	0.999299	0.665265	1.501054	0.999088
neoplasms	Non-Hodgkins lymphoma	Wald ratio	1	-0.00079	0.250535	0.997492	0.999213	0.611503	1.632743	0.999088
digestive	Ulcer of esophagus	Wald ratio	1	0.000186	0.150321	0.999012	1.000186	0.744946	1.342878	0.999088
digestive	Regional enteritis	Wald ratio	1	0.000286	0.250535	0.999088	1.000286	0.612159	1.634497	0.999088

Table S16. The phenome-wide MR analysis results of MMP12 (IVW or Wald ratio methods).

Category	Phenostring	Method	Nsnp	beta	se	P value	OR	LL	LR	FDR
injuries & poisonings	Mechanical complication of unspecified genitourinary device, implant, and graft	Inverse variance weighted	2	-0.24199	0.075914	0.001434	0.78506	0.676525	0.911008	0.593718
injuries & poisonings	Complication of colostomy or enterostomy	Inverse variance weighted	2	-0.37985	0.119769	0.001517	0.683965	0.540859	0.864935	0.593718
neurological	Convulsions	Inverse variance weighted	2	-0.16889	0.060916	0.005564	0.844606	0.749551	0.951715	0.92159
symptoms	Pain in limb	Inverse variance weighted	2	0.096135	0.035981	0.007545	1.100907	1.025942	1.18135	0.92159
circulatory system	Nonspecific chest pain	Inverse variance weighted	2	-0.04258	0.016184	0.008505	0.958309	0.928389	0.989194	0.92159
circulatory system	Occlusion and stenosis of precerebral arteries	Inverse variance weighted	2	-0.2001	0.077393	0.009724	0.818651	0.703429	0.952745	0.92159
endocrine/metabolic	Hyperlipidemia	Inverse variance weighted	2	-0.04158	0.016184	0.010187	0.95927	0.929319	0.990186	0.92159
endocrine/metabolic	Disorders of lipoid metabolism	Inverse variance weighted	2	-0.04124	0.016184	0.010827	0.959599	0.929638	0.990525	0.92159
congenital anomalies	Cardiac shunt/ heart septal defect	Inverse variance weighted	2	-0.27677	0.109764	0.011686	0.75823	0.611459	0.94023	0.92159
circulatory system	Abdominal aortic aneurysm	Inverse variance weighted	2	0.225697	0.089753	0.011915	1.253196	1.051041	1.494233	0.92159
neurological	Abnormality of gait	Inverse variance weighted	2	-0.16592	0.067388	0.013809	0.847111	0.742298	0.966723	0.92159
endocrine/metabolic	Hypercholesterolemia	Inverse variance weighted	2	-0.03972	0.016184	0.014124	0.961062	0.931055	0.992036	0.92159
sense organs	Ectropion or entropion	Inverse variance weighted	2	-0.18956	0.082087	0.020933	0.827327	0.704373	0.971743	0.996292
musculoskeletal	Hallux rigidus	Inverse variance weighted	2	0.157868	0.068566	0.02131	1.171012	1.023757	1.339446	0.996292
respiratory	Other diseases of respiratory system, NEC	Inverse variance weighted	2	-0.0656	0.028662	0.022095	0.936506	0.885346	0.990622	0.996292
respiratory	Other diseases of respiratory system, not elsewhere classified	Inverse variance weighted	2	-0.05999	0.027426	0.028707	0.94177	0.892481	0.99378	0.996292
injuries & poisonings	Skull and face fracture and other intercranial injury	Inverse variance weighted	2	-0.10862	0.049849	0.029336	0.897073	0.81357	0.989145	0.996292
neoplasms	Benign neoplasm of other endocrine glands and related structures	Inverse variance weighted	2	0.192189	0.089753	0.032249	1.211899	1.016406	1.444993	0.996292
genitourinary	Other nonmalignant breast conditions	Inverse variance weighted	2	-0.13063	0.061205	0.032817	0.877541	0.778338	0.989388	0.996292
genitourinary	Prolapse of vaginal walls	Inverse variance weighted	2	0.065799	0.031193	0.034909	1.068012	1.004671	1.135345	0.996292
musculoskeletal	Acquired toe deformities	Inverse variance weighted	2	0.078128	0.037402	0.03672	1.081261	1.004831	1.163504	0.996292
sense organs	Myopia	Inverse variance weighted	2	0.158085	0.075914	0.037304	1.171266	1.009337	1.359173	0.996292
hematopoietic	Anemia of chronic disease	Inverse variance weighted	2	-0.2063	0.100934	0.040961	0.813588	0.667556	0.991565	0.996292
pregnancy complications	Hemorrhage during pregnancy; childbirth and postpartum	Inverse variance weighted	2	-0.12604	0.062652	0.044254	0.881583	0.77971	0.996768	0.996292
digestive	Diverticulosis and diverticulitis	Inverse variance weighted	2	0.035126	0.017473	0.044406	1.03575	1.000878	1.071837	0.996292
digestive	Diverticulosis	Inverse variance weighted	2	0.034999	0.01745	0.044894	1.035619	1.000797	1.071652	0.996292
dermatologic	Cellulitis and abscess of fingers/toes	Inverse variance weighted	2	-0.22486	0.112114	0.0449	0.798632	0.641081	0.994902	0.996292
musculoskeletal	Joint effusions	Inverse variance weighted	2	-0.1419	0.070909	0.045378	0.867709	0.755119	0.997087	0.996292
neoplasms	Hemangioma and lymphangioma, any site	Inverse variance weighted	2	-0.1408	0.072628	0.052538	0.868659	0.753402	1.001548	0.996292
endocrine/metabolic	Secondary hypothyroidism	Inverse variance weighted	2	0.155204	0.080919	0.055107	1.167897	0.99661	1.368623	0.996292
injuries & poisonings	Fracture of lower limb	Inverse variance weighted	2	-0.06659	0.034951	0.056764	0.935582	0.873637	1.00192	0.996292
neoplasms	Nevus, non-neoplastic	Inverse variance weighted	2	-0.20872	0.109764	0.057235	0.811624	0.654518	1.006441	0.996292
sense organs	Disorders of refraction and accommodation; blindness and low vision	Inverse variance weighted	2	0.101781	0.053555	0.057369	1.107141	0.996818	1.229675	0.996292
neoplasms	Cancer of brain and nervous system	Inverse variance weighted	2	-0.28037	0.148129	0.058395	0.755506	0.565129	1.010015	0.996292
circulatory system	Cardiac arrest	Inverse variance weighted	2	0.165192	0.087397	0.05874	1.17962	0.993913	1.400026	0.996292
circulatory system	Varicose veins of lower extremity	Inverse variance weighted	2	0.049335	0.02616	0.059308	1.050573	0.998064	1.105844	0.996292
dermatologic	Diseases of sebaceous glands	Inverse variance weighted	2	0.054041	0.028662	0.059366	1.055528	0.997866	1.116522	0.996292
circulatory system	Hypertension	Inverse variance weighted	2	-0.02263	0.012095	0.061295	0.977621	0.954718	1.001073	0.996292
injuries & poisonings	Fracture of ankle and foot	Inverse variance weighted	2	-0.10454	0.05589	0.061426	0.900741	0.807283	1.005019	0.996292
mental disorders	Depression	Inverse variance weighted	2	-0.04667	0.024954	0.061475	0.954406	0.908849	1.002247	0.996292
sense organs	Primary angle-closure glaucoma	Inverse variance weighted	2	0.188195	0.100934	0.062246	1.207069	0.990411	1.471123	0.996292
musculoskeletal	Other acquired musculoskeletal deformity	Inverse variance weighted	2	0.113772	0.061205	0.063048	1.120497	0.993829	1.26331	0.996292
circulatory system	Cerebrovascular disease	Inverse variance weighted	2	-0.05909	0.031795	0.063096	0.942621	0.885673	1.003232	0.996292
dermatologic	Sebaceous cyst	Inverse variance weighted	2	0.053215	0.028662	0.063363	1.054656	0.997042	1.115599	0.996292
digestive	Constipation	Inverse variance weighted	2	0.048308	0.02619	0.065113	1.049493	0.996979	1.104774	0.996292
circulatory system	Essential hypertension	Inverse variance weighted	2	-0.02226	0.012095	0.065728	0.977988	0.955077	1.001449	0.996292
neoplasms	Other benign neoplasm of connective and other soft tissue	Inverse variance weighted	2	-0.14655	0.079746	0.066103	0.863681	0.738706	1.009799	0.996292
symptoms	Cervicalgia	Inverse variance weighted	2	-0.11665	0.063562	0.066477	0.889897	0.78566	1.007965	0.996292
mental disorders	Mood disorders	Inverse variance weighted	2	-0.04561	0.024893	0.066908	0.955413	0.909917	1.003184	0.996292
circulatory system	Varicose veins	Inverse variance weighted	2	0.045625	0.024954	0.067496	1.046682	0.99672	1.099149	0.996292
circulatory system	Hemorrhoids	Inverse variance weighted	2	0.034089	0.018653	0.067624	1.034676	0.997532	1.073204	0.996292
digestive	Duodenal ulcer	Inverse variance weighted	2	-0.0905	0.049818	0.069273	0.913473	0.828495	1.007168	0.996292
genitourinary	Urethral stricture (not specified as infectious)	Inverse variance weighted	2	-0.08353	0.046142	0.070257	0.919865	0.840324	1.006934	0.996292
musculoskeletal	Intervertebral disc disorders	Inverse variance weighted	2	0.051642	0.028662	0.071581	1.052999	0.995475	1.113846	0.996292
circulatory system	Heart valve replaced	Inverse variance weighted	2	-0.12355	0.069739	0.076471	0.883781	0.770871	1.01323	0.996292
congenital anomalies	Congenital anomalies of urinary system	Inverse variance weighted	2	-0.16057	0.090927	0.07741	0.851659	0.712635	1.017804	0.996292
injuries & poisonings	Poisoning by anticonvulsants and anti-Parkinsonism drugs	Inverse variance weighted	2	-0.21118	0.119769	0.07786	0.809627	0.640229	1.023846	0.996292
mental disorders	Schizophrenia and other psychotic disorders	Inverse variance weighted	2	-0.21327	0.121078	0.07817	0.80794	0.637258	1.024337	0.996292
neoplasms	Manlignant and unknown neoplasms of brain and nervous system	Inverse variance weighted	2	-0.23861	0.13581	0.078933	0.787725	0.603629	1.027966	0.996292
circulatory system	Cardiac arrest and ventricular fibrillation	Inverse variance weighted	2	0.13974	0.079746	0.079722	1.149974	0.983573	1.344528	0.996292
musculoskeletal	Unspecified monoarthritis	Inverse variance weighted	2	0.038974	0.022423	0.082189	1.039743	0.995037	1.086458	0.996292
circulatory system	Peripheral vascular disease	Inverse variance weighted	2	-0.07554	0.043579	0.08301	0.92724	0.851328	1.00992	0.996292
digestive	Celiac disease	Inverse variance weighted	2	-0.10807	0.062386	0.083228	0.897566	0.79426	1.014309	0.996292
symptoms	Sciatica	Inverse variance weighted	2	0.094605	0.054725	0.083857	1.099225	0.987424	1.223684	0.996292
mental disorders	Delirium dementia and amnestic and other cognitive disorders	Inverse variance weighted	2	-0.11049	0.064095	0.084738	0.895396	0.789689	1.015252	0.996292
digestive	Other specified gastritis	Inverse variance weighted	2	0.051407	0.029928	0.08585	1.052751	0.992775	1.116351	0.996292
neoplasms	Vascular hamartomas and non-neoplastic nevi	Inverse variance weighted	2	-0.22417	0.131825	0.089028	0.799176	0.617207	1.034794	0.996292
infectious diseases	Intestinal infection due to C. difficile	Inverse variance weighted	2	-0.17695	0.104762	0.091209	0.837823	0.682303	1.028791	0.996292
neoplasms	Secondary malignant neoplasm of liver	Inverse variance weighted	2	-0.09296	0.055083	0.091468	0.911227	0.817973	1.015112	0.996292
dermatologic	Erythematous conditions	Inverse variance weighted	2	0.092043	0.054725	0.092585	1.096412	0.984897	1.220553	0.996292
digestive	Ventral hernia	Inverse variance weighted	2	-0.07745	0.046081	0.092804	0.925471	0.845547	1.012949	0.996292
neurological	Epilepsy	Inverse variance weighted	2	-0.14825	0.088576	0.094201	0.86222	0.724803	1.025689	0.996292
injuries & poisonings	Adverse drug events and drug allergies	Inverse variance weighted	2	-0.22322	0.133625	0.094825	0.799941	0.615622	1.039446	0.996292
genitourinary	Other disorders of urethra and urinary tract	Inverse variance weighted	2	-0.07042	0.042355	0.096549	0.932003	0.857723	1.012715	0.996292
circulatory system	Hypotension NOS	Inverse variance weighted	2	-0.07436	0.044876	0.097517	0.928337	0.85017	1.01369	0.996292
neurological	Epilepsy, recurrent seizures, convulsions	Inverse variance weighted	2	-0.06196	0.037402	0.097609	0.939922	0.873483	1.011414	0.996292
circulatory system	Aortic aneurysm	Inverse variance weighted	2	0.156542	0.096242	0.103834	1.169459	0.968417	1.412238	0.996292
injuries & poisonings	Opiates and related narcotics causing adverse effects in therapeutic use	Inverse variance weighted	2	0.129621	0.079746	0.104074	1.138397	0.973671	1.330992	0.996292
dermatologic	Carbuncle and furuncle	Inverse variance weighted	2	0.097425	0.059955	0.104169	1.102329	0.980114	1.239784	0.996292
digestive	Symptoms involving digestive system	Inverse variance weighted	2	0.034457	0.021217	0.104371	1.035057	0.992897	1.079008	0.996292
endocrine/metabolic	Anorexia	Inverse variance weighted	2	-0.14867	0.092098	0.106463	0.861851	0.71951	1.032351	0.996292
circulatory system	Vascular insufficiency of intestine	Inverse variance weighted	2	-0.17812	0.11094	0.108378	0.836844	0.673302	1.040109	0.996292
circulatory system	Atrial fibrillation and flutter	Inverse variance weighted	2	-0.03732	0.023658	0.11466	0.963365	0.919714	1.009088	0.996292
symptoms	Pain	Inverse variance weighted	2	0.151007	0.09593	0.115457	1.163005	0.96366	1.403587	0.996292
neoplasms	Uterine leiomyoma	Inverse variance weighted	2	-0.04284	0.027461	0.118748	0.958064	0.907861	1.011044	0.996292
circulatory system	Other specified cardiac dysrhythmias	Inverse variance weighted	2	-0.083	0.053509	0.120881	0.920354	0.828719	1.022122	0.996292
genitourinary	Other disorders of bladder	Inverse variance weighted	2	-0.04234	0.027426	0.122613	0.958541	0.908374	1.011477	0.996292
circulatory system	Nonrheumatic mitral valve disorders	Inverse variance weighted	2	0.076715	0.049849	0.123821	1.079734	0.979229	1.190554	0.996292
injuries & poisonings	Complication due to other implant and internal device	Inverse variance weighted	2	-0.0727	0.047348	0.124698	0.929884	0.847473	1.02031	0.996292
endocrine/metabolic	Diabetes mellitus	Inverse variance weighted	2	-0.05814	0.038191	0.127904	0.943515	0.875467	1.016852	0.996292
circulatory system	Rheumatic disease of the heart valves	Inverse variance weighted	2	0.065168	0.042817	0.128004	1.067339	0.981422	1.160777	0.996292
digestive	Cholecystitis without cholelithiasis	Inverse variance weighted	2	0.077733	0.051115	0.128327	1.080834	0.977797	1.194728	0.996292
digestive	Diseases of the salivary glands	Inverse variance weighted	2	-0.15072	0.099759	0.13083	0.860089	0.707338	1.045827	0.996292
circulatory system	Peripheral vascular disease, unspecified	Inverse variance weighted	2	-0.09078	0.060096	0.130878	0.913215	0.811744	1.027372	0.996292
injuries & poisonings	Fracture of unspecified bones	Inverse variance weighted	2	0.110771	0.073568	0.132148	1.117139	0.967129	1.290417	0.996292
circulatory system	Cardiac dysrhythmias	Inverse variance weighted	2	-0.02802	0.018653	0.133022	0.972366	0.937459	1.008574	0.996292
circulatory system	Paroxysmal supraventricular tachycardia	Inverse variance weighted	2	-0.08984	0.060284	0.136139	0.914075	0.812207	1.028719	0.996292
endocrine/metabolic	Type 2 diabetes	Inverse variance weighted	2	-0.06038	0.040649	0.137439	0.941406	0.869312	1.01948	0.996292
injuries & poisonings	Nonspecific abnormal findings on radiological and other examination of musculoskeletal system	Inverse variance weighted	2	-0.23096	0.15581	0.138251	0.793769	0.584879	1.077264	0.996292
dermatologic	Actinic keratosis	Inverse variance weighted	2	0.077391	0.052351	0.139324	1.080464	0.975099	1.197216	0.996292
endocrine/metabolic	Hypovolemia	Inverse variance weighted	2	-0.07535	0.051053	0.139948	0.927415	0.839106	1.025018	0.996292
congenital anomalies	Congenital anomalies of genital organs	Inverse variance weighted	2	0.187158	0.126938	0.14037	1.205818	0.940222	1.546441	0.996292
musculoskeletal	Ganglion and cyst of synovium, tendon, and bursa	Inverse variance weighted	2	-0.06972	0.047348	0.140909	0.932659	0.850002	1.023355	0.996292
musculoskeletal	Acquired spondylolisthesis	Inverse variance weighted	2	0.100697	0.068566	0.141933	1.105942	0.96687	1.265017	0.996292
symptoms	Fever of unknown origin	Inverse variance weighted	2	0.061816	0.042375	0.144626	1.063766	0.978985	1.15589	0.996292
mental disorders	Other mental disorder	Inverse variance weighted	2	-0.02515	0.017419	0.148801	0.975165	0.942434	1.009032	0.996292
digestive	Malposition and malpresentation of fetus or obstruction	Inverse variance weighted	2	0.097122	0.067388	0.149521	1.101995	0.965645	1.257597	0.996292
pregnancy complications	Antepartum hemorrhage, abruptio placentae, and placenta previa	Inverse variance weighted	2	-0.15384	0.106839	0.14989	0.85741	0.695418	1.057137	0.996292
musculoskeletal	Symptoms and disorders of the joints	Inverse variance weighted	2	-0.06437	0.044846	0.151152	0.937653	0.858754	1.023802	0.996292
circulatory system	Intracranial hemorrhage	Inverse variance weighted	2	0.090853	0.063562	0.152905	1.095108	0.966833	1.240401	0.996292
digestive	Peptic ulcer (excl. esophageal)	Inverse variance weighted	2	-0.04442	0.031163	0.154053	0.956553	0.899875	1.016801	0.996292

digestive	Blood in stool	Inverse variance weighted	2	-0.07447	0.052351	0.15487	0.928234	0.837714	1.028536	0.996292
mental disorders	Neurological disorders	Inverse variance weighted	2	-0.08118	0.057301	0.156568	0.922029	0.82408	1.031621	0.996292
digestive	Intestinal malabsorption (non-celiac)	Inverse variance weighted	2	-0.08267	0.058559	0.158015	0.920653	0.820823	1.032624	0.996292
neoplasms	Chemotherapy	Inverse variance weighted	2	-0.02637	0.018686	0.158175	0.973975	0.938949	1.010307	0.996292
dermatologic	Other local infections of skin and subcutaneous tissue	Inverse variance weighted	2	0.055927	0.039873	0.160732	1.05752	0.97802	1.143482	0.996292
digestive	Abnormal results of function study of liver	Inverse variance weighted	2	0.068773	0.049067	0.161033	1.071193	0.972973	1.179328	0.996292
neoplasms	Benign neoplasm of uterus	Inverse variance weighted	2	-0.04031	0.028933	0.163532	0.960489	0.907537	1.016532	0.996292
musculoskeletal	Curvature of spine	Inverse variance weighted	2	0.11055	0.079746	0.165664	1.116893	0.955278	1.305849	0.996292
pregnancy complications	Hemorrhage in early pregnancy	Inverse variance weighted	2	-0.18594	0.134658	0.167337	0.830326	0.637713	1.081114	0.996292
musculoskeletal	Kyphoscoliosis and scoliosis	Inverse variance weighted	2	0.112986	0.082087	0.168696	1.119616	0.953224	1.315053	0.996292
injuries & poisonings	Fracture of vertebral column without mention of spinal cord injury	Inverse variance weighted	2	0.099476	0.072391	0.169394	1.104592	0.958477	1.272981	0.996292
circulatory system	Premature beats	Inverse variance weighted	2	-0.25034	0.182651	0.170496	0.778534	0.544255	1.11366	0.996292
dermatologic	Psoriatic arthropathy	Inverse variance weighted	2	0.156756	0.114369	0.170497	1.16971	0.934813	1.46363	0.996292
circulatory system	Cerebral artery occlusion, with cerebral infarction	Inverse variance weighted	2	-0.09521	0.069739	0.172182	0.909182	0.793027	1.042351	0.996292
mental disorders	Memory loss	Inverse variance weighted	2	-0.17269	0.126837	0.173362	0.841401	0.656201	1.07887	0.996292
dermatologic	Degenerative skin conditions and other dermatoses	Inverse variance weighted	2	0.048988	0.036166	0.175567	1.050208	0.97834	1.127355	0.996292
mental disorders	Alcoholism	Inverse variance weighted	2	-0.04449	0.032898	0.1763	0.956489	0.896762	1.020195	0.996292
endocrine/metabolic	Gout	Inverse variance weighted	2	0.076911	0.056996	0.177202	1.079946	0.965798	1.207586	0.996292
genitourinary	Chronic cystitis	Inverse variance weighted	2	-0.12051	0.089753	0.179384	0.886471	0.743473	1.056973	0.996292
neurological	Other paralytic syndromes	Inverse variance weighted	2	0.139867	0.104762	0.181844	1.150121	0.936631	1.412272	0.996292
circulatory system	Ill-defined descriptions and complications of heart disease	Inverse variance weighted	2	0.130637	0.098005	0.182542	1.139554	0.940398	1.380887	0.996292
neoplasms	Other non-epithelial cancer of skin	Inverse variance weighted	2	0.034729	0.02619	0.184835	1.035339	0.983533	1.089874	0.996292
genitourinary	Inflammatory disease of breast	Inverse variance weighted	2	-0.18907	0.142881	0.185753	0.827731	0.625556	1.095247	0.996292
injuries & poisonings	Foreign body injury	Inverse variance weighted	2	-0.10227	0.077393	0.186359	0.902787	0.775724	1.050663	0.996292
digestive	Other disorders of gallbladder	Inverse variance weighted	2	0.093432	0.071209	0.189496	1.097936	0.95491	1.262384	0.996292
endocrine/metabolic	Gout and other crystal arthropathies	Inverse variance weighted	2	0.06957	0.053311	0.191897	1.072047	0.965683	1.190126	0.996292
endocrine/metabolic	Protein-calorie malnutrition	Inverse variance weighted	2	-0.1068	0.082087	0.193224	0.898702	0.765141	1.055577	0.996292
circulatory system	Precordial pain	Inverse variance weighted	2	-0.05834	0.044876	0.193588	0.943328	0.863899	1.03006	0.996292
neoplasms	Cancer within the respiratory system	Inverse variance weighted	2	0.067927	0.05232	0.194184	1.070287	0.965972	1.185867	0.996292
genitourinary	Functional disorders of bladder	Inverse variance weighted	2	-0.0853	0.065903	0.195554	0.918237	0.806968	1.044847	0.996292
circulatory system	Left bundle branch block	Inverse variance weighted	2	-0.09681	0.075775	0.201396	0.907729	0.782447	1.053071	0.996292
mental disorders	Altered mental status	Inverse variance weighted	2	-0.08884	0.069654	0.202127	0.914988	0.798225	1.048832	0.996292
musculoskeletal	Degeneration of intervertebral disc	Inverse variance weighted	2	0.065103	0.051053	0.202239	1.067269	0.965643	1.179591	0.996292
neurological	Inflammatory and toxic neuropathy	Inverse variance weighted	2	0.092192	0.072391	0.202827	1.096576	0.951521	1.263743	0.996292
circulatory system	Occlusion of cerebral arteries	Inverse variance weighted	2	-0.07994	0.062865	0.203537	0.923176	0.816155	1.044231	0.996292
respiratory	Chronic pharyngitis and nasopharyngitis	Inverse variance weighted	2	0.10915	0.085923	0.203971	1.115329	0.942462	1.319903	0.996292
circulatory system	Mitral valve disease	Inverse variance weighted	2	0.068466	0.054161	0.206193	1.070864	0.963011	1.190796	0.996292
sense organs	Retinal vascular changes and abnomalities	Inverse variance weighted	2	0.116392	0.092098	0.206304	1.123437	0.937893	1.345686	0.996292
circulatory system	Paroxysmal ventricular tachycardia	Inverse variance weighted	2	0.110211	0.087397	0.207295	1.116514	0.940741	1.325128	0.996292
injuries & poisonings	Fracture of radius and ulna	Inverse variance weighted	2	-0.05742	0.045556	0.207523	0.944198	0.863546	1.032384	0.996292
musculoskeletal	Osteoporosis NOS	Inverse variance weighted	2	-0.04516	0.036166	0.211798	0.955846	0.890436	1.026061	0.996292
injuries & poisonings	Fracture of hand or wrist	Inverse variance weighted	2	-0.06246	0.050066	0.212195	0.939451	0.851643	1.036313	0.996292
mental disorders	Other specified nonpsychotic and/or transient mental disorders	Inverse variance weighted	2	-0.13118	0.105937	0.215615	0.877061	0.712613	1.079457	0.996292
pregnancy complications	Placenta previa and abruptio placenta	Inverse variance weighted	2	-0.09494	0.077393	0.219908	0.909425	0.781427	1.058387	0.996292
circulatory system	Abnormal function study of cardiovascular system	Inverse variance weighted	2	0.143274	0.117118	0.221204	1.154046	0.91734	1.451831	0.996292
digestive	Chronic periodontitis	Inverse variance weighted	2	-0.13709	0.112114	0.221414	0.871891	0.699888	1.086165	0.996292
endocrine/metabolic	Acidosis	Inverse variance weighted	2	0.099973	0.082087	0.22327	1.105141	0.9409	1.298051	0.996292
digestive	Hemorrhage from gastrointestinal ulcer	Inverse variance weighted	2	0.13146	0.108281	0.224724	1.140493	0.922405	1.410144	0.996292
infectious diseases	Infection/inflammation of internal prosthetic device; implant; and graft	Inverse variance weighted	2	-0.06486	0.053555	0.225857	0.937198	0.843809	1.040923	0.996292
genitourinary	Frequency of urination and polyuria	Inverse variance weighted	2	-0.05131	0.042375	0.225967	0.949986	0.874273	1.032256	0.996292
genitourinary	Mucous polyp of cervix	Inverse variance weighted	2	-0.08208	0.067813	0.226148	0.921201	0.806549	1.052151	0.996292
circulatory system	Aneurysm and dissection of heart	Inverse variance weighted	2	0.121848	0.100934	0.227352	1.129583	0.926833	1.376686	0.996292
dermatologic	Cellulitis and abscess of foot, toe	Inverse variance weighted	2	-0.04352	0.036136	0.228466	0.957414	0.891949	1.027684	0.996292
neoplasms	Cancer of bronchus; lung	Inverse variance weighted	2	0.070293	0.058559	0.229992	1.072823	0.956492	1.203301	0.996292
neurological	Abnormal movement	Inverse variance weighted	2	-0.09057	0.075456	0.230014	0.913409	0.787837	1.058997	0.996292
musculoskeletal	Aseptic necrosis of bone	Inverse variance weighted	2	-0.29869	0.249849	0.231901	0.74179	0.454575	1.210478	0.996292
hematopoietic	Diseases of spleen	Inverse variance weighted	2	0.141339	0.11829	0.232145	1.151815	0.913465	1.452358	0.996292
respiratory	Acute upper respiratory infections of multiple or unspecified sites	Inverse variance weighted	2	0.065252	0.054725	0.233117	1.067428	0.958862	1.188288	0.996292
circulatory system	Stricture of artery	Inverse variance weighted	2	-0.1562	0.131878	0.236241	0.855388	0.660551	1.107693	0.996292
neoplasms	Multiple myeloma	Inverse variance weighted	2	0.134125	0.113286	0.236434	1.143535	0.915838	1.427843	0.996292
mental disorders	Dementias	Inverse variance weighted	2	-0.10134	0.085923	0.238229	0.903626	0.763572	1.06937	0.996292
hematopoietic	Lymphadenitis	Inverse variance weighted	2	-0.07006	0.059562	0.239501	0.932339	0.82961	1.047789	0.996292
respiratory	Abnormal findings examination of lungs	Inverse variance weighted	2	0.070152	0.059737	0.240256	1.072671	0.954152	1.205912	0.996292
digestive	Reflux esophagitis	Inverse variance weighted	2	-0.03244	0.027662	0.240944	0.968083	0.916994	1.022019	0.996292
digestive	Cholelithiasis and cholecystitis	Inverse variance weighted	2	0.026106	0.022423	0.244317	1.02645	0.982316	1.072567	0.996292
musculoskeletal	Osteoporosis, osteopenia and pathological fracture	Inverse variance weighted	2	-0.03605	0.031163	0.247393	0.964595	0.907441	1.025349	0.996292
dermatologic	Cellulitis and abscess of leg, except foot	Inverse variance weighted	2	-0.04155	0.036136	0.250161	0.959297	0.893703	1.029704	0.996292
musculoskeletal	Osteoporosis	Inverse variance weighted	2	-0.0387	0.033665	0.250273	0.962035	0.900606	1.027655	0.996292
neoplasms	Benign neoplasm of colon	Inverse variance weighted	2	0.035797	0.031214	0.251452	1.036446	0.974937	1.101836	0.996292
genitourinary	Breast conditions, congenital or relating to hormones	Inverse variance weighted	2	0.116789	0.101992	0.252173	1.123883	0.920246	1.372581	0.996292
neoplasms	Malignant neoplasm, other	Inverse variance weighted	2	-0.02426	0.021217	0.252773	0.976027	0.936272	1.017472	0.996292
mental disorders	Schizophrenia	Inverse variance weighted	2	-0.15805	0.138454	0.253634	0.853804	0.650884	1.119985	0.996292
neurological	Other conditions of brain	Inverse variance weighted	2	0.079924	0.070909	0.259685	1.083205	0.942653	1.244714	0.996292
digestive	Regional enteritis	Inverse variance weighted	2	0.072832	0.064735	0.260554	1.07555	0.947386	1.221052	0.996292
symptoms	Myalgia and myositis unspecified	Inverse variance weighted	2	-0.11861	0.105937	0.262856	0.88815	0.721624	1.093106	0.996292
hematopoietic	Iron deficiency anemias, unspecified or not due to blood loss	Inverse variance weighted	2	-0.03474	0.031163	0.264994	0.96586	0.90863	1.026694	0.996292
musculoskeletal	Other and unspecified disc disorder	Inverse variance weighted	2	0.045725	0.041077	0.265639	1.046787	0.965813	1.134549	0.996292
genitourinary	Dyspareunia	Inverse variance weighted	2	-0.08868	0.079699	0.265862	0.915141	0.782792	1.069867	0.996292
hematopoietic	Iron deficiency anemias	Inverse variance weighted	2	-0.03454	0.031132	0.267253	0.966051	0.908866	1.026835	0.996292
neoplasms	Cancer of other lymphoid, histiocytic tissue	Inverse variance weighted	2	-0.06162	0.056204	0.272913	0.94024	0.842165	1.049736	0.996292
injuries & poisonings	Internal derangement of knee	Inverse variance weighted	2	-0.02455	0.022423	0.273531	0.975747	0.933792	1.019586	0.996292
mental disorders	Anxiety disorder	Inverse variance weighted	2	-0.03683	0.033665	0.273916	0.963838	0.902293	1.02958	0.996292
neurological	Abnormal involuntary movements	Inverse variance weighted	2	0.09794	0.090053	0.276782	1.102896	0.924442	1.315798	0.996292
circulatory system	Hypotension	Inverse variance weighted	2	-0.03788	0.034901	0.277737	0.962827	0.899167	1.030994	0.996292
symptoms	Other abnormal blood chemistry	Inverse variance weighted	2	-0.06805	0.062742	0.278103	0.934214	0.826112	1.056462	0.996292
circulatory system	Atherosclerosis of the extremities	Inverse variance weighted	2	-0.10082	0.093579	0.281323	0.904098	0.752592	1.086104	0.996292
genitourinary	Cervicitis and endocervicitis	Inverse variance weighted	2	-0.07924	0.073568	0.281418	0.923815	0.799765	1.067106	0.996292
circulatory system	Coronary atherosclerosis	Inverse variance weighted	2	-0.02276	0.021156	0.281972	0.977496	0.937792	1.01888	0.996292
respiratory	Septal Deviations/Turbinate Hypertrophy	Inverse variance weighted	2	-0.04193	0.039246	0.285363	0.958939	0.887941	1.035613	0.996292
musculoskeletal	Spondylosis and allied disorders	Inverse variance weighted	2	-0.0331	0.031132	0.287649	0.967439	0.910171	1.02831	0.996292
digestive	Paralytic ileus	Inverse variance weighted	2	0.124399	0.117118	0.288159	1.132468	0.900188	1.424685	0.996292
genitourinary	Other specified benign mammary dysplasias	Inverse variance weighted	2	-0.10706	0.100934	0.288839	0.898474	0.737206	1.09502	0.996292
hematopoietic	Pernicious anemia	Inverse variance weighted	2	0.134817	0.127347	0.289753	1.144328	0.89156	1.468758	0.996292
injuries & poisonings	Allergy/adverse effect of penicillin	Inverse variance weighted	2	-0.02369	0.022391	0.290087	0.976591	0.934659	1.020403	0.996292
genitourinary	Hypertrophy of female genital organs	Inverse variance weighted	2	-0.07885	0.074743	0.291438	0.924177	0.798239	1.069985	0.996292
injuries & poisonings	Fracture of upper limb	Inverse variance weighted	2	-0.03124	0.029897	0.296036	0.969241	0.914077	1.027734	0.996292
dermatologic	Cellulitis and abscess of arm/hand	Inverse variance weighted	2	-0.03773	0.036136	0.296456	0.962974	0.897129	1.033652	0.996292
genitourinary	Chronic renal failure [CKD]	Inverse variance weighted	2	-0.05397	0.052351	0.302587	0.947462	0.855066	1.049841	0.996292
congenital anomalies	Congenital anomalies of great vessels	Inverse variance weighted	2	0.065276	0.063562	0.304442	1.067453	0.942418	1.209078	0.996292
circulatory system	Angina pectoris	Inverse variance weighted	2	-0.02299	0.022423	0.305162	0.977269	0.93525	1.021177	0.996292
sense organs	Primary open angle glaucoma	Inverse variance weighted	2	0.095422	0.09314	0.305597	1.100123	0.916556	1.320454	0.996292
genitourinary	Urinary incontinence	Inverse variance weighted	2	-0.02934	0.028692	0.306547	0.971089	0.917986	1.027264	0.996292
respiratory	Bacterial pneumonia	Inverse variance weighted	2	0.034345	0.033602	0.306731	1.034942	0.968976	1.105398	0.996292
infectious diseases	Viral Enteritis	Inverse variance weighted	2	0.092809	0.090927	0.307395	1.097252	0.918138	1.311309	0.996292
musculoskeletal	Other disorders of synovium, tendon, and bursa	Inverse variance weighted	2	-0.03154	0.031132	0.311078	0.968956	0.911599	1.029922	0.996292
neoplasms	Benign neoplasm of lip, oral cavity, and pharynx	Inverse variance weighted	2	-0.0858	0.08475	0.311344	0.917777	0.777314	1.083621	0.996292
digestive	Diseases of pancreas	Inverse variance weighted	2	0.05286	0.05232	0.312343	1.054282	0.951527	1.168134	0.996292
genitourinary	Calculus of ureter	Inverse variance weighted	2	-0.05522	0.054725	0.312952	0.946277	0.850032	1.053419	0.996292
musculoskeletal	Malunion and nonunion of fracture	Inverse variance weighted	2	0.075401	0.074743	0.313069	1.078316	0.931373	1.248443	0.996292
symptoms	Effects of other external causes	Inverse variance weighted	2	-0.04249	0.042228	0.314296	0.958399	0.882269	1.041097	0.996292
digestive	Hemorrhage of rectum and anus	Inverse variance weighted	2	0.023703	0.023658	0.3164	1.023986	0.977588	1.072586	0.996292
musculoskeletal	Other disorders of bone and cartilage	Inverse variance weighted	2	-0.04347	0.04361	0.318829	0.957458	0.879018	1.042897	0.996292
circulatory system	Elevated blood pressure reading without diagnosis of hypertension	Inverse variance weighted	2	-0.12349	0.123901	0.318908	0.883828	0.693268	1.126768	0.996292
circulatory system	Late effects of cerebrovascular disease	Inverse variance weighted	2	-0.07566	0.075914	0.318921	0.92713	0.798953	1.07587	0.996292

digestive	Personal history of diseases of digestive system	Inverse variance weighted	2	0.022347	0.022423	0.318948	1.022599	0.97863	1.068543	0.996292
symptoms	Nonspecific findings on examination of blood	Inverse variance weighted	2	-0.05545	0.055645	0.318986	0.946057	0.848303	1.055075	0.996292
sense organs	Open-angle glaucoma	Inverse variance weighted	2	0.091786	0.092254	0.319769	1.09613	0.914817	1.31338	0.996292
pregnancy complications	Late pregnancy and failed induction	Inverse variance weighted	2	-0.07654	0.077393	0.322684	0.926318	0.795943	1.078048	0.996292
sense organs	Labyrinthitis	Inverse variance weighted	2	-0.10225	0.103668	0.323983	0.902806	0.736801	1.106211	0.996292
genitourinary	Pain and other symptoms associated with female genital organs	Inverse variance weighted	2	0.065418	0.066458	0.324943	1.067606	0.937217	1.216134	0.996292
sense organs	Vertiginous syndromes and other disorders of vestibular system	Inverse variance weighted	2	-0.03428	0.03487	0.325609	0.966304	0.902469	1.034655	0.996292
dermatologic	Superficial cellulitis and abscess	Inverse variance weighted	2	-0.03033	0.031163	0.33043	0.970126	0.912643	1.031228	0.996292
musculoskeletal	Stiffness of joint	Inverse variance weighted	2	-0.10385	0.107111	0.332245	0.901356	0.730671	1.111913	0.996292
dermatologic	Atopic/contact dermatitis due to other or unspecified	Inverse variance weighted	2	0.05667	0.058559	0.333177	1.058306	0.94355	1.187019	0.996292
digestive	Acute appendicitis	Inverse variance weighted	2	-0.05032	0.052381	0.336704	0.950923	0.85814	1.053738	0.996292
digestive	Other disorders of peritoneum	Inverse variance weighted	2	-0.06718	0.069934	0.336749	0.935028	0.815258	1.072392	0.996292
neurological	Sleep disorders	Inverse variance weighted	2	-0.03584	0.037402	0.337909	0.964792	0.896595	1.038176	0.996292
injuries & poisonings	Fracture of tibia and fibula	Inverse variance weighted	2	-0.07919	0.08274	0.338523	0.923865	0.785558	1.086522	0.996292
respiratory	Chronic sinusitis	Inverse variance weighted	2	-0.05889	0.061718	0.340026	0.942814	0.835393	1.064049	0.996292
neoplasms	Cancer of esophagus	Inverse variance weighted	2	-0.09515	0.099759	0.340178	0.909235	0.747756	1.105586	0.996292
digestive	Diseases of lips	Inverse variance weighted	2	-0.09774	0.102898	0.342199	0.906889	0.741252	1.109539	0.996292
symptoms	Neuralgia, neuritis, and radiculitis NOS	Inverse variance weighted	2	0.073409	0.077393	0.342864	1.07617	0.924704	1.252446	0.996292
endocrine/metabolic	Disorders of the pituitary gland and its hypothalamic control	Inverse variance weighted	2	0.095727	0.100934	0.342919	1.100459	0.902936	1.341191	0.996292
injuries & poisonings	Poisoning by psychotropic agents	Inverse variance weighted	2	-0.10266	0.10827	0.343024	0.902431	0.729882	1.115773	0.996292
digestive	Inguinal hernia	Inverse variance weighted	2	0.021274	0.022453	0.343399	1.021502	0.977522	1.06746	0.996292
neoplasms	Benign neoplasm of unspecified sites	Inverse variance weighted	2	0.051811	0.054725	0.343769	1.053176	0.946059	1.172422	0.996292
sense organs	Strabismus (not specified as paralytic)	Inverse variance weighted	2	-0.08353	0.088576	0.345682	0.919866	0.773262	1.094264	0.996292
genitourinary	Vaginal enterocele, congenital or acquired	Inverse variance weighted	2	0.096276	0.102107	0.345732	1.101063	0.901358	1.345015	0.996292
circulatory system	First degree AV block	Inverse variance weighted	2	-0.08099	0.085923	0.345883	0.922202	0.779268	1.091352	0.996292
neoplasms	Cancer of stomach	Inverse variance weighted	2	-0.24549	0.260781	0.346513	0.782319	0.469249	1.304262	0.996292
musculoskeletal	Other unspecified back disorders	Inverse variance weighted	2	0.062005	0.065903	0.346784	1.063968	0.93504	1.210672	0.996292
endocrine/metabolic	Acid-base balance disorder	Inverse variance weighted	2	0.073497	0.078571	0.349571	1.076266	0.922653	1.255453	0.996292
digestive	Appendiceal conditions	Inverse variance weighted	2	-0.04311	0.046112	0.349877	0.957809	0.875039	1.048408	0.996292
neoplasms	Malignant neoplasm of testis	Inverse variance weighted	2	-0.04072	0.043579	0.350142	0.960101	0.881499	1.045712	0.996292
digestive	Abnormal findings on exam of gastrointestinal tract/ abdominal area	Inverse variance weighted	2	0.072615	0.077736	0.350239	1.075317	0.92335	1.252294	0.996292
neoplasms	Secondary malignant neoplasm	Inverse variance weighted	2	-0.02563	0.027456	0.350555	0.974696	0.923631	1.028584	0.996292
musculoskeletal	Osteoarthrosis, generalized	Inverse variance weighted	2	0.110822	0.118756	0.350721	1.117196	0.885202	1.40999	0.996292
endocrine/metabolic	Abnormal glucose	Inverse variance weighted	2	0.094671	0.102107	0.353834	1.099297	0.899912	1.342858	0.996292
symptoms	Swelling of limb	Inverse variance weighted	2	0.036936	0.039842	0.353896	1.037626	0.959681	1.121902	0.996292
injuries & poisonings	Fracture of ribs	Inverse variance weighted	2	-0.08883	0.09593	0.354454	0.915001	0.758165	1.10428	0.996292
genitourinary	Calculus of kidney	Inverse variance weighted	2	-0.04381	0.047378	0.35516	0.957139	0.87226	1.050277	0.996292
neoplasms	Secondary malignancy of bone	Inverse variance weighted	2	-0.05302	0.057383	0.355488	0.948359	0.847475	1.061252	0.996292
injuries & poisonings	Fracture of foot	Inverse variance weighted	2	-0.06305	0.068246	0.355525	0.938893	0.821342	1.073268	0.996292
circulatory system	Intracerebral hemorrhage	Inverse variance weighted	2	0.131635	0.142494	0.355592	1.140692	0.862731	1.508209	0.996292
sense organs	Hearing loss	Inverse variance weighted	2	-0.03798	0.041139	0.355903	0.962732	0.888152	1.043576	0.996292
circulatory system	Noninfectious disorders of lymphatic channels	Inverse variance weighted	2	-0.14764	0.160418	0.357405	0.862745	0.629988	1.181498	0.996292
neoplasms	Cancer, suspected or other	Inverse variance weighted	2	-0.01942	0.021187	0.359275	0.980764	0.940869	1.02235	0.996292
musculoskeletal	Rupture of synovium	Inverse variance weighted	2	-0.15206	0.166195	0.360225	0.858939	0.620147	1.18968	0.996292
sense organs	Other disorders of eyelids	Inverse variance weighted	2	-0.03286	0.036105	0.362803	0.967677	0.901566	1.038636	0.996292
neoplasms	Secondary malignant neoplasm of digestive systems	Inverse variance weighted	2	0.062238	0.068566	0.364029	1.064216	0.930391	1.217289	0.996292
sense organs	Other retinal disorders	Inverse variance weighted	2	0.071305	0.07895	0.366439	1.073909	0.919949	1.253635	0.996292
circulatory system	Primary/intrinsic cardiomyopathies	Inverse variance weighted	2	-0.07874	0.087195	0.366484	0.924277	0.779077	1.096538	0.996292
endocrine/metabolic	Electrolyte imbalance	Inverse variance weighted	2	0.038141	0.042344	0.36772	1.038878	0.956138	1.128778	0.996292
injuries & poisonings	Fracture of clavicle or scapula	Inverse variance weighted	2	-0.06103	0.068566	0.373434	0.940797	0.822493	1.076119	0.996292
musculoskeletal	Spinal stenosis	Inverse variance weighted	2	0.038835	0.043641	0.373528	1.039599	0.954373	1.132436	0.996292
injuries & poisonings	Poisoning by analgesics, antipyretics, and antirheumatics	Inverse variance weighted	2	-0.04029	0.045364	0.374508	0.960515	0.878799	1.049829	0.996292
genitourinary	Genital prolapse	Inverse variance weighted	2	0.023186	0.026128	0.374864	1.023457	0.972364	1.077234	0.996292
circulatory system	Paroxysmal tachycardia, unspecified	Inverse variance weighted	2	-0.04912	0.055508	0.376204	0.952067	0.853923	1.061492	0.996292
dermatologic	Psoriasis and related disorders	Inverse variance weighted	2	0.049497	0.056204	0.378495	1.050742	0.941141	1.173107	0.996292
digestive	Appendicitis	Inverse variance weighted	2	-0.0417	0.047378	0.378724	0.959153	0.874096	1.052487	0.996292
respiratory	Epistaxis or throat hemorrhage	Inverse variance weighted	2	0.091516	0.104077	0.379231	1.095835	0.89362	1.343808	0.996292
circulatory system	Cardiomyopathy	Inverse variance weighted	2	-0.07224	0.082378	0.380529	0.930309	0.7916	1.093323	0.996292
mental disorders	Anxiety disorders	Inverse variance weighted	2	-0.02841	0.032399	0.380578	0.971991	0.912187	1.035717	0.996292
endocrine/metabolic	Vitamin deficiency	Inverse variance weighted	2	0.067221	0.077082	0.383168	1.069532	0.919561	1.243961	0.996292
sense organs	Visual disturbances	Inverse variance weighted	2	0.040231	0.046142	0.383269	1.041051	0.951032	1.139591	0.996292
digestive	Gastric ulcer	Inverse variance weighted	2	-0.03679	0.042344	0.384996	0.963883	0.887116	1.047293	0.996292
digestive	Nonspecific abnormal findings in stool contents	Inverse variance weighted	2	-0.05711	0.065903	0.386171	0.94449	0.83004	1.07472	0.996292
injuries & poisonings	Fracture of patella	Inverse variance weighted	2	-0.08381	0.097102	0.388077	0.919606	0.760233	1.11239	0.996292
genitourinary	Irregular menstrual bleeding	Inverse variance weighted	2	0.05387	0.062577	0.389319	1.055347	0.933531	1.193059	0.996292
symptoms	Musculoskeletal symptoms referable to limbs	Inverse variance weighted	2	0.031082	0.036166	0.390105	1.03157	0.960978	1.107348	0.996292
musculoskeletal	Other and unspecified disorders of back	Inverse variance weighted	2	0.050198	0.058559	0.391319	1.05148	0.937464	1.179362	0.996292
musculoskeletal	Derangement of joint, non-traumatic	Inverse variance weighted	2	0.042664	0.049849	0.392077	1.043587	0.946447	1.150698	0.996292
musculoskeletal	Polymyalgia Rheumatica	Inverse variance weighted	2	0.067219	0.078571	0.392262	1.06953	0.916879	1.247597	0.996292
endocrine/metabolic	Disorders of mineral metabolism	Inverse variance weighted	2	0.050048	0.058559	0.392741	1.051321	0.937323	1.179185	0.996292
dermatologic	Rash and other nonspecific skin eruption	Inverse variance weighted	2	0.048646	0.057383	0.396587	1.049848	0.938169	1.174823	0.996292
sense organs	Disorders of external ear	Inverse variance weighted	2	0.060066	0.070909	0.39695	1.061906	0.924118	1.22024	0.996292
digestive	Ileostomy status	Inverse variance weighted	2	-0.05582	0.065903	0.397027	0.945713	0.831115	1.076112	0.996292
neurological	Sleep apnea	Inverse variance weighted	2	-0.0347	0.041109	0.398562	0.965892	0.89112	1.046938	0.996292
musculoskeletal	Osteomyelitis	Inverse variance weighted	2	-0.09449	0.112114	0.399335	0.909836	0.730347	1.133435	0.996292
sense organs	Otitis media and Eustachian tube disorders	Inverse variance weighted	2	-0.04728	0.056204	0.400211	0.953819	0.854328	1.064897	0.996292
sense organs	Strabismus and other disorders of binocular eye movements	Inverse variance weighted	2	-0.05964	0.070909	0.400329	0.942107	0.819863	1.082577	0.996292
endocrine/metabolic	Disorders of calcium/phosphorus metabolism	Inverse variance weighted	2	0.064802	0.077082	0.400518	1.066948	0.91734	1.240956	0.996292
respiratory	Bronchiectasis	Inverse variance weighted	2	-0.05596	0.066674	0.401263	0.945573	0.829739	1.077579	0.996292
neurological	Parkinson's disease	Inverse variance weighted	2	-0.06652	0.079746	0.404191	0.935643	0.800255	1.093936	0.996292
digestive	Other diseases of the teeth and supporting structures	Inverse variance weighted	2	-0.0426	0.051085	0.404351	0.958296	0.866993	1.059215	0.996292
neoplasms	Malignant neoplasm of bladder	Inverse variance weighted	2	-0.04865	0.058559	0.406063	0.952511	0.849227	1.068357	0.996292
pregnancy complications	Missed abortion/Hydatidiform mole	Inverse variance weighted	2	0.064223	0.077393	0.406632	1.06633	0.916249	1.240994	0.996292
neurological	Extrapyramidal disease and abnormal movement disorders	Inverse variance weighted	2	0.085942	0.103885	0.408079	1.089743	0.888987	1.335834	0.996292
symptoms	Abdominal pain	Inverse variance weighted	2	-0.01132	0.013713	0.409058	0.988743	0.962522	1.015678	0.996292
sense organs	Dizziness and giddiness (Light-headedness and vertigo)	Inverse variance weighted	2	-0.03283	0.039873	0.410369	0.967707	0.894959	1.046369	0.996292
symptoms	Symptoms of the muscles	Inverse variance weighted	2	-0.08268	0.100934	0.412692	0.920644	0.755397	1.122041	0.996292
digestive	Diseases of the jaws	Inverse variance weighted	2	-0.12778	0.156184	0.413293	0.88005	0.647978	1.195238	0.996292
digestive	Liver abscess and sequelae of chronic liver disease	Inverse variance weighted	2	0.07092	0.087093	0.415471	1.073495	0.905035	1.273313	0.996292
digestive	Peritoneal adhesions (postoperative) (postinfection)	Inverse variance weighted	2	-0.04045	0.04969	0.415595	0.960355	0.871233	1.058593	0.996292
sense organs	Corneal opacity and other disorders of cornea	Inverse variance weighted	2	0.080229	0.098581	0.415737	1.083536	0.89316	1.31449	0.996292
endocrine/metabolic	Hypopotassemia	Inverse variance weighted	2	0.057593	0.070909	0.416671	1.059284	0.921835	1.217226	0.996292
circulatory system	Arterial embolism and thrombosis	Inverse variance weighted	2	-0.07191	0.088576	0.416851	0.93061	0.782294	1.107046	0.996292
musculoskeletal	Hallux valgus (Bunion)	Inverse variance weighted	2	-0.02723	0.033634	0.418209	0.973139	0.911056	1.039454	0.996292
respiratory	Acute and chronic tonsillitis	Inverse variance weighted	2	-0.06839	0.084592	0.418805	0.933894	0.791209	1.10231	0.996292
circulatory system	Disease of tricuspid valve	Inverse variance weighted	2	0.066305	0.082087	0.419243	1.068552	0.909749	1.255076	0.996292
endocrine/metabolic	Disorders of adrenal glands	Inverse variance weighted	2	-0.17597	0.218393	0.420381	0.838641	0.546609	1.286696	0.996292
genitourinary	Retention of urine	Inverse variance weighted	2	0.027015	0.033602	0.421416	1.027383	0.961899	1.097325	0.996292
circulatory system	Cardiomegaly	Inverse variance weighted	2	-0.04304	0.053555	0.421582	0.957872	0.862423	1.063885	0.996292
sense organs	Aphakia and other disorders of lens	Inverse variance weighted	2	-0.05099	0.063562	0.42245	0.95029	0.838978	1.07637	0.996292
genitourinary	Glomerulonephritis	Inverse variance weighted	2	-0.06685	0.083574	0.423749	0.935332	0.794011	1.101807	0.996292
symptoms	Symptoms involving nervous and musculoskeletal systems	Inverse variance weighted	2	-0.04969	0.062386	0.425724	0.951522	0.842006	1.075283	0.996292
neurological	Degenerative disease of the spinal cord	Inverse variance weighted	2	0.068925	0.086628	0.426241	1.071355	0.904054	1.269617	0.996292
genitourinary	Hypertrophy of breast (Gynecomastia)	Inverse variance weighted	2	0.116844	0.146954	0.426551	1.123944	0.842665	1.499112	0.996292
digestive	Other symptoms involving abdomen and pelvis	Inverse variance weighted	2	-0.03758	0.047348	0.42739	0.963119	0.877762	1.056776	0.996292
pregnancy complications	Umbilical cord complications during labor and delivery	Inverse variance weighted	2	-0.09313	0.117409	0.427649	0.911074	0.723791	1.146817	0.996292
neoplasms	Colorectal cancer	Inverse variance weighted	2	0.046056	0.058313	0.429645	1.047133	0.934038	1.17392	0.996292
infectious diseases	Streptococcus infection	Inverse variance weighted	2	0.081858	0.103712	0.429948	1.085302	0.885664	1.329941	0.996292
circulatory system	Subarachnoid hemorrhage	Inverse variance weighted	2	0.073638	0.093579	0.431338	1.076417	0.896034	1.293112	0.996292
dermatologic	Disorders of sweat glands	Inverse variance weighted	2	0.073002	0.093579	0.435327	1.075732	0.895464	1.29229	0.996292
infectious diseases	Postoperative infection	Inverse variance weighted	2	0.031126	0.039904	0.435373	1.031615	0.954006	1.115538	0.996292
genitourinary	Postmenopausal atrophic vaginitis	Inverse variance weighted	2	-0.06205	0.079746	0.436523	0.939837	0.803842	1.098839	0.996292
sense organs	Degeneration of macula and posterior pole of retina	Inverse variance weighted	2	0.08691	0.112418	0.439461	1.090799	0.87509	1.35968	0.996292
digestive	GERD	Inverse variance weighted	2	0.017314	0.022453	0.440624	1.017465	0.97366	1.063242	0.996292

respiratory	Chronic tonsillitis and adenoiditis	Inverse variance weighted	2	-0.06116	0.079746	0.443127	0.940673	0.804558	1.099817	0.996292
circulatory system	Polyarteritis nodosa and allied conditions	Inverse variance weighted	2	-0.07121	0.093266	0.445159	0.931267	0.775684	1.118056	0.996292
respiratory	Pleurisy; pleural effusion	Inverse variance weighted	2	-0.02567	0.033634	0.445361	0.974658	0.912477	1.041076	0.996292
sense organs	Macular degeneration (senile) of retina NOS	Inverse variance weighted	2	0.084486	0.111828	0.449947	1.088158	0.873981	1.354821	0.996292
genitourinary	Urinary calculus	Inverse variance weighted	2	-0.02539	0.033634	0.450283	0.974928	0.91273	1.041364	0.996292
genitourinary	Prolapse of vaginal vault after hysterectomy	Inverse variance weighted	2	-0.08926	0.11829	0.45052	0.914612	0.725347	1.153261	0.996292
musculoskeletal	Enthesopathy	Inverse variance weighted	2	-0.02068	0.027426	0.450831	0.979532	0.928267	1.033628	0.996292
circulatory system	Other disorders of arteries and arterioles	Inverse variance weighted	2	-0.08946	0.118739	0.451214	0.914427	0.724564	1.154043	0.996292
neoplasms	Lymphoid leukemia, chronic	Inverse variance weighted	2	-0.08865	0.11829	0.453619	0.915169	0.725789	1.153964	0.996292
injuries & poisonings	Injury, NOS	Inverse variance weighted	2	0.022393	0.029897	0.453851	1.022646	0.964442	1.084362	0.996292
sense organs	Suppurative and unspecified otitis media	Inverse variance weighted	2	-0.06794	0.090927	0.454972	0.93432	0.781803	1.116591	0.996292
injuries & poisonings	Burns	Inverse variance weighted	2	0.082621	0.11094	0.456435	1.08613	0.873871	1.349945	0.996292
neoplasms	Leukemia	Inverse variance weighted	2	0.04892	0.065903	0.457907	1.050136	0.922885	1.194933	0.996292
musculoskeletal	Osteomyelitis, periostitis, and other infections involving bone	Inverse variance weighted	2	-0.07938	0.108281	0.463517	0.923691	0.74706	1.142083	0.996292
dermatologic	Sarcoidosis	Inverse variance weighted	2	-0.08661	0.118517	0.464907	0.917034	0.726945	1.156828	0.996292
musculoskeletal	Other disorders of soft tissues	Inverse variance weighted	2	0.029336	0.040209	0.465645	1.029771	0.95173	1.114211	0.996292
respiratory	Respiratory abnormalities	Inverse variance weighted	2	0.114234	0.157363	0.467884	1.121015	0.823495	1.526025	0.996292
sense organs	Ptosis of eyelid	Inverse variance weighted	2	0.045075	0.062386	0.46998	1.046106	0.925703	1.182169	0.996292
mental disorders	Tobacco use disorder	Inverse variance weighted	2	-0.01439	0.019952	0.470847	0.985716	0.947913	1.025026	0.996292
dermatologic	Psoriasis	Inverse variance weighted	2	0.041279	0.057383	0.471921	1.042143	0.931283	1.1662	0.996292
genitourinary	Cystic mastopathy	Inverse variance weighted	2	0.088153	0.1233	0.474643	1.092155	0.857688	1.390718	0.996292
injuries & poisonings	Open wounds of extremities	Inverse variance weighted	2	-0.02933	0.041139	0.475915	0.971098	0.895869	1.052644	0.996292
digestive	Obstruction of bile duct	Inverse variance weighted	2	-0.06828	0.09593	0.476579	0.933994	0.773903	1.127203	0.996292
genitourinary	Postmenopausal bleeding	Inverse variance weighted	2	-0.0203	0.028662	0.478768	0.979904	0.926373	1.036528	0.996292
genitourinary	Noninflammatory disorders of ovary, fallopian tube, and broad ligament	Inverse variance weighted	2	0.069816	0.098581	0.478818	1.072311	0.883907	1.300872	0.996292
genitourinary	Other disorders of the kidney and ureters	Inverse variance weighted	2	-0.03947	0.055753	0.478944	0.961295	0.861784	1.072297	0.996292
congenital anomalies	Cardiac congenital anomalies	Inverse variance weighted	2	-0.03693	0.052351	0.480514	0.963741	0.869758	1.06788	0.996292
pregnancy complications	Problems associated with amniotic cavity and membranes	Inverse variance weighted	2	0.082835	0.117702	0.481577	1.086362	0.862551	1.368247	0.996292
genitourinary	Chronic glomerulonephritis, NOS	Inverse variance weighted	2	0.064713	0.092098	0.482271	1.066853	0.890654	1.277908	0.996292
respiratory	Other upper respiratory disease	Inverse variance weighted	2	-0.02975	0.042344	0.482378	0.970692	0.893383	1.054691	0.996292
neoplasms	Secondary malignancy of respiratory organs	Inverse variance weighted	2	-0.04025	0.057383	0.482996	0.960545	0.858365	1.074889	0.996292
genitourinary	Pelvic peritoneal adhesions, female (postoperative) (postinfection)	Inverse variance weighted	2	0.03767	0.054694	0.490981	1.038389	0.932833	1.15589	0.996292
genitourinary	Uterine/Uterovaginal prolapse	Inverse variance weighted	2	0.02561	0.037371	0.49317	1.02594	0.953479	1.103909	0.996292
circulatory system	Raynaud's syndrome	Inverse variance weighted	2	-0.05384	0.078571	0.493199	0.947584	0.812338	1.105348	0.996292
digestive	Functional digestive disorders	Inverse variance weighted	2	0.012773	0.018686	0.494231	1.012855	0.976432	1.050638	0.996292
digestive	Peritonitis and retroperitoneal infections	Inverse variance weighted	2	0.061071	0.089753	0.496226	1.062975	0.891505	1.267425	0.996292
neoplasms	Lymphoid leukemia	Inverse variance weighted	2	-0.07537	0.11094	0.496879	0.927397	0.746158	1.152657	0.996292
genitourinary	Noninflammatory disorders of cervix	Inverse variance weighted	2	-0.07847	0.115919	0.498449	0.92453	0.736629	1.160362	0.996292
digestive	Umbilical hernia	Inverse variance weighted	2	-0.03053	0.045208	0.499457	0.96993	0.887684	1.059797	0.996292
dermatologic	Chronic ulcer of skin	Inverse variance weighted	2	-0.0401	0.05973	0.501957	0.96069	0.854555	1.080007	0.996292
circulatory system	Circulatory disease NEC	Inverse variance weighted	2	-0.0142	0.021217	0.503419	0.985904	0.945745	1.027767	0.996292
circulatory system	Bundle branch block	Inverse variance weighted	2	-0.03569	0.053535	0.504986	0.964939	0.868821	1.071692	0.996292
genitourinary	Hematuria	Inverse variance weighted	2	0.024147	0.036229	0.505089	1.024441	0.954219	1.09983	0.996292
musculoskeletal	Hammer toe (acquired)	Inverse variance weighted	2	0.066205	0.099608	0.506275	1.068445	0.87895	1.298795	0.996292
sense organs	Diplopia and disorders of binocular vision	Inverse variance weighted	2	-0.06529	0.098581	0.507754	0.936792	0.772199	1.136468	0.996292
neoplasms	Cancer of bladder	Inverse variance weighted	2	-0.03608	0.054725	0.509703	0.964563	0.866458	1.073775	0.996292
infectious diseases	Septicemia	Inverse variance weighted	2	-0.02775	0.042375	0.51255	0.972631	0.895113	1.056863	0.996292
genitourinary	stress incontinence, female	Inverse variance weighted	2	-0.0236	0.036105	0.513296	0.976674	0.909949	1.048293	0.996292
injuries & poisonings	Poisoning by antibiotics	Inverse variance weighted	2	-0.01381	0.021156	0.513765	0.986281	0.94622	1.028037	0.996292
mental disorders	Psychogenic and somatoform disorders	Inverse variance weighted	2	-0.0756	0.115944	0.514399	0.927191	0.738714	1.163757	0.996292
injuries & poisonings	Injuries to the nervous system	Inverse variance weighted	2	0.047595	0.073568	0.517669	1.048745	0.907919	1.211414	0.996292
pregnancy complications	Known or suspected fetal abnormality affecting management of mother	Inverse variance weighted	2	-0.0502	0.077621	0.517803	0.951039	0.816819	1.107314	0.996292
neurological	Facial nerve disorders [CN7]	Inverse variance weighted	2	0.078838	0.121948	0.517961	1.082029	0.851991	1.374178	0.996292
digestive	Cholelithiasis with acute cholecystitis	Inverse variance weighted	2	0.044231	0.068566	0.518869	1.045224	0.913787	1.195565	0.996292
digestive	Diseases of the oral soft tissues, excluding lesions specific for gingiva and tongue	Inverse variance weighted	2	0.033345	0.051775	0.519552	1.033907	0.934135	1.144335	0.996292
symptoms	Gangrene	Inverse variance weighted	2	-0.08468	0.132618	0.523143	0.918808	0.708497	1.191548	0.996292
genitourinary	Abnormal findings on mammogram or breast exam	Inverse variance weighted	2	-0.04293	0.067388	0.524109	0.95798	0.83945	1.093248	0.996292
genitourinary	Cyst or abscess of Bartholin's gland	Inverse variance weighted	2	-0.06174	0.097102	0.524861	0.940123	0.777194	1.137208	0.996292
infectious diseases	Bacterial infection NOS	Inverse variance weighted	2	0.015783	0.024893	0.526072	1.015908	0.967531	1.066703	0.996292
digestive	Chronic pancreatitis	Inverse variance weighted	2	0.074644	0.11829	0.528023	1.077501	0.854529	1.358652	0.996292
respiratory	Acute tonsillitis	Inverse variance weighted	2	-0.06827	0.108281	0.528347	0.934004	0.755401	1.154834	0.996292
infectious diseases	E. coli	Inverse variance weighted	2	0.032076	0.051115	0.53032	1.032596	0.934158	1.141407	0.996292
circulatory system	Other aneurysm	Inverse variance weighted	2	0.081039	0.129168	0.530404	1.084413	0.841869	1.396833	0.996292
endocrine/metabolic	Hypothyroidism NOS	Inverse variance weighted	2	-0.01481	0.023689	0.531893	0.985301	0.940599	1.032127	0.996292
genitourinary	Cystitis	Inverse variance weighted	2	-0.05317	0.085092	0.532072	0.94822	0.80256	1.120316	0.996292
dermatologic	Pilonidal cyst	Inverse variance weighted	2	-0.06927	0.11094	0.532384	0.933077	0.750728	1.159716	0.996292
neoplasms	Non-Hodgkins lymphoma	Inverse variance weighted	2	-0.03968	0.063562	0.532418	0.961094	0.848517	1.088607	0.996292
genitourinary	Hydronephrosis	Inverse variance weighted	2	0.037848	0.060897	0.534268	1.038573	0.921723	1.170237	0.996292
circulatory system	Aortic valve disease	Inverse variance weighted	2	0.046429	0.074743	0.53448	1.047524	0.904777	1.212792	0.996292
genitourinary	Renal failure NOS	Inverse variance weighted	2	0.043958	0.070909	0.535306	1.044939	0.909352	1.200742	0.996292
circulatory system	Orthostatic hypotension	Inverse variance weighted	2	0.045253	0.073568	0.538477	1.046293	0.905796	1.208582	0.996292
musculoskeletal	Pain in joint	Inverse variance weighted	2	-0.01824	0.029928	0.542127	0.981922	0.92598	1.041242	0.996292
sense organs	Tinnitus	Inverse variance weighted	2	-0.07209	0.11829	0.542214	0.930443	0.737903	1.173223	0.996292
mental disorders	Delirium due to conditions classified elsewhere	Inverse variance weighted	2	-0.0638	0.104762	0.542513	0.938191	0.764041	1.152036	0.996292
circulatory system	Arrhythmia (cardiac) NOS	Inverse variance weighted	2	-0.05392	0.088576	0.542677	0.947505	0.796497	1.127144	0.996292
circulatory system	Other disorders of circulatory system	Inverse variance weighted	2	-0.01274	0.021217	0.548175	0.98734	0.947123	1.029265	0.996292
digestive	Hematemesis	Inverse variance weighted	2	-0.04678	0.078007	0.548728	0.954299	0.818998	1.111951	0.996292
neoplasms	Melanomas of skin, dx or hx	Inverse variance weighted	2	-0.06152	0.103378	0.551753	0.94033	0.767862	1.151536	0.996292
neoplasms	Melanomas of skin	Inverse variance weighted	2	-0.06152	0.103378	0.551753	0.94033	0.767862	1.151536	0.996292
infectious diseases	Intestinal infection	Inverse variance weighted	2	0.016964	0.02863	0.553513	1.017108	0.961605	1.075815	0.996292
neoplasms	Benign neoplasm of skin	Inverse variance weighted	2	-0.01809	0.031132	0.561284	0.982077	0.923943	1.043869	0.996292
genitourinary	Cystitis and urethritis	Inverse variance weighted	2	-0.04848	0.083618	0.562084	0.952679	0.808667	1.122338	0.996292
genitourinary	Calculus of lower urinary tract	Inverse variance weighted	2	-0.10775	0.186088	0.562586	0.897856	0.623455	1.293028	0.996292
genitourinary	Lump or mass in breast	Inverse variance weighted	2	-0.03946	0.068566	0.564996	0.961313	0.840428	1.099586	0.996292
circulatory system	Varicose veins of lower extremity, symptomtic	Inverse variance weighted	2	-0.09036	0.157718	0.566716	0.913606	0.670667	1.244547	0.996292
circulatory system	Hypertensive heart and/or renal disease	Inverse variance weighted	2	-0.07797	0.136116	0.566763	0.924991	0.708391	1.20782	0.996292
pregnancy complications	Obstetrical/birth trauma	Inverse variance weighted	2	-0.02358	0.041197	0.567046	0.976694	0.900929	1.058831	0.996292
digestive	Irritable Bowel Syndrome	Inverse variance weighted	2	-0.0207	0.036166	0.56713	0.979515	0.912485	1.051469	0.996292
digestive	Periapical abscess	Inverse variance weighted	2	-0.04486	0.078571	0.568024	0.95613	0.819664	1.115316	0.996292
injuries & poisonings	Poisoning by other anti-infectives	Inverse variance weighted	2	-0.04333	0.075914	0.568173	0.957598	0.825209	1.111226	0.996292
digestive	Other disorders of liver	Inverse variance weighted	2	0.040297	0.070682	0.568604	1.04112	0.906431	1.195822	0.996292
neoplasms	Carcinoma in situ of skin	Inverse variance weighted	2	-0.05904	0.103584	0.568681	0.942667	0.76946	1.154863	0.996292
respiratory	Pneumococcal pneumonia	Inverse variance weighted	2	0.019698	0.034901	0.572489	1.019893	0.95246	1.0921	0.996292
hematopoietic	Other deficiency anemia	Inverse variance weighted	2	0.089435	0.158565	0.572735	1.093556	0.801434	1.492157	0.996292
injuries & poisonings	Complications of cardiac/vascular device, implant, and graft	Inverse variance weighted	2	-0.04282	0.076065	0.573512	0.958088	0.825387	1.112123	0.996292
genitourinary	Endometrial hyperplasia	Inverse variance weighted	2	0.044653	0.079746	0.575523	1.045665	0.894357	1.222571	0.996292
digestive	Inflammatory bowel disease and other gastroenteritis and colitis	Inverse variance weighted	2	0.041933	0.075142	0.576812	1.042824	0.900014	1.208295	0.996292
circulatory system	Nonspecific abnormal findings on radiological and other examination of other intrathoracic organs (echo	Inverse variance weighted	2	-0.12401	0.223502	0.579005	0.883373	0.570027	1.368967	0.996292
musculoskeletal	Osteoarthritis; localized	Inverse variance weighted	2	0.011755	0.021187	0.57901	1.011825	0.970667	1.054728	0.996292
endocrine/metabolic	Vitamin B-complex deficiencies	Inverse variance weighted	2	0.053806	0.097102	0.579498	1.05528	0.872394	1.276506	0.996292
hematopoietic	Megaloblastic anemia	Inverse variance weighted	2	0.0957	0.173214	0.580612	1.100428	0.783644	1.545272	0.996292
neurological	Multiple sclerosis	Inverse variance weighted	2	0.040538	0.073568	0.581616	1.041371	0.901535	1.202896	0.996292
circulatory system	Cardiac pacemaker/device in situ	Inverse variance weighted	2	0.05007	0.092364	0.587754	1.051345	0.877249	1.25999	0.996292
musculoskeletal	Ankylosing spondylitis	Inverse variance weighted	2	0.114175	0.210746	0.58798	1.120948	0.741643	1.694244	0.996292
digestive	Other disorders of stomach and duodenum	Inverse variance weighted	2	0.024959	0.046112	0.588323	1.025273	0.936673	1.122253	0.996292
pregnancy complications	Early or threatened labor; hemorrhage in early pregnancy	Inverse variance weighted	2	-0.05314	0.098339	0.588942	0.948248	0.782013	1.14982	0.996292
digestive	Cholelithiasis	Inverse variance weighted	2	0.012787	0.023689	0.589343	1.012869	0.966916	1.061006	0.996292
circulatory system	Transient cerebral ischemia	Inverse variance weighted	2	-0.03096	0.057383	0.589502	0.969513	0.866379	1.084924	0.996292
respiratory	Cough	Inverse variance weighted	2	-0.02854	0.05303	0.590498	0.971867	0.875924	1.078319	0.996292
injuries & poisonings	Fracture of humerus	Inverse variance weighted	2	0.060512	0.112701	0.591318	1.062381	0.851818	1.324992	0.996292
musculoskeletal	Fasciitis	Inverse variance weighted	2	-0.02317	0.043641	0.5954	0.977092	0.89699	1.064347	0.996292
digestive	Diaphragmatic hernia	Inverse variance weighted	2	-0.00926	0.01745	0.595669	0.990783	0.957469	1.025256	0.996292
neoplasms	Skin cancer	Inverse variance weighted	2	0.012539	0.023689	0.596591	1.012618	0.966676	1.060743	0.996292
digestive	Dysphagia	Inverse variance weighted	2	0.023666	0.044806	0.597376	1.023948	0.93786	1.117939	0.996292

circulatory system	Cardiac pacemaker in situ	Inverse variance weighted	2	0.053974	0.1027	0.599199	1.055458	0.86302	1.290806	0.996292
genitourinary	Disorders of menstruation and other abnormal bleeding from female genital tract	Inverse variance weighted	2	-0.01104	0.021187	0.602464	0.989025	0.948794	1.030961	0.996292
endocrine/metabolic	Chondrocalcinosis	Inverse variance weighted	2	0.058858	0.113286	0.603375	1.060625	0.849436	1.32432	0.996292
mental disorders	Alcoholic liver damage	Inverse variance weighted	2	0.049205	0.094756	0.603568	1.050435	0.872391	1.264816	0.996292
digestive	Duodenitis	Inverse variance weighted	2	-0.01609	0.031163	0.605528	0.984034	0.925728	1.046013	0.996292
digestive	Acute pancreatitis	Inverse variance weighted	2	0.030842	0.05973	0.605609	1.031322	0.917384	1.159412	0.996292
musculoskeletal	Pathologic fracture	Inverse variance weighted	2	-0.06105	0.11829	0.605767	0.940774	0.746096	1.186249	0.996292
circulatory system	Other forms of chronic heart disease	Inverse variance weighted	2	-0.03266	0.063562	0.607384	0.967869	0.854498	1.096281	0.996292
circulatory system	Cerebral ischemia	Inverse variance weighted	2	-0.02558	0.049849	0.607833	0.974743	0.884011	1.074788	0.996292
genitourinary	Endometriosis	Inverse variance weighted	2	-0.02944	0.057477	0.60846	0.970985	0.867535	1.086772	0.996292
endocrine/metabolic	Hyperpotassemia	Inverse variance weighted	2	0.055082	0.107944	0.609856	1.056627	0.855141	1.305587	0.996292
respiratory	Other dyspnea	Inverse variance weighted	2	0.045161	0.088576	0.610151	1.046197	0.879459	1.244546	0.996292
sense organs	Infection of the eye	Inverse variance weighted	2	0.057539	0.113286	0.611516	1.059227	0.848317	1.322574	0.996292
hematopoietic	Decreased white blood cell count	Inverse variance weighted	2	0.024935	0.0495	0.614445	1.025248	0.930452	1.129702	0.996292
hematopoietic	Neutropenia	Inverse variance weighted	2	0.024935	0.0495	0.614445	1.025248	0.930452	1.129702	0.996292
genitourinary	Noninflammatory disorders of vulva and perineum	Inverse variance weighted	2	0.042491	0.084706	0.615932	1.043406	0.883792	1.231848	0.996292
genitourinary	Other symptoms/disorders or the urinary system	Inverse variance weighted	2	-0.01117	0.022365	0.617488	0.988893	0.94648	1.033206	0.996292
digestive	Heartburn	Inverse variance weighted	2	-0.02924	0.058559	0.617491	0.971179	0.86587	1.089295	0.996292
digestive	Ascites (non malignant)	Inverse variance weighted	2	0.033637	0.067388	0.617671	1.034209	0.906247	1.18024	0.996292
endocrine/metabolic	Nontoxic nodular goiter	Inverse variance weighted	2	-0.05109	0.102352	0.61769	0.950197	0.777481	1.16128	0.996292
mental disorders	Swelling, mass, or lump in head and neck [Space-occupying lesion, intracranial NOS]	Inverse variance weighted	2	-0.0435	0.088576	0.623348	0.957432	0.804841	1.138952	0.996292
circulatory system	Tachycardia NOS	Inverse variance weighted	2	-0.02809	0.057383	0.62452	0.972304	0.868873	1.088048	0.996292
circulatory system	Endocarditis	Inverse variance weighted	2	0.110931	0.227024	0.625104	1.117318	0.716028	1.743507	0.996292
sense organs	Other disorders of eye	Inverse variance weighted	2	-0.02638	0.05439	0.627603	0.97396	0.875475	1.083524	0.996292
circulatory system	Pericarditis	Inverse variance weighted	2	-0.03621	0.074743	0.628013	0.964433	0.833009	1.116592	0.996292
endocrine/metabolic	Thyrotoxicosis with or without goiter	Inverse variance weighted	2	-0.02987	0.062386	0.632045	0.970568	0.85886	1.096807	0.996292
respiratory	Shortness of breath	Inverse variance weighted	2	0.016707	0.034901	0.632141	1.016848	0.949616	1.08884	0.996292
neurological	Other peripheral nerve disorders	Inverse variance weighted	2	-0.02161	0.045329	0.633556	0.978622	0.895426	1.069548	0.996292
genitourinary	Symptoms involving female genital tract	Inverse variance weighted	2	-0.01593	0.033634	0.635711	0.984194	0.921405	1.051261	0.996292
circulatory system	Pulmonary heart disease	Inverse variance weighted	2	-0.02091	0.04428	0.636699	0.979303	0.897894	1.068093	0.996292
injuries & poisonings	Torus fracture	Inverse variance weighted	2	-0.03102	0.065903	0.637889	0.969459	0.851984	1.103132	0.996292
congenital anomalies	Cardiac and circulatory congenital anomalies	Inverse variance weighted	2	-0.02998	0.06383	0.638535	0.970461	0.856337	1.099794	0.996292
genitourinary	Polyp of corpus uteri	Inverse variance weighted	2	0.015899	0.03387	0.638782	1.016026	0.950766	1.085765	0.996292
injuries & poisonings	Contusion	Inverse variance weighted	2	-0.03229	0.069739	0.643331	0.968223	0.844525	1.11004	0.996292
genitourinary	Other signs and symptoms in breast	Inverse variance weighted	2	-0.04646	0.100934	0.645305	0.954603	0.78326	1.163428	0.996292
respiratory	Empyema and pneumothorax	Inverse variance weighted	2	0.092232	0.200482	0.645481	1.096619	0.74029	1.624463	0.996292
dermatologic	Psoriasis vulgaris	Inverse variance weighted	2	0.029671	0.064735	0.646707	1.030115	0.907366	1.16947	0.996292
digestive	Flatulence	Inverse variance weighted	2	-0.03427	0.074811	0.646902	0.966312	0.834521	1.118916	0.996292
musculoskeletal	Bursitis	Inverse variance weighted	2	-0.04146	0.090927	0.648383	0.959384	0.802776	1.146545	0.996292
respiratory	Obstructive chronic bronchitis	Inverse variance weighted	2	-0.02377	0.05232	0.649651	0.976514	0.881338	1.081967	0.996292
pregnancy complications	Complications of labor and delivery NEC	Inverse variance weighted	2	-0.05042	0.111793	0.651968	0.950828	0.763734	1.183756	0.996292
injuries & poisonings	Complications of surgical and medical procedures	Inverse variance weighted	2	-0.01238	0.027456	0.652019	0.987695	0.935949	1.042302	0.996292
neoplasms	Benign neoplasm of brain, cranial nerves, meninges	Inverse variance weighted	2	-0.04325	0.09593	0.652077	0.957669	0.79352	1.155775	0.996292
sense organs	Subjective visual disturbances	Inverse variance weighted	2	0.058487	0.130566	0.654187	1.060232	0.820844	1.369433	0.996292
sense organs	Otitis media	Inverse variance weighted	2	-0.02826	0.063562	0.656621	0.972137	0.858266	1.101115	0.996292
dermatologic	Diseases of hair and hair follicles	Inverse variance weighted	2	0.032847	0.074218	0.658071	1.033393	0.89349	1.195201	0.996292
symptoms	Malaise and fatigue	Inverse variance weighted	2	0.020375	0.046112	0.658591	1.020584	0.93239	1.117121	0.996292
endocrine/metabolic	Type 1 diabetes	Inverse variance weighted	2	-0.02962	0.067226	0.659451	0.97081	0.850962	1.107537	0.996292
respiratory	Pneumonitis due to inhalation of food or vomitus	Inverse variance weighted	2	-0.04877	0.11094	0.660205	0.952397	0.766273	1.18373	0.996292
neoplasms	Malignant neoplasm of female breast	Inverse variance weighted	2	-0.0115	0.02616	0.660321	0.988569	0.939159	1.040579	0.996292
musculoskeletal	Arthropathy NOS	Inverse variance weighted	2	0.006567	0.014978	0.661055	1.006589	0.977468	1.036578	0.996292
neoplasms	Benign neoplasm of brain and other parts of nervous system	Inverse variance weighted	2	-0.04062	0.093266	0.663191	0.960196	0.799779	1.152787	0.996292
musculoskeletal	Osteoarthrosis	Inverse variance weighted	2	0.007581	0.017419	0.663387	1.00761	0.97379	1.042604	0.996292
musculoskeletal	Other disorders of cervical region	Inverse variance weighted	2	-0.07996	0.184447	0.664629	0.92315	0.643084	1.325186	0.996292
sense organs	Retinal detachments and defects	Inverse variance weighted	2	0.020463	0.047348	0.665601	1.020674	0.930217	1.119928	0.996292
pregnancy complications	Hypertension complicating pregnancy, childbirth, and the puerperium	Inverse variance weighted	2	0.035516	0.082395	0.666432	1.036155	0.881634	1.217757	0.996292
musculoskeletal	Acquired foot deformities	Inverse variance weighted	2	0.011724	0.027426	0.669029	1.011793	0.95884	1.067671	0.996292
sense organs	Otitis externa	Inverse variance weighted	2	0.04987	0.117118	0.670246	1.051135	0.835537	1.322364	0.996292
musculoskeletal	Osteoarthrosis, localized, primary	Inverse variance weighted	2	0.012203	0.028662	0.670278	1.012278	0.956979	1.070772	0.996292
genitourinary	Other abnormality of urination	Inverse variance weighted	2	-0.037	0.087428	0.672127	0.963674	0.811914	1.1438	0.996292
symptoms	Other ill-defined and unknown causes of morbidity and mortality	Inverse variance weighted	2	-0.00947	0.022391	0.672354	0.990575	0.948044	1.035015	0.996292
digestive	Acute gastritis	Inverse variance weighted	2	0.033222	0.078571	0.672421	1.03378	0.886231	1.205895	0.996292
genitourinary	Disorders of uterus, NEC	Inverse variance weighted	2	0.019306	0.046142	0.675656	1.019493	0.931338	1.115993	0.996292
neoplasms	Cervical cancer	Inverse variance weighted	2	0.027445	0.065903	0.677087	1.027825	0.903277	1.169546	0.996292
neoplasms	Malignant neoplasm of rectum, rectosigmoid junction, and anus	Inverse variance weighted	2	0.024346	0.058559	0.677591	1.024645	0.913539	1.149264	0.996292
pregnancy complications	Other complications of pregnancy NEC	Inverse variance weighted	2	0.035135	0.08452	0.677632	1.035759	0.877635	1.222374	0.996292
neoplasms	Colon cancer	Inverse variance weighted	2	0.040265	0.097011	0.678099	1.041087	0.860815	1.259112	0.996292
circulatory system	Chronic pulmonary heart disease	Inverse variance weighted	2	-0.11051	0.268954	0.68115	0.895376	0.528526	1.516854	0.996292
neurological	Other cerebral degenerations	Inverse variance weighted	2	-0.03816	0.093072	0.681812	0.96256	0.802053	1.155187	0.996292
musculoskeletal	Other derangement of joint	Inverse variance weighted	2	0.024263	0.05973	0.684591	1.02456	0.911368	1.151809	0.996292
musculoskeletal	Other arthropathies	Inverse variance weighted	2	0.005938	0.014949	0.691219	1.005955	0.976909	1.035865	0.996292
digestive	Other disorders of biliary tract	Inverse variance weighted	2	-0.03314	0.083574	0.691687	0.967401	0.821234	1.139583	0.996292
digestive	Gastritis and duodenitis	Inverse variance weighted	2	-0.00643	0.016243	0.692166	0.99359	0.962457	1.02573	0.996292
neoplasms	Large cell lymphoma	Inverse variance weighted	2	0.044825	0.114093	0.694409	1.045844	0.836276	1.30793	0.996292
respiratory	Nasal polyps	Inverse variance weighted	2	0.018577	0.047348	0.694792	1.018751	0.928464	1.117818	0.996292
endocrine/metabolic	Crystal arthropathies	Inverse variance weighted	2	0.041553	0.107111	0.698058	1.042428	0.845029	1.28594	0.996292
sense organs	Inflammation of the eye	Inverse variance weighted	2	0.018285	0.047348	0.699354	1.018453	0.928193	1.117492	0.996292
circulatory system	Right bundle branch block	Inverse variance weighted	2	-0.02681	0.069739	0.700675	0.973548	0.849169	1.116144	0.996292
digestive	Jaundice (not of newborn)	Inverse variance weighted	2	-0.07809	0.204119	0.702054	0.924885	0.619924	1.379867	0.996292
digestive	Noninfectious gastroenteritis	Inverse variance weighted	2	-0.01941	0.050844	0.702683	0.98078	0.887754	1.083554	0.996292
genitourinary	Menopausal and postmenopausal disorders	Inverse variance weighted	2	-0.00994	0.02619	0.704372	0.990112	0.940569	1.042265	0.996292
genitourinary	Infertility, female	Inverse variance weighted	2	-0.0282	0.074743	0.705986	0.972197	0.839715	1.125581	0.996292
neoplasms	Lipoma of skin and subcutaneous tissue	Inverse variance weighted	2	0.034628	0.09199	0.706595	1.035235	0.864441	1.239774	0.996292
symptoms	Symptoms concerning nutrition, metabolism, and development	Inverse variance weighted	2	-0.01343	0.036166	0.710454	0.986663	0.919144	1.059142	0.996292
mental disorders	Aphasia/speech disturbance	Inverse variance weighted	2	-0.04978	0.134403	0.711125	0.951443	0.731099	1.238195	0.996292
dermatologic	Seborrheic keratosis	Inverse variance weighted	2	0.017909	0.048583	0.712404	1.01807	0.9256	1.119779	0.996292
endocrine/metabolic	Nontoxic multinodular goiter	Inverse variance weighted	2	-0.0896	0.246432	0.716171	0.914299	0.564054	1.482024	0.996292
genitourinary	Cyst of kidney, acquired	Inverse variance weighted	2	0.027127	0.074743	0.716656	1.027498	0.88748	1.189607	0.996292
sense organs	Perforation of tympanic membrane	Inverse variance weighted	2	0.030224	0.083574	0.717622	1.030685	0.874956	1.214131	0.996292
symptoms	Nausea and vomiting	Inverse variance weighted	2	-0.01209	0.033445	0.717774	0.987984	0.925296	1.05492	0.996292
musculoskeletal	Osteopenia or other disorder of bone and cartilage	Inverse variance weighted	2	-0.03912	0.108903	0.719399	0.961631	0.776799	1.190442	0.996292
sense organs	Inflammation of eyelids	Inverse variance weighted	2	-0.01943	0.054725	0.722509	0.980754	0.881003	1.0918	0.996292
symptoms	Symptoms involving skin and other integumentary tissue	Inverse variance weighted	2	0.022452	0.063562	0.723921	1.022706	0.902912	1.158393	0.996292
genitourinary	Stricture/obstruction of ureter	Inverse variance weighted	2	0.031132	0.088576	0.725236	1.031622	0.867207	1.227208	0.996292
injuries & poisonings	Hemorrhage or hematoma complicating a procedure	Inverse variance weighted	2	0.016865	0.048066	0.725692	1.017008	0.92557	1.117478	0.996292
dermatologic	Cellulitis and abscess of face/neck	Inverse variance weighted	2	0.070721	0.201842	0.726055	1.073282	0.722608	1.594135	0.996292
sense organs	Disorders of conjunctiva	Inverse variance weighted	2	0.033889	0.097102	0.727087	1.03447	0.85519	1.251333	0.996292
circulatory system	Hypertensive chronic kidney disease	Inverse variance weighted	2	-0.05235	0.150066	0.727217	0.948999	0.707176	1.273516	0.996292
endocrine/metabolic	Other disorders of pancreatic internal secretion	Inverse variance weighted	2	0.05631	0.161513	0.727359	1.057926	0.770855	1.451903	0.996292
infectious diseases	Viral hepatitis	Inverse variance weighted	2	-0.03578	0.104493	0.732011	0.964849	0.786164	1.184146	0.996292
circulatory system	Congestive heart failure (CHF) NOS	Inverse variance weighted	2	-0.02036	0.05973	0.733228	0.979848	0.871596	1.101544	0.996292
circulatory system	Phlebitis and thrombophlebitis of lower extremities	Inverse variance weighted	2	0.020047	0.059316	0.735382	1.020249	0.908272	1.146032	0.996292
injuries & poisonings	Open wounds of head; neck; and trunk	Inverse variance weighted	2	0.014734	0.04361	0.735477	1.014843	0.931702	1.105403	0.996292
neoplasms	Cancer of larynx, pharynx, nasal cavities	Inverse variance weighted	2	-0.03614	0.107111	0.735822	0.964507	0.781864	1.189816	0.996292
sense organs	Retinal detachment with retinal defect	Inverse variance weighted	2	0.024375	0.072391	0.73633	1.024675	0.889131	1.180881	0.996292
circulatory system	Atrioventricular block, complete	Inverse variance weighted	2	0.062532	0.185902	0.736591	1.064529	0.73946	1.532499	0.996292
genitourinary	Benign mammary dysplasias	Inverse variance weighted	2	-0.01843	0.05487	0.736951	0.981738	0.881637	1.093206	0.996292
respiratory	Asthma	Inverse variance weighted	2	-0.00583	0.01745	0.738291	0.994187	0.960758	1.028778	0.996292
circulatory system	Atrioventricular [AV] block	Inverse variance weighted	2	-0.02797	0.083751	0.738443	0.972422	0.82521	1.145895	0.996292
injuries & poisonings	Effects radiation NOS	Inverse variance weighted	2	-0.01621	0.048583	0.738617	0.983919	0.894551	1.082216	0.996292
digestive	Stricture and stenosis of esophagus	Inverse variance weighted	2	0.020773	0.062386	0.739147	1.020991	0.903479	1.153787	0.996292
digestive	Ulcer of esophagus	Inverse variance weighted	2	-0.02492	0.075245	0.740522	0.975389	0.841643	1.13039	0.996292
genitourinary	Pelvic inflammatory disease (PID)	Inverse variance weighted	2	0.043632	0.132282	0.741517	1.044598	0.806026	1.353785	0.996292
injuries & poisonings	Traumatic arthropathy	Inverse variance weighted	2	0.038203	0.115944	0.74178	1.038942	0.827748	1.304021	0.996292

symptoms	Chronic fatigue syndrome	Inverse variance weighted	2	0.035822	0.109764	0.744154	1.036472	0.835842	1.285259	0.996292
digestive	Chronic liver disease and cirrhosis	Inverse variance weighted	2	0.01624	0.049849	0.744583	1.016373	0.921766	1.12069	0.996292
circulatory system	Carditis	Inverse variance weighted	2	0.019284	0.05973	0.746809	1.019471	0.906842	1.146089	0.996292
digestive	Gastrointestinal hemorrhage	Inverse variance weighted	2	0.008692	0.026928	0.746864	1.008729	0.956871	1.063398	0.996292
neurological	Disorders of other cranial nerves	Inverse variance weighted	2	0.023277	0.072391	0.747799	1.02355	0.888155	1.179584	0.996292
digestive	Ulceration of the lower GI tract	Inverse variance weighted	2	0.026322	0.082087	0.74847	1.026671	0.874092	1.205885	0.996292
neoplasms	Malignant neoplasm of other and ill-defined sites within the digestive organs and peritoneum	Inverse variance weighted	2	0.011499	0.036136	0.75033	1.011565	0.942398	1.085809	0.996292
endocrine/metabolic	Hypoglycemia	Inverse variance weighted	2	0.052684	0.16593	0.75086	1.054096	0.761443	1.459228	0.996292
hematopoietic	Purpura and other hemorrhagic conditions	Inverse variance weighted	2	-0.0201	0.063562	0.751857	0.980103	0.865299	1.110138	0.996292
circulatory system	Cardiac conduction disorders	Inverse variance weighted	2	-0.02387	0.075815	0.752894	0.976414	0.841587	1.132841	0.996292
dermatologic	Other hypertrophic and atrophic conditions of skin	Inverse variance weighted	2	-0.01559	0.050122	0.755708	0.984527	0.892407	1.086156	0.996292
respiratory	Chronic bronchitis	Inverse variance weighted	2	0.015445	0.049849	0.75668	1.015565	0.921033	1.1198	0.996292
genitourinary	Noninflammatory disorders of vagina	Inverse variance weighted	2	0.019474	0.063562	0.759322	1.019665	0.900227	1.154949	0.996292
digestive	Abdominal hernia	Inverse variance weighted	2	-0.00418	0.013681	0.760208	0.995833	0.969484	1.022898	0.996292
respiratory	Bronchitis	Inverse variance weighted	2	-0.03193	0.105937	0.763129	0.968577	0.786971	1.192093	0.996292
circulatory system	Heart valve disorders	Inverse variance weighted	2	0.014124	0.046931	0.763441	1.014225	0.925094	1.111943	0.996292
pregnancy complications	Miscarriage; stillbirth	Inverse variance weighted	2	-0.0256	0.085755	0.765345	0.97473	0.823926	1.153135	0.996292
injuries & poisonings	Other open wound of head and face	Inverse variance weighted	2	-0.01483	0.049849	0.76606	0.985278	0.893565	1.086404	0.996292
hematopoietic	Thrombocytopenia	Inverse variance weighted	2	-0.02787	0.094591	0.768281	0.972516	0.807941	1.170615	0.996292
neoplasms	Neoplasm of unspecified nature of digestive system	Inverse variance weighted	2	0.024182	0.082395	0.769148	1.024477	0.871698	1.204032	0.996292
digestive	Other biliary tract disease	Inverse variance weighted	2	0.012715	0.04361	0.770632	1.012796	0.929822	1.103173	0.996292
neoplasms	Malignant neoplasm of ovary	Inverse variance weighted	2	0.017024	0.058559	0.771268	1.01717	0.906874	1.14088	0.996292
respiratory	Abnormal sputum	Inverse variance weighted	2	-0.01668	0.057383	0.771348	0.983462	0.878844	1.100534	0.996292
digestive	Other chronic nonalcoholic liver disease	Inverse variance weighted	2	-0.01895	0.065903	0.773744	0.981232	0.862331	1.116529	0.996292
genitourinary	Pyelonephritis	Inverse variance weighted	2	0.043478	0.151656	0.774351	1.044437	0.775872	1.405964	0.996292
musculoskeletal	Rheumatism, unspecified and fibrositis	Inverse variance weighted	2	0.032087	0.112106	0.774708	1.032608	0.828912	1.286359	0.996292
dermatologic	Diseases of nail, NOS	Inverse variance weighted	2	-0.02136	0.074743	0.775048	0.978867	0.845476	1.133303	0.996292
endocrine/metabolic	Hypothyroidism	Inverse variance weighted	2	-0.00672	0.023658	0.776425	0.993304	0.948296	1.040448	0.996292
symptoms	Edema	Inverse variance weighted	2	0.018663	0.065903	0.777027	1.018839	0.89538	1.15932	0.996292
circulatory system	Ischemic Heart Disease	Inverse variance weighted	2	-0.00459	0.016243	0.777608	0.995423	0.964232	1.027623	0.996292
respiratory	Other diseases of lung	Inverse variance weighted	2	0.03617	0.128299	0.778002	1.036833	0.806303	1.333273	0.996292
infectious diseases	Gram negative septicemia	Inverse variance weighted	2	-0.02589	0.092098	0.778589	0.974438	0.813503	1.167211	0.996292
infectious diseases	Viral infection	Inverse variance weighted	2	-0.01432	0.051053	0.779102	0.985782	0.891915	1.089528	0.996292
dermatologic	Symptoms affecting skin	Inverse variance weighted	2	0.010119	0.036136	0.779466	1.01017	0.941098	1.084312	0.996292
digestive	Esophageal bleeding (varices/hemorrhage)	Inverse variance weighted	2	0.017883	0.064735	0.782352	1.018044	0.896733	1.155766	0.996292
symptoms	Syncope and collapse	Inverse variance weighted	2	-0.0079	0.02863	0.782665	0.992134	0.937993	1.049399	0.996292
sense organs	Cholesteatoma	Inverse variance weighted	2	0.029843	0.108281	0.782854	1.030292	0.833277	1.273889	0.996292
genitourinary	Nephritis; nephrosis; renal sclerosis	Inverse variance weighted	2	-0.01874	0.068566	0.784623	0.981436	0.85802	1.122602	0.996292
hematopoietic	Disorders of iron metabolism	Inverse variance weighted	2	0.02793	0.103584	0.787441	1.028323	0.839378	1.259801	0.996292
respiratory	Respiratory failure, insufficiency, arrest	Inverse variance weighted	2	-0.02509	0.093094	0.787544	0.975223	0.81257	1.170434	0.996292
symptoms	Other symptoms	Inverse variance weighted	2	0.020452	0.075914	0.787615	1.020662	0.879555	1.184408	0.996292
endocrine/metabolic	Type 2 diabetes with neurological manifestations	Inverse variance weighted	2	0.029931	0.112114	0.78949	1.030384	0.827114	1.283609	0.996292
neoplasms	Cancer of mouth	Inverse variance weighted	2	-0.02824	0.105937	0.78983	0.972159	0.789881	1.196501	0.996292
digestive	Periodontitis (acute or chronic)	Inverse variance weighted	2	-0.02236	0.083984	0.790056	0.977888	0.82947	1.152864	0.996292
genitourinary	Irregular menstrual cycle/bleeding	Inverse variance weighted	2	-0.00596	0.022453	0.790653	0.994057	0.951259	1.038781	0.996292
circulatory system	Congestive heart failure; nonhypertensive	Inverse variance weighted	2	-0.00992	0.037371	0.79077	0.990134	0.920201	1.065382	0.996292
musculoskeletal	Rheumatoid arthritis	Inverse variance weighted	2	0.013194	0.050059	0.792109	1.013282	0.918584	1.117742	0.996292
genitourinary	Ovarian cyst	Inverse variance weighted	2	0.010429	0.039842	0.793515	1.010483	0.934577	1.092554	0.996292
circulatory system	Other venous embolism and thrombosis	Inverse variance weighted	2	-0.03266	0.125066	0.793984	0.967868	0.757456	1.236729	0.996292
genitourinary	Urinary tract infection	Inverse variance weighted	2	0.006064	0.023689	0.797973	1.006082	0.960437	1.053896	0.996292
neoplasms	Cancer of urinary organs (incl. kidney and bladder)	Inverse variance weighted	2	-0.01083	0.042344	0.798129	0.989228	0.910443	1.074831	0.996292
dermatologic	Disturbance of skin sensation	Inverse variance weighted	2	-0.01268	0.049849	0.799161	0.987397	0.895487	1.08874	0.996292
musculoskeletal	Peripheral enthesopathies and allied syndromes	Inverse variance weighted	2	-0.00564	0.022453	0.801613	0.994374	0.951563	1.039112	0.996292
musculoskeletal	Other specified osteoporosis	Inverse variance weighted	2	-0.02911	0.115944	0.801732	0.971306	0.773861	1.219127	0.996292
respiratory	Emphysema	Inverse variance weighted	2	0.016178	0.064735	0.802657	1.016309	0.895205	1.153797	0.996292
dermatologic	Cellulitis and abscess of trunk	Inverse variance weighted	2	-0.04461	0.178834	0.803027	0.956373	0.673598	1.357857	0.996292
genitourinary	Inflammatory diseases of female pelvic organs	Inverse variance weighted	2	0.008692	0.03487	0.803147	1.00873	0.942092	1.080081	0.996292
genitourinary	Dysmenorrhea	Inverse variance weighted	2	-0.01581	0.063562	0.803624	0.984319	0.869021	1.114913	0.996292
endocrine/metabolic	Hyposmolality and/or hyponatremia	Inverse variance weighted	2	0.015484	0.062386	0.803987	1.015604	0.898712	1.1477	0.996292
neoplasms	Malignant neoplasm of ovary and other uterine adnexa	Inverse variance weighted	2	0.014492	0.058559	0.804533	1.014598	0.904581	1.137995	0.996292
circulatory system	Unstable angina (intermediate coronary syndrome)	Inverse variance weighted	2	0.009048	0.037402	0.808842	1.009089	0.937761	1.085843	0.996292
pregnancy complications	Fetal distress and abnormal forces of labor	Inverse variance weighted	2	0.015851	0.068207	0.816228	1.015978	0.888843	1.161297	0.996292
injuries & poisonings	Poisoning/allergy of sulfonamides	Inverse variance weighted	2	-0.02069	0.090927	0.81996	0.979518	0.819623	1.170606	0.996292
dermatologic	Disorder of skin and subcutaneous tissue NOS	Inverse variance weighted	2	-0.00791	0.034901	0.820712	0.992122	0.926525	1.062363	0.996292
digestive	Femoral hernia	Inverse variance weighted	2	0.023719	0.104762	0.820884	1.024002	0.833923	1.257407	0.996292
respiratory	Diseases of the larynx and vocal cords	Inverse variance weighted	2	-0.0118	0.05232	0.821507	0.988266	0.891945	1.094988	0.996292
hematopoietic	Other diseases of blood and blood-forming organs	Inverse variance weighted	2	-0.01357	0.061058	0.824133	0.986523	0.875253	1.111938	0.996292
dermatologic	Other dyschromia	Inverse variance weighted	2	0.021683	0.097667	0.824308	1.02192	0.84388	1.237522	0.996292
musculoskeletal	Unspecified polyarthropathy or polyarthritis	Inverse variance weighted	2	-0.01011	0.046049	0.826273	0.989944	0.904509	1.083448	0.996292
sense organs	Disorders of vitreous body	Inverse variance weighted	2	-0.02641	0.121286	0.827614	0.973934	0.767872	1.235294	0.996292
neurological	Other headache syndromes	Inverse variance weighted	2	-0.00677	0.031132	0.827745	0.993249	0.934453	1.055743	0.996292
respiratory	Postinflammatory pulmonary fibrosis	Inverse variance weighted	2	-0.01952	0.089753	0.827872	0.980674	0.82248	1.169295	0.996292
circulatory system	Phlebitis and thrombophlebitis	Inverse variance weighted	2	0.012576	0.057922	0.828114	1.012656	0.903977	1.1344	0.996292
neoplasms	Cervical intraepithelial neoplasia [CIN] [Cervical dysplasia]	Inverse variance weighted	2	-0.0251	0.115951	0.828648	0.975216	0.776965	1.224053	0.996292
infectious diseases	Candidiasis	Inverse variance weighted	2	-0.02916	0.135376	0.829445	0.971259	0.744906	1.266395	0.996292
hematopoietic	Diseases of white blood cells	Inverse variance weighted	2	0.009166	0.04361	0.833526	1.009208	0.926529	1.099266	0.996292
dermatologic	Decubitus ulcer	Inverse variance weighted	2	0.018607	0.088576	0.833611	1.018782	0.856413	1.211934	0.996292
sense organs	Amblyopia	Inverse variance weighted	2	0.023976	0.114767	0.834521	1.024265	0.817939	1.282638	0.996292
neoplasms	Secondary malignancy of lymph nodes	Inverse variance weighted	2	-0.00779	0.03734	0.834645	0.992236	0.922212	1.067576	0.996292
injuries & poisonings	Crushing or internal injury to organs	Inverse variance weighted	2	-0.02707	0.129716	0.834694	0.973293	0.754791	1.255049	0.996292
mental disorders	Symptoms involving head and neck	Inverse variance weighted	2	0.010111	0.048614	0.835237	1.010162	0.918355	1.111148	0.996292
injuries & poisonings	Anaphylactic shock NOS	Inverse variance weighted	2	-0.02297	0.114455	0.840923	0.977289	0.780902	1.223065	0.996292
pregnancy complications	Normal delivery	Inverse variance weighted	2	0.01952	0.097586	0.84146	1.019712	0.842191	1.234651	0.996292
digestive	Ulcerative colitis (chronic)	Inverse variance weighted	2	0.022818	0.114767	0.842405	1.02308	0.816993	1.281153	0.996292
genitourinary	Dysuria	Inverse variance weighted	2	-0.0153	0.077082	0.842621	0.984813	0.846721	1.145425	0.996292
neoplasms	Breast cancer [female]	Inverse variance weighted	2	-0.00493	0.024925	0.843222	0.995083	0.947639	1.044902	0.996292
respiratory	Pneumonia	Inverse variance weighted	2	-0.00538	0.027395	0.844274	0.994633	0.942635	1.0495	0.996292
endocrine/metabolic	Disorders of parathyroid gland	Inverse variance weighted	2	-0.01756	0.089753	0.844843	0.982589	0.824086	1.171577	0.996292
neoplasms	Myeloproliferative disease	Inverse variance weighted	2	0.016198	0.08475	0.848422	1.01633	0.860784	1.199984	0.996292
neoplasms	Neoplasm of uncertain behavior	Inverse variance weighted	2	-0.01336	0.070909	0.850585	0.986732	0.858697	1.133856	0.996292
pregnancy complications	Early onset of delivery	Inverse variance weighted	2	-0.02066	0.109764	0.850672	0.979548	0.789937	1.214672	0.996292
dermatologic	Ingrowing nail	Inverse variance weighted	2	0.016148	0.085923	0.85093	1.016279	0.858764	1.202685	0.996292
genitourinary	Noninflammatory female genital disorders	Inverse variance weighted	2	0.006169	0.032932	0.851416	1.006188	0.943293	1.073276	0.996292
circulatory system	Other acute and subacute forms of ischemic heart disease	Inverse variance weighted	2	-0.02194	0.11866	0.853333	0.978302	0.775296	1.234465	0.996292
neurological	Hemiplegia	Inverse variance weighted	2	-0.01287	0.069739	0.853558	0.98721	0.861086	1.131808	0.996292
mental disorders	Phobia	Inverse variance weighted	2	-0.02174	0.11946	0.855609	0.978497	0.774235	1.236648	0.996292
dermatologic	Scar conditions and fibrosis of skin	Inverse variance weighted	2	-0.0127	0.070184	0.856358	0.987376	0.860479	1.132987	0.996292
circulatory system	Palpitations	Inverse variance weighted	2	-0.00754	0.04361	0.86267	0.992485	0.911175	1.08105	0.996292
genitourinary	Polyp of female genital organs	Inverse variance weighted	2	0.004524	0.02619	0.862856	1.004534	0.95427	1.057447	0.996292
sense organs	Cataract	Inverse variance weighted	2	-0.0082	0.047903	0.864062	0.991832	0.902947	1.089467	0.996292
neoplasms	Pancreatic cancer	Inverse variance weighted	2	0.018999	0.113727	0.867326	1.019181	0.815538	1.273673	0.996292
infectious diseases	Sepsis	Inverse variance weighted	2	-0.00832	0.051053	0.870479	0.99171	0.897279	1.09608	0.996292
infectious diseases	Sepsis and SIRS	Inverse variance weighted	2	-0.00832	0.051053	0.870479	0.99171	0.897279	1.09608	0.996292
hematopoietic	Coagulation defects	Inverse variance weighted	2	0.019884	0.122112	0.87065	1.020083	0.802956	1.295923	0.996292
congenital anomalies	Digestive congenital anomalies	Inverse variance weighted	2	-0.01834	0.115863	0.874224	0.981827	0.782366	1.232139	0.996292
digestive	Other intestinal obstruction	Inverse variance weighted	2	-0.01663	0.105087	0.874247	0.983506	0.800434	1.208449	0.996292
mental disorders	Agorophobia, social phobia, and panic disorder	Inverse variance weighted	2	-0.01596	0.100934	0.874377	0.984169	0.807519	1.199462	0.996292
injuries & poisonings	Fracture of pelvis	Inverse variance weighted	2	-0.01543	0.099759	0.877059	0.984686	0.809807	1.197331	0.996292
sense organs	Other disorders of middle ear and mastoid	Inverse variance weighted	2	-0.01933	0.125313	0.877417	0.980857	0.767251	1.253932	0.996292
musculoskeletal	Spondylosis without myelopathy	Inverse variance weighted	2	-0.01033	0.067637	0.878586	0.989721	0.866839	1.130022	0.996292
genitourinary	Benign neoplasm of breast	Inverse variance weighted	2	-0.01074	0.072065	0.881514	0.989316	0.858997	1.139406	0.996292
genitourinary	Abnormal findings on examination of urine	Inverse variance weighted	2	0.010665	0.073497	0.884621	1.010723	0.875125	1.167331	0.996292
sense organs	Senile cataract	Inverse variance weighted	2	-0.01035	0.073146	0.88753	0.989708	0.85752	1.142274	0.996292
endocrine/metabolic	Other disorders of metabolism	Inverse variance weighted	2	0.018409	0.134169	0.890864	1.01858	0.783047	1.324958	0.996292

digestive	Disorders of tooth development	Inverse variance weighted	2	-0.00722	0.053555	0.892773	0.992807	0.893877	1.102686	0.996292
dermatologic	Sicca syndrome	Inverse variance weighted	2	0.015698	0.11829	0.894425	1.015822	0.805614	1.28088	0.996292
symptoms	Other tests	Inverse variance weighted	2	0.004515	0.034901	0.897057	1.004526	0.938108	1.075645	0.996292
mental disorders	Bipolar	Inverse variance weighted	2	-0.01056	0.082087	0.897654	0.989497	0.842442	1.162221	0.996292
respiratory	Hemoptysis	Inverse variance weighted	2	-0.00748	0.058559	0.898404	0.992551	0.884925	1.113267	0.996292
digestive	Hemorrhage of gastrointestinal tract	Inverse variance weighted	2	0.010842	0.085237	0.898789	1.0109	0.855368	1.194714	0.996292
digestive	Ulcerative colitis	Inverse variance weighted	2	0.012506	0.100646	0.901108	1.012585	0.831304	1.233397	0.996292
musculoskeletal	Contracture of palmar fascia [Dupuytren's disease]	Inverse variance weighted	2	-0.00568	0.046081	0.901933	0.994338	0.908467	1.088326	0.996292
digestive	Other disorders of intestine	Inverse variance weighted	2	-0.00494	0.041139	0.904395	0.995071	0.917985	1.07863	0.996292
hematopoietic	Other anemias	Inverse variance weighted	2	-0.00298	0.024893	0.904705	0.997024	0.949547	1.046876	0.996292
sense organs	Glaucoma	Inverse variance weighted	2	0.006247	0.052178	0.904709	1.006266	0.908443	1.114623	0.996292
congenital anomalies	Other congenital musculoskeletal anomalies	Inverse variance weighted	2	-0.03543	0.298021	0.905362	0.965189	0.538185	1.730981	0.996292
neurological	Nerve root and plexus disorders	Inverse variance weighted	2	-0.00943	0.079746	0.905874	0.990615	0.847273	1.158208	0.996292
dermatologic	Pruritus and related conditions	Inverse variance weighted	2	0.01115	0.09593	0.907467	1.011213	0.837886	1.220394	0.996292
digestive	Disorders of esophageal motility	Inverse variance weighted	2	-0.01211	0.105937	0.908987	0.987963	0.802722	1.215951	0.996292
musculoskeletal	Rheumatoid arthritis and other inflammatory polyarthropathies	Inverse variance weighted	2	0.005192	0.046535	0.911159	1.005206	0.91758	1.1012	0.996292
sense organs	Meniere's disease	Inverse variance weighted	2	0.012019	0.11094	0.913726	1.012092	0.814302	1.257924	0.996292
musculoskeletal	Disorders of muscle, ligament, and fascia	Inverse variance weighted	2	-0.00439	0.041077	0.914896	0.99562	0.918604	1.079093	0.996292
dermatologic	Unspecified diffuse connective tissue disease	Inverse variance weighted	2	0.005292	0.051115	0.917544	1.005306	0.909469	1.111241	0.996292
endocrine/metabolic	Simple and unspecified goiter	Inverse variance weighted	2	0.010945	0.108586	0.919712	1.011005	0.81719	1.250787	0.996292
digestive	Intestinal obstruction without mention of hernia	Inverse variance weighted	2	0.011411	0.113367	0.919825	1.011476	0.809945	1.263152	0.996292
musculoskeletal	Osteoarthrosis NOS	Inverse variance weighted	2	-0.00245	0.024925	0.921575	0.997549	0.949988	1.047492	0.996292
circulatory system	Abnormal heart sounds	Inverse variance weighted	2	0.008089	0.082395	0.921798	1.008121	0.857782	1.18481	0.996292
digestive	Peritoneal or intestinal adhesions	Inverse variance weighted	2	-0.009	0.092098	0.922141	0.991039	0.827362	1.187096	0.996292
digestive	Acute periodontitis	Inverse variance weighted	2	0.010974	0.114567	0.923691	1.011034	0.80769	1.265573	0.996292
neoplasms	Benign neoplasm of other parts of digestive system	Inverse variance weighted	2	0.004907	0.051598	0.92424	1.004919	0.908259	1.111865	0.996292
endocrine/metabolic	Type 2 diabetes with ophthalmic manifestations	Inverse variance weighted	2	0.011469	0.12391	0.926254	1.011535	0.793427	1.2896	0.996292
circulatory system	Arterial embolism and thrombosis of lower extremity artery	Inverse variance weighted	2	0.010184	0.113286	0.928366	1.010236	0.809081	1.261404	0.996292
digestive	Dental caries	Inverse variance weighted	2	-0.00423	0.048583	0.930581	0.995777	0.905331	1.095258	0.996292
digestive	Diseases of hard tissues of teeth	Inverse variance weighted	2	0.004171	0.048583	0.931591	1.004179	0.91297	1.1045	0.996292
injuries & poisonings	Intracranial hemorrhage (injury)	Inverse variance weighted	2	0.012929	0.152935	0.932626	1.013013	0.750644	1.367087	0.996292
genitourinary	Renal failure	Inverse variance weighted	2	0.002732	0.032957	0.933939	1.002736	0.940012	1.069645	0.996292
genitourinary	Inflammatory disease of cervix, vagina, and vulva	Inverse variance weighted	2	0.004125	0.049879	0.934097	1.004133	0.910611	1.10726	0.996292
genitourinary	Bladder neck obstruction	Inverse variance weighted	2	0.004943	0.060897	0.935301	1.004956	0.891888	1.132357	0.996292
neoplasms	Malignant neoplasm of kidney, except pelvis	Inverse variance weighted	2	-0.00664	0.08475	0.937526	0.993379	0.841346	1.172886	0.996292
circulatory system	Myocardial infarction	Inverse variance weighted	2	0.002014	0.02616	0.938627	1.002016	0.951934	1.054733	0.996292
mental disorders	Alcohol-related disorders	Inverse variance weighted	2	-0.00245	0.032437	0.939788	0.997553	0.936105	1.063034	0.996292
genitourinary	Renal colic	Inverse variance weighted	2	-0.00448	0.05973	0.940247	0.995533	0.885548	1.119177	0.996292
congenital anomalies	Genitourinary congenital anomalies	Inverse variance weighted	2	-0.00503	0.067388	0.940491	0.994982	0.871873	1.135474	0.996292
neurological	Migraine	Inverse variance weighted	2	-0.00378	0.051053	0.941021	0.99623	0.901368	1.101075	0.996292
sense organs	Epiphora	Inverse variance weighted	2	0.012719	0.173856	0.941679	1.0128	0.720336	1.424009	0.996292
endocrine/metabolic	Disorders of fluid, electrolyte, and acid-base balance	Inverse variance weighted	2	-0.00221	0.031163	0.943393	0.99779	0.938668	1.060635	0.996292
digestive	Cholelithiasis with other cholecystitis	Inverse variance weighted	2	-0.00256	0.036166	0.943574	0.997443	0.929187	1.070714	0.996292
respiratory	Other symptoms of respiratory system	Inverse variance weighted	2	0.001989	0.028662	0.944672	1.001991	0.947254	1.059891	0.996292
dermatologic	Prurigo and Lichen	Inverse variance weighted	2	0.006651	0.09593	0.944726	1.006673	0.834124	1.214916	0.996292
digestive	Anal and rectal polyp	Inverse variance weighted	2	0.002149	0.031163	0.945018	1.002151	0.942772	1.065271	0.996292
sense organs	Other disorders of tympanic membrane	Inverse variance weighted	2	0.004989	0.072391	0.94505	1.005002	0.872061	1.158209	0.996292
injuries & poisonings	Dislocation	Inverse variance weighted	2	-0.00461	0.070168	0.947667	0.995405	0.867505	1.142162	0.996292
musculoskeletal	Other acquired deformities of limbs	Inverse variance weighted	2	0.004301	0.065903	0.947963	1.00431	0.882612	1.142789	0.996292
sense organs	Disorders of lacrimal system	Inverse variance weighted	2	0.005167	0.079276	0.948035	1.00518	0.860524	1.174154	0.996292
endocrine/metabolic	Overweight, obesity and other hyperalimentation	Inverse variance weighted	2	-0.00294	0.045116	0.948096	0.997067	0.912685	1.089251	0.996292
injuries & poisonings	Superficial injury without mention of infection	Inverse variance weighted	2	0.002644	0.041109	0.948711	1.002648	0.925031	1.086778	0.996292
digestive	Gingival and periodontal diseases	Inverse variance weighted	2	-0.00408	0.063562	0.948819	0.995928	0.879271	1.128063	0.996292
circulatory system	Atherosclerosis	Inverse variance weighted	2	-0.00466	0.073568	0.949512	0.995353	0.861696	1.14974	0.996292
dermatologic	Hyperhidrosis	Inverse variance weighted	2	-0.0069	0.109764	0.94986	0.993121	0.800883	1.231503	0.996292
neoplasms	Breast cancer	Inverse variance weighted	2	-0.00156	0.024925	0.950246	0.998446	0.950842	1.048433	0.996292
infectious diseases	Viral warts & HPV	Inverse variance weighted	2	-0.0082	0.132981	0.950836	0.991834	0.764263	1.287168	0.996292
respiratory	Voice disturbance	Inverse variance weighted	2	-0.00477	0.080919	0.953033	0.995245	0.84928	1.166298	0.996292
genitourinary	Excessive or frequent menstruation	Inverse variance weighted	2	-0.00168	0.028662	0.953186	0.998319	0.943782	1.056007	0.996292
respiratory	Respiratory failure	Inverse variance weighted	2	0.004339	0.073932	0.953204	1.004348	0.868865	1.160957	0.996292
neoplasms	Cervical cancer and dysplasia	Inverse variance weighted	2	-0.00384	0.066984	0.954304	0.996169	0.873604	1.135929	0.996292
respiratory	Pulmonary collapse; interstitial and compensatory emphysema	Inverse variance weighted	2	-0.00821	0.143576	0.954409	0.991825	0.74855	1.314163	0.996292
digestive	Diseases of esophagus	Inverse variance weighted	2	0.000986	0.017769	0.955758	1.000986	0.966725	1.036461	0.996292
endocrine/metabolic	Obesity	Inverse variance weighted	2	0.002301	0.041543	0.955823	1.002304	0.923926	1.087331	0.996292
neoplasms	Acquired absence of breast	Inverse variance weighted	2	0.003015	0.054725	0.95606	1.00302	0.901004	1.116586	0.996292
musculoskeletal	Displacement of intervertebral disc	Inverse variance weighted	2	-0.0063	0.11829	0.957532	0.993721	0.788086	1.253012	0.996292
digestive	Anal and rectal conditions	Inverse variance weighted	2	-0.00151	0.028438	0.957642	0.998491	0.944359	1.055726	0.996292
musculoskeletal	Synovitis and tenosynovitis	Inverse variance weighted	2	0.004397	0.085745	0.959099	1.004407	0.849029	1.188221	0.996292
neoplasms	Cancer of kidney and renal pelvis	Inverse variance weighted	2	0.004023	0.082087	0.960908	1.004032	0.854817	1.179293	0.996292
genitourinary	Other disorders of male genital organs	Inverse variance weighted	2	-0.00595	0.125946	0.962324	0.994068	0.77662	1.2724	0.996292
infectious diseases	Staphylococcus infections	Inverse variance weighted	2	0.002676	0.058971	0.963809	1.002679	0.893234	1.125535	0.996292
genitourinary	Acute renal failure	Inverse variance weighted	2	0.001781	0.039904	0.964394	1.001783	0.926418	1.083279	0.996292
digestive	Ulceration of intestine	Inverse variance weighted	2	0.004566	0.103277	0.964736	1.004576	0.820488	1.229968	0.996292
respiratory	Respiratory insufficiency	Inverse variance weighted	2	-0.00328	0.075578	0.965353	0.996722	0.85949	1.155866	0.996292
symptoms	Back pain	Inverse variance weighted	2	-0.00109	0.02616	0.966625	0.998906	0.948979	1.051459	0.996292
neoplasms	Lipoma	Inverse variance weighted	2	0.003506	0.088911	0.968543	1.003512	0.843025	1.194552	0.996292
infectious diseases	Bacterial enteritis	Inverse variance weighted	2	-0.00199	0.051085	0.968937	0.998013	0.902925	1.103114	0.996292
respiratory	Chronic airway obstruction	Inverse variance weighted	2	-0.00231	0.060342	0.969507	0.997696	0.88641	1.122954	0.996292
musculoskeletal	Other inflammatory spondylopathies	Inverse variance weighted	2	0.004137	0.1088	0.96967	1.004145	0.811304	1.242823	0.996292
circulatory system	Other chronic ischemic heart disease, unspecified	Inverse variance weighted	2	-0.0009	0.023658	0.969689	0.999101	0.953831	1.04652	0.996292
endocrine/metabolic	Diabetic retinopathy	Inverse variance weighted	2	0.005045	0.136501	0.970518	1.005058	0.769129	1.313358	0.996292
injuries & poisonings	Fracture of neck of femur	Inverse variance weighted	2	-0.00199	0.063562	0.975018	0.998011	0.88111	1.130423	0.996292
neoplasms	Malignant neoplasm of uterus	Inverse variance weighted	2	0.003257	0.105355	0.975337	1.003262	0.816083	1.233374	0.996292
endocrine/metabolic	Hyperparathyroidism	Inverse variance weighted	2	-0.00287	0.09593	0.976145	0.997136	0.826222	1.203405	0.996292
circulatory system	Heart failure NOS	Inverse variance weighted	2	0.001214	0.041139	0.976462	1.001215	0.923653	1.08529	0.996292
sense organs	Blindness and low vision	Inverse variance weighted	2	0.002881	0.099759	0.976964	1.002885	0.824773	1.21946	0.996292
neoplasms	Secondary malignancy of brain/spine	Inverse variance weighted	2	-0.00271	0.094756	0.977206	0.997296	0.828259	1.200832	0.996292
neoplasms	Cancer of other female genital organs	Inverse variance weighted	2	-0.00131	0.053555	0.980494	0.998691	0.899175	1.109222	0.998344
genitourinary	Irregular menstrual cycle	Inverse variance weighted	2	-0.00131	0.05973	0.982487	0.99869	0.888356	1.122727	0.998648
respiratory	Allergic rhinitis	Inverse variance weighted	2	0.003366	0.168608	0.984072	1.003372	0.721008	1.396317	0.998648
digestive	Portal hypertension	Inverse variance weighted	2	0.001843	0.115944	0.987316	1.001845	0.798192	1.257458	0.998648
digestive	Esophagitis, GERD and related diseases	Inverse variance weighted	2	-0.00032	0.021618	0.98837	0.999685	0.958212	1.042953	0.998648
injuries & poisonings	Complication of internal orthopedic device	Inverse variance weighted	2	-0.00066	0.047378	0.988805	0.999335	0.910715	1.09658	0.998648
digestive	Diseases and other conditions of the tongue	Inverse variance weighted	2	-0.00088	0.075914	0.990782	0.999123	0.860993	1.159414	0.998648
digestive	Calculus of bile duct	Inverse variance weighted	2	0.000971	0.092354	0.991608	1.000972	0.835235	1.199596	0.998648
digestive	Diseases of pulp and periapical tissues	Inverse variance weighted	2	-0.00059	0.063562	0.992532	0.999405	0.882341	1.132002	0.998648
dermatologic	Dyschromia and Vitiligo	Inverse variance weighted	2	0.00074	0.08475	0.993035	1.00074	0.84758	1.181576	0.998648
injuries & poisonings	Adverse effects of sedatives or other central nervous system depressants and anesthetics	Inverse variance weighted	2	-0.00079	0.11094	0.994335	0.999213	0.80394	1.241916	0.998648
neoplasms	Benign neoplasm of ovary	Inverse variance weighted	2	-0.0004	0.069739	0.995396	0.999598	0.871891	1.14601	0.998648
respiratory	Acute pharyngitis	Inverse variance weighted	2	0.000567	0.115916	0.996097	1.000567	0.797217	1.255786	0.998648
dermatologic	Diffuse diseases of connective tissue	Inverse variance weighted	2	-0.0001	0.046081	0.998236	0.999898	0.913547	1.094412	0.999513
digestive	Disturbances in tooth eruption	Inverse variance weighted	2	6.83E-06	0.054725	0.9999	1.000007	0.898297	1.113232	0.9999

Table S17. The phenome-wide MR analysis results of HSD17B12 (IVW or Wald ratio methods).

Category	Phenostring	Method	Nsnp	beta	se	P value	OR	LL	LR	FDR
circulatory system	Essential hypertension	Wald ratio	1	-0.32771	0.069513	2.426E-06	0.720575	0.628794	0.825753	0.001896
circulatory system	Hypertension	Wald ratio	1	-0.31778	0.069513	4.844E-06	0.727766	0.635069	0.833994	0.001896
circulatory system	Cardiomegaly	Wald ratio	1	-1.19166	0.307845	0.0001084	0.303717	0.166122	0.55528	0.028293
endocrine/metabolic	Disorders of calcium/phosphorus metabolism	Wald ratio	1	-1.48957	0.446872	0.0008581	0.225469	0.093908	0.541342	0.167977
endocrine/metabolic	Type 2 diabetes	Wald ratio	1	-0.37736	0.119166	0.001542	0.68567	0.542849	0.866067	0.241472
injuries & poisonings	Fracture of unspecified bones	Wald ratio	1	1.290963	0.427011	0.0025007	3.636288	1.574632	8.397253	0.301991
endocrine/metabolic	Diabetes mellitus	Wald ratio	1	-0.3575	0.119166	0.0026998	0.699424	0.553739	0.883439	0.301991
musculoskeletal	Arthropathy NOS	Wald ratio	1	-0.25819	0.088381	0.0034852	0.772446	0.649586	0.918544	0.309925
mental disorders	Other specified nonpsychotic and/or transient mental disorders	Wald ratio	1	-1.78749	0.61569	0.0036934	0.16738	0.050075	0.559487	0.309925
injuries & poisonings	Internal derangement of knee	Wald ratio	1	-0.36743	0.129096	0.0044251	0.692513	0.537699	0.891902	0.309925
respiratory	Diseases of the larynx and vocal cords	Wald ratio	1	-0.84409	0.297915	0.0046065	0.429948	0.239787	0.770914	0.309925
sense organs	Macular degeneration (senile) of retina NOS	Wald ratio	1	-0.95333	0.337637	0.0047498	0.385457	0.198872	0.747098	0.309925
dermatologic	Pilonidal cyst	Wald ratio	1	1.787488	0.645482	0.0056189	5.974423	1.685974	21.17098	0.318648
sense organs	Degeneration of macula and posterior pole of retina	Wald ratio	1	-0.93347	0.337637	0.0056974	0.393189	0.202861	0.762084	0.318648
musculoskeletal	Other arthropathies	Wald ratio	1	-0.23833	0.087388	0.006386	0.787941	0.663907	0.935148	0.319473
sense organs	Other retinal disorders	Wald ratio	1	-0.67527	0.248262	0.0065282	0.509017	0.312901	0.828052	0.319473
genitourinary	Dyspareunia	Wald ratio	1	-1.19166	0.446872	0.0076608	0.303717	0.126498	0.729213	0.346979
sense organs	Open-angle glaucoma	Wald ratio	1	-1.29096	0.486594	0.0079765	0.275006	0.105961	0.713738	0.346979
genitourinary	Benign mammary dysplasias	Wald ratio	1	0.695134	0.268123	0.0095256	2.003978	1.184846	3.389408	0.392553
respiratory	Asthma	Wald ratio	1	-0.24826	0.099305	0.0124193	0.780155	0.642172	0.947788	0.486217
infectious diseases	Staphylococcus infections	Wald ratio	1	-0.6852	0.278054	0.0137287	0.503988	0.292237	0.869168	0.500005
sense organs	Primary open angle glaucoma	Wald ratio	1	-1.19166	0.486594	0.0143262	0.303717	0.117023	0.788254	0.500005
genitourinary	Endometriosis	Wald ratio	1	-0.60576	0.248262	0.0146873	0.54566	0.335426	0.887661	0.500005
musculoskeletal	Peripheral enthesopathies and allied syndromes	Wald ratio	1	-0.30785	0.129096	0.017097	0.735029	0.57071	0.946659	0.541579
digestive	Ulcer of esophagus	Wald ratio	1	-0.51639	0.218471	0.0180966	0.596673	0.38884	0.915594	0.541579
digestive	Intestinal obstruction without mention of hernia	Wald ratio	1	0.585899	0.248262	0.0182749	1.796605	1.104403	2.922655	0.541579
circulatory system	Ischemic Heart Disease	Wald ratio	1	-0.2284	0.097319	0.0189284	0.795805	0.657608	0.963044	0.541579
symptoms	Nausea and vomiting	Wald ratio	1	-0.34757	0.148957	0.0196307	0.706405	0.527544	0.945907	0.541579
hematopoietic	Diseases of spleen	Wald ratio	1	1.588878	0.685204	0.0204037	4.898249	1.278745	18.76281	0.541579
musculoskeletal	Enthesopathy	Wald ratio	1	-0.36743	0.158888	0.0207501	0.692513	0.507201	0.945532	0.541579
neurological	Disorders of other cranial nerves	Wald ratio	1	-0.95333	0.41708	0.022271	0.385457	0.170196	0.872974	0.556667
genitourinary	Ovarian cyst	Wald ratio	1	-0.51639	0.228401	0.0237673	0.596673	0.381345	0.933589	0.556667
musculoskeletal	Unspecified monoarthritis	Wald ratio	1	-0.28798	0.129096	0.0256964	0.749774	0.582158	0.965649	0.556667
sense organs	Retinal vascular changes and abnomalities	Wald ratio	1	-1.19166	0.536246	0.0262683	0.303717	0.106171	0.868823	0.556667
sense organs	Other disorders of middle ear and mastoid	Wald ratio	1	1.191658	0.536246	0.0262683	3.292537	1.150982	9.418738	0.556667
musculoskeletal	Contracture of palmar fascia [Dupuytren's disease]	Wald ratio	1	-0.59583	0.268123	0.0262683	0.551105	0.325839	0.932107	0.556667
circulatory system	Other chronic ischemic heart disease, unspecified	Wald ratio	1	-0.30785	0.139027	0.0268091	0.735029	0.559709	0.965265	0.556667
genitourinary	Other specified benign mammary dysplasias	Wald ratio	1	1.290963	0.585899	0.0275673	3.636288	1.15327	11.4653	0.556667
digestive	Abnormal results of function study of liver	Wald ratio	1	-0.5859	0.268123	0.0288753	0.556605	0.329091	0.941409	0.556667
infectious diseases	Bacterial infection NOS	Wald ratio	1	-0.29791	0.139027	0.0321246	0.742365	0.565295	0.974898	0.556667
digestive	Dental caries	Wald ratio	1	-0.59583	0.278054	0.0321246	0.551105	0.319559	0.950427	0.556667
endocrine/metabolic	Overweight, obesity and other hyperalimentation	Wald ratio	1	-0.31778	0.148957	0.0328974	0.727766	0.543497	0.974511	0.556667
genitourinary	Cyst or abscess of Bartholin's gland	Wald ratio	1	1.191658	0.566038	0.0352684	3.292537	1.085699	9.985084	0.556667
neoplasms	Lipoma	Wald ratio	1	0.41708	0.19861	0.0357288	1.517525	1.028196	2.23973	0.556667
circulatory system	Varicose veins of lower extremity, symptomtic	Wald ratio	1	-1.29096	0.61569	0.0360136	0.275006	0.082273	0.919238	0.556667
neoplasms	Lipoma of skin and subcutaneous tissue	Wald ratio	1	0.476663	0.228401	0.0368921	1.610691	1.029422	2.520178	0.556667
circulatory system	Angina pectoris	Wald ratio	1	-0.26812	0.129096	0.0378087	0.764814	0.593836	0.985019	0.556667
digestive	Diseases of hard tissues of teeth	Wald ratio	1	-0.57597	0.278054	0.0383188	0.56216	0.325969	0.969492	0.556667
sense organs	Blindness and low vision	Wald ratio	1	1.191658	0.575968	0.0385493	3.292537	1.064772	10.18133	0.556667
endocrine/metabolic	Disorders of lipoid metabolism	Wald ratio	1	-0.18868	0.09136	0.0389024	0.828052	0.692293	0.990433	0.556667
digestive	Hemorrhage from gastrointestinal ulcer	Wald ratio	1	1.290963	0.625621	0.0390659	3.636288	1.066889	12.39359	0.556667
digestive	Constipation	Wald ratio	1	-0.32771	0.158888	0.0391602	0.720575	0.527753	0.983846	0.556667
musculoskeletal	Other disorders of cervical region	Wald ratio	1	-1.39027	0.675273	0.0395111	0.249009	0.066284	0.935445	0.556667
dermatologic	Symptoms affecting skin	Wald ratio	1	-0.42701	0.20854	0.0405973	0.652456	0.433549	0.981894	0.556667
sense organs	Dizziness and giddiness (Light-headedness and vertigo)	Wald ratio	1	0.466733	0.228401	0.0410051	1.594775	1.019249	2.495276	0.556667
dermatologic	Rash and other nonspecific skin eruption	Wald ratio	1	-0.6852	0.337637	0.0424164	0.503988	0.260027	0.976837	0.556667
respiratory	Bronchiectasis	Wald ratio	1	-0.72493	0.357498	0.0425829	0.484361	0.240359	0.976061	0.556667
circulatory system	Nonspecific chest pain	Wald ratio	1	-0.18868	0.093347	0.0432511	0.828052	0.689603	0.994296	0.556667
circulatory system	Unstable angina (intermediate coronary syndrome)	Wald ratio	1	-0.43694	0.218471	0.0455003	0.646009	0.420991	0.991299	0.556667
circulatory system	Coronary atherosclerosis	Wald ratio	1	-0.23833	0.119166	0.0455003	0.787941	0.623818	0.995245	0.556667
digestive	GERD	Wald ratio	1	-0.25819	0.129096	0.0455003	0.772446	0.599763	0.994849	0.556667
digestive	Other intestinal obstruction	Wald ratio	1	0.536246	0.268123	0.0455003	1.709578	1.010783	2.891478	0.556667
musculoskeletal	Fasciitis	Wald ratio	1	-0.51639	0.258193	0.0455003	0.596673	0.359715	0.989725	0.556667
musculoskeletal	Disorders of muscle, ligament, and fascia	Wald ratio	1	-0.47666	0.238332	0.0455003	0.620851	0.389149	0.990512	0.556667
endocrine/metabolic	Type 2 diabetes with ophthalmic manifestations	Wald ratio	1	-0.84409	0.427011	0.0480706	0.429948	0.186182	0.992876	0.57441
infectious diseases	Streptococcus infection	Wald ratio	1	-0.74479	0.377358	0.0484177	0.474836	0.226636	0.994849	0.57441
neurological	Other paralytic syndromes	Wald ratio	1	-1.19166	0.60576	0.0491586	0.303717	0.092648	0.99564	0.574496
endocrine/metabolic	Hyperlipidemia	Wald ratio	1	-0.17875	0.09136	0.0504037	0.836316	0.699202	1.000318	0.580384
circulatory system	Abnormal heart sounds	Wald ratio	1	-0.9136	0.476663	0.0552803	0.401076	0.157573	1.02087	0.611811
digestive	Heartburn	Wald ratio	1	0.645482	0.337637	0.0559064	1.906905	0.983846	3.695993	0.611811
circulatory system	Hemorrhoids	Wald ratio	1	0.20854	0.109235	0.0562504	1.231878	0.994454	1.525987	0.611811
digestive	Chronic liver disease and cirrhosis	Wald ratio	1	-0.54618	0.287984	0.0578871	0.57916	0.329353	1.01844	0.611811
pregnancy complicati	Other complications of pregnancy NEC	Wald ratio	1	0.635551	0.337637	0.0597881	1.888062	0.974124	3.659472	0.611811
musculoskeletal	Joint effusions	Wald ratio	1	-0.76465	0.40715	0.0603745	0.465498	0.209578	1.033929	0.611811
neoplasms	Malignant neoplasm of kidney, except pelvis	Wald ratio	1	0.913605	0.486594	0.0604426	2.493294	0.960675	6.470987	0.611811
endocrine/metabolic	Obesity	Wald ratio	1	-0.29791	0.158888	0.0607927	0.742365	0.543713	1.013597	0.611811
genitourinary	Acute renal failure	Wald ratio	1	-0.42701	0.228401	0.0615442	0.652456	0.416997	1.02087	0.611811
musculoskeletal	Osteoarthrosis NOS	Wald ratio	1	-0.27805	0.148957	0.0619482	0.757256	0.56552	1.014	0.611811
dermatologic	Cellulitis and abscess of arm/hand	Wald ratio	1	-0.38729	0.20854	0.0632908	0.678895	0.451117	1.021682	0.611811
dermatologic	Cellulitis and abscess of leg, except foot	Wald ratio	1	-0.38729	0.20854	0.0632908	0.678895	0.451117	1.021682	0.611811
sense organs	Inflammation of the eye	Wald ratio	1	-0.51639	0.278054	0.0632908	0.596673	0.345981	1.029013	0.611811
digestive	Other chronic nonalcoholic liver disease	Wald ratio	1	-0.69513	0.377358	0.0654598	0.499008	0.238173	1.045493	0.622233
genitourinary	Polyp of corpus uteri	Wald ratio	1	0.327706	0.178749	0.066753	1.387781	0.977613	1.97004	0.622233
musculoskeletal	Spondylosis and allied disorders	Wald ratio	1	-0.32771	0.178749	0.066753	0.720575	0.507604	1.0229	0.622233
dermatologic	Cellulitis and abscess of foot, toe	Wald ratio	1	-0.37736	0.20854	0.0703697	0.68567	0.455619	1.031878	0.63589
digestive	Esophagitis, GERD and related diseases	Wald ratio	1	-0.16882	0.093347	0.0705271	0.844662	0.703437	1.014241	0.63589
genitourinary	Stricture/obstruction of ureter	Wald ratio	1	0.933466	0.516385	0.0706544	2.543308	0.924364	6.997696	0.63589
endocrine/metabolic	Hypercholesterolemia	Wald ratio	1	-0.16882	0.09434	0.0735386	0.844662	0.702069	1.016217	0.654326
endocrine/metabolic	Disorders of adrenal glands	Wald ratio	1	1.092354	0.61569	0.0760311	2.981282	0.891902	9.965272	0.668903
endocrine/metabolic	Disorders of mineral metabolism	Wald ratio	1	-0.59583	0.337637	0.0776132	0.551105	0.284337	1.068161	0.675235
sense organs	Cholesteatoma	Wald ratio	1	1.092354	0.625621	0.0808054	2.981282	0.87471	10.16113	0.695282
digestive	Diseases of the salivary glands	Wald ratio	1	-0.99305	0.575968	0.0846829	0.370446	0.119798	1.145509	0.712759
injuries & poisonings	Fracture of vertebral column without mention of spinal cord injury	Wald ratio	1	0.714995	0.41708	0.0864763	2.044177	0.902594	4.62961	0.712759
sense organs	Glaucoma	Wald ratio	1	-0.40715	0.238332	0.0875745	0.665544	0.417162	1.061815	0.712759
circulatory system	Paroxysmal ventricular tachycardia	Wald ratio	1	-0.86395	0.506455	0.08803	0.421493	0.156202	1.137348	0.712759
digestive	Cholelithiasis and cholecystitis	Wald ratio	1	-0.21847	0.129096	0.0905873	0.803747	0.624066	1.035162	0.712759
genitourinary	Hematuria	Wald ratio	1	-0.21847	0.129096	0.0905873	0.803747	0.624066	1.035162	0.712759
mental disorders	Symptoms involving head and neck	Wald ratio	1	-0.48659	0.287984	0.0910939	0.614717	0.349573	1.080966	0.712759
genitourinary	Pain and other symptoms associated with female genital organs	Wald ratio	1	-0.48659	0.287984	0.0910939	0.614717	0.349573	1.080966	0.712759
respiratory	Allergic rhinitis	Wald ratio	1	-0.80437	0.476663	0.0915072	0.44737	0.175761	1.138704	0.712759
neurological	Other conditions of brain	Wald ratio	1	-0.6852	0.40715	0.0923893	0.503988	0.226906	1.11942	0.712759
respiratory	Acute pharyngitis	Wald ratio	1	0.883813	0.526316	0.0931043	2.420111	0.862633	6.789602	0.712759
respiratory	Other upper respiratory disease	Wald ratio	1	-0.39722	0.238332	0.0955807	0.672186	0.421326	1.072412	0.712759
respiratory	Shortness of breath	Wald ratio	1	-0.34757	0.20854	0.0955807	0.706405	0.469397	1.063082	0.712759
digestive	Other disorders of liver	Wald ratio	1	-0.34757	0.20854	0.0955807	0.706405	0.469397	1.063082	0.712759
musculoskeletal	Osteoporosis	Wald ratio	1	0.327706	0.19861	0.0989429	1.387781	0.940288	2.048241	0.714996

circulatory system	Transient cerebral ischemia	Wald ratio	1	0.556107	0.337637	0.099546	1.743871	0.89973	3.379997	0.714996
hematopoietic	Other anemias	Wald ratio	1	0.228401	0.139027	0.1004125	1.256589	0.956867	1.650195	0.714996
mental disorders	Alcohol-related disorders	Wald ratio	1	-0.2284	0.139027	0.1004125	0.795805	0.605989	1.045078	0.714996
endocrine/metabolic	Secondary hypothyroidism	Wald ratio	1	-0.76465	0.466733	0.1013596	0.465498	0.186478	1.162007	0.714996
neurological	Other cerebral degenerations	Wald ratio	1	-0.76465	0.466733	0.1013596	0.465498	0.186478	1.162007	0.714996
respiratory	Voice disturbance	Wald ratio	1	-0.75472	0.466733	0.1058737	0.470144	0.188339	1.173604	0.735989
digestive	Inguinal hernia	Wald ratio	1	-0.20854	0.129096	0.1062274	0.811768	0.630294	1.045493	0.735989
digestive	Peritoneal or intestinal adhesions	Wald ratio	1	0.863952	0.536246	0.1071555	2.372519	0.829369	6.786905	0.735989
neoplasms	Cancer of kidney and renal pelvis	Wald ratio	1	0.764647	0.476663	0.1086773	2.148237	0.843992	5.467971	0.736498
sense organs	Vertiginous syndromes and other disorders of vestibular system	Wald ratio	1	0.317776	0.19861	0.1095986	1.374068	0.930997	2.028001	0.736498
musculoskeletal	Displacement of intervertebral disc	Wald ratio	1	1.092354	0.685204	0.1108906	2.981282	0.778298	11.41984	0.736498
sense organs	Inflammation of eyelids	Wald ratio	1	-0.50645	0.317776	0.110992	0.602628	0.323261	1.123429	0.736498
endocrine/metabolic	Diabetic retinopathy	Wald ratio	1	-0.67527	0.427011	0.1137877	0.509017	0.220421	1.17547	0.748704
neurological	Parkinson's disease	Wald ratio	1	-0.73486	0.466733	0.1153793	0.479575	0.192117	1.197146	0.751183
musculoskeletal	Osteoporosis NOS	Wald ratio	1	0.327706	0.20854	0.1160831	1.387781	0.922163	2.088497	0.751183
musculoskeletal	Other derangement of joint	Wald ratio	1	-0.53625	0.347567	0.1228654	0.58494	0.295976	1.156022	0.785385
respiratory	Postinflammatory pulmonary fibrosis	Wald ratio	1	-0.79444	0.516385	0.1239358	0.451835	0.164219	1.243185	0.785385
neoplasms	Carcinoma in situ of skin	Wald ratio	1	-0.9136	0.595829	0.1251937	0.401076	0.124752	1.289456	0.785385
sense organs	Disorders of refraction and accommodation; blindness and low vision	Wald ratio	1	0.486594	0.317776	0.1257076	1.626766	0.872628	3.032641	0.785385
neoplasms	Secondary malignancy of lymph nodes	Wald ratio	1	-0.31778	0.20854	0.1275563	0.727766	0.483592	1.095229	0.785385
neoplasms	Secondary malignant neoplasm of liver	Wald ratio	1	-0.46673	0.307845	0.1294867	0.627048	0.342971	1.146419	0.785385
dermatologic	Cellulitis and abscess of fingers/toes	Wald ratio	1	0.993049	0.655412	0.129734	2.699452	0.747098	9.753795	0.785385
genitourinary	Other nonmalignant breast conditions	Wald ratio	1	0.526316	0.347567	0.1299534	1.692685	0.856487	3.345269	0.785385
sense organs	Disorders of conjunctiva	Wald ratio	1	-0.85402	0.566038	0.1313571	0.425699	0.140372	1.290994	0.785385
neoplasms	Leukemia	Wald ratio	1	-0.56604	0.377358	0.1336144	0.567771	0.270994	1.189562	0.785385
digestive	Dysphagia	Wald ratio	1	0.297915	0.19861	0.1336144	1.347047	0.912689	1.98812	0.785385
neurological	Facial nerve disorders [CN7]	Wald ratio	1	-0.78451	0.526316	0.1360755	0.456344	0.162661	1.280269	0.785385
injuries & poisonings	Poisoning/allergy of sulfonamides	Wald ratio	1	-0.78451	0.526316	0.1360755	0.456344	0.162661	1.280269	0.785385
infectious diseases	Viral infection	Wald ratio	1	-0.42701	0.287984	0.1381386	0.652456	0.371035	1.14733	0.785385
circulatory system	Occlusion of cerebral arteries	Wald ratio	1	-0.36743	0.248262	0.1388732	0.692513	0.425699	1.126557	0.785385
respiratory	Bacterial pneumonia	Wald ratio	1	-0.27805	0.188679	0.1405666	0.757256	0.523162	1.096099	0.785385
musculoskeletal	Other inflammatory spondylopathies	Wald ratio	1	-0.55611	0.377358	0.1405666	0.573437	0.273698	1.201433	0.785385
respiratory	Other symptoms of respiratory system	Wald ratio	1	-0.24826	0.168818	0.1414025	0.780155	0.560377	1.086131	0.785385
digestive	Paralytic ileus	Wald ratio	1	0.993049	0.675273	0.1414025	2.699452	0.718574	10.14097	0.785385
infectious diseases	E. coli	Wald ratio	1	-0.43694	0.297915	0.1424668	0.646009	0.360287	1.158321	0.785385
symptoms	Other ill-defined and unknown causes of morbidity and mortality	Wald ratio	1	0.188679	0.129096	0.1438677	1.207654	0.937677	1.555362	0.785385
digestive	Diseases of esophagus	Wald ratio	1	-0.1291	0.088381	0.1441049	0.878889	0.739099	1.045119	0.785385
genitourinary	Noninflammatory disorders of vagina	Wald ratio	1	0.536246	0.367428	0.1444387	1.709578	0.832009	3.512771	0.785385
neoplasms	Lymphoid leukemia, chronic	Wald ratio	1	-0.99305	0.685204	0.1472607	0.370446	0.096709	1.418997	0.795208
neoplasms	Benign neoplasm of uterus	Wald ratio	1	-0.2284	0.158888	0.150576	0.795805	0.582852	1.086562	0.79698
mental disorders	Bipolar	Wald ratio	1	0.685204	0.476663	0.150576	1.984176	0.779536	5.050381	0.79698
sense organs	Retinal detachments and defects	Wald ratio	1	-0.39722	0.278054	0.1531275	0.672186	0.389768	1.159241	0.79698
digestive	Cholelithiasis	Wald ratio	1	-0.19861	0.139027	0.1531275	0.81987	0.624314	1.076681	0.79698
injuries & poisonings	Complication due to other implant and internal device	Wald ratio	1	0.397219	0.278054	0.1531275	1.487682	0.862633	2.565631	0.79698
sense organs	Disorders of lacrimal system	Wald ratio	1	-0.46673	0.327706	0.1543763	0.627048	0.329877	1.191927	0.79698
pregnancy complicati	Known or suspected fetal abnormality affecting management of mother	Wald ratio	1	0.367428	0.258193	0.1547139	1.444016	0.87055	2.395245	0.79698
injuries & poisonings	Poisoning by antibiotics	Wald ratio	1	-0.16882	0.119166	0.1565804	0.844662	0.668724	1.066889	0.801323
circulatory system	Abdominal aortic aneurysm	Wald ratio	1	-0.72493	0.516385	0.1603647	0.484361	0.176041	1.332677	0.811573
genitourinary	Other symptoms/disorders or the urinary system	Wald ratio	1	-0.13903	0.099305	0.1615133	0.870205	0.716294	1.057186	0.811573
digestive	Regional enteritis	Wald ratio	1	0.526316	0.377358	0.1630952	1.692685	0.807908	3.546419	0.811573
infectious diseases	Infection/inflammation of internal prosthetic device; implant; and graft	Wald ratio	1	-0.42701	0.307845	0.1654123	0.652456	0.356869	1.192874	0.811573
respiratory	Chronic sinusitis	Wald ratio	1	-0.42701	0.307845	0.1654123	0.652456	0.356869	1.192874	0.811573
respiratory	Hemoptysis	Wald ratio	1	0.466733	0.337637	0.1668634	1.594775	0.822806	3.091018	0.811573
respiratory	Pneumonia	Wald ratio	1	-0.21847	0.158888	0.1691314	0.803747	0.588669	1.097406	0.811573
genitourinary	Irregular menstrual cycle	Wald ratio	1	0.476663	0.347567	0.1702414	1.610691	0.814999	3.183225	0.811573
genitourinary	Other disorders of the kidney and ureters	Wald ratio	1	0.367428	0.268123	0.1705713	1.444016	0.85377	2.442322	0.811573
endocrine/metabolic	Crystal arthropathies	Wald ratio	1	-0.85402	0.625621	0.1722281	0.425699	0.1249	1.450915	0.811573
digestive	Celiac disease	Wald ratio	1	0.486594	0.357498	0.1734786	1.626766	0.807267	3.278182	0.811573
circulatory system	Palpitations	Wald ratio	1	-0.33764	0.248262	0.1738299	0.713455	0.438572	1.160623	0.811573
genitourinary	Urinary tract infection	Wald ratio	1	-0.18868	0.139027	0.1747358	0.828052	0.630544	1.087426	0.811573
mental disorders	Memory loss	Wald ratio	1	0.794439	0.585899	0.1751207	2.213199	0.701929	6.978266	0.811573
pregnancy complicati	Missed abortion/Hydatidiform mole	Wald ratio	1	-0.60576	0.446872	0.1752406	0.54566	0.227267	1.310108	0.811573
pregnancy complicati	Antepartum hemorrhage, abruptio placentae, and placenta previa	Wald ratio	1	-0.72493	0.536246	0.1764227	0.484361	0.169319	1.385578	0.811573
genitourinary	Other disorders of male genital organs	Wald ratio	1	0.387289	0.287984	0.1786809	1.472982	0.837646	2.590207	0.811573
digestive	Ulcerative colitis (chronic)	Wald ratio	1	0.893744	0.665343	0.1791802	2.444263	0.663433	9.005318	0.811573
neoplasms	Other non-epithelial cancer of skin	Wald ratio	1	-0.19861	0.148957	0.1824224	0.81987	0.61228	1.097842	0.811573
circulatory system	Myocardial infarction	Wald ratio	1	-0.19861	0.148957	0.1824224	0.81987	0.61228	1.097842	0.811573
endocrine/metabolic	Chondrocalcinosis	Wald ratio	1	-0.87388	0.655412	0.1824224	0.417328	0.115499	1.507911	0.811573
digestive	Chronic periodontitis	Wald ratio	1	-0.87388	0.655412	0.1824224	0.417328	0.115499	1.507911	0.811573
pregnancy complicati	Complications of labor and delivery NEC	Wald ratio	1	0.436941	0.327706	0.1824224	1.547965	0.814352	2.942458	0.811573
circulatory system	Other aneurysm	Wald ratio	1	-0.48659	0.367428	0.1853954	0.614717	0.299167	1.263095	0.812693
musculoskeletal	Spondylosis without myelopathy	Wald ratio	1	-0.28798	0.218471	0.1874428	0.749774	0.488612	1.150525	0.812693
mental disorders	Schizophrenia and other psychotic disorders	Wald ratio	1	-0.70506	0.536246	0.1885721	0.494077	0.172716	1.413372	0.812693
neoplasms	Uterine leiomyoma	Wald ratio	1	-0.20854	0.158888	0.1893515	0.811768	0.594544	1.108358	0.812693
respiratory	Other diseases of respiratory system, not elsewhere classified	Wald ratio	1	-0.20854	0.158888	0.1893515	0.811768	0.594544	1.108358	0.812693
endocrine/metabolic	Hypovolemia	Wald ratio	1	-0.37736	0.287984	0.1900792	0.68567	0.389922	1.205736	0.812693
sense organs	Epiphora	Wald ratio	1	-0.67527	0.516385	0.1909777	0.509017	0.185002	1.400518	0.812693
neoplasms	Lymphoid leukemia	Wald ratio	1	-0.84409	0.645482	0.1909777	0.429948	0.121331	1.523564	0.812693
respiratory	Abnormal sputum	Wald ratio	1	0.436941	0.337637	0.1956248	1.547965	0.798655	3.00029	0.8267
dermatologic	Actinic keratosis	Wald ratio	1	0.397219	0.307845	0.1969387	1.487682	0.813705	2.719902	0.8267
digestive	Periapical abscess	Wald ratio	1	-0.57597	0.446872	0.1974367	0.56216	0.23414	1.349725	0.8267
dermatologic	Superficial cellulitis and abscess	Wald ratio	1	-0.2284	0.178749	0.2013278	0.795805	0.560599	1.129694	0.838509
digestive	Diseases of pulp and periapical tissues	Wald ratio	1	-0.46673	0.367428	0.2039884	0.627048	0.305168	1.288432	0.841518
symptoms	Other symptoms	Wald ratio	1	0.566038	0.446872	0.2052745	1.761275	0.733571	4.228751	0.841518
symptoms	Back pain	Wald ratio	1	-0.18868	0.148957	0.2052745	0.828052	0.61839	1.108799	0.841518
circulatory system	Noninfectious disorders of lymphatic channels	Wald ratio	1	-0.73486	0.585899	0.2097558	0.479575	0.1521	1.512109	0.84412
hematopoietic	Other diseases of blood and blood-forming organs	Wald ratio	1	0.297915	0.238332	0.2112995	1.347047	0.844327	2.14909	0.84412
circulatory system	Cardiac pacemaker/device in situ	Wald ratio	1	-0.39722	0.317776	0.2112995	0.672186	0.360573	1.2531	0.84412
genitourinary	Vaginal enterocele, congenital or acquired	Wald ratio	1	-0.74479	0.595829	0.2112995	0.474836	0.147694	1.526593	0.84412
injuries & poisonings	Complication of internal orthopedic device	Wald ratio	1	-0.34757	0.278054	0.2112995	0.706405	0.409609	1.218253	0.84412
circulatory system	Atherosclerosis of the extremities	Wald ratio	1	-0.67527	0.546177	0.2163234	0.509017	0.174509	1.484731	0.858346
dermatologic	Unspecified diffuse connective tissue disease	Wald ratio	1	0.367428	0.297915	0.2174514	1.444016	0.805345	2.589178	0.858346
circulatory system	Congestive heart failure (CHF) NOS	Wald ratio	1	-0.42701	0.347567	0.2192325	0.652456	0.330139	1.289456	0.858346
neoplasms	Benign neoplasm of skin	Wald ratio	1	-0.21847	0.178749	0.2216236	0.803747	0.566194	1.140968	0.858346
musculoskeletal	Other disorders of synovium, tendon, and bursa	Wald ratio	1	0.218471	0.178749	0.2216236	1.244173	0.876449	1.766179	0.858346
endocrine/metabolic	Nontoxic nodular goiter	Wald ratio	1	-0.55611	0.456802	0.2234554	0.573437	0.234233	1.40386	0.858346
symptoms	Swelling of limb	Wald ratio	1	-0.27805	0.228401	0.2234554	0.757256	0.483976	1.184846	0.858346
circulatory system	Cardiac pacemaker in situ	Wald ratio	1	-0.39722	0.327706	0.225466	0.672186	0.353623	1.277729	0.858346
mental disorders	Swelling, mass, or lump in head and neck [Space-occupying lesion, intracranial NOS]	Wald ratio	1	-0.62562	0.516385	0.2256891	0.534929	0.19442	1.471812	0.858346
genitourinary	Retention of urine	Wald ratio	1	-0.2284	0.188679	0.226077	0.795805	0.549794	1.151897	0.858346
digestive	Inflammatory bowel disease and other gastroenteritis and colitis	Wald ratio	1	0.287984	0.238332	0.2269191	1.333736	0.835984	2.127855	0.858346
symptoms	Musculoskeletal symptoms referable to limbs	Wald ratio	1	-0.24826	0.20854	0.2338593	0.780155	0.518404	1.17407	0.872175
neoplasms	Benign neoplasm of unspecified sites	Wald ratio	1	-0.37736	0.317776	0.2350305	0.68567	0.367806	1.278237	0.872175
respiratory	Chronic airway obstruction	Wald ratio	1	-0.18868	0.158888	0.2350305	0.828052	0.60647	1.130591	0.872175
dermatologic	Cellulitis and abscess of trunk	Wald ratio	1	0.754717	0.635551	0.2350305	2.127009	0.612037	7.391992	0.872175
dermatologic	Ingrowing nail	Wald ratio	1	0.585899	0.496524	0.2380002	1.796605	0.678895	4.754476	0.879029
endocrine/metabolic	Disorders of parathyroid gland	Wald ratio	1	-0.61569	0.526316	0.2420769	0.540268	0.192575	1.515717	0.886229
digestive	Other specified gastritis	Wald ratio	1	-0.20854	0.178749	0.243345	0.811768	0.571845	1.152355	0.886229
musculoskeletal	Osteoporosis, osteopenia and pathological fracture	Wald ratio	1	0.20854	0.178749	0.243345	1.231878	0.867788	1.748726	0.886229

digestive	Chronic pancreatitis	Wald ratio	1	0.794439	0.685204	0.2462849	2.213199	0.577781	8.477689	0.886827
mental disorders	Anxiety disorders	Wald ratio	1	-0.21847	0.188679	0.246907	0.803747	0.55528	1.163393	0.886827
digestive	Flatulence	Wald ratio	1	0.436941	0.377358	0.246907	1.547965	0.738835	3.243212	0.886827
neurological	Nerve root and plexus disorders	Wald ratio	1	-0.52632	0.456802	0.2492496	0.590778	0.241316	1.446312	0.891153
respiratory	Pneumococcal pneumonia	Wald ratio	1	-0.23833	0.20854	0.2530979	0.787941	0.523577	1.185788	0.894173
infectious diseases	Sepsis	Wald ratio	1	-0.32771	0.287984	0.2551493	0.720575	0.409772	1.267115	0.894173
infectious diseases	Sepsis and SIRS	Wald ratio	1	-0.32771	0.287984	0.2551493	0.720575	0.409772	1.267115	0.894173
digestive	Hemorrhage of gastrointestinal tract	Wald ratio	1	-0.24826	0.218471	0.2558044	0.780155	0.508411	1.197146	0.894173
genitourinary	Hypertrophy of female genital organs	Wald ratio	1	-0.49652	0.436941	0.2558044	0.608642	0.258482	1.433159	0.894173
neoplasms	Breast cancer	Wald ratio	1	-0.16882	0.148957	0.2570743	0.844662	0.630795	1.131041	0.894619
genitourinary	Benign neoplasm of breast	Wald ratio	1	0.446872	0.397219	0.260589	1.563414	0.717718	3.405603	0.89886
neoplasms	Cancer of other female genital organs	Wald ratio	1	0.357498	0.317776	0.260589	1.429747	0.766943	2.665356	0.89886
sense organs	Infection of the eye	Wald ratio	1	0.734856	0.655412	0.2621976	2.085182	0.577093	7.534284	0.900442
injuries & poisonings	Fracture of hand or wrist	Wald ratio	1	-0.29791	0.268123	0.2665205	0.742365	0.438921	1.255591	0.909318
genitourinary	Renal failure	Wald ratio	1	-0.20854	0.188679	0.2690456	0.811768	0.560822	1.175003	0.909318
mental disorders	Anxiety disorder	Wald ratio	1	-0.21847	0.19861	0.2713321	0.803747	0.544577	1.186259	0.909318
neoplasms	Benign neoplasm of ovary	Wald ratio	1	-0.44687	0.40715	0.2723962	0.639626	0.287974	1.420689	0.909318
dermatologic	Disorder of skin and subcutaneous tissue NOS	Wald ratio	1	0.228401	0.20854	0.2734123	1.256589	0.834988	1.891065	0.909318
endocrine/metabolic	Simple and unspecified goiter	Wald ratio	1	-0.69513	0.635551	0.2740646	0.499008	0.143587	1.7342	0.909318
respiratory	Acute upper respiratory infections of multiple or unspecified sites	Wald ratio	1	0.347567	0.317776	0.2740646	1.415619	0.759365	2.639019	0.909318
neoplasms	Chemotherapy	Wald ratio	1	-0.11917	0.109235	0.2753129	0.887661	0.716579	1.099588	0.909318
circulatory system	Congestive heart failure; nonhypertensive	Wald ratio	1	-0.23833	0.218471	0.2753129	0.787941	0.513485	1.209093	0.909318
genitourinary	Disorders of menstruation and other abnormal bleeding from female genital tract	Wald ratio	1	0.129096	0.119166	0.2786605	1.1378	0.900803	1.437149	0.909318
musculoskeletal	Other and unspecified disc disorder	Wald ratio	1	0.258193	0.238332	0.2786605	1.294588	0.811446	2.065398	0.909318
neoplasms	Cancer of urinary organs (incl. kidney and bladder)	Wald ratio	1	0.268123	0.248262	0.2801422	1.307508	0.803747	2.127009	0.909318
respiratory	Chronic pharyngitis and nasopharyngitis	Wald ratio	1	0.536246	0.496524	0.2801422	1.709578	0.646009	4.524169	0.909318
musculoskeletal	Hallux rigidus	Wald ratio	1	0.427011	0.397219	0.2823747	1.532669	0.703604	3.338632	0.909318
symptoms	Malaise and fatigue	Wald ratio	1	0.287984	0.268123	0.2827895	1.333736	0.788568	2.255802	0.909318
digestive	Peritoneal adhesions (postoperative) (postinfection)	Wald ratio	1	-0.29791	0.278054	0.2839768	0.742365	0.430461	1.280269	0.909318
congenital anomalies	Digestive congenital anomalies	Wald ratio	1	-0.62562	0.585899	0.2856123	0.534929	0.169656	1.686644	0.909318
circulatory system	Cardiac arrest and ventricular fibrillation	Wald ratio	1	-0.48659	0.456802	0.2867776	0.614717	0.251094	1.504919	0.909318
respiratory	Epistaxis or throat hemorrhage	Wald ratio	1	0.337637	0.317776	0.2880088	1.401631	0.751861	2.612942	0.909318
genitourinary	Other disorders of bladder	Wald ratio	1	0.168818	0.158888	0.2880088	1.183905	0.867099	1.61646	0.909318
dermatologic	Cellulitis and abscess of face/neck	Wald ratio	1	-0.715	0.675273	0.2896802	0.489195	0.13022	1.837747	0.910922
sense organs	Disorders of external ear	Wald ratio	1	-0.42701	0.40715	0.2942792	0.652456	0.29375	1.449187	0.911523
injuries & poisonings	Fracture of radius and ulna	Wald ratio	1	-0.2284	0.218471	0.2958129	0.795805	0.51861	1.22116	0.911523
infectious diseases	Septicemia	Wald ratio	1	-0.25819	0.248262	0.2983399	0.772446	0.474836	1.256589	0.911523
neoplasms	Malignant neoplasm of testis	Wald ratio	1	0.258193	0.248262	0.2983399	1.294588	0.795805	2.105992	0.911523
mental disorders	Agorophobia, social phobia, and panic disorder	Wald ratio	1	0.60576	0.585899	0.3011837	1.832644	0.581234	5.778367	0.911523
respiratory	Obstructive chronic bronchitis	Wald ratio	1	0.307845	0.297915	0.3014479	1.36049	0.758762	2.439414	0.911523
digestive	Blood in stool	Wald ratio	1	0.317776	0.307845	0.3019512	1.374068	0.751563	2.512183	0.911523
neoplasms	Malignant neoplasm of ovary and other uterine adnexa	Wald ratio	1	0.347567	0.337637	0.3032862	1.415619	0.730373	2.743775	0.911523
musculoskeletal	Other and unspecified disorders of back	Wald ratio	1	0.347567	0.337637	0.3032862	1.415619	0.730373	2.743775	0.911523
dermatologic	Chronic ulcer of skin	Wald ratio	1	-0.3575	0.347567	0.3036811	0.699424	0.353904	1.382279	0.911523
injuries & poisonings	Torus fracture	Wald ratio	1	-0.38729	0.377358	0.3047428	0.678895	0.324032	1.422383	0.911523
circulatory system	Atherosclerosis	Wald ratio	1	-0.43694	0.427011	0.3061869	0.646009	0.279743	1.491825	0.911523
genitourinary	Cyst of kidney, acquired	Wald ratio	1	0.446872	0.436941	0.3064368	1.563414	0.66396	3.681341	0.911523
injuries & poisonings	Opiates and related narcotics causing adverse effects in therapeutic use	Wald ratio	1	0.466733	0.456802	0.3069044	1.594775	0.651421	3.904249	0.911523
neoplasms	Neoplasm of unspecified nature of digestive system	Wald ratio	1	0.486594	0.476663	0.3073334	1.626766	0.639118	4.140655	0.911523
neoplasms	Malignant neoplasm of female breast	Wald ratio	1	-0.14896	0.148957	0.3173105	0.861606	0.643448	1.153729	0.913434
mental disorders	Dementias	Wald ratio	1	-0.49652	0.496524	0.3173105	0.608642	0.229992	1.610691	0.913434
sense organs	Diplopia and disorders of binocular vision	Wald ratio	1	-0.57597	0.575968	0.3173105	0.56216	0.181797	1.738338	0.913434
genitourinary	Renal failure NOS	Wald ratio	1	0.41708	0.41708	0.3173105	1.517525	0.670054	3.436859	0.913434
pregnancy complicati	Placenta previa and abruptio placenta	Wald ratio	1	0.446872	0.446872	0.3173105	1.563414	0.651162	3.753695	0.913434
musculoskeletal	Ganglion and cyst of synovium, tendon, and bursa	Wald ratio	1	0.278054	0.278054	0.3173105	1.320557	0.765726	2.27741	0.913434
injuries & poisonings	Fracture of lower limb	Wald ratio	1	-0.18868	0.188679	0.3173105	0.828052	0.572072	1.198573	0.913434
injuries & poisonings	Effects radiation NOS	Wald ratio	1	-0.27805	0.278054	0.3173105	0.757256	0.439095	1.305951	0.913434
endocrine/metabolic	Nontoxic multinodular goiter	Wald ratio	1	-0.5859	0.595829	0.3254434	0.556605	0.173128	1.789483	0.926012
digestive	Periodontitis (acute or chronic)	Wald ratio	1	-0.43694	0.446872	0.3281842	0.646009	0.269063	1.551043	0.926012
injuries & poisonings	Poisoning by other anti-infectives	Wald ratio	1	-0.42701	0.436941	0.3284342	0.652456	0.277089	1.536327	0.926012
pregnancy complicati	Umbilical cord complications during labor and delivery	Wald ratio	1	-0.66534	0.685204	0.3315411	0.514097	0.134211	1.969257	0.926012
circulatory system	Nonrheumatic mitral valve disorders	Wald ratio	1	-0.27805	0.287984	0.3342858	0.757256	0.430632	1.331619	0.926012
musculoskeletal	Derangement of joint, non-traumatic	Wald ratio	1	-0.27805	0.287984	0.3342858	0.757256	0.430632	1.331619	0.926012
endocrine/metabolic	Vitamin B-complex deficiencies	Wald ratio	1	0.546177	0.566038	0.3345887	1.726639	0.569352	5.236277	0.926012
mental disorders	Alcoholic liver damage	Wald ratio	1	-0.52632	0.546177	0.3352283	0.590778	0.202539	1.723213	0.926012
circulatory system	Heart failure NOS	Wald ratio	1	-0.2284	0.238332	0.3378947	0.795805	0.498809	1.269634	0.926012
neurological	Epilepsy, recurrent seizures, convulsions	Wald ratio	1	-0.20854	0.218471	0.3398076	0.811768	0.529013	1.245656	0.926012
digestive	Diseases and other conditions of the tongue	Wald ratio	1	0.41708	0.436941	0.3398076	1.517525	0.644471	3.573286	0.926012
circulatory system	Hypotension	Wald ratio	1	-0.19861	0.20854	0.3409038	0.81987	0.544793	1.233837	0.926012
digestive	Disorders of esophageal motility	Wald ratio	1	-0.5859	0.61569	0.3412933	0.556605	0.166518	1.860516	0.926012
sense organs	Corneal opacity and other disorders of cornea	Wald ratio	1	-0.54618	0.575968	0.342989	0.57916	0.187294	1.790905	0.926012
mental disorders	Delirium dementia and amnestic and other cognitive disorders	Wald ratio	1	-0.32771	0.347567	0.345754	0.720575	0.364606	1.424079	0.926012
neoplasms	Malignant neoplasm of ovary	Wald ratio	1	0.317776	0.337637	0.3466144	1.374068	0.708935	2.663239	0.926012
mental disorders	Alcoholism	Wald ratio	1	-0.15889	0.168818	0.3466144	0.853092	0.612766	1.187673	0.926012
respiratory	Other diseases of respiratory system, NEC	Wald ratio	1	-0.15889	0.168818	0.3466144	0.853092	0.612766	1.187673	0.926012
injuries & poisonings	Fracture of tibia and fibula	Wald ratio	1	-0.31778	0.337637	0.3466144	0.727766	0.375483	1.410567	0.926012
genitourinary	Dysuria	Wald ratio	1	-0.41708	0.446872	0.3506479	0.658968	0.27446	1.582156	0.926012
musculoskeletal	Degeneration of intervertebral disc	Wald ratio	1	-0.26812	0.287984	0.3518357	0.764814	0.434929	1.344908	0.926012
neoplasms	Skin cancer	Wald ratio	1	-0.1291	0.139027	0.3531112	0.878889	0.669256	1.154187	0.926012
digestive	Other disorders of stomach and duodenum	Wald ratio	1	-0.24826	0.268123	0.3544845	0.780155	0.461265	1.319508	0.926012
digestive	Other disorders of peritoneum	Wald ratio	1	-0.24826	0.268123	0.3544845	0.780155	0.461265	1.319508	0.926012
symptoms	Chronic fatigue syndrome	Wald ratio	1	0.585899	0.635551	0.3565938	1.796605	0.516964	6.243737	0.926012
neoplasms	Benign neoplasm of colon	Wald ratio	1	0.109235	0.119166	0.3593173	1.115425	0.883089	1.408888	0.926012
musculoskeletal	Kyphoscoliosis and scoliosis	Wald ratio	1	-0.43694	0.476663	0.3593173	0.646009	0.253802	1.644307	0.926012
dermatologic	Other hypertrophic and atrophic conditions of skin	Wald ratio	1	-0.20854	0.228401	0.3612197	0.811768	0.518816	1.270139	0.926012
genitourinary	Prolapse of vaginal vault after hysterectomy	Wald ratio	1	0.625621	0.685204	0.3612197	1.869406	0.48803	7.160785	0.926012
dermatologic	Carbuncle and furuncle	Wald ratio	1	-0.29791	0.327706	0.3633021	0.742365	0.390543	1.411128	0.926012
mental disorders	Schizophrenia	Wald ratio	1	-0.5859	0.645482	0.3640408	0.556605	0.157073	1.972389	0.926012
injuries & poisonings	Adverse effects of sedatives or other central nervous system depressants and anesthetic	Wald ratio	1	-0.57597	0.635551	0.3648035	0.56216	0.161759	1.953675	0.926012
sense organs	Disorders of vitreous body	Wald ratio	1	-0.37736	0.41708	0.3655915	0.68567	0.302754	1.552892	0.926012
neoplasms	Cancer of larynx, pharynx, nasal cavities	Wald ratio	1	0.556107	0.61569	0.3664061	1.743871	0.521709	5.829085	0.926012
circulatory system	Hypertensive chronic kidney disease	Wald ratio	1	0.357498	0.397219	0.3681203	1.429747	0.656356	3.114435	0.926012
injuries & poisonings	Anaphylactic shock NOS	Wald ratio	1	-0.59583	0.665343	0.3705079	0.551105	0.149583	2.030419	0.926012
digestive	Abnormal findings on exam of gastrointestinal tract/ abdominal area	Wald ratio	1	0.337637	0.377358	0.3709278	1.401631	0.66899	2.93662	0.926012
neoplasms	Other benign neoplasm of connective and other soft tissue	Wald ratio	1	0.41708	0.466733	0.3715268	1.517525	0.607918	3.788147	0.926012
endocrine/metabolic	Hyperparathyroidism	Wald ratio	1	-0.49652	0.556107	0.3719337	0.608642	0.204642	1.810215	0.926012
circulatory system	Other specified cardiac dysrhythmias	Wald ratio	1	0.248262	0.278054	0.3719337	1.281796	0.74325	2.210563	0.926012
circulatory system	Other acute and subacute forms of ischemic heart disease	Wald ratio	1	-0.40715	0.456802	0.3727659	0.665544	0.271856	1.629353	0.926012
musculoskeletal	Curvature of spine	Wald ratio	1	-0.40715	0.456802	0.3727659	0.665544	0.271856	1.629353	0.926012
genitourinary	Irregular menstrual bleeding	Wald ratio	1	0.228401	0.258193	0.3763641	1.256589	0.757557	2.084354	0.926012
injuries & poisonings	Nonspecific abnormal findings on radiological and other examination of musculoske	Wald ratio	1	0.516385	0.585899	0.3781252	1.675959	0.53154	5.284335	0.926012
endocrine/metabolic	Hyperpotassemia	Wald ratio	1	0.436941	0.496524	0.3788593	1.547965	0.58494	4.096484	0.926012
sense organs	Other disorders of eye	Wald ratio	1	-0.21847	0.248262	0.3788593	0.803747	0.494077	1.307508	0.926012
digestive	Diseases of the jaws	Wald ratio	1	-0.43694	0.496524	0.3788593	0.646009	0.244112	1.709578	0.926012
injuries & poisonings	Crushing or internal injury to organs	Wald ratio	1	0.427011	0.486594	0.3801874	1.532669	0.590543	3.977824	0.926012
endocrine/metabolic	Protein-calorie malnutrition	Wald ratio	1	0.41708	0.476663	0.3815739	1.517525	0.5962	3.8626	0.926012
neoplasms	Secondary malignant neoplasm	Wald ratio	1	-0.13903	0.158888	0.3815739	0.870205	0.637343	1.188145	0.926012
genitourinary	Pelvic peritoneal adhesions, female (postoperative) (postinfection)	Wald ratio	1	-0.26812	0.307845	0.3837718	0.764814	0.418324	1.398294	0.926012
injuries & poisonings	Fracture of humerus	Wald ratio	1	-0.32771	0.377358	0.3851639	0.720575	0.343926	1.509708	0.926012

neoplasms	Breast cancer [female]	Wald ratio	1	-0.1291	0.148957	0.3861247	0.878889	0.656356	1.176872	0.926012
genitourinary	Menopausal and postmenopausal disorders	Wald ratio	1	0.129096	0.148957	0.3861247	1.1378	0.84971	1.523564	0.926012
respiratory	Chronic bronchitis	Wald ratio	1	0.248262	0.287984	0.3886496	1.281796	0.728923	2.25401	0.926012
dermatologic	Prurigo and Lichen	Wald ratio	1	0.476663	0.556107	0.3913659	1.610691	0.541557	4.790494	0.926012
endocrine/metabolic	Hypopotassemia	Wald ratio	1	-0.34757	0.40715	0.3932942	0.706405	0.318039	1.569013	0.926012
circulatory system	Cerebral artery occlusion, with cerebral infarction	Wald ratio	1	-0.34757	0.40715	0.3932942	0.706405	0.318039	1.569013	0.926012
genitourinary	Mucous polyp of cervix	Wald ratio	1	-0.2284	0.268123	0.3942963	0.795805	0.470517	1.345977	0.926012
dermatologic	Diffuse diseases of connective tissue	Wald ratio	1	0.228401	0.268123	0.3942963	1.256589	0.742955	2.12532	0.926012
genitourinary	Irregular menstrual cycle/bleeding	Wald ratio	1	0.109235	0.129096	0.3974669	1.115425	0.866067	1.436578	0.926012
digestive	Abdominal hernia	Wald ratio	1	-0.06455	0.076465	0.3985823	0.937491	0.80701	1.089068	0.926012
congenital anomalies	Cardiac shunt/ heart septal defect	Wald ratio	1	0.536246	0.635551	0.3988091	1.709578	0.491923	5.941291	0.926012
circulatory system	Arterial embolism and thrombosis	Wald ratio	1	-0.42701	0.506455	0.3991517	0.652456	0.241796	1.760575	0.926012
dermatologic	Sarcoidosis	Wald ratio	1	-0.55611	0.665343	0.4032557	0.573437	0.155645	2.112695	0.926012
digestive	Anal and rectal polyp	Wald ratio	1	-0.14896	0.178749	0.4046568	0.861606	0.606952	1.223102	0.926012
symptoms	Symptoms involving nervous and musculoskeletal systems	Wald ratio	1	-0.29791	0.357498	0.4046568	0.742365	0.368391	1.495979	0.926012
circulatory system	Aortic aneurysm	Wald ratio	1	-0.34757	0.41708	0.4046568	0.706405	0.311909	1.599851	0.926012
circulatory system	Mitral valve disease	Wald ratio	1	-0.23833	0.287984	0.4079049	0.787941	0.448081	1.385578	0.926012
musculoskeletal	Rheumatoid arthritis and other inflammatory polyarthropathies	Wald ratio	1	0.188679	0.228401	0.4087548	1.207654	0.771833	1.889563	0.926012
endocrine/metabolic	Vitamin deficiency	Wald ratio	1	0.367428	0.446872	0.4109504	1.444016	0.601433	3.467025	0.926012
sense organs	Labyrinthitis	Wald ratio	1	-0.4568	0.556107	0.4114022	0.633305	0.212934	1.883568	0.926012
digestive	Other symptoms involving abdomen and pelvis	Wald ratio	1	0.228401	0.278054	0.4114022	1.256589	0.728634	2.167092	0.926012
digestive	Malposition and malpresentation of fetus or obstruction	Wald ratio	1	0.317776	0.387289	0.4119238	1.374068	0.643193	2.935454	0.926012
circulatory system	Late effects of cerebrovascular disease	Wald ratio	1	-0.3575	0.436941	0.4132534	0.699424	0.297036	1.646921	0.926012
hematopoietic	Disorders of iron metabolism	Wald ratio	1	0.486594	0.595829	0.4141189	1.626766	0.505994	5.230041	0.926012
neoplasms	Cervical cancer	Wald ratio	1	0.307845	0.377358	0.4146206	1.36049	0.649354	2.850424	0.926012
circulatory system	Polyarteritis nodosa and allied conditions	Wald ratio	1	0.436941	0.536246	0.4151783	1.547965	0.541127	4.42816	0.926012
circulatory system	Disease of tricuspid valve	Wald ratio	1	-0.38729	0.476663	0.4165048	0.678895	0.266722	1.728011	0.926012
dermatologic	Hyperhidrosis	Wald ratio	1	-0.51639	0.635551	0.4165048	0.596673	0.17169	2.073618	0.926012
sense organs	Aphakia and other disorders of lens	Wald ratio	1	0.297915	0.367428	0.4174743	1.347047	0.655574	2.767857	0.926012
digestive	Calculus of bile duct	Wald ratio	1	0.248262	0.307845	0.4199825	1.281796	0.701093	2.343484	0.926719
neoplasms	Melanomas of skin, dx or hx	Wald ratio	1	0.238332	0.297915	0.4237108	1.26913	0.707809	2.275601	0.926719
neoplasms	Melanomas of skin	Wald ratio	1	0.238332	0.297915	0.4237108	1.26913	0.707809	2.275601	0.926719
hematopoietic	Lymphadenitis	Wald ratio	1	0.238332	0.297915	0.4237108	1.26913	0.707809	2.275601	0.926719
injuries & poisonings	Burns	Wald ratio	1	-0.51639	0.645482	0.4237108	0.596673	0.16838	2.114374	0.926719
dermatologic	Disorders of sweat glands	Wald ratio	1	-0.42701	0.536246	0.4258598	0.652456	0.228081	1.866438	0.926723
musculoskeletal	Rheumatism, unspecified and fibrositis	Wald ratio	1	0.387289	0.486594	0.4260795	1.472982	0.567545	3.822914	0.926723
sense organs	Ectropion or entropion	Wald ratio	1	0.377358	0.476663	0.4285551	1.458427	0.572982	3.712178	0.928654
respiratory	Bronchitis	Wald ratio	1	-0.48659	0.61569	0.4293394	0.614717	0.183903	2.05476	0.928654
hematopoietic	Coagulation defects	Wald ratio	1	-0.39722	0.506455	0.432856	0.672186	0.249107	1.813814	0.931926
neoplasms	Cancer of bladder	Wald ratio	1	0.248262	0.317776	0.4346555	1.281796	0.687579	2.389543	0.931926
symptoms	Sciatica	Wald ratio	1	0.248262	0.317776	0.4346555	1.281796	0.687579	2.389543	0.931926
dermatologic	Diseases of nail, NOS	Wald ratio	1	0.337637	0.436941	0.4396838	1.401631	0.595253	3.300394	0.931926
injuries & poisonings	Allergy/adverse effect of penicillin	Wald ratio	1	-0.0993	0.129096	0.4417563	0.905467	0.703046	1.166169	0.931926
circulatory system	Atrioventricular block, complete	Wald ratio	1	-0.49652	0.645482	0.4417563	0.608642	0.171758	2.156787	0.931926
neoplasms	Secondary malignancy of bone	Wald ratio	1	-0.25819	0.337637	0.4444467	0.772446	0.398535	1.497167	0.931926
sense organs	Otitis externa	Wald ratio	1	-0.51639	0.675273	0.4444467	0.596673	0.15883	2.24151	0.931926
circulatory system	Cardiac arrest	Wald ratio	1	-0.38729	0.506455	0.4444467	0.678895	0.251594	1.831916	0.931926
circulatory system	Cerebrovascular disease	Wald ratio	1	-0.1291	0.168818	0.4444467	0.878889	0.631296	1.223588	0.931926
injuries & poisonings	Fracture of upper limb	Wald ratio	1	-0.1291	0.168818	0.4444467	0.878889	0.631296	1.223588	0.931926
genitourinary	Other signs and symptoms in breast	Wald ratio	1	0.446872	0.585899	0.4456353	1.563414	0.495846	4.929479	0.931926
circulatory system	Phlebitis and thrombophlebitis	Wald ratio	1	-0.18868	0.248262	0.4472546	0.828052	0.509017	1.347047	0.931926
mental disorders	Tobacco use disorder	Wald ratio	1	-0.09037	0.119166	0.4482515	0.913595	0.723299	1.153958	0.931926
neoplasms	Secondary malignancy of respiratory organs	Wald ratio	1	0.248262	0.327706	0.448705	1.281796	0.674326	2.436508	0.931926
musculoskeletal	Other specified osteoporosis	Wald ratio	1	0.506455	0.675273	0.4532547	1.659398	0.441719	6.233825	0.933943
injuries & poisonings	Open wounds of extremities	Wald ratio	1	0.178749	0.238332	0.4532547	1.19572	0.749476	1.907663	0.933943
injuries & poisonings	Complications of surgical and medical procedures	Wald ratio	1	-0.11917	0.158888	0.4532547	0.887661	0.650128	1.211979	0.933943
circulatory system	Subarachnoid hemorrhage	Wald ratio	1	0.40715	0.546177	0.455997	1.502529	0.515119	4.382662	0.93507
circulatory system	Arterial embolism and thrombosis of lower extremity artery	Wald ratio	1	-0.48659	0.655412	0.4578303	0.614717	0.170128	2.221125	0.93507
genitourinary	Endometrial hyperplasia	Wald ratio	1	-0.33764	0.456802	0.4598278	0.713455	0.291426	1.746644	0.93507
respiratory	Pneumonitis due to inhalation of food or vomitus	Wald ratio	1	-0.47666	0.645482	0.460234	0.620851	0.175203	2.200051	0.93507
digestive	Acute gastritis	Wald ratio	1	-0.32771	0.446872	0.4633551	0.720575	0.300119	1.730072	0.93507
pregnancy complicati	Late pregnancy and failed induction	Wald ratio	1	0.327706	0.446872	0.4633551	1.387781	0.578011	3.332007	0.93507
neoplasms	Cancer within the respiratory system	Wald ratio	1	-0.21847	0.297915	0.4633551	0.803747	0.448259	1.441151	0.93507
circulatory system	Varicose veins of lower extremity	Wald ratio	1	-0.10924	0.148957	0.4633551	0.896519	0.669522	1.200479	0.93507
circulatory system	Endocarditis	Wald ratio	1	0.427011	0.585899	0.4661157	1.532669	0.486095	4.832541	0.936347
circulatory system	Cardiomyopathy	Wald ratio	1	-0.31778	0.436941	0.4670589	0.727766	0.309072	1.713657	0.936347
musculoskeletal	Osteoarthritis; localized	Wald ratio	1	0.086395	0.119166	0.468452	1.090237	0.863147	1.377073	0.936347
dermatologic	Disturbance of skin sensation	Wald ratio	1	-0.20854	0.287984	0.4689811	0.811768	0.461631	1.427477	0.936347
endocrine/metabolic	Thyrotoxicosis with or without goiter	Wald ratio	1	-0.25819	0.357498	0.4701579	0.772446	0.383319	1.556598	0.936347
digestive	Portal hypertension	Wald ratio	1	0.486594	0.675273	0.4711629	1.626766	0.433033	6.111236	0.936347
circulatory system	Circulatory disease NEC	Wald ratio	1	-0.09235	0.129096	0.4743714	0.911783	0.70795	1.174304	0.939304
musculoskeletal	Stiffness of joint	Wald ratio	1	0.446872	0.625621	0.4750505	1.563414	0.458707	5.328599	0.939304
digestive	Nonspecific abnormal findings in stool contents	Wald ratio	1	0.268123	0.377358	0.4773778	1.307508	0.624066	2.739419	0.939391
musculoskeletal	Rheumatoid arthritis	Wald ratio	1	0.168818	0.238332	0.4787383	1.183905	0.74207	1.888813	0.939391
infectious diseases	Candidiasis	Wald ratio	1	-0.23833	0.337637	0.4802613	0.787941	0.406529	1.5272	0.939391
neoplasms	Malignant neoplasm of bladder	Wald ratio	1	0.238332	0.337637	0.4802613	1.26913	0.654793	2.459847	0.939391
musculoskeletal	Malunion and nonunion of fracture	Wald ratio	1	-0.30785	0.436941	0.4810932	0.735029	0.312157	1.730759	0.939391
circulatory system	Intracerebral hemorrhage	Wald ratio	1	-0.40715	0.585899	0.4871084	0.665544	0.211081	2.098476	0.943703
injuries & poisonings	Adverse drug events and drug allergies	Wald ratio	1	0.40715	0.585899	0.4871084	1.502529	0.476536	4.737509	0.943703
neoplasms	Hemangioma and lymphangioma, any site	Wald ratio	1	-0.26812	0.387289	0.4887441	0.764814	0.358005	1.633889	0.943703
neoplasms	Secondary malignancy of brain/spine	Wald ratio	1	-0.37736	0.546177	0.4896227	0.68567	0.235072	2.000002	0.943703
circulatory system	Ill-defined descriptions and complications of heart disease	Wald ratio	1	-0.37736	0.546177	0.4896227	0.68567	0.235072	2.000002	0.943703
injuries & poisonings	Foreign body injury	Wald ratio	1	-0.30785	0.446872	0.4908932	0.735029	0.30614	1.764776	0.943703
genitourinary	Noninflammatory female genital disorders	Wald ratio	1	0.109235	0.158888	0.4917677	1.115425	0.816944	1.522959	0.943703
circulatory system	Cardiac conduction disorders	Wald ratio	1	-0.1291	0.188679	0.4938422	0.878889	0.607194	1.272158	0.943703
respiratory	Acute tonsillitis	Wald ratio	1	0.427011	0.625621	0.4948978	1.532669	0.449686	5.223812	0.943703
musculoskeletal	Acquired toe deformities	Wald ratio	1	0.148957	0.218471	0.4953539	1.160623	0.756354	1.780973	0.943703
digestive	Ulcerative colitis	Wald ratio	1	0.188679	0.278054	0.4974095	1.207654	0.700258	2.082698	0.945199
mental disorders	Other mental disorder	Wald ratio	1	-0.06653	0.098312	0.4985534	0.935631	0.771649	1.13446	0.945199
musculoskeletal	Polymyalgia Rheumatica	Wald ratio	1	-0.30785	0.456802	0.5003666	0.735029	0.300239	1.799462	0.946346
endocrine/metabolic	Hypoglycemia	Wald ratio	1	0.337637	0.506455	0.5049851	1.401631	0.519434	3.782132	0.948209
circulatory system	Pulmonary heart disease	Wald ratio	1	0.158888	0.238332	0.5049851	1.172206	0.734737	1.870149	0.948209
circulatory system	Primary/intrinsic cardiomyopathies	Wald ratio	1	-0.29791	0.446872	0.5049851	0.742365	0.309195	1.782389	0.948209
dermatologic	Erythematous conditions	Wald ratio	1	0.20854	0.317776	0.5116633	1.231878	0.660803	2.296486	0.957459
neurological	Migraine	Wald ratio	1	0.188679	0.287984	0.5123568	1.207654	0.686761	2.123633	0.957459
musculoskeletal	Other disorders of soft tissues	Wald ratio	1	-0.1291	0.19861	0.5156922	0.878889	0.59549	1.297162	0.961398
sense organs	Other disorders of tympanic membrane	Wald ratio	1	0.268123	0.41708	0.5203168	1.307508	0.577322	2.961218	0.966387
circulatory system	Peripheral vascular disease	Wald ratio	1	-0.15889	0.248262	0.5221726	0.853092	0.52441	1.387781	0.966387
digestive	Personal history of diseases of digestive system	Wald ratio	1	-0.08242	0.129096	0.5231733	0.920882	0.715015	1.186023	0.966387
neurological	Sleep disorders	Wald ratio	1	-0.13903	0.218471	0.5245394	0.870205	0.567094	1.335326	0.966387
dermatologic	Diseases of hair and hair follicles	Wald ratio	1	0.139027	0.218471	0.5245394	1.149155	0.748881	1.763375	0.966387
digestive	Ileostomy status	Wald ratio	1	-0.23833	0.377358	0.527662	0.787941	0.37608	1.650851	0.968557
digestive	Symptoms involving digestive system	Wald ratio	1	-0.08143	0.129096	0.5281914	0.921797	0.715725	1.187201	0.968557
musculoskeletal	Osteoarthrosis	Wald ratio	1	-0.06157	0.098312	0.5311427	0.940288	0.77549	1.140107	0.968682
digestive	Reflux esophagitis	Wald ratio	1	0.099305	0.158888	0.5319711	1.104403	0.808872	1.507911	0.968682
digestive	Other disorders of intestine	Wald ratio	1	0.148957	0.238332	0.5319711	1.160623	0.727477	1.851669	0.968682
mental disorders	Depression	Wald ratio	1	-0.09235	0.148957	0.5352578	0.911783	0.68092	1.220918	0.971174
digestive	Intestinal malabsorption (non-celiac)	Wald ratio	1	0.20854	0.337637	0.536808	1.231878	0.635574	2.387646	0.971174
circulatory system	Hypotension NOS	Wald ratio	1	-0.15889	0.258193	0.5383007	0.853092	0.514302	1.415057	0.971174

digestive	Umbilical hernia	Wald ratio	1	-0.15889	0.258193	0.5383007	0.853092	0.514302	1.415057	0.971174
endocrine/metabolic	Disorders of fluid, electrolyte, and acid-base balance	Wald ratio	1	-0.10924	0.178749	0.541126	0.896519	0.631547	1.272664	0.974027
dermatologic	Seborrheic keratosis	Wald ratio	1	-0.16882	0.278054	0.5437561	0.844662	0.489778	1.45669	0.976516
genitourinary	Symptoms involving female genital tract	Wald ratio	1	-0.11917	0.19861	0.5485062	0.887661	0.601433	1.310108	0.982793
digestive	Noninfectious gastroenteritis	Wald ratio	1	0.076465	0.129096	0.5536446	1.079464	0.838145	1.390264	0.985613
injuries & poisonings	Hemorrhage or hematoma complicating a procedure	Wald ratio	1	-0.1291	0.218471	0.5545813	0.878889	0.572754	1.348653	0.985613
neoplasms	Cancer of mouth	Wald ratio	1	0.357498	0.60576	0.5550807	1.429747	0.43614	4.686971	0.985613
digestive	Liver abscess and sequelae of chronic liver disease	Wald ratio	1	0.297915	0.506455	0.5563744	1.347047	0.499206	3.634843	0.985613
genitourinary	Excessive or frequent menstruation	Wald ratio	1	-0.0993	0.168818	0.5563744	0.905467	0.650386	1.260589	0.985613
circulatory system	Other disorders of circulatory system	Wald ratio	1	-0.07547	0.129096	0.5588064	0.927306	0.720003	1.194296	0.985996
neurological	Sleep apnea	Wald ratio	1	-0.13903	0.238332	0.5596689	0.870205	0.545443	1.388332	0.985996
neurological	Other peripheral nerve disorders	Wald ratio	1	-0.08044	0.139027	0.5628784	0.922713	0.702627	1.211738	0.985996
circulatory system	Phlebitis and thrombophlebitis of lower extremities	Wald ratio	1	-0.14896	0.258193	0.5639914	0.861606	0.519434	1.429179	0.985996
symptoms	Symptoms concerning nutrition, metabolism, and development	Wald ratio	1	0.119166	0.20854	0.5677092	1.126557	0.748583	1.695376	0.985996
sense organs	Other disorders of eyelids	Wald ratio	1	0.119166	0.20854	0.5677092	1.126557	0.748583	1.695376	0.985996
digestive	Other disorders of biliary tract	Wald ratio	1	0.278054	0.486594	0.5677092	1.320557	0.508815	3.427316	0.985996
neoplasms	Cancer of esophagus	Wald ratio	1	0.327706	0.575968	0.5693795	1.387781	0.448794	4.29136	0.985996
endocrine/metabolic	Other disorders of pancreatic internal secretion	Wald ratio	1	0.287984	0.506455	0.569609	1.333736	0.494273	3.598926	0.985996
circulatory system	Other forms of chronic heart disease	Wald ratio	1	-0.20854	0.367428	0.5703286	0.811768	0.395067	1.667989	0.985996
musculoskeletal	Other unspecified back disorders	Wald ratio	1	0.218471	0.387289	0.5726843	1.244173	0.58239	2.657955	0.985996
circulatory system	Paroxysmal supraventricular tachycardia	Wald ratio	1	0.178749	0.317776	0.5737754	1.19572	0.641407	2.22908	0.985996
pregnancy complicati	Hemorrhage in early pregnancy	Wald ratio	1	-0.26812	0.476663	0.5737754	0.764814	0.300477	1.946703	0.985996
genitourinary	Other disorders of urethra and urinary tract	Wald ratio	1	-0.13903	0.248262	0.5754794	0.870205	0.534929	1.415619	0.985996
genitourinary	Polyp of female genital organs	Wald ratio	1	0.083416	0.148957	0.5754794	1.086994	0.811768	1.455533	0.985996
genitourinary	Pyelonephritis	Wald ratio	1	-0.23833	0.427011	0.5767491	0.787941	0.341205	1.819587	0.986014
sense organs	Subjective visual disturbances	Wald ratio	1	-0.34757	0.625621	0.5785147	0.706405	0.20726	2.407646	0.986077
injuries & poisonings	Fracture of pelvis	Wald ratio	1	0.317776	0.575968	0.5811374	1.374068	0.444359	4.248956	0.986077
injuries & poisonings	Contusion	Wald ratio	1	0.218471	0.397219	0.5823194	1.244173	0.571164	2.710196	0.986077
neoplasms	Benign neoplasm of other parts of digestive system	Wald ratio	1	0.119166	0.218471	0.5854409	1.126557	0.734154	1.728698	0.986077
sense organs	Hearing loss	Wald ratio	1	0.129096	0.238332	0.5880482	1.1378	0.713171	1.815256	0.986077
digestive	Gingival and periodontal diseases	Wald ratio	1	-0.19861	0.367428	0.5888243	0.81987	0.39901	1.684635	0.986077
neoplasms	Cervical cancer and dysplasia	Wald ratio	1	0.139027	0.258193	0.5902585	1.149155	0.692788	1.906148	0.986077
musculoskeletal	Symptoms and disorders of the joints	Wald ratio	1	-0.13903	0.258193	0.5902585	0.870205	0.524618	1.443442	0.986077
genitourinary	Calculus of ureter	Wald ratio	1	-0.16882	0.317776	0.5952455	0.844662	0.453093	1.574632	0.986077
digestive	Esophageal bleeding (varices/hemorrhage)	Wald ratio	1	-0.19861	0.377358	0.5986688	0.81987	0.391319	1.717746	0.986077
endocrine/metabolic	Other disorders of metabolism	Wald ratio	1	0.218471	0.41708	0.600411	1.244173	0.549357	2.817777	0.986077
digestive	Cholelithiasis with other cholecystitis	Wald ratio	1	-0.10924	0.20854	0.600411	0.896519	0.595726	1.349189	0.986077
neoplasms	Malignant neoplasm of uterus	Wald ratio	1	0.228401	0.436941	0.6011641	1.256589	0.533656	2.958867	0.986077
sense organs	Myopia	Wald ratio	1	0.228401	0.436941	0.6011641	1.256589	0.533656	2.958867	0.986077
circulatory system	Rheumatic disease of the heart valves	Wald ratio	1	-0.11917	0.228401	0.601852	0.887661	0.56732	1.388884	0.986077
genitourinary	Calculus of lower urinary tract	Wald ratio	1	0.287984	0.556107	0.6045579	1.333736	0.448437	3.966779	0.986077
genitourinary	Postmenopausal bleeding	Wald ratio	1	0.087388	0.168818	0.6047045	1.09132	0.783883	1.519334	0.986077
pregnancy complicati	Problems associated with amniotic cavity and membranes	Wald ratio	1	-0.21847	0.427011	0.6089115	0.803747	0.348049	1.856087	0.986077
genitourinary	Cystic mastopathy	Wald ratio	1	0.258193	0.506455	0.6101888	1.294588	0.479765	3.49329	0.986077
sense organs	Meniere's disease	Wald ratio	1	0.327706	0.645482	0.6116691	1.387781	0.39163	4.917745	0.986077
infectious diseases	Postoperative infection	Wald ratio	1	-0.11917	0.238332	0.6170751	0.887661	0.556384	1.416182	0.986077
neoplasms	Cancer of bronchus; lung	Wald ratio	1	-0.16882	0.337637	0.6170751	0.844662	0.435794	1.637138	0.986077
neoplasms	Myeloproliferative disease	Wald ratio	1	0.248262	0.496524	0.6170751	1.281796	0.484361	3.392102	0.986077
endocrine/metabolic	Gout and other crystal arthropathies	Wald ratio	1	-0.1291	0.258193	0.6170751	0.878889	0.529854	1.457848	0.986077
circulatory system	Hypertensive heart and/or renal disease	Wald ratio	1	0.188679	0.377358	0.6170751	1.207654	0.576406	2.530209	0.986077
respiratory	Septal Deviations/Turbinate Hypertrophy	Wald ratio	1	-0.10924	0.218471	0.6170751	0.896519	0.584243	1.375706	0.986077
digestive	Disturbances in tooth eruption	Wald ratio	1	0.158888	0.317776	0.6170751	1.172206	0.628794	2.185245	0.986077
digestive	Stricture and stenosis of esophagus	Wald ratio	1	0.178749	0.357498	0.6170751	1.19572	0.593365	2.409559	0.986077
digestive	Cholecystitis without cholelithiasis	Wald ratio	1	-0.14896	0.297915	0.6170751	0.861606	0.480528	1.544894	0.986077
symptoms	Pain in limb	Wald ratio	1	0.099305	0.19861	0.6170751	1.104403	0.748286	1.63	0.986077
symptoms	Gangrene	Wald ratio	1	0.327706	0.665343	0.6223396	1.387781	0.376678	5.112955	0.986077
circulatory system	Aneurysm and dissection of heart	Wald ratio	1	-0.28798	0.585899	0.6230549	0.749774	0.237795	2.364053	0.986077
symptoms	Abdominal pain	Wald ratio	1	-0.03873	0.079444	0.625904	0.962011	0.823296	1.124098	0.986077
sense organs	Otitis media	Wald ratio	1	0.178749	0.367428	0.6266223	1.19572	0.581927	2.456918	0.986077
pregnancy complicati	Normal delivery	Wald ratio	1	0.178749	0.367428	0.6266223	1.19572	0.581927	2.456918	0.986077
digestive	Acute appendicitis	Wald ratio	1	-0.14896	0.307845	0.6284774	0.861606	0.471265	1.575258	0.986077
digestive	Duodenal ulcer	Wald ratio	1	0.139027	0.287984	0.6292671	1.149155	0.653494	2.020764	0.986077
genitourinary	Inflammatory disease of breast	Wald ratio	1	0.278054	0.575968	0.6292671	1.320557	0.427054	4.083487	0.986077
injuries & poisonings	Other open wound of head and face	Wald ratio	1	0.139027	0.287984	0.6292671	1.149155	0.653494	2.020764	0.986077
circulatory system	Cardiac dysrhythmias	Wald ratio	1	-0.05263	0.109235	0.6299351	0.948729	0.765878	1.175237	0.986077
respiratory	Other dyspnea	Wald ratio	1	-0.24826	0.516385	0.6306805	0.780155	0.283547	2.146531	0.986077
genitourinary	Chronic cystitis	Wald ratio	1	-0.24826	0.516385	0.6306805	0.780155	0.283547	2.146531	0.986077
injuries & poisonings	Poisoning by analgesics, antipyretics, and antirheumatics	Wald ratio	1	-0.09037	0.188679	0.6319761	0.913595	0.631171	1.322394	0.986077
injuries & poisonings	Intracranial hemorrhage (injury)	Wald ratio	1	0.327706	0.685204	0.6324645	1.387781	0.362296	5.315915	0.986077
mental disorders	Delirium due to conditions classified elsewhere	Wald ratio	1	-0.28798	0.60576	0.6344949	0.749774	0.228716	2.457894	0.986077
symptoms	Symptoms of the muscles	Wald ratio	1	-0.27805	0.585899	0.635089	0.757256	0.240168	2.387646	0.986077
circulatory system	Varicose veins	Wald ratio	1	-0.07051	0.148957	0.6359754	0.931922	0.69596	1.247885	0.986077
neoplasms	Acquired absence of breast	Wald ratio	1	-0.14896	0.317776	0.6392483	0.861606	0.462182	1.606219	0.987794
respiratory	Chronic tonsillitis and adenoiditis	Wald ratio	1	0.218471	0.466733	0.6397237	1.244173	0.498413	3.105787	0.987794
genitourinary	Noninflammatory disorders of ovary, fallopian tube, and broad ligament	Wald ratio	1	0.268123	0.575968	0.6415611	1.307508	0.422834	4.043137	0.987794
circulatory system	Heart valve replaced	Wald ratio	1	-0.18868	0.40715	0.6430672	0.828052	0.372807	1.839207	0.987794
musculoskeletal	Osteopenia or other disorder of bone and cartilage	Wald ratio	1	-0.24826	0.536246	0.6433909	0.780155	0.272721	2.231738	0.987794
dermatologic	Dyschromia and Vitiligo	Wald ratio	1	-0.2284	0.496524	0.6455162	0.795805	0.300716	2.105992	0.988871
mental disorders	Psychogenic and somatoform disorders	Wald ratio	1	-0.30785	0.675273	0.6484746	0.735029	0.195659	2.761268	0.988871
musculoskeletal	Bursitis	Wald ratio	1	0.238332	0.526316	0.650671	1.26913	0.452373	3.560534	0.988871
symptoms	Other tests	Wald ratio	1	0.089374	0.19861	0.6527104	1.09349	0.740892	1.613893	0.988871
digestive	Duodenitis	Wald ratio	1	0.080437	0.178749	0.6527104	1.083761	0.763448	1.538464	0.988871
injuries & poisonings	Fracture of ribs	Wald ratio	1	-0.24826	0.556107	0.6552877	0.780155	0.262309	2.320327	0.988871
infectious diseases	Gram negative septicemia	Wald ratio	1	-0.23833	0.536246	0.6567213	0.787941	0.275443	2.25401	0.988871
respiratory	Empyema and pneumothorax	Wald ratio	1	-0.19861	0.446872	0.6567213	0.81987	0.341476	1.968475	0.988871
genitourinary	Other abnormality of urination	Wald ratio	1	0.158888	0.357498	0.6567213	1.172206	0.581696	2.362175	0.988871
genitourinary	Disorders of uterus, NEC	Wald ratio	1	0.119166	0.268123	0.6567213	1.126557	0.666073	1.905391	0.988871
digestive	Gastric ulcer	Wald ratio	1	-0.10924	0.248262	0.6599371	0.896519	0.551105	1.458427	0.991806
neoplasms	Malignant neoplasm, other	Wald ratio	1	-0.05561	0.129096	0.6666362	0.945907	0.734445	1.218253	0.995249
sense organs	Senile cataract	Wald ratio	1	0.076465	0.178749	0.6688129	1.079464	0.760421	1.532365	0.995249
circulatory system	Abnormal function study of cardiovascular system	Wald ratio	1	-0.28798	0.675273	0.669765	0.749774	0.199584	2.816658	0.995249
musculoskeletal	Acquired spondylolisthesis	Wald ratio	1	-0.16882	0.397219	0.6708367	0.844662	0.38776	1.839938	0.995249
neurological	Abnormal involuntary movements	Wald ratio	1	-0.21847	0.516385	0.6722391	0.803747	0.292121	2.211441	0.995249
dermatologic	Sebaceous cyst	Wald ratio	1	-0.07051	0.168818	0.6762052	0.931922	0.669389	1.29742	0.995249
dermatologic	Diseases of sebaceous glands	Wald ratio	1	-0.07051	0.168818	0.6762052	0.931922	0.669389	1.29742	0.995249
neoplasms	Cancer, suspected or other	Wald ratio	1	-0.04965	0.119166	0.6769222	0.95156	0.753356	1.201911	0.995249
injuries & poisonings	Superficial injury without mention of infection	Wald ratio	1	-0.0993	0.238332	0.6769222	0.905467	0.567545	1.44459	0.995249
infectious diseases	Viral Enteritis	Wald ratio	1	-0.21847	0.526316	0.6780728	0.803747	0.286491	2.254906	0.995249
circulatory system	Elevated blood pressure reading without diagnosis of hypertension	Wald ratio	1	0.168818	0.40715	0.6784097	1.183905	0.53302	2.629601	0.995249
congenital anomalies	Congenital anomalies of genital organs	Wald ratio	1	-0.23833	0.575968	0.6790256	0.787941	0.254812	2.436508	0.995249
symptoms	Cervicalgia	Wald ratio	1	0.148957	0.367428	0.6851795	1.160623	0.564846	2.384802	0.995249
circulatory system	Occlusion and stenosis of precerebral arteries	Wald ratio	1	-0.17875	0.446872	0.6891565	0.836316	0.348326	2.007962	0.995249
genitourinary	Renal colic	Wald ratio	1	-0.13903	0.347567	0.6891565	0.870205	0.440318	1.719794	0.995249
genitourinary	Urinary incontinence	Wald ratio	1	-0.06753	0.168818	0.6891565	0.934702	0.671386	1.301291	0.995249
pregnancy complicati	Fetal distress and abnormal forces of labor	Wald ratio	1	0.139027	0.347567	0.6891565	1.149155	0.581465	2.271086	0.995249
musculoskeletal	Synovitis and tenosynovitis	Wald ratio	1	0.119166	0.297915	0.6891565	1.126557	0.628294	2.019961	0.995249
sense organs	Suppurative and unspecified otitis media	Wald ratio	1	-0.20854	0.526316	0.691938	0.811768	0.28935	2.27741	0.995249
digestive	Other biliary tract disease	Wald ratio	1	-0.09831	0.248262	0.692105	0.906366	0.557158	1.474446	0.995249
symptoms	Syncope and collapse	Wald ratio	1	0.066534	0.168818	0.6934942	1.068798	0.767705	1.487978	0.995249

sense organs	Otitis media and Eustachian tube disorders	Wald ratio	1	0.129096	0.327706	0.6936258	1.1378	0.598573	2.162792	0.995249
musculoskeletal	Osteomyelitis	Wald ratio	1	-0.25819	0.655412	0.6936258	0.772446	0.213782	2.791042	0.995249
symptoms	Myalgia and myositis unspecified	Wald ratio	1	-0.23833	0.60576	0.6939926	0.787941	0.240359	2.583015	0.995249
circulatory system	Paroxysmal tachycardia, unspecified	Wald ratio	1	-0.10924	0.278054	0.694425	0.896519	0.519847	1.546122	0.995249
endocrine/metabolic	Anorexia	Wald ratio	1	0.20854	0.536246	0.6973583	1.231878	0.430632	3.523951	0.995249
genitourinary	Abnormal findings on examination of urine	Wald ratio	1	-0.0993	0.258193	0.7005224	0.905467	0.545877	1.501933	0.995249
musculoskeletal	Osteoarthrosis, localized, primary	Wald ratio	1	0.064548	0.168818	0.7021996	1.066677	0.766182	1.485026	0.995249
digestive	Diaphragmatic hernia	Wald ratio	1	-0.03774	0.099305	0.7039454	0.962967	0.79265	1.169881	0.995249
circulatory system	Atrial fibrillation and flutter	Wald ratio	1	-0.05263	0.139027	0.7050061	0.948729	0.722438	1.245904	0.995249
neoplasms	Vascular hamartomas and non-neoplastic nevi	Wald ratio	1	-0.2284	0.60576	0.7061371	0.795805	0.242758	2.608793	0.995249
pregnancy complicati	Hypertension complicating pregnancy, childbirth, and the puerperium	Wald ratio	1	0.178749	0.476663	0.7076605	1.19572	0.46977	3.043502	0.995249
respiratory	Acute and chronic tonsillitis	Wald ratio	1	0.129096	0.347567	0.7103183	1.1378	0.575719	2.248645	0.995249
neoplasms	Benign neoplasm of brain and other parts of nervous system	Wald ratio	1	0.19861	0.536246	0.7111065	1.219706	0.426376	3.48913	0.995249
genitourinary	Urethral stricture (not specified as infectious)	Wald ratio	1	-0.0993	0.268123	0.7111065	0.905467	0.535354	1.531452	0.995249
digestive	Peritonitis and retroperitoneal infections	Wald ratio	1	0.188679	0.516385	0.7148243	1.207654	0.438921	3.322756	0.995249
neoplasms	Multiple myeloma	Wald ratio	1	-0.23833	0.655412	0.7161296	0.787941	0.21807	2.847029	0.995249
pregnancy complicati	Early onset of delivery	Wald ratio	1	0.228401	0.635551	0.7193146	1.256589	0.361577	4.367023	0.995249
neoplasms	Benign neoplasm of brain, cranial nerves, meninges	Wald ratio	1	0.19861	0.556107	0.7209849	1.219706	0.410097	3.627631	0.995249
respiratory	Nasal polyps	Wald ratio	1	-0.0993	0.278054	0.7209849	0.905467	0.525035	1.561552	0.995249
injuries & poisonings	Fracture of patella	Wald ratio	1	-0.19861	0.556107	0.7209849	0.81987	0.275662	2.438445	0.995249
injuries & poisonings	Complication of colostomy or enterostomy	Wald ratio	1	-0.24826	0.695134	0.7209849	0.780155	0.199743	3.047131	0.995249
genitourinary	Chronic renal failure [CKD]	Wald ratio	1	-0.10924	0.307845	0.7227104	0.896519	0.490362	1.63909	0.995249
neoplasms	Malignant neoplasm of rectum, rectosigmoid junction, and anus	Wald ratio	1	0.119166	0.337637	0.7241325	1.126557	0.581234	2.18351	0.995249
circulatory system	Left bundle branch block	Wald ratio	1	0.129096	0.367428	0.7253248	1.1378	0.553739	2.337905	0.995249
endocrine/metabolic	Hypothyroidism NOS	Wald ratio	1	0.048659	0.139027	0.7263387	1.049863	0.799448	1.378715	0.995249
endocrine/metabolic	Electrolyte imbalance	Wald ratio	1	0.086395	0.248262	0.7278402	1.090237	0.670187	1.77356	0.995249
digestive	Gastritis and duodenitis	Wald ratio	1	-0.03376	0.097319	0.7286373	0.9668	0.798909	1.169974	0.995249
neoplasms	Pancreatic cancer	Wald ratio	1	-0.21847	0.635551	0.7310343	0.803747	0.231274	2.79326	0.995249
digestive	Disorders of tooth development	Wald ratio	1	0.109235	0.317776	0.7310343	1.115425	0.598335	2.079392	0.995249
neoplasms	Benign neoplasm of lip, oral cavity, and pharynx	Wald ratio	1	0.168818	0.496524	0.7338565	1.183905	0.44737	3.133047	0.995249
circulatory system	Stricture of artery	Wald ratio	1	0.178749	0.526316	0.7341407	1.19572	0.426207	3.354584	0.995249
genitourinary	Prolapse of vaginal walls	Wald ratio	1	0.060576	0.178749	0.7346934	1.062448	0.748434	1.50821	0.995249
digestive	Anal and rectal conditions	Wald ratio	1	-0.04369	0.129096	0.7350154	0.957247	0.74325	1.232858	0.995249
injuries & poisonings	Poisoning by psychotropic agents	Wald ratio	1	0.119166	0.357498	0.7388827	1.126557	0.559043	2.270184	0.995249
neurological	Abnormality of gait	Wald ratio	1	-0.1291	0.387289	0.7388827	0.878889	0.411403	1.877592	0.995249
congenital anomalies	Other congenital musculoskeletal anomalies	Wald ratio	1	0.20854	0.635551	0.7428172	1.231878	0.354467	4.281145	0.995249
neurological	Hemiplegia	Wald ratio	1	-0.1291	0.397219	0.7451811	0.878889	0.403473	1.914495	0.995249
genitourinary	Nephritis; nephrosis; renal sclerosis	Wald ratio	1	0.129096	0.397219	0.7451811	1.1378	0.522331	2.478482	0.995249
neurological	Degenerative disease of the spinal cord	Wald ratio	1	-0.11917	0.367428	0.7456925	0.887661	0.432002	1.823929	0.995249
sense organs	Ptosis of eyelid	Wald ratio	1	0.119166	0.367428	0.7456925	1.126557	0.548267	2.314803	0.995249
injuries & poisonings	Fracture of neck of femur	Wald ratio	1	-0.11917	0.367428	0.7456925	0.887661	0.432002	1.823929	0.995249
musculoskeletal	Intervertebral disc disorders	Wald ratio	1	0.054618	0.168818	0.7462943	1.056137	0.758611	1.470352	0.995249
neoplasms	Large cell lymphoma	Wald ratio	1	0.20854	0.645482	0.746637	1.231878	0.347634	4.365288	0.995249
circulatory system	Peripheral vascular disease, unspecified	Wald ratio	1	-0.0993	0.307845	0.7470129	0.905467	0.495256	1.655448	0.995249
hematopoietic	Anemia of chronic disease	Wald ratio	1	0.188679	0.585899	0.747427	1.207654	0.383015	3.807759	0.995249
circulatory system	Heart valve disorders	Wald ratio	1	-0.07646	0.238332	0.7483367	0.926386	0.580657	1.477964	0.995249
digestive	Gastrointestinal hemorrhage	Wald ratio	1	-0.03476	0.109235	0.750347	0.96584	0.779691	1.196433	0.995249
digestive	Diseases of the oral soft tissues, excluding lesions specific for gingiva and tongue	Wald ratio	1	0.078451	0.248262	0.7520025	1.08161	0.664884	1.759526	0.995249
genitourinary	Functional disorders of bladder	Wald ratio	1	0.119166	0.377358	0.7521623	1.126557	0.537699	2.360299	0.995249
sense organs	Strabismus (not specified as paralytic)	Wald ratio	1	-0.15889	0.506455	0.7537295	0.853092	0.31615	2.301966	0.995249
neurological	Abnormal movement	Wald ratio	1	-0.09335	0.297915	0.7540274	0.910878	0.508007	1.63324	0.995249
neoplasms	Neoplasm of uncertain behavior	Wald ratio	1	-0.1291	0.41708	0.7569231	0.878889	0.388068	1.990491	0.995249
neurological	Extrapyramidal disease and abnormal movement disorders	Wald ratio	1	0.158888	0.516385	0.7583165	1.172206	0.426038	3.225226	0.995249
neoplasms	Cancer of stomach	Wald ratio	1	0.19861	0.655412	0.7618668	1.219706	0.337565	4.407103	0.995249
genitourinary	Hypertrophy of breast (Gynecomastia)	Wald ratio	1	-0.15889	0.526316	0.7627384	0.853092	0.304079	2.393343	0.995249
mental disorders	Aphasia/speech disturbance	Wald ratio	1	-0.11917	0.397219	0.7641772	0.887661	0.407499	1.933601	0.995249
hematopoietic	Pernicious anemia	Wald ratio	1	-0.16882	0.566038	0.7655157	0.844662	0.278524	2.561558	0.995249
congenital anomalies	Congenital anomalies of great vessels	Wald ratio	1	-0.10924	0.367428	0.7662395	0.896519	0.436314	1.842132	0.995249
digestive	Femoral hernia	Wald ratio	1	-0.17875	0.60576	0.7679313	0.836316	0.255116	2.741596	0.995249
circulatory system	Right bundle branch block	Wald ratio	1	-0.11917	0.40715	0.7697645	0.887661	0.399645	1.971605	0.995249
circulatory system	Precordial pain	Wald ratio	1	0.075472	0.258193	0.7700514	1.078393	0.650128	1.788772	0.995249
musculoskeletal	Other acquired deformities of limbs	Wald ratio	1	0.109235	0.377358	0.7722189	1.115425	0.532386	2.336976	0.995249
sense organs	Retinal detachment with retinal defect	Wald ratio	1	-0.11917	0.41708	0.775097	0.887661	0.391941	2.010356	0.995249
digestive	Other disorders of gallbladder	Wald ratio	1	-0.11917	0.41708	0.775097	0.887661	0.391941	2.010356	0.995249
neoplasms	Colorectal cancer	Wald ratio	1	-0.06753	0.238332	0.7769213	0.934702	0.58587	1.491232	0.995249
circulatory system	First degree AV block	Wald ratio	1	0.139027	0.496524	0.7794775	1.149155	0.434239	3.041085	0.995249
genitourinary	Inflammatory diseases of female pelvic organs	Wald ratio	1	-0.05561	0.19861	0.7794775	0.945907	0.640897	1.396074	0.995249
digestive	Peptic ulcer (excl. esophageal)	Wald ratio	1	0.049652	0.178749	0.781183	1.050906	0.740303	1.491825	0.995249
digestive	Ventral hernia	Wald ratio	1	-0.07448	0.268123	0.781183	0.928227	0.548812	1.569948	0.995249
dermatologic	Sicca syndrome	Wald ratio	1	-0.18868	0.685204	0.7830379	0.828052	0.216173	3.171865	0.995249
musculoskeletal	Hammer toe (acquired)	Wald ratio	1	0.097319	0.357498	0.7854512	1.102212	0.546962	2.221125	0.995249
injuries & poisonings	Open wounds of head; neck; and trunk	Wald ratio	1	0.067527	0.248262	0.785622	1.069859	0.65766	1.740411	0.995249
musculoskeletal	Unspecified polyarthropathy or polyarthritis	Wald ratio	1	0.0715	0.268123	0.7897258	1.074118	0.635069	1.816699	0.995249
neoplasms	Nevus, non-neoplastic	Wald ratio	1	-0.16882	0.635551	0.790528	0.844662	0.243047	2.935454	0.995249
pregnancy complicati	Miscarriage; stillbirth	Wald ratio	1	-0.06058	0.228401	0.790842	0.941222	0.601552	1.47269	0.995249
symptoms	Fever of unknown origin	Wald ratio	1	-0.06554	0.248262	0.7917799	0.93656	0.575719	1.523564	0.995249
injuries & poisonings	Complications of cardiac/vascular device, implant, and graft	Wald ratio	1	-0.09633	0.367428	0.7931964	0.908168	0.441983	1.866067	0.995249
mental disorders	Mood disorders	Wald ratio	1	-0.03575	0.139027	0.7970685	0.964882	0.734737	1.267115	0.995249
sense organs	Primary angle-closure glaucoma	Wald ratio	1	-0.14896	0.585899	0.7993122	0.861606	0.273263	2.716663	0.995249
musculoskeletal	Ankylosing spondylitis	Wald ratio	1	-0.15889	0.625621	0.7995201	0.853092	0.250298	2.907602	0.995249
injuries & poisonings	Mechanical complication of unspecified genitourinary device, implant, and graft	Wald ratio	1	0.109235	0.436941	0.8025873	1.115425	0.473705	2.626469	0.995249
neoplasms	Non-Hodgkins lymphoma	Wald ratio	1	-0.09136	0.367428	0.8036326	0.912689	0.444183	1.875356	0.995249
neoplasms	Cancer of other lymphoid, histiocytic tissue	Wald ratio	1	-0.08143	0.327706	0.8037593	0.921797	0.484938	1.752203	0.995249
circulatory system	Bundle branch block	Wald ratio	1	0.066534	0.268123	0.8040198	1.068798	0.631923	1.807701	0.995249
genitourinary	Uterine/Uterovaginal prolapse	Wald ratio	1	0.053625	0.218471	0.8061045	1.055088	0.687579	1.61903	0.995249
respiratory	Abnormal findings examination of lungs	Wald ratio	1	-0.07448	0.307845	0.8088302	0.928227	0.507705	1.697061	0.995249
circulatory system	Premature beats	Wald ratio	1	0.158888	0.665343	0.811256	1.172206	0.318165	4.318721	0.995249
genitourinary	Noninflammatory disorders of vulva and perineum	Wald ratio	1	-0.0993	0.41708	0.8118072	0.905467	0.399803	2.050683	0.995249
musculoskeletal	Pathologic fracture	Wald ratio	1	-0.15889	0.685204	0.8166281	0.853092	0.22271	3.267781	0.995249
injuries & poisonings	Fracture of foot	Wald ratio	1	-0.08441	0.367428	0.8183018	0.919055	0.447281	1.888437	0.995249
endocrine/metabolic	Acidosis	Wald ratio	1	-0.10924	0.476663	0.8187394	0.896519	0.352221	2.281937	0.995249
neurological	Other headache syndromes	Wald ratio	1	-0.04071	0.178749	0.819819	0.960103	0.676338	1.362924	0.995249
circulatory system	Other venous embolism and thrombosis	Wald ratio	1	0.148957	0.655412	0.8202117	1.160623	0.321213	4.193623	0.995249
musculoskeletal	Acquired foot deformities	Wald ratio	1	0.03575	0.158888	0.8219793	1.036396	0.759063	1.415057	0.995249
circulatory system	Tachycardia NOS	Wald ratio	1	-0.07547	0.337637	0.8231235	0.927306	0.478433	1.797319	0.995249
symptoms	Other abnormal blood chemistry	Wald ratio	1	-0.04369	0.19861	0.8258712	0.957247	0.64858	1.41281	0.995249
respiratory	Respiratory failure, insufficiency, arrest	Wald ratio	1	-0.06753	0.307845	0.8263736	0.934702	0.511246	1.708899	0.995249
neoplasms	Manlignant and unknown neoplasms of brain and nervous system	Wald ratio	1	-0.1291	0.60576	0.8312375	0.878889	0.268103	2.881159	0.995249
sense organs	Strabismus and other disorders of binocular eye movements	Wald ratio	1	-0.0864	0.40715	0.8319548	0.917232	0.412958	2.037286	0.995249
genitourinary	Cystitis	Wald ratio	1	0.060576	0.287984	0.8333986	1.062448	0.604186	1.868292	0.995249
endocrine/metabolic	Type 1 diabetes	Wald ratio	1	0.064548	0.307845	0.8339195	1.066677	0.583432	1.950186	0.995249
dermatologic	Psoriasis vulgaris	Wald ratio	1	0.078451	0.377358	0.8353112	1.08161	0.516246	2.26613	0.995249
injuries & poisonings	Fracture of clavicle or scapula	Wald ratio	1	0.082423	0.397219	0.8356194	1.085915	0.498512	2.365462	0.995249
genitourinary	stress incontinence, female	Wald ratio	1	-0.0427	0.20854	0.8377582	0.958198	0.636711	1.44201	0.995249
sense organs	Visual disturbances	Wald ratio	1	-0.05462	0.268123	0.838585	0.946847	0.559821	1.601441	0.995249
endocrine/metabolic	Hyposmolality and/or hyponatremia	Wald ratio	1	0.074479	0.367428	0.8393674	1.077322	0.524306	2.213638	0.995249
infectious diseases	Intestinal infection	Wald ratio	1	-0.03376	0.168818	0.8414806	0.9668	0.694441	1.345977	0.995249
genitourinary	Frequency of urination and polyuria	Wald ratio	1	-0.04965	0.248262	0.8414806	0.95156	0.58494	1.547965	0.995249
hematopoietic	Diseases of white blood cells	Wald ratio	1	-0.04866	0.248262	0.8446102	0.952506	0.585521	1.549503	0.995249

hematopoietic	Megaloblastic anemia	Wald ratio	1	0.093347	0.476663	0.8447406	1.097842	0.431316	2.79437	0.995249
sense organs	Perforation of tympanic membrane	Wald ratio	1	0.093347	0.476663	0.8447406	1.097842	0.431316	2.79437	0.995249
hematopoietic	Purpura and other hemorrhagic conditions	Wald ratio	1	-0.07051	0.367428	0.8478269	0.931922	0.453543	1.914875	0.995249
genitourinary	Inflammatory disease of cervix, vagina, and vulva	Wald ratio	1	-0.05462	0.287984	0.8495794	0.946847	0.538447	1.66501	0.995249
genitourinary	Cystitis and urethritis	Wald ratio	1	0.052632	0.278054	0.8498689	1.054041	0.611186	1.817781	0.995249
musculoskeletal	Osteoarthrosis, generalized	Wald ratio	1	-0.11917	0.635551	0.8512686	0.887661	0.25542	3.084885	0.995249
endocrine/metabolic	Acid-base balance disorder	Wald ratio	1	0.084409	0.456802	0.8533995	1.088074	0.444447	2.663768	0.995249
circulatory system	Aortic valve disease	Wald ratio	1	-0.08044	0.436941	0.8539421	0.922713	0.391863	2.172695	0.995249
infectious diseases	Intestinal infection due to C. difficile	Wald ratio	1	-0.10924	0.60576	0.8568952	0.896519	0.273481	2.938954	0.995249
genitourinary	Dysmenorrhea	Wald ratio	1	0.065541	0.367428	0.8584258	1.067737	0.519641	2.193942	0.995249
digestive	Acute periodontitis	Wald ratio	1	-0.0993	0.595829	0.8676323	0.905467	0.281639	2.911069	0.995249
endocrine/metabolic	Gout	Wald ratio	1	0.04568	0.278054	0.8695062	1.04674	0.606952	1.805189	0.995249
neoplasms	Cancer of brain and nervous system	Wald ratio	1	-0.10924	0.675273	0.8714911	0.896519	0.238647	3.367935	0.995249
injuries & poisonings	Traumatic arthropathy	Wald ratio	1	0.109235	0.675273	0.8714911	1.115425	0.296918	4.190293	0.995249
genitourinary	Cervicitis and endocervicitis	Wald ratio	1	-0.06852	0.427011	0.8725147	0.933774	0.404355	2.156359	0.995249
injuries & poisonings	Skull and face fracture and other intercranial injury	Wald ratio	1	0.04568	0.287984	0.8739677	1.04674	0.595253	1.840669	0.995249
hematopoietic	Other deficiency anemia	Wald ratio	1	0.0715	0.456802	0.8756218	1.074118	0.438747	2.629601	0.995249
dermatologic	Decubitus ulcer	Wald ratio	1	-0.08044	0.516385	0.8762149	0.922713	0.33536	2.538766	0.995249
musculoskeletal	Other acquired musculoskeletal deformity	Wald ratio	1	-0.05561	0.357498	0.8763834	0.945907	0.469397	1.906148	0.995249
pregnancy complicati	Early or threatened labor; hemorrhage in early pregnancy	Wald ratio	1	-0.04866	0.317776	0.8782997	0.952506	0.510942	1.775675	0.995249
musculoskeletal	Other disorders of bone and cartilage	Wald ratio	1	0.037736	0.248262	0.8791869	1.038457	0.638357	1.689326	0.995249
respiratory	Other diseases of lung	Wald ratio	1	0.082423	0.556107	0.8821737	1.085915	0.365113	3.229713	0.995249
genitourinary	Urinary calculus	Wald ratio	1	0.027805	0.188679	0.8828412	1.028196	0.710344	1.488273	0.995249
dermatologic	Scar conditions and fibrosis of skin	Wald ratio	1	0.046673	0.317776	0.8832307	1.04778	0.562049	1.953287	0.995249
musculoskeletal	Hallux valgus (Bunion)	Wald ratio	1	0.028798	0.19861	0.8847109	1.029217	0.697344	1.519032	0.995249
symptoms	Edema	Wald ratio	1	0.054618	0.377358	0.8849186	1.056137	0.504088	2.212759	0.995249
mental disorders	Neurological disorders	Wald ratio	1	0.032771	0.228401	0.8859125	1.033313	0.660409	1.616781	0.995249
circulatory system	Carditis	Wald ratio	1	0.049652	0.347567	0.886403	1.050906	0.531752	2.076915	0.995249
digestive	Hemorrhage of rectum and anus	Wald ratio	1	-0.01986	0.139027	0.886403	0.980335	0.746505	1.287409	0.995249
digestive	Hematemesis	Wald ratio	1	0.048659	0.347567	0.88866	1.049863	0.531224	2.074854	0.995249
circulatory system	Vascular insufficiency of intestine	Wald ratio	1	-0.08937	0.645482	0.8898757	0.914503	0.258072	3.240636	0.995249
digestive	Irritable Bowel Syndrome	Wald ratio	1	0.028798	0.20854	0.8901651	1.029217	0.683902	1.548888	0.995249
sense organs	Tinnitus	Wald ratio	1	-0.09335	0.685204	0.891638	0.910878	0.237795	3.48913	0.995249
digestive	Cholelithiasis with acute cholecystitis	Wald ratio	1	-0.05362	0.397219	0.8926119	0.947788	0.435102	2.064577	0.995249
neurological	Convulsions	Wald ratio	1	-0.04369	0.327706	0.8939298	0.957247	0.503587	1.819587	0.995249
symptoms	Neuralgia, neuritis, and radiculitis NOS	Wald ratio	1	-0.05859	0.446872	0.8956874	0.943093	0.392798	2.26433	0.995249
digestive	Functional digestive disorders	Wald ratio	1	0.013903	0.109235	0.8987245	1.014	0.818568	1.25609	0.995249
circulatory system	Raynaud's syndrome	Wald ratio	1	0.057597	0.456802	0.8996631	1.059288	0.432689	2.593296	0.995249
circulatory system	Arrhythmia (cardiac) NOS	Wald ratio	1	0.062562	0.506455	0.9016879	1.064561	0.394518	2.872588	0.995249
respiratory	Pleurisy; pleural effusion	Wald ratio	1	-0.02284	0.188679	0.9036493	0.977419	0.675264	1.414776	0.995249
injuries & poisonings	Injuries to the nervous system	Wald ratio	1	0.050645	0.427011	0.9055886	1.05195	0.455529	2.429261	0.995249
circulatory system	Pericarditis	Wald ratio	1	0.051639	0.436941	0.9059236	1.052995	0.447192	2.479467	0.995249
genitourinary	Chronic glomerulonephritis, NOS	Wald ratio	1	-0.06157	0.536246	0.9085919	0.940288	0.328699	2.689818	0.995249
neoplasms	Malignant neoplasm of other and ill-defined sites within the digestive organs and perit	Wald ratio	1	-0.02383	0.20854	0.9090113	0.976449	0.648838	1.469476	0.995249
dermatologic	Psoriatic arthropathy	Wald ratio	1	-0.06653	0.585899	0.9095871	0.935631	0.296741	2.950065	0.995249
infectious diseases	Viral hepatitis	Wald ratio	1	-0.05065	0.446872	0.9097663	0.950616	0.395931	2.282391	0.995249
congenital anomalies	Genitourinary congenital anomalies	Wald ratio	1	-0.04369	0.387289	0.9101729	0.957247	0.448081	2.044989	0.995249
circulatory system	Atrioventricular [AV] block	Wald ratio	1	-0.03674	0.337637	0.9133425	0.963924	0.497326	1.868292	0.995249
respiratory	Pulmonary collapse; interstitial and compensatory emphysema	Wald ratio	1	-0.03774	0.347567	0.9135424	0.962967	0.487255	1.903122	0.995249
musculoskeletal	Rupture of synovium	Wald ratio	1	0.066534	0.635551	0.9166238	1.068798	0.307541	3.71439	0.995249
congenital anomalies	Cardiac congenital anomalies	Wald ratio	1	0.031778	0.307845	0.9177838	1.032288	0.564622	1.887313	0.995249
mental disorders	Phobia	Wald ratio	1	-0.07051	0.695134	0.9192103	0.931922	0.238599	3.6399	0.995249
endocrine/metabolic	Disorders of the pituitary gland and its hypothalamic control	Wald ratio	1	-0.05859	0.585899	0.9203443	0.943093	0.299108	2.973595	0.995249
digestive	Jaundice (not of newborn)	Wald ratio	1	-0.05065	0.516385	0.9218712	0.950616	0.345501	2.615538	0.995249
hematopoietic	Iron deficiency anemias, unspecified or not due to blood loss	Wald ratio	1	-0.01688	0.178749	0.9247561	0.98326	0.692651	1.395797	0.995249
digestive	Diseases of lips	Wald ratio	1	-0.05362	0.595829	0.9282872	0.947788	0.294802	3.047131	0.995249
symptoms	Nonspecific findings on examination of blood	Wald ratio	1	-0.01787	0.19861	0.9282872	0.982284	0.665544	1.449763	0.995249
musculoskeletal	Spinal stenosis	Wald ratio	1	0.02284	0.258193	0.9295099	1.023103	0.616796	1.697061	0.995249
circulatory system	Cerebral ischemia	Wald ratio	1	0.024826	0.287984	0.9313019	1.025137	0.582968	1.802681	0.995249
infectious diseases	Viral warts & HPV	Wald ratio	1	0.041708	0.486594	0.9316935	1.04259	0.401714	2.705893	0.995249
sense organs	Cataract	Wald ratio	1	-0.00993	0.119166	0.9335865	0.990119	0.783883	1.250614	0.995249
neoplasms	Benign neoplasm of other endocrine glands and related structures	Wald ratio	1	0.043694	0.526316	0.9338365	1.044663	0.372363	2.930793	0.995249
hematopoietic	Decreased white blood cell count	Wald ratio	1	-0.02284	0.278054	0.9345331	0.977419	0.566757	1.685639	0.995249
hematopoietic	Neutropenia	Wald ratio	1	-0.02284	0.278054	0.9345331	0.977419	0.566757	1.685639	0.995249
digestive	Ulceration of intestine	Wald ratio	1	0.048659	0.595829	0.9349118	1.049863	0.326552	3.375301	0.995249
digestive	Ulceration of the lower GI tract	Wald ratio	1	0.038729	0.476663	0.9352431	1.039489	0.408391	2.645841	0.995249
circulatory system	Intracranial hemorrhage	Wald ratio	1	-0.02979	0.367428	0.9353775	0.970648	0.47239	1.994448	0.995249
genitourinary	Breast conditions, congenital or relating to hormones	Wald ratio	1	-0.03972	0.506455	0.9374849	0.961057	0.356161	2.593296	0.995249
digestive	Obstruction of bile duct	Wald ratio	1	0.042701	0.556107	0.938794	1.043626	0.350895	3.103937	0.995249
symptoms	Effects of other external causes	Wald ratio	1	-0.01787	0.238332	0.9402147	0.982284	0.615694	1.567145	0.995249
pregnancy complicati	Obstetrical/birth trauma	Wald ratio	1	0.017875	0.238332	0.9402147	1.018036	0.638103	1.624183	0.995249
injuries & poisonings	Poisoning by anticonvulsants and anti-Parkinsonism drugs	Wald ratio	1	-0.04965	0.685204	0.9422328	0.95156	0.248416	3.644964	0.995249
digestive	Appendiceal conditions	Wald ratio	1	0.018868	0.268123	0.9438989	1.019047	0.602509	1.723555	0.995249
digestive	Other diseases of the teeth and supporting structures	Wald ratio	1	0.020854	0.297915	0.9441937	1.021073	0.569465	1.830825	0.995249
dermatologic	Other dyschromia	Wald ratio	1	0.038729	0.556107	0.9444779	1.039489	0.349504	3.091632	0.995249
pregnancy complicati	Hemorrhage during pregnancy; childbirth and postpartum	Wald ratio	1	0.024826	0.357498	0.9446359	1.025137	0.508714	2.065808	0.995249
respiratory	Cough	Wald ratio	1	0.019861	0.287984	0.9450171	1.02006	0.580081	1.793753	0.995249
symptoms	Pain	Wald ratio	1	-0.03774	0.556107	0.9458994	0.962967	0.323775	2.864043	0.995249
mental disorders	Altered mental status	Wald ratio	1	0.021847	0.327706	0.9468471	1.022087	0.537699	1.94284	0.995249
musculoskeletal	Osteomyelitis, periostitis, and other infections involving bone	Wald ratio	1	0.041708	0.625621	0.9468471	1.04259	0.305897	3.55347	0.995249
musculoskeletal	Pain in joint	Wald ratio	1	0.011917	0.178749	0.9468471	1.011988	0.712888	1.436578	0.995249
genitourinary	Hydronephrosis	Wald ratio	1	0.02284	0.347567	0.9476053	1.023103	0.517683	2.021968	0.995249
digestive	Appendicitis	Wald ratio	1	-0.01787	0.278054	0.9487427	0.982284	0.569578	1.69403	0.995249
genitourinary	Abnormal findings on mammogram or breast exam	Wald ratio	1	-0.02483	0.387289	0.9488886	0.975479	0.456616	2.08394	0.995249
dermatologic	Psoriasis and related disorders	Wald ratio	1	0.019861	0.327706	0.9516729	1.02006	0.536632	1.938985	0.995249
congenital anomalies	Cardiac and circulatory congenital anomalies	Wald ratio	1	-0.01787	0.297915	0.9521556	0.982284	0.547832	1.761275	0.995249
circulatory system	Chronic pulmonary heart disease	Wald ratio	1	-0.03774	0.635551	0.9526534	0.962967	0.277089	3.346598	0.995249
digestive	Diverticulosis and diverticulitis	Wald ratio	1	-0.00586	0.099305	0.9529521	0.994158	0.818324	1.207773	0.995249
injuries & poisonings	Dislocation	Wald ratio	1	-0.01986	0.337637	0.9530927	0.980335	0.505793	1.9001	0.995249
neurological	Multiple sclerosis	Wald ratio	1	0.024826	0.427011	0.9536375	1.025137	0.443918	2.367342	0.995249
injuries & poisonings	Fracture of ankle and foot	Wald ratio	1	0.018868	0.327706	0.9540866	1.019047	0.536099	1.937061	0.995249
digestive	Ascites (non malignant)	Wald ratio	1	0.02284	0.397219	0.9541469	1.023103	0.469677	2.228637	0.995249
neoplasms	Colon cancer	Wald ratio	1	-0.01589	0.287984	0.9560011	0.984237	0.559709	1.730759	0.995249
genitourinary	Lump or mass in breast	Wald ratio	1	0.021847	0.397219	0.9561385	1.022087	0.469211	2.226425	0.995249
respiratory	Respiratory failure	Wald ratio	1	0.017875	0.347567	0.958984	1.018036	0.515119	2.011954	0.995249
genitourinary	Glomerulonephritis	Wald ratio	1	0.023833	0.486594	0.9609356	1.024119	0.394597	2.657955	0.995249
sense organs	Amblyopia	Wald ratio	1	0.030785	0.665343	0.9630961	1.031263	0.27991	3.799449	0.995249
genitourinary	Postmenopausal atrophic vaginitis	Wald ratio	1	0.020854	0.466733	0.9643617	1.021073	0.40904	2.548871	0.995249
genitourinary	Calculus of kidney	Wald ratio	1	0.011917	0.278054	0.9658154	1.011988	0.586802	1.745257	0.995249
genitourinary	Pelvic inflammatory disease (PID)	Wald ratio	1	-0.02085	0.506455	0.9671552	0.979362	0.362944	2.64269	0.995249
respiratory	Respiratory insufficiency	Wald ratio	1	0.014896	0.367428	0.9676622	1.015007	0.493979	2.085596	0.995249
hematopoietic	Iron deficiency anemias	Wald ratio	1	-0.00705	0.178749	0.968536	0.992974	0.699494	1.409587	0.995249
digestive	Diverticulosis	Wald ratio	1	-0.00387	0.099305	0.9688904	0.996135	0.819951	1.210175	0.995249
digestive	Acute pancreatitis	Wald ratio	1	-0.01291	0.347567	0.9703711	0.987173	0.499503	1.95096	0.995249
respiratory	Emphysema	Wald ratio	1	0.01291	0.377358	0.9727093	1.012993	0.483496	2.122368	0.995249
dermatologic	Pruritus and related conditions	Wald ratio	1	-0.01887	0.556107	0.9729341	0.981309	0.329942	2.918595	0.995249
injuries & poisonings	Injury, NOS	Wald ratio	1	0.00566	0.168818	0.9732524	1.005676	0.722366	1.400101	0.995249
dermatologic	Atopic/contact dermatitis due to other or unspecified	Wald ratio	1	0.010924	0.337637	0.9741906	1.010983	0.521605	1.959504	0.995249
digestive	Diseases of pancreas	Wald ratio	1	-0.00924	0.297915	0.9752695	0.990807	0.552585	1.776557	0.995249

dermatologic	Degenerative skin conditions and other dermatoses	Wald ratio	1	-0.00636	0.20854	0.9756873	0.993665	0.660278	1.495384	0.995249
circulatory system	Orthostatic hypotension	Wald ratio	1	0.01291	0.427011	0.9758816	1.012993	0.43866	2.339298	0.995249
neoplasms	Cervical intraepithelial neoplasia [CIN] [Cervical dysplasia]	Wald ratio	1	0.00993	0.337637	0.9765362	1.00998	0.521088	1.957559	0.995249
neurological	Inflammatory and toxic neuropathy	Wald ratio	1	-0.01192	0.41708	0.9772064	0.988154	0.436314	2.237952	0.995249
dermatologic	Other local infections of skin and subcutaneous tissue	Wald ratio	1	0.005561	0.228401	0.9805752	1.005577	0.642682	1.573382	0.995249
genitourinary	Genital prolapse	Wald ratio	1	0.003575	0.148957	0.9808526	1.003581	0.749476	1.34384	0.995249
neoplasms	Secondary malignant neoplasm of digestive systems	Wald ratio	1	0.009335	0.397219	0.9812514	1.009378	0.463376	2.198741	0.995249
genitourinary	Noninflammatory disorders of cervix	Wald ratio	1	0.005859	0.287984	0.9837683	1.005876	0.572015	1.768811	0.995249
neurological	Epilepsy	Wald ratio	1	0.009831	0.516385	0.9848104	1.00988	0.36704	2.778598	0.995249
endocrine/metabolic	Type 2 diabetes with neurological manifestations	Wald ratio	1	0.00993	0.645482	0.9877253	1.00998	0.285015	3.578968	0.995249
infectious diseases	Bacterial enteritis	Wald ratio	1	-0.00457	0.297915	0.9877662	0.995442	0.55517	1.784868	0.995249
symptoms	Symptoms involving skin and other integumentary tissue	Wald ratio	1	-0.00546	0.367428	0.98814	0.994553	0.484024	2.043568	0.995249
musculoskeletal	Aseptic necrosis of bone	Wald ratio	1	0.009434	0.665343	0.9886871	1.009479	0.273997	3.719188	0.995249
genitourinary	Bladder neck obstruction	Wald ratio	1	0.004767	0.347567	0.9890579	1.004778	0.508411	1.985753	0.995249
endocrine/metabolic	Hypothyroidism	Wald ratio	1	-0.00189	0.139027	0.9891719	0.998115	0.760044	1.310758	0.995249
circulatory system	Nonspecific abnormal findings on radiological and other examination of other intratho	Wald ratio	1	-0.00844	0.655412	0.9897245	0.991595	0.274433	3.58288	0.995249
circulatory system	Other disorders of arteries and arterioles	Wald ratio	1	-0.00546	0.427011	0.9897948	0.994553	0.430674	2.296715	0.995249
dermatologic	Psoriasis	Wald ratio	1	0.003972	0.327706	0.9903289	1.00398	0.528173	1.908421	0.995249
endocrine/metabolic	Abnormal glucose	Wald ratio	1	0.00576	0.595829	0.9922872	1.005776	0.312839	3.233564	0.995249
respiratory	Respiratory abnormalities	Wald ratio	1	-0.00556	0.625621	0.9929078	0.994454	0.291773	3.389408	0.995249
genitourinary	Infertility, female	Wald ratio	1	0.003476	0.427011	0.9935057	1.003482	0.434541	2.317333	0.995249
congenital anomalies	Congenital anomalies of urinary system	Wald ratio	1	-0.00397	0.526316	0.9939783	0.996036	0.355031	2.79437	0.995249
hematopoietic	Thrombocytopenia	Wald ratio	1	0.000288	0.387289	0.9994067	1.000288	0.468229	2.136939	0.999407
